# Discovery and Engineering of a Rat Endogenous Retrovirus Reverse Transcriptase for Efficient Prime Editing

**DOI:** 10.1002/advs.75888

**Published:** 2026-06-26

**Authors:** Linsha Ma, Pengcheng Yao, Shengqi Wu, Yuanyuan Shi, Lang Qin, Baitao Li, Jiayi Zhu, Minhua Huang, Yichong Zhu, Yuwen Song, Jinhuan Pang, Ziping Guo, Guochuan Wu, Chen Wang, Kewei Xu, Ruihua Huang, Quan Kuang, Liang Qu, Changtian Pan, Xianrong Xie, Qinlong Zhu, Jiaying Huang, Qiupeng Lin

**Affiliations:** ^1^ Guangdong Basic Research Center of Excellence for Precise Breeding of Future Crops Key Laboratory for Enhancing Resource Use Efficiency of Crops in South China State Key Laboratory For Conservation and Utilization of Subtropical Agro‐Bioresources Guangdong Provincial Key Laboratory of Plant Molecular Breeding Ministry of Agriculture and Rural Affairs College of Agriculture South China Agricultural University Guangzhou China; ^2^ Department of Radiology The Affiliated Panyu Central Hospital GMU‐GIBH Joint School of Life Sciences Guangdong Provincial Key Laboratory of Protein Modification and Disease The Guangdong‐Hong Kong‐Macao Joint Laboratory for Cell Fate Regulation and Diseases Guangzhou Medical University Guangzhou China; ^3^ State Key Laboratory of Respiratory Disease National Clinical Research Center for Respiratory Disease National Center for Respiratory Medicine Joint International Research Laboratory of Respiratory Health Guangdong Basic Research Center of Excellence for Respiratory Medicine Guangzhou Institute of Respiratory Health Guangzhou Medical University Guangzhou China; ^4^ College of Fisheries Southwest University Chongqing China; ^5^ Department of Life Sciences Beijing Normal‐Hong Kong Baptist University Zhuhai China; ^6^ Dermatology Hospital of Southern Medical University Guangzhou China; ^7^ Guangdong Key Laboratory of Biotechnology for Plant Development College of Life Sciences South China Normal University Guangzhou China; ^8^ State Key Laboratory of Quantitative Synthetic Biology Shenzhen Institute of Synthetic Biology Shenzhen Institutes of Advanced Technology Chinese Academy of Sciences Shenzhen China; ^9^ College of Life Sciences Nanchang Normal University Nanchang China; ^10^ Frontier Innovation Center Department of Systems Biology For Medicine Qidong‐Fudan Innovative Institution of Medical Sciences School of Basic Medical Sciences Fudan University Shanghai China; ^11^ Clinical Center for Biotherapy Zhongshan Hospital Fudan University Shanghai China; ^12^ Department of Horticulture College of Agriculture and Biotechnology Zhejiang University Hangzhou China; ^13^ Key Laboratory of Horticultural Plant Growth Development and Quality Improvement Ministry of Agricultural Zhejiang University Hangzhou China; ^14^ Institute of BioFoundry College of Life Sciences South China Agricultural University Guangzhou China

**Keywords:** crop breeding, gene therapy, hard‐to‐edit, prime editors, protein engineering, RERV (Rattus norvegicus endogenous retrovirus), reverse transcriptase, TRAP‐seq‐PE

## Abstract

CRISPR‐based prime editors (PEs) install precise edits into genomic DNA without generating double‐strand breaks. Their editing efficiency is highly dependent on reverse transcriptases (RTs), but efficient RT candidates remain limited. Here, we identified 19 novel active RTs by screening 558 candidates. Among them, RERV‐RT, derived from *Rattus norvegicus*, exhibited the highest activity. Through structure‐guided engineering and deep mutational scanning, we developed an optimized variant, enRERV‐RT, which outperforms conventional M‐MLV‐RT‐based PE systems by 1.20‐fold in mammalian and plant cells, and by 1.88‐fold at hard‐to‐edit loci, while enabling precise multiplex editing of functionally relevant genes. Additionally, we developed a high‐throughput platform, TRAP‐seq‐PE, to systematically evaluate prime editor performance. Across diverse mutation types, we found that PE systems based on enRERV‐RT exhibited higher editing efficiencies than those based on M‐MLV‐RT. Collectively, our work establishes a versatile, high‐efficiency PE system, thereby facilitating advances in clinical gene therapy and precise crop breeding.

## Graphical abstract

We screened 558 reverse transcriptases and engineered an optimized rat endogenous retrovirus‐derived variant, enRERV‐RT, via structure‐guided engineering and deep mutational scanning. This enhanced prime editor, based on the engineered RT, outperforms conventional M‐MLV‐RT systems across plant and animal cells, particularly at hard‐to‐edit loci, enabling efficient precise genome editing for agricultural applications and clinical gene therapy.

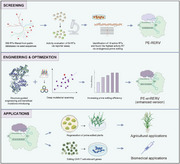

## 1 Background

Clustered Regularly Interspaced Short Palindromic Repeats (CRISPR)‐based technologies can modify specific target genomic DNA sequences in living cells, and have revolutionized fundamental biological research and enabled diverse clinical and agricultural applications [1, 2, 3, 4]. Prime editing, a CRISPR/Cas‐based system, can introduce precise DNA substitutions and install small indels (insertions/deletions) at target genomic loci without requiring donor DNA or generating double‐strand breaks [5], representing a significant advance in genome editing due to its unique combination of versatility, specificity, and precision [6, 7, 8, 9, 10, 11]. First developed in 2019, the prime editor (PE) consists of a Cas9 nickase (whose HNH domain is inactivated via the H840A amino acid substitution) and a Moloney murine leukemia virus reverse transcriptase (M‐MLV‐RT). It also includes an elongated prime editing guide RNA (pegRNA), which contains a single‐guide RNA (sgRNA), a primer binding site (PBS) for hybridizing with the 3′ end of the nicked DNA strand, and an RT template (RTT) encoding the desired edits [5]. The design parameters of these components remain an active area of research, with optimization studies ongoing [12, 13, 14, 15, 16, 17, 18, 19].

Reverse transcriptases (RNA‐dependent DNA polymerases, RTs) are key components of PEs. Previous studies have shown that RT activity exerts a critical impact on PE efficiency [20]. Two primary strategies for enhancing RT enzymatic activity have been leveraged to improve PE performance. The first entails engineering RTs through rational design or directed evolution [5, 21, 22, 23, 24]. For example, the most widely used engineered RT in PE systems is an M‐MLV‐RT variant harboring five amino acid substitutions (D200N/T306K/W313F/T330P/L603W), which boosts the editing efficiency by 1.6‐ to 5.1‐fold compared with the wild‐type M‐MLV‐RT utilized in the original PE1 system [5]. The second approach entails identifying novel RTs from diverse sources [13, 25, 26, 27, 28, 29]. For instance, Liu et al. developed pvPE by discovering an RT from PERV (porcine endogenous retrovirus) of Bama mini‐pigs [25], and Doman et al. generated the PE6a‐c series using a panel of novel RTs [26]. To date, the variety of highly efficient RTs suitable for prime editing remains limited and requires further exploration. Exploring and engineering more efficient RTs will help us further break through efficiency bottlenecks, improve editing performance, and expand editing scenarios.

Here, we identified 19 novel active RTs from 558 candidates and found that RERV (*Rattus norvegicus* endogenous retrovirus), an uncharacterized RT, exhibited the highest PE efficiency. By comparing the protein sequence and structure of RERV‐RT with M‐MLV‐RT and PERV‐RT, we introduced mutations at five amino acid residues of RERV‐RT, resulting in a 2.80‐fold higher editing efficiency than wild‐type (wt) RERV‐RT. Through deep mutational scanning (DMS) of the core regions of RERV‐RT, we identified the beneficial W149F mutation. By combining another two functionally improving mutations, we developed an optimized variant, enRERV‐RT, which outperformed conventional M‐MLV‐RT‐based PE systems by an average of 1.20‐fold in both mammalian and plant cells. We also established a high‐throughput evaluation library for prime editor properties, which enables a comprehensive assessment of prime editor performance across distinct mutation types. This novel enRERV‐RT‐based prime editor (PE‐enRERV) exhibits universal and highly efficient editing across diverse species, serving as a potential editing tool for therapeutic and agricultural applications.

## 2 Results

### 2.1 Discovery of Active RTs for Prime Editing via Protein Database Mining

**FIGURE 1:**
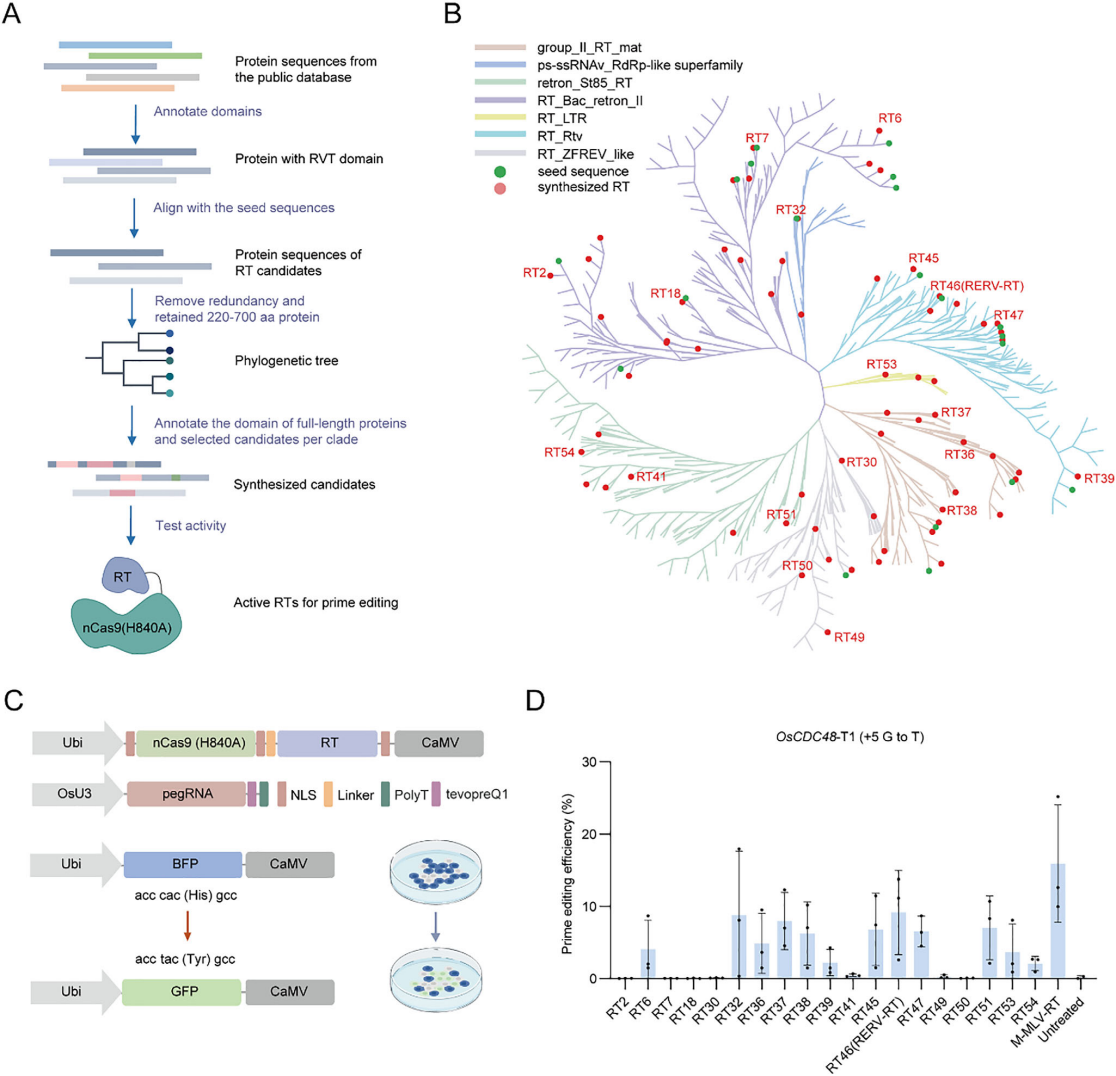
Discovery of active reverse transcriptase for prime editing. (A) Workflow for the discovery of candidate RTs with prime editing potential. (B) Phylogenetic classification of 558 RTs identified in this study. RTs harboring functional domains in distinct clades based on the evolutionary characteristics are color‑coded for visualization. Selected candidates and seed RTs are labeled with red dots and green dots, respectively. (C) Schematic representation of the BFP‐to‐GFP reporter system. Vectors for expression of nCas9‐RT, transcription of pegRNA, and expression of BFP protein were co‐transformed into rice protoplasts for prime editor activity validation. (D) Prime editing efficiencies of RTs at the OsCDC48‐T1 endogenous target site in rice protoplasts. The 19 validated RTs are annotated on the phylogenetic tree shown in Figure 1B. Data are presented as means ± standard deviations from three independent biological replicates.

To identify novel RTs capable of mediating efficient prime editing, we screened RT domain‐containing proteins from the NCBI database and identified 1943 RT candidates with 40%–90% sequence similarity to the 21 seed sequences of RTs currently used in prime editing [26]. After eliminating sequence redundancy, we selected the 558 unique full‐length proteins (length: 220–700 aa) for phylogenetic tree construction. We constructed a phylogenetic tree and found that the candidates could be clustered into seven distinct clades based on the evolutionary characteristics of their functional RT domains. The sequences of 558 unique sequences in the phylogenetic tree and their corresponding seed RTs are provided in Table S1. We selected a minimum of three candidates from each of the seven clades, ultimately yielding 54 RT candidates (designated as RT1‐RT54) with highly intact RVT domains (Figure 1A,B). In this study, the RVT domain is defined broadly as a functional module responsible for reverse transcription, corresponding to the core domain found in retrotransposons and retroviruses. This domain is widely distributed across diverse reverse‐transcribing elements, including retrotransposons, retroviruses, group II introns, bacterial msDNAs, hepadnaviruses, and caulimoviruses.

These RTs were then synthesized and cloned into the plant PE vector ePPEplus to construct the corresponding RT‐ePPEplus vectors [30]. We co‐transformed these vectors with an established BFP‐to‐GFP reporter system [13] into rice protoplasts to rapidly assess the activity of 54 RTs (Figure 1C). Microscopic fluorescence analysis showed that 19 out of 54 RTs induced green fluorescence, confirming the editing activity of their corresponding prime editors (Figure S1). We then annotated the domain architectures of these 19 functional RTs with the HMMER web server [31] and found that they mainly contain five types of domains accompanying the conserved RVT catalytic core: RT_RNaseH_2 and RNase H for supporting complete cDNA synthesis, RVT_Thumb that can enhance substrate handling, GIIM stabilizing RNA structures, and peptidase_A2B that may be involved in activation of the reverse transcription process (Figure S2).

We further evaluated the editing efficiency of these active RTs at endogenous loci in rice protoplasts. Results showed that RT46 (RERV‐RT) exhibited the highest editing activity (Figure 1D), reaching 9.2% efficiency at the *OsCDC48* locus for G‐to‐T editing, which was 1.04‐fold and 1.15‐fold higher than that of the second‐ (RT32) and third‐ (RT37) most efficient RTs, respectively (Figures 1D and S3). We thus used RERV‐RT for subsequent optimization.

### 2.2 Enhancing Prime Editing Efficiency via Structure‐Guided Engineering of RERV‐RT

**FIGURE 2:**
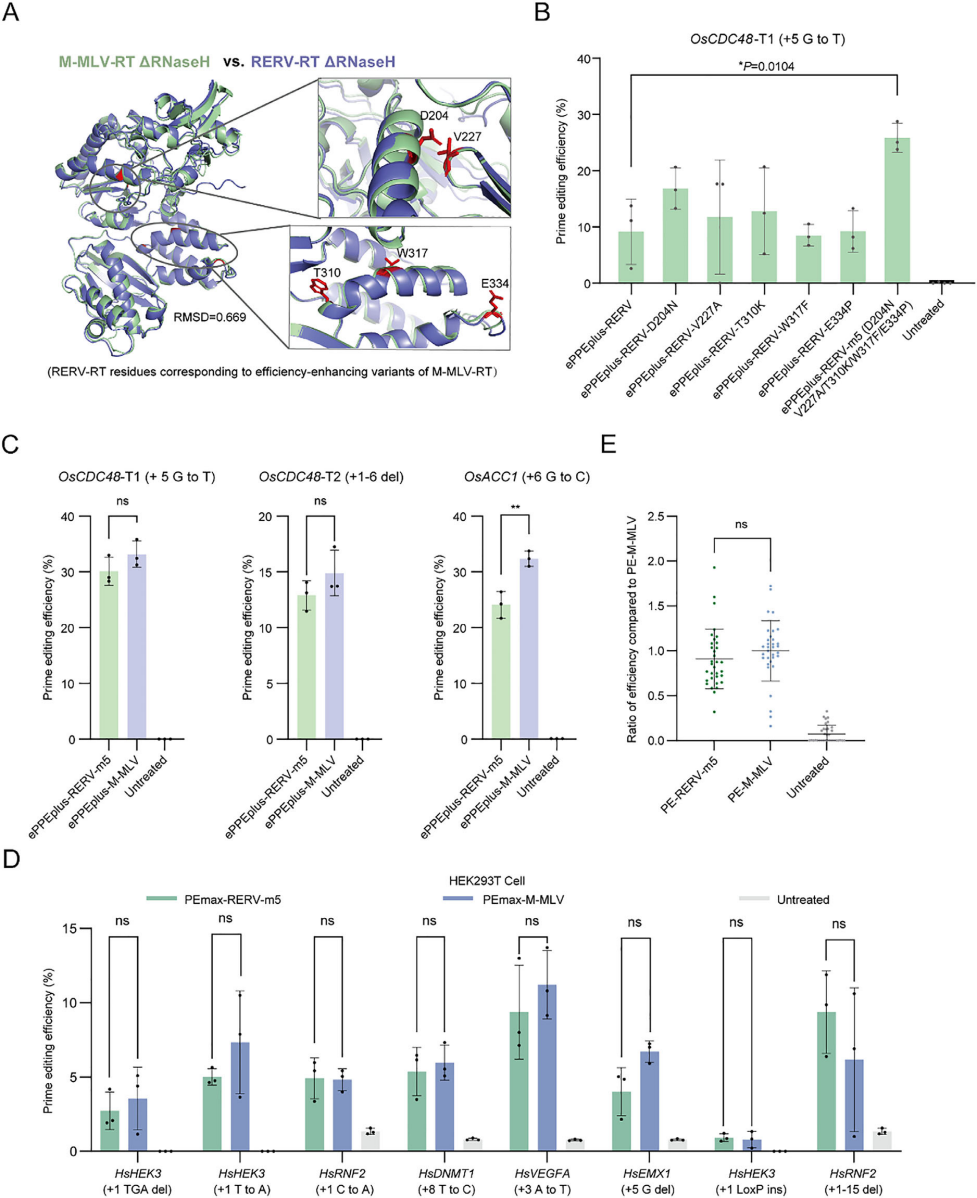
Engineered RERV‐RT for prime editing in rice and human cells. (A) Structural comparison of M‐MLV‐RT ΔRNaseH and RERV‐RT ΔRNaseH (RMSD = 0.669 Å). The local structures surrounding residues D204, V227, T310, W317 and E334 in RERV‐RT are enlarged and colored in red. The protein structure of RERV‐RT ΔRNaseH was predicted by AlphaFold 3 and colored in purple. The cryo‐electron microscopy structure of M‐MLV RT ΔRNaseH (PDB: 8WUV) is colored in green. (B) Editing efficiencies of ePPEplus‐RERV and its engineered variants at the OsCDC48‐T1 site. (C) Comparison of editing efficiencies between M‐MLV‐RT and RERV‐RT‐m5 in the ePPEplus vector architecture in rice protoplast. (D) Comparison of editing efficiencies between M‐MLV‐RT and RERV‐RT‐m5 in the PEmax vector architecture in HEK293T cells. (E) Summary of editing efficiency comparisons between PE‐RERV‐m5 and PE‐M‐MLV across 11 endogenous sites in rice protoplasts and HEK293T cells. Data are presented as means ± standard deviations from three independent biological replicates. Two‐tailed Student's *t*‐test was used for statistical analysis. ns: no significance, *p* > 0.05; ^*^ *p* < 0.05, ^**^ *p* < 0.01.

We then predicted the protein structure of RERV‐RT using AlphaFold 3 [32] and compared it with the cryo‐electron microscopy (cryo‐EM) structure of M‐MLV‐RT (PDB: 8WUV) [33]. High structural similarity was observed between RERV‐RT and M‐MLV‐RT (Figures 2A andS4), suggesting that the efficiency‐enhancing mutations identified in M‐MLV‐RT may also be applicable to RERV‐RT. To improve the activity of RERV‐RT, we introduced five previously optimized mutations implemented in PE2 [5] and ePPEplus [30] into RERV‐RT (D204N, V227A, T310K, W317F, E334P) to generate engineered variants. We constructed the corresponding plant prime editors harboring RERV‐RT with these five mutations and tested their editing efficiencies in rice protoplasts. Results showed that the aforementioned amino acid substitutions enhanced RERV‐RT activity, and the editor carrying all five mutations (designated RERV‐RT‐m5) exhibited a 2.82‐fold increase in efficiency compared with the wild‐type RERV‐RT (Figure 2B). We further compared the performance of RERV‐RT‐m5‐based prime editors (PE‐RERV‐m5) with M‐MLV‐RT‐based prime editors (PE‐M‐MLV) in rice protoplasts and human embryonic kidney 293T (HEK293T) cells. These two prime editors displayed comparable editing efficiencies in both plant and mammalian cells, highlighting the engineering potential of PE‐RERV (Figure 2C–E).

To further explore whether RERV‐RT can be miniaturized while retaining its activity, we evaluated seven truncated RERV‑RT variants using the BFP‑to‑GFP reporter system in rice protoplasts. Our data showed that deletion of the first 51 N‑terminal amino acids or the 174 C‑terminal amino acids did not impair the enzymatic activity of RERV‑RT. This conclusion was further validated at the endogenous *OsCDC48*‑T1 locus in rice protoplasts (Figure S5).

Previous studies demonstrated that MLH1dn protein [34, 35, 36] and MLH1 small binders (MLH1‐SBs) [37], which are AI‐designed proteins that specifically binds to MLH1, can improve PE efficiency by inhibiting the DNA mismatch repair (MMR) pathway. To further enhance the efficiency of RERV‐RT‐m5, we constructed PE‐RERV‐m5‐OsMLHdn and PE‐RERV‐m5‐SB2 vectors and tested their performance at three endogenous loci in rice protoplasts. However, no significant efficiency enhancement was observed for these variants across all tested loci (Figure S6). Additionally, we evaluated the MS2 coat protein (MCP)‐mediated pegRNA recruitment system [38, 39, 40] by constructing PE‐RERV‐m5‐MCP vectors, which were co‐transfected with pegRNA‐MS2 vectors into rice protoplasts. Unfortunately, this approach showed no statistically significant differences in editing efficiency compared with the original RERV‐RT‐m5 (Figure S7). Notably, previous research has shown that the composite promoter 35S‐CmYLCV‐U6 (35C) outperforms the OsU3 promoter [41, 42]. Consistently, our 35C‐epegRNA vectors exhibited improved PE efficiency at the *OsCDC48*‐T1, *OsEPSPS*‐T1 and *OsGAPDH* loci compared with those using the OsU3 promoter (Figure S8). Therefore, the 35C promoter was selected for pegRNA expression in subsequent plant vector designs.

**FIGURE 3:**
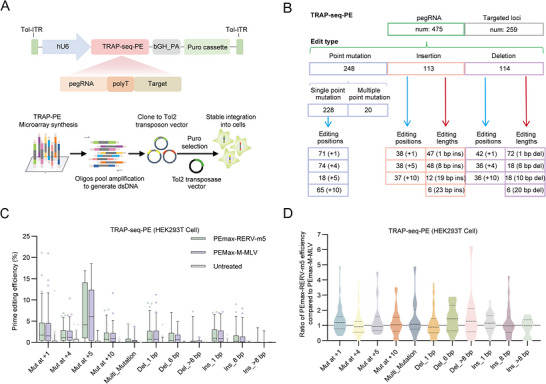
Evaluation of editing efficiency and properties of PEmax‐RERV‐RT‐m5 and PEmax‐M‐MLV‐RT using TRAP‐seq‐PE in HEK293T cell line. (A) Pipeline for the construction of pegRNA library and the workflow of cell line construction. (B) Schematic diagram of the TRAP‐seq‐PE library system, consisting of 475 constructs. (C) Comparison of editing efficiencies between PE‐M‐MLV and PE‐RERV‐m5 via TRAP‐seq‐PE analysis. (D) Summary of editing efficiency ratios: PE‐RERV‐m5 versus PE‐M‐MLV. Values were calculated based on the editing efficiency of each edit type (derived from TRAP‐seq‐PE analysis), with the efficiency of the M‐MLV‐RT‐based editor at each target locus normalized to 1. num: number; Mut: single point mutation; Multi: multiple point mutation; Del: deletion; Ins: insertion.

To systematically evaluate the editing properties of different prime editors, we designed 475 pegRNAs for various types of edits (Figure 3A). Specifically, these comprised: 228 pegRNAs for 12 types of single base substitutions at positions +1, +4, +5, and +10; 20 pegRNAs for multi‐nucleotide mutations within the +1 to +10 region; 113 pegRNAs for insertions of 1 bp, 8 bp, 19 bp, and 23 bp fragments at positions +1, +5, and +10; 114 pegRNAs for deletions of 1 bp, 6 bp, 10 bp, and 20 bp fragments at positions +1, +4, and +10 (Figure 3B).

We adopted the TRAP library construction method reported previously [43, 44] and established TRAP‐seq‐PE, which employs a library of 475 constructs using the Tol2 transposase‐mediated genomic integration system [45]. Each construct contained the aforementioned pegRNA (driven by a human U6 promoter) and its corresponding target sequence. Upon the addition of Tol2 transposase, these Tol2 constructs integrated into the genome; after resistance selection, stable integration of the pegRNA library was achieved in HEK293T cells (Figure 3A). We then transiently transfected plasmids encoding the RTs and quantified prime editing efficiency at all targeted sites via deep amplicon sequencing. We found that RERV‐RT‐m5 exhibited comparable editing efficiency to that of M‐MLV‐RT (Figure 3C,D). This highlights that further modification and optimization of RERV‐RT‐m5 hold great promise for broadening its application potential in prime editing.

### 2.3 Generation of Prime‐Edited Plants Using the PE‐RERV‐m5 System

**FIGURE 4:**
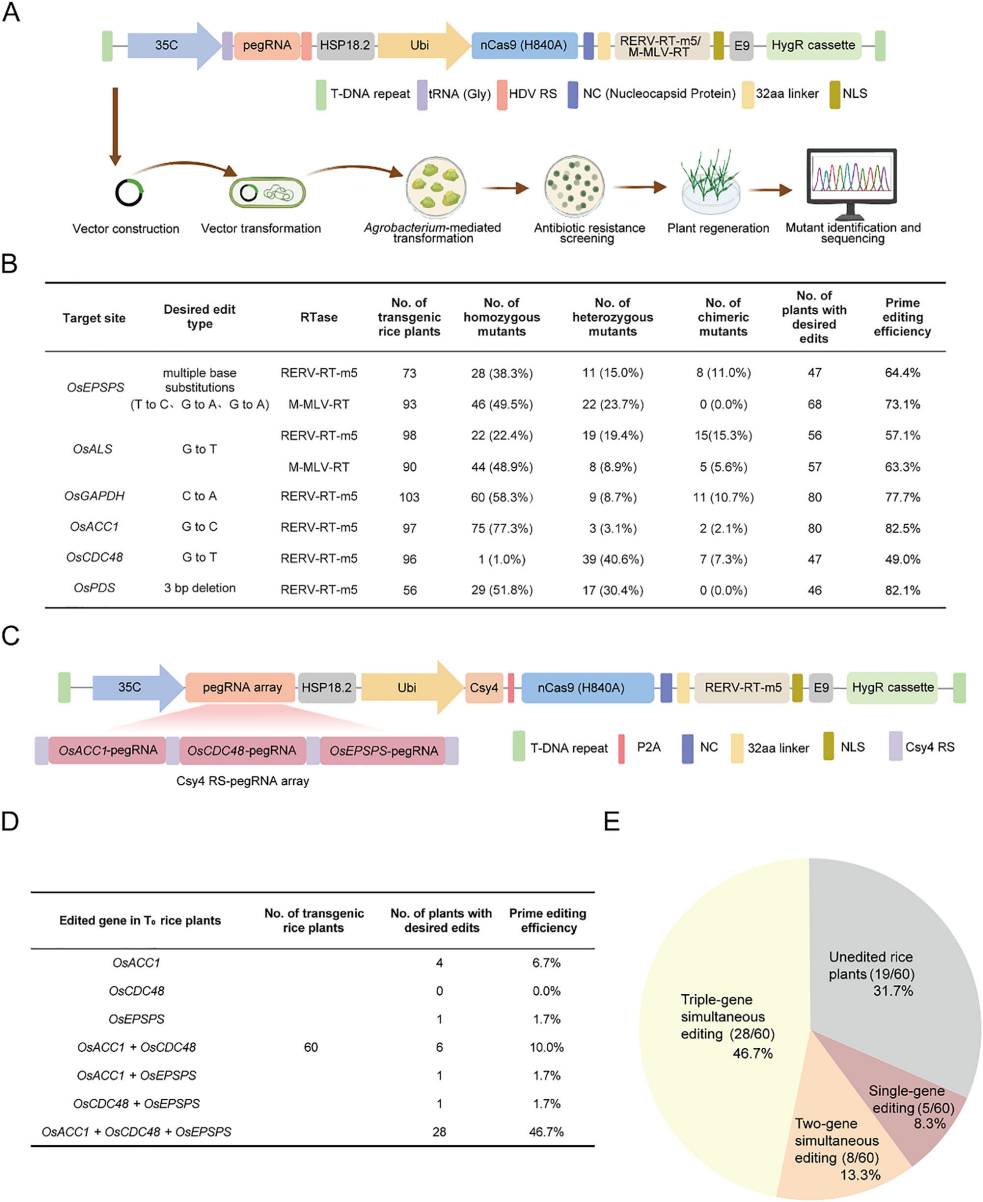
*Agrobacterium*‐mediated transformation of RERV‐RT‐m5 and M‐MLV‐RT‐based plant prime editors and plant regeneration. (A) Schematic diagram of the prime editing binary expression vectors pH‐35C‐RERV‐m5 and pH‐35C‐M‐MLV, along with the workflow of *Agrobacterium*‐mediated genetic transformation in rice and the corresponding transformation efficiency analysis. (B) Prime editing efficiency in regenerated rice plants. (C) Schematic diagram of the binary expression vector for multiplex gene prime editing binary expression vector. (D) Editing frequencies of each gene in multiplex prime editing among 60 regenerated rice plants. (E) Simultaneous editing events at multiple target loci in 60 regenerated rice plants.

To investigate the potential of RERV‐based prime editors in generating prime‐edited plants, we constructed the corresponding binary vector pH‐35C‐RERV‐m5. Six endogenous rice loci were tested: *OsACC1*, *OsPDS*, *OsALS*, *OsCDC48*, *OsGAPDH*, and *OsEPSPS*. These vectors were introduced into rice calli via *Agrobacterium*‐mediated transformation (Figure 4A). Sanger sequencing was performed to identify genotypes of regenerated plants.

The average editing efficiency of pH‐35C‐RERV‐m5 across the six endogenous loci was 68.8%, with the highest efficiency observed at the *OsACC1* locus (82.5%, 80 out of 97 plants). Compared with the efficiency of M‐MLV‐based PE (73.1%, 68 out of 93 at *OsEPSPS*; and 63.3%, 57 out of 90 at *OsALS*), the RERV‐m5‐based prime editor exhibited comparable PE efficiency (64.4%, 47 out of 73 at *OsEPSPS*; and 57.1%, 56 out of 98 at *OsALS*) (Figure 4B).

To evaluate the off‐target effects of PE‐RERV‐m5, we predicted potential off‐target sites using CRISPR‐OFFinder [46] and performed next‐generation sequencing (NGS) to detect such sites in the aforementioned regenerated rice plants. Notably, across all tested plants, no off‐target mutations were detected at 12 potential off‐target sites (Table S2), demonstrating the high specificity of PE‐RERV‐m5 prime editors in rice.

Meanwhile, we assessed the efficiency of PE‐RERV‐m5 in multiplex genome editing. We constructed a binary vector with a Csy4‐processed‐pegRNA array that simultaneously targets three endogenous loci: *OsACC1*, *OsCDC48*, and *OsEPSPS* (Figure 4C). This vector was then transformed into rice calli via *Agrobacterium*‐mediated transformation. Sanger sequencing of 60 regenerated rice seedlings revealed that 28 of them exhibited concurrent edits at all three loci, corresponding to a multiplex editing efficiency of 46.7% (Figures 4D,E and S9). These results demonstrated the potential of PE‐RERV‐m5 for multiple trait pyramiding.

### 2.4 Deep Mutational Scanning‐Driven Evolution of RERV‐RT to Enhance PE Efficiency

To further enhance the editing activity of RERV‐RT, we performed deep mutational scanning (DMS) for its engineering. We leveraged AlphaFold 3 to predict the structure of RERV‐RT, and identified key amino acids crucial for its interactions with substrate DNA in the reverse transcription domain. Based on the predicted structure of the RERV‑RT‑Cas9‑target DNA‑pegRNA complex, we pinpointed candidate amino acids located within 4 Å of the target DNA. By selecting regions encompassing these proximity‑defined amino acids and the aforementioned five beneficial mutations (Figure 2B), and considering mutant library size constraints and sequencing feasibility, we defined four core regions of RERV‐RT for DMS analysis (Figure S10).

**FIGURE 5:**
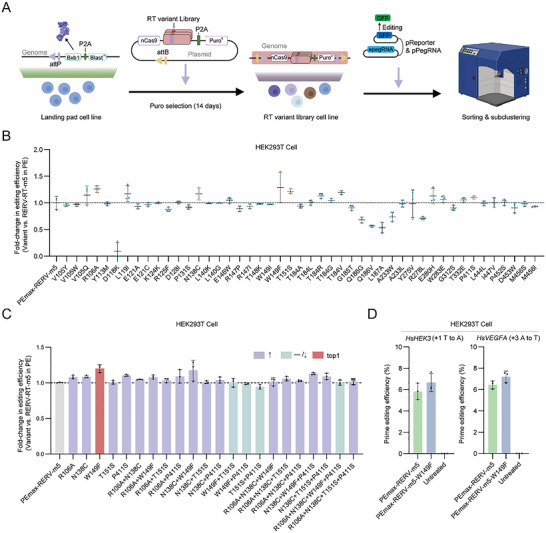
Deep mutational scanning (DMS) of RERV‐RT to improve its editing efficiency. (A) Schematic workflow of the DMS strategy employed for RERV‐RT engineering. (B) Functional validation of point mutations identified via DMS, using a BFP‑to‑GFP fluorescent reporter assay and flow cytometry analysis. Both blue and orange dots denote the point mutations identified in DMS, with orange dots indicating the high‐efficiency mutations selected for further combinatorial analysis. (C) Comparison of editing efficiencies between RERV‐RT‐m5 and its combinatorial variants from (B). RERV‐RT‐m5 serves as the reference control (gray bar). Purple bars indicate variants with enhanced efficiency relative to the reference, while cyan bars denote variants with comparable or reduced activity. The W149F mutation, which conferred the most substantial improvement in efficiency, is highlighted in red. (D) Endogenous gene editing efficiency of PE‐RERV‐m5 and PE‐RERV‐m5‐W149F in HEK293T cells. Data are presented as mean ± standard deviation from three independent biological replicates, with statistical significance determined by a two‐tailed Student's *t*‐test.

We constructed a vector library containing all 20 possible amino acid substitutions at each residue within the four core regions of RERV‐RT. Each PE‐RERV mutant was integrated into a specific locus by transfecting the landing pad cell line with BxbI integrase [47, 48] (Figure 5A,B). The transfected landing pad cell line was selected with puromycin and cultured, generating a cell line bearing the RERV‐mutant library. Next, our previously constructed BFP reporter plasmid was transfected into this cell line. Cells expressing green fluorescent protein (GFP), which indicated successful prime editing, were sorted and enriched by flow cytometry. Cells with different GFP fluorescence intensities were separately enriched and NGS was performed to validate mutations corresponding to each enriched cell population.

Using this strategy, cluster analysis and enrichment scoring of mutations within GFP‐stratified cell populations allowed us to identify mutations with the capacity to improve PE efficiency (Figure S10). We performed a common expression pattern analysis with k‐means clustering (k = 6 or 7). Based on GFP enrichment fold, enrichment trends across different clusters, and amino acid positional effects, we selected 47 high‐confidence candidates for subsequent experimental validation. Notably, five of the variants (R106A, N138C, W149F, T151S and P411S) were selected for further testing because they exhibited higher activity than RERV‐RT‐m5. Among them, the W149F variant (based on RERV‐RT‐m5) showed the most significant improvement in editing efficiency compared with PE‐RERV‐m5, with a 1.2‐fold increase in the reporter system (Figure 5B). We also tested combinations of these five variants. Unfortunately, combining multiple mutants did not lead to a further enhancement of editing efficiency (Figure 5C). We further compared the performance of PE‐RERV‐m5 with PE‐RERV‐m5‐W149F at two additional endogenous loci in HEK293T cells, and found that PE‐RERV‐m5‐W149F exhibited higher PE efficiency than PE‐RERV‐m5 (Figure 5D).

**FIGURE 6:**
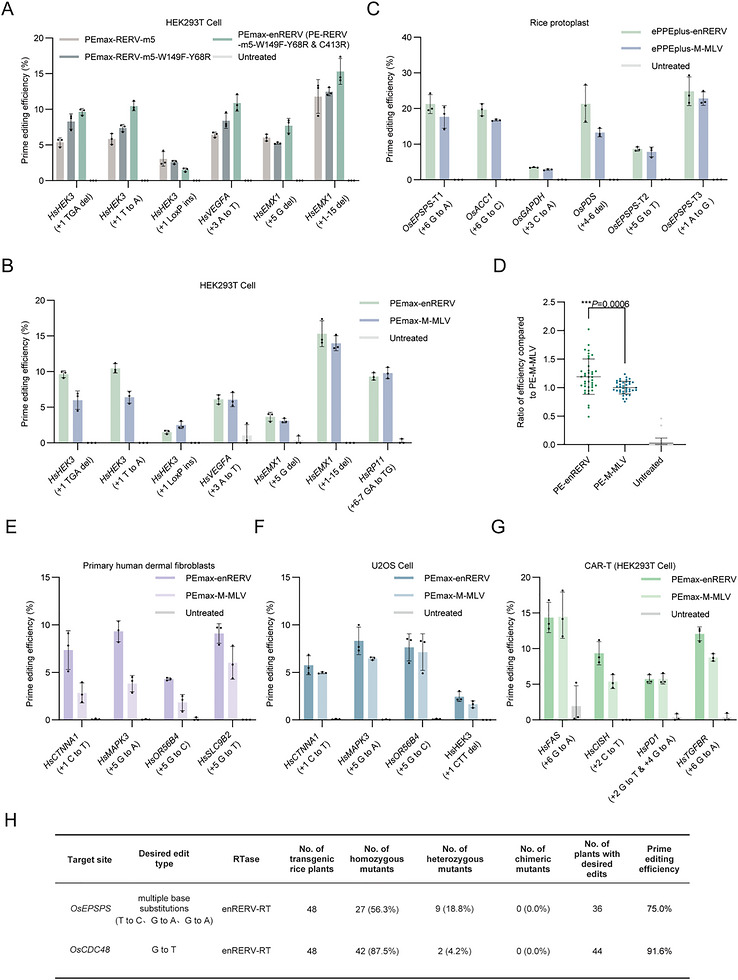
Prime editing in human and plant cells using the enRERV‐RT‐based prime editor. (A) Comparison of prime editing efficiencies among PE‐RERV‐m5, PE‐RERV‐m5‐W149F‐Y68R, and PE‐RERV‐W149F‐Y68R & C413R (PE‐enRERV) at six endogenous loci in HEK293T cells. (B) Comparison of prime editing efficiencies between PE‐enRERV and PE‐M‐MLV at seven endogenous loci in HEK293T cells. (C) Comparison of prime editing efficiencies between PE‐enRERV and PE‐M‐MLV at six endogenous loci in rice protoplasts. (D) Summary of editing efficiencies of PE‐enRERV and PE‐M‐MLV. The average editing efficiency of the M‐MLV‐RT vector at each endogenous site was normalized to 1. Statistical analysis was performed using a two‐tailed Student's *t*‐test. ^***^ *p* < 0.001. (E) Comparison of prime editing efficiencies between PEmax‐enRERV and PEmax‐M‐MLV at four endogenous loci in primary human dermal fibroblasts. (F) Comparison of prime editing efficiencies between PEmax‐enRERV and PEmax‐M‐MLV at four endogenous loci in U2OS cells. (G) Application of PEmax‐enRERV for gene silencing to enhance the durability and potency of CAR‐T cells. (H) Prime editing efficiency of ePPEplus‐enRERV at two endogenous loci in regenerated rice plants. Data are presented as means ± standard deviations from three independent biological replicates.

We further introduced two beneficial mutations, Y68R and C413R, that were recently identified in PERV‐RT [25] into RERV‐RT‐m5‐W149F, and found that these two mutations further enhanced prime editing efficiency (Figure 6A). This variant (RERV‐RT‐m5‐W149F‐Y68R&C413R) was designated enRERV‐RT. We evaluated its editing performance at 13 endogenous sites in total, in rice protoplasts and HEK293T cells (Figure 6B,C). Results showed that the enRERV‐RT‐based prime editor (PE‐enRERV) achieved a 1.17‐fold increase in editing efficiency across seven canonical target sites in HEK293T cells compared to PE‐M‐MLV (Figure 6B). A similar trend was observed across six endogenous sites in rice protoplasts, with a 1.22‐fold increase (Figure 6C). Collectively, PE‐enRERV exhibited an overall 1.20‐fold efficiency enhancement relative to PE‐M‐MLV (Figure 6D). Similar results were observed based on another newly reported La‐fused PE system, which is also known as “PE7” (Figure S11) [49].

We further tested the editing performance of PE‐enRERV and PE‐M‐MLV across an additional immortalized cell line (U2OS cell) and a primary cell line (primary human dermal fibroblasts). PE‐enRERV outperformed PE‐M‐MLV across eight endogenous sites in the two cell lines (Figure 6E,F). Especially in primary human dermal fibroblasts, PE‐enRERV yielded a better editing performance than PE‐M‐MLV across four tested loci (Figure 6E). These results suggested that enRERV‐RT maintains superior editing capability over M‐MLV‐RT even in primary cell types.

To compare byproducts levels between PE systems based on enRERV‐RT and M‐MLV‐RT, we systematically analyzed editing byproducts in the 17 validated editing events in plant and animal cells and observed no substantial differences between the two systems (Figures S12 and S13).

### 2.5 Application of PE‐enRERV for Enhancing CAR‐T Immunotherapies and Regenerating Prime‐Edited Plants

Previous studies have demonstrated that knockout of *HsPD‐1*, *HsFAS*, *HsCISH*, and *HsTGFBR* enhances the durability and potency of CAR‐T cells [50, 51, 52]. These four genes are key regulatory targets associated with immune checkpoints, the tumor microenvironment and graft‐versus‐host disease. Multiplexed targeting of these genes endows CAR‐T cells with superior persistence, anti‐tumor potency, and resistance to the tumor microenvironment [52, 53, 54, 55]. To explore the therapeutic potential of PE‐enRERV (which does not induce double‐strand breaks (DSBs) [5, 56]) in cancer immunotherapy, we evaluated its genome editing efficiency in CAR‐T cell‐relevant genes. We constructed prime editing vectors to introduce stop codons into each of the four genes: *HsPD‐1* (R94*), *HsFAS* (W5*), *HsCISH* (Q6*), and *HsTGFBR* (W249*). Our results showed that PE‐enRERV achieved efficient editing at all target sites, highlighting its potential for advancing next‐generation CAR‐T immunotherapies (Figure 6G).

To investigate the potential of PE‐enRERV for generating prime edited plants, we evaluated editing at two endogenous rice loci, *OsEPSPS* and *OsCDC48*. pH‐35C‐enRERV exhibited higher editing efficiencies than pH‐35C‐RERV‐m5 at both loci (Figures 4B and 6H). These results demonstrated the effective editing activity and broad application potential of enRERV‐RT in the regeneration of prime‐edited plant.

### 2.6 PE‐enRERV Outperforms PE‐M‐MLV in Hard‐to‐Edit Prime Editing Events

**FIGURE 7:**
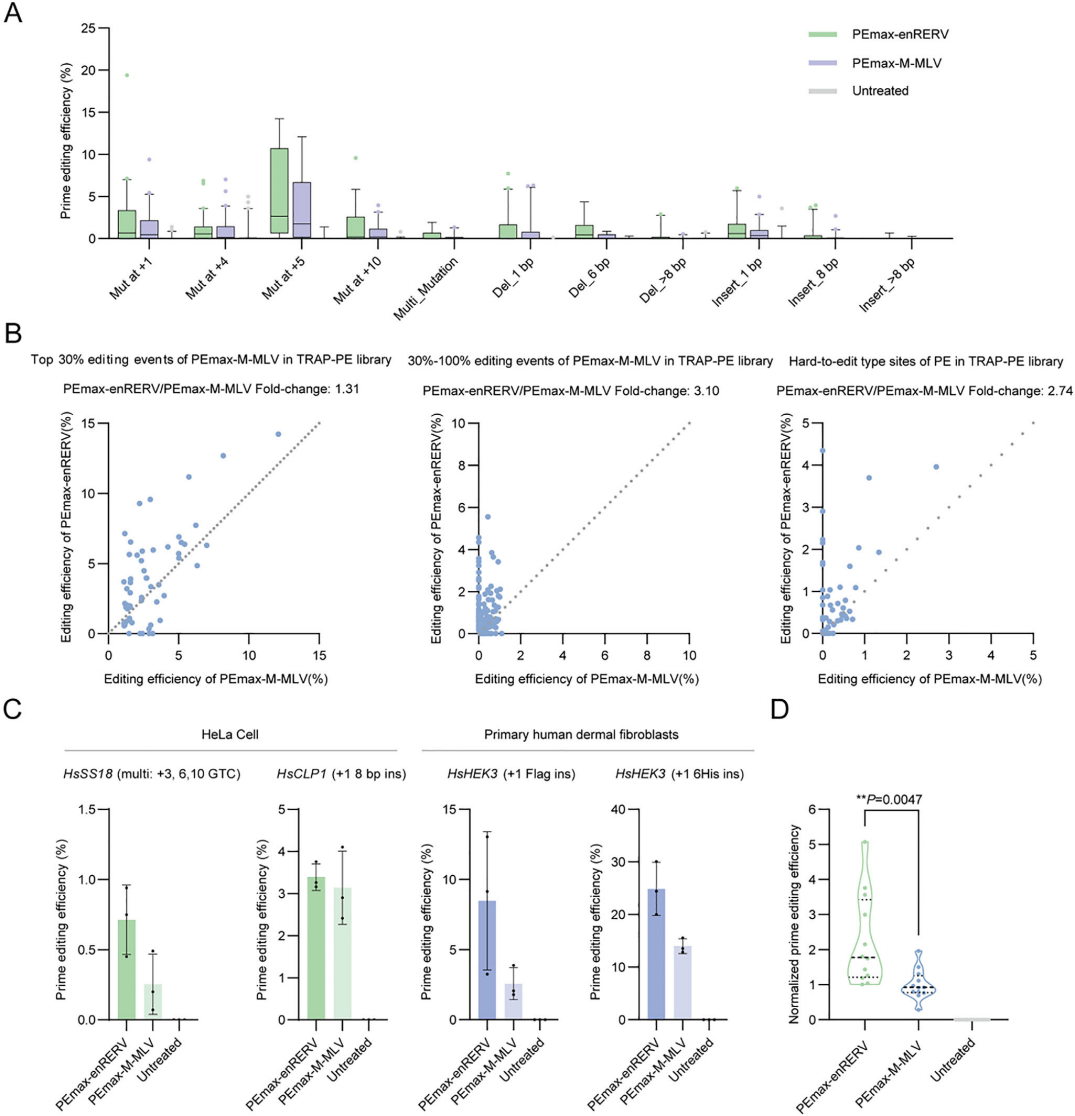
PE‐enRERV outperforms PE‐M‐MLV in hard‐to‐edit prime editing events.(A) Comparison of editing properties of PEmax‐enRERV and PEmax‐M‐MLV via TRAP‐seq‐PE. (B) Stratified analysis of editing efficiency between PE‐enRERV and PE‐M‐MLV using TRAP‐seq‐PE. (C) Comparison of prime editing efficiencies between PE‐enRERV and PE‐M‐MLV at four endogenous loci in HeLa and Primary human dermal fibroblasts. (D) Overall editing efficiency between PE‐enRERV and PE‐M‐MLV across the four endogenous target sites shown in (C). The average editing efficiency of PE‐M‐MLV at each endogenous site was normalized to 1. Statistical analysis was performed using a two‐tailed Student's *t*‐test. ^***^ *p* < 0.001.

We further systematically compared the editing performance of PE‐enRERV and PE‐M‐MLV using TRAP‐seq‐PE. On average, PE‐enRERV exhibited a 1.8‐fold higher editing efficiency than PE‐M‐MLV (Figure 7A). Stratified analysis of the library data showed that even among the top 30% most efficiently edited loci by PE‐M‐MLV, PE‐enRERV still achieved an approximately 1.3‐fold efficiency improvement. Notably, at hard‐to‐edit targets, PE‐enRERV exhibited a ∼2.74‐fold enhancement compared with PE‐M‐MLV (Figure 7B). Together, these results indicate that PE‐enRERV is particularly advantageous for challenging prime editing events compared with routine edits.

We then compared the performance of PE‐enRERV and PE‐M‐MLV in challenging edits across HeLa cells and primary human dermal fibroblasts. PE‐enRERV outperformed PE‐M‐MLV by an average of 1.88‐fold at all four tested loci, and showed a particularly strong advantage for the targeted insertion of two long fragments (a 24‐bp Flag tag and an 18‐bp 6×His tag) in fibroblasts (Figure 7C,D). These results underscore the robustness of PE‐enRERV in diverse cell types and its exceptional ability to edit challenging genomic regions, supporting its broad potential for efficient and versatile genome editing.

## 3 Discussion

Prime editors enable the generation of all types of base conversions, as well as small insertions and deletions, and exhibit greater safety in gene therapy compared with nuclease‐based CRISPR technologies (which induce double‐strand breaks, DSBs) and base editors (which may cause genome‐wide off‐target effects) [5, 57, 58, 59, 60, 61, 62]. However, achieving high prime editing efficiency across diverse species and various edit types remains a challenge in the field.

Previous studies (from 2019 to the present) have reported approximately 50 active RT enzymes, which are primarily of viral origin [13, 25, 26, 27]. However, the Pfam database phylogenetic distribution analysis revealed that the RVT domain is widely conserved across taxa: 53.8% (421 713 sequences) originate from viruses, 42.5% (332 705 sequences) from 3618 eukaryotic species, and 3.7% from bacteria. This broad taxonomic distribution highlights RT enzymes as an untapped, rich resource for expanding prime editing capabilities. Among the 558 unique RT sequence we screened, a substantial proportion were mined from eukaryotes and bacteria (216/558 and 261/558, respectively). Notably, functionally active RTs identified to date—including the rat‐derived RERV‐RT, yeast‐derived RT (Tf1‐RT) [26], and the previously characterized porcine endogenous retrovirus RT (PERV‐RT) [25]—underscore the immense potential of these species‐specific enzymes (numbering in the tens of thousands) for PE optimization. Importantly, leveraging endogenous RTs from target species may mitigate immune responses during editing, which will address a critical barrier to the clinical translation of PE technologies.

To ensure phylogenetic diversity, 54 RT candidates were selected from the 558 unique RT sequences, with at least three representatives from each of the seven clades in the phylogenetic tree. From these, 19 active RTs were confirmed via an established BFP‐to‐GFP reporter system in rice protoplasts. Among these 19 active RTs, RERV‐RT exhibited the highest PE activity, but it required further engineering for diverse applications. To ensure cross‐species utility (in both plants and mammals), we performed parallel optimization in both systems. Building on our previous work on deaminase structure‐function relationships [44]—which showed that functionally conserved proteins often share structural similarity—AlphaFold 3‐predicted models and structural alignments revealed high similarity among RERV‐RT, M‐MLV‐RT [5] and PERV‐RT [25]. We therefore hypothesized that efficiency‐enhancing mutations from M‐MLV‐RT and PERV‐RT could be applied to RERV‐RT, a strategy that significantly improved its activity. Additionally, deep mutational scanning (DMS) of four key regions in RERV‐RT identified additional efficiency‐enhancing mutations.

Given the lack of a comprehensive evaluation system for PE editing characteristics, we established the TRAP‐seq‐PE assay by drawing on our experience with TRAP‐seq in base editing [41]. We applied this assay with over 470 pegRNAs covering all major editing types to evaluate PE performance. Using this system, we found that enRERV‐RT and M‐MLV‐RT exhibited consistent editing patterns across various edit types, including single‐base substitutions, multi‐nucleotide mutations, and fragment insertions/deletions. Notably, enRERV‐RT displayed enhanced editing efficiency, demonstrating that it is a well‐suited, precise, and promising alternative tool for diverse prime editing applications. This assay comprehensively assessed all editing characteristics achievable by current PE systems and enables rapid, quantitative comparison of PE variants while providing a comprehensive view of editing capabilities, thus accelerating optimal PE selection for specific genomic contexts and editing goals.

While optimized PE‐enRERV delivers only ∼20% higher efficiency than PE‐M‐MLV for canonical target events, TRAP‐seq analysis reveals a greater performance advantage at hard‐to‐edit genomic targets, which is further validated in challenging endogenous edits in primary human dermal fibroblasts and HeLa cells. These results indicate that enRERV‐RT is a promising candidate for further optimization and modification for prime editing applications. Going forward, strategies such as AI‐driven protein design and directed evolution could further boost the performance of enRERV‐RT, as advanced AI models enable rapid protein optimization by fine‐tuning binding specificity and enhancing stability [63, 64, 65]. Additionally, directed evolution constitutes a versatile and powerful approach for optimizing target protein properties, enabled by the construction of diverse gene libraries and efficient screening platforms [66, 67, 68]. For instance, PACE and PANCE are two representative systems employed for genome editor optimization [69].

To date, numerous beneficial mutations in M‐MLV‐RT (e.g., V223M and L435K in PEmax**; Q291I and D339E in rPE) have been continuously identified. Such mutations could provide valuable candidate references and rational design clues for further optimizing the enzymatic activity and editing efficiency of enRERV‐RT. Moreover, as previously reported, other RT‐based prime editors such as evo‐Ec48‐RT‐derived PE6a and the engineered, evolved Tf1‐RT‐derived PE6c each exhibit distinct preferences of editing types, which highlights that the performance of enRERV‐RT across diverse scenarios is worthy of further in‐depth evaluation.

The efficiency difference between TRAP‐PE library and endogenous sites was observed during this study. We speculate that the observed discrepancy is primarily driven by two key factors: First, the discrepancy arises from the distinct delivery and expression modalities of the prime editor across different assays. Our TRAP‐seq assay uses transposon‐mediated stable integration to achieve sustained long‐term editing, while the validation at endogenous genomic loci employs transient transfection for acute short‐term editing. These two modalities have fundamentally distinct editing kinetics and editor expression profiles, leading to the observed difference in the magnitude of efficiency enhancement. Second, this discrepancy may be attributed to differences in chromatin status and cellular microenvironment. We hypothesize that the integrated TRAP‐PE library may lack strong epigenetic modifications, resulting in higher editing efficiency relative to endogenous loci and thereby causing the observed differences in editing performance. Nevertheless, our results demonstrate that the TRAP‐PE library exhibits consistent efficiency trends with endogenous target sites, and this approach can still accurately and objectively evaluate the functional improvements of distinct prime editing systems.

Beyond optimizing core components (RT and Cas enzymes), engineering pegRNAs, refining prime editor architecture, and modulating cellular DNA repair pathways have also proven effective for improving PE outcomes [1]. Collectively, integrating a comprehensive PE properties evaluation system with diverse optimization strategies to enhance PE performance will further broaden its applications in therapeutic and agricultural breeding scenarios.

## 4 Conclusion

In summary, this study identified and engineered a novel reverse transcriptase (RERV‐RT) from *Rattus norvegicus*, addressing the scarcity of efficient RT variants for CRISPR‐based prime editing. Multi‐layered engineering of RERV‐RT yielded enRERV‐RT, which achieved a 1.20‐fold editing improvement over M‐MLV‐based editors in mammalian cells and rice, and a 1.88‐fold improvement in hard‐to‐edit events. Functional validation confirmed PE‐enRERV's superior properties, including high specificity, efficient multiplex editing, and potent editing of CAR‐T regulatory genes, highlighting its cross‐species versatility in both biomedical and agricultural applications. Collectively, our work establishes a high‐efficiency PE platform based on enRERV‐RT, expanding the RT toolkit for prime editing in gene therapy, crop breeding, and basic research.

## 5 Methods

### 5.1 Mammalian Cell Lines Culture Conditions

HEK293T cells, U2OS cells, HeLa cells and primary human dermal fibroblasts were cultured in Dulbecco's Modified Eagle's medium (DMEM, Basalmedia) supplemented with 10% (vol/vol) fetal bovine serum (FBS, Sigma‐Aldrich) and 1% (vol/vol) Penicillin‐Streptomycin (Gibco) in a humidified incubator at 37°C with 5% CO_2_. Cells were plated on 75 cm^2^ Cell Culture Flask (NEST).

### 5.2 Rice Protoplast Isolation and Transformation

We used the Japonica rice (*Oryza sativa*) variety Zhonghua11 for protoplast preparation. Protoplast isolation and transformation were performed as described previously [70]. Plasmids (5 µg per construct) were transformed into protoplasts by PEG‐4000 (Sigma). The transformed protoplasts were normally incubated at 26°C for 48 h for fluorescence detection and 72 h for DNA extraction.

Fluorescence imaging of GFP‐expressing cells was performed using an inverted fluorescence microscope (Mshot, Guangzhou). For documentation and analysis, raw image data were recorded and processed using the microscope's proprietary software (Mshot image analysis system) with consistent acquisition parameters maintained across all samples. Image processing was performed using Photoshop software.

For DNA extraction, the transformed protoplasts were harvested by centrifugation, and genomic DNA was subsequently extracted using a CTAB (cetyltrimethylammonium bromide) with chloroform extraction method [71].

### 5.3 Protein Clustering and Analyzing

Protein sequences were searched and downloaded by BLAST (https://blast.ncbi.nlm.nih.gov/Blast.cgi) in the NCBI database, with criteria of 40%–90% sequence identity to the seed sequence and a length of 220–700 amino acids. We used CD‐HIT to reduce redundant sequences with a threshold of 90% sequence identity and 90% coverage. The 40%–90% sequence similarity range was set to achieve two key screening objectives: first, to filter out sequences with <40% similarity to reference RTs, which lack sufficient conservation of the core reverse transcriptase catalytic domain and are highly unlikely to retain functional reverse transcription activity; second, to exclude sequences with excessive homology to well‐characterized RTs, while reducing the complexity of the subsequent experimental screening workflow. We did exclude sequences with >90% similarity to commonly used, well‐studied RTs, as we reasoned that variants sharing over 90% sequence identity with these reference RTs would be highly unlikely to exhibit substantial differences in core protein structure and catalytic editing efficiency. Then, the CD‐Search was utilized to annotate RT domains to reduce the accumulation of unrelated information (https://www.ncbi.nlm.nih.gov/Structure/cdd) [72]. Finally, 558 RT candidates were selected and listed in Table S1 for downstream phylogenetic analyses.

The Clustal Omega tool [73] (https://www.ebi.ac.uk/jdispatcher/msa/clustal) was used to generate a multiple sequence alignment of all 558 RT sequences, and an unrooted tree was generated using the neighbor‐joining tree build method with 1000 Bootstrap. iTOL [74] (https://itol.embl.de/) was used for visualization. We randomly chose several proteins from each branch, and total of 54 new RT enzymes were mined for efficiency test (Table S1). The protein structure model was predicted by AlphaFold 3 and visualized in PyMOL.

### 5.4 Removal of Redundant Sequences From Reverse Transcriptase Genes and Their Synthesis

To obtain functional gene constructs, selected RT candidates were firstly subjected to domain annotation (https://www.novopro.cn/). Then the extra N‐ and C‐terminal sequences were removed and the remaining sequences were codon‐optimized specifically for expression in either plants (rice and wheat) or mammals (human and mouse) and synthesized by GenScript.

### 5.5 Structure Prediction and Plasmid Construction

The atomic models for the protein and its ternary complex with target DNA and pegRNA were generated using AlphaFold 3 (https://alphafoldserver.com) [30]. The top‐predicted model, selected based on the highest confidence score (pTM), was subjected to structural analysis. Visualization, structural alignment with reference coordinates, annotation of functional sites, and figure preparation were carried out using the PyMOL software suite.

The GFP reporter system we previously constructed comprises two plasmids: one expressing the BFP protein, and the other containing a pegRNA that targets the 66th amino acid of BFP to induce the H66T mutation [13]. All new RTs in this study and pTol5‐hU6‐pegRNA‐puro‐Tol3 vector were commercially synthesized and cloned by GenScript. For plant PE vectors (maize ubiquitin‐1 promoter‐driven PEs), synthesized RT were cloned into ePPEplus vector, yielding vectors with Ubi‐1::NLS‐nCas9(H840A)‐XTEN‐NCprotein‐NLS‐32aaLinker‐RT‐NLS::CaMV‐T expression cassettes. For PE vectors used in mammalian cells (CMV promoter‐driven PEs), synthesized RT were cloned into pCMV‐PEmax (Addgene #174820), yielding vectors with CMV::NLS‐nCas9(H840A)‐NLS‐Linker‐RT‐NLS::bGH expression cassettes. The plant pegRNA and epegRNA vectors (rice U3 or 35C promoter drives pegRNA) were constructed as reported previously using the pOsU3‐esgRNA (Addgene #115629) backbone or p35C‐esgRNA (a gift from Professor Qi‐jun Chen of China Agricultural University) backbone [41, 42], and the resulting recombinant plasmids were designated pOsU3‐epegRNA and p35C‐epegRNA. For plant‐targeted pegRNA constructs used in this study, PCR‐amplified pegRNA fragments were seamlessly cloned into the *Bsa*I/*Sac*II‐digested pOsU3‐epegRNA backbone and *Bsa*I‐linearized p35C‐epegRNA backbone, respectively. The human pegRNA or epegRNA vectors (human U6 promoter drives pegRNA), were constructed as reported previously using the phU6‐gRNA (developed by replacing the OsU3 promoter of the pOsU3‐esgRNA vector with the U6 promoter) backbone, yielding the recombinant plasmid phU6‐epegRNA. To generate pegRNA vectors targeting endogenous loci in human cells, PCR‐amplified pegRNA fragments were seamlessly inserted into the phU6‐epegRNA backbone pre‐digested with *Bsa*I and *Sac*II. To construct the Agrobacterium‐mediated rice transformation binary vector, we chemically synthesized a DNA expression cassette: 35C::tGly‐epegRNA (*OsCDC48*) ‐HDV::HSP18.2‐Ubi‐1::NLS‐nCas9(H840A)‐XTEN‐NCprotein‐NLS‐32aaLinker‐RERV‐NLS::E9‐T. This synthetic fragment was seamlessly inserted into the vector pH‐ePPE (Addgene #183097) to yield the recombinant plasmid pH‐35C‐RERV‐OsCDC48. For binary vectors targeting alternative genomic loci, construction was performed as follows. First, PCR‐amplified pegRNA fragments were seamlessly assembled into the *Bsa*I‐digested p35C‐epegRNA backbone to generate an intermediate plasmid. Next, the pegRNA‐containing target fragment was amplified from this intermediate construct using the primer pair pegF (gcggcgaagtattcaggcac) and pegR (caggcatgcagattacgcca). The resulting PCR product was finally seamlessly cloned into the pH‐35C‐RERV backbone linearized by double digestion with *Pml*I and *Hind*III. For multi‐gene editing binary transformation vectors, a synthetic DNA fragment 35C::Csy4 RS‐pegRNA array::HSP18.2‐Ubi‐1::Csy4 was synthesized. This synthetic sequence was seamlessly cloned into the pH‐35C‐RERV‐OsCDC48 backbone, and the recombinant vector pH‐35C‐Csy4‐RERV was obtained.

All plasmids for human cell line transfection were extracted with the Endo‐free Plasmid Purification Kit II (Tiangen). All plasmids for plant protoplasts transformation were extracted with the Wizard Plus Midipreps DNA Purification System (Promega). All plasmids for *Agrobacterium*‐mediated transformation were extracted using the Wizard Plus Midipreps DNA Purification System (Promega). All primer sets used for plasmid construction in this work were synthesized by Sangon, Guangzhou. All seamless cloning assembly reactions during vector construction were performed using 2 × MultiF Seamless Assembly Mix (ABclonal). The core plasmids constructed in this study, including ePPEplus‐enRERV (Addgene #258134, WeKwikGene #0002690), PEmax‐enRERV (Addgene #258135, WeKwikGene #0002691), pH‐35C‐enRERV‐OsCDC48 (Addgene #258136, WeKwikGene #0002692), p35C‐epegRNA (Addgene #259452, WeKwikGene #0002760), and phU6‐epegRNA (Addgene #259451, WeKwikGene #0002759) have been deposited in the WeKwikGene repository of Westlake University and Addgene.

### 5.6 Cell Transfection for Endogenous Sites Editing

For HEK293T cells, U2OS cells and HeLa cells, approximately 5 × 10^4^ cells were seeded into 48‐well plates. Upon reaching 60%–70% confluency, 750 ng of PE editor plasmid, 250 ng of pegRNA plasmid, 0.5 µL of Lipo8000 (Beyotime) were mixed and filled with Optimem (ThermoFisher) to 25 µL for each well.

For primary human dermal fibroblasts (HDF), approximately 5 × 10^4^ cells were seeded into 48‐well plates. Upon reaching 60%–70% confluency, 750 ng of PE editor plasmid, 250 ng of pegRNA plasmid, 1 µL of Lipofectamine LTX (ThermoFisher) and 1 µL PLUS reagent were mixed and filled with Optimem (ThermoFisher) to 100 µL for each well.

For DNA extraction, genomic DNA was extracted with Lysis Buffer (Genesand) and Proteinase K (Genesand) following the lysis kit protocol. For protoplasts, genomic DNA was extracted with the CTAB method after 72 h incubation [71]. All DNA samples were quantified with a NanoDrop 2000 spectrophotometer (Thermo Scientific). Specific primers were used for mutant identification (amplification and sequencing of target sites).

### 5.7 DMS Library Construction

First, we cultured the landing pad cell line (a gift from Xiaoyan Jia's lab of Fudan University), which contains an attP site and expression cassettes for BxbI integrase and blasticidin resistance. In parallel, an oligonucleotide library encoding the RERV‐RT mutants was synthesized by Beijing Genomics Institute (BGI) using massively parallel synthesis. The library coverage was verified by next‐generation sequencing (NGS) to ensure it was approximately 100%. These oligonucleotides were then cloned into an attB‐containing vector using 2 × MultiF Seamless Assembly Mix (ABclonal). The landing pad cells were induced with 2 µg/mL doxycycline for 3 days. For each pool, 5 × 10^6^ cells were plated in a 100 mm dish and transfected with 15 µg of the RERV‐RT mutant library plasmid DNA using Lipo8000 transfection reagent. After 72 h, transfected cells were selected with 1 µg/mL puromycin, which was maintained thereafter. Following a 14‐days selection, the cells were transfected with a total of 12 µg plasmid DNA at a 1:1 ratio of BFP to BFP‐pegRNA, again using Lipo8000. After 72 h, the cells were detached with trypsin, resuspended in PBS, and subjected to fluorescence‐activated cell sorting (FACS; BD Aria III) to isolate GFP‐positive cells.

Genomic DNA was extracted from both pre‐sorted and sorted GFP‐positive populations. The RERV‐RT mutation region was amplified by targeted PCR and analyzed by NGS to identify the mutants enriched in the GFP‐positive cells. The analysis of mutation clusters for determining fluorescence enhancement trends referenced co‐expression analysis methods; see details in the R package ‘cluster’: https://cran.r‐project.org/web/packages/cluster/index.html, and amino acid positional effects. All primers used are listed in Table S3.

### 5.8 TRAP‐seq‐PE Library

To evaluate the editing characteristics of the novel reverse transcriptase mined in this study, we synthesized 475 pegRNAs, along with their corresponding target sequences and their flanking genomic context. These pegRNA‐target sequence pairs were tandemly linked to form library constructs. All pegRNAs were designed with a 13 bp PBS sequence, while RTT lengths remained consistent for the same category of edits. PegRNAs designed for the same type of edit were directed against different target sites. Following exclusion of constructs with design or synthesis errors, the final library comprised 475 valid constructs for subsequent analysis. The 475 pegRNA fragments above were integrated into the pTol2‐hU6‐ccdB‐puro using 2 × MultiF Seamless Assembly Mix (ABclonal), to construct the TRAP library plasmid. Then, 5 × 10^6^ HEK293T cells were plated in a 10 cm dish and transfected with 15 µg plasmid (1.0:1.0 /TRAP library plasmid: Tol2 Transposase plasmid) and 24 µL Lipo8000 in 750 µL OptiMEM. After 72 h, transfected cells were selected with1 µg/mL puromycin for 14 days. NGS sequencing was used to ensure each pegRNA was integrated into the plasmid and cell line.

We evaluated the efficiency of PEs using the pegRNA TRAP‐seq library cell line. Briefly, 5 × 10^6^ cells were plated in a 100 mm dish 24 h before PE transfection. For transfections, 15 µg of PE plasmid DNA and 24 µL Lipo8000 were mixed in 750 µL OptiMEM. After 72 h, cells were washed with PBS and DNA was extracted using StarSpin Universal DNA Kit (GenStar). The extracted DNA was then amplified and subjected to NGS to assess of prime editing efficiency.

### 5.9 TRAP‐seq‐PE Library Sequencing and Bioinformatic Analysis

Raw paired‐end sequencing reads were merged using FLASH to generate full‐length amplicon sequences. The resulting merged reads were processed through a custom Python pipeline (https://github.com/BaitaoU‐U/PE‐TRAP‐library.git) to identify constructs and quantify editing efficiencies.

Reads were oriented and aligned based on the presence of the pegRNA scaffold. Each read was assigned to its specific pegRNA‐target construct within the TRAP‐seq‐PE library using the spacer and PBS‐RTT sequences as unique molecular identifiers. To precisely define the editing window, the target region was extracted using a spatial anchor sequence located upstream of the target site.

The extracted target sequences were compared against the designed wild‐type (WT) and intended edited reference sequences. To ensure statistical robustness, only target sites with a total valid read depth of 50 were included in the downstream analysis. Prime editing efficiency was calculated as the percentage of intended edited reads relative to the total number of valid reads at each site.

### 5.10 Flow Cytometry Analysis

To assess the editing efficiency of mutants with fluorescent reporter systems, cells were harvested 72 h post‐transfection, resuspended in PBS, and analyzed on a CytoFLEX flow cytometer (Beckman Coulter Life Sciences). Data were collected and analyzed using CytExpert software (version 2.5.0.77).

### 5.11 Amplicon Deep Sequencing and Data Analysis

Genomic DNA was extracted from three independent replicates of each target locus, and amplicon sequencing was performed on each replicate. 2 × KeyPo SE Master Mix (Dye Plus) (Vazyme) was used for amplification of the target sequence.

A nested PCR strategy was employed. In the first round, the target region was amplified from genomic DNA using site‐specific primers. In the second round, both forward and reverse barcodes containing sequencing adapters were added to the amplicon ends for library construction. Equal amounts of PCR products were pooled and sequenced commercially (Illumina Novaseq X plus; Genewiz, Suzhou). Analysis of prime‐editing processivity and indels were performed as described previously, and the analysis pipeline for sequencing data followed previously described methods [75]. The workflow for NovaSeq data is available on GitHub (https://github.com/ReiGao/GEanalysis/tree/master/Scripts), and the code for processing MiSeq data has also been shared (https://github.com/ReiGao/Miseq_BEanalysis).

All primers used for amplicon deep sequencing in this work are listed in Table S3 and were synthesized by Sangon, Shanghai.

### 5.12 *Agrobacterium*‐Mediated Transformation of Rice Calli

*Agrobacterium* vector and the *Agrobacterium* strain EHA105 were used in this study. Rice calli (Zhonghua 11) were induced from mature embryos, and the plant was screened for resistance to hygromycin (Sangon). The callus transformation was performed by Wuhan Boyuan Biotechnology Co., LTD. The calli were grown in the medium for 3–4 weeks for obtaining regenerated plants. The leaf samples from each plant were used for DNA extraction and genotyping.

### 5.13 Prediction of off‐target Edits

The off‐target sites were predicted with CRISPR‐OFFinder. The maximum mismatch was set at three. All primers used for off‐target sites’ identification in this work are listed in Table S3 and were synthesized by Sangon, Shanghai.

### 5.14 Quantification and Statistical Analysis

All data were presented as mean ± standard deviation (SD). Statistical significance between two groups was calculated using independent two‐tailed student's *t*‐test with GraphPad Prism. *p* < 0.05 was considered statistically significant, *p* < 0.01 was considered highly statistically significant and *p* < 0.001 was considered extremely statistically significant.

## Author Contributions

**Linsha Ma**, **Pengcheng Yao**, **Shengqi Wu**, **Yuanyuan Shi**, **Lang Qin**, and **Baitao Li**: Conceptualization, Investigation, Formal analysis, Validation, Writing – Original draft. **Jiayi Zhu**, **Minhua Huang**, **Yichong Zhu**, **Yuwen Song**, **Jinhuan Pang**, **Ziping Guo** and **Guochuan Wu**: Methodology, Software, Formal analysis. **Chen Wang**: Resources. **Kewei Xu**: Writing and Editing. **Ruihua Huang**, **Quan Kuang**, **Liang Qu**, **Changtian Pan** and **Xianrong Xie**: Supervision. **Qinlong Zhu**: Supervision, Project administration. **Jiaying Huang** and **Qiupeng Lin**: Writing – Review and Editing, Supervision, Project administration, Funding acquisition.

## Funding

This work was supported by the National Key R&D Program of China (2024YFC3408200, 2024YFF1000800), the STI 2030—Major Projects (2023ZD04074), the Frontier Technology Program of Jiangxi Provincial Natural Science Foundation (20253BAC260005), the Invigorate the Seed Industry of Guangdong Province (2023‐NJS‐00‐012), the National Natural Science Foundation of China (32422050, 32401250), the Young Elite Scientists Sponsorship Program of the China Association for Science and Technology, the Guangdong Provincial “Pearl River Talent Program” Innovation and Entrepreneurship Team Project (2023ZT10N019), the Key R&D Program of Guangxi Province (GKN AB24153006), Science and Technology Projects in Guangzhou (2025A04J7124, 2025A04J3669) and a specific university discipline construction project (2023B10564004).

## Conflicts of Interest

The authors have submitted two patent applications based on the results reported in this paper.

## Supporting information




**Supporting File 1**: advs75888‐sup‐0001‐DataS1.docx.

**Supporting File 2**: advs75888‐sup‐0002‐DataS2.pdf.

**Supporting File 3**: advs75888‐sup‐0003‐FigureS1.pdf.

**Supporting File 4**: advs75888‐sup‐0004‐TableS1.pdf.

**Supporting File 5**: advs75888‐sup‐0005‐TableS2.docx.

**Supporting File 6**: advs75888‐sup‐0006‐TableS3.docx.

**Supporting File 7**: advs75888‐sup‐0007‐TableS4.xlsx.

**Supporting File 8**: advs75888‐sup‐0008‐TableS5.xlsx.

## Data Availability

All data supporting the findings of this study are available in the article and its supplementary figures and tables. The NGS data can be accessed in the PRJCA057472.
