# Supplementary material for: Discovery and Engineering of a Rat Endogenous Retrovirus Reverse Transcriptase for Efficient Prime Editing
Source: Adv Sci (Weinh). 2026 Jun 26;13(49):e75888. doi: 10.1002/advs.75888 (PMC13335913; doi:10.1002/advs.75888)
Supplement: Supplementary file 2 [file ADVS-13-e75888-s005.pdf]

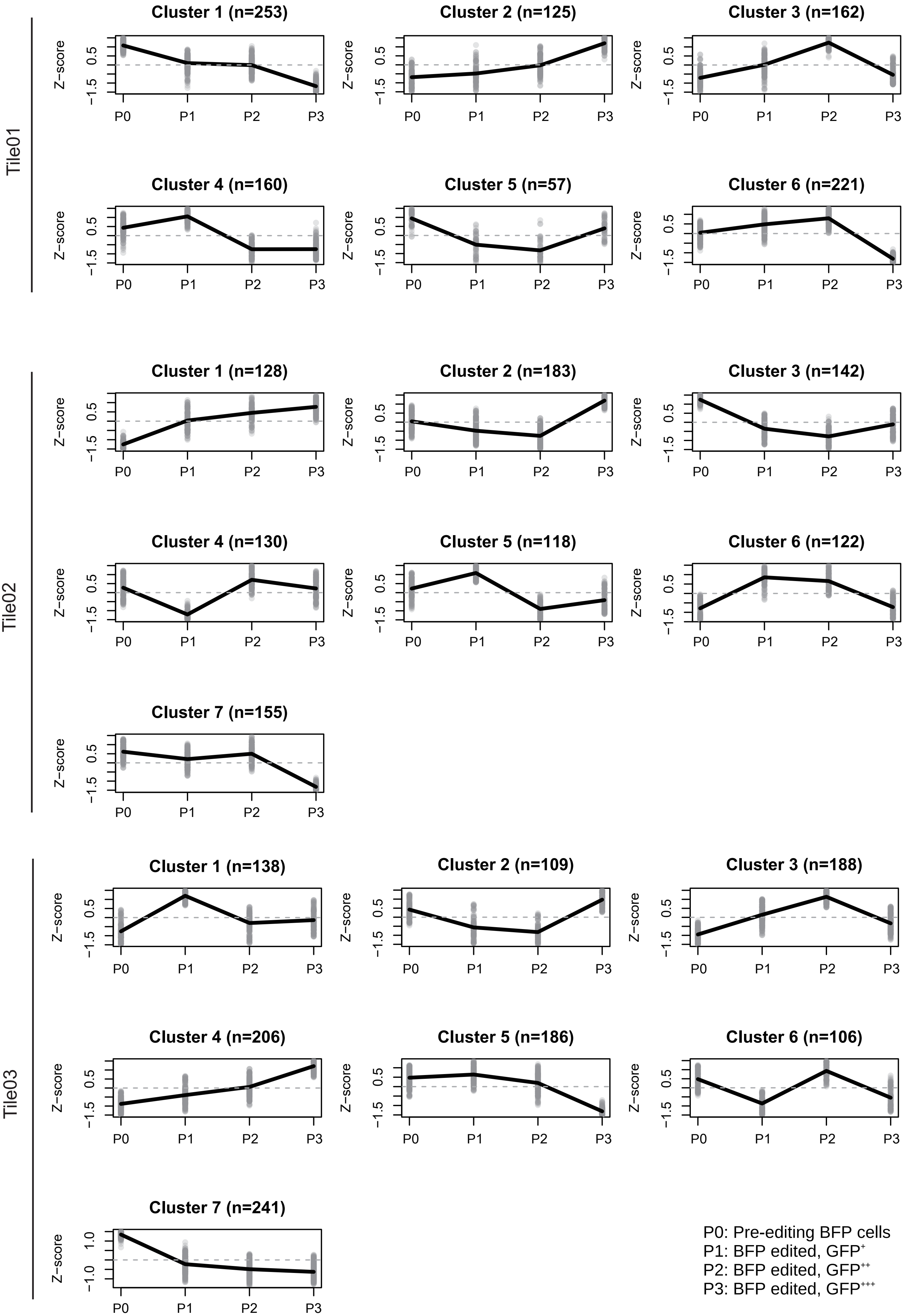
Tile01
Cluster 1 (n=253)
Cluster 2 (n=125)
Cluster 3 (n=162)
Cluster 4 (n=160)
Cluster 5 (n=57)
Cluster 6 (n=221)
Tile02
Cluster 1 (n=128)
Cluster 2 (n=183)
Cluster 3 (n=142)
Cluster 4 (n=130)
Cluster 5 (n=118)
Cluster 6 (n=122)
Cluster 7 (n=155)
Tile03
Cluster 1 (n=138)
Cluster 2 (n=109)
Cluster 3 (n=188)
Cluster 4 (n=206)
Cluster 5 (n=186)
Cluster 6 (n=106)
Cluster 7 (n=241)
Z−score
P0
P1
P2
P3
P0: Pre-editing BFP cells
P1: BFP edited, GFP+
P2: BFP edited, GFP++
P3: BFP edited, GFP+++

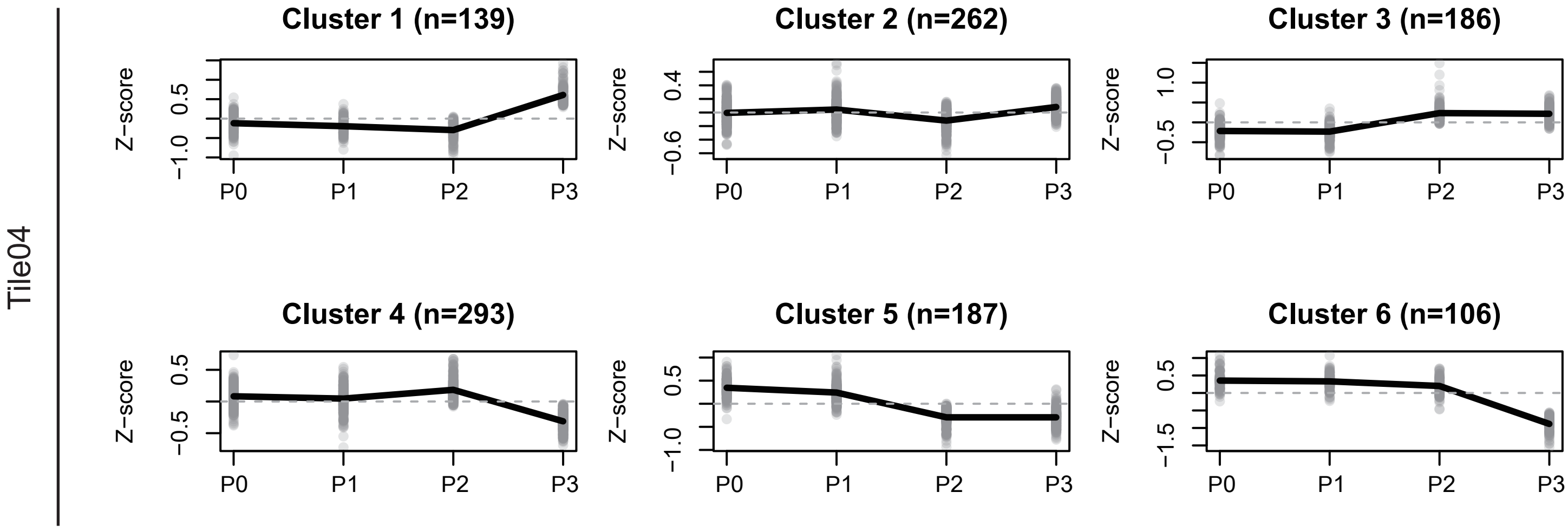
Tile04
Cluster 1 (n=139)
Cluster 2 (n=262)
Cluster 3 (n=186)
Cluster 4 (n=293)
Cluster 5 (n=187)
Cluster 6 (n=106)
Z−score
P0
P1
P2
P3
−1.0
0.5
−0.6
0.4
−0.5
1.0
−1.5
