# Supplementary material for: Discovery and Engineering of a Rat Endogenous Retrovirus Reverse Transcriptase for Efficient Prime Editing
Source: Adv Sci (Weinh). 2026 Jun 26;13(49):e75888. doi: 10.1002/advs.75888 (PMC13335913; doi:10.1002/advs.75888)
Supplement: Supplementary file 3 [file ADVS-13-e75888-s006.pdf]

Supporting Information

Figure S1. Activity analysis of newly identified reverse transcriptases.

Figure S2. Schematic diagrams of domains in 19 functionally active RTs identified.

Figure S3. Prime editing efficiencies at endogenous rice loci of RT32.

Figure S4. Sequence alignment of RERV-RT, PERV-RT and M-MLV-RT, and BFP-to-GFP reporter system fluorescence for engineered RERV-RT mutants.

Figure S5. Prime editing efficiencies at endogenous rice loci between RERV-RT and its truncated variants.

Figure S6. Comparison of prime editing efficiency among PE-RERV-m5, PE-enRERV and their optimized version via SB2 and OsMLH1dn.

Figure S7. Optimization of plant prime editing efficiency via the MCP-MS2 recruitment system.

Figure S8. Enhancement of plant prime editing efficiency by utilizing the 35C promoter.

Figure S9. Multiplex prime editing frequencies of three target genes in 60 regenerated T_0 rice plants.

Figure S10. Saturation mutagenesis of core amino acid regions in RERV-RT and screening of potential effective mutation sites.

Figure S11. Comparison of prime editing efficiency between RERV-RT-m5 and M-MLV-RT in ePPE7plus architecture.

Figure S12. Comparison of byproducts between PE-enRERV and PE-M-MLV .

Figure S13. Analysis of byproduct types and abundances at the prime-edited target sites corresponding to Figure 6.

A

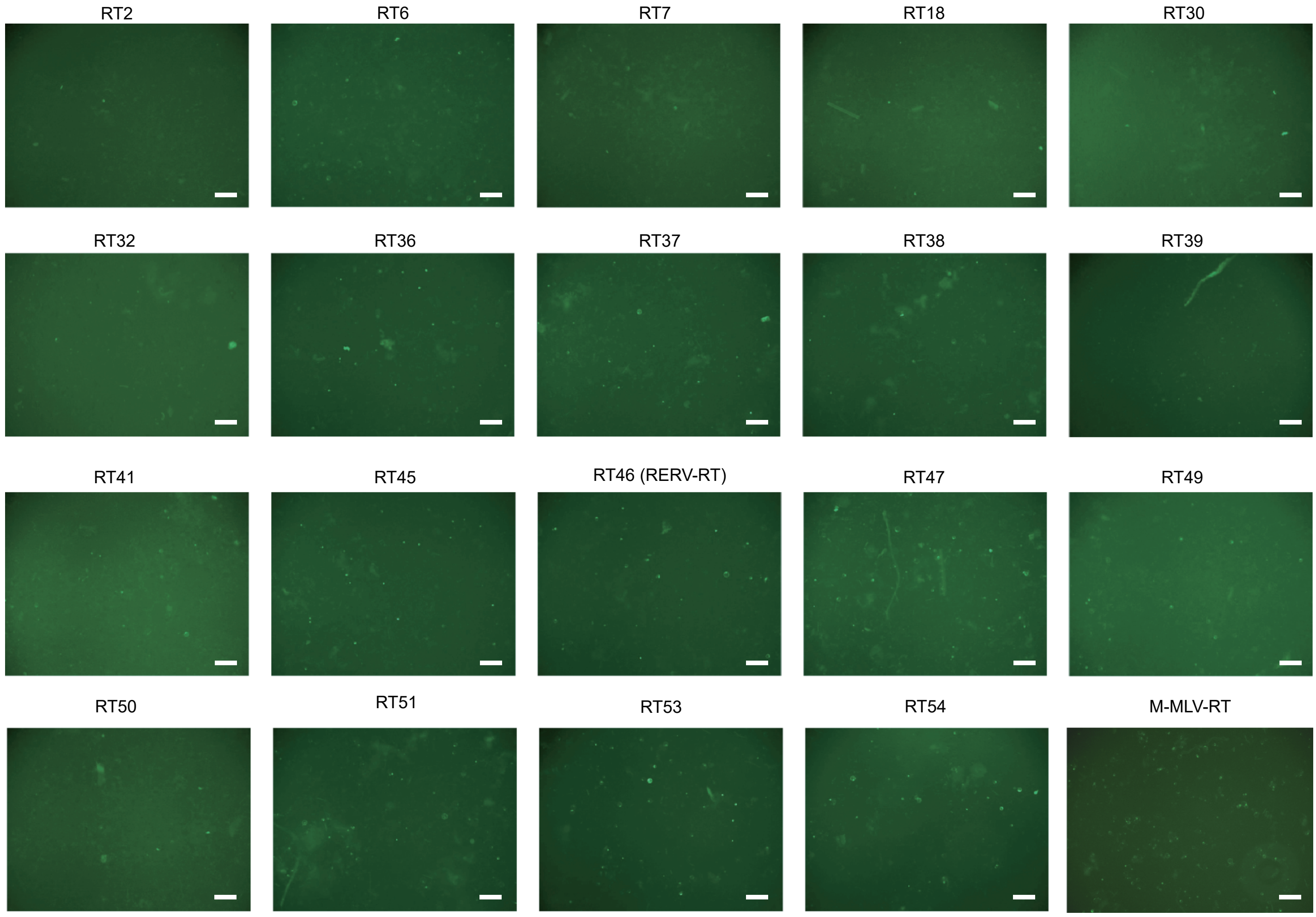

B

RTase	enzyme activity	RTase	enzyme activity	RTase	enzyme activity	RTase	enzyme activity	RTase	enzyme activity
RT1		RT12		RT23		RT34		RT45	* *
RT2	*	RT13		RT24		RT35		RT46(RERV-RT)	* * *
RT3		RT14		RT25		RT36	* *	RT47	* *
RT4		RT15		RT26		RT37	* *	RT48	
RT5		RT16		RT27		RT38	*	RT49	*
RT6	* *	RT17		RT28		RT39	*	RT50	*
RT7	*	RT18	*	RT29		RT40		RT51	* *
RT8		RT19		RT30	*	RT41	*	RT52	
RT9		RT20		RT31		RT42		RT53	* *
RT10		RT21		RT32	* *	RT43		RT54	*
RT11		RT22		RT33		RT44		M-MLV-RT	* * * *

Figure S1. Activity analysis of newly identified reverse transcriptases.
A Evaluation of prime editing efficiency of the newly identified RTs in rice protoplasts via the BFP-to-GFP reporter system using microscopy. Microscopic images of BFP-to-GFP conversion in rice protoplasts expressing 54 RTs, showing fluorescence signals from 19 RTs with biological activity. M-MLV-RT was used as a positive control. *Scale bars,* 150 μm. **B** Enzyme activity was measured based on the fluorescence intensity of reverse transcriptases in the rice fluorescent reporter system, where the number of asterisks (*) indicates the level of fluorescence intensity.

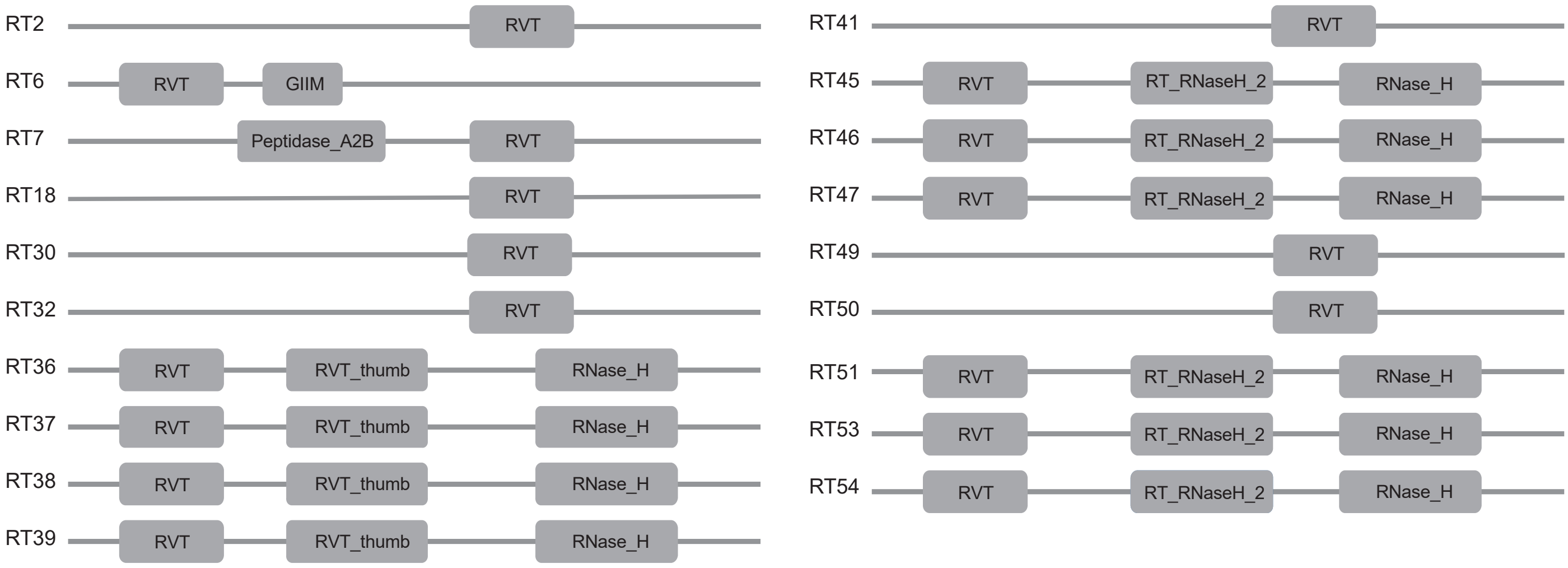

Figure S2. Schematic diagrams of domains in 19 functionally active RTs identified.
Domain prediction of the 19 active reverse transcriptases via the HMMERSCAN web server revealed that all these enzymes harbor the conserved RVT domain, along with other different domains.

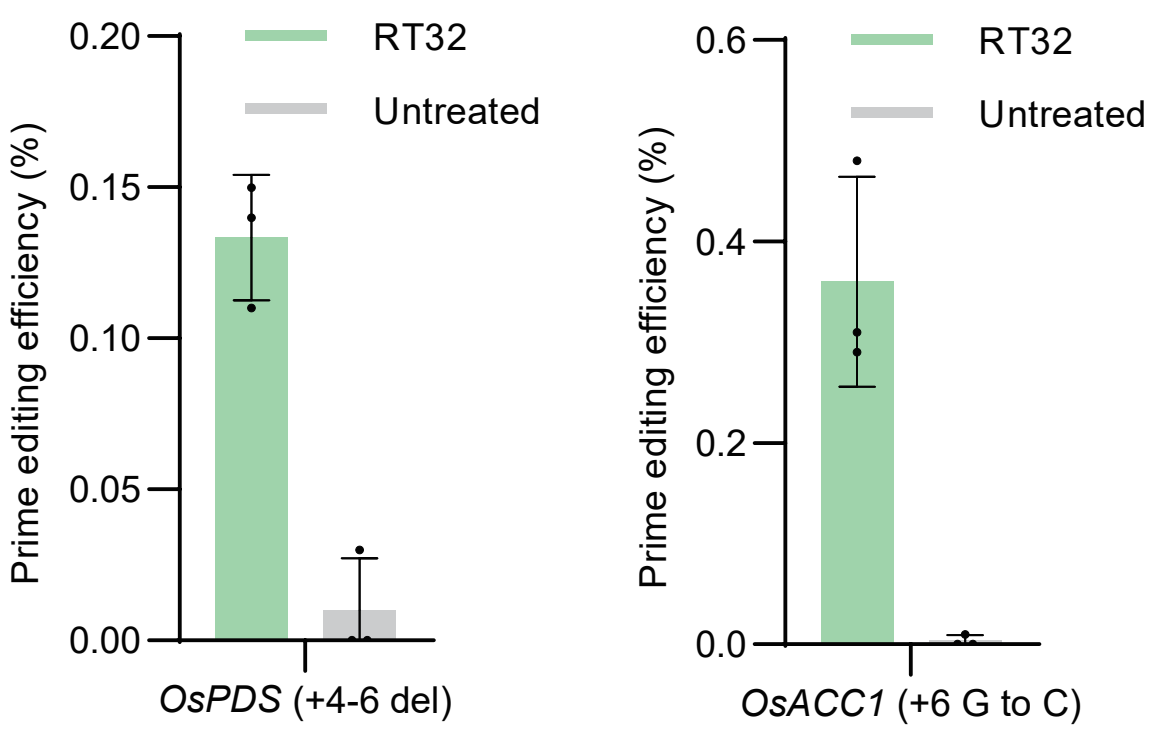

Figure S3. Prime editing efficiencies at endogenous rice loci of RT32.
Data are presented as means ± standard deviations from three independent biological replicates.

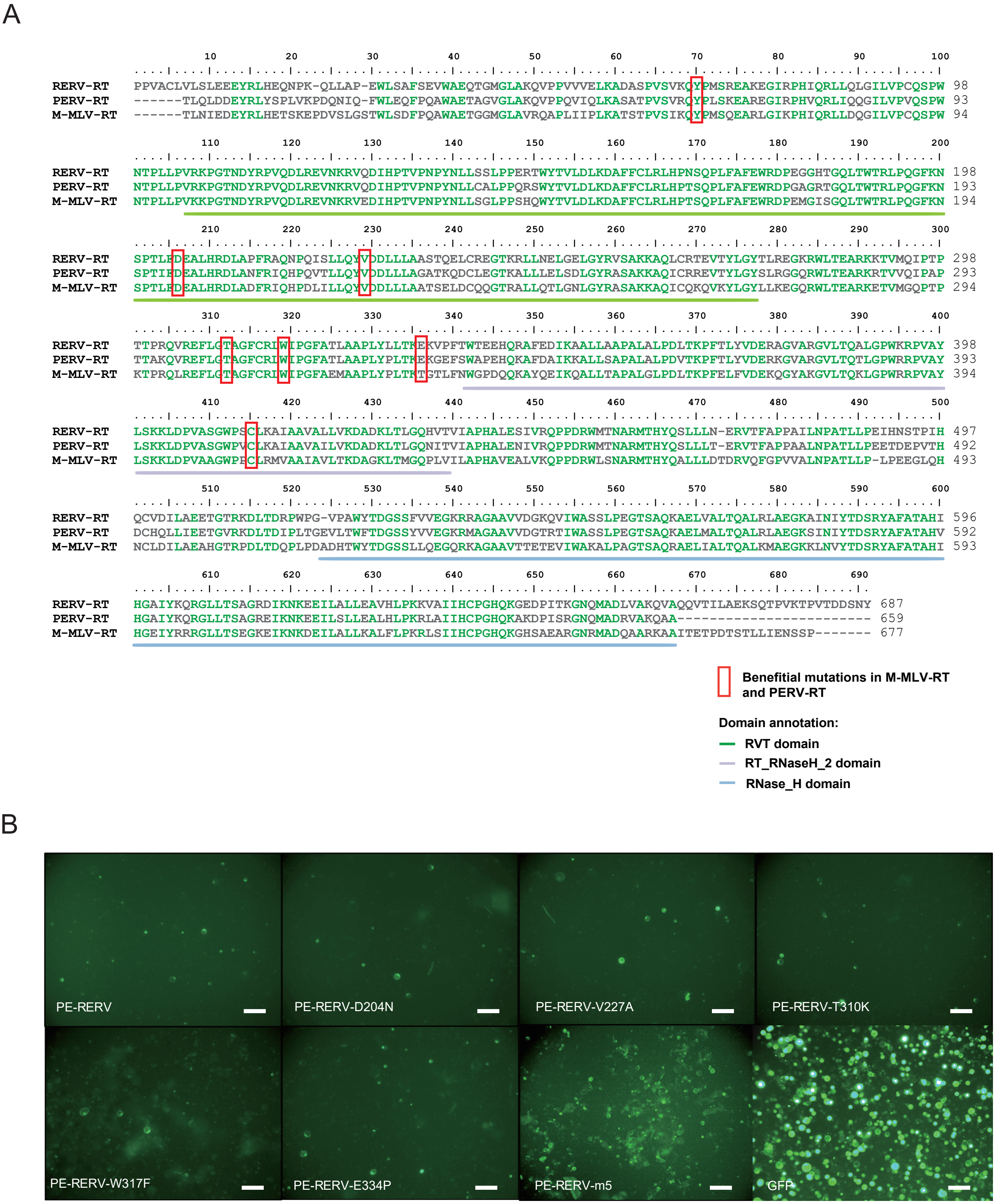

Figure S4 Sequence alignment of RERV-RT, PERV-RT and M-MLV-RT, and BFP-to-GFP reporter system fluorescence for engineered RERV-RT mutants.

A Amino acid sequence alignment of RERV-RT, PERV-RT and M-MLV-RT. In the alignment results, red boxes indicate the positions of beneficial mutations in M-MLV-RT and PERV-RT and their corresponding residues in RERV-RT; green underlines indicate the amino acid residues of the RVT domain in RERV-RT; purple underlines indicate the amino acid residues of the RT_RNaseH domain; and blue underlines indicate the amino acid residues of the RNase_H domain. **B** Microscopic evaluation of BFP-to-GFP prime editing efficiencies of distinct engineered RERV-RT mutants in rice protoplasts. GFP indicates the expression of GFP protein in rice protoplasts, which serves as a positive control for this system. *Scale bars*, 150 µm. One of three independent experiments is shown.

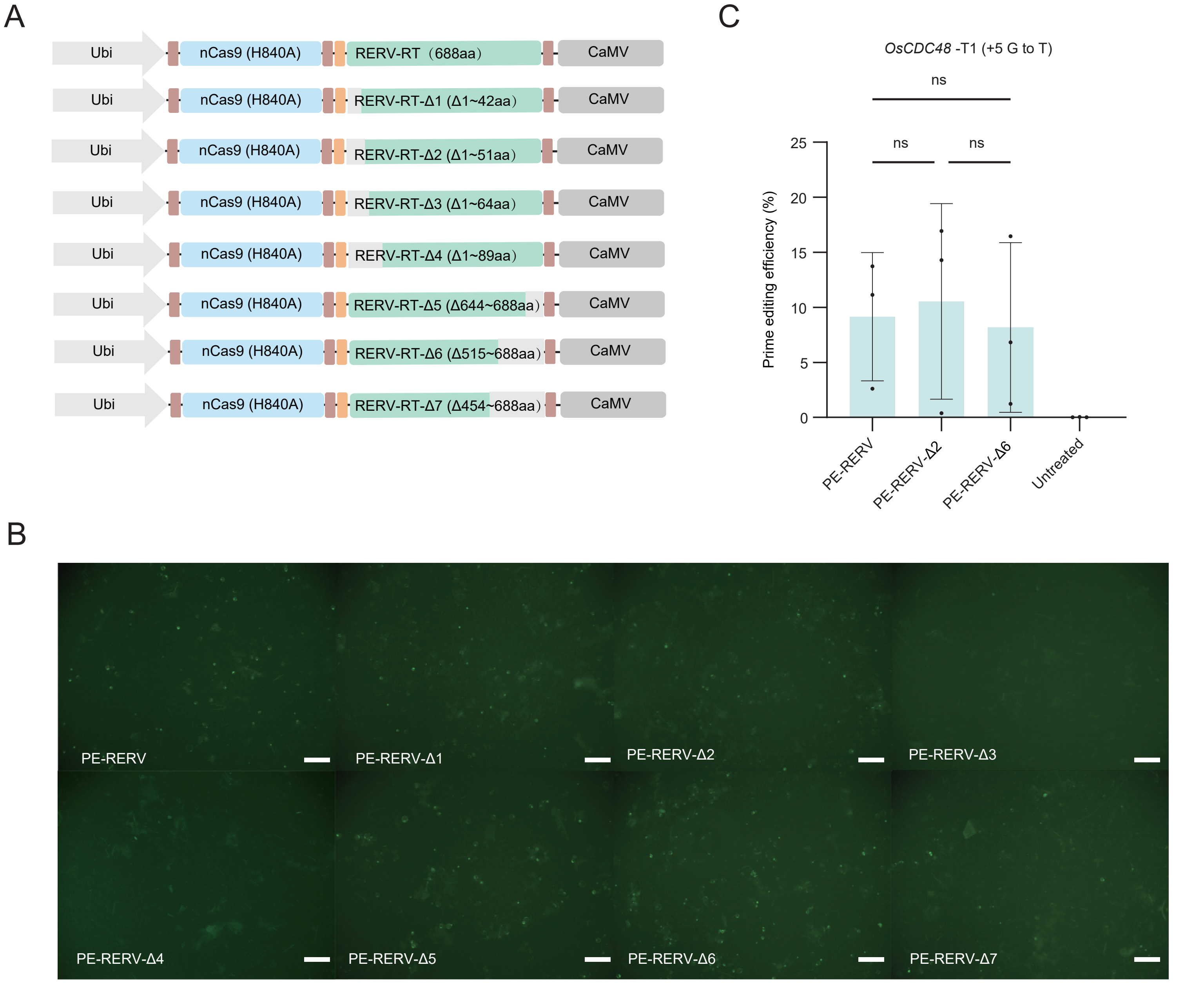

Figure S5. Prime editing efficiencies at endogenous rice loci between RERV-RT and its truncated variants.

A Schematic diagram of vectors encoding distinct truncated variants of RERV-RT. **B** Microscopic evaluation of prime editing efficiency of different truncated variants in rice protoplasts via the BFP-to-GFP reporter system, *Scale bars*, 150 µm. The image shows results from one of three independent experiments. **C** Prime editing efficiencies of PE-RERV-Δ2 and PE-RERV-Δ6 (N-terminal and C-terminal truncated variants of RERV-RT, respectively) at endogenous sites in rice protoplasts.

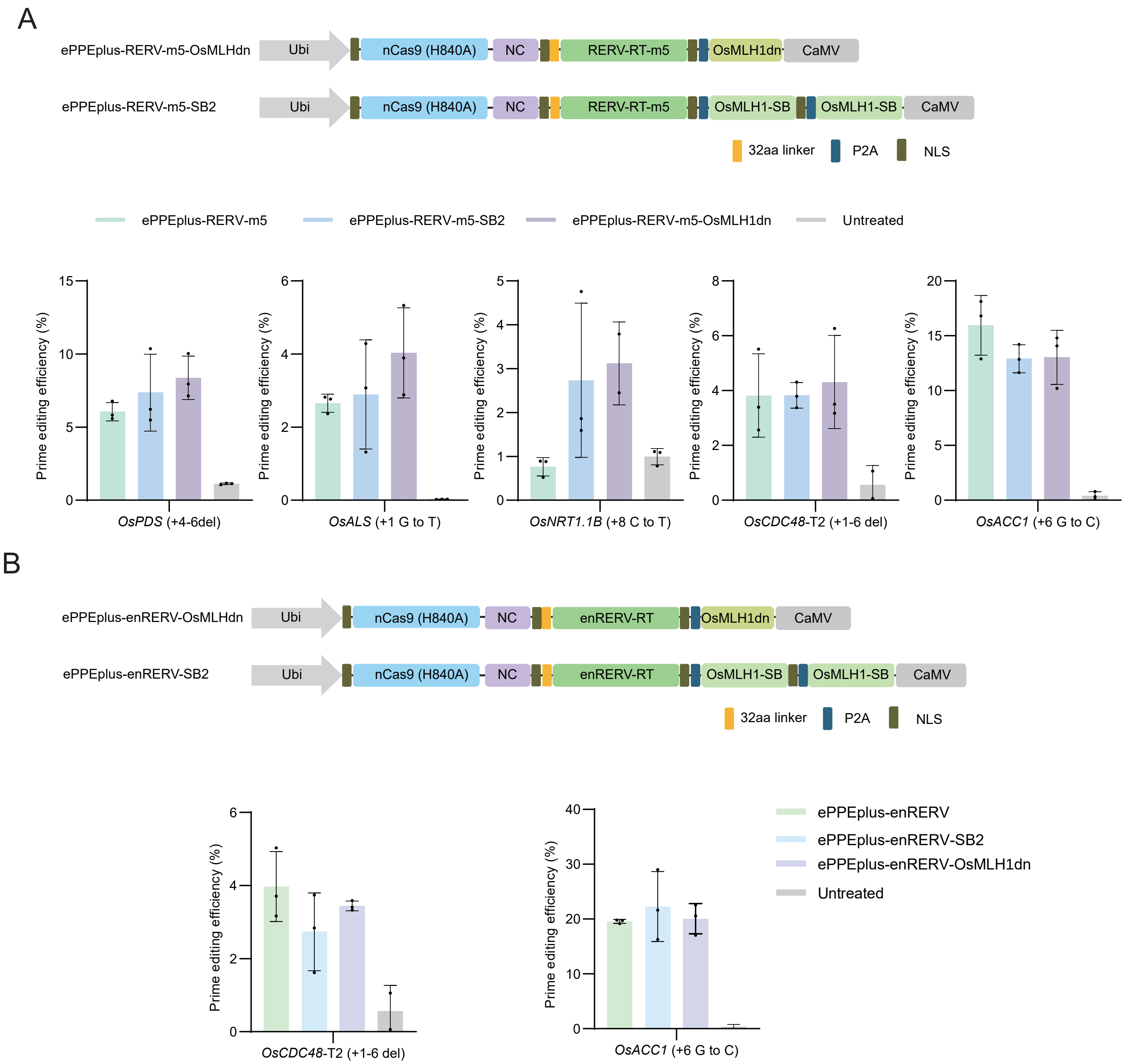

Figure S6. Comparison of prime editing efficiency among PE-RERV-m5, PE-enRERV and their optimized version via SB2 and OsMLH1dn. **A** Schematic diagram of vectors encoding PE-RERV-m5 fused with DNA mismatch repair inhibitors (SB2 and OsMLH1dn), along with prime editing efficiencies at five endogenous sites in rice protoplasts. **B** Schematic diagram of vectors encoding PE-enRERV fused with DNA mismatch repair inhibitors (SB2 and OsMLH1dn), along with prime editing efficiencies at two endogenous sites in rice protoplasts.

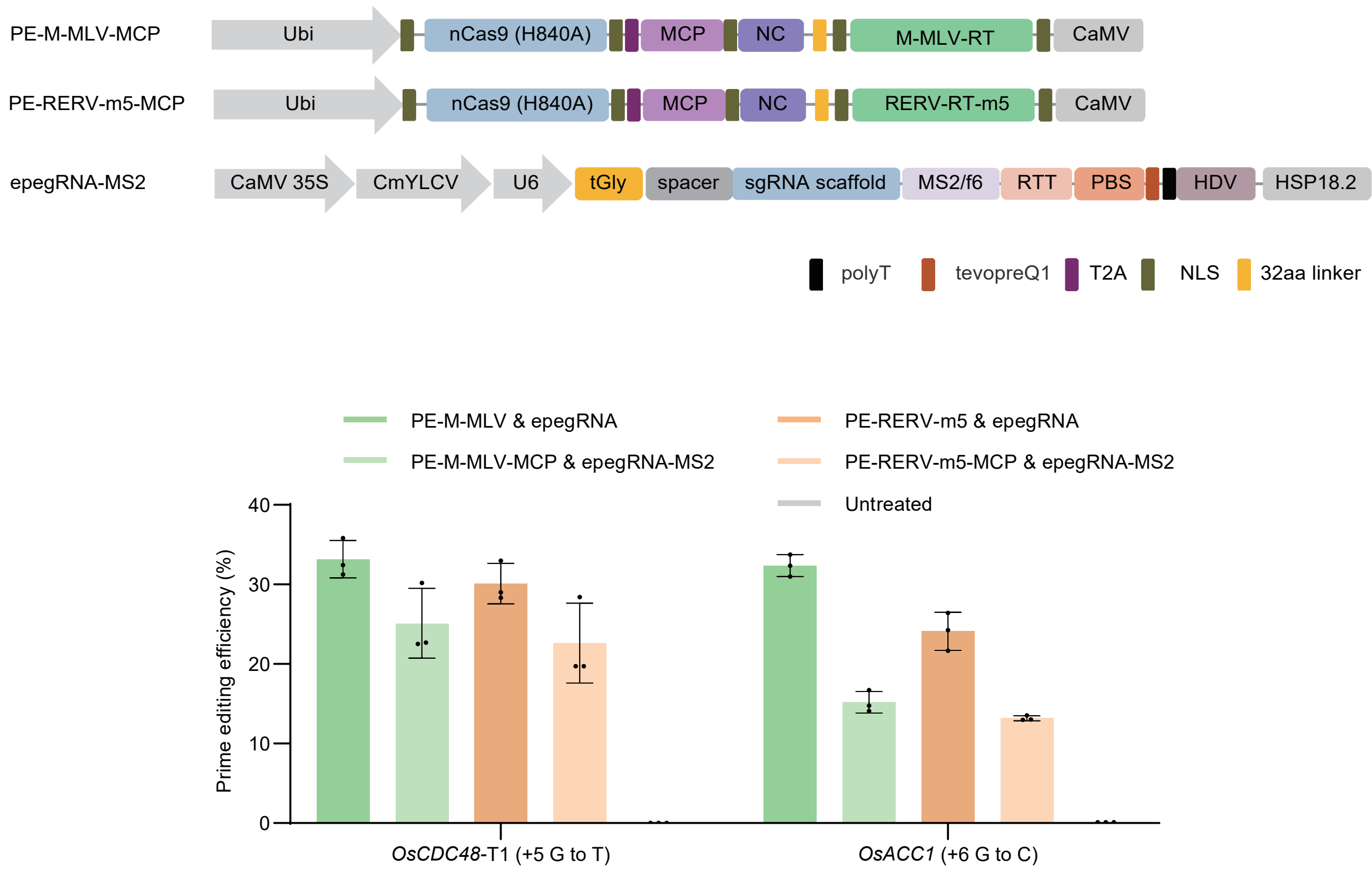

Figure S7 Optimization of plant prime editing efficiency via the MCP-MS2 recruitment system.
Schematic diagram of vector structures for PE-M-MLV-MCP, PE-RERV-m5-MCP and epegRNA-MS2, along with a comparison of prime editing efficiencies with the non-MCP-MS2 recruitment system at endogenous sites in rice protoplasts.

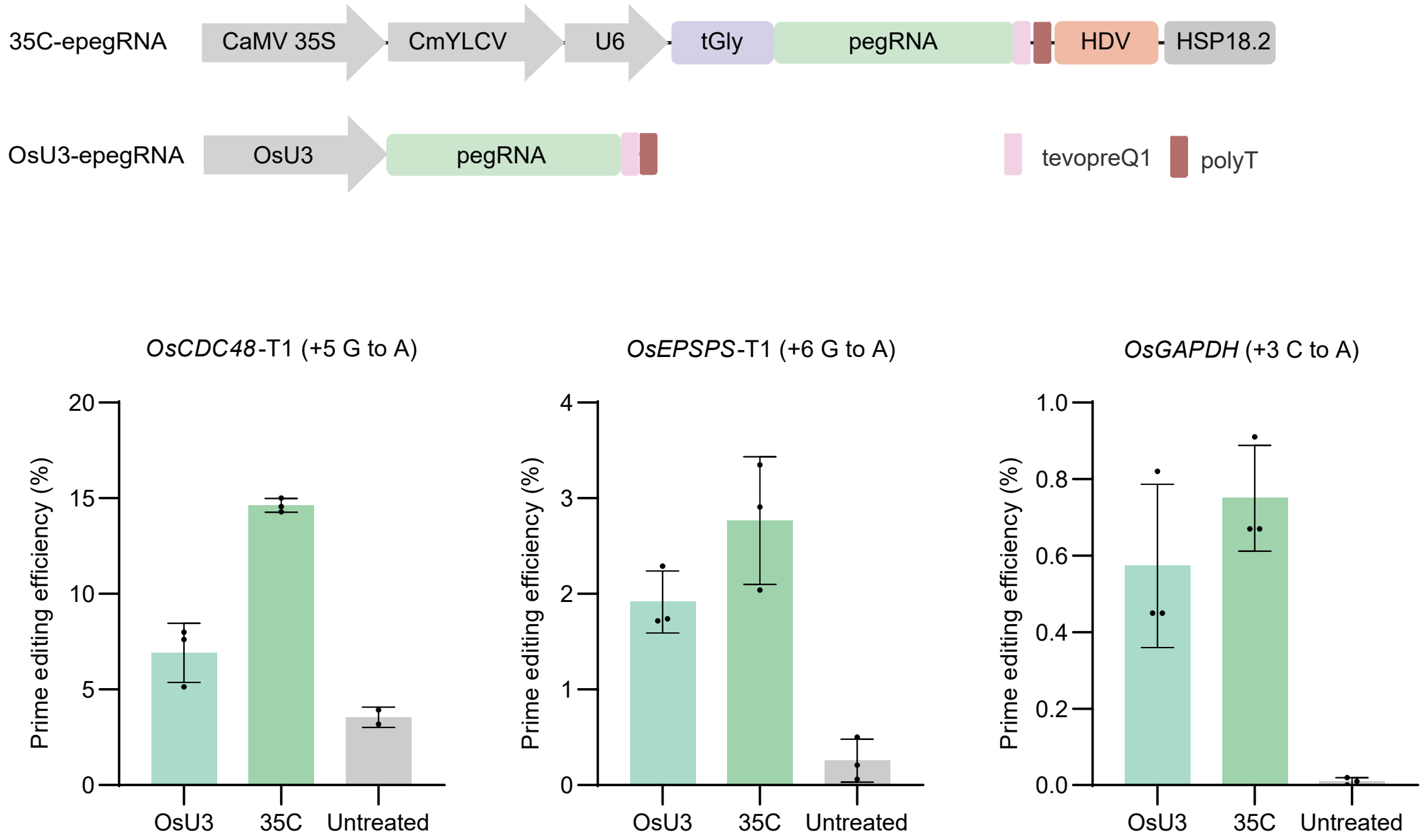

Figure S8. Enhancement of plant prime editing efficiency by utilizing the 35C promoter.

Schematic diagram of vector structures for epegRNA driven by the 35C promoter, along with prime editing efficiencies at three endogenous sites in rice protoplasts.

A

Edited gene in T_0 plants	Desired edit type	No. of transgenic rice plants	No. of plants with desired edits	Prime editing efficiency
OsACC1	G to C	60	39	65.0%
OsCDC48	G to T	60	35	58.3%
OsEPSPS	multiple base substitutions (T to C、G to A、G to A)	60	31	51.2%

B

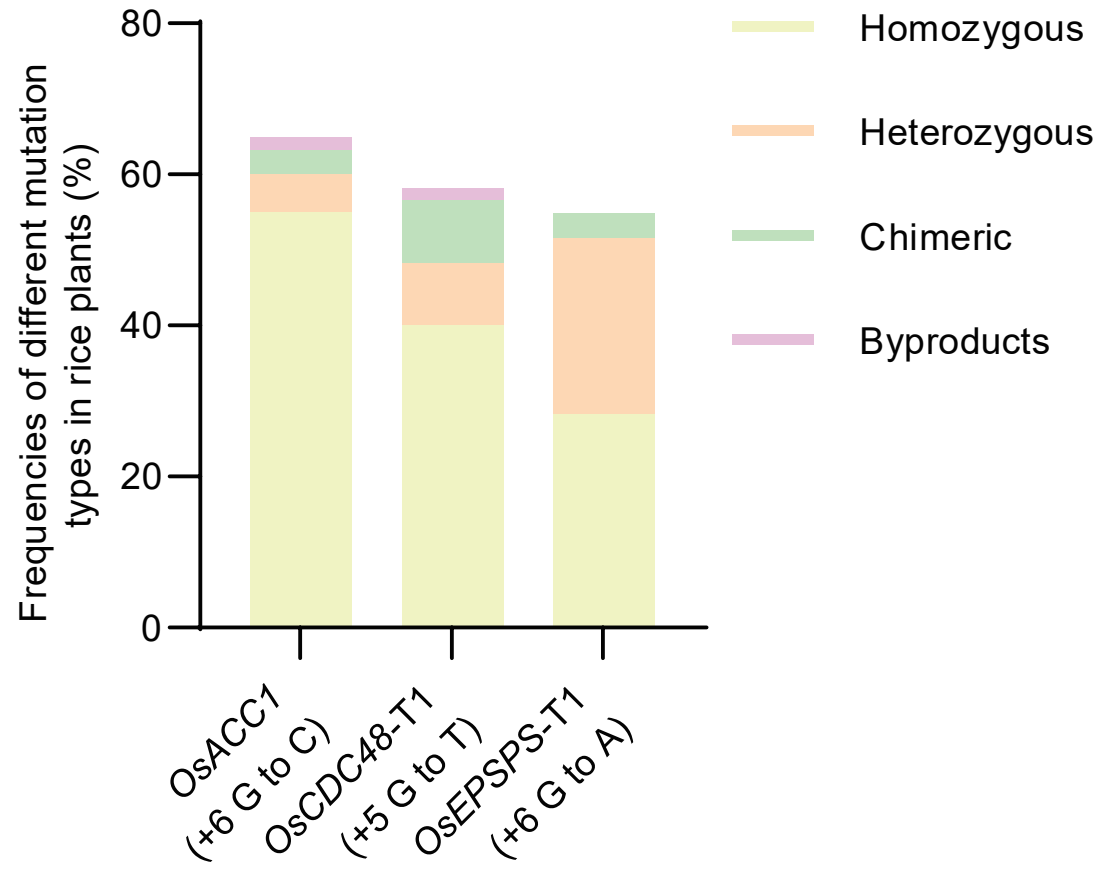

Figure S9. Multiplex prime editing frequencies of three target genes in 60 regenerated T_0 rice plants.
A Editing frequencies of the three target genes in multiplex gene editing, along with the proportion of regenerated rice plants harboring edits for each target gene. **B** In the bar charts, yellow represents the proportion of homozygous edits for each gene in regenerated rice plants, orange represents heterozygous edits, green represents chimeric edits, and pink represents edits with byproducts.

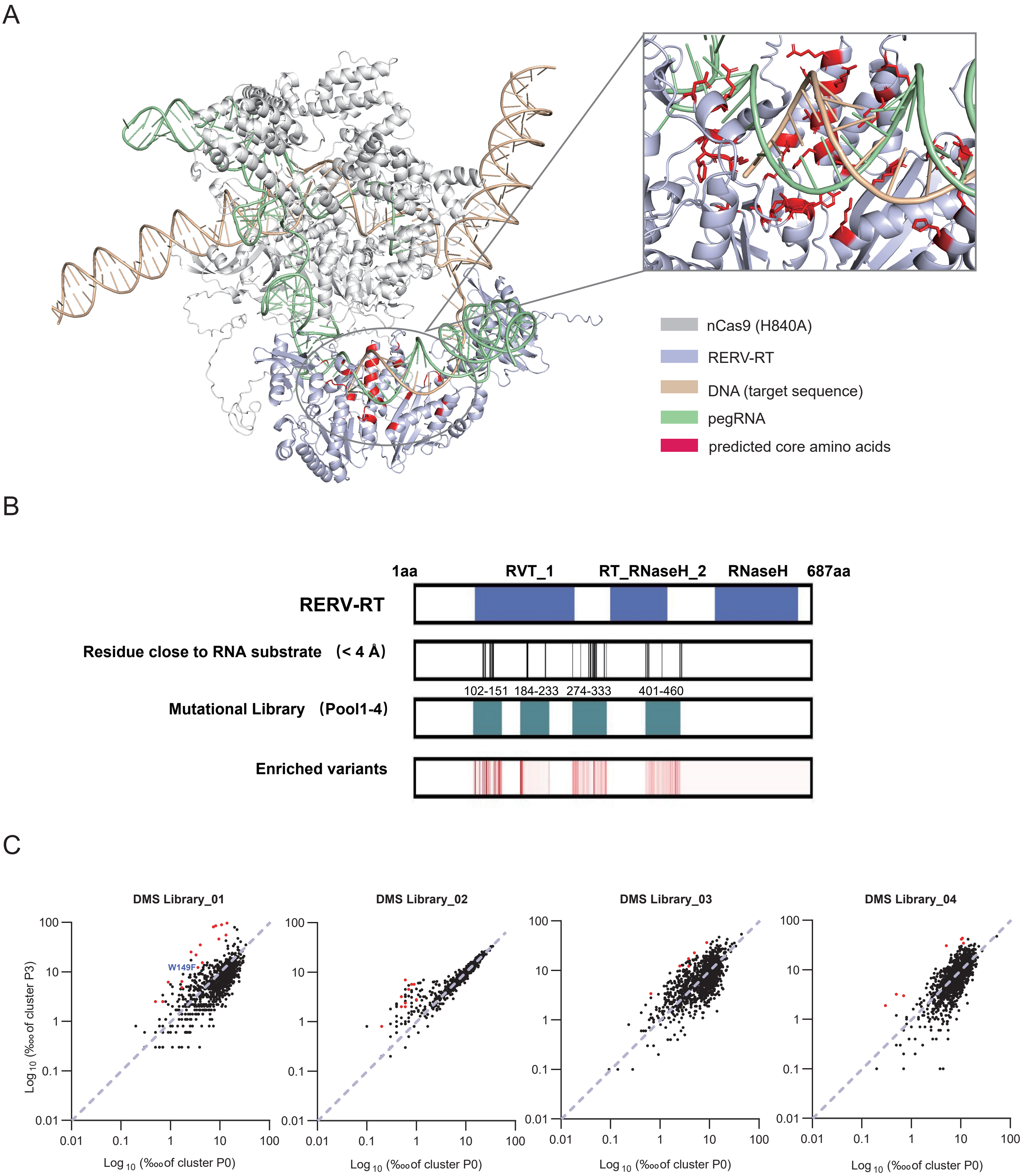

Figure S10.Saturation mutagenesis of coreaminoacidregionsinRERV-RT andscreeningof potential effective mutation sites.
A Structure of the RERV-RT-nCas9-target DNA-pegRNA complex predicted by AlphaFold 3, where red indicates predicted core amino acids involvedin interaction segments and locatedwithin 4 Å of the nucleic acid substrate.**B** Design of the DMS library, including the core domains of RERV-RT, candidate amino acids within 4 Å (as shown in A), defined ranges of the four DMS libraries and positions of fluorescence-enriched mutation sites. **C** Results of the four DMS libraries, where P0 represents the proportion of fluorescentcellsat the initial stage of mutagenesis and P3 denotes the proportion of fluorescentcellsat the final stage of mutagenesis, both expressed in log_{10}format.Cell lines transfected with the four respectively constructed mutant vector libraries were subjected to sorting and enrichment of GFP-positive cells (indicating successful prime editing) using the BFP-GFP reporter system combined with flow cytometry; the corresponding mutation sites of each enriched cell population were verified by next-generation sequencing (NGS). The red dots denote the candidate high-efficiency point mutations verified in this study.

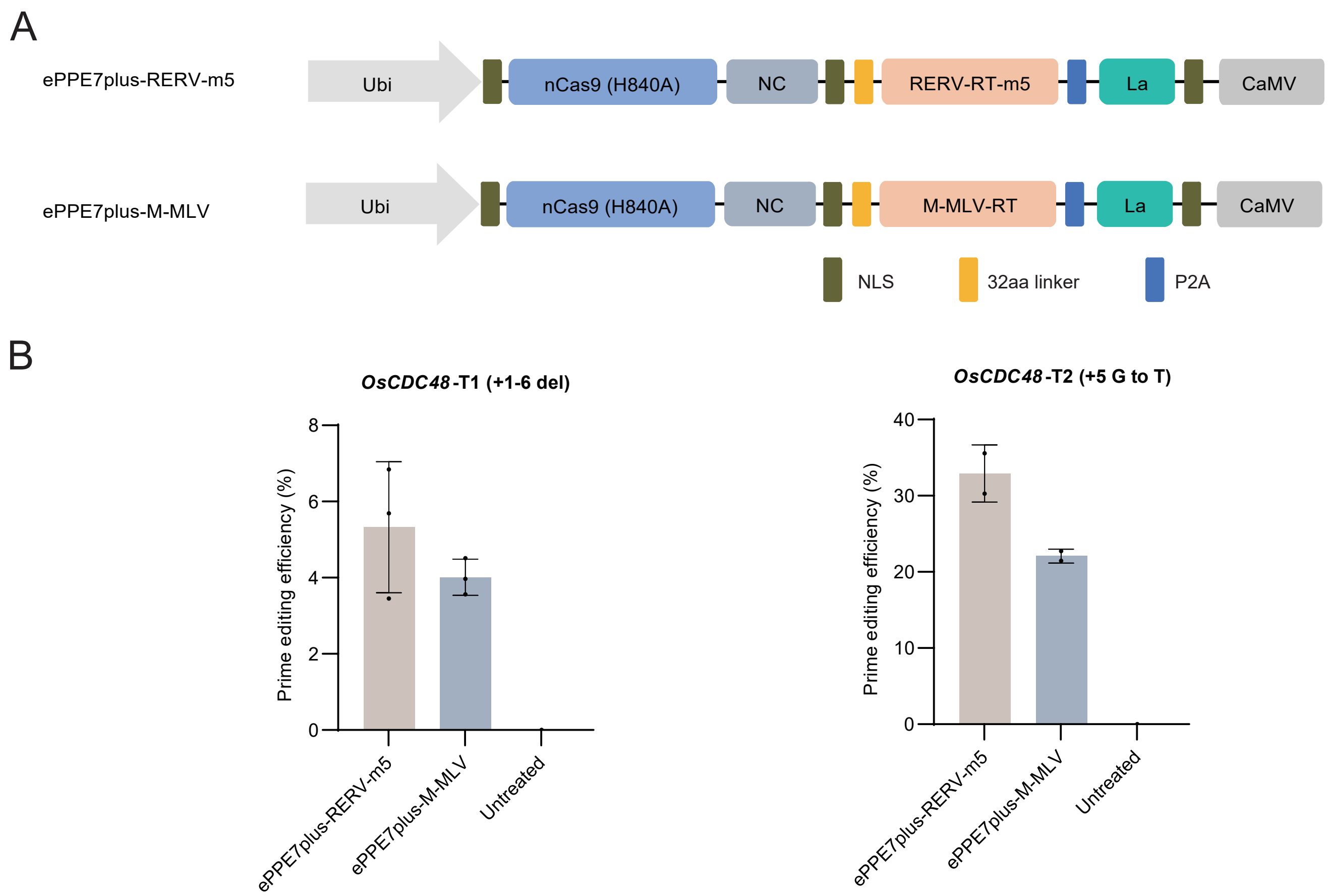

Figure S11. Comparison of prime editing efficiency between RERV-RT-m5 and M-MLV-RT in ePPE7plus architecture.
A Schematic diagram of the ePPE7plus-RERV-m5 and ePPE7plus-M-MLV vectors. **B** Comparison of efficiency between ePPE7plus-RERV-M5 and ePPE7plus-M-MLV at two endogenous loci.

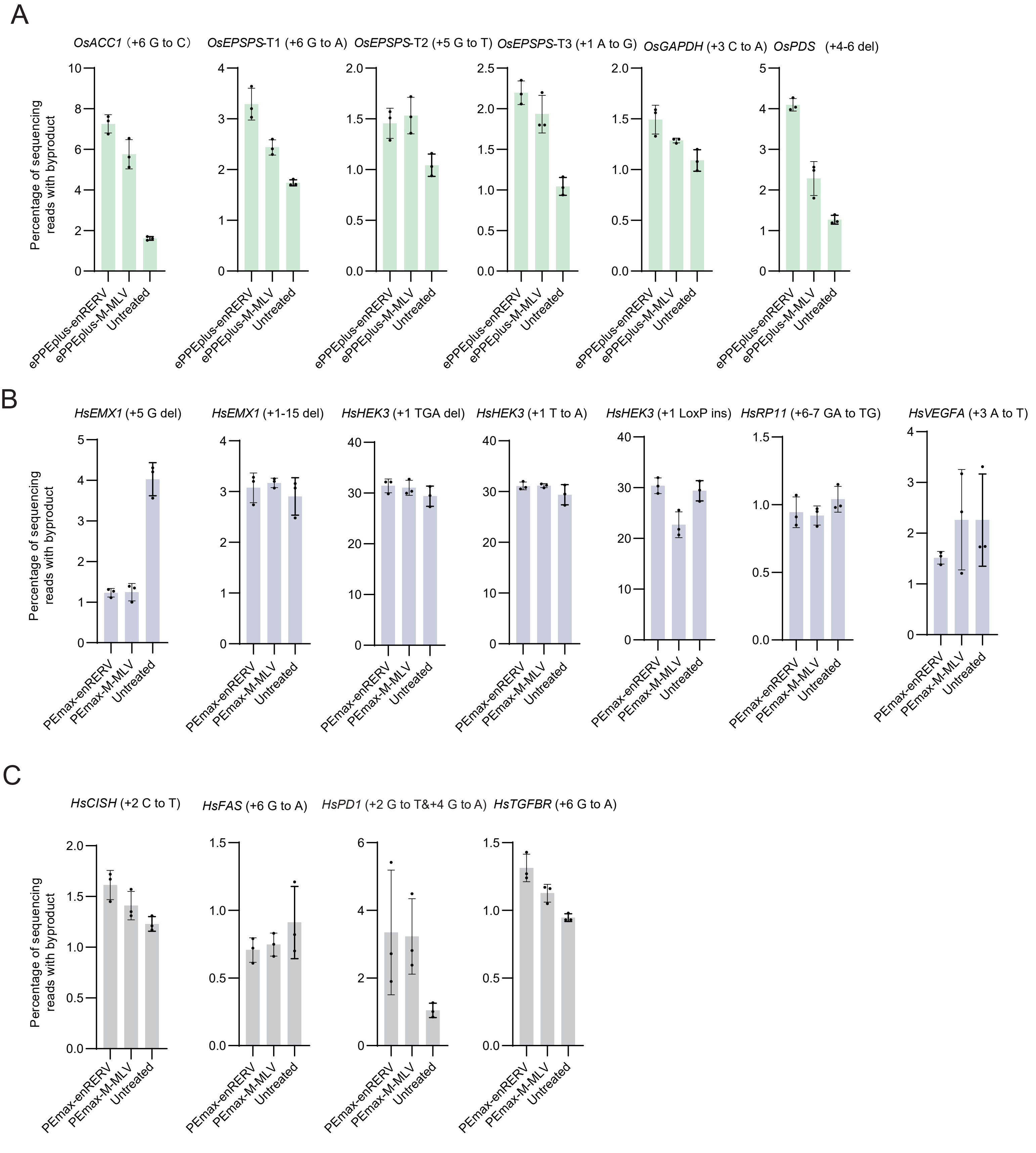

Figure S12. Comparison of byproducts between PE-enRERV and PE-M-MLV.
A Byproducts between PE-enRERV and PE-M-MLV across rice endogenous sites, corresponding to Figure 6C in main text. **B** Byproducts between PE-enRERV and PE-M-MLV across human endogenous targets, corresponding to Figure 6B in main text. **C** Byproducts between PE-enRERV and PE-M-MLV across endogenous targets for enhances the durability and potency of CAR-T cells, corresponding to Figure 6 G inmain text. Data are presented as means ± standard deviations from three independent biological replicates.

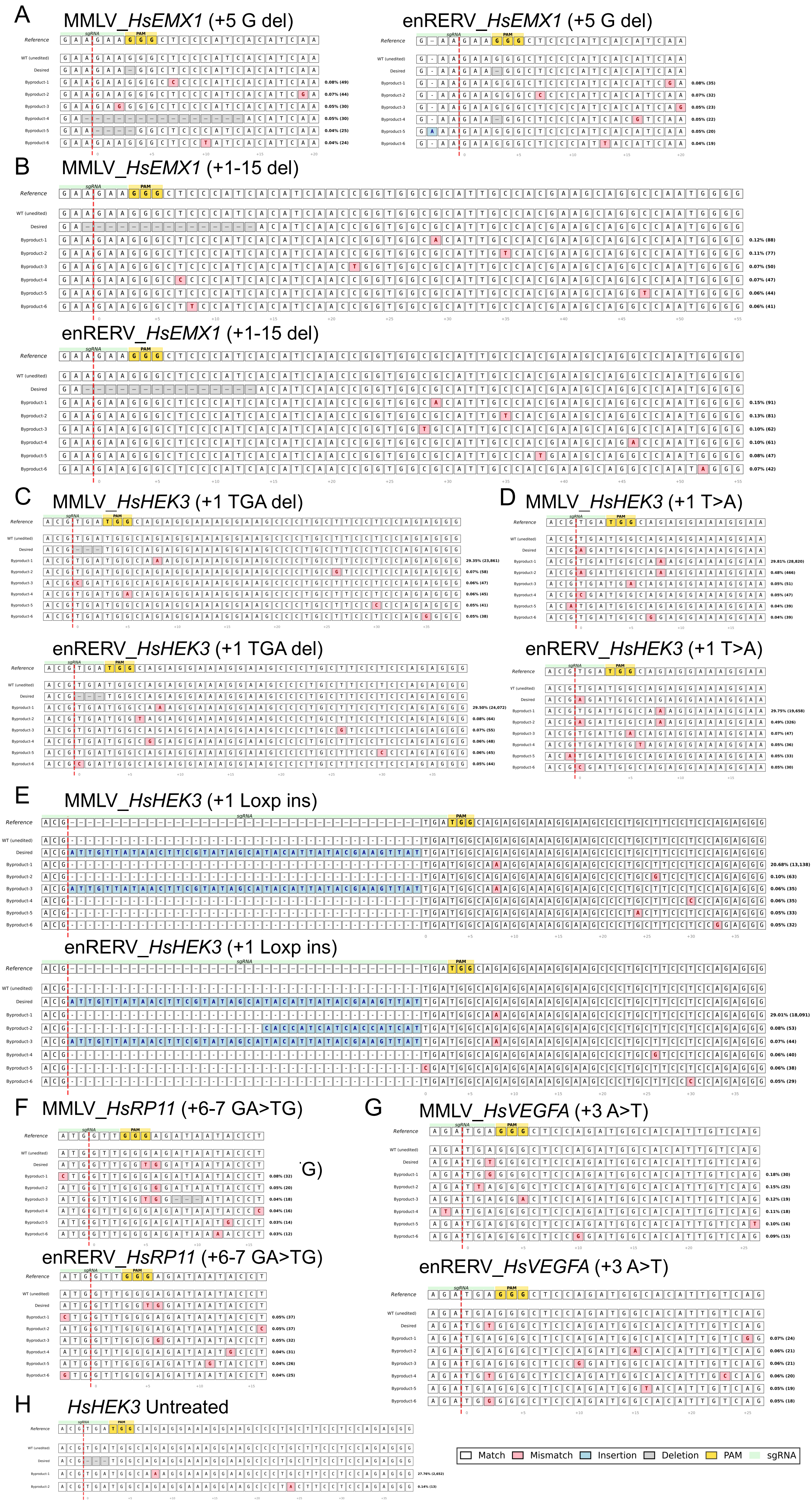
A
MMLV_HsEMX1 (+5 G del)
enRERV_HsEMX1 (+5 G del)
B
MMLV_HsEMX1 (+1-15 del)
enRERV_HsEMX1 (+1-15 del)
C
MMLV_HsHEK3 (+1 TGA del)
enRERV_HsHEK3 (+1 TGA del)
D
MMLV_HsHEK3 (+1 T>A)
enRERV_HsHEK3 (+1 T>A)
E
MMLV_HsHEK3 (+1 Loxp ins)
enRERV_HsHEK3 (+1 Loxp ins)
F
MMLV_HsRP11 (+6-7 GA>TG)
enRERV_HsRP11 (+6-7 GA>TG)
G
MMLV_HsVEGFA (+3 A>T)
enRERV_HsVEGFA (+3 A>T)
H
HsHEK3 Untreated
Reference
WT (unedited)
Desired
Byproduct-1
Byproduct-2
Byproduct-3
Byproduct-4
Byproduct-5
Byproduct-6
sgRNA
PAM
Match
Mismatch
Insertion
Deletion
PAM
sgRNA

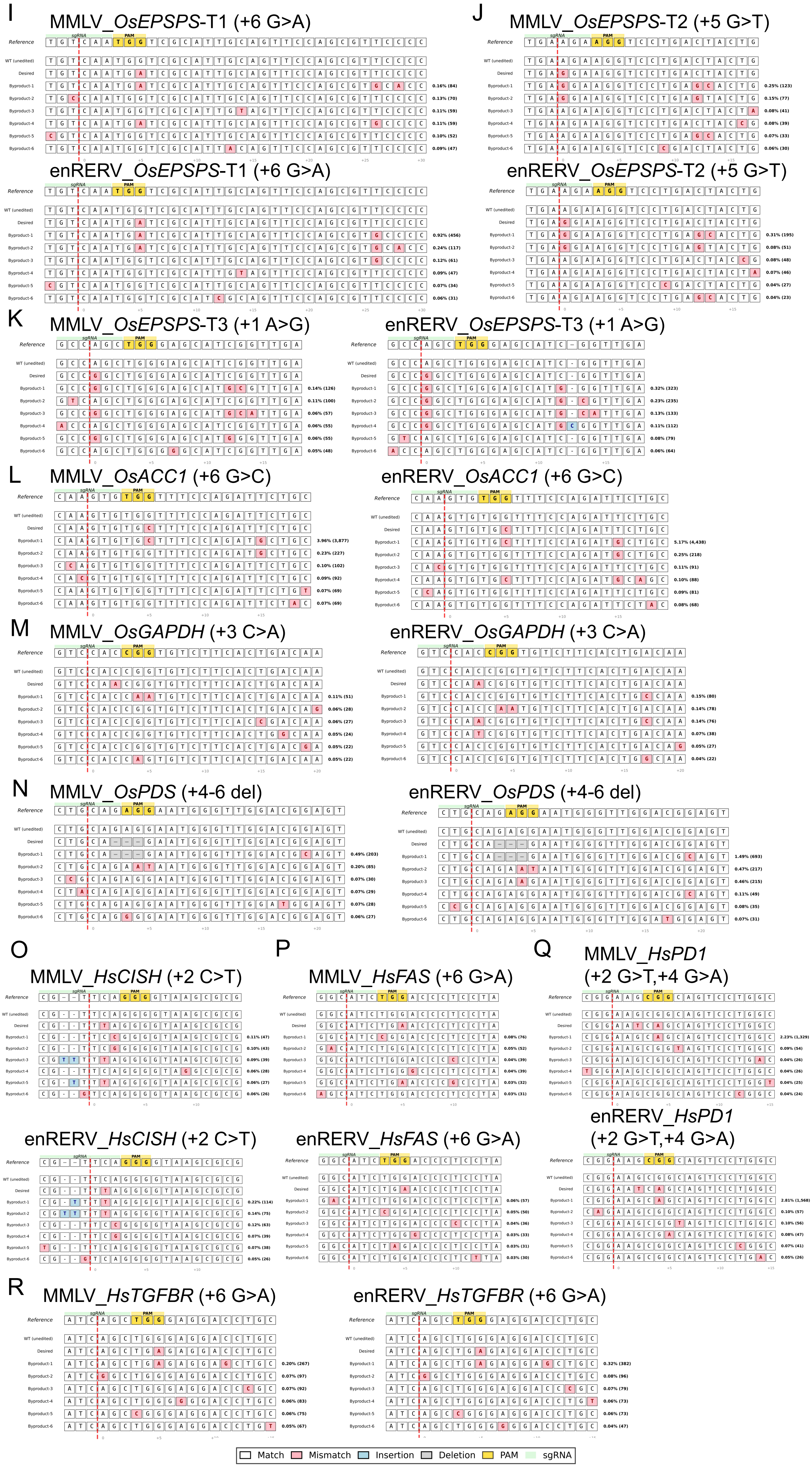
I
MMLV_OsEPSPS-T1 (+6 G>A)
enRERV_OsEPSPS-T1 (+6 G>A)
J
MMLV_OsEPSPS-T2 (+5 G>T)
enRERV_OsEPSPS-T2 (+5 G>T)
K
MMLV_OsEPSPS-T3 (+1 A>G)
enRERV_OsEPSPS-T3 (+1 A>G)
L
MMLV_OsACC1 (+6 G>C)
enRERV_OsACC1 (+6 G>C)
M
MMLV_OsGAPDH (+3 C>A)
enRERV_OsGAPDH (+3 C>A)
N
MMLV_OsPDS (+4-6 del)
enRERV_OsPDS (+4-6 del)
O
MMLV_HsCISH (+2 C>T)
enRERV_HsCISH (+2 C>T)
P
MMLV_HsFAS (+6 G>A)
enRERV_HsFAS (+6 G>A)
Q
MMLV_HsPD1 (+2 G>T,+4 G>A)
enRERV_HsPD1 (+2 G>T,+4 G>A)
R
MMLV_HsTGFBR (+6 G>A)
enRERV_HsTGFBR (+6 G>A)
Match
Mismatch
Insertion
Deletion
PAM
sgRNA

Figure S13. Analysis of byproduct types and abundances at the prime-edited target sites corresponding to Figure 6.
A-G & I-R Analysis and Visualization of NGS data showing the specific byproduct mutations generated by PE-M-MLV and PE-enRERV at various target genes (*OsEPSPS*, *OsACC1*, *OsGAPDH*, *OsPDS*, *HsPD1*, *HsCISH*, *HsFAS*, *HsTGFBR*, *HsEMX1*, *HsHEK3*, *HsRP11*, and *HsVEGFA*). Detected byproducts include base substitutions (e.g., +6 G>A, +5 G>T, +3 A>T) and small indels (e.g., +1-15 del, +4-6 del, +1 Loxp ins). In the sequence alignments, sgRNAs, PAM sequences, matched bases, mismatches, and insertions/deletions (indels) are color-coded as indicated in the legend. A-G are corresponding to Figure 6B, I-N are corresponding to Figure 6C, O-R are corresponding to Figure 6G. **H** Byproduct analysis of the untreated control group at the *HsHEK3* locus. High levels of these specific byproducts were consistently detected across all *HsHEK3* editing conditions. Their persistent presence in the untreated sample indicates that these signals represent inherent background noise rather than genuine PE-induced off-target or byproduct events.
