# Supplementary material for: Discovery and Engineering of a Rat Endogenous Retrovirus Reverse Transcriptase for Efficient Prime Editing
Source: Adv Sci (Weinh). 2026 Jun 26;13(49):e75888. doi: 10.1002/advs.75888 (PMC13335913; doi:10.1002/advs.75888)
Supplement: Supplementary file 6 [file ADVS-13-e75888-s008.docx]

**Data S1. Sequence information of plasmids for primer editors used in this study.**

**Sequence 1 Plasmids sequence of ePPEplus**

(NLSSV40-nCas9(H840A/R221K/N394K)-XTEN-NC-NLS-32aa Linker-M-MLV-RT-NLSvbp)

CCTAAGAAAAAGAGAAAAGTGGACAAGAAGTACTCGATCGGCCTCGATATTGGGACTAACTCTGTTGGCTGGGCCGTGATCACCGACGAGTACAAGGTGCCCTCAAAGAAGTTCAAGGTCCTGGGCAACACCGATCGGCATTCCATCAAGAAGAATCTCATTGGCGCTCTCCTGTTCGACAGCGGCGAGACGGCTGAGGCTACGCGGCTCAAGCGCACCGCCCGCAGGCGGTACACGCGCAGGAAGAATCGCATCTGCTACCTGCAGGAGATTTTCTCCAACGAGATGGCGAAGGTTGACGATTCTTTCTTCCACAGGCTGGAGGAGTCATTCCTCGTGGAGGAGGATAAGAAGCACGAGCGGCATCCAATCTTCGGCAACATTGTCGACGAGGTTGCCTACCACGAGAAGTACCCTACGATCTACCATCTGCGGAAGAAGCTCGTGGACTCCACAGATAAGGCGGACCTCCGCCTGATCTACCTCGCTCTGGCCCACATGATTAAGTTCAGGGGCCATTTCCTGATCGAGGGGGATCTCAACCCGGACAATAGCGATGTTGACAAGCTGTTCATCCAGCTCGTGCAGACGTACAACCAGCTCTTCGAGGAGAACCCCATTAATGCGTCAGGCGTCGACGCGAAGGCTATCCTGTCCGCTAGGCTCTCGAAGTCTCGGAAGCTCGAGAACCTGATCGCCCAGCTGCCGGGCGAGAAGAAGAACGGCCTGTTCGGGAATCTCATTGCGCTCAGCCTGGGGCTCACGCCCAACTTCAAGTCGAATTTCGATCTCGCTGAGGACGCCAAGCTGCAGCTCTCCAAGGACACATACGACGATGACCTGGATAACCTCCTGGCCCAGATCGGCGATCAGTACGCGGACCTGTTCCTCGCTGCCAAGAATCTGTCGGACGCCATCCTCCTGTCTGATATTCTCAGGGTGAACACCGAGATTACGAAGGCTCCGCTCTCAGCCTCCATGATCAAGCGCTACGACGAGCACCATCAGGATCTGACCCTCCTGAAGGCGCTGGTCAGGCAGCAGCTCCCCGAGAAGTACAAGGAGATCTTCTTCGATCAGTCGAAGAACGGCTACGCTGGGTACATTGACGGCGGGGCCTCTCAGGAGGAGTTCTACAAGTTCATCAAGCCGATTCTGGAGAAGATGGACGGCACGGAGGAGCTGCTGGTGAAGCTCAAGCGCGAGGACCTCCTGAGGAAGCAGCGGACATTCGATAACGGCAGCATCCCACACCAGATTCATCTCGGGGAGCTGCACGCTATCCTGAGGAGGCAGGAGGACTTCTACCCTTTCCTCAAGGATAACCGCGAGAAGATCGAGAAGATTCTGACTTTCAGGATCCCGTACTACGTCGGCCCACTCGCTAGGGGCAACTCCCGCTTCGCTTGGATGACCCGCAAGTCAGAGGAGACGATCACGCCGTGGAACTTCGAGGAGGTGGTCGACAAGGGCGCTAGCGCTCAGTCGTTCATCGAGAGGATGACGAATTTCGACAAGAACCTGCCAAATGAGAAGGTGCTCCCTAAGCACTCGCTCCTGTACGAGTACTTCACAGTCTACAACGAGCTGACTAAGGTGAAGTATGTGACCGAGGGCATGAGGAAGCCGGCTTTCCTGTCTGGGGAGCAGAAGAAGGCCATCGTGGACCTCCTGTTCAAGACCAACCGGAAGGTCACGGTTAAGCAGCTCAAGGAGGACTACTTCAAGAAGATTGAGTGCTTCGATTCGGTCGAGATCTCTGGCGTTGAGGACCGCTTCAACGCCTCCCTGGGGACCTACCACGATCTCCTGAAGATCATTAAGGATAAGGACTTCCTGGACAACGAGGAGAATGAGGATATCCTCGAGGACATTGTGCTGACACTCACTCTGTTCGAGGACCGGGAGATGATCGAGGAGCGCCTGAAGACTTACGCCCATCTCTTCGATGACAAGGTCATGAAGCAGCTCAAGAGGAGGAGGTACACCGGCTGGGGGAGGCTGAGCAGGAAGCTCATCAACGGCATTCGGGACAAGCAGTCCGGGAAGACGATCCTCGACTTCCTGAAGAGCGATGGCTTCGCGAACCGCAATTTCATGCAGCTGATTCACGATGACAGCCTCACATTCAAGGAGGATATCCAGAAGGCTCAGGTGAGCGGCCAGGGGGACTCGCTGCACGAGCATATCGCGAACCTCGCTGGCTCGCCAGCTATCAAGAAGGGGATTCTGCAGACCGTGAAGGTTGTGGACGAGCTGGTGAAGGTCATGGGCAGGCACAAGCCTGAGAACATCGTCATTGAGATGGCCCGGGAGAATCAGACCACGCAGAAGGGCCAGAAGAACTCACGCGAGAGGATGAAGAGGATCGAGGAGGGCATTAAGGAGCTGGGGTCCCAGATCCTCAAGGAGCACCCGGTGGAGAACACGCAGCTGCAGAATGAGAAGCTCTACCTGTACTACCTCCAGAATGGCCGCGATATGTATGTGGACCAGGAGCTGGATATTAACAGGCTCAGCGATTACGACGTCGATGCCATCGTTCCACAGTCATTCCTGAAGGATGACTCCATTGACAACAAGGTCCTCACCAGGTCGGACAAGAACCGGGGCAAGTCTGATAATGTTCCTTCAGAGGAGGTCGTTAAGAAGATGAAGAACTACTGGCGCCAGCTCCTGAATGCCAAGCTGATCACGCAGCGGAAGTTCGATAACCTCACAAAGGCTGAGAGGGGCGGGCTCTCTGAGCTGGACAAGGCGGGCTTCATCAAGAGGCAGCTGGTCGAGACACGGCAGATCACTAAGCACGTTGCGCAGATTCTCGACTCACGGATGAACACTAAGTACGATGAGAATGACAAGCTGATCCGCGAGGTGAAGGTCATCACCCTGAAGTCAAAGCTCGTCTCCGACTTCAGGAAGGATTTCCAGTTCTACAAGGTTCGGGAGATCAACAATTACCACCATGCCCATGACGCGTACCTGAACGCGGTGGTCGGCACAGCTCTGATCAAGAAGTACCCAAAGCTCGAGAGCGAGTTCGTGTACGGGGACTACAAGGTTTACGATGTGAGGAAGATGATCGCCAAGTCGGAGCAGGAGATTGGCAAGGCTACCGCCAAGTACTTCTTCTACTCTAACATTATGAATTTCTTCAAGACAGAGATCACTCTGGCCAATGGCGAGATCCGGAAGCGCCCCCTCATCGAGACGAACGGCGAGACGGGGGAGATCGTGTGGGACAAGGGCAGGGATTTCGCGACCGTCAGGAAGGTTCTCTCCATGCCACAAGTGAATATCGTCAAGAAGACAGAGGTCCAGACTGGCGGGTTCTCTAAGGAGTCAATTCTGCCTAAGCGGAACAGCGACAAGCTCATCGCCCGCAAGAAGGACTGGGATCCGAAGAAGTACGGCGGGTTCGACAGCCCCACTGTGGCCTACTCGGTCCTGGTTGTGGCGAAGGTTGAGAAGGGCAAGTCCAAGAAGCTCAAGAGCGTGAAGGAGCTGCTGGGGATCACGATTATGGAGCGCTCCAGCTTCGAGAAGAACCCGATCGATTTCCTGGAGGCGAAGGGCTACAAGGAGGTGAAGAAGGACCTGATCATTAAGCTCCCCAAGTACTCACTCTTCGAGCTGGAGAACGGCAGGAAGCGGATGCTGGCTTCCGCTGGCGAGCTGCAGAAGGGGAACGAGCTGGCTCTGCCGTCCAAGTATGTGAACTTCCTCTACCTGGCCTCCCACTACGAGAAGCTCAAGGGCAGCCCCGAGGACAACGAGCAGAAGCAGCTGTTCGTCGAGCAGCACAAGCATTACCTCGACGAGATCATTGAGCAGATTTCCGAGTTCTCCAAGCGCGTGATCCTGGCCGACGCGAATCTGGATAAGGTCCTCTCCGCGTACAACAAGCACCGCGACAAGCCAATCAGGGAGCAGGCTGAGAATATCATTCATCTCTTCACCCTGACGAACCTCGGCGCCCCTGCTGCTTTCAAGTACTTCGACACAACTATCGATCGCAAGAGGTACACAAGCACTAAGGAGGTCCTGGACGCGACCCTCATCCACCAGTCGATTACCGGCCTCTACGAGACGCGCATCGACCTGTCTCAGCTCGGGGGCGACGAATTCTCCGGGAGCGAGACGCCAGGCACCTCCGAGTCGGCCACCCCAGAATCTGCCACAGTGGTGTCCGGCCAAAAGCAGGACCGCCAGGGCGGAGAACGCAGAAGGTCCCAGCTCGATAGGGATCAGTGTGCCTACTGCAAGGAGAAGGGCCACTGGGCCAAAGACTGCCCGAAAAAGCCGCGCGGCCCACGCGGCCCAAGGCCACAAACATCCCTCCTTCCAAAGAAGAAGCGGAAGGTGGAGCTCAGCGGAGGATCTTCCGGAGGATCTAGCGGCTCCGAGACACCAGGAACATCCGAAAGCGCTACACCAGAATCTAGCGGAGGCTCTTCCGGAGGATCTAGGCCTACCCTCAACATCGAGGATGAGTATCGCCTCCACGAAACCTCCAAAGAACCGGACGTGTCCCTCGGCAGCACATGGCTCAGCGACTTCCCACAAGCGTGGGCCGAAACCGGCGGCATGGGCCTCGCCGTCCGCCAAGCCCCACTCATTATCCCGCTGAAGGCGACCTCCACACCGGTGTCCATCAAGCAGTACCCGATGAGCCAAGAGGCGAGGCTCGGGATTAAGCCGCACATTCAGCGCCTCCTCGATCAAGGCATTCTCGTGCCGTGCCAATCCCCGTGGAATACACCACTCCTCCCGGTCAAAAAGCCGGGCACCAACGACTATCGCCCGGTCCAAGATCTCCGCGAGGTCAACAAGCGCGTGGAAGACATCCACCCGACCGTCCCGAACCCGTATAATCTGCTCTCCGGGCTCCCACCATCCCACCAGTGGTATACAGTGCTGGACCTCAAAGACGCCTTCTTCTGTCTCCGCCTCCACCCAACAAGCCAGCCGCTCTTCGCCTTCGAGTGGCGCGACCCGGAGATGGGCATCTCCGGCCAACTGACATGGACACGCCTCCCGCAAGGCTTCAAGAACAGCCCGACACTCTTCAACGAGGCGCTCCATAGGGACCTCGCGGATTTTCGCATCCAGCATCCGGACCTCATCCTCCTCCAGTATGCGGATGATCTCCTCCTCGCCGCGACCTCCGAGCTGGATTGTCAACAAGGCACACGCGCGCTCCTCCAAACACTCGGGAACCTCGGCTATCGCGCGTCCGCGAAAAAGGCCCAAATCTGCCAGAAGCAAGTGAAGTACCTCGGGTATCTGCTCAAGGAAGGCCAACGCTGGCTCACCGAAGCGCGCAAAGAAACAGTGATGGGGCAACCGACACCGAAAACACCACGCCAGCTGCGCGAGTTTCTCGGCAAAGCCGGCTTCTGTCGCCTCTTCATCCCGGGCTTTGCCGAGATGGCCGCGCCACTCTACCCACTCACCAAGCCGGGCACACTGTTTAACTGGGGGCCGGATCAGCAGAAAGCCTACCAAGAGATCAAACAAGCGCTCCTCACCGCCCCAGCGCTCGGGCTCCCAGATCTCACAAAGCCGTTCGAGCTGTTCGTCGATGAGAAGCAAGGCTACGCGAAGGGCGTGCTCACACAGAAGCTCGGCCCGTGGAGGAGGCCAGTGGCCTATCTCTCCAAAAAACTCGATCCAGTGGCCGCCGGCTGGCCACCGTGTCTGCGCATGGTCGCCGCGATTGCCGTGCTCACAAAGGATGCCGGCAAACTCACAATGGGCCAGCCGCTGGTGATCCTCGCGCCACATGCCGTGGAAGCCCTCGTCAAACAGCCGCCGGATAGGTGGCTCTCCAATGCGCGCATGACCCATTACCAAGCGCTCCTCCTCGACACCGATCGCGTCCAGTTCGGCCCAGTGGTCGCCCTCAATCCGGCGACACTGCTGCCACTCCCAGAGGAGGGCCTCCAACACAACTGTCTGGATATTCTCGCGGAAGCGCATGGCACAAGGCCAGACCTCACAGATCAACCGCTCAGCGGCGGCAGCAAAAGAACGGCGGACGGCTCTGAGAAGCGCACCGCTGATAGCCAGCATTCAACTCCTCCGAAAACAAAGAGGAAAGTTGAGTTCGAACCGAAGAAGAAAAGGAAGGTGTGA

**Sequence 2 Plasmids sequence of ePPEplus-RT1**

(NLSSV40-nCas9(H840A/R221K/N394K)-XTEN-NC-NLS-32aa Linker-RT1-NLSvbp)

CCTAAGAAAAAGAGAAAAGTGGACAAGAAGTACTCGATCGGCCTCGATATTGGGACTAACTCTGTTGGCTGGGCCGTGATCACCGACGAGTACAAGGTGCCCTCAAAGAAGTTCAAGGTCCTGGGCAACACCGATCGGCATTCCATCAAGAAGAATCTCATTGGCGCTCTCCTGTTCGACAGCGGCGAGACGGCTGAGGCTACGCGGCTCAAGCGCACCGCCCGCAGGCGGTACACGCGCAGGAAGAATCGCATCTGCTACCTGCAGGAGATTTTCTCCAACGAGATGGCGAAGGTTGACGATTCTTTCTTCCACAGGCTGGAGGAGTCATTCCTCGTGGAGGAGGATAAGAAGCACGAGCGGCATCCAATCTTCGGCAACATTGTCGACGAGGTTGCCTACCACGAGAAGTACCCTACGATCTACCATCTGCGGAAGAAGCTCGTGGACTCCACAGATAAGGCGGACCTCCGCCTGATCTACCTCGCTCTGGCCCACATGATTAAGTTCAGGGGCCATTTCCTGATCGAGGGGGATCTCAACCCGGACAATAGCGATGTTGACAAGCTGTTCATCCAGCTCGTGCAGACGTACAACCAGCTCTTCGAGGAGAACCCCATTAATGCGTCAGGCGTCGACGCGAAGGCTATCCTGTCCGCTAGGCTCTCGAAGTCTCGGAAGCTCGAGAACCTGATCGCCCAGCTGCCGGGCGAGAAGAAGAACGGCCTGTTCGGGAATCTCATTGCGCTCAGCCTGGGGCTCACGCCCAACTTCAAGTCGAATTTCGATCTCGCTGAGGACGCCAAGCTGCAGCTCTCCAAGGACACATACGACGATGACCTGGATAACCTCCTGGCCCAGATCGGCGATCAGTACGCGGACCTGTTCCTCGCTGCCAAGAATCTGTCGGACGCCATCCTCCTGTCTGATATTCTCAGGGTGAACACCGAGATTACGAAGGCTCCGCTCTCAGCCTCCATGATCAAGCGCTACGACGAGCACCATCAGGATCTGACCCTCCTGAAGGCGCTGGTCAGGCAGCAGCTCCCCGAGAAGTACAAGGAGATCTTCTTCGATCAGTCGAAGAACGGCTACGCTGGGTACATTGACGGCGGGGCCTCTCAGGAGGAGTTCTACAAGTTCATCAAGCCGATTCTGGAGAAGATGGACGGCACGGAGGAGCTGCTGGTGAAGCTCAAGCGCGAGGACCTCCTGAGGAAGCAGCGGACATTCGATAACGGCAGCATCCCACACCAGATTCATCTCGGGGAGCTGCACGCTATCCTGAGGAGGCAGGAGGACTTCTACCCTTTCCTCAAGGATAACCGCGAGAAGATCGAGAAGATTCTGACTTTCAGGATCCCGTACTACGTCGGCCCACTCGCTAGGGGCAACTCCCGCTTCGCTTGGATGACCCGCAAGTCAGAGGAGACGATCACGCCGTGGAACTTCGAGGAGGTGGTCGACAAGGGCGCTAGCGCTCAGTCGTTCATCGAGAGGATGACGAATTTCGACAAGAACCTGCCAAATGAGAAGGTGCTCCCTAAGCACTCGCTCCTGTACGAGTACTTCACAGTCTACAACGAGCTGACTAAGGTGAAGTATGTGACCGAGGGCATGAGGAAGCCGGCTTTCCTGTCTGGGGAGCAGAAGAAGGCCATCGTGGACCTCCTGTTCAAGACCAACCGGAAGGTCACGGTTAAGCAGCTCAAGGAGGACTACTTCAAGAAGATTGAGTGCTTCGATTCGGTCGAGATCTCTGGCGTTGAGGACCGCTTCAACGCCTCCCTGGGGACCTACCACGATCTCCTGAAGATCATTAAGGATAAGGACTTCCTGGACAACGAGGAGAATGAGGATATCCTCGAGGACATTGTGCTGACACTCACTCTGTTCGAGGACCGGGAGATGATCGAGGAGCGCCTGAAGACTTACGCCCATCTCTTCGATGACAAGGTCATGAAGCAGCTCAAGAGGAGGAGGTACACCGGCTGGGGGAGGCTGAGCAGGAAGCTCATCAACGGCATTCGGGACAAGCAGTCCGGGAAGACGATCCTCGACTTCCTGAAGAGCGATGGCTTCGCGAACCGCAATTTCATGCAGCTGATTCACGATGACAGCCTCACATTCAAGGAGGATATCCAGAAGGCTCAGGTGAGCGGCCAGGGGGACTCGCTGCACGAGCATATCGCGAACCTCGCTGGCTCGCCAGCTATCAAGAAGGGGATTCTGCAGACCGTGAAGGTTGTGGACGAGCTGGTGAAGGTCATGGGCAGGCACAAGCCTGAGAACATCGTCATTGAGATGGCCCGGGAGAATCAGACCACGCAGAAGGGCCAGAAGAACTCACGCGAGAGGATGAAGAGGATCGAGGAGGGCATTAAGGAGCTGGGGTCCCAGATCCTCAAGGAGCACCCGGTGGAGAACACGCAGCTGCAGAATGAGAAGCTCTACCTGTACTACCTCCAGAATGGCCGCGATATGTATGTGGACCAGGAGCTGGATATTAACAGGCTCAGCGATTACGACGTCGATGCCATCGTTCCACAGTCATTCCTGAAGGATGACTCCATTGACAACAAGGTCCTCACCAGGTCGGACAAGAACCGGGGCAAGTCTGATAATGTTCCTTCAGAGGAGGTCGTTAAGAAGATGAAGAACTACTGGCGCCAGCTCCTGAATGCCAAGCTGATCACGCAGCGGAAGTTCGATAACCTCACAAAGGCTGAGAGGGGCGGGCTCTCTGAGCTGGACAAGGCGGGCTTCATCAAGAGGCAGCTGGTCGAGACACGGCAGATCACTAAGCACGTTGCGCAGATTCTCGACTCACGGATGAACACTAAGTACGATGAGAATGACAAGCTGATCCGCGAGGTGAAGGTCATCACCCTGAAGTCAAAGCTCGTCTCCGACTTCAGGAAGGATTTCCAGTTCTACAAGGTTCGGGAGATCAACAATTACCACCATGCCCATGACGCGTACCTGAACGCGGTGGTCGGCACAGCTCTGATCAAGAAGTACCCAAAGCTCGAGAGCGAGTTCGTGTACGGGGACTACAAGGTTTACGATGTGAGGAAGATGATCGCCAAGTCGGAGCAGGAGATTGGCAAGGCTACCGCCAAGTACTTCTTCTACTCTAACATTATGAATTTCTTCAAGACAGAGATCACTCTGGCCAATGGCGAGATCCGGAAGCGCCCCCTCATCGAGACGAACGGCGAGACGGGGGAGATCGTGTGGGACAAGGGCAGGGATTTCGCGACCGTCAGGAAGGTTCTCTCCATGCCACAAGTGAATATCGTCAAGAAGACAGAGGTCCAGACTGGCGGGTTCTCTAAGGAGTCAATTCTGCCTAAGCGGAACAGCGACAAGCTCATCGCCCGCAAGAAGGACTGGGATCCGAAGAAGTACGGCGGGTTCGACAGCCCCACTGTGGCCTACTCGGTCCTGGTTGTGGCGAAGGTTGAGAAGGGCAAGTCCAAGAAGCTCAAGAGCGTGAAGGAGCTGCTGGGGATCACGATTATGGAGCGCTCCAGCTTCGAGAAGAACCCGATCGATTTCCTGGAGGCGAAGGGCTACAAGGAGGTGAAGAAGGACCTGATCATTAAGCTCCCCAAGTACTCACTCTTCGAGCTGGAGAACGGCAGGAAGCGGATGCTGGCTTCCGCTGGCGAGCTGCAGAAGGGGAACGAGCTGGCTCTGCCGTCCAAGTATGTGAACTTCCTCTACCTGGCCTCCCACTACGAGAAGCTCAAGGGCAGCCCCGAGGACAACGAGCAGAAGCAGCTGTTCGTCGAGCAGCACAAGCATTACCTCGACGAGATCATTGAGCAGATTTCCGAGTTCTCCAAGCGCGTGATCCTGGCCGACGCGAATCTGGATAAGGTCCTCTCCGCGTACAACAAGCACCGCGACAAGCCAATCAGGGAGCAGGCTGAGAATATCATTCATCTCTTCACCCTGACGAACCTCGGCGCCCCTGCTGCTTTCAAGTACTTCGACACAACTATCGATCGCAAGAGGTACACAAGCACTAAGGAGGTCCTGGACGCGACCCTCATCCACCAGTCGATTACCGGCCTCTACGAGACGCGCATCGACCTGTCTCAGCTCGGGGGCGACGAATTCTCCGGGAGCGAGACGCCAGGCACCTCCGAGTCGGCCACCCCAGAATCTGCCACAGTGGTGTCCGGCCAAAAGCAGGACCGCCAGGGCGGAGAACGCAGAAGGTCCCAGCTCGATAGGGATCAGTGTGCCTACTGCAAGGAGAAGGGCCACTGGGCCAAAGACTGCCCGAAAAAGCCGCGCGGCCCACGCGGCCCAAGGCCACAAACATCCCTCCTTCCAAAGAAGAAGCGGAAGGTGGAGCTCAGCGGAGGATCTTCCGGAGGATCTAGCGGCTCCGAGACACCAGGAACATCCGAAAGCGCTACACCAGAATCTAGCGGAGGCTCTTCCGGAGGATCTAGGCCTGCCCTGCTGGAAAATATTTTGTCTAGATCTAACTTGACCAAAGCTCTAAAAAGAGTAGAGGCGAACAAGGGCGCCCCCGGCATGGATGGAGTGAGCACTGAGCACCTCAGAGACTACCTTAGGGAGCACTGGCCAGCCATCAAACAAAAGCTCCTGGAGGGAACCTACCAGCCAGCCCCTGTGCGCAGAGTGGAGATACCAAAACCTGACGGCGGCGTGAGACTACTGGGCATTCCTACCGTCATCGACAGGTTTATACAGCAGGCTATCCTTCAGGTGCTCACGCCTATCTTTGACCCACACTTCTCATCTCACTCCTACGGCTTCCGTCCGCAGAGAAGGGCACATGATGCCGTGAGACAGGCGCAGCGCTACATTCATGAAGGATACAAGTATGTTGTGGACATCGATCTGGAGAAATTCTTCGACCGCGTCCACCACGATATCCTTATGTCGAGGGTGGCACGGAGAGTCAAGGATAAGCGGGTACTCAAGCTGATTAGAGCTTACTTGAAGGCCGGCATTATGATCGCCGGCATTAAGGTTAGATCCGAAGAAGGAACCCCCCAAGGGGGCACCCTGTCCCCCCTGCTTGCAAACATCCTCTTAGATGATCTCGACAAGGAGCTGGAGAAGCGCGGCCTGCGGTTCTGTAGATACGCCGACGACTGCAACATCTATGCTAGGAGCCGTAGAGCGGGCCAGCGGGTGAAGCAGAGCATTCAGAAGTTCTTGGAAAAGAAATTGAAGCTGAAGGTGAATGAAGAGAAGAGCGCCGTCGACCGGCCATGGCGAAGGAAGTTTCTTGGTTTCTCCTTCACAAGCCAGAGGCAAGCTCGCATCAGACTCGCCCCTCAGTCGATTCAAAGATTCAAGAACAAAATTAGGCAGCTCACCAATCCTAACTGGAGCATCTCAATGGAGGAGCGCATAAAGAAGTTGAACCAGTACATGATGGGGTGGATAGGTTACTTCGCCTTAATCGAGACCCCGAGCCCGCTGAAGCGCCTGGAGGAGTGGATCCGGAGACGGCTGAGACTCTGCAGGTGGCACCAATGGAAGAGAGTGCGGACCCGTATCCGGGAGCTCCGCGCCCTGGGCCTCAAGGAACATGAGGTGTTTGAAATCGCAAACACAAGAAAGGGCGCGTGGAGGACAACAAAAACACCCCAGTTGCACAAGGCCCTGTCAAAGGCTTACTGGCTCGCCCAGGGGCTGCGTAGCCTGACCGAAAGATATTTTGATGTGAGACAAGATTGGCGCACTGCCAGCGGCGGCAGCAAAAGAACGGCGGACGGCTCTGAGAAGCGCACCGCTGATAGCCAGCATTCAACTCCTCCGAAAACAAAGAGGAAAGTTGAGTTCGAACCGAAGAAGAAAAGGAAGGTGTGA

**Sequence 3 Plasmids sequence of ePPEplus-RT2**

(NLSSV40-nCas9(H840A/R221K/N394K)-XTEN-NC-NLS-32aa Linker-RT2-NLSvbp)

CCTAAGAAAAAGAGAAAAGTGGACAAGAAGTACTCGATCGGCCTCGATATTGGGACTAACTCTGTTGGCTGGGCCGTGATCACCGACGAGTACAAGGTGCCCTCAAAGAAGTTCAAGGTCCTGGGCAACACCGATCGGCATTCCATCAAGAAGAATCTCATTGGCGCTCTCCTGTTCGACAGCGGCGAGACGGCTGAGGCTACGCGGCTCAAGCGCACCGCCCGCAGGCGGTACACGCGCAGGAAGAATCGCATCTGCTACCTGCAGGAGATTTTCTCCAACGAGATGGCGAAGGTTGACGATTCTTTCTTCCACAGGCTGGAGGAGTCATTCCTCGTGGAGGAGGATAAGAAGCACGAGCGGCATCCAATCTTCGGCAACATTGTCGACGAGGTTGCCTACCACGAGAAGTACCCTACGATCTACCATCTGCGGAAGAAGCTCGTGGACTCCACAGATAAGGCGGACCTCCGCCTGATCTACCTCGCTCTGGCCCACATGATTAAGTTCAGGGGCCATTTCCTGATCGAGGGGGATCTCAACCCGGACAATAGCGATGTTGACAAGCTGTTCATCCAGCTCGTGCAGACGTACAACCAGCTCTTCGAGGAGAACCCCATTAATGCGTCAGGCGTCGACGCGAAGGCTATCCTGTCCGCTAGGCTCTCGAAGTCTCGGAAGCTCGAGAACCTGATCGCCCAGCTGCCGGGCGAGAAGAAGAACGGCCTGTTCGGGAATCTCATTGCGCTCAGCCTGGGGCTCACGCCCAACTTCAAGTCGAATTTCGATCTCGCTGAGGACGCCAAGCTGCAGCTCTCCAAGGACACATACGACGATGACCTGGATAACCTCCTGGCCCAGATCGGCGATCAGTACGCGGACCTGTTCCTCGCTGCCAAGAATCTGTCGGACGCCATCCTCCTGTCTGATATTCTCAGGGTGAACACCGAGATTACGAAGGCTCCGCTCTCAGCCTCCATGATCAAGCGCTACGACGAGCACCATCAGGATCTGACCCTCCTGAAGGCGCTGGTCAGGCAGCAGCTCCCCGAGAAGTACAAGGAGATCTTCTTCGATCAGTCGAAGAACGGCTACGCTGGGTACATTGACGGCGGGGCCTCTCAGGAGGAGTTCTACAAGTTCATCAAGCCGATTCTGGAGAAGATGGACGGCACGGAGGAGCTGCTGGTGAAGCTCAAGCGCGAGGACCTCCTGAGGAAGCAGCGGACATTCGATAACGGCAGCATCCCACACCAGATTCATCTCGGGGAGCTGCACGCTATCCTGAGGAGGCAGGAGGACTTCTACCCTTTCCTCAAGGATAACCGCGAGAAGATCGAGAAGATTCTGACTTTCAGGATCCCGTACTACGTCGGCCCACTCGCTAGGGGCAACTCCCGCTTCGCTTGGATGACCCGCAAGTCAGAGGAGACGATCACGCCGTGGAACTTCGAGGAGGTGGTCGACAAGGGCGCTAGCGCTCAGTCGTTCATCGAGAGGATGACGAATTTCGACAAGAACCTGCCAAATGAGAAGGTGCTCCCTAAGCACTCGCTCCTGTACGAGTACTTCACAGTCTACAACGAGCTGACTAAGGTGAAGTATGTGACCGAGGGCATGAGGAAGCCGGCTTTCCTGTCTGGGGAGCAGAAGAAGGCCATCGTGGACCTCCTGTTCAAGACCAACCGGAAGGTCACGGTTAAGCAGCTCAAGGAGGACTACTTCAAGAAGATTGAGTGCTTCGATTCGGTCGAGATCTCTGGCGTTGAGGACCGCTTCAACGCCTCCCTGGGGACCTACCACGATCTCCTGAAGATCATTAAGGATAAGGACTTCCTGGACAACGAGGAGAATGAGGATATCCTCGAGGACATTGTGCTGACACTCACTCTGTTCGAGGACCGGGAGATGATCGAGGAGCGCCTGAAGACTTACGCCCATCTCTTCGATGACAAGGTCATGAAGCAGCTCAAGAGGAGGAGGTACACCGGCTGGGGGAGGCTGAGCAGGAAGCTCATCAACGGCATTCGGGACAAGCAGTCCGGGAAGACGATCCTCGACTTCCTGAAGAGCGATGGCTTCGCGAACCGCAATTTCATGCAGCTGATTCACGATGACAGCCTCACATTCAAGGAGGATATCCAGAAGGCTCAGGTGAGCGGCCAGGGGGACTCGCTGCACGAGCATATCGCGAACCTCGCTGGCTCGCCAGCTATCAAGAAGGGGATTCTGCAGACCGTGAAGGTTGTGGACGAGCTGGTGAAGGTCATGGGCAGGCACAAGCCTGAGAACATCGTCATTGAGATGGCCCGGGAGAATCAGACCACGCAGAAGGGCCAGAAGAACTCACGCGAGAGGATGAAGAGGATCGAGGAGGGCATTAAGGAGCTGGGGTCCCAGATCCTCAAGGAGCACCCGGTGGAGAACACGCAGCTGCAGAATGAGAAGCTCTACCTGTACTACCTCCAGAATGGCCGCGATATGTATGTGGACCAGGAGCTGGATATTAACAGGCTCAGCGATTACGACGTCGATGCCATCGTTCCACAGTCATTCCTGAAGGATGACTCCATTGACAACAAGGTCCTCACCAGGTCGGACAAGAACCGGGGCAAGTCTGATAATGTTCCTTCAGAGGAGGTCGTTAAGAAGATGAAGAACTACTGGCGCCAGCTCCTGAATGCCAAGCTGATCACGCAGCGGAAGTTCGATAACCTCACAAAGGCTGAGAGGGGCGGGCTCTCTGAGCTGGACAAGGCGGGCTTCATCAAGAGGCAGCTGGTCGAGACACGGCAGATCACTAAGCACGTTGCGCAGATTCTCGACTCACGGATGAACACTAAGTACGATGAGAATGACAAGCTGATCCGCGAGGTGAAGGTCATCACCCTGAAGTCAAAGCTCGTCTCCGACTTCAGGAAGGATTTCCAGTTCTACAAGGTTCGGGAGATCAACAATTACCACCATGCCCATGACGCGTACCTGAACGCGGTGGTCGGCACAGCTCTGATCAAGAAGTACCCAAAGCTCGAGAGCGAGTTCGTGTACGGGGACTACAAGGTTTACGATGTGAGGAAGATGATCGCCAAGTCGGAGCAGGAGATTGGCAAGGCTACCGCCAAGTACTTCTTCTACTCTAACATTATGAATTTCTTCAAGACAGAGATCACTCTGGCCAATGGCGAGATCCGGAAGCGCCCCCTCATCGAGACGAACGGCGAGACGGGGGAGATCGTGTGGGACAAGGGCAGGGATTTCGCGACCGTCAGGAAGGTTCTCTCCATGCCACAAGTGAATATCGTCAAGAAGACAGAGGTCCAGACTGGCGGGTTCTCTAAGGAGTCAATTCTGCCTAAGCGGAACAGCGACAAGCTCATCGCCCGCAAGAAGGACTGGGATCCGAAGAAGTACGGCGGGTTCGACAGCCCCACTGTGGCCTACTCGGTCCTGGTTGTGGCGAAGGTTGAGAAGGGCAAGTCCAAGAAGCTCAAGAGCGTGAAGGAGCTGCTGGGGATCACGATTATGGAGCGCTCCAGCTTCGAGAAGAACCCGATCGATTTCCTGGAGGCGAAGGGCTACAAGGAGGTGAAGAAGGACCTGATCATTAAGCTCCCCAAGTACTCACTCTTCGAGCTGGAGAACGGCAGGAAGCGGATGCTGGCTTCCGCTGGCGAGCTGCAGAAGGGGAACGAGCTGGCTCTGCCGTCCAAGTATGTGAACTTCCTCTACCTGGCCTCCCACTACGAGAAGCTCAAGGGCAGCCCCGAGGACAACGAGCAGAAGCAGCTGTTCGTCGAGCAGCACAAGCATTACCTCGACGAGATCATTGAGCAGATTTCCGAGTTCTCCAAGCGCGTGATCCTGGCCGACGCGAATCTGGATAAGGTCCTCTCCGCGTACAACAAGCACCGCGACAAGCCAATCAGGGAGCAGGCTGAGAATATCATTCATCTCTTCACCCTGACGAACCTCGGCGCCCCTGCTGCTTTCAAGTACTTCGACACAACTATCGATCGCAAGAGGTACACAAGCACTAAGGAGGTCCTGGACGCGACCCTCATCCACCAGTCGATTACCGGCCTCTACGAGACGCGCATCGACCTGTCTCAGCTCGGGGGCGACGAATTCTCCGGGAGCGAGACGCCAGGCACCTCCGAGTCGGCCACCCCAGAATCTGCCACAGTGGTGTCCGGCCAAAAGCAGGACCGCCAGGGCGGAGAACGCAGAAGGTCCCAGCTCGATAGGGATCAGTGTGCCTACTGCAAGGAGAAGGGCCACTGGGCCAAAGACTGCCCGAAAAAGCCGCGCGGCCCACGCGGCCCAAGGCCACAAACATCCCTCCTTCCAAAGAAGAAGCGGAAGGTGGAGCTCAGCGGAGGATCTTCCGGAGGATCTAGCGGCTCCGAGACACCAGGAACATCCGAAAGCGCTACACCAGAATCTAGCGGAGGCTCTTCCGGAGGATCTAGGCCTAACCTGGTGTCAAAATTGGCACTGTATTTGGAAAAGAGTGAGTCTGAAGTCATCCGCTTCCTCTCTGATGCACCAAATAAATATCGAGTGTACAAGATTCCAAAGAGATCTCATGGCCATAGGATTATAGCTCAACCTTCTAAGGAACTGAAGGAGTACCAGCGGGCGTTCTTGGAGCTGTACGAGTTCCCCGTCCACGACAGCGCCATGGCGTACTGCAAGGGGAAGGGCATCAAAGAAAATGCTCTCGCCCACAGCAAGAACTCGTACCTTCTCAAGACTGACCTCGAGAATTTTTTCAACAGCATCACGCCGCCGATTTTCTGGCAGTGCATGGAGAAGTGCGCCTCCACAGTGCCGCAGTTCACCAAGCAGGAAAAGGTGCTTGTGGAGAAATTGATTTTTTGGTGCCCTTCCAAGGATCGGAACGGCAAGCTTGTCCTCTCTATTGGAGCACCTTCGTCGCCCGCCATCAGCAACTTTTGTTTATATCAATTTGACGACTTTATGTCAGATGTTTGTTATGATCACAAGATTACTTATACTCGCTATGCTGATGATCTTACCTTTTCAACAAATGAAAAAGACCTGCTCCACACCATCATCAATTCAATTCAGTACTCCTTGAACTACTTCTTCTCCAATTCTTTAAAACTGAATCATAGTAAAACGGTGTTCAGCTCGCGTGCGCACAACCGCCATGTTACTGGGATAACAATCAACAACCATGGTAGGTTGTCCCTCGGCAGGGAGAGGAAGAGGTACATCAAGCACCTTGTAAATCAATTCAAGTACAACCAGCTATCCGAGTCCGACATCTTCCACCTGCAGGGTCTTCTCTCATTCGCCAGACATATTGAACCAAAATTTATATTTAGACTGAAAGATAAATACACCAACGAGCTGGTTCAACGGATATATGAGGCGGGAAATGAGCAGCAGAACAAGAAGCAAAGCGGCGGCAGCAAAAGAACGGCGGACGGCTCTGAGAAGCGCACCGCTGATAGCCAGCATTCAACTCCTCCGAAAACAAAGAGGAAAGTTGAGTTCGAACCGAAGAAGAAAAGGAAGGTGTGA

**Sequence 4 Plasmids sequence of ePPEplus-RT3**

(NLSSV40-nCas9(H840A/R221K/N394K)-XTEN-NC-NLS-32aa Linker-RT3-NLSvbp)

CCTAAGAAAAAGAGAAAAGTGGACAAGAAGTACTCGATCGGCCTCGATATTGGGACTAACTCTGTTGGCTGGGCCGTGATCACCGACGAGTACAAGGTGCCCTCAAAGAAGTTCAAGGTCCTGGGCAACACCGATCGGCATTCCATCAAGAAGAATCTCATTGGCGCTCTCCTGTTCGACAGCGGCGAGACGGCTGAGGCTACGCGGCTCAAGCGCACCGCCCGCAGGCGGTACACGCGCAGGAAGAATCGCATCTGCTACCTGCAGGAGATTTTCTCCAACGAGATGGCGAAGGTTGACGATTCTTTCTTCCACAGGCTGGAGGAGTCATTCCTCGTGGAGGAGGATAAGAAGCACGAGCGGCATCCAATCTTCGGCAACATTGTCGACGAGGTTGCCTACCACGAGAAGTACCCTACGATCTACCATCTGCGGAAGAAGCTCGTGGACTCCACAGATAAGGCGGACCTCCGCCTGATCTACCTCGCTCTGGCCCACATGATTAAGTTCAGGGGCCATTTCCTGATCGAGGGGGATCTCAACCCGGACAATAGCGATGTTGACAAGCTGTTCATCCAGCTCGTGCAGACGTACAACCAGCTCTTCGAGGAGAACCCCATTAATGCGTCAGGCGTCGACGCGAAGGCTATCCTGTCCGCTAGGCTCTCGAAGTCTCGGAAGCTCGAGAACCTGATCGCCCAGCTGCCGGGCGAGAAGAAGAACGGCCTGTTCGGGAATCTCATTGCGCTCAGCCTGGGGCTCACGCCCAACTTCAAGTCGAATTTCGATCTCGCTGAGGACGCCAAGCTGCAGCTCTCCAAGGACACATACGACGATGACCTGGATAACCTCCTGGCCCAGATCGGCGATCAGTACGCGGACCTGTTCCTCGCTGCCAAGAATCTGTCGGACGCCATCCTCCTGTCTGATATTCTCAGGGTGAACACCGAGATTACGAAGGCTCCGCTCTCAGCCTCCATGATCAAGCGCTACGACGAGCACCATCAGGATCTGACCCTCCTGAAGGCGCTGGTCAGGCAGCAGCTCCCCGAGAAGTACAAGGAGATCTTCTTCGATCAGTCGAAGAACGGCTACGCTGGGTACATTGACGGCGGGGCCTCTCAGGAGGAGTTCTACAAGTTCATCAAGCCGATTCTGGAGAAGATGGACGGCACGGAGGAGCTGCTGGTGAAGCTCAAGCGCGAGGACCTCCTGAGGAAGCAGCGGACATTCGATAACGGCAGCATCCCACACCAGATTCATCTCGGGGAGCTGCACGCTATCCTGAGGAGGCAGGAGGACTTCTACCCTTTCCTCAAGGATAACCGCGAGAAGATCGAGAAGATTCTGACTTTCAGGATCCCGTACTACGTCGGCCCACTCGCTAGGGGCAACTCCCGCTTCGCTTGGATGACCCGCAAGTCAGAGGAGACGATCACGCCGTGGAACTTCGAGGAGGTGGTCGACAAGGGCGCTAGCGCTCAGTCGTTCATCGAGAGGATGACGAATTTCGACAAGAACCTGCCAAATGAGAAGGTGCTCCCTAAGCACTCGCTCCTGTACGAGTACTTCACAGTCTACAACGAGCTGACTAAGGTGAAGTATGTGACCGAGGGCATGAGGAAGCCGGCTTTCCTGTCTGGGGAGCAGAAGAAGGCCATCGTGGACCTCCTGTTCAAGACCAACCGGAAGGTCACGGTTAAGCAGCTCAAGGAGGACTACTTCAAGAAGATTGAGTGCTTCGATTCGGTCGAGATCTCTGGCGTTGAGGACCGCTTCAACGCCTCCCTGGGGACCTACCACGATCTCCTGAAGATCATTAAGGATAAGGACTTCCTGGACAACGAGGAGAATGAGGATATCCTCGAGGACATTGTGCTGACACTCACTCTGTTCGAGGACCGGGAGATGATCGAGGAGCGCCTGAAGACTTACGCCCATCTCTTCGATGACAAGGTCATGAAGCAGCTCAAGAGGAGGAGGTACACCGGCTGGGGGAGGCTGAGCAGGAAGCTCATCAACGGCATTCGGGACAAGCAGTCCGGGAAGACGATCCTCGACTTCCTGAAGAGCGATGGCTTCGCGAACCGCAATTTCATGCAGCTGATTCACGATGACAGCCTCACATTCAAGGAGGATATCCAGAAGGCTCAGGTGAGCGGCCAGGGGGACTCGCTGCACGAGCATATCGCGAACCTCGCTGGCTCGCCAGCTATCAAGAAGGGGATTCTGCAGACCGTGAAGGTTGTGGACGAGCTGGTGAAGGTCATGGGCAGGCACAAGCCTGAGAACATCGTCATTGAGATGGCCCGGGAGAATCAGACCACGCAGAAGGGCCAGAAGAACTCACGCGAGAGGATGAAGAGGATCGAGGAGGGCATTAAGGAGCTGGGGTCCCAGATCCTCAAGGAGCACCCGGTGGAGAACACGCAGCTGCAGAATGAGAAGCTCTACCTGTACTACCTCCAGAATGGCCGCGATATGTATGTGGACCAGGAGCTGGATATTAACAGGCTCAGCGATTACGACGTCGATGCCATCGTTCCACAGTCATTCCTGAAGGATGACTCCATTGACAACAAGGTCCTCACCAGGTCGGACAAGAACCGGGGCAAGTCTGATAATGTTCCTTCAGAGGAGGTCGTTAAGAAGATGAAGAACTACTGGCGCCAGCTCCTGAATGCCAAGCTGATCACGCAGCGGAAGTTCGATAACCTCACAAAGGCTGAGAGGGGCGGGCTCTCTGAGCTGGACAAGGCGGGCTTCATCAAGAGGCAGCTGGTCGAGACACGGCAGATCACTAAGCACGTTGCGCAGATTCTCGACTCACGGATGAACACTAAGTACGATGAGAATGACAAGCTGATCCGCGAGGTGAAGGTCATCACCCTGAAGTCAAAGCTCGTCTCCGACTTCAGGAAGGATTTCCAGTTCTACAAGGTTCGGGAGATCAACAATTACCACCATGCCCATGACGCGTACCTGAACGCGGTGGTCGGCACAGCTCTGATCAAGAAGTACCCAAAGCTCGAGAGCGAGTTCGTGTACGGGGACTACAAGGTTTACGATGTGAGGAAGATGATCGCCAAGTCGGAGCAGGAGATTGGCAAGGCTACCGCCAAGTACTTCTTCTACTCTAACATTATGAATTTCTTCAAGACAGAGATCACTCTGGCCAATGGCGAGATCCGGAAGCGCCCCCTCATCGAGACGAACGGCGAGACGGGGGAGATCGTGTGGGACAAGGGCAGGGATTTCGCGACCGTCAGGAAGGTTCTCTCCATGCCACAAGTGAATATCGTCAAGAAGACAGAGGTCCAGACTGGCGGGTTCTCTAAGGAGTCAATTCTGCCTAAGCGGAACAGCGACAAGCTCATCGCCCGCAAGAAGGACTGGGATCCGAAGAAGTACGGCGGGTTCGACAGCCCCACTGTGGCCTACTCGGTCCTGGTTGTGGCGAAGGTTGAGAAGGGCAAGTCCAAGAAGCTCAAGAGCGTGAAGGAGCTGCTGGGGATCACGATTATGGAGCGCTCCAGCTTCGAGAAGAACCCGATCGATTTCCTGGAGGCGAAGGGCTACAAGGAGGTGAAGAAGGACCTGATCATTAAGCTCCCCAAGTACTCACTCTTCGAGCTGGAGAACGGCAGGAAGCGGATGCTGGCTTCCGCTGGCGAGCTGCAGAAGGGGAACGAGCTGGCTCTGCCGTCCAAGTATGTGAACTTCCTCTACCTGGCCTCCCACTACGAGAAGCTCAAGGGCAGCCCCGAGGACAACGAGCAGAAGCAGCTGTTCGTCGAGCAGCACAAGCATTACCTCGACGAGATCATTGAGCAGATTTCCGAGTTCTCCAAGCGCGTGATCCTGGCCGACGCGAATCTGGATAAGGTCCTCTCCGCGTACAACAAGCACCGCGACAAGCCAATCAGGGAGCAGGCTGAGAATATCATTCATCTCTTCACCCTGACGAACCTCGGCGCCCCTGCTGCTTTCAAGTACTTCGACACAACTATCGATCGCAAGAGGTACACAAGCACTAAGGAGGTCCTGGACGCGACCCTCATCCACCAGTCGATTACCGGCCTCTACGAGACGCGCATCGACCTGTCTCAGCTCGGGGGCGACGAATTCTCCGGGAGCGAGACGCCAGGCACCTCCGAGTCGGCCACCCCAGAATCTGCCACAGTGGTGTCCGGCCAAAAGCAGGACCGCCAGGGCGGAGAACGCAGAAGGTCCCAGCTCGATAGGGATCAGTGTGCCTACTGCAAGGAGAAGGGCCACTGGGCCAAAGACTGCCCGAAAAAGCCGCGCGGCCCACGCGGCCCAAGGCCACAAACATCCCTCCTTCCAAAGAAGAAGCGGAAGGTGGAGCTCAGCGGAGGATCTTCCGGAGGATCTAGCGGCTCCGAGACACCAGGAACATCCGAAAGCGCTACACCAGAATCTAGCGGAGGCTCTTCCGGAGGATCTAGGCCTCCTGACACTTCCTCCCTCATGAAGCAGATACTGTCCAGCGACAATCTGAACCGTGCCTACCTCCAGGTGGTGCGGAACAAGGGAGCAGAGGGCGTTGACGGAATGAAATACACCGAGCTCAAGGAGCACCTCGTGAAGGATGGAGAAATCATCAAGGAGCAACTGCGTACCCGGAAGTACAAACCACAGCCAGTGCGGAGAGTTGAAATCCCTAAGCCTGACGGCGGCGTGCGCAACCTGGGCGTGCCTACCGTGACAGATAGACTGATCCAGCAAGCTATCGCGCAGGTTTTGACCCCCATCTATGAGGAGCAGTTCCACGACCACAGCTATGGGTTCAGACCCAACCGGTGTGCACAGCAGGCAATCCTGACCGCCCTCGACATGATGAATGATGGGAACGACTGGATCGTTGATATTGATCTCGAGAAGTTCTTCGACACGGTGAATCATGATAAATTGATGACGATCATTGGCCGCACCATTAAAGATGGCGACGTGATCAGCATAGTGCGCAAGTACCTGGTGAGCGGCATCATGATTGATGATGAATATGAGGACAGCATCGTCGGCACTCCTCAGGGAGGCAACCTCTCTCCACTCCTCGCCAATATAATGTTGAATGAACTTGACAAGGAAATGGAGAAGCGCGGCCTGAACTTTGTGAGGTACGCCGATGACTGCATTATCATGGTGGGCTCAGAGATGTCGGCCAACAGAGTGATGAGAAACATTTCACGGTTCATCGAAGAAAAACTGGGTTTGAAAGTTAACATGACAAAGAGCAAAGTCGACCGCCCCTCTGGCCTTAAGTATCTGGGCTTCGGGTTCTACTTTGACAGCCGAGCTCACCAATTTAAAGCAAAGCCGCACGCCAAGTCTGTGGCGAAGTTTAAGAAGAGGATGAAAGGTCTGACCTGCCGGTCATGGGGTGTCAGCAATTCCTACAAGGTGGAGAAGCTCAACCAGCTCATCAGAGGATGGATTAACTACTTCAAAATCGGTTCCATGAAGATTCTCTGTGCGAAGCTGGATGCTAACATCCGGTATCGCCTGCGCATGTGCATTTGGAAGCATTGGAAAACACCCCAGAATCGGGCCAAGAATCTGATGAAGCTGGGCATGGATAGAATAACGGCCTACAAAGTTGGGTATTGCAGTAGAGCCTACGCGCACGTCTGCAGCTGCGGCGCCGTGAACATTGCCATCACCAACAAAAGACTGGCCTCCTTCGGGCTGATCAGTATGCTGGACTACTACACAGAAAGATGTGTGACTTGTAGCGGCGGCAGCAAAAGAACGGCGGACGGCTCTGAGAAGCGCACCGCTGATAGCCAGCATTCAACTCCTCCGAAAACAAAGAGGAAAGTTGAGTTCGAACCGAAGAAGAAAAGGAAGGTGTGA

**Sequence 5 Plasmids sequence of ePPEplus-RT4**

(NLSSV40-nCas9(H840A/R221K/N394K)-XTEN-NC-NLS-32aa Linker-RT4-NLSvbp)

CCTAAGAAAAAGAGAAAAGTGGACAAGAAGTACTCGATCGGCCTCGATATTGGGACTAACTCTGTTGGCTGGGCCGTGATCACCGACGAGTACAAGGTGCCCTCAAAGAAGTTCAAGGTCCTGGGCAACACCGATCGGCATTCCATCAAGAAGAATCTCATTGGCGCTCTCCTGTTCGACAGCGGCGAGACGGCTGAGGCTACGCGGCTCAAGCGCACCGCCCGCAGGCGGTACACGCGCAGGAAGAATCGCATCTGCTACCTGCAGGAGATTTTCTCCAACGAGATGGCGAAGGTTGACGATTCTTTCTTCCACAGGCTGGAGGAGTCATTCCTCGTGGAGGAGGATAAGAAGCACGAGCGGCATCCAATCTTCGGCAACATTGTCGACGAGGTTGCCTACCACGAGAAGTACCCTACGATCTACCATCTGCGGAAGAAGCTCGTGGACTCCACAGATAAGGCGGACCTCCGCCTGATCTACCTCGCTCTGGCCCACATGATTAAGTTCAGGGGCCATTTCCTGATCGAGGGGGATCTCAACCCGGACAATAGCGATGTTGACAAGCTGTTCATCCAGCTCGTGCAGACGTACAACCAGCTCTTCGAGGAGAACCCCATTAATGCGTCAGGCGTCGACGCGAAGGCTATCCTGTCCGCTAGGCTCTCGAAGTCTCGGAAGCTCGAGAACCTGATCGCCCAGCTGCCGGGCGAGAAGAAGAACGGCCTGTTCGGGAATCTCATTGCGCTCAGCCTGGGGCTCACGCCCAACTTCAAGTCGAATTTCGATCTCGCTGAGGACGCCAAGCTGCAGCTCTCCAAGGACACATACGACGATGACCTGGATAACCTCCTGGCCCAGATCGGCGATCAGTACGCGGACCTGTTCCTCGCTGCCAAGAATCTGTCGGACGCCATCCTCCTGTCTGATATTCTCAGGGTGAACACCGAGATTACGAAGGCTCCGCTCTCAGCCTCCATGATCAAGCGCTACGACGAGCACCATCAGGATCTGACCCTCCTGAAGGCGCTGGTCAGGCAGCAGCTCCCCGAGAAGTACAAGGAGATCTTCTTCGATCAGTCGAAGAACGGCTACGCTGGGTACATTGACGGCGGGGCCTCTCAGGAGGAGTTCTACAAGTTCATCAAGCCGATTCTGGAGAAGATGGACGGCACGGAGGAGCTGCTGGTGAAGCTCAAGCGCGAGGACCTCCTGAGGAAGCAGCGGACATTCGATAACGGCAGCATCCCACACCAGATTCATCTCGGGGAGCTGCACGCTATCCTGAGGAGGCAGGAGGACTTCTACCCTTTCCTCAAGGATAACCGCGAGAAGATCGAGAAGATTCTGACTTTCAGGATCCCGTACTACGTCGGCCCACTCGCTAGGGGCAACTCCCGCTTCGCTTGGATGACCCGCAAGTCAGAGGAGACGATCACGCCGTGGAACTTCGAGGAGGTGGTCGACAAGGGCGCTAGCGCTCAGTCGTTCATCGAGAGGATGACGAATTTCGACAAGAACCTGCCAAATGAGAAGGTGCTCCCTAAGCACTCGCTCCTGTACGAGTACTTCACAGTCTACAACGAGCTGACTAAGGTGAAGTATGTGACCGAGGGCATGAGGAAGCCGGCTTTCCTGTCTGGGGAGCAGAAGAAGGCCATCGTGGACCTCCTGTTCAAGACCAACCGGAAGGTCACGGTTAAGCAGCTCAAGGAGGACTACTTCAAGAAGATTGAGTGCTTCGATTCGGTCGAGATCTCTGGCGTTGAGGACCGCTTCAACGCCTCCCTGGGGACCTACCACGATCTCCTGAAGATCATTAAGGATAAGGACTTCCTGGACAACGAGGAGAATGAGGATATCCTCGAGGACATTGTGCTGACACTCACTCTGTTCGAGGACCGGGAGATGATCGAGGAGCGCCTGAAGACTTACGCCCATCTCTTCGATGACAAGGTCATGAAGCAGCTCAAGAGGAGGAGGTACACCGGCTGGGGGAGGCTGAGCAGGAAGCTCATCAACGGCATTCGGGACAAGCAGTCCGGGAAGACGATCCTCGACTTCCTGAAGAGCGATGGCTTCGCGAACCGCAATTTCATGCAGCTGATTCACGATGACAGCCTCACATTCAAGGAGGATATCCAGAAGGCTCAGGTGAGCGGCCAGGGGGACTCGCTGCACGAGCATATCGCGAACCTCGCTGGCTCGCCAGCTATCAAGAAGGGGATTCTGCAGACCGTGAAGGTTGTGGACGAGCTGGTGAAGGTCATGGGCAGGCACAAGCCTGAGAACATCGTCATTGAGATGGCCCGGGAGAATCAGACCACGCAGAAGGGCCAGAAGAACTCACGCGAGAGGATGAAGAGGATCGAGGAGGGCATTAAGGAGCTGGGGTCCCAGATCCTCAAGGAGCACCCGGTGGAGAACACGCAGCTGCAGAATGAGAAGCTCTACCTGTACTACCTCCAGAATGGCCGCGATATGTATGTGGACCAGGAGCTGGATATTAACAGGCTCAGCGATTACGACGTCGATGCCATCGTTCCACAGTCATTCCTGAAGGATGACTCCATTGACAACAAGGTCCTCACCAGGTCGGACAAGAACCGGGGCAAGTCTGATAATGTTCCTTCAGAGGAGGTCGTTAAGAAGATGAAGAACTACTGGCGCCAGCTCCTGAATGCCAAGCTGATCACGCAGCGGAAGTTCGATAACCTCACAAAGGCTGAGAGGGGCGGGCTCTCTGAGCTGGACAAGGCGGGCTTCATCAAGAGGCAGCTGGTCGAGACACGGCAGATCACTAAGCACGTTGCGCAGATTCTCGACTCACGGATGAACACTAAGTACGATGAGAATGACAAGCTGATCCGCGAGGTGAAGGTCATCACCCTGAAGTCAAAGCTCGTCTCCGACTTCAGGAAGGATTTCCAGTTCTACAAGGTTCGGGAGATCAACAATTACCACCATGCCCATGACGCGTACCTGAACGCGGTGGTCGGCACAGCTCTGATCAAGAAGTACCCAAAGCTCGAGAGCGAGTTCGTGTACGGGGACTACAAGGTTTACGATGTGAGGAAGATGATCGCCAAGTCGGAGCAGGAGATTGGCAAGGCTACCGCCAAGTACTTCTTCTACTCTAACATTATGAATTTCTTCAAGACAGAGATCACTCTGGCCAATGGCGAGATCCGGAAGCGCCCCCTCATCGAGACGAACGGCGAGACGGGGGAGATCGTGTGGGACAAGGGCAGGGATTTCGCGACCGTCAGGAAGGTTCTCTCCATGCCACAAGTGAATATCGTCAAGAAGACAGAGGTCCAGACTGGCGGGTTCTCTAAGGAGTCAATTCTGCCTAAGCGGAACAGCGACAAGCTCATCGCCCGCAAGAAGGACTGGGATCCGAAGAAGTACGGCGGGTTCGACAGCCCCACTGTGGCCTACTCGGTCCTGGTTGTGGCGAAGGTTGAGAAGGGCAAGTCCAAGAAGCTCAAGAGCGTGAAGGAGCTGCTGGGGATCACGATTATGGAGCGCTCCAGCTTCGAGAAGAACCCGATCGATTTCCTGGAGGCGAAGGGCTACAAGGAGGTGAAGAAGGACCTGATCATTAAGCTCCCCAAGTACTCACTCTTCGAGCTGGAGAACGGCAGGAAGCGGATGCTGGCTTCCGCTGGCGAGCTGCAGAAGGGGAACGAGCTGGCTCTGCCGTCCAAGTATGTGAACTTCCTCTACCTGGCCTCCCACTACGAGAAGCTCAAGGGCAGCCCCGAGGACAACGAGCAGAAGCAGCTGTTCGTCGAGCAGCACAAGCATTACCTCGACGAGATCATTGAGCAGATTTCCGAGTTCTCCAAGCGCGTGATCCTGGCCGACGCGAATCTGGATAAGGTCCTCTCCGCGTACAACAAGCACCGCGACAAGCCAATCAGGGAGCAGGCTGAGAATATCATTCATCTCTTCACCCTGACGAACCTCGGCGCCCCTGCTGCTTTCAAGTACTTCGACACAACTATCGATCGCAAGAGGTACACAAGCACTAAGGAGGTCCTGGACGCGACCCTCATCCACCAGTCGATTACCGGCCTCTACGAGACGCGCATCGACCTGTCTCAGCTCGGGGGCGACGAATTCTCCGGGAGCGAGACGCCAGGCACCTCCGAGTCGGCCACCCCAGAATCTGCCACAGTGGTGTCCGGCCAAAAGCAGGACCGCCAGGGCGGAGAACGCAGAAGGTCCCAGCTCGATAGGGATCAGTGTGCCTACTGCAAGGAGAAGGGCCACTGGGCCAAAGACTGCCCGAAAAAGCCGCGCGGCCCACGCGGCCCAAGGCCACAAACATCCCTCCTTCCAAAGAAGAAGCGGAAGGTGGAGCTCAGCGGAGGATCTTCCGGAGGATCTAGCGGCTCCGAGACACCAGGAACATCCGAAAGCGCTACACCAGAATCTAGCGGAGGCTCTTCCGGAGGATCTAGGCCTGTCCTGAAGAAACCTCTGGGCCCGCTCTCCAGCAAGCAGAGCCTAGTTCAAGGTGCAAATGGTTCTAGCTATAGGTCATGGACGACGAGCAGGACTATGGATTTAGGGAAGGGGGAGATCAAGCATTCATTTCTTGTTATCCCAAATTGTCCTTCCCCTTTGATGGGACAAGATCTGCTGACCAAGTTGCGAGCACAGATCACCTTCGAGGAAACTGGACCTGAAATTGCCTTCCTCAACCCTTCTGTCCAGCCGCTCACCACTTCAGTCTTGACCATCAGGGCTGAGGATGAGTATCGTCTATTCACCAATGATCCACTGCCGGTTGAATCGCAAGACACCGACTTCTGGCTGCGGCTGGTCCCGGAGGCATGGGCAGAGACGGCCGGGCTTGGCCTATCCACACTTCAGGCCCCTGTTGTTGTCGAGCTCAAGACGACTGCTGTTCCTGTAAGAGTGAGGCAGTACCCGATGTCACAAGAAGCAAAAAAAGGCATTACACCGCACATCAGAAAATTGCTGCAGCAAGGCGTCCTCGTGAAATGCCAGTCTGCGTGGAACACACCGTTGTTGCCCGTCAAGAAGCCCGGAACTGGAGACTACCGGCCGGTGCAGGATCTCCGCGCCGTTAACTCACAAGTTGAGGTGATACACCCGACCGTGCCCAACCCATACAACCTCCTTTCCACGCTCTCTCCTGAGAGGGTGTGGTACAGCGTACTCGACCTCAAAGATGCTTTCTTCTGTTTGAGCCTGCATCCAGCAAGTCAACCACTCTTCGCCTTTGAGTGGTCCGACCCCGAAGCTGGCATAAGTGGGCAGCTGACATGGACACGACTACCGCAGGGCTTCAAGAATTCTCCAACAATTTTTGACGAGGCGCTGCACCAGGACCTGAGTTTGTTTCGTTCCCAGCACCCACAGGTGACGCTATTACAATATGTGGATGATATCCTTCTCGCCGGCAAGACTGAGGAGGACTGGGGTAAGCGCTGGCTGACGGAGGCGAGGAAGAAGGTGGTGGCCCAGATACCTGCCCCAACTACAAGAAGACAGCTTCGGGAGTTCTTGGGGACGGCGGGTTTCTGCCGCCTTTGGATCCCAGAATTAGCAATAGTGCACTGCCCAGGACATCAAACTGGTAAAGAAACAGTCGCCTCTGGCAACCGCCGGGCCGACGAAGCGGCGAAGGCCGCCGCATCGTGCATGAAGGAGATACCGGCGCTGATTGTCACCGAGCCCGACAACCTCCCGCCGGTCTCCACCACTGTTGATCCCCTTTCAGAAGAACCAGAAGTTGGCGACGTGAAACTGATGGTGCTCCGCCAGCAATATCAAGAGCTGGCTCAAGCTGATAATTTTGATCAGGAGTACCAGGAGGTGGAGGCTCCTGATCATATTGATTTTAGCGGCGGCAGCAAAAGAACGGCGGACGGCTCTGAGAAGCGCACCGCTGATAGCCAGCATTCAACTCCTCCGAAAACAAAGAGGAAAGTTGAGTTCGAACCGAAGAAGAAAAGGAAGGTGTGA

**Sequence 6 Plasmids sequence of ePPEplus-RT5**

(NLSSV40-nCas9(H840A/R221K/N394K)-XTEN-NC-NLS-32aa Linker-RT9-NLSvbp)

CCTAAGAAAAAGAGAAAAGTGGACAAGAAGTACTCGATCGGCCTCGATATTGGGACTAACTCTGTTGGCTGGGCCGTGATCACCGACGAGTACAAGGTGCCCTCAAAGAAGTTCAAGGTCCTGGGCAACACCGATCGGCATTCCATCAAGAAGAATCTCATTGGCGCTCTCCTGTTCGACAGCGGCGAGACGGCTGAGGCTACGCGGCTCAAGCGCACCGCCCGCAGGCGGTACACGCGCAGGAAGAATCGCATCTGCTACCTGCAGGAGATTTTCTCCAACGAGATGGCGAAGGTTGACGATTCTTTCTTCCACAGGCTGGAGGAGTCATTCCTCGTGGAGGAGGATAAGAAGCACGAGCGGCATCCAATCTTCGGCAACATTGTCGACGAGGTTGCCTACCACGAGAAGTACCCTACGATCTACCATCTGCGGAAGAAGCTCGTGGACTCCACAGATAAGGCGGACCTCCGCCTGATCTACCTCGCTCTGGCCCACATGATTAAGTTCAGGGGCCATTTCCTGATCGAGGGGGATCTCAACCCGGACAATAGCGATGTTGACAAGCTGTTCATCCAGCTCGTGCAGACGTACAACCAGCTCTTCGAGGAGAACCCCATTAATGCGTCAGGCGTCGACGCGAAGGCTATCCTGTCCGCTAGGCTCTCGAAGTCTCGGAAGCTCGAGAACCTGATCGCCCAGCTGCCGGGCGAGAAGAAGAACGGCCTGTTCGGGAATCTCATTGCGCTCAGCCTGGGGCTCACGCCCAACTTCAAGTCGAATTTCGATCTCGCTGAGGACGCCAAGCTGCAGCTCTCCAAGGACACATACGACGATGACCTGGATAACCTCCTGGCCCAGATCGGCGATCAGTACGCGGACCTGTTCCTCGCTGCCAAGAATCTGTCGGACGCCATCCTCCTGTCTGATATTCTCAGGGTGAACACCGAGATTACGAAGGCTCCGCTCTCAGCCTCCATGATCAAGCGCTACGACGAGCACCATCAGGATCTGACCCTCCTGAAGGCGCTGGTCAGGCAGCAGCTCCCCGAGAAGTACAAGGAGATCTTCTTCGATCAGTCGAAGAACGGCTACGCTGGGTACATTGACGGCGGGGCCTCTCAGGAGGAGTTCTACAAGTTCATCAAGCCGATTCTGGAGAAGATGGACGGCACGGAGGAGCTGCTGGTGAAGCTCAAGCGCGAGGACCTCCTGAGGAAGCAGCGGACATTCGATAACGGCAGCATCCCACACCAGATTCATCTCGGGGAGCTGCACGCTATCCTGAGGAGGCAGGAGGACTTCTACCCTTTCCTCAAGGATAACCGCGAGAAGATCGAGAAGATTCTGACTTTCAGGATCCCGTACTACGTCGGCCCACTCGCTAGGGGCAACTCCCGCTTCGCTTGGATGACCCGCAAGTCAGAGGAGACGATCACGCCGTGGAACTTCGAGGAGGTGGTCGACAAGGGCGCTAGCGCTCAGTCGTTCATCGAGAGGATGACGAATTTCGACAAGAACCTGCCAAATGAGAAGGTGCTCCCTAAGCACTCGCTCCTGTACGAGTACTTCACAGTCTACAACGAGCTGACTAAGGTGAAGTATGTGACCGAGGGCATGAGGAAGCCGGCTTTCCTGTCTGGGGAGCAGAAGAAGGCCATCGTGGACCTCCTGTTCAAGACCAACCGGAAGGTCACGGTTAAGCAGCTCAAGGAGGACTACTTCAAGAAGATTGAGTGCTTCGATTCGGTCGAGATCTCTGGCGTTGAGGACCGCTTCAACGCCTCCCTGGGGACCTACCACGATCTCCTGAAGATCATTAAGGATAAGGACTTCCTGGACAACGAGGAGAATGAGGATATCCTCGAGGACATTGTGCTGACACTCACTCTGTTCGAGGACCGGGAGATGATCGAGGAGCGCCTGAAGACTTACGCCCATCTCTTCGATGACAAGGTCATGAAGCAGCTCAAGAGGAGGAGGTACACCGGCTGGGGGAGGCTGAGCAGGAAGCTCATCAACGGCATTCGGGACAAGCAGTCCGGGAAGACGATCCTCGACTTCCTGAAGAGCGATGGCTTCGCGAACCGCAATTTCATGCAGCTGATTCACGATGACAGCCTCACATTCAAGGAGGATATCCAGAAGGCTCAGGTGAGCGGCCAGGGGGACTCGCTGCACGAGCATATCGCGAACCTCGCTGGCTCGCCAGCTATCAAGAAGGGGATTCTGCAGACCGTGAAGGTTGTGGACGAGCTGGTGAAGGTCATGGGCAGGCACAAGCCTGAGAACATCGTCATTGAGATGGCCCGGGAGAATCAGACCACGCAGAAGGGCCAGAAGAACTCACGCGAGAGGATGAAGAGGATCGAGGAGGGCATTAAGGAGCTGGGGTCCCAGATCCTCAAGGAGCACCCGGTGGAGAACACGCAGCTGCAGAATGAGAAGCTCTACCTGTACTACCTCCAGAATGGCCGCGATATGTATGTGGACCAGGAGCTGGATATTAACAGGCTCAGCGATTACGACGTCGATGCCATCGTTCCACAGTCATTCCTGAAGGATGACTCCATTGACAACAAGGTCCTCACCAGGTCGGACAAGAACCGGGGCAAGTCTGATAATGTTCCTTCAGAGGAGGTCGTTAAGAAGATGAAGAACTACTGGCGCCAGCTCCTGAATGCCAAGCTGATCACGCAGCGGAAGTTCGATAACCTCACAAAGGCTGAGAGGGGCGGGCTCTCTGAGCTGGACAAGGCGGGCTTCATCAAGAGGCAGCTGGTCGAGACACGGCAGATCACTAAGCACGTTGCGCAGATTCTCGACTCACGGATGAACACTAAGTACGATGAGAATGACAAGCTGATCCGCGAGGTGAAGGTCATCACCCTGAAGTCAAAGCTCGTCTCCGACTTCAGGAAGGATTTCCAGTTCTACAAGGTTCGGGAGATCAACAATTACCACCATGCCCATGACGCGTACCTGAACGCGGTGGTCGGCACAGCTCTGATCAAGAAGTACCCAAAGCTCGAGAGCGAGTTCGTGTACGGGGACTACAAGGTTTACGATGTGAGGAAGATGATCGCCAAGTCGGAGCAGGAGATTGGCAAGGCTACCGCCAAGTACTTCTTCTACTCTAACATTATGAATTTCTTCAAGACAGAGATCACTCTGGCCAATGGCGAGATCCGGAAGCGCCCCCTCATCGAGACGAACGGCGAGACGGGGGAGATCGTGTGGGACAAGGGCAGGGATTTCGCGACCGTCAGGAAGGTTCTCTCCATGCCACAAGTGAATATCGTCAAGAAGACAGAGGTCCAGACTGGCGGGTTCTCTAAGGAGTCAATTCTGCCTAAGCGGAACAGCGACAAGCTCATCGCCCGCAAGAAGGACTGGGATCCGAAGAAGTACGGCGGGTTCGACAGCCCCACTGTGGCCTACTCGGTCCTGGTTGTGGCGAAGGTTGAGAAGGGCAAGTCCAAGAAGCTCAAGAGCGTGAAGGAGCTGCTGGGGATCACGATTATGGAGCGCTCCAGCTTCGAGAAGAACCCGATCGATTTCCTGGAGGCGAAGGGCTACAAGGAGGTGAAGAAGGACCTGATCATTAAGCTCCCCAAGTACTCACTCTTCGAGCTGGAGAACGGCAGGAAGCGGATGCTGGCTTCCGCTGGCGAGCTGCAGAAGGGGAACGAGCTGGCTCTGCCGTCCAAGTATGTGAACTTCCTCTACCTGGCCTCCCACTACGAGAAGCTCAAGGGCAGCCCCGAGGACAACGAGCAGAAGCAGCTGTTCGTCGAGCAGCACAAGCATTACCTCGACGAGATCATTGAGCAGATTTCCGAGTTCTCCAAGCGCGTGATCCTGGCCGACGCGAATCTGGATAAGGTCCTCTCCGCGTACAACAAGCACCGCGACAAGCCAATCAGGGAGCAGGCTGAGAATATCATTCATCTCTTCACCCTGACGAACCTCGGCGCCCCTGCTGCTTTCAAGTACTTCGACACAACTATCGATCGCAAGAGGTACACAAGCACTAAGGAGGTCCTGGACGCGACCCTCATCCACCAGTCGATTACCGGCCTCTACGAGACGCGCATCGACCTGTCTCAGCTCGGGGGCGACGAATTCTCCGGGAGCGAGACGCCAGGCACCTCCGAGTCGGCCACCCCAGAATCTGCCACAGTGGTGTCCGGCCAAAAGCAGGACCGCCAGGGCGGAGAACGCAGAAGGTCCCAGCTCGATAGGGATCAGTGTGCCTACTGCAAGGAGAAGGGCCACTGGGCCAAAGACTGCCCGAAAAAGCCGCGCGGCCCACGCGGCCCAAGGCCACAAACATCCCTCCTTCCAAAGAAGAAGCGGAAGGTGGAGCTCAGCGGAGGATCTTCCGGAGGATCTAGCGGCTCCGAGACACCAGGAACATCCGAAAGCGCTACACCAGAATCTAGCGGAGGCTCTTCCGGAGGATCTAGGCCTCCTGCCCTTCTCGAGACAATTCTCAGCAGGAACAACCTGATCACCGCCCTCAAGAGAGTGGAGGCGAACAAGGGCGCCCCGGGCATTGATGGTGTACCAACAGAGCAGCTCAGAGATGATATCAGAAAGCACTGGAAAAGCATCAAGAGGCAGCTGCTTGAAGGCACATACAAGCCTGCACCAGTCCGGAGAGTGGAGATACCCAAACCAAATGGCGGCGTCAGACTGCTGGGCATCCCCACCGTCATGGATCGGTTCATCCAGCAGGCCATTTTGCAGGTGCTCACCCCTATCTTTGATCCGCACTTTTCGCCCTACTCCTACGGCTTCCGCCCAAAACGCCGCGCACACGACGCGGTGCGGCAGGCGCAGAAGTATATTCAGGAGGGCTACCGCTACGTCGTGGACATCGACCTGGAGAAATTCTTCGACAGAGTGAATCATGATATCCTCATGTCTCGTGTGGCGAGGAAGGTTGAAGACAAGAGAGTTCTGAAGCTGATCCGTGCCTACCTCAAAGCCGGAGTGATGTTAGAGGGTGTGAGAGTAAGGAGTGAAGAAGGAACCCCTCAAGGTGGCCCTTTGAGCCCTCTGCTTGCCAACATTCTGCTGGACGACCTCGACAAGGAACTCGAGAAGAGGGGCCTCAAATTCTGCCGGTATGCTGATGACTGCAACATATATGTGAGGAGCCCACGTGCCGGCCAACGTGTGAAGCAATCTGTTCAGAAGTACCTGGAAAAGAAGCTGAAGCTGAAGGTGAATGAAGAGAAAAGCGCCGTCGACAGACCATGGAAGCGCAAGTTTCTGGGATTCTCCTTCACAAGCCAGAGAGAAGCTAGGATCAGACTGGCACCAAAGAGCGTGCAGCGCTTCAAGAACAAAATCCGCCAATTGACAAATCCAAACTGGAGCCTGCCGATGGAGGAGAGAATCAGGAAGCTGAATCAATACACTATGGGGTGGATGGGGTATTTCGCGCTCATCGAGACTCCCTCGCCCCTGAAACGCCTGGAGGAGTGGATCAGAAGACGCCTGAGATTGTGTCGGTGGCACCAGTGGAAGAGAGTCCGCACGCGGATTCGAGAGCTCCGAGCACTGGGCTTGAAGGAGCATGAAGTTTTTGAAATCGCTAACACGCGAAAGGGCGCATGGAGGACCACCCGGACGCCGCAGCTCCACAAAGCGCTGGGTAAGGCTTACTGGCTCAAACAGGGGAGCGGCGGCAGCAAAAGAACGGCGGACGGCTCTGAGAAGCGCACCGCTGATAGCCAGCATTCAACTCCTCCGAAAACAAAGAGGAAAGTTGAGTTCGAACCGAAGAAGAAAAGGAAGGTGTGA

**Sequence 7 Plasmids sequence of ePPEplus-RT6**

(NLSSV40-nCas9(H840A/R221K/N394K)-XTEN-NC-NLS-32aa Linker-RT6-NLSvbp)

CCTAAGAAAAAGAGAAAAGTGGACAAGAAGTACTCGATCGGCCTCGATATTGGGACTAACTCTGTTGGCTGGGCCGTGATCACCGACGAGTACAAGGTGCCCTCAAAGAAGTTCAAGGTCCTGGGCAACACCGATCGGCATTCCATCAAGAAGAATCTCATTGGCGCTCTCCTGTTCGACAGCGGCGAGACGGCTGAGGCTACGCGGCTCAAGCGCACCGCCCGCAGGCGGTACACGCGCAGGAAGAATCGCATCTGCTACCTGCAGGAGATTTTCTCCAACGAGATGGCGAAGGTTGACGATTCTTTCTTCCACAGGCTGGAGGAGTCATTCCTCGTGGAGGAGGATAAGAAGCACGAGCGGCATCCAATCTTCGGCAACATTGTCGACGAGGTTGCCTACCACGAGAAGTACCCTACGATCTACCATCTGCGGAAGAAGCTCGTGGACTCCACAGATAAGGCGGACCTCCGCCTGATCTACCTCGCTCTGGCCCACATGATTAAGTTCAGGGGCCATTTCCTGATCGAGGGGGATCTCAACCCGGACAATAGCGATGTTGACAAGCTGTTCATCCAGCTCGTGCAGACGTACAACCAGCTCTTCGAGGAGAACCCCATTAATGCGTCAGGCGTCGACGCGAAGGCTATCCTGTCCGCTAGGCTCTCGAAGTCTCGGAAGCTCGAGAACCTGATCGCCCAGCTGCCGGGCGAGAAGAAGAACGGCCTGTTCGGGAATCTCATTGCGCTCAGCCTGGGGCTCACGCCCAACTTCAAGTCGAATTTCGATCTCGCTGAGGACGCCAAGCTGCAGCTCTCCAAGGACACATACGACGATGACCTGGATAACCTCCTGGCCCAGATCGGCGATCAGTACGCGGACCTGTTCCTCGCTGCCAAGAATCTGTCGGACGCCATCCTCCTGTCTGATATTCTCAGGGTGAACACCGAGATTACGAAGGCTCCGCTCTCAGCCTCCATGATCAAGCGCTACGACGAGCACCATCAGGATCTGACCCTCCTGAAGGCGCTGGTCAGGCAGCAGCTCCCCGAGAAGTACAAGGAGATCTTCTTCGATCAGTCGAAGAACGGCTACGCTGGGTACATTGACGGCGGGGCCTCTCAGGAGGAGTTCTACAAGTTCATCAAGCCGATTCTGGAGAAGATGGACGGCACGGAGGAGCTGCTGGTGAAGCTCAAGCGCGAGGACCTCCTGAGGAAGCAGCGGACATTCGATAACGGCAGCATCCCACACCAGATTCATCTCGGGGAGCTGCACGCTATCCTGAGGAGGCAGGAGGACTTCTACCCTTTCCTCAAGGATAACCGCGAGAAGATCGAGAAGATTCTGACTTTCAGGATCCCGTACTACGTCGGCCCACTCGCTAGGGGCAACTCCCGCTTCGCTTGGATGACCCGCAAGTCAGAGGAGACGATCACGCCGTGGAACTTCGAGGAGGTGGTCGACAAGGGCGCTAGCGCTCAGTCGTTCATCGAGAGGATGACGAATTTCGACAAGAACCTGCCAAATGAGAAGGTGCTCCCTAAGCACTCGCTCCTGTACGAGTACTTCACAGTCTACAACGAGCTGACTAAGGTGAAGTATGTGACCGAGGGCATGAGGAAGCCGGCTTTCCTGTCTGGGGAGCAGAAGAAGGCCATCGTGGACCTCCTGTTCAAGACCAACCGGAAGGTCACGGTTAAGCAGCTCAAGGAGGACTACTTCAAGAAGATTGAGTGCTTCGATTCGGTCGAGATCTCTGGCGTTGAGGACCGCTTCAACGCCTCCCTGGGGACCTACCACGATCTCCTGAAGATCATTAAGGATAAGGACTTCCTGGACAACGAGGAGAATGAGGATATCCTCGAGGACATTGTGCTGACACTCACTCTGTTCGAGGACCGGGAGATGATCGAGGAGCGCCTGAAGACTTACGCCCATCTCTTCGATGACAAGGTCATGAAGCAGCTCAAGAGGAGGAGGTACACCGGCTGGGGGAGGCTGAGCAGGAAGCTCATCAACGGCATTCGGGACAAGCAGTCCGGGAAGACGATCCTCGACTTCCTGAAGAGCGATGGCTTCGCGAACCGCAATTTCATGCAGCTGATTCACGATGACAGCCTCACATTCAAGGAGGATATCCAGAAGGCTCAGGTGAGCGGCCAGGGGGACTCGCTGCACGAGCATATCGCGAACCTCGCTGGCTCGCCAGCTATCAAGAAGGGGATTCTGCAGACCGTGAAGGTTGTGGACGAGCTGGTGAAGGTCATGGGCAGGCACAAGCCTGAGAACATCGTCATTGAGATGGCCCGGGAGAATCAGACCACGCAGAAGGGCCAGAAGAACTCACGCGAGAGGATGAAGAGGATCGAGGAGGGCATTAAGGAGCTGGGGTCCCAGATCCTCAAGGAGCACCCGGTGGAGAACACGCAGCTGCAGAATGAGAAGCTCTACCTGTACTACCTCCAGAATGGCCGCGATATGTATGTGGACCAGGAGCTGGATATTAACAGGCTCAGCGATTACGACGTCGATGCCATCGTTCCACAGTCATTCCTGAAGGATGACTCCATTGACAACAAGGTCCTCACCAGGTCGGACAAGAACCGGGGCAAGTCTGATAATGTTCCTTCAGAGGAGGTCGTTAAGAAGATGAAGAACTACTGGCGCCAGCTCCTGAATGCCAAGCTGATCACGCAGCGGAAGTTCGATAACCTCACAAAGGCTGAGAGGGGCGGGCTCTCTGAGCTGGACAAGGCGGGCTTCATCAAGAGGCAGCTGGTCGAGACACGGCAGATCACTAAGCACGTTGCGCAGATTCTCGACTCACGGATGAACACTAAGTACGATGAGAATGACAAGCTGATCCGCGAGGTGAAGGTCATCACCCTGAAGTCAAAGCTCGTCTCCGACTTCAGGAAGGATTTCCAGTTCTACAAGGTTCGGGAGATCAACAATTACCACCATGCCCATGACGCGTACCTGAACGCGGTGGTCGGCACAGCTCTGATCAAGAAGTACCCAAAGCTCGAGAGCGAGTTCGTGTACGGGGACTACAAGGTTTACGATGTGAGGAAGATGATCGCCAAGTCGGAGCAGGAGATTGGCAAGGCTACCGCCAAGTACTTCTTCTACTCTAACATTATGAATTTCTTCAAGACAGAGATCACTCTGGCCAATGGCGAGATCCGGAAGCGCCCCCTCATCGAGACGAACGGCGAGACGGGGGAGATCGTGTGGGACAAGGGCAGGGATTTCGCGACCGTCAGGAAGGTTCTCTCCATGCCACAAGTGAATATCGTCAAGAAGACAGAGGTCCAGACTGGCGGGTTCTCTAAGGAGTCAATTCTGCCTAAGCGGAACAGCGACAAGCTCATCGCCCGCAAGAAGGACTGGGATCCGAAGAAGTACGGCGGGTTCGACAGCCCCACTGTGGCCTACTCGGTCCTGGTTGTGGCGAAGGTTGAGAAGGGCAAGTCCAAGAAGCTCAAGAGCGTGAAGGAGCTGCTGGGGATCACGATTATGGAGCGCTCCAGCTTCGAGAAGAACCCGATCGATTTCCTGGAGGCGAAGGGCTACAAGGAGGTGAAGAAGGACCTGATCATTAAGCTCCCCAAGTACTCACTCTTCGAGCTGGAGAACGGCAGGAAGCGGATGCTGGCTTCCGCTGGCGAGCTGCAGAAGGGGAACGAGCTGGCTCTGCCGTCCAAGTATGTGAACTTCCTCTACCTGGCCTCCCACTACGAGAAGCTCAAGGGCAGCCCCGAGGACAACGAGCAGAAGCAGCTGTTCGTCGAGCAGCACAAGCATTACCTCGACGAGATCATTGAGCAGATTTCCGAGTTCTCCAAGCGCGTGATCCTGGCCGACGCGAATCTGGATAAGGTCCTCTCCGCGTACAACAAGCACCGCGACAAGCCAATCAGGGAGCAGGCTGAGAATATCATTCATCTCTTCACCCTGACGAACCTCGGCGCCCCTGCTGCTTTCAAGTACTTCGACACAACTATCGATCGCAAGAGGTACACAAGCACTAAGGAGGTCCTGGACGCGACCCTCATCCACCAGTCGATTACCGGCCTCTACGAGACGCGCATCGACCTGTCTCAGCTCGGGGGCGACGAATTCTCCGGGAGCGAGACGCCAGGCACCTCCGAGTCGGCCACCCCAGAATCTGCCACAGTGGTGTCCGGCCAAAAGCAGGACCGCCAGGGCGGAGAACGCAGAAGGTCCCAGCTCGATAGGGATCAGTGTGCCTACTGCAAGGAGAAGGGCCACTGGGCCAAAGACTGCCCGAAAAAGCCGCGCGGCCCACGCGGCCCAAGGCCACAAACATCCCTCCTTCCAAAGAAGAAGCGGAAGGTGGAGCTCAGCGGAGGATCTTCCGGAGGATCTAGCGGCTCCGAGACACCAGGAACATCCGAAAGCGCTACACCAGAATCTAGCGGAGGCTCTTCCGGAGGATCTAGGCCTATGAACCTCGCGTTAAAGCGGGTAATTTCGAACAAGGGTTCTCATGGTGTTGATGGCATGACCGTCTACAAGCTCAAGCAATTTCTAAAAACAAATTGGATCAGCATCCGGGAATCAATCTTGAATGGCGAGTACCGCCCCATGCCGGTGAGACGGGTGGAAATTCCAAAGCCAAATGGTGGGACGCGCCTGTTGGGAATACCTACTGTTTTGGACCGGCTGATCCAGCAGGCGATTGCGCAGGAACTGAACAGTATATATGATCGTCGCTTCTCCGAAAGTTCCTTCGGCTTCAGGCCACAGCGTGGCGCCAAGGACGCCATAAAGAAGGCGGAGCAGTATATAAATGATGGATATCGATGGGTGGTCGACATGGATTTGGAGAAGTTCTTCGACAAAGTGAACCATGATATTCTGATGCATAAACTCTCTCGGAGCATAAAGGATAAACGAGTCCTATCCCTGATCAGGAAGTACCTCCAATCTGGTATAATGATCAACGGCGTGGTGGTTCGGAATGAGGAGGGAACTCCTCAAGGCGGCCCTCTTTCGCCGCTCCTCAGCAACATCATGCTGGATGAACTTGACAAGGAGCTTGAGATGAGGGGCCACAAGTTCTGCCGGTATGCTGATGATTGTAATATTTATGTGAAGAGTGAACGTGCCGGCAAGAGGGTGATGGAGAACATTACTAATTTCATCGAGGGGAAGCTGAAGCTTAAAGTCAACCGCGACAAGTCAGCTGTTGACCGGCCGTGGAAACGCAAATTCCTCGGATTCACTCTAAATCTCATGTTTGGAAAGGCATATTCTTCGATTTCTAAACAAAGTCTTAAAAGATACAAAGACAAGATTAGAGAAGTTCTTAGCCGCAGCAAGCCCATCACCCTGGAGCAAAGGATTGAAAAGCTCAACCAGATCAATATAGGGTGGATAAACTACTACGGTATTGCTAAGTGCAAGGGCATCGTCGAGCACCTGGACATATGGATTCGAAAACGTCTCAGGATGTGCATCTGGAAGCAGTGGAAGAAAGTTCGTACGAGGTACAAGAACCTTAAGAAGCTGGGTTTAGAACACTACGAGGCCATCAAGTTTGCAAATACAAGAAAAGGGTACTGGCGCGTCGCCAATTCTGCAATATTGAACACAACGCTCACCAACAAATTTTTTTCAGACCTCGGCCTCAAATCATTGACACATCAGTACATCAAAATTCACTCCACCAGCGGCGGCAGCAAAAGAACGGCGGACGGCTCTGAGAAGCGCACCGCTGATAGCCAGCATTCAACTCCTCCGAAAACAAAGAGGAAAGTTGAGTTCGAACCGAAGAAGAAAAGGAAGGTGTGA

**Sequence 8 Plasmids sequence of ePPEplus-RT7**

(NLSSV40-nCas9(H840A/R221K/N394K)-XTEN-NC-NLS-32aa Linker-RT7-NLSvbp)

CCTAAGAAAAAGAGAAAAGTGGACAAGAAGTACTCGATCGGCCTCGATATTGGGACTAACTCTGTTGGCTGGGCCGTGATCACCGACGAGTACAAGGTGCCCTCAAAGAAGTTCAAGGTCCTGGGCAACACCGATCGGCATTCCATCAAGAAGAATCTCATTGGCGCTCTCCTGTTCGACAGCGGCGAGACGGCTGAGGCTACGCGGCTCAAGCGCACCGCCCGCAGGCGGTACACGCGCAGGAAGAATCGCATCTGCTACCTGCAGGAGATTTTCTCCAACGAGATGGCGAAGGTTGACGATTCTTTCTTCCACAGGCTGGAGGAGTCATTCCTCGTGGAGGAGGATAAGAAGCACGAGCGGCATCCAATCTTCGGCAACATTGTCGACGAGGTTGCCTACCACGAGAAGTACCCTACGATCTACCATCTGCGGAAGAAGCTCGTGGACTCCACAGATAAGGCGGACCTCCGCCTGATCTACCTCGCTCTGGCCCACATGATTAAGTTCAGGGGCCATTTCCTGATCGAGGGGGATCTCAACCCGGACAATAGCGATGTTGACAAGCTGTTCATCCAGCTCGTGCAGACGTACAACCAGCTCTTCGAGGAGAACCCCATTAATGCGTCAGGCGTCGACGCGAAGGCTATCCTGTCCGCTAGGCTCTCGAAGTCTCGGAAGCTCGAGAACCTGATCGCCCAGCTGCCGGGCGAGAAGAAGAACGGCCTGTTCGGGAATCTCATTGCGCTCAGCCTGGGGCTCACGCCCAACTTCAAGTCGAATTTCGATCTCGCTGAGGACGCCAAGCTGCAGCTCTCCAAGGACACATACGACGATGACCTGGATAACCTCCTGGCCCAGATCGGCGATCAGTACGCGGACCTGTTCCTCGCTGCCAAGAATCTGTCGGACGCCATCCTCCTGTCTGATATTCTCAGGGTGAACACCGAGATTACGAAGGCTCCGCTCTCAGCCTCCATGATCAAGCGCTACGACGAGCACCATCAGGATCTGACCCTCCTGAAGGCGCTGGTCAGGCAGCAGCTCCCCGAGAAGTACAAGGAGATCTTCTTCGATCAGTCGAAGAACGGCTACGCTGGGTACATTGACGGCGGGGCCTCTCAGGAGGAGTTCTACAAGTTCATCAAGCCGATTCTGGAGAAGATGGACGGCACGGAGGAGCTGCTGGTGAAGCTCAAGCGCGAGGACCTCCTGAGGAAGCAGCGGACATTCGATAACGGCAGCATCCCACACCAGATTCATCTCGGGGAGCTGCACGCTATCCTGAGGAGGCAGGAGGACTTCTACCCTTTCCTCAAGGATAACCGCGAGAAGATCGAGAAGATTCTGACTTTCAGGATCCCGTACTACGTCGGCCCACTCGCTAGGGGCAACTCCCGCTTCGCTTGGATGACCCGCAAGTCAGAGGAGACGATCACGCCGTGGAACTTCGAGGAGGTGGTCGACAAGGGCGCTAGCGCTCAGTCGTTCATCGAGAGGATGACGAATTTCGACAAGAACCTGCCAAATGAGAAGGTGCTCCCTAAGCACTCGCTCCTGTACGAGTACTTCACAGTCTACAACGAGCTGACTAAGGTGAAGTATGTGACCGAGGGCATGAGGAAGCCGGCTTTCCTGTCTGGGGAGCAGAAGAAGGCCATCGTGGACCTCCTGTTCAAGACCAACCGGAAGGTCACGGTTAAGCAGCTCAAGGAGGACTACTTCAAGAAGATTGAGTGCTTCGATTCGGTCGAGATCTCTGGCGTTGAGGACCGCTTCAACGCCTCCCTGGGGACCTACCACGATCTCCTGAAGATCATTAAGGATAAGGACTTCCTGGACAACGAGGAGAATGAGGATATCCTCGAGGACATTGTGCTGACACTCACTCTGTTCGAGGACCGGGAGATGATCGAGGAGCGCCTGAAGACTTACGCCCATCTCTTCGATGACAAGGTCATGAAGCAGCTCAAGAGGAGGAGGTACACCGGCTGGGGGAGGCTGAGCAGGAAGCTCATCAACGGCATTCGGGACAAGCAGTCCGGGAAGACGATCCTCGACTTCCTGAAGAGCGATGGCTTCGCGAACCGCAATTTCATGCAGCTGATTCACGATGACAGCCTCACATTCAAGGAGGATATCCAGAAGGCTCAGGTGAGCGGCCAGGGGGACTCGCTGCACGAGCATATCGCGAACCTCGCTGGCTCGCCAGCTATCAAGAAGGGGATTCTGCAGACCGTGAAGGTTGTGGACGAGCTGGTGAAGGTCATGGGCAGGCACAAGCCTGAGAACATCGTCATTGAGATGGCCCGGGAGAATCAGACCACGCAGAAGGGCCAGAAGAACTCACGCGAGAGGATGAAGAGGATCGAGGAGGGCATTAAGGAGCTGGGGTCCCAGATCCTCAAGGAGCACCCGGTGGAGAACACGCAGCTGCAGAATGAGAAGCTCTACCTGTACTACCTCCAGAATGGCCGCGATATGTATGTGGACCAGGAGCTGGATATTAACAGGCTCAGCGATTACGACGTCGATGCCATCGTTCCACAGTCATTCCTGAAGGATGACTCCATTGACAACAAGGTCCTCACCAGGTCGGACAAGAACCGGGGCAAGTCTGATAATGTTCCTTCAGAGGAGGTCGTTAAGAAGATGAAGAACTACTGGCGCCAGCTCCTGAATGCCAAGCTGATCACGCAGCGGAAGTTCGATAACCTCACAAAGGCTGAGAGGGGCGGGCTCTCTGAGCTGGACAAGGCGGGCTTCATCAAGAGGCAGCTGGTCGAGACACGGCAGATCACTAAGCACGTTGCGCAGATTCTCGACTCACGGATGAACACTAAGTACGATGAGAATGACAAGCTGATCCGCGAGGTGAAGGTCATCACCCTGAAGTCAAAGCTCGTCTCCGACTTCAGGAAGGATTTCCAGTTCTACAAGGTTCGGGAGATCAACAATTACCACCATGCCCATGACGCGTACCTGAACGCGGTGGTCGGCACAGCTCTGATCAAGAAGTACCCAAAGCTCGAGAGCGAGTTCGTGTACGGGGACTACAAGGTTTACGATGTGAGGAAGATGATCGCCAAGTCGGAGCAGGAGATTGGCAAGGCTACCGCCAAGTACTTCTTCTACTCTAACATTATGAATTTCTTCAAGACAGAGATCACTCTGGCCAATGGCGAGATCCGGAAGCGCCCCCTCATCGAGACGAACGGCGAGACGGGGGAGATCGTGTGGGACAAGGGCAGGGATTTCGCGACCGTCAGGAAGGTTCTCTCCATGCCACAAGTGAATATCGTCAAGAAGACAGAGGTCCAGACTGGCGGGTTCTCTAAGGAGTCAATTCTGCCTAAGCGGAACAGCGACAAGCTCATCGCCCGCAAGAAGGACTGGGATCCGAAGAAGTACGGCGGGTTCGACAGCCCCACTGTGGCCTACTCGGTCCTGGTTGTGGCGAAGGTTGAGAAGGGCAAGTCCAAGAAGCTCAAGAGCGTGAAGGAGCTGCTGGGGATCACGATTATGGAGCGCTCCAGCTTCGAGAAGAACCCGATCGATTTCCTGGAGGCGAAGGGCTACAAGGAGGTGAAGAAGGACCTGATCATTAAGCTCCCCAAGTACTCACTCTTCGAGCTGGAGAACGGCAGGAAGCGGATGCTGGCTTCCGCTGGCGAGCTGCAGAAGGGGAACGAGCTGGCTCTGCCGTCCAAGTATGTGAACTTCCTCTACCTGGCCTCCCACTACGAGAAGCTCAAGGGCAGCCCCGAGGACAACGAGCAGAAGCAGCTGTTCGTCGAGCAGCACAAGCATTACCTCGACGAGATCATTGAGCAGATTTCCGAGTTCTCCAAGCGCGTGATCCTGGCCGACGCGAATCTGGATAAGGTCCTCTCCGCGTACAACAAGCACCGCGACAAGCCAATCAGGGAGCAGGCTGAGAATATCATTCATCTCTTCACCCTGACGAACCTCGGCGCCCCTGCTGCTTTCAAGTACTTCGACACAACTATCGATCGCAAGAGGTACACAAGCACTAAGGAGGTCCTGGACGCGACCCTCATCCACCAGTCGATTACCGGCCTCTACGAGACGCGCATCGACCTGTCTCAGCTCGGGGGCGACGAATTCTCCGGGAGCGAGACGCCAGGCACCTCCGAGTCGGCCACCCCAGAATCTGCCACAGTGGTGTCCGGCCAAAAGCAGGACCGCCAGGGCGGAGAACGCAGAAGGTCCCAGCTCGATAGGGATCAGTGTGCCTACTGCAAGGAGAAGGGCCACTGGGCCAAAGACTGCCCGAAAAAGCCGCGCGGCCCACGCGGCCCAAGGCCACAAACATCCCTCCTTCCAAAGAAGAAGCGGAAGGTGGAGCTCAGCGGAGGATCTTCCGGAGGATCTAGCGGCTCCGAGACACCAGGAACATCCGAAAGCGCTACACCAGAATCTAGCGGAGGCTCTTCCGGAGGATCTAGGCCTTTCATCAAGGAGAAACCAGTGAATGTTCTGGTGGATACAGGTTCACCAACCTCCTTTATCAGGGCTGATATTGCAGATAATCTGAAGCTCACGCGGTCCACGGCGCCGCCGTTCCGGTTCCGTGGCGTCGTCTCCTCTGAAAGCTCGTATACAAATGAATCAACAGAAATTTTGCTGGAGCTTGATGACATCAAGATAAAAACACCCGTCTACATCACCGACGCCATCACTTTTGAGATAATAATTGGCCACCCCATCATCAGTAGCCACCCGCTGCTCCACAAGATACTGAACAACAAGAAGCCGGTGCCTGAATATATTGTTTCTGTGCTCACTGAAAATGATACTGAGGAGTTGGATGCTGAGAACGCCGCCGCAGTCTTCGCCATTAAAATTTCCGAGACGGACACAGATGATAGGTTCGACAAGCTGCCGCGGTGGCTCAAGGAAAAATATTCAGCAACTGTTAGAGACGATCTTCCTCCAAAAAAATTTGCTGAACCTCCCAAGGTGGAGCATGAGATTGAAATAAAGCCTGGCGCGCGCCTCCCCCGCCGGCAGCCCTATCAAACAACTCCAAAAATTGAGCAAGAAATCAACCTGATAGTTGCTGACCTACTTGAGAAGAAGTTCATAGTTCCAAGCAAGTCGCCGTGCTCCAGCCCTGTGGTTCTTGTAAAAAAGAAGGACGGCACCTATAGATTATGTGTCGACTACCGCGCGCTCAACACCGTCACCATCAAAGATCCATTTCCGCTCCCTCGCATCGATAACCTCCTCGCCAAGATTGGAAGTTCAACTATCTTCTCCACGCTGGATCTACATTCCGGGTACCACCAGATCCCAATGAAGATGGACGACCGCTTCAAGACCGCCTTTGTCACTCCAAATGGAAAGTACGAGTACACGGTGATGCCGTTTGGTCTTGTCAATGCACCTAGCACATTTGCGAGATACATGGCCGACCTCTTCCGTGATTTGCCCTACGTGTGCGTGTACCTCGACGACATTCTCATTCATTCTCGATCTGTTGATGAGCACTGGAAGCATATTGATATCGTCCTCCAGAGGCTCACGCAGGAGGGGCTGATCGTGAAGAAAAAGAAATGCAACTTCGCGGCGAAATCAGTTGAGTTCTGGGACACCCTATCGTTGTTAATCAAGTCGAATAGAAGCGGCGGCAGCAAAAGAACGGCGGACGGCTCTGAGAAGCGCACCGCTGATAGCCAGCATTCAACTCCTCCGAAAACAAAGAGGAAAGTTGAGTTCGAACCGAAGAAGAAAAGGAAGGTGTGA

**Sequence 9 Plasmids sequence of ePPEplus-RT8**

(NLSSV40-nCas9(H840A/R221K/N394K)-XTEN-NC-NLS-32aa Linker-RT8-NLSvbp)

CCTAAGAAAAAGAGAAAAGTGGACAAGAAGTACTCGATCGGCCTCGATATTGGGACTAACTCTGTTGGCTGGGCCGTGATCACCGACGAGTACAAGGTGCCCTCAAAGAAGTTCAAGGTCCTGGGCAACACCGATCGGCATTCCATCAAGAAGAATCTCATTGGCGCTCTCCTGTTCGACAGCGGCGAGACGGCTGAGGCTACGCGGCTCAAGCGCACCGCCCGCAGGCGGTACACGCGCAGGAAGAATCGCATCTGCTACCTGCAGGAGATTTTCTCCAACGAGATGGCGAAGGTTGACGATTCTTTCTTCCACAGGCTGGAGGAGTCATTCCTCGTGGAGGAGGATAAGAAGCACGAGCGGCATCCAATCTTCGGCAACATTGTCGACGAGGTTGCCTACCACGAGAAGTACCCTACGATCTACCATCTGCGGAAGAAGCTCGTGGACTCCACAGATAAGGCGGACCTCCGCCTGATCTACCTCGCTCTGGCCCACATGATTAAGTTCAGGGGCCATTTCCTGATCGAGGGGGATCTCAACCCGGACAATAGCGATGTTGACAAGCTGTTCATCCAGCTCGTGCAGACGTACAACCAGCTCTTCGAGGAGAACCCCATTAATGCGTCAGGCGTCGACGCGAAGGCTATCCTGTCCGCTAGGCTCTCGAAGTCTCGGAAGCTCGAGAACCTGATCGCCCAGCTGCCGGGCGAGAAGAAGAACGGCCTGTTCGGGAATCTCATTGCGCTCAGCCTGGGGCTCACGCCCAACTTCAAGTCGAATTTCGATCTCGCTGAGGACGCCAAGCTGCAGCTCTCCAAGGACACATACGACGATGACCTGGATAACCTCCTGGCCCAGATCGGCGATCAGTACGCGGACCTGTTCCTCGCTGCCAAGAATCTGTCGGACGCCATCCTCCTGTCTGATATTCTCAGGGTGAACACCGAGATTACGAAGGCTCCGCTCTCAGCCTCCATGATCAAGCGCTACGACGAGCACCATCAGGATCTGACCCTCCTGAAGGCGCTGGTCAGGCAGCAGCTCCCCGAGAAGTACAAGGAGATCTTCTTCGATCAGTCGAAGAACGGCTACGCTGGGTACATTGACGGCGGGGCCTCTCAGGAGGAGTTCTACAAGTTCATCAAGCCGATTCTGGAGAAGATGGACGGCACGGAGGAGCTGCTGGTGAAGCTCAAGCGCGAGGACCTCCTGAGGAAGCAGCGGACATTCGATAACGGCAGCATCCCACACCAGATTCATCTCGGGGAGCTGCACGCTATCCTGAGGAGGCAGGAGGACTTCTACCCTTTCCTCAAGGATAACCGCGAGAAGATCGAGAAGATTCTGACTTTCAGGATCCCGTACTACGTCGGCCCACTCGCTAGGGGCAACTCCCGCTTCGCTTGGATGACCCGCAAGTCAGAGGAGACGATCACGCCGTGGAACTTCGAGGAGGTGGTCGACAAGGGCGCTAGCGCTCAGTCGTTCATCGAGAGGATGACGAATTTCGACAAGAACCTGCCAAATGAGAAGGTGCTCCCTAAGCACTCGCTCCTGTACGAGTACTTCACAGTCTACAACGAGCTGACTAAGGTGAAGTATGTGACCGAGGGCATGAGGAAGCCGGCTTTCCTGTCTGGGGAGCAGAAGAAGGCCATCGTGGACCTCCTGTTCAAGACCAACCGGAAGGTCACGGTTAAGCAGCTCAAGGAGGACTACTTCAAGAAGATTGAGTGCTTCGATTCGGTCGAGATCTCTGGCGTTGAGGACCGCTTCAACGCCTCCCTGGGGACCTACCACGATCTCCTGAAGATCATTAAGGATAAGGACTTCCTGGACAACGAGGAGAATGAGGATATCCTCGAGGACATTGTGCTGACACTCACTCTGTTCGAGGACCGGGAGATGATCGAGGAGCGCCTGAAGACTTACGCCCATCTCTTCGATGACAAGGTCATGAAGCAGCTCAAGAGGAGGAGGTACACCGGCTGGGGGAGGCTGAGCAGGAAGCTCATCAACGGCATTCGGGACAAGCAGTCCGGGAAGACGATCCTCGACTTCCTGAAGAGCGATGGCTTCGCGAACCGCAATTTCATGCAGCTGATTCACGATGACAGCCTCACATTCAAGGAGGATATCCAGAAGGCTCAGGTGAGCGGCCAGGGGGACTCGCTGCACGAGCATATCGCGAACCTCGCTGGCTCGCCAGCTATCAAGAAGGGGATTCTGCAGACCGTGAAGGTTGTGGACGAGCTGGTGAAGGTCATGGGCAGGCACAAGCCTGAGAACATCGTCATTGAGATGGCCCGGGAGAATCAGACCACGCAGAAGGGCCAGAAGAACTCACGCGAGAGGATGAAGAGGATCGAGGAGGGCATTAAGGAGCTGGGGTCCCAGATCCTCAAGGAGCACCCGGTGGAGAACACGCAGCTGCAGAATGAGAAGCTCTACCTGTACTACCTCCAGAATGGCCGCGATATGTATGTGGACCAGGAGCTGGATATTAACAGGCTCAGCGATTACGACGTCGATGCCATCGTTCCACAGTCATTCCTGAAGGATGACTCCATTGACAACAAGGTCCTCACCAGGTCGGACAAGAACCGGGGCAAGTCTGATAATGTTCCTTCAGAGGAGGTCGTTAAGAAGATGAAGAACTACTGGCGCCAGCTCCTGAATGCCAAGCTGATCACGCAGCGGAAGTTCGATAACCTCACAAAGGCTGAGAGGGGCGGGCTCTCTGAGCTGGACAAGGCGGGCTTCATCAAGAGGCAGCTGGTCGAGACACGGCAGATCACTAAGCACGTTGCGCAGATTCTCGACTCACGGATGAACACTAAGTACGATGAGAATGACAAGCTGATCCGCGAGGTGAAGGTCATCACCCTGAAGTCAAAGCTCGTCTCCGACTTCAGGAAGGATTTCCAGTTCTACAAGGTTCGGGAGATCAACAATTACCACCATGCCCATGACGCGTACCTGAACGCGGTGGTCGGCACAGCTCTGATCAAGAAGTACCCAAAGCTCGAGAGCGAGTTCGTGTACGGGGACTACAAGGTTTACGATGTGAGGAAGATGATCGCCAAGTCGGAGCAGGAGATTGGCAAGGCTACCGCCAAGTACTTCTTCTACTCTAACATTATGAATTTCTTCAAGACAGAGATCACTCTGGCCAATGGCGAGATCCGGAAGCGCCCCCTCATCGAGACGAACGGCGAGACGGGGGAGATCGTGTGGGACAAGGGCAGGGATTTCGCGACCGTCAGGAAGGTTCTCTCCATGCCACAAGTGAATATCGTCAAGAAGACAGAGGTCCAGACTGGCGGGTTCTCTAAGGAGTCAATTCTGCCTAAGCGGAACAGCGACAAGCTCATCGCCCGCAAGAAGGACTGGGATCCGAAGAAGTACGGCGGGTTCGACAGCCCCACTGTGGCCTACTCGGTCCTGGTTGTGGCGAAGGTTGAGAAGGGCAAGTCCAAGAAGCTCAAGAGCGTGAAGGAGCTGCTGGGGATCACGATTATGGAGCGCTCCAGCTTCGAGAAGAACCCGATCGATTTCCTGGAGGCGAAGGGCTACAAGGAGGTGAAGAAGGACCTGATCATTAAGCTCCCCAAGTACTCACTCTTCGAGCTGGAGAACGGCAGGAAGCGGATGCTGGCTTCCGCTGGCGAGCTGCAGAAGGGGAACGAGCTGGCTCTGCCGTCCAAGTATGTGAACTTCCTCTACCTGGCCTCCCACTACGAGAAGCTCAAGGGCAGCCCCGAGGACAACGAGCAGAAGCAGCTGTTCGTCGAGCAGCACAAGCATTACCTCGACGAGATCATTGAGCAGATTTCCGAGTTCTCCAAGCGCGTGATCCTGGCCGACGCGAATCTGGATAAGGTCCTCTCCGCGTACAACAAGCACCGCGACAAGCCAATCAGGGAGCAGGCTGAGAATATCATTCATCTCTTCACCCTGACGAACCTCGGCGCCCCTGCTGCTTTCAAGTACTTCGACACAACTATCGATCGCAAGAGGTACACAAGCACTAAGGAGGTCCTGGACGCGACCCTCATCCACCAGTCGATTACCGGCCTCTACGAGACGCGCATCGACCTGTCTCAGCTCGGGGGCGACGAATTCTCCGGGAGCGAGACGCCAGGCACCTCCGAGTCGGCCACCCCAGAATCTGCCACAGTGGTGTCCGGCCAAAAGCAGGACCGCCAGGGCGGAGAACGCAGAAGGTCCCAGCTCGATAGGGATCAGTGTGCCTACTGCAAGGAGAAGGGCCACTGGGCCAAAGACTGCCCGAAAAAGCCGCGCGGCCCACGCGGCCCAAGGCCACAAACATCCCTCCTTCCAAAGAAGAAGCGGAAGGTGGAGCTCAGCGGAGGATCTTCCGGAGGATCTAGCGGCTCCGAGACACCAGGAACATCCGAAAGCGCTACACCAGAATCTAGCGGAGGCTCTTCCGGAGGATCTAGGCCTTACGAGCACTTCGCCGTCAGTAGCAAGGAACTCTCGCCGCGCGCGTCGCTGCTCCTCGGGTTCTTTGTTGAGCGGAACATGAACCTTGTGAAGCGATTGGCGCGGCACCTCGGGAAGAGCGAACTTGAGGTGATTCATTTCCTCGCCGACGCCCCAAATAAATATCGTGTGTACAAGATTCCAAAACGGAGCTATGGCCACCGCATCATTGCTCAACCAACAAGAGAGTTGAAGCAGTACCAAAGAGCATTTCTGGAGCTCTACACCTTTCCGGTGCATTCTTCAGCGACGGCCTACTGCAAAGGAAAATCCATCAAGGACAATGCACTCAGCCACGTCAAGAACCACTACCTGCTGAAGACAGATCTAGAAAATTTCTTCAATTCAATCACCCCTTCGATCTTCTGGAAGTCGATTGAAACAAATAGCATAACGACTCCCAAGTTCAGTGCATCTGAAATCGATCTTGTGGAGCGACTGATATTTTGGAGGCCCTCAAAGCTACAGGGCGGCAAGCTAGTGTTGTCAGTTGGTGCGCCGTCGTCGCCTACTATATCAAACTTCTGCCTATACCAGTTTGATGAGTACCTCAGCCTCATCTGCAAGGAGCAGAACATATCTTATACGAGGTATGCTGACGACCTCACCTTCAGCACTTGTGAGAAAGATGTTCTGCACACCGTCATCCCTCTCATTCAGTCCCTTCTTGACTACATCTTTGCATCAGAGTTGAAGCTTAATAGATCTAAAACTGTCTTCTCCTCCAAGGCGCACAACCGCCATGTAACCGGTGTCACGCTGAGCAACAAGGGAACCTTGAGTTTGGGAAGGGAAAGGAAGAGATATATTAAACATCTTGTTCATAGTTTCAAGTATGGTAAACTTGATGATTCTGAGATCCGCCATTTGCAAGGCATGCTGTCTTTTGCCAAGCACATCGAGCCGATTTTCATCGACCGGCTGAAAGAGAAGTACACAAATGAGTTAATAAAGAGGATTTATGAGGCCGGCCATGAAAGCGGCGGCAGCAAAAGAACGGCGGACGGCTCTGAGAAGCGCACCGCTGATAGCCAGCATTCAACTCCTCCGAAAACAAAGAGGAAAGTTGAGTTCGAACCGAAGAAGAAAAGGAAGGTGTGA

**Sequence 10 Plasmids sequence of ePPEplus-RT9**

(NLSSV40-nCas9(H840A/R221K/N394K)-XTEN-NC-NLS-32aa Linker-RT9-NLSvbp)

CCTAAGAAAAAGAGAAAAGTGGACAAGAAGTACTCGATCGGCCTCGATATTGGGACTAACTCTGTTGGCTGGGCCGTGATCACCGACGAGTACAAGGTGCCCTCAAAGAAGTTCAAGGTCCTGGGCAACACCGATCGGCATTCCATCAAGAAGAATCTCATTGGCGCTCTCCTGTTCGACAGCGGCGAGACGGCTGAGGCTACGCGGCTCAAGCGCACCGCCCGCAGGCGGTACACGCGCAGGAAGAATCGCATCTGCTACCTGCAGGAGATTTTCTCCAACGAGATGGCGAAGGTTGACGATTCTTTCTTCCACAGGCTGGAGGAGTCATTCCTCGTGGAGGAGGATAAGAAGCACGAGCGGCATCCAATCTTCGGCAACATTGTCGACGAGGTTGCCTACCACGAGAAGTACCCTACGATCTACCATCTGCGGAAGAAGCTCGTGGACTCCACAGATAAGGCGGACCTCCGCCTGATCTACCTCGCTCTGGCCCACATGATTAAGTTCAGGGGCCATTTCCTGATCGAGGGGGATCTCAACCCGGACAATAGCGATGTTGACAAGCTGTTCATCCAGCTCGTGCAGACGTACAACCAGCTCTTCGAGGAGAACCCCATTAATGCGTCAGGCGTCGACGCGAAGGCTATCCTGTCCGCTAGGCTCTCGAAGTCTCGGAAGCTCGAGAACCTGATCGCCCAGCTGCCGGGCGAGAAGAAGAACGGCCTGTTCGGGAATCTCATTGCGCTCAGCCTGGGGCTCACGCCCAACTTCAAGTCGAATTTCGATCTCGCTGAGGACGCCAAGCTGCAGCTCTCCAAGGACACATACGACGATGACCTGGATAACCTCCTGGCCCAGATCGGCGATCAGTACGCGGACCTGTTCCTCGCTGCCAAGAATCTGTCGGACGCCATCCTCCTGTCTGATATTCTCAGGGTGAACACCGAGATTACGAAGGCTCCGCTCTCAGCCTCCATGATCAAGCGCTACGACGAGCACCATCAGGATCTGACCCTCCTGAAGGCGCTGGTCAGGCAGCAGCTCCCCGAGAAGTACAAGGAGATCTTCTTCGATCAGTCGAAGAACGGCTACGCTGGGTACATTGACGGCGGGGCCTCTCAGGAGGAGTTCTACAAGTTCATCAAGCCGATTCTGGAGAAGATGGACGGCACGGAGGAGCTGCTGGTGAAGCTCAAGCGCGAGGACCTCCTGAGGAAGCAGCGGACATTCGATAACGGCAGCATCCCACACCAGATTCATCTCGGGGAGCTGCACGCTATCCTGAGGAGGCAGGAGGACTTCTACCCTTTCCTCAAGGATAACCGCGAGAAGATCGAGAAGATTCTGACTTTCAGGATCCCGTACTACGTCGGCCCACTCGCTAGGGGCAACTCCCGCTTCGCTTGGATGACCCGCAAGTCAGAGGAGACGATCACGCCGTGGAACTTCGAGGAGGTGGTCGACAAGGGCGCTAGCGCTCAGTCGTTCATCGAGAGGATGACGAATTTCGACAAGAACCTGCCAAATGAGAAGGTGCTCCCTAAGCACTCGCTCCTGTACGAGTACTTCACAGTCTACAACGAGCTGACTAAGGTGAAGTATGTGACCGAGGGCATGAGGAAGCCGGCTTTCCTGTCTGGGGAGCAGAAGAAGGCCATCGTGGACCTCCTGTTCAAGACCAACCGGAAGGTCACGGTTAAGCAGCTCAAGGAGGACTACTTCAAGAAGATTGAGTGCTTCGATTCGGTCGAGATCTCTGGCGTTGAGGACCGCTTCAACGCCTCCCTGGGGACCTACCACGATCTCCTGAAGATCATTAAGGATAAGGACTTCCTGGACAACGAGGAGAATGAGGATATCCTCGAGGACATTGTGCTGACACTCACTCTGTTCGAGGACCGGGAGATGATCGAGGAGCGCCTGAAGACTTACGCCCATCTCTTCGATGACAAGGTCATGAAGCAGCTCAAGAGGAGGAGGTACACCGGCTGGGGGAGGCTGAGCAGGAAGCTCATCAACGGCATTCGGGACAAGCAGTCCGGGAAGACGATCCTCGACTTCCTGAAGAGCGATGGCTTCGCGAACCGCAATTTCATGCAGCTGATTCACGATGACAGCCTCACATTCAAGGAGGATATCCAGAAGGCTCAGGTGAGCGGCCAGGGGGACTCGCTGCACGAGCATATCGCGAACCTCGCTGGCTCGCCAGCTATCAAGAAGGGGATTCTGCAGACCGTGAAGGTTGTGGACGAGCTGGTGAAGGTCATGGGCAGGCACAAGCCTGAGAACATCGTCATTGAGATGGCCCGGGAGAATCAGACCACGCAGAAGGGCCAGAAGAACTCACGCGAGAGGATGAAGAGGATCGAGGAGGGCATTAAGGAGCTGGGGTCCCAGATCCTCAAGGAGCACCCGGTGGAGAACACGCAGCTGCAGAATGAGAAGCTCTACCTGTACTACCTCCAGAATGGCCGCGATATGTATGTGGACCAGGAGCTGGATATTAACAGGCTCAGCGATTACGACGTCGATGCCATCGTTCCACAGTCATTCCTGAAGGATGACTCCATTGACAACAAGGTCCTCACCAGGTCGGACAAGAACCGGGGCAAGTCTGATAATGTTCCTTCAGAGGAGGTCGTTAAGAAGATGAAGAACTACTGGCGCCAGCTCCTGAATGCCAAGCTGATCACGCAGCGGAAGTTCGATAACCTCACAAAGGCTGAGAGGGGCGGGCTCTCTGAGCTGGACAAGGCGGGCTTCATCAAGAGGCAGCTGGTCGAGACACGGCAGATCACTAAGCACGTTGCGCAGATTCTCGACTCACGGATGAACACTAAGTACGATGAGAATGACAAGCTGATCCGCGAGGTGAAGGTCATCACCCTGAAGTCAAAGCTCGTCTCCGACTTCAGGAAGGATTTCCAGTTCTACAAGGTTCGGGAGATCAACAATTACCACCATGCCCATGACGCGTACCTGAACGCGGTGGTCGGCACAGCTCTGATCAAGAAGTACCCAAAGCTCGAGAGCGAGTTCGTGTACGGGGACTACAAGGTTTACGATGTGAGGAAGATGATCGCCAAGTCGGAGCAGGAGATTGGCAAGGCTACCGCCAAGTACTTCTTCTACTCTAACATTATGAATTTCTTCAAGACAGAGATCACTCTGGCCAATGGCGAGATCCGGAAGCGCCCCCTCATCGAGACGAACGGCGAGACGGGGGAGATCGTGTGGGACAAGGGCAGGGATTTCGCGACCGTCAGGAAGGTTCTCTCCATGCCACAAGTGAATATCGTCAAGAAGACAGAGGTCCAGACTGGCGGGTTCTCTAAGGAGTCAATTCTGCCTAAGCGGAACAGCGACAAGCTCATCGCCCGCAAGAAGGACTGGGATCCGAAGAAGTACGGCGGGTTCGACAGCCCCACTGTGGCCTACTCGGTCCTGGTTGTGGCGAAGGTTGAGAAGGGCAAGTCCAAGAAGCTCAAGAGCGTGAAGGAGCTGCTGGGGATCACGATTATGGAGCGCTCCAGCTTCGAGAAGAACCCGATCGATTTCCTGGAGGCGAAGGGCTACAAGGAGGTGAAGAAGGACCTGATCATTAAGCTCCCCAAGTACTCACTCTTCGAGCTGGAGAACGGCAGGAAGCGGATGCTGGCTTCCGCTGGCGAGCTGCAGAAGGGGAACGAGCTGGCTCTGCCGTCCAAGTATGTGAACTTCCTCTACCTGGCCTCCCACTACGAGAAGCTCAAGGGCAGCCCCGAGGACAACGAGCAGAAGCAGCTGTTCGTCGAGCAGCACAAGCATTACCTCGACGAGATCATTGAGCAGATTTCCGAGTTCTCCAAGCGCGTGATCCTGGCCGACGCGAATCTGGATAAGGTCCTCTCCGCGTACAACAAGCACCGCGACAAGCCAATCAGGGAGCAGGCTGAGAATATCATTCATCTCTTCACCCTGACGAACCTCGGCGCCCCTGCTGCTTTCAAGTACTTCGACACAACTATCGATCGCAAGAGGTACACAAGCACTAAGGAGGTCCTGGACGCGACCCTCATCCACCAGTCGATTACCGGCCTCTACGAGACGCGCATCGACCTGTCTCAGCTCGGGGGCGACGAATTCTCCGGGAGCGAGACGCCAGGCACCTCCGAGTCGGCCACCCCAGAATCTGCCACAGTGGTGTCCGGCCAAAAGCAGGACCGCCAGGGCGGAGAACGCAGAAGGTCCCAGCTCGATAGGGATCAGTGTGCCTACTGCAAGGAGAAGGGCCACTGGGCCAAAGACTGCCCGAAAAAGCCGCGCGGCCCACGCGGCCCAAGGCCACAAACATCCCTCCTTCCAAAGAAGAAGCGGAAGGTGGAGCTCAGCGGAGGATCTTCCGGAGGATCTAGCGGCTCCGAGACACCAGGAACATCCGAAAGCGCTACACCAGAATCTAGCGGAGGCTCTTCCGGAGGATCTAGGCCTAGGCCTATGCTGCTGCTTTCCTTGGGTTCCTACAGCCAGCTGGTCGCGCCGCTCTCCAAGATTGAGCCCATTCTTGCCCCGTGCCGCGGGCCTAATGTTAGACTGGGCCCCGGAACACTGGGTTCGCGGCCGGAGGCAAAAGGTCGCTGCTCCACGGCCGAGCCGCCCAGACATCCAATTCTCTCCATTCTGCTAATATTTCAGAAGGCCATCTTCTGTATTGTTCACCAGGTTTTTTTGCTCGTCTTCTTCTTCCTGTTTCTTCTCCTCTTCTGCTGTTGCTGCTTCTACTTCCTCTCAACACAGCACTCAGTGCTGACTGAAGCAAAAGGACCACTCTCTTCAAAAACGTCATGGGTTCAAGGAGCAACTGGCGGAAAGCTCTACAGGTGGACTACTGAAAGGAAGGTGCACCTCTCGACGGGTCAAGTCACACATTCATTTCTCCTTGTGCCGGACTGTCCATATCCTCTTCTTGGAAGGGATCTGCTGAGTAAAGTTGGCGCGCAGATCCCTTTCCAGCAGAAAGGTGCTACCATCACCGGCGCCGGCGGCCAGCCACTGCAAGTCCTGACACTGCGGCTTGAGGATGAACACCGTCTACATGAGGACAGCCCTCCCTCCGTACAACCACTGGACTCTGAATGGCTGACCAATTATCCACAGGCGTGGGTGGAGACGGCGGGCATGGGGCTTGCCGTCAACCAGCCGCCAATAATCATCAACCTGAAACCATCGGCGACGCCGATCAGCATCCGGCAGTATTCCATGAGCAAGGAAGCTAAGGAGGGCATCAGGCCGCACATCCAGAGGTTGCTGCAACTCGGTATTTTAATTCCGTGCCAGTCACCCTGGAACACCCCGTTGCTACCAGTGAAGAAGCCAGGGACCGGGGACTACCGCCCGGTGCAGGATTTAAGGGAGGTGAACTGGAGAACAGAAGATATTCACCCAACAGTACCAAATCCTTACAATTTGTTAAGCACCCTCCCGCCCTCTCATGTTTGGTACACCGTCCTCGATTTGAAGGACGCCTTCTTTTGCCTCCGCCTATCGTCACAGAGCCAACCCATATTTGCATTTGAGTGGAAGGATCCAGAGACAGGCTTCTCTGGGCAGCTCACATGGACAAGACTTCCTCAAGGCTTCAAGAACTCCCCCACCCTGTTCGACGAGGCGCTCCACCGGGATTTGGCCGACTTCCGCGTCGGCCACCCCGACCTCGTTCTCCTTCAGTACGTGGACGACTTATTGCTCGCGGCGCGCACCGAGCAAGATTGTGTGAAAGGGACAGGAGCTCTCCTCGAGAGGCTTGGCGAGCTCGGCTATCGAGCATCCGCCAAGAAAGCTCAAGTGGTGATGCATGACTGCCATCAAATATTAGCTGAAGTTCATGGGACCCGTGGTGATCTTACAGATCAACCTTTGCCTGATGCAGAAGCCACCTGGTATACTGATGGAAGTAGTTTCCTTAGAAATGGGTGGACGGAGGCTTTCCCGACCAAGAGGGAAACTGCTCAGGTGGTCGTCAAGAAGATCCTGGAGGAGATATTTCCTGGTCTTGATTGCCCCCGTGGCGCCAGCGGCGGCAGCAAAAGAACGGCGGACGGCTCTGAGAAGCGCACCGCTGATAGCCAGCATTCAACTCCTCCGAAAACAAAGAGGAAAGTTGAGTTCGAACCGAAGAAGAAAAGGAAGGTGTGA

**Sequence 11 Plasmids sequence of ePPEplus-RT10**

(NLSSV40-nCas9(H840A/R221K/N394K)-XTEN-NC-NLS-32aa Linker-RT10-NLSvbp)

CCTAAGAAAAAGAGAAAAGTGGACAAGAAGTACTCGATCGGCCTCGATATTGGGACTAACTCTGTTGGCTGGGCCGTGATCACCGACGAGTACAAGGTGCCCTCAAAGAAGTTCAAGGTCCTGGGCAACACCGATCGGCATTCCATCAAGAAGAATCTCATTGGCGCTCTCCTGTTCGACAGCGGCGAGACGGCTGAGGCTACGCGGCTCAAGCGCACCGCCCGCAGGCGGTACACGCGCAGGAAGAATCGCATCTGCTACCTGCAGGAGATTTTCTCCAACGAGATGGCGAAGGTTGACGATTCTTTCTTCCACAGGCTGGAGGAGTCATTCCTCGTGGAGGAGGATAAGAAGCACGAGCGGCATCCAATCTTCGGCAACATTGTCGACGAGGTTGCCTACCACGAGAAGTACCCTACGATCTACCATCTGCGGAAGAAGCTCGTGGACTCCACAGATAAGGCGGACCTCCGCCTGATCTACCTCGCTCTGGCCCACATGATTAAGTTCAGGGGCCATTTCCTGATCGAGGGGGATCTCAACCCGGACAATAGCGATGTTGACAAGCTGTTCATCCAGCTCGTGCAGACGTACAACCAGCTCTTCGAGGAGAACCCCATTAATGCGTCAGGCGTCGACGCGAAGGCTATCCTGTCCGCTAGGCTCTCGAAGTCTCGGAAGCTCGAGAACCTGATCGCCCAGCTGCCGGGCGAGAAGAAGAACGGCCTGTTCGGGAATCTCATTGCGCTCAGCCTGGGGCTCACGCCCAACTTCAAGTCGAATTTCGATCTCGCTGAGGACGCCAAGCTGCAGCTCTCCAAGGACACATACGACGATGACCTGGATAACCTCCTGGCCCAGATCGGCGATCAGTACGCGGACCTGTTCCTCGCTGCCAAGAATCTGTCGGACGCCATCCTCCTGTCTGATATTCTCAGGGTGAACACCGAGATTACGAAGGCTCCGCTCTCAGCCTCCATGATCAAGCGCTACGACGAGCACCATCAGGATCTGACCCTCCTGAAGGCGCTGGTCAGGCAGCAGCTCCCCGAGAAGTACAAGGAGATCTTCTTCGATCAGTCGAAGAACGGCTACGCTGGGTACATTGACGGCGGGGCCTCTCAGGAGGAGTTCTACAAGTTCATCAAGCCGATTCTGGAGAAGATGGACGGCACGGAGGAGCTGCTGGTGAAGCTCAAGCGCGAGGACCTCCTGAGGAAGCAGCGGACATTCGATAACGGCAGCATCCCACACCAGATTCATCTCGGGGAGCTGCACGCTATCCTGAGGAGGCAGGAGGACTTCTACCCTTTCCTCAAGGATAACCGCGAGAAGATCGAGAAGATTCTGACTTTCAGGATCCCGTACTACGTCGGCCCACTCGCTAGGGGCAACTCCCGCTTCGCTTGGATGACCCGCAAGTCAGAGGAGACGATCACGCCGTGGAACTTCGAGGAGGTGGTCGACAAGGGCGCTAGCGCTCAGTCGTTCATCGAGAGGATGACGAATTTCGACAAGAACCTGCCAAATGAGAAGGTGCTCCCTAAGCACTCGCTCCTGTACGAGTACTTCACAGTCTACAACGAGCTGACTAAGGTGAAGTATGTGACCGAGGGCATGAGGAAGCCGGCTTTCCTGTCTGGGGAGCAGAAGAAGGCCATCGTGGACCTCCTGTTCAAGACCAACCGGAAGGTCACGGTTAAGCAGCTCAAGGAGGACTACTTCAAGAAGATTGAGTGCTTCGATTCGGTCGAGATCTCTGGCGTTGAGGACCGCTTCAACGCCTCCCTGGGGACCTACCACGATCTCCTGAAGATCATTAAGGATAAGGACTTCCTGGACAACGAGGAGAATGAGGATATCCTCGAGGACATTGTGCTGACACTCACTCTGTTCGAGGACCGGGAGATGATCGAGGAGCGCCTGAAGACTTACGCCCATCTCTTCGATGACAAGGTCATGAAGCAGCTCAAGAGGAGGAGGTACACCGGCTGGGGGAGGCTGAGCAGGAAGCTCATCAACGGCATTCGGGACAAGCAGTCCGGGAAGACGATCCTCGACTTCCTGAAGAGCGATGGCTTCGCGAACCGCAATTTCATGCAGCTGATTCACGATGACAGCCTCACATTCAAGGAGGATATCCAGAAGGCTCAGGTGAGCGGCCAGGGGGACTCGCTGCACGAGCATATCGCGAACCTCGCTGGCTCGCCAGCTATCAAGAAGGGGATTCTGCAGACCGTGAAGGTTGTGGACGAGCTGGTGAAGGTCATGGGCAGGCACAAGCCTGAGAACATCGTCATTGAGATGGCCCGGGAGAATCAGACCACGCAGAAGGGCCAGAAGAACTCACGCGAGAGGATGAAGAGGATCGAGGAGGGCATTAAGGAGCTGGGGTCCCAGATCCTCAAGGAGCACCCGGTGGAGAACACGCAGCTGCAGAATGAGAAGCTCTACCTGTACTACCTCCAGAATGGCCGCGATATGTATGTGGACCAGGAGCTGGATATTAACAGGCTCAGCGATTACGACGTCGATGCCATCGTTCCACAGTCATTCCTGAAGGATGACTCCATTGACAACAAGGTCCTCACCAGGTCGGACAAGAACCGGGGCAAGTCTGATAATGTTCCTTCAGAGGAGGTCGTTAAGAAGATGAAGAACTACTGGCGCCAGCTCCTGAATGCCAAGCTGATCACGCAGCGGAAGTTCGATAACCTCACAAAGGCTGAGAGGGGCGGGCTCTCTGAGCTGGACAAGGCGGGCTTCATCAAGAGGCAGCTGGTCGAGACACGGCAGATCACTAAGCACGTTGCGCAGATTCTCGACTCACGGATGAACACTAAGTACGATGAGAATGACAAGCTGATCCGCGAGGTGAAGGTCATCACCCTGAAGTCAAAGCTCGTCTCCGACTTCAGGAAGGATTTCCAGTTCTACAAGGTTCGGGAGATCAACAATTACCACCATGCCCATGACGCGTACCTGAACGCGGTGGTCGGCACAGCTCTGATCAAGAAGTACCCAAAGCTCGAGAGCGAGTTCGTGTACGGGGACTACAAGGTTTACGATGTGAGGAAGATGATCGCCAAGTCGGAGCAGGAGATTGGCAAGGCTACCGCCAAGTACTTCTTCTACTCTAACATTATGAATTTCTTCAAGACAGAGATCACTCTGGCCAATGGCGAGATCCGGAAGCGCCCCCTCATCGAGACGAACGGCGAGACGGGGGAGATCGTGTGGGACAAGGGCAGGGATTTCGCGACCGTCAGGAAGGTTCTCTCCATGCCACAAGTGAATATCGTCAAGAAGACAGAGGTCCAGACTGGCGGGTTCTCTAAGGAGTCAATTCTGCCTAAGCGGAACAGCGACAAGCTCATCGCCCGCAAGAAGGACTGGGATCCGAAGAAGTACGGCGGGTTCGACAGCCCCACTGTGGCCTACTCGGTCCTGGTTGTGGCGAAGGTTGAGAAGGGCAAGTCCAAGAAGCTCAAGAGCGTGAAGGAGCTGCTGGGGATCACGATTATGGAGCGCTCCAGCTTCGAGAAGAACCCGATCGATTTCCTGGAGGCGAAGGGCTACAAGGAGGTGAAGAAGGACCTGATCATTAAGCTCCCCAAGTACTCACTCTTCGAGCTGGAGAACGGCAGGAAGCGGATGCTGGCTTCCGCTGGCGAGCTGCAGAAGGGGAACGAGCTGGCTCTGCCGTCCAAGTATGTGAACTTCCTCTACCTGGCCTCCCACTACGAGAAGCTCAAGGGCAGCCCCGAGGACAACGAGCAGAAGCAGCTGTTCGTCGAGCAGCACAAGCATTACCTCGACGAGATCATTGAGCAGATTTCCGAGTTCTCCAAGCGCGTGATCCTGGCCGACGCGAATCTGGATAAGGTCCTCTCCGCGTACAACAAGCACCGCGACAAGCCAATCAGGGAGCAGGCTGAGAATATCATTCATCTCTTCACCCTGACGAACCTCGGCGCCCCTGCTGCTTTCAAGTACTTCGACACAACTATCGATCGCAAGAGGTACACAAGCACTAAGGAGGTCCTGGACGCGACCCTCATCCACCAGTCGATTACCGGCCTCTACGAGACGCGCATCGACCTGTCTCAGCTCGGGGGCGACGAATTCTCCGGGAGCGAGACGCCAGGCACCTCCGAGTCGGCCACCCCAGAATCTGCCACAGTGGTGTCCGGCCAAAAGCAGGACCGCCAGGGCGGAGAACGCAGAAGGTCCCAGCTCGATAGGGATCAGTGTGCCTACTGCAAGGAGAAGGGCCACTGGGCCAAAGACTGCCCGAAAAAGCCGCGCGGCCCACGCGGCCCAAGGCCACAAACATCCCTCCTTCCAAAGAAGAAGCGGAAGGTGGAGCTCAGCGGAGGATCTTCCGGAGGATCTAGCGGCTCCGAGACACCAGGAACATCCGAAAGCGCTACACCAGAATCTAGCGGAGGCTCTTCCGGAGGATCTAGGCCTATGGAGACACCCAGCGACAGGTTGCCGCCGCACGGCCTCTCCCGTTTTTCACCCTTCCAGCGCCCCCGCCCACCACCTACTGCACCTCCTCCGCCGCTGGCACCGCCGACGTCGTCGCTGTATCCAATTCTTCCTAGGAAAGATCCACCAAAAACTCCAGTTCTGCCAGCTGATCCGAATTCACCATTGATTGATCTCCTCACATTATTGACTGGCGGCGGCTCGCCGCGGATTTCAAGCACTGGTACAGCCGGCGGTGGAGGGCCAGGCGCCTTAAGCAACTGTAGATCTCCAACCAGGAAGGCCAGGGATGAGCCTGCAAAAGGAGCTACAGGCTCCCGCAGATATAGATGGACCACCGAGCGGAAGGTTCAGCTGGCATCCGGGCAAGTCACTCATTCTTTCCTGCATATTCCTGATTGTAGCCACCCGCTGCTCGGCCGCGACCTCCTCACCAAGCTGAAAGCACAGATCTATTTCGACGACAAGGGGTCGACGGTGATGGGTCCCAAGGGCACGCCGCTCCAAGTTTTGGCGCTAAAACTGGAAGAGGAGTACAGGCTCTTCGAGAGTGAATCATCAAAGGAGCCGTCAGAAGGAATCCAGGGCTGGCTGCGGGAATTCCCCTCCAGCTGGGCTGAAACCGGAGGGCTTGGTCTTGCGCGGGACCAGCCTCCACTGGTGATTCAACTGAAGGCGAGCGCCACCCATGTTTCTATCAAGCAGTATCCCATGAGCCGCGAGGCGCATGAGGGCATCAAACCACACATTAGACGGCTGCTGGATCAAGGAGTGCTCGTACCGTGCCGCTCCCCATGGAACACACCTTTGTTGCCGGTAAAGAAACCTGGTATTGGCGACTACCGACCTGTTCAAGATTTGAGAGAGGTGAACAAGAGGGTGGAGGACATCCACCCAATGGTGCTGAATCCCTACAACTTACTTTGTACTCTTCCCCCAACACATATATGGTACACCGTCCTCGACCTCAAGGACGCCTTTTTCTGCCTCCGCCTCCATCCACAATCTCAACTACTTTTTGCTTTTGAGTGGAGGGACCCGGAGATGGGGCTTTCCGGCCAACTTACATGGACAAGACTTCCTCAAGGGTTCAAGAACTCCCCCACCCTTTTTGATGAAGCTCTGCACAGCGATCTCGCAGAATTTCGCGTCAAGCACCCCGCCCTCATACTCCTGCAGTACGTCGATGATCTATTATTGGCCGCGCGCACGCAGGCCGAGTGCTTGAGGGGAACTAGAGCGCTATTAACGAAATTGGGTCAGAAGAGCTATCGCGCCTCCGCTAAGAAGGCACAAATCTGCCAGAGCAAGGTCATCTACCTCGTGCAGCCGGCGACCGCTGCTTCCGGCAGTCAGGTATTGCTCAGGTGGCTTCCCCTGTACATCCTAAGTCCCTCTCTTGGAAGCTGCAGCACTGGTGAAAGTTCAAATAGTCGTCCGTTCAATGAGTTCCGGGAGCTTTATTCTTCCCACCAGCTTCTGGCATACCAGATAAGCGGCGGCAGCAAAAGAACGGCGGACGGCTCTGAGAAGCGCACCGCTGATAGCCAGCATTCAACTCCTCCGAAAACAAAGAGGAAAGTTGAGTTCGAACCGAAGAAGAAAAGGAAGGTGTGA

**Sequence 12 Plasmids sequence of ePPEplus-RT11**

(NLSSV40-nCas9(H840A/R221K/N394K)-XTEN-NC-NLS-32aa Linker-RT11-NLSvbp)

CCTAAGAAAAAGAGAAAAGTGGACAAGAAGTACTCGATCGGCCTCGATATTGGGACTAACTCTGTTGGCTGGGCCGTGATCACCGACGAGTACAAGGTGCCCTCAAAGAAGTTCAAGGTCCTGGGCAACACCGATCGGCATTCCATCAAGAAGAATCTCATTGGCGCTCTCCTGTTCGACAGCGGCGAGACGGCTGAGGCTACGCGGCTCAAGCGCACCGCCCGCAGGCGGTACACGCGCAGGAAGAATCGCATCTGCTACCTGCAGGAGATTTTCTCCAACGAGATGGCGAAGGTTGACGATTCTTTCTTCCACAGGCTGGAGGAGTCATTCCTCGTGGAGGAGGATAAGAAGCACGAGCGGCATCCAATCTTCGGCAACATTGTCGACGAGGTTGCCTACCACGAGAAGTACCCTACGATCTACCATCTGCGGAAGAAGCTCGTGGACTCCACAGATAAGGCGGACCTCCGCCTGATCTACCTCGCTCTGGCCCACATGATTAAGTTCAGGGGCCATTTCCTGATCGAGGGGGATCTCAACCCGGACAATAGCGATGTTGACAAGCTGTTCATCCAGCTCGTGCAGACGTACAACCAGCTCTTCGAGGAGAACCCCATTAATGCGTCAGGCGTCGACGCGAAGGCTATCCTGTCCGCTAGGCTCTCGAAGTCTCGGAAGCTCGAGAACCTGATCGCCCAGCTGCCGGGCGAGAAGAAGAACGGCCTGTTCGGGAATCTCATTGCGCTCAGCCTGGGGCTCACGCCCAACTTCAAGTCGAATTTCGATCTCGCTGAGGACGCCAAGCTGCAGCTCTCCAAGGACACATACGACGATGACCTGGATAACCTCCTGGCCCAGATCGGCGATCAGTACGCGGACCTGTTCCTCGCTGCCAAGAATCTGTCGGACGCCATCCTCCTGTCTGATATTCTCAGGGTGAACACCGAGATTACGAAGGCTCCGCTCTCAGCCTCCATGATCAAGCGCTACGACGAGCACCATCAGGATCTGACCCTCCTGAAGGCGCTGGTCAGGCAGCAGCTCCCCGAGAAGTACAAGGAGATCTTCTTCGATCAGTCGAAGAACGGCTACGCTGGGTACATTGACGGCGGGGCCTCTCAGGAGGAGTTCTACAAGTTCATCAAGCCGATTCTGGAGAAGATGGACGGCACGGAGGAGCTGCTGGTGAAGCTCAAGCGCGAGGACCTCCTGAGGAAGCAGCGGACATTCGATAACGGCAGCATCCCACACCAGATTCATCTCGGGGAGCTGCACGCTATCCTGAGGAGGCAGGAGGACTTCTACCCTTTCCTCAAGGATAACCGCGAGAAGATCGAGAAGATTCTGACTTTCAGGATCCCGTACTACGTCGGCCCACTCGCTAGGGGCAACTCCCGCTTCGCTTGGATGACCCGCAAGTCAGAGGAGACGATCACGCCGTGGAACTTCGAGGAGGTGGTCGACAAGGGCGCTAGCGCTCAGTCGTTCATCGAGAGGATGACGAATTTCGACAAGAACCTGCCAAATGAGAAGGTGCTCCCTAAGCACTCGCTCCTGTACGAGTACTTCACAGTCTACAACGAGCTGACTAAGGTGAAGTATGTGACCGAGGGCATGAGGAAGCCGGCTTTCCTGTCTGGGGAGCAGAAGAAGGCCATCGTGGACCTCCTGTTCAAGACCAACCGGAAGGTCACGGTTAAGCAGCTCAAGGAGGACTACTTCAAGAAGATTGAGTGCTTCGATTCGGTCGAGATCTCTGGCGTTGAGGACCGCTTCAACGCCTCCCTGGGGACCTACCACGATCTCCTGAAGATCATTAAGGATAAGGACTTCCTGGACAACGAGGAGAATGAGGATATCCTCGAGGACATTGTGCTGACACTCACTCTGTTCGAGGACCGGGAGATGATCGAGGAGCGCCTGAAGACTTACGCCCATCTCTTCGATGACAAGGTCATGAAGCAGCTCAAGAGGAGGAGGTACACCGGCTGGGGGAGGCTGAGCAGGAAGCTCATCAACGGCATTCGGGACAAGCAGTCCGGGAAGACGATCCTCGACTTCCTGAAGAGCGATGGCTTCGCGAACCGCAATTTCATGCAGCTGATTCACGATGACAGCCTCACATTCAAGGAGGATATCCAGAAGGCTCAGGTGAGCGGCCAGGGGGACTCGCTGCACGAGCATATCGCGAACCTCGCTGGCTCGCCAGCTATCAAGAAGGGGATTCTGCAGACCGTGAAGGTTGTGGACGAGCTGGTGAAGGTCATGGGCAGGCACAAGCCTGAGAACATCGTCATTGAGATGGCCCGGGAGAATCAGACCACGCAGAAGGGCCAGAAGAACTCACGCGAGAGGATGAAGAGGATCGAGGAGGGCATTAAGGAGCTGGGGTCCCAGATCCTCAAGGAGCACCCGGTGGAGAACACGCAGCTGCAGAATGAGAAGCTCTACCTGTACTACCTCCAGAATGGCCGCGATATGTATGTGGACCAGGAGCTGGATATTAACAGGCTCAGCGATTACGACGTCGATGCCATCGTTCCACAGTCATTCCTGAAGGATGACTCCATTGACAACAAGGTCCTCACCAGGTCGGACAAGAACCGGGGCAAGTCTGATAATGTTCCTTCAGAGGAGGTCGTTAAGAAGATGAAGAACTACTGGCGCCAGCTCCTGAATGCCAAGCTGATCACGCAGCGGAAGTTCGATAACCTCACAAAGGCTGAGAGGGGCGGGCTCTCTGAGCTGGACAAGGCGGGCTTCATCAAGAGGCAGCTGGTCGAGACACGGCAGATCACTAAGCACGTTGCGCAGATTCTCGACTCACGGATGAACACTAAGTACGATGAGAATGACAAGCTGATCCGCGAGGTGAAGGTCATCACCCTGAAGTCAAAGCTCGTCTCCGACTTCAGGAAGGATTTCCAGTTCTACAAGGTTCGGGAGATCAACAATTACCACCATGCCCATGACGCGTACCTGAACGCGGTGGTCGGCACAGCTCTGATCAAGAAGTACCCAAAGCTCGAGAGCGAGTTCGTGTACGGGGACTACAAGGTTTACGATGTGAGGAAGATGATCGCCAAGTCGGAGCAGGAGATTGGCAAGGCTACCGCCAAGTACTTCTTCTACTCTAACATTATGAATTTCTTCAAGACAGAGATCACTCTGGCCAATGGCGAGATCCGGAAGCGCCCCCTCATCGAGACGAACGGCGAGACGGGGGAGATCGTGTGGGACAAGGGCAGGGATTTCGCGACCGTCAGGAAGGTTCTCTCCATGCCACAAGTGAATATCGTCAAGAAGACAGAGGTCCAGACTGGCGGGTTCTCTAAGGAGTCAATTCTGCCTAAGCGGAACAGCGACAAGCTCATCGCCCGCAAGAAGGACTGGGATCCGAAGAAGTACGGCGGGTTCGACAGCCCCACTGTGGCCTACTCGGTCCTGGTTGTGGCGAAGGTTGAGAAGGGCAAGTCCAAGAAGCTCAAGAGCGTGAAGGAGCTGCTGGGGATCACGATTATGGAGCGCTCCAGCTTCGAGAAGAACCCGATCGATTTCCTGGAGGCGAAGGGCTACAAGGAGGTGAAGAAGGACCTGATCATTAAGCTCCCCAAGTACTCACTCTTCGAGCTGGAGAACGGCAGGAAGCGGATGCTGGCTTCCGCTGGCGAGCTGCAGAAGGGGAACGAGCTGGCTCTGCCGTCCAAGTATGTGAACTTCCTCTACCTGGCCTCCCACTACGAGAAGCTCAAGGGCAGCCCCGAGGACAACGAGCAGAAGCAGCTGTTCGTCGAGCAGCACAAGCATTACCTCGACGAGATCATTGAGCAGATTTCCGAGTTCTCCAAGCGCGTGATCCTGGCCGACGCGAATCTGGATAAGGTCCTCTCCGCGTACAACAAGCACCGCGACAAGCCAATCAGGGAGCAGGCTGAGAATATCATTCATCTCTTCACCCTGACGAACCTCGGCGCCCCTGCTGCTTTCAAGTACTTCGACACAACTATCGATCGCAAGAGGTACACAAGCACTAAGGAGGTCCTGGACGCGACCCTCATCCACCAGTCGATTACCGGCCTCTACGAGACGCGCATCGACCTGTCTCAGCTCGGGGGCGACGAATTCTCCGGGAGCGAGACGCCAGGCACCTCCGAGTCGGCCACCCCAGAATCTGCCACAGTGGTGTCCGGCCAAAAGCAGGACCGCCAGGGCGGAGAACGCAGAAGGTCCCAGCTCGATAGGGATCAGTGTGCCTACTGCAAGGAGAAGGGCCACTGGGCCAAAGACTGCCCGAAAAAGCCGCGCGGCCCACGCGGCCCAAGGCCACAAACATCCCTCCTTCCAAAGAAGAAGCGGAAGGTGGAGCTCAGCGGAGGATCTTCCGGAGGATCTAGCGGCTCCGAGACACCAGGAACATCCGAAAGCGCTACACCAGAATCTAGCGGAGGCTCTTCCGGAGGATCTAGGCCTCCTCTCAGCTCCCCACTGGTGGAGGAATACCGCCTGTTCGTTGAGCAGCCGGCTCAGAACCTTGCCTTACTCGACCTTTGGAGGGAGGACATACCTGAGGTGTGGGCAGAATCCAACCCACCTGGACTGGCCACCACACAGGTTCCTGTGCATGTCCAGCTGACCTCAACCGCCTTACCAATTAGAATACGCCAGTACCCTATATCCCTGGAGGCTAGACGGTCCCTCCGCGGCAGCATCAGGAAATTTAAAGCCGCGGGCATCCTGAAACCTGTTCATAGTCCTTGGAATACGCCTCTGCTGCCAGTGCGCAAGACTGGGACCTCTGAGTATAGGATGGTACAAGATCTGCGAGAAGTGAATAAACGGGTGGAGACCATCCACCCAACTGTGCCTAATCCCTATACGCTGCTGAGCCTGCTCCCTCCTGATAGAACATGGTACTCGGTACTTGATCTCAAGGACGCTTTCTTCTGCATCCCACTCACTTGCCAATCACAGCTGCTTTTCGCATTTGAGTGGATTGATATCGAGGAGGGTGAATCTGGCCAGCTCACGTGGACGAGGCTGCCACAGGGTTTCAAGAATTCTCCGACTCTATTTGATGAGGCTCTGAGTCGCGATCTGCAGGGATATCGTTTCGACCACCCCACTGTGACCCTGCTTCAATATGTGGACGACCTGCTCATAGCTGCGAGGAGCCGGGATGAGTGCCTCCAGGCGACCAGAGACCTCCTCGTCACCCTGGGTTCGATGGGTTACAGGGTGAGCGGCTCTAAGGCGCAACTATGCCAAGAGGAGGTCACTTATCTGGGCTTCCGGATCAAAGATGGCACCCGTACACTGGCTCAGAGCCGGGTTCAGGCGATCTTGCAAATCCCTGCCCCCAAGACTAAGAAGCAGGTCCGGGAGTTCCTGGGAACCGTCGGGTACTGCAGGCTATGGATCCCATCCTTCGCCGAGCTGGCTCAACCTCTCTACGCTGCGACACGGGGAGCCGACGCGCCGCTCCGGTGGACAGGGACAGAGGAAGAAGCCTTCCAGAGACTGAAAACAGCCTTGTTGCAGCCCCCTGCACTAGCTTTGCCCAACCTTGACAAACCTTTCCAACTGTTTGTGGATGAAGCGAAGGGTGTGGCAAAAGGAGTGCTCATGCAGACACTTGGCCCATGGAAGAGACCAGTGGCCTACCTCTCTCGGAAGCTCGACCCGCTGGCCGCCGGCTGGCCTCGATGTTTGAGGGCCATCGCTGCCGCCGCCCTCCTGTCTAAGGAAGCTAGCAAACTTACATTTGAGCAAAGCCTCGAAATTACTTCCAGCCACAATCTTGAGGGCCTACTGCGCACCCCGCCAGACAAATGGCTGACCAACGCACGCGTGACACAGTACCAGGTGCTGCTGCTGGATCCTCCCAGAGTGATCTTCAAGCAAACAGCGGCCCTCAACCCGGCCACCTTGCTGCCTGCGACTGATGATTCGCTGCCGCTCCACCACTGCGCGGATACCTTGGACGCCCTGACCACCACTCGGCCAGATCTAACAGACCAGCCGCTCGCCGATGCCGAAGCTACACTTTTCACAGACGGCTCCTCCTACGTGAAGAAGGCTGAATACGCGGGCGCCGCGGTGGTGACCACAAACAGCATCGTTTGGGCCGAGGCCCTGCCCAGAGGTACTTCCGCGCAGAGAGCTGAGCTGATCGCCCTGACTAAGGCTTTAGAATGGAGCAGAGATAAGACAGTCAACATCTACACGGACTCTAGATATGCTTTTGCTACACTCCACGTCCACGCGATGATTTACAAGGAGCGTGGCCTGCTCACTGCGGGCGGGAAGGCCATCAAGAACGCGAGCGAAATTCTTGCCCTGCTCACTGCCATCTGGCTACCTAAACGGGTGGCCGTCATCCATTGTCGAGGCCACCAGCAGGGCGAGAGCCTCGAGGCTCTGGGAAACCGCCTGGCAGACAAGACTGCTAGAGAAGTAGCCAAGAAGTCTCCCGCCATTCAGGCTTCTCTTTGTGATCCACCTAGAACGCCCGTTGATTGGGTGCCGGCTGACACCCCGCAGTACACTAAACAAGAAGAAGCCCTGGGCCAGAGACTAGGCGGCACCACAGACCCAACAAGCGGCGGCAGCAAAAGAACGGCGGACGGCTCTGAGAAGCGCACCGCTGATAGCCAGCATTCAACTCCTCCGAAAACAAAGAGGAAAGTTGAGTTCGAACCGAAGAAGAAAAGGAAGGTGTGA

**Sequence 13 Plasmids sequence of ePPEplus-RT12**

(NLSSV40-nCas9(H840A/R221K/N394K)-XTEN-NC-NLS-32aa Linker-RT12-NLSvbp)

CCTAAGAAAAAGAGAAAAGTGGACAAGAAGTACTCGATCGGCCTCGATATTGGGACTAACTCTGTTGGCTGGGCCGTGATCACCGACGAGTACAAGGTGCCCTCAAAGAAGTTCAAGGTCCTGGGCAACACCGATCGGCATTCCATCAAGAAGAATCTCATTGGCGCTCTCCTGTTCGACAGCGGCGAGACGGCTGAGGCTACGCGGCTCAAGCGCACCGCCCGCAGGCGGTACACGCGCAGGAAGAATCGCATCTGCTACCTGCAGGAGATTTTCTCCAACGAGATGGCGAAGGTTGACGATTCTTTCTTCCACAGGCTGGAGGAGTCATTCCTCGTGGAGGAGGATAAGAAGCACGAGCGGCATCCAATCTTCGGCAACATTGTCGACGAGGTTGCCTACCACGAGAAGTACCCTACGATCTACCATCTGCGGAAGAAGCTCGTGGACTCCACAGATAAGGCGGACCTCCGCCTGATCTACCTCGCTCTGGCCCACATGATTAAGTTCAGGGGCCATTTCCTGATCGAGGGGGATCTCAACCCGGACAATAGCGATGTTGACAAGCTGTTCATCCAGCTCGTGCAGACGTACAACCAGCTCTTCGAGGAGAACCCCATTAATGCGTCAGGCGTCGACGCGAAGGCTATCCTGTCCGCTAGGCTCTCGAAGTCTCGGAAGCTCGAGAACCTGATCGCCCAGCTGCCGGGCGAGAAGAAGAACGGCCTGTTCGGGAATCTCATTGCGCTCAGCCTGGGGCTCACGCCCAACTTCAAGTCGAATTTCGATCTCGCTGAGGACGCCAAGCTGCAGCTCTCCAAGGACACATACGACGATGACCTGGATAACCTCCTGGCCCAGATCGGCGATCAGTACGCGGACCTGTTCCTCGCTGCCAAGAATCTGTCGGACGCCATCCTCCTGTCTGATATTCTCAGGGTGAACACCGAGATTACGAAGGCTCCGCTCTCAGCCTCCATGATCAAGCGCTACGACGAGCACCATCAGGATCTGACCCTCCTGAAGGCGCTGGTCAGGCAGCAGCTCCCCGAGAAGTACAAGGAGATCTTCTTCGATCAGTCGAAGAACGGCTACGCTGGGTACATTGACGGCGGGGCCTCTCAGGAGGAGTTCTACAAGTTCATCAAGCCGATTCTGGAGAAGATGGACGGCACGGAGGAGCTGCTGGTGAAGCTCAAGCGCGAGGACCTCCTGAGGAAGCAGCGGACATTCGATAACGGCAGCATCCCACACCAGATTCATCTCGGGGAGCTGCACGCTATCCTGAGGAGGCAGGAGGACTTCTACCCTTTCCTCAAGGATAACCGCGAGAAGATCGAGAAGATTCTGACTTTCAGGATCCCGTACTACGTCGGCCCACTCGCTAGGGGCAACTCCCGCTTCGCTTGGATGACCCGCAAGTCAGAGGAGACGATCACGCCGTGGAACTTCGAGGAGGTGGTCGACAAGGGCGCTAGCGCTCAGTCGTTCATCGAGAGGATGACGAATTTCGACAAGAACCTGCCAAATGAGAAGGTGCTCCCTAAGCACTCGCTCCTGTACGAGTACTTCACAGTCTACAACGAGCTGACTAAGGTGAAGTATGTGACCGAGGGCATGAGGAAGCCGGCTTTCCTGTCTGGGGAGCAGAAGAAGGCCATCGTGGACCTCCTGTTCAAGACCAACCGGAAGGTCACGGTTAAGCAGCTCAAGGAGGACTACTTCAAGAAGATTGAGTGCTTCGATTCGGTCGAGATCTCTGGCGTTGAGGACCGCTTCAACGCCTCCCTGGGGACCTACCACGATCTCCTGAAGATCATTAAGGATAAGGACTTCCTGGACAACGAGGAGAATGAGGATATCCTCGAGGACATTGTGCTGACACTCACTCTGTTCGAGGACCGGGAGATGATCGAGGAGCGCCTGAAGACTTACGCCCATCTCTTCGATGACAAGGTCATGAAGCAGCTCAAGAGGAGGAGGTACACCGGCTGGGGGAGGCTGAGCAGGAAGCTCATCAACGGCATTCGGGACAAGCAGTCCGGGAAGACGATCCTCGACTTCCTGAAGAGCGATGGCTTCGCGAACCGCAATTTCATGCAGCTGATTCACGATGACAGCCTCACATTCAAGGAGGATATCCAGAAGGCTCAGGTGAGCGGCCAGGGGGACTCGCTGCACGAGCATATCGCGAACCTCGCTGGCTCGCCAGCTATCAAGAAGGGGATTCTGCAGACCGTGAAGGTTGTGGACGAGCTGGTGAAGGTCATGGGCAGGCACAAGCCTGAGAACATCGTCATTGAGATGGCCCGGGAGAATCAGACCACGCAGAAGGGCCAGAAGAACTCACGCGAGAGGATGAAGAGGATCGAGGAGGGCATTAAGGAGCTGGGGTCCCAGATCCTCAAGGAGCACCCGGTGGAGAACACGCAGCTGCAGAATGAGAAGCTCTACCTGTACTACCTCCAGAATGGCCGCGATATGTATGTGGACCAGGAGCTGGATATTAACAGGCTCAGCGATTACGACGTCGATGCCATCGTTCCACAGTCATTCCTGAAGGATGACTCCATTGACAACAAGGTCCTCACCAGGTCGGACAAGAACCGGGGCAAGTCTGATAATGTTCCTTCAGAGGAGGTCGTTAAGAAGATGAAGAACTACTGGCGCCAGCTCCTGAATGCCAAGCTGATCACGCAGCGGAAGTTCGATAACCTCACAAAGGCTGAGAGGGGCGGGCTCTCTGAGCTGGACAAGGCGGGCTTCATCAAGAGGCAGCTGGTCGAGACACGGCAGATCACTAAGCACGTTGCGCAGATTCTCGACTCACGGATGAACACTAAGTACGATGAGAATGACAAGCTGATCCGCGAGGTGAAGGTCATCACCCTGAAGTCAAAGCTCGTCTCCGACTTCAGGAAGGATTTCCAGTTCTACAAGGTTCGGGAGATCAACAATTACCACCATGCCCATGACGCGTACCTGAACGCGGTGGTCGGCACAGCTCTGATCAAGAAGTACCCAAAGCTCGAGAGCGAGTTCGTGTACGGGGACTACAAGGTTTACGATGTGAGGAAGATGATCGCCAAGTCGGAGCAGGAGATTGGCAAGGCTACCGCCAAGTACTTCTTCTACTCTAACATTATGAATTTCTTCAAGACAGAGATCACTCTGGCCAATGGCGAGATCCGGAAGCGCCCCCTCATCGAGACGAACGGCGAGACGGGGGAGATCGTGTGGGACAAGGGCAGGGATTTCGCGACCGTCAGGAAGGTTCTCTCCATGCCACAAGTGAATATCGTCAAGAAGACAGAGGTCCAGACTGGCGGGTTCTCTAAGGAGTCAATTCTGCCTAAGCGGAACAGCGACAAGCTCATCGCCCGCAAGAAGGACTGGGATCCGAAGAAGTACGGCGGGTTCGACAGCCCCACTGTGGCCTACTCGGTCCTGGTTGTGGCGAAGGTTGAGAAGGGCAAGTCCAAGAAGCTCAAGAGCGTGAAGGAGCTGCTGGGGATCACGATTATGGAGCGCTCCAGCTTCGAGAAGAACCCGATCGATTTCCTGGAGGCGAAGGGCTACAAGGAGGTGAAGAAGGACCTGATCATTAAGCTCCCCAAGTACTCACTCTTCGAGCTGGAGAACGGCAGGAAGCGGATGCTGGCTTCCGCTGGCGAGCTGCAGAAGGGGAACGAGCTGGCTCTGCCGTCCAAGTATGTGAACTTCCTCTACCTGGCCTCCCACTACGAGAAGCTCAAGGGCAGCCCCGAGGACAACGAGCAGAAGCAGCTGTTCGTCGAGCAGCACAAGCATTACCTCGACGAGATCATTGAGCAGATTTCCGAGTTCTCCAAGCGCGTGATCCTGGCCGACGCGAATCTGGATAAGGTCCTCTCCGCGTACAACAAGCACCGCGACAAGCCAATCAGGGAGCAGGCTGAGAATATCATTCATCTCTTCACCCTGACGAACCTCGGCGCCCCTGCTGCTTTCAAGTACTTCGACACAACTATCGATCGCAAGAGGTACACAAGCACTAAGGAGGTCCTGGACGCGACCCTCATCCACCAGTCGATTACCGGCCTCTACGAGACGCGCATCGACCTGTCTCAGCTCGGGGGCGACGAATTCTCCGGGAGCGAGACGCCAGGCACCTCCGAGTCGGCCACCCCAGAATCTGCCACAGTGGTGTCCGGCCAAAAGCAGGACCGCCAGGGCGGAGAACGCAGAAGGTCCCAGCTCGATAGGGATCAGTGTGCCTACTGCAAGGAGAAGGGCCACTGGGCCAAAGACTGCCCGAAAAAGCCGCGCGGCCCACGCGGCCCAAGGCCACAAACATCCCTCCTTCCAAAGAAGAAGCGGAAGGTGGAGCTCAGCGGAGGATCTTCCGGAGGATCTAGCGGCTCCGAGACACCAGGAACATCCGAAAGCGCTACACCAGAATCTAGCGGAGGCTCTTCCGGAGGATCTAGGCCTTATTCGCCGCAGGTGAAGCCGGATCAAGATATTCAAAGCTGGCTCGAGCAGTTCCCTCAGGCGTGGGCGGAGACGGCCGGCATGGGCCTCGCCAAGCAGGTTCCCCCACAGGTCATCCAGCTCAAAGCGTCCGCCACCCCTGTTTCCGTACGGCAGTATCCTTTAAGCCGGGAGGCCAGAGAAGGTATATGGCCACATGTCCAGAGGTTGATACAGCAGGGCATCCTGGTGCCGGTCCAATCTCCATGGAACACACCACTCCTTCCGGTGAGAAAGCCCGGGACAAATGATTATAGGCCAGTGCAGGATCTGCGAGAAGTCAACAAGAGGGTTCAGGACATCCACCCGACTGTTCCAAATCCCTACAACCTCCTCTCGGCGCTGCCGCCGGAGAGGAACTGGTACACCGTCCTTGACCTCAAGGACGCCTTCTTCTGCCTCCGCCTGCATCCTACTTCACAGCCGCTCTTCACCTTTGAGTGGAGGGATCCTGGAACTGGAAGAACAGGTCAATTGACATGGACGCGCTTGCCGCAAGGGTTCAAGAATTCACCCACCATCTTTGATGAGGCGCTCCACCGCGACTTGGCAAATTTTCGTATTCAACACCCACAAGTCACGCTGCTGCAGTACGTGGACGACCTTCTTCTTGCTGGCGCTACAAAACAAGACTGCTTGGAAGGCACGAAAGCACTTTTGCTCGAACTTTCTGATCTTGGGTACCGCGCTAGTGCAAAGAAGGCGCAGATTTGTAGACGCGAGGTGAGCGGCGGCAGCAAAAGAACGGCGGACGGCTCTGAGAAGCGCACCGCTGATAGCCAGCATTCAACTCCTCCGAAAACAAAGAGGAAAGTTGAGTTCGAACCGAAGAAGAAAAGGAAGGTGTGA

**Sequence 14 Plasmids sequence of ePPEplus-RT13**

(NLSSV40-nCas9(H840A/R221K/N394K)-XTEN-NC-NLS-32aa Linker-RT13-NLSvbp)

CCTAAGAAAAAGAGAAAAGTGGACAAGAAGTACTCGATCGGCCTCGATATTGGGACTAACTCTGTTGGCTGGGCCGTGATCACCGACGAGTACAAGGTGCCCTCAAAGAAGTTCAAGGTCCTGGGCAACACCGATCGGCATTCCATCAAGAAGAATCTCATTGGCGCTCTCCTGTTCGACAGCGGCGAGACGGCTGAGGCTACGCGGCTCAAGCGCACCGCCCGCAGGCGGTACACGCGCAGGAAGAATCGCATCTGCTACCTGCAGGAGATTTTCTCCAACGAGATGGCGAAGGTTGACGATTCTTTCTTCCACAGGCTGGAGGAGTCATTCCTCGTGGAGGAGGATAAGAAGCACGAGCGGCATCCAATCTTCGGCAACATTGTCGACGAGGTTGCCTACCACGAGAAGTACCCTACGATCTACCATCTGCGGAAGAAGCTCGTGGACTCCACAGATAAGGCGGACCTCCGCCTGATCTACCTCGCTCTGGCCCACATGATTAAGTTCAGGGGCCATTTCCTGATCGAGGGGGATCTCAACCCGGACAATAGCGATGTTGACAAGCTGTTCATCCAGCTCGTGCAGACGTACAACCAGCTCTTCGAGGAGAACCCCATTAATGCGTCAGGCGTCGACGCGAAGGCTATCCTGTCCGCTAGGCTCTCGAAGTCTCGGAAGCTCGAGAACCTGATCGCCCAGCTGCCGGGCGAGAAGAAGAACGGCCTGTTCGGGAATCTCATTGCGCTCAGCCTGGGGCTCACGCCCAACTTCAAGTCGAATTTCGATCTCGCTGAGGACGCCAAGCTGCAGCTCTCCAAGGACACATACGACGATGACCTGGATAACCTCCTGGCCCAGATCGGCGATCAGTACGCGGACCTGTTCCTCGCTGCCAAGAATCTGTCGGACGCCATCCTCCTGTCTGATATTCTCAGGGTGAACACCGAGATTACGAAGGCTCCGCTCTCAGCCTCCATGATCAAGCGCTACGACGAGCACCATCAGGATCTGACCCTCCTGAAGGCGCTGGTCAGGCAGCAGCTCCCCGAGAAGTACAAGGAGATCTTCTTCGATCAGTCGAAGAACGGCTACGCTGGGTACATTGACGGCGGGGCCTCTCAGGAGGAGTTCTACAAGTTCATCAAGCCGATTCTGGAGAAGATGGACGGCACGGAGGAGCTGCTGGTGAAGCTCAAGCGCGAGGACCTCCTGAGGAAGCAGCGGACATTCGATAACGGCAGCATCCCACACCAGATTCATCTCGGGGAGCTGCACGCTATCCTGAGGAGGCAGGAGGACTTCTACCCTTTCCTCAAGGATAACCGCGAGAAGATCGAGAAGATTCTGACTTTCAGGATCCCGTACTACGTCGGCCCACTCGCTAGGGGCAACTCCCGCTTCGCTTGGATGACCCGCAAGTCAGAGGAGACGATCACGCCGTGGAACTTCGAGGAGGTGGTCGACAAGGGCGCTAGCGCTCAGTCGTTCATCGAGAGGATGACGAATTTCGACAAGAACCTGCCAAATGAGAAGGTGCTCCCTAAGCACTCGCTCCTGTACGAGTACTTCACAGTCTACAACGAGCTGACTAAGGTGAAGTATGTGACCGAGGGCATGAGGAAGCCGGCTTTCCTGTCTGGGGAGCAGAAGAAGGCCATCGTGGACCTCCTGTTCAAGACCAACCGGAAGGTCACGGTTAAGCAGCTCAAGGAGGACTACTTCAAGAAGATTGAGTGCTTCGATTCGGTCGAGATCTCTGGCGTTGAGGACCGCTTCAACGCCTCCCTGGGGACCTACCACGATCTCCTGAAGATCATTAAGGATAAGGACTTCCTGGACAACGAGGAGAATGAGGATATCCTCGAGGACATTGTGCTGACACTCACTCTGTTCGAGGACCGGGAGATGATCGAGGAGCGCCTGAAGACTTACGCCCATCTCTTCGATGACAAGGTCATGAAGCAGCTCAAGAGGAGGAGGTACACCGGCTGGGGGAGGCTGAGCAGGAAGCTCATCAACGGCATTCGGGACAAGCAGTCCGGGAAGACGATCCTCGACTTCCTGAAGAGCGATGGCTTCGCGAACCGCAATTTCATGCAGCTGATTCACGATGACAGCCTCACATTCAAGGAGGATATCCAGAAGGCTCAGGTGAGCGGCCAGGGGGACTCGCTGCACGAGCATATCGCGAACCTCGCTGGCTCGCCAGCTATCAAGAAGGGGATTCTGCAGACCGTGAAGGTTGTGGACGAGCTGGTGAAGGTCATGGGCAGGCACAAGCCTGAGAACATCGTCATTGAGATGGCCCGGGAGAATCAGACCACGCAGAAGGGCCAGAAGAACTCACGCGAGAGGATGAAGAGGATCGAGGAGGGCATTAAGGAGCTGGGGTCCCAGATCCTCAAGGAGCACCCGGTGGAGAACACGCAGCTGCAGAATGAGAAGCTCTACCTGTACTACCTCCAGAATGGCCGCGATATGTATGTGGACCAGGAGCTGGATATTAACAGGCTCAGCGATTACGACGTCGATGCCATCGTTCCACAGTCATTCCTGAAGGATGACTCCATTGACAACAAGGTCCTCACCAGGTCGGACAAGAACCGGGGCAAGTCTGATAATGTTCCTTCAGAGGAGGTCGTTAAGAAGATGAAGAACTACTGGCGCCAGCTCCTGAATGCCAAGCTGATCACGCAGCGGAAGTTCGATAACCTCACAAAGGCTGAGAGGGGCGGGCTCTCTGAGCTGGACAAGGCGGGCTTCATCAAGAGGCAGCTGGTCGAGACACGGCAGATCACTAAGCACGTTGCGCAGATTCTCGACTCACGGATGAACACTAAGTACGATGAGAATGACAAGCTGATCCGCGAGGTGAAGGTCATCACCCTGAAGTCAAAGCTCGTCTCCGACTTCAGGAAGGATTTCCAGTTCTACAAGGTTCGGGAGATCAACAATTACCACCATGCCCATGACGCGTACCTGAACGCGGTGGTCGGCACAGCTCTGATCAAGAAGTACCCAAAGCTCGAGAGCGAGTTCGTGTACGGGGACTACAAGGTTTACGATGTGAGGAAGATGATCGCCAAGTCGGAGCAGGAGATTGGCAAGGCTACCGCCAAGTACTTCTTCTACTCTAACATTATGAATTTCTTCAAGACAGAGATCACTCTGGCCAATGGCGAGATCCGGAAGCGCCCCCTCATCGAGACGAACGGCGAGACGGGGGAGATCGTGTGGGACAAGGGCAGGGATTTCGCGACCGTCAGGAAGGTTCTCTCCATGCCACAAGTGAATATCGTCAAGAAGACAGAGGTCCAGACTGGCGGGTTCTCTAAGGAGTCAATTCTGCCTAAGCGGAACAGCGACAAGCTCATCGCCCGCAAGAAGGACTGGGATCCGAAGAAGTACGGCGGGTTCGACAGCCCCACTGTGGCCTACTCGGTCCTGGTTGTGGCGAAGGTTGAGAAGGGCAAGTCCAAGAAGCTCAAGAGCGTGAAGGAGCTGCTGGGGATCACGATTATGGAGCGCTCCAGCTTCGAGAAGAACCCGATCGATTTCCTGGAGGCGAAGGGCTACAAGGAGGTGAAGAAGGACCTGATCATTAAGCTCCCCAAGTACTCACTCTTCGAGCTGGAGAACGGCAGGAAGCGGATGCTGGCTTCCGCTGGCGAGCTGCAGAAGGGGAACGAGCTGGCTCTGCCGTCCAAGTATGTGAACTTCCTCTACCTGGCCTCCCACTACGAGAAGCTCAAGGGCAGCCCCGAGGACAACGAGCAGAAGCAGCTGTTCGTCGAGCAGCACAAGCATTACCTCGACGAGATCATTGAGCAGATTTCCGAGTTCTCCAAGCGCGTGATCCTGGCCGACGCGAATCTGGATAAGGTCCTCTCCGCGTACAACAAGCACCGCGACAAGCCAATCAGGGAGCAGGCTGAGAATATCATTCATCTCTTCACCCTGACGAACCTCGGCGCCCCTGCTGCTTTCAAGTACTTCGACACAACTATCGATCGCAAGAGGTACACAAGCACTAAGGAGGTCCTGGACGCGACCCTCATCCACCAGTCGATTACCGGCCTCTACGAGACGCGCATCGACCTGTCTCAGCTCGGGGGCGACGAATTCTCCGGGAGCGAGACGCCAGGCACCTCCGAGTCGGCCACCCCAGAATCTGCCACAGTGGTGTCCGGCCAAAAGCAGGACCGCCAGGGCGGAGAACGCAGAAGGTCCCAGCTCGATAGGGATCAGTGTGCCTACTGCAAGGAGAAGGGCCACTGGGCCAAAGACTGCCCGAAAAAGCCGCGCGGCCCACGCGGCCCAAGGCCACAAACATCCCTCCTTCCAAAGAAGAAGCGGAAGGTGGAGCTCAGCGGAGGATCTTCCGGAGGATCTAGCGGCTCCGAGACACCAGGAACATCCGAAAGCGCTACACCAGAATCTAGCGGAGGCTCTTCCGGAGGATCTAGGCCTGGAAGCCACATGACCTGGTTGTCAGATTTCCCGCAGGCGTGGGCAGAAACTGGCGGCATGGGCCTGGCGGTGCGGCAAGCACCGCTCATCATCCCCCTTAAAGCTACTTCTACACCAGTTTCCATCAAGCAGTACCCCATGTCGCAGGAGGCGAGATTAGGTATAAAACCACACATCCAGAGGCTTCTTGATCAAGGAATTCTTGTTCCTTGTCAATCACCATGGAACACCCCTCTTTTACCGGTGAAGAAACCTGGAACAAATGATTATAGACCTGTCCAAGATCTGAGGGAGGTGAACAAGAGGGTGGAGGATATTCATCCAACTGTTCCAAATCCATACAACCTGTTGAGTGGGCTGCCGCCGAGCCATCAGTGGTACACGGTGCTCGACCTGAAAGACGCCTTCTTCTGCCTGCGGCTCCACCCCACGAGTCAACCATTGTTTGCATTTGAGTGGAGGGACCCGGAGATGGGTATTTCTGGTCAGCTGACATGGACAAGGTTGCCACAAGGCTTCAAGAATTCGCCGACGCTCTTTGATGAAGCTTTGCACCGCGACCTCGCCGACTTCCGCATTCAGCATCCTGATTTAATACTCCTCCAGTATGTCGACGACCTTCTCCTGGCCGCCACCTCCGAGCTCGACTGCCAGCAGGGCACCCGGGCGCTCCTGCAAACACTAGGCAACCTCGGGTACCGCGCTTCCGCCAAGAAGGCGCAGATATGTCAGAAGCAAGTCAAGTACCTCGGATATCTATTGAAAGAAGGGCAGCGCCTCACTAGAGGAAGCGGCTGCAGCGGCGGCAGCAAAAGAACGGCGGACGGCTCTGAGAAGCGCACCGCTGATAGCCAGCATTCAACTCCTCCGAAAACAAAGAGGAAAGTTGAGTTCGAACCGAAGAAGAAAAGGAAGGTGTGA

**Sequence 15 Plasmids sequence of ePPEplus-RT14**

(NLSSV40-nCas9(H840A/R221K/N394K)-XTEN-NC-NLS-32aa Linker-RT14-NLSvbp)

CCTAAGAAAAAGAGAAAAGTGGACAAGAAGTACTCGATCGGCCTCGATATTGGGACTAACTCTGTTGGCTGGGCCGTGATCACCGACGAGTACAAGGTGCCCTCAAAGAAGTTCAAGGTCCTGGGCAACACCGATCGGCATTCCATCAAGAAGAATCTCATTGGCGCTCTCCTGTTCGACAGCGGCGAGACGGCTGAGGCTACGCGGCTCAAGCGCACCGCCCGCAGGCGGTACACGCGCAGGAAGAATCGCATCTGCTACCTGCAGGAGATTTTCTCCAACGAGATGGCGAAGGTTGACGATTCTTTCTTCCACAGGCTGGAGGAGTCATTCCTCGTGGAGGAGGATAAGAAGCACGAGCGGCATCCAATCTTCGGCAACATTGTCGACGAGGTTGCCTACCACGAGAAGTACCCTACGATCTACCATCTGCGGAAGAAGCTCGTGGACTCCACAGATAAGGCGGACCTCCGCCTGATCTACCTCGCTCTGGCCCACATGATTAAGTTCAGGGGCCATTTCCTGATCGAGGGGGATCTCAACCCGGACAATAGCGATGTTGACAAGCTGTTCATCCAGCTCGTGCAGACGTACAACCAGCTCTTCGAGGAGAACCCCATTAATGCGTCAGGCGTCGACGCGAAGGCTATCCTGTCCGCTAGGCTCTCGAAGTCTCGGAAGCTCGAGAACCTGATCGCCCAGCTGCCGGGCGAGAAGAAGAACGGCCTGTTCGGGAATCTCATTGCGCTCAGCCTGGGGCTCACGCCCAACTTCAAGTCGAATTTCGATCTCGCTGAGGACGCCAAGCTGCAGCTCTCCAAGGACACATACGACGATGACCTGGATAACCTCCTGGCCCAGATCGGCGATCAGTACGCGGACCTGTTCCTCGCTGCCAAGAATCTGTCGGACGCCATCCTCCTGTCTGATATTCTCAGGGTGAACACCGAGATTACGAAGGCTCCGCTCTCAGCCTCCATGATCAAGCGCTACGACGAGCACCATCAGGATCTGACCCTCCTGAAGGCGCTGGTCAGGCAGCAGCTCCCCGAGAAGTACAAGGAGATCTTCTTCGATCAGTCGAAGAACGGCTACGCTGGGTACATTGACGGCGGGGCCTCTCAGGAGGAGTTCTACAAGTTCATCAAGCCGATTCTGGAGAAGATGGACGGCACGGAGGAGCTGCTGGTGAAGCTCAAGCGCGAGGACCTCCTGAGGAAGCAGCGGACATTCGATAACGGCAGCATCCCACACCAGATTCATCTCGGGGAGCTGCACGCTATCCTGAGGAGGCAGGAGGACTTCTACCCTTTCCTCAAGGATAACCGCGAGAAGATCGAGAAGATTCTGACTTTCAGGATCCCGTACTACGTCGGCCCACTCGCTAGGGGCAACTCCCGCTTCGCTTGGATGACCCGCAAGTCAGAGGAGACGATCACGCCGTGGAACTTCGAGGAGGTGGTCGACAAGGGCGCTAGCGCTCAGTCGTTCATCGAGAGGATGACGAATTTCGACAAGAACCTGCCAAATGAGAAGGTGCTCCCTAAGCACTCGCTCCTGTACGAGTACTTCACAGTCTACAACGAGCTGACTAAGGTGAAGTATGTGACCGAGGGCATGAGGAAGCCGGCTTTCCTGTCTGGGGAGCAGAAGAAGGCCATCGTGGACCTCCTGTTCAAGACCAACCGGAAGGTCACGGTTAAGCAGCTCAAGGAGGACTACTTCAAGAAGATTGAGTGCTTCGATTCGGTCGAGATCTCTGGCGTTGAGGACCGCTTCAACGCCTCCCTGGGGACCTACCACGATCTCCTGAAGATCATTAAGGATAAGGACTTCCTGGACAACGAGGAGAATGAGGATATCCTCGAGGACATTGTGCTGACACTCACTCTGTTCGAGGACCGGGAGATGATCGAGGAGCGCCTGAAGACTTACGCCCATCTCTTCGATGACAAGGTCATGAAGCAGCTCAAGAGGAGGAGGTACACCGGCTGGGGGAGGCTGAGCAGGAAGCTCATCAACGGCATTCGGGACAAGCAGTCCGGGAAGACGATCCTCGACTTCCTGAAGAGCGATGGCTTCGCGAACCGCAATTTCATGCAGCTGATTCACGATGACAGCCTCACATTCAAGGAGGATATCCAGAAGGCTCAGGTGAGCGGCCAGGGGGACTCGCTGCACGAGCATATCGCGAACCTCGCTGGCTCGCCAGCTATCAAGAAGGGGATTCTGCAGACCGTGAAGGTTGTGGACGAGCTGGTGAAGGTCATGGGCAGGCACAAGCCTGAGAACATCGTCATTGAGATGGCCCGGGAGAATCAGACCACGCAGAAGGGCCAGAAGAACTCACGCGAGAGGATGAAGAGGATCGAGGAGGGCATTAAGGAGCTGGGGTCCCAGATCCTCAAGGAGCACCCGGTGGAGAACACGCAGCTGCAGAATGAGAAGCTCTACCTGTACTACCTCCAGAATGGCCGCGATATGTATGTGGACCAGGAGCTGGATATTAACAGGCTCAGCGATTACGACGTCGATGCCATCGTTCCACAGTCATTCCTGAAGGATGACTCCATTGACAACAAGGTCCTCACCAGGTCGGACAAGAACCGGGGCAAGTCTGATAATGTTCCTTCAGAGGAGGTCGTTAAGAAGATGAAGAACTACTGGCGCCAGCTCCTGAATGCCAAGCTGATCACGCAGCGGAAGTTCGATAACCTCACAAAGGCTGAGAGGGGCGGGCTCTCTGAGCTGGACAAGGCGGGCTTCATCAAGAGGCAGCTGGTCGAGACACGGCAGATCACTAAGCACGTTGCGCAGATTCTCGACTCACGGATGAACACTAAGTACGATGAGAATGACAAGCTGATCCGCGAGGTGAAGGTCATCACCCTGAAGTCAAAGCTCGTCTCCGACTTCAGGAAGGATTTCCAGTTCTACAAGGTTCGGGAGATCAACAATTACCACCATGCCCATGACGCGTACCTGAACGCGGTGGTCGGCACAGCTCTGATCAAGAAGTACCCAAAGCTCGAGAGCGAGTTCGTGTACGGGGACTACAAGGTTTACGATGTGAGGAAGATGATCGCCAAGTCGGAGCAGGAGATTGGCAAGGCTACCGCCAAGTACTTCTTCTACTCTAACATTATGAATTTCTTCAAGACAGAGATCACTCTGGCCAATGGCGAGATCCGGAAGCGCCCCCTCATCGAGACGAACGGCGAGACGGGGGAGATCGTGTGGGACAAGGGCAGGGATTTCGCGACCGTCAGGAAGGTTCTCTCCATGCCACAAGTGAATATCGTCAAGAAGACAGAGGTCCAGACTGGCGGGTTCTCTAAGGAGTCAATTCTGCCTAAGCGGAACAGCGACAAGCTCATCGCCCGCAAGAAGGACTGGGATCCGAAGAAGTACGGCGGGTTCGACAGCCCCACTGTGGCCTACTCGGTCCTGGTTGTGGCGAAGGTTGAGAAGGGCAAGTCCAAGAAGCTCAAGAGCGTGAAGGAGCTGCTGGGGATCACGATTATGGAGCGCTCCAGCTTCGAGAAGAACCCGATCGATTTCCTGGAGGCGAAGGGCTACAAGGAGGTGAAGAAGGACCTGATCATTAAGCTCCCCAAGTACTCACTCTTCGAGCTGGAGAACGGCAGGAAGCGGATGCTGGCTTCCGCTGGCGAGCTGCAGAAGGGGAACGAGCTGGCTCTGCCGTCCAAGTATGTGAACTTCCTCTACCTGGCCTCCCACTACGAGAAGCTCAAGGGCAGCCCCGAGGACAACGAGCAGAAGCAGCTGTTCGTCGAGCAGCACAAGCATTACCTCGACGAGATCATTGAGCAGATTTCCGAGTTCTCCAAGCGCGTGATCCTGGCCGACGCGAATCTGGATAAGGTCCTCTCCGCGTACAACAAGCACCGCGACAAGCCAATCAGGGAGCAGGCTGAGAATATCATTCATCTCTTCACCCTGACGAACCTCGGCGCCCCTGCTGCTTTCAAGTACTTCGACACAACTATCGATCGCAAGAGGTACACAAGCACTAAGGAGGTCCTGGACGCGACCCTCATCCACCAGTCGATTACCGGCCTCTACGAGACGCGCATCGACCTGTCTCAGCTCGGGGGCGACGAATTCTCCGGGAGCGAGACGCCAGGCACCTCCGAGTCGGCCACCCCAGAATCTGCCACAGTGGTGTCCGGCCAAAAGCAGGACCGCCAGGGCGGAGAACGCAGAAGGTCCCAGCTCGATAGGGATCAGTGTGCCTACTGCAAGGAGAAGGGCCACTGGGCCAAAGACTGCCCGAAAAAGCCGCGCGGCCCACGCGGCCCAAGGCCACAAACATCCCTCCTTCCAAAGAAGAAGCGGAAGGTGGAGCTCAGCGGAGGATCTTCCGGAGGATCTAGCGGCTCCGAGACACCAGGAACATCCGAAAGCGCTACACCAGAATCTAGCGGAGGCTCTTCCGGAGGATCTAGGCCTGAAGATGAATACAAGCTCTTCGACAAGCCGTCGGTAAGCATGAAGGACATGGATTACTGGCTCTCAAGTTATCCTGAGGCGTGGGCGGAGACTGGTGGAATGGGCATGGCCAAGCAGAGACCGCCGATAGTCATCCATCTTCGCGCGGCGGCAATTCCCATCAACATCAAGCAATACCCCATGAGCAGAGAAGCATATCAAGGAATAAAACCGCACATCAAAAGACTGCTTGACCAGGGGATTCTTACTCCATGTCAATCTCCATGGAACACCCCACTTCTGCCTGTGAAGAAGCCTGGTACAAATGATTATAGGCCGGTGCAGGACCTGCGAGAGGTGAACAAGAGGGTGGAGGACATTCATCCAACTGTTCCAAATCCATACAACCTGCTGTCCACACTTCCGCCCACGCACACCTGGTACACCGTCCTCGACCTCAAAGATGCTTTCTTCTGCCTCCGGTTGTCACCTCAATCACAGGCGCTCTTTGCATTTGAGTGGAAGGATCCGGAAGGCGGCATTAGCGGGCAGCTGACATGGACGCGGCTCCCGCAGGGTTTCAAGAATTCCCCCACCCTGTTTGATGAAGCTCTGCACCAGGATTTGGCTGACTTCCGTGTCAGGCATCCCTCCCTCATCATGCTACAGTATGTTGATGATATTCTCCTCGCCGCCACCAGTGAAGAGGACTGCCAGGCCGGCACGGAGGAGCTGTTGCAAACATTAGGACTTCTCGGCTACCGCGCCTCCGCAAAAAAAGCGCAGATATGCCAAACTAGCGGCGGCAGCAAAAGAACGGCGGACGGCTCTGAGAAGCGCACCGCTGATAGCCAGCATTCAACTCCTCCGAAAACAAAGAGGAAAGTTGAGTTCGAACCGAAGAAGAAAAGGAAGGTGTGA

**Sequence 16 Plasmids sequence of ePPEplus-RT15**

(NLSSV40-nCas9(H840A/R221K/N394K)-XTEN-NC-NLS-32aa Linker-RT15-NLSvbp)

CCTAAGAAAAAGAGAAAAGTGGACAAGAAGTACTCGATCGGCCTCGATATTGGGACTAACTCTGTTGGCTGGGCCGTGATCACCGACGAGTACAAGGTGCCCTCAAAGAAGTTCAAGGTCCTGGGCAACACCGATCGGCATTCCATCAAGAAGAATCTCATTGGCGCTCTCCTGTTCGACAGCGGCGAGACGGCTGAGGCTACGCGGCTCAAGCGCACCGCCCGCAGGCGGTACACGCGCAGGAAGAATCGCATCTGCTACCTGCAGGAGATTTTCTCCAACGAGATGGCGAAGGTTGACGATTCTTTCTTCCACAGGCTGGAGGAGTCATTCCTCGTGGAGGAGGATAAGAAGCACGAGCGGCATCCAATCTTCGGCAACATTGTCGACGAGGTTGCCTACCACGAGAAGTACCCTACGATCTACCATCTGCGGAAGAAGCTCGTGGACTCCACAGATAAGGCGGACCTCCGCCTGATCTACCTCGCTCTGGCCCACATGATTAAGTTCAGGGGCCATTTCCTGATCGAGGGGGATCTCAACCCGGACAATAGCGATGTTGACAAGCTGTTCATCCAGCTCGTGCAGACGTACAACCAGCTCTTCGAGGAGAACCCCATTAATGCGTCAGGCGTCGACGCGAAGGCTATCCTGTCCGCTAGGCTCTCGAAGTCTCGGAAGCTCGAGAACCTGATCGCCCAGCTGCCGGGCGAGAAGAAGAACGGCCTGTTCGGGAATCTCATTGCGCTCAGCCTGGGGCTCACGCCCAACTTCAAGTCGAATTTCGATCTCGCTGAGGACGCCAAGCTGCAGCTCTCCAAGGACACATACGACGATGACCTGGATAACCTCCTGGCCCAGATCGGCGATCAGTACGCGGACCTGTTCCTCGCTGCCAAGAATCTGTCGGACGCCATCCTCCTGTCTGATATTCTCAGGGTGAACACCGAGATTACGAAGGCTCCGCTCTCAGCCTCCATGATCAAGCGCTACGACGAGCACCATCAGGATCTGACCCTCCTGAAGGCGCTGGTCAGGCAGCAGCTCCCCGAGAAGTACAAGGAGATCTTCTTCGATCAGTCGAAGAACGGCTACGCTGGGTACATTGACGGCGGGGCCTCTCAGGAGGAGTTCTACAAGTTCATCAAGCCGATTCTGGAGAAGATGGACGGCACGGAGGAGCTGCTGGTGAAGCTCAAGCGCGAGGACCTCCTGAGGAAGCAGCGGACATTCGATAACGGCAGCATCCCACACCAGATTCATCTCGGGGAGCTGCACGCTATCCTGAGGAGGCAGGAGGACTTCTACCCTTTCCTCAAGGATAACCGCGAGAAGATCGAGAAGATTCTGACTTTCAGGATCCCGTACTACGTCGGCCCACTCGCTAGGGGCAACTCCCGCTTCGCTTGGATGACCCGCAAGTCAGAGGAGACGATCACGCCGTGGAACTTCGAGGAGGTGGTCGACAAGGGCGCTAGCGCTCAGTCGTTCATCGAGAGGATGACGAATTTCGACAAGAACCTGCCAAATGAGAAGGTGCTCCCTAAGCACTCGCTCCTGTACGAGTACTTCACAGTCTACAACGAGCTGACTAAGGTGAAGTATGTGACCGAGGGCATGAGGAAGCCGGCTTTCCTGTCTGGGGAGCAGAAGAAGGCCATCGTGGACCTCCTGTTCAAGACCAACCGGAAGGTCACGGTTAAGCAGCTCAAGGAGGACTACTTCAAGAAGATTGAGTGCTTCGATTCGGTCGAGATCTCTGGCGTTGAGGACCGCTTCAACGCCTCCCTGGGGACCTACCACGATCTCCTGAAGATCATTAAGGATAAGGACTTCCTGGACAACGAGGAGAATGAGGATATCCTCGAGGACATTGTGCTGACACTCACTCTGTTCGAGGACCGGGAGATGATCGAGGAGCGCCTGAAGACTTACGCCCATCTCTTCGATGACAAGGTCATGAAGCAGCTCAAGAGGAGGAGGTACACCGGCTGGGGGAGGCTGAGCAGGAAGCTCATCAACGGCATTCGGGACAAGCAGTCCGGGAAGACGATCCTCGACTTCCTGAAGAGCGATGGCTTCGCGAACCGCAATTTCATGCAGCTGATTCACGATGACAGCCTCACATTCAAGGAGGATATCCAGAAGGCTCAGGTGAGCGGCCAGGGGGACTCGCTGCACGAGCATATCGCGAACCTCGCTGGCTCGCCAGCTATCAAGAAGGGGATTCTGCAGACCGTGAAGGTTGTGGACGAGCTGGTGAAGGTCATGGGCAGGCACAAGCCTGAGAACATCGTCATTGAGATGGCCCGGGAGAATCAGACCACGCAGAAGGGCCAGAAGAACTCACGCGAGAGGATGAAGAGGATCGAGGAGGGCATTAAGGAGCTGGGGTCCCAGATCCTCAAGGAGCACCCGGTGGAGAACACGCAGCTGCAGAATGAGAAGCTCTACCTGTACTACCTCCAGAATGGCCGCGATATGTATGTGGACCAGGAGCTGGATATTAACAGGCTCAGCGATTACGACGTCGATGCCATCGTTCCACAGTCATTCCTGAAGGATGACTCCATTGACAACAAGGTCCTCACCAGGTCGGACAAGAACCGGGGCAAGTCTGATAATGTTCCTTCAGAGGAGGTCGTTAAGAAGATGAAGAACTACTGGCGCCAGCTCCTGAATGCCAAGCTGATCACGCAGCGGAAGTTCGATAACCTCACAAAGGCTGAGAGGGGCGGGCTCTCTGAGCTGGACAAGGCGGGCTTCATCAAGAGGCAGCTGGTCGAGACACGGCAGATCACTAAGCACGTTGCGCAGATTCTCGACTCACGGATGAACACTAAGTACGATGAGAATGACAAGCTGATCCGCGAGGTGAAGGTCATCACCCTGAAGTCAAAGCTCGTCTCCGACTTCAGGAAGGATTTCCAGTTCTACAAGGTTCGGGAGATCAACAATTACCACCATGCCCATGACGCGTACCTGAACGCGGTGGTCGGCACAGCTCTGATCAAGAAGTACCCAAAGCTCGAGAGCGAGTTCGTGTACGGGGACTACAAGGTTTACGATGTGAGGAAGATGATCGCCAAGTCGGAGCAGGAGATTGGCAAGGCTACCGCCAAGTACTTCTTCTACTCTAACATTATGAATTTCTTCAAGACAGAGATCACTCTGGCCAATGGCGAGATCCGGAAGCGCCCCCTCATCGAGACGAACGGCGAGACGGGGGAGATCGTGTGGGACAAGGGCAGGGATTTCGCGACCGTCAGGAAGGTTCTCTCCATGCCACAAGTGAATATCGTCAAGAAGACAGAGGTCCAGACTGGCGGGTTCTCTAAGGAGTCAATTCTGCCTAAGCGGAACAGCGACAAGCTCATCGCCCGCAAGAAGGACTGGGATCCGAAGAAGTACGGCGGGTTCGACAGCCCCACTGTGGCCTACTCGGTCCTGGTTGTGGCGAAGGTTGAGAAGGGCAAGTCCAAGAAGCTCAAGAGCGTGAAGGAGCTGCTGGGGATCACGATTATGGAGCGCTCCAGCTTCGAGAAGAACCCGATCGATTTCCTGGAGGCGAAGGGCTACAAGGAGGTGAAGAAGGACCTGATCATTAAGCTCCCCAAGTACTCACTCTTCGAGCTGGAGAACGGCAGGAAGCGGATGCTGGCTTCCGCTGGCGAGCTGCAGAAGGGGAACGAGCTGGCTCTGCCGTCCAAGTATGTGAACTTCCTCTACCTGGCCTCCCACTACGAGAAGCTCAAGGGCAGCCCCGAGGACAACGAGCAGAAGCAGCTGTTCGTCGAGCAGCACAAGCATTACCTCGACGAGATCATTGAGCAGATTTCCGAGTTCTCCAAGCGCGTGATCCTGGCCGACGCGAATCTGGATAAGGTCCTCTCCGCGTACAACAAGCACCGCGACAAGCCAATCAGGGAGCAGGCTGAGAATATCATTCATCTCTTCACCCTGACGAACCTCGGCGCCCCTGCTGCTTTCAAGTACTTCGACACAACTATCGATCGCAAGAGGTACACAAGCACTAAGGAGGTCCTGGACGCGACCCTCATCCACCAGTCGATTACCGGCCTCTACGAGACGCGCATCGACCTGTCTCAGCTCGGGGGCGACGAATTCTCCGGGAGCGAGACGCCAGGCACCTCCGAGTCGGCCACCCCAGAATCTGCCACAGTGGTGTCCGGCCAAAAGCAGGACCGCCAGGGCGGAGAACGCAGAAGGTCCCAGCTCGATAGGGATCAGTGTGCCTACTGCAAGGAGAAGGGCCACTGGGCCAAAGACTGCCCGAAAAAGCCGCGCGGCCCACGCGGCCCAAGGCCACAAACATCCCTCCTTCCAAAGAAGAAGCGGAAGGTGGAGCTCAGCGGAGGATCTTCCGGAGGATCTAGCGGCTCCGAGACACCAGGAACATCCGAAAGCGCTACACCAGAATCTAGCGGAGGCTCTTCCGGAGGATCTAGGCCTTGGCTGCGGCTGGTACCTGAAGCGTGGGCGGAGACGGCGGGGCTTGGTCTCTCGACCCTTCAAGCTCCTGTTGTTGTCGAACTCAAGACGACCGCCGTTCCAGTTCGCGTCCGGCAGTACCCGATGAGCCAGGAGGCCAAGAAAGGAATTACTGGATTTCTTGTCAAGTGTCAAAGTGCATGGAACACCCCGCTCCTCCCCGTCAAAAAACCAGGAACCGGCGACTATCGGCCGGTGCAAGATCTCCGGGCCGTGAACAGTCAAGTGGAGGTGATCCATCCAACTGTTCCAAATCCCTACAACTTGTTGTCAACGCTGTCCCCCGAGAGGGTGTGGTACACCGTCCTCGACTTGAAAGATGCCTTCTTCTGCCTCTCCCTCCACCCTGCATCTCAACCTCTCTTCGCCTTTGAGTGGAGCGACCCAGAAGCCGGCATTTCTGGTCAGCTGACATGGACAAGACTGCCGCAGGGCTTCAAGAATTCACCCACTATATTTGATGAAGCACTGCACCAGGACTTGTCATTGTTTCGATCGCAGCACCCACAAGTTACGCTGCTGCAGTATGTAGATGATATTCTCATCGCCGGGAAAACTGAAGAGGACTGCAAGCAGGCAACACATAATCTTCTGAAGGAGCTGGCCCGCCTCGGCTATCGTGCTTCCGCGAAGAAGGCGCAGCTCTGCCAGAGGGAGGTCACCTTCCTCGGGTACATGCTGCGCGGCGGCAAGAGATGGCTTACTGAAGCGAGGAAGAAGGTGGTGGCTCAGATCCCGGCGCCGACAACGAGCGGCGGCAGCAAAAGAACGGCGGACGGCTCTGAGAAGCGCACCGCTGATAGCCAGCATTCAACTCCTCCGAAAACAAAGAGGAAAGTTGAGTTCGAACCGAAGAAGAAAAGGAAGGTGTGA

**Sequence 17 Plasmids sequence of ePPEplus-RT16**

(NLSSV40-nCas9(H840A/R221K/N394K)-XTEN-NC-NLS-32aa Linker-RT16-NLSvbp)

CCTAAGAAAAAGAGAAAAGTGGACAAGAAGTACTCGATCGGCCTCGATATTGGGACTAACTCTGTTGGCTGGGCCGTGATCACCGACGAGTACAAGGTGCCCTCAAAGAAGTTCAAGGTCCTGGGCAACACCGATCGGCATTCCATCAAGAAGAATCTCATTGGCGCTCTCCTGTTCGACAGCGGCGAGACGGCTGAGGCTACGCGGCTCAAGCGCACCGCCCGCAGGCGGTACACGCGCAGGAAGAATCGCATCTGCTACCTGCAGGAGATTTTCTCCAACGAGATGGCGAAGGTTGACGATTCTTTCTTCCACAGGCTGGAGGAGTCATTCCTCGTGGAGGAGGATAAGAAGCACGAGCGGCATCCAATCTTCGGCAACATTGTCGACGAGGTTGCCTACCACGAGAAGTACCCTACGATCTACCATCTGCGGAAGAAGCTCGTGGACTCCACAGATAAGGCGGACCTCCGCCTGATCTACCTCGCTCTGGCCCACATGATTAAGTTCAGGGGCCATTTCCTGATCGAGGGGGATCTCAACCCGGACAATAGCGATGTTGACAAGCTGTTCATCCAGCTCGTGCAGACGTACAACCAGCTCTTCGAGGAGAACCCCATTAATGCGTCAGGCGTCGACGCGAAGGCTATCCTGTCCGCTAGGCTCTCGAAGTCTCGGAAGCTCGAGAACCTGATCGCCCAGCTGCCGGGCGAGAAGAAGAACGGCCTGTTCGGGAATCTCATTGCGCTCAGCCTGGGGCTCACGCCCAACTTCAAGTCGAATTTCGATCTCGCTGAGGACGCCAAGCTGCAGCTCTCCAAGGACACATACGACGATGACCTGGATAACCTCCTGGCCCAGATCGGCGATCAGTACGCGGACCTGTTCCTCGCTGCCAAGAATCTGTCGGACGCCATCCTCCTGTCTGATATTCTCAGGGTGAACACCGAGATTACGAAGGCTCCGCTCTCAGCCTCCATGATCAAGCGCTACGACGAGCACCATCAGGATCTGACCCTCCTGAAGGCGCTGGTCAGGCAGCAGCTCCCCGAGAAGTACAAGGAGATCTTCTTCGATCAGTCGAAGAACGGCTACGCTGGGTACATTGACGGCGGGGCCTCTCAGGAGGAGTTCTACAAGTTCATCAAGCCGATTCTGGAGAAGATGGACGGCACGGAGGAGCTGCTGGTGAAGCTCAAGCGCGAGGACCTCCTGAGGAAGCAGCGGACATTCGATAACGGCAGCATCCCACACCAGATTCATCTCGGGGAGCTGCACGCTATCCTGAGGAGGCAGGAGGACTTCTACCCTTTCCTCAAGGATAACCGCGAGAAGATCGAGAAGATTCTGACTTTCAGGATCCCGTACTACGTCGGCCCACTCGCTAGGGGCAACTCCCGCTTCGCTTGGATGACCCGCAAGTCAGAGGAGACGATCACGCCGTGGAACTTCGAGGAGGTGGTCGACAAGGGCGCTAGCGCTCAGTCGTTCATCGAGAGGATGACGAATTTCGACAAGAACCTGCCAAATGAGAAGGTGCTCCCTAAGCACTCGCTCCTGTACGAGTACTTCACAGTCTACAACGAGCTGACTAAGGTGAAGTATGTGACCGAGGGCATGAGGAAGCCGGCTTTCCTGTCTGGGGAGCAGAAGAAGGCCATCGTGGACCTCCTGTTCAAGACCAACCGGAAGGTCACGGTTAAGCAGCTCAAGGAGGACTACTTCAAGAAGATTGAGTGCTTCGATTCGGTCGAGATCTCTGGCGTTGAGGACCGCTTCAACGCCTCCCTGGGGACCTACCACGATCTCCTGAAGATCATTAAGGATAAGGACTTCCTGGACAACGAGGAGAATGAGGATATCCTCGAGGACATTGTGCTGACACTCACTCTGTTCGAGGACCGGGAGATGATCGAGGAGCGCCTGAAGACTTACGCCCATCTCTTCGATGACAAGGTCATGAAGCAGCTCAAGAGGAGGAGGTACACCGGCTGGGGGAGGCTGAGCAGGAAGCTCATCAACGGCATTCGGGACAAGCAGTCCGGGAAGACGATCCTCGACTTCCTGAAGAGCGATGGCTTCGCGAACCGCAATTTCATGCAGCTGATTCACGATGACAGCCTCACATTCAAGGAGGATATCCAGAAGGCTCAGGTGAGCGGCCAGGGGGACTCGCTGCACGAGCATATCGCGAACCTCGCTGGCTCGCCAGCTATCAAGAAGGGGATTCTGCAGACCGTGAAGGTTGTGGACGAGCTGGTGAAGGTCATGGGCAGGCACAAGCCTGAGAACATCGTCATTGAGATGGCCCGGGAGAATCAGACCACGCAGAAGGGCCAGAAGAACTCACGCGAGAGGATGAAGAGGATCGAGGAGGGCATTAAGGAGCTGGGGTCCCAGATCCTCAAGGAGCACCCGGTGGAGAACACGCAGCTGCAGAATGAGAAGCTCTACCTGTACTACCTCCAGAATGGCCGCGATATGTATGTGGACCAGGAGCTGGATATTAACAGGCTCAGCGATTACGACGTCGATGCCATCGTTCCACAGTCATTCCTGAAGGATGACTCCATTGACAACAAGGTCCTCACCAGGTCGGACAAGAACCGGGGCAAGTCTGATAATGTTCCTTCAGAGGAGGTCGTTAAGAAGATGAAGAACTACTGGCGCCAGCTCCTGAATGCCAAGCTGATCACGCAGCGGAAGTTCGATAACCTCACAAAGGCTGAGAGGGGCGGGCTCTCTGAGCTGGACAAGGCGGGCTTCATCAAGAGGCAGCTGGTCGAGACACGGCAGATCACTAAGCACGTTGCGCAGATTCTCGACTCACGGATGAACACTAAGTACGATGAGAATGACAAGCTGATCCGCGAGGTGAAGGTCATCACCCTGAAGTCAAAGCTCGTCTCCGACTTCAGGAAGGATTTCCAGTTCTACAAGGTTCGGGAGATCAACAATTACCACCATGCCCATGACGCGTACCTGAACGCGGTGGTCGGCACAGCTCTGATCAAGAAGTACCCAAAGCTCGAGAGCGAGTTCGTGTACGGGGACTACAAGGTTTACGATGTGAGGAAGATGATCGCCAAGTCGGAGCAGGAGATTGGCAAGGCTACCGCCAAGTACTTCTTCTACTCTAACATTATGAATTTCTTCAAGACAGAGATCACTCTGGCCAATGGCGAGATCCGGAAGCGCCCCCTCATCGAGACGAACGGCGAGACGGGGGAGATCGTGTGGGACAAGGGCAGGGATTTCGCGACCGTCAGGAAGGTTCTCTCCATGCCACAAGTGAATATCGTCAAGAAGACAGAGGTCCAGACTGGCGGGTTCTCTAAGGAGTCAATTCTGCCTAAGCGGAACAGCGACAAGCTCATCGCCCGCAAGAAGGACTGGGATCCGAAGAAGTACGGCGGGTTCGACAGCCCCACTGTGGCCTACTCGGTCCTGGTTGTGGCGAAGGTTGAGAAGGGCAAGTCCAAGAAGCTCAAGAGCGTGAAGGAGCTGCTGGGGATCACGATTATGGAGCGCTCCAGCTTCGAGAAGAACCCGATCGATTTCCTGGAGGCGAAGGGCTACAAGGAGGTGAAGAAGGACCTGATCATTAAGCTCCCCAAGTACTCACTCTTCGAGCTGGAGAACGGCAGGAAGCGGATGCTGGCTTCCGCTGGCGAGCTGCAGAAGGGGAACGAGCTGGCTCTGCCGTCCAAGTATGTGAACTTCCTCTACCTGGCCTCCCACTACGAGAAGCTCAAGGGCAGCCCCGAGGACAACGAGCAGAAGCAGCTGTTCGTCGAGCAGCACAAGCATTACCTCGACGAGATCATTGAGCAGATTTCCGAGTTCTCCAAGCGCGTGATCCTGGCCGACGCGAATCTGGATAAGGTCCTCTCCGCGTACAACAAGCACCGCGACAAGCCAATCAGGGAGCAGGCTGAGAATATCATTCATCTCTTCACCCTGACGAACCTCGGCGCCCCTGCTGCTTTCAAGTACTTCGACACAACTATCGATCGCAAGAGGTACACAAGCACTAAGGAGGTCCTGGACGCGACCCTCATCCACCAGTCGATTACCGGCCTCTACGAGACGCGCATCGACCTGTCTCAGCTCGGGGGCGACGAATTCTCCGGGAGCGAGACGCCAGGCACCTCCGAGTCGGCCACCCCAGAATCTGCCACAGTGGTGTCCGGCCAAAAGCAGGACCGCCAGGGCGGAGAACGCAGAAGGTCCCAGCTCGATAGGGATCAGTGTGCCTACTGCAAGGAGAAGGGCCACTGGGCCAAAGACTGCCCGAAAAAGCCGCGCGGCCCACGCGGCCCAAGGCCACAAACATCCCTCCTTCCAAAGAAGAAGCGGAAGGTGGAGCTCAGCGGAGGATCTTCCGGAGGATCTAGCGGCTCCGAGACACCAGGAACATCCGAAAGCGCTACACCAGAATCTAGCGGAGGCTCTTCCGGAGGATCTAGGCCTATCAAGGAGCAACTTAGGACCAGGAAGTACAAGCCGCAGCCGGTGCGCCGTGTGGAGATCCCCAAGCCAGATGGCGGCGTCAGAAATCTTGGCGTGCCGACGGTAACTGATAGATTTGTGCAGCAAGCAATTGCTCAGGTGCTCACCCCCATCTACGAGGAGCAGTTCCATGCGCACTCCTACGGCTTTCGGCCAAATCGGTGCGCGCAGCAGGCCATCCTCACGGCGCTCGACATGATGAACGACGGAAATGACTGGATTGTTGACATCGACCTGGAGAAATTTTTCGACACCGTCAATCATGACAAGCTGATGACAATAATTGGTCGCACCATTAAAGATGGTGATGTTATATCCATCGTCCGCAAATATCTTGTCTCTGGAATAATGATTGATGATGAATATGAAGATAGTATAGTTGGGACTCCACAAGGTGGCAATTTGAGTCCTCTTCTTGCAAATATTATGCTGAATGAGCTGGACATGGAAATGGAGACAAGAGGGCTGAACTTCGTCCGGTACGCCGACGACTGTATCATCATGGTGCGGTCCGAGATGTCGGCCAACCGCGTCATGAGGAACATTTCACGATTCATTGAAGAAAAATTAGGGCTCAAGGTGAACATGACCAAGAGCAAGGTTGATAGGCCGGACGGCTTGAAGTATTTGGGATTTGGGTTCTTCTTTGATACTCAAGCTCAACAGTATAAGGCGCGGCCTCACGCCAAGTCAATAGCAAAACTAAAAACAAAGATGAAATGGCTCACAAGAAGGAACTGGTCTGTTAGCAATAGGTACAAGATTGAGAAGCTCAACCAGCTCACTCGTGGGTGGATCAACTACTTCGGCATCGGCTACATGAAGTGGCTGTGCAAGGATATGGATGCTTTAATCCGGAGACGCCTCAGGATGTGCATTTGGGTAAGCGGCGGCAGCAAAAGAACGGCGGACGGCTCTGAGAAGCGCACCGCTGATAGCCAGCATTCAACTCCTCCGAAAACAAAGAGGAAAGTTGAGTTCGAACCGAAGAAGAAAAGGAAGGTGTGA

**Sequence 18 Plasmids sequence of ePPEplus-RT17**

(NLSSV40-nCas9(H840A/R221K/N394K)-XTEN-NC-NLS-32aa Linker-RT17-NLSvbp)

CCTAAGAAAAAGAGAAAAGTGGACAAGAAGTACTCGATCGGCCTCGATATTGGGACTAACTCTGTTGGCTGGGCCGTGATCACCGACGAGTACAAGGTGCCCTCAAAGAAGTTCAAGGTCCTGGGCAACACCGATCGGCATTCCATCAAGAAGAATCTCATTGGCGCTCTCCTGTTCGACAGCGGCGAGACGGCTGAGGCTACGCGGCTCAAGCGCACCGCCCGCAGGCGGTACACGCGCAGGAAGAATCGCATCTGCTACCTGCAGGAGATTTTCTCCAACGAGATGGCGAAGGTTGACGATTCTTTCTTCCACAGGCTGGAGGAGTCATTCCTCGTGGAGGAGGATAAGAAGCACGAGCGGCATCCAATCTTCGGCAACATTGTCGACGAGGTTGCCTACCACGAGAAGTACCCTACGATCTACCATCTGCGGAAGAAGCTCGTGGACTCCACAGATAAGGCGGACCTCCGCCTGATCTACCTCGCTCTGGCCCACATGATTAAGTTCAGGGGCCATTTCCTGATCGAGGGGGATCTCAACCCGGACAATAGCGATGTTGACAAGCTGTTCATCCAGCTCGTGCAGACGTACAACCAGCTCTTCGAGGAGAACCCCATTAATGCGTCAGGCGTCGACGCGAAGGCTATCCTGTCCGCTAGGCTCTCGAAGTCTCGGAAGCTCGAGAACCTGATCGCCCAGCTGCCGGGCGAGAAGAAGAACGGCCTGTTCGGGAATCTCATTGCGCTCAGCCTGGGGCTCACGCCCAACTTCAAGTCGAATTTCGATCTCGCTGAGGACGCCAAGCTGCAGCTCTCCAAGGACACATACGACGATGACCTGGATAACCTCCTGGCCCAGATCGGCGATCAGTACGCGGACCTGTTCCTCGCTGCCAAGAATCTGTCGGACGCCATCCTCCTGTCTGATATTCTCAGGGTGAACACCGAGATTACGAAGGCTCCGCTCTCAGCCTCCATGATCAAGCGCTACGACGAGCACCATCAGGATCTGACCCTCCTGAAGGCGCTGGTCAGGCAGCAGCTCCCCGAGAAGTACAAGGAGATCTTCTTCGATCAGTCGAAGAACGGCTACGCTGGGTACATTGACGGCGGGGCCTCTCAGGAGGAGTTCTACAAGTTCATCAAGCCGATTCTGGAGAAGATGGACGGCACGGAGGAGCTGCTGGTGAAGCTCAAGCGCGAGGACCTCCTGAGGAAGCAGCGGACATTCGATAACGGCAGCATCCCACACCAGATTCATCTCGGGGAGCTGCACGCTATCCTGAGGAGGCAGGAGGACTTCTACCCTTTCCTCAAGGATAACCGCGAGAAGATCGAGAAGATTCTGACTTTCAGGATCCCGTACTACGTCGGCCCACTCGCTAGGGGCAACTCCCGCTTCGCTTGGATGACCCGCAAGTCAGAGGAGACGATCACGCCGTGGAACTTCGAGGAGGTGGTCGACAAGGGCGCTAGCGCTCAGTCGTTCATCGAGAGGATGACGAATTTCGACAAGAACCTGCCAAATGAGAAGGTGCTCCCTAAGCACTCGCTCCTGTACGAGTACTTCACAGTCTACAACGAGCTGACTAAGGTGAAGTATGTGACCGAGGGCATGAGGAAGCCGGCTTTCCTGTCTGGGGAGCAGAAGAAGGCCATCGTGGACCTCCTGTTCAAGACCAACCGGAAGGTCACGGTTAAGCAGCTCAAGGAGGACTACTTCAAGAAGATTGAGTGCTTCGATTCGGTCGAGATCTCTGGCGTTGAGGACCGCTTCAACGCCTCCCTGGGGACCTACCACGATCTCCTGAAGATCATTAAGGATAAGGACTTCCTGGACAACGAGGAGAATGAGGATATCCTCGAGGACATTGTGCTGACACTCACTCTGTTCGAGGACCGGGAGATGATCGAGGAGCGCCTGAAGACTTACGCCCATCTCTTCGATGACAAGGTCATGAAGCAGCTCAAGAGGAGGAGGTACACCGGCTGGGGGAGGCTGAGCAGGAAGCTCATCAACGGCATTCGGGACAAGCAGTCCGGGAAGACGATCCTCGACTTCCTGAAGAGCGATGGCTTCGCGAACCGCAATTTCATGCAGCTGATTCACGATGACAGCCTCACATTCAAGGAGGATATCCAGAAGGCTCAGGTGAGCGGCCAGGGGGACTCGCTGCACGAGCATATCGCGAACCTCGCTGGCTCGCCAGCTATCAAGAAGGGGATTCTGCAGACCGTGAAGGTTGTGGACGAGCTGGTGAAGGTCATGGGCAGGCACAAGCCTGAGAACATCGTCATTGAGATGGCCCGGGAGAATCAGACCACGCAGAAGGGCCAGAAGAACTCACGCGAGAGGATGAAGAGGATCGAGGAGGGCATTAAGGAGCTGGGGTCCCAGATCCTCAAGGAGCACCCGGTGGAGAACACGCAGCTGCAGAATGAGAAGCTCTACCTGTACTACCTCCAGAATGGCCGCGATATGTATGTGGACCAGGAGCTGGATATTAACAGGCTCAGCGATTACGACGTCGATGCCATCGTTCCACAGTCATTCCTGAAGGATGACTCCATTGACAACAAGGTCCTCACCAGGTCGGACAAGAACCGGGGCAAGTCTGATAATGTTCCTTCAGAGGAGGTCGTTAAGAAGATGAAGAACTACTGGCGCCAGCTCCTGAATGCCAAGCTGATCACGCAGCGGAAGTTCGATAACCTCACAAAGGCTGAGAGGGGCGGGCTCTCTGAGCTGGACAAGGCGGGCTTCATCAAGAGGCAGCTGGTCGAGACACGGCAGATCACTAAGCACGTTGCGCAGATTCTCGACTCACGGATGAACACTAAGTACGATGAGAATGACAAGCTGATCCGCGAGGTGAAGGTCATCACCCTGAAGTCAAAGCTCGTCTCCGACTTCAGGAAGGATTTCCAGTTCTACAAGGTTCGGGAGATCAACAATTACCACCATGCCCATGACGCGTACCTGAACGCGGTGGTCGGCACAGCTCTGATCAAGAAGTACCCAAAGCTCGAGAGCGAGTTCGTGTACGGGGACTACAAGGTTTACGATGTGAGGAAGATGATCGCCAAGTCGGAGCAGGAGATTGGCAAGGCTACCGCCAAGTACTTCTTCTACTCTAACATTATGAATTTCTTCAAGACAGAGATCACTCTGGCCAATGGCGAGATCCGGAAGCGCCCCCTCATCGAGACGAACGGCGAGACGGGGGAGATCGTGTGGGACAAGGGCAGGGATTTCGCGACCGTCAGGAAGGTTCTCTCCATGCCACAAGTGAATATCGTCAAGAAGACAGAGGTCCAGACTGGCGGGTTCTCTAAGGAGTCAATTCTGCCTAAGCGGAACAGCGACAAGCTCATCGCCCGCAAGAAGGACTGGGATCCGAAGAAGTACGGCGGGTTCGACAGCCCCACTGTGGCCTACTCGGTCCTGGTTGTGGCGAAGGTTGAGAAGGGCAAGTCCAAGAAGCTCAAGAGCGTGAAGGAGCTGCTGGGGATCACGATTATGGAGCGCTCCAGCTTCGAGAAGAACCCGATCGATTTCCTGGAGGCGAAGGGCTACAAGGAGGTGAAGAAGGACCTGATCATTAAGCTCCCCAAGTACTCACTCTTCGAGCTGGAGAACGGCAGGAAGCGGATGCTGGCTTCCGCTGGCGAGCTGCAGAAGGGGAACGAGCTGGCTCTGCCGTCCAAGTATGTGAACTTCCTCTACCTGGCCTCCCACTACGAGAAGCTCAAGGGCAGCCCCGAGGACAACGAGCAGAAGCAGCTGTTCGTCGAGCAGCACAAGCATTACCTCGACGAGATCATTGAGCAGATTTCCGAGTTCTCCAAGCGCGTGATCCTGGCCGACGCGAATCTGGATAAGGTCCTCTCCGCGTACAACAAGCACCGCGACAAGCCAATCAGGGAGCAGGCTGAGAATATCATTCATCTCTTCACCCTGACGAACCTCGGCGCCCCTGCTGCTTTCAAGTACTTCGACACAACTATCGATCGCAAGAGGTACACAAGCACTAAGGAGGTCCTGGACGCGACCCTCATCCACCAGTCGATTACCGGCCTCTACGAGACGCGCATCGACCTGTCTCAGCTCGGGGGCGACGAATTCTCCGGGAGCGAGACGCCAGGCACCTCCGAGTCGGCCACCCCAGAATCTGCCACAGTGGTGTCCGGCCAAAAGCAGGACCGCCAGGGCGGAGAACGCAGAAGGTCCCAGCTCGATAGGGATCAGTGTGCCTACTGCAAGGAGAAGGGCCACTGGGCCAAAGACTGCCCGAAAAAGCCGCGCGGCCCACGCGGCCCAAGGCCACAAACATCCCTCCTTCCAAAGAAGAAGCGGAAGGTGGAGCTCAGCGGAGGATCTTCCGGAGGATCTAGCGGCTCCGAGACACCAGGAACATCCGAAAGCGCTACACCAGAATCTAGCGGAGGCTCTTCCGGAGGATCTAGGCCTATGGTGCCAATGGGTGCCTTACAGCCGGGCATTCCCAGCCCTGTTGCCATCCCGCGGGGCTACGTGAAGCTCATGATTGACCTGAAGGATTGCTTCTTCAGTATTCCTCTCCACCCAAAGGACTGTAAAAGGTTCGCTTTCACGCTACCGATGATCAACTGCATCGGCCCTTCTCCACGATTTCAGTGGAAGGTGCTGCCACAGGGTATGGCCAACTCGCCCACCCTATGCCAAAGATATGTAGCTCAAGTCATCGACCCCTTTAGGATGTCCTATCCTGACCTGTACGTGGTGCACTACATGGATGATATCTTGGTGGCCGGCCCTGACCAAGACCAGCTTTACTCTGCCTCACAGGAGCTGATCAATGCCCTGCAGAATCAGGGCCTCCAGGTCTCCCCGGAGAAGGTGCAAATTCACCCCCCACACCTCTTCCTTGGGTTTGAACTCTTCCCCAACAAGATACTGTCCCAGAAGGTTCAGGTGAGACAAGATTCACTGCAAACACTTAACGACTTCCAGAGGCTCCTCGGCGACATCAACTGGCTAAGACCATACCTTAAATTAACCACCGGCGAGCTGAAACCTCTGTTTGACATACTAAGAGGAGACCCTGATCCATCTTCACCTCGCATGCTCACACAGGAAGCGAGACGTAGCCTTGCCAAGGTGGAGCAAGCTATCTCCGAGCAGAACATTGGGTATTTCTCCCCTGAACTGCCTCTGCAGCTCCTCGTCTTCCCTACTCCGTTCTCTCCCACCGGCCTGCTCTGGCAACTGAAGCCGCTGTTCTGGGTTCACATGAGCGCCAGCTCGTCTAAAGTGTTACCTACATACCCACAGCTCGTGGCAAATGTTCTCCGCCTGGGTCGGGAGGCCGCCCTAAAGCTGTTTGGAAGGGACCCAGACGTAATCGTGTTGCCTTATGATGCATCCCAGGTTCAATGGCTGCTGAAGAATAATGATGACTGGGCGGTAAACTGTATCTCCTTCCAGGGTGTGATAGACAACCATTACCCTGCCGATAAGCTGGTGCAGTTCCTCCATCCGGTGGTGTTCCCCAAGAAGAGCAGCCCAATCCCTGGCGCCATGTTGGTCTTCACCGACGGCTCCAGCAGCGGAATGGCGGCGTTCTACATCAATGGGAAGGTTTCACGGTTCATGACGGATTTCAGCTCGGCGCAGCTAGTGAAACTCGCCGCCATCGTAAAGGTGTTTGAACAACTCCCTAAAACATCTTTCAACCTTTACACCGATTCTGCATACGTCGCTGCCTCCGTGCCGTTGCTGGAGACCGTCCCTTACATCAGGCCTTCAACCAACGCCTCTCCGATGTTTGCTAAGCTGCAGTCTCTAATCCTGGCAAGAAACTTTCCTTTTTTTATTGGACACATCAGAGCACACTCAGGACTTCCTGGCCCCCTGTCTGAAGGCAATGACATCGTGGACCAGGCTACTCAGGTGATTGCCAGCGGCGGCAGCAAAAGAACGGCGGACGGCTCTGAGAAGCGCACCGCTGATAGCCAGCATTCAACTCCTCCGAAAACAAAGAGGAAAGTTGAGTTCGAACCGAAGAAGAAAAGGAAGGTGTGA

**Sequence 19 Plasmids sequence of ePPEplus-RT18**

(NLSSV40-nCas9(H840A/R221K/N394K)-XTEN-NC-NLS-32aa Linker-RT18-NLSvbp)

CCTAAGAAAAAGAGAAAAGTGGACAAGAAGTACTCGATCGGCCTCGATATTGGGACTAACTCTGTTGGCTGGGCCGTGATCACCGACGAGTACAAGGTGCCCTCAAAGAAGTTCAAGGTCCTGGGCAACACCGATCGGCATTCCATCAAGAAGAATCTCATTGGCGCTCTCCTGTTCGACAGCGGCGAGACGGCTGAGGCTACGCGGCTCAAGCGCACCGCCCGCAGGCGGTACACGCGCAGGAAGAATCGCATCTGCTACCTGCAGGAGATTTTCTCCAACGAGATGGCGAAGGTTGACGATTCTTTCTTCCACAGGCTGGAGGAGTCATTCCTCGTGGAGGAGGATAAGAAGCACGAGCGGCATCCAATCTTCGGCAACATTGTCGACGAGGTTGCCTACCACGAGAAGTACCCTACGATCTACCATCTGCGGAAGAAGCTCGTGGACTCCACAGATAAGGCGGACCTCCGCCTGATCTACCTCGCTCTGGCCCACATGATTAAGTTCAGGGGCCATTTCCTGATCGAGGGGGATCTCAACCCGGACAATAGCGATGTTGACAAGCTGTTCATCCAGCTCGTGCAGACGTACAACCAGCTCTTCGAGGAGAACCCCATTAATGCGTCAGGCGTCGACGCGAAGGCTATCCTGTCCGCTAGGCTCTCGAAGTCTCGGAAGCTCGAGAACCTGATCGCCCAGCTGCCGGGCGAGAAGAAGAACGGCCTGTTCGGGAATCTCATTGCGCTCAGCCTGGGGCTCACGCCCAACTTCAAGTCGAATTTCGATCTCGCTGAGGACGCCAAGCTGCAGCTCTCCAAGGACACATACGACGATGACCTGGATAACCTCCTGGCCCAGATCGGCGATCAGTACGCGGACCTGTTCCTCGCTGCCAAGAATCTGTCGGACGCCATCCTCCTGTCTGATATTCTCAGGGTGAACACCGAGATTACGAAGGCTCCGCTCTCAGCCTCCATGATCAAGCGCTACGACGAGCACCATCAGGATCTGACCCTCCTGAAGGCGCTGGTCAGGCAGCAGCTCCCCGAGAAGTACAAGGAGATCTTCTTCGATCAGTCGAAGAACGGCTACGCTGGGTACATTGACGGCGGGGCCTCTCAGGAGGAGTTCTACAAGTTCATCAAGCCGATTCTGGAGAAGATGGACGGCACGGAGGAGCTGCTGGTGAAGCTCAAGCGCGAGGACCTCCTGAGGAAGCAGCGGACATTCGATAACGGCAGCATCCCACACCAGATTCATCTCGGGGAGCTGCACGCTATCCTGAGGAGGCAGGAGGACTTCTACCCTTTCCTCAAGGATAACCGCGAGAAGATCGAGAAGATTCTGACTTTCAGGATCCCGTACTACGTCGGCCCACTCGCTAGGGGCAACTCCCGCTTCGCTTGGATGACCCGCAAGTCAGAGGAGACGATCACGCCGTGGAACTTCGAGGAGGTGGTCGACAAGGGCGCTAGCGCTCAGTCGTTCATCGAGAGGATGACGAATTTCGACAAGAACCTGCCAAATGAGAAGGTGCTCCCTAAGCACTCGCTCCTGTACGAGTACTTCACAGTCTACAACGAGCTGACTAAGGTGAAGTATGTGACCGAGGGCATGAGGAAGCCGGCTTTCCTGTCTGGGGAGCAGAAGAAGGCCATCGTGGACCTCCTGTTCAAGACCAACCGGAAGGTCACGGTTAAGCAGCTCAAGGAGGACTACTTCAAGAAGATTGAGTGCTTCGATTCGGTCGAGATCTCTGGCGTTGAGGACCGCTTCAACGCCTCCCTGGGGACCTACCACGATCTCCTGAAGATCATTAAGGATAAGGACTTCCTGGACAACGAGGAGAATGAGGATATCCTCGAGGACATTGTGCTGACACTCACTCTGTTCGAGGACCGGGAGATGATCGAGGAGCGCCTGAAGACTTACGCCCATCTCTTCGATGACAAGGTCATGAAGCAGCTCAAGAGGAGGAGGTACACCGGCTGGGGGAGGCTGAGCAGGAAGCTCATCAACGGCATTCGGGACAAGCAGTCCGGGAAGACGATCCTCGACTTCCTGAAGAGCGATGGCTTCGCGAACCGCAATTTCATGCAGCTGATTCACGATGACAGCCTCACATTCAAGGAGGATATCCAGAAGGCTCAGGTGAGCGGCCAGGGGGACTCGCTGCACGAGCATATCGCGAACCTCGCTGGCTCGCCAGCTATCAAGAAGGGGATTCTGCAGACCGTGAAGGTTGTGGACGAGCTGGTGAAGGTCATGGGCAGGCACAAGCCTGAGAACATCGTCATTGAGATGGCCCGGGAGAATCAGACCACGCAGAAGGGCCAGAAGAACTCACGCGAGAGGATGAAGAGGATCGAGGAGGGCATTAAGGAGCTGGGGTCCCAGATCCTCAAGGAGCACCCGGTGGAGAACACGCAGCTGCAGAATGAGAAGCTCTACCTGTACTACCTCCAGAATGGCCGCGATATGTATGTGGACCAGGAGCTGGATATTAACAGGCTCAGCGATTACGACGTCGATGCCATCGTTCCACAGTCATTCCTGAAGGATGACTCCATTGACAACAAGGTCCTCACCAGGTCGGACAAGAACCGGGGCAAGTCTGATAATGTTCCTTCAGAGGAGGTCGTTAAGAAGATGAAGAACTACTGGCGCCAGCTCCTGAATGCCAAGCTGATCACGCAGCGGAAGTTCGATAACCTCACAAAGGCTGAGAGGGGCGGGCTCTCTGAGCTGGACAAGGCGGGCTTCATCAAGAGGCAGCTGGTCGAGACACGGCAGATCACTAAGCACGTTGCGCAGATTCTCGACTCACGGATGAACACTAAGTACGATGAGAATGACAAGCTGATCCGCGAGGTGAAGGTCATCACCCTGAAGTCAAAGCTCGTCTCCGACTTCAGGAAGGATTTCCAGTTCTACAAGGTTCGGGAGATCAACAATTACCACCATGCCCATGACGCGTACCTGAACGCGGTGGTCGGCACAGCTCTGATCAAGAAGTACCCAAAGCTCGAGAGCGAGTTCGTGTACGGGGACTACAAGGTTTACGATGTGAGGAAGATGATCGCCAAGTCGGAGCAGGAGATTGGCAAGGCTACCGCCAAGTACTTCTTCTACTCTAACATTATGAATTTCTTCAAGACAGAGATCACTCTGGCCAATGGCGAGATCCGGAAGCGCCCCCTCATCGAGACGAACGGCGAGACGGGGGAGATCGTGTGGGACAAGGGCAGGGATTTCGCGACCGTCAGGAAGGTTCTCTCCATGCCACAAGTGAATATCGTCAAGAAGACAGAGGTCCAGACTGGCGGGTTCTCTAAGGAGTCAATTCTGCCTAAGCGGAACAGCGACAAGCTCATCGCCCGCAAGAAGGACTGGGATCCGAAGAAGTACGGCGGGTTCGACAGCCCCACTGTGGCCTACTCGGTCCTGGTTGTGGCGAAGGTTGAGAAGGGCAAGTCCAAGAAGCTCAAGAGCGTGAAGGAGCTGCTGGGGATCACGATTATGGAGCGCTCCAGCTTCGAGAAGAACCCGATCGATTTCCTGGAGGCGAAGGGCTACAAGGAGGTGAAGAAGGACCTGATCATTAAGCTCCCCAAGTACTCACTCTTCGAGCTGGAGAACGGCAGGAAGCGGATGCTGGCTTCCGCTGGCGAGCTGCAGAAGGGGAACGAGCTGGCTCTGCCGTCCAAGTATGTGAACTTCCTCTACCTGGCCTCCCACTACGAGAAGCTCAAGGGCAGCCCCGAGGACAACGAGCAGAAGCAGCTGTTCGTCGAGCAGCACAAGCATTACCTCGACGAGATCATTGAGCAGATTTCCGAGTTCTCCAAGCGCGTGATCCTGGCCGACGCGAATCTGGATAAGGTCCTCTCCGCGTACAACAAGCACCGCGACAAGCCAATCAGGGAGCAGGCTGAGAATATCATTCATCTCTTCACCCTGACGAACCTCGGCGCCCCTGCTGCTTTCAAGTACTTCGACACAACTATCGATCGCAAGAGGTACACAAGCACTAAGGAGGTCCTGGACGCGACCCTCATCCACCAGTCGATTACCGGCCTCTACGAGACGCGCATCGACCTGTCTCAGCTCGGGGGCGACGAATTCTCCGGGAGCGAGACGCCAGGCACCTCCGAGTCGGCCACCCCAGAATCTGCCACAGTGGTGTCCGGCCAAAAGCAGGACCGCCAGGGCGGAGAACGCAGAAGGTCCCAGCTCGATAGGGATCAGTGTGCCTACTGCAAGGAGAAGGGCCACTGGGCCAAAGACTGCCCGAAAAAGCCGCGCGGCCCACGCGGCCCAAGGCCACAAACATCCCTCCTTCCAAAGAAGAAGCGGAAGGTGGAGCTCAGCGGAGGATCTTCCGGAGGATCTAGCGGCTCCGAGACACCAGGAACATCCGAAAGCGCTACACCAGAATCTAGCGGAGGCTCTTCCGGAGGATCTAGGCCTGAGGAGGAAATAGATCACTGGCACAAGGCAAAGAAATGGATTCCTTCACTTAGAGATCGCAAGGAGATCACCTCCATGTACGAGGAGAAGCTGCTCAATAGGATCCTTGACAAGGATAATCTCAACCAGGCGTTCAAGCAGGTGAAGAAGAACAAGGGCGCCGCTGGCGTAGATGGCATGACAGTTGAAGAGCTCGGGAGCTACATGGCGATCAACAAAGAAGAAATTATATCACAAATACGTCAAAGGAAATATGAGCCCAACCCGGTCCTCAGAGTGGAGATACCAAAACCAAATGGAGGAGTAAGGTTATTGGGAATTCCAACTGTCAAGGACCGCCTCATCCAGCAGGCTATTGCACAAGTTCTAACACCTATTTTTGATCGTAAATTTTCAGAATATTCTTATGGATTTCGCCCCAAGCGGTACGCGGAGATGGCCATCCTGCAAACACTGGAGTTCTTGAATGAAGGGCATGACTGGATCGTCGACATCGACCTCGAGAGGTTCTTCGACACCGTCAACCATGATCGGCTAATGAACCTTATCAGCAAGACGGTGAATGATGGTGATGTTATAAGTTTAATTAGGAAGTTCCTCGTCTCCGGTGTGCAGATCGATGAGGAGTACAAGGAAACTGTGATCGGCACGCCGCAGGGCGGCAACCTCTCGCCGCTGCTTAGCAATATTATGCTGAATGAGCTGGATATGGAACTTGAGAATAGAGGGTTGCATTTTGTTCGCTACGCCGACGACTGCATCATTATGGTGAAATCTGAAATGAGCGGCGGCAGCAAAAGAACGGCGGACGGCTCTGAGAAGCGCACCGCTGATAGCCAGCATTCAACTCCTCCGAAAACAAAGAGGAAAGTTGAGTTCGAACCGAAGAAGAAAAGGAAGGTGTGA

**Sequence 20 Plasmids sequence of ePPEplus-RT19**

(NLSSV40-nCas9(H840A/R221K/N394K)-XTEN-NC-NLS-32aa Linker-RT19-NLSvbp)

CCTAAGAAAAAGAGAAAAGTGGACAAGAAGTACTCGATCGGCCTCGATATTGGGACTAACTCTGTTGGCTGGGCCGTGATCACCGACGAGTACAAGGTGCCCTCAAAGAAGTTCAAGGTCCTGGGCAACACCGATCGGCATTCCATCAAGAAGAATCTCATTGGCGCTCTCCTGTTCGACAGCGGCGAGACGGCTGAGGCTACGCGGCTCAAGCGCACCGCCCGCAGGCGGTACACGCGCAGGAAGAATCGCATCTGCTACCTGCAGGAGATTTTCTCCAACGAGATGGCGAAGGTTGACGATTCTTTCTTCCACAGGCTGGAGGAGTCATTCCTCGTGGAGGAGGATAAGAAGCACGAGCGGCATCCAATCTTCGGCAACATTGTCGACGAGGTTGCCTACCACGAGAAGTACCCTACGATCTACCATCTGCGGAAGAAGCTCGTGGACTCCACAGATAAGGCGGACCTCCGCCTGATCTACCTCGCTCTGGCCCACATGATTAAGTTCAGGGGCCATTTCCTGATCGAGGGGGATCTCAACCCGGACAATAGCGATGTTGACAAGCTGTTCATCCAGCTCGTGCAGACGTACAACCAGCTCTTCGAGGAGAACCCCATTAATGCGTCAGGCGTCGACGCGAAGGCTATCCTGTCCGCTAGGCTCTCGAAGTCTCGGAAGCTCGAGAACCTGATCGCCCAGCTGCCGGGCGAGAAGAAGAACGGCCTGTTCGGGAATCTCATTGCGCTCAGCCTGGGGCTCACGCCCAACTTCAAGTCGAATTTCGATCTCGCTGAGGACGCCAAGCTGCAGCTCTCCAAGGACACATACGACGATGACCTGGATAACCTCCTGGCCCAGATCGGCGATCAGTACGCGGACCTGTTCCTCGCTGCCAAGAATCTGTCGGACGCCATCCTCCTGTCTGATATTCTCAGGGTGAACACCGAGATTACGAAGGCTCCGCTCTCAGCCTCCATGATCAAGCGCTACGACGAGCACCATCAGGATCTGACCCTCCTGAAGGCGCTGGTCAGGCAGCAGCTCCCCGAGAAGTACAAGGAGATCTTCTTCGATCAGTCGAAGAACGGCTACGCTGGGTACATTGACGGCGGGGCCTCTCAGGAGGAGTTCTACAAGTTCATCAAGCCGATTCTGGAGAAGATGGACGGCACGGAGGAGCTGCTGGTGAAGCTCAAGCGCGAGGACCTCCTGAGGAAGCAGCGGACATTCGATAACGGCAGCATCCCACACCAGATTCATCTCGGGGAGCTGCACGCTATCCTGAGGAGGCAGGAGGACTTCTACCCTTTCCTCAAGGATAACCGCGAGAAGATCGAGAAGATTCTGACTTTCAGGATCCCGTACTACGTCGGCCCACTCGCTAGGGGCAACTCCCGCTTCGCTTGGATGACCCGCAAGTCAGAGGAGACGATCACGCCGTGGAACTTCGAGGAGGTGGTCGACAAGGGCGCTAGCGCTCAGTCGTTCATCGAGAGGATGACGAATTTCGACAAGAACCTGCCAAATGAGAAGGTGCTCCCTAAGCACTCGCTCCTGTACGAGTACTTCACAGTCTACAACGAGCTGACTAAGGTGAAGTATGTGACCGAGGGCATGAGGAAGCCGGCTTTCCTGTCTGGGGAGCAGAAGAAGGCCATCGTGGACCTCCTGTTCAAGACCAACCGGAAGGTCACGGTTAAGCAGCTCAAGGAGGACTACTTCAAGAAGATTGAGTGCTTCGATTCGGTCGAGATCTCTGGCGTTGAGGACCGCTTCAACGCCTCCCTGGGGACCTACCACGATCTCCTGAAGATCATTAAGGATAAGGACTTCCTGGACAACGAGGAGAATGAGGATATCCTCGAGGACATTGTGCTGACACTCACTCTGTTCGAGGACCGGGAGATGATCGAGGAGCGCCTGAAGACTTACGCCCATCTCTTCGATGACAAGGTCATGAAGCAGCTCAAGAGGAGGAGGTACACCGGCTGGGGGAGGCTGAGCAGGAAGCTCATCAACGGCATTCGGGACAAGCAGTCCGGGAAGACGATCCTCGACTTCCTGAAGAGCGATGGCTTCGCGAACCGCAATTTCATGCAGCTGATTCACGATGACAGCCTCACATTCAAGGAGGATATCCAGAAGGCTCAGGTGAGCGGCCAGGGGGACTCGCTGCACGAGCATATCGCGAACCTCGCTGGCTCGCCAGCTATCAAGAAGGGGATTCTGCAGACCGTGAAGGTTGTGGACGAGCTGGTGAAGGTCATGGGCAGGCACAAGCCTGAGAACATCGTCATTGAGATGGCCCGGGAGAATCAGACCACGCAGAAGGGCCAGAAGAACTCACGCGAGAGGATGAAGAGGATCGAGGAGGGCATTAAGGAGCTGGGGTCCCAGATCCTCAAGGAGCACCCGGTGGAGAACACGCAGCTGCAGAATGAGAAGCTCTACCTGTACTACCTCCAGAATGGCCGCGATATGTATGTGGACCAGGAGCTGGATATTAACAGGCTCAGCGATTACGACGTCGATGCCATCGTTCCACAGTCATTCCTGAAGGATGACTCCATTGACAACAAGGTCCTCACCAGGTCGGACAAGAACCGGGGCAAGTCTGATAATGTTCCTTCAGAGGAGGTCGTTAAGAAGATGAAGAACTACTGGCGCCAGCTCCTGAATGCCAAGCTGATCACGCAGCGGAAGTTCGATAACCTCACAAAGGCTGAGAGGGGCGGGCTCTCTGAGCTGGACAAGGCGGGCTTCATCAAGAGGCAGCTGGTCGAGACACGGCAGATCACTAAGCACGTTGCGCAGATTCTCGACTCACGGATGAACACTAAGTACGATGAGAATGACAAGCTGATCCGCGAGGTGAAGGTCATCACCCTGAAGTCAAAGCTCGTCTCCGACTTCAGGAAGGATTTCCAGTTCTACAAGGTTCGGGAGATCAACAATTACCACCATGCCCATGACGCGTACCTGAACGCGGTGGTCGGCACAGCTCTGATCAAGAAGTACCCAAAGCTCGAGAGCGAGTTCGTGTACGGGGACTACAAGGTTTACGATGTGAGGAAGATGATCGCCAAGTCGGAGCAGGAGATTGGCAAGGCTACCGCCAAGTACTTCTTCTACTCTAACATTATGAATTTCTTCAAGACAGAGATCACTCTGGCCAATGGCGAGATCCGGAAGCGCCCCCTCATCGAGACGAACGGCGAGACGGGGGAGATCGTGTGGGACAAGGGCAGGGATTTCGCGACCGTCAGGAAGGTTCTCTCCATGCCACAAGTGAATATCGTCAAGAAGACAGAGGTCCAGACTGGCGGGTTCTCTAAGGAGTCAATTCTGCCTAAGCGGAACAGCGACAAGCTCATCGCCCGCAAGAAGGACTGGGATCCGAAGAAGTACGGCGGGTTCGACAGCCCCACTGTGGCCTACTCGGTCCTGGTTGTGGCGAAGGTTGAGAAGGGCAAGTCCAAGAAGCTCAAGAGCGTGAAGGAGCTGCTGGGGATCACGATTATGGAGCGCTCCAGCTTCGAGAAGAACCCGATCGATTTCCTGGAGGCGAAGGGCTACAAGGAGGTGAAGAAGGACCTGATCATTAAGCTCCCCAAGTACTCACTCTTCGAGCTGGAGAACGGCAGGAAGCGGATGCTGGCTTCCGCTGGCGAGCTGCAGAAGGGGAACGAGCTGGCTCTGCCGTCCAAGTATGTGAACTTCCTCTACCTGGCCTCCCACTACGAGAAGCTCAAGGGCAGCCCCGAGGACAACGAGCAGAAGCAGCTGTTCGTCGAGCAGCACAAGCATTACCTCGACGAGATCATTGAGCAGATTTCCGAGTTCTCCAAGCGCGTGATCCTGGCCGACGCGAATCTGGATAAGGTCCTCTCCGCGTACAACAAGCACCGCGACAAGCCAATCAGGGAGCAGGCTGAGAATATCATTCATCTCTTCACCCTGACGAACCTCGGCGCCCCTGCTGCTTTCAAGTACTTCGACACAACTATCGATCGCAAGAGGTACACAAGCACTAAGGAGGTCCTGGACGCGACCCTCATCCACCAGTCGATTACCGGCCTCTACGAGACGCGCATCGACCTGTCTCAGCTCGGGGGCGACGAATTCTCCGGGAGCGAGACGCCAGGCACCTCCGAGTCGGCCACCCCAGAATCTGCCACAGTGGTGTCCGGCCAAAAGCAGGACCGCCAGGGCGGAGAACGCAGAAGGTCCCAGCTCGATAGGGATCAGTGTGCCTACTGCAAGGAGAAGGGCCACTGGGCCAAAGACTGCCCGAAAAAGCCGCGCGGCCCACGCGGCCCAAGGCCACAAACATCCCTCCTTCCAAAGAAGAAGCGGAAGGTGGAGCTCAGCGGAGGATCTTCCGGAGGATCTAGCGGCTCCGAGACACCAGGAACATCCGAAAGCGCTACACCAGAATCTAGCGGAGGCTCTTCCGGAGGATCTAGGCCTACGCCGGTGTGGATCGACCAGTGGCCGCTGCCGGAAATCAAGCTCGTCGCGCTGATCCACCTCGTGGAGCGGGAACTGCAGCTTGGGCACCTGGAACCAAGTCTCAGCTGCTGGAACACCCCTGTATTTGTTATAAGGAAAGCAAGTGGTAGCTACCGCCTGTTGCATGATCTCCGCGCCGTCAATGCAAAACTTATTCCTTTTGGTGCTGTGCAGCAGGGCGCGCCGGTTCTCTCAGCTTTGCCTCGCGGCTGGCCACTAATGGTGCTCGACCTCAAGGACTGCTTCTTCTCCATTCCCCTCGCCGGGCAAGATAGAGAAGCATTCGCCTTCACTTTGCCCTCGGTGAACAATCAAGCTCCAGCGCGGAGATTTCAATGGAAGGTTCTTCCACAAGGCATGGCGTGTTCCCCCACCATATGCCAGCTGGTGGTGGGCCGTGTCCTTGAACCTTTAAGAAGAAGGCATCCATCCCTCTGCATGCTGCACTACATGGATGATCTTCTCATCGCCGCCTCAGATTGTGAGAAGCTGGAGATGGTTGGAAAGGAAATTATTTCTGTTTTGGAGGAGGCCGGCTTCACAATATCGCCGGACAAGATCCAGAGGGAGCCCGGCGTCGAGTACCTCGGATATCGTTTAGGATCAACATATGTCATCCCGGCGGGGAGCGGCGGCAGCAAAAGAACGGCGGACGGCTCTGAGAAGCGCACCGCTGATAGCCAGCATTCAACTCCTCCGAAAACAAAGAGGAAAGTTGAGTTCGAACCGAAGAAGAAAAGGAAGGTGTGA

**Sequence 21 Plasmids sequence of ePPEplus-RT20**

(NLSSV40-nCas9(H840A/R221K/N394K)-XTEN-NC-NLS-32aa Linker-RT20-NLSvbp)

CCTAAGAAAAAGAGAAAAGTGGACAAGAAGTACTCGATCGGCCTCGATATTGGGACTAACTCTGTTGGCTGGGCCGTGATCACCGACGAGTACAAGGTGCCCTCAAAGAAGTTCAAGGTCCTGGGCAACACCGATCGGCATTCCATCAAGAAGAATCTCATTGGCGCTCTCCTGTTCGACAGCGGCGAGACGGCTGAGGCTACGCGGCTCAAGCGCACCGCCCGCAGGCGGTACACGCGCAGGAAGAATCGCATCTGCTACCTGCAGGAGATTTTCTCCAACGAGATGGCGAAGGTTGACGATTCTTTCTTCCACAGGCTGGAGGAGTCATTCCTCGTGGAGGAGGATAAGAAGCACGAGCGGCATCCAATCTTCGGCAACATTGTCGACGAGGTTGCCTACCACGAGAAGTACCCTACGATCTACCATCTGCGGAAGAAGCTCGTGGACTCCACAGATAAGGCGGACCTCCGCCTGATCTACCTCGCTCTGGCCCACATGATTAAGTTCAGGGGCCATTTCCTGATCGAGGGGGATCTCAACCCGGACAATAGCGATGTTGACAAGCTGTTCATCCAGCTCGTGCAGACGTACAACCAGCTCTTCGAGGAGAACCCCATTAATGCGTCAGGCGTCGACGCGAAGGCTATCCTGTCCGCTAGGCTCTCGAAGTCTCGGAAGCTCGAGAACCTGATCGCCCAGCTGCCGGGCGAGAAGAAGAACGGCCTGTTCGGGAATCTCATTGCGCTCAGCCTGGGGCTCACGCCCAACTTCAAGTCGAATTTCGATCTCGCTGAGGACGCCAAGCTGCAGCTCTCCAAGGACACATACGACGATGACCTGGATAACCTCCTGGCCCAGATCGGCGATCAGTACGCGGACCTGTTCCTCGCTGCCAAGAATCTGTCGGACGCCATCCTCCTGTCTGATATTCTCAGGGTGAACACCGAGATTACGAAGGCTCCGCTCTCAGCCTCCATGATCAAGCGCTACGACGAGCACCATCAGGATCTGACCCTCCTGAAGGCGCTGGTCAGGCAGCAGCTCCCCGAGAAGTACAAGGAGATCTTCTTCGATCAGTCGAAGAACGGCTACGCTGGGTACATTGACGGCGGGGCCTCTCAGGAGGAGTTCTACAAGTTCATCAAGCCGATTCTGGAGAAGATGGACGGCACGGAGGAGCTGCTGGTGAAGCTCAAGCGCGAGGACCTCCTGAGGAAGCAGCGGACATTCGATAACGGCAGCATCCCACACCAGATTCATCTCGGGGAGCTGCACGCTATCCTGAGGAGGCAGGAGGACTTCTACCCTTTCCTCAAGGATAACCGCGAGAAGATCGAGAAGATTCTGACTTTCAGGATCCCGTACTACGTCGGCCCACTCGCTAGGGGCAACTCCCGCTTCGCTTGGATGACCCGCAAGTCAGAGGAGACGATCACGCCGTGGAACTTCGAGGAGGTGGTCGACAAGGGCGCTAGCGCTCAGTCGTTCATCGAGAGGATGACGAATTTCGACAAGAACCTGCCAAATGAGAAGGTGCTCCCTAAGCACTCGCTCCTGTACGAGTACTTCACAGTCTACAACGAGCTGACTAAGGTGAAGTATGTGACCGAGGGCATGAGGAAGCCGGCTTTCCTGTCTGGGGAGCAGAAGAAGGCCATCGTGGACCTCCTGTTCAAGACCAACCGGAAGGTCACGGTTAAGCAGCTCAAGGAGGACTACTTCAAGAAGATTGAGTGCTTCGATTCGGTCGAGATCTCTGGCGTTGAGGACCGCTTCAACGCCTCCCTGGGGACCTACCACGATCTCCTGAAGATCATTAAGGATAAGGACTTCCTGGACAACGAGGAGAATGAGGATATCCTCGAGGACATTGTGCTGACACTCACTCTGTTCGAGGACCGGGAGATGATCGAGGAGCGCCTGAAGACTTACGCCCATCTCTTCGATGACAAGGTCATGAAGCAGCTCAAGAGGAGGAGGTACACCGGCTGGGGGAGGCTGAGCAGGAAGCTCATCAACGGCATTCGGGACAAGCAGTCCGGGAAGACGATCCTCGACTTCCTGAAGAGCGATGGCTTCGCGAACCGCAATTTCATGCAGCTGATTCACGATGACAGCCTCACATTCAAGGAGGATATCCAGAAGGCTCAGGTGAGCGGCCAGGGGGACTCGCTGCACGAGCATATCGCGAACCTCGCTGGCTCGCCAGCTATCAAGAAGGGGATTCTGCAGACCGTGAAGGTTGTGGACGAGCTGGTGAAGGTCATGGGCAGGCACAAGCCTGAGAACATCGTCATTGAGATGGCCCGGGAGAATCAGACCACGCAGAAGGGCCAGAAGAACTCACGCGAGAGGATGAAGAGGATCGAGGAGGGCATTAAGGAGCTGGGGTCCCAGATCCTCAAGGAGCACCCGGTGGAGAACACGCAGCTGCAGAATGAGAAGCTCTACCTGTACTACCTCCAGAATGGCCGCGATATGTATGTGGACCAGGAGCTGGATATTAACAGGCTCAGCGATTACGACGTCGATGCCATCGTTCCACAGTCATTCCTGAAGGATGACTCCATTGACAACAAGGTCCTCACCAGGTCGGACAAGAACCGGGGCAAGTCTGATAATGTTCCTTCAGAGGAGGTCGTTAAGAAGATGAAGAACTACTGGCGCCAGCTCCTGAATGCCAAGCTGATCACGCAGCGGAAGTTCGATAACCTCACAAAGGCTGAGAGGGGCGGGCTCTCTGAGCTGGACAAGGCGGGCTTCATCAAGAGGCAGCTGGTCGAGACACGGCAGATCACTAAGCACGTTGCGCAGATTCTCGACTCACGGATGAACACTAAGTACGATGAGAATGACAAGCTGATCCGCGAGGTGAAGGTCATCACCCTGAAGTCAAAGCTCGTCTCCGACTTCAGGAAGGATTTCCAGTTCTACAAGGTTCGGGAGATCAACAATTACCACCATGCCCATGACGCGTACCTGAACGCGGTGGTCGGCACAGCTCTGATCAAGAAGTACCCAAAGCTCGAGAGCGAGTTCGTGTACGGGGACTACAAGGTTTACGATGTGAGGAAGATGATCGCCAAGTCGGAGCAGGAGATTGGCAAGGCTACCGCCAAGTACTTCTTCTACTCTAACATTATGAATTTCTTCAAGACAGAGATCACTCTGGCCAATGGCGAGATCCGGAAGCGCCCCCTCATCGAGACGAACGGCGAGACGGGGGAGATCGTGTGGGACAAGGGCAGGGATTTCGCGACCGTCAGGAAGGTTCTCTCCATGCCACAAGTGAATATCGTCAAGAAGACAGAGGTCCAGACTGGCGGGTTCTCTAAGGAGTCAATTCTGCCTAAGCGGAACAGCGACAAGCTCATCGCCCGCAAGAAGGACTGGGATCCGAAGAAGTACGGCGGGTTCGACAGCCCCACTGTGGCCTACTCGGTCCTGGTTGTGGCGAAGGTTGAGAAGGGCAAGTCCAAGAAGCTCAAGAGCGTGAAGGAGCTGCTGGGGATCACGATTATGGAGCGCTCCAGCTTCGAGAAGAACCCGATCGATTTCCTGGAGGCGAAGGGCTACAAGGAGGTGAAGAAGGACCTGATCATTAAGCTCCCCAAGTACTCACTCTTCGAGCTGGAGAACGGCAGGAAGCGGATGCTGGCTTCCGCTGGCGAGCTGCAGAAGGGGAACGAGCTGGCTCTGCCGTCCAAGTATGTGAACTTCCTCTACCTGGCCTCCCACTACGAGAAGCTCAAGGGCAGCCCCGAGGACAACGAGCAGAAGCAGCTGTTCGTCGAGCAGCACAAGCATTACCTCGACGAGATCATTGAGCAGATTTCCGAGTTCTCCAAGCGCGTGATCCTGGCCGACGCGAATCTGGATAAGGTCCTCTCCGCGTACAACAAGCACCGCGACAAGCCAATCAGGGAGCAGGCTGAGAATATCATTCATCTCTTCACCCTGACGAACCTCGGCGCCCCTGCTGCTTTCAAGTACTTCGACACAACTATCGATCGCAAGAGGTACACAAGCACTAAGGAGGTCCTGGACGCGACCCTCATCCACCAGTCGATTACCGGCCTCTACGAGACGCGCATCGACCTGTCTCAGCTCGGGGGCGACGAATTCTCCGGGAGCGAGACGCCAGGCACCTCCGAGTCGGCCACCCCAGAATCTGCCACAGTGGTGTCCGGCCAAAAGCAGGACCGCCAGGGCGGAGAACGCAGAAGGTCCCAGCTCGATAGGGATCAGTGTGCCTACTGCAAGGAGAAGGGCCACTGGGCCAAAGACTGCCCGAAAAAGCCGCGCGGCCCACGCGGCCCAAGGCCACAAACATCCCTCCTTCCAAAGAAGAAGCGGAAGGTGGAGCTCAGCGGAGGATCTTCCGGAGGATCTAGCGGCTCCGAGACACCAGGAACATCCGAAAGCGCTACACCAGAATCTAGCGGAGGCTCTTCCGGAGGATCTAGGCCTATGGACAAGTTTAAGCCGTACAGCAAATCAAAAGCACCAATCAGTACATTACACAAGCTAGCGCAGACCCTATCAATAAGTATAGATGAACTGAATGAAATTGCGGCGCTTTCAGAAGATGAAAAGTACTCCAGAAAAGAACTGCCGAAGGCCGACGGCAGCAAGCGTGTCGTCTACTCTCTTCATCCAAAGATGAGATTGCTCCAATCAAGGATTAATAAACGAATATTTAAAGAGCTCGTCATCTTCCCGCCGTTCTTATTTGGTAGTGTGCCCGGGAAGAATGATGGGGTGAACAGCAATATAAAGAGAGATTATATTTCGTGTGCCAAGGCTCACTGCGGCGCTCGAACAGTATTGAAGGTTGATATCTCTAACTTCTTCGACAACATTCATAAGGATCTTGTCAGAGATGTGTTCAATGACGTGCTCAACATCCGTGGCGAGGCGCTGGAGTATGTCACTAACCTGTGCTGCAAGGGCGACTTTGTGGTCCAGGGGGCACTTACTTCAAGCTACATTGCTACTCTGTGTCTTTATGAGCAGGAGGGCTGCGTCTACCAACGCGCCCTCCGGAAAAATTTGGTTTACACCCGCTTGGTGGACGACATAACTGTTTCTTCGAAAATAAATGGATATGACTTCTCCCAGATCCTTAGTCATATTGAGAACATGCTGTCAAATTATGATCTGCCTGTTAATAAACGCAAGACAAAAGTATTCCACTGTTCTTCCGAGCCACTCAAGATCCATGGACTCCGCGTCGACTACAACTCACCTAGGCTTCCTGGTGACGAGGTGAAAAGGATCAGGGCGTCGCTCCACAACCTGAAGAAACTGGCCGTCAAGAACAACACCAAGACCTCCATGGCATATCGCAAGGAGTTCAACCGGTGCATGGGGCGGATCAACAAGCTCGGCCGCGTTGGACATGATAAGTACGACCTCTTCAAGGAACAACTCCTCGCCATCAAGCCCCTCCCCTCGCTGAGGGATATTAAGATTGTGGAGTCTGCTGTGACGAGCTTGGAAAGTTCCTGCCTCATCGGTAATGGCAACAAACACTGGTACAAGAGGAAATATAATTTGACGTTATACAAACTCATCATCCTAACACGTTCCAAGGCATTTGATGGAGTTGTCAACGCCTTCAAGGAGAGGTTAAAGAAGGTGCGTCCAGCTAGCGGCGGCAGCAAAAGAACGGCGGACGGCTCTGAGAAGCGCACCGCTGATAGCCAGCATTCAACTCCTCCGAAAACAAAGAGGAAAGTTGAGTTCGAACCGAAGAAGAAAAGGAAGGTGTGA

**Sequence 22 Plasmids sequence of ePPEplus-RT21**

(NLSSV40-nCas9(H840A/R221K/N394K)-XTEN-NC-NLS-32aa Linker-RT21-NLSvbp)

CCTAAGAAAAAGAGAAAAGTGGACAAGAAGTACTCGATCGGCCTCGATATTGGGACTAACTCTGTTGGCTGGGCCGTGATCACCGACGAGTACAAGGTGCCCTCAAAGAAGTTCAAGGTCCTGGGCAACACCGATCGGCATTCCATCAAGAAGAATCTCATTGGCGCTCTCCTGTTCGACAGCGGCGAGACGGCTGAGGCTACGCGGCTCAAGCGCACCGCCCGCAGGCGGTACACGCGCAGGAAGAATCGCATCTGCTACCTGCAGGAGATTTTCTCCAACGAGATGGCGAAGGTTGACGATTCTTTCTTCCACAGGCTGGAGGAGTCATTCCTCGTGGAGGAGGATAAGAAGCACGAGCGGCATCCAATCTTCGGCAACATTGTCGACGAGGTTGCCTACCACGAGAAGTACCCTACGATCTACCATCTGCGGAAGAAGCTCGTGGACTCCACAGATAAGGCGGACCTCCGCCTGATCTACCTCGCTCTGGCCCACATGATTAAGTTCAGGGGCCATTTCCTGATCGAGGGGGATCTCAACCCGGACAATAGCGATGTTGACAAGCTGTTCATCCAGCTCGTGCAGACGTACAACCAGCTCTTCGAGGAGAACCCCATTAATGCGTCAGGCGTCGACGCGAAGGCTATCCTGTCCGCTAGGCTCTCGAAGTCTCGGAAGCTCGAGAACCTGATCGCCCAGCTGCCGGGCGAGAAGAAGAACGGCCTGTTCGGGAATCTCATTGCGCTCAGCCTGGGGCTCACGCCCAACTTCAAGTCGAATTTCGATCTCGCTGAGGACGCCAAGCTGCAGCTCTCCAAGGACACATACGACGATGACCTGGATAACCTCCTGGCCCAGATCGGCGATCAGTACGCGGACCTGTTCCTCGCTGCCAAGAATCTGTCGGACGCCATCCTCCTGTCTGATATTCTCAGGGTGAACACCGAGATTACGAAGGCTCCGCTCTCAGCCTCCATGATCAAGCGCTACGACGAGCACCATCAGGATCTGACCCTCCTGAAGGCGCTGGTCAGGCAGCAGCTCCCCGAGAAGTACAAGGAGATCTTCTTCGATCAGTCGAAGAACGGCTACGCTGGGTACATTGACGGCGGGGCCTCTCAGGAGGAGTTCTACAAGTTCATCAAGCCGATTCTGGAGAAGATGGACGGCACGGAGGAGCTGCTGGTGAAGCTCAAGCGCGAGGACCTCCTGAGGAAGCAGCGGACATTCGATAACGGCAGCATCCCACACCAGATTCATCTCGGGGAGCTGCACGCTATCCTGAGGAGGCAGGAGGACTTCTACCCTTTCCTCAAGGATAACCGCGAGAAGATCGAGAAGATTCTGACTTTCAGGATCCCGTACTACGTCGGCCCACTCGCTAGGGGCAACTCCCGCTTCGCTTGGATGACCCGCAAGTCAGAGGAGACGATCACGCCGTGGAACTTCGAGGAGGTGGTCGACAAGGGCGCTAGCGCTCAGTCGTTCATCGAGAGGATGACGAATTTCGACAAGAACCTGCCAAATGAGAAGGTGCTCCCTAAGCACTCGCTCCTGTACGAGTACTTCACAGTCTACAACGAGCTGACTAAGGTGAAGTATGTGACCGAGGGCATGAGGAAGCCGGCTTTCCTGTCTGGGGAGCAGAAGAAGGCCATCGTGGACCTCCTGTTCAAGACCAACCGGAAGGTCACGGTTAAGCAGCTCAAGGAGGACTACTTCAAGAAGATTGAGTGCTTCGATTCGGTCGAGATCTCTGGCGTTGAGGACCGCTTCAACGCCTCCCTGGGGACCTACCACGATCTCCTGAAGATCATTAAGGATAAGGACTTCCTGGACAACGAGGAGAATGAGGATATCCTCGAGGACATTGTGCTGACACTCACTCTGTTCGAGGACCGGGAGATGATCGAGGAGCGCCTGAAGACTTACGCCCATCTCTTCGATGACAAGGTCATGAAGCAGCTCAAGAGGAGGAGGTACACCGGCTGGGGGAGGCTGAGCAGGAAGCTCATCAACGGCATTCGGGACAAGCAGTCCGGGAAGACGATCCTCGACTTCCTGAAGAGCGATGGCTTCGCGAACCGCAATTTCATGCAGCTGATTCACGATGACAGCCTCACATTCAAGGAGGATATCCAGAAGGCTCAGGTGAGCGGCCAGGGGGACTCGCTGCACGAGCATATCGCGAACCTCGCTGGCTCGCCAGCTATCAAGAAGGGGATTCTGCAGACCGTGAAGGTTGTGGACGAGCTGGTGAAGGTCATGGGCAGGCACAAGCCTGAGAACATCGTCATTGAGATGGCCCGGGAGAATCAGACCACGCAGAAGGGCCAGAAGAACTCACGCGAGAGGATGAAGAGGATCGAGGAGGGCATTAAGGAGCTGGGGTCCCAGATCCTCAAGGAGCACCCGGTGGAGAACACGCAGCTGCAGAATGAGAAGCTCTACCTGTACTACCTCCAGAATGGCCGCGATATGTATGTGGACCAGGAGCTGGATATTAACAGGCTCAGCGATTACGACGTCGATGCCATCGTTCCACAGTCATTCCTGAAGGATGACTCCATTGACAACAAGGTCCTCACCAGGTCGGACAAGAACCGGGGCAAGTCTGATAATGTTCCTTCAGAGGAGGTCGTTAAGAAGATGAAGAACTACTGGCGCCAGCTCCTGAATGCCAAGCTGATCACGCAGCGGAAGTTCGATAACCTCACAAAGGCTGAGAGGGGCGGGCTCTCTGAGCTGGACAAGGCGGGCTTCATCAAGAGGCAGCTGGTCGAGACACGGCAGATCACTAAGCACGTTGCGCAGATTCTCGACTCACGGATGAACACTAAGTACGATGAGAATGACAAGCTGATCCGCGAGGTGAAGGTCATCACCCTGAAGTCAAAGCTCGTCTCCGACTTCAGGAAGGATTTCCAGTTCTACAAGGTTCGGGAGATCAACAATTACCACCATGCCCATGACGCGTACCTGAACGCGGTGGTCGGCACAGCTCTGATCAAGAAGTACCCAAAGCTCGAGAGCGAGTTCGTGTACGGGGACTACAAGGTTTACGATGTGAGGAAGATGATCGCCAAGTCGGAGCAGGAGATTGGCAAGGCTACCGCCAAGTACTTCTTCTACTCTAACATTATGAATTTCTTCAAGACAGAGATCACTCTGGCCAATGGCGAGATCCGGAAGCGCCCCCTCATCGAGACGAACGGCGAGACGGGGGAGATCGTGTGGGACAAGGGCAGGGATTTCGCGACCGTCAGGAAGGTTCTCTCCATGCCACAAGTGAATATCGTCAAGAAGACAGAGGTCCAGACTGGCGGGTTCTCTAAGGAGTCAATTCTGCCTAAGCGGAACAGCGACAAGCTCATCGCCCGCAAGAAGGACTGGGATCCGAAGAAGTACGGCGGGTTCGACAGCCCCACTGTGGCCTACTCGGTCCTGGTTGTGGCGAAGGTTGAGAAGGGCAAGTCCAAGAAGCTCAAGAGCGTGAAGGAGCTGCTGGGGATCACGATTATGGAGCGCTCCAGCTTCGAGAAGAACCCGATCGATTTCCTGGAGGCGAAGGGCTACAAGGAGGTGAAGAAGGACCTGATCATTAAGCTCCCCAAGTACTCACTCTTCGAGCTGGAGAACGGCAGGAAGCGGATGCTGGCTTCCGCTGGCGAGCTGCAGAAGGGGAACGAGCTGGCTCTGCCGTCCAAGTATGTGAACTTCCTCTACCTGGCCTCCCACTACGAGAAGCTCAAGGGCAGCCCCGAGGACAACGAGCAGAAGCAGCTGTTCGTCGAGCAGCACAAGCATTACCTCGACGAGATCATTGAGCAGATTTCCGAGTTCTCCAAGCGCGTGATCCTGGCCGACGCGAATCTGGATAAGGTCCTCTCCGCGTACAACAAGCACCGCGACAAGCCAATCAGGGAGCAGGCTGAGAATATCATTCATCTCTTCACCCTGACGAACCTCGGCGCCCCTGCTGCTTTCAAGTACTTCGACACAACTATCGATCGCAAGAGGTACACAAGCACTAAGGAGGTCCTGGACGCGACCCTCATCCACCAGTCGATTACCGGCCTCTACGAGACGCGCATCGACCTGTCTCAGCTCGGGGGCGACGAATTCTCCGGGAGCGAGACGCCAGGCACCTCCGAGTCGGCCACCCCAGAATCTGCCACAGTGGTGTCCGGCCAAAAGCAGGACCGCCAGGGCGGAGAACGCAGAAGGTCCCAGCTCGATAGGGATCAGTGTGCCTACTGCAAGGAGAAGGGCCACTGGGCCAAAGACTGCCCGAAAAAGCCGCGCGGCCCACGCGGCCCAAGGCCACAAACATCCCTCCTTCCAAAGAAGAAGCGGAAGGTGGAGCTCAGCGGAGGATCTTCCGGAGGATCTAGCGGCTCCGAGACACCAGGAACATCCGAAAGCGCTACACCAGAATCTAGCGGAGGCTCTTCCGGAGGATCTAGGCCTCAGGAGGGAACATACCGCCCCATGCCGGTGAAGAGAGTGGGCATACCAAAACCTGGTGGCGGCATCCGCTTACTTGGAATACCTACAGTAATGGATCGGTTCATCCAGCAGGCGCTCATGCAGGTCATGACTCCAATATTTGATCCTCACTTCTCCCCAAATTCATATGGATTTAGACCCAACAAGAGGGCACATGACGCCGTCAAGCAAGCGCAAAGCTACATCAAGGAGGGGTTCCGTTGGGTGGTGGACATGGACCTGGAGAAGTTCTTCGACCGCGTCAATCATGATATTCTTATGGCGCGGGTCGTCAGGAAGGTTTCTGACAAGCGGGTACTGAAGCTCGTCCGTGCCTACCTACAAGCTGGTGTTATTGCTGATGGTATTGAGCTGGAAACCATCGAAGGGACGCCGCAGGGCGGGCCGCTGTCGCCGCTACTCGCCAACATCCTCCTTGATGATTTGGATAAAGAATTGACTGCAAGGGGGCTCCGCTTCGTGAGGTATGCCGACGACTGCAACATTTTTGTTCGAAGCCGCCGCGCCGGCGAGAGGGTGATGGAGAGTGTCATCAGATTTGCTGAAGGCAAGCTGCGCCTCAAGGTGAATCGGGACAAATCAGCGGTTGATAGGCCATGGAACAGAAAATTCCTCGGCTTCAGCGGCGGCAGCAAAAGAACGGCGGACGGCTCTGAGAAGCGCACCGCTGATAGCCAGCATTCAACTCCTCCGAAAACAAAGAGGAAAGTTGAGTTCGAACCGAAGAAGAAAAGGAAGGTGTGA

**Sequence 23 Plasmids sequence of ePPEplus-RT22**

(NLSSV40-nCas9(H840A/R221K/N394K)-XTEN-NC-NLS-32aa Linker-RT22-NLSvbp)

CCTAAGAAAAAGAGAAAAGTGGACAAGAAGTACTCGATCGGCCTCGATATTGGGACTAACTCTGTTGGCTGGGCCGTGATCACCGACGAGTACAAGGTGCCCTCAAAGAAGTTCAAGGTCCTGGGCAACACCGATCGGCATTCCATCAAGAAGAATCTCATTGGCGCTCTCCTGTTCGACAGCGGCGAGACGGCTGAGGCTACGCGGCTCAAGCGCACCGCCCGCAGGCGGTACACGCGCAGGAAGAATCGCATCTGCTACCTGCAGGAGATTTTCTCCAACGAGATGGCGAAGGTTGACGATTCTTTCTTCCACAGGCTGGAGGAGTCATTCCTCGTGGAGGAGGATAAGAAGCACGAGCGGCATCCAATCTTCGGCAACATTGTCGACGAGGTTGCCTACCACGAGAAGTACCCTACGATCTACCATCTGCGGAAGAAGCTCGTGGACTCCACAGATAAGGCGGACCTCCGCCTGATCTACCTCGCTCTGGCCCACATGATTAAGTTCAGGGGCCATTTCCTGATCGAGGGGGATCTCAACCCGGACAATAGCGATGTTGACAAGCTGTTCATCCAGCTCGTGCAGACGTACAACCAGCTCTTCGAGGAGAACCCCATTAATGCGTCAGGCGTCGACGCGAAGGCTATCCTGTCCGCTAGGCTCTCGAAGTCTCGGAAGCTCGAGAACCTGATCGCCCAGCTGCCGGGCGAGAAGAAGAACGGCCTGTTCGGGAATCTCATTGCGCTCAGCCTGGGGCTCACGCCCAACTTCAAGTCGAATTTCGATCTCGCTGAGGACGCCAAGCTGCAGCTCTCCAAGGACACATACGACGATGACCTGGATAACCTCCTGGCCCAGATCGGCGATCAGTACGCGGACCTGTTCCTCGCTGCCAAGAATCTGTCGGACGCCATCCTCCTGTCTGATATTCTCAGGGTGAACACCGAGATTACGAAGGCTCCGCTCTCAGCCTCCATGATCAAGCGCTACGACGAGCACCATCAGGATCTGACCCTCCTGAAGGCGCTGGTCAGGCAGCAGCTCCCCGAGAAGTACAAGGAGATCTTCTTCGATCAGTCGAAGAACGGCTACGCTGGGTACATTGACGGCGGGGCCTCTCAGGAGGAGTTCTACAAGTTCATCAAGCCGATTCTGGAGAAGATGGACGGCACGGAGGAGCTGCTGGTGAAGCTCAAGCGCGAGGACCTCCTGAGGAAGCAGCGGACATTCGATAACGGCAGCATCCCACACCAGATTCATCTCGGGGAGCTGCACGCTATCCTGAGGAGGCAGGAGGACTTCTACCCTTTCCTCAAGGATAACCGCGAGAAGATCGAGAAGATTCTGACTTTCAGGATCCCGTACTACGTCGGCCCACTCGCTAGGGGCAACTCCCGCTTCGCTTGGATGACCCGCAAGTCAGAGGAGACGATCACGCCGTGGAACTTCGAGGAGGTGGTCGACAAGGGCGCTAGCGCTCAGTCGTTCATCGAGAGGATGACGAATTTCGACAAGAACCTGCCAAATGAGAAGGTGCTCCCTAAGCACTCGCTCCTGTACGAGTACTTCACAGTCTACAACGAGCTGACTAAGGTGAAGTATGTGACCGAGGGCATGAGGAAGCCGGCTTTCCTGTCTGGGGAGCAGAAGAAGGCCATCGTGGACCTCCTGTTCAAGACCAACCGGAAGGTCACGGTTAAGCAGCTCAAGGAGGACTACTTCAAGAAGATTGAGTGCTTCGATTCGGTCGAGATCTCTGGCGTTGAGGACCGCTTCAACGCCTCCCTGGGGACCTACCACGATCTCCTGAAGATCATTAAGGATAAGGACTTCCTGGACAACGAGGAGAATGAGGATATCCTCGAGGACATTGTGCTGACACTCACTCTGTTCGAGGACCGGGAGATGATCGAGGAGCGCCTGAAGACTTACGCCCATCTCTTCGATGACAAGGTCATGAAGCAGCTCAAGAGGAGGAGGTACACCGGCTGGGGGAGGCTGAGCAGGAAGCTCATCAACGGCATTCGGGACAAGCAGTCCGGGAAGACGATCCTCGACTTCCTGAAGAGCGATGGCTTCGCGAACCGCAATTTCATGCAGCTGATTCACGATGACAGCCTCACATTCAAGGAGGATATCCAGAAGGCTCAGGTGAGCGGCCAGGGGGACTCGCTGCACGAGCATATCGCGAACCTCGCTGGCTCGCCAGCTATCAAGAAGGGGATTCTGCAGACCGTGAAGGTTGTGGACGAGCTGGTGAAGGTCATGGGCAGGCACAAGCCTGAGAACATCGTCATTGAGATGGCCCGGGAGAATCAGACCACGCAGAAGGGCCAGAAGAACTCACGCGAGAGGATGAAGAGGATCGAGGAGGGCATTAAGGAGCTGGGGTCCCAGATCCTCAAGGAGCACCCGGTGGAGAACACGCAGCTGCAGAATGAGAAGCTCTACCTGTACTACCTCCAGAATGGCCGCGATATGTATGTGGACCAGGAGCTGGATATTAACAGGCTCAGCGATTACGACGTCGATGCCATCGTTCCACAGTCATTCCTGAAGGATGACTCCATTGACAACAAGGTCCTCACCAGGTCGGACAAGAACCGGGGCAAGTCTGATAATGTTCCTTCAGAGGAGGTCGTTAAGAAGATGAAGAACTACTGGCGCCAGCTCCTGAATGCCAAGCTGATCACGCAGCGGAAGTTCGATAACCTCACAAAGGCTGAGAGGGGCGGGCTCTCTGAGCTGGACAAGGCGGGCTTCATCAAGAGGCAGCTGGTCGAGACACGGCAGATCACTAAGCACGTTGCGCAGATTCTCGACTCACGGATGAACACTAAGTACGATGAGAATGACAAGCTGATCCGCGAGGTGAAGGTCATCACCCTGAAGTCAAAGCTCGTCTCCGACTTCAGGAAGGATTTCCAGTTCTACAAGGTTCGGGAGATCAACAATTACCACCATGCCCATGACGCGTACCTGAACGCGGTGGTCGGCACAGCTCTGATCAAGAAGTACCCAAAGCTCGAGAGCGAGTTCGTGTACGGGGACTACAAGGTTTACGATGTGAGGAAGATGATCGCCAAGTCGGAGCAGGAGATTGGCAAGGCTACCGCCAAGTACTTCTTCTACTCTAACATTATGAATTTCTTCAAGACAGAGATCACTCTGGCCAATGGCGAGATCCGGAAGCGCCCCCTCATCGAGACGAACGGCGAGACGGGGGAGATCGTGTGGGACAAGGGCAGGGATTTCGCGACCGTCAGGAAGGTTCTCTCCATGCCACAAGTGAATATCGTCAAGAAGACAGAGGTCCAGACTGGCGGGTTCTCTAAGGAGTCAATTCTGCCTAAGCGGAACAGCGACAAGCTCATCGCCCGCAAGAAGGACTGGGATCCGAAGAAGTACGGCGGGTTCGACAGCCCCACTGTGGCCTACTCGGTCCTGGTTGTGGCGAAGGTTGAGAAGGGCAAGTCCAAGAAGCTCAAGAGCGTGAAGGAGCTGCTGGGGATCACGATTATGGAGCGCTCCAGCTTCGAGAAGAACCCGATCGATTTCCTGGAGGCGAAGGGCTACAAGGAGGTGAAGAAGGACCTGATCATTAAGCTCCCCAAGTACTCACTCTTCGAGCTGGAGAACGGCAGGAAGCGGATGCTGGCTTCCGCTGGCGAGCTGCAGAAGGGGAACGAGCTGGCTCTGCCGTCCAAGTATGTGAACTTCCTCTACCTGGCCTCCCACTACGAGAAGCTCAAGGGCAGCCCCGAGGACAACGAGCAGAAGCAGCTGTTCGTCGAGCAGCACAAGCATTACCTCGACGAGATCATTGAGCAGATTTCCGAGTTCTCCAAGCGCGTGATCCTGGCCGACGCGAATCTGGATAAGGTCCTCTCCGCGTACAACAAGCACCGCGACAAGCCAATCAGGGAGCAGGCTGAGAATATCATTCATCTCTTCACCCTGACGAACCTCGGCGCCCCTGCTGCTTTCAAGTACTTCGACACAACTATCGATCGCAAGAGGTACACAAGCACTAAGGAGGTCCTGGACGCGACCCTCATCCACCAGTCGATTACCGGCCTCTACGAGACGCGCATCGACCTGTCTCAGCTCGGGGGCGACGAATTCTCCGGGAGCGAGACGCCAGGCACCTCCGAGTCGGCCACCCCAGAATCTGCCACAGTGGTGTCCGGCCAAAAGCAGGACCGCCAGGGCGGAGAACGCAGAAGGTCCCAGCTCGATAGGGATCAGTGTGCCTACTGCAAGGAGAAGGGCCACTGGGCCAAAGACTGCCCGAAAAAGCCGCGCGGCCCACGCGGCCCAAGGCCACAAACATCCCTCCTTCCAAAGAAGAAGCGGAAGGTGGAGCTCAGCGGAGGATCTTCCGGAGGATCTAGCGGCTCCGAGACACCAGGAACATCCGAAAGCGCTACACCAGAATCTAGCGGAGGCTCTTCCGGAGGATCTAGGCCTGAAAAGCTGCGCAACAGGAAGTACAAGCCTCAGCCGGTACGGCGGGTGGAGATACCCAAGCCCGACGGCGGCATCGGCAACTTGGGCGTGCCGACTGTTACTGTCCGCTTCATCCAACAAGCTGTTGCTCAAGTGCTGATACCAATATATGAGGAGCAGTTCCATGAGCACAGCTACGGCTTCCGTCCAAATAGGTGCGCGCAGCAGGCCATTATGACTGCTTTGGACATGATCAATGAAGGAAATACATGGATCGTGGATATTGGGCTGGAGAAGTTCTTCGACACGGTGAATCATGATAAGCTCGTCACCGTCATTGGTCGGACCATCAAGGATGGTGATGTCATCTCCATCATTAGAAAATTTCTTGTTTCTGGATCCATGGTTGATGATGAGTACAAGGAGAGCGTAATTGGAACGCCGCAGGGCGGGAACCTTTCTCCTCTCCTCGCCAACATCATGCTAAATGAGCTCGACAAGGAAATGGAGAAGAGAGGTCTAAACTTCGTCAGATATGCCGACGACTGCATCATAATGGTGGGGTCGGAGATGTCAGCAAAGCGGGTGATGAGGAATCTCACCAAGTTTATTGAAGAAAAACTTGGCCTCAAGGTGAACATGAAGAAGTCAAAAGTTGACCGCCCTGGTGGGCTCAAGTATTCCGGTTTTGATATCTACTACGACAGCCACGCGCACGGGTTCAAGGCAAAACCACATACAAAAAGTGTAGAAAAATTTAAGGCGAGGATGAAACAACTGACATGTCGTTCATGGGGCGTCTCCAACAGCTATAAAATTGAGAAGTTAAGCGGCGGCAGCAAAAGAACGGCGGACGGCTCTGAGAAGCGCACCGCTGATAGCCAGCATTCAACTCCTCCGAAAACAAAGAGGAAAGTTGAGTTCGAACCGAAGAAGAAAAGGAAGGTGTGA

**Sequence 24 Plasmids sequence of ePPEplus-RT23**

(NLSSV40-nCas9(H840A/R221K/N394K)-XTEN-NC-NLS-32aa Linker-RT23-NLSvbp)

CCTAAGAAAAAGAGAAAAGTGGACAAGAAGTACTCGATCGGCCTCGATATTGGGACTAACTCTGTTGGCTGGGCCGTGATCACCGACGAGTACAAGGTGCCCTCAAAGAAGTTCAAGGTCCTGGGCAACACCGATCGGCATTCCATCAAGAAGAATCTCATTGGCGCTCTCCTGTTCGACAGCGGCGAGACGGCTGAGGCTACGCGGCTCAAGCGCACCGCCCGCAGGCGGTACACGCGCAGGAAGAATCGCATCTGCTACCTGCAGGAGATTTTCTCCAACGAGATGGCGAAGGTTGACGATTCTTTCTTCCACAGGCTGGAGGAGTCATTCCTCGTGGAGGAGGATAAGAAGCACGAGCGGCATCCAATCTTCGGCAACATTGTCGACGAGGTTGCCTACCACGAGAAGTACCCTACGATCTACCATCTGCGGAAGAAGCTCGTGGACTCCACAGATAAGGCGGACCTCCGCCTGATCTACCTCGCTCTGGCCCACATGATTAAGTTCAGGGGCCATTTCCTGATCGAGGGGGATCTCAACCCGGACAATAGCGATGTTGACAAGCTGTTCATCCAGCTCGTGCAGACGTACAACCAGCTCTTCGAGGAGAACCCCATTAATGCGTCAGGCGTCGACGCGAAGGCTATCCTGTCCGCTAGGCTCTCGAAGTCTCGGAAGCTCGAGAACCTGATCGCCCAGCTGCCGGGCGAGAAGAAGAACGGCCTGTTCGGGAATCTCATTGCGCTCAGCCTGGGGCTCACGCCCAACTTCAAGTCGAATTTCGATCTCGCTGAGGACGCCAAGCTGCAGCTCTCCAAGGACACATACGACGATGACCTGGATAACCTCCTGGCCCAGATCGGCGATCAGTACGCGGACCTGTTCCTCGCTGCCAAGAATCTGTCGGACGCCATCCTCCTGTCTGATATTCTCAGGGTGAACACCGAGATTACGAAGGCTCCGCTCTCAGCCTCCATGATCAAGCGCTACGACGAGCACCATCAGGATCTGACCCTCCTGAAGGCGCTGGTCAGGCAGCAGCTCCCCGAGAAGTACAAGGAGATCTTCTTCGATCAGTCGAAGAACGGCTACGCTGGGTACATTGACGGCGGGGCCTCTCAGGAGGAGTTCTACAAGTTCATCAAGCCGATTCTGGAGAAGATGGACGGCACGGAGGAGCTGCTGGTGAAGCTCAAGCGCGAGGACCTCCTGAGGAAGCAGCGGACATTCGATAACGGCAGCATCCCACACCAGATTCATCTCGGGGAGCTGCACGCTATCCTGAGGAGGCAGGAGGACTTCTACCCTTTCCTCAAGGATAACCGCGAGAAGATCGAGAAGATTCTGACTTTCAGGATCCCGTACTACGTCGGCCCACTCGCTAGGGGCAACTCCCGCTTCGCTTGGATGACCCGCAAGTCAGAGGAGACGATCACGCCGTGGAACTTCGAGGAGGTGGTCGACAAGGGCGCTAGCGCTCAGTCGTTCATCGAGAGGATGACGAATTTCGACAAGAACCTGCCAAATGAGAAGGTGCTCCCTAAGCACTCGCTCCTGTACGAGTACTTCACAGTCTACAACGAGCTGACTAAGGTGAAGTATGTGACCGAGGGCATGAGGAAGCCGGCTTTCCTGTCTGGGGAGCAGAAGAAGGCCATCGTGGACCTCCTGTTCAAGACCAACCGGAAGGTCACGGTTAAGCAGCTCAAGGAGGACTACTTCAAGAAGATTGAGTGCTTCGATTCGGTCGAGATCTCTGGCGTTGAGGACCGCTTCAACGCCTCCCTGGGGACCTACCACGATCTCCTGAAGATCATTAAGGATAAGGACTTCCTGGACAACGAGGAGAATGAGGATATCCTCGAGGACATTGTGCTGACACTCACTCTGTTCGAGGACCGGGAGATGATCGAGGAGCGCCTGAAGACTTACGCCCATCTCTTCGATGACAAGGTCATGAAGCAGCTCAAGAGGAGGAGGTACACCGGCTGGGGGAGGCTGAGCAGGAAGCTCATCAACGGCATTCGGGACAAGCAGTCCGGGAAGACGATCCTCGACTTCCTGAAGAGCGATGGCTTCGCGAACCGCAATTTCATGCAGCTGATTCACGATGACAGCCTCACATTCAAGGAGGATATCCAGAAGGCTCAGGTGAGCGGCCAGGGGGACTCGCTGCACGAGCATATCGCGAACCTCGCTGGCTCGCCAGCTATCAAGAAGGGGATTCTGCAGACCGTGAAGGTTGTGGACGAGCTGGTGAAGGTCATGGGCAGGCACAAGCCTGAGAACATCGTCATTGAGATGGCCCGGGAGAATCAGACCACGCAGAAGGGCCAGAAGAACTCACGCGAGAGGATGAAGAGGATCGAGGAGGGCATTAAGGAGCTGGGGTCCCAGATCCTCAAGGAGCACCCGGTGGAGAACACGCAGCTGCAGAATGAGAAGCTCTACCTGTACTACCTCCAGAATGGCCGCGATATGTATGTGGACCAGGAGCTGGATATTAACAGGCTCAGCGATTACGACGTCGATGCCATCGTTCCACAGTCATTCCTGAAGGATGACTCCATTGACAACAAGGTCCTCACCAGGTCGGACAAGAACCGGGGCAAGTCTGATAATGTTCCTTCAGAGGAGGTCGTTAAGAAGATGAAGAACTACTGGCGCCAGCTCCTGAATGCCAAGCTGATCACGCAGCGGAAGTTCGATAACCTCACAAAGGCTGAGAGGGGCGGGCTCTCTGAGCTGGACAAGGCGGGCTTCATCAAGAGGCAGCTGGTCGAGACACGGCAGATCACTAAGCACGTTGCGCAGATTCTCGACTCACGGATGAACACTAAGTACGATGAGAATGACAAGCTGATCCGCGAGGTGAAGGTCATCACCCTGAAGTCAAAGCTCGTCTCCGACTTCAGGAAGGATTTCCAGTTCTACAAGGTTCGGGAGATCAACAATTACCACCATGCCCATGACGCGTACCTGAACGCGGTGGTCGGCACAGCTCTGATCAAGAAGTACCCAAAGCTCGAGAGCGAGTTCGTGTACGGGGACTACAAGGTTTACGATGTGAGGAAGATGATCGCCAAGTCGGAGCAGGAGATTGGCAAGGCTACCGCCAAGTACTTCTTCTACTCTAACATTATGAATTTCTTCAAGACAGAGATCACTCTGGCCAATGGCGAGATCCGGAAGCGCCCCCTCATCGAGACGAACGGCGAGACGGGGGAGATCGTGTGGGACAAGGGCAGGGATTTCGCGACCGTCAGGAAGGTTCTCTCCATGCCACAAGTGAATATCGTCAAGAAGACAGAGGTCCAGACTGGCGGGTTCTCTAAGGAGTCAATTCTGCCTAAGCGGAACAGCGACAAGCTCATCGCCCGCAAGAAGGACTGGGATCCGAAGAAGTACGGCGGGTTCGACAGCCCCACTGTGGCCTACTCGGTCCTGGTTGTGGCGAAGGTTGAGAAGGGCAAGTCCAAGAAGCTCAAGAGCGTGAAGGAGCTGCTGGGGATCACGATTATGGAGCGCTCCAGCTTCGAGAAGAACCCGATCGATTTCCTGGAGGCGAAGGGCTACAAGGAGGTGAAGAAGGACCTGATCATTAAGCTCCCCAAGTACTCACTCTTCGAGCTGGAGAACGGCAGGAAGCGGATGCTGGCTTCCGCTGGCGAGCTGCAGAAGGGGAACGAGCTGGCTCTGCCGTCCAAGTATGTGAACTTCCTCTACCTGGCCTCCCACTACGAGAAGCTCAAGGGCAGCCCCGAGGACAACGAGCAGAAGCAGCTGTTCGTCGAGCAGCACAAGCATTACCTCGACGAGATCATTGAGCAGATTTCCGAGTTCTCCAAGCGCGTGATCCTGGCCGACGCGAATCTGGATAAGGTCCTCTCCGCGTACAACAAGCACCGCGACAAGCCAATCAGGGAGCAGGCTGAGAATATCATTCATCTCTTCACCCTGACGAACCTCGGCGCCCCTGCTGCTTTCAAGTACTTCGACACAACTATCGATCGCAAGAGGTACACAAGCACTAAGGAGGTCCTGGACGCGACCCTCATCCACCAGTCGATTACCGGCCTCTACGAGACGCGCATCGACCTGTCTCAGCTCGGGGGCGACGAATTCTCCGGGAGCGAGACGCCAGGCACCTCCGAGTCGGCCACCCCAGAATCTGCCACAGTGGTGTCCGGCCAAAAGCAGGACCGCCAGGGCGGAGAACGCAGAAGGTCCCAGCTCGATAGGGATCAGTGTGCCTACTGCAAGGAGAAGGGCCACTGGGCCAAAGACTGCCCGAAAAAGCCGCGCGGCCCACGCGGCCCAAGGCCACAAACATCCCTCCTTCCAAAGAAGAAGCGGAAGGTGGAGCTCAGCGGAGGATCTTCCGGAGGATCTAGCGGCTCCGAGACACCAGGAACATCCGAAAGCGCTACACCAGAATCTAGCGGAGGCTCTTCCGGAGGATCTAGGCCTGAGAGACTGGCAAGGGACTTGGACATTTCAGAGCAAGATGTGAGGGGATTTGCCCTCACCGCCCCCAACAGATACAAGATTTACCGTATCCCAAAAAGCGGTAGCAGAGTAATCGCACATCCGAGTAGAATGCTCAAGGCTTACCAGCGCCTCATCATCAGCTACGTCACGGAGTTACTGCCCGTTCACTCCTGCGCCTACGCATACAGGCAGGGCGTGGGCATCAAAGACAATGCTAGGGTGCATGCCAAGTCATCTTACCTCCTGAAGATGGATTTACAGAATTTCTTCAACTCTATAAACCCTTCCCTATTCTTTGACGTGGTTGAAAAGGTTGGGCTGGAGGTGGCTGACCGCGACAAGTGGCTCTTGACAAAGCTCCTGTTCTGGAGCCCTAATAAAAGCTCTAGTGGCCGGCTGATCTTGAGTGTAGGTGCGCCGTCCTCCCCTCTTGTGAGCAACTTTATCATGTATCCTTTTGACATCAGGATGTCAGAGTACTGCTTCGCCAATGGCCTCAAATACACAAGATATGCCGACGACATCACCCTGTCAACCAACAAGCGCGGCTGTCTCTTCGATTTGCCTGATTATATCAAAGGGGTGCTCGCCAACGAATATCAAGGGAAGCTGTCGGTGAATGAAAGCAAAACAGTCTTCTCCAGCAAGGCGCACAACCGTCACGTCACCGGCATCACTATTACTAACAAAGGATCACTGAGCATAGGCCGGCAGCGCAAGAGATACATTAGCTCCCTGATATACCAGTTTTCTTCTGATCAGTTAGACCCAGATGATACCCTCCACCTGAAGGGCCTGCTGTCCTTCGCGTTCGACGTGGAGCCCGACCTGCGCCGGAGATTTGAGAAGAAGTACACCTCGAAAGTGATCCACCAAATCCTTAGCGGCGGCAGCAAAAGAACGGCGGACGGCTCTGAGAAGCGCACCGCTGATAGCCAGCATTCAACTCCTCCGAAAACAAAGAGGAAAGTTGAGTTCGAACCGAAGAAGAAAAGGAAGGTGTGA

**Sequence 25 Plasmids sequence of ePPEplus-RT24**

(NLSSV40-nCas9(H840A/R221K/N394K)-XTEN-NC-NLS-32aa Linker-RT24-NLSvbp)

CCTAAGAAAAAGAGAAAAGTGGACAAGAAGTACTCGATCGGCCTCGATATTGGGACTAACTCTGTTGGCTGGGCCGTGATCACCGACGAGTACAAGGTGCCCTCAAAGAAGTTCAAGGTCCTGGGCAACACCGATCGGCATTCCATCAAGAAGAATCTCATTGGCGCTCTCCTGTTCGACAGCGGCGAGACGGCTGAGGCTACGCGGCTCAAGCGCACCGCCCGCAGGCGGTACACGCGCAGGAAGAATCGCATCTGCTACCTGCAGGAGATTTTCTCCAACGAGATGGCGAAGGTTGACGATTCTTTCTTCCACAGGCTGGAGGAGTCATTCCTCGTGGAGGAGGATAAGAAGCACGAGCGGCATCCAATCTTCGGCAACATTGTCGACGAGGTTGCCTACCACGAGAAGTACCCTACGATCTACCATCTGCGGAAGAAGCTCGTGGACTCCACAGATAAGGCGGACCTCCGCCTGATCTACCTCGCTCTGGCCCACATGATTAAGTTCAGGGGCCATTTCCTGATCGAGGGGGATCTCAACCCGGACAATAGCGATGTTGACAAGCTGTTCATCCAGCTCGTGCAGACGTACAACCAGCTCTTCGAGGAGAACCCCATTAATGCGTCAGGCGTCGACGCGAAGGCTATCCTGTCCGCTAGGCTCTCGAAGTCTCGGAAGCTCGAGAACCTGATCGCCCAGCTGCCGGGCGAGAAGAAGAACGGCCTGTTCGGGAATCTCATTGCGCTCAGCCTGGGGCTCACGCCCAACTTCAAGTCGAATTTCGATCTCGCTGAGGACGCCAAGCTGCAGCTCTCCAAGGACACATACGACGATGACCTGGATAACCTCCTGGCCCAGATCGGCGATCAGTACGCGGACCTGTTCCTCGCTGCCAAGAATCTGTCGGACGCCATCCTCCTGTCTGATATTCTCAGGGTGAACACCGAGATTACGAAGGCTCCGCTCTCAGCCTCCATGATCAAGCGCTACGACGAGCACCATCAGGATCTGACCCTCCTGAAGGCGCTGGTCAGGCAGCAGCTCCCCGAGAAGTACAAGGAGATCTTCTTCGATCAGTCGAAGAACGGCTACGCTGGGTACATTGACGGCGGGGCCTCTCAGGAGGAGTTCTACAAGTTCATCAAGCCGATTCTGGAGAAGATGGACGGCACGGAGGAGCTGCTGGTGAAGCTCAAGCGCGAGGACCTCCTGAGGAAGCAGCGGACATTCGATAACGGCAGCATCCCACACCAGATTCATCTCGGGGAGCTGCACGCTATCCTGAGGAGGCAGGAGGACTTCTACCCTTTCCTCAAGGATAACCGCGAGAAGATCGAGAAGATTCTGACTTTCAGGATCCCGTACTACGTCGGCCCACTCGCTAGGGGCAACTCCCGCTTCGCTTGGATGACCCGCAAGTCAGAGGAGACGATCACGCCGTGGAACTTCGAGGAGGTGGTCGACAAGGGCGCTAGCGCTCAGTCGTTCATCGAGAGGATGACGAATTTCGACAAGAACCTGCCAAATGAGAAGGTGCTCCCTAAGCACTCGCTCCTGTACGAGTACTTCACAGTCTACAACGAGCTGACTAAGGTGAAGTATGTGACCGAGGGCATGAGGAAGCCGGCTTTCCTGTCTGGGGAGCAGAAGAAGGCCATCGTGGACCTCCTGTTCAAGACCAACCGGAAGGTCACGGTTAAGCAGCTCAAGGAGGACTACTTCAAGAAGATTGAGTGCTTCGATTCGGTCGAGATCTCTGGCGTTGAGGACCGCTTCAACGCCTCCCTGGGGACCTACCACGATCTCCTGAAGATCATTAAGGATAAGGACTTCCTGGACAACGAGGAGAATGAGGATATCCTCGAGGACATTGTGCTGACACTCACTCTGTTCGAGGACCGGGAGATGATCGAGGAGCGCCTGAAGACTTACGCCCATCTCTTCGATGACAAGGTCATGAAGCAGCTCAAGAGGAGGAGGTACACCGGCTGGGGGAGGCTGAGCAGGAAGCTCATCAACGGCATTCGGGACAAGCAGTCCGGGAAGACGATCCTCGACTTCCTGAAGAGCGATGGCTTCGCGAACCGCAATTTCATGCAGCTGATTCACGATGACAGCCTCACATTCAAGGAGGATATCCAGAAGGCTCAGGTGAGCGGCCAGGGGGACTCGCTGCACGAGCATATCGCGAACCTCGCTGGCTCGCCAGCTATCAAGAAGGGGATTCTGCAGACCGTGAAGGTTGTGGACGAGCTGGTGAAGGTCATGGGCAGGCACAAGCCTGAGAACATCGTCATTGAGATGGCCCGGGAGAATCAGACCACGCAGAAGGGCCAGAAGAACTCACGCGAGAGGATGAAGAGGATCGAGGAGGGCATTAAGGAGCTGGGGTCCCAGATCCTCAAGGAGCACCCGGTGGAGAACACGCAGCTGCAGAATGAGAAGCTCTACCTGTACTACCTCCAGAATGGCCGCGATATGTATGTGGACCAGGAGCTGGATATTAACAGGCTCAGCGATTACGACGTCGATGCCATCGTTCCACAGTCATTCCTGAAGGATGACTCCATTGACAACAAGGTCCTCACCAGGTCGGACAAGAACCGGGGCAAGTCTGATAATGTTCCTTCAGAGGAGGTCGTTAAGAAGATGAAGAACTACTGGCGCCAGCTCCTGAATGCCAAGCTGATCACGCAGCGGAAGTTCGATAACCTCACAAAGGCTGAGAGGGGCGGGCTCTCTGAGCTGGACAAGGCGGGCTTCATCAAGAGGCAGCTGGTCGAGACACGGCAGATCACTAAGCACGTTGCGCAGATTCTCGACTCACGGATGAACACTAAGTACGATGAGAATGACAAGCTGATCCGCGAGGTGAAGGTCATCACCCTGAAGTCAAAGCTCGTCTCCGACTTCAGGAAGGATTTCCAGTTCTACAAGGTTCGGGAGATCAACAATTACCACCATGCCCATGACGCGTACCTGAACGCGGTGGTCGGCACAGCTCTGATCAAGAAGTACCCAAAGCTCGAGAGCGAGTTCGTGTACGGGGACTACAAGGTTTACGATGTGAGGAAGATGATCGCCAAGTCGGAGCAGGAGATTGGCAAGGCTACCGCCAAGTACTTCTTCTACTCTAACATTATGAATTTCTTCAAGACAGAGATCACTCTGGCCAATGGCGAGATCCGGAAGCGCCCCCTCATCGAGACGAACGGCGAGACGGGGGAGATCGTGTGGGACAAGGGCAGGGATTTCGCGACCGTCAGGAAGGTTCTCTCCATGCCACAAGTGAATATCGTCAAGAAGACAGAGGTCCAGACTGGCGGGTTCTCTAAGGAGTCAATTCTGCCTAAGCGGAACAGCGACAAGCTCATCGCCCGCAAGAAGGACTGGGATCCGAAGAAGTACGGCGGGTTCGACAGCCCCACTGTGGCCTACTCGGTCCTGGTTGTGGCGAAGGTTGAGAAGGGCAAGTCCAAGAAGCTCAAGAGCGTGAAGGAGCTGCTGGGGATCACGATTATGGAGCGCTCCAGCTTCGAGAAGAACCCGATCGATTTCCTGGAGGCGAAGGGCTACAAGGAGGTGAAGAAGGACCTGATCATTAAGCTCCCCAAGTACTCACTCTTCGAGCTGGAGAACGGCAGGAAGCGGATGCTGGCTTCCGCTGGCGAGCTGCAGAAGGGGAACGAGCTGGCTCTGCCGTCCAAGTATGTGAACTTCCTCTACCTGGCCTCCCACTACGAGAAGCTCAAGGGCAGCCCCGAGGACAACGAGCAGAAGCAGCTGTTCGTCGAGCAGCACAAGCATTACCTCGACGAGATCATTGAGCAGATTTCCGAGTTCTCCAAGCGCGTGATCCTGGCCGACGCGAATCTGGATAAGGTCCTCTCCGCGTACAACAAGCACCGCGACAAGCCAATCAGGGAGCAGGCTGAGAATATCATTCATCTCTTCACCCTGACGAACCTCGGCGCCCCTGCTGCTTTCAAGTACTTCGACACAACTATCGATCGCAAGAGGTACACAAGCACTAAGGAGGTCCTGGACGCGACCCTCATCCACCAGTCGATTACCGGCCTCTACGAGACGCGCATCGACCTGTCTCAGCTCGGGGGCGACGAATTCTCCGGGAGCGAGACGCCAGGCACCTCCGAGTCGGCCACCCCAGAATCTGCCACAGTGGTGTCCGGCCAAAAGCAGGACCGCCAGGGCGGAGAACGCAGAAGGTCCCAGCTCGATAGGGATCAGTGTGCCTACTGCAAGGAGAAGGGCCACTGGGCCAAAGACTGCCCGAAAAAGCCGCGCGGCCCACGCGGCCCAAGGCCACAAACATCCCTCCTTCCAAAGAAGAAGCGGAAGGTGGAGCTCAGCGGAGGATCTTCCGGAGGATCTAGCGGCTCCGAGACACCAGGAACATCCGAAAGCGCTACACCAGAATCTAGCGGAGGCTCTTCCGGAGGATCTAGGCCTCTGGAGGCGACCCCCTCCAAGCATTACAAAGTCTACAAGATTCCTAAGAAAAGACTCGGCTTCATCGCCCAGCCTACACCAGCCGTAAAGATTATTCAAAAGGAGATCGTCGACTACCTCACCCCCAAGGTGAGCATTCACACCAGCGCCACCGCCTACGTGCCTGGAAGATCTGTGAAGGATAATGCATTGGTGCATGTAAAATCGAACTTCCTTCTGAAGGTCGATCTTGAAAATTTCTTCAACAATATAACACCAAAAATGCTCTTCAAGAGCCTGAAGAAACAGGGTATCGTGGTGAGCGGGGTGGACCTCATAGTGCTGCAGCAGTTCCTCTTCTGGAACATCTCAAAGAAGCGGTCTGGGAAGCTCGCCCTCAGTGTCGGCGCACCTTCTTCGCCGTTCGTGAGCAATGTTGTCATGCTGAGCTTTGATGAAAGGATGACAAAGTTGTGCCGCAAGTCGGGTATCAACTACAGCAGATATGCTGACGACCTGACATTCTCCACCACCCAAAAGGGACTGCTGTTTGAGCACCTGTTTGTTGTGAGAAAAGCTCTGAAGAAGGAATTTGGCGCCCGACTGGTGCTGAATGAGTCAAAAACCGTGTTCTCTAGCAAGGCGCACAACCGCCACGTCACCGGCATTACCTTGACAAATAACAACAAAATCTCCCTGGGCCGGGAGAGGAAGAGGTACATCTCCGCGCTGGTCCATAAGTTTAAGCTGGGGCTCCTAGACGTGGAGGATACTCTTCACTTGCAGGGACTGATCAGCTACGCCAACCACATTGAGGCCAGCTTCGTCCAGAAGATGTCAATCAAATATGGTGACAACGTTCTGACAGAAATCATCAAGTACAGCGGCAGCGGCGGCAGCAAAAGAACGGCGGACGGCTCTGAGAAGCGCACCGCTGATAGCCAGCATTCAACTCCTCCGAAAACAAAGAGGAAAGTTGAGTTCGAACCGAAGAAGAAAAGGAAGGTGTGA

**Sequence 26 Plasmids sequence of ePPEplus-RT25**

(NLSSV40-nCas9(H840A/R221K/N394K)-XTEN-NC-NLS-32aa Linker-RT25-NLSvbp)

CCTAAGAAAAAGAGAAAAGTGGACAAGAAGTACTCGATCGGCCTCGATATTGGGACTAACTCTGTTGGCTGGGCCGTGATCACCGACGAGTACAAGGTGCCCTCAAAGAAGTTCAAGGTCCTGGGCAACACCGATCGGCATTCCATCAAGAAGAATCTCATTGGCGCTCTCCTGTTCGACAGCGGCGAGACGGCTGAGGCTACGCGGCTCAAGCGCACCGCCCGCAGGCGGTACACGCGCAGGAAGAATCGCATCTGCTACCTGCAGGAGATTTTCTCCAACGAGATGGCGAAGGTTGACGATTCTTTCTTCCACAGGCTGGAGGAGTCATTCCTCGTGGAGGAGGATAAGAAGCACGAGCGGCATCCAATCTTCGGCAACATTGTCGACGAGGTTGCCTACCACGAGAAGTACCCTACGATCTACCATCTGCGGAAGAAGCTCGTGGACTCCACAGATAAGGCGGACCTCCGCCTGATCTACCTCGCTCTGGCCCACATGATTAAGTTCAGGGGCCATTTCCTGATCGAGGGGGATCTCAACCCGGACAATAGCGATGTTGACAAGCTGTTCATCCAGCTCGTGCAGACGTACAACCAGCTCTTCGAGGAGAACCCCATTAATGCGTCAGGCGTCGACGCGAAGGCTATCCTGTCCGCTAGGCTCTCGAAGTCTCGGAAGCTCGAGAACCTGATCGCCCAGCTGCCGGGCGAGAAGAAGAACGGCCTGTTCGGGAATCTCATTGCGCTCAGCCTGGGGCTCACGCCCAACTTCAAGTCGAATTTCGATCTCGCTGAGGACGCCAAGCTGCAGCTCTCCAAGGACACATACGACGATGACCTGGATAACCTCCTGGCCCAGATCGGCGATCAGTACGCGGACCTGTTCCTCGCTGCCAAGAATCTGTCGGACGCCATCCTCCTGTCTGATATTCTCAGGGTGAACACCGAGATTACGAAGGCTCCGCTCTCAGCCTCCATGATCAAGCGCTACGACGAGCACCATCAGGATCTGACCCTCCTGAAGGCGCTGGTCAGGCAGCAGCTCCCCGAGAAGTACAAGGAGATCTTCTTCGATCAGTCGAAGAACGGCTACGCTGGGTACATTGACGGCGGGGCCTCTCAGGAGGAGTTCTACAAGTTCATCAAGCCGATTCTGGAGAAGATGGACGGCACGGAGGAGCTGCTGGTGAAGCTCAAGCGCGAGGACCTCCTGAGGAAGCAGCGGACATTCGATAACGGCAGCATCCCACACCAGATTCATCTCGGGGAGCTGCACGCTATCCTGAGGAGGCAGGAGGACTTCTACCCTTTCCTCAAGGATAACCGCGAGAAGATCGAGAAGATTCTGACTTTCAGGATCCCGTACTACGTCGGCCCACTCGCTAGGGGCAACTCCCGCTTCGCTTGGATGACCCGCAAGTCAGAGGAGACGATCACGCCGTGGAACTTCGAGGAGGTGGTCGACAAGGGCGCTAGCGCTCAGTCGTTCATCGAGAGGATGACGAATTTCGACAAGAACCTGCCAAATGAGAAGGTGCTCCCTAAGCACTCGCTCCTGTACGAGTACTTCACAGTCTACAACGAGCTGACTAAGGTGAAGTATGTGACCGAGGGCATGAGGAAGCCGGCTTTCCTGTCTGGGGAGCAGAAGAAGGCCATCGTGGACCTCCTGTTCAAGACCAACCGGAAGGTCACGGTTAAGCAGCTCAAGGAGGACTACTTCAAGAAGATTGAGTGCTTCGATTCGGTCGAGATCTCTGGCGTTGAGGACCGCTTCAACGCCTCCCTGGGGACCTACCACGATCTCCTGAAGATCATTAAGGATAAGGACTTCCTGGACAACGAGGAGAATGAGGATATCCTCGAGGACATTGTGCTGACACTCACTCTGTTCGAGGACCGGGAGATGATCGAGGAGCGCCTGAAGACTTACGCCCATCTCTTCGATGACAAGGTCATGAAGCAGCTCAAGAGGAGGAGGTACACCGGCTGGGGGAGGCTGAGCAGGAAGCTCATCAACGGCATTCGGGACAAGCAGTCCGGGAAGACGATCCTCGACTTCCTGAAGAGCGATGGCTTCGCGAACCGCAATTTCATGCAGCTGATTCACGATGACAGCCTCACATTCAAGGAGGATATCCAGAAGGCTCAGGTGAGCGGCCAGGGGGACTCGCTGCACGAGCATATCGCGAACCTCGCTGGCTCGCCAGCTATCAAGAAGGGGATTCTGCAGACCGTGAAGGTTGTGGACGAGCTGGTGAAGGTCATGGGCAGGCACAAGCCTGAGAACATCGTCATTGAGATGGCCCGGGAGAATCAGACCACGCAGAAGGGCCAGAAGAACTCACGCGAGAGGATGAAGAGGATCGAGGAGGGCATTAAGGAGCTGGGGTCCCAGATCCTCAAGGAGCACCCGGTGGAGAACACGCAGCTGCAGAATGAGAAGCTCTACCTGTACTACCTCCAGAATGGCCGCGATATGTATGTGGACCAGGAGCTGGATATTAACAGGCTCAGCGATTACGACGTCGATGCCATCGTTCCACAGTCATTCCTGAAGGATGACTCCATTGACAACAAGGTCCTCACCAGGTCGGACAAGAACCGGGGCAAGTCTGATAATGTTCCTTCAGAGGAGGTCGTTAAGAAGATGAAGAACTACTGGCGCCAGCTCCTGAATGCCAAGCTGATCACGCAGCGGAAGTTCGATAACCTCACAAAGGCTGAGAGGGGCGGGCTCTCTGAGCTGGACAAGGCGGGCTTCATCAAGAGGCAGCTGGTCGAGACACGGCAGATCACTAAGCACGTTGCGCAGATTCTCGACTCACGGATGAACACTAAGTACGATGAGAATGACAAGCTGATCCGCGAGGTGAAGGTCATCACCCTGAAGTCAAAGCTCGTCTCCGACTTCAGGAAGGATTTCCAGTTCTACAAGGTTCGGGAGATCAACAATTACCACCATGCCCATGACGCGTACCTGAACGCGGTGGTCGGCACAGCTCTGATCAAGAAGTACCCAAAGCTCGAGAGCGAGTTCGTGTACGGGGACTACAAGGTTTACGATGTGAGGAAGATGATCGCCAAGTCGGAGCAGGAGATTGGCAAGGCTACCGCCAAGTACTTCTTCTACTCTAACATTATGAATTTCTTCAAGACAGAGATCACTCTGGCCAATGGCGAGATCCGGAAGCGCCCCCTCATCGAGACGAACGGCGAGACGGGGGAGATCGTGTGGGACAAGGGCAGGGATTTCGCGACCGTCAGGAAGGTTCTCTCCATGCCACAAGTGAATATCGTCAAGAAGACAGAGGTCCAGACTGGCGGGTTCTCTAAGGAGTCAATTCTGCCTAAGCGGAACAGCGACAAGCTCATCGCCCGCAAGAAGGACTGGGATCCGAAGAAGTACGGCGGGTTCGACAGCCCCACTGTGGCCTACTCGGTCCTGGTTGTGGCGAAGGTTGAGAAGGGCAAGTCCAAGAAGCTCAAGAGCGTGAAGGAGCTGCTGGGGATCACGATTATGGAGCGCTCCAGCTTCGAGAAGAACCCGATCGATTTCCTGGAGGCGAAGGGCTACAAGGAGGTGAAGAAGGACCTGATCATTAAGCTCCCCAAGTACTCACTCTTCGAGCTGGAGAACGGCAGGAAGCGGATGCTGGCTTCCGCTGGCGAGCTGCAGAAGGGGAACGAGCTGGCTCTGCCGTCCAAGTATGTGAACTTCCTCTACCTGGCCTCCCACTACGAGAAGCTCAAGGGCAGCCCCGAGGACAACGAGCAGAAGCAGCTGTTCGTCGAGCAGCACAAGCATTACCTCGACGAGATCATTGAGCAGATTTCCGAGTTCTCCAAGCGCGTGATCCTGGCCGACGCGAATCTGGATAAGGTCCTCTCCGCGTACAACAAGCACCGCGACAAGCCAATCAGGGAGCAGGCTGAGAATATCATTCATCTCTTCACCCTGACGAACCTCGGCGCCCCTGCTGCTTTCAAGTACTTCGACACAACTATCGATCGCAAGAGGTACACAAGCACTAAGGAGGTCCTGGACGCGACCCTCATCCACCAGTCGATTACCGGCCTCTACGAGACGCGCATCGACCTGTCTCAGCTCGGGGGCGACGAATTCTCCGGGAGCGAGACGCCAGGCACCTCCGAGTCGGCCACCCCAGAATCTGCCACAGTGGTGTCCGGCCAAAAGCAGGACCGCCAGGGCGGAGAACGCAGAAGGTCCCAGCTCGATAGGGATCAGTGTGCCTACTGCAAGGAGAAGGGCCACTGGGCCAAAGACTGCCCGAAAAAGCCGCGCGGCCCACGCGGCCCAAGGCCACAAACATCCCTCCTTCCAAAGAAGAAGCGGAAGGTGGAGCTCAGCGGAGGATCTTCCGGAGGATCTAGCGGCTCCGAGACACCAGGAACATCCGAAAGCGCTACACCAGAATCTAGCGGAGGCTCTTCCGGAGGATCTAGGCCTGGCCTAATCAAACAACTCGCCCAGAACCTCTGCAAGAGCGAAGCTGAAGTCGCCCATTTCCTCCTCAACGCCCCGAAGATGTACAAGGTTTACACCATTCCAAAGTCCGGCCACATCGCGCAGCCGAGCAAGGAACTAAAGGAGTACCAGCGGAAGTATCTGGAGCTCCAGCAGCTACCTATCCATGAGGCCGCCATGGCGTACAGGGAGGGGATAGGGATAAAGCAAAATGCTATCGCTCACAAAAATAACCCTTACCTGCTGAAGCTCGACCTCGAGAACTTCTTTAACAGTATCTCCAACTTGCTGTTTTGGGAGGTGTGGAAGAGCATACAACCTCTCCCTTCTTCCAAGGATCAGAAGGCGCTGGAGCAGCTGCTGTTCTGGTGCCCTAGCAAAATTACTGGCGGCCGTCTGGTACTCAGCATTGGCGCCCCCTCTTCACCCCTTGTGAGCAATTTTTTCATGTATCAGTTTGATTGCACCATCTCTGAAATATGTCGGCACAAGAATATCGTGTACACAAGATATGCCGATGACCTCACCTTCTCTACTAAGATCAAGGGAATCCTCTTCGACATGCCAAACATCGTGAAAGACCACCTGTCGTTGCACTTCGGCGACGCCATTAACATTAATAAGAAGAAAACAAAGTTCTCCTCCAAAGCTCACAACCGCCACGTCACCGGTATCACGCTGAACAATGATGGAAAGCTTTCCCTGGGCCGGGAGAGAAAAAGATACATCAAGCACTTGGTGCACCAGGTGAAGCTGGGCTGTCTTGAAAATGAAGACAAACAGCATCTGGTAGGCTTACTGGCTTTCGTGAAGCATATCGAGCCGATGTTCCTGGAGAGCTTGAAGAATAAATACTCTGTGGAGTTGATTACCAAGATCATCGAGGGAAGCGGCGGCAGCAAAAGAACGGCGGACGGCTCTGAGAAGCGCACCGCTGATAGCCAGCATTCAACTCCTCCGAAAACAAAGAGGAAAGTTGAGTTCGAACCGAAGAAGAAAAGGAAGGTGTGA

**Sequence 27 Plasmids sequence of ePPEplus-RT26**

(NLSSV40-nCas9(H840A/R221K/N394K)-XTEN-NC-NLS-32aa Linker-RT26-NLSvbp)

CCTAAGAAAAAGAGAAAAGTGGACAAGAAGTACTCGATCGGCCTCGATATTGGGACTAACTCTGTTGGCTGGGCCGTGATCACCGACGAGTACAAGGTGCCCTCAAAGAAGTTCAAGGTCCTGGGCAACACCGATCGGCATTCCATCAAGAAGAATCTCATTGGCGCTCTCCTGTTCGACAGCGGCGAGACGGCTGAGGCTACGCGGCTCAAGCGCACCGCCCGCAGGCGGTACACGCGCAGGAAGAATCGCATCTGCTACCTGCAGGAGATTTTCTCCAACGAGATGGCGAAGGTTGACGATTCTTTCTTCCACAGGCTGGAGGAGTCATTCCTCGTGGAGGAGGATAAGAAGCACGAGCGGCATCCAATCTTCGGCAACATTGTCGACGAGGTTGCCTACCACGAGAAGTACCCTACGATCTACCATCTGCGGAAGAAGCTCGTGGACTCCACAGATAAGGCGGACCTCCGCCTGATCTACCTCGCTCTGGCCCACATGATTAAGTTCAGGGGCCATTTCCTGATCGAGGGGGATCTCAACCCGGACAATAGCGATGTTGACAAGCTGTTCATCCAGCTCGTGCAGACGTACAACCAGCTCTTCGAGGAGAACCCCATTAATGCGTCAGGCGTCGACGCGAAGGCTATCCTGTCCGCTAGGCTCTCGAAGTCTCGGAAGCTCGAGAACCTGATCGCCCAGCTGCCGGGCGAGAAGAAGAACGGCCTGTTCGGGAATCTCATTGCGCTCAGCCTGGGGCTCACGCCCAACTTCAAGTCGAATTTCGATCTCGCTGAGGACGCCAAGCTGCAGCTCTCCAAGGACACATACGACGATGACCTGGATAACCTCCTGGCCCAGATCGGCGATCAGTACGCGGACCTGTTCCTCGCTGCCAAGAATCTGTCGGACGCCATCCTCCTGTCTGATATTCTCAGGGTGAACACCGAGATTACGAAGGCTCCGCTCTCAGCCTCCATGATCAAGCGCTACGACGAGCACCATCAGGATCTGACCCTCCTGAAGGCGCTGGTCAGGCAGCAGCTCCCCGAGAAGTACAAGGAGATCTTCTTCGATCAGTCGAAGAACGGCTACGCTGGGTACATTGACGGCGGGGCCTCTCAGGAGGAGTTCTACAAGTTCATCAAGCCGATTCTGGAGAAGATGGACGGCACGGAGGAGCTGCTGGTGAAGCTCAAGCGCGAGGACCTCCTGAGGAAGCAGCGGACATTCGATAACGGCAGCATCCCACACCAGATTCATCTCGGGGAGCTGCACGCTATCCTGAGGAGGCAGGAGGACTTCTACCCTTTCCTCAAGGATAACCGCGAGAAGATCGAGAAGATTCTGACTTTCAGGATCCCGTACTACGTCGGCCCACTCGCTAGGGGCAACTCCCGCTTCGCTTGGATGACCCGCAAGTCAGAGGAGACGATCACGCCGTGGAACTTCGAGGAGGTGGTCGACAAGGGCGCTAGCGCTCAGTCGTTCATCGAGAGGATGACGAATTTCGACAAGAACCTGCCAAATGAGAAGGTGCTCCCTAAGCACTCGCTCCTGTACGAGTACTTCACAGTCTACAACGAGCTGACTAAGGTGAAGTATGTGACCGAGGGCATGAGGAAGCCGGCTTTCCTGTCTGGGGAGCAGAAGAAGGCCATCGTGGACCTCCTGTTCAAGACCAACCGGAAGGTCACGGTTAAGCAGCTCAAGGAGGACTACTTCAAGAAGATTGAGTGCTTCGATTCGGTCGAGATCTCTGGCGTTGAGGACCGCTTCAACGCCTCCCTGGGGACCTACCACGATCTCCTGAAGATCATTAAGGATAAGGACTTCCTGGACAACGAGGAGAATGAGGATATCCTCGAGGACATTGTGCTGACACTCACTCTGTTCGAGGACCGGGAGATGATCGAGGAGCGCCTGAAGACTTACGCCCATCTCTTCGATGACAAGGTCATGAAGCAGCTCAAGAGGAGGAGGTACACCGGCTGGGGGAGGCTGAGCAGGAAGCTCATCAACGGCATTCGGGACAAGCAGTCCGGGAAGACGATCCTCGACTTCCTGAAGAGCGATGGCTTCGCGAACCGCAATTTCATGCAGCTGATTCACGATGACAGCCTCACATTCAAGGAGGATATCCAGAAGGCTCAGGTGAGCGGCCAGGGGGACTCGCTGCACGAGCATATCGCGAACCTCGCTGGCTCGCCAGCTATCAAGAAGGGGATTCTGCAGACCGTGAAGGTTGTGGACGAGCTGGTGAAGGTCATGGGCAGGCACAAGCCTGAGAACATCGTCATTGAGATGGCCCGGGAGAATCAGACCACGCAGAAGGGCCAGAAGAACTCACGCGAGAGGATGAAGAGGATCGAGGAGGGCATTAAGGAGCTGGGGTCCCAGATCCTCAAGGAGCACCCGGTGGAGAACACGCAGCTGCAGAATGAGAAGCTCTACCTGTACTACCTCCAGAATGGCCGCGATATGTATGTGGACCAGGAGCTGGATATTAACAGGCTCAGCGATTACGACGTCGATGCCATCGTTCCACAGTCATTCCTGAAGGATGACTCCATTGACAACAAGGTCCTCACCAGGTCGGACAAGAACCGGGGCAAGTCTGATAATGTTCCTTCAGAGGAGGTCGTTAAGAAGATGAAGAACTACTGGCGCCAGCTCCTGAATGCCAAGCTGATCACGCAGCGGAAGTTCGATAACCTCACAAAGGCTGAGAGGGGCGGGCTCTCTGAGCTGGACAAGGCGGGCTTCATCAAGAGGCAGCTGGTCGAGACACGGCAGATCACTAAGCACGTTGCGCAGATTCTCGACTCACGGATGAACACTAAGTACGATGAGAATGACAAGCTGATCCGCGAGGTGAAGGTCATCACCCTGAAGTCAAAGCTCGTCTCCGACTTCAGGAAGGATTTCCAGTTCTACAAGGTTCGGGAGATCAACAATTACCACCATGCCCATGACGCGTACCTGAACGCGGTGGTCGGCACAGCTCTGATCAAGAAGTACCCAAAGCTCGAGAGCGAGTTCGTGTACGGGGACTACAAGGTTTACGATGTGAGGAAGATGATCGCCAAGTCGGAGCAGGAGATTGGCAAGGCTACCGCCAAGTACTTCTTCTACTCTAACATTATGAATTTCTTCAAGACAGAGATCACTCTGGCCAATGGCGAGATCCGGAAGCGCCCCCTCATCGAGACGAACGGCGAGACGGGGGAGATCGTGTGGGACAAGGGCAGGGATTTCGCGACCGTCAGGAAGGTTCTCTCCATGCCACAAGTGAATATCGTCAAGAAGACAGAGGTCCAGACTGGCGGGTTCTCTAAGGAGTCAATTCTGCCTAAGCGGAACAGCGACAAGCTCATCGCCCGCAAGAAGGACTGGGATCCGAAGAAGTACGGCGGGTTCGACAGCCCCACTGTGGCCTACTCGGTCCTGGTTGTGGCGAAGGTTGAGAAGGGCAAGTCCAAGAAGCTCAAGAGCGTGAAGGAGCTGCTGGGGATCACGATTATGGAGCGCTCCAGCTTCGAGAAGAACCCGATCGATTTCCTGGAGGCGAAGGGCTACAAGGAGGTGAAGAAGGACCTGATCATTAAGCTCCCCAAGTACTCACTCTTCGAGCTGGAGAACGGCAGGAAGCGGATGCTGGCTTCCGCTGGCGAGCTGCAGAAGGGGAACGAGCTGGCTCTGCCGTCCAAGTATGTGAACTTCCTCTACCTGGCCTCCCACTACGAGAAGCTCAAGGGCAGCCCCGAGGACAACGAGCAGAAGCAGCTGTTCGTCGAGCAGCACAAGCATTACCTCGACGAGATCATTGAGCAGATTTCCGAGTTCTCCAAGCGCGTGATCCTGGCCGACGCGAATCTGGATAAGGTCCTCTCCGCGTACAACAAGCACCGCGACAAGCCAATCAGGGAGCAGGCTGAGAATATCATTCATCTCTTCACCCTGACGAACCTCGGCGCCCCTGCTGCTTTCAAGTACTTCGACACAACTATCGATCGCAAGAGGTACACAAGCACTAAGGAGGTCCTGGACGCGACCCTCATCCACCAGTCGATTACCGGCCTCTACGAGACGCGCATCGACCTGTCTCAGCTCGGGGGCGACGAATTCTCCGGGAGCGAGACGCCAGGCACCTCCGAGTCGGCCACCCCAGAATCTGCCACAGTGGTGTCCGGCCAAAAGCAGGACCGCCAGGGCGGAGAACGCAGAAGGTCCCAGCTCGATAGGGATCAGTGTGCCTACTGCAAGGAGAAGGGCCACTGGGCCAAAGACTGCCCGAAAAAGCCGCGCGGCCCACGCGGCCCAAGGCCACAAACATCCCTCCTTCCAAAGAAGAAGCGGAAGGTGGAGCTCAGCGGAGGATCTTCCGGAGGATCTAGCGGCTCCGAGACACCAGGAACATCCGAAAGCGCTACACCAGAATCTAGCGGAGGCTCTTCCGGAGGATCTAGGCCTAGCTTCCTGGAGGAGCTCGCCTCCGTGCTGCAAAAATCAGAAGCTGAAGTGGAGGGATTCTTGGAGGGTGCCCCCAAGAAGTACTACAAGATACCTAAAAGTGGCTTCATCGCGCAGCCATCAAAGGAGCTGAAGGAGTACCAGCGGGCGTTCCTCAAGATTCAGTCTCTGCCCATCCATAACGCCGCCAAAGCTTACAGAAAGAACCTGAGCATCAAAGATAATGCCACCGTCCACAAGAGCAGCAGGTACTTCCTTTCCATGGATCTGGAAAATTTCTTCAATTCGATCTCCAGCGACCTCTTCTGGAGAGTCTACTCGCGCCATTTTGATTCATTCTCCGGCCAAGACAAGAGGGTGATGGAGTCCCTCCTCTTTTGGAGCCCTGGGAAGTCCAACAAGCAAGGCCTGGTGCTGAGCGTAGGCGCCCCTTCTTCTCCTCTGCTATCGAACTTCTTCATGTACACATTCGACCTCATCATGTCGAACTATTGTGATGAAACAAACATTCTGTACACTAGGTATGCCGACGACTTCACCTTCTCCACCAATGATAAGAATATTCTGTTCAGCGTGCCGGAGGTGGTGAAAGGTTTTCTGGAGAAGTGCTTTGGAAGAGGTATCAACGTTAATAAAAGAAAGACGGTGTTCAGTAGCAAGGCCCACAATCGCCACGTCACCGGCATCACGATCAACAACAATGGCGACCTGTCTTTGGGCCGGGAGAGAAAGAGATATATTAAGCATCTGATCTTCCAGTATAGCATTAATAAGCTTGAAGAAGGAATGCTGCAGCACCTTCAGGGGCTGCTGGCATTCAGCAACCACATTGAGCCAATGTTTATATTTACACTGAATGAAAAATACACCAAGGAATTGATGTTGAGAATCTCAGAGGAGCGGCACAGCGGCGGCAGCAAAAGAACGGCGGACGGCTCTGAGAAGCGCACCGCTGATAGCCAGCATTCAACTCCTCCGAAAACAAAGAGGAAAGTTGAGTTCGAACCGAAGAAGAAAAGGAAGGTGTGA

**Sequence 28 Plasmids sequence of ePPEplus-RT27**

(NLSSV40-nCas9(H840A/R221K/N394K)-XTEN-NC-NLS-32aa Linker-RT27-NLSvbp)

CCTAAGAAAAAGAGAAAAGTGGACAAGAAGTACTCGATCGGCCTCGATATTGGGACTAACTCTGTTGGCTGGGCCGTGATCACCGACGAGTACAAGGTGCCCTCAAAGAAGTTCAAGGTCCTGGGCAACACCGATCGGCATTCCATCAAGAAGAATCTCATTGGCGCTCTCCTGTTCGACAGCGGCGAGACGGCTGAGGCTACGCGGCTCAAGCGCACCGCCCGCAGGCGGTACACGCGCAGGAAGAATCGCATCTGCTACCTGCAGGAGATTTTCTCCAACGAGATGGCGAAGGTTGACGATTCTTTCTTCCACAGGCTGGAGGAGTCATTCCTCGTGGAGGAGGATAAGAAGCACGAGCGGCATCCAATCTTCGGCAACATTGTCGACGAGGTTGCCTACCACGAGAAGTACCCTACGATCTACCATCTGCGGAAGAAGCTCGTGGACTCCACAGATAAGGCGGACCTCCGCCTGATCTACCTCGCTCTGGCCCACATGATTAAGTTCAGGGGCCATTTCCTGATCGAGGGGGATCTCAACCCGGACAATAGCGATGTTGACAAGCTGTTCATCCAGCTCGTGCAGACGTACAACCAGCTCTTCGAGGAGAACCCCATTAATGCGTCAGGCGTCGACGCGAAGGCTATCCTGTCCGCTAGGCTCTCGAAGTCTCGGAAGCTCGAGAACCTGATCGCCCAGCTGCCGGGCGAGAAGAAGAACGGCCTGTTCGGGAATCTCATTGCGCTCAGCCTGGGGCTCACGCCCAACTTCAAGTCGAATTTCGATCTCGCTGAGGACGCCAAGCTGCAGCTCTCCAAGGACACATACGACGATGACCTGGATAACCTCCTGGCCCAGATCGGCGATCAGTACGCGGACCTGTTCCTCGCTGCCAAGAATCTGTCGGACGCCATCCTCCTGTCTGATATTCTCAGGGTGAACACCGAGATTACGAAGGCTCCGCTCTCAGCCTCCATGATCAAGCGCTACGACGAGCACCATCAGGATCTGACCCTCCTGAAGGCGCTGGTCAGGCAGCAGCTCCCCGAGAAGTACAAGGAGATCTTCTTCGATCAGTCGAAGAACGGCTACGCTGGGTACATTGACGGCGGGGCCTCTCAGGAGGAGTTCTACAAGTTCATCAAGCCGATTCTGGAGAAGATGGACGGCACGGAGGAGCTGCTGGTGAAGCTCAAGCGCGAGGACCTCCTGAGGAAGCAGCGGACATTCGATAACGGCAGCATCCCACACCAGATTCATCTCGGGGAGCTGCACGCTATCCTGAGGAGGCAGGAGGACTTCTACCCTTTCCTCAAGGATAACCGCGAGAAGATCGAGAAGATTCTGACTTTCAGGATCCCGTACTACGTCGGCCCACTCGCTAGGGGCAACTCCCGCTTCGCTTGGATGACCCGCAAGTCAGAGGAGACGATCACGCCGTGGAACTTCGAGGAGGTGGTCGACAAGGGCGCTAGCGCTCAGTCGTTCATCGAGAGGATGACGAATTTCGACAAGAACCTGCCAAATGAGAAGGTGCTCCCTAAGCACTCGCTCCTGTACGAGTACTTCACAGTCTACAACGAGCTGACTAAGGTGAAGTATGTGACCGAGGGCATGAGGAAGCCGGCTTTCCTGTCTGGGGAGCAGAAGAAGGCCATCGTGGACCTCCTGTTCAAGACCAACCGGAAGGTCACGGTTAAGCAGCTCAAGGAGGACTACTTCAAGAAGATTGAGTGCTTCGATTCGGTCGAGATCTCTGGCGTTGAGGACCGCTTCAACGCCTCCCTGGGGACCTACCACGATCTCCTGAAGATCATTAAGGATAAGGACTTCCTGGACAACGAGGAGAATGAGGATATCCTCGAGGACATTGTGCTGACACTCACTCTGTTCGAGGACCGGGAGATGATCGAGGAGCGCCTGAAGACTTACGCCCATCTCTTCGATGACAAGGTCATGAAGCAGCTCAAGAGGAGGAGGTACACCGGCTGGGGGAGGCTGAGCAGGAAGCTCATCAACGGCATTCGGGACAAGCAGTCCGGGAAGACGATCCTCGACTTCCTGAAGAGCGATGGCTTCGCGAACCGCAATTTCATGCAGCTGATTCACGATGACAGCCTCACATTCAAGGAGGATATCCAGAAGGCTCAGGTGAGCGGCCAGGGGGACTCGCTGCACGAGCATATCGCGAACCTCGCTGGCTCGCCAGCTATCAAGAAGGGGATTCTGCAGACCGTGAAGGTTGTGGACGAGCTGGTGAAGGTCATGGGCAGGCACAAGCCTGAGAACATCGTCATTGAGATGGCCCGGGAGAATCAGACCACGCAGAAGGGCCAGAAGAACTCACGCGAGAGGATGAAGAGGATCGAGGAGGGCATTAAGGAGCTGGGGTCCCAGATCCTCAAGGAGCACCCGGTGGAGAACACGCAGCTGCAGAATGAGAAGCTCTACCTGTACTACCTCCAGAATGGCCGCGATATGTATGTGGACCAGGAGCTGGATATTAACAGGCTCAGCGATTACGACGTCGATGCCATCGTTCCACAGTCATTCCTGAAGGATGACTCCATTGACAACAAGGTCCTCACCAGGTCGGACAAGAACCGGGGCAAGTCTGATAATGTTCCTTCAGAGGAGGTCGTTAAGAAGATGAAGAACTACTGGCGCCAGCTCCTGAATGCCAAGCTGATCACGCAGCGGAAGTTCGATAACCTCACAAAGGCTGAGAGGGGCGGGCTCTCTGAGCTGGACAAGGCGGGCTTCATCAAGAGGCAGCTGGTCGAGACACGGCAGATCACTAAGCACGTTGCGCAGATTCTCGACTCACGGATGAACACTAAGTACGATGAGAATGACAAGCTGATCCGCGAGGTGAAGGTCATCACCCTGAAGTCAAAGCTCGTCTCCGACTTCAGGAAGGATTTCCAGTTCTACAAGGTTCGGGAGATCAACAATTACCACCATGCCCATGACGCGTACCTGAACGCGGTGGTCGGCACAGCTCTGATCAAGAAGTACCCAAAGCTCGAGAGCGAGTTCGTGTACGGGGACTACAAGGTTTACGATGTGAGGAAGATGATCGCCAAGTCGGAGCAGGAGATTGGCAAGGCTACCGCCAAGTACTTCTTCTACTCTAACATTATGAATTTCTTCAAGACAGAGATCACTCTGGCCAATGGCGAGATCCGGAAGCGCCCCCTCATCGAGACGAACGGCGAGACGGGGGAGATCGTGTGGGACAAGGGCAGGGATTTCGCGACCGTCAGGAAGGTTCTCTCCATGCCACAAGTGAATATCGTCAAGAAGACAGAGGTCCAGACTGGCGGGTTCTCTAAGGAGTCAATTCTGCCTAAGCGGAACAGCGACAAGCTCATCGCCCGCAAGAAGGACTGGGATCCGAAGAAGTACGGCGGGTTCGACAGCCCCACTGTGGCCTACTCGGTCCTGGTTGTGGCGAAGGTTGAGAAGGGCAAGTCCAAGAAGCTCAAGAGCGTGAAGGAGCTGCTGGGGATCACGATTATGGAGCGCTCCAGCTTCGAGAAGAACCCGATCGATTTCCTGGAGGCGAAGGGCTACAAGGAGGTGAAGAAGGACCTGATCATTAAGCTCCCCAAGTACTCACTCTTCGAGCTGGAGAACGGCAGGAAGCGGATGCTGGCTTCCGCTGGCGAGCTGCAGAAGGGGAACGAGCTGGCTCTGCCGTCCAAGTATGTGAACTTCCTCTACCTGGCCTCCCACTACGAGAAGCTCAAGGGCAGCCCCGAGGACAACGAGCAGAAGCAGCTGTTCGTCGAGCAGCACAAGCATTACCTCGACGAGATCATTGAGCAGATTTCCGAGTTCTCCAAGCGCGTGATCCTGGCCGACGCGAATCTGGATAAGGTCCTCTCCGCGTACAACAAGCACCGCGACAAGCCAATCAGGGAGCAGGCTGAGAATATCATTCATCTCTTCACCCTGACGAACCTCGGCGCCCCTGCTGCTTTCAAGTACTTCGACACAACTATCGATCGCAAGAGGTACACAAGCACTAAGGAGGTCCTGGACGCGACCCTCATCCACCAGTCGATTACCGGCCTCTACGAGACGCGCATCGACCTGTCTCAGCTCGGGGGCGACGAATTCTCCGGGAGCGAGACGCCAGGCACCTCCGAGTCGGCCACCCCAGAATCTGCCACAGTGGTGTCCGGCCAAAAGCAGGACCGCCAGGGCGGAGAACGCAGAAGGTCCCAGCTCGATAGGGATCAGTGTGCCTACTGCAAGGAGAAGGGCCACTGGGCCAAAGACTGCCCGAAAAAGCCGCGCGGCCCACGCGGCCCAAGGCCACAAACATCCCTCCTTCCAAAGAAGAAGCGGAAGGTGGAGCTCAGCGGAGGATCTTCCGGAGGATCTAGCGGCTCCGAGACACCAGGAACATCCGAAAGCGCTACACCAGAATCTAGCGGAGGCTCTTCCGGAGGATCTAGGCCTATGAGCTCCGACCGCCCTATCAAAACAACGGCGAGCGCTATTCCTTCTCTGAAAAAGCTGTCGCAGACTCTAGGATTCTCTATCGAAGAACTGGAGGAGATCAGGGCTATCCCTTTGGAGAAGAGGTATGTGAAATTGGAAAAACCAAAAATCGACGGCACGATGGTGTATAGACCCCACTTCAAGCTGAAGAGACTGCAGCGTAGAATTAACTCCAGGATATTTCGTCCTCTGGTGATATGGCCGGTGTACCTCTACGGAAGCGTACCTTGTGATAATGATGATGAAAGCATAAAGCGCGACTACATCACCTGCGCCAGCCAGCACTGCGCCGCCAAGACAATCCTCAAGATTGATATCAAGAATTTCTTCGACAACATCCACCAAGATATTGTGAGAGACGTGTTTGACAAGGTGCTCAACATCAAGGATGATGCCTTGGATTACATTGTTGATGTCTGCTGTGCCGGCGACTTCATCGTACAGGGCGCCCTCACCAGTTCTTACATAGCCTCTCTGTGTCTGCATGATGTTGAGGCCGACATCTATGCCCGGGCCAAACGGAAAAAGCTGGTCTACACCAGGCTGGTGGACGACATAACCGTAAGCAGCAAAATCCATGATTTTGATTTCTCGCAGATAAGGAAGCACATCGAGGACATGCTTGCCAAGAAAGATTTACCCATCAATGTGGAGAAAAGCGGCGTCTTCATTACAAGTACAAAACCGCTGGTGGTACACGGGTTGAGAATAGACCACAGCAAGCCACGGCTCCCTTCTGATGAAGTTAAGAAGATCCGCGCTTCTCTGCACAACCTTATTGCAAATGCCTCCAAGAACAACAATAAGACCAGTCTGGCATACAGGATCGAGTTCAATAGGTGCATGGGCCGCATCAACAAGCTCGGCCGGGTCGGCCATGAGAAGCATCTGGTGTTTATGTCGAAGATCAAACTGATCCGGCCCATGCCGTCTTACAAGGACATTGTCGACTGCAAGGGAAAGCTGGTGAAGCTGGAAAAGCTCCACGCTAAGGGTTACAACACCTCCGACATCTACTTCCGTAGGTACCAGCTCGTATCGCACCTCCTGTCGCTGATTAACAGATCAAGTTCCTTCCACGAGGTGGTGAAAGATCTGAGAAAGCGGCTGAACAGAATCAAGCCTAGCGGCGGCAGCAAAAGAACGGCGGACGGCTCTGAGAAGCGCACCGCTGATAGCCAGCATTCAACTCCTCCGAAAACAAAGAGGAAAGTTGAGTTCGAACCGAAGAAGAAAAGGAAGGTGTGA

**Sequence 29 Plasmids sequence of ePPEplus-RT28**

(NLSSV40-nCas9(H840A/R221K/N394K)-XTEN-NC-NLS-32aa Linker-RT28-NLSvbp)

CCTAAGAAAAAGAGAAAAGTGGACAAGAAGTACTCGATCGGCCTCGATATTGGGACTAACTCTGTTGGCTGGGCCGTGATCACCGACGAGTACAAGGTGCCCTCAAAGAAGTTCAAGGTCCTGGGCAACACCGATCGGCATTCCATCAAGAAGAATCTCATTGGCGCTCTCCTGTTCGACAGCGGCGAGACGGCTGAGGCTACGCGGCTCAAGCGCACCGCCCGCAGGCGGTACACGCGCAGGAAGAATCGCATCTGCTACCTGCAGGAGATTTTCTCCAACGAGATGGCGAAGGTTGACGATTCTTTCTTCCACAGGCTGGAGGAGTCATTCCTCGTGGAGGAGGATAAGAAGCACGAGCGGCATCCAATCTTCGGCAACATTGTCGACGAGGTTGCCTACCACGAGAAGTACCCTACGATCTACCATCTGCGGAAGAAGCTCGTGGACTCCACAGATAAGGCGGACCTCCGCCTGATCTACCTCGCTCTGGCCCACATGATTAAGTTCAGGGGCCATTTCCTGATCGAGGGGGATCTCAACCCGGACAATAGCGATGTTGACAAGCTGTTCATCCAGCTCGTGCAGACGTACAACCAGCTCTTCGAGGAGAACCCCATTAATGCGTCAGGCGTCGACGCGAAGGCTATCCTGTCCGCTAGGCTCTCGAAGTCTCGGAAGCTCGAGAACCTGATCGCCCAGCTGCCGGGCGAGAAGAAGAACGGCCTGTTCGGGAATCTCATTGCGCTCAGCCTGGGGCTCACGCCCAACTTCAAGTCGAATTTCGATCTCGCTGAGGACGCCAAGCTGCAGCTCTCCAAGGACACATACGACGATGACCTGGATAACCTCCTGGCCCAGATCGGCGATCAGTACGCGGACCTGTTCCTCGCTGCCAAGAATCTGTCGGACGCCATCCTCCTGTCTGATATTCTCAGGGTGAACACCGAGATTACGAAGGCTCCGCTCTCAGCCTCCATGATCAAGCGCTACGACGAGCACCATCAGGATCTGACCCTCCTGAAGGCGCTGGTCAGGCAGCAGCTCCCCGAGAAGTACAAGGAGATCTTCTTCGATCAGTCGAAGAACGGCTACGCTGGGTACATTGACGGCGGGGCCTCTCAGGAGGAGTTCTACAAGTTCATCAAGCCGATTCTGGAGAAGATGGACGGCACGGAGGAGCTGCTGGTGAAGCTCAAGCGCGAGGACCTCCTGAGGAAGCAGCGGACATTCGATAACGGCAGCATCCCACACCAGATTCATCTCGGGGAGCTGCACGCTATCCTGAGGAGGCAGGAGGACTTCTACCCTTTCCTCAAGGATAACCGCGAGAAGATCGAGAAGATTCTGACTTTCAGGATCCCGTACTACGTCGGCCCACTCGCTAGGGGCAACTCCCGCTTCGCTTGGATGACCCGCAAGTCAGAGGAGACGATCACGCCGTGGAACTTCGAGGAGGTGGTCGACAAGGGCGCTAGCGCTCAGTCGTTCATCGAGAGGATGACGAATTTCGACAAGAACCTGCCAAATGAGAAGGTGCTCCCTAAGCACTCGCTCCTGTACGAGTACTTCACAGTCTACAACGAGCTGACTAAGGTGAAGTATGTGACCGAGGGCATGAGGAAGCCGGCTTTCCTGTCTGGGGAGCAGAAGAAGGCCATCGTGGACCTCCTGTTCAAGACCAACCGGAAGGTCACGGTTAAGCAGCTCAAGGAGGACTACTTCAAGAAGATTGAGTGCTTCGATTCGGTCGAGATCTCTGGCGTTGAGGACCGCTTCAACGCCTCCCTGGGGACCTACCACGATCTCCTGAAGATCATTAAGGATAAGGACTTCCTGGACAACGAGGAGAATGAGGATATCCTCGAGGACATTGTGCTGACACTCACTCTGTTCGAGGACCGGGAGATGATCGAGGAGCGCCTGAAGACTTACGCCCATCTCTTCGATGACAAGGTCATGAAGCAGCTCAAGAGGAGGAGGTACACCGGCTGGGGGAGGCTGAGCAGGAAGCTCATCAACGGCATTCGGGACAAGCAGTCCGGGAAGACGATCCTCGACTTCCTGAAGAGCGATGGCTTCGCGAACCGCAATTTCATGCAGCTGATTCACGATGACAGCCTCACATTCAAGGAGGATATCCAGAAGGCTCAGGTGAGCGGCCAGGGGGACTCGCTGCACGAGCATATCGCGAACCTCGCTGGCTCGCCAGCTATCAAGAAGGGGATTCTGCAGACCGTGAAGGTTGTGGACGAGCTGGTGAAGGTCATGGGCAGGCACAAGCCTGAGAACATCGTCATTGAGATGGCCCGGGAGAATCAGACCACGCAGAAGGGCCAGAAGAACTCACGCGAGAGGATGAAGAGGATCGAGGAGGGCATTAAGGAGCTGGGGTCCCAGATCCTCAAGGAGCACCCGGTGGAGAACACGCAGCTGCAGAATGAGAAGCTCTACCTGTACTACCTCCAGAATGGCCGCGATATGTATGTGGACCAGGAGCTGGATATTAACAGGCTCAGCGATTACGACGTCGATGCCATCGTTCCACAGTCATTCCTGAAGGATGACTCCATTGACAACAAGGTCCTCACCAGGTCGGACAAGAACCGGGGCAAGTCTGATAATGTTCCTTCAGAGGAGGTCGTTAAGAAGATGAAGAACTACTGGCGCCAGCTCCTGAATGCCAAGCTGATCACGCAGCGGAAGTTCGATAACCTCACAAAGGCTGAGAGGGGCGGGCTCTCTGAGCTGGACAAGGCGGGCTTCATCAAGAGGCAGCTGGTCGAGACACGGCAGATCACTAAGCACGTTGCGCAGATTCTCGACTCACGGATGAACACTAAGTACGATGAGAATGACAAGCTGATCCGCGAGGTGAAGGTCATCACCCTGAAGTCAAAGCTCGTCTCCGACTTCAGGAAGGATTTCCAGTTCTACAAGGTTCGGGAGATCAACAATTACCACCATGCCCATGACGCGTACCTGAACGCGGTGGTCGGCACAGCTCTGATCAAGAAGTACCCAAAGCTCGAGAGCGAGTTCGTGTACGGGGACTACAAGGTTTACGATGTGAGGAAGATGATCGCCAAGTCGGAGCAGGAGATTGGCAAGGCTACCGCCAAGTACTTCTTCTACTCTAACATTATGAATTTCTTCAAGACAGAGATCACTCTGGCCAATGGCGAGATCCGGAAGCGCCCCCTCATCGAGACGAACGGCGAGACGGGGGAGATCGTGTGGGACAAGGGCAGGGATTTCGCGACCGTCAGGAAGGTTCTCTCCATGCCACAAGTGAATATCGTCAAGAAGACAGAGGTCCAGACTGGCGGGTTCTCTAAGGAGTCAATTCTGCCTAAGCGGAACAGCGACAAGCTCATCGCCCGCAAGAAGGACTGGGATCCGAAGAAGTACGGCGGGTTCGACAGCCCCACTGTGGCCTACTCGGTCCTGGTTGTGGCGAAGGTTGAGAAGGGCAAGTCCAAGAAGCTCAAGAGCGTGAAGGAGCTGCTGGGGATCACGATTATGGAGCGCTCCAGCTTCGAGAAGAACCCGATCGATTTCCTGGAGGCGAAGGGCTACAAGGAGGTGAAGAAGGACCTGATCATTAAGCTCCCCAAGTACTCACTCTTCGAGCTGGAGAACGGCAGGAAGCGGATGCTGGCTTCCGCTGGCGAGCTGCAGAAGGGGAACGAGCTGGCTCTGCCGTCCAAGTATGTGAACTTCCTCTACCTGGCCTCCCACTACGAGAAGCTCAAGGGCAGCCCCGAGGACAACGAGCAGAAGCAGCTGTTCGTCGAGCAGCACAAGCATTACCTCGACGAGATCATTGAGCAGATTTCCGAGTTCTCCAAGCGCGTGATCCTGGCCGACGCGAATCTGGATAAGGTCCTCTCCGCGTACAACAAGCACCGCGACAAGCCAATCAGGGAGCAGGCTGAGAATATCATTCATCTCTTCACCCTGACGAACCTCGGCGCCCCTGCTGCTTTCAAGTACTTCGACACAACTATCGATCGCAAGAGGTACACAAGCACTAAGGAGGTCCTGGACGCGACCCTCATCCACCAGTCGATTACCGGCCTCTACGAGACGCGCATCGACCTGTCTCAGCTCGGGGGCGACGAATTCTCCGGGAGCGAGACGCCAGGCACCTCCGAGTCGGCCACCCCAGAATCTGCCACAGTGGTGTCCGGCCAAAAGCAGGACCGCCAGGGCGGAGAACGCAGAAGGTCCCAGCTCGATAGGGATCAGTGTGCCTACTGCAAGGAGAAGGGCCACTGGGCCAAAGACTGCCCGAAAAAGCCGCGCGGCCCACGCGGCCCAAGGCCACAAACATCCCTCCTTCCAAAGAAGAAGCGGAAGGTGGAGCTCAGCGGAGGATCTTCCGGAGGATCTAGCGGCTCCGAGACACCAGGAACATCCGAAAGCGCTACACCAGAATCTAGCGGAGGCTCTTCCGGAGGATCTAGGCCTCCTTCTAACAAATCTAACTCGGGCATCTCCACACTTACCAGCCTGTCCAAGGCTCTTGGTTTCAGCATTGATGAACTTGTGGAGATCAGATCCATCCCTCTTGACCGGCGCTACCACAAACTGGAGAAGCCCAAGTCAGACGGCAGTATGAGGATAGTGTACCGTCCCCACCACAAGCTGAAAAGGCTGCAGAGGAGAATCAACAATAGAATATTCCGAGAGCTGGTGATATGGCCGAATTTCCTCTTCGGCAGCGTTCCTAACGACAATGACGGAGAGGATTCCCTCAACCGTGACTACATAACTTGTGCCAACCAACACTGCGGCGCCAAGACGCTGCTCAAAGTGGACATTAAAAACTTCTTTGATAACATCCATAAGGAGCTGGTATCTGATATCTTCTCCGACTTCTTCTACATCAATGCTGAAGCTAATGATTACATAACAGATATTTGCTGCGCCGGCGAGTTCATTGTCCAGGGCGCGCTCACCTCCTCTTACATCGCCTCACTGTGTCTCCACGACGTTGAGACCACCGTCTATAGACGTGCAAAGCGCAAGGGGCTAGTGTATACCAGACTGGTTGACGACATCACGGTGAGCTCGAAGGTTTACGATTATGATTTTTCTCAGATCAAGAAGCATATAGAAGACATGCTGGCCGAGAAAGATCTGCCCATCAACATCGTGAAAAGCGGCGTCTTCCAGGTTTCGACGAAGCCACTGCTGGTGCACGGTCTGAGAGTCGACCACAATAAGCCTCGTTTGCCTTCTTATGAAGTGCGCAAGATCCGGGCCAGCCTCAACAACCTCATCGGAAACTCCATTAAGAATAACAACAAGACAAGCCTGGCGTACAGGGTGGAGTACAATCGCTGCATGGGAAGAATCAACAAGCTTGGCCGGCTGGGCCATGAAAAGCATGCGGTGTTCATGGGCCGCATCAAGAAGGTGAGACCGATGCCATCATTTCAAGATGTGAGGGAGTGCAAGAAGAAAATAAAATGGCTCGAGATCCAGTACTCGAAGGGATTCAATAAGAGCGACAAGTACTTCCGGGTGTTTCAGATCACCGGCCACGTCATCAGTCTGATTAACAGAAGCGACGCCTTTTCTACCATCGCCAAGGAACTGCGGCTGAACAAAGTGAAACCTAGCGGCGGCAGCAAAAGAACGGCGGACGGCTCTGAGAAGCGCACCGCTGATAGCCAGCATTCAACTCCTCCGAAAACAAAGAGGAAAGTTGAGTTCGAACCGAAGAAGAAAAGGAAGGTGTGA

**Sequence 30 Plasmids sequence of ePPEplus-RT29**

(NLSSV40-nCas9(H840A/R221K/N394K)-XTEN-NC-NLS-32aa Linker-RT29-NLSvbp)

CCTAAGAAAAAGAGAAAAGTGGACAAGAAGTACTCGATCGGCCTCGATATTGGGACTAACTCTGTTGGCTGGGCCGTGATCACCGACGAGTACAAGGTGCCCTCAAAGAAGTTCAAGGTCCTGGGCAACACCGATCGGCATTCCATCAAGAAGAATCTCATTGGCGCTCTCCTGTTCGACAGCGGCGAGACGGCTGAGGCTACGCGGCTCAAGCGCACCGCCCGCAGGCGGTACACGCGCAGGAAGAATCGCATCTGCTACCTGCAGGAGATTTTCTCCAACGAGATGGCGAAGGTTGACGATTCTTTCTTCCACAGGCTGGAGGAGTCATTCCTCGTGGAGGAGGATAAGAAGCACGAGCGGCATCCAATCTTCGGCAACATTGTCGACGAGGTTGCCTACCACGAGAAGTACCCTACGATCTACCATCTGCGGAAGAAGCTCGTGGACTCCACAGATAAGGCGGACCTCCGCCTGATCTACCTCGCTCTGGCCCACATGATTAAGTTCAGGGGCCATTTCCTGATCGAGGGGGATCTCAACCCGGACAATAGCGATGTTGACAAGCTGTTCATCCAGCTCGTGCAGACGTACAACCAGCTCTTCGAGGAGAACCCCATTAATGCGTCAGGCGTCGACGCGAAGGCTATCCTGTCCGCTAGGCTCTCGAAGTCTCGGAAGCTCGAGAACCTGATCGCCCAGCTGCCGGGCGAGAAGAAGAACGGCCTGTTCGGGAATCTCATTGCGCTCAGCCTGGGGCTCACGCCCAACTTCAAGTCGAATTTCGATCTCGCTGAGGACGCCAAGCTGCAGCTCTCCAAGGACACATACGACGATGACCTGGATAACCTCCTGGCCCAGATCGGCGATCAGTACGCGGACCTGTTCCTCGCTGCCAAGAATCTGTCGGACGCCATCCTCCTGTCTGATATTCTCAGGGTGAACACCGAGATTACGAAGGCTCCGCTCTCAGCCTCCATGATCAAGCGCTACGACGAGCACCATCAGGATCTGACCCTCCTGAAGGCGCTGGTCAGGCAGCAGCTCCCCGAGAAGTACAAGGAGATCTTCTTCGATCAGTCGAAGAACGGCTACGCTGGGTACATTGACGGCGGGGCCTCTCAGGAGGAGTTCTACAAGTTCATCAAGCCGATTCTGGAGAAGATGGACGGCACGGAGGAGCTGCTGGTGAAGCTCAAGCGCGAGGACCTCCTGAGGAAGCAGCGGACATTCGATAACGGCAGCATCCCACACCAGATTCATCTCGGGGAGCTGCACGCTATCCTGAGGAGGCAGGAGGACTTCTACCCTTTCCTCAAGGATAACCGCGAGAAGATCGAGAAGATTCTGACTTTCAGGATCCCGTACTACGTCGGCCCACTCGCTAGGGGCAACTCCCGCTTCGCTTGGATGACCCGCAAGTCAGAGGAGACGATCACGCCGTGGAACTTCGAGGAGGTGGTCGACAAGGGCGCTAGCGCTCAGTCGTTCATCGAGAGGATGACGAATTTCGACAAGAACCTGCCAAATGAGAAGGTGCTCCCTAAGCACTCGCTCCTGTACGAGTACTTCACAGTCTACAACGAGCTGACTAAGGTGAAGTATGTGACCGAGGGCATGAGGAAGCCGGCTTTCCTGTCTGGGGAGCAGAAGAAGGCCATCGTGGACCTCCTGTTCAAGACCAACCGGAAGGTCACGGTTAAGCAGCTCAAGGAGGACTACTTCAAGAAGATTGAGTGCTTCGATTCGGTCGAGATCTCTGGCGTTGAGGACCGCTTCAACGCCTCCCTGGGGACCTACCACGATCTCCTGAAGATCATTAAGGATAAGGACTTCCTGGACAACGAGGAGAATGAGGATATCCTCGAGGACATTGTGCTGACACTCACTCTGTTCGAGGACCGGGAGATGATCGAGGAGCGCCTGAAGACTTACGCCCATCTCTTCGATGACAAGGTCATGAAGCAGCTCAAGAGGAGGAGGTACACCGGCTGGGGGAGGCTGAGCAGGAAGCTCATCAACGGCATTCGGGACAAGCAGTCCGGGAAGACGATCCTCGACTTCCTGAAGAGCGATGGCTTCGCGAACCGCAATTTCATGCAGCTGATTCACGATGACAGCCTCACATTCAAGGAGGATATCCAGAAGGCTCAGGTGAGCGGCCAGGGGGACTCGCTGCACGAGCATATCGCGAACCTCGCTGGCTCGCCAGCTATCAAGAAGGGGATTCTGCAGACCGTGAAGGTTGTGGACGAGCTGGTGAAGGTCATGGGCAGGCACAAGCCTGAGAACATCGTCATTGAGATGGCCCGGGAGAATCAGACCACGCAGAAGGGCCAGAAGAACTCACGCGAGAGGATGAAGAGGATCGAGGAGGGCATTAAGGAGCTGGGGTCCCAGATCCTCAAGGAGCACCCGGTGGAGAACACGCAGCTGCAGAATGAGAAGCTCTACCTGTACTACCTCCAGAATGGCCGCGATATGTATGTGGACCAGGAGCTGGATATTAACAGGCTCAGCGATTACGACGTCGATGCCATCGTTCCACAGTCATTCCTGAAGGATGACTCCATTGACAACAAGGTCCTCACCAGGTCGGACAAGAACCGGGGCAAGTCTGATAATGTTCCTTCAGAGGAGGTCGTTAAGAAGATGAAGAACTACTGGCGCCAGCTCCTGAATGCCAAGCTGATCACGCAGCGGAAGTTCGATAACCTCACAAAGGCTGAGAGGGGCGGGCTCTCTGAGCTGGACAAGGCGGGCTTCATCAAGAGGCAGCTGGTCGAGACACGGCAGATCACTAAGCACGTTGCGCAGATTCTCGACTCACGGATGAACACTAAGTACGATGAGAATGACAAGCTGATCCGCGAGGTGAAGGTCATCACCCTGAAGTCAAAGCTCGTCTCCGACTTCAGGAAGGATTTCCAGTTCTACAAGGTTCGGGAGATCAACAATTACCACCATGCCCATGACGCGTACCTGAACGCGGTGGTCGGCACAGCTCTGATCAAGAAGTACCCAAAGCTCGAGAGCGAGTTCGTGTACGGGGACTACAAGGTTTACGATGTGAGGAAGATGATCGCCAAGTCGGAGCAGGAGATTGGCAAGGCTACCGCCAAGTACTTCTTCTACTCTAACATTATGAATTTCTTCAAGACAGAGATCACTCTGGCCAATGGCGAGATCCGGAAGCGCCCCCTCATCGAGACGAACGGCGAGACGGGGGAGATCGTGTGGGACAAGGGCAGGGATTTCGCGACCGTCAGGAAGGTTCTCTCCATGCCACAAGTGAATATCGTCAAGAAGACAGAGGTCCAGACTGGCGGGTTCTCTAAGGAGTCAATTCTGCCTAAGCGGAACAGCGACAAGCTCATCGCCCGCAAGAAGGACTGGGATCCGAAGAAGTACGGCGGGTTCGACAGCCCCACTGTGGCCTACTCGGTCCTGGTTGTGGCGAAGGTTGAGAAGGGCAAGTCCAAGAAGCTCAAGAGCGTGAAGGAGCTGCTGGGGATCACGATTATGGAGCGCTCCAGCTTCGAGAAGAACCCGATCGATTTCCTGGAGGCGAAGGGCTACAAGGAGGTGAAGAAGGACCTGATCATTAAGCTCCCCAAGTACTCACTCTTCGAGCTGGAGAACGGCAGGAAGCGGATGCTGGCTTCCGCTGGCGAGCTGCAGAAGGGGAACGAGCTGGCTCTGCCGTCCAAGTATGTGAACTTCCTCTACCTGGCCTCCCACTACGAGAAGCTCAAGGGCAGCCCCGAGGACAACGAGCAGAAGCAGCTGTTCGTCGAGCAGCACAAGCATTACCTCGACGAGATCATTGAGCAGATTTCCGAGTTCTCCAAGCGCGTGATCCTGGCCGACGCGAATCTGGATAAGGTCCTCTCCGCGTACAACAAGCACCGCGACAAGCCAATCAGGGAGCAGGCTGAGAATATCATTCATCTCTTCACCCTGACGAACCTCGGCGCCCCTGCTGCTTTCAAGTACTTCGACACAACTATCGATCGCAAGAGGTACACAAGCACTAAGGAGGTCCTGGACGCGACCCTCATCCACCAGTCGATTACCGGCCTCTACGAGACGCGCATCGACCTGTCTCAGCTCGGGGGCGACGAATTCTCCGGGAGCGAGACGCCAGGCACCTCCGAGTCGGCCACCCCAGAATCTGCCACAGTGGTGTCCGGCCAAAAGCAGGACCGCCAGGGCGGAGAACGCAGAAGGTCCCAGCTCGATAGGGATCAGTGTGCCTACTGCAAGGAGAAGGGCCACTGGGCCAAAGACTGCCCGAAAAAGCCGCGCGGCCCACGCGGCCCAAGGCCACAAACATCCCTCCTTCCAAAGAAGAAGCGGAAGGTGGAGCTCAGCGGAGGATCTTCCGGAGGATCTAGCGGCTCCGAGACACCAGGAACATCCGAAAGCGCTACACCAGAATCTAGCGGAGGCTCTTCCGGAGGATCTAGGCCTCCTATTGATCCAAGATTCCCCCTATTCCCTGAAAAAACCAGCAAGCAAGCAATCAGTAGCCTTGAGAGCCTGGCTGAGGCGCTGAGCGTCACCGTGAAGGATCTCGAGGAGGTCAGATCCCTGGAATCTGACAAGTGTTACCGGCTGAAGGAGATCCCAAAGAATGGCGGCGGCCTCAGGAAGGTTTATGATCCTCATCCTCTGATCAGACGGCTACAGTCCAGAATCAATAAAAGAATTTTCCTCGACCTGATCCAGTGGCCGGGCTACCTCTTCGGGTCGTTGCCTAACATAAACATCGACGGACAGCTGGTGTCCAGAGACTACATAGCCTGCGCCCAAAAGCATTGCAAAGCAAAAAGCCTGTTAAAGGTCGATGTGAACAACTTCTTTGACAATATCCACCGCGACCACGTTTTCGACATCTTCAGGATGTTTTTAAAGTTCTCAGATGAAGTAAGCAACTGTCTTGCCGACATTTGCTGTTATAATGGCTTCCTGGTGCAGGGCGCCCTGACATCCAGCTACATTGCCACCCTCTGTTTCTGGCAAAATGAAGGTTACGTCGTTAAAAGACTAAGTAGAAAGGGACTTATTTACACCCGCCTGGTGGATGATATCACTGTGTCTAGTAAGCGCCACGACTTCAACTTTGAGTACGCAGAGCACCACATCAAGATGATGCTCGTCGACCTGGACCTCCCTGCTAACAAGAGAAAGAGAGAAATCTTACGCGACGGCATTGAACCTTTACAGGTGCACGGCCTGAGAGTTAACTATGCTTCCCCCAGACTGCCCGCCGATGAAGTGAGAAGAATTCGGGCCGCCGTCAAGAACGTGGTAAGGATGTCAAAAATCAATAACTACAGGACCTCCCTGTCTTACCGAGCCATGCATGATCGCTGCATGGGACGGGTGAACAAGCTGGCCCGAGTTGGGCACAACAAGCACGGTGATTTTAAGCGGGAGCTGGTGGACGTTATCCCTCTACCGTCCGTGAGAGATCTCGAGAAGGCTGAAAGCTCCATCTCCTTCTTGGAGAAACTCGACCCGGAGAGGTTAAAGCTGCGAAAATACACAAGACGCTACCACCTCGCCAAATACCGTGTGTCTATCGTCGCCAGGACATACTGCCAGGAGGCTGACGACCTCTCATTCCGGCTGCAGAAGCTCAGCGTGTTTGTCGGCGACTATGAGCTGAGCGGCGGCAGCAAAAGAACGGCGGACGGCTCTGAGAAGCGCACCGCTGATAGCCAGCATTCAACTCCTCCGAAAACAAAGAGGAAAGTTGAGTTCGAACCGAAGAAGAAAAGGAAGGTGTGA

**Sequence 31 Plasmids sequence of ePPEplus-RT30**

(NLSSV40-nCas9(H840A/R221K/N394K)-XTEN-NC-NLS-32aa Linker-RT30-NLSvbp)

CCTAAGAAAAAGAGAAAAGTGGACAAGAAGTACTCGATCGGCCTCGATATTGGGACTAACTCTGTTGGCTGGGCCGTGATCACCGACGAGTACAAGGTGCCCTCAAAGAAGTTCAAGGTCCTGGGCAACACCGATCGGCATTCCATCAAGAAGAATCTCATTGGCGCTCTCCTGTTCGACAGCGGCGAGACGGCTGAGGCTACGCGGCTCAAGCGCACCGCCCGCAGGCGGTACACGCGCAGGAAGAATCGCATCTGCTACCTGCAGGAGATTTTCTCCAACGAGATGGCGAAGGTTGACGATTCTTTCTTCCACAGGCTGGAGGAGTCATTCCTCGTGGAGGAGGATAAGAAGCACGAGCGGCATCCAATCTTCGGCAACATTGTCGACGAGGTTGCCTACCACGAGAAGTACCCTACGATCTACCATCTGCGGAAGAAGCTCGTGGACTCCACAGATAAGGCGGACCTCCGCCTGATCTACCTCGCTCTGGCCCACATGATTAAGTTCAGGGGCCATTTCCTGATCGAGGGGGATCTCAACCCGGACAATAGCGATGTTGACAAGCTGTTCATCCAGCTCGTGCAGACGTACAACCAGCTCTTCGAGGAGAACCCCATTAATGCGTCAGGCGTCGACGCGAAGGCTATCCTGTCCGCTAGGCTCTCGAAGTCTCGGAAGCTCGAGAACCTGATCGCCCAGCTGCCGGGCGAGAAGAAGAACGGCCTGTTCGGGAATCTCATTGCGCTCAGCCTGGGGCTCACGCCCAACTTCAAGTCGAATTTCGATCTCGCTGAGGACGCCAAGCTGCAGCTCTCCAAGGACACATACGACGATGACCTGGATAACCTCCTGGCCCAGATCGGCGATCAGTACGCGGACCTGTTCCTCGCTGCCAAGAATCTGTCGGACGCCATCCTCCTGTCTGATATTCTCAGGGTGAACACCGAGATTACGAAGGCTCCGCTCTCAGCCTCCATGATCAAGCGCTACGACGAGCACCATCAGGATCTGACCCTCCTGAAGGCGCTGGTCAGGCAGCAGCTCCCCGAGAAGTACAAGGAGATCTTCTTCGATCAGTCGAAGAACGGCTACGCTGGGTACATTGACGGCGGGGCCTCTCAGGAGGAGTTCTACAAGTTCATCAAGCCGATTCTGGAGAAGATGGACGGCACGGAGGAGCTGCTGGTGAAGCTCAAGCGCGAGGACCTCCTGAGGAAGCAGCGGACATTCGATAACGGCAGCATCCCACACCAGATTCATCTCGGGGAGCTGCACGCTATCCTGAGGAGGCAGGAGGACTTCTACCCTTTCCTCAAGGATAACCGCGAGAAGATCGAGAAGATTCTGACTTTCAGGATCCCGTACTACGTCGGCCCACTCGCTAGGGGCAACTCCCGCTTCGCTTGGATGACCCGCAAGTCAGAGGAGACGATCACGCCGTGGAACTTCGAGGAGGTGGTCGACAAGGGCGCTAGCGCTCAGTCGTTCATCGAGAGGATGACGAATTTCGACAAGAACCTGCCAAATGAGAAGGTGCTCCCTAAGCACTCGCTCCTGTACGAGTACTTCACAGTCTACAACGAGCTGACTAAGGTGAAGTATGTGACCGAGGGCATGAGGAAGCCGGCTTTCCTGTCTGGGGAGCAGAAGAAGGCCATCGTGGACCTCCTGTTCAAGACCAACCGGAAGGTCACGGTTAAGCAGCTCAAGGAGGACTACTTCAAGAAGATTGAGTGCTTCGATTCGGTCGAGATCTCTGGCGTTGAGGACCGCTTCAACGCCTCCCTGGGGACCTACCACGATCTCCTGAAGATCATTAAGGATAAGGACTTCCTGGACAACGAGGAGAATGAGGATATCCTCGAGGACATTGTGCTGACACTCACTCTGTTCGAGGACCGGGAGATGATCGAGGAGCGCCTGAAGACTTACGCCCATCTCTTCGATGACAAGGTCATGAAGCAGCTCAAGAGGAGGAGGTACACCGGCTGGGGGAGGCTGAGCAGGAAGCTCATCAACGGCATTCGGGACAAGCAGTCCGGGAAGACGATCCTCGACTTCCTGAAGAGCGATGGCTTCGCGAACCGCAATTTCATGCAGCTGATTCACGATGACAGCCTCACATTCAAGGAGGATATCCAGAAGGCTCAGGTGAGCGGCCAGGGGGACTCGCTGCACGAGCATATCGCGAACCTCGCTGGCTCGCCAGCTATCAAGAAGGGGATTCTGCAGACCGTGAAGGTTGTGGACGAGCTGGTGAAGGTCATGGGCAGGCACAAGCCTGAGAACATCGTCATTGAGATGGCCCGGGAGAATCAGACCACGCAGAAGGGCCAGAAGAACTCACGCGAGAGGATGAAGAGGATCGAGGAGGGCATTAAGGAGCTGGGGTCCCAGATCCTCAAGGAGCACCCGGTGGAGAACACGCAGCTGCAGAATGAGAAGCTCTACCTGTACTACCTCCAGAATGGCCGCGATATGTATGTGGACCAGGAGCTGGATATTAACAGGCTCAGCGATTACGACGTCGATGCCATCGTTCCACAGTCATTCCTGAAGGATGACTCCATTGACAACAAGGTCCTCACCAGGTCGGACAAGAACCGGGGCAAGTCTGATAATGTTCCTTCAGAGGAGGTCGTTAAGAAGATGAAGAACTACTGGCGCCAGCTCCTGAATGCCAAGCTGATCACGCAGCGGAAGTTCGATAACCTCACAAAGGCTGAGAGGGGCGGGCTCTCTGAGCTGGACAAGGCGGGCTTCATCAAGAGGCAGCTGGTCGAGACACGGCAGATCACTAAGCACGTTGCGCAGATTCTCGACTCACGGATGAACACTAAGTACGATGAGAATGACAAGCTGATCCGCGAGGTGAAGGTCATCACCCTGAAGTCAAAGCTCGTCTCCGACTTCAGGAAGGATTTCCAGTTCTACAAGGTTCGGGAGATCAACAATTACCACCATGCCCATGACGCGTACCTGAACGCGGTGGTCGGCACAGCTCTGATCAAGAAGTACCCAAAGCTCGAGAGCGAGTTCGTGTACGGGGACTACAAGGTTTACGATGTGAGGAAGATGATCGCCAAGTCGGAGCAGGAGATTGGCAAGGCTACCGCCAAGTACTTCTTCTACTCTAACATTATGAATTTCTTCAAGACAGAGATCACTCTGGCCAATGGCGAGATCCGGAAGCGCCCCCTCATCGAGACGAACGGCGAGACGGGGGAGATCGTGTGGGACAAGGGCAGGGATTTCGCGACCGTCAGGAAGGTTCTCTCCATGCCACAAGTGAATATCGTCAAGAAGACAGAGGTCCAGACTGGCGGGTTCTCTAAGGAGTCAATTCTGCCTAAGCGGAACAGCGACAAGCTCATCGCCCGCAAGAAGGACTGGGATCCGAAGAAGTACGGCGGGTTCGACAGCCCCACTGTGGCCTACTCGGTCCTGGTTGTGGCGAAGGTTGAGAAGGGCAAGTCCAAGAAGCTCAAGAGCGTGAAGGAGCTGCTGGGGATCACGATTATGGAGCGCTCCAGCTTCGAGAAGAACCCGATCGATTTCCTGGAGGCGAAGGGCTACAAGGAGGTGAAGAAGGACCTGATCATTAAGCTCCCCAAGTACTCACTCTTCGAGCTGGAGAACGGCAGGAAGCGGATGCTGGCTTCCGCTGGCGAGCTGCAGAAGGGGAACGAGCTGGCTCTGCCGTCCAAGTATGTGAACTTCCTCTACCTGGCCTCCCACTACGAGAAGCTCAAGGGCAGCCCCGAGGACAACGAGCAGAAGCAGCTGTTCGTCGAGCAGCACAAGCATTACCTCGACGAGATCATTGAGCAGATTTCCGAGTTCTCCAAGCGCGTGATCCTGGCCGACGCGAATCTGGATAAGGTCCTCTCCGCGTACAACAAGCACCGCGACAAGCCAATCAGGGAGCAGGCTGAGAATATCATTCATCTCTTCACCCTGACGAACCTCGGCGCCCCTGCTGCTTTCAAGTACTTCGACACAACTATCGATCGCAAGAGGTACACAAGCACTAAGGAGGTCCTGGACGCGACCCTCATCCACCAGTCGATTACCGGCCTCTACGAGACGCGCATCGACCTGTCTCAGCTCGGGGGCGACGAATTCTCCGGGAGCGAGACGCCAGGCACCTCCGAGTCGGCCACCCCAGAATCTGCCACAGTGGTGTCCGGCCAAAAGCAGGACCGCCAGGGCGGAGAACGCAGAAGGTCCCAGCTCGATAGGGATCAGTGTGCCTACTGCAAGGAGAAGGGCCACTGGGCCAAAGACTGCCCGAAAAAGCCGCGCGGCCCACGCGGCCCAAGGCCACAAACATCCCTCCTTCCAAAGAAGAAGCGGAAGGTGGAGCTCAGCGGAGGATCTTCCGGAGGATCTAGCGGCTCCGAGACACCAGGAACATCCGAAAGCGCTACACCAGAATCTAGCGGAGGCTCTTCCGGAGGATCTAGGCCTCTGGCCAAGGAGAATCTGAACACTGCGTACTTGCAAGTTGTCCGGAACAAGGGCGCGGAGGGGGTGGATGGCATGACATATACTGAACTGAAAGAGCACCTGGAGAACAATGGTGAGATTATCAAGGACCAGCTGCGCGCGAGGAAGTACACCCCGCGGCCGGTGCGCCGGGTTGAAATACCAAAGCCCGACGGCGGCGTAAGAAATTTGGGTGTCCCAACGGTGACCGACAGATTTGTGCAACAAGCAATTGCACAAGTGCTCACTCCAATTTATGAGAAGCAGTTCCATGATCATTCGTACGGCTTCAGACCTGACCGATGTGCTCAGCAGGCCATCATCACCGCCTTAAATCTCATGAATGAAGGAAATGATTGGATCGTCGACATTGATCTTGAAAAGTTCTTTGATACCGTCAACCACGACAAGCTGATGACGCTCATCGGCCGTACAATAAAAGAAGGTGATGTTATAAGCATCATTCGCAAATTCCTCGTCTCCGGCATTATGGTTGATGATGAGTACAAGGAAGCTGTCATCGGGACGCCGCAGGGAGGCAACCTTTCACCCCTCCTCGCCAACATCATGCTCAATGAGCTTGATAAGGAGATGGAGCAGCGCGGCCTCAACTTCGTGAGGTATGCTGACGACTGCATCATAATGGTGGGTTCTGAAATGTCAGCAAAGAGGGTGATGAGGAACTTGACAAAGTTTATTGAGGAGAAACTGGGGCTCAAGGTGAACATGACCAAGAGCAAAGTAGACCGCCCTAGTGGATTGAAATATCTTGGATTTGGGTTCTACTTCGACTCCAAGGCGCACCAGAGCGGCGGCAGCAAAAGAACGGCGGACGGCTCTGAGAAGCGCACCGCTGATAGCCAGCATTCAACTCCTCCGAAAACAAAGAGGAAAGTTGAGTTCGAACCGAAGAAGAAAAGGAAGGTGTGA

**Sequence 32 Plasmids sequence of ePPEplus-RT31**

(NLSSV40-nCas9(H840A/R221K/N394K)-XTEN-NC-NLS-32aa Linker-RT31-NLSvbp)

CCTAAGAAAAAGAGAAAAGTGGACAAGAAGTACTCGATCGGCCTCGATATTGGGACTAACTCTGTTGGCTGGGCCGTGATCACCGACGAGTACAAGGTGCCCTCAAAGAAGTTCAAGGTCCTGGGCAACACCGATCGGCATTCCATCAAGAAGAATCTCATTGGCGCTCTCCTGTTCGACAGCGGCGAGACGGCTGAGGCTACGCGGCTCAAGCGCACCGCCCGCAGGCGGTACACGCGCAGGAAGAATCGCATCTGCTACCTGCAGGAGATTTTCTCCAACGAGATGGCGAAGGTTGACGATTCTTTCTTCCACAGGCTGGAGGAGTCATTCCTCGTGGAGGAGGATAAGAAGCACGAGCGGCATCCAATCTTCGGCAACATTGTCGACGAGGTTGCCTACCACGAGAAGTACCCTACGATCTACCATCTGCGGAAGAAGCTCGTGGACTCCACAGATAAGGCGGACCTCCGCCTGATCTACCTCGCTCTGGCCCACATGATTAAGTTCAGGGGCCATTTCCTGATCGAGGGGGATCTCAACCCGGACAATAGCGATGTTGACAAGCTGTTCATCCAGCTCGTGCAGACGTACAACCAGCTCTTCGAGGAGAACCCCATTAATGCGTCAGGCGTCGACGCGAAGGCTATCCTGTCCGCTAGGCTCTCGAAGTCTCGGAAGCTCGAGAACCTGATCGCCCAGCTGCCGGGCGAGAAGAAGAACGGCCTGTTCGGGAATCTCATTGCGCTCAGCCTGGGGCTCACGCCCAACTTCAAGTCGAATTTCGATCTCGCTGAGGACGCCAAGCTGCAGCTCTCCAAGGACACATACGACGATGACCTGGATAACCTCCTGGCCCAGATCGGCGATCAGTACGCGGACCTGTTCCTCGCTGCCAAGAATCTGTCGGACGCCATCCTCCTGTCTGATATTCTCAGGGTGAACACCGAGATTACGAAGGCTCCGCTCTCAGCCTCCATGATCAAGCGCTACGACGAGCACCATCAGGATCTGACCCTCCTGAAGGCGCTGGTCAGGCAGCAGCTCCCCGAGAAGTACAAGGAGATCTTCTTCGATCAGTCGAAGAACGGCTACGCTGGGTACATTGACGGCGGGGCCTCTCAGGAGGAGTTCTACAAGTTCATCAAGCCGATTCTGGAGAAGATGGACGGCACGGAGGAGCTGCTGGTGAAGCTCAAGCGCGAGGACCTCCTGAGGAAGCAGCGGACATTCGATAACGGCAGCATCCCACACCAGATTCATCTCGGGGAGCTGCACGCTATCCTGAGGAGGCAGGAGGACTTCTACCCTTTCCTCAAGGATAACCGCGAGAAGATCGAGAAGATTCTGACTTTCAGGATCCCGTACTACGTCGGCCCACTCGCTAGGGGCAACTCCCGCTTCGCTTGGATGACCCGCAAGTCAGAGGAGACGATCACGCCGTGGAACTTCGAGGAGGTGGTCGACAAGGGCGCTAGCGCTCAGTCGTTCATCGAGAGGATGACGAATTTCGACAAGAACCTGCCAAATGAGAAGGTGCTCCCTAAGCACTCGCTCCTGTACGAGTACTTCACAGTCTACAACGAGCTGACTAAGGTGAAGTATGTGACCGAGGGCATGAGGAAGCCGGCTTTCCTGTCTGGGGAGCAGAAGAAGGCCATCGTGGACCTCCTGTTCAAGACCAACCGGAAGGTCACGGTTAAGCAGCTCAAGGAGGACTACTTCAAGAAGATTGAGTGCTTCGATTCGGTCGAGATCTCTGGCGTTGAGGACCGCTTCAACGCCTCCCTGGGGACCTACCACGATCTCCTGAAGATCATTAAGGATAAGGACTTCCTGGACAACGAGGAGAATGAGGATATCCTCGAGGACATTGTGCTGACACTCACTCTGTTCGAGGACCGGGAGATGATCGAGGAGCGCCTGAAGACTTACGCCCATCTCTTCGATGACAAGGTCATGAAGCAGCTCAAGAGGAGGAGGTACACCGGCTGGGGGAGGCTGAGCAGGAAGCTCATCAACGGCATTCGGGACAAGCAGTCCGGGAAGACGATCCTCGACTTCCTGAAGAGCGATGGCTTCGCGAACCGCAATTTCATGCAGCTGATTCACGATGACAGCCTCACATTCAAGGAGGATATCCAGAAGGCTCAGGTGAGCGGCCAGGGGGACTCGCTGCACGAGCATATCGCGAACCTCGCTGGCTCGCCAGCTATCAAGAAGGGGATTCTGCAGACCGTGAAGGTTGTGGACGAGCTGGTGAAGGTCATGGGCAGGCACAAGCCTGAGAACATCGTCATTGAGATGGCCCGGGAGAATCAGACCACGCAGAAGGGCCAGAAGAACTCACGCGAGAGGATGAAGAGGATCGAGGAGGGCATTAAGGAGCTGGGGTCCCAGATCCTCAAGGAGCACCCGGTGGAGAACACGCAGCTGCAGAATGAGAAGCTCTACCTGTACTACCTCCAGAATGGCCGCGATATGTATGTGGACCAGGAGCTGGATATTAACAGGCTCAGCGATTACGACGTCGATGCCATCGTTCCACAGTCATTCCTGAAGGATGACTCCATTGACAACAAGGTCCTCACCAGGTCGGACAAGAACCGGGGCAAGTCTGATAATGTTCCTTCAGAGGAGGTCGTTAAGAAGATGAAGAACTACTGGCGCCAGCTCCTGAATGCCAAGCTGATCACGCAGCGGAAGTTCGATAACCTCACAAAGGCTGAGAGGGGCGGGCTCTCTGAGCTGGACAAGGCGGGCTTCATCAAGAGGCAGCTGGTCGAGACACGGCAGATCACTAAGCACGTTGCGCAGATTCTCGACTCACGGATGAACACTAAGTACGATGAGAATGACAAGCTGATCCGCGAGGTGAAGGTCATCACCCTGAAGTCAAAGCTCGTCTCCGACTTCAGGAAGGATTTCCAGTTCTACAAGGTTCGGGAGATCAACAATTACCACCATGCCCATGACGCGTACCTGAACGCGGTGGTCGGCACAGCTCTGATCAAGAAGTACCCAAAGCTCGAGAGCGAGTTCGTGTACGGGGACTACAAGGTTTACGATGTGAGGAAGATGATCGCCAAGTCGGAGCAGGAGATTGGCAAGGCTACCGCCAAGTACTTCTTCTACTCTAACATTATGAATTTCTTCAAGACAGAGATCACTCTGGCCAATGGCGAGATCCGGAAGCGCCCCCTCATCGAGACGAACGGCGAGACGGGGGAGATCGTGTGGGACAAGGGCAGGGATTTCGCGACCGTCAGGAAGGTTCTCTCCATGCCACAAGTGAATATCGTCAAGAAGACAGAGGTCCAGACTGGCGGGTTCTCTAAGGAGTCAATTCTGCCTAAGCGGAACAGCGACAAGCTCATCGCCCGCAAGAAGGACTGGGATCCGAAGAAGTACGGCGGGTTCGACAGCCCCACTGTGGCCTACTCGGTCCTGGTTGTGGCGAAGGTTGAGAAGGGCAAGTCCAAGAAGCTCAAGAGCGTGAAGGAGCTGCTGGGGATCACGATTATGGAGCGCTCCAGCTTCGAGAAGAACCCGATCGATTTCCTGGAGGCGAAGGGCTACAAGGAGGTGAAGAAGGACCTGATCATTAAGCTCCCCAAGTACTCACTCTTCGAGCTGGAGAACGGCAGGAAGCGGATGCTGGCTTCCGCTGGCGAGCTGCAGAAGGGGAACGAGCTGGCTCTGCCGTCCAAGTATGTGAACTTCCTCTACCTGGCCTCCCACTACGAGAAGCTCAAGGGCAGCCCCGAGGACAACGAGCAGAAGCAGCTGTTCGTCGAGCAGCACAAGCATTACCTCGACGAGATCATTGAGCAGATTTCCGAGTTCTCCAAGCGCGTGATCCTGGCCGACGCGAATCTGGATAAGGTCCTCTCCGCGTACAACAAGCACCGCGACAAGCCAATCAGGGAGCAGGCTGAGAATATCATTCATCTCTTCACCCTGACGAACCTCGGCGCCCCTGCTGCTTTCAAGTACTTCGACACAACTATCGATCGCAAGAGGTACACAAGCACTAAGGAGGTCCTGGACGCGACCCTCATCCACCAGTCGATTACCGGCCTCTACGAGACGCGCATCGACCTGTCTCAGCTCGGGGGCGACGAATTCTCCGGGAGCGAGACGCCAGGCACCTCCGAGTCGGCCACCCCAGAATCTGCCACAGTGGTGTCCGGCCAAAAGCAGGACCGCCAGGGCGGAGAACGCAGAAGGTCCCAGCTCGATAGGGATCAGTGTGCCTACTGCAAGGAGAAGGGCCACTGGGCCAAAGACTGCCCGAAAAAGCCGCGCGGCCCACGCGGCCCAAGGCCACAAACATCCCTCCTTCCAAAGAAGAAGCGGAAGGTGGAGCTCAGCGGAGGATCTTCCGGAGGATCTAGCGGCTCCGAGACACCAGGAACATCCGAAAGCGCTACACCAGAATCTAGCGGAGGCTCTTCCGGAGGATCTAGGCCTGTGCCTGACTGGATTCGGAAGGATTACGGCACGGTGCTGAGGGAGGAGCTCCCTCCTCAGATGCCGCCCACCAGATCAGTGGATCATCAGATTCCTTTGAAGCCTGACATGCCTCCGCCGTTCAAGGGAATCTTTCGTCTTTCTCAGCTGGAGCTGCGGGAATTGAAAAGGCAGTTGGATCAGCTGCTTAAAGACGGCAAAATCAAGCCCTCCACCAGCCCATACGGCGCGCCGGTGCTCTTCGTCAAGAAGAAAGATGATAAGTTGAGGATGTGCATCGACTACCGCGCCCTAAATAGTCAGACAATCCAAAACAGGTACGCCCTGCCTAGGATCGATGAATTGTTTGATCGGCTGCACGGCGCCAAAATCTTCAGCAAACTCGATCTGACCTCAGGCTACTATCAGATCGCTATCAAGCCAAAAGACAGACACAAGACCGCCTTCCGCTATGGTCACTACGAGTTCAACGTCATGCCATTTGGTCTTACCAACGCCCCCGCCACCTTCCAGACACTGATGAATGATATCTTCAGAGACCTTCTAGACGTCTGCGTCATCGTCTATCTCGACGACATTCTGGTATACTCCAAGAACAAGGAAGAGCATGAGCAACACCTCCGGCAGGTGTTGCAGAGACTCAAGAATAACCAACTATACGCAAAGCTCTCAAAATGTACATTCTTCACAAACAGCATCGAGTACCTCGGCCATATTGTGGACGGGGAAGGCCTGCGGCCAAATCCACGGCTGGTTCAGGCGCTGAAGGACTTCCCGCAGCCCAAGACCCTCAAGGAGCTGCAGTCATTCCTGGGCCTGGCCAACTACTACAGAAAATTCATCGCGAACTTCTCTCACATTGCCCTGCCCCTGACAGACGCAACCCGCAATAACACACAGAGCAACCTGAGACCAATAGAGTGGACACAGTCGATGCAGACTGCTTTTGAAAAGCTAAAGGAAGCCTTAACGTCGGCCCCCTGCCTGGCCCTGCCGGACCCTGATGGAGAGTTTGAGGTGACAACCGACGCATCCGAGGACACCAAGGCCGTTGGCGCGGTGCTCATGCAAAATGGACACCCAGTGGCATACGAAAGCACAAAGTTAAACTCCCACCAGCTCAACTATGCTGTGCACGACAAGGAGATGTGTGCTATTATGCATGCCCTGGAGAGATGGCGCCCTTTCCTCCTTGGCCGTCACTTCAAGGTGTACACGGACCATAGAAGCCTCGTCCACTTTAAGACACAAAGCAACCTAAATCAGCGGCAACTGAGATGGCAGGAGAAGGCCGCCGATTATGATATGGAGATCCTCTACAAGCCTGGAAAGGAAAATGTTGTGGCTGACGCCCTGTCCCGAAGCGGCGGCAGCAAAAGAACGGCGGACGGCTCTGAGAAGCGCACCGCTGATAGCCAGCATTCAACTCCTCCGAAAACAAAGAGGAAAGTTGAGTTCGAACCGAAGAAGAAAAGGAAGGTGTGA

**Sequence 33 Plasmids sequence of ePPEplus-RT32**

(NLSSV40-nCas9(H840A/R221K/N394K)-XTEN-NC-NLS-32aa Linker-RT32-NLSvbp)

CCTAAGAAAAAGAGAAAAGTGGACAAGAAGTACTCGATCGGCCTCGATATTGGGACTAACTCTGTTGGCTGGGCCGTGATCACCGACGAGTACAAGGTGCCCTCAAAGAAGTTCAAGGTCCTGGGCAACACCGATCGGCATTCCATCAAGAAGAATCTCATTGGCGCTCTCCTGTTCGACAGCGGCGAGACGGCTGAGGCTACGCGGCTCAAGCGCACCGCCCGCAGGCGGTACACGCGCAGGAAGAATCGCATCTGCTACCTGCAGGAGATTTTCTCCAACGAGATGGCGAAGGTTGACGATTCTTTCTTCCACAGGCTGGAGGAGTCATTCCTCGTGGAGGAGGATAAGAAGCACGAGCGGCATCCAATCTTCGGCAACATTGTCGACGAGGTTGCCTACCACGAGAAGTACCCTACGATCTACCATCTGCGGAAGAAGCTCGTGGACTCCACAGATAAGGCGGACCTCCGCCTGATCTACCTCGCTCTGGCCCACATGATTAAGTTCAGGGGCCATTTCCTGATCGAGGGGGATCTCAACCCGGACAATAGCGATGTTGACAAGCTGTTCATCCAGCTCGTGCAGACGTACAACCAGCTCTTCGAGGAGAACCCCATTAATGCGTCAGGCGTCGACGCGAAGGCTATCCTGTCCGCTAGGCTCTCGAAGTCTCGGAAGCTCGAGAACCTGATCGCCCAGCTGCCGGGCGAGAAGAAGAACGGCCTGTTCGGGAATCTCATTGCGCTCAGCCTGGGGCTCACGCCCAACTTCAAGTCGAATTTCGATCTCGCTGAGGACGCCAAGCTGCAGCTCTCCAAGGACACATACGACGATGACCTGGATAACCTCCTGGCCCAGATCGGCGATCAGTACGCGGACCTGTTCCTCGCTGCCAAGAATCTGTCGGACGCCATCCTCCTGTCTGATATTCTCAGGGTGAACACCGAGATTACGAAGGCTCCGCTCTCAGCCTCCATGATCAAGCGCTACGACGAGCACCATCAGGATCTGACCCTCCTGAAGGCGCTGGTCAGGCAGCAGCTCCCCGAGAAGTACAAGGAGATCTTCTTCGATCAGTCGAAGAACGGCTACGCTGGGTACATTGACGGCGGGGCCTCTCAGGAGGAGTTCTACAAGTTCATCAAGCCGATTCTGGAGAAGATGGACGGCACGGAGGAGCTGCTGGTGAAGCTCAAGCGCGAGGACCTCCTGAGGAAGCAGCGGACATTCGATAACGGCAGCATCCCACACCAGATTCATCTCGGGGAGCTGCACGCTATCCTGAGGAGGCAGGAGGACTTCTACCCTTTCCTCAAGGATAACCGCGAGAAGATCGAGAAGATTCTGACTTTCAGGATCCCGTACTACGTCGGCCCACTCGCTAGGGGCAACTCCCGCTTCGCTTGGATGACCCGCAAGTCAGAGGAGACGATCACGCCGTGGAACTTCGAGGAGGTGGTCGACAAGGGCGCTAGCGCTCAGTCGTTCATCGAGAGGATGACGAATTTCGACAAGAACCTGCCAAATGAGAAGGTGCTCCCTAAGCACTCGCTCCTGTACGAGTACTTCACAGTCTACAACGAGCTGACTAAGGTGAAGTATGTGACCGAGGGCATGAGGAAGCCGGCTTTCCTGTCTGGGGAGCAGAAGAAGGCCATCGTGGACCTCCTGTTCAAGACCAACCGGAAGGTCACGGTTAAGCAGCTCAAGGAGGACTACTTCAAGAAGATTGAGTGCTTCGATTCGGTCGAGATCTCTGGCGTTGAGGACCGCTTCAACGCCTCCCTGGGGACCTACCACGATCTCCTGAAGATCATTAAGGATAAGGACTTCCTGGACAACGAGGAGAATGAGGATATCCTCGAGGACATTGTGCTGACACTCACTCTGTTCGAGGACCGGGAGATGATCGAGGAGCGCCTGAAGACTTACGCCCATCTCTTCGATGACAAGGTCATGAAGCAGCTCAAGAGGAGGAGGTACACCGGCTGGGGGAGGCTGAGCAGGAAGCTCATCAACGGCATTCGGGACAAGCAGTCCGGGAAGACGATCCTCGACTTCCTGAAGAGCGATGGCTTCGCGAACCGCAATTTCATGCAGCTGATTCACGATGACAGCCTCACATTCAAGGAGGATATCCAGAAGGCTCAGGTGAGCGGCCAGGGGGACTCGCTGCACGAGCATATCGCGAACCTCGCTGGCTCGCCAGCTATCAAGAAGGGGATTCTGCAGACCGTGAAGGTTGTGGACGAGCTGGTGAAGGTCATGGGCAGGCACAAGCCTGAGAACATCGTCATTGAGATGGCCCGGGAGAATCAGACCACGCAGAAGGGCCAGAAGAACTCACGCGAGAGGATGAAGAGGATCGAGGAGGGCATTAAGGAGCTGGGGTCCCAGATCCTCAAGGAGCACCCGGTGGAGAACACGCAGCTGCAGAATGAGAAGCTCTACCTGTACTACCTCCAGAATGGCCGCGATATGTATGTGGACCAGGAGCTGGATATTAACAGGCTCAGCGATTACGACGTCGATGCCATCGTTCCACAGTCATTCCTGAAGGATGACTCCATTGACAACAAGGTCCTCACCAGGTCGGACAAGAACCGGGGCAAGTCTGATAATGTTCCTTCAGAGGAGGTCGTTAAGAAGATGAAGAACTACTGGCGCCAGCTCCTGAATGCCAAGCTGATCACGCAGCGGAAGTTCGATAACCTCACAAAGGCTGAGAGGGGCGGGCTCTCTGAGCTGGACAAGGCGGGCTTCATCAAGAGGCAGCTGGTCGAGACACGGCAGATCACTAAGCACGTTGCGCAGATTCTCGACTCACGGATGAACACTAAGTACGATGAGAATGACAAGCTGATCCGCGAGGTGAAGGTCATCACCCTGAAGTCAAAGCTCGTCTCCGACTTCAGGAAGGATTTCCAGTTCTACAAGGTTCGGGAGATCAACAATTACCACCATGCCCATGACGCGTACCTGAACGCGGTGGTCGGCACAGCTCTGATCAAGAAGTACCCAAAGCTCGAGAGCGAGTTCGTGTACGGGGACTACAAGGTTTACGATGTGAGGAAGATGATCGCCAAGTCGGAGCAGGAGATTGGCAAGGCTACCGCCAAGTACTTCTTCTACTCTAACATTATGAATTTCTTCAAGACAGAGATCACTCTGGCCAATGGCGAGATCCGGAAGCGCCCCCTCATCGAGACGAACGGCGAGACGGGGGAGATCGTGTGGGACAAGGGCAGGGATTTCGCGACCGTCAGGAAGGTTCTCTCCATGCCACAAGTGAATATCGTCAAGAAGACAGAGGTCCAGACTGGCGGGTTCTCTAAGGAGTCAATTCTGCCTAAGCGGAACAGCGACAAGCTCATCGCCCGCAAGAAGGACTGGGATCCGAAGAAGTACGGCGGGTTCGACAGCCCCACTGTGGCCTACTCGGTCCTGGTTGTGGCGAAGGTTGAGAAGGGCAAGTCCAAGAAGCTCAAGAGCGTGAAGGAGCTGCTGGGGATCACGATTATGGAGCGCTCCAGCTTCGAGAAGAACCCGATCGATTTCCTGGAGGCGAAGGGCTACAAGGAGGTGAAGAAGGACCTGATCATTAAGCTCCCCAAGTACTCACTCTTCGAGCTGGAGAACGGCAGGAAGCGGATGCTGGCTTCCGCTGGCGAGCTGCAGAAGGGGAACGAGCTGGCTCTGCCGTCCAAGTATGTGAACTTCCTCTACCTGGCCTCCCACTACGAGAAGCTCAAGGGCAGCCCCGAGGACAACGAGCAGAAGCAGCTGTTCGTCGAGCAGCACAAGCATTACCTCGACGAGATCATTGAGCAGATTTCCGAGTTCTCCAAGCGCGTGATCCTGGCCGACGCGAATCTGGATAAGGTCCTCTCCGCGTACAACAAGCACCGCGACAAGCCAATCAGGGAGCAGGCTGAGAATATCATTCATCTCTTCACCCTGACGAACCTCGGCGCCCCTGCTGCTTTCAAGTACTTCGACACAACTATCGATCGCAAGAGGTACACAAGCACTAAGGAGGTCCTGGACGCGACCCTCATCCACCAGTCGATTACCGGCCTCTACGAGACGCGCATCGACCTGTCTCAGCTCGGGGGCGACGAATTCTCCGGGAGCGAGACGCCAGGCACCTCCGAGTCGGCCACCCCAGAATCTGCCACAGTGGTGTCCGGCCAAAAGCAGGACCGCCAGGGCGGAGAACGCAGAAGGTCCCAGCTCGATAGGGATCAGTGTGCCTACTGCAAGGAGAAGGGCCACTGGGCCAAAGACTGCCCGAAAAAGCCGCGCGGCCCACGCGGCCCAAGGCCACAAACATCCCTCCTTCCAAAGAAGAAGCGGAAGGTGGAGCTCAGCGGAGGATCTTCCGGAGGATCTAGCGGCTCCGAGACACCAGGAACATCCGAAAGCGCTACACCAGAATCTAGCGGAGGCTCTTCCGGAGGATCTAGGCCTATAGATCAGTGGCTCTCCAAATACCCGGACACGTGGGCTGAAACTGGCGGCATGGGGCTGGCCATCCATCAGCCGCCGCTGATTGTGGAACTCAAAGCTACTGAAACACCTGTTGCGGTGAAGCAGTATCCAATGACAGCTGAGGCCCGCCAAGGTATTAGACCGCACATCAAGAGGTTGCTCGAGCAAGGAATACTTATTCCCTGCTGGGCGGCGTGGAACACACCATTGCTTCCTGTCAAGAAACCTGGAAGCGGCGACTACCGCCCCGTCCAAGATCTCCGCGAGATCAACAAACGTGTAGAAGATATTCATCCAACTGTTCCAAATCCTTATAATTTGCTATCGACTTTAAGTCCATCTCACACATGGTACAGTGTTCTGGACCTGAAAGATGCATTCTTCTGTCTTAAGCTGCACCACCAATCACAACCTCTGTTCACCTTTGAGTGGAAGGATTCTGATCTTGGAATCTCAGGGCAGCTGACCTGGACAAGACTGCCGCAGGGCTTCAAGAACAGCCCCACACTCTTTGATGAAGCACTCCACCAGGACTTAACGGCCTTCCGCACCCTCCATCCTCATCTCATTCTGCTGCAGTACGTCGACGACATCCTGCTCGCTGCCGAGACGAGGGAGGAGTGCCTCACCGGCACCGAGGCGCTCCTTCAAGAACTTGGTCTCATGGGCTATCGGGCATCCGCCAAGAAGGCGCAGCTCTGCCAGCAGGAGGTGACCAGCGGCGGCAGCAAAAGAACGGCGGACGGCTCTGAGAAGCGCACCGCTGATAGCCAGCATTCAACTCCTCCGAAAACAAAGAGGAAAGTTGAGTTCGAACCGAAGAAGAAAAGGAAGGTGTGA

**Sequence 34 Plasmids sequence of ePPEplus-RT33**

(NLSSV40-nCas9(H840A/R221K/N394K)-XTEN-NC-NLS-32aa Linker-RT33-NLSvbp)

CCTAAGAAAAAGAGAAAAGTGGACAAGAAGTACTCGATCGGCCTCGATATTGGGACTAACTCTGTTGGCTGGGCCGTGATCACCGACGAGTACAAGGTGCCCTCAAAGAAGTTCAAGGTCCTGGGCAACACCGATCGGCATTCCATCAAGAAGAATCTCATTGGCGCTCTCCTGTTCGACAGCGGCGAGACGGCTGAGGCTACGCGGCTCAAGCGCACCGCCCGCAGGCGGTACACGCGCAGGAAGAATCGCATCTGCTACCTGCAGGAGATTTTCTCCAACGAGATGGCGAAGGTTGACGATTCTTTCTTCCACAGGCTGGAGGAGTCATTCCTCGTGGAGGAGGATAAGAAGCACGAGCGGCATCCAATCTTCGGCAACATTGTCGACGAGGTTGCCTACCACGAGAAGTACCCTACGATCTACCATCTGCGGAAGAAGCTCGTGGACTCCACAGATAAGGCGGACCTCCGCCTGATCTACCTCGCTCTGGCCCACATGATTAAGTTCAGGGGCCATTTCCTGATCGAGGGGGATCTCAACCCGGACAATAGCGATGTTGACAAGCTGTTCATCCAGCTCGTGCAGACGTACAACCAGCTCTTCGAGGAGAACCCCATTAATGCGTCAGGCGTCGACGCGAAGGCTATCCTGTCCGCTAGGCTCTCGAAGTCTCGGAAGCTCGAGAACCTGATCGCCCAGCTGCCGGGCGAGAAGAAGAACGGCCTGTTCGGGAATCTCATTGCGCTCAGCCTGGGGCTCACGCCCAACTTCAAGTCGAATTTCGATCTCGCTGAGGACGCCAAGCTGCAGCTCTCCAAGGACACATACGACGATGACCTGGATAACCTCCTGGCCCAGATCGGCGATCAGTACGCGGACCTGTTCCTCGCTGCCAAGAATCTGTCGGACGCCATCCTCCTGTCTGATATTCTCAGGGTGAACACCGAGATTACGAAGGCTCCGCTCTCAGCCTCCATGATCAAGCGCTACGACGAGCACCATCAGGATCTGACCCTCCTGAAGGCGCTGGTCAGGCAGCAGCTCCCCGAGAAGTACAAGGAGATCTTCTTCGATCAGTCGAAGAACGGCTACGCTGGGTACATTGACGGCGGGGCCTCTCAGGAGGAGTTCTACAAGTTCATCAAGCCGATTCTGGAGAAGATGGACGGCACGGAGGAGCTGCTGGTGAAGCTCAAGCGCGAGGACCTCCTGAGGAAGCAGCGGACATTCGATAACGGCAGCATCCCACACCAGATTCATCTCGGGGAGCTGCACGCTATCCTGAGGAGGCAGGAGGACTTCTACCCTTTCCTCAAGGATAACCGCGAGAAGATCGAGAAGATTCTGACTTTCAGGATCCCGTACTACGTCGGCCCACTCGCTAGGGGCAACTCCCGCTTCGCTTGGATGACCCGCAAGTCAGAGGAGACGATCACGCCGTGGAACTTCGAGGAGGTGGTCGACAAGGGCGCTAGCGCTCAGTCGTTCATCGAGAGGATGACGAATTTCGACAAGAACCTGCCAAATGAGAAGGTGCTCCCTAAGCACTCGCTCCTGTACGAGTACTTCACAGTCTACAACGAGCTGACTAAGGTGAAGTATGTGACCGAGGGCATGAGGAAGCCGGCTTTCCTGTCTGGGGAGCAGAAGAAGGCCATCGTGGACCTCCTGTTCAAGACCAACCGGAAGGTCACGGTTAAGCAGCTCAAGGAGGACTACTTCAAGAAGATTGAGTGCTTCGATTCGGTCGAGATCTCTGGCGTTGAGGACCGCTTCAACGCCTCCCTGGGGACCTACCACGATCTCCTGAAGATCATTAAGGATAAGGACTTCCTGGACAACGAGGAGAATGAGGATATCCTCGAGGACATTGTGCTGACACTCACTCTGTTCGAGGACCGGGAGATGATCGAGGAGCGCCTGAAGACTTACGCCCATCTCTTCGATGACAAGGTCATGAAGCAGCTCAAGAGGAGGAGGTACACCGGCTGGGGGAGGCTGAGCAGGAAGCTCATCAACGGCATTCGGGACAAGCAGTCCGGGAAGACGATCCTCGACTTCCTGAAGAGCGATGGCTTCGCGAACCGCAATTTCATGCAGCTGATTCACGATGACAGCCTCACATTCAAGGAGGATATCCAGAAGGCTCAGGTGAGCGGCCAGGGGGACTCGCTGCACGAGCATATCGCGAACCTCGCTGGCTCGCCAGCTATCAAGAAGGGGATTCTGCAGACCGTGAAGGTTGTGGACGAGCTGGTGAAGGTCATGGGCAGGCACAAGCCTGAGAACATCGTCATTGAGATGGCCCGGGAGAATCAGACCACGCAGAAGGGCCAGAAGAACTCACGCGAGAGGATGAAGAGGATCGAGGAGGGCATTAAGGAGCTGGGGTCCCAGATCCTCAAGGAGCACCCGGTGGAGAACACGCAGCTGCAGAATGAGAAGCTCTACCTGTACTACCTCCAGAATGGCCGCGATATGTATGTGGACCAGGAGCTGGATATTAACAGGCTCAGCGATTACGACGTCGATGCCATCGTTCCACAGTCATTCCTGAAGGATGACTCCATTGACAACAAGGTCCTCACCAGGTCGGACAAGAACCGGGGCAAGTCTGATAATGTTCCTTCAGAGGAGGTCGTTAAGAAGATGAAGAACTACTGGCGCCAGCTCCTGAATGCCAAGCTGATCACGCAGCGGAAGTTCGATAACCTCACAAAGGCTGAGAGGGGCGGGCTCTCTGAGCTGGACAAGGCGGGCTTCATCAAGAGGCAGCTGGTCGAGACACGGCAGATCACTAAGCACGTTGCGCAGATTCTCGACTCACGGATGAACACTAAGTACGATGAGAATGACAAGCTGATCCGCGAGGTGAAGGTCATCACCCTGAAGTCAAAGCTCGTCTCCGACTTCAGGAAGGATTTCCAGTTCTACAAGGTTCGGGAGATCAACAATTACCACCATGCCCATGACGCGTACCTGAACGCGGTGGTCGGCACAGCTCTGATCAAGAAGTACCCAAAGCTCGAGAGCGAGTTCGTGTACGGGGACTACAAGGTTTACGATGTGAGGAAGATGATCGCCAAGTCGGAGCAGGAGATTGGCAAGGCTACCGCCAAGTACTTCTTCTACTCTAACATTATGAATTTCTTCAAGACAGAGATCACTCTGGCCAATGGCGAGATCCGGAAGCGCCCCCTCATCGAGACGAACGGCGAGACGGGGGAGATCGTGTGGGACAAGGGCAGGGATTTCGCGACCGTCAGGAAGGTTCTCTCCATGCCACAAGTGAATATCGTCAAGAAGACAGAGGTCCAGACTGGCGGGTTCTCTAAGGAGTCAATTCTGCCTAAGCGGAACAGCGACAAGCTCATCGCCCGCAAGAAGGACTGGGATCCGAAGAAGTACGGCGGGTTCGACAGCCCCACTGTGGCCTACTCGGTCCTGGTTGTGGCGAAGGTTGAGAAGGGCAAGTCCAAGAAGCTCAAGAGCGTGAAGGAGCTGCTGGGGATCACGATTATGGAGCGCTCCAGCTTCGAGAAGAACCCGATCGATTTCCTGGAGGCGAAGGGCTACAAGGAGGTGAAGAAGGACCTGATCATTAAGCTCCCCAAGTACTCACTCTTCGAGCTGGAGAACGGCAGGAAGCGGATGCTGGCTTCCGCTGGCGAGCTGCAGAAGGGGAACGAGCTGGCTCTGCCGTCCAAGTATGTGAACTTCCTCTACCTGGCCTCCCACTACGAGAAGCTCAAGGGCAGCCCCGAGGACAACGAGCAGAAGCAGCTGTTCGTCGAGCAGCACAAGCATTACCTCGACGAGATCATTGAGCAGATTTCCGAGTTCTCCAAGCGCGTGATCCTGGCCGACGCGAATCTGGATAAGGTCCTCTCCGCGTACAACAAGCACCGCGACAAGCCAATCAGGGAGCAGGCTGAGAATATCATTCATCTCTTCACCCTGACGAACCTCGGCGCCCCTGCTGCTTTCAAGTACTTCGACACAACTATCGATCGCAAGAGGTACACAAGCACTAAGGAGGTCCTGGACGCGACCCTCATCCACCAGTCGATTACCGGCCTCTACGAGACGCGCATCGACCTGTCTCAGCTCGGGGGCGACGAATTCTCCGGGAGCGAGACGCCAGGCACCTCCGAGTCGGCCACCCCAGAATCTGCCACAGTGGTGTCCGGCCAAAAGCAGGACCGCCAGGGCGGAGAACGCAGAAGGTCCCAGCTCGATAGGGATCAGTGTGCCTACTGCAAGGAGAAGGGCCACTGGGCCAAAGACTGCCCGAAAAAGCCGCGCGGCCCACGCGGCCCAAGGCCACAAACATCCCTCCTTCCAAAGAAGAAGCGGAAGGTGGAGCTCAGCGGAGGATCTTCCGGAGGATCTAGCGGCTCCGAGACACCAGGAACATCCGAAAGCGCTACACCAGAATCTAGCGGAGGCTCTTCCGGAGGATCTAGGCCTATGCATGAGCCGACACTTCTTTCACAAATCATCCATCCTGCGAATTTGAATCAAGCATACAAACAAGTAATGAAGAACAAGGGCGCTCCTGGAATTGATGATATGCCAATTACGGCGCTGAAAGCACATTTGGCGCTCCACAAGGAGACTCTTGTGCACCAGCTGCAGCGACGGGAATACAAGCCACAACCAGTGAAGAGGGTGGAGATACCAAAGGCATCTGGAGGGGTGCGCCTCCTCGGCATACCCACAGTCACCGACCGCTTCATCCAGCAGGCCATCGCACAGGTGCTCACTCCTATATTTGACAAGCAGTTCCATGATAATAGCTATGGTTTCCGCCCCAAGAGATATGCTGAGATGGCGATACTTCAAGCTTTGGAGAACATGAATGAAGGATATGGCTGGCTGGTGGACATCGACCTGGAGAGGTTCTTCGACACCGTCCACCACGACAGGCTCATGAACATTGTAGCCAGAACAGTTACTGATGGCGACGTCATTTCACTCGTCAGGAAGTTTCTAGTTTCTGGTGTGATGGTGCAGGATGAGTACCAGGAGACAATCATTGGGACGCCGCAGGGCGGCAACCTGTCGCCGCTGCTGTCCAACATCATGCTGCATGAATTGGATAAAGAGCTCGCCAACAGAGATTTGAGATTTGTTCGTTACGCCGACGACTGCCTCATTTTCGTCAAGAGCGACATGGCCGCGCGCCGCGTCATGAGGAGTGTTTCTAAATTCATTGAGGAGAAGCTCGGATTAATAGTTAATGTTACCAAGTCCAAGGTGCGGCGGCCGGAAGATGAAGAAACAAAATTTCTTGGCTTCGGGTTCTACTTTGATTGGAATGATCGGAGCGGCGGCAGCAAAAGAACGGCGGACGGCTCTGAGAAGCGCACCGCTGATAGCCAGCATTCAACTCCTCCGAAAACAAAGAGGAAAGTTGAGTTCGAACCGAAGAAGAAAAGGAAGGTGTGA

**Sequence 35 Plasmids sequence of ePPEplus-RT34**

(NLSSV40-nCas9(H840A/R221K/N394K)-XTEN-NC-NLS-32aa Linker-RT34-NLSvbp)

CCTAAGAAAAAGAGAAAAGTGGACAAGAAGTACTCGATCGGCCTCGATATTGGGACTAACTCTGTTGGCTGGGCCGTGATCACCGACGAGTACAAGGTGCCCTCAAAGAAGTTCAAGGTCCTGGGCAACACCGATCGGCATTCCATCAAGAAGAATCTCATTGGCGCTCTCCTGTTCGACAGCGGCGAGACGGCTGAGGCTACGCGGCTCAAGCGCACCGCCCGCAGGCGGTACACGCGCAGGAAGAATCGCATCTGCTACCTGCAGGAGATTTTCTCCAACGAGATGGCGAAGGTTGACGATTCTTTCTTCCACAGGCTGGAGGAGTCATTCCTCGTGGAGGAGGATAAGAAGCACGAGCGGCATCCAATCTTCGGCAACATTGTCGACGAGGTTGCCTACCACGAGAAGTACCCTACGATCTACCATCTGCGGAAGAAGCTCGTGGACTCCACAGATAAGGCGGACCTCCGCCTGATCTACCTCGCTCTGGCCCACATGATTAAGTTCAGGGGCCATTTCCTGATCGAGGGGGATCTCAACCCGGACAATAGCGATGTTGACAAGCTGTTCATCCAGCTCGTGCAGACGTACAACCAGCTCTTCGAGGAGAACCCCATTAATGCGTCAGGCGTCGACGCGAAGGCTATCCTGTCCGCTAGGCTCTCGAAGTCTCGGAAGCTCGAGAACCTGATCGCCCAGCTGCCGGGCGAGAAGAAGAACGGCCTGTTCGGGAATCTCATTGCGCTCAGCCTGGGGCTCACGCCCAACTTCAAGTCGAATTTCGATCTCGCTGAGGACGCCAAGCTGCAGCTCTCCAAGGACACATACGACGATGACCTGGATAACCTCCTGGCCCAGATCGGCGATCAGTACGCGGACCTGTTCCTCGCTGCCAAGAATCTGTCGGACGCCATCCTCCTGTCTGATATTCTCAGGGTGAACACCGAGATTACGAAGGCTCCGCTCTCAGCCTCCATGATCAAGCGCTACGACGAGCACCATCAGGATCTGACCCTCCTGAAGGCGCTGGTCAGGCAGCAGCTCCCCGAGAAGTACAAGGAGATCTTCTTCGATCAGTCGAAGAACGGCTACGCTGGGTACATTGACGGCGGGGCCTCTCAGGAGGAGTTCTACAAGTTCATCAAGCCGATTCTGGAGAAGATGGACGGCACGGAGGAGCTGCTGGTGAAGCTCAAGCGCGAGGACCTCCTGAGGAAGCAGCGGACATTCGATAACGGCAGCATCCCACACCAGATTCATCTCGGGGAGCTGCACGCTATCCTGAGGAGGCAGGAGGACTTCTACCCTTTCCTCAAGGATAACCGCGAGAAGATCGAGAAGATTCTGACTTTCAGGATCCCGTACTACGTCGGCCCACTCGCTAGGGGCAACTCCCGCTTCGCTTGGATGACCCGCAAGTCAGAGGAGACGATCACGCCGTGGAACTTCGAGGAGGTGGTCGACAAGGGCGCTAGCGCTCAGTCGTTCATCGAGAGGATGACGAATTTCGACAAGAACCTGCCAAATGAGAAGGTGCTCCCTAAGCACTCGCTCCTGTACGAGTACTTCACAGTCTACAACGAGCTGACTAAGGTGAAGTATGTGACCGAGGGCATGAGGAAGCCGGCTTTCCTGTCTGGGGAGCAGAAGAAGGCCATCGTGGACCTCCTGTTCAAGACCAACCGGAAGGTCACGGTTAAGCAGCTCAAGGAGGACTACTTCAAGAAGATTGAGTGCTTCGATTCGGTCGAGATCTCTGGCGTTGAGGACCGCTTCAACGCCTCCCTGGGGACCTACCACGATCTCCTGAAGATCATTAAGGATAAGGACTTCCTGGACAACGAGGAGAATGAGGATATCCTCGAGGACATTGTGCTGACACTCACTCTGTTCGAGGACCGGGAGATGATCGAGGAGCGCCTGAAGACTTACGCCCATCTCTTCGATGACAAGGTCATGAAGCAGCTCAAGAGGAGGAGGTACACCGGCTGGGGGAGGCTGAGCAGGAAGCTCATCAACGGCATTCGGGACAAGCAGTCCGGGAAGACGATCCTCGACTTCCTGAAGAGCGATGGCTTCGCGAACCGCAATTTCATGCAGCTGATTCACGATGACAGCCTCACATTCAAGGAGGATATCCAGAAGGCTCAGGTGAGCGGCCAGGGGGACTCGCTGCACGAGCATATCGCGAACCTCGCTGGCTCGCCAGCTATCAAGAAGGGGATTCTGCAGACCGTGAAGGTTGTGGACGAGCTGGTGAAGGTCATGGGCAGGCACAAGCCTGAGAACATCGTCATTGAGATGGCCCGGGAGAATCAGACCACGCAGAAGGGCCAGAAGAACTCACGCGAGAGGATGAAGAGGATCGAGGAGGGCATTAAGGAGCTGGGGTCCCAGATCCTCAAGGAGCACCCGGTGGAGAACACGCAGCTGCAGAATGAGAAGCTCTACCTGTACTACCTCCAGAATGGCCGCGATATGTATGTGGACCAGGAGCTGGATATTAACAGGCTCAGCGATTACGACGTCGATGCCATCGTTCCACAGTCATTCCTGAAGGATGACTCCATTGACAACAAGGTCCTCACCAGGTCGGACAAGAACCGGGGCAAGTCTGATAATGTTCCTTCAGAGGAGGTCGTTAAGAAGATGAAGAACTACTGGCGCCAGCTCCTGAATGCCAAGCTGATCACGCAGCGGAAGTTCGATAACCTCACAAAGGCTGAGAGGGGCGGGCTCTCTGAGCTGGACAAGGCGGGCTTCATCAAGAGGCAGCTGGTCGAGACACGGCAGATCACTAAGCACGTTGCGCAGATTCTCGACTCACGGATGAACACTAAGTACGATGAGAATGACAAGCTGATCCGCGAGGTGAAGGTCATCACCCTGAAGTCAAAGCTCGTCTCCGACTTCAGGAAGGATTTCCAGTTCTACAAGGTTCGGGAGATCAACAATTACCACCATGCCCATGACGCGTACCTGAACGCGGTGGTCGGCACAGCTCTGATCAAGAAGTACCCAAAGCTCGAGAGCGAGTTCGTGTACGGGGACTACAAGGTTTACGATGTGAGGAAGATGATCGCCAAGTCGGAGCAGGAGATTGGCAAGGCTACCGCCAAGTACTTCTTCTACTCTAACATTATGAATTTCTTCAAGACAGAGATCACTCTGGCCAATGGCGAGATCCGGAAGCGCCCCCTCATCGAGACGAACGGCGAGACGGGGGAGATCGTGTGGGACAAGGGCAGGGATTTCGCGACCGTCAGGAAGGTTCTCTCCATGCCACAAGTGAATATCGTCAAGAAGACAGAGGTCCAGACTGGCGGGTTCTCTAAGGAGTCAATTCTGCCTAAGCGGAACAGCGACAAGCTCATCGCCCGCAAGAAGGACTGGGATCCGAAGAAGTACGGCGGGTTCGACAGCCCCACTGTGGCCTACTCGGTCCTGGTTGTGGCGAAGGTTGAGAAGGGCAAGTCCAAGAAGCTCAAGAGCGTGAAGGAGCTGCTGGGGATCACGATTATGGAGCGCTCCAGCTTCGAGAAGAACCCGATCGATTTCCTGGAGGCGAAGGGCTACAAGGAGGTGAAGAAGGACCTGATCATTAAGCTCCCCAAGTACTCACTCTTCGAGCTGGAGAACGGCAGGAAGCGGATGCTGGCTTCCGCTGGCGAGCTGCAGAAGGGGAACGAGCTGGCTCTGCCGTCCAAGTATGTGAACTTCCTCTACCTGGCCTCCCACTACGAGAAGCTCAAGGGCAGCCCCGAGGACAACGAGCAGAAGCAGCTGTTCGTCGAGCAGCACAAGCATTACCTCGACGAGATCATTGAGCAGATTTCCGAGTTCTCCAAGCGCGTGATCCTGGCCGACGCGAATCTGGATAAGGTCCTCTCCGCGTACAACAAGCACCGCGACAAGCCAATCAGGGAGCAGGCTGAGAATATCATTCATCTCTTCACCCTGACGAACCTCGGCGCCCCTGCTGCTTTCAAGTACTTCGACACAACTATCGATCGCAAGAGGTACACAAGCACTAAGGAGGTCCTGGACGCGACCCTCATCCACCAGTCGATTACCGGCCTCTACGAGACGCGCATCGACCTGTCTCAGCTCGGGGGCGACGAATTCTCCGGGAGCGAGACGCCAGGCACCTCCGAGTCGGCCACCCCAGAATCTGCCACAGTGGTGTCCGGCCAAAAGCAGGACCGCCAGGGCGGAGAACGCAGAAGGTCCCAGCTCGATAGGGATCAGTGTGCCTACTGCAAGGAGAAGGGCCACTGGGCCAAAGACTGCCCGAAAAAGCCGCGCGGCCCACGCGGCCCAAGGCCACAAACATCCCTCCTTCCAAAGAAGAAGCGGAAGGTGGAGCTCAGCGGAGGATCTTCCGGAGGATCTAGCGGCTCCGAGACACCAGGAACATCCGAAAGCGCTACACCAGAATCTAGCGGAGGCTCTTCCGGAGGATCTAGGCCTCCTGGCGCCACCGTATGTCAGCTGAGTGCCCAGTACGCGATACCTCTTCGGTGGAAGCCGGACACAAGACCTGTATGGGTGGATCAATGGCCACTCCCAAAGGAGAAGCTCGACGCGCTGCAGACGCTTGTTGCTAGAGAACTGCGGCTGGGCCATATCGAACCATCCCTCAGCAGATGGAACACTCCTGTATTCGTCATCCAAAAGAAATCTGGAGCTTTTCGGCTGCTGCATGATTTAAGAGCTGTCAATTCACAGCTGATACCTTTCGGGGTGGTGCAGCAAGGAGCCCCAGTACTTTCCGCCGTCCCCGAGGAGTGGGAGGTAACCGCTATCGATCTGAAGGATTGCTTCTTCTCCATCCCCCTCGCGGAGCAGGATCGGGAGGCGTTCGCGTTCACCGTCCCAGTGAGCAACAACCAGAGACCGACCCAGCGATACCAGTGGCGTGTCCTGCCTCAGGGCATGGCGTGTTCCCCTACCATCTGCCAGATGGTTGTGGGCAAGATCCTTGGACCTCTCCACCACACTTCAGAGGCCAGCGAATGTATAATCCTTCACTACATGGATGATTTACTGCTGGCCGCGCCTACACTCGCCCGTCTGCAAGACCTGGAGACCTGCGTGATCTCCCTGCTGACGAAGGCAGGATTTACAGTGAGCTCCGAGAAAATTCAGCGCGGCAGCGGCGTCCAATTCCTGGGCTACAAGTTTGACGAAGGAACCGTCCGTCCCACCGGAGTGAATATCACTCCTCGCATCAGGACCCTCTGGGATGTGCAGAAGCTGGTGGGGGCCCTCCAGTGGATTCGAGGGGCGCTGGGCATCCCTCCAAGACTCATGCAACCCTTCTATGATCAGTTGAAGGGTTCCGACCCCAGAGAACCCCGGACGTTCACCCCCGACATGTCAGAGGCGTGGGACGAGATTGTCACGAGCTGCCTCCACACGGCCCTGTCGCAATACAACTGCAAGAAGGAACTTGAAGTAGCTGTGTGCAAATGTGATTCTGGCGCTACCGGTTTGCTGGGCCAAGACCTGGGCGCCAAGCCACAGCCGCTGTGGTGGAATTATTCTGTCCAGCCAGTCCACGCGTTCACCAGCTGGCTAGAAGTGTTAGCCAAGCTGATCTGTAAGTGCCGGCTCTCCGCCGTGAGGATTTTCGGCCAGGAGCCATCTGTGATATACTTGCCCAGAAGTTTCAGAGACACGTCTCCTCTTCCGGAGCCTATTCTGCTCGCCTTAGTTGGGAGCTCGGCTACCGTGACATTTACGGACTCCCTGTCGATATTTGAGCTGGCGCGGCCAATGCAGCTCAGTCTTCACTGCCGGGTGCAGGCCAGCCCCGTCGATGGCCCTACACTATTCACAGATGCGAGCAGCTCTACGGGCCAGGGCGCCGTAGTTTGGGGCAGAGGTTCCTGCTGGAAATCCGTTATATTCCAGGATGTTACTGCCTCGGTGCAGATCCTGGAGGTGCGCGCAGTGGCATTGGCCCTGCGCCTCTGGCCGACAATCCCCTGCAACATTGTGACCGACAGCGCATTTGCCGCTAAATTGCTGCTGTCGATGGGACAGGAAGGTATCTCATCCACAGAGGCCGCCATGGTCCTCGAGGATTCGCTCTGCACAAGAACCGCTCCGGTGGCCGTGATGCATGTGCGGTCTCATTCGGAGGTGCCTGGATTTTTTTCCGAAGGCAACGCGGCCGCGGACAGAGCGGCCGGCACCCACGTCTTCACCCTTAAGACGGCCAGGGAGTTGCACAGCACCCTGCACATCGGAGCCCGGGCTCTGAGCAGAACTTGCGGCATCCCCAGCGGCGGCAGCAAAAGAACGGCGGACGGCTCTGAGAAGCGCACCGCTGATAGCCAGCATTCAACTCCTCCGAAAACAAAGAGGAAAGTTGAGTTCGAACCGAAGAAGAAAAGGAAGGTGTGA

**Sequence 36 Plasmids sequence of ePPEplus-RT35**

(NLSSV40-nCas9(H840A/R221K/N394K)-XTEN-NC-NLS-32aa Linker-RT35-Linker-NLSvbp)

CCTAAGAAAAAGAGAAAAGTGGACAAGAAGTACTCGATCGGCCTCGATATTGGGACTAACTCTGTTGGCTGGGCCGTGATCACCGACGAGTACAAGGTGCCCTCAAAGAAGTTCAAGGTCCTGGGCAACACCGATCGGCATTCCATCAAGAAGAATCTCATTGGCGCTCTCCTGTTCGACAGCGGCGAGACGGCTGAGGCTACGCGGCTCAAGCGCACCGCCCGCAGGCGGTACACGCGCAGGAAGAATCGCATCTGCTACCTGCAGGAGATTTTCTCCAACGAGATGGCGAAGGTTGACGATTCTTTCTTCCACAGGCTGGAGGAGTCATTCCTCGTGGAGGAGGATAAGAAGCACGAGCGGCATCCAATCTTCGGCAACATTGTCGACGAGGTTGCCTACCACGAGAAGTACCCTACGATCTACCATCTGCGGAAGAAGCTCGTGGACTCCACAGATAAGGCGGACCTCCGCCTGATCTACCTCGCTCTGGCCCACATGATTAAGTTCAGGGGCCATTTCCTGATCGAGGGGGATCTCAACCCGGACAATAGCGATGTTGACAAGCTGTTCATCCAGCTCGTGCAGACGTACAACCAGCTCTTCGAGGAGAACCCCATTAATGCGTCAGGCGTCGACGCGAAGGCTATCCTGTCCGCTAGGCTCTCGAAGTCTCGGAAGCTCGAGAACCTGATCGCCCAGCTGCCGGGCGAGAAGAAGAACGGCCTGTTCGGGAATCTCATTGCGCTCAGCCTGGGGCTCACGCCCAACTTCAAGTCGAATTTCGATCTCGCTGAGGACGCCAAGCTGCAGCTCTCCAAGGACACATACGACGATGACCTGGATAACCTCCTGGCCCAGATCGGCGATCAGTACGCGGACCTGTTCCTCGCTGCCAAGAATCTGTCGGACGCCATCCTCCTGTCTGATATTCTCAGGGTGAACACCGAGATTACGAAGGCTCCGCTCTCAGCCTCCATGATCAAGCGCTACGACGAGCACCATCAGGATCTGACCCTCCTGAAGGCGCTGGTCAGGCAGCAGCTCCCCGAGAAGTACAAGGAGATCTTCTTCGATCAGTCGAAGAACGGCTACGCTGGGTACATTGACGGCGGGGCCTCTCAGGAGGAGTTCTACAAGTTCATCAAGCCGATTCTGGAGAAGATGGACGGCACGGAGGAGCTGCTGGTGAAGCTCAAGCGCGAGGACCTCCTGAGGAAGCAGCGGACATTCGATAACGGCAGCATCCCACACCAGATTCATCTCGGGGAGCTGCACGCTATCCTGAGGAGGCAGGAGGACTTCTACCCTTTCCTCAAGGATAACCGCGAGAAGATCGAGAAGATTCTGACTTTCAGGATCCCGTACTACGTCGGCCCACTCGCTAGGGGCAACTCCCGCTTCGCTTGGATGACCCGCAAGTCAGAGGAGACGATCACGCCGTGGAACTTCGAGGAGGTGGTCGACAAGGGCGCTAGCGCTCAGTCGTTCATCGAGAGGATGACGAATTTCGACAAGAACCTGCCAAATGAGAAGGTGCTCCCTAAGCACTCGCTCCTGTACGAGTACTTCACAGTCTACAACGAGCTGACTAAGGTGAAGTATGTGACCGAGGGCATGAGGAAGCCGGCTTTCCTGTCTGGGGAGCAGAAGAAGGCCATCGTGGACCTCCTGTTCAAGACCAACCGGAAGGTCACGGTTAAGCAGCTCAAGGAGGACTACTTCAAGAAGATTGAGTGCTTCGATTCGGTCGAGATCTCTGGCGTTGAGGACCGCTTCAACGCCTCCCTGGGGACCTACCACGATCTCCTGAAGATCATTAAGGATAAGGACTTCCTGGACAACGAGGAGAATGAGGATATCCTCGAGGACATTGTGCTGACACTCACTCTGTTCGAGGACCGGGAGATGATCGAGGAGCGCCTGAAGACTTACGCCCATCTCTTCGATGACAAGGTCATGAAGCAGCTCAAGAGGAGGAGGTACACCGGCTGGGGGAGGCTGAGCAGGAAGCTCATCAACGGCATTCGGGACAAGCAGTCCGGGAAGACGATCCTCGACTTCCTGAAGAGCGATGGCTTCGCGAACCGCAATTTCATGCAGCTGATTCACGATGACAGCCTCACATTCAAGGAGGATATCCAGAAGGCTCAGGTGAGCGGCCAGGGGGACTCGCTGCACGAGCATATCGCGAACCTCGCTGGCTCGCCAGCTATCAAGAAGGGGATTCTGCAGACCGTGAAGGTTGTGGACGAGCTGGTGAAGGTCATGGGCAGGCACAAGCCTGAGAACATCGTCATTGAGATGGCCCGGGAGAATCAGACCACGCAGAAGGGCCAGAAGAACTCACGCGAGAGGATGAAGAGGATCGAGGAGGGCATTAAGGAGCTGGGGTCCCAGATCCTCAAGGAGCACCCGGTGGAGAACACGCAGCTGCAGAATGAGAAGCTCTACCTGTACTACCTCCAGAATGGCCGCGATATGTATGTGGACCAGGAGCTGGATATTAACAGGCTCAGCGATTACGACGTCGATGCCATCGTTCCACAGTCATTCCTGAAGGATGACTCCATTGACAACAAGGTCCTCACCAGGTCGGACAAGAACCGGGGCAAGTCTGATAATGTTCCTTCAGAGGAGGTCGTTAAGAAGATGAAGAACTACTGGCGCCAGCTCCTGAATGCCAAGCTGATCACGCAGCGGAAGTTCGATAACCTCACAAAGGCTGAGAGGGGCGGGCTCTCTGAGCTGGACAAGGCGGGCTTCATCAAGAGGCAGCTGGTCGAGACACGGCAGATCACTAAGCACGTTGCGCAGATTCTCGACTCACGGATGAACACTAAGTACGATGAGAATGACAAGCTGATCCGCGAGGTGAAGGTCATCACCCTGAAGTCAAAGCTCGTCTCCGACTTCAGGAAGGATTTCCAGTTCTACAAGGTTCGGGAGATCAACAATTACCACCATGCCCATGACGCGTACCTGAACGCGGTGGTCGGCACAGCTCTGATCAAGAAGTACCCAAAGCTCGAGAGCGAGTTCGTGTACGGGGACTACAAGGTTTACGATGTGAGGAAGATGATCGCCAAGTCGGAGCAGGAGATTGGCAAGGCTACCGCCAAGTACTTCTTCTACTCTAACATTATGAATTTCTTCAAGACAGAGATCACTCTGGCCAATGGCGAGATCCGGAAGCGCCCCCTCATCGAGACGAACGGCGAGACGGGGGAGATCGTGTGGGACAAGGGCAGGGATTTCGCGACCGTCAGGAAGGTTCTCTCCATGCCACAAGTGAATATCGTCAAGAAGACAGAGGTCCAGACTGGCGGGTTCTCTAAGGAGTCAATTCTGCCTAAGCGGAACAGCGACAAGCTCATCGCCCGCAAGAAGGACTGGGATCCGAAGAAGTACGGCGGGTTCGACAGCCCCACTGTGGCCTACTCGGTCCTGGTTGTGGCGAAGGTTGAGAAGGGCAAGTCCAAGAAGCTCAAGAGCGTGAAGGAGCTGCTGGGGATCACGATTATGGAGCGCTCCAGCTTCGAGAAGAACCCGATCGATTTCCTGGAGGCGAAGGGCTACAAGGAGGTGAAGAAGGACCTGATCATTAAGCTCCCCAAGTACTCACTCTTCGAGCTGGAGAACGGCAGGAAGCGGATGCTGGCTTCCGCTGGCGAGCTGCAGAAGGGGAACGAGCTGGCTCTGCCGTCCAAGTATGTGAACTTCCTCTACCTGGCCTCCCACTACGAGAAGCTCAAGGGCAGCCCCGAGGACAACGAGCAGAAGCAGCTGTTCGTCGAGCAGCACAAGCATTACCTCGACGAGATCATTGAGCAGATTTCCGAGTTCTCCAAGCGCGTGATCCTGGCCGACGCGAATCTGGATAAGGTCCTCTCCGCGTACAACAAGCACCGCGACAAGCCAATCAGGGAGCAGGCTGAGAATATCATTCATCTCTTCACCCTGACGAACCTCGGCGCCCCTGCTGCTTTCAAGTACTTCGACACAACTATCGATCGCAAGAGGTACACAAGCACTAAGGAGGTCCTGGACGCGACCCTCATCCACCAGTCGATTACCGGCCTCTACGAGACGCGCATCGACCTGTCTCAGCTCGGGGGCGACGAATTCTCCGGGAGCGAGACGCCAGGCACCTCCGAGTCGGCCACCCCAGAATCTGCCACAGTGGTGTCCGGCCAAAAGCAGGACCGCCAGGGCGGAGAACGCAGAAGGTCCCAGCTCGATAGGGATCAGTGTGCCTACTGCAAGGAGAAGGGCCACTGGGCCAAAGACTGCCCGAAAAAGCCGCGCGGCCCACGCGGCCCAAGGCCACAAACATCCCTCCTTCCAAAGAAGAAGCGGAAGGTGGAGCTCAGCGGAGGATCTTCCGGAGGATCTAGCGGCTCCGAGACACCAGGAACATCCGAAAGCGCTACACCAGAATCTAGCGGAGGCTCTTCCGGAGGATCTAGGCCTCCTATCCCTTCCCTGCCCTTTTCCCTGTGGGGCAGAGACATTATGGAGTACATGAGCGTGCGGCTGATCACGGACGCCCGGAAATTCTTTCTGGGCGCCATCATGAGCAGCTTCTACGCGAAGAAGATCTCGTGGAAGAATGATGAGCCTGTTTGGCTGAACCAGTGGCCCCTGACGACCGAGAAGATCCAAGCTATCGAAGAAATCATTCAGGAACAGCTGCAGGCCGGCCATCTTGAAGAATCGTTCTCCCCTTGGAACACTCCTATATTCGTCATCAGAAAGAAGAGCGGGAAGTGGAGGCTCTTCCAGGATCTTCGCGCGGTGAATGCCACCATGTACGACATGGGTGCGCTGCAGCCCGGCTTGCCTTCACCGGTCGCCGTGCCGCAGGGATGGAAAGTGATCTTTATCGACCTGCAGGATTGTTTCTTCACCATCCAGCTGCACCCCGATGACTGCCAGCGCTTCGCCTTCTCCGTTCCTAGCGTGAATTACAAGGAGCCTTTCAAGAGATACCAATGGAGGGTGCTGCCTCAAGGAATGAAGAACAGCCCAACCCTGTGCCAATATTTCGTCGCCAAGGCCATCGAGCAGCTAAGAAAGGATCATTCCGACGCCTACCTGGTCCATTACATGGATGATATACTCATCGCTCACCCTGACATTCAGGAGTTGGATAAGCTGGTGGTGAAGCTGATCAATAATCTGAAGGACCATGGCCTAGTAGTGGCCCCGGAAAAGGTACAGCAGAAATGGCCAATCAATTACCTGGGCCATACAATGCAAGAAGACTACGTGAGCACACAAAAGATTGAAATAAGACGGGATAAGATGAGGACACTGAACGATTTTCAGAAGGTCCTGGGCAACATCAATTGGATCCGACCATACTTGAAGATTACCACAGGAGACCTCCGTCCTCTGTTCATGACATTGAACGGGGACCCCGACCCAAAGTCCGCCCGCATTCTGACCTCTAAGGCTTGTGAGGCGCTGCAGCTCGTGGAGGAGAAACTGACCGAGGCCAAGGTCATCAGAATTGATTACAACAAACCTCTCAGCTTACTCATAATCAGAACCGAATATACCCCGACCGGTTGCCTCTGGCAGGACGGCGTACTGGAGTGGATCCACCTCCCGCACACCAACTCTAGAATTATCTCTACCTATGATATCCTCTGCGCTGCCCTAATTGTCAAGGCCCGGAACAGAAGCAAGGAAATCTTTGGCCGGGATATGCAGGAGATCGTTGTGCCATACACACGCCAGCAATTTGAGCACCTCCTGCAGGAATCCTATGACTGGGGCTTGGCCCTGATCGGATACCTTGGCATCATCAAGTTCCACCTGCCCAACATCCAAAGCCTGATCCATAAAAGAACACCTATTATCTTTCCTTCTAACTGGAGCACTGAGCCTATCGAAAAAGCAAAGGTGATTTTCACCGACGGCAGCAGCAAAGGAAACTCTGTGATGATTGTGCAGGGCGAACCCCCCATCATCAAGTATGAGAAGGGCCTGAGCGCTCAGAAGGCAGAGATCATTGCCGTCATCCTCGCGTTTGAAAAGGTGAAGGAGCCGTTCAACCTCTACACAGACTCTCGGTACGTTGCTGGGCTGTTCCCTGCAATCGAGACCGCCATGATCAGTATAAGGACCGAAATCAACGACTTACTGTCCAAGCTGAAGGAGCTGGCGCAGAAAAGGAAATTTAAGTACCATGTGGCGCACATAAGAGGCCACACCAAGCTGCCAGAGCCTCTGGCCCAAGGGAACGCCATCGCCGACTCGTACACGCAGGTGTTCATCGCTCTGGAGAATGCAATGGCGTCCCACAAGCTCCACCACCAGAATGCCAGCGGCGGCAGCAAAAGAACGGCGGACGGCTCTGAGAAGCGCACCGCTGATAGCCAGCATTCAACTCCTCCGAAAACAAAGAGGAAAGTTGAGTTCGAACCGAAGAAGAAAAGGAAGGTGTGA

**Sequence 37 Plasmids sequence of ePPEplus-RT36**

(NLSSV40-nCas9(H840A/R221K/N394K)-XTEN-NC-NLS-32aa Linker-RT36-NLSvbp)

CCTAAGAAAAAGAGAAAAGTGGACAAGAAGTACTCGATCGGCCTCGATATTGGGACTAACTCTGTTGGCTGGGCCGTGATCACCGACGAGTACAAGGTGCCCTCAAAGAAGTTCAAGGTCCTGGGCAACACCGATCGGCATTCCATCAAGAAGAATCTCATTGGCGCTCTCCTGTTCGACAGCGGCGAGACGGCTGAGGCTACGCGGCTCAAGCGCACCGCCCGCAGGCGGTACACGCGCAGGAAGAATCGCATCTGCTACCTGCAGGAGATTTTCTCCAACGAGATGGCGAAGGTTGACGATTCTTTCTTCCACAGGCTGGAGGAGTCATTCCTCGTGGAGGAGGATAAGAAGCACGAGCGGCATCCAATCTTCGGCAACATTGTCGACGAGGTTGCCTACCACGAGAAGTACCCTACGATCTACCATCTGCGGAAGAAGCTCGTGGACTCCACAGATAAGGCGGACCTCCGCCTGATCTACCTCGCTCTGGCCCACATGATTAAGTTCAGGGGCCATTTCCTGATCGAGGGGGATCTCAACCCGGACAATAGCGATGTTGACAAGCTGTTCATCCAGCTCGTGCAGACGTACAACCAGCTCTTCGAGGAGAACCCCATTAATGCGTCAGGCGTCGACGCGAAGGCTATCCTGTCCGCTAGGCTCTCGAAGTCTCGGAAGCTCGAGAACCTGATCGCCCAGCTGCCGGGCGAGAAGAAGAACGGCCTGTTCGGGAATCTCATTGCGCTCAGCCTGGGGCTCACGCCCAACTTCAAGTCGAATTTCGATCTCGCTGAGGACGCCAAGCTGCAGCTCTCCAAGGACACATACGACGATGACCTGGATAACCTCCTGGCCCAGATCGGCGATCAGTACGCGGACCTGTTCCTCGCTGCCAAGAATCTGTCGGACGCCATCCTCCTGTCTGATATTCTCAGGGTGAACACCGAGATTACGAAGGCTCCGCTCTCAGCCTCCATGATCAAGCGCTACGACGAGCACCATCAGGATCTGACCCTCCTGAAGGCGCTGGTCAGGCAGCAGCTCCCCGAGAAGTACAAGGAGATCTTCTTCGATCAGTCGAAGAACGGCTACGCTGGGTACATTGACGGCGGGGCCTCTCAGGAGGAGTTCTACAAGTTCATCAAGCCGATTCTGGAGAAGATGGACGGCACGGAGGAGCTGCTGGTGAAGCTCAAGCGCGAGGACCTCCTGAGGAAGCAGCGGACATTCGATAACGGCAGCATCCCACACCAGATTCATCTCGGGGAGCTGCACGCTATCCTGAGGAGGCAGGAGGACTTCTACCCTTTCCTCAAGGATAACCGCGAGAAGATCGAGAAGATTCTGACTTTCAGGATCCCGTACTACGTCGGCCCACTCGCTAGGGGCAACTCCCGCTTCGCTTGGATGACCCGCAAGTCAGAGGAGACGATCACGCCGTGGAACTTCGAGGAGGTGGTCGACAAGGGCGCTAGCGCTCAGTCGTTCATCGAGAGGATGACGAATTTCGACAAGAACCTGCCAAATGAGAAGGTGCTCCCTAAGCACTCGCTCCTGTACGAGTACTTCACAGTCTACAACGAGCTGACTAAGGTGAAGTATGTGACCGAGGGCATGAGGAAGCCGGCTTTCCTGTCTGGGGAGCAGAAGAAGGCCATCGTGGACCTCCTGTTCAAGACCAACCGGAAGGTCACGGTTAAGCAGCTCAAGGAGGACTACTTCAAGAAGATTGAGTGCTTCGATTCGGTCGAGATCTCTGGCGTTGAGGACCGCTTCAACGCCTCCCTGGGGACCTACCACGATCTCCTGAAGATCATTAAGGATAAGGACTTCCTGGACAACGAGGAGAATGAGGATATCCTCGAGGACATTGTGCTGACACTCACTCTGTTCGAGGACCGGGAGATGATCGAGGAGCGCCTGAAGACTTACGCCCATCTCTTCGATGACAAGGTCATGAAGCAGCTCAAGAGGAGGAGGTACACCGGCTGGGGGAGGCTGAGCAGGAAGCTCATCAACGGCATTCGGGACAAGCAGTCCGGGAAGACGATCCTCGACTTCCTGAAGAGCGATGGCTTCGCGAACCGCAATTTCATGCAGCTGATTCACGATGACAGCCTCACATTCAAGGAGGATATCCAGAAGGCTCAGGTGAGCGGCCAGGGGGACTCGCTGCACGAGCATATCGCGAACCTCGCTGGCTCGCCAGCTATCAAGAAGGGGATTCTGCAGACCGTGAAGGTTGTGGACGAGCTGGTGAAGGTCATGGGCAGGCACAAGCCTGAGAACATCGTCATTGAGATGGCCCGGGAGAATCAGACCACGCAGAAGGGCCAGAAGAACTCACGCGAGAGGATGAAGAGGATCGAGGAGGGCATTAAGGAGCTGGGGTCCCAGATCCTCAAGGAGCACCCGGTGGAGAACACGCAGCTGCAGAATGAGAAGCTCTACCTGTACTACCTCCAGAATGGCCGCGATATGTATGTGGACCAGGAGCTGGATATTAACAGGCTCAGCGATTACGACGTCGATGCCATCGTTCCACAGTCATTCCTGAAGGATGACTCCATTGACAACAAGGTCCTCACCAGGTCGGACAAGAACCGGGGCAAGTCTGATAATGTTCCTTCAGAGGAGGTCGTTAAGAAGATGAAGAACTACTGGCGCCAGCTCCTGAATGCCAAGCTGATCACGCAGCGGAAGTTCGATAACCTCACAAAGGCTGAGAGGGGCGGGCTCTCTGAGCTGGACAAGGCGGGCTTCATCAAGAGGCAGCTGGTCGAGACACGGCAGATCACTAAGCACGTTGCGCAGATTCTCGACTCACGGATGAACACTAAGTACGATGAGAATGACAAGCTGATCCGCGAGGTGAAGGTCATCACCCTGAAGTCAAAGCTCGTCTCCGACTTCAGGAAGGATTTCCAGTTCTACAAGGTTCGGGAGATCAACAATTACCACCATGCCCATGACGCGTACCTGAACGCGGTGGTCGGCACAGCTCTGATCAAGAAGTACCCAAAGCTCGAGAGCGAGTTCGTGTACGGGGACTACAAGGTTTACGATGTGAGGAAGATGATCGCCAAGTCGGAGCAGGAGATTGGCAAGGCTACCGCCAAGTACTTCTTCTACTCTAACATTATGAATTTCTTCAAGACAGAGATCACTCTGGCCAATGGCGAGATCCGGAAGCGCCCCCTCATCGAGACGAACGGCGAGACGGGGGAGATCGTGTGGGACAAGGGCAGGGATTTCGCGACCGTCAGGAAGGTTCTCTCCATGCCACAAGTGAATATCGTCAAGAAGACAGAGGTCCAGACTGGCGGGTTCTCTAAGGAGTCAATTCTGCCTAAGCGGAACAGCGACAAGCTCATCGCCCGCAAGAAGGACTGGGATCCGAAGAAGTACGGCGGGTTCGACAGCCCCACTGTGGCCTACTCGGTCCTGGTTGTGGCGAAGGTTGAGAAGGGCAAGTCCAAGAAGCTCAAGAGCGTGAAGGAGCTGCTGGGGATCACGATTATGGAGCGCTCCAGCTTCGAGAAGAACCCGATCGATTTCCTGGAGGCGAAGGGCTACAAGGAGGTGAAGAAGGACCTGATCATTAAGCTCCCCAAGTACTCACTCTTCGAGCTGGAGAACGGCAGGAAGCGGATGCTGGCTTCCGCTGGCGAGCTGCAGAAGGGGAACGAGCTGGCTCTGCCGTCCAAGTATGTGAACTTCCTCTACCTGGCCTCCCACTACGAGAAGCTCAAGGGCAGCCCCGAGGACAACGAGCAGAAGCAGCTGTTCGTCGAGCAGCACAAGCATTACCTCGACGAGATCATTGAGCAGATTTCCGAGTTCTCCAAGCGCGTGATCCTGGCCGACGCGAATCTGGATAAGGTCCTCTCCGCGTACAACAAGCACCGCGACAAGCCAATCAGGGAGCAGGCTGAGAATATCATTCATCTCTTCACCCTGACGAACCTCGGCGCCCCTGCTGCTTTCAAGTACTTCGACACAACTATCGATCGCAAGAGGTACACAAGCACTAAGGAGGTCCTGGACGCGACCCTCATCCACCAGTCGATTACCGGCCTCTACGAGACGCGCATCGACCTGTCTCAGCTCGGGGGCGACGAATTCTCCGGGAGCGAGACGCCAGGCACCTCCGAGTCGGCCACCCCAGAATCTGCCACAGTGGTGTCCGGCCAAAAGCAGGACCGCCAGGGCGGAGAACGCAGAAGGTCCCAGCTCGATAGGGATCAGTGTGCCTACTGCAAGGAGAAGGGCCACTGGGCCAAAGACTGCCCGAAAAAGCCGCGCGGCCCACGCGGCCCAAGGCCACAAACATCCCTCCTTCCAAAGAAGAAGCGGAAGGTGGAGCTCAGCGGAGGATCTTCCGGAGGATCTAGCGGCTCCGAGACACCAGGAACATCCGAAAGCGCTACACCAGAATCTAGCGGAGGCTCTTCCGGAGGATCTAGGCCTCCTGAAGCCATCGTCTTCGCCGCTGACCCCATCACATGGAAATCACAGGATCCTGTCTGGGTGGAGCAATGGCCACTGACTGAGGAGAAGCTGCTGGCGGCAAAGGCCCTGATTTCTGAGCAGCTTGAGCTTGGGCATATTGAACCTAGCAATAGCCCTTGGAACACACCAATTTTTGTGATCAAGAAGAAAAGCGGGAAATGGAGGCTCCTGCAGGACTTGAGAGCCATCAATGCCACAATGGAGGATATGGGCGCCCTACAGCCCGGCCTGCCTAGCCCAGTGGCCATCCCTGAAGGATACAACATAATCGTGATCGATCTGCAGGACTGCTTCTTCACCATCCCTCTGAACGCCGAGGACAAGAAGCGTTTCGCCTTCTCGCTGCCCGCTGAAAATTTCAAGCAACCATACCTCAGATTCCAGTGGAAGGTGCTTCCTCAGGGAATGAAAAACAGCCCTACTCTGTGCCAAAAGTTTGTTAACGCGGCCATCGAAGACATCCGTGCAAAGTATGAGCAGCTATACATGATCCACTATATGGATGATATCCTAATTGCGCACCCCGACAGGGCCCACCTGCAGACGGTACTGCAGGATCTAACACAAGCCTTGACAGACCGGGGCTTGAAAATCGCCCCTGAGAAGATACAGGTCAACCCGCCGATTACATACCTCGGTCGGGTGATCAACTCCGAGACCGTGACCCACGCGCCTCTAAAGCTGAGAAAGGATCACTTAGTGACCTTAAATGACTACCAGAAGCTGTTAGGGGACATTAACTGGATCCGGCCATACTTAAAACTGACCACCGCCGAACTAAAGCCGCTGTTCAACATCCTACGGGGAGACCCAGATCCCACCTCTAAGAGACAGTTAACCGCCGAAGCACAGGAGGCTCTCGAGAAGGTGGAAGCGGCGCTCTCAGACAGCTATGTGAAGAGAGTCAACTTACAAACAAATTGGCAGTTCTTGTGTCTCGCCACCCCGACCGCGCCCACCGGCGTGCTCTGGCAAAACGGCCCTCTGGAGTGGGTACATCTACCCGCACAGGCCAAGAAGGTGGTCGCCTCTTACCCGGCCCTCATAGCCACCCTTATCCTTAAGGGTAGAAAGCGCTCCATCGAGCTCTTCGGCAAGGAGCCGGCCGAGATCGTGATACCTTACAATAAAGAACAGCTCGACGCTCTGCTGATGTTCGATGAGGACTGGCAGATTGCCATCGGAAACTATTTCGGCCAGATACTGCACCATCTTCCCAGCCATGTCCTGCTGAACTTCATGTCAAAGCACCCGGTGATTTTTCCGGTGAGGTGCAAGTACAGCCCAATTTCCGACGCCCAGATGGTGTTTACAGACGGCAGCGCAAATGGAAGGGCAAGCATCGTGACTAAGGATGAAAGAAAAATCCTCCACACACAGGAAACTTCTGTGCAACGGGCTGAGCTGACCGCCGTTATCGAAGCGTTCGTCATGTTTGCTGAGGAGGAGTTCAATCTCTACAGTGATAGCCAATACGTCGTGCGGTTGTTCCCCCACATAGAGACCGCGATATTGCCGGAGAACAAGACCGCCCTGTTCCATCTGCTGACAAAGCTGCAGCAGCAAATCTGGAAGAGAAGTCGCGCCTACTTCATCGGCCACATCAGGGCCCACAGTGGCCTCCCTGGCCCTCTCAACGCATTAAATGACCTGGCCGACAGCCTCACAAAAGTTACCGTAGCGAGTGCTTTTGAGGAGGCTAGAGCCAGCCACTCGCTGCACCACCAGAACGCGACTGCTAGCGGCGGCAGCAAAAGAACGGCGGACGGCTCTGAGAAGCGCACCGCTGATAGCCAGCATTCAACTCCTCCGAAAACAAAGAGGAAAGTTGAGTTCGAACCGAAGAAGAAAAGGAAGGTGTGA

**Sequence 38 Plasmids sequence of ePPEplus-RT37**

(NLSSV40-nCas9(H840A/R221K/N394K)-XTEN-NC-NLS-32aa Linker-RT37-NLSvbp)

CCTAAGAAAAAGAGAAAAGTGGACAAGAAGTACTCGATCGGCCTCGATATTGGGACTAACTCTGTTGGCTGGGCCGTGATCACCGACGAGTACAAGGTGCCCTCAAAGAAGTTCAAGGTCCTGGGCAACACCGATCGGCATTCCATCAAGAAGAATCTCATTGGCGCTCTCCTGTTCGACAGCGGCGAGACGGCTGAGGCTACGCGGCTCAAGCGCACCGCCCGCAGGCGGTACACGCGCAGGAAGAATCGCATCTGCTACCTGCAGGAGATTTTCTCCAACGAGATGGCGAAGGTTGACGATTCTTTCTTCCACAGGCTGGAGGAGTCATTCCTCGTGGAGGAGGATAAGAAGCACGAGCGGCATCCAATCTTCGGCAACATTGTCGACGAGGTTGCCTACCACGAGAAGTACCCTACGATCTACCATCTGCGGAAGAAGCTCGTGGACTCCACAGATAAGGCGGACCTCCGCCTGATCTACCTCGCTCTGGCCCACATGATTAAGTTCAGGGGCCATTTCCTGATCGAGGGGGATCTCAACCCGGACAATAGCGATGTTGACAAGCTGTTCATCCAGCTCGTGCAGACGTACAACCAGCTCTTCGAGGAGAACCCCATTAATGCGTCAGGCGTCGACGCGAAGGCTATCCTGTCCGCTAGGCTCTCGAAGTCTCGGAAGCTCGAGAACCTGATCGCCCAGCTGCCGGGCGAGAAGAAGAACGGCCTGTTCGGGAATCTCATTGCGCTCAGCCTGGGGCTCACGCCCAACTTCAAGTCGAATTTCGATCTCGCTGAGGACGCCAAGCTGCAGCTCTCCAAGGACACATACGACGATGACCTGGATAACCTCCTGGCCCAGATCGGCGATCAGTACGCGGACCTGTTCCTCGCTGCCAAGAATCTGTCGGACGCCATCCTCCTGTCTGATATTCTCAGGGTGAACACCGAGATTACGAAGGCTCCGCTCTCAGCCTCCATGATCAAGCGCTACGACGAGCACCATCAGGATCTGACCCTCCTGAAGGCGCTGGTCAGGCAGCAGCTCCCCGAGAAGTACAAGGAGATCTTCTTCGATCAGTCGAAGAACGGCTACGCTGGGTACATTGACGGCGGGGCCTCTCAGGAGGAGTTCTACAAGTTCATCAAGCCGATTCTGGAGAAGATGGACGGCACGGAGGAGCTGCTGGTGAAGCTCAAGCGCGAGGACCTCCTGAGGAAGCAGCGGACATTCGATAACGGCAGCATCCCACACCAGATTCATCTCGGGGAGCTGCACGCTATCCTGAGGAGGCAGGAGGACTTCTACCCTTTCCTCAAGGATAACCGCGAGAAGATCGAGAAGATTCTGACTTTCAGGATCCCGTACTACGTCGGCCCACTCGCTAGGGGCAACTCCCGCTTCGCTTGGATGACCCGCAAGTCAGAGGAGACGATCACGCCGTGGAACTTCGAGGAGGTGGTCGACAAGGGCGCTAGCGCTCAGTCGTTCATCGAGAGGATGACGAATTTCGACAAGAACCTGCCAAATGAGAAGGTGCTCCCTAAGCACTCGCTCCTGTACGAGTACTTCACAGTCTACAACGAGCTGACTAAGGTGAAGTATGTGACCGAGGGCATGAGGAAGCCGGCTTTCCTGTCTGGGGAGCAGAAGAAGGCCATCGTGGACCTCCTGTTCAAGACCAACCGGAAGGTCACGGTTAAGCAGCTCAAGGAGGACTACTTCAAGAAGATTGAGTGCTTCGATTCGGTCGAGATCTCTGGCGTTGAGGACCGCTTCAACGCCTCCCTGGGGACCTACCACGATCTCCTGAAGATCATTAAGGATAAGGACTTCCTGGACAACGAGGAGAATGAGGATATCCTCGAGGACATTGTGCTGACACTCACTCTGTTCGAGGACCGGGAGATGATCGAGGAGCGCCTGAAGACTTACGCCCATCTCTTCGATGACAAGGTCATGAAGCAGCTCAAGAGGAGGAGGTACACCGGCTGGGGGAGGCTGAGCAGGAAGCTCATCAACGGCATTCGGGACAAGCAGTCCGGGAAGACGATCCTCGACTTCCTGAAGAGCGATGGCTTCGCGAACCGCAATTTCATGCAGCTGATTCACGATGACAGCCTCACATTCAAGGAGGATATCCAGAAGGCTCAGGTGAGCGGCCAGGGGGACTCGCTGCACGAGCATATCGCGAACCTCGCTGGCTCGCCAGCTATCAAGAAGGGGATTCTGCAGACCGTGAAGGTTGTGGACGAGCTGGTGAAGGTCATGGGCAGGCACAAGCCTGAGAACATCGTCATTGAGATGGCCCGGGAGAATCAGACCACGCAGAAGGGCCAGAAGAACTCACGCGAGAGGATGAAGAGGATCGAGGAGGGCATTAAGGAGCTGGGGTCCCAGATCCTCAAGGAGCACCCGGTGGAGAACACGCAGCTGCAGAATGAGAAGCTCTACCTGTACTACCTCCAGAATGGCCGCGATATGTATGTGGACCAGGAGCTGGATATTAACAGGCTCAGCGATTACGACGTCGATGCCATCGTTCCACAGTCATTCCTGAAGGATGACTCCATTGACAACAAGGTCCTCACCAGGTCGGACAAGAACCGGGGCAAGTCTGATAATGTTCCTTCAGAGGAGGTCGTTAAGAAGATGAAGAACTACTGGCGCCAGCTCCTGAATGCCAAGCTGATCACGCAGCGGAAGTTCGATAACCTCACAAAGGCTGAGAGGGGCGGGCTCTCTGAGCTGGACAAGGCGGGCTTCATCAAGAGGCAGCTGGTCGAGACACGGCAGATCACTAAGCACGTTGCGCAGATTCTCGACTCACGGATGAACACTAAGTACGATGAGAATGACAAGCTGATCCGCGAGGTGAAGGTCATCACCCTGAAGTCAAAGCTCGTCTCCGACTTCAGGAAGGATTTCCAGTTCTACAAGGTTCGGGAGATCAACAATTACCACCATGCCCATGACGCGTACCTGAACGCGGTGGTCGGCACAGCTCTGATCAAGAAGTACCCAAAGCTCGAGAGCGAGTTCGTGTACGGGGACTACAAGGTTTACGATGTGAGGAAGATGATCGCCAAGTCGGAGCAGGAGATTGGCAAGGCTACCGCCAAGTACTTCTTCTACTCTAACATTATGAATTTCTTCAAGACAGAGATCACTCTGGCCAATGGCGAGATCCGGAAGCGCCCCCTCATCGAGACGAACGGCGAGACGGGGGAGATCGTGTGGGACAAGGGCAGGGATTTCGCGACCGTCAGGAAGGTTCTCTCCATGCCACAAGTGAATATCGTCAAGAAGACAGAGGTCCAGACTGGCGGGTTCTCTAAGGAGTCAATTCTGCCTAAGCGGAACAGCGACAAGCTCATCGCCCGCAAGAAGGACTGGGATCCGAAGAAGTACGGCGGGTTCGACAGCCCCACTGTGGCCTACTCGGTCCTGGTTGTGGCGAAGGTTGAGAAGGGCAAGTCCAAGAAGCTCAAGAGCGTGAAGGAGCTGCTGGGGATCACGATTATGGAGCGCTCCAGCTTCGAGAAGAACCCGATCGATTTCCTGGAGGCGAAGGGCTACAAGGAGGTGAAGAAGGACCTGATCATTAAGCTCCCCAAGTACTCACTCTTCGAGCTGGAGAACGGCAGGAAGCGGATGCTGGCTTCCGCTGGCGAGCTGCAGAAGGGGAACGAGCTGGCTCTGCCGTCCAAGTATGTGAACTTCCTCTACCTGGCCTCCCACTACGAGAAGCTCAAGGGCAGCCCCGAGGACAACGAGCAGAAGCAGCTGTTCGTCGAGCAGCACAAGCATTACCTCGACGAGATCATTGAGCAGATTTCCGAGTTCTCCAAGCGCGTGATCCTGGCCGACGCGAATCTGGATAAGGTCCTCTCCGCGTACAACAAGCACCGCGACAAGCCAATCAGGGAGCAGGCTGAGAATATCATTCATCTCTTCACCCTGACGAACCTCGGCGCCCCTGCTGCTTTCAAGTACTTCGACACAACTATCGATCGCAAGAGGTACACAAGCACTAAGGAGGTCCTGGACGCGACCCTCATCCACCAGTCGATTACCGGCCTCTACGAGACGCGCATCGACCTGTCTCAGCTCGGGGGCGACGAATTCTCCGGGAGCGAGACGCCAGGCACCTCCGAGTCGGCCACCCCAGAATCTGCCACAGTGGTGTCCGGCCAAAAGCAGGACCGCCAGGGCGGAGAACGCAGAAGGTCCCAGCTCGATAGGGATCAGTGTGCCTACTGCAAGGAGAAGGGCCACTGGGCCAAAGACTGCCCGAAAAAGCCGCGCGGCCCACGCGGCCCAAGGCCACAAACATCCCTCCTTCCAAAGAAGAAGCGGAAGGTGGAGCTCAGCGGAGGATCTTCCGGAGGATCTAGCGGCTCCGAGACACCAGGAACATCCGAAAGCGCTACACCAGAATCTAGCGGAGGCTCTTCCGGAGGATCTAGGCCTCCTTTGGACACCGGGAGCAATCAGGAAAAAGGTCTGGGCAAGGAATCAAACACTAGCCACAGCAGTACCGGGTTCATCATCGGGGCCATCGCGCACCGCACCGCCGACCCTATTGTCTGGAAGAGCTCACAGCCGGTGTGGGTTGAGCAATGGCCACTGACCTCTGAGAAGATAGCCGCTGCCCGACAGCTGATTGATCAGCAGCTTGCCGAGGGGCACATTGAACCATCCAACTCGCCGTGGAATACCCCTATATTTGTGATAAAGAAGAAGAGCGGGAAGTGGCGATTGCTGCAGGACCTGCGCGCTATCAATGATACGATGGAGGACATGGGATCTCTGCAGCCCGGCCTGCCTTCTCCTGTGGCAATACCTCAGGAGTACTGCGTGGTGGTCATCGACCTTCAGGATTGCTTCTTCACCATCCCTCTTCACCCCGCGGACTGCCAGCGCTTCGCCTTCAGCATACCCAGCGAAAATTTCAAACAACCTTACCAGAGGTACCAGTGGAAAGTTCTGCCGCAGGGGATGAAGAATTCTCCTACCCTTTGTCAGAAGTTCGTCGACCAGGCCCTCCAGGTCATAAGAAAGAAATTTTCCGACCTCTACCTGATCCACTACATGGATGATATCCTTCTGGCACATAAGGATAGGGCCACCCTTCAAGAGATTCTGACCCAGACGGTGCTCGCCTTGGAGGAGCATGGGCTCAAGATCGCCCCAGAGAAAATCCAAACAGAACCTTCGTTCTCGTACCTGGGCAGAATTTTACACACCAGCACAATCACACACCAGCCTCTGCAACTGAGGAAGGACCACCTCAACACACTGAACGACTACCAAAAGCTGCTGGGCGACATCAACTGGGTGAGAAGCTATCTGAAGATAACCACCCTGGATCTGAAACCACTGTTTGATATCTTGAAGGGCGATTCCAACCCGAAGAGTCCTCGGCAGCTAACGGTGGAGGGAGAGAAGGCTATCGCTAAGATTGAGCAGGCTATCAACAAGCAGCAACTGCAGTATCTTGATTACTCAAAATCTTGGGCGCTGATCATTCTCGCCACGAAATATACCCCAACTGGCTGCCTGTGGCAAGAAGGCCCTCTGGAGTGGATACACTTGCCTGTTACCCCTAGAAAGATTGTGCCCTCATACCCTAGCCTTGTGGCCACCTTGATTATCAAAGGAAGACGTAGATCTATCGAGCTGTTTGGCCGGGAGGTGACTGAAATCATCATCCCATACAAGAGAGAACAGCTTGACACACTTCTCCAGTTTGAAGAAGAATGGCAGATTGCCATCGGCAACTTCCCCGGCCAAATTCTTCACCACCTACCGCGGAGCCCAATCCTGCAGTTCCTCAGCCTGCACCCATTTATCTTCCCCATCAAATGTTCTAAGGAGCCTCTCTCCCCCCCCGCCTCGCTGGTATTCACGGACGGTTCTAGCAATGGCCGCGCCGTCACCATCATCGACTCCATTACTCATGTCCAACAGACCCTGGAGACCAGCGCGCAGAGAACTGAGCTGCTCGCCGTGATCTACGCGTTTGAACAACTGCAGCAGGTGCCGTTCAATCTGTACACAGATTCTCAGTACATCGTGAAGCTATTTCCGCACATTGAAACTGCCTCCCTGCCCCAGGGAAGAACAGCCATCTTCTCCTTGCTGACTCAGTTACAGACATACATACATAAGAGAGAGAAGGCATTCTACATAGGCCACATCCGGGCCCACAGCTGCCTTCCTGGCCCACTGAGTGAAGGCAACTATCAAGCTGACTTACTTACTCGGCCTGTGGTGTGTACAGCTCTGGAGGAGGCAAGGAGATCCCACGCTATCCATCACCAAAACAGCGGCGGCAGCAAAAGAACGGCGGACGGCTCTGAGAAGCGCACCGCTGATAGCCAGCATTCAACTCCTCCGAAAACAAAGAGGAAAGTTGAGTTCGAACCGAAGAAGAAAAGGAAGGTGTGA

**Sequence 39 Plasmids sequence of ePPEplus-RT38**

(NLSSV40-nCas9(H840A/R221K/N394K)-XTEN-NC-NLS-32aa Linker-RT38-NLSvbp)

CCTAAGAAAAAGAGAAAAGTGGACAAGAAGTACTCGATCGGCCTCGATATTGGGACTAACTCTGTTGGCTGGGCCGTGATCACCGACGAGTACAAGGTGCCCTCAAAGAAGTTCAAGGTCCTGGGCAACACCGATCGGCATTCCATCAAGAAGAATCTCATTGGCGCTCTCCTGTTCGACAGCGGCGAGACGGCTGAGGCTACGCGGCTCAAGCGCACCGCCCGCAGGCGGTACACGCGCAGGAAGAATCGCATCTGCTACCTGCAGGAGATTTTCTCCAACGAGATGGCGAAGGTTGACGATTCTTTCTTCCACAGGCTGGAGGAGTCATTCCTCGTGGAGGAGGATAAGAAGCACGAGCGGCATCCAATCTTCGGCAACATTGTCGACGAGGTTGCCTACCACGAGAAGTACCCTACGATCTACCATCTGCGGAAGAAGCTCGTGGACTCCACAGATAAGGCGGACCTCCGCCTGATCTACCTCGCTCTGGCCCACATGATTAAGTTCAGGGGCCATTTCCTGATCGAGGGGGATCTCAACCCGGACAATAGCGATGTTGACAAGCTGTTCATCCAGCTCGTGCAGACGTACAACCAGCTCTTCGAGGAGAACCCCATTAATGCGTCAGGCGTCGACGCGAAGGCTATCCTGTCCGCTAGGCTCTCGAAGTCTCGGAAGCTCGAGAACCTGATCGCCCAGCTGCCGGGCGAGAAGAAGAACGGCCTGTTCGGGAATCTCATTGCGCTCAGCCTGGGGCTCACGCCCAACTTCAAGTCGAATTTCGATCTCGCTGAGGACGCCAAGCTGCAGCTCTCCAAGGACACATACGACGATGACCTGGATAACCTCCTGGCCCAGATCGGCGATCAGTACGCGGACCTGTTCCTCGCTGCCAAGAATCTGTCGGACGCCATCCTCCTGTCTGATATTCTCAGGGTGAACACCGAGATTACGAAGGCTCCGCTCTCAGCCTCCATGATCAAGCGCTACGACGAGCACCATCAGGATCTGACCCTCCTGAAGGCGCTGGTCAGGCAGCAGCTCCCCGAGAAGTACAAGGAGATCTTCTTCGATCAGTCGAAGAACGGCTACGCTGGGTACATTGACGGCGGGGCCTCTCAGGAGGAGTTCTACAAGTTCATCAAGCCGATTCTGGAGAAGATGGACGGCACGGAGGAGCTGCTGGTGAAGCTCAAGCGCGAGGACCTCCTGAGGAAGCAGCGGACATTCGATAACGGCAGCATCCCACACCAGATTCATCTCGGGGAGCTGCACGCTATCCTGAGGAGGCAGGAGGACTTCTACCCTTTCCTCAAGGATAACCGCGAGAAGATCGAGAAGATTCTGACTTTCAGGATCCCGTACTACGTCGGCCCACTCGCTAGGGGCAACTCCCGCTTCGCTTGGATGACCCGCAAGTCAGAGGAGACGATCACGCCGTGGAACTTCGAGGAGGTGGTCGACAAGGGCGCTAGCGCTCAGTCGTTCATCGAGAGGATGACGAATTTCGACAAGAACCTGCCAAATGAGAAGGTGCTCCCTAAGCACTCGCTCCTGTACGAGTACTTCACAGTCTACAACGAGCTGACTAAGGTGAAGTATGTGACCGAGGGCATGAGGAAGCCGGCTTTCCTGTCTGGGGAGCAGAAGAAGGCCATCGTGGACCTCCTGTTCAAGACCAACCGGAAGGTCACGGTTAAGCAGCTCAAGGAGGACTACTTCAAGAAGATTGAGTGCTTCGATTCGGTCGAGATCTCTGGCGTTGAGGACCGCTTCAACGCCTCCCTGGGGACCTACCACGATCTCCTGAAGATCATTAAGGATAAGGACTTCCTGGACAACGAGGAGAATGAGGATATCCTCGAGGACATTGTGCTGACACTCACTCTGTTCGAGGACCGGGAGATGATCGAGGAGCGCCTGAAGACTTACGCCCATCTCTTCGATGACAAGGTCATGAAGCAGCTCAAGAGGAGGAGGTACACCGGCTGGGGGAGGCTGAGCAGGAAGCTCATCAACGGCATTCGGGACAAGCAGTCCGGGAAGACGATCCTCGACTTCCTGAAGAGCGATGGCTTCGCGAACCGCAATTTCATGCAGCTGATTCACGATGACAGCCTCACATTCAAGGAGGATATCCAGAAGGCTCAGGTGAGCGGCCAGGGGGACTCGCTGCACGAGCATATCGCGAACCTCGCTGGCTCGCCAGCTATCAAGAAGGGGATTCTGCAGACCGTGAAGGTTGTGGACGAGCTGGTGAAGGTCATGGGCAGGCACAAGCCTGAGAACATCGTCATTGAGATGGCCCGGGAGAATCAGACCACGCAGAAGGGCCAGAAGAACTCACGCGAGAGGATGAAGAGGATCGAGGAGGGCATTAAGGAGCTGGGGTCCCAGATCCTCAAGGAGCACCCGGTGGAGAACACGCAGCTGCAGAATGAGAAGCTCTACCTGTACTACCTCCAGAATGGCCGCGATATGTATGTGGACCAGGAGCTGGATATTAACAGGCTCAGCGATTACGACGTCGATGCCATCGTTCCACAGTCATTCCTGAAGGATGACTCCATTGACAACAAGGTCCTCACCAGGTCGGACAAGAACCGGGGCAAGTCTGATAATGTTCCTTCAGAGGAGGTCGTTAAGAAGATGAAGAACTACTGGCGCCAGCTCCTGAATGCCAAGCTGATCACGCAGCGGAAGTTCGATAACCTCACAAAGGCTGAGAGGGGCGGGCTCTCTGAGCTGGACAAGGCGGGCTTCATCAAGAGGCAGCTGGTCGAGACACGGCAGATCACTAAGCACGTTGCGCAGATTCTCGACTCACGGATGAACACTAAGTACGATGAGAATGACAAGCTGATCCGCGAGGTGAAGGTCATCACCCTGAAGTCAAAGCTCGTCTCCGACTTCAGGAAGGATTTCCAGTTCTACAAGGTTCGGGAGATCAACAATTACCACCATGCCCATGACGCGTACCTGAACGCGGTGGTCGGCACAGCTCTGATCAAGAAGTACCCAAAGCTCGAGAGCGAGTTCGTGTACGGGGACTACAAGGTTTACGATGTGAGGAAGATGATCGCCAAGTCGGAGCAGGAGATTGGCAAGGCTACCGCCAAGTACTTCTTCTACTCTAACATTATGAATTTCTTCAAGACAGAGATCACTCTGGCCAATGGCGAGATCCGGAAGCGCCCCCTCATCGAGACGAACGGCGAGACGGGGGAGATCGTGTGGGACAAGGGCAGGGATTTCGCGACCGTCAGGAAGGTTCTCTCCATGCCACAAGTGAATATCGTCAAGAAGACAGAGGTCCAGACTGGCGGGTTCTCTAAGGAGTCAATTCTGCCTAAGCGGAACAGCGACAAGCTCATCGCCCGCAAGAAGGACTGGGATCCGAAGAAGTACGGCGGGTTCGACAGCCCCACTGTGGCCTACTCGGTCCTGGTTGTGGCGAAGGTTGAGAAGGGCAAGTCCAAGAAGCTCAAGAGCGTGAAGGAGCTGCTGGGGATCACGATTATGGAGCGCTCCAGCTTCGAGAAGAACCCGATCGATTTCCTGGAGGCGAAGGGCTACAAGGAGGTGAAGAAGGACCTGATCATTAAGCTCCCCAAGTACTCACTCTTCGAGCTGGAGAACGGCAGGAAGCGGATGCTGGCTTCCGCTGGCGAGCTGCAGAAGGGGAACGAGCTGGCTCTGCCGTCCAAGTATGTGAACTTCCTCTACCTGGCCTCCCACTACGAGAAGCTCAAGGGCAGCCCCGAGGACAACGAGCAGAAGCAGCTGTTCGTCGAGCAGCACAAGCATTACCTCGACGAGATCATTGAGCAGATTTCCGAGTTCTCCAAGCGCGTGATCCTGGCCGACGCGAATCTGGATAAGGTCCTCTCCGCGTACAACAAGCACCGCGACAAGCCAATCAGGGAGCAGGCTGAGAATATCATTCATCTCTTCACCCTGACGAACCTCGGCGCCCCTGCTGCTTTCAAGTACTTCGACACAACTATCGATCGCAAGAGGTACACAAGCACTAAGGAGGTCCTGGACGCGACCCTCATCCACCAGTCGATTACCGGCCTCTACGAGACGCGCATCGACCTGTCTCAGCTCGGGGGCGACGAATTCTCCGGGAGCGAGACGCCAGGCACCTCCGAGTCGGCCACCCCAGAATCTGCCACAGTGGTGTCCGGCCAAAAGCAGGACCGCCAGGGCGGAGAACGCAGAAGGTCCCAGCTCGATAGGGATCAGTGTGCCTACTGCAAGGAGAAGGGCCACTGGGCCAAAGACTGCCCGAAAAAGCCGCGCGGCCCACGCGGCCCAAGGCCACAAACATCCCTCCTTCCAAAGAAGAAGCGGAAGGTGGAGCTCAGCGGAGGATCTTCCGGAGGATCTAGCGGCTCCGAGACACCAGGAACATCCGAAAGCGCTACACCAGAATCTAGCGGAGGCTCTTCCGGAGGATCTAGGCCTCCTGCCCCGCAGCAATGCGCCGAACCCATCACATGGAAGAGTGATGAACCTGTGTGGGTGGATCAATGGCCGCTGACGAATGATAAGCTGGCCGCCGCGCAGCAGTTAGTTCAGGAACAACTGGAGGCGGGCCATATCACCGAGTCTAGCAGTCCATGGAACACTCCTATCTTTGTGATCAAGAAGAAAAGCGGGAAGTGGCGGCTGCTGCAAGACCTGCGGGCGGTCAACGCTACCATGGTGCTGATGGGGGCGCTGCAGCCCGGCCTCCCAAGCCCCGTCGCCATCCCACAGGGTTATCTTAAGATAATCATCGACTTGAAGGACTGCTTCTTCTCAATCCCTCTTCACCCTTCGGACCAGAAGAGGTTCGCCTTCTCCCTGCCTAGCACCAACTTCAAGGAACCAATGCAGAGATTTCAATGGAAGGTTCTGCCTCAGGGCATGGCAAACTCCCCCACACTGTGCCAAAAATACGTGGCCACCGCCATCCACAAGGTGCGCCACGCGTGGAAACAAATGTACATTATCCATTACATGGATGATATACTAATTGCGGGCAAGGATGGCCAGCAGGTGCTGCAGTGCTTTGATCAACTCAAGCAGGAGCTCACCGCCGCCGGCCTGCACATCGCGCCTGAGAAAGTGCAGTTGCAGGACCCATACACCTACCTGGGTTTTGAACTGAATGGTCCTAAAATCACTAACCAGAAAGCTGTTATTCGGAAAGACAAGCTGCAAACCCTGAACGATTTCCAGAAGCTACTCGGCGACATCAACTGGCTCCGGCCCTACCTGAAGCTGACCACCGGAGACTTGAAGCCGCTGTTCGACACCCTGAAGGGTGATTCTGATCCTAACTCCCACCGAAGCCTGAGCAAGGAGGCGCTAGCCAGCCTTGAAAAGGTGGAAACCGCGATCGCGGAGCAGTTTGTGACGCACATCAACTATAGCCTGCCGCTAATTTTCCTCATCTTCAATACCGCCCTAACACCAACGGGCCTCTTCTGGCAGGATAACCCTATCATGTGGATTCACTTACCTGCTTCCCCGAAAAAGGTGCTGCTGCCATATTATGATGCCATCGCCGACCTAATCATACTTGGCCGCGACCACTCCAAGAAGTACTTCGGCATCGAGCCTTCTACAATTATCCAGCCTTACTCGAAATCTCAAATTGACTGGTTGATGCAAAATACAGAGATGTGGCCAATCGCTTGTGCCAGCTTCGTCGGTATCCTCGACAACCACTACCCGCCGAACAAGCTCATCCAGTTCTGCAAGCTGCACACTTTTGTGTTCCCTCAAATCATTAGCAAGACGCCCCTCAACAATGCCCTTCTCGTTTTCACTGATGGCTCAAGCACTGGAATGGCCGCCTACACCCTGACAGACACAACCATCAAGTTTCAAACAAATCTGAACTCGGCTCAGCTGGTGGAACTGCAGGCACTGATCGCTGTCTTGAGCGCCTTCCCAAATCAACCACTGAACATTTACACAGACTCTGCCTACCTAGCACACAGCATCCCTCTGCTGGAGACGGTGGCCCAGATCAAGCATATCTCAGAAACTGCTAAACTTTTCCTGCAGTGTCAACAGCTGATATACAACAGATCTATCCCGTTCTACATTGGCCATGTCAGAGCTCATTCGGGCTTGCCCGGCCCTATTGCCCAGGGAAACCAAAGAGCCGACCTTGCGACCAAGATCGTTGCCTCCAACATTAACACAAACAGCGGCGGCAGCAAAAGAACGGCGGACGGCTCTGAGAAGCGCACCGCTGATAGCCAGCATTCAACTCCTCCGAAAACAAAGAGGAAAGTTGAGTTCGAACCGAAGAAGAAAAGGAAGGTGTGA

**Sequence 40 Plasmids sequence of ePPEplus-RT39**

(NLSSV40-nCas9(H840A/R221K/N394K)-XTEN-NC-NLS-32aa Linker-RT39-NLSvbp)

CCTAAGAAAAAGAGAAAAGTGGACAAGAAGTACTCGATCGGCCTCGATATTGGGACTAACTCTGTTGGCTGGGCCGTGATCACCGACGAGTACAAGGTGCCCTCAAAGAAGTTCAAGGTCCTGGGCAACACCGATCGGCATTCCATCAAGAAGAATCTCATTGGCGCTCTCCTGTTCGACAGCGGCGAGACGGCTGAGGCTACGCGGCTCAAGCGCACCGCCCGCAGGCGGTACACGCGCAGGAAGAATCGCATCTGCTACCTGCAGGAGATTTTCTCCAACGAGATGGCGAAGGTTGACGATTCTTTCTTCCACAGGCTGGAGGAGTCATTCCTCGTGGAGGAGGATAAGAAGCACGAGCGGCATCCAATCTTCGGCAACATTGTCGACGAGGTTGCCTACCACGAGAAGTACCCTACGATCTACCATCTGCGGAAGAAGCTCGTGGACTCCACAGATAAGGCGGACCTCCGCCTGATCTACCTCGCTCTGGCCCACATGATTAAGTTCAGGGGCCATTTCCTGATCGAGGGGGATCTCAACCCGGACAATAGCGATGTTGACAAGCTGTTCATCCAGCTCGTGCAGACGTACAACCAGCTCTTCGAGGAGAACCCCATTAATGCGTCAGGCGTCGACGCGAAGGCTATCCTGTCCGCTAGGCTCTCGAAGTCTCGGAAGCTCGAGAACCTGATCGCCCAGCTGCCGGGCGAGAAGAAGAACGGCCTGTTCGGGAATCTCATTGCGCTCAGCCTGGGGCTCACGCCCAACTTCAAGTCGAATTTCGATCTCGCTGAGGACGCCAAGCTGCAGCTCTCCAAGGACACATACGACGATGACCTGGATAACCTCCTGGCCCAGATCGGCGATCAGTACGCGGACCTGTTCCTCGCTGCCAAGAATCTGTCGGACGCCATCCTCCTGTCTGATATTCTCAGGGTGAACACCGAGATTACGAAGGCTCCGCTCTCAGCCTCCATGATCAAGCGCTACGACGAGCACCATCAGGATCTGACCCTCCTGAAGGCGCTGGTCAGGCAGCAGCTCCCCGAGAAGTACAAGGAGATCTTCTTCGATCAGTCGAAGAACGGCTACGCTGGGTACATTGACGGCGGGGCCTCTCAGGAGGAGTTCTACAAGTTCATCAAGCCGATTCTGGAGAAGATGGACGGCACGGAGGAGCTGCTGGTGAAGCTCAAGCGCGAGGACCTCCTGAGGAAGCAGCGGACATTCGATAACGGCAGCATCCCACACCAGATTCATCTCGGGGAGCTGCACGCTATCCTGAGGAGGCAGGAGGACTTCTACCCTTTCCTCAAGGATAACCGCGAGAAGATCGAGAAGATTCTGACTTTCAGGATCCCGTACTACGTCGGCCCACTCGCTAGGGGCAACTCCCGCTTCGCTTGGATGACCCGCAAGTCAGAGGAGACGATCACGCCGTGGAACTTCGAGGAGGTGGTCGACAAGGGCGCTAGCGCTCAGTCGTTCATCGAGAGGATGACGAATTTCGACAAGAACCTGCCAAATGAGAAGGTGCTCCCTAAGCACTCGCTCCTGTACGAGTACTTCACAGTCTACAACGAGCTGACTAAGGTGAAGTATGTGACCGAGGGCATGAGGAAGCCGGCTTTCCTGTCTGGGGAGCAGAAGAAGGCCATCGTGGACCTCCTGTTCAAGACCAACCGGAAGGTCACGGTTAAGCAGCTCAAGGAGGACTACTTCAAGAAGATTGAGTGCTTCGATTCGGTCGAGATCTCTGGCGTTGAGGACCGCTTCAACGCCTCCCTGGGGACCTACCACGATCTCCTGAAGATCATTAAGGATAAGGACTTCCTGGACAACGAGGAGAATGAGGATATCCTCGAGGACATTGTGCTGACACTCACTCTGTTCGAGGACCGGGAGATGATCGAGGAGCGCCTGAAGACTTACGCCCATCTCTTCGATGACAAGGTCATGAAGCAGCTCAAGAGGAGGAGGTACACCGGCTGGGGGAGGCTGAGCAGGAAGCTCATCAACGGCATTCGGGACAAGCAGTCCGGGAAGACGATCCTCGACTTCCTGAAGAGCGATGGCTTCGCGAACCGCAATTTCATGCAGCTGATTCACGATGACAGCCTCACATTCAAGGAGGATATCCAGAAGGCTCAGGTGAGCGGCCAGGGGGACTCGCTGCACGAGCATATCGCGAACCTCGCTGGCTCGCCAGCTATCAAGAAGGGGATTCTGCAGACCGTGAAGGTTGTGGACGAGCTGGTGAAGGTCATGGGCAGGCACAAGCCTGAGAACATCGTCATTGAGATGGCCCGGGAGAATCAGACCACGCAGAAGGGCCAGAAGAACTCACGCGAGAGGATGAAGAGGATCGAGGAGGGCATTAAGGAGCTGGGGTCCCAGATCCTCAAGGAGCACCCGGTGGAGAACACGCAGCTGCAGAATGAGAAGCTCTACCTGTACTACCTCCAGAATGGCCGCGATATGTATGTGGACCAGGAGCTGGATATTAACAGGCTCAGCGATTACGACGTCGATGCCATCGTTCCACAGTCATTCCTGAAGGATGACTCCATTGACAACAAGGTCCTCACCAGGTCGGACAAGAACCGGGGCAAGTCTGATAATGTTCCTTCAGAGGAGGTCGTTAAGAAGATGAAGAACTACTGGCGCCAGCTCCTGAATGCCAAGCTGATCACGCAGCGGAAGTTCGATAACCTCACAAAGGCTGAGAGGGGCGGGCTCTCTGAGCTGGACAAGGCGGGCTTCATCAAGAGGCAGCTGGTCGAGACACGGCAGATCACTAAGCACGTTGCGCAGATTCTCGACTCACGGATGAACACTAAGTACGATGAGAATGACAAGCTGATCCGCGAGGTGAAGGTCATCACCCTGAAGTCAAAGCTCGTCTCCGACTTCAGGAAGGATTTCCAGTTCTACAAGGTTCGGGAGATCAACAATTACCACCATGCCCATGACGCGTACCTGAACGCGGTGGTCGGCACAGCTCTGATCAAGAAGTACCCAAAGCTCGAGAGCGAGTTCGTGTACGGGGACTACAAGGTTTACGATGTGAGGAAGATGATCGCCAAGTCGGAGCAGGAGATTGGCAAGGCTACCGCCAAGTACTTCTTCTACTCTAACATTATGAATTTCTTCAAGACAGAGATCACTCTGGCCAATGGCGAGATCCGGAAGCGCCCCCTCATCGAGACGAACGGCGAGACGGGGGAGATCGTGTGGGACAAGGGCAGGGATTTCGCGACCGTCAGGAAGGTTCTCTCCATGCCACAAGTGAATATCGTCAAGAAGACAGAGGTCCAGACTGGCGGGTTCTCTAAGGAGTCAATTCTGCCTAAGCGGAACAGCGACAAGCTCATCGCCCGCAAGAAGGACTGGGATCCGAAGAAGTACGGCGGGTTCGACAGCCCCACTGTGGCCTACTCGGTCCTGGTTGTGGCGAAGGTTGAGAAGGGCAAGTCCAAGAAGCTCAAGAGCGTGAAGGAGCTGCTGGGGATCACGATTATGGAGCGCTCCAGCTTCGAGAAGAACCCGATCGATTTCCTGGAGGCGAAGGGCTACAAGGAGGTGAAGAAGGACCTGATCATTAAGCTCCCCAAGTACTCACTCTTCGAGCTGGAGAACGGCAGGAAGCGGATGCTGGCTTCCGCTGGCGAGCTGCAGAAGGGGAACGAGCTGGCTCTGCCGTCCAAGTATGTGAACTTCCTCTACCTGGCCTCCCACTACGAGAAGCTCAAGGGCAGCCCCGAGGACAACGAGCAGAAGCAGCTGTTCGTCGAGCAGCACAAGCATTACCTCGACGAGATCATTGAGCAGATTTCCGAGTTCTCCAAGCGCGTGATCCTGGCCGACGCGAATCTGGATAAGGTCCTCTCCGCGTACAACAAGCACCGCGACAAGCCAATCAGGGAGCAGGCTGAGAATATCATTCATCTCTTCACCCTGACGAACCTCGGCGCCCCTGCTGCTTTCAAGTACTTCGACACAACTATCGATCGCAAGAGGTACACAAGCACTAAGGAGGTCCTGGACGCGACCCTCATCCACCAGTCGATTACCGGCCTCTACGAGACGCGCATCGACCTGTCTCAGCTCGGGGGCGACGAATTCTCCGGGAGCGAGACGCCAGGCACCTCCGAGTCGGCCACCCCAGAATCTGCCACAGTGGTGTCCGGCCAAAAGCAGGACCGCCAGGGCGGAGAACGCAGAAGGTCCCAGCTCGATAGGGATCAGTGTGCCTACTGCAAGGAGAAGGGCCACTGGGCCAAAGACTGCCCGAAAAAGCCGCGCGGCCCACGCGGCCCAAGGCCACAAACATCCCTCCTTCCAAAGAAGAAGCGGAAGGTGGAGCTCAGCGGAGGATCTTCCGGAGGATCTAGCGGCTCCGAGACACCAGGAACATCCGAAAGCGCTACACCAGAATCTAGCGGAGGCTCTTCCGGAGGATCTAGGCCTCCTGAGCCAATCGCTCTGTCATGGAAGTCAAATGAACCCATTTGGATTGAACAGTGGCCTTTAACCCGGGAGAAGTTACAGGCCGCCGAAGAGCTCGTCGAGGAGCAGCTGCAGAAGGGCCACATCGAGACCTCCACAAGCCCGTGGAACTCTCCCATATTTGTGATCCGAAAGAAAAGCGGCAAATGGAGATTACTGACTGATCTTAGAGCTGTCAATGCGTCCATGTTCCCAATGGGCGCCTTGCAGCCGGGCCTCCCCGCTCCGGTGATGCTCCCTCGCGACTGGCCGCTCCTGATAATCGATCTGCTCGATTGCTTCTTCACCATACCTATCCATCCCCAAGATAAACAGAGATTTGCATTCTCTATCCCAAAGATCAATAATGCAGGGCCACACACCAGATACCAATGGAGGGTGCTGCCTCAGGGAATGATGAATTCCCCTACCATCTGCCAGTTGTACGTCGACGCCGCCTTGAAACCTATCAGGAAGAGATATCCATTTCTCCACATTTACCATTACATGGACGATATCCTGTTCGCCGGCCCCAAGAAGGAATTTCTGCAGGATCTGCTGGAGCAACTGCCGCTGTACTTTAAGCCATATGGACTCATTATTGCTCCTGAAAAGATTCAGCTGGAGGACATCAGTCACTATCTCGGTTTCGTGGTGAGCAGAACGACGGTCCACCCACAGAAGATGACAATACGGCGTGACAACCTGAAAACACTGAATGACTTCCAGAAGCTGTTGGGCGACATCAACTGGCTGCGCCCGAGTCTGGGCATTCCTACTTACCAGCTTCACAACCTGTTCGATACCCTGAAGGGGGATACGGACCTCAACAGCGCGAGATGCCTGACCCCCGCCGCCGAAGAAGAATTGCAGTTCTTTGAGCATCAGCTGAAATCCAAATTTCTCATGAGAATTGATCCTCTCCTGCCTCTTACCCTGTACCTGATCAACACTTTCATGTACCCAACAGCCCTGCTCGGCCAAAATGAGGAGCCTTGTGAGTGGCTCTACACCCGCCACCGCTTCCACCGAAGCATTATGTCATATGAACAGCAAATCGCCATCCTGATACAGCAGGGCAGACAGCGGTGTATAAGCCTCTCGGGCTATGATGTGAGCACAATTGTGCTACCTCTGACTAAGAGCCAATTCTTCTTCCTGCTGCAACGGAGCGAAGCACTGCAAATCGCCCTGGCGCAGTTTGTAGGAAACATTGAGTATACCCTGCCGAAAGGAAAGCTCTGGGATTTTTTCAAGAGACATAATTTCGTTATTAACCAAATCATCTCTGACATCCCGCTGGAGGGCCCCAACGTCTTCACAGATGGGAGCCGGCAGAGATCCGCGTATTGGGCAGAGAAGAAATACTGGTCTCAGAAGCAGAACACAAACTCTATTCAAAAGAATGAACTTCTCGCCATCATCCAGGTTCTGAAAGATTTCAAGCAAGACATCAATATCATCGCCGACTCCGCTTACGTGGTGGGTGTGGTGAAGAACATCATCGGCGCGGTGATCAACTCTCTGGACAAGGAGCTGACAACCCTGTTCAAAACCTTACAGGTGTTACTGACCAGCCGTAACGCTCGCATCTTCATCGTGCATATCAGATCACACAGTAAGCTGCCCGGCCCTTTGGTCTACGGCAACGAGCAGGTAGACAAATTAGCCGCCTTCGCTAGCCCGGAGGAAGAGCACAGATACTTCCATAACAACAGCGGCGGCAGCAAAAGAACGGCGGACGGCTCTGAGAAGCGCACCGCTGATAGCCAGCATTCAACTCCTCCGAAAACAAAGAGGAAAGTTGAGTTCGAACCGAAGAAGAAAAGGAAGGTGTGA

**Sequence 41 Plasmids sequence of ePPEplus-RT40**

(NLSSV40-nCas9(H840A/R221K/N394K)-XTEN-NC-NLS-32aa Linker-RT40-NLSvbp)

CCTAAGAAAAAGAGAAAAGTGGACAAGAAGTACTCGATCGGCCTCGATATTGGGACTAACTCTGTTGGCTGGGCCGTGATCACCGACGAGTACAAGGTGCCCTCAAAGAAGTTCAAGGTCCTGGGCAACACCGATCGGCATTCCATCAAGAAGAATCTCATTGGCGCTCTCCTGTTCGACAGCGGCGAGACGGCTGAGGCTACGCGGCTCAAGCGCACCGCCCGCAGGCGGTACACGCGCAGGAAGAATCGCATCTGCTACCTGCAGGAGATTTTCTCCAACGAGATGGCGAAGGTTGACGATTCTTTCTTCCACAGGCTGGAGGAGTCATTCCTCGTGGAGGAGGATAAGAAGCACGAGCGGCATCCAATCTTCGGCAACATTGTCGACGAGGTTGCCTACCACGAGAAGTACCCTACGATCTACCATCTGCGGAAGAAGCTCGTGGACTCCACAGATAAGGCGGACCTCCGCCTGATCTACCTCGCTCTGGCCCACATGATTAAGTTCAGGGGCCATTTCCTGATCGAGGGGGATCTCAACCCGGACAATAGCGATGTTGACAAGCTGTTCATCCAGCTCGTGCAGACGTACAACCAGCTCTTCGAGGAGAACCCCATTAATGCGTCAGGCGTCGACGCGAAGGCTATCCTGTCCGCTAGGCTCTCGAAGTCTCGGAAGCTCGAGAACCTGATCGCCCAGCTGCCGGGCGAGAAGAAGAACGGCCTGTTCGGGAATCTCATTGCGCTCAGCCTGGGGCTCACGCCCAACTTCAAGTCGAATTTCGATCTCGCTGAGGACGCCAAGCTGCAGCTCTCCAAGGACACATACGACGATGACCTGGATAACCTCCTGGCCCAGATCGGCGATCAGTACGCGGACCTGTTCCTCGCTGCCAAGAATCTGTCGGACGCCATCCTCCTGTCTGATATTCTCAGGGTGAACACCGAGATTACGAAGGCTCCGCTCTCAGCCTCCATGATCAAGCGCTACGACGAGCACCATCAGGATCTGACCCTCCTGAAGGCGCTGGTCAGGCAGCAGCTCCCCGAGAAGTACAAGGAGATCTTCTTCGATCAGTCGAAGAACGGCTACGCTGGGTACATTGACGGCGGGGCCTCTCAGGAGGAGTTCTACAAGTTCATCAAGCCGATTCTGGAGAAGATGGACGGCACGGAGGAGCTGCTGGTGAAGCTCAAGCGCGAGGACCTCCTGAGGAAGCAGCGGACATTCGATAACGGCAGCATCCCACACCAGATTCATCTCGGGGAGCTGCACGCTATCCTGAGGAGGCAGGAGGACTTCTACCCTTTCCTCAAGGATAACCGCGAGAAGATCGAGAAGATTCTGACTTTCAGGATCCCGTACTACGTCGGCCCACTCGCTAGGGGCAACTCCCGCTTCGCTTGGATGACCCGCAAGTCAGAGGAGACGATCACGCCGTGGAACTTCGAGGAGGTGGTCGACAAGGGCGCTAGCGCTCAGTCGTTCATCGAGAGGATGACGAATTTCGACAAGAACCTGCCAAATGAGAAGGTGCTCCCTAAGCACTCGCTCCTGTACGAGTACTTCACAGTCTACAACGAGCTGACTAAGGTGAAGTATGTGACCGAGGGCATGAGGAAGCCGGCTTTCCTGTCTGGGGAGCAGAAGAAGGCCATCGTGGACCTCCTGTTCAAGACCAACCGGAAGGTCACGGTTAAGCAGCTCAAGGAGGACTACTTCAAGAAGATTGAGTGCTTCGATTCGGTCGAGATCTCTGGCGTTGAGGACCGCTTCAACGCCTCCCTGGGGACCTACCACGATCTCCTGAAGATCATTAAGGATAAGGACTTCCTGGACAACGAGGAGAATGAGGATATCCTCGAGGACATTGTGCTGACACTCACTCTGTTCGAGGACCGGGAGATGATCGAGGAGCGCCTGAAGACTTACGCCCATCTCTTCGATGACAAGGTCATGAAGCAGCTCAAGAGGAGGAGGTACACCGGCTGGGGGAGGCTGAGCAGGAAGCTCATCAACGGCATTCGGGACAAGCAGTCCGGGAAGACGATCCTCGACTTCCTGAAGAGCGATGGCTTCGCGAACCGCAATTTCATGCAGCTGATTCACGATGACAGCCTCACATTCAAGGAGGATATCCAGAAGGCTCAGGTGAGCGGCCAGGGGGACTCGCTGCACGAGCATATCGCGAACCTCGCTGGCTCGCCAGCTATCAAGAAGGGGATTCTGCAGACCGTGAAGGTTGTGGACGAGCTGGTGAAGGTCATGGGCAGGCACAAGCCTGAGAACATCGTCATTGAGATGGCCCGGGAGAATCAGACCACGCAGAAGGGCCAGAAGAACTCACGCGAGAGGATGAAGAGGATCGAGGAGGGCATTAAGGAGCTGGGGTCCCAGATCCTCAAGGAGCACCCGGTGGAGAACACGCAGCTGCAGAATGAGAAGCTCTACCTGTACTACCTCCAGAATGGCCGCGATATGTATGTGGACCAGGAGCTGGATATTAACAGGCTCAGCGATTACGACGTCGATGCCATCGTTCCACAGTCATTCCTGAAGGATGACTCCATTGACAACAAGGTCCTCACCAGGTCGGACAAGAACCGGGGCAAGTCTGATAATGTTCCTTCAGAGGAGGTCGTTAAGAAGATGAAGAACTACTGGCGCCAGCTCCTGAATGCCAAGCTGATCACGCAGCGGAAGTTCGATAACCTCACAAAGGCTGAGAGGGGCGGGCTCTCTGAGCTGGACAAGGCGGGCTTCATCAAGAGGCAGCTGGTCGAGACACGGCAGATCACTAAGCACGTTGCGCAGATTCTCGACTCACGGATGAACACTAAGTACGATGAGAATGACAAGCTGATCCGCGAGGTGAAGGTCATCACCCTGAAGTCAAAGCTCGTCTCCGACTTCAGGAAGGATTTCCAGTTCTACAAGGTTCGGGAGATCAACAATTACCACCATGCCCATGACGCGTACCTGAACGCGGTGGTCGGCACAGCTCTGATCAAGAAGTACCCAAAGCTCGAGAGCGAGTTCGTGTACGGGGACTACAAGGTTTACGATGTGAGGAAGATGATCGCCAAGTCGGAGCAGGAGATTGGCAAGGCTACCGCCAAGTACTTCTTCTACTCTAACATTATGAATTTCTTCAAGACAGAGATCACTCTGGCCAATGGCGAGATCCGGAAGCGCCCCCTCATCGAGACGAACGGCGAGACGGGGGAGATCGTGTGGGACAAGGGCAGGGATTTCGCGACCGTCAGGAAGGTTCTCTCCATGCCACAAGTGAATATCGTCAAGAAGACAGAGGTCCAGACTGGCGGGTTCTCTAAGGAGTCAATTCTGCCTAAGCGGAACAGCGACAAGCTCATCGCCCGCAAGAAGGACTGGGATCCGAAGAAGTACGGCGGGTTCGACAGCCCCACTGTGGCCTACTCGGTCCTGGTTGTGGCGAAGGTTGAGAAGGGCAAGTCCAAGAAGCTCAAGAGCGTGAAGGAGCTGCTGGGGATCACGATTATGGAGCGCTCCAGCTTCGAGAAGAACCCGATCGATTTCCTGGAGGCGAAGGGCTACAAGGAGGTGAAGAAGGACCTGATCATTAAGCTCCCCAAGTACTCACTCTTCGAGCTGGAGAACGGCAGGAAGCGGATGCTGGCTTCCGCTGGCGAGCTGCAGAAGGGGAACGAGCTGGCTCTGCCGTCCAAGTATGTGAACTTCCTCTACCTGGCCTCCCACTACGAGAAGCTCAAGGGCAGCCCCGAGGACAACGAGCAGAAGCAGCTGTTCGTCGAGCAGCACAAGCATTACCTCGACGAGATCATTGAGCAGATTTCCGAGTTCTCCAAGCGCGTGATCCTGGCCGACGCGAATCTGGATAAGGTCCTCTCCGCGTACAACAAGCACCGCGACAAGCCAATCAGGGAGCAGGCTGAGAATATCATTCATCTCTTCACCCTGACGAACCTCGGCGCCCCTGCTGCTTTCAAGTACTTCGACACAACTATCGATCGCAAGAGGTACACAAGCACTAAGGAGGTCCTGGACGCGACCCTCATCCACCAGTCGATTACCGGCCTCTACGAGACGCGCATCGACCTGTCTCAGCTCGGGGGCGACGAATTCTCCGGGAGCGAGACGCCAGGCACCTCCGAGTCGGCCACCCCAGAATCTGCCACAGTGGTGTCCGGCCAAAAGCAGGACCGCCAGGGCGGAGAACGCAGAAGGTCCCAGCTCGATAGGGATCAGTGTGCCTACTGCAAGGAGAAGGGCCACTGGGCCAAAGACTGCCCGAAAAAGCCGCGCGGCCCACGCGGCCCAAGGCCACAAACATCCCTCCTTCCAAAGAAGAAGCGGAAGGTGGAGCTCAGCGGAGGATCTTCCGGAGGATCTAGCGGCTCCGAGACACCAGGAACATCCGAAAGCGCTACACCAGAATCTAGCGGAGGCTCTTCCGGAGGATCTAGGCCTGCCGAAAAGATTCAGTGGAGAAATGACATCCCTGTTTGGGTGGATCAATGGAGCCTGCCGAAGGAAAAAATCGAAGCCGCCTCACTGCTCGTCCAGGAGCAGCTGGAAGCGGGCCACCTGGTAGAAAGCCACAGCCCGTGGAACACACCGATTTTCATTATTAGAAAGAAGAGTGGCAAATGGAGACTGCTTCAGGACCTGAGGAAGGTGAATGAGACCATGGTGTTGATGGGAACCCTGCAACCTGGCTTGCCTAGCCCGGTGGCCATCCCAAAGGGCTACTACAAGATCGTCATCGATCTAAAGGACTGCTTCTTCACAATACCTTTGCACCCCAAAGATTGTGAGAGATTCGCGTTCTCCGTGCCGTCTGTCAACTTCAAGGAGCCAATGAAGAGGTACCACTGGACCGTCCTGCCCCAGGGCATGGCGAACTCTCCCACGCTTTGCCAGAGATTTGTCGCCAAGGCTATCCAGCCGGTGCGTCAGCAGTGGCCAAACATCTACATAATCCATTTTACAGATGACGTGCTCATGGCCGGGAAGGATCCACAGGACCTGTTGTTGTGTTATGGTGACCTGCAGAAGGCCCTCGCCGACAAGGGACTCCAAATCGCAAGTGAGAAGATCCAGACTCAGGATCCTTACAACTACCTCGGCTTCCGTCTCACCGACCAGGCGGTGTTCCCGCAGAAGATCGTGATCCGACGCGACAACCTCCTAAACGACTTCCAAAAACTCCTTGGCGATATTAATTGGCTGCGGCCGTACTTAAAGTTAACAACTGGCGAATTAAAGCCTTTGTTCGACATTCTCAAAGGAAGCTCAGACCCAACCTCGCCTAGAAGCCTCACCTCAGAGGGGCTGCTGGCTCTGCAGCTTGTGGAGAAGGCTATCGAGGAACAGTTTGTCACGTACATTGATTATTCACTGCCTCTCCACCTCCTGATATTCAACACCACTCACGTTCCCACCGGCCTGCTTTGGCAGAAGTTTCCTATCATGTGGATACACTCGCGGATCTCGCCTAAAAGAAACATCCTACCTTACCATGAGGCGGTGGCCCAAATGATCATAACTGGTCGGAGGCAGGCTCTCACCTACTTCGGCAAGGAGCCGGACATAATAGTGCAACCATATTCTGTGTCTCAGGACACATGGCTGAAACAACACTCCACCGACTGGCTTCTCGCGCAGCTGGGCTTCGAGGGTACATTAGACTCCCACTATCCCCAAGATCGGTTGATCAAGTTTCTCAACGTGCATGACATGATATTCCCTAAAATGACTAGCCTGCAGCCTTTAAACAATGCACTGCTGATCTTCACCGACGGCTCTTCGAAGGGACGTGCCGGGTACCTCATTTCGAACCAACAGGTGATCGTTGAAACACCCGGCCTGAGCGCACAGCTCGCCGAGCTGACAGCTGTACTGAAGGTTTTCCAGAGTGTGCATGAAGCATTCAATATCTTCACCGACAGCCTCTACGTGGCACAAAGCGTCCCTCTGCTGGAGACCTGCGGCACGTTCAACTTTAACACCCCTTCCGGCAGTCTTTTCTCTGAGTTGCAAAACATCATTCTGGCAAGAAAAAATCCATTCTATATCGGGCACATTAGATCTCATAGCGGACTGCCCGGCCCGCTGGCTGAGGGAAATGATAGAATTGACAGCGGCGGCAGCAAAAGAACGGCGGACGGCTCTGAGAAGCGCACCGCTGATAGCCAGCATTCAACTCCTCCGAAAACAAAGAGGAAAGTTGAGTTCGAACCGAAGAAGAAAAGGAAGGTGTGA

**Sequence 42 Plasmids sequence of ePPEplus-RT41**

(NLSSV40-nCas9(H840A/R221K/N394K)-XTEN-NC-NLS-32aa Linker-RT41-NLSvbp)

CCTAAGAAAAAGAGAAAAGTGGACAAGAAGTACTCGATCGGCCTCGATATTGGGACTAACTCTGTTGGCTGGGCCGTGATCACCGACGAGTACAAGGTGCCCTCAAAGAAGTTCAAGGTCCTGGGCAACACCGATCGGCATTCCATCAAGAAGAATCTCATTGGCGCTCTCCTGTTCGACAGCGGCGAGACGGCTGAGGCTACGCGGCTCAAGCGCACCGCCCGCAGGCGGTACACGCGCAGGAAGAATCGCATCTGCTACCTGCAGGAGATTTTCTCCAACGAGATGGCGAAGGTTGACGATTCTTTCTTCCACAGGCTGGAGGAGTCATTCCTCGTGGAGGAGGATAAGAAGCACGAGCGGCATCCAATCTTCGGCAACATTGTCGACGAGGTTGCCTACCACGAGAAGTACCCTACGATCTACCATCTGCGGAAGAAGCTCGTGGACTCCACAGATAAGGCGGACCTCCGCCTGATCTACCTCGCTCTGGCCCACATGATTAAGTTCAGGGGCCATTTCCTGATCGAGGGGGATCTCAACCCGGACAATAGCGATGTTGACAAGCTGTTCATCCAGCTCGTGCAGACGTACAACCAGCTCTTCGAGGAGAACCCCATTAATGCGTCAGGCGTCGACGCGAAGGCTATCCTGTCCGCTAGGCTCTCGAAGTCTCGGAAGCTCGAGAACCTGATCGCCCAGCTGCCGGGCGAGAAGAAGAACGGCCTGTTCGGGAATCTCATTGCGCTCAGCCTGGGGCTCACGCCCAACTTCAAGTCGAATTTCGATCTCGCTGAGGACGCCAAGCTGCAGCTCTCCAAGGACACATACGACGATGACCTGGATAACCTCCTGGCCCAGATCGGCGATCAGTACGCGGACCTGTTCCTCGCTGCCAAGAATCTGTCGGACGCCATCCTCCTGTCTGATATTCTCAGGGTGAACACCGAGATTACGAAGGCTCCGCTCTCAGCCTCCATGATCAAGCGCTACGACGAGCACCATCAGGATCTGACCCTCCTGAAGGCGCTGGTCAGGCAGCAGCTCCCCGAGAAGTACAAGGAGATCTTCTTCGATCAGTCGAAGAACGGCTACGCTGGGTACATTGACGGCGGGGCCTCTCAGGAGGAGTTCTACAAGTTCATCAAGCCGATTCTGGAGAAGATGGACGGCACGGAGGAGCTGCTGGTGAAGCTCAAGCGCGAGGACCTCCTGAGGAAGCAGCGGACATTCGATAACGGCAGCATCCCACACCAGATTCATCTCGGGGAGCTGCACGCTATCCTGAGGAGGCAGGAGGACTTCTACCCTTTCCTCAAGGATAACCGCGAGAAGATCGAGAAGATTCTGACTTTCAGGATCCCGTACTACGTCGGCCCACTCGCTAGGGGCAACTCCCGCTTCGCTTGGATGACCCGCAAGTCAGAGGAGACGATCACGCCGTGGAACTTCGAGGAGGTGGTCGACAAGGGCGCTAGCGCTCAGTCGTTCATCGAGAGGATGACGAATTTCGACAAGAACCTGCCAAATGAGAAGGTGCTCCCTAAGCACTCGCTCCTGTACGAGTACTTCACAGTCTACAACGAGCTGACTAAGGTGAAGTATGTGACCGAGGGCATGAGGAAGCCGGCTTTCCTGTCTGGGGAGCAGAAGAAGGCCATCGTGGACCTCCTGTTCAAGACCAACCGGAAGGTCACGGTTAAGCAGCTCAAGGAGGACTACTTCAAGAAGATTGAGTGCTTCGATTCGGTCGAGATCTCTGGCGTTGAGGACCGCTTCAACGCCTCCCTGGGGACCTACCACGATCTCCTGAAGATCATTAAGGATAAGGACTTCCTGGACAACGAGGAGAATGAGGATATCCTCGAGGACATTGTGCTGACACTCACTCTGTTCGAGGACCGGGAGATGATCGAGGAGCGCCTGAAGACTTACGCCCATCTCTTCGATGACAAGGTCATGAAGCAGCTCAAGAGGAGGAGGTACACCGGCTGGGGGAGGCTGAGCAGGAAGCTCATCAACGGCATTCGGGACAAGCAGTCCGGGAAGACGATCCTCGACTTCCTGAAGAGCGATGGCTTCGCGAACCGCAATTTCATGCAGCTGATTCACGATGACAGCCTCACATTCAAGGAGGATATCCAGAAGGCTCAGGTGAGCGGCCAGGGGGACTCGCTGCACGAGCATATCGCGAACCTCGCTGGCTCGCCAGCTATCAAGAAGGGGATTCTGCAGACCGTGAAGGTTGTGGACGAGCTGGTGAAGGTCATGGGCAGGCACAAGCCTGAGAACATCGTCATTGAGATGGCCCGGGAGAATCAGACCACGCAGAAGGGCCAGAAGAACTCACGCGAGAGGATGAAGAGGATCGAGGAGGGCATTAAGGAGCTGGGGTCCCAGATCCTCAAGGAGCACCCGGTGGAGAACACGCAGCTGCAGAATGAGAAGCTCTACCTGTACTACCTCCAGAATGGCCGCGATATGTATGTGGACCAGGAGCTGGATATTAACAGGCTCAGCGATTACGACGTCGATGCCATCGTTCCACAGTCATTCCTGAAGGATGACTCCATTGACAACAAGGTCCTCACCAGGTCGGACAAGAACCGGGGCAAGTCTGATAATGTTCCTTCAGAGGAGGTCGTTAAGAAGATGAAGAACTACTGGCGCCAGCTCCTGAATGCCAAGCTGATCACGCAGCGGAAGTTCGATAACCTCACAAAGGCTGAGAGGGGCGGGCTCTCTGAGCTGGACAAGGCGGGCTTCATCAAGAGGCAGCTGGTCGAGACACGGCAGATCACTAAGCACGTTGCGCAGATTCTCGACTCACGGATGAACACTAAGTACGATGAGAATGACAAGCTGATCCGCGAGGTGAAGGTCATCACCCTGAAGTCAAAGCTCGTCTCCGACTTCAGGAAGGATTTCCAGTTCTACAAGGTTCGGGAGATCAACAATTACCACCATGCCCATGACGCGTACCTGAACGCGGTGGTCGGCACAGCTCTGATCAAGAAGTACCCAAAGCTCGAGAGCGAGTTCGTGTACGGGGACTACAAGGTTTACGATGTGAGGAAGATGATCGCCAAGTCGGAGCAGGAGATTGGCAAGGCTACCGCCAAGTACTTCTTCTACTCTAACATTATGAATTTCTTCAAGACAGAGATCACTCTGGCCAATGGCGAGATCCGGAAGCGCCCCCTCATCGAGACGAACGGCGAGACGGGGGAGATCGTGTGGGACAAGGGCAGGGATTTCGCGACCGTCAGGAAGGTTCTCTCCATGCCACAAGTGAATATCGTCAAGAAGACAGAGGTCCAGACTGGCGGGTTCTCTAAGGAGTCAATTCTGCCTAAGCGGAACAGCGACAAGCTCATCGCCCGCAAGAAGGACTGGGATCCGAAGAAGTACGGCGGGTTCGACAGCCCCACTGTGGCCTACTCGGTCCTGGTTGTGGCGAAGGTTGAGAAGGGCAAGTCCAAGAAGCTCAAGAGCGTGAAGGAGCTGCTGGGGATCACGATTATGGAGCGCTCCAGCTTCGAGAAGAACCCGATCGATTTCCTGGAGGCGAAGGGCTACAAGGAGGTGAAGAAGGACCTGATCATTAAGCTCCCCAAGTACTCACTCTTCGAGCTGGAGAACGGCAGGAAGCGGATGCTGGCTTCCGCTGGCGAGCTGCAGAAGGGGAACGAGCTGGCTCTGCCGTCCAAGTATGTGAACTTCCTCTACCTGGCCTCCCACTACGAGAAGCTCAAGGGCAGCCCCGAGGACAACGAGCAGAAGCAGCTGTTCGTCGAGCAGCACAAGCATTACCTCGACGAGATCATTGAGCAGATTTCCGAGTTCTCCAAGCGCGTGATCCTGGCCGACGCGAATCTGGATAAGGTCCTCTCCGCGTACAACAAGCACCGCGACAAGCCAATCAGGGAGCAGGCTGAGAATATCATTCATCTCTTCACCCTGACGAACCTCGGCGCCCCTGCTGCTTTCAAGTACTTCGACACAACTATCGATCGCAAGAGGTACACAAGCACTAAGGAGGTCCTGGACGCGACCCTCATCCACCAGTCGATTACCGGCCTCTACGAGACGCGCATCGACCTGTCTCAGCTCGGGGGCGACGAATTCTCCGGGAGCGAGACGCCAGGCACCTCCGAGTCGGCCACCCCAGAATCTGCCACAGTGGTGTCCGGCCAAAAGCAGGACCGCCAGGGCGGAGAACGCAGAAGGTCCCAGCTCGATAGGGATCAGTGTGCCTACTGCAAGGAGAAGGGCCACTGGGCCAAAGACTGCCCGAAAAAGCCGCGCGGCCCACGCGGCCCAAGGCCACAAACATCCCTCCTTCCAAAGAAGAAGCGGAAGGTGGAGCTCAGCGGAGGATCTTCCGGAGGATCTAGCGGCTCCGAGACACCAGGAACATCCGAAAGCGCTACACCAGAATCTAGCGGAGGCTCTTCCGGAGGATCTAGGCCTGAGAAGAATCTTTTGAATATTATCCTCTCTGATGACAACCTCAATGAAGCGTACAAACGAGTCCACAAGAACAAAGGCGCCTCGGGTGTCGACAAGATGATGACATCCGACCTCAAGCAGCATTTAAAAGAGCACGGCCAGGAGATTAAGGAGCAGATACGGCAAAGGAAGTATAAACCACAACCTGTGCTGAGAGTGGAGATCCCCAAGCCTGATGGAGGTGTACGGCTGCTCGGGATTCCGACGGTGACTGACCGCTTCATCCAGCAGGCGATATCTCAGGTGATCGGGCCCATCTTTGATGAACAGTTCAGTGACTACAGCTATGGATTTAGACCAAAGAGATATGCTGAGATGGCGATAGTACGAGCGCTGGAGCTCATGAATGATGGCTACAAGTGGATTGTTGATATTGATCTTGAGAAGTTCTTCGACACCGTGCACCATGATAAGCTGATGAGGATTGTGAGCAAGACGATAAAGGATGGTGATGTTATTTCTTTGATAAGGAAATATCTCGTCTCCGGGATCGTCATCGACGAGCAATATCATGAATCAATCATTGGAACTCCTCAAGGCGGCAACTTGTCGCCGCTTCTCAGCAACATTATGCTGAATGAGCTGGACAAAGAATTGGAAATCCGCGGCCTCAACTTCGTCCGCTACGCCGACGACTGCCTCATCCTAGTTGGAAGTGAGAAAGCAGCCAAGAGGGTCATGGAACGTATCACAAAATTTATTGAGGAGGAATTGGGCCTGAAGGTTAATGCTACCAAGTCAAAGGTGTCCGAACCAAAAGATATCAAGTTTCTTGGCTTCGCCTTCTACTGGAACAAGTACCAGTACCAATTCAAGGCTAGCGGCGGCAGCAAAAGAACGGCGGACGGCTCTGAGAAGCGCACCGCTGATAGCCAGCATTCAACTCCTCCGAAAACAAAGAGGAAAGTTGAGTTCGAACCGAAGAAGAAAAGGAAGGTGTGA

**Sequence 43 Plasmids sequence of ePPEplus-RT42**

(NLSSV40-nCas9(H840A/R221K/N394K)-XTEN-NC-NLS-32aa Linker-RT42-NLSvbp)

CCTAAGAAAAAGAGAAAAGTGGACAAGAAGTACTCGATCGGCCTCGATATTGGGACTAACTCTGTTGGCTGGGCCGTGATCACCGACGAGTACAAGGTGCCCTCAAAGAAGTTCAAGGTCCTGGGCAACACCGATCGGCATTCCATCAAGAAGAATCTCATTGGCGCTCTCCTGTTCGACAGCGGCGAGACGGCTGAGGCTACGCGGCTCAAGCGCACCGCCCGCAGGCGGTACACGCGCAGGAAGAATCGCATCTGCTACCTGCAGGAGATTTTCTCCAACGAGATGGCGAAGGTTGACGATTCTTTCTTCCACAGGCTGGAGGAGTCATTCCTCGTGGAGGAGGATAAGAAGCACGAGCGGCATCCAATCTTCGGCAACATTGTCGACGAGGTTGCCTACCACGAGAAGTACCCTACGATCTACCATCTGCGGAAGAAGCTCGTGGACTCCACAGATAAGGCGGACCTCCGCCTGATCTACCTCGCTCTGGCCCACATGATTAAGTTCAGGGGCCATTTCCTGATCGAGGGGGATCTCAACCCGGACAATAGCGATGTTGACAAGCTGTTCATCCAGCTCGTGCAGACGTACAACCAGCTCTTCGAGGAGAACCCCATTAATGCGTCAGGCGTCGACGCGAAGGCTATCCTGTCCGCTAGGCTCTCGAAGTCTCGGAAGCTCGAGAACCTGATCGCCCAGCTGCCGGGCGAGAAGAAGAACGGCCTGTTCGGGAATCTCATTGCGCTCAGCCTGGGGCTCACGCCCAACTTCAAGTCGAATTTCGATCTCGCTGAGGACGCCAAGCTGCAGCTCTCCAAGGACACATACGACGATGACCTGGATAACCTCCTGGCCCAGATCGGCGATCAGTACGCGGACCTGTTCCTCGCTGCCAAGAATCTGTCGGACGCCATCCTCCTGTCTGATATTCTCAGGGTGAACACCGAGATTACGAAGGCTCCGCTCTCAGCCTCCATGATCAAGCGCTACGACGAGCACCATCAGGATCTGACCCTCCTGAAGGCGCTGGTCAGGCAGCAGCTCCCCGAGAAGTACAAGGAGATCTTCTTCGATCAGTCGAAGAACGGCTACGCTGGGTACATTGACGGCGGGGCCTCTCAGGAGGAGTTCTACAAGTTCATCAAGCCGATTCTGGAGAAGATGGACGGCACGGAGGAGCTGCTGGTGAAGCTCAAGCGCGAGGACCTCCTGAGGAAGCAGCGGACATTCGATAACGGCAGCATCCCACACCAGATTCATCTCGGGGAGCTGCACGCTATCCTGAGGAGGCAGGAGGACTTCTACCCTTTCCTCAAGGATAACCGCGAGAAGATCGAGAAGATTCTGACTTTCAGGATCCCGTACTACGTCGGCCCACTCGCTAGGGGCAACTCCCGCTTCGCTTGGATGACCCGCAAGTCAGAGGAGACGATCACGCCGTGGAACTTCGAGGAGGTGGTCGACAAGGGCGCTAGCGCTCAGTCGTTCATCGAGAGGATGACGAATTTCGACAAGAACCTGCCAAATGAGAAGGTGCTCCCTAAGCACTCGCTCCTGTACGAGTACTTCACAGTCTACAACGAGCTGACTAAGGTGAAGTATGTGACCGAGGGCATGAGGAAGCCGGCTTTCCTGTCTGGGGAGCAGAAGAAGGCCATCGTGGACCTCCTGTTCAAGACCAACCGGAAGGTCACGGTTAAGCAGCTCAAGGAGGACTACTTCAAGAAGATTGAGTGCTTCGATTCGGTCGAGATCTCTGGCGTTGAGGACCGCTTCAACGCCTCCCTGGGGACCTACCACGATCTCCTGAAGATCATTAAGGATAAGGACTTCCTGGACAACGAGGAGAATGAGGATATCCTCGAGGACATTGTGCTGACACTCACTCTGTTCGAGGACCGGGAGATGATCGAGGAGCGCCTGAAGACTTACGCCCATCTCTTCGATGACAAGGTCATGAAGCAGCTCAAGAGGAGGAGGTACACCGGCTGGGGGAGGCTGAGCAGGAAGCTCATCAACGGCATTCGGGACAAGCAGTCCGGGAAGACGATCCTCGACTTCCTGAAGAGCGATGGCTTCGCGAACCGCAATTTCATGCAGCTGATTCACGATGACAGCCTCACATTCAAGGAGGATATCCAGAAGGCTCAGGTGAGCGGCCAGGGGGACTCGCTGCACGAGCATATCGCGAACCTCGCTGGCTCGCCAGCTATCAAGAAGGGGATTCTGCAGACCGTGAAGGTTGTGGACGAGCTGGTGAAGGTCATGGGCAGGCACAAGCCTGAGAACATCGTCATTGAGATGGCCCGGGAGAATCAGACCACGCAGAAGGGCCAGAAGAACTCACGCGAGAGGATGAAGAGGATCGAGGAGGGCATTAAGGAGCTGGGGTCCCAGATCCTCAAGGAGCACCCGGTGGAGAACACGCAGCTGCAGAATGAGAAGCTCTACCTGTACTACCTCCAGAATGGCCGCGATATGTATGTGGACCAGGAGCTGGATATTAACAGGCTCAGCGATTACGACGTCGATGCCATCGTTCCACAGTCATTCCTGAAGGATGACTCCATTGACAACAAGGTCCTCACCAGGTCGGACAAGAACCGGGGCAAGTCTGATAATGTTCCTTCAGAGGAGGTCGTTAAGAAGATGAAGAACTACTGGCGCCAGCTCCTGAATGCCAAGCTGATCACGCAGCGGAAGTTCGATAACCTCACAAAGGCTGAGAGGGGCGGGCTCTCTGAGCTGGACAAGGCGGGCTTCATCAAGAGGCAGCTGGTCGAGACACGGCAGATCACTAAGCACGTTGCGCAGATTCTCGACTCACGGATGAACACTAAGTACGATGAGAATGACAAGCTGATCCGCGAGGTGAAGGTCATCACCCTGAAGTCAAAGCTCGTCTCCGACTTCAGGAAGGATTTCCAGTTCTACAAGGTTCGGGAGATCAACAATTACCACCATGCCCATGACGCGTACCTGAACGCGGTGGTCGGCACAGCTCTGATCAAGAAGTACCCAAAGCTCGAGAGCGAGTTCGTGTACGGGGACTACAAGGTTTACGATGTGAGGAAGATGATCGCCAAGTCGGAGCAGGAGATTGGCAAGGCTACCGCCAAGTACTTCTTCTACTCTAACATTATGAATTTCTTCAAGACAGAGATCACTCTGGCCAATGGCGAGATCCGGAAGCGCCCCCTCATCGAGACGAACGGCGAGACGGGGGAGATCGTGTGGGACAAGGGCAGGGATTTCGCGACCGTCAGGAAGGTTCTCTCCATGCCACAAGTGAATATCGTCAAGAAGACAGAGGTCCAGACTGGCGGGTTCTCTAAGGAGTCAATTCTGCCTAAGCGGAACAGCGACAAGCTCATCGCCCGCAAGAAGGACTGGGATCCGAAGAAGTACGGCGGGTTCGACAGCCCCACTGTGGCCTACTCGGTCCTGGTTGTGGCGAAGGTTGAGAAGGGCAAGTCCAAGAAGCTCAAGAGCGTGAAGGAGCTGCTGGGGATCACGATTATGGAGCGCTCCAGCTTCGAGAAGAACCCGATCGATTTCCTGGAGGCGAAGGGCTACAAGGAGGTGAAGAAGGACCTGATCATTAAGCTCCCCAAGTACTCACTCTTCGAGCTGGAGAACGGCAGGAAGCGGATGCTGGCTTCCGCTGGCGAGCTGCAGAAGGGGAACGAGCTGGCTCTGCCGTCCAAGTATGTGAACTTCCTCTACCTGGCCTCCCACTACGAGAAGCTCAAGGGCAGCCCCGAGGACAACGAGCAGAAGCAGCTGTTCGTCGAGCAGCACAAGCATTACCTCGACGAGATCATTGAGCAGATTTCCGAGTTCTCCAAGCGCGTGATCCTGGCCGACGCGAATCTGGATAAGGTCCTCTCCGCGTACAACAAGCACCGCGACAAGCCAATCAGGGAGCAGGCTGAGAATATCATTCATCTCTTCACCCTGACGAACCTCGGCGCCCCTGCTGCTTTCAAGTACTTCGACACAACTATCGATCGCAAGAGGTACACAAGCACTAAGGAGGTCCTGGACGCGACCCTCATCCACCAGTCGATTACCGGCCTCTACGAGACGCGCATCGACCTGTCTCAGCTCGGGGGCGACGAATTCTCCGGGAGCGAGACGCCAGGCACCTCCGAGTCGGCCACCCCAGAATCTGCCACAGTGGTGTCCGGCCAAAAGCAGGACCGCCAGGGCGGAGAACGCAGAAGGTCCCAGCTCGATAGGGATCAGTGTGCCTACTGCAAGGAGAAGGGCCACTGGGCCAAAGACTGCCCGAAAAAGCCGCGCGGCCCACGCGGCCCAAGGCCACAAACATCCCTCCTTCCAAAGAAGAAGCGGAAGGTGGAGCTCAGCGGAGGATCTTCCGGAGGATCTAGCGGCTCCGAGACACCAGGAACATCCGAAAGCGCTACACCAGAATCTAGCGGAGGCTCTTCCGGAGGATCTAGGCCTGTTCCACTGGAAGAGGAGTTCAAACTTATCGGAGAGGAGGGCAAATCGCCTGAACAGAAGTGGAATTATCTGCGGGAAAAGTTCCCTCAGGTCTGGGCTGAAACAAATTTGCCGGGCCTGGCAAAGCATCAGGTACCCATTGTAGTGCAGGTGAGAGCGACAGCAAGCCCGGTGCGCGTGCGGCAATACCCCCTGAGACTGGAAGCCAAAATTAAGATATCGCAGCACATCCATCTCCTGAAGGCTGGCATCTTGATCCCTTGTCAGAGCGCATGGAACACCCCCCTGCTGCCTGTGCGCAAGCCAGGAACCTCTGACTACCGGCCAGTCCAAGACCTGAGAGAAGTTAACTCGCGTGTAGAGACGATTCACCCTACGGTGCCCAACCCGTACACCTTGCTCAGCCTCATCCCGCCTGTGCACATTTGGTACTCAGTGCTCGACCTTAAGGATGCGTTCTTCTCGCTCCCCTTAGCTCCTGTGAGCCAGCCTCTCTTCGCATTTGAGTGGACAGACCCGGACACCGGCACAACCGGGCAGCTGACTTGGACTAGACTACCACAAGGATTCAAGAATTCTCCTACCCTGTTTGGAGAAGCTCTTTCCAAAGATCTGCAGGAGGTGAGGGAGGCCTTCCCTTTCGCTACCATCTTGCAGTATGTGGATGACCTGCTGGTGGCCACCAGCACAGAGAAGGAATGTTTGACAGACACCGAGGGACTGCTGGAGGCGCTGCAGGATTTAGGTTACAGGGTGTCGTGGAAGAAGGCGCAGCTGTGTCAAACAAAGGTGACTTACCTTGGCTACATCTTAGAAAAGGGCGAGAGGAAACTGGCCGAGTCTAGAGTAAGCGCCATCCTGCAGATACCGGTGCCGTGCAGCAAGAAACAAGTTCGAGAGTTCCTCGGCGCCATCGGATACTGCCGGATATGGATTCCTAGTTTTGCCGAAATCGCGCGGCCTCTGCATGAGGCCACCGCGGGCGGCAACACACCTTTGCAGTGGACTGAAGCATGCCAACAGGCGTTCCAGACACTTAAGACGGCGCTGACCACCGCCCCCGCCTTAGCCCTTCCTGATCTGACTAAACCGTTCACCCTATACGTGGCAGAAAAAAATGGTGTTGCCAAAGGAGTCGTCACGCAGCCGCTCGGACCATGGAAAAGACCTATCGCATATTTAAGTAAGAGACTCGATACTGTTGCGAGAGGGTGGCCGAGCTGCCTCCGCATGATCGCAGCCGCCGCCACGTTGACACGGGAAGCCGACAAGTTGACATTTGGTCAAGACCTGACCCTGATAACGCCTCACGCCATTGAACCTCTTCTCCACTCCAGCCCTAGCCGGTGGCTCTCCAACGCCAGACTGCTGCAGTATCAAGGCCTTCTGATTGAGCAGCCCCGCATCAAATTCGCAACCACCAGCAACCTTAACCCTGCCTCTCTGCTGCCAGAGTCAGATGATCAGCTGCCTATCCATGACTGCCTGGAGACCCTTGAGAACCTTAGAGGCGGCCGCCCCGACTTAACCGATCTTGCTTTGCCGCAGCCAGAGGCAACATATTACACTGATGGTTGCTCCTTCGTTCGTGAGGGTGTGAGATATGCCGGCGCCGCCATAGTCGACCAAAATCAGCAGATGGTGTGGGAGCAAGGCCTGCCTCAAGGCACCAGCGCTCAGAAAGCTGAGTTGATCGCCCTAACAAAAGCCCTGGAGCTCGGAAAGGGAAAGAGAATCAATATCTTCACAGATTCTCGCTACGCGTTTGCCACGGCGCACGTCCACGGTCAGATATACCAGAACAGAGGCCTGCTCACCAGCTCGGGGAAGGAAATCAAGAACAAGACAGAAATTCTTGCCCTGCTGCAGGCCATCTGGCTGCCCCGGGAGGTGGCCATCATCCACTGCCCTGGCCACCAAAGAAACAGTTCCCCAGAGGCTATCGGCAACAATGCTGCTGACCGCGCTGCCAAGCAGGCCGCTCAGAAGAGTGTGGGCAAGGAGCTTGTGCTATTACCAACACCAGTGCTGCCCGAAAGACCCGAGTACACCGAGGAAGAAAGCGGCGGCAGCAAAAGAACGGCGGACGGCTCTGAGAAGCGCACCGCTGATAGCCAGCATTCAACTCCTCCGAAAACAAAGAGGAAAGTTGAGTTCGAACCGAAGAAGAAAAGGAAGGTGTGA

**Sequence 44 Plasmids sequence of ePPEplus-RT43**

(NLSSV40-nCas9(H840A/R221K/N394K)-XTEN-NC-NLS-32aa Linker-RT43-NLSvbp)

CCTAAGAAAAAGAGAAAAGTGGACAAGAAGTACTCGATCGGCCTCGATATTGGGACTAACTCTGTTGGCTGGGCCGTGATCACCGACGAGTACAAGGTGCCCTCAAAGAAGTTCAAGGTCCTGGGCAACACCGATCGGCATTCCATCAAGAAGAATCTCATTGGCGCTCTCCTGTTCGACAGCGGCGAGACGGCTGAGGCTACGCGGCTCAAGCGCACCGCCCGCAGGCGGTACACGCGCAGGAAGAATCGCATCTGCTACCTGCAGGAGATTTTCTCCAACGAGATGGCGAAGGTTGACGATTCTTTCTTCCACAGGCTGGAGGAGTCATTCCTCGTGGAGGAGGATAAGAAGCACGAGCGGCATCCAATCTTCGGCAACATTGTCGACGAGGTTGCCTACCACGAGAAGTACCCTACGATCTACCATCTGCGGAAGAAGCTCGTGGACTCCACAGATAAGGCGGACCTCCGCCTGATCTACCTCGCTCTGGCCCACATGATTAAGTTCAGGGGCCATTTCCTGATCGAGGGGGATCTCAACCCGGACAATAGCGATGTTGACAAGCTGTTCATCCAGCTCGTGCAGACGTACAACCAGCTCTTCGAGGAGAACCCCATTAATGCGTCAGGCGTCGACGCGAAGGCTATCCTGTCCGCTAGGCTCTCGAAGTCTCGGAAGCTCGAGAACCTGATCGCCCAGCTGCCGGGCGAGAAGAAGAACGGCCTGTTCGGGAATCTCATTGCGCTCAGCCTGGGGCTCACGCCCAACTTCAAGTCGAATTTCGATCTCGCTGAGGACGCCAAGCTGCAGCTCTCCAAGGACACATACGACGATGACCTGGATAACCTCCTGGCCCAGATCGGCGATCAGTACGCGGACCTGTTCCTCGCTGCCAAGAATCTGTCGGACGCCATCCTCCTGTCTGATATTCTCAGGGTGAACACCGAGATTACGAAGGCTCCGCTCTCAGCCTCCATGATCAAGCGCTACGACGAGCACCATCAGGATCTGACCCTCCTGAAGGCGCTGGTCAGGCAGCAGCTCCCCGAGAAGTACAAGGAGATCTTCTTCGATCAGTCGAAGAACGGCTACGCTGGGTACATTGACGGCGGGGCCTCTCAGGAGGAGTTCTACAAGTTCATCAAGCCGATTCTGGAGAAGATGGACGGCACGGAGGAGCTGCTGGTGAAGCTCAAGCGCGAGGACCTCCTGAGGAAGCAGCGGACATTCGATAACGGCAGCATCCCACACCAGATTCATCTCGGGGAGCTGCACGCTATCCTGAGGAGGCAGGAGGACTTCTACCCTTTCCTCAAGGATAACCGCGAGAAGATCGAGAAGATTCTGACTTTCAGGATCCCGTACTACGTCGGCCCACTCGCTAGGGGCAACTCCCGCTTCGCTTGGATGACCCGCAAGTCAGAGGAGACGATCACGCCGTGGAACTTCGAGGAGGTGGTCGACAAGGGCGCTAGCGCTCAGTCGTTCATCGAGAGGATGACGAATTTCGACAAGAACCTGCCAAATGAGAAGGTGCTCCCTAAGCACTCGCTCCTGTACGAGTACTTCACAGTCTACAACGAGCTGACTAAGGTGAAGTATGTGACCGAGGGCATGAGGAAGCCGGCTTTCCTGTCTGGGGAGCAGAAGAAGGCCATCGTGGACCTCCTGTTCAAGACCAACCGGAAGGTCACGGTTAAGCAGCTCAAGGAGGACTACTTCAAGAAGATTGAGTGCTTCGATTCGGTCGAGATCTCTGGCGTTGAGGACCGCTTCAACGCCTCCCTGGGGACCTACCACGATCTCCTGAAGATCATTAAGGATAAGGACTTCCTGGACAACGAGGAGAATGAGGATATCCTCGAGGACATTGTGCTGACACTCACTCTGTTCGAGGACCGGGAGATGATCGAGGAGCGCCTGAAGACTTACGCCCATCTCTTCGATGACAAGGTCATGAAGCAGCTCAAGAGGAGGAGGTACACCGGCTGGGGGAGGCTGAGCAGGAAGCTCATCAACGGCATTCGGGACAAGCAGTCCGGGAAGACGATCCTCGACTTCCTGAAGAGCGATGGCTTCGCGAACCGCAATTTCATGCAGCTGATTCACGATGACAGCCTCACATTCAAGGAGGATATCCAGAAGGCTCAGGTGAGCGGCCAGGGGGACTCGCTGCACGAGCATATCGCGAACCTCGCTGGCTCGCCAGCTATCAAGAAGGGGATTCTGCAGACCGTGAAGGTTGTGGACGAGCTGGTGAAGGTCATGGGCAGGCACAAGCCTGAGAACATCGTCATTGAGATGGCCCGGGAGAATCAGACCACGCAGAAGGGCCAGAAGAACTCACGCGAGAGGATGAAGAGGATCGAGGAGGGCATTAAGGAGCTGGGGTCCCAGATCCTCAAGGAGCACCCGGTGGAGAACACGCAGCTGCAGAATGAGAAGCTCTACCTGTACTACCTCCAGAATGGCCGCGATATGTATGTGGACCAGGAGCTGGATATTAACAGGCTCAGCGATTACGACGTCGATGCCATCGTTCCACAGTCATTCCTGAAGGATGACTCCATTGACAACAAGGTCCTCACCAGGTCGGACAAGAACCGGGGCAAGTCTGATAATGTTCCTTCAGAGGAGGTCGTTAAGAAGATGAAGAACTACTGGCGCCAGCTCCTGAATGCCAAGCTGATCACGCAGCGGAAGTTCGATAACCTCACAAAGGCTGAGAGGGGCGGGCTCTCTGAGCTGGACAAGGCGGGCTTCATCAAGAGGCAGCTGGTCGAGACACGGCAGATCACTAAGCACGTTGCGCAGATTCTCGACTCACGGATGAACACTAAGTACGATGAGAATGACAAGCTGATCCGCGAGGTGAAGGTCATCACCCTGAAGTCAAAGCTCGTCTCCGACTTCAGGAAGGATTTCCAGTTCTACAAGGTTCGGGAGATCAACAATTACCACCATGCCCATGACGCGTACCTGAACGCGGTGGTCGGCACAGCTCTGATCAAGAAGTACCCAAAGCTCGAGAGCGAGTTCGTGTACGGGGACTACAAGGTTTACGATGTGAGGAAGATGATCGCCAAGTCGGAGCAGGAGATTGGCAAGGCTACCGCCAAGTACTTCTTCTACTCTAACATTATGAATTTCTTCAAGACAGAGATCACTCTGGCCAATGGCGAGATCCGGAAGCGCCCCCTCATCGAGACGAACGGCGAGACGGGGGAGATCGTGTGGGACAAGGGCAGGGATTTCGCGACCGTCAGGAAGGTTCTCTCCATGCCACAAGTGAATATCGTCAAGAAGACAGAGGTCCAGACTGGCGGGTTCTCTAAGGAGTCAATTCTGCCTAAGCGGAACAGCGACAAGCTCATCGCCCGCAAGAAGGACTGGGATCCGAAGAAGTACGGCGGGTTCGACAGCCCCACTGTGGCCTACTCGGTCCTGGTTGTGGCGAAGGTTGAGAAGGGCAAGTCCAAGAAGCTCAAGAGCGTGAAGGAGCTGCTGGGGATCACGATTATGGAGCGCTCCAGCTTCGAGAAGAACCCGATCGATTTCCTGGAGGCGAAGGGCTACAAGGAGGTGAAGAAGGACCTGATCATTAAGCTCCCCAAGTACTCACTCTTCGAGCTGGAGAACGGCAGGAAGCGGATGCTGGCTTCCGCTGGCGAGCTGCAGAAGGGGAACGAGCTGGCTCTGCCGTCCAAGTATGTGAACTTCCTCTACCTGGCCTCCCACTACGAGAAGCTCAAGGGCAGCCCCGAGGACAACGAGCAGAAGCAGCTGTTCGTCGAGCAGCACAAGCATTACCTCGACGAGATCATTGAGCAGATTTCCGAGTTCTCCAAGCGCGTGATCCTGGCCGACGCGAATCTGGATAAGGTCCTCTCCGCGTACAACAAGCACCGCGACAAGCCAATCAGGGAGCAGGCTGAGAATATCATTCATCTCTTCACCCTGACGAACCTCGGCGCCCCTGCTGCTTTCAAGTACTTCGACACAACTATCGATCGCAAGAGGTACACAAGCACTAAGGAGGTCCTGGACGCGACCCTCATCCACCAGTCGATTACCGGCCTCTACGAGACGCGCATCGACCTGTCTCAGCTCGGGGGCGACGAATTCTCCGGGAGCGAGACGCCAGGCACCTCCGAGTCGGCCACCCCAGAATCTGCCACAGTGGTGTCCGGCCAAAAGCAGGACCGCCAGGGCGGAGAACGCAGAAGGTCCCAGCTCGATAGGGATCAGTGTGCCTACTGCAAGGAGAAGGGCCACTGGGCCAAAGACTGCCCGAAAAAGCCGCGCGGCCCACGCGGCCCAAGGCCACAAACATCCCTCCTTCCAAAGAAGAAGCGGAAGGTGGAGCTCAGCGGAGGATCTTCCGGAGGATCTAGCGGCTCCGAGACACCAGGAACATCCGAAAGCGCTACACCAGAATCTAGCGGAGGCTCTTCCGGAGGATCTAGGCCTCCTAGCAAGGGCCCCAAGGTGACTTGGGAGGAGGCGCCCGTGGCGTGCTTAGTGCTCAGCCTGGAGGAAGAGTATCGGCTACATGAACAAAATCCAAAGCAACTGCTGGCTCCTGAATGGCTCTCCGCCTTCTCTGAAGTTTGGGCTGAGCAGACGGGCATGGGGTTAGCCAAACAGGTGCCGCCGGTGGTTGTGGAGCTGAAGGCCGACGCTTCCCCGGTGAGCGTAAAGCAGTACCCGATGTCGCGGGAAGCAAAGGAAGGCATCAGACCTCACATTCAAAGGCTGCTGCAGCTCGGGATCCTGGTGCCATGCCAGAGCCCATGGAACACACCTCTCCTTCCTGTGAGGAAGCCGGGCACCAATGACTACCGGCCCGTGCAGGATTTGCGGGAGGTCAATAAGAGGGTTCAGGACATCCACCCTACCGTGCCAAACCCCTACAACCTTCTGAGCAGCCTGCCACCGGAGAGGACCTGGTACACCGTGCTTGACCTCAAGGATGCATTCTTCTGCCTCAGATTGCACCCGAACTCCCAGCCACTGTTTGCTTTCGAGTGGAGGGATCCTGAGGGTGGCCATACCGGTCAGCTGACTTGGACGCGACTGCCACAGGGATTTAAGAATAGCCCAACCTTATTTGATGAGGCACTCCACCGCGACCTGGCGCCCTTCAGAGCTCAGAACCCTCAGATTAGTTTGCTGCAGTATGTGGACGACCTGCTGCTGGCCGCAAGCACTCAGGAACTCTGCAGGGAAGGCACCAAACGGCTGCTGAACGAACTGGGCGAGTTGGGCTATCGCGTCTCAGCTAAGAAGGCCCAGCTTTGTCGTACTGAGGTAACATACCTGGGGTACACCCTGAGAGAGGGCAAGCGGTGGCTGACCGAAGCCAGAAAGAAGACGGTGATGCAAATCCCAACTCCCACAACCCCTCGCCAGGTGAGAGAATTTCTGGGTACCGCCGGCTTCTGTCGGCTATGGATTCCTGGATTCGCCACCCTGGCTGCACCCCTCTACCTTCTGACCAAGGAGAAGGTTCCCTTCACATGGACAGAAGAGCATCAGCGTGCTTTTGAAGATATCAAGGCTGCTCTGCTCGCCGCCCCTGCCCTGGCTCTGCCAGACCTCACTAAGCCCTTCACGCTTTACGTGGATGAAAGAGCCGGCGTCGCCCGTGGCGTGCTCACCCAGGCCCTCGGCCCGTGGAAGAGGCCCGTCGCCTACCTGAGCAAGAAGCTGGACCCGGTGGCCAGCGGCTGGCCATCCTGCCTGAAAGCCATCGCCGCCGTGGCGCTGCTGGTGAAGGACGCCGACAAGCTGACCCTGGGACAGCACGTCACAGTCATCGCCCCCCACGCCCTGGAGTCCATCGTCAGACAACCACCTGATCGATGGATGACAAATGCTAGAATGACGCACTACCAGTCCCTACTTCTAAATGAGCGGGTGACGTTCGCGCCTCCTGCAATTCTCAACCCCGCCACGCTGCTGCCAGAGATCCACAACTCGACTCCTATCCACCAGTGTGTCGACATCCTGGCGGAGGAGACCGGCACCAGGAAAGATCTGACCGACAGACCTTGGCCTGGAGTCCCGGCGTGGTATACAGACGGCAGTTCTTTTGTGGTAGAGGGAAAGCGCCGGGCCGGCGCAGCCGTGGTGGATGGGAAGCAGGTAATCTGGGCCAGCAGTCTGCCAGAGGGAACGTCTGCACAGAAAGCTGAGCTCGTGGCCCTAACCCAGGCTCTCCGGCTGGCCGAAGGCAAGGCCATCAACATTTACACTGACAGCCGCTATGCGTTTGCTACAGCCCACATCCACGGCGCCATCTACAAGCAACGTGGACTACTCACAAGCGCGGGCAGAGACATTAAAAACAAAGAAGAAATCCTCGCCCTGCTGGAGGCCGTCCATCTCCCGAAGAAGGTCGCTATAATTCACTGCCCTGGGCACCAAAAAGGAGAGGACCCCATCACAAAAGGGAACCAAATGGCGGACCTCGTTGCAAAACAGGTCGCCCAGCAAGTGACCATCCTCGCAGAGAAATCTCAAACACCTGTGAAAACACCTGTGACCGATGATAGTAACTACATCTACTCAGCCAATGACTTCAAGATTCTGGAGAACATGGTATACAGAAAACAGGCGCAGGAGTTCTCCTTCGACGGTAGCGGCGGCAGCAAAAGAACGGCGGACGGCTCTGAGAAGCGCACCGCTGATAGCCAGCATTCAACTCCTCCGAAAACAAAGAGGAAAGTTGAGTTCGAACCGAAGAAGAAAAGGAAGGTGTGA

**Sequence 45 Plasmids sequence of ePPEplus-RT44**

(NLSSV40-nCas9(H840A/R221K/N394K)-XTEN-NC-NLS-32aa Linker-RT44-NLSvbp)

CCTAAGAAAAAGAGAAAAGTGGACAAGAAGTACTCGATCGGCCTCGATATTGGGACTAACTCTGTTGGCTGGGCCGTGATCACCGACGAGTACAAGGTGCCCTCAAAGAAGTTCAAGGTCCTGGGCAACACCGATCGGCATTCCATCAAGAAGAATCTCATTGGCGCTCTCCTGTTCGACAGCGGCGAGACGGCTGAGGCTACGCGGCTCAAGCGCACCGCCCGCAGGCGGTACACGCGCAGGAAGAATCGCATCTGCTACCTGCAGGAGATTTTCTCCAACGAGATGGCGAAGGTTGACGATTCTTTCTTCCACAGGCTGGAGGAGTCATTCCTCGTGGAGGAGGATAAGAAGCACGAGCGGCATCCAATCTTCGGCAACATTGTCGACGAGGTTGCCTACCACGAGAAGTACCCTACGATCTACCATCTGCGGAAGAAGCTCGTGGACTCCACAGATAAGGCGGACCTCCGCCTGATCTACCTCGCTCTGGCCCACATGATTAAGTTCAGGGGCCATTTCCTGATCGAGGGGGATCTCAACCCGGACAATAGCGATGTTGACAAGCTGTTCATCCAGCTCGTGCAGACGTACAACCAGCTCTTCGAGGAGAACCCCATTAATGCGTCAGGCGTCGACGCGAAGGCTATCCTGTCCGCTAGGCTCTCGAAGTCTCGGAAGCTCGAGAACCTGATCGCCCAGCTGCCGGGCGAGAAGAAGAACGGCCTGTTCGGGAATCTCATTGCGCTCAGCCTGGGGCTCACGCCCAACTTCAAGTCGAATTTCGATCTCGCTGAGGACGCCAAGCTGCAGCTCTCCAAGGACACATACGACGATGACCTGGATAACCTCCTGGCCCAGATCGGCGATCAGTACGCGGACCTGTTCCTCGCTGCCAAGAATCTGTCGGACGCCATCCTCCTGTCTGATATTCTCAGGGTGAACACCGAGATTACGAAGGCTCCGCTCTCAGCCTCCATGATCAAGCGCTACGACGAGCACCATCAGGATCTGACCCTCCTGAAGGCGCTGGTCAGGCAGCAGCTCCCCGAGAAGTACAAGGAGATCTTCTTCGATCAGTCGAAGAACGGCTACGCTGGGTACATTGACGGCGGGGCCTCTCAGGAGGAGTTCTACAAGTTCATCAAGCCGATTCTGGAGAAGATGGACGGCACGGAGGAGCTGCTGGTGAAGCTCAAGCGCGAGGACCTCCTGAGGAAGCAGCGGACATTCGATAACGGCAGCATCCCACACCAGATTCATCTCGGGGAGCTGCACGCTATCCTGAGGAGGCAGGAGGACTTCTACCCTTTCCTCAAGGATAACCGCGAGAAGATCGAGAAGATTCTGACTTTCAGGATCCCGTACTACGTCGGCCCACTCGCTAGGGGCAACTCCCGCTTCGCTTGGATGACCCGCAAGTCAGAGGAGACGATCACGCCGTGGAACTTCGAGGAGGTGGTCGACAAGGGCGCTAGCGCTCAGTCGTTCATCGAGAGGATGACGAATTTCGACAAGAACCTGCCAAATGAGAAGGTGCTCCCTAAGCACTCGCTCCTGTACGAGTACTTCACAGTCTACAACGAGCTGACTAAGGTGAAGTATGTGACCGAGGGCATGAGGAAGCCGGCTTTCCTGTCTGGGGAGCAGAAGAAGGCCATCGTGGACCTCCTGTTCAAGACCAACCGGAAGGTCACGGTTAAGCAGCTCAAGGAGGACTACTTCAAGAAGATTGAGTGCTTCGATTCGGTCGAGATCTCTGGCGTTGAGGACCGCTTCAACGCCTCCCTGGGGACCTACCACGATCTCCTGAAGATCATTAAGGATAAGGACTTCCTGGACAACGAGGAGAATGAGGATATCCTCGAGGACATTGTGCTGACACTCACTCTGTTCGAGGACCGGGAGATGATCGAGGAGCGCCTGAAGACTTACGCCCATCTCTTCGATGACAAGGTCATGAAGCAGCTCAAGAGGAGGAGGTACACCGGCTGGGGGAGGCTGAGCAGGAAGCTCATCAACGGCATTCGGGACAAGCAGTCCGGGAAGACGATCCTCGACTTCCTGAAGAGCGATGGCTTCGCGAACCGCAATTTCATGCAGCTGATTCACGATGACAGCCTCACATTCAAGGAGGATATCCAGAAGGCTCAGGTGAGCGGCCAGGGGGACTCGCTGCACGAGCATATCGCGAACCTCGCTGGCTCGCCAGCTATCAAGAAGGGGATTCTGCAGACCGTGAAGGTTGTGGACGAGCTGGTGAAGGTCATGGGCAGGCACAAGCCTGAGAACATCGTCATTGAGATGGCCCGGGAGAATCAGACCACGCAGAAGGGCCAGAAGAACTCACGCGAGAGGATGAAGAGGATCGAGGAGGGCATTAAGGAGCTGGGGTCCCAGATCCTCAAGGAGCACCCGGTGGAGAACACGCAGCTGCAGAATGAGAAGCTCTACCTGTACTACCTCCAGAATGGCCGCGATATGTATGTGGACCAGGAGCTGGATATTAACAGGCTCAGCGATTACGACGTCGATGCCATCGTTCCACAGTCATTCCTGAAGGATGACTCCATTGACAACAAGGTCCTCACCAGGTCGGACAAGAACCGGGGCAAGTCTGATAATGTTCCTTCAGAGGAGGTCGTTAAGAAGATGAAGAACTACTGGCGCCAGCTCCTGAATGCCAAGCTGATCACGCAGCGGAAGTTCGATAACCTCACAAAGGCTGAGAGGGGCGGGCTCTCTGAGCTGGACAAGGCGGGCTTCATCAAGAGGCAGCTGGTCGAGACACGGCAGATCACTAAGCACGTTGCGCAGATTCTCGACTCACGGATGAACACTAAGTACGATGAGAATGACAAGCTGATCCGCGAGGTGAAGGTCATCACCCTGAAGTCAAAGCTCGTCTCCGACTTCAGGAAGGATTTCCAGTTCTACAAGGTTCGGGAGATCAACAATTACCACCATGCCCATGACGCGTACCTGAACGCGGTGGTCGGCACAGCTCTGATCAAGAAGTACCCAAAGCTCGAGAGCGAGTTCGTGTACGGGGACTACAAGGTTTACGATGTGAGGAAGATGATCGCCAAGTCGGAGCAGGAGATTGGCAAGGCTACCGCCAAGTACTTCTTCTACTCTAACATTATGAATTTCTTCAAGACAGAGATCACTCTGGCCAATGGCGAGATCCGGAAGCGCCCCCTCATCGAGACGAACGGCGAGACGGGGGAGATCGTGTGGGACAAGGGCAGGGATTTCGCGACCGTCAGGAAGGTTCTCTCCATGCCACAAGTGAATATCGTCAAGAAGACAGAGGTCCAGACTGGCGGGTTCTCTAAGGAGTCAATTCTGCCTAAGCGGAACAGCGACAAGCTCATCGCCCGCAAGAAGGACTGGGATCCGAAGAAGTACGGCGGGTTCGACAGCCCCACTGTGGCCTACTCGGTCCTGGTTGTGGCGAAGGTTGAGAAGGGCAAGTCCAAGAAGCTCAAGAGCGTGAAGGAGCTGCTGGGGATCACGATTATGGAGCGCTCCAGCTTCGAGAAGAACCCGATCGATTTCCTGGAGGCGAAGGGCTACAAGGAGGTGAAGAAGGACCTGATCATTAAGCTCCCCAAGTACTCACTCTTCGAGCTGGAGAACGGCAGGAAGCGGATGCTGGCTTCCGCTGGCGAGCTGCAGAAGGGGAACGAGCTGGCTCTGCCGTCCAAGTATGTGAACTTCCTCTACCTGGCCTCCCACTACGAGAAGCTCAAGGGCAGCCCCGAGGACAACGAGCAGAAGCAGCTGTTCGTCGAGCAGCACAAGCATTACCTCGACGAGATCATTGAGCAGATTTCCGAGTTCTCCAAGCGCGTGATCCTGGCCGACGCGAATCTGGATAAGGTCCTCTCCGCGTACAACAAGCACCGCGACAAGCCAATCAGGGAGCAGGCTGAGAATATCATTCATCTCTTCACCCTGACGAACCTCGGCGCCCCTGCTGCTTTCAAGTACTTCGACACAACTATCGATCGCAAGAGGTACACAAGCACTAAGGAGGTCCTGGACGCGACCCTCATCCACCAGTCGATTACCGGCCTCTACGAGACGCGCATCGACCTGTCTCAGCTCGGGGGCGACGAATTCTCCGGGAGCGAGACGCCAGGCACCTCCGAGTCGGCCACCCCAGAATCTGCCACAGTGGTGTCCGGCCAAAAGCAGGACCGCCAGGGCGGAGAACGCAGAAGGTCCCAGCTCGATAGGGATCAGTGTGCCTACTGCAAGGAGAAGGGCCACTGGGCCAAAGACTGCCCGAAAAAGCCGCGCGGCCCACGCGGCCCAAGGCCACAAACATCCCTCCTTCCAAAGAAGAAGCGGAAGGTGGAGCTCAGCGGAGGATCTTCCGGAGGATCTAGCGGCTCCGAGACACCAGGAACATCCGAAAGCGCTACACCAGAATCTAGCGGAGGCTCTTCCGGAGGATCTAGGCCTCCTAGATCTGAAGTTAAGGTTACAGGGCCGGAAGGAATCCCTTTAACTATCCTGACAATGTCTATTGAGGATGAATACCGCCTGCATGAAAAGAGGACCAACAGCAACAATCAGGAGACCCTCGACCACTGGCTTGCGGAGTTTCCTCAGGCGTGGGCAGAAACCGGCGGGATGGGTTTAGCCATCAATCAGGCGCCCATAATAGTTACTCTGAAGGCCGCCATTCTGCCCGCCTCTGTGAGACAATATCCTATGCCAAAAGAGGCGAGGGAAGGTATACGGCCACACATAAAGAGATTGCTGGAGCAAGGGATCCTGGTCCCGTGCAAAAGCCCGTGGAACACGCCGCTGCTGCCTGTGAGGAAACCAGGCACTAATGATTACCGCCCCGTGCAAGATTTACGAGAGGTGAATAAGAGGATCGAAGACATCCACCCAACCGTGCCAAATCCATACAACTTACTGTCTGGTCTGCCGCCTAACTACACATGGTACACGGTATTGGACTTGAAGGACGCCTTCTTCTGCCTTAGATTACACCCGACCTCTCAGCCGATTTTTGCATTCGAATGGCAGGACGCTGATCTCGGCATCTCCGGCCAGCTCACCTGGACCAGACTGCCCCAGGGTTTCAAAAACAGCCCTACACTGTTTGATGAGGCCCTTCATCAAGACCTCGCCGGGTTCCGCGTAAGATATCCCGCCCTGATTCTCCTGCAGTACGTTGATGATATCCTGTTGGCCGCGAAGACAAAGGAAGAGTGCAAGGAGGGCACAAGAGCCCTGTTACAGACGCTTGGCTCGCTGGGCTATAGGGCTTCCGCTAAGAAGGCTCAGATTTGTCAAAAGCAGGTGACGTATCTGGGATACAAAATCAAGGATGGCAGAAGATGGTTGACCGAAGCCAGAATGCGCGCGATCCTGGACATTCCAACCCCACAAAACCCTAGACAACTACGGGAATTTCTGGGCACAGCTGGCTTCTGTAGGCTGTGGATCCCTGGCTTCGCGGAGATGGCGGCCCCATTGTACCCTCTGACACGGCCTGGCGTCGCTTTCAAGTGGGAGGAACCACAGAAAAAAGCGTTCACTAATATTAAAAAGGCCCTGCTCGAGTCTCCCGCGCTGGGCTTACCTGACCTGGCAAAGCCTTTTGAGCTCTTCATCGACGAGAAGGGCGGCTACGCCAAGGGAGTCCTGACCCAGAAGCTGGGCCCATGGAGACGGCCCACCGCGTACCTCAGCAAAAAGCTCGATCCGGTGGCTAGCGGATGGCCACCTTGTCTAAGGATGATTGCGGCAATCGCCCTGCTGGTGAAAGATTCCTACAAACTCACCCTGGGCCAACCCTTAACTATACACGCCCCCCACGCCGTGGAGGCCGTTATCCGCCAGCCGCCCGATAGATGGTTAACCAATGCTCGCATGACCCACTACCAGACCATGTTACTGGACAAGGACCGGGTGCATTTTGGCCCCCTCGTGACACTCAACCCAGCGACCTTGTTGCCTCTCCCCGGAGAGCCGGAGGCTCACAACTGCCTGCAGGTGCTGGCCGAGGCGCACGGCGCTAGACCCGACCTGACTGATCAGCCACTGCCAAGCCCTGACCATATTTGGTTCACCGACGGGAGTAGCTTCCTCCACCAAGGAAAAAGACGGGCTGGTGCCGCTGTGACTACAGAGAACCAGGTGGTCTGGGCGCAGGCACTGCCACCCGGAACCAGCGCCCAAAGAGCTGAGCTCATCGCTCTGACGCAGGCCCTGAAGCTGGCCGAAGGCAAGCGCCTGACCGTCTATACTGACAGCCGGTATGCTTTCGCCACAGCACATATCCACGGTGAGATCTACAGAAGAAGGGGCTTACTGACTAGCGAAGGAAAGGATATCAAGAACAAGGAAGAAATTTTAGCCTTGCTCCGGGCGTTGCACCTGCCCTCTGCCCTCAGCATCATCCATTGCCCTGGCCACCAGAAAGGCGACTCCTTAGAGGCTCGTGGGAACCGGCGCGCCGACCTGGCTGCCAGAGAAGCAGCCTTGACAACTGACACAACCTCTCTGCTGGCCCTGGAGCCTACAAACGACCGACCTACGCCTTCATGGGACAGCGGCGGCAGCAAAAGAACGGCGGACGGCTCTGAGAAGCGCACCGCTGATAGCCAGCATTCAACTCCTCCGAAAACAAAGAGGAAAGTTGAGTTCGAACCGAAGAAGAAAAGGAAGGTGTGA

**Sequence 46 Plasmids sequence of ePPEplus-RT45**

(NLSSV40-nCas9(H840A/R221K/N394K)-XTEN-NC-NLS-32aa Linker-RT45-NLSvbp)

CCTAAGAAAAAGAGAAAAGTGGACAAGAAGTACTCGATCGGCCTCGATATTGGGACTAACTCTGTTGGCTGGGCCGTGATCACCGACGAGTACAAGGTGCCCTCAAAGAAGTTCAAGGTCCTGGGCAACACCGATCGGCATTCCATCAAGAAGAATCTCATTGGCGCTCTCCTGTTCGACAGCGGCGAGACGGCTGAGGCTACGCGGCTCAAGCGCACCGCCCGCAGGCGGTACACGCGCAGGAAGAATCGCATCTGCTACCTGCAGGAGATTTTCTCCAACGAGATGGCGAAGGTTGACGATTCTTTCTTCCACAGGCTGGAGGAGTCATTCCTCGTGGAGGAGGATAAGAAGCACGAGCGGCATCCAATCTTCGGCAACATTGTCGACGAGGTTGCCTACCACGAGAAGTACCCTACGATCTACCATCTGCGGAAGAAGCTCGTGGACTCCACAGATAAGGCGGACCTCCGCCTGATCTACCTCGCTCTGGCCCACATGATTAAGTTCAGGGGCCATTTCCTGATCGAGGGGGATCTCAACCCGGACAATAGCGATGTTGACAAGCTGTTCATCCAGCTCGTGCAGACGTACAACCAGCTCTTCGAGGAGAACCCCATTAATGCGTCAGGCGTCGACGCGAAGGCTATCCTGTCCGCTAGGCTCTCGAAGTCTCGGAAGCTCGAGAACCTGATCGCCCAGCTGCCGGGCGAGAAGAAGAACGGCCTGTTCGGGAATCTCATTGCGCTCAGCCTGGGGCTCACGCCCAACTTCAAGTCGAATTTCGATCTCGCTGAGGACGCCAAGCTGCAGCTCTCCAAGGACACATACGACGATGACCTGGATAACCTCCTGGCCCAGATCGGCGATCAGTACGCGGACCTGTTCCTCGCTGCCAAGAATCTGTCGGACGCCATCCTCCTGTCTGATATTCTCAGGGTGAACACCGAGATTACGAAGGCTCCGCTCTCAGCCTCCATGATCAAGCGCTACGACGAGCACCATCAGGATCTGACCCTCCTGAAGGCGCTGGTCAGGCAGCAGCTCCCCGAGAAGTACAAGGAGATCTTCTTCGATCAGTCGAAGAACGGCTACGCTGGGTACATTGACGGCGGGGCCTCTCAGGAGGAGTTCTACAAGTTCATCAAGCCGATTCTGGAGAAGATGGACGGCACGGAGGAGCTGCTGGTGAAGCTCAAGCGCGAGGACCTCCTGAGGAAGCAGCGGACATTCGATAACGGCAGCATCCCACACCAGATTCATCTCGGGGAGCTGCACGCTATCCTGAGGAGGCAGGAGGACTTCTACCCTTTCCTCAAGGATAACCGCGAGAAGATCGAGAAGATTCTGACTTTCAGGATCCCGTACTACGTCGGCCCACTCGCTAGGGGCAACTCCCGCTTCGCTTGGATGACCCGCAAGTCAGAGGAGACGATCACGCCGTGGAACTTCGAGGAGGTGGTCGACAAGGGCGCTAGCGCTCAGTCGTTCATCGAGAGGATGACGAATTTCGACAAGAACCTGCCAAATGAGAAGGTGCTCCCTAAGCACTCGCTCCTGTACGAGTACTTCACAGTCTACAACGAGCTGACTAAGGTGAAGTATGTGACCGAGGGCATGAGGAAGCCGGCTTTCCTGTCTGGGGAGCAGAAGAAGGCCATCGTGGACCTCCTGTTCAAGACCAACCGGAAGGTCACGGTTAAGCAGCTCAAGGAGGACTACTTCAAGAAGATTGAGTGCTTCGATTCGGTCGAGATCTCTGGCGTTGAGGACCGCTTCAACGCCTCCCTGGGGACCTACCACGATCTCCTGAAGATCATTAAGGATAAGGACTTCCTGGACAACGAGGAGAATGAGGATATCCTCGAGGACATTGTGCTGACACTCACTCTGTTCGAGGACCGGGAGATGATCGAGGAGCGCCTGAAGACTTACGCCCATCTCTTCGATGACAAGGTCATGAAGCAGCTCAAGAGGAGGAGGTACACCGGCTGGGGGAGGCTGAGCAGGAAGCTCATCAACGGCATTCGGGACAAGCAGTCCGGGAAGACGATCCTCGACTTCCTGAAGAGCGATGGCTTCGCGAACCGCAATTTCATGCAGCTGATTCACGATGACAGCCTCACATTCAAGGAGGATATCCAGAAGGCTCAGGTGAGCGGCCAGGGGGACTCGCTGCACGAGCATATCGCGAACCTCGCTGGCTCGCCAGCTATCAAGAAGGGGATTCTGCAGACCGTGAAGGTTGTGGACGAGCTGGTGAAGGTCATGGGCAGGCACAAGCCTGAGAACATCGTCATTGAGATGGCCCGGGAGAATCAGACCACGCAGAAGGGCCAGAAGAACTCACGCGAGAGGATGAAGAGGATCGAGGAGGGCATTAAGGAGCTGGGGTCCCAGATCCTCAAGGAGCACCCGGTGGAGAACACGCAGCTGCAGAATGAGAAGCTCTACCTGTACTACCTCCAGAATGGCCGCGATATGTATGTGGACCAGGAGCTGGATATTAACAGGCTCAGCGATTACGACGTCGATGCCATCGTTCCACAGTCATTCCTGAAGGATGACTCCATTGACAACAAGGTCCTCACCAGGTCGGACAAGAACCGGGGCAAGTCTGATAATGTTCCTTCAGAGGAGGTCGTTAAGAAGATGAAGAACTACTGGCGCCAGCTCCTGAATGCCAAGCTGATCACGCAGCGGAAGTTCGATAACCTCACAAAGGCTGAGAGGGGCGGGCTCTCTGAGCTGGACAAGGCGGGCTTCATCAAGAGGCAGCTGGTCGAGACACGGCAGATCACTAAGCACGTTGCGCAGATTCTCGACTCACGGATGAACACTAAGTACGATGAGAATGACAAGCTGATCCGCGAGGTGAAGGTCATCACCCTGAAGTCAAAGCTCGTCTCCGACTTCAGGAAGGATTTCCAGTTCTACAAGGTTCGGGAGATCAACAATTACCACCATGCCCATGACGCGTACCTGAACGCGGTGGTCGGCACAGCTCTGATCAAGAAGTACCCAAAGCTCGAGAGCGAGTTCGTGTACGGGGACTACAAGGTTTACGATGTGAGGAAGATGATCGCCAAGTCGGAGCAGGAGATTGGCAAGGCTACCGCCAAGTACTTCTTCTACTCTAACATTATGAATTTCTTCAAGACAGAGATCACTCTGGCCAATGGCGAGATCCGGAAGCGCCCCCTCATCGAGACGAACGGCGAGACGGGGGAGATCGTGTGGGACAAGGGCAGGGATTTCGCGACCGTCAGGAAGGTTCTCTCCATGCCACAAGTGAATATCGTCAAGAAGACAGAGGTCCAGACTGGCGGGTTCTCTAAGGAGTCAATTCTGCCTAAGCGGAACAGCGACAAGCTCATCGCCCGCAAGAAGGACTGGGATCCGAAGAAGTACGGCGGGTTCGACAGCCCCACTGTGGCCTACTCGGTCCTGGTTGTGGCGAAGGTTGAGAAGGGCAAGTCCAAGAAGCTCAAGAGCGTGAAGGAGCTGCTGGGGATCACGATTATGGAGCGCTCCAGCTTCGAGAAGAACCCGATCGATTTCCTGGAGGCGAAGGGCTACAAGGAGGTGAAGAAGGACCTGATCATTAAGCTCCCCAAGTACTCACTCTTCGAGCTGGAGAACGGCAGGAAGCGGATGCTGGCTTCCGCTGGCGAGCTGCAGAAGGGGAACGAGCTGGCTCTGCCGTCCAAGTATGTGAACTTCCTCTACCTGGCCTCCCACTACGAGAAGCTCAAGGGCAGCCCCGAGGACAACGAGCAGAAGCAGCTGTTCGTCGAGCAGCACAAGCATTACCTCGACGAGATCATTGAGCAGATTTCCGAGTTCTCCAAGCGCGTGATCCTGGCCGACGCGAATCTGGATAAGGTCCTCTCCGCGTACAACAAGCACCGCGACAAGCCAATCAGGGAGCAGGCTGAGAATATCATTCATCTCTTCACCCTGACGAACCTCGGCGCCCCTGCTGCTTTCAAGTACTTCGACACAACTATCGATCGCAAGAGGTACACAAGCACTAAGGAGGTCCTGGACGCGACCCTCATCCACCAGTCGATTACCGGCCTCTACGAGACGCGCATCGACCTGTCTCAGCTCGGGGGCGACGAATTCTCCGGGAGCGAGACGCCAGGCACCTCCGAGTCGGCCACCCCAGAATCTGCCACAGTGGTGTCCGGCCAAAAGCAGGACCGCCAGGGCGGAGAACGCAGAAGGTCCCAGCTCGATAGGGATCAGTGTGCCTACTGCAAGGAGAAGGGCCACTGGGCCAAAGACTGCCCGAAAAAGCCGCGCGGCCCACGCGGCCCAAGGCCACAAACATCCCTCCTTCCAAAGAAGAAGCGGAAGGTGGAGCTCAGCGGAGGATCTTCCGGAGGATCTAGCGGCTCCGAGACACCAGGAACATCCGAAAGCGCTACACCAGAATCTAGCGGAGGCTCTTCCGGAGGATCTAGGCCTCCTGGCCCGCAGGGACAGCCATTGCACGTCCTGACCCTGAACTTGGAAGATGAGTACAGACTGCATGAACCTCCACGCGACCCAAAAACCGCCCTGGAAACATTCTGGTTGAGCAAGTTCCCTCAGGCGTGGGCGGAAACTGGCGGCATGGGGCTTGCAACGCAGCAGGCTCCTCTCATCATCAGTTTGAAGGCGACCGCCACTCCTGTAAGCATAAAACAATACCCAATGTCACTGGAAGCCAAGACAGGAATCCGCCCCCACATTCGGAGACTGTTGGACCAGGGCATATTAACACCATGTCAAAGCCCCTGGAACACTCCTCTGCTGCCTGTAAAGAAACCTGGCACGGGCGACTACAGGCCGGTGCAGGACTTAAGAGAGGTGAACAAGAGGGTGGAGGACATCCACCCTACAGTCCCTAATCCTTACAACCTTCTGAATGGCCTCAGCCCCTCCCACAACTGGTATTCCGTGTTAGACCTCAAGGACGCCTTCTTCTGTCTGAGACTGCACCCTGAAAGCCAACCAATCTTTGCTTTCGAGTGGAAGGATCCAGAGTTGGGAATAAGCGGCCAGCTAACATGGACGAGACTGCCCCAGGGTTTCAAGAATTCACCCACGCTGTTTGATGAAGCCTTACACCGTGACCTGGCCGACTTCCGCATCCAGCACCCGACTCTCATCCTCCTGCAGTATGTTGATGATTTGCTGCTCGCCGCCACCTCGGAGCAGGAATGCAAGGAGGGCACCAAGGCCCTGCTGCTGACTCTGGGAAACCTAGGATACAAGGCTTCCGCAAAAAAGGCTCAGATCTGCCAGAAACAGGTGACATACCTTGGATATCAAATCAAGGAGGGGCAGAGATGGCTTACCGAAGCCAGGAAGGCTACAGTGATGAATATGCCAGTGCCCGGCACCCCGAGGCAGCTCAGAGAGTTCCTTGGCACCGCCGGTTTTTGTCGGCTCTGGATCCCGGGCTTCGCTGAGATCGCCGCCCCTCTGTACCCGCTAACCAAAAGCGGCACCCTGTTCCGGTGGGGTGAGGATCAGCAAAAGGCTTATCAGGAGATCAAGCGGGCCTTACTTACCGCGCCCGCGCTGGGCTTGCCCGACCTGACAAAGCCATTCGAGTTGTTCGTCGACGAGAAGCAAGGCTACGCTAAAGGCGTGCTGACCCAGAAGCTCGGCCCATGGAGACGGCCGGTCGCCTACCTAAGCAAGAAGCTGGACCCTGTCGCGAGCGGGTGGCCGCCGTGCCTGCGCATGGTGGCGGCCATCGCGGTGCTGACCAAGGATGCCGGAAAGCTGACCCTCGGTCAACCTCTCACCATCCTGGCGCCCCACGCGGTCGAGGCCCTAGTTAAGCAGCCGCCTGACAGATGGCTCTCCAACGCACGGATGACACACTACCAAGCAATGCTACTGGATACCGATCGAGTGCATTTTGGTCCCGTGGTGACATTAAACCCTGCCACTCTGCTGCCATTACCTGAAGGCGGTGCCAAGCATGACTGCTTGGAGATTCTGGCGGAGATGCATGGAACTCGGCCTGATCTGACTGATCAACCACTGCAGAATGCTGATTTCACCTGGTACACCGATGGTAGTTCTTTTTTGCAGGACGGTCAGCGTCGAGCCGGGGCCGCCGTAACGACAGAGACCGAAGTCATTTGGGCCGAGACTCTGCCAACCGGAACAAGTGCGCAGCGTGCCGAGCTGATAGCTCTTACACAAGCGCTGCGACTGGCCGAAGGCAAGAAGTTGAACGTCTACACAGACAGCAGATACGCGTTCGCCACGGCGCACATTCACGGCGAGATATATCGAAGACGGGGCCTCTTGACCTCTGAAGGAAAGGAGATTAAGAACAGAAATGAAATCCTGGCCCTGCTGAAGGCGCTGTTCCTGCCTCAGAGACTGTCTATTATTCATTGCCCCGGCCACCAGAAAGGTGACTCCCCTAAAGCTAGAGGCAACCGCATGGCAGACCGCGCAGCACGGGAAGCGGCCATGGGCACTGAAATATTGAAAAGACTAGGCGCCAGCCAAGATTCTGTGAAGGGAAGCGGCGGCAGCAAAAGAACGGCGGACGGCTCTGAGAAGCGCACCGCTGATAGCCAGCATTCAACTCCTCCGAAAACAAAGAGGAAAGTTGAGTTCGAACCGAAGAAGAAAAGGAAGGTGTGA

**Sequence 47 Plasmids sequence of ePPEplus-RT46(PE-RERV)**

(NLSSV40-nCas9(H840A/R221K/N394K)-XTEN-NC-NLS-32aa Linker-RT46(RERV)-NLSvbp)

CCTAAGAAAAAGAGAAAAGTGGACAAGAAGTACTCGATCGGCCTCGATATTGGGACTAACTCTGTTGGCTGGGCCGTGATCACCGACGAGTACAAGGTGCCCTCAAAGAAGTTCAAGGTCCTGGGCAACACCGATCGGCATTCCATCAAGAAGAATCTCATTGGCGCTCTCCTGTTCGACAGCGGCGAGACGGCTGAGGCTACGCGGCTCAAGCGCACCGCCCGCAGGCGGTACACGCGCAGGAAGAATCGCATCTGCTACCTGCAGGAGATTTTCTCCAACGAGATGGCGAAGGTTGACGATTCTTTCTTCCACAGGCTGGAGGAGTCATTCCTCGTGGAGGAGGATAAGAAGCACGAGCGGCATCCAATCTTCGGCAACATTGTCGACGAGGTTGCCTACCACGAGAAGTACCCTACGATCTACCATCTGCGGAAGAAGCTCGTGGACTCCACAGATAAGGCGGACCTCCGCCTGATCTACCTCGCTCTGGCCCACATGATTAAGTTCAGGGGCCATTTCCTGATCGAGGGGGATCTCAACCCGGACAATAGCGATGTTGACAAGCTGTTCATCCAGCTCGTGCAGACGTACAACCAGCTCTTCGAGGAGAACCCCATTAATGCGTCAGGCGTCGACGCGAAGGCTATCCTGTCCGCTAGGCTCTCGAAGTCTCGGAAGCTCGAGAACCTGATCGCCCAGCTGCCGGGCGAGAAGAAGAACGGCCTGTTCGGGAATCTCATTGCGCTCAGCCTGGGGCTCACGCCCAACTTCAAGTCGAATTTCGATCTCGCTGAGGACGCCAAGCTGCAGCTCTCCAAGGACACATACGACGATGACCTGGATAACCTCCTGGCCCAGATCGGCGATCAGTACGCGGACCTGTTCCTCGCTGCCAAGAATCTGTCGGACGCCATCCTCCTGTCTGATATTCTCAGGGTGAACACCGAGATTACGAAGGCTCCGCTCTCAGCCTCCATGATCAAGCGCTACGACGAGCACCATCAGGATCTGACCCTCCTGAAGGCGCTGGTCAGGCAGCAGCTCCCCGAGAAGTACAAGGAGATCTTCTTCGATCAGTCGAAGAACGGCTACGCTGGGTACATTGACGGCGGGGCCTCTCAGGAGGAGTTCTACAAGTTCATCAAGCCGATTCTGGAGAAGATGGACGGCACGGAGGAGCTGCTGGTGAAGCTCAAGCGCGAGGACCTCCTGAGGAAGCAGCGGACATTCGATAACGGCAGCATCCCACACCAGATTCATCTCGGGGAGCTGCACGCTATCCTGAGGAGGCAGGAGGACTTCTACCCTTTCCTCAAGGATAACCGCGAGAAGATCGAGAAGATTCTGACTTTCAGGATCCCGTACTACGTCGGCCCACTCGCTAGGGGCAACTCCCGCTTCGCTTGGATGACCCGCAAGTCAGAGGAGACGATCACGCCGTGGAACTTCGAGGAGGTGGTCGACAAGGGCGCTAGCGCTCAGTCGTTCATCGAGAGGATGACGAATTTCGACAAGAACCTGCCAAATGAGAAGGTGCTCCCTAAGCACTCGCTCCTGTACGAGTACTTCACAGTCTACAACGAGCTGACTAAGGTGAAGTATGTGACCGAGGGCATGAGGAAGCCGGCTTTCCTGTCTGGGGAGCAGAAGAAGGCCATCGTGGACCTCCTGTTCAAGACCAACCGGAAGGTCACGGTTAAGCAGCTCAAGGAGGACTACTTCAAGAAGATTGAGTGCTTCGATTCGGTCGAGATCTCTGGCGTTGAGGACCGCTTCAACGCCTCCCTGGGGACCTACCACGATCTCCTGAAGATCATTAAGGATAAGGACTTCCTGGACAACGAGGAGAATGAGGATATCCTCGAGGACATTGTGCTGACACTCACTCTGTTCGAGGACCGGGAGATGATCGAGGAGCGCCTGAAGACTTACGCCCATCTCTTCGATGACAAGGTCATGAAGCAGCTCAAGAGGAGGAGGTACACCGGCTGGGGGAGGCTGAGCAGGAAGCTCATCAACGGCATTCGGGACAAGCAGTCCGGGAAGACGATCCTCGACTTCCTGAAGAGCGATGGCTTCGCGAACCGCAATTTCATGCAGCTGATTCACGATGACAGCCTCACATTCAAGGAGGATATCCAGAAGGCTCAGGTGAGCGGCCAGGGGGACTCGCTGCACGAGCATATCGCGAACCTCGCTGGCTCGCCAGCTATCAAGAAGGGGATTCTGCAGACCGTGAAGGTTGTGGACGAGCTGGTGAAGGTCATGGGCAGGCACAAGCCTGAGAACATCGTCATTGAGATGGCCCGGGAGAATCAGACCACGCAGAAGGGCCAGAAGAACTCACGCGAGAGGATGAAGAGGATCGAGGAGGGCATTAAGGAGCTGGGGTCCCAGATCCTCAAGGAGCACCCGGTGGAGAACACGCAGCTGCAGAATGAGAAGCTCTACCTGTACTACCTCCAGAATGGCCGCGATATGTATGTGGACCAGGAGCTGGATATTAACAGGCTCAGCGATTACGACGTCGATGCCATCGTTCCACAGTCATTCCTGAAGGATGACTCCATTGACAACAAGGTCCTCACCAGGTCGGACAAGAACCGGGGCAAGTCTGATAATGTTCCTTCAGAGGAGGTCGTTAAGAAGATGAAGAACTACTGGCGCCAGCTCCTGAATGCCAAGCTGATCACGCAGCGGAAGTTCGATAACCTCACAAAGGCTGAGAGGGGCGGGCTCTCTGAGCTGGACAAGGCGGGCTTCATCAAGAGGCAGCTGGTCGAGACACGGCAGATCACTAAGCACGTTGCGCAGATTCTCGACTCACGGATGAACACTAAGTACGATGAGAATGACAAGCTGATCCGCGAGGTGAAGGTCATCACCCTGAAGTCAAAGCTCGTCTCCGACTTCAGGAAGGATTTCCAGTTCTACAAGGTTCGGGAGATCAACAATTACCACCATGCCCATGACGCGTACCTGAACGCGGTGGTCGGCACAGCTCTGATCAAGAAGTACCCAAAGCTCGAGAGCGAGTTCGTGTACGGGGACTACAAGGTTTACGATGTGAGGAAGATGATCGCCAAGTCGGAGCAGGAGATTGGCAAGGCTACCGCCAAGTACTTCTTCTACTCTAACATTATGAATTTCTTCAAGACAGAGATCACTCTGGCCAATGGCGAGATCCGGAAGCGCCCCCTCATCGAGACGAACGGCGAGACGGGGGAGATCGTGTGGGACAAGGGCAGGGATTTCGCGACCGTCAGGAAGGTTCTCTCCATGCCACAAGTGAATATCGTCAAGAAGACAGAGGTCCAGACTGGCGGGTTCTCTAAGGAGTCAATTCTGCCTAAGCGGAACAGCGACAAGCTCATCGCCCGCAAGAAGGACTGGGATCCGAAGAAGTACGGCGGGTTCGACAGCCCCACTGTGGCCTACTCGGTCCTGGTTGTGGCGAAGGTTGAGAAGGGCAAGTCCAAGAAGCTCAAGAGCGTGAAGGAGCTGCTGGGGATCACGATTATGGAGCGCTCCAGCTTCGAGAAGAACCCGATCGATTTCCTGGAGGCGAAGGGCTACAAGGAGGTGAAGAAGGACCTGATCATTAAGCTCCCCAAGTACTCACTCTTCGAGCTGGAGAACGGCAGGAAGCGGATGCTGGCTTCCGCTGGCGAGCTGCAGAAGGGGAACGAGCTGGCTCTGCCGTCCAAGTATGTGAACTTCCTCTACCTGGCCTCCCACTACGAGAAGCTCAAGGGCAGCCCCGAGGACAACGAGCAGAAGCAGCTGTTCGTCGAGCAGCACAAGCATTACCTCGACGAGATCATTGAGCAGATTTCCGAGTTCTCCAAGCGCGTGATCCTGGCCGACGCGAATCTGGATAAGGTCCTCTCCGCGTACAACAAGCACCGCGACAAGCCAATCAGGGAGCAGGCTGAGAATATCATTCATCTCTTCACCCTGACGAACCTCGGCGCCCCTGCTGCTTTCAAGTACTTCGACACAACTATCGATCGCAAGAGGTACACAAGCACTAAGGAGGTCCTGGACGCGACCCTCATCCACCAGTCGATTACCGGCCTCTACGAGACGCGCATCGACCTGTCTCAGCTCGGGGGCGACGAATTCTCCGGGAGCGAGACGCCAGGCACCTCCGAGTCGGCCACCCCAGAATCTGCCACAGTGGTGTCCGGCCAAAAGCAGGACCGCCAGGGCGGAGAACGCAGAAGGTCCCAGCTCGATAGGGATCAGTGTGCCTACTGCAAGGAGAAGGGCCACTGGGCCAAAGACTGCCCGAAAAAGCCGCGCGGCCCACGCGGCCCAAGGCCACAAACATCCCTCCTTCCAAAGAAGAAGCGGAAGGTGGAGCTCAGCGGAGGATCTTCCGGAGGATCTAGCGGCTCCGAGACACCAGGAACATCCGAAAGCGCTACACCAGAATCTAGCGGAGGCTCTTCCGGAGGATCTAGGCCTCCCGTCGCGTGCCTCGTACTGAGCCTAGAGGAGGAGTACAGGCTGCATGAGCAAAATCCGAAGCAGCTGCTAGCCCCTGAATGGCTGAGCGCCTTCTCCGAGGTATGGGCGGAGCAGACTGGCATGGGACTGGCCAAGCAGGTGCCTCCTGTGGTGGTAGAGCTGAAGGCGGACGCCAGCCCAGTGAGCGTGAAACAATACCCGATGTCGAGGGAAGCAAAGGAAGGCATTAGACCCCACATCCAGAGACTGCTGCAATTGGGAATCCTGGTACCCTGCCAAAGCCCTTGGAACACGCCACTGCTGCCTGTGCGCAAACCAGGAACTAATGATTACAGACCTGTACAGGATCTAAGGGAAGTTAACAAGCGGGTGCAGGACATACATCCTACGGTCCCAAACCCCTACAACTTGCTGAGCTCTCTGCCCCCCGAGCGCACATGGTACACTGTGCTCGACTTAAAAGATGCGTTCTTCTGCCTTCGCCTCCACCCGAACTCACAGCCACTGTTCGCGTTCGAGTGGCGCGACCCTGAGGGCGGCCACACCGGGCAACTGACGTGGACACGGCTGCCACAGGGTTTCAAGAACAGCCCTACTCTATTTGATGAGGCCCTGCACCGTGATCTGGCCCCCTTCCGGGCGCAGAACCCTCAAATCAGTCTACTGCAGTATGTTGATGACCTGCTGCTAGCCGCTTCTACTCAGGAGCTCTGCCGGGAGGGCACAAAGCGGCTGTTAAATGAGCTGGGCGAGCTGGGATACCGCGTGTCTGCCAAGAAGGCACAATTGTGTCGGACAGAGGTGACATATCTCGGCTACACATTAAGAGAAGGTAAGAGATGGCTCACTGAAGCACGTAAGAAGACTGTCATGCAGATTCCTACCCCTACCACGCCCCGTCAGGTGCGGGAGTTCCTCGGCACCGCCGGCTTCTGTAGACTGTGGATTCCTGGATTTGCCACCCTGGCCGCCCCACTCTACCTGCTGACGAAGGAAAAGGTTCCTTTCACCTGGACTGAGGAACATCAGCGGGCTTTCGAAGACATCAAAGCCGCCCTGCTGGCGGCTCCAGCTCTGGCCCTCCCTGACCTCACCAAGCCCTTCACACTCTATGTGGACGAGAGAGCTGGAGTGGCCAGAGGTGTGCTGACACAGGCACTGGGGCCGTGGAAGAGACCCGTGGCGTACCTATCTAAAAAGCTCGACCCCGTGGCCTCTGGGTGGCCGAGCTGTCTAAAGGCCATCGCTGCTGTAGCTCTACTGGTCAAAGATGCTGACAAGCTGACCCTGGGCCAGCATGTCACCGTCATCGCCCCGCACGCCTTAGAATCCATAGTCCGGCAACCACCAGATCGGTGGATGACAAACGCTAGGATGACGCATTATCAGAGCTTGCTGTTAAACGAAAGAGTGACATTTGCGCCCCCTGCCATCCTTAACCCAGCGACGCTACTGCCGGAGATCCACAACTCAACCCCCATCCACCAGTGCGTCGACATTCTGGCTGAGGAGACCGGCACGCGCAAGGACCTCACGGACAGACCATGGCCTGGTGTGCCGGCATGGTACACCGACGGCAGCTCCTTTGTCGTGGAGGGAAAGCGACGCGCAGGCGCGGCTGTGGTGGACGGCAAGCAAGTCATCTGGGCATCCTCCCTGCCAGAAGGCACCAGCGCTCAGAAAGCGGAGTTGGTGGCCTTGACCCAGGCCCTCAGATTAGCTGAAGGGAAGGCTATTAATATCTACACCGATTCTAGATACGCCTTCGCCACGGCGCACATCCACGGCGCGATCTACAAGCAGCGGGGCCTGCTGACCAGTGCCGGGCGAGACATCAAGAATAAGGAAGAGATTCTTGCCCTGCTGGAGGCCGTCCACCTCCCCAAAAAGGTGGCCATTATCCACTGCCCCGGCCACCAGAAGGGAGAGGATCCAATTACAAAGGGGAACCAGATGGCCGACCTCGTGGCGAAGCAGGTCGCTCAGCAGGTTACCATCCTGGCAGAAAAATCGCAGACCCCTGTGAAGACACCTGTGACAGATGATTCTAACTATAGCGGCGGCAGCAAAAGAACGGCGGACGGCTCTGAGAAGCGCACCGCTGATAGCCAGCATTCAACTCCTCCGAAAACAAAGAGGAAAGTTGAGTTCGAACCGAAGAAGAAAAGGAAGGTGTGA

**Sequence 48 Plasmids sequence of ePPEplus-RT47**

(NLSSV40-nCas9(H840A/R221K/N394K)-XTEN-NC-NLS-32aa Linker-RT47-NLSvbp)

CCTAAGAAAAAGAGAAAAGTGGACAAGAAGTACTCGATCGGCCTCGATATTGGGACTAACTCTGTTGGCTGGGCCGTGATCACCGACGAGTACAAGGTGCCCTCAAAGAAGTTCAAGGTCCTGGGCAACACCGATCGGCATTCCATCAAGAAGAATCTCATTGGCGCTCTCCTGTTCGACAGCGGCGAGACGGCTGAGGCTACGCGGCTCAAGCGCACCGCCCGCAGGCGGTACACGCGCAGGAAGAATCGCATCTGCTACCTGCAGGAGATTTTCTCCAACGAGATGGCGAAGGTTGACGATTCTTTCTTCCACAGGCTGGAGGAGTCATTCCTCGTGGAGGAGGATAAGAAGCACGAGCGGCATCCAATCTTCGGCAACATTGTCGACGAGGTTGCCTACCACGAGAAGTACCCTACGATCTACCATCTGCGGAAGAAGCTCGTGGACTCCACAGATAAGGCGGACCTCCGCCTGATCTACCTCGCTCTGGCCCACATGATTAAGTTCAGGGGCCATTTCCTGATCGAGGGGGATCTCAACCCGGACAATAGCGATGTTGACAAGCTGTTCATCCAGCTCGTGCAGACGTACAACCAGCTCTTCGAGGAGAACCCCATTAATGCGTCAGGCGTCGACGCGAAGGCTATCCTGTCCGCTAGGCTCTCGAAGTCTCGGAAGCTCGAGAACCTGATCGCCCAGCTGCCGGGCGAGAAGAAGAACGGCCTGTTCGGGAATCTCATTGCGCTCAGCCTGGGGCTCACGCCCAACTTCAAGTCGAATTTCGATCTCGCTGAGGACGCCAAGCTGCAGCTCTCCAAGGACACATACGACGATGACCTGGATAACCTCCTGGCCCAGATCGGCGATCAGTACGCGGACCTGTTCCTCGCTGCCAAGAATCTGTCGGACGCCATCCTCCTGTCTGATATTCTCAGGGTGAACACCGAGATTACGAAGGCTCCGCTCTCAGCCTCCATGATCAAGCGCTACGACGAGCACCATCAGGATCTGACCCTCCTGAAGGCGCTGGTCAGGCAGCAGCTCCCCGAGAAGTACAAGGAGATCTTCTTCGATCAGTCGAAGAACGGCTACGCTGGGTACATTGACGGCGGGGCCTCTCAGGAGGAGTTCTACAAGTTCATCAAGCCGATTCTGGAGAAGATGGACGGCACGGAGGAGCTGCTGGTGAAGCTCAAGCGCGAGGACCTCCTGAGGAAGCAGCGGACATTCGATAACGGCAGCATCCCACACCAGATTCATCTCGGGGAGCTGCACGCTATCCTGAGGAGGCAGGAGGACTTCTACCCTTTCCTCAAGGATAACCGCGAGAAGATCGAGAAGATTCTGACTTTCAGGATCCCGTACTACGTCGGCCCACTCGCTAGGGGCAACTCCCGCTTCGCTTGGATGACCCGCAAGTCAGAGGAGACGATCACGCCGTGGAACTTCGAGGAGGTGGTCGACAAGGGCGCTAGCGCTCAGTCGTTCATCGAGAGGATGACGAATTTCGACAAGAACCTGCCAAATGAGAAGGTGCTCCCTAAGCACTCGCTCCTGTACGAGTACTTCACAGTCTACAACGAGCTGACTAAGGTGAAGTATGTGACCGAGGGCATGAGGAAGCCGGCTTTCCTGTCTGGGGAGCAGAAGAAGGCCATCGTGGACCTCCTGTTCAAGACCAACCGGAAGGTCACGGTTAAGCAGCTCAAGGAGGACTACTTCAAGAAGATTGAGTGCTTCGATTCGGTCGAGATCTCTGGCGTTGAGGACCGCTTCAACGCCTCCCTGGGGACCTACCACGATCTCCTGAAGATCATTAAGGATAAGGACTTCCTGGACAACGAGGAGAATGAGGATATCCTCGAGGACATTGTGCTGACACTCACTCTGTTCGAGGACCGGGAGATGATCGAGGAGCGCCTGAAGACTTACGCCCATCTCTTCGATGACAAGGTCATGAAGCAGCTCAAGAGGAGGAGGTACACCGGCTGGGGGAGGCTGAGCAGGAAGCTCATCAACGGCATTCGGGACAAGCAGTCCGGGAAGACGATCCTCGACTTCCTGAAGAGCGATGGCTTCGCGAACCGCAATTTCATGCAGCTGATTCACGATGACAGCCTCACATTCAAGGAGGATATCCAGAAGGCTCAGGTGAGCGGCCAGGGGGACTCGCTGCACGAGCATATCGCGAACCTCGCTGGCTCGCCAGCTATCAAGAAGGGGATTCTGCAGACCGTGAAGGTTGTGGACGAGCTGGTGAAGGTCATGGGCAGGCACAAGCCTGAGAACATCGTCATTGAGATGGCCCGGGAGAATCAGACCACGCAGAAGGGCCAGAAGAACTCACGCGAGAGGATGAAGAGGATCGAGGAGGGCATTAAGGAGCTGGGGTCCCAGATCCTCAAGGAGCACCCGGTGGAGAACACGCAGCTGCAGAATGAGAAGCTCTACCTGTACTACCTCCAGAATGGCCGCGATATGTATGTGGACCAGGAGCTGGATATTAACAGGCTCAGCGATTACGACGTCGATGCCATCGTTCCACAGTCATTCCTGAAGGATGACTCCATTGACAACAAGGTCCTCACCAGGTCGGACAAGAACCGGGGCAAGTCTGATAATGTTCCTTCAGAGGAGGTCGTTAAGAAGATGAAGAACTACTGGCGCCAGCTCCTGAATGCCAAGCTGATCACGCAGCGGAAGTTCGATAACCTCACAAAGGCTGAGAGGGGCGGGCTCTCTGAGCTGGACAAGGCGGGCTTCATCAAGAGGCAGCTGGTCGAGACACGGCAGATCACTAAGCACGTTGCGCAGATTCTCGACTCACGGATGAACACTAAGTACGATGAGAATGACAAGCTGATCCGCGAGGTGAAGGTCATCACCCTGAAGTCAAAGCTCGTCTCCGACTTCAGGAAGGATTTCCAGTTCTACAAGGTTCGGGAGATCAACAATTACCACCATGCCCATGACGCGTACCTGAACGCGGTGGTCGGCACAGCTCTGATCAAGAAGTACCCAAAGCTCGAGAGCGAGTTCGTGTACGGGGACTACAAGGTTTACGATGTGAGGAAGATGATCGCCAAGTCGGAGCAGGAGATTGGCAAGGCTACCGCCAAGTACTTCTTCTACTCTAACATTATGAATTTCTTCAAGACAGAGATCACTCTGGCCAATGGCGAGATCCGGAAGCGCCCCCTCATCGAGACGAACGGCGAGACGGGGGAGATCGTGTGGGACAAGGGCAGGGATTTCGCGACCGTCAGGAAGGTTCTCTCCATGCCACAAGTGAATATCGTCAAGAAGACAGAGGTCCAGACTGGCGGGTTCTCTAAGGAGTCAATTCTGCCTAAGCGGAACAGCGACAAGCTCATCGCCCGCAAGAAGGACTGGGATCCGAAGAAGTACGGCGGGTTCGACAGCCCCACTGTGGCCTACTCGGTCCTGGTTGTGGCGAAGGTTGAGAAGGGCAAGTCCAAGAAGCTCAAGAGCGTGAAGGAGCTGCTGGGGATCACGATTATGGAGCGCTCCAGCTTCGAGAAGAACCCGATCGATTTCCTGGAGGCGAAGGGCTACAAGGAGGTGAAGAAGGACCTGATCATTAAGCTCCCCAAGTACTCACTCTTCGAGCTGGAGAACGGCAGGAAGCGGATGCTGGCTTCCGCTGGCGAGCTGCAGAAGGGGAACGAGCTGGCTCTGCCGTCCAAGTATGTGAACTTCCTCTACCTGGCCTCCCACTACGAGAAGCTCAAGGGCAGCCCCGAGGACAACGAGCAGAAGCAGCTGTTCGTCGAGCAGCACAAGCATTACCTCGACGAGATCATTGAGCAGATTTCCGAGTTCTCCAAGCGCGTGATCCTGGCCGACGCGAATCTGGATAAGGTCCTCTCCGCGTACAACAAGCACCGCGACAAGCCAATCAGGGAGCAGGCTGAGAATATCATTCATCTCTTCACCCTGACGAACCTCGGCGCCCCTGCTGCTTTCAAGTACTTCGACACAACTATCGATCGCAAGAGGTACACAAGCACTAAGGAGGTCCTGGACGCGACCCTCATCCACCAGTCGATTACCGGCCTCTACGAGACGCGCATCGACCTGTCTCAGCTCGGGGGCGACGAATTCTCCGGGAGCGAGACGCCAGGCACCTCCGAGTCGGCCACCCCAGAATCTGCCACAGTGGTGTCCGGCCAAAAGCAGGACCGCCAGGGCGGAGAACGCAGAAGGTCCCAGCTCGATAGGGATCAGTGTGCCTACTGCAAGGAGAAGGGCCACTGGGCCAAAGACTGCCCGAAAAAGCCGCGCGGCCCACGCGGCCCAAGGCCACAAACATCCCTCCTTCCAAAGAAGAAGCGGAAGGTGGAGCTCAGCGGAGGATCTTCCGGAGGATCTAGCGGCTCCGAGACACCAGGAACATCCGAAAGCGCTACACCAGAATCTAGCGGAGGCTCTTCCGGAGGATCTAGGCCTCCTAACAGGGATGGCCAACCAATTCAGGTGCTCACCGTGAGCCTGCAGGATGAACATAGACTTTTTGAAACACCGGTGACCACAAACCTGCTCGAAGCCTGGCTCCAAGACTTCCCACAAGCGTGGGCAGAGACCGGCGGACTAGGCAGGGCCAAATGTCAAGCACCCATCATCATTGATCTCAAACCTACGGCGATGCCAGTCTCCATACGCCAGTACCCGATGTCGAAGGAAGCGCACATGGGAATCCAGCCGCATATCACAAGATTTCTCGAATTAGGAGTCCTGCGTCCCTGTCGTAGCCCATGGAACACACCTCTGCTGCCTGTCAAGAAGCCAGGCACCAGGGACTACAGACCTGTTCAAGATCTGCGTGAGGTGAACAAGCGGACAATGGATATTCACCCAACAGTGCCGAACCCATACAATCTTCTGTCTACTCTGAGCCCAGACCGCACCTGGTACACAGTGCTGGACTTGAAGGACGCGTTCTTCTGTCTGCCTCTCGCCCCACAAAGCCAGGAACTCTTCGCTTTCGAGTGGAGGGACCCGAAGAGAGGGATAAGCGGCCAGCTCACATGGACACGGCTGCCCCAGGGATTCAAGAACTCCCCTACATTATTTGATGAGGCCCTACACCGCGACCTCACCGACTTTAGAACACAGCACCCGGAGGTTACTCTGTTGCAGTATGTGGATGATTTGCTCCTGGCCGCCCCTACCAAGGAGGCGTGCATTCTAGGCACCAGGCACCTGTTGAGAGAACTTGGTGAAAAGGGGTACCGCGCCTCAGCCAAGAAAGCGCAGATCTGCCAGACTAAGGTGACATACCTGGGCTACATCCTGTCTGAGGGAAAGAGATGGCTGACTCCCGGTCGGATCGAGACGGTGGCAAGAATCCCACCCCCTCAGAACCCTAGGGAAGTAAGGGAGTTCCTGGGCACCGCTGGATTCTGCAGACTCTGGATCCCTGGCTTTGCCGAGCTGGCAGCCCCCCTCTATGCCCTCACGAAAGAAAGTGCCCCGTTCACCTGGCAAGAGAAGCATCAAAGCGCATTTGAGGCTCTCAAGGAGGCTCTGTTATCCGCCCCTGCCCTGGGTCTGCCGGACACCTCTAAGCCGTTCACGTTGTTCATCGACGAGAAGCAGGGCATCGCTAAGGGAGTGCTCACTCAGAAATTAGGCCCGTGGAAGCGCCCAGTCGCCTACCTGAGCAAGAAACTCGACCCTGTGGCCGCCGGGTGGCCGCCGTGCTTGAGAATTATGGCCGCGACCGCCATGCTGGTTAAGGACTCCGCTAAGCTGACGCTGGGTCAGCCTCTGACTGTTATCACTCCCCACGCGTTAGAGGCGGTGGTGCGACAACCTCCAGACCGATGGATCACAAATGCTAGACTGACTCACTACCAAGCCCTGCTGCTCGATACAGATCGGATCCAATTTGGCCCTCCTGTCACCCTCAACCCGGCCACCCTGCTGCCTGCGCCTGAGGACCAGCAATCTGCTCATGATTGCCGCCAGGTGTTAGCGGAGATACATGGCACGAGGGAGGATCTGAAAGACCAGGAGCTGCCTGACGCCGACCACAGCTGGTACACGGACGGGAGCTCTTACATCGATTCTGGCACAAGACGAGCCGGGGCCGCCGTGGTGGACGGCCACCACATTATATGGGCACAGTCACTGCCCCCCGGAACCTCCGCCCAAAAAGCTGAGCTAATCGCCCTGACTAAGGCCCTGGAGCTAAGCGAGGGCAAAAAGGCAAATATCTATACTGACAGCAGATATGCTTTCGCCACCGCCCACACGCACGGTTCCATCTACGAGCGGCGGGGCCTGCTGACTTCAGAAGGCAAAGAGATCAAGAATAAGGCCGAGATCATCGCGCTGTTGAAGGCACTGTTCCTACCCCAGAGAGTGGCGATCATTCACTGCCCTGGCCACCAGAAGGGGCAGGGTCCTATTGCAACCGGCAACAGACAGGCAGACCAAGTCGCCAGACAGGTCGCGGCCATCGGCGCAGTTCTCAACCAGGATACAAAAGACTGGGAGAAAGAAGGCAAGATTGTGCTGCCCAGAAAGGAAGCCCTAGCAATGATTCAGCAGATGCATGCTTGGACGCACCTCTCTAATCAGAAATTGAAGAGCCTTATTGAAAAAACAGATTTCCTTATCCCGAAGAGCGGCGGCAGCAAAAGAACGGCGGACGGCTCTGAGAAGCGCACCGCTGATAGCCAGCATTCAACTCCTCCGAAAACAAAGAGGAAAGTTGAGTTCGAACCGAAGAAGAAAAGGAAGGTGTGA

**Sequence 49 Plasmids sequence of ePPEplus-RT48**

(NLSSV40-nCas9(H840A/R221K/N394K)-XTEN-NC-NLS-32aa Linker-RT48-NLSvbp)

CCTAAGAAAAAGAGAAAAGTGGACAAGAAGTACTCGATCGGCCTCGATATTGGGACTAACTCTGTTGGCTGGGCCGTGATCACCGACGAGTACAAGGTGCCCTCAAAGAAGTTCAAGGTCCTGGGCAACACCGATCGGCATTCCATCAAGAAGAATCTCATTGGCGCTCTCCTGTTCGACAGCGGCGAGACGGCTGAGGCTACGCGGCTCAAGCGCACCGCCCGCAGGCGGTACACGCGCAGGAAGAATCGCATCTGCTACCTGCAGGAGATTTTCTCCAACGAGATGGCGAAGGTTGACGATTCTTTCTTCCACAGGCTGGAGGAGTCATTCCTCGTGGAGGAGGATAAGAAGCACGAGCGGCATCCAATCTTCGGCAACATTGTCGACGAGGTTGCCTACCACGAGAAGTACCCTACGATCTACCATCTGCGGAAGAAGCTCGTGGACTCCACAGATAAGGCGGACCTCCGCCTGATCTACCTCGCTCTGGCCCACATGATTAAGTTCAGGGGCCATTTCCTGATCGAGGGGGATCTCAACCCGGACAATAGCGATGTTGACAAGCTGTTCATCCAGCTCGTGCAGACGTACAACCAGCTCTTCGAGGAGAACCCCATTAATGCGTCAGGCGTCGACGCGAAGGCTATCCTGTCCGCTAGGCTCTCGAAGTCTCGGAAGCTCGAGAACCTGATCGCCCAGCTGCCGGGCGAGAAGAAGAACGGCCTGTTCGGGAATCTCATTGCGCTCAGCCTGGGGCTCACGCCCAACTTCAAGTCGAATTTCGATCTCGCTGAGGACGCCAAGCTGCAGCTCTCCAAGGACACATACGACGATGACCTGGATAACCTCCTGGCCCAGATCGGCGATCAGTACGCGGACCTGTTCCTCGCTGCCAAGAATCTGTCGGACGCCATCCTCCTGTCTGATATTCTCAGGGTGAACACCGAGATTACGAAGGCTCCGCTCTCAGCCTCCATGATCAAGCGCTACGACGAGCACCATCAGGATCTGACCCTCCTGAAGGCGCTGGTCAGGCAGCAGCTCCCCGAGAAGTACAAGGAGATCTTCTTCGATCAGTCGAAGAACGGCTACGCTGGGTACATTGACGGCGGGGCCTCTCAGGAGGAGTTCTACAAGTTCATCAAGCCGATTCTGGAGAAGATGGACGGCACGGAGGAGCTGCTGGTGAAGCTCAAGCGCGAGGACCTCCTGAGGAAGCAGCGGACATTCGATAACGGCAGCATCCCACACCAGATTCATCTCGGGGAGCTGCACGCTATCCTGAGGAGGCAGGAGGACTTCTACCCTTTCCTCAAGGATAACCGCGAGAAGATCGAGAAGATTCTGACTTTCAGGATCCCGTACTACGTCGGCCCACTCGCTAGGGGCAACTCCCGCTTCGCTTGGATGACCCGCAAGTCAGAGGAGACGATCACGCCGTGGAACTTCGAGGAGGTGGTCGACAAGGGCGCTAGCGCTCAGTCGTTCATCGAGAGGATGACGAATTTCGACAAGAACCTGCCAAATGAGAAGGTGCTCCCTAAGCACTCGCTCCTGTACGAGTACTTCACAGTCTACAACGAGCTGACTAAGGTGAAGTATGTGACCGAGGGCATGAGGAAGCCGGCTTTCCTGTCTGGGGAGCAGAAGAAGGCCATCGTGGACCTCCTGTTCAAGACCAACCGGAAGGTCACGGTTAAGCAGCTCAAGGAGGACTACTTCAAGAAGATTGAGTGCTTCGATTCGGTCGAGATCTCTGGCGTTGAGGACCGCTTCAACGCCTCCCTGGGGACCTACCACGATCTCCTGAAGATCATTAAGGATAAGGACTTCCTGGACAACGAGGAGAATGAGGATATCCTCGAGGACATTGTGCTGACACTCACTCTGTTCGAGGACCGGGAGATGATCGAGGAGCGCCTGAAGACTTACGCCCATCTCTTCGATGACAAGGTCATGAAGCAGCTCAAGAGGAGGAGGTACACCGGCTGGGGGAGGCTGAGCAGGAAGCTCATCAACGGCATTCGGGACAAGCAGTCCGGGAAGACGATCCTCGACTTCCTGAAGAGCGATGGCTTCGCGAACCGCAATTTCATGCAGCTGATTCACGATGACAGCCTCACATTCAAGGAGGATATCCAGAAGGCTCAGGTGAGCGGCCAGGGGGACTCGCTGCACGAGCATATCGCGAACCTCGCTGGCTCGCCAGCTATCAAGAAGGGGATTCTGCAGACCGTGAAGGTTGTGGACGAGCTGGTGAAGGTCATGGGCAGGCACAAGCCTGAGAACATCGTCATTGAGATGGCCCGGGAGAATCAGACCACGCAGAAGGGCCAGAAGAACTCACGCGAGAGGATGAAGAGGATCGAGGAGGGCATTAAGGAGCTGGGGTCCCAGATCCTCAAGGAGCACCCGGTGGAGAACACGCAGCTGCAGAATGAGAAGCTCTACCTGTACTACCTCCAGAATGGCCGCGATATGTATGTGGACCAGGAGCTGGATATTAACAGGCTCAGCGATTACGACGTCGATGCCATCGTTCCACAGTCATTCCTGAAGGATGACTCCATTGACAACAAGGTCCTCACCAGGTCGGACAAGAACCGGGGCAAGTCTGATAATGTTCCTTCAGAGGAGGTCGTTAAGAAGATGAAGAACTACTGGCGCCAGCTCCTGAATGCCAAGCTGATCACGCAGCGGAAGTTCGATAACCTCACAAAGGCTGAGAGGGGCGGGCTCTCTGAGCTGGACAAGGCGGGCTTCATCAAGAGGCAGCTGGTCGAGACACGGCAGATCACTAAGCACGTTGCGCAGATTCTCGACTCACGGATGAACACTAAGTACGATGAGAATGACAAGCTGATCCGCGAGGTGAAGGTCATCACCCTGAAGTCAAAGCTCGTCTCCGACTTCAGGAAGGATTTCCAGTTCTACAAGGTTCGGGAGATCAACAATTACCACCATGCCCATGACGCGTACCTGAACGCGGTGGTCGGCACAGCTCTGATCAAGAAGTACCCAAAGCTCGAGAGCGAGTTCGTGTACGGGGACTACAAGGTTTACGATGTGAGGAAGATGATCGCCAAGTCGGAGCAGGAGATTGGCAAGGCTACCGCCAAGTACTTCTTCTACTCTAACATTATGAATTTCTTCAAGACAGAGATCACTCTGGCCAATGGCGAGATCCGGAAGCGCCCCCTCATCGAGACGAACGGCGAGACGGGGGAGATCGTGTGGGACAAGGGCAGGGATTTCGCGACCGTCAGGAAGGTTCTCTCCATGCCACAAGTGAATATCGTCAAGAAGACAGAGGTCCAGACTGGCGGGTTCTCTAAGGAGTCAATTCTGCCTAAGCGGAACAGCGACAAGCTCATCGCCCGCAAGAAGGACTGGGATCCGAAGAAGTACGGCGGGTTCGACAGCCCCACTGTGGCCTACTCGGTCCTGGTTGTGGCGAAGGTTGAGAAGGGCAAGTCCAAGAAGCTCAAGAGCGTGAAGGAGCTGCTGGGGATCACGATTATGGAGCGCTCCAGCTTCGAGAAGAACCCGATCGATTTCCTGGAGGCGAAGGGCTACAAGGAGGTGAAGAAGGACCTGATCATTAAGCTCCCCAAGTACTCACTCTTCGAGCTGGAGAACGGCAGGAAGCGGATGCTGGCTTCCGCTGGCGAGCTGCAGAAGGGGAACGAGCTGGCTCTGCCGTCCAAGTATGTGAACTTCCTCTACCTGGCCTCCCACTACGAGAAGCTCAAGGGCAGCCCCGAGGACAACGAGCAGAAGCAGCTGTTCGTCGAGCAGCACAAGCATTACCTCGACGAGATCATTGAGCAGATTTCCGAGTTCTCCAAGCGCGTGATCCTGGCCGACGCGAATCTGGATAAGGTCCTCTCCGCGTACAACAAGCACCGCGACAAGCCAATCAGGGAGCAGGCTGAGAATATCATTCATCTCTTCACCCTGACGAACCTCGGCGCCCCTGCTGCTTTCAAGTACTTCGACACAACTATCGATCGCAAGAGGTACACAAGCACTAAGGAGGTCCTGGACGCGACCCTCATCCACCAGTCGATTACCGGCCTCTACGAGACGCGCATCGACCTGTCTCAGCTCGGGGGCGACGAATTCTCCGGGAGCGAGACGCCAGGCACCTCCGAGTCGGCCACCCCAGAATCTGCCACAGTGGTGTCCGGCCAAAAGCAGGACCGCCAGGGCGGAGAACGCAGAAGGTCCCAGCTCGATAGGGATCAGTGTGCCTACTGCAAGGAGAAGGGCCACTGGGCCAAAGACTGCCCGAAAAAGCCGCGCGGCCCACGCGGCCCAAGGCCACAAACATCCCTCCTTCCAAAGAAGAAGCGGAAGGTGGAGCTCAGCGGAGGATCTTCCGGAGGATCTAGCGGCTCCGAGACACCAGGAACATCCGAAAGCGCTACACCAGAATCTAGCGGAGGCTCTTCCGGAGGATCTAGGCCTTTCAATCTCGCCGACGAATACCGTATTCATGAGAAGGGAAAGAAGATGGATAACCCACAGTGGTGGTTGAGTAGGTTCCCGGAGGCGTGGGCAGAAACGGGCGGCGTGGGTATGGCAAGCCAGGTGCCTCCAATTGTTATCGCTGTGAAGTCTGGCGCCACCCCGATCTCTGTCAGGCAGTATCCCATGGGGAAAGAGGCAAAAGAGGGGATACGACCTCATATTAACAAATTTCTTCAGCTGGGCATCTTGACCCCATGTAGAAGCAGCTGGAATACTCCCCTGCTTCCCGTGAAGAAACCTGGCACTAGAGACTACCGGCCCGTGCAAGATTTGAGAGAGGTGAACAAAAGAGTAGAAGATGTGCACCCTATCGTGCCGAACCCTTATACCCTGCTGAGCACGCTGCCTCCAGAGAGGATATGGTACACCGTCCTTGACCTCAAGGACGCCTTCTTCTCCATGAGGCTCCACCCTACCAGCCAGCACCTGTTTGCGTTTGAGTGGAAGGATCCAGAAACCGGCCACTCTGGACAGTTAACATGGACCAGACTGCCGCAGGGATTCAAGAATAGTCCGACGCTCTTTGATGAAGCTTTGCACCGCGACCTCATGATATTCAACAATCCTCAGCTGACGCTGCTTCAGTACGTGGACGATCTGCTGCTGGGCGCCGAAACCCAGGACGAGTGCCAAAAGGGCACAGAGCAGCTGCTGTCTGAGCTGGGTCGACTAGGGTACAGAGCTTCGGCCAAAAAAGCACAGCTTTGTGAGGTGACATATCTGGGCTACATCCTCAGAGATGGCAAGAGATGGTTAACGGAGGCCCGGAAGAAGACAGTGCTGCAAATCCCTGTCCCTCAGACACCGCGTCAGGTTAGAGAATTCCTCGGCACGGCCGGTTTCTGCCGCCTGTGGATCCCTGGCTTCGCGGCCCTGGCGGCGCCCCTCCACACATTAACACGGCTGGAAACAGAATTTCAATGGAAGCCAGAGCACCAGATGGCGTTCGACGCCATCAAGCGGGCCCTCCTCACGGCTCCGGCTTTGGTGCTGCCAGACCTGACCAAGCCGTTCATGTTGTTCGTCGACGAGAGAGCCGGCGTAGCCAAAGGAGTGCTGACACAGACACTAGGCCCATGGAAGCGCCCTGTGGCATACCTCAGCAAAAAGCTGGACCCCGTCGCGAGCGGCTGGCCATCGTGCCTCAAAATAATCGCCGCCGTGGCCACCCTCATCAAAGACTCCGACAAGCTCACCCTCGGCCAACCGGTCACTATCATCGCCCCCCATGCCCTCGAATCCGTGATCAGACAGCCTCCTGATCGGTGGATAACGAACGCGAGACTGACCCACTACCAATCCCTTCTGCTAAATGATAGAGTGTCCTTTGGGACGCCCGTAATTCTCAACCCCGCCACTCTGCTGCCGGAGACCACGGGCCAGAACCCTATCCATGTCTGCACCGACATTCTTGCCGAGGCCAGCTCAACAAGAGAGGACCTCACCGACGTACCTCTCCCCGGCTGCCCTTCATGGTTCACAGACGGAAGTAATTTCCTGGTGGAGGGGAAAAGGAAGGCGGGCGCCGCCGTGGTTGATGGTAGGACTATCTGGAGCAGCGGCCTGCCTGAAGGTACCAGCGCACAGAAGGCCGAGCTCATCGCTCTAACACAAGCATTACGGCTGGCAGAAGGAAAAAATATTAACATTTACACAGATTCTCGATATGCTTTCGCCACCGCGCACGTGCATGGGGCCATCTACAGGCAGCGAGGCTTACTGACTAGCACAGGCAAGGAAATAAAAAACAAGGATGAGATCTTATCCCTGCTAGAGGCCATCCACCTGCCTCGCAAGGTTGCCGTTATCCACTGCCCCGGTCACCAAGGCGGCCAGGATGCCGTGGCCAAGGGGAACAGAATGGCCGATGCCACCGCTAAGCAGGCTGCTTTGGGTGCCGTGATCCTCACCGTGGTAAACAAGAGCGGCGGCAGCAAAAGAACGGCGGACGGCTCTGAGAAGCGCACCGCTGATAGCCAGCATTCAACTCCTCCGAAAACAAAGAGGAAAGTTGAGTTCGAACCGAAGAAGAAAAGGAAGGTGTGA

**Sequence 50 Plasmids sequence of ePPEplus-RT49**

(NLSSV40-nCas9(H840A/R221K/N394K)-XTEN-NC-NLS-32aa Linker-RT49-NLSvbp)

CCTAAGAAAAAGAGAAAAGTGGACAAGAAGTACTCGATCGGCCTCGATATTGGGACTAACTCTGTTGGCTGGGCCGTGATCACCGACGAGTACAAGGTGCCCTCAAAGAAGTTCAAGGTCCTGGGCAACACCGATCGGCATTCCATCAAGAAGAATCTCATTGGCGCTCTCCTGTTCGACAGCGGCGAGACGGCTGAGGCTACGCGGCTCAAGCGCACCGCCCGCAGGCGGTACACGCGCAGGAAGAATCGCATCTGCTACCTGCAGGAGATTTTCTCCAACGAGATGGCGAAGGTTGACGATTCTTTCTTCCACAGGCTGGAGGAGTCATTCCTCGTGGAGGAGGATAAGAAGCACGAGCGGCATCCAATCTTCGGCAACATTGTCGACGAGGTTGCCTACCACGAGAAGTACCCTACGATCTACCATCTGCGGAAGAAGCTCGTGGACTCCACAGATAAGGCGGACCTCCGCCTGATCTACCTCGCTCTGGCCCACATGATTAAGTTCAGGGGCCATTTCCTGATCGAGGGGGATCTCAACCCGGACAATAGCGATGTTGACAAGCTGTTCATCCAGCTCGTGCAGACGTACAACCAGCTCTTCGAGGAGAACCCCATTAATGCGTCAGGCGTCGACGCGAAGGCTATCCTGTCCGCTAGGCTCTCGAAGTCTCGGAAGCTCGAGAACCTGATCGCCCAGCTGCCGGGCGAGAAGAAGAACGGCCTGTTCGGGAATCTCATTGCGCTCAGCCTGGGGCTCACGCCCAACTTCAAGTCGAATTTCGATCTCGCTGAGGACGCCAAGCTGCAGCTCTCCAAGGACACATACGACGATGACCTGGATAACCTCCTGGCCCAGATCGGCGATCAGTACGCGGACCTGTTCCTCGCTGCCAAGAATCTGTCGGACGCCATCCTCCTGTCTGATATTCTCAGGGTGAACACCGAGATTACGAAGGCTCCGCTCTCAGCCTCCATGATCAAGCGCTACGACGAGCACCATCAGGATCTGACCCTCCTGAAGGCGCTGGTCAGGCAGCAGCTCCCCGAGAAGTACAAGGAGATCTTCTTCGATCAGTCGAAGAACGGCTACGCTGGGTACATTGACGGCGGGGCCTCTCAGGAGGAGTTCTACAAGTTCATCAAGCCGATTCTGGAGAAGATGGACGGCACGGAGGAGCTGCTGGTGAAGCTCAAGCGCGAGGACCTCCTGAGGAAGCAGCGGACATTCGATAACGGCAGCATCCCACACCAGATTCATCTCGGGGAGCTGCACGCTATCCTGAGGAGGCAGGAGGACTTCTACCCTTTCCTCAAGGATAACCGCGAGAAGATCGAGAAGATTCTGACTTTCAGGATCCCGTACTACGTCGGCCCACTCGCTAGGGGCAACTCCCGCTTCGCTTGGATGACCCGCAAGTCAGAGGAGACGATCACGCCGTGGAACTTCGAGGAGGTGGTCGACAAGGGCGCTAGCGCTCAGTCGTTCATCGAGAGGATGACGAATTTCGACAAGAACCTGCCAAATGAGAAGGTGCTCCCTAAGCACTCGCTCCTGTACGAGTACTTCACAGTCTACAACGAGCTGACTAAGGTGAAGTATGTGACCGAGGGCATGAGGAAGCCGGCTTTCCTGTCTGGGGAGCAGAAGAAGGCCATCGTGGACCTCCTGTTCAAGACCAACCGGAAGGTCACGGTTAAGCAGCTCAAGGAGGACTACTTCAAGAAGATTGAGTGCTTCGATTCGGTCGAGATCTCTGGCGTTGAGGACCGCTTCAACGCCTCCCTGGGGACCTACCACGATCTCCTGAAGATCATTAAGGATAAGGACTTCCTGGACAACGAGGAGAATGAGGATATCCTCGAGGACATTGTGCTGACACTCACTCTGTTCGAGGACCGGGAGATGATCGAGGAGCGCCTGAAGACTTACGCCCATCTCTTCGATGACAAGGTCATGAAGCAGCTCAAGAGGAGGAGGTACACCGGCTGGGGGAGGCTGAGCAGGAAGCTCATCAACGGCATTCGGGACAAGCAGTCCGGGAAGACGATCCTCGACTTCCTGAAGAGCGATGGCTTCGCGAACCGCAATTTCATGCAGCTGATTCACGATGACAGCCTCACATTCAAGGAGGATATCCAGAAGGCTCAGGTGAGCGGCCAGGGGGACTCGCTGCACGAGCATATCGCGAACCTCGCTGGCTCGCCAGCTATCAAGAAGGGGATTCTGCAGACCGTGAAGGTTGTGGACGAGCTGGTGAAGGTCATGGGCAGGCACAAGCCTGAGAACATCGTCATTGAGATGGCCCGGGAGAATCAGACCACGCAGAAGGGCCAGAAGAACTCACGCGAGAGGATGAAGAGGATCGAGGAGGGCATTAAGGAGCTGGGGTCCCAGATCCTCAAGGAGCACCCGGTGGAGAACACGCAGCTGCAGAATGAGAAGCTCTACCTGTACTACCTCCAGAATGGCCGCGATATGTATGTGGACCAGGAGCTGGATATTAACAGGCTCAGCGATTACGACGTCGATGCCATCGTTCCACAGTCATTCCTGAAGGATGACTCCATTGACAACAAGGTCCTCACCAGGTCGGACAAGAACCGGGGCAAGTCTGATAATGTTCCTTCAGAGGAGGTCGTTAAGAAGATGAAGAACTACTGGCGCCAGCTCCTGAATGCCAAGCTGATCACGCAGCGGAAGTTCGATAACCTCACAAAGGCTGAGAGGGGCGGGCTCTCTGAGCTGGACAAGGCGGGCTTCATCAAGAGGCAGCTGGTCGAGACACGGCAGATCACTAAGCACGTTGCGCAGATTCTCGACTCACGGATGAACACTAAGTACGATGAGAATGACAAGCTGATCCGCGAGGTGAAGGTCATCACCCTGAAGTCAAAGCTCGTCTCCGACTTCAGGAAGGATTTCCAGTTCTACAAGGTTCGGGAGATCAACAATTACCACCATGCCCATGACGCGTACCTGAACGCGGTGGTCGGCACAGCTCTGATCAAGAAGTACCCAAAGCTCGAGAGCGAGTTCGTGTACGGGGACTACAAGGTTTACGATGTGAGGAAGATGATCGCCAAGTCGGAGCAGGAGATTGGCAAGGCTACCGCCAAGTACTTCTTCTACTCTAACATTATGAATTTCTTCAAGACAGAGATCACTCTGGCCAATGGCGAGATCCGGAAGCGCCCCCTCATCGAGACGAACGGCGAGACGGGGGAGATCGTGTGGGACAAGGGCAGGGATTTCGCGACCGTCAGGAAGGTTCTCTCCATGCCACAAGTGAATATCGTCAAGAAGACAGAGGTCCAGACTGGCGGGTTCTCTAAGGAGTCAATTCTGCCTAAGCGGAACAGCGACAAGCTCATCGCCCGCAAGAAGGACTGGGATCCGAAGAAGTACGGCGGGTTCGACAGCCCCACTGTGGCCTACTCGGTCCTGGTTGTGGCGAAGGTTGAGAAGGGCAAGTCCAAGAAGCTCAAGAGCGTGAAGGAGCTGCTGGGGATCACGATTATGGAGCGCTCCAGCTTCGAGAAGAACCCGATCGATTTCCTGGAGGCGAAGGGCTACAAGGAGGTGAAGAAGGACCTGATCATTAAGCTCCCCAAGTACTCACTCTTCGAGCTGGAGAACGGCAGGAAGCGGATGCTGGCTTCCGCTGGCGAGCTGCAGAAGGGGAACGAGCTGGCTCTGCCGTCCAAGTATGTGAACTTCCTCTACCTGGCCTCCCACTACGAGAAGCTCAAGGGCAGCCCCGAGGACAACGAGCAGAAGCAGCTGTTCGTCGAGCAGCACAAGCATTACCTCGACGAGATCATTGAGCAGATTTCCGAGTTCTCCAAGCGCGTGATCCTGGCCGACGCGAATCTGGATAAGGTCCTCTCCGCGTACAACAAGCACCGCGACAAGCCAATCAGGGAGCAGGCTGAGAATATCATTCATCTCTTCACCCTGACGAACCTCGGCGCCCCTGCTGCTTTCAAGTACTTCGACACAACTATCGATCGCAAGAGGTACACAAGCACTAAGGAGGTCCTGGACGCGACCCTCATCCACCAGTCGATTACCGGCCTCTACGAGACGCGCATCGACCTGTCTCAGCTCGGGGGCGACGAATTCTCCGGGAGCGAGACGCCAGGCACCTCCGAGTCGGCCACCCCAGAATCTGCCACAGTGGTGTCCGGCCAAAAGCAGGACCGCCAGGGCGGAGAACGCAGAAGGTCCCAGCTCGATAGGGATCAGTGTGCCTACTGCAAGGAGAAGGGCCACTGGGCCAAAGACTGCCCGAAAAAGCCGCGCGGCCCACGCGGCCCAAGGCCACAAACATCCCTCCTTCCAAAGAAGAAGCGGAAGGTGGAGCTCAGCGGAGGATCTTCCGGAGGATCTAGCGGCTCCGAGACACCAGGAACATCCGAAAGCGCTACACCAGAATCTAGCGGAGGCTCTTCCGGAGGATCTAGGCCTAACCTCGTCAAGCAGCTGGCGCAGCACCTCGGCAAGTCTGAGTACGAGGTGAGTTTGTTCCTCGTCGACGCGCCCAACAAGTACCGCGTGTACAAGATCCCCAAGAGGACATATGGCCACCGCGTCATCGCTCAACCATCAAAGGAGCTGAAAAAATACCAGCGGGCATTTCTTGACATTTACCCGTTCCCGGTGCACTCCGCCGCCATGGCGTACTGCGAGGGGAAGAGTATAAAAGATAATGCTCAAGCACATGTGCAGAATGATTATCTTCTCAAAACTGATTTAGAAAATTTCTTCAACTCGATAACTCCAACTATATTTTGGAGCTGTATTGAATCATGTAGTCTCGACACGCCTTCTTTTACAACAAATGAGAAGAAACTGGTGGAGAGCCTGCTCTTCTGGTGCCCTTCCAAAACAAAGAATGGAAAATTGGTTCTTTCAGTTGGTGCACCATCGTCGCCGACGATAAGCAACTTCTGCCTCTATGAGTTTGATAGATACCTCTCTACCATCTGCAACTTTCAAAATATCACCTACACACGGTATGCTGACGACCTCACCTTCTCCACCAAGAAGAAGGATATTCTGTACACTGTTATTCCTTCACTGCAGGATCTTTTGGTGAAGCTCTTTTCTCAATCTATTCGTCTTAACCATTCCAAGACGGTGTTCTCATCCCGCGCGCACAACAGACATGTCACCGGCGTCACTCTGAACAATGACCGCCGTCTCTCTTTGGGGAGGCAGAGGAAACGGTACATCAAGCATCTTGTAAACCAGTTCAAGTATGAGCAGCTTGATGAAGTTGACACAAGGCATCTGCAAGGTCTTCTCGCCTTCGCCAAGCACATCGAGCCGACGTTCATCAAACGGCTGAAGGACAAGTACAGCTCGGAACTGATCAAAAGAATTTATGAAGCTGGAAATGAAAGCGGCGGCAGCAAAAGAACGGCGGACGGCTCTGAGAAGCGCACCGCTGATAGCCAGCATTCAACTCCTCCGAAAACAAAGAGGAAAGTTGAGTTCGAACCGAAGAAGAAAAGGAAGGTGTGA

**Sequence 51 Plasmids sequence of ePPEplus-RT50**

(NLSSV40-nCas9(H840A/R221K/N394K)-XTEN-NC-NLS-32aa Linker-RT50-NLSvbp)

CCTAAGAAAAAGAGAAAAGTGGACAAGAAGTACTCGATCGGCCTCGATATTGGGACTAACTCTGTTGGCTGGGCCGTGATCACCGACGAGTACAAGGTGCCCTCAAAGAAGTTCAAGGTCCTGGGCAACACCGATCGGCATTCCATCAAGAAGAATCTCATTGGCGCTCTCCTGTTCGACAGCGGCGAGACGGCTGAGGCTACGCGGCTCAAGCGCACCGCCCGCAGGCGGTACACGCGCAGGAAGAATCGCATCTGCTACCTGCAGGAGATTTTCTCCAACGAGATGGCGAAGGTTGACGATTCTTTCTTCCACAGGCTGGAGGAGTCATTCCTCGTGGAGGAGGATAAGAAGCACGAGCGGCATCCAATCTTCGGCAACATTGTCGACGAGGTTGCCTACCACGAGAAGTACCCTACGATCTACCATCTGCGGAAGAAGCTCGTGGACTCCACAGATAAGGCGGACCTCCGCCTGATCTACCTCGCTCTGGCCCACATGATTAAGTTCAGGGGCCATTTCCTGATCGAGGGGGATCTCAACCCGGACAATAGCGATGTTGACAAGCTGTTCATCCAGCTCGTGCAGACGTACAACCAGCTCTTCGAGGAGAACCCCATTAATGCGTCAGGCGTCGACGCGAAGGCTATCCTGTCCGCTAGGCTCTCGAAGTCTCGGAAGCTCGAGAACCTGATCGCCCAGCTGCCGGGCGAGAAGAAGAACGGCCTGTTCGGGAATCTCATTGCGCTCAGCCTGGGGCTCACGCCCAACTTCAAGTCGAATTTCGATCTCGCTGAGGACGCCAAGCTGCAGCTCTCCAAGGACACATACGACGATGACCTGGATAACCTCCTGGCCCAGATCGGCGATCAGTACGCGGACCTGTTCCTCGCTGCCAAGAATCTGTCGGACGCCATCCTCCTGTCTGATATTCTCAGGGTGAACACCGAGATTACGAAGGCTCCGCTCTCAGCCTCCATGATCAAGCGCTACGACGAGCACCATCAGGATCTGACCCTCCTGAAGGCGCTGGTCAGGCAGCAGCTCCCCGAGAAGTACAAGGAGATCTTCTTCGATCAGTCGAAGAACGGCTACGCTGGGTACATTGACGGCGGGGCCTCTCAGGAGGAGTTCTACAAGTTCATCAAGCCGATTCTGGAGAAGATGGACGGCACGGAGGAGCTGCTGGTGAAGCTCAAGCGCGAGGACCTCCTGAGGAAGCAGCGGACATTCGATAACGGCAGCATCCCACACCAGATTCATCTCGGGGAGCTGCACGCTATCCTGAGGAGGCAGGAGGACTTCTACCCTTTCCTCAAGGATAACCGCGAGAAGATCGAGAAGATTCTGACTTTCAGGATCCCGTACTACGTCGGCCCACTCGCTAGGGGCAACTCCCGCTTCGCTTGGATGACCCGCAAGTCAGAGGAGACGATCACGCCGTGGAACTTCGAGGAGGTGGTCGACAAGGGCGCTAGCGCTCAGTCGTTCATCGAGAGGATGACGAATTTCGACAAGAACCTGCCAAATGAGAAGGTGCTCCCTAAGCACTCGCTCCTGTACGAGTACTTCACAGTCTACAACGAGCTGACTAAGGTGAAGTATGTGACCGAGGGCATGAGGAAGCCGGCTTTCCTGTCTGGGGAGCAGAAGAAGGCCATCGTGGACCTCCTGTTCAAGACCAACCGGAAGGTCACGGTTAAGCAGCTCAAGGAGGACTACTTCAAGAAGATTGAGTGCTTCGATTCGGTCGAGATCTCTGGCGTTGAGGACCGCTTCAACGCCTCCCTGGGGACCTACCACGATCTCCTGAAGATCATTAAGGATAAGGACTTCCTGGACAACGAGGAGAATGAGGATATCCTCGAGGACATTGTGCTGACACTCACTCTGTTCGAGGACCGGGAGATGATCGAGGAGCGCCTGAAGACTTACGCCCATCTCTTCGATGACAAGGTCATGAAGCAGCTCAAGAGGAGGAGGTACACCGGCTGGGGGAGGCTGAGCAGGAAGCTCATCAACGGCATTCGGGACAAGCAGTCCGGGAAGACGATCCTCGACTTCCTGAAGAGCGATGGCTTCGCGAACCGCAATTTCATGCAGCTGATTCACGATGACAGCCTCACATTCAAGGAGGATATCCAGAAGGCTCAGGTGAGCGGCCAGGGGGACTCGCTGCACGAGCATATCGCGAACCTCGCTGGCTCGCCAGCTATCAAGAAGGGGATTCTGCAGACCGTGAAGGTTGTGGACGAGCTGGTGAAGGTCATGGGCAGGCACAAGCCTGAGAACATCGTCATTGAGATGGCCCGGGAGAATCAGACCACGCAGAAGGGCCAGAAGAACTCACGCGAGAGGATGAAGAGGATCGAGGAGGGCATTAAGGAGCTGGGGTCCCAGATCCTCAAGGAGCACCCGGTGGAGAACACGCAGCTGCAGAATGAGAAGCTCTACCTGTACTACCTCCAGAATGGCCGCGATATGTATGTGGACCAGGAGCTGGATATTAACAGGCTCAGCGATTACGACGTCGATGCCATCGTTCCACAGTCATTCCTGAAGGATGACTCCATTGACAACAAGGTCCTCACCAGGTCGGACAAGAACCGGGGCAAGTCTGATAATGTTCCTTCAGAGGAGGTCGTTAAGAAGATGAAGAACTACTGGCGCCAGCTCCTGAATGCCAAGCTGATCACGCAGCGGAAGTTCGATAACCTCACAAAGGCTGAGAGGGGCGGGCTCTCTGAGCTGGACAAGGCGGGCTTCATCAAGAGGCAGCTGGTCGAGACACGGCAGATCACTAAGCACGTTGCGCAGATTCTCGACTCACGGATGAACACTAAGTACGATGAGAATGACAAGCTGATCCGCGAGGTGAAGGTCATCACCCTGAAGTCAAAGCTCGTCTCCGACTTCAGGAAGGATTTCCAGTTCTACAAGGTTCGGGAGATCAACAATTACCACCATGCCCATGACGCGTACCTGAACGCGGTGGTCGGCACAGCTCTGATCAAGAAGTACCCAAAGCTCGAGAGCGAGTTCGTGTACGGGGACTACAAGGTTTACGATGTGAGGAAGATGATCGCCAAGTCGGAGCAGGAGATTGGCAAGGCTACCGCCAAGTACTTCTTCTACTCTAACATTATGAATTTCTTCAAGACAGAGATCACTCTGGCCAATGGCGAGATCCGGAAGCGCCCCCTCATCGAGACGAACGGCGAGACGGGGGAGATCGTGTGGGACAAGGGCAGGGATTTCGCGACCGTCAGGAAGGTTCTCTCCATGCCACAAGTGAATATCGTCAAGAAGACAGAGGTCCAGACTGGCGGGTTCTCTAAGGAGTCAATTCTGCCTAAGCGGAACAGCGACAAGCTCATCGCCCGCAAGAAGGACTGGGATCCGAAGAAGTACGGCGGGTTCGACAGCCCCACTGTGGCCTACTCGGTCCTGGTTGTGGCGAAGGTTGAGAAGGGCAAGTCCAAGAAGCTCAAGAGCGTGAAGGAGCTGCTGGGGATCACGATTATGGAGCGCTCCAGCTTCGAGAAGAACCCGATCGATTTCCTGGAGGCGAAGGGCTACAAGGAGGTGAAGAAGGACCTGATCATTAAGCTCCCCAAGTACTCACTCTTCGAGCTGGAGAACGGCAGGAAGCGGATGCTGGCTTCCGCTGGCGAGCTGCAGAAGGGGAACGAGCTGGCTCTGCCGTCCAAGTATGTGAACTTCCTCTACCTGGCCTCCCACTACGAGAAGCTCAAGGGCAGCCCCGAGGACAACGAGCAGAAGCAGCTGTTCGTCGAGCAGCACAAGCATTACCTCGACGAGATCATTGAGCAGATTTCCGAGTTCTCCAAGCGCGTGATCCTGGCCGACGCGAATCTGGATAAGGTCCTCTCCGCGTACAACAAGCACCGCGACAAGCCAATCAGGGAGCAGGCTGAGAATATCATTCATCTCTTCACCCTGACGAACCTCGGCGCCCCTGCTGCTTTCAAGTACTTCGACACAACTATCGATCGCAAGAGGTACACAAGCACTAAGGAGGTCCTGGACGCGACCCTCATCCACCAGTCGATTACCGGCCTCTACGAGACGCGCATCGACCTGTCTCAGCTCGGGGGCGACGAATTCTCCGGGAGCGAGACGCCAGGCACCTCCGAGTCGGCCACCCCAGAATCTGCCACAGTGGTGTCCGGCCAAAAGCAGGACCGCCAGGGCGGAGAACGCAGAAGGTCCCAGCTCGATAGGGATCAGTGTGCCTACTGCAAGGAGAAGGGCCACTGGGCCAAAGACTGCCCGAAAAAGCCGCGCGGCCCACGCGGCCCAAGGCCACAAACATCCCTCCTTCCAAAGAAGAAGCGGAAGGTGGAGCTCAGCGGAGGATCTTCCGGAGGATCTAGCGGCTCCGAGACACCAGGAACATCCGAAAGCGCTACACCAGAATCTAGCGGAGGCTCTTCCGGAGGATCTAGGCCTACAAGGAAATACAAGCCGCAACCTGTGCGCCGGGTGGAGATTCCCAAGCCTGATGGTGGCGTAAGAAATTTAGGTGTTCCTACGGTGACTGATAGATTTATTCAGCAAGCTATTGCTCAAGTGCTGACACCAATATATGAGGAGCAGTTCCACGACCACTCATATGGTTTTCGTCCCAACCGTTGTGCGCAGCAGGCCATCCTCACTGCGCTCGACATGATGAATGAAGGAAATGATTGGATCGTGGACATCGACCTTGAAAAATTCTTCGACACGGTCAATCATGATAAGCTGATGACAATAATTGGCCGCACCATCAAGGACGGCGACGTCATCTCCATCGTCAGGAAGTACCTGGTATCCGGGATAATGATTGATGATGAGTACGAGGATTCTATTGTTGGAACGCCGCAGGGCGGCAACTTGTCGCCGCTTCTTGCAAATATTATGCTGAATGAACTGGACAAGGAAATGGAGAAGCGGGGCCTCAACTTCGTCCGCTACGCCGACGACTGCATCATCATGGTTGGATCAGAGATGAGCGCCAACAGAGTGATGAGGAACATTTCTCGCTTCATCAAAGAAAAGCTCGGTCTCAAGGTGAACATGACCAAGAGTAAGGTTGATAAACCACAAGGGTTGAAATATCTTGGATTTGGCTTCTACTTTGATTCGCGGGCGCACCAGTTTAAAGCAAAGCCACATGCTAAATCAGTCGCCAAGTTCAAGAAGAGGATGAAGGAGTTGACCTGCCGAAGCAGCGGCGGCAGCAAAAGAACGGCGGACGGCTCTGAGAAGCGCACCGCTGATAGCCAGCATTCAACTCCTCCGAAAACAAAGAGGAAAGTTGAGTTCGAACCGAAGAAGAAAAGGAAGGTGTGA

**Sequence 52 Plasmids sequence of ePPEplus-RT51**

(NLSSV40-nCas9(H840A/R221K/N394K)-XTEN-NC-NLS-32aa Linker-RT51-NLSvbp)

CCTAAGAAAAAGAGAAAAGTGGACAAGAAGTACTCGATCGGCCTCGATATTGGGACTAACTCTGTTGGCTGGGCCGTGATCACCGACGAGTACAAGGTGCCCTCAAAGAAGTTCAAGGTCCTGGGCAACACCGATCGGCATTCCATCAAGAAGAATCTCATTGGCGCTCTCCTGTTCGACAGCGGCGAGACGGCTGAGGCTACGCGGCTCAAGCGCACCGCCCGCAGGCGGTACACGCGCAGGAAGAATCGCATCTGCTACCTGCAGGAGATTTTCTCCAACGAGATGGCGAAGGTTGACGATTCTTTCTTCCACAGGCTGGAGGAGTCATTCCTCGTGGAGGAGGATAAGAAGCACGAGCGGCATCCAATCTTCGGCAACATTGTCGACGAGGTTGCCTACCACGAGAAGTACCCTACGATCTACCATCTGCGGAAGAAGCTCGTGGACTCCACAGATAAGGCGGACCTCCGCCTGATCTACCTCGCTCTGGCCCACATGATTAAGTTCAGGGGCCATTTCCTGATCGAGGGGGATCTCAACCCGGACAATAGCGATGTTGACAAGCTGTTCATCCAGCTCGTGCAGACGTACAACCAGCTCTTCGAGGAGAACCCCATTAATGCGTCAGGCGTCGACGCGAAGGCTATCCTGTCCGCTAGGCTCTCGAAGTCTCGGAAGCTCGAGAACCTGATCGCCCAGCTGCCGGGCGAGAAGAAGAACGGCCTGTTCGGGAATCTCATTGCGCTCAGCCTGGGGCTCACGCCCAACTTCAAGTCGAATTTCGATCTCGCTGAGGACGCCAAGCTGCAGCTCTCCAAGGACACATACGACGATGACCTGGATAACCTCCTGGCCCAGATCGGCGATCAGTACGCGGACCTGTTCCTCGCTGCCAAGAATCTGTCGGACGCCATCCTCCTGTCTGATATTCTCAGGGTGAACACCGAGATTACGAAGGCTCCGCTCTCAGCCTCCATGATCAAGCGCTACGACGAGCACCATCAGGATCTGACCCTCCTGAAGGCGCTGGTCAGGCAGCAGCTCCCCGAGAAGTACAAGGAGATCTTCTTCGATCAGTCGAAGAACGGCTACGCTGGGTACATTGACGGCGGGGCCTCTCAGGAGGAGTTCTACAAGTTCATCAAGCCGATTCTGGAGAAGATGGACGGCACGGAGGAGCTGCTGGTGAAGCTCAAGCGCGAGGACCTCCTGAGGAAGCAGCGGACATTCGATAACGGCAGCATCCCACACCAGATTCATCTCGGGGAGCTGCACGCTATCCTGAGGAGGCAGGAGGACTTCTACCCTTTCCTCAAGGATAACCGCGAGAAGATCGAGAAGATTCTGACTTTCAGGATCCCGTACTACGTCGGCCCACTCGCTAGGGGCAACTCCCGCTTCGCTTGGATGACCCGCAAGTCAGAGGAGACGATCACGCCGTGGAACTTCGAGGAGGTGGTCGACAAGGGCGCTAGCGCTCAGTCGTTCATCGAGAGGATGACGAATTTCGACAAGAACCTGCCAAATGAGAAGGTGCTCCCTAAGCACTCGCTCCTGTACGAGTACTTCACAGTCTACAACGAGCTGACTAAGGTGAAGTATGTGACCGAGGGCATGAGGAAGCCGGCTTTCCTGTCTGGGGAGCAGAAGAAGGCCATCGTGGACCTCCTGTTCAAGACCAACCGGAAGGTCACGGTTAAGCAGCTCAAGGAGGACTACTTCAAGAAGATTGAGTGCTTCGATTCGGTCGAGATCTCTGGCGTTGAGGACCGCTTCAACGCCTCCCTGGGGACCTACCACGATCTCCTGAAGATCATTAAGGATAAGGACTTCCTGGACAACGAGGAGAATGAGGATATCCTCGAGGACATTGTGCTGACACTCACTCTGTTCGAGGACCGGGAGATGATCGAGGAGCGCCTGAAGACTTACGCCCATCTCTTCGATGACAAGGTCATGAAGCAGCTCAAGAGGAGGAGGTACACCGGCTGGGGGAGGCTGAGCAGGAAGCTCATCAACGGCATTCGGGACAAGCAGTCCGGGAAGACGATCCTCGACTTCCTGAAGAGCGATGGCTTCGCGAACCGCAATTTCATGCAGCTGATTCACGATGACAGCCTCACATTCAAGGAGGATATCCAGAAGGCTCAGGTGAGCGGCCAGGGGGACTCGCTGCACGAGCATATCGCGAACCTCGCTGGCTCGCCAGCTATCAAGAAGGGGATTCTGCAGACCGTGAAGGTTGTGGACGAGCTGGTGAAGGTCATGGGCAGGCACAAGCCTGAGAACATCGTCATTGAGATGGCCCGGGAGAATCAGACCACGCAGAAGGGCCAGAAGAACTCACGCGAGAGGATGAAGAGGATCGAGGAGGGCATTAAGGAGCTGGGGTCCCAGATCCTCAAGGAGCACCCGGTGGAGAACACGCAGCTGCAGAATGAGAAGCTCTACCTGTACTACCTCCAGAATGGCCGCGATATGTATGTGGACCAGGAGCTGGATATTAACAGGCTCAGCGATTACGACGTCGATGCCATCGTTCCACAGTCATTCCTGAAGGATGACTCCATTGACAACAAGGTCCTCACCAGGTCGGACAAGAACCGGGGCAAGTCTGATAATGTTCCTTCAGAGGAGGTCGTTAAGAAGATGAAGAACTACTGGCGCCAGCTCCTGAATGCCAAGCTGATCACGCAGCGGAAGTTCGATAACCTCACAAAGGCTGAGAGGGGCGGGCTCTCTGAGCTGGACAAGGCGGGCTTCATCAAGAGGCAGCTGGTCGAGACACGGCAGATCACTAAGCACGTTGCGCAGATTCTCGACTCACGGATGAACACTAAGTACGATGAGAATGACAAGCTGATCCGCGAGGTGAAGGTCATCACCCTGAAGTCAAAGCTCGTCTCCGACTTCAGGAAGGATTTCCAGTTCTACAAGGTTCGGGAGATCAACAATTACCACCATGCCCATGACGCGTACCTGAACGCGGTGGTCGGCACAGCTCTGATCAAGAAGTACCCAAAGCTCGAGAGCGAGTTCGTGTACGGGGACTACAAGGTTTACGATGTGAGGAAGATGATCGCCAAGTCGGAGCAGGAGATTGGCAAGGCTACCGCCAAGTACTTCTTCTACTCTAACATTATGAATTTCTTCAAGACAGAGATCACTCTGGCCAATGGCGAGATCCGGAAGCGCCCCCTCATCGAGACGAACGGCGAGACGGGGGAGATCGTGTGGGACAAGGGCAGGGATTTCGCGACCGTCAGGAAGGTTCTCTCCATGCCACAAGTGAATATCGTCAAGAAGACAGAGGTCCAGACTGGCGGGTTCTCTAAGGAGTCAATTCTGCCTAAGCGGAACAGCGACAAGCTCATCGCCCGCAAGAAGGACTGGGATCCGAAGAAGTACGGCGGGTTCGACAGCCCCACTGTGGCCTACTCGGTCCTGGTTGTGGCGAAGGTTGAGAAGGGCAAGTCCAAGAAGCTCAAGAGCGTGAAGGAGCTGCTGGGGATCACGATTATGGAGCGCTCCAGCTTCGAGAAGAACCCGATCGATTTCCTGGAGGCGAAGGGCTACAAGGAGGTGAAGAAGGACCTGATCATTAAGCTCCCCAAGTACTCACTCTTCGAGCTGGAGAACGGCAGGAAGCGGATGCTGGCTTCCGCTGGCGAGCTGCAGAAGGGGAACGAGCTGGCTCTGCCGTCCAAGTATGTGAACTTCCTCTACCTGGCCTCCCACTACGAGAAGCTCAAGGGCAGCCCCGAGGACAACGAGCAGAAGCAGCTGTTCGTCGAGCAGCACAAGCATTACCTCGACGAGATCATTGAGCAGATTTCCGAGTTCTCCAAGCGCGTGATCCTGGCCGACGCGAATCTGGATAAGGTCCTCTCCGCGTACAACAAGCACCGCGACAAGCCAATCAGGGAGCAGGCTGAGAATATCATTCATCTCTTCACCCTGACGAACCTCGGCGCCCCTGCTGCTTTCAAGTACTTCGACACAACTATCGATCGCAAGAGGTACACAAGCACTAAGGAGGTCCTGGACGCGACCCTCATCCACCAGTCGATTACCGGCCTCTACGAGACGCGCATCGACCTGTCTCAGCTCGGGGGCGACGAATTCTCCGGGAGCGAGACGCCAGGCACCTCCGAGTCGGCCACCCCAGAATCTGCCACAGTGGTGTCCGGCCAAAAGCAGGACCGCCAGGGCGGAGAACGCAGAAGGTCCCAGCTCGATAGGGATCAGTGTGCCTACTGCAAGGAGAAGGGCCACTGGGCCAAAGACTGCCCGAAAAAGCCGCGCGGCCCACGCGGCCCAAGGCCACAAACATCCCTCCTTCCAAAGAAGAAGCGGAAGGTGGAGCTCAGCGGAGGATCTTCCGGAGGATCTAGCGGCTCCGAGACACCAGGAACATCCGAAAGCGCTACACCAGAATCTAGCGGAGGCTCTTCCGGAGGATCTAGGCCTCCTACACCCATCGCTTGCCTGGTACTCAACCTCGAGGAGGAGTATCGGCTTCATGAACAAGGCCCTAAACAGCTGCCAGACCCTGAATGGCTGACCGCCTTTTCCGGCGTATGGGCCGAGCAGGCGGGCATGGGACTGGCAAAGCAGGTACCTCCTGTAGTCGTGGAGCTGAAGGCTGACGCCAGCCCCGTCTCCGTGCGTCAGTACCCCATGTCAAGAGAAGCTAAGGAAGGCATCAGACCACACATCCAGAGACTGATTGAACAGGGCATCCTAGTCCCGTGCCAGTCGCCTTGGAACACTCCACTGTTACCTGTGAGAAAGCCGGGCACCAACGACTACAGACCCGTGCAAGACCTGCGGGAGGTCAACAAGAGAGTGCAGGACATCCATCCCACCGTGCCAAATCCGTATAACCTTCTGAGCAGCCTTCCTCCTGAGAGAACGTGGTACACTGTGCTCGATTTAAAAGACGCGTTCTTCTGTCTGCGTCTTCACCCGAACAGCCAGCCTCTCTTCGCATTTGAGTGGCGTGACCCGGAGGGGGGCCACACCGGCCAGCTGACCTGGACACGGCTGCCGCAAGGATTCAAGAACTCACCTACCCTGTTTGACGAAGCGCTGCACCGCGACCTGGCACCCTTCAGAGCTCAGAACCCACAAATCAGCTTACTCCAATATGTGGATGATTTACTGGTGGCAGCCTCCACAAGAGAACTGTGCTTGGATGGAACTGAAAAACTGCTTAATGAACTTGGTGAACTGGGCTACCGTGTGAGCGCCAAGAAGGCTCAGCTCTGCCGGACAGAGGTGACATACCTGGGATACACCTTGAGAGAGGGAAAGAGGTGGCTCACTGAAGCAAGAAAGAAAACTGTGATGCTCATTCCAACACCTACGACCCCCAGACAGGTGAGAGAGTTCCTGGGCACAGCAGGGTTCTGCAGGCTGTGGATCCCAGGCTTCGCAACCCTGGCGGCTCCACTCTACCCTCTGACCAAGGAGAAGGTTCCGTTCACTTGGACAGAGGAGCATCAGAAGGCTTTCGATTCTATCAAGGCCGCCCTCTTAGCTGCCCCTGCCCTCGCGTTACCTGACCTCACCAAGAGCTTCACCCTATACGTGGACGAGAGGGCCGGCGTAGCCCGCGGCGTGCTGACACAAACACTGGGCCCGTGGAAAAGGCCCGTCGCGTACTTAAGCAAGAAATTGGATCCAGTGGCCAGCGGCTGGCCGTCCTGTCTGAAGGCTATTGCTGCGGTCGCCCTGCTGGTGAAGGATGCTGACAAGTTGACCTTGGGCCAGCACGTCACTGTCATCGCGCCCCATGCCTTGGAGTCTATCGTCCGGCAGCCGCCTGACCGCTGGACCACCAACGCCCGGATGACGCACTACCAGTCCCTGCTGCTGAATGATCGGGTTACATTTGCCCCTCCTGCTATCCTCAACCCTGCCACCCTGCTGCCTCTTGTCGATGATTCCGTGCCTGTTCACAGATGCGCCGATGTCCTGGCAGAAGAGGCCGGCACCAGGAAGGACCTGATCGACCAGCCATGGCCTGGCGTGCCCAACTGGTATACTGATGGATCCTCGTTTGTGGTGGAAGGGAAGCGGCGTGCAGGCGCCGCCGTGGTGGACGGCAAAAAGGTGATATGGGCCTCCTCACTGCCAGAGGGCACCAGTGCTCAGAAGGCAGAGCTGCTGGCCCTAACCCAGGCACTCCAGATGGCTGAAGGTAAGGCCATCAATATATACACCGACAGCAGATATGCCTTCGCCACCGCGCACATCCATGGAGCCATCTACAAGCAGCGCGGCTTGCTCACAAGTGCCGGCAAGGATATTAAAAATAAAGAAGAGATCCTCGCCCTTCTGGAGGCGATCCACCTCCCAAAAAAGGTGGCTATAATTCACTGCCCCGGGCACCAGAAAGGACAAGATCCTGTTGCCCAGGGCAACCAAATGGCAGATATTGCTGCTAAGCAAGCAGCACAAGGCGTCATGGCGTTGGCCGGCAAAAGCGGCCCTAGCTCCGGTCCGTCACCTGTGACTCACCCGGACTGCATCTTTTGTCAAAAGGAAAGCGGCGGCAGCAAAAGAACGGCGGACGGCTCTGAGAAGCGCACCGCTGATAGCCAGCATTCAACTCCTCCGAAAACAAAGAGGAAAGTTGAGTTCGAACCGAAGAAGAAAAGGAAGGTGTGA

**Sequence 53 Plasmids sequence of ePPEplus-RT52**

(NLSSV40-nCas9(H840A/R221K/N394K)-XTEN-NC-NLS-32aa Linker-RT52-NLSvbp)

CCTAAGAAAAAGAGAAAAGTGGACAAGAAGTACTCGATCGGCCTCGATATTGGGACTAACTCTGTTGGCTGGGCCGTGATCACCGACGAGTACAAGGTGCCCTCAAAGAAGTTCAAGGTCCTGGGCAACACCGATCGGCATTCCATCAAGAAGAATCTCATTGGCGCTCTCCTGTTCGACAGCGGCGAGACGGCTGAGGCTACGCGGCTCAAGCGCACCGCCCGCAGGCGGTACACGCGCAGGAAGAATCGCATCTGCTACCTGCAGGAGATTTTCTCCAACGAGATGGCGAAGGTTGACGATTCTTTCTTCCACAGGCTGGAGGAGTCATTCCTCGTGGAGGAGGATAAGAAGCACGAGCGGCATCCAATCTTCGGCAACATTGTCGACGAGGTTGCCTACCACGAGAAGTACCCTACGATCTACCATCTGCGGAAGAAGCTCGTGGACTCCACAGATAAGGCGGACCTCCGCCTGATCTACCTCGCTCTGGCCCACATGATTAAGTTCAGGGGCCATTTCCTGATCGAGGGGGATCTCAACCCGGACAATAGCGATGTTGACAAGCTGTTCATCCAGCTCGTGCAGACGTACAACCAGCTCTTCGAGGAGAACCCCATTAATGCGTCAGGCGTCGACGCGAAGGCTATCCTGTCCGCTAGGCTCTCGAAGTCTCGGAAGCTCGAGAACCTGATCGCCCAGCTGCCGGGCGAGAAGAAGAACGGCCTGTTCGGGAATCTCATTGCGCTCAGCCTGGGGCTCACGCCCAACTTCAAGTCGAATTTCGATCTCGCTGAGGACGCCAAGCTGCAGCTCTCCAAGGACACATACGACGATGACCTGGATAACCTCCTGGCCCAGATCGGCGATCAGTACGCGGACCTGTTCCTCGCTGCCAAGAATCTGTCGGACGCCATCCTCCTGTCTGATATTCTCAGGGTGAACACCGAGATTACGAAGGCTCCGCTCTCAGCCTCCATGATCAAGCGCTACGACGAGCACCATCAGGATCTGACCCTCCTGAAGGCGCTGGTCAGGCAGCAGCTCCCCGAGAAGTACAAGGAGATCTTCTTCGATCAGTCGAAGAACGGCTACGCTGGGTACATTGACGGCGGGGCCTCTCAGGAGGAGTTCTACAAGTTCATCAAGCCGATTCTGGAGAAGATGGACGGCACGGAGGAGCTGCTGGTGAAGCTCAAGCGCGAGGACCTCCTGAGGAAGCAGCGGACATTCGATAACGGCAGCATCCCACACCAGATTCATCTCGGGGAGCTGCACGCTATCCTGAGGAGGCAGGAGGACTTCTACCCTTTCCTCAAGGATAACCGCGAGAAGATCGAGAAGATTCTGACTTTCAGGATCCCGTACTACGTCGGCCCACTCGCTAGGGGCAACTCCCGCTTCGCTTGGATGACCCGCAAGTCAGAGGAGACGATCACGCCGTGGAACTTCGAGGAGGTGGTCGACAAGGGCGCTAGCGCTCAGTCGTTCATCGAGAGGATGACGAATTTCGACAAGAACCTGCCAAATGAGAAGGTGCTCCCTAAGCACTCGCTCCTGTACGAGTACTTCACAGTCTACAACGAGCTGACTAAGGTGAAGTATGTGACCGAGGGCATGAGGAAGCCGGCTTTCCTGTCTGGGGAGCAGAAGAAGGCCATCGTGGACCTCCTGTTCAAGACCAACCGGAAGGTCACGGTTAAGCAGCTCAAGGAGGACTACTTCAAGAAGATTGAGTGCTTCGATTCGGTCGAGATCTCTGGCGTTGAGGACCGCTTCAACGCCTCCCTGGGGACCTACCACGATCTCCTGAAGATCATTAAGGATAAGGACTTCCTGGACAACGAGGAGAATGAGGATATCCTCGAGGACATTGTGCTGACACTCACTCTGTTCGAGGACCGGGAGATGATCGAGGAGCGCCTGAAGACTTACGCCCATCTCTTCGATGACAAGGTCATGAAGCAGCTCAAGAGGAGGAGGTACACCGGCTGGGGGAGGCTGAGCAGGAAGCTCATCAACGGCATTCGGGACAAGCAGTCCGGGAAGACGATCCTCGACTTCCTGAAGAGCGATGGCTTCGCGAACCGCAATTTCATGCAGCTGATTCACGATGACAGCCTCACATTCAAGGAGGATATCCAGAAGGCTCAGGTGAGCGGCCAGGGGGACTCGCTGCACGAGCATATCGCGAACCTCGCTGGCTCGCCAGCTATCAAGAAGGGGATTCTGCAGACCGTGAAGGTTGTGGACGAGCTGGTGAAGGTCATGGGCAGGCACAAGCCTGAGAACATCGTCATTGAGATGGCCCGGGAGAATCAGACCACGCAGAAGGGCCAGAAGAACTCACGCGAGAGGATGAAGAGGATCGAGGAGGGCATTAAGGAGCTGGGGTCCCAGATCCTCAAGGAGCACCCGGTGGAGAACACGCAGCTGCAGAATGAGAAGCTCTACCTGTACTACCTCCAGAATGGCCGCGATATGTATGTGGACCAGGAGCTGGATATTAACAGGCTCAGCGATTACGACGTCGATGCCATCGTTCCACAGTCATTCCTGAAGGATGACTCCATTGACAACAAGGTCCTCACCAGGTCGGACAAGAACCGGGGCAAGTCTGATAATGTTCCTTCAGAGGAGGTCGTTAAGAAGATGAAGAACTACTGGCGCCAGCTCCTGAATGCCAAGCTGATCACGCAGCGGAAGTTCGATAACCTCACAAAGGCTGAGAGGGGCGGGCTCTCTGAGCTGGACAAGGCGGGCTTCATCAAGAGGCAGCTGGTCGAGACACGGCAGATCACTAAGCACGTTGCGCAGATTCTCGACTCACGGATGAACACTAAGTACGATGAGAATGACAAGCTGATCCGCGAGGTGAAGGTCATCACCCTGAAGTCAAAGCTCGTCTCCGACTTCAGGAAGGATTTCCAGTTCTACAAGGTTCGGGAGATCAACAATTACCACCATGCCCATGACGCGTACCTGAACGCGGTGGTCGGCACAGCTCTGATCAAGAAGTACCCAAAGCTCGAGAGCGAGTTCGTGTACGGGGACTACAAGGTTTACGATGTGAGGAAGATGATCGCCAAGTCGGAGCAGGAGATTGGCAAGGCTACCGCCAAGTACTTCTTCTACTCTAACATTATGAATTTCTTCAAGACAGAGATCACTCTGGCCAATGGCGAGATCCGGAAGCGCCCCCTCATCGAGACGAACGGCGAGACGGGGGAGATCGTGTGGGACAAGGGCAGGGATTTCGCGACCGTCAGGAAGGTTCTCTCCATGCCACAAGTGAATATCGTCAAGAAGACAGAGGTCCAGACTGGCGGGTTCTCTAAGGAGTCAATTCTGCCTAAGCGGAACAGCGACAAGCTCATCGCCCGCAAGAAGGACTGGGATCCGAAGAAGTACGGCGGGTTCGACAGCCCCACTGTGGCCTACTCGGTCCTGGTTGTGGCGAAGGTTGAGAAGGGCAAGTCCAAGAAGCTCAAGAGCGTGAAGGAGCTGCTGGGGATCACGATTATGGAGCGCTCCAGCTTCGAGAAGAACCCGATCGATTTCCTGGAGGCGAAGGGCTACAAGGAGGTGAAGAAGGACCTGATCATTAAGCTCCCCAAGTACTCACTCTTCGAGCTGGAGAACGGCAGGAAGCGGATGCTGGCTTCCGCTGGCGAGCTGCAGAAGGGGAACGAGCTGGCTCTGCCGTCCAAGTATGTGAACTTCCTCTACCTGGCCTCCCACTACGAGAAGCTCAAGGGCAGCCCCGAGGACAACGAGCAGAAGCAGCTGTTCGTCGAGCAGCACAAGCATTACCTCGACGAGATCATTGAGCAGATTTCCGAGTTCTCCAAGCGCGTGATCCTGGCCGACGCGAATCTGGATAAGGTCCTCTCCGCGTACAACAAGCACCGCGACAAGCCAATCAGGGAGCAGGCTGAGAATATCATTCATCTCTTCACCCTGACGAACCTCGGCGCCCCTGCTGCTTTCAAGTACTTCGACACAACTATCGATCGCAAGAGGTACACAAGCACTAAGGAGGTCCTGGACGCGACCCTCATCCACCAGTCGATTACCGGCCTCTACGAGACGCGCATCGACCTGTCTCAGCTCGGGGGCGACGAATTCTCCGGGAGCGAGACGCCAGGCACCTCCGAGTCGGCCACCCCAGAATCTGCCACAGTGGTGTCCGGCCAAAAGCAGGACCGCCAGGGCGGAGAACGCAGAAGGTCCCAGCTCGATAGGGATCAGTGTGCCTACTGCAAGGAGAAGGGCCACTGGGCCAAAGACTGCCCGAAAAAGCCGCGCGGCCCACGCGGCCCAAGGCCACAAACATCCCTCCTTCCAAAGAAGAAGCGGAAGGTGGAGCTCAGCGGAGGATCTTCCGGAGGATCTAGCGGCTCCGAGACACCAGGAACATCCGAAAGCGCTACACCAGAATCTAGCGGAGGCTCTTCCGGAGGATCTAGGCCTGACAAGCTGAAACCATATAGCAAGAGCAAAGCTCCTATAACTTCGTTGTCAAAGCTTGCTCAGACGCTGTCTGTATCAGTAGACCAGCTGAATGAGATTGCAGGGCTCCCTGATGAGGAGAAGTATGTGTTGAAGGAGTTGCCCAAGGCCGACGGCTCAAAAAGGATCGTCTACTCCCTTCATCCAAAAATGAGGTTATTGCAAAGTCGTGTCAATAAGCGCATCTTCAAGGAACTCGTGGTGTTCCCGTGCTTCCTCTATGGTTCTGTGCCGGGAAAAAATGACGGTCTTAATTCTCATATAAAAAGAGACTATGTTTCTTGTGCCAAGGCTCATTGTGGAGCAAAAACTGTTCTGAAGGTTGATATCAGCAACTTCTTCGACAACATCCACAAGGATTTAGTTTCTGATGTACTTGAAAATGTTTTGAATATAAAGGACGAGGCCCTTAAGTACCTCACCACCCTGTGCTGCAAGGGCGACTTCATCGTGCAGGGTGCGCTAACAAGCTCCTACATCGCGACGCTCTGTCTGTACGAGAGGGAGGGGGATATTGTTCAACGCGCGCTGAGAAAGAATCTTGTGTACACGAGATTGGTCGACGACATCACCGTCTCCTCCAAGATAAATGATTATGATTTTTCACAGATACTCAACCACATTGAGAACATGCTTTCCGAGCATGATCTCCCGATCAACAAGAGGAAGACGAAAATTTTCCACTGCTCCTCTGAGCCCATCAAGATTCATGGTCTACGTATTGATTACAAATCGCCGCGCCTCCCTAGTGATGAGGTGAAGAGGATACGGGCATCGCTCCACAACCTCAAGCAACTGTCTATAAAGAACAATACAAAGACATCCATCGCCTACAGAAAAGAGTTCAACCGGTGCATGGGGCGAGTCCACAAACTAGGACGCGTCGGCCATGAAAAATATGAACAATTTAAGCAGCAGCTCCTCGCCATCAAACCCATGCCAAGTAAGCGGGACGTCGCTGTCGTGGAGGCGGCGGTGAATTCATTGGAAATTTCCTTTTTTAATGGCAACAACGGCAAGCACTGGTACAAGCGAAAGTACAACCTGACCCTCTACAAGATGATCATTTTTACTCGCAGCGATGCTTTTGATGAAGTTGTTGAAGGCTTCCGCGTGAGATTAAGCAAGGTGCGGCCCATTAGCGGCGGCAGCAAAAGAACGGCGGACGGCTCTGAGAAGCGCACCGCTGATAGCCAGCATTCAACTCCTCCGAAAACAAAGAGGAAAGTTGAGTTCGAACCGAAGAAGAAAAGGAAGGTGTGA

**Sequence 54 Plasmids sequence of ePPEplus-RT53**

(NLSSV40-nCas9(H840A/R221K/N394K)-XTEN-NC-NLS-32aa Linker-RT53-NLSvbp)

CCTAAGAAAAAGAGAAAAGTGGACAAGAAGTACTCGATCGGCCTCGATATTGGGACTAACTCTGTTGGCTGGGCCGTGATCACCGACGAGTACAAGGTGCCCTCAAAGAAGTTCAAGGTCCTGGGCAACACCGATCGGCATTCCATCAAGAAGAATCTCATTGGCGCTCTCCTGTTCGACAGCGGCGAGACGGCTGAGGCTACGCGGCTCAAGCGCACCGCCCGCAGGCGGTACACGCGCAGGAAGAATCGCATCTGCTACCTGCAGGAGATTTTCTCCAACGAGATGGCGAAGGTTGACGATTCTTTCTTCCACAGGCTGGAGGAGTCATTCCTCGTGGAGGAGGATAAGAAGCACGAGCGGCATCCAATCTTCGGCAACATTGTCGACGAGGTTGCCTACCACGAGAAGTACCCTACGATCTACCATCTGCGGAAGAAGCTCGTGGACTCCACAGATAAGGCGGACCTCCGCCTGATCTACCTCGCTCTGGCCCACATGATTAAGTTCAGGGGCCATTTCCTGATCGAGGGGGATCTCAACCCGGACAATAGCGATGTTGACAAGCTGTTCATCCAGCTCGTGCAGACGTACAACCAGCTCTTCGAGGAGAACCCCATTAATGCGTCAGGCGTCGACGCGAAGGCTATCCTGTCCGCTAGGCTCTCGAAGTCTCGGAAGCTCGAGAACCTGATCGCCCAGCTGCCGGGCGAGAAGAAGAACGGCCTGTTCGGGAATCTCATTGCGCTCAGCCTGGGGCTCACGCCCAACTTCAAGTCGAATTTCGATCTCGCTGAGGACGCCAAGCTGCAGCTCTCCAAGGACACATACGACGATGACCTGGATAACCTCCTGGCCCAGATCGGCGATCAGTACGCGGACCTGTTCCTCGCTGCCAAGAATCTGTCGGACGCCATCCTCCTGTCTGATATTCTCAGGGTGAACACCGAGATTACGAAGGCTCCGCTCTCAGCCTCCATGATCAAGCGCTACGACGAGCACCATCAGGATCTGACCCTCCTGAAGGCGCTGGTCAGGCAGCAGCTCCCCGAGAAGTACAAGGAGATCTTCTTCGATCAGTCGAAGAACGGCTACGCTGGGTACATTGACGGCGGGGCCTCTCAGGAGGAGTTCTACAAGTTCATCAAGCCGATTCTGGAGAAGATGGACGGCACGGAGGAGCTGCTGGTGAAGCTCAAGCGCGAGGACCTCCTGAGGAAGCAGCGGACATTCGATAACGGCAGCATCCCACACCAGATTCATCTCGGGGAGCTGCACGCTATCCTGAGGAGGCAGGAGGACTTCTACCCTTTCCTCAAGGATAACCGCGAGAAGATCGAGAAGATTCTGACTTTCAGGATCCCGTACTACGTCGGCCCACTCGCTAGGGGCAACTCCCGCTTCGCTTGGATGACCCGCAAGTCAGAGGAGACGATCACGCCGTGGAACTTCGAGGAGGTGGTCGACAAGGGCGCTAGCGCTCAGTCGTTCATCGAGAGGATGACGAATTTCGACAAGAACCTGCCAAATGAGAAGGTGCTCCCTAAGCACTCGCTCCTGTACGAGTACTTCACAGTCTACAACGAGCTGACTAAGGTGAAGTATGTGACCGAGGGCATGAGGAAGCCGGCTTTCCTGTCTGGGGAGCAGAAGAAGGCCATCGTGGACCTCCTGTTCAAGACCAACCGGAAGGTCACGGTTAAGCAGCTCAAGGAGGACTACTTCAAGAAGATTGAGTGCTTCGATTCGGTCGAGATCTCTGGCGTTGAGGACCGCTTCAACGCCTCCCTGGGGACCTACCACGATCTCCTGAAGATCATTAAGGATAAGGACTTCCTGGACAACGAGGAGAATGAGGATATCCTCGAGGACATTGTGCTGACACTCACTCTGTTCGAGGACCGGGAGATGATCGAGGAGCGCCTGAAGACTTACGCCCATCTCTTCGATGACAAGGTCATGAAGCAGCTCAAGAGGAGGAGGTACACCGGCTGGGGGAGGCTGAGCAGGAAGCTCATCAACGGCATTCGGGACAAGCAGTCCGGGAAGACGATCCTCGACTTCCTGAAGAGCGATGGCTTCGCGAACCGCAATTTCATGCAGCTGATTCACGATGACAGCCTCACATTCAAGGAGGATATCCAGAAGGCTCAGGTGAGCGGCCAGGGGGACTCGCTGCACGAGCATATCGCGAACCTCGCTGGCTCGCCAGCTATCAAGAAGGGGATTCTGCAGACCGTGAAGGTTGTGGACGAGCTGGTGAAGGTCATGGGCAGGCACAAGCCTGAGAACATCGTCATTGAGATGGCCCGGGAGAATCAGACCACGCAGAAGGGCCAGAAGAACTCACGCGAGAGGATGAAGAGGATCGAGGAGGGCATTAAGGAGCTGGGGTCCCAGATCCTCAAGGAGCACCCGGTGGAGAACACGCAGCTGCAGAATGAGAAGCTCTACCTGTACTACCTCCAGAATGGCCGCGATATGTATGTGGACCAGGAGCTGGATATTAACAGGCTCAGCGATTACGACGTCGATGCCATCGTTCCACAGTCATTCCTGAAGGATGACTCCATTGACAACAAGGTCCTCACCAGGTCGGACAAGAACCGGGGCAAGTCTGATAATGTTCCTTCAGAGGAGGTCGTTAAGAAGATGAAGAACTACTGGCGCCAGCTCCTGAATGCCAAGCTGATCACGCAGCGGAAGTTCGATAACCTCACAAAGGCTGAGAGGGGCGGGCTCTCTGAGCTGGACAAGGCGGGCTTCATCAAGAGGCAGCTGGTCGAGACACGGCAGATCACTAAGCACGTTGCGCAGATTCTCGACTCACGGATGAACACTAAGTACGATGAGAATGACAAGCTGATCCGCGAGGTGAAGGTCATCACCCTGAAGTCAAAGCTCGTCTCCGACTTCAGGAAGGATTTCCAGTTCTACAAGGTTCGGGAGATCAACAATTACCACCATGCCCATGACGCGTACCTGAACGCGGTGGTCGGCACAGCTCTGATCAAGAAGTACCCAAAGCTCGAGAGCGAGTTCGTGTACGGGGACTACAAGGTTTACGATGTGAGGAAGATGATCGCCAAGTCGGAGCAGGAGATTGGCAAGGCTACCGCCAAGTACTTCTTCTACTCTAACATTATGAATTTCTTCAAGACAGAGATCACTCTGGCCAATGGCGAGATCCGGAAGCGCCCCCTCATCGAGACGAACGGCGAGACGGGGGAGATCGTGTGGGACAAGGGCAGGGATTTCGCGACCGTCAGGAAGGTTCTCTCCATGCCACAAGTGAATATCGTCAAGAAGACAGAGGTCCAGACTGGCGGGTTCTCTAAGGAGTCAATTCTGCCTAAGCGGAACAGCGACAAGCTCATCGCCCGCAAGAAGGACTGGGATCCGAAGAAGTACGGCGGGTTCGACAGCCCCACTGTGGCCTACTCGGTCCTGGTTGTGGCGAAGGTTGAGAAGGGCAAGTCCAAGAAGCTCAAGAGCGTGAAGGAGCTGCTGGGGATCACGATTATGGAGCGCTCCAGCTTCGAGAAGAACCCGATCGATTTCCTGGAGGCGAAGGGCTACAAGGAGGTGAAGAAGGACCTGATCATTAAGCTCCCCAAGTACTCACTCTTCGAGCTGGAGAACGGCAGGAAGCGGATGCTGGCTTCCGCTGGCGAGCTGCAGAAGGGGAACGAGCTGGCTCTGCCGTCCAAGTATGTGAACTTCCTCTACCTGGCCTCCCACTACGAGAAGCTCAAGGGCAGCCCCGAGGACAACGAGCAGAAGCAGCTGTTCGTCGAGCAGCACAAGCATTACCTCGACGAGATCATTGAGCAGATTTCCGAGTTCTCCAAGCGCGTGATCCTGGCCGACGCGAATCTGGATAAGGTCCTCTCCGCGTACAACAAGCACCGCGACAAGCCAATCAGGGAGCAGGCTGAGAATATCATTCATCTCTTCACCCTGACGAACCTCGGCGCCCCTGCTGCTTTCAAGTACTTCGACACAACTATCGATCGCAAGAGGTACACAAGCACTAAGGAGGTCCTGGACGCGACCCTCATCCACCAGTCGATTACCGGCCTCTACGAGACGCGCATCGACCTGTCTCAGCTCGGGGGCGACGAATTCTCCGGGAGCGAGACGCCAGGCACCTCCGAGTCGGCCACCCCAGAATCTGCCACAGTGGTGTCCGGCCAAAAGCAGGACCGCCAGGGCGGAGAACGCAGAAGGTCCCAGCTCGATAGGGATCAGTGTGCCTACTGCAAGGAGAAGGGCCACTGGGCCAAAGACTGCCCGAAAAAGCCGCGCGGCCCACGCGGCCCAAGGCCACAAACATCCCTCCTTCCAAAGAAGAAGCGGAAGGTGGAGCTCAGCGGAGGATCTTCCGGAGGATCTAGCGGCTCCGAGACACCAGGAACATCCGAAAGCGCTACACCAGAATCTAGCGGAGGCTCTTCCGGAGGATCTAGGCCTCCTGAGGAAGACCGCGCTACATTGTGTCTTGTGCTTGGCCTGGAGGAGGAGTACCGCCTACATGAGAAGCCTGTGCCTTCCTCCGTCGACCCCTCTTGGCTCCAGCTGTTCCCGGACGTTTGGGCGGAGAAAGGCGGCATGGGGCTAGCCAACCGGGTCCCCCCTATCGTAGTGGAGCTAAAGAGTGATGCCCTGCCAGTCGCCGTAAGGCAGTACCCGATGTCGCGAGAAGCTAGAGAAGGTATCCGGCCGCACATTCAGCGCTTCCTGGACCTTGGTGTTCTAGTGCCATGCCAGAGTCCGTGGAACACACCACTGCTTCCTGTGAAGAAGCCCGGCACCTCTGACTACCGCCCCGTGCAGGATCTGAGAGAAATTAATAAGAGAGTTCAAGATATACACCCAACAGTGCCCAACCCATACAACTTGCTGTCCAGCCTGCCCCCAAATCATACGTGGTATAGCGTTCTAGATCTCAAAGACGCGTTCTTCTGCCTCAAACTGCATCCGAACTCCCAGCTCCTCTTCGCCTTTGAGTGGAGGGATCCGGAGAAAGGACACACCGGCCAACTAACATGGACACGGCTGCCGCAGGGCTTCAAGAACTCTCCTACTCTCTTCGATGAGGCACTCCACAGAGACCTGGCCTCCTTCCGCGCCAGCAACCCCCAGGTGGTGCTGCTGCAATATGTGGACGACCTGCTGGTGGCGGCCCCTACCTACAAGGACTGCAAGGAGGGAACTCAAAAACTACTCCAGGAGCTCTCGGAATTAGGTTACAGGGTCAGCGCGAAGAAGGCGCAGCTTTGTCAGAGAGAGGTAACGTACCTCGGATACCTGCTAAAGGAGGGCAAGCGGTGGCTGACCCCTGCTCGTAAGGCCACGGTGATGGAGATCCCCACCCCCACCACGCCTCGCCAGGTGCGTGAGTTCCTTGGTACTGCCGGTTTCTGTAGACTCTGGATTCCTGGTTTTGCTAGTCTGGCCGCCCCGCTGTATCCACTCACCAAGGAAAGCACTCCCTTCCTCTGGACAGAGGAACACCGCCGCGCATTCGACCAAATCAAGGAGGCCCTGCTCACGGCCCCTGCCCTCGCCCTCCCAGACCTGACTAAGCCGTTTGCCCTCTATGTGGATGAACGGGCCGGCGTGGCGAGAGGCGTGTTAACGCAGACACTGGGGCCTTGGCGGCGGCCTGTGGCCTACCTGTCAAAGAAGCTGGACCCCGTGGCTTCCGGCTGGCCCACGTGCCTCAAGGCAGTTGCTGCCGTCGCGCTGTTACTCAAGGATGCAGATAAGCTGACCCTCGGACAATCAGTGACAGTCATCGCTAGCCACAGCCTAGAGTCTATCGTGAGACAGCCGCCAGACAGGTGGATGACGAACGCCAGGATGACACACTACCAGTCGCTGCTGCTTAATGAACGTGTGAGCTTTGCCCCCCCGGCCGTACTGAACCCTGCCACCTTGCTGCCAGCAGAGTCTGGCGCGGCGCCTGTTCACGAATGCTCTGAAATTCTGGCTGAGGAGACCGGCACAAGACAGGACCTCACCGATCAACCTCTGCCGGGAGTGCCCGCGTGGTACACCGACGGCTCTAGCTTCATCACTGAAGGCAAAAGAAGGGCCGGCGCGGCCATTGTGGATGGAAAGCGGACGGTATGGATGTCCTCCCTGCCTGAAGGAACGAGCGCACAAAAGGCTGAACTGATCGCACTGACACAGGCTTTGCGCCTGGCCGACGGGAAGGATATCAATATTTACACTGATTCTAGATACGCGTTCGCCACGGCCCATATCCATGGCGCCATATATCGGCAGCGGGGTCTGCTTACCTCCGCCGGCAAGGAAATCAAAAATAAAGAAGAGATTCTCGCACTGCTGGAGGCTATCCACCTGCCTAAAAGAGTAGCCATCATTCACTGCCCTGGCCACCAGAAAGGCAATGATCCAGTGGCTATCGGAAACCGGCGGGCTGACGAGGCCGCCAAGCAGGCCGCGCTAGCCGTGAGGGTGCTCGCAGAGACTATCGAACCCCAGGGACAGCTGGGCTCGACCCAAGATAGAACGCGGCCAGGTGAATTAACCCCTGATCAGGGAAAGAATTTTATCAGGAGAGTGCATCAGCTGACGCACCTCGGCAGCGGCGGCAGCAAAAGAACGGCGGACGGCTCTGAGAAGCGCACCGCTGATAGCCAGCATTCAACTCCTCCGAAAACAAAGAGGAAAGTTGAGTTCGAACCGAAGAAGAAAAGGAAGGTGTGA

**Sequence 55 Plasmids sequence of ePPEplus-RT54**

(NLSSV40-nCas9(H840A/R221K/N394K)-XTEN-NC-NLS-32aa Linker-RT54-NLSvbp)

CCTAAGAAAAAGAGAAAAGTGGACAAGAAGTACTCGATCGGCCTCGATATTGGGACTAACTCTGTTGGCTGGGCCGTGATCACCGACGAGTACAAGGTGCCCTCAAAGAAGTTCAAGGTCCTGGGCAACACCGATCGGCATTCCATCAAGAAGAATCTCATTGGCGCTCTCCTGTTCGACAGCGGCGAGACGGCTGAGGCTACGCGGCTCAAGCGCACCGCCCGCAGGCGGTACACGCGCAGGAAGAATCGCATCTGCTACCTGCAGGAGATTTTCTCCAACGAGATGGCGAAGGTTGACGATTCTTTCTTCCACAGGCTGGAGGAGTCATTCCTCGTGGAGGAGGATAAGAAGCACGAGCGGCATCCAATCTTCGGCAACATTGTCGACGAGGTTGCCTACCACGAGAAGTACCCTACGATCTACCATCTGCGGAAGAAGCTCGTGGACTCCACAGATAAGGCGGACCTCCGCCTGATCTACCTCGCTCTGGCCCACATGATTAAGTTCAGGGGCCATTTCCTGATCGAGGGGGATCTCAACCCGGACAATAGCGATGTTGACAAGCTGTTCATCCAGCTCGTGCAGACGTACAACCAGCTCTTCGAGGAGAACCCCATTAATGCGTCAGGCGTCGACGCGAAGGCTATCCTGTCCGCTAGGCTCTCGAAGTCTCGGAAGCTCGAGAACCTGATCGCCCAGCTGCCGGGCGAGAAGAAGAACGGCCTGTTCGGGAATCTCATTGCGCTCAGCCTGGGGCTCACGCCCAACTTCAAGTCGAATTTCGATCTCGCTGAGGACGCCAAGCTGCAGCTCTCCAAGGACACATACGACGATGACCTGGATAACCTCCTGGCCCAGATCGGCGATCAGTACGCGGACCTGTTCCTCGCTGCCAAGAATCTGTCGGACGCCATCCTCCTGTCTGATATTCTCAGGGTGAACACCGAGATTACGAAGGCTCCGCTCTCAGCCTCCATGATCAAGCGCTACGACGAGCACCATCAGGATCTGACCCTCCTGAAGGCGCTGGTCAGGCAGCAGCTCCCCGAGAAGTACAAGGAGATCTTCTTCGATCAGTCGAAGAACGGCTACGCTGGGTACATTGACGGCGGGGCCTCTCAGGAGGAGTTCTACAAGTTCATCAAGCCGATTCTGGAGAAGATGGACGGCACGGAGGAGCTGCTGGTGAAGCTCAAGCGCGAGGACCTCCTGAGGAAGCAGCGGACATTCGATAACGGCAGCATCCCACACCAGATTCATCTCGGGGAGCTGCACGCTATCCTGAGGAGGCAGGAGGACTTCTACCCTTTCCTCAAGGATAACCGCGAGAAGATCGAGAAGATTCTGACTTTCAGGATCCCGTACTACGTCGGCCCACTCGCTAGGGGCAACTCCCGCTTCGCTTGGATGACCCGCAAGTCAGAGGAGACGATCACGCCGTGGAACTTCGAGGAGGTGGTCGACAAGGGCGCTAGCGCTCAGTCGTTCATCGAGAGGATGACGAATTTCGACAAGAACCTGCCAAATGAGAAGGTGCTCCCTAAGCACTCGCTCCTGTACGAGTACTTCACAGTCTACAACGAGCTGACTAAGGTGAAGTATGTGACCGAGGGCATGAGGAAGCCGGCTTTCCTGTCTGGGGAGCAGAAGAAGGCCATCGTGGACCTCCTGTTCAAGACCAACCGGAAGGTCACGGTTAAGCAGCTCAAGGAGGACTACTTCAAGAAGATTGAGTGCTTCGATTCGGTCGAGATCTCTGGCGTTGAGGACCGCTTCAACGCCTCCCTGGGGACCTACCACGATCTCCTGAAGATCATTAAGGATAAGGACTTCCTGGACAACGAGGAGAATGAGGATATCCTCGAGGACATTGTGCTGACACTCACTCTGTTCGAGGACCGGGAGATGATCGAGGAGCGCCTGAAGACTTACGCCCATCTCTTCGATGACAAGGTCATGAAGCAGCTCAAGAGGAGGAGGTACACCGGCTGGGGGAGGCTGAGCAGGAAGCTCATCAACGGCATTCGGGACAAGCAGTCCGGGAAGACGATCCTCGACTTCCTGAAGAGCGATGGCTTCGCGAACCGCAATTTCATGCAGCTGATTCACGATGACAGCCTCACATTCAAGGAGGATATCCAGAAGGCTCAGGTGAGCGGCCAGGGGGACTCGCTGCACGAGCATATCGCGAACCTCGCTGGCTCGCCAGCTATCAAGAAGGGGATTCTGCAGACCGTGAAGGTTGTGGACGAGCTGGTGAAGGTCATGGGCAGGCACAAGCCTGAGAACATCGTCATTGAGATGGCCCGGGAGAATCAGACCACGCAGAAGGGCCAGAAGAACTCACGCGAGAGGATGAAGAGGATCGAGGAGGGCATTAAGGAGCTGGGGTCCCAGATCCTCAAGGAGCACCCGGTGGAGAACACGCAGCTGCAGAATGAGAAGCTCTACCTGTACTACCTCCAGAATGGCCGCGATATGTATGTGGACCAGGAGCTGGATATTAACAGGCTCAGCGATTACGACGTCGATGCCATCGTTCCACAGTCATTCCTGAAGGATGACTCCATTGACAACAAGGTCCTCACCAGGTCGGACAAGAACCGGGGCAAGTCTGATAATGTTCCTTCAGAGGAGGTCGTTAAGAAGATGAAGAACTACTGGCGCCAGCTCCTGAATGCCAAGCTGATCACGCAGCGGAAGTTCGATAACCTCACAAAGGCTGAGAGGGGCGGGCTCTCTGAGCTGGACAAGGCGGGCTTCATCAAGAGGCAGCTGGTCGAGACACGGCAGATCACTAAGCACGTTGCGCAGATTCTCGACTCACGGATGAACACTAAGTACGATGAGAATGACAAGCTGATCCGCGAGGTGAAGGTCATCACCCTGAAGTCAAAGCTCGTCTCCGACTTCAGGAAGGATTTCCAGTTCTACAAGGTTCGGGAGATCAACAATTACCACCATGCCCATGACGCGTACCTGAACGCGGTGGTCGGCACAGCTCTGATCAAGAAGTACCCAAAGCTCGAGAGCGAGTTCGTGTACGGGGACTACAAGGTTTACGATGTGAGGAAGATGATCGCCAAGTCGGAGCAGGAGATTGGCAAGGCTACCGCCAAGTACTTCTTCTACTCTAACATTATGAATTTCTTCAAGACAGAGATCACTCTGGCCAATGGCGAGATCCGGAAGCGCCCCCTCATCGAGACGAACGGCGAGACGGGGGAGATCGTGTGGGACAAGGGCAGGGATTTCGCGACCGTCAGGAAGGTTCTCTCCATGCCACAAGTGAATATCGTCAAGAAGACAGAGGTCCAGACTGGCGGGTTCTCTAAGGAGTCAATTCTGCCTAAGCGGAACAGCGACAAGCTCATCGCCCGCAAGAAGGACTGGGATCCGAAGAAGTACGGCGGGTTCGACAGCCCCACTGTGGCCTACTCGGTCCTGGTTGTGGCGAAGGTTGAGAAGGGCAAGTCCAAGAAGCTCAAGAGCGTGAAGGAGCTGCTGGGGATCACGATTATGGAGCGCTCCAGCTTCGAGAAGAACCCGATCGATTTCCTGGAGGCGAAGGGCTACAAGGAGGTGAAGAAGGACCTGATCATTAAGCTCCCCAAGTACTCACTCTTCGAGCTGGAGAACGGCAGGAAGCGGATGCTGGCTTCCGCTGGCGAGCTGCAGAAGGGGAACGAGCTGGCTCTGCCGTCCAAGTATGTGAACTTCCTCTACCTGGCCTCCCACTACGAGAAGCTCAAGGGCAGCCCCGAGGACAACGAGCAGAAGCAGCTGTTCGTCGAGCAGCACAAGCATTACCTCGACGAGATCATTGAGCAGATTTCCGAGTTCTCCAAGCGCGTGATCCTGGCCGACGCGAATCTGGATAAGGTCCTCTCCGCGTACAACAAGCACCGCGACAAGCCAATCAGGGAGCAGGCTGAGAATATCATTCATCTCTTCACCCTGACGAACCTCGGCGCCCCTGCTGCTTTCAAGTACTTCGACACAACTATCGATCGCAAGAGGTACACAAGCACTAAGGAGGTCCTGGACGCGACCCTCATCCACCAGTCGATTACCGGCCTCTACGAGACGCGCATCGACCTGTCTCAGCTCGGGGGCGACGAATTCTCCGGGAGCGAGACGCCAGGCACCTCCGAGTCGGCCACCCCAGAATCTGCCACAGTGGTGTCCGGCCAAAAGCAGGACCGCCAGGGCGGAGAACGCAGAAGGTCCCAGCTCGATAGGGATCAGTGTGCCTACTGCAAGGAGAAGGGCCACTGGGCCAAAGACTGCCCGAAAAAGCCGCGCGGCCCACGCGGCCCAAGGCCACAAACATCCCTCCTTCCAAAGAAGAAGCGGAAGGTGGAGCTCAGCGGAGGATCTTCCGGAGGATCTAGCGGCTCCGAGACACCAGGAACATCCGAAAGCGCTACACCAGAATCTAGCGGAGGCTCTTCCGGAGGATCTAGGCCTCCTCGGCCGGCGATGTGCTTGGTGCTCAACCTCGAGGAAGAATACAGGCTCCATGAGAAACCAGTGCCCCCCAGCATAGATCCTTCGTGGCTTCAGCTGTTCCCGATGGTGTGGGCGGAGAAGGCTGGGATGGGGCTGGCCAACCAGGTTCCTCCTGTGGTGGTCGAACTCAAGTCAGACGCCAGCCCGGTTGCCGTGAGGCAGTACCCGATGTCAAAGGAGGCAAGAGAGGGTATTCGCCCTCACATCCAGAGATTTCTGGATTTAGGCATCTTGGTCCCATGTCAGAGCCCATGGAACACCCCTCTTCTTCCCGTGAAAAAGCCCGGCACAAATGATTACAGACCAGTACAAGATCTTAGAGAAGTCAATAAGCGTGTGCAGGACATTCACCCGACAGTGCCTAACCCATACAATTTGCTGTCGAGCCTACCGCCTAGCCACACCTGGTATTCAGTGCTAGATTTAAAGGACGCATTCTTCTGTCTGAAGCTCCATCCTAACTCTCAACCCCTTTTCGCCTTCGAATGGAGAGATCCTGAAAAGGGCAACACTGGGCAGCTCACATGGACGAGACTGCCACAGGGATTCAAGAATAGCCCTACCCTTTTTGATGAGGCTCTGCACAGGGACCTAGCCAGCTTCCGCGCCCTGAACCCTCAGGTCGTGATGCTTCAATATGTTGACGACCTACTGGTCGCGGCTCCAACATATCGCGACTGCAAAGAGGGCACACGTAGGCTGTTACAGGAGCTGTCTAAGCTGGGCTACCGCGTGAGCGCTAAAAAGGCTCAGCTTTGCCGGGAGGAGGTAACTTATCTGGGCTACTTGCTGAAAGGTGGCAAAAGATGGCTCACACCAGCTCGGAAGGCGACGGTCATGAAAATCCCTACCCCCACCACTCCTCGTCAGGTTCGGGAGTTCCTCGGCACGGCGGGCTTCTGCAGGTTATGGATCCCTGGATTCGCCAGCTTAGCTGCCCCTCTCTACCCGCTGACCAGAGAGAAGGTGCCTTTCACATGGACAGAGGCCCACCAAGAGGCATTCGGCAGGATAAAGGAGGCGCTGTTGAGCGCTCCGGCCTTAGCTCTGCCGGACCTGACTAAGCCGTTCGCCCTGTACGTTGATGAAAAGGAAGGAGTGGCGCGCGGGGTGCTGACACAGACTCTCGGCCCGTGGAGACGCCCTGTCGCGTACCTTAGCAAAAAACTAGACCCGGTGGCTTCTGGGTGGCCGACTTGCCTCAAAGCAATCGCCGCCGTCGCCCTCCTGCTGAAGGACGCCGACAAGTTAACTCTGGGTCAGAATGTACTCGTGATCGCACCACACAACCTGGAGTCCATCGTGAGACAGCCGCCTGATCGGTGGATGACCAACGCCAGGATGACGCACTACCAGAGTCTGCTGCTGAATGAACGCGTCTCTTTTGCTCCTCCTGCAATTTTGAACCCTGCCACTCTACTGCCCGTGGAGTCAGATGACACCCCTATACATATTTGCTCTGAAATCCTGGCCGAGGAGACCGGCACGCGACCTGATCTGCGAGACCAACCACTTCCTGGAGTGCCGGCGTGGTACACCGACGGCAGCTCTTTCATCATGGATGGGAGACGCCAGGCCGGCGCGGCCATCGTCGACAACAAGCGCACCGTCTGGGCATCCAACCTGCCCGAGGGAACATCGGCCCAGAAGGCCGAGCTCATCGCCCTGACCCAGGCTCTGAGGCTAGCTGAAGGGAAGTCTATCAATATCTACACAGACAGCCGGTATGCATTTGCTACCGCGCACGTCCATGGGGCCATTTACAAGCAAAGAGGACTGTTGACATCTGCCGGTAAAGATATCAAAAATAAGGAAGAAATACTCGCTCTGCTAGAGGCCATCCATTTGCCAAAGAGAGTCGCCATTATCCACTGTCCTGGCCATCAGCGCGGCACAGATCCAGTGGCCACCGGAAACAGGAAGGCTGACGAAGCAGCCAAGCAGGCTGCGCAGTCCACCCGGATCCTCACCGAAACAACAAAGAACCAAGAACACTTTGAACCCACTCGGGGGAAGATCAAGCCGCGGGAGCTGACCCCTGACCAGGGCCGGGAGTTTATTCAGCGGTTACACCAGTTAACCCACCTCGGCAGCGGCGGCAGCAAAAGAACGGCGGACGGCTCTGAGAAGCGCACCGCTGATAGCCAGCATTCAACTCCTCCGAAAACAAAGAGGAAAGTTGAGTTCGAACCGAAGAAGAAAAGGAAGGTGTGA

**Sequence 56 Plasmids sequence of PE-RERV-m5(ePPEplus-RERV-m5)**

(NLSSV40-nCas9(H840A/R221K/N394K)-XTEN-NC-NLS-32aa Linker-RERV-RT-m5(D204N/V227A/T310K/W317F/E334P)-NLSvbp)

CCTAAGAAAAAGAGAAAAGTGGACAAGAAGTACTCGATCGGCCTCGATATTGGGACTAACTCTGTTGGCTGGGCCGTGATCACCGACGAGTACAAGGTGCCCTCAAAGAAGTTCAAGGTCCTGGGCAACACCGATCGGCATTCCATCAAGAAGAATCTCATTGGCGCTCTCCTGTTCGACAGCGGCGAGACGGCTGAGGCTACGCGGCTCAAGCGCACCGCCCGCAGGCGGTACACGCGCAGGAAGAATCGCATCTGCTACCTGCAGGAGATTTTCTCCAACGAGATGGCGAAGGTTGACGATTCTTTCTTCCACAGGCTGGAGGAGTCATTCCTCGTGGAGGAGGATAAGAAGCACGAGCGGCATCCAATCTTCGGCAACATTGTCGACGAGGTTGCCTACCACGAGAAGTACCCTACGATCTACCATCTGCGGAAGAAGCTCGTGGACTCCACAGATAAGGCGGACCTCCGCCTGATCTACCTCGCTCTGGCCCACATGATTAAGTTCAGGGGCCATTTCCTGATCGAGGGGGATCTCAACCCGGACAATAGCGATGTTGACAAGCTGTTCATCCAGCTCGTGCAGACGTACAACCAGCTCTTCGAGGAGAACCCCATTAATGCGTCAGGCGTCGACGCGAAGGCTATCCTGTCCGCTAGGCTCTCGAAGTCTCGGAAGCTCGAGAACCTGATCGCCCAGCTGCCGGGCGAGAAGAAGAACGGCCTGTTCGGGAATCTCATTGCGCTCAGCCTGGGGCTCACGCCCAACTTCAAGTCGAATTTCGATCTCGCTGAGGACGCCAAGCTGCAGCTCTCCAAGGACACATACGACGATGACCTGGATAACCTCCTGGCCCAGATCGGCGATCAGTACGCGGACCTGTTCCTCGCTGCCAAGAATCTGTCGGACGCCATCCTCCTGTCTGATATTCTCAGGGTGAACACCGAGATTACGAAGGCTCCGCTCTCAGCCTCCATGATCAAGCGCTACGACGAGCACCATCAGGATCTGACCCTCCTGAAGGCGCTGGTCAGGCAGCAGCTCCCCGAGAAGTACAAGGAGATCTTCTTCGATCAGTCGAAGAACGGCTACGCTGGGTACATTGACGGCGGGGCCTCTCAGGAGGAGTTCTACAAGTTCATCAAGCCGATTCTGGAGAAGATGGACGGCACGGAGGAGCTGCTGGTGAAGCTCAAGCGCGAGGACCTCCTGAGGAAGCAGCGGACATTCGATAACGGCAGCATCCCACACCAGATTCATCTCGGGGAGCTGCACGCTATCCTGAGGAGGCAGGAGGACTTCTACCCTTTCCTCAAGGATAACCGCGAGAAGATCGAGAAGATTCTGACTTTCAGGATCCCGTACTACGTCGGCCCACTCGCTAGGGGCAACTCCCGCTTCGCTTGGATGACCCGCAAGTCAGAGGAGACGATCACGCCGTGGAACTTCGAGGAGGTGGTCGACAAGGGCGCTAGCGCTCAGTCGTTCATCGAGAGGATGACGAATTTCGACAAGAACCTGCCAAATGAGAAGGTGCTCCCTAAGCACTCGCTCCTGTACGAGTACTTCACAGTCTACAACGAGCTGACTAAGGTGAAGTATGTGACCGAGGGCATGAGGAAGCCGGCTTTCCTGTCTGGGGAGCAGAAGAAGGCCATCGTGGACCTCCTGTTCAAGACCAACCGGAAGGTCACGGTTAAGCAGCTCAAGGAGGACTACTTCAAGAAGATTGAGTGCTTCGATTCGGTCGAGATCTCTGGCGTTGAGGACCGCTTCAACGCCTCCCTGGGGACCTACCACGATCTCCTGAAGATCATTAAGGATAAGGACTTCCTGGACAACGAGGAGAATGAGGATATCCTCGAGGACATTGTGCTGACACTCACTCTGTTCGAGGACCGGGAGATGATCGAGGAGCGCCTGAAGACTTACGCCCATCTCTTCGATGACAAGGTCATGAAGCAGCTCAAGAGGAGGAGGTACACCGGCTGGGGGAGGCTGAGCAGGAAGCTCATCAACGGCATTCGGGACAAGCAGTCCGGGAAGACGATCCTCGACTTCCTGAAGAGCGATGGCTTCGCGAACCGCAATTTCATGCAGCTGATTCACGATGACAGCCTCACATTCAAGGAGGATATCCAGAAGGCTCAGGTGAGCGGCCAGGGGGACTCGCTGCACGAGCATATCGCGAACCTCGCTGGCTCGCCAGCTATCAAGAAGGGGATTCTGCAGACCGTGAAGGTTGTGGACGAGCTGGTGAAGGTCATGGGCAGGCACAAGCCTGAGAACATCGTCATTGAGATGGCCCGGGAGAATCAGACCACGCAGAAGGGCCAGAAGAACTCACGCGAGAGGATGAAGAGGATCGAGGAGGGCATTAAGGAGCTGGGGTCCCAGATCCTCAAGGAGCACCCGGTGGAGAACACGCAGCTGCAGAATGAGAAGCTCTACCTGTACTACCTCCAGAATGGCCGCGATATGTATGTGGACCAGGAGCTGGATATTAACAGGCTCAGCGATTACGACGTCGATGCCATCGTTCCACAGTCATTCCTGAAGGATGACTCCATTGACAACAAGGTCCTCACCAGGTCGGACAAGAACCGGGGCAAGTCTGATAATGTTCCTTCAGAGGAGGTCGTTAAGAAGATGAAGAACTACTGGCGCCAGCTCCTGAATGCCAAGCTGATCACGCAGCGGAAGTTCGATAACCTCACAAAGGCTGAGAGGGGCGGGCTCTCTGAGCTGGACAAGGCGGGCTTCATCAAGAGGCAGCTGGTCGAGACACGGCAGATCACTAAGCACGTTGCGCAGATTCTCGACTCACGGATGAACACTAAGTACGATGAGAATGACAAGCTGATCCGCGAGGTGAAGGTCATCACCCTGAAGTCAAAGCTCGTCTCCGACTTCAGGAAGGATTTCCAGTTCTACAAGGTTCGGGAGATCAACAATTACCACCATGCCCATGACGCGTACCTGAACGCGGTGGTCGGCACAGCTCTGATCAAGAAGTACCCAAAGCTCGAGAGCGAGTTCGTGTACGGGGACTACAAGGTTTACGATGTGAGGAAGATGATCGCCAAGTCGGAGCAGGAGATTGGCAAGGCTACCGCCAAGTACTTCTTCTACTCTAACATTATGAATTTCTTCAAGACAGAGATCACTCTGGCCAATGGCGAGATCCGGAAGCGCCCCCTCATCGAGACGAACGGCGAGACGGGGGAGATCGTGTGGGACAAGGGCAGGGATTTCGCGACCGTCAGGAAGGTTCTCTCCATGCCACAAGTGAATATCGTCAAGAAGACAGAGGTCCAGACTGGCGGGTTCTCTAAGGAGTCAATTCTGCCTAAGCGGAACAGCGACAAGCTCATCGCCCGCAAGAAGGACTGGGATCCGAAGAAGTACGGCGGGTTCGACAGCCCCACTGTGGCCTACTCGGTCCTGGTTGTGGCGAAGGTTGAGAAGGGCAAGTCCAAGAAGCTCAAGAGCGTGAAGGAGCTGCTGGGGATCACGATTATGGAGCGCTCCAGCTTCGAGAAGAACCCGATCGATTTCCTGGAGGCGAAGGGCTACAAGGAGGTGAAGAAGGACCTGATCATTAAGCTCCCCAAGTACTCACTCTTCGAGCTGGAGAACGGCAGGAAGCGGATGCTGGCTTCCGCTGGCGAGCTGCAGAAGGGGAACGAGCTGGCTCTGCCGTCCAAGTATGTGAACTTCCTCTACCTGGCCTCCCACTACGAGAAGCTCAAGGGCAGCCCCGAGGACAACGAGCAGAAGCAGCTGTTCGTCGAGCAGCACAAGCATTACCTCGACGAGATCATTGAGCAGATTTCCGAGTTCTCCAAGCGCGTGATCCTGGCCGACGCGAATCTGGATAAGGTCCTCTCCGCGTACAACAAGCACCGCGACAAGCCAATCAGGGAGCAGGCTGAGAATATCATTCATCTCTTCACCCTGACGAACCTCGGCGCCCCTGCTGCTTTCAAGTACTTCGACACAACTATCGATCGCAAGAGGTACACAAGCACTAAGGAGGTCCTGGACGCGACCCTCATCCACCAGTCGATTACCGGCCTCTACGAGACGCGCATCGACCTGTCTCAGCTCGGGGGCGACGAATTCTCCGGGAGCGAGACGCCAGGCACCTCCGAGTCGGCCACCCCAGAATCTGCCACAGTGGTGTCCGGCCAAAAGCAGGACCGCCAGGGCGGAGAACGCAGAAGGTCCCAGCTCGATAGGGATCAGTGTGCCTACTGCAAGGAGAAGGGCCACTGGGCCAAAGACTGCCCGAAAAAGCCGCGCGGCCCACGCGGCCCAAGGCCACAAACATCCCTCCTTCCAAAGAAGAAGCGGAAGGTGGAGCTCAGCGGAGGATCTTCCGGAGGATCTAGCGGCTCCGAGACACCAGGAACATCCGAAAGCGCTACACCAGAATCTAGCGGAGGCTCTTCCGGAGGATCTAGGCCTCCCGTCGCGTGCCTCGTACTGAGCCTAGAGGAGGAGTACAGGCTGCATGAGCAAAATCCGAAGCAGCTGCTAGCCCCTGAATGGCTGAGCGCCTTCTCCGAGGTATGGGCGGAGCAGACTGGCATGGGACTGGCCAAGCAGGTGCCTCCTGTGGTGGTAGAGCTGAAGGCGGACGCCAGCCCAGTGAGCGTGAAACAATACCCGATGTCGAGGGAAGCAAAGGAAGGCATTAGACCCCACATCCAGAGACTGCTGCAATTGGGAATCCTGGTACCCTGCCAAAGCCCTTGGAACACGCCACTGCTGCCTGTGCGCAAACCAGGAACTAATGATTACAGACCTGTACAGGATCTAAGGGAAGTTAACAAGCGGGTGCAGGACATACATCCTACGGTCCCAAACCCCTACAACTTGCTGAGCTCTCTGCCCCCCGAGCGCACATGGTACACTGTGCTCGACTTAAAAGATGCGTTCTTCTGCCTTCGCCTCCACCCGAACTCACAGCCACTGTTCGCGTTCGAGTGGCGCGACCCTGAGGGCGGCCACACCGGGCAACTGACGTGGACACGGCTGCCACAGGGTTTCAAGAACAGCCCTACTCTATTTGATGAGGCCCTGCACCGTGATCTGGCCCCCTTCCGGGCGCAGAACCCTCAAATCAGTCTACTGCAGTATGTTGATGACCTGCTGCTAGCCGCTTCTACTCAGGAGCTCTGCCGGGAGGGCACAAAGCGGCTGTTAAATGAGCTGGGCGAGCTGGGATACCGCGTGTCTGCCAAGAAGGCACAATTGTGTCGGACAGAGGTGACATATCTCGGCTACACATTAAGAGAAGGTAAGAGATGGCTCACTGAAGCACGTAAGAAGACTGTCATGCAGATTCCTACCCCTACCACGCCCCGTCAGGTGCGGGAGTTCCTCGGCACCGCCGGCTTCTGTAGACTGTGGATTCCTGGATTTGCCACCCTGGCCGCCCCACTCTACCTGCTGACGAAGGAAAAGGTTCCTTTCACCTGGACTGAGGAACATCAGCGGGCTTTCGAAGACATCAAAGCCGCCCTGCTGGCGGCTCCAGCTCTGGCCCTCCCTGACCTCACCAAGCCCTTCACACTCTATGTGGACGAGAGAGCTGGAGTGGCCAGAGGTGTGCTGACACAGGCACTGGGGCCGTGGAAGAGACCCGTGGCGTACCTATCTAAAAAGCTCGACCCCGTGGCCTCTGGGTGGCCGAGCTGTCTAAAGGCCATCGCTGCTGTAGCTCTACTGGTCAAAGATGCTGACAAGCTGACCCTGGGCCAGCATGTCACCGTCATCGCCCCGCACGCCTTAGAATCCATAGTCCGGCAACCACCAGATCGGTGGATGACAAACGCTAGGATGACGCATTATCAGAGCTTGCTGTTAAACGAAAGAGTGACATTTGCGCCCCCTGCCATCCTTAACCCAGCGACGCTACTGCCGGAGATCCACAACTCAACCCCCATCCACCAGTGCGTCGACATTCTGGCTGAGGAGACCGGCACGCGCAAGGACCTCACGGACAGACCATGGCCTGGTGTGCCGGCATGGTACACCGACGGCAGCTCCTTTGTCGTGGAGGGAAAGCGACGCGCAGGCGCGGCTGTGGTGGACGGCAAGCAAGTCATCTGGGCATCCTCCCTGCCAGAAGGCACCAGCGCTCAGAAAGCGGAGTTGGTGGCCTTGACCCAGGCCCTCAGATTAGCTGAAGGGAAGGCTATTAATATCTACACCGATTCTAGATACGCCTTCGCCACGGCGCACATCCACGGCGCGATCTACAAGCAGCGGGGCCTGCTGACCAGTGCCGGGCGAGACATCAAGAATAAGGAAGAGATTCTTGCCCTGCTGGAGGCCGTCCACCTCCCCAAAAAGGTGGCCATTATCCACTGCCCCGGCCACCAGAAGGGAGAGGATCCAATTACAAAGGGGAACCAGATGGCCGACCTCGTGGCGAAGCAGGTCGCTCAGCAGGTTACCATCCTGGCAGAAAAATCGCAGACCCCTGTGAAGACACCTGTGACAGATGATTCTAACTATAGCGGCGGCAGCAAAAGAACGGCGGACGGCTCTGAGAAGCGCACCGCTGATAGCCAGCATTCAACTCCTCCGAAAACAAAGAGGAAAGTTGAGTTCGAACCGAAGAAGAAAAGGAAGGTGTGA

**Sequence 57 Plasmids sequence of PE-enRERV (ePPEplus-enRERV)**

(NLSSV40-nCas9(H840A/R221K/N394K)-XTEN-NC-NLS-32aa Linker-enRERV-RT(Y68R/W149F/D204N/V227A/T310K/W317F/E334P/C413R)-NLSvbp)

CCTAAGAAAAAGAGAAAAGTGGACAAGAAGTACTCGATCGGCCTCGATATTGGGACTAACTCTGTTGGCTGGGCCGTGATCACCGACGAGTACAAGGTGCCCTCAAAGAAGTTCAAGGTCCTGGGCAACACCGATCGGCATTCCATCAAGAAGAATCTCATTGGCGCTCTCCTGTTCGACAGCGGCGAGACGGCTGAGGCTACGCGGCTCAAGCGCACCGCCCGCAGGCGGTACACGCGCAGGAAGAATCGCATCTGCTACCTGCAGGAGATTTTCTCCAACGAGATGGCGAAGGTTGACGATTCTTTCTTCCACAGGCTGGAGGAGTCATTCCTCGTGGAGGAGGATAAGAAGCACGAGCGGCATCCAATCTTCGGCAACATTGTCGACGAGGTTGCCTACCACGAGAAGTACCCTACGATCTACCATCTGCGGAAGAAGCTCGTGGACTCCACAGATAAGGCGGACCTCCGCCTGATCTACCTCGCTCTGGCCCACATGATTAAGTTCAGGGGCCATTTCCTGATCGAGGGGGATCTCAACCCGGACAATAGCGATGTTGACAAGCTGTTCATCCAGCTCGTGCAGACGTACAACCAGCTCTTCGAGGAGAACCCCATTAATGCGTCAGGCGTCGACGCGAAGGCTATCCTGTCCGCTAGGCTCTCGAAGTCTCGGAAGCTCGAGAACCTGATCGCCCAGCTGCCGGGCGAGAAGAAGAACGGCCTGTTCGGGAATCTCATTGCGCTCAGCCTGGGGCTCACGCCCAACTTCAAGTCGAATTTCGATCTCGCTGAGGACGCCAAGCTGCAGCTCTCCAAGGACACATACGACGATGACCTGGATAACCTCCTGGCCCAGATCGGCGATCAGTACGCGGACCTGTTCCTCGCTGCCAAGAATCTGTCGGACGCCATCCTCCTGTCTGATATTCTCAGGGTGAACACCGAGATTACGAAGGCTCCGCTCTCAGCCTCCATGATCAAGCGCTACGACGAGCACCATCAGGATCTGACCCTCCTGAAGGCGCTGGTCAGGCAGCAGCTCCCCGAGAAGTACAAGGAGATCTTCTTCGATCAGTCGAAGAACGGCTACGCTGGGTACATTGACGGCGGGGCCTCTCAGGAGGAGTTCTACAAGTTCATCAAGCCGATTCTGGAGAAGATGGACGGCACGGAGGAGCTGCTGGTGAAGCTCAAGCGCGAGGACCTCCTGAGGAAGCAGCGGACATTCGATAACGGCAGCATCCCACACCAGATTCATCTCGGGGAGCTGCACGCTATCCTGAGGAGGCAGGAGGACTTCTACCCTTTCCTCAAGGATAACCGCGAGAAGATCGAGAAGATTCTGACTTTCAGGATCCCGTACTACGTCGGCCCACTCGCTAGGGGCAACTCCCGCTTCGCTTGGATGACCCGCAAGTCAGAGGAGACGATCACGCCGTGGAACTTCGAGGAGGTGGTCGACAAGGGCGCTAGCGCTCAGTCGTTCATCGAGAGGATGACGAATTTCGACAAGAACCTGCCAAATGAGAAGGTGCTCCCTAAGCACTCGCTCCTGTACGAGTACTTCACAGTCTACAACGAGCTGACTAAGGTGAAGTATGTGACCGAGGGCATGAGGAAGCCGGCTTTCCTGTCTGGGGAGCAGAAGAAGGCCATCGTGGACCTCCTGTTCAAGACCAACCGGAAGGTCACGGTTAAGCAGCTCAAGGAGGACTACTTCAAGAAGATTGAGTGCTTCGATTCGGTCGAGATCTCTGGCGTTGAGGACCGCTTCAACGCCTCCCTGGGGACCTACCACGATCTCCTGAAGATCATTAAGGATAAGGACTTCCTGGACAACGAGGAGAATGAGGATATCCTCGAGGACATTGTGCTGACACTCACTCTGTTCGAGGACCGGGAGATGATCGAGGAGCGCCTGAAGACTTACGCCCATCTCTTCGATGACAAGGTCATGAAGCAGCTCAAGAGGAGGAGGTACACCGGCTGGGGGAGGCTGAGCAGGAAGCTCATCAACGGCATTCGGGACAAGCAGTCCGGGAAGACGATCCTCGACTTCCTGAAGAGCGATGGCTTCGCGAACCGCAATTTCATGCAGCTGATTCACGATGACAGCCTCACATTCAAGGAGGATATCCAGAAGGCTCAGGTGAGCGGCCAGGGGGACTCGCTGCACGAGCATATCGCGAACCTCGCTGGCTCGCCAGCTATCAAGAAGGGGATTCTGCAGACCGTGAAGGTTGTGGACGAGCTGGTGAAGGTCATGGGCAGGCACAAGCCTGAGAACATCGTCATTGAGATGGCCCGGGAGAATCAGACCACGCAGAAGGGCCAGAAGAACTCACGCGAGAGGATGAAGAGGATCGAGGAGGGCATTAAGGAGCTGGGGTCCCAGATCCTCAAGGAGCACCCGGTGGAGAACACGCAGCTGCAGAATGAGAAGCTCTACCTGTACTACCTCCAGAATGGCCGCGATATGTATGTGGACCAGGAGCTGGATATTAACAGGCTCAGCGATTACGACGTCGATGCCATCGTTCCACAGTCATTCCTGAAGGATGACTCCATTGACAACAAGGTCCTCACCAGGTCGGACAAGAACCGGGGCAAGTCTGATAATGTTCCTTCAGAGGAGGTCGTTAAGAAGATGAAGAACTACTGGCGCCAGCTCCTGAATGCCAAGCTGATCACGCAGCGGAAGTTCGATAACCTCACAAAGGCTGAGAGGGGCGGGCTCTCTGAGCTGGACAAGGCGGGCTTCATCAAGAGGCAGCTGGTCGAGACACGGCAGATCACTAAGCACGTTGCGCAGATTCTCGACTCACGGATGAACACTAAGTACGATGAGAATGACAAGCTGATCCGCGAGGTGAAGGTCATCACCCTGAAGTCAAAGCTCGTCTCCGACTTCAGGAAGGATTTCCAGTTCTACAAGGTTCGGGAGATCAACAATTACCACCATGCCCATGACGCGTACCTGAACGCGGTGGTCGGCACAGCTCTGATCAAGAAGTACCCAAAGCTCGAGAGCGAGTTCGTGTACGGGGACTACAAGGTTTACGATGTGAGGAAGATGATCGCCAAGTCGGAGCAGGAGATTGGCAAGGCTACCGCCAAGTACTTCTTCTACTCTAACATTATGAATTTCTTCAAGACAGAGATCACTCTGGCCAATGGCGAGATCCGGAAGCGCCCCCTCATCGAGACGAACGGCGAGACGGGGGAGATCGTGTGGGACAAGGGCAGGGATTTCGCGACCGTCAGGAAGGTTCTCTCCATGCCACAAGTGAATATCGTCAAGAAGACAGAGGTCCAGACTGGCGGGTTCTCTAAGGAGTCAATTCTGCCTAAGCGGAACAGCGACAAGCTCATCGCCCGCAAGAAGGACTGGGATCCGAAGAAGTACGGCGGGTTCGACAGCCCCACTGTGGCCTACTCGGTCCTGGTTGTGGCGAAGGTTGAGAAGGGCAAGTCCAAGAAGCTCAAGAGCGTGAAGGAGCTGCTGGGGATCACGATTATGGAGCGCTCCAGCTTCGAGAAGAACCCGATCGATTTCCTGGAGGCGAAGGGCTACAAGGAGGTGAAGAAGGACCTGATCATTAAGCTCCCCAAGTACTCACTCTTCGAGCTGGAGAACGGCAGGAAGCGGATGCTGGCTTCCGCTGGCGAGCTGCAGAAGGGGAACGAGCTGGCTCTGCCGTCCAAGTATGTGAACTTCCTCTACCTGGCCTCCCACTACGAGAAGCTCAAGGGCAGCCCCGAGGACAACGAGCAGAAGCAGCTGTTCGTCGAGCAGCACAAGCATTACCTCGACGAGATCATTGAGCAGATTTCCGAGTTCTCCAAGCGCGTGATCCTGGCCGACGCGAATCTGGATAAGGTCCTCTCCGCGTACAACAAGCACCGCGACAAGCCAATCAGGGAGCAGGCTGAGAATATCATTCATCTCTTCACCCTGACGAACCTCGGCGCCCCTGCTGCTTTCAAGTACTTCGACACAACTATCGATCGCAAGAGGTACACAAGCACTAAGGAGGTCCTGGACGCGACCCTCATCCACCAGTCGATTACCGGCCTCTACGAGACGCGCATCGACCTGTCTCAGCTCGGGGGCGACGAATTCTCCGGGAGCGAGACGCCAGGCACCTCCGAGTCGGCCACCCCAGAATCTGCCACAGTGGTGTCCGGCCAAAAGCAGGACCGCCAGGGCGGAGAACGCAGAAGGTCCCAGCTCGATAGGGATCAGTGTGCCTACTGCAAGGAGAAGGGCCACTGGGCCAAAGACTGCCCGAAAAAGCCGCGCGGCCCACGCGGCCCAAGGCCACAAACATCCCTCCTTCCAAAGAAGAAGCGGAAGGTGGAGCTCAGCGGAGGATCTTCCGGAGGATCTAGCGGCTCCGAGACACCAGGAACATCCGAAAGCGCTACACCAGAATCTAGCGGAGGCTCTTCCGGAGGATCTAGGCCTCCCGTCGCGTGCCTCGTACTGAGCCTAGAGGAGGAGTACAGGCTGCATGAGCAAAATCCGAAGCAGCTGCTAGCCCCTGAATGGCTGAGCGCCTTCTCCGAGGTATGGGCGGAGCAGACTGGCATGGGACTGGCCAAGCAGGTGCCTCCTGTGGTGGTAGAGCTGAAGGCGGACGCCAGCCCAGTGAGCGTGAAACAAcgcCCGATGTCGAGGGAAGCAAAGGAAGGCATTAGACCCCACATCCAGAGACTGCTGCAATTGGGAATCCTGGTACCCTGCCAAAGCCCTTGGAACACGCCACTGCTGCCTGTGCGCAAACCAGGAACTAATGATTACAGACCTGTACAGGATCTAAGGGAAGTTAACAAGCGGGTGCAGGACATACATCCTACGGTCCCAAACCCCTACAACTTGCTGAGCTCTCTGCCCCCCGAGCGCACAttcTACACTGTGCTCGACTTAAAAGATGCGTTCTTCTGCCTTCGCCTCCACCCGAACTCACAGCCACTGTTCGCGTTCGAGTGGCGCGACCCTGAGGGCGGCCACACCGGGCAACTGACGTGGACACGGCTGCCACAGGGTTTCAAGAACAGCCCTACTCTATTTAACGAGGCCCTGCACCGTGATCTGGCCCCCTTCCGGGCGCAGAACCCTCAAATCAGTCTACTGCAGTATGCGGATGACCTGCTGCTAGCCGCTTCTACTCAGGAGCTCTGCCGGGAGGGCACAAAGCGGCTGTTAAATGAGCTGGGCGAGCTGGGATACCGCGTGTCTGCCAAGAAGGCACAATTGTGTCGGACAGAGGTGACATATCTCGGCTACACATTAAGAGAAGGTAAGAGATGGCTCACTGAAGCACGTAAGAAGACTGTCATGCAGATTCCTACCCCTACCACGCCCCGTCAGGTGCGGGAGTTCCTCGGCAAGGCCGGCTTCTGTAGACTGTTCATTCCTGGATTTGCCACCCTGGCCGCCCCACTCTACCTGCTGACGAAGCCAAAGGTTCCTTTCACCTGGACTGAGGAACATCAGCGGGCTTTCGAAGACATCAAAGCCGCCCTGCTGGCGGCTCCAGCTCTGGCCCTCCCTGACCTCACCAAGCCCTTCACACTCTATGTGGACGAGAGAGCTGGAGTGGCCAGAGGTGTGCTGACACAGGCACTGGGGCCGTGGAAGAGACCCGTGGCGTACCTATCTAAAAAGCTCGACCCCGTGGCCTCTGGGTGGCCGAGCcgcCTAAAGGCCATCGCTGCTGTAGCTCTACTGGTCAAAGATGCTGACAAGCTGACCCTGGGCCAGCATGTCACCGTCATCGCCCCGCACGCCTTAGAATCCATAGTCCGGCAACCACCAGATCGGTGGATGACAAACGCTAGGATGACGCATTATCAGAGCTTGCTGTTAAACGAAAGAGTGACATTTGCGCCCCCTGCCATCCTTAACCCAGCGACGCTACTGCCGGAGATCCACAACTCAACCCCCATCCACCAGTGCGTCGACATTCTGGCTGAGGAGACCGGCACGCGCAAGGACCTCACGGACAGACCATGGCCTGGTGTGCCGGCATGGTACACCGACGGCAGCTCCTTTGTCGTGGAGGGAAAGCGACGCGCAGGCGCGGCTGTGGTGGACGGCAAGCAAGTCATCTGGGCATCCTCCCTGCCAGAAGGCACCAGCGCTCAGAAAGCGGAGTTGGTGGCCTTGACCCAGGCCCTCAGATTAGCTGAAGGGAAGGCTATTAATATCTACACCGATTCTAGATACGCCTTCGCCACGGCGCACATCCACGGCGCGATCTACAAGCAGCGGGGCCTGCTGACCAGTGCCGGGCGAGACATCAAGAATAAGGAAGAGATTCTTGCCCTGCTGGAGGCCGTCCACCTCCCCAAAAAGGTGGCCATTATCCACTGCCCCGGCCACCAGAAGGGAGAGGATCCAATTACAAAGGGGAACCAGATGGCCGACCTCGTGGCGAAGCAGGTCGCTCAGCAGGTTACCATCCTGGCAGAAAAATCGCAGACCCCTGTGAAGACACCTGTGACAGATGATTCTAACTATAGCGGCGGCAGCAAAAGAACGGCGGACGGCTCTGAGAAGCGCACCGCTGATAGCCAGCATTCAACTCCTCCGAAAACAAAGAGGAAAGTTGAGTTCGAACCGAAGAAGAAAAGGAAGGTGTGA

**Sequence 58 Plasmids sequence of PE-M-MLV (PEmax)**

(NLSSV40-nCas9(H840A/R221K/N394K)-NLSSV40-M-MLV-RT-NLSSV40-NLSc-myc)

CCAAAGAAGAAGCGGAAAGTCGACAAGAAGTACAGCATCGGCCTGGACATCGGCACCAACTCTGTGGGCTGGGCCGTGATCACCGACGAGTACAAGGTGCCCAGCAAGAAATTCAAGGTGCTGGGCAACACCGACCGGCACAGCATCAAGAAGAACCTGATCGGAGCCCTGCTGTTCGACAGCGGCGAAACAGCCGAGGCCACCCGGCTGAAGAGAACCGCCAGAAGAAGATACACCAGACGGAAGAACCGGATCTGCTATCTGCAAGAGATCTTCAGCAACGAGATGGCCAAGGTGGACGACAGCTTCTTCCACAGACTGGAAGAGTCCTTCCTGGTGGAAGAGGATAAGAAGCACGAGCGGCACCCCATCTTCGGCAACATCGTGGACGAGGTGGCCTACCACGAGAAGTACCCCACCATCTACCACCTGAGAAAGAAACTGGTGGACAGCACCGACAAGGCCGACCTGCGGCTGATCTATCTGGCCCTGGCCCACATGATCAAGTTCCGGGGCCACTTCCTGATCGAGGGCGACCTGAACCCCGACAACAGCGACGTGGACAAGCTGTTCATCCAGCTGGTGCAGACCTACAACCAGCTGTTCGAGGAAAACCCCATCAACGCCAGCGGCGTGGACGCCAAGGCCATCCTGTCTGCCAGACTGAGCAAGAGCAGAAAGCTGGAAAATCTGATCGCCCAGCTGCCCGGCGAGAAGAAGAATGGCCTGTTCGGAAACCTGATTGCCCTGAGCCTGGGCCTGACCCCCAACTTCAAGAGCAACTTCGACCTGGCCGAGGATGCCAAACTGCAGCTGAGCAAGGACACCTACGACGACGACCTGGACAACCTGCTGGCCCAGATCGGCGACCAGTACGCCGACCTGTTTCTGGCCGCCAAGAACCTGTCCGACGCCATCCTGCTGAGCGACATCCTGAGAGTGAACACCGAGATCACCAAGGCCCCCCTGAGCGCCTCTATGATCAAGAGATACGACGAGCACCACCAGGACCTGACCCTGCTGAAAGCTCTCGTGCGGCAGCAGCTGCCTGAGAAGTACAAAGAGATTTTCTTCGACCAGAGCAAGAACGGCTACGCCGGCTACATTGACGGCGGAGCCAGCCAGGAAGAGTTCTACAAGTTCATCAAGCCCATCCTGGAAAAGATGGACGGCACCGAGGAACTGCTCGTGAAGCTGAAGAGAGAGGACCTGCTGCGGAAGCAGCGGACCTTCGACAACGGCAGCATCCCCCACCAGATCCACCTGGGAGAGCTGCACGCCATTCTGCGGCGGCAGGAAGATTTTTACCCATTCCTGAAGGACAACCGGGAAAAGATCGAGAAGATCCTGACCTTCCGCATCCCCTACTACGTGGGCCCTCTGGCCAGGGGAAACAGCAGATTCGCCTGGATGACCAGAAAGAGCGAGGAAACCATCACCCCCTGGAACTTCGAGGAAGTGGTGGACAAGGGCGCTTCCGCCCAGAGCTTCATCGAGCGGATGACCAACTTCGATAAGAACCTGCCCAACGAGAAGGTGCTGCCCAAGCACAGCCTGCTGTACGAGTACTTCACCGTGTATAACGAGCTGACCAAAGTGAAATACGTGACCGAGGGAATGAGAAAGCCCGCCTTCCTGAGCGGCGAGCAGAAAAAGGCCATCGTGGACCTGCTGTTCAAGACCAACCGGAAAGTGACCGTGAAGCAGCTGAAAGAGGACTACTTCAAGAAAATCGAGTGCTTCGACTCCGTGGAAATCTCCGGCGTGGAAGATCGGTTCAACGCCTCCCTGGGCACATACCACGATCTGCTGAAAATTATCAAGGACAAGGACTTCCTGGACAATGAGGAAAACGAGGACATTCTGGAAGATATCGTGCTGACCCTGACACTGTTTGAGGACAGAGAGATGATCGAGGAACGGCTGAAAACCTATGCCCACCTGTTCGACGACAAAGTGATGAAGCAGCTGAAGCGGCGGAGATACACCGGCTGGGGCAGGCTGAGCCGGAAGCTGATCAACGGCATCCGGGACAAGCAGTCCGGCAAGACAATCCTGGATTTCCTGAAGTCCGACGGCTTCGCCAACAGAAACTTCATGCAGCTGATCCACGACGACAGCCTGACCTTTAAAGAGGACATCCAGAAAGCCCAGGTGTCCGGCCAGGGCGATAGCCTGCACGAGCACATTGCCAATCTGGCCGGCAGCCCCGCCATTAAGAAGGGCATCCTGCAGACAGTGAAGGTGGTGGACGAGCTCGTGAAAGTGATGGGCCGGCACAAGCCCGAGAACATCGTGATCGAAATGGCCAGAGAGAACCAGACCACCCAGAAGGGACAGAAGAACAGCCGCGAGAGAATGAAGCGGATCGAAGAGGGCATCAAAGAGCTGGGCAGCCAGATCCTGAAAGAACACCCCGTGGAAAACACCCAGCTGCAGAACGAGAAGCTGTACCTGTACTACCTGCAGAATGGGCGGGATATGTACGTGGACCAGGAACTGGACATCAACCGGCTGTCCGACTACGATGTGGACGCTATCGTGCCTCAGAGCTTTCTGAAGGACGACTCCATCGACAACAAGGTGCTGACCAGAAGCGACAAGAACCGGGGCAAGAGCGACAACGTGCCCTCCGAAGAGGTCGTGAAGAAGATGAAGAACTACTGGCGGCAGCTGCTGAACGCCAAGCTGATTACCCAGAGAAAGTTCGACAATCTGACCAAGGCCGAGAGAGGCGGCCTGAGCGAACTGGATAAGGCCGGCTTCATCAAGAGACAGCTGGTGGAAACCCGGCAGATCACAAAGCACGTGGCACAGATCCTGGACTCCCGGATGAACACTAAGTACGACGAGAATGACAAGCTGATCCGGGAAGTGAAAGTGATCACCCTGAAGTCCAAGCTGGTGTCCGATTTCCGGAAGGATTTCCAGTTTTACAAAGTGCGCGAGATCAACAACTACCACCACGCCCACGACGCCTACCTGAACGCCGTCGTGGGAACCGCCCTGATCAAAAAGTACCCTAAGCTGGAAAGCGAGTTCGTGTACGGCGACTACAAGGTGTACGACGTGCGGAAGATGATCGCCAAGAGCGAGCAGGAAATCGGCAAGGCTACCGCCAAGTACTTCTTCTACAGCAACATCATGAACTTTTTCAAGACCGAGATTACCCTGGCCAACGGCGAGATCCGGAAGCGGCCTCTGATCGAGACAAACGGCGAAACCGGGGAGATCGTGTGGGATAAGGGCCGGGATTTTGCCACCGTGCGGAAAGTGCTGAGCATGCCCCAAGTGAATATCGTGAAAAAGACCGAGGTGCAGACAGGCGGCTTCAGCAAAGAGTCTATCCTGCCCAAGAGGAACAGCGATAAGCTGATCGCCAGAAAGAAGGACTGGGACCCTAAGAAGTACGGCGGCTTCGACAGCCCCACCGTGGCCTATTCTGTGCTGGTGGTGGCCAAAGTGGAAAAGGGCAAGTCCAAGAAACTGAAGAGTGTGAAAGAGCTGCTGGGGATCACCATCATGGAAAGAAGCAGCTTCGAGAAGAATCCCATCGACTTTCTGGAAGCCAAGGGCTACAAAGAAGTGAAAAAGGACCTGATCATCAAGCTGCCTAAGTACTCCCTGTTCGAGCTGGAAAACGGCCGGAAGAGAATGCTGGCCTCTGCCGGCGAACTGCAGAAGGGAAACGAACTGGCCCTGCCCTCCAAATATGTGAACTTCCTGTACCTGGCCAGCCACTATGAGAAGCTGAAGGGCTCCCCCGAGGATAATGAGCAGAAACAGCTGTTTGTGGAACAGCACAAGCACTACCTGGACGAGATCATCGAGCAGATCAGCGAGTTCTCCAAGAGAGTGATCCTGGCCGACGCTAATCTGGACAAAGTGCTGTCCGCCTACAACAAGCACCGGGATAAGCCCATCAGAGAGCAGGCCGAGAATATCATCCACCTGTTTACCCTGACCAATCTGGGAGCCCCTGCCGCCTTCAAGTACTTTGACACCACCATCGACCGGAAGAGGTACACCAGCACCAAAGAGGTGCTGGACGCCACCCTGATCCACCAGAGCATCACCGGCCTGTACGAGACACGGATCGACCTGTCTCAGCTGGGAGGTGACTCCGGCGGAAGCTCTGGTGGCAGCAAGCGGACCGCCGACGGCTCTGAATTCGAGAGCCCTAAGAAGAAAAGAAAGGTGAGCGGAGGCTCTAGCGGCGGAAGCACCCTGAACATTGAAGACGAGTATAGACTGCATGAAACAAGCAAGGAACCCGACGTGTCCCTGGGCTCCACCTGGCTGTCCGACTTTCCCCAGGCCTGGGCCGAGACAGGAGGAATGGGCCTGGCCGTGCGGCAGGCACCCCTGATCATCCCTCTGAAGGCCACCTCTACACCCGTGAGCATCAAGCAGTACCCTATGTCTCAGGAGGCCAGACTGGGCATCAAGCCTCACATCCAGAGGCTGCTGGACCAGGGCATCCTGGTGCCATGCCAGAGCCCCTGGAACACACCACTGCTGCCCGTGAAGAAGCCAGGCACCAATGACTATAGACCCGTGCAGGATCTGAGAGAGGTGAACAAGAGGGTGGAGGATATCCACCCCACCGTGCCCAACCCTTACAATCTGCTGTCCGGCCTGCCCCCTTCTCACCAGTGGTATACAGTGCTGGACCTGAAGGATGCCTTCTTTTGTCTGAGACTGCACCCTACCAGCCAGCCACTGTTCGCCTTTGAGTGGAGGGACCCTGAGATGGGCATCTCTGGCCAGCTGACCTGGACACGCCTGCCTCAGGGCTTCAAGAATAGCCCAACACTGTTTAACGAGGCCCTGCACCGCGACCTGGCAGATTTCCGGATCCAGCACCCAGATCTGATCCTGCTGCAGTACGTGGACGATCTGCTGCTGGCCGCCACCAGCGAGCTGGATTGCCAGCAGGGAACACGCGCCCTGCTGCAGACCCTGGGAAACCTGGGATATAGGGCATCCGCCAAGAAGGCCCAGATCTGTCAGAAGCAGGTGAAGTACCTGGGCTATCTGCTGAAGGAGGGCCAGAGATGGCTGACAGAGGCCAGGAAGGAGACAGTGATGGGCCAGCCAACACCCAAGACCCCAAGACAGCTGAGGGAGTTCCTGGGCAAAGCAGGATTTTGCAGGCTGTTCATCCCAGGATTCGCAGAGATGGCAGCACCTCTGTACCCACTGACCAAGCCGGGCACCCTGTTTAATTGGGGCCCTGACCAGCAGAAGGCCTATCAGGAGATCAAGCAGGCCCTGCTGACAGCACCAGCCCTGGGCCTGCCAGACCTGACCAAGCCTTTCGAGCTGTTTGTGGATGAGAAGCAGGGCTACGCCAAGGGCGTGCTGACCCAGAAGCTGGGACCATGGAGACGGCCCGTGGCCTATCTGTCCAAGAAGCTGGACCCAGTGGCAGCAGGATGGCCACCATGCCTGAGGATGGTGGCAGCAATCGCCGTGCTGACAAAGGATGCCGGCAAGCTGACCATGGGACAGCCACTGGTCATCCTGGCACCACACGCAGTGGAGGCCCTGGTGAAGCAGCCTCCAGATCGCTGGCTGTCTAACGCCCGGATGACACACTACCAGGCCCTGCTGCTGGACACCGATCGCGTGCAGTTTGGCCCTGTGGTGGCCCTGAATCCAGCCACCCTGCTGCCTCTGCCAGAGGAGGGCCTGCAGCACAACTGTCTGGACATCCTGGCAGAGGCACACGGAACAAGGCCAGACCTGACCGATCAGCCCCTGCCTGACGCCGATCACACATGGTATACCGATGGAAGCTCCCTGCTGCAGGAGGGCCAGAGGAAGGCAGGAGCAGCAGTGACCACAGAGACAGAAGTGATCTGGGCCAAGGCCCTGCCAGCAGGCACATCCGCCCAGCGGGCCGAGCTGATCGCCCTGACCCAGGCCCTGAAGATGGCCGAGGGCAAGAAGCTGAACGTGTACACAGACTCCAGATATGCCTTCGCCACCGCACACATCCACGGAGAGATCTACAGGCGCCGGGGCTGGCTGACCTCTGAGGGCAAGGAGATCAAGAACAAGGATGAGATCCTGGCCCTGCTGAAGGCCCTGTTTCTGCCCAAGCGGCTGAGCATCATCCACTGTCCTGGACACCAGAAGGGACACTCCGCCGAGGCAAGGGGCAATCGGATGGCCGACCAGGCCGCCAGAAAGGCTGCTATTACTGAAACTCCCGACACTTCCACTCTGCTGATTGAAAACTCCTCCCCTTCTGGCGGCTCAAAAAGAACCGCCGACGGCAGCGAATTCGAGTCTCCCAAGAAGAAGAGGAAAGTCGGCTCTGGCCCTGCCGCTAAGAGAGTGAAGCTGGAC

**Sequence 59 Plasmids sequence of PE-RERV-m5(PEmax-RERV-m5)**

(NLSSV40-nCas9(H840A/R221K/N394K)-NLSSV40-RERV-RT-m5(D204N/V227A/T310K/W317F/E334P)-NLSSV40-NLSc-myc)

CCAAAGAAGAAGCGGAAAGTCAAGCGAAGAAATGATGAAGAAGCCGGCCCGTCCGGCGCCAACCGGAAGGGCCTGAAAGACAAGAAGTACAGCATCGGCCTGGACATCGGCACCAACTCTGTGGGCTGGGCCGTGATCACCGACGAGTACAAGGTGCCCAGCAAGAAATTCAAGGTGCTGGGCAACACCGACCGGCACAGCATCAAGAAGAACCTGATCGGAGCCCTGCTGTTCGACAGCGGCGAAACAGCCGAGGCCACCCGGCTGAAGAGAACCGCCAGAAGAAGATACACCAGACGGAAGAACCGGATCTGCTATCTGCAAGAGATCTTCAGCAACGAGATGGCCAAGGTGGACGACAGCTTCTTCCACAGACTGGAAGAGTCCTTCCTGGTGGAAGAGGATAAGAAGCACGAGCGGCACCCCATCTTCGGCAACATCGTGGACGAGGTGGCCTACCACGAGAAGTACCCCACCATCTACCACCTGAGAAAGAAACTGGTGGACAGCACCGACAAGGCCGACCTGCGGCTGATCTATCTGGCCCTGGCCCACATGATCAAGTTCCGGGGCCACTTCCTGATCGAGGGCGACCTGAACCCCGACAACAGCGACGTGGACAAGCTGTTCATCCAGCTGGTGCAGACCTACAACCAGCTGTTCGAGGAAAACCCCATCAACGCCAGCGGCGTGGACGCCAAGGCCATCCTGTCTGCCAGACTGAGCAAGAGCAGAAAGCTGGAAAATCTGATCGCCCAGCTGCCCGGCGAGAAGAAGAATGGCCTGTTCGGAAACCTGATTGCCCTGAGCCTGGGCCTGACCCCCAACTTCAAGAGCAACTTCGACCTGGCCGAGGATGCCAAACTGCAGCTGAGCAAGGACACCTACGACGACGACCTGGACAACCTGCTGGCCCAGATCGGCGACCAGTACGCCGACCTGTTTCTGGCCGCCAAGAACCTGTCCGACGCCATCCTGCTGAGCGACATCCTGAGAGTGAACACCGAGATCACCAAGGCCCCCCTGAGCGCCTCTATGATCAAGAGATACGACGAGCACCACCAGGACCTGACCCTGCTGAAAGCTCTCGTGCGGCAGCAGCTGCCTGAGAAGTACAAAGAGATTTTCTTCGACCAGAGCAAGAACGGCTACGCCGGCTACATTGACGGCGGAGCCAGCCAGGAAGAGTTCTACAAGTTCATCAAGCCCATCCTGGAAAAGATGGACGGCACCGAGGAACTGCTCGTGAAGCTGAAGAGAGAGGACCTGCTGCGGAAGCAGCGGACCTTCGACAACGGCAGCATCCCCCACCAGATCCACCTGGGAGAGCTGCACGCCATTCTGCGGCGGCAGGAAGATTTTTACCCATTCCTGAAGGACAACCGGGAAAAGATCGAGAAGATCCTGACCTTCCGCATCCCCTACTACGTGGGCCCTCTGGCCAGGGGAAACAGCAGATTCGCCTGGATGACCAGAAAGAGCGAGGAAACCATCACCCCCTGGAACTTCGAGGAAGTGGTGGACAAGGGCGCTTCCGCCCAGAGCTTCATCGAGCGGATGACCAACTTCGATAAGAACCTGCCCAACGAGAAGGTGCTGCCCAAGCACAGCCTGCTGTACGAGTACTTCACCGTGTATAACGAGCTGACCAAAGTGAAATACGTGACCGAGGGAATGAGAAAGCCCGCCTTCCTGAGCGGCGAGCAGAAAAAGGCCATCGTGGACCTGCTGTTCAAGACCAACCGGAAAGTGACCGTGAAGCAGCTGAAAGAGGACTACTTCAAGAAAATCGAGTGCTTCGACTCCGTGGAAATCTCCGGCGTGGAAGATCGGTTCAACGCCTCCCTGGGCACATACCACGATCTGCTGAAAATTATCAAGGACAAGGACTTCCTGGACAATGAGGAAAACGAGGACATTCTGGAAGATATCGTGCTGACCCTGACACTGTTTGAGGACAGAGAGATGATCGAGGAACGGCTGAAAACCTATGCCCACCTGTTCGACGACAAAGTGATGAAGCAGCTGAAGCGGCGGAGATACACCGGCTGGGGCAGGCTGAGCCGGAAGCTGATCAACGGCATCCGGGACAAGCAGTCCGGCAAGACAATCCTGGATTTCCTGAAGTCCGACGGCTTCGCCAACAGAAACTTCATGCAGCTGATCCACGACGACAGCCTGACCTTTAAAGAGGACATCCAGAAAGCCCAGGTGTCCGGCCAGGGCGATAGCCTGCACGAGCACATTGCCAATCTGGCCGGCAGCCCCGCCATTAAGAAGGGCATCCTGCAGACAGTGAAGGTGGTGGACGAGCTCGTGAAAGTGATGGGCCGGCACAAGCCCGAGAACATCGTGATCGAAATGGCCAGAGAGAACCAGACCACCCAGAAGGGACAGAAGAACAGCCGCGAGAGAATGAAGCGGATCGAAGAGGGCATCAAAGAGCTGGGCAGCCAGATCCTGAAAGAACACCCCGTGGAAAACACCCAGCTGCAGAACGAGAAGCTGTACCTGTACTACCTGCAGAATGGGCGGGATATGTACGTGGACCAGGAACTGGACATCAACCGGCTGTCCGACTACGATGTGGACGCTATCGTGCCTCAGAGCTTTCTGAAGGACGACTCCATCGACAACAAGGTGCTGACCAGAAGCGACAAGAACCGGGGCAAGAGCGACAACGTGCCCTCCGAAGAGGTCGTGAAGAAGATGAAGAACTACTGGCGGCAGCTGCTGAACGCCAAGCTGATTACCCAGAGAAAGTTCGACAATCTGACCAAGGCCGAGAGAGGCGGCCTGAGCGAACTGGATAAGGCCGGCTTCATCAAGAGACAGCTGGTGGAAACCCGGCAGATCACAAAGCACGTGGCACAGATCCTGGACTCCCGGATGAACACTAAGTACGACGAGAATGACAAGCTGATCCGGGAAGTGAAAGTGATCACCCTGAAGTCCAAGCTGGTGTCCGATTTCCGGAAGGATTTCCAGTTTTACAAAGTGCGCGAGATCAACAACTACCACCACGCCCACGACGCCTACCTGAACGCCGTCGTGGGAACCGCCCTGATCAAAAAGTACCCTAAGCTGGAAAGCGAGTTCGTGTACGGCGACTACAAGGTGTACGACGTGCGGAAGATGATCGCCAAGAGCGAGCAGGAAATCGGCAAGGCTACCGCCAAGTACTTCTTCTACAGCAACATCATGAACTTTTTCAAGACCGAGATTACCCTGGCCAACGGCGAGATCCGGAAGCGGCCTCTGATCGAGACAAACGGCGAAACCGGGGAGATCGTGTGGGATAAGGGCCGGGATTTTGCCACCGTGCGGAAAGTGCTGAGCATGCCCCAAGTGAATATCGTGAAAAAGACCGAGGTGCAGACAGGCGGCTTCAGCAAAGAGTCTATCCTGCCCAAGAGGAACAGCGATAAGCTGATCGCCAGAAAGAAGGACTGGGACCCTAAGAAGTACGGCGGCTTCGACAGCCCCACCGTGGCCTATTCTGTGCTGGTGGTGGCCAAAGTGGAAAAGGGCAAGTCCAAGAAACTGAAGAGTGTGAAAGAGCTGCTGGGGATCACCATCATGGAAAGAAGCAGCTTCGAGAAGAATCCCATCGACTTTCTGGAAGCCAAGGGCTACAAAGAAGTGAAAAAGGACCTGATCATCAAGCTGCCTAAGTACTCCCTGTTCGAGCTGGAAAACGGCCGGAAGAGAATGCTGGCCTCTGCCGGCGAACTGCAGAAGGGAAACGAACTGGCCCTGCCCTCCAAATATGTGAACTTCCTGTACCTGGCCAGCCACTATGAGAAGCTGAAGGGCTCCCCCGAGGATAATGAGCAGAAACAGCTGTTTGTGGAACAGCACAAGCACTACCTGGACGAGATCATCGAGCAGATCAGCGAGTTCTCCAAGAGAGTGATCCTGGCCGACGCTAATCTGGACAAAGTGCTGTCCGCCTACAACAAGCACCGGGATAAGCCCATCAGAGAGCAGGCCGAGAATATCATCCACCTGTTTACCCTGACCAATCTGGGAGCCCCTGCCGCCTTCAAGTACTTTGACACCACCATCGACCGGAAGAGGTACACCAGCACCAAAGAGGTGCTGGACGCCACCCTGATCCACCAGAGCATCACCGGCCTGTACGAGACACGGATCGACCTGTCTCAGCTGGGAGGTGACTCCGGCGGAAGCTCTGGTGGCAGCAAGCGGACCGCCGACGGCTCTGAATTCGAGAGCCCTAAGAAGAAAAGAAAGGTGAGCGGAGGCTCTAGCGGCGGAAGCCCTCCTGTGGCCTGCCTGGTCTTAAGCCTCGAGGAGGAATACCGCCTGCATGAGCAGAACCCCAAGCAGCTCCTGGCCCCAGAATGGTTGAGCGCTTTCTCTGAGGTCTGGGCTGAGCAAACTGGAATGGGCCTGGCCAAGCAGGTGCCCCCTGTCGTAGTGGAGCTGAAGGCTGACGCTAGCCCAGTGTCAGTGAAGCAGTACCCCATGTCTAGAGAAGCCAAGGAGGGCATCCGGCCACACATCCAGCGGCTTCTTCAGTTGGGCATCCTGGTACCCTGCCAGTCTCCCTGGAACACTCCGCTGCTGCCTGTGAGGAAGCCTGGGACCAATGACTATAGGCCTGTCCAGGATTTACGGGAAGTGAACAAAAGAGTACAAGACATTCACCCTACTGTGCCCAACCCCTACAATTTGCTGAGCTCTCTGCCTCCTGAGCGCACATGGTACACAGTCCTGGACCTCAAGGATGCCTTCTTCTGCCTCCGCCTGCATCCAAACTCCCAGCCACTCTTCGCCTTTGAGTGGAGAGACCCAGAGGGAGGCCACACAGGCCAGCTGACGTGGACTCGGCTGCCCCAGGGTTTCAAAAACTCCCCGACTCTGTTTAATGAAGCTCTGCACAGGGACCTGGCGCCTTTCCGGGCTCAAAACCCACAAATCTCATTACTGCAGTACGCTGATGATTTGCTGCTAGCTGCGTCCACTCAGGAGTTGTGTAGAGAAGGCACCAAGAGACTGCTGAATGAACTCGGAGAGCTCGGCTACAGGGTGTCCGCGAAGAAAGCTCAGCTGTGCAGGACTGAGGTGACCTACCTCGGCTACACTCTCCGGGAGGGTAAACGTTGGCTGACAGAGGCCCGAAAGAAGACAGTCATGCAGATCCCTACCCCTACGACCCCCCGTCAGGTGCGGGAGTTCCTGGGGAAGGCAGGCTTCTGTAGACTCTTCATCCCAGGCTTTGCTACCCTGGCAGCACCTCTGTACCTGCTGACTAAGCCGAAGGTGCCATTCACTTGGACAGAAGAACACCAGAGAGCGTTTGAAGACATAAAAGCCGCCCTCCTGGCAGCCCCAGCCCTAGCCCTCCCAGACCTGACCAAGCCATTCACCCTGTATGTGGATGAGAGAGCTGGCGTTGCCCGTGGTGTACTTACCCAGGCCCTTGGCCCGTGGAAGAGACCCGTGGCATACTTAAGTAAGAAGCTGGACCCTGTCGCCAGTGGCTGGCCAAGCTGCCTCAAGGCCATTGCAGCTGTGGCTCTGCTCGTGAAGGATGCTGACAAGCTTACGCTGGGCCAGCATGTGACCGTGATCGCTCCCCACGCCCTAGAGTCTATTGTCCGGCAGCCACCCGATAGGTGGATGACCAACGCAAGGATGACTCACTATCAGTCACTGTTACTGAATGAGAGGGTGACGTTTGCTCCTCCTGCGATCTTGAATCCTGCAACCCTGCTCCCCGAAATCCACAATAGCACACCCATCCATCAGTGTGTGGACATCTTGGCCGAAGAAACAGGCACTAGAAAGGACCTTACAGACCGGCCTTGGCCTGGAGTGCCTGCCTGGTACACTGATGGCAGTTCCTTTGTGGTAGAGGGCAAAAGAAGAGCCGGCGCAGCTGTAGTTGATGGCAAACAGGTCATCTGGGCCAGCAGCCTACCAGAAGGGACGTCTGCACAGAAAGCAGAGCTGGTGGCCCTGACTCAAGCCCTGCGTCTGGCGGAAGGAAAGGCCATCAACATTTATACAGACTCCCGCTATGCCTTCGCCACAGCCCACATTCATGGGGCCATCTACAAACAGCGAGGCTTACTGACGTCAGCTGGAAGAGATATCAAAAACAAGGAGGAGATCCTGGCCCTTCTGGAAGCTGTCCACCTTCCTAAAAAGGTTGCCATTATTCACTGCCCTGGGCACCAGAAGGGAGAAGATCCCATAACCAAGGGAAACCAGATGGCAGACCTAGTGGCTAAACAGGTGGCCCAGCAGGTGACCATCCTGGCGGAAAAGTCTCAAACCCCCGTGAAAACACCTGTTACAGATGACAGCAACTATTCTGGCGGCTCAAAAAGAACCGCCGACGGCAGCGAATTCGAGTCTCCCAAGAAGAAGAGGAAAGTCGGCTCTGGCCCTGCCGCTAAGAGAGTGAAGCTGGAC

**Sequence 60 Plasmids sequence of PE-enRERV(PEmax-enRERV)**

(NLSSV40-nCas9(H840A/R221K/N394K)-NLSSV40-enRERV-RT(Y68R/W149F/D204N/V227A/T310K/W317F/E334P/C413R)-NLSSV40-NLSc-myc)

CCAAAGAAGAAGCGGAAAGTCAAGCGAAGAAATGATGAAGAAGCCGGCCCGTCCGGCGCCAACCGGAAGGGCCTGAAAGACAAGAAGTACAGCATCGGCCTGGACATCGGCACCAACTCTGTGGGCTGGGCCGTGATCACCGACGAGTACAAGGTGCCCAGCAAGAAATTCAAGGTGCTGGGCAACACCGACCGGCACAGCATCAAGAAGAACCTGATCGGAGCCCTGCTGTTCGACAGCGGCGAAACAGCCGAGGCCACCCGGCTGAAGAGAACCGCCAGAAGAAGATACACCAGACGGAAGAACCGGATCTGCTATCTGCAAGAGATCTTCAGCAACGAGATGGCCAAGGTGGACGACAGCTTCTTCCACAGACTGGAAGAGTCCTTCCTGGTGGAAGAGGATAAGAAGCACGAGCGGCACCCCATCTTCGGCAACATCGTGGACGAGGTGGCCTACCACGAGAAGTACCCCACCATCTACCACCTGAGAAAGAAACTGGTGGACAGCACCGACAAGGCCGACCTGCGGCTGATCTATCTGGCCCTGGCCCACATGATCAAGTTCCGGGGCCACTTCCTGATCGAGGGCGACCTGAACCCCGACAACAGCGACGTGGACAAGCTGTTCATCCAGCTGGTGCAGACCTACAACCAGCTGTTCGAGGAAAACCCCATCAACGCCAGCGGCGTGGACGCCAAGGCCATCCTGTCTGCCAGACTGAGCAAGAGCAGAAAGCTGGAAAATCTGATCGCCCAGCTGCCCGGCGAGAAGAAGAATGGCCTGTTCGGAAACCTGATTGCCCTGAGCCTGGGCCTGACCCCCAACTTCAAGAGCAACTTCGACCTGGCCGAGGATGCCAAACTGCAGCTGAGCAAGGACACCTACGACGACGACCTGGACAACCTGCTGGCCCAGATCGGCGACCAGTACGCCGACCTGTTTCTGGCCGCCAAGAACCTGTCCGACGCCATCCTGCTGAGCGACATCCTGAGAGTGAACACCGAGATCACCAAGGCCCCCCTGAGCGCCTCTATGATCAAGAGATACGACGAGCACCACCAGGACCTGACCCTGCTGAAAGCTCTCGTGCGGCAGCAGCTGCCTGAGAAGTACAAAGAGATTTTCTTCGACCAGAGCAAGAACGGCTACGCCGGCTACATTGACGGCGGAGCCAGCCAGGAAGAGTTCTACAAGTTCATCAAGCCCATCCTGGAAAAGATGGACGGCACCGAGGAACTGCTCGTGAAGCTGAAGAGAGAGGACCTGCTGCGGAAGCAGCGGACCTTCGACAACGGCAGCATCCCCCACCAGATCCACCTGGGAGAGCTGCACGCCATTCTGCGGCGGCAGGAAGATTTTTACCCATTCCTGAAGGACAACCGGGAAAAGATCGAGAAGATCCTGACCTTCCGCATCCCCTACTACGTGGGCCCTCTGGCCAGGGGAAACAGCAGATTCGCCTGGATGACCAGAAAGAGCGAGGAAACCATCACCCCCTGGAACTTCGAGGAAGTGGTGGACAAGGGCGCTTCCGCCCAGAGCTTCATCGAGCGGATGACCAACTTCGATAAGAACCTGCCCAACGAGAAGGTGCTGCCCAAGCACAGCCTGCTGTACGAGTACTTCACCGTGTATAACGAGCTGACCAAAGTGAAATACGTGACCGAGGGAATGAGAAAGCCCGCCTTCCTGAGCGGCGAGCAGAAAAAGGCCATCGTGGACCTGCTGTTCAAGACCAACCGGAAAGTGACCGTGAAGCAGCTGAAAGAGGACTACTTCAAGAAAATCGAGTGCTTCGACTCCGTGGAAATCTCCGGCGTGGAAGATCGGTTCAACGCCTCCCTGGGCACATACCACGATCTGCTGAAAATTATCAAGGACAAGGACTTCCTGGACAATGAGGAAAACGAGGACATTCTGGAAGATATCGTGCTGACCCTGACACTGTTTGAGGACAGAGAGATGATCGAGGAACGGCTGAAAACCTATGCCCACCTGTTCGACGACAAAGTGATGAAGCAGCTGAAGCGGCGGAGATACACCGGCTGGGGCAGGCTGAGCCGGAAGCTGATCAACGGCATCCGGGACAAGCAGTCCGGCAAGACAATCCTGGATTTCCTGAAGTCCGACGGCTTCGCCAACAGAAACTTCATGCAGCTGATCCACGACGACAGCCTGACCTTTAAAGAGGACATCCAGAAAGCCCAGGTGTCCGGCCAGGGCGATAGCCTGCACGAGCACATTGCCAATCTGGCCGGCAGCCCCGCCATTAAGAAGGGCATCCTGCAGACAGTGAAGGTGGTGGACGAGCTCGTGAAAGTGATGGGCCGGCACAAGCCCGAGAACATCGTGATCGAAATGGCCAGAGAGAACCAGACCACCCAGAAGGGACAGAAGAACAGCCGCGAGAGAATGAAGCGGATCGAAGAGGGCATCAAAGAGCTGGGCAGCCAGATCCTGAAAGAACACCCCGTGGAAAACACCCAGCTGCAGAACGAGAAGCTGTACCTGTACTACCTGCAGAATGGGCGGGATATGTACGTGGACCAGGAACTGGACATCAACCGGCTGTCCGACTACGATGTGGACGCTATCGTGCCTCAGAGCTTTCTGAAGGACGACTCCATCGACAACAAGGTGCTGACCAGAAGCGACAAGAACCGGGGCAAGAGCGACAACGTGCCCTCCGAAGAGGTCGTGAAGAAGATGAAGAACTACTGGCGGCAGCTGCTGAACGCCAAGCTGATTACCCAGAGAAAGTTCGACAATCTGACCAAGGCCGAGAGAGGCGGCCTGAGCGAACTGGATAAGGCCGGCTTCATCAAGAGACAGCTGGTGGAAACCCGGCAGATCACAAAGCACGTGGCACAGATCCTGGACTCCCGGATGAACACTAAGTACGACGAGAATGACAAGCTGATCCGGGAAGTGAAAGTGATCACCCTGAAGTCCAAGCTGGTGTCCGATTTCCGGAAGGATTTCCAGTTTTACAAAGTGCGCGAGATCAACAACTACCACCACGCCCACGACGCCTACCTGAACGCCGTCGTGGGAACCGCCCTGATCAAAAAGTACCCTAAGCTGGAAAGCGAGTTCGTGTACGGCGACTACAAGGTGTACGACGTGCGGAAGATGATCGCCAAGAGCGAGCAGGAAATCGGCAAGGCTACCGCCAAGTACTTCTTCTACAGCAACATCATGAACTTTTTCAAGACCGAGATTACCCTGGCCAACGGCGAGATCCGGAAGCGGCCTCTGATCGAGACAAACGGCGAAACCGGGGAGATCGTGTGGGATAAGGGCCGGGATTTTGCCACCGTGCGGAAAGTGCTGAGCATGCCCCAAGTGAATATCGTGAAAAAGACCGAGGTGCAGACAGGCGGCTTCAGCAAAGAGTCTATCCTGCCCAAGAGGAACAGCGATAAGCTGATCGCCAGAAAGAAGGACTGGGACCCTAAGAAGTACGGCGGCTTCGACAGCCCCACCGTGGCCTATTCTGTGCTGGTGGTGGCCAAAGTGGAAAAGGGCAAGTCCAAGAAACTGAAGAGTGTGAAAGAGCTGCTGGGGATCACCATCATGGAAAGAAGCAGCTTCGAGAAGAATCCCATCGACTTTCTGGAAGCCAAGGGCTACAAAGAAGTGAAAAAGGACCTGATCATCAAGCTGCCTAAGTACTCCCTGTTCGAGCTGGAAAACGGCCGGAAGAGAATGCTGGCCTCTGCCGGCGAACTGCAGAAGGGAAACGAACTGGCCCTGCCCTCCAAATATGTGAACTTCCTGTACCTGGCCAGCCACTATGAGAAGCTGAAGGGCTCCCCCGAGGATAATGAGCAGAAACAGCTGTTTGTGGAACAGCACAAGCACTACCTGGACGAGATCATCGAGCAGATCAGCGAGTTCTCCAAGAGAGTGATCCTGGCCGACGCTAATCTGGACAAAGTGCTGTCCGCCTACAACAAGCACCGGGATAAGCCCATCAGAGAGCAGGCCGAGAATATCATCCACCTGTTTACCCTGACCAATCTGGGAGCCCCTGCCGCCTTCAAGTACTTTGACACCACCATCGACCGGAAGAGGTACACCAGCACCAAAGAGGTGCTGGACGCCACCCTGATCCACCAGAGCATCACCGGCCTGTACGAGACACGGATCGACCTGTCTCAGCTGGGAGGTGACTCCGGCGGAAGCTCTGGTGGCAGCAAGCGGACCGCCGACGGCTCTGAATTCGAGAGCCCTAAGAAGAAAAGAAAGGTGAGCGGAGGCTCTAGCGGCGGAAGCCCTCCTGTGGCCTGCCTGGTCTTAAGCCTCGAGGAGGAATACCGCCTGCATGAGCAGAACCCCAAGCAGCTCCTGGCCCCAGAATGGTTGAGCGCTTTCTCTGAGGTCTGGGCTGAGCAAACTGGAATGGGCCTGGCCAAGCAGGTGCCCCCTGTCGTAGTGGAGCTGAAGGCTGACGCTAGCCCAGTGTCAGTGAAGCAGAGACCCATGTCTAGAGAAGCCAAGGAGGGCATCCGGCCACACATCCAGCGGCTTCTTCAGTTGGGCATCCTGGTACCCTGCCAGTCTCCCTGGAACACTCCGCTGCTGCCTGTGAGGAAGCCTGGGACCAATGACTATAGGCCTGTCCAGGATTTACGGGAAGTGAACAAAAGAGTACAAGACATTCACCCTACTGTGCCCAACCCCTACAATTTGCTGAGCTCTCTGCCTCCTGAGCGCACATTCTACACAGTCCTGGACCTCAAGGATGCCTTCTTCTGCCTCCGCCTGCATCCAAACTCCCAGCCACTCTTCGCCTTTGAGTGGAGAGACCCAGAGGGAGGCCACACAGGCCAGCTGACGTGGACTCGGCTGCCCCAGGGTTTCAAAAACTCCCCGACTCTGTTTAATGAAGCTCTGCACAGGGACCTGGCGCCTTTCCGGGCTCAAAACCCACAAATCTCATTACTGCAGTACGCTGATGATTTGCTGCTAGCTGCGTCCACTCAGGAGTTGTGTAGAGAAGGCACCAAGAGACTGCTGAATGAACTCGGAGAGCTCGGCTACAGGGTGTCCGCGAAGAAAGCTCAGCTGTGCAGGACTGAGGTGACCTACCTCGGCTACACTCTCCGGGAGGGTAAACGTTGGCTGACAGAGGCCCGAAAGAAGACAGTCATGCAGATCCCTACCCCTACGACCCCCCGTCAGGTGCGGGAGTTCCTGGGGAAGGCAGGCTTCTGTAGACTCTTCATCCCAGGCTTTGCTACCCTGGCAGCACCTCTGTACCTGCTGACTAAGCCGAAGGTGCCATTCACTTGGACAGAAGAACACCAGAGAGCGTTTGAAGACATAAAAGCCGCCCTCCTGGCAGCCCCAGCCCTAGCCCTCCCAGACCTGACCAAGCCATTCACCCTGTATGTGGATGAGAGAGCTGGCGTTGCCCGTGGTGTACTTACCCAGGCCCTTGGCCCGTGGAAGAGACCCGTGGCATACTTAAGTAAGAAGCTGGACCCTGTCGCCAGTGGCTGGCCAAGCAGACTCAAGGCCATTGCAGCTGTGGCTCTGCTCGTGAAGGATGCTGACAAGCTTACGCTGGGCCAGCATGTGACCGTGATCGCTCCCCACGCCCTAGAGTCTATTGTCCGGCAGCCACCCGATAGGTGGATGACCAACGCAAGGATGACTCACTATCAGTCACTGTTACTGAATGAGAGGGTGACGTTTGCTCCTCCTGCGATCTTGAATCCTGCAACCCTGCTCCCCGAAATCCACAATAGCACACCCATCCATCAGTGTGTGGACATCTTGGCCGAAGAAACAGGCACTAGAAAGGACCTTACAGACCGGCCTTGGCCTGGAGTGCCTGCCTGGTACACTGATGGCAGTTCCTTTGTGGTAGAGGGCAAAAGAAGAGCCGGCGCAGCTGTAGTTGATGGCAAACAGGTCATCTGGGCCAGCAGCCTACCAGAAGGGACGTCTGCACAGAAAGCAGAGCTGGTGGCCCTGACTCAAGCCCTGCGTCTGGCGGAAGGAAAGGCCATCAACATTTATACAGACTCCCGCTATGCCTTCGCCACAGCCCACATTCATGGGGCCATCTACAAACAGCGAGGCTTACTGACGTCAGCTGGAAGAGATATCAAAAACAAGGAGGAGATCCTGGCCCTTCTGGAAGCTGTCCACCTTCCTAAAAAGGTTGCCATTATTCACTGCCCTGGGCACCAGAAGGGAGAAGATCCCATAACCAAGGGAAACCAGATGGCAGACCTAGTGGCTAAACAGGTGGCCCAGCAGGTGACCATCCTGGCGGAAAAGTCTCAAACCCCCGTGAAAACACCTGTTACAGATGACAGCAACTATTCTGGCGGCTCAAAAAGAACCGCCGACGGCAGCGAATTCGAGTCTCCCAAGAAGAAGAGGAAAGTCGGCTCTGGCCCTGCCGCTAAGAGAGTGAAGCTGGAC

**Sequence 61 Plasmids sequence of PE-RERV-Δ1**

(NLSc-myc-NLSSV40-nCas9(H840A/R221K/N394K)-XTEN-NC-NLS-32aa Linker-RERV-RT(Δ1~42aa)-NLSvbp)

AAGCTTACTGGTTGTACAATGCCGGCGGCCAAGAGAGTCAAGCTCGACGGCGGGAAGCGCACAGCTGATGGTTCTGAATTCGAGTCCCCTAAGAAAAAGAGAAAAGTGGACAAGAAGTACTCGATCGGCCTCGATATTGGGACTAACTCTGTTGGCTGGGCCGTGATCACCGACGAGTACAAGGTGCCCTCAAAGAAGTTCAAGGTCCTGGGCAACACCGATCGGCATTCCATCAAGAAGAATCTCATTGGCGCTCTCCTGTTCGACAGCGGCGAGACGGCTGAGGCTACGCGGCTCAAGCGCACCGCCCGCAGGCGGTACACGCGCAGGAAGAATCGCATCTGCTACCTGCAGGAGATTTTCTCCAACGAGATGGCGAAGGTTGACGATTCTTTCTTCCACAGGCTGGAGGAGTCATTCCTCGTGGAGGAGGATAAGAAGCACGAGCGGCATCCAATCTTCGGCAACATTGTCGACGAGGTTGCCTACCACGAGAAGTACCCTACGATCTACCATCTGCGGAAGAAGCTCGTGGACTCCACAGATAAGGCGGACCTCCGCCTGATCTACCTCGCTCTGGCCCACATGATTAAGTTCAGGGGCCATTTCCTGATCGAGGGGGATCTCAACCCGGACAATAGCGATGTTGACAAGCTGTTCATCCAGCTCGTGCAGACGTACAACCAGCTCTTCGAGGAGAACCCCATTAATGCGTCAGGCGTCGACGCGAAGGCTATCCTGTCCGCTAGGCTCTCGAAGTCTCGGAAGCTCGAGAACCTGATCGCCCAGCTGCCGGGCGAGAAGAAGAACGGCCTGTTCGGGAATCTCATTGCGCTCAGCCTGGGGCTCACGCCCAACTTCAAGTCGAATTTCGATCTCGCTGAGGACGCCAAGCTGCAGCTCTCCAAGGACACATACGACGATGACCTGGATAACCTCCTGGCCCAGATCGGCGATCAGTACGCGGACCTGTTCCTCGCTGCCAAGAATCTGTCGGACGCCATCCTCCTGTCTGATATTCTCAGGGTGAACACCGAGATTACGAAGGCTCCGCTCTCAGCCTCCATGATCAAGCGCTACGACGAGCACCATCAGGATCTGACCCTCCTGAAGGCGCTGGTCAGGCAGCAGCTCCCCGAGAAGTACAAGGAGATCTTCTTCGATCAGTCGAAGAACGGCTACGCTGGGTACATTGACGGCGGGGCCTCTCAGGAGGAGTTCTACAAGTTCATCAAGCCGATTCTGGAGAAGATGGACGGCACGGAGGAGCTGCTGGTGAAGCTCAAGCGCGAGGACCTCCTGAGGAAGCAGCGGACATTCGATAACGGCAGCATCCCACACCAGATTCATCTCGGGGAGCTGCACGCTATCCTGAGGAGGCAGGAGGACTTCTACCCTTTCCTCAAGGATAACCGCGAGAAGATCGAGAAGATTCTGACTTTCAGGATCCCGTACTACGTCGGCCCACTCGCTAGGGGCAACTCCCGCTTCGCTTGGATGACCCGCAAGTCAGAGGAGACGATCACGCCGTGGAACTTCGAGGAGGTGGTCGACAAGGGCGCTAGCGCTCAGTCGTTCATCGAGAGGATGACGAATTTCGACAAGAACCTGCCAAATGAGAAGGTGCTCCCTAAGCACTCGCTCCTGTACGAGTACTTCACAGTCTACAACGAGCTGACTAAGGTGAAGTATGTGACCGAGGGCATGAGGAAGCCGGCTTTCCTGTCTGGGGAGCAGAAGAAGGCCATCGTGGACCTCCTGTTCAAGACCAACCGGAAGGTCACGGTTAAGCAGCTCAAGGAGGACTACTTCAAGAAGATTGAGTGCTTCGATTCGGTCGAGATCTCTGGCGTTGAGGACCGCTTCAACGCCTCCCTGGGGACCTACCACGATCTCCTGAAGATCATTAAGGATAAGGACTTCCTGGACAACGAGGAGAATGAGGATATCCTCGAGGACATTGTGCTGACACTCACTCTGTTCGAGGACCGGGAGATGATCGAGGAGCGCCTGAAGACTTACGCCCATCTCTTCGATGACAAGGTCATGAAGCAGCTCAAGAGGAGGAGGTACACCGGCTGGGGGAGGCTGAGCAGGAAGCTCATCAACGGCATTCGGGACAAGCAGTCCGGGAAGACGATCCTCGACTTCCTGAAGAGCGATGGCTTCGCGAACCGCAATTTCATGCAGCTGATTCACGATGACAGCCTCACATTCAAGGAGGATATCCAGAAGGCTCAGGTGAGCGGCCAGGGGGACTCGCTGCACGAGCATATCGCGAACCTCGCTGGCTCGCCAGCTATCAAGAAGGGGATTCTGCAGACCGTGAAGGTTGTGGACGAGCTGGTGAAGGTCATGGGCAGGCACAAGCCTGAGAACATCGTCATTGAGATGGCCCGGGAGAATCAGACCACGCAGAAGGGCCAGAAGAACTCACGCGAGAGGATGAAGAGGATCGAGGAGGGCATTAAGGAGCTGGGGTCCCAGATCCTCAAGGAGCACCCGGTGGAGAACACGCAGCTGCAGAATGAGAAGCTCTACCTGTACTACCTCCAGAATGGCCGCGATATGTATGTGGACCAGGAGCTGGATATTAACAGGCTCAGCGATTACGACGTCGATGCCATCGTTCCACAGTCATTCCTGAAGGATGACTCCATTGACAACAAGGTCCTCACCAGGTCGGACAAGAACCGGGGCAAGTCTGATAATGTTCCTTCAGAGGAGGTCGTTAAGAAGATGAAGAACTACTGGCGCCAGCTCCTGAATGCCAAGCTGATCACGCAGCGGAAGTTCGATAACCTCACAAAGGCTGAGAGGGGCGGGCTCTCTGAGCTGGACAAGGCGGGCTTCATCAAGAGGCAGCTGGTCGAGACACGGCAGATCACTAAGCACGTTGCGCAGATTCTCGACTCACGGATGAACACTAAGTACGATGAGAATGACAAGCTGATCCGCGAGGTGAAGGTCATCACCCTGAAGTCAAAGCTCGTCTCCGACTTCAGGAAGGATTTCCAGTTCTACAAGGTTCGGGAGATCAACAATTACCACCATGCCCATGACGCGTACCTGAACGCGGTGGTCGGCACAGCTCTGATCAAGAAGTACCCAAAGCTCGAGAGCGAGTTCGTGTACGGGGACTACAAGGTTTACGATGTGAGGAAGATGATCGCCAAGTCGGAGCAGGAGATTGGCAAGGCTACCGCCAAGTACTTCTTCTACTCTAACATTATGAATTTCTTCAAGACAGAGATCACTCTGGCCAATGGCGAGATCCGGAAGCGCCCCCTCATCGAGACGAACGGCGAGACGGGGGAGATCGTGTGGGACAAGGGCAGGGATTTCGCGACCGTCAGGAAGGTTCTCTCCATGCCACAAGTGAATATCGTCAAGAAGACAGAGGTCCAGACTGGCGGGTTCTCTAAGGAGTCAATTCTGCCTAAGCGGAACAGCGACAAGCTCATCGCCCGCAAGAAGGACTGGGATCCGAAGAAGTACGGCGGGTTCGACAGCCCCACTGTGGCCTACTCGGTCCTGGTTGTGGCGAAGGTTGAGAAGGGCAAGTCCAAGAAGCTCAAGAGCGTGAAGGAGCTGCTGGGGATCACGATTATGGAGCGCTCCAGCTTCGAGAAGAACCCGATCGATTTCCTGGAGGCGAAGGGCTACAAGGAGGTGAAGAAGGACCTGATCATTAAGCTCCCCAAGTACTCACTCTTCGAGCTGGAGAACGGCAGGAAGCGGATGCTGGCTTCCGCTGGCGAGCTGCAGAAGGGGAACGAGCTGGCTCTGCCGTCCAAGTATGTGAACTTCCTCTACCTGGCCTCCCACTACGAGAAGCTCAAGGGCAGCCCCGAGGACAACGAGCAGAAGCAGCTGTTCGTCGAGCAGCACAAGCATTACCTCGACGAGATCATTGAGCAGATTTCCGAGTTCTCCAAGCGCGTGATCCTGGCCGACGCGAATCTGGATAAGGTCCTCTCCGCGTACAACAAGCACCGCGACAAGCCAATCAGGGAGCAGGCTGAGAATATCATTCATCTCTTCACCCTGACGAACCTCGGCGCCCCTGCTGCTTTCAAGTACTTCGACACAACTATCGATCGCAAGAGGTACACAAGCACTAAGGAGGTCCTGGACGCGACCCTCATCCACCAGTCGATTACCGGCCTCTACGAGACGCGCATCGACCTGTCTCAGCTCGGGGGCGACGAATTCTCCGGGAGCGAGACGCCAGGCACCTCCGAGTCGGCCACCCCAGAATCTGCCACAGTGGTGTCCGGCCAAAAGCAGGACCGCCAGGGCGGAGAACGCAGAAGGTCCCAGCTCGATAGGGATCAGTGTGCCTACTGCAAGGAGAAGGGCCACTGGGCCAAAGACTGCCCGAAAAAGCCGCGCGGCCCACGCGGCCCAAGGCCACAAACATCCCTCCTTCCAAAGAAGAAGCGGAAGGTGGAGCTCAGCGGAGGATCTTCCGGAGGATCTAGCGGCTCCGAGACACCAGGAACATCCGAAAGCGCTACACCAGAATCTAGCGGAGGCTCTTCCGGAGGATCTAGG ATGGGACTGGCCAAGCAGGTGCCTCCTGTGGTGGTAGAGCTGAAGGCGGACGCCAGCCCAGTGAGCGTGAAACAATACCCGATGTCGAGGGAAGCAAAGGAAGGCATTAGACCCCACATCCAGAGACTGCTGCAATTGGGAATCCTGGTACCCTGCCAAAGCCCTTGGAACACGCCACTGCTGCCTGTGCGCAAACCAGGAACTAATGATTACAGACCTGTACAGGATCTAAGGGAAGTTAACAAGCGGGTGCAGGACATACATCCTACGGTCCCAAACCCCTACAACTTGCTGAGCTCTCTGCCCCCCGAGCGCACATGGTACACTGTGCTCGACTTAAAAGATGCGTTCTTCTGCCTTCGCCTCCACCCGAACTCACAGCCACTGTTCGCGTTCGAGTGGCGCGACCCTGAGGGCGGCCACACCGGGCAACTGACGTGGACACGGCTGCCACAGGGTTTCAAGAACAGCCCTACTCTATTTGATGAGGCCCTGCACCGTGATCTGGCCCCCTTCCGGGCGCAGAACCCTCAAATCAGTCTACTGCAGTATGTTGATGACCTGCTGCTAGCCGCTTCTACTCAGGAGCTCTGCCGGGAGGGCACAAAGCGGCTGTTAAATGAGCTGGGCGAGCTGGGATACCGCGTGTCTGCCAAGAAGGCACAATTGTGTCGGACAGAGGTGACATATCTCGGCTACACATTAAGAGAAGGTAAGAGATGGCTCACTGAAGCACGTAAGAAGACTGTCATGCAGATTCCTACCCCTACCACGCCCCGTCAGGTGCGGGAGTTCCTCGGCACCGCCGGCTTCTGTAGACTGTGGATTCCTGGATTTGCCACCCTGGCCGCCCCACTCTACCTGCTGACGAAGGAAAAGGTTCCTTTCACCTGGACTGAGGAACATCAGCGGGCTTTCGAAGACATCAAAGCCGCCCTGCTGGCGGCTCCAGCTCTGGCCCTCCCTGACCTCACCAAGCCCTTCACACTCTATGTGGACGAGAGAGCTGGAGTGGCCAGAGGTGTGCTGACACAGGCACTGGGGCCGTGGAAGAGACCCGTGGCGTACCTATCTAAAAAGCTCGACCCCGTGGCCTCTGGGTGGCCGAGCTGTCTAAAGGCCATCGCTGCTGTAGCTCTACTGGTCAAAGATGCTGACAAGCTGACCCTGGGCCAGCATGTCACCGTCATCGCCCCGCACGCCTTAGAATCCATAGTCCGGCAACCACCAGATCGGTGGATGACAAACGCTAGGATGACGCATTATCAGAGCTTGCTGTTAAACGAAAGAGTGACATTTGCGCCCCCTGCCATCCTTAACCCAGCGACGCTACTGCCGGAGATCCACAACTCAACCCCCATCCACCAGTGCGTCGACATTCTGGCTGAGGAGACCGGCACGCGCAAGGACCTCACGGACAGACCATGGCCTGGTGTGCCGGCATGGTACACCGACGGCAGCTCCTTTGTCGTGGAGGGAAAGCGACGCGCAGGCGCGGCTGTGGTGGACGGCAAGCAAGTCATCTGGGCATCCTCCCTGCCAGAAGGCACCAGCGCTCAGAAAGCGGAGTTGGTGGCCTTGACCCAGGCCCTCAGATTAGCTGAAGGGAAGGCTATTAATATCTACACCGATTCTAGATACGCCTTCGCCACGGCGCACATCCACGGCGCGATCTACAAGCAGCGGGGCCTGCTGACCAGTGCCGGGCGAGACATCAAGAATAAGGAAGAGATTCTTGCCCTGCTGGAGGCCGTCCACCTCCCCAAAAAGGTGGCCATTATCCACTGCCCCGGCCACCAGAAGGGAGAGGATCCAATTACAAAGGGGAACCAGATGGCCGACCTCGTGGCGAAGCAGGTCGCTCAGCAGGTTACCATCCTGGCAGAAAAATCGCAGACCCCTGTGAAGACACCTGTGACAGATGATTCTAACTATAGCGGCGGCAGCAAAAGAACGGCGGACGGCTCTGAGAAGCGCACCGCTGATAGCCAGCATTCAACTCCTCCGAAAACAAAGAGGAAAGTTGAGTTCGAACCGAAGAAGAAAAGGAAGGTGTGA

**Sequence 62 Plasmids sequence of PE-RERV-Δ2**

(NLSc-myc-NLSSV40-nCas9(H840A/R221K/N394K)-XTEN-NC-NLS-32aa Linker-RERV-RT(Δ1~51aa)-NLSvbp)

AAGCTTACTGGTTGTACAATGCCGGCGGCCAAGAGAGTCAAGCTCGACGGCGGGAAGCGCACAGCTGATGGTTCTGAATTCGAGTCCCCTAAGAAAAAGAGAAAAGTGGACAAGAAGTACTCGATCGGCCTCGATATTGGGACTAACTCTGTTGGCTGGGCCGTGATCACCGACGAGTACAAGGTGCCCTCAAAGAAGTTCAAGGTCCTGGGCAACACCGATCGGCATTCCATCAAGAAGAATCTCATTGGCGCTCTCCTGTTCGACAGCGGCGAGACGGCTGAGGCTACGCGGCTCAAGCGCACCGCCCGCAGGCGGTACACGCGCAGGAAGAATCGCATCTGCTACCTGCAGGAGATTTTCTCCAACGAGATGGCGAAGGTTGACGATTCTTTCTTCCACAGGCTGGAGGAGTCATTCCTCGTGGAGGAGGATAAGAAGCACGAGCGGCATCCAATCTTCGGCAACATTGTCGACGAGGTTGCCTACCACGAGAAGTACCCTACGATCTACCATCTGCGGAAGAAGCTCGTGGACTCCACAGATAAGGCGGACCTCCGCCTGATCTACCTCGCTCTGGCCCACATGATTAAGTTCAGGGGCCATTTCCTGATCGAGGGGGATCTCAACCCGGACAATAGCGATGTTGACAAGCTGTTCATCCAGCTCGTGCAGACGTACAACCAGCTCTTCGAGGAGAACCCCATTAATGCGTCAGGCGTCGACGCGAAGGCTATCCTGTCCGCTAGGCTCTCGAAGTCTCGGAAGCTCGAGAACCTGATCGCCCAGCTGCCGGGCGAGAAGAAGAACGGCCTGTTCGGGAATCTCATTGCGCTCAGCCTGGGGCTCACGCCCAACTTCAAGTCGAATTTCGATCTCGCTGAGGACGCCAAGCTGCAGCTCTCCAAGGACACATACGACGATGACCTGGATAACCTCCTGGCCCAGATCGGCGATCAGTACGCGGACCTGTTCCTCGCTGCCAAGAATCTGTCGGACGCCATCCTCCTGTCTGATATTCTCAGGGTGAACACCGAGATTACGAAGGCTCCGCTCTCAGCCTCCATGATCAAGCGCTACGACGAGCACCATCAGGATCTGACCCTCCTGAAGGCGCTGGTCAGGCAGCAGCTCCCCGAGAAGTACAAGGAGATCTTCTTCGATCAGTCGAAGAACGGCTACGCTGGGTACATTGACGGCGGGGCCTCTCAGGAGGAGTTCTACAAGTTCATCAAGCCGATTCTGGAGAAGATGGACGGCACGGAGGAGCTGCTGGTGAAGCTCAAGCGCGAGGACCTCCTGAGGAAGCAGCGGACATTCGATAACGGCAGCATCCCACACCAGATTCATCTCGGGGAGCTGCACGCTATCCTGAGGAGGCAGGAGGACTTCTACCCTTTCCTCAAGGATAACCGCGAGAAGATCGAGAAGATTCTGACTTTCAGGATCCCGTACTACGTCGGCCCACTCGCTAGGGGCAACTCCCGCTTCGCTTGGATGACCCGCAAGTCAGAGGAGACGATCACGCCGTGGAACTTCGAGGAGGTGGTCGACAAGGGCGCTAGCGCTCAGTCGTTCATCGAGAGGATGACGAATTTCGACAAGAACCTGCCAAATGAGAAGGTGCTCCCTAAGCACTCGCTCCTGTACGAGTACTTCACAGTCTACAACGAGCTGACTAAGGTGAAGTATGTGACCGAGGGCATGAGGAAGCCGGCTTTCCTGTCTGGGGAGCAGAAGAAGGCCATCGTGGACCTCCTGTTCAAGACCAACCGGAAGGTCACGGTTAAGCAGCTCAAGGAGGACTACTTCAAGAAGATTGAGTGCTTCGATTCGGTCGAGATCTCTGGCGTTGAGGACCGCTTCAACGCCTCCCTGGGGACCTACCACGATCTCCTGAAGATCATTAAGGATAAGGACTTCCTGGACAACGAGGAGAATGAGGATATCCTCGAGGACATTGTGCTGACACTCACTCTGTTCGAGGACCGGGAGATGATCGAGGAGCGCCTGAAGACTTACGCCCATCTCTTCGATGACAAGGTCATGAAGCAGCTCAAGAGGAGGAGGTACACCGGCTGGGGGAGGCTGAGCAGGAAGCTCATCAACGGCATTCGGGACAAGCAGTCCGGGAAGACGATCCTCGACTTCCTGAAGAGCGATGGCTTCGCGAACCGCAATTTCATGCAGCTGATTCACGATGACAGCCTCACATTCAAGGAGGATATCCAGAAGGCTCAGGTGAGCGGCCAGGGGGACTCGCTGCACGAGCATATCGCGAACCTCGCTGGCTCGCCAGCTATCAAGAAGGGGATTCTGCAGACCGTGAAGGTTGTGGACGAGCTGGTGAAGGTCATGGGCAGGCACAAGCCTGAGAACATCGTCATTGAGATGGCCCGGGAGAATCAGACCACGCAGAAGGGCCAGAAGAACTCACGCGAGAGGATGAAGAGGATCGAGGAGGGCATTAAGGAGCTGGGGTCCCAGATCCTCAAGGAGCACCCGGTGGAGAACACGCAGCTGCAGAATGAGAAGCTCTACCTGTACTACCTCCAGAATGGCCGCGATATGTATGTGGACCAGGAGCTGGATATTAACAGGCTCAGCGATTACGACGTCGATGCCATCGTTCCACAGTCATTCCTGAAGGATGACTCCATTGACAACAAGGTCCTCACCAGGTCGGACAAGAACCGGGGCAAGTCTGATAATGTTCCTTCAGAGGAGGTCGTTAAGAAGATGAAGAACTACTGGCGCCAGCTCCTGAATGCCAAGCTGATCACGCAGCGGAAGTTCGATAACCTCACAAAGGCTGAGAGGGGCGGGCTCTCTGAGCTGGACAAGGCGGGCTTCATCAAGAGGCAGCTGGTCGAGACACGGCAGATCACTAAGCACGTTGCGCAGATTCTCGACTCACGGATGAACACTAAGTACGATGAGAATGACAAGCTGATCCGCGAGGTGAAGGTCATCACCCTGAAGTCAAAGCTCGTCTCCGACTTCAGGAAGGATTTCCAGTTCTACAAGGTTCGGGAGATCAACAATTACCACCATGCCCATGACGCGTACCTGAACGCGGTGGTCGGCACAGCTCTGATCAAGAAGTACCCAAAGCTCGAGAGCGAGTTCGTGTACGGGGACTACAAGGTTTACGATGTGAGGAAGATGATCGCCAAGTCGGAGCAGGAGATTGGCAAGGCTACCGCCAAGTACTTCTTCTACTCTAACATTATGAATTTCTTCAAGACAGAGATCACTCTGGCCAATGGCGAGATCCGGAAGCGCCCCCTCATCGAGACGAACGGCGAGACGGGGGAGATCGTGTGGGACAAGGGCAGGGATTTCGCGACCGTCAGGAAGGTTCTCTCCATGCCACAAGTGAATATCGTCAAGAAGACAGAGGTCCAGACTGGCGGGTTCTCTAAGGAGTCAATTCTGCCTAAGCGGAACAGCGACAAGCTCATCGCCCGCAAGAAGGACTGGGATCCGAAGAAGTACGGCGGGTTCGACAGCCCCACTGTGGCCTACTCGGTCCTGGTTGTGGCGAAGGTTGAGAAGGGCAAGTCCAAGAAGCTCAAGAGCGTGAAGGAGCTGCTGGGGATCACGATTATGGAGCGCTCCAGCTTCGAGAAGAACCCGATCGATTTCCTGGAGGCGAAGGGCTACAAGGAGGTGAAGAAGGACCTGATCATTAAGCTCCCCAAGTACTCACTCTTCGAGCTGGAGAACGGCAGGAAGCGGATGCTGGCTTCCGCTGGCGAGCTGCAGAAGGGGAACGAGCTGGCTCTGCCGTCCAAGTATGTGAACTTCCTCTACCTGGCCTCCCACTACGAGAAGCTCAAGGGCAGCCCCGAGGACAACGAGCAGAAGCAGCTGTTCGTCGAGCAGCACAAGCATTACCTCGACGAGATCATTGAGCAGATTTCCGAGTTCTCCAAGCGCGTGATCCTGGCCGACGCGAATCTGGATAAGGTCCTCTCCGCGTACAACAAGCACCGCGACAAGCCAATCAGGGAGCAGGCTGAGAATATCATTCATCTCTTCACCCTGACGAACCTCGGCGCCCCTGCTGCTTTCAAGTACTTCGACACAACTATCGATCGCAAGAGGTACACAAGCACTAAGGAGGTCCTGGACGCGACCCTCATCCACCAGTCGATTACCGGCCTCTACGAGACGCGCATCGACCTGTCTCAGCTCGGGGGCGACGAATTCTCCGGGAGCGAGACGCCAGGCACCTCCGAGTCGGCCACCCCAGAATCTGCCACAGTGGTGTCCGGCCAAAAGCAGGACCGCCAGGGCGGAGAACGCAGAAGGTCCCAGCTCGATAGGGATCAGTGTGCCTACTGCAAGGAGAAGGGCCACTGGGCCAAAGACTGCCCGAAAAAGCCGCGCGGCCCACGCGGCCCAAGGCCACAAACATCCCTCCTTCCAAAGAAGAAGCGGAAGGTGGAGCTCAGCGGAGGATCTTCCGGAGGATCTAGCGGCTCCGAGACACCAGGAACATCCGAAAGCGCTACACCAGAATCTAGCGGAGGCTCTTCCGGAGGATCTAGG GTGGTGGTAGAGCTGAAGGCGGACGCCAGCCCAGTGAGCGTGAAACAATACCCGATGTCGAGGGAAGCAAAGGAAGGCATTAGACCCCACATCCAGAGACTGCTGCAATTGGGAATCCTGGTACCCTGCCAAAGCCCTTGGAACACGCCACTGCTGCCTGTGCGCAAACCAGGAACTAATGATTACAGACCTGTACAGGATCTAAGGGAAGTTAACAAGCGGGTGCAGGACATACATCCTACGGTCCCAAACCCCTACAACTTGCTGAGCTCTCTGCCCCCCGAGCGCACATGGTACACTGTGCTCGACTTAAAAGATGCGTTCTTCTGCCTTCGCCTCCACCCGAACTCACAGCCACTGTTCGCGTTCGAGTGGCGCGACCCTGAGGGCGGCCACACCGGGCAACTGACGTGGACACGGCTGCCACAGGGTTTCAAGAACAGCCCTACTCTATTTGATGAGGCCCTGCACCGTGATCTGGCCCCCTTCCGGGCGCAGAACCCTCAAATCAGTCTACTGCAGTATGTTGATGACCTGCTGCTAGCCGCTTCTACTCAGGAGCTCTGCCGGGAGGGCACAAAGCGGCTGTTAAATGAGCTGGGCGAGCTGGGATACCGCGTGTCTGCCAAGAAGGCACAATTGTGTCGGACAGAGGTGACATATCTCGGCTACACATTAAGAGAAGGTAAGAGATGGCTCACTGAAGCACGTAAGAAGACTGTCATGCAGATTCCTACCCCTACCACGCCCCGTCAGGTGCGGGAGTTCCTCGGCACCGCCGGCTTCTGTAGACTGTGGATTCCTGGATTTGCCACCCTGGCCGCCCCACTCTACCTGCTGACGAAGGAAAAGGTTCCTTTCACCTGGACTGAGGAACATCAGCGGGCTTTCGAAGACATCAAAGCCGCCCTGCTGGCGGCTCCAGCTCTGGCCCTCCCTGACCTCACCAAGCCCTTCACACTCTATGTGGACGAGAGAGCTGGAGTGGCCAGAGGTGTGCTGACACAGGCACTGGGGCCGTGGAAGAGACCCGTGGCGTACCTATCTAAAAAGCTCGACCCCGTGGCCTCTGGGTGGCCGAGCTGTCTAAAGGCCATCGCTGCTGTAGCTCTACTGGTCAAAGATGCTGACAAGCTGACCCTGGGCCAGCATGTCACCGTCATCGCCCCGCACGCCTTAGAATCCATAGTCCGGCAACCACCAGATCGGTGGATGACAAACGCTAGGATGACGCATTATCAGAGCTTGCTGTTAAACGAAAGAGTGACATTTGCGCCCCCTGCCATCCTTAACCCAGCGACGCTACTGCCGGAGATCCACAACTCAACCCCCATCCACCAGTGCGTCGACATTCTGGCTGAGGAGACCGGCACGCGCAAGGACCTCACGGACAGACCATGGCCTGGTGTGCCGGCATGGTACACCGACGGCAGCTCCTTTGTCGTGGAGGGAAAGCGACGCGCAGGCGCGGCTGTGGTGGACGGCAAGCAAGTCATCTGGGCATCCTCCCTGCCAGAAGGCACCAGCGCTCAGAAAGCGGAGTTGGTGGCCTTGACCCAGGCCCTCAGATTAGCTGAAGGGAAGGCTATTAATATCTACACCGATTCTAGATACGCCTTCGCCACGGCGCACATCCACGGCGCGATCTACAAGCAGCGGGGCCTGCTGACCAGTGCCGGGCGAGACATCAAGAATAAGGAAGAGATTCTTGCCCTGCTGGAGGCCGTCCACCTCCCCAAAAAGGTGGCCATTATCCACTGCCCCGGCCACCAGAAGGGAGAGGATCCAATTACAAAGGGGAACCAGATGGCCGACCTCGTGGCGAAGCAGGTCGCTCAGCAGGTTACCATCCTGGCAGAAAAATCGCAGACCCCTGTGAAGACACCTGTGACAGATGATTCTAACTATAGCGGCGGCAGCAAAAGAACGGCGGACGGCTCTGAGAAGCGCACCGCTGATAGCCAGCATTCAACTCCTCCGAAAACAAAGAGGAAAGTTGAGTTCGAACCGAAGAAGAAAAGGAAGGTGTGA

**Sequence 63 Plasmids sequence of PE-RERV-Δ3**

(NLSc-myc-NLSSV40-nCas9(H840A/R221K/N394K)-XTEN-NC-NLS-32aa Linker-RERV-RT(Δ1~64aa)-NLSvbp)

AAGCTTACTGGTTGTACAATGCCGGCGGCCAAGAGAGTCAAGCTCGACGGCGGGAAGCGCACAGCTGATGGTTCTGAATTCGAGTCCCCTAAGAAAAAGAGAAAAGTGGACAAGAAGTACTCGATCGGCCTCGATATTGGGACTAACTCTGTTGGCTGGGCCGTGATCACCGACGAGTACAAGGTGCCCTCAAAGAAGTTCAAGGTCCTGGGCAACACCGATCGGCATTCCATCAAGAAGAATCTCATTGGCGCTCTCCTGTTCGACAGCGGCGAGACGGCTGAGGCTACGCGGCTCAAGCGCACCGCCCGCAGGCGGTACACGCGCAGGAAGAATCGCATCTGCTACCTGCAGGAGATTTTCTCCAACGAGATGGCGAAGGTTGACGATTCTTTCTTCCACAGGCTGGAGGAGTCATTCCTCGTGGAGGAGGATAAGAAGCACGAGCGGCATCCAATCTTCGGCAACATTGTCGACGAGGTTGCCTACCACGAGAAGTACCCTACGATCTACCATCTGCGGAAGAAGCTCGTGGACTCCACAGATAAGGCGGACCTCCGCCTGATCTACCTCGCTCTGGCCCACATGATTAAGTTCAGGGGCCATTTCCTGATCGAGGGGGATCTCAACCCGGACAATAGCGATGTTGACAAGCTGTTCATCCAGCTCGTGCAGACGTACAACCAGCTCTTCGAGGAGAACCCCATTAATGCGTCAGGCGTCGACGCGAAGGCTATCCTGTCCGCTAGGCTCTCGAAGTCTCGGAAGCTCGAGAACCTGATCGCCCAGCTGCCGGGCGAGAAGAAGAACGGCCTGTTCGGGAATCTCATTGCGCTCAGCCTGGGGCTCACGCCCAACTTCAAGTCGAATTTCGATCTCGCTGAGGACGCCAAGCTGCAGCTCTCCAAGGACACATACGACGATGACCTGGATAACCTCCTGGCCCAGATCGGCGATCAGTACGCGGACCTGTTCCTCGCTGCCAAGAATCTGTCGGACGCCATCCTCCTGTCTGATATTCTCAGGGTGAACACCGAGATTACGAAGGCTCCGCTCTCAGCCTCCATGATCAAGCGCTACGACGAGCACCATCAGGATCTGACCCTCCTGAAGGCGCTGGTCAGGCAGCAGCTCCCCGAGAAGTACAAGGAGATCTTCTTCGATCAGTCGAAGAACGGCTACGCTGGGTACATTGACGGCGGGGCCTCTCAGGAGGAGTTCTACAAGTTCATCAAGCCGATTCTGGAGAAGATGGACGGCACGGAGGAGCTGCTGGTGAAGCTCAAGCGCGAGGACCTCCTGAGGAAGCAGCGGACATTCGATAACGGCAGCATCCCACACCAGATTCATCTCGGGGAGCTGCACGCTATCCTGAGGAGGCAGGAGGACTTCTACCCTTTCCTCAAGGATAACCGCGAGAAGATCGAGAAGATTCTGACTTTCAGGATCCCGTACTACGTCGGCCCACTCGCTAGGGGCAACTCCCGCTTCGCTTGGATGACCCGCAAGTCAGAGGAGACGATCACGCCGTGGAACTTCGAGGAGGTGGTCGACAAGGGCGCTAGCGCTCAGTCGTTCATCGAGAGGATGACGAATTTCGACAAGAACCTGCCAAATGAGAAGGTGCTCCCTAAGCACTCGCTCCTGTACGAGTACTTCACAGTCTACAACGAGCTGACTAAGGTGAAGTATGTGACCGAGGGCATGAGGAAGCCGGCTTTCCTGTCTGGGGAGCAGAAGAAGGCCATCGTGGACCTCCTGTTCAAGACCAACCGGAAGGTCACGGTTAAGCAGCTCAAGGAGGACTACTTCAAGAAGATTGAGTGCTTCGATTCGGTCGAGATCTCTGGCGTTGAGGACCGCTTCAACGCCTCCCTGGGGACCTACCACGATCTCCTGAAGATCATTAAGGATAAGGACTTCCTGGACAACGAGGAGAATGAGGATATCCTCGAGGACATTGTGCTGACACTCACTCTGTTCGAGGACCGGGAGATGATCGAGGAGCGCCTGAAGACTTACGCCCATCTCTTCGATGACAAGGTCATGAAGCAGCTCAAGAGGAGGAGGTACACCGGCTGGGGGAGGCTGAGCAGGAAGCTCATCAACGGCATTCGGGACAAGCAGTCCGGGAAGACGATCCTCGACTTCCTGAAGAGCGATGGCTTCGCGAACCGCAATTTCATGCAGCTGATTCACGATGACAGCCTCACATTCAAGGAGGATATCCAGAAGGCTCAGGTGAGCGGCCAGGGGGACTCGCTGCACGAGCATATCGCGAACCTCGCTGGCTCGCCAGCTATCAAGAAGGGGATTCTGCAGACCGTGAAGGTTGTGGACGAGCTGGTGAAGGTCATGGGCAGGCACAAGCCTGAGAACATCGTCATTGAGATGGCCCGGGAGAATCAGACCACGCAGAAGGGCCAGAAGAACTCACGCGAGAGGATGAAGAGGATCGAGGAGGGCATTAAGGAGCTGGGGTCCCAGATCCTCAAGGAGCACCCGGTGGAGAACACGCAGCTGCAGAATGAGAAGCTCTACCTGTACTACCTCCAGAATGGCCGCGATATGTATGTGGACCAGGAGCTGGATATTAACAGGCTCAGCGATTACGACGTCGATGCCATCGTTCCACAGTCATTCCTGAAGGATGACTCCATTGACAACAAGGTCCTCACCAGGTCGGACAAGAACCGGGGCAAGTCTGATAATGTTCCTTCAGAGGAGGTCGTTAAGAAGATGAAGAACTACTGGCGCCAGCTCCTGAATGCCAAGCTGATCACGCAGCGGAAGTTCGATAACCTCACAAAGGCTGAGAGGGGCGGGCTCTCTGAGCTGGACAAGGCGGGCTTCATCAAGAGGCAGCTGGTCGAGACACGGCAGATCACTAAGCACGTTGCGCAGATTCTCGACTCACGGATGAACACTAAGTACGATGAGAATGACAAGCTGATCCGCGAGGTGAAGGTCATCACCCTGAAGTCAAAGCTCGTCTCCGACTTCAGGAAGGATTTCCAGTTCTACAAGGTTCGGGAGATCAACAATTACCACCATGCCCATGACGCGTACCTGAACGCGGTGGTCGGCACAGCTCTGATCAAGAAGTACCCAAAGCTCGAGAGCGAGTTCGTGTACGGGGACTACAAGGTTTACGATGTGAGGAAGATGATCGCCAAGTCGGAGCAGGAGATTGGCAAGGCTACCGCCAAGTACTTCTTCTACTCTAACATTATGAATTTCTTCAAGACAGAGATCACTCTGGCCAATGGCGAGATCCGGAAGCGCCCCCTCATCGAGACGAACGGCGAGACGGGGGAGATCGTGTGGGACAAGGGCAGGGATTTCGCGACCGTCAGGAAGGTTCTCTCCATGCCACAAGTGAATATCGTCAAGAAGACAGAGGTCCAGACTGGCGGGTTCTCTAAGGAGTCAATTCTGCCTAAGCGGAACAGCGACAAGCTCATCGCCCGCAAGAAGGACTGGGATCCGAAGAAGTACGGCGGGTTCGACAGCCCCACTGTGGCCTACTCGGTCCTGGTTGTGGCGAAGGTTGAGAAGGGCAAGTCCAAGAAGCTCAAGAGCGTGAAGGAGCTGCTGGGGATCACGATTATGGAGCGCTCCAGCTTCGAGAAGAACCCGATCGATTTCCTGGAGGCGAAGGGCTACAAGGAGGTGAAGAAGGACCTGATCATTAAGCTCCCCAAGTACTCACTCTTCGAGCTGGAGAACGGCAGGAAGCGGATGCTGGCTTCCGCTGGCGAGCTGCAGAAGGGGAACGAGCTGGCTCTGCCGTCCAAGTATGTGAACTTCCTCTACCTGGCCTCCCACTACGAGAAGCTCAAGGGCAGCCCCGAGGACAACGAGCAGAAGCAGCTGTTCGTCGAGCAGCACAAGCATTACCTCGACGAGATCATTGAGCAGATTTCCGAGTTCTCCAAGCGCGTGATCCTGGCCGACGCGAATCTGGATAAGGTCCTCTCCGCGTACAACAAGCACCGCGACAAGCCAATCAGGGAGCAGGCTGAGAATATCATTCATCTCTTCACCCTGACGAACCTCGGCGCCCCTGCTGCTTTCAAGTACTTCGACACAACTATCGATCGCAAGAGGTACACAAGCACTAAGGAGGTCCTGGACGCGACCCTCATCCACCAGTCGATTACCGGCCTCTACGAGACGCGCATCGACCTGTCTCAGCTCGGGGGCGACGAATTCTCCGGGAGCGAGACGCCAGGCACCTCCGAGTCGGCCACCCCAGAATCTGCCACAGTGGTGTCCGGCCAAAAGCAGGACCGCCAGGGCGGAGAACGCAGAAGGTCCCAGCTCGATAGGGATCAGTGTGCCTACTGCAAGGAGAAGGGCCACTGGGCCAAAGACTGCCCGAAAAAGCCGCGCGGCCCACGCGGCCCAAGGCCACAAACATCCCTCCTTCCAAAGAAGAAGCGGAAGGTGGAGCTCAGCGGAGGATCTTCCGGAGGATCTAGCGGCTCCGAGACACCAGGAACATCCGAAAGCGCTACACCAGAATCTAGCGGAGGCTCTTCCGGAGGATCTAGG GTGAAACAATACCCGATGTCGAGGGAAGCAAAGGAAGGCATTAGACCCCACATCCAGAGACTGCTGCAATTGGGAATCCTGGTACCCTGCCAAAGCCCTTGGAACACGCCACTGCTGCCTGTGCGCAAACCAGGAACTAATGATTACAGACCTGTACAGGATCTAAGGGAAGTTAACAAGCGGGTGCAGGACATACATCCTACGGTCCCAAACCCCTACAACTTGCTGAGCTCTCTGCCCCCCGAGCGCACATGGTACACTGTGCTCGACTTAAAAGATGCGTTCTTCTGCCTTCGCCTCCACCCGAACTCACAGCCACTGTTCGCGTTCGAGTGGCGCGACCCTGAGGGCGGCCACACCGGGCAACTGACGTGGACACGGCTGCCACAGGGTTTCAAGAACAGCCCTACTCTATTTGATGAGGCCCTGCACCGTGATCTGGCCCCCTTCCGGGCGCAGAACCCTCAAATCAGTCTACTGCAGTATGTTGATGACCTGCTGCTAGCCGCTTCTACTCAGGAGCTCTGCCGGGAGGGCACAAAGCGGCTGTTAAATGAGCTGGGCGAGCTGGGATACCGCGTGTCTGCCAAGAAGGCACAATTGTGTCGGACAGAGGTGACATATCTCGGCTACACATTAAGAGAAGGTAAGAGATGGCTCACTGAAGCACGTAAGAAGACTGTCATGCAGATTCCTACCCCTACCACGCCCCGTCAGGTGCGGGAGTTCCTCGGCACCGCCGGCTTCTGTAGACTGTGGATTCCTGGATTTGCCACCCTGGCCGCCCCACTCTACCTGCTGACGAAGGAAAAGGTTCCTTTCACCTGGACTGAGGAACATCAGCGGGCTTTCGAAGACATCAAAGCCGCCCTGCTGGCGGCTCCAGCTCTGGCCCTCCCTGACCTCACCAAGCCCTTCACACTCTATGTGGACGAGAGAGCTGGAGTGGCCAGAGGTGTGCTGACACAGGCACTGGGGCCGTGGAAGAGACCCGTGGCGTACCTATCTAAAAAGCTCGACCCCGTGGCCTCTGGGTGGCCGAGCTGTCTAAAGGCCATCGCTGCTGTAGCTCTACTGGTCAAAGATGCTGACAAGCTGACCCTGGGCCAGCATGTCACCGTCATCGCCCCGCACGCCTTAGAATCCATAGTCCGGCAACCACCAGATCGGTGGATGACAAACGCTAGGATGACGCATTATCAGAGCTTGCTGTTAAACGAAAGAGTGACATTTGCGCCCCCTGCCATCCTTAACCCAGCGACGCTACTGCCGGAGATCCACAACTCAACCCCCATCCACCAGTGCGTCGACATTCTGGCTGAGGAGACCGGCACGCGCAAGGACCTCACGGACAGACCATGGCCTGGTGTGCCGGCATGGTACACCGACGGCAGCTCCTTTGTCGTGGAGGGAAAGCGACGCGCAGGCGCGGCTGTGGTGGACGGCAAGCAAGTCATCTGGGCATCCTCCCTGCCAGAAGGCACCAGCGCTCAGAAAGCGGAGTTGGTGGCCTTGACCCAGGCCCTCAGATTAGCTGAAGGGAAGGCTATTAATATCTACACCGATTCTAGATACGCCTTCGCCACGGCGCACATCCACGGCGCGATCTACAAGCAGCGGGGCCTGCTGACCAGTGCCGGGCGAGACATCAAGAATAAGGAAGAGATTCTTGCCCTGCTGGAGGCCGTCCACCTCCCCAAAAAGGTGGCCATTATCCACTGCCCCGGCCACCAGAAGGGAGAGGATCCAATTACAAAGGGGAACCAGATGGCCGACCTCGTGGCGAAGCAGGTCGCTCAGCAGGTTACCATCCTGGCAGAAAAATCGCAGACCCCTGTGAAGACACCTGTGACAGATGATTCTAACTATAGCGGCGGCAGCAAAAGAACGGCGGACGGCTCTGAGAAGCGCACCGCTGATAGCCAGCATTCAACTCCTCCGAAAACAAAGAGGAAAGTTGAGTTCGAACCGAAGAAGAAAAGGAAGGTGTGA

**Sequence 64 Plasmids sequence of PE-RERV-Δ4**

(NLSc-myc-NLSSV40-nCas9(H840A/R221K/N394K)-XTEN-NC-NLS-32aa Linker-RERV-RT(Δ1~89aa)-NLSvbp)

AAGCTTACTGGTTGTACAATGCCGGCGGCCAAGAGAGTCAAGCTCGACGGCGGGAAGCGCACAGCTGATGGTTCTGAATTCGAGTCCCCTAAGAAAAAGAGAAAAGTGGACAAGAAGTACTCGATCGGCCTCGATATTGGGACTAACTCTGTTGGCTGGGCCGTGATCACCGACGAGTACAAGGTGCCCTCAAAGAAGTTCAAGGTCCTGGGCAACACCGATCGGCATTCCATCAAGAAGAATCTCATTGGCGCTCTCCTGTTCGACAGCGGCGAGACGGCTGAGGCTACGCGGCTCAAGCGCACCGCCCGCAGGCGGTACACGCGCAGGAAGAATCGCATCTGCTACCTGCAGGAGATTTTCTCCAACGAGATGGCGAAGGTTGACGATTCTTTCTTCCACAGGCTGGAGGAGTCATTCCTCGTGGAGGAGGATAAGAAGCACGAGCGGCATCCAATCTTCGGCAACATTGTCGACGAGGTTGCCTACCACGAGAAGTACCCTACGATCTACCATCTGCGGAAGAAGCTCGTGGACTCCACAGATAAGGCGGACCTCCGCCTGATCTACCTCGCTCTGGCCCACATGATTAAGTTCAGGGGCCATTTCCTGATCGAGGGGGATCTCAACCCGGACAATAGCGATGTTGACAAGCTGTTCATCCAGCTCGTGCAGACGTACAACCAGCTCTTCGAGGAGAACCCCATTAATGCGTCAGGCGTCGACGCGAAGGCTATCCTGTCCGCTAGGCTCTCGAAGTCTCGGAAGCTCGAGAACCTGATCGCCCAGCTGCCGGGCGAGAAGAAGAACGGCCTGTTCGGGAATCTCATTGCGCTCAGCCTGGGGCTCACGCCCAACTTCAAGTCGAATTTCGATCTCGCTGAGGACGCCAAGCTGCAGCTCTCCAAGGACACATACGACGATGACCTGGATAACCTCCTGGCCCAGATCGGCGATCAGTACGCGGACCTGTTCCTCGCTGCCAAGAATCTGTCGGACGCCATCCTCCTGTCTGATATTCTCAGGGTGAACACCGAGATTACGAAGGCTCCGCTCTCAGCCTCCATGATCAAGCGCTACGACGAGCACCATCAGGATCTGACCCTCCTGAAGGCGCTGGTCAGGCAGCAGCTCCCCGAGAAGTACAAGGAGATCTTCTTCGATCAGTCGAAGAACGGCTACGCTGGGTACATTGACGGCGGGGCCTCTCAGGAGGAGTTCTACAAGTTCATCAAGCCGATTCTGGAGAAGATGGACGGCACGGAGGAGCTGCTGGTGAAGCTCAAGCGCGAGGACCTCCTGAGGAAGCAGCGGACATTCGATAACGGCAGCATCCCACACCAGATTCATCTCGGGGAGCTGCACGCTATCCTGAGGAGGCAGGAGGACTTCTACCCTTTCCTCAAGGATAACCGCGAGAAGATCGAGAAGATTCTGACTTTCAGGATCCCGTACTACGTCGGCCCACTCGCTAGGGGCAACTCCCGCTTCGCTTGGATGACCCGCAAGTCAGAGGAGACGATCACGCCGTGGAACTTCGAGGAGGTGGTCGACAAGGGCGCTAGCGCTCAGTCGTTCATCGAGAGGATGACGAATTTCGACAAGAACCTGCCAAATGAGAAGGTGCTCCCTAAGCACTCGCTCCTGTACGAGTACTTCACAGTCTACAACGAGCTGACTAAGGTGAAGTATGTGACCGAGGGCATGAGGAAGCCGGCTTTCCTGTCTGGGGAGCAGAAGAAGGCCATCGTGGACCTCCTGTTCAAGACCAACCGGAAGGTCACGGTTAAGCAGCTCAAGGAGGACTACTTCAAGAAGATTGAGTGCTTCGATTCGGTCGAGATCTCTGGCGTTGAGGACCGCTTCAACGCCTCCCTGGGGACCTACCACGATCTCCTGAAGATCATTAAGGATAAGGACTTCCTGGACAACGAGGAGAATGAGGATATCCTCGAGGACATTGTGCTGACACTCACTCTGTTCGAGGACCGGGAGATGATCGAGGAGCGCCTGAAGACTTACGCCCATCTCTTCGATGACAAGGTCATGAAGCAGCTCAAGAGGAGGAGGTACACCGGCTGGGGGAGGCTGAGCAGGAAGCTCATCAACGGCATTCGGGACAAGCAGTCCGGGAAGACGATCCTCGACTTCCTGAAGAGCGATGGCTTCGCGAACCGCAATTTCATGCAGCTGATTCACGATGACAGCCTCACATTCAAGGAGGATATCCAGAAGGCTCAGGTGAGCGGCCAGGGGGACTCGCTGCACGAGCATATCGCGAACCTCGCTGGCTCGCCAGCTATCAAGAAGGGGATTCTGCAGACCGTGAAGGTTGTGGACGAGCTGGTGAAGGTCATGGGCAGGCACAAGCCTGAGAACATCGTCATTGAGATGGCCCGGGAGAATCAGACCACGCAGAAGGGCCAGAAGAACTCACGCGAGAGGATGAAGAGGATCGAGGAGGGCATTAAGGAGCTGGGGTCCCAGATCCTCAAGGAGCACCCGGTGGAGAACACGCAGCTGCAGAATGAGAAGCTCTACCTGTACTACCTCCAGAATGGCCGCGATATGTATGTGGACCAGGAGCTGGATATTAACAGGCTCAGCGATTACGACGTCGATGCCATCGTTCCACAGTCATTCCTGAAGGATGACTCCATTGACAACAAGGTCCTCACCAGGTCGGACAAGAACCGGGGCAAGTCTGATAATGTTCCTTCAGAGGAGGTCGTTAAGAAGATGAAGAACTACTGGCGCCAGCTCCTGAATGCCAAGCTGATCACGCAGCGGAAGTTCGATAACCTCACAAAGGCTGAGAGGGGCGGGCTCTCTGAGCTGGACAAGGCGGGCTTCATCAAGAGGCAGCTGGTCGAGACACGGCAGATCACTAAGCACGTTGCGCAGATTCTCGACTCACGGATGAACACTAAGTACGATGAGAATGACAAGCTGATCCGCGAGGTGAAGGTCATCACCCTGAAGTCAAAGCTCGTCTCCGACTTCAGGAAGGATTTCCAGTTCTACAAGGTTCGGGAGATCAACAATTACCACCATGCCCATGACGCGTACCTGAACGCGGTGGTCGGCACAGCTCTGATCAAGAAGTACCCAAAGCTCGAGAGCGAGTTCGTGTACGGGGACTACAAGGTTTACGATGTGAGGAAGATGATCGCCAAGTCGGAGCAGGAGATTGGCAAGGCTACCGCCAAGTACTTCTTCTACTCTAACATTATGAATTTCTTCAAGACAGAGATCACTCTGGCCAATGGCGAGATCCGGAAGCGCCCCCTCATCGAGACGAACGGCGAGACGGGGGAGATCGTGTGGGACAAGGGCAGGGATTTCGCGACCGTCAGGAAGGTTCTCTCCATGCCACAAGTGAATATCGTCAAGAAGACAGAGGTCCAGACTGGCGGGTTCTCTAAGGAGTCAATTCTGCCTAAGCGGAACAGCGACAAGCTCATCGCCCGCAAGAAGGACTGGGATCCGAAGAAGTACGGCGGGTTCGACAGCCCCACTGTGGCCTACTCGGTCCTGGTTGTGGCGAAGGTTGAGAAGGGCAAGTCCAAGAAGCTCAAGAGCGTGAAGGAGCTGCTGGGGATCACGATTATGGAGCGCTCCAGCTTCGAGAAGAACCCGATCGATTTCCTGGAGGCGAAGGGCTACAAGGAGGTGAAGAAGGACCTGATCATTAAGCTCCCCAAGTACTCACTCTTCGAGCTGGAGAACGGCAGGAAGCGGATGCTGGCTTCCGCTGGCGAGCTGCAGAAGGGGAACGAGCTGGCTCTGCCGTCCAAGTATGTGAACTTCCTCTACCTGGCCTCCCACTACGAGAAGCTCAAGGGCAGCCCCGAGGACAACGAGCAGAAGCAGCTGTTCGTCGAGCAGCACAAGCATTACCTCGACGAGATCATTGAGCAGATTTCCGAGTTCTCCAAGCGCGTGATCCTGGCCGACGCGAATCTGGATAAGGTCCTCTCCGCGTACAACAAGCACCGCGACAAGCCAATCAGGGAGCAGGCTGAGAATATCATTCATCTCTTCACCCTGACGAACCTCGGCGCCCCTGCTGCTTTCAAGTACTTCGACACAACTATCGATCGCAAGAGGTACACAAGCACTAAGGAGGTCCTGGACGCGACCCTCATCCACCAGTCGATTACCGGCCTCTACGAGACGCGCATCGACCTGTCTCAGCTCGGGGGCGACGAATTCTCCGGGAGCGAGACGCCAGGCACCTCCGAGTCGGCCACCCCAGAATCTGCCACAGTGGTGTCCGGCCAAAAGCAGGACCGCCAGGGCGGAGAACGCAGAAGGTCCCAGCTCGATAGGGATCAGTGTGCCTACTGCAAGGAGAAGGGCCACTGGGCCAAAGACTGCCCGAAAAAGCCGCGCGGCCCACGCGGCCCAAGGCCACAAACATCCCTCCTTCCAAAGAAGAAGCGGAAGGTGGAGCTCAGCGGAGGATCTTCCGGAGGATCTAGCGGCTCCGAGACACCAGGAACATCCGAAAGCGCTACACCAGAATCTAGCGGAGGCTCTTCCGGAGGATCTAGG ATCCTGGTACCCTGCCAAAGCCCTTGGAACACGCCACTGCTGCCTGTGCGCAAACCAGGAACTAATGATTACAGACCTGTACAGGATCTAAGGGAAGTTAACAAGCGGGTGCAGGACATACATCCTACGGTCCCAAACCCCTACAACTTGCTGAGCTCTCTGCCCCCCGAGCGCACATGGTACACTGTGCTCGACTTAAAAGATGCGTTCTTCTGCCTTCGCCTCCACCCGAACTCACAGCCACTGTTCGCGTTCGAGTGGCGCGACCCTGAGGGCGGCCACACCGGGCAACTGACGTGGACACGGCTGCCACAGGGTTTCAAGAACAGCCCTACTCTATTTGATGAGGCCCTGCACCGTGATCTGGCCCCCTTCCGGGCGCAGAACCCTCAAATCAGTCTACTGCAGTATGTTGATGACCTGCTGCTAGCCGCTTCTACTCAGGAGCTCTGCCGGGAGGGCACAAAGCGGCTGTTAAATGAGCTGGGCGAGCTGGGATACCGCGTGTCTGCCAAGAAGGCACAATTGTGTCGGACAGAGGTGACATATCTCGGCTACACATTAAGAGAAGGTAAGAGATGGCTCACTGAAGCACGTAAGAAGACTGTCATGCAGATTCCTACCCCTACCACGCCCCGTCAGGTGCGGGAGTTCCTCGGCACCGCCGGCTTCTGTAGACTGTGGATTCCTGGATTTGCCACCCTGGCCGCCCCACTCTACCTGCTGACGAAGGAAAAGGTTCCTTTCACCTGGACTGAGGAACATCAGCGGGCTTTCGAAGACATCAAAGCCGCCCTGCTGGCGGCTCCAGCTCTGGCCCTCCCTGACCTCACCAAGCCCTTCACACTCTATGTGGACGAGAGAGCTGGAGTGGCCAGAGGTGTGCTGACACAGGCACTGGGGCCGTGGAAGAGACCCGTGGCGTACCTATCTAAAAAGCTCGACCCCGTGGCCTCTGGGTGGCCGAGCTGTCTAAAGGCCATCGCTGCTGTAGCTCTACTGGTCAAAGATGCTGACAAGCTGACCCTGGGCCAGCATGTCACCGTCATCGCCCCGCACGCCTTAGAATCCATAGTCCGGCAACCACCAGATCGGTGGATGACAAACGCTAGGATGACGCATTATCAGAGCTTGCTGTTAAACGAAAGAGTGACATTTGCGCCCCCTGCCATCCTTAACCCAGCGACGCTACTGCCGGAGATCCACAACTCAACCCCCATCCACCAGTGCGTCGACATTCTGGCTGAGGAGACCGGCACGCGCAAGGACCTCACGGACAGACCATGGCCTGGTGTGCCGGCATGGTACACCGACGGCAGCTCCTTTGTCGTGGAGGGAAAGCGACGCGCAGGCGCGGCTGTGGTGGACGGCAAGCAAGTCATCTGGGCATCCTCCCTGCCAGAAGGCACCAGCGCTCAGAAAGCGGAGTTGGTGGCCTTGACCCAGGCCCTCAGATTAGCTGAAGGGAAGGCTATTAATATCTACACCGATTCTAGATACGCCTTCGCCACGGCGCACATCCACGGCGCGATCTACAAGCAGCGGGGCCTGCTGACCAGTGCCGGGCGAGACATCAAGAATAAGGAAGAGATTCTTGCCCTGCTGGAGGCCGTCCACCTCCCCAAAAAGGTGGCCATTATCCACTGCCCCGGCCACCAGAAGGGAGAGGATCCAATTACAAAGGGGAACCAGATGGCCGACCTCGTGGCGAAGCAGGTCGCTCAGCAGGTTACCATCCTGGCAGAAAAATCGCAGACCCCTGTGAAGACACCTGTGACAGATGATTCTAACTATAGCGGCGGCAGCAAAAGAACGGCGGACGGCTCTGAGAAGCGCACCGCTGATAGCCAGCATTCAACTCCTCCGAAAACAAAGAGGAAAGTTGAGTTCGAACCGAAGAAGAAAAGGAAGGTGTGA

**Sequence 65 Plasmids sequence of PE-RERV-Δ5**

(NLSc-myc-NLSSV40-nCas9(H840A/R221K/N394K)-XTEN-NC-NLS-32aa Linker-RERV-RT(Δ644~688aa)-NLSvbp)

AAGCTTACTGGTTGTACAATGCCGGCGGCCAAGAGAGTCAAGCTCGACGGCGGGAAGCGCACAGCTGATGGTTCTGAATTCGAGTCCCCTAAGAAAAAGAGAAAAGTGGACAAGAAGTACTCGATCGGCCTCGATATTGGGACTAACTCTGTTGGCTGGGCCGTGATCACCGACGAGTACAAGGTGCCCTCAAAGAAGTTCAAGGTCCTGGGCAACACCGATCGGCATTCCATCAAGAAGAATCTCATTGGCGCTCTCCTGTTCGACAGCGGCGAGACGGCTGAGGCTACGCGGCTCAAGCGCACCGCCCGCAGGCGGTACACGCGCAGGAAGAATCGCATCTGCTACCTGCAGGAGATTTTCTCCAACGAGATGGCGAAGGTTGACGATTCTTTCTTCCACAGGCTGGAGGAGTCATTCCTCGTGGAGGAGGATAAGAAGCACGAGCGGCATCCAATCTTCGGCAACATTGTCGACGAGGTTGCCTACCACGAGAAGTACCCTACGATCTACCATCTGCGGAAGAAGCTCGTGGACTCCACAGATAAGGCGGACCTCCGCCTGATCTACCTCGCTCTGGCCCACATGATTAAGTTCAGGGGCCATTTCCTGATCGAGGGGGATCTCAACCCGGACAATAGCGATGTTGACAAGCTGTTCATCCAGCTCGTGCAGACGTACAACCAGCTCTTCGAGGAGAACCCCATTAATGCGTCAGGCGTCGACGCGAAGGCTATCCTGTCCGCTAGGCTCTCGAAGTCTCGGAAGCTCGAGAACCTGATCGCCCAGCTGCCGGGCGAGAAGAAGAACGGCCTGTTCGGGAATCTCATTGCGCTCAGCCTGGGGCTCACGCCCAACTTCAAGTCGAATTTCGATCTCGCTGAGGACGCCAAGCTGCAGCTCTCCAAGGACACATACGACGATGACCTGGATAACCTCCTGGCCCAGATCGGCGATCAGTACGCGGACCTGTTCCTCGCTGCCAAGAATCTGTCGGACGCCATCCTCCTGTCTGATATTCTCAGGGTGAACACCGAGATTACGAAGGCTCCGCTCTCAGCCTCCATGATCAAGCGCTACGACGAGCACCATCAGGATCTGACCCTCCTGAAGGCGCTGGTCAGGCAGCAGCTCCCCGAGAAGTACAAGGAGATCTTCTTCGATCAGTCGAAGAACGGCTACGCTGGGTACATTGACGGCGGGGCCTCTCAGGAGGAGTTCTACAAGTTCATCAAGCCGATTCTGGAGAAGATGGACGGCACGGAGGAGCTGCTGGTGAAGCTCAAGCGCGAGGACCTCCTGAGGAAGCAGCGGACATTCGATAACGGCAGCATCCCACACCAGATTCATCTCGGGGAGCTGCACGCTATCCTGAGGAGGCAGGAGGACTTCTACCCTTTCCTCAAGGATAACCGCGAGAAGATCGAGAAGATTCTGACTTTCAGGATCCCGTACTACGTCGGCCCACTCGCTAGGGGCAACTCCCGCTTCGCTTGGATGACCCGCAAGTCAGAGGAGACGATCACGCCGTGGAACTTCGAGGAGGTGGTCGACAAGGGCGCTAGCGCTCAGTCGTTCATCGAGAGGATGACGAATTTCGACAAGAACCTGCCAAATGAGAAGGTGCTCCCTAAGCACTCGCTCCTGTACGAGTACTTCACAGTCTACAACGAGCTGACTAAGGTGAAGTATGTGACCGAGGGCATGAGGAAGCCGGCTTTCCTGTCTGGGGAGCAGAAGAAGGCCATCGTGGACCTCCTGTTCAAGACCAACCGGAAGGTCACGGTTAAGCAGCTCAAGGAGGACTACTTCAAGAAGATTGAGTGCTTCGATTCGGTCGAGATCTCTGGCGTTGAGGACCGCTTCAACGCCTCCCTGGGGACCTACCACGATCTCCTGAAGATCATTAAGGATAAGGACTTCCTGGACAACGAGGAGAATGAGGATATCCTCGAGGACATTGTGCTGACACTCACTCTGTTCGAGGACCGGGAGATGATCGAGGAGCGCCTGAAGACTTACGCCCATCTCTTCGATGACAAGGTCATGAAGCAGCTCAAGAGGAGGAGGTACACCGGCTGGGGGAGGCTGAGCAGGAAGCTCATCAACGGCATTCGGGACAAGCAGTCCGGGAAGACGATCCTCGACTTCCTGAAGAGCGATGGCTTCGCGAACCGCAATTTCATGCAGCTGATTCACGATGACAGCCTCACATTCAAGGAGGATATCCAGAAGGCTCAGGTGAGCGGCCAGGGGGACTCGCTGCACGAGCATATCGCGAACCTCGCTGGCTCGCCAGCTATCAAGAAGGGGATTCTGCAGACCGTGAAGGTTGTGGACGAGCTGGTGAAGGTCATGGGCAGGCACAAGCCTGAGAACATCGTCATTGAGATGGCCCGGGAGAATCAGACCACGCAGAAGGGCCAGAAGAACTCACGCGAGAGGATGAAGAGGATCGAGGAGGGCATTAAGGAGCTGGGGTCCCAGATCCTCAAGGAGCACCCGGTGGAGAACACGCAGCTGCAGAATGAGAAGCTCTACCTGTACTACCTCCAGAATGGCCGCGATATGTATGTGGACCAGGAGCTGGATATTAACAGGCTCAGCGATTACGACGTCGATGCCATCGTTCCACAGTCATTCCTGAAGGATGACTCCATTGACAACAAGGTCCTCACCAGGTCGGACAAGAACCGGGGCAAGTCTGATAATGTTCCTTCAGAGGAGGTCGTTAAGAAGATGAAGAACTACTGGCGCCAGCTCCTGAATGCCAAGCTGATCACGCAGCGGAAGTTCGATAACCTCACAAAGGCTGAGAGGGGCGGGCTCTCTGAGCTGGACAAGGCGGGCTTCATCAAGAGGCAGCTGGTCGAGACACGGCAGATCACTAAGCACGTTGCGCAGATTCTCGACTCACGGATGAACACTAAGTACGATGAGAATGACAAGCTGATCCGCGAGGTGAAGGTCATCACCCTGAAGTCAAAGCTCGTCTCCGACTTCAGGAAGGATTTCCAGTTCTACAAGGTTCGGGAGATCAACAATTACCACCATGCCCATGACGCGTACCTGAACGCGGTGGTCGGCACAGCTCTGATCAAGAAGTACCCAAAGCTCGAGAGCGAGTTCGTGTACGGGGACTACAAGGTTTACGATGTGAGGAAGATGATCGCCAAGTCGGAGCAGGAGATTGGCAAGGCTACCGCCAAGTACTTCTTCTACTCTAACATTATGAATTTCTTCAAGACAGAGATCACTCTGGCCAATGGCGAGATCCGGAAGCGCCCCCTCATCGAGACGAACGGCGAGACGGGGGAGATCGTGTGGGACAAGGGCAGGGATTTCGCGACCGTCAGGAAGGTTCTCTCCATGCCACAAGTGAATATCGTCAAGAAGACAGAGGTCCAGACTGGCGGGTTCTCTAAGGAGTCAATTCTGCCTAAGCGGAACAGCGACAAGCTCATCGCCCGCAAGAAGGACTGGGATCCGAAGAAGTACGGCGGGTTCGACAGCCCCACTGTGGCCTACTCGGTCCTGGTTGTGGCGAAGGTTGAGAAGGGCAAGTCCAAGAAGCTCAAGAGCGTGAAGGAGCTGCTGGGGATCACGATTATGGAGCGCTCCAGCTTCGAGAAGAACCCGATCGATTTCCTGGAGGCGAAGGGCTACAAGGAGGTGAAGAAGGACCTGATCATTAAGCTCCCCAAGTACTCACTCTTCGAGCTGGAGAACGGCAGGAAGCGGATGCTGGCTTCCGCTGGCGAGCTGCAGAAGGGGAACGAGCTGGCTCTGCCGTCCAAGTATGTGAACTTCCTCTACCTGGCCTCCCACTACGAGAAGCTCAAGGGCAGCCCCGAGGACAACGAGCAGAAGCAGCTGTTCGTCGAGCAGCACAAGCATTACCTCGACGAGATCATTGAGCAGATTTCCGAGTTCTCCAAGCGCGTGATCCTGGCCGACGCGAATCTGGATAAGGTCCTCTCCGCGTACAACAAGCACCGCGACAAGCCAATCAGGGAGCAGGCTGAGAATATCATTCATCTCTTCACCCTGACGAACCTCGGCGCCCCTGCTGCTTTCAAGTACTTCGACACAACTATCGATCGCAAGAGGTACACAAGCACTAAGGAGGTCCTGGACGCGACCCTCATCCACCAGTCGATTACCGGCCTCTACGAGACGCGCATCGACCTGTCTCAGCTCGGGGGCGACGAATTCTCCGGGAGCGAGACGCCAGGCACCTCCGAGTCGGCCACCCCAGAATCTGCCACAGTGGTGTCCGGCCAAAAGCAGGACCGCCAGGGCGGAGAACGCAGAAGGTCCCAGCTCGATAGGGATCAGTGTGCCTACTGCAAGGAGAAGGGCCACTGGGCCAAAGACTGCCCGAAAAAGCCGCGCGGCCCACGCGGCCCAAGGCCACAAACATCCCTCCTTCCAAAGAAGAAGCGGAAGGTGGAGCTCAGCGGAGGATCTTCCGGAGGATCTAGCGGCTCCGAGACACCAGGAACATCCGAAAGCGCTACACCAGAATCTAGCGGAGGCTCTTCCGGAGGATCTAGG CCTCCCGTCGCGTGCCTCGTACTGAGCCTAGAGGAGGAGTACAGGCTGCATGAGCAAAATCCGAAGCAGCTGCTAGCCCCTGAATGGCTGAGCGCCTTCTCCGAGGTATGGGCGGAGCAGACTGGCATGGGACTGGCCAAGCAGGTGCCTCCTGTGGTGGTAGAGCTGAAGGCGGACGCCAGCCCAGTGAGCGTGAAACAATACCCGATGTCGAGGGAAGCAAAGGAAGGCATTAGACCCCACATCCAGAGACTGCTGCAATTGGGAATCCTGGTACCCTGCCAAAGCCCTTGGAACACGCCACTGCTGCCTGTGCGCAAACCAGGAACTAATGATTACAGACCTGTACAGGATCTAAGGGAAGTTAACAAGCGGGTGCAGGACATACATCCTACGGTCCCAAACCCCTACAACTTGCTGAGCTCTCTGCCCCCCGAGCGCACATGGTACACTGTGCTCGACTTAAAAGATGCGTTCTTCTGCCTTCGCCTCCACCCGAACTCACAGCCACTGTTCGCGTTCGAGTGGCGCGACCCTGAGGGCGGCCACACCGGGCAACTGACGTGGACACGGCTGCCACAGGGTTTCAAGAACAGCCCTACTCTATTTGATGAGGCCCTGCACCGTGATCTGGCCCCCTTCCGGGCGCAGAACCCTCAAATCAGTCTACTGCAGTATGTTGATGACCTGCTGCTAGCCGCTTCTACTCAGGAGCTCTGCCGGGAGGGCACAAAGCGGCTGTTAAATGAGCTGGGCGAGCTGGGATACCGCGTGTCTGCCAAGAAGGCACAATTGTGTCGGACAGAGGTGACATATCTCGGCTACACATTAAGAGAAGGTAAGAGATGGCTCACTGAAGCACGTAAGAAGACTGTCATGCAGATTCCTACCCCTACCACGCCCCGTCAGGTGCGGGAGTTCCTCGGCACCGCCGGCTTCTGTAGACTGTGGATTCCTGGATTTGCCACCCTGGCCGCCCCACTCTACCTGCTGACGAAGGAAAAGGTTCCTTTCACCTGGACTGAGGAACATCAGCGGGCTTTCGAAGACATCAAAGCCGCCCTGCTGGCGGCTCCAGCTCTGGCCCTCCCTGACCTCACCAAGCCCTTCACACTCTATGTGGACGAGAGAGCTGGAGTGGCCAGAGGTGTGCTGACACAGGCACTGGGGCCGTGGAAGAGACCCGTGGCGTACCTATCTAAAAAGCTCGACCCCGTGGCCTCTGGGTGGCCGAGCTGTCTAAAGGCCATCGCTGCTGTAGCTCTACTGGTCAAAGATGCTGACAAGCTGACCCTGGGCCAGCATGTCACCGTCATCGCCCCGCACGCCTTAGAATCCATAGTCCGGCAACCACCAGATCGGTGGATGACAAACGCTAGGATGACGCATTATCAGAGCTTGCTGTTAAACGAAAGAGTGACATTTGCGCCCCCTGCCATCCTTAACCCAGCGACGCTACTGCCGGAGATCCACAACTCAACCCCCATCCACCAGTGCGTCGACATTCTGGCTGAGGAGACCGGCACGCGCAAGGACCTCACGGACAGACCATGGCCTGGTGTGCCGGCATGGTACACCGACGGCAGCTCCTTTGTCGTGGAGGGAAAGCGACGCGCAGGCGCGGCTGTGGTGGACGGCAAGCAAGTCATCTGGGCATCCTCCCTGCCAGAAGGCACCAGCGCTCAGAAAGCGGAGTTGGTGGCCTTGACCCAGGCCCTCAGATTAGCTGAAGGGAAGGCTATTAATATCTACACCGATTCTAGATACGCCTTCGCCACGGCGCACATCCACGGCGCGATCTACAAGCAGCGGGGCCTGCTGACCAGTGCCGGGCGAGACATCAAGAATAAGGAAGAGATTCTTGCCCTGCTGGAGGCCGTCCACCTCCCCAAAAAGGTGGCCATTATCCACTGCCCCGGCCACCAGAAGGGAAGCGGCGGCAGCAAAAGAACGGCGGACGGCTCTGAGAAGCGCACCGCTGATAGCCAGCATTCAACTCCTCCGAAAACAAAGAGGAAAGTTGAGTTCGAACCGAAGAAGAAAAGGAAGGTGTGA

**Sequence 66 Plasmids sequence of PE-RERV-Δ6**

(NLSc-myc-NLSSV40-nCas9(H840A/R221K/N394K)-XTEN-NC-NLS-32aa Linker-RERV-RT(Δ515~688aa)-NLSvbp)

AAGCTTACTGGTTGTACAATGCCGGCGGCCAAGAGAGTCAAGCTCGACGGCGGGAAGCGCACAGCTGATGGTTCTGAATTCGAGTCCCCTAAGAAAAAGAGAAAAGTGGACAAGAAGTACTCGATCGGCCTCGATATTGGGACTAACTCTGTTGGCTGGGCCGTGATCACCGACGAGTACAAGGTGCCCTCAAAGAAGTTCAAGGTCCTGGGCAACACCGATCGGCATTCCATCAAGAAGAATCTCATTGGCGCTCTCCTGTTCGACAGCGGCGAGACGGCTGAGGCTACGCGGCTCAAGCGCACCGCCCGCAGGCGGTACACGCGCAGGAAGAATCGCATCTGCTACCTGCAGGAGATTTTCTCCAACGAGATGGCGAAGGTTGACGATTCTTTCTTCCACAGGCTGGAGGAGTCATTCCTCGTGGAGGAGGATAAGAAGCACGAGCGGCATCCAATCTTCGGCAACATTGTCGACGAGGTTGCCTACCACGAGAAGTACCCTACGATCTACCATCTGCGGAAGAAGCTCGTGGACTCCACAGATAAGGCGGACCTCCGCCTGATCTACCTCGCTCTGGCCCACATGATTAAGTTCAGGGGCCATTTCCTGATCGAGGGGGATCTCAACCCGGACAATAGCGATGTTGACAAGCTGTTCATCCAGCTCGTGCAGACGTACAACCAGCTCTTCGAGGAGAACCCCATTAATGCGTCAGGCGTCGACGCGAAGGCTATCCTGTCCGCTAGGCTCTCGAAGTCTCGGAAGCTCGAGAACCTGATCGCCCAGCTGCCGGGCGAGAAGAAGAACGGCCTGTTCGGGAATCTCATTGCGCTCAGCCTGGGGCTCACGCCCAACTTCAAGTCGAATTTCGATCTCGCTGAGGACGCCAAGCTGCAGCTCTCCAAGGACACATACGACGATGACCTGGATAACCTCCTGGCCCAGATCGGCGATCAGTACGCGGACCTGTTCCTCGCTGCCAAGAATCTGTCGGACGCCATCCTCCTGTCTGATATTCTCAGGGTGAACACCGAGATTACGAAGGCTCCGCTCTCAGCCTCCATGATCAAGCGCTACGACGAGCACCATCAGGATCTGACCCTCCTGAAGGCGCTGGTCAGGCAGCAGCTCCCCGAGAAGTACAAGGAGATCTTCTTCGATCAGTCGAAGAACGGCTACGCTGGGTACATTGACGGCGGGGCCTCTCAGGAGGAGTTCTACAAGTTCATCAAGCCGATTCTGGAGAAGATGGACGGCACGGAGGAGCTGCTGGTGAAGCTCAAGCGCGAGGACCTCCTGAGGAAGCAGCGGACATTCGATAACGGCAGCATCCCACACCAGATTCATCTCGGGGAGCTGCACGCTATCCTGAGGAGGCAGGAGGACTTCTACCCTTTCCTCAAGGATAACCGCGAGAAGATCGAGAAGATTCTGACTTTCAGGATCCCGTACTACGTCGGCCCACTCGCTAGGGGCAACTCCCGCTTCGCTTGGATGACCCGCAAGTCAGAGGAGACGATCACGCCGTGGAACTTCGAGGAGGTGGTCGACAAGGGCGCTAGCGCTCAGTCGTTCATCGAGAGGATGACGAATTTCGACAAGAACCTGCCAAATGAGAAGGTGCTCCCTAAGCACTCGCTCCTGTACGAGTACTTCACAGTCTACAACGAGCTGACTAAGGTGAAGTATGTGACCGAGGGCATGAGGAAGCCGGCTTTCCTGTCTGGGGAGCAGAAGAAGGCCATCGTGGACCTCCTGTTCAAGACCAACCGGAAGGTCACGGTTAAGCAGCTCAAGGAGGACTACTTCAAGAAGATTGAGTGCTTCGATTCGGTCGAGATCTCTGGCGTTGAGGACCGCTTCAACGCCTCCCTGGGGACCTACCACGATCTCCTGAAGATCATTAAGGATAAGGACTTCCTGGACAACGAGGAGAATGAGGATATCCTCGAGGACATTGTGCTGACACTCACTCTGTTCGAGGACCGGGAGATGATCGAGGAGCGCCTGAAGACTTACGCCCATCTCTTCGATGACAAGGTCATGAAGCAGCTCAAGAGGAGGAGGTACACCGGCTGGGGGAGGCTGAGCAGGAAGCTCATCAACGGCATTCGGGACAAGCAGTCCGGGAAGACGATCCTCGACTTCCTGAAGAGCGATGGCTTCGCGAACCGCAATTTCATGCAGCTGATTCACGATGACAGCCTCACATTCAAGGAGGATATCCAGAAGGCTCAGGTGAGCGGCCAGGGGGACTCGCTGCACGAGCATATCGCGAACCTCGCTGGCTCGCCAGCTATCAAGAAGGGGATTCTGCAGACCGTGAAGGTTGTGGACGAGCTGGTGAAGGTCATGGGCAGGCACAAGCCTGAGAACATCGTCATTGAGATGGCCCGGGAGAATCAGACCACGCAGAAGGGCCAGAAGAACTCACGCGAGAGGATGAAGAGGATCGAGGAGGGCATTAAGGAGCTGGGGTCCCAGATCCTCAAGGAGCACCCGGTGGAGAACACGCAGCTGCAGAATGAGAAGCTCTACCTGTACTACCTCCAGAATGGCCGCGATATGTATGTGGACCAGGAGCTGGATATTAACAGGCTCAGCGATTACGACGTCGATGCCATCGTTCCACAGTCATTCCTGAAGGATGACTCCATTGACAACAAGGTCCTCACCAGGTCGGACAAGAACCGGGGCAAGTCTGATAATGTTCCTTCAGAGGAGGTCGTTAAGAAGATGAAGAACTACTGGCGCCAGCTCCTGAATGCCAAGCTGATCACGCAGCGGAAGTTCGATAACCTCACAAAGGCTGAGAGGGGCGGGCTCTCTGAGCTGGACAAGGCGGGCTTCATCAAGAGGCAGCTGGTCGAGACACGGCAGATCACTAAGCACGTTGCGCAGATTCTCGACTCACGGATGAACACTAAGTACGATGAGAATGACAAGCTGATCCGCGAGGTGAAGGTCATCACCCTGAAGTCAAAGCTCGTCTCCGACTTCAGGAAGGATTTCCAGTTCTACAAGGTTCGGGAGATCAACAATTACCACCATGCCCATGACGCGTACCTGAACGCGGTGGTCGGCACAGCTCTGATCAAGAAGTACCCAAAGCTCGAGAGCGAGTTCGTGTACGGGGACTACAAGGTTTACGATGTGAGGAAGATGATCGCCAAGTCGGAGCAGGAGATTGGCAAGGCTACCGCCAAGTACTTCTTCTACTCTAACATTATGAATTTCTTCAAGACAGAGATCACTCTGGCCAATGGCGAGATCCGGAAGCGCCCCCTCATCGAGACGAACGGCGAGACGGGGGAGATCGTGTGGGACAAGGGCAGGGATTTCGCGACCGTCAGGAAGGTTCTCTCCATGCCACAAGTGAATATCGTCAAGAAGACAGAGGTCCAGACTGGCGGGTTCTCTAAGGAGTCAATTCTGCCTAAGCGGAACAGCGACAAGCTCATCGCCCGCAAGAAGGACTGGGATCCGAAGAAGTACGGCGGGTTCGACAGCCCCACTGTGGCCTACTCGGTCCTGGTTGTGGCGAAGGTTGAGAAGGGCAAGTCCAAGAAGCTCAAGAGCGTGAAGGAGCTGCTGGGGATCACGATTATGGAGCGCTCCAGCTTCGAGAAGAACCCGATCGATTTCCTGGAGGCGAAGGGCTACAAGGAGGTGAAGAAGGACCTGATCATTAAGCTCCCCAAGTACTCACTCTTCGAGCTGGAGAACGGCAGGAAGCGGATGCTGGCTTCCGCTGGCGAGCTGCAGAAGGGGAACGAGCTGGCTCTGCCGTCCAAGTATGTGAACTTCCTCTACCTGGCCTCCCACTACGAGAAGCTCAAGGGCAGCCCCGAGGACAACGAGCAGAAGCAGCTGTTCGTCGAGCAGCACAAGCATTACCTCGACGAGATCATTGAGCAGATTTCCGAGTTCTCCAAGCGCGTGATCCTGGCCGACGCGAATCTGGATAAGGTCCTCTCCGCGTACAACAAGCACCGCGACAAGCCAATCAGGGAGCAGGCTGAGAATATCATTCATCTCTTCACCCTGACGAACCTCGGCGCCCCTGCTGCTTTCAAGTACTTCGACACAACTATCGATCGCAAGAGGTACACAAGCACTAAGGAGGTCCTGGACGCGACCCTCATCCACCAGTCGATTACCGGCCTCTACGAGACGCGCATCGACCTGTCTCAGCTCGGGGGCGACGAATTCTCCGGGAGCGAGACGCCAGGCACCTCCGAGTCGGCCACCCCAGAATCTGCCACAGTGGTGTCCGGCCAAAAGCAGGACCGCCAGGGCGGAGAACGCAGAAGGTCCCAGCTCGATAGGGATCAGTGTGCCTACTGCAAGGAGAAGGGCCACTGGGCCAAAGACTGCCCGAAAAAGCCGCGCGGCCCACGCGGCCCAAGGCCACAAACATCCCTCCTTCCAAAGAAGAAGCGGAAGGTGGAGCTCAGCGGAGGATCTTCCGGAGGATCTAGCGGCTCCGAGACACCAGGAACATCCGAAAGCGCTACACCAGAATCTAGCGGAGGCTCTTCCGGAGGATCTAGG CCTCCCGTCGCGTGCCTCGTACTGAGCCTAGAGGAGGAGTACAGGCTGCATGAGCAAAATCCGAAGCAGCTGCTAGCCCCTGAATGGCTGAGCGCCTTCTCCGAGGTATGGGCGGAGCAGACTGGCATGGGACTGGCCAAGCAGGTGCCTCCTGTGGTGGTAGAGCTGAAGGCGGACGCCAGCCCAGTGAGCGTGAAACAATACCCGATGTCGAGGGAAGCAAAGGAAGGCATTAGACCCCACATCCAGAGACTGCTGCAATTGGGAATCCTGGTACCCTGCCAAAGCCCTTGGAACACGCCACTGCTGCCTGTGCGCAAACCAGGAACTAATGATTACAGACCTGTACAGGATCTAAGGGAAGTTAACAAGCGGGTGCAGGACATACATCCTACGGTCCCAAACCCCTACAACTTGCTGAGCTCTCTGCCCCCCGAGCGCACATGGTACACTGTGCTCGACTTAAAAGATGCGTTCTTCTGCCTTCGCCTCCACCCGAACTCACAGCCACTGTTCGCGTTCGAGTGGCGCGACCCTGAGGGCGGCCACACCGGGCAACTGACGTGGACACGGCTGCCACAGGGTTTCAAGAACAGCCCTACTCTATTTGATGAGGCCCTGCACCGTGATCTGGCCCCCTTCCGGGCGCAGAACCCTCAAATCAGTCTACTGCAGTATGTTGATGACCTGCTGCTAGCCGCTTCTACTCAGGAGCTCTGCCGGGAGGGCACAAAGCGGCTGTTAAATGAGCTGGGCGAGCTGGGATACCGCGTGTCTGCCAAGAAGGCACAATTGTGTCGGACAGAGGTGACATATCTCGGCTACACATTAAGAGAAGGTAAGAGATGGCTCACTGAAGCACGTAAGAAGACTGTCATGCAGATTCCTACCCCTACCACGCCCCGTCAGGTGCGGGAGTTCCTCGGCACCGCCGGCTTCTGTAGACTGTGGATTCCTGGATTTGCCACCCTGGCCGCCCCACTCTACCTGCTGACGAAGGAAAAGGTTCCTTTCACCTGGACTGAGGAACATCAGCGGGCTTTCGAAGACATCAAAGCCGCCCTGCTGGCGGCTCCAGCTCTGGCCCTCCCTGACCTCACCAAGCCCTTCACACTCTATGTGGACGAGAGAGCTGGAGTGGCCAGAGGTGTGCTGACACAGGCACTGGGGCCGTGGAAGAGACCCGTGGCGTACCTATCTAAAAAGCTCGACCCCGTGGCCTCTGGGTGGCCGAGCTGTCTAAAGGCCATCGCTGCTGTAGCTCTACTGGTCAAAGATGCTGACAAGCTGACCCTGGGCCAGCATGTCACCGTCATCGCCCCGCACGCCTTAGAATCCATAGTCCGGCAACCACCAGATCGGTGGATGACAAACGCTAGGATGACGCATTATCAGAGCTTGCTGTTAAACGAAAGAGTGACATTTGCGCCCCCTGCCATCCTTAACCCAGCGACGCTACTGCCGGAGATCCACAACTCAACCCCCATCCACCAGTGCGTCGACATTCTGGCTGAGGAGACCGGCACGCGCAAGGACCTCACGAGCGGCGGCAGCAAAAGAACGGCGGACGGCTCTGAGAAGCGCACCGCTGATAGCCAGCATTCAACTCCTCCGAAAACAAAGAGGAAAGTTGAGTTCGAACCGAAGAAGAAAAGGAAGGTGTGA

**Sequence 67 Plasmids sequence of PE-RERV-Δ7**

(NLSc-myc-NLSSV40-nCas9(H840A/R221K/N394K)-XTEN-NC-NLS-32aa Linker-RERV-RT(Δ454~688aa)-NLSvbp)

AAGCTTACTGGTTGTACAATGCCGGCGGCCAAGAGAGTCAAGCTCGACGGCGGGAAGCGCACAGCTGATGGTTCTGAATTCGAGTCCCCTAAGAAAAAGAGAAAAGTGGACAAGAAGTACTCGATCGGCCTCGATATTGGGACTAACTCTGTTGGCTGGGCCGTGATCACCGACGAGTACAAGGTGCCCTCAAAGAAGTTCAAGGTCCTGGGCAACACCGATCGGCATTCCATCAAGAAGAATCTCATTGGCGCTCTCCTGTTCGACAGCGGCGAGACGGCTGAGGCTACGCGGCTCAAGCGCACCGCCCGCAGGCGGTACACGCGCAGGAAGAATCGCATCTGCTACCTGCAGGAGATTTTCTCCAACGAGATGGCGAAGGTTGACGATTCTTTCTTCCACAGGCTGGAGGAGTCATTCCTCGTGGAGGAGGATAAGAAGCACGAGCGGCATCCAATCTTCGGCAACATTGTCGACGAGGTTGCCTACCACGAGAAGTACCCTACGATCTACCATCTGCGGAAGAAGCTCGTGGACTCCACAGATAAGGCGGACCTCCGCCTGATCTACCTCGCTCTGGCCCACATGATTAAGTTCAGGGGCCATTTCCTGATCGAGGGGGATCTCAACCCGGACAATAGCGATGTTGACAAGCTGTTCATCCAGCTCGTGCAGACGTACAACCAGCTCTTCGAGGAGAACCCCATTAATGCGTCAGGCGTCGACGCGAAGGCTATCCTGTCCGCTAGGCTCTCGAAGTCTCGGAAGCTCGAGAACCTGATCGCCCAGCTGCCGGGCGAGAAGAAGAACGGCCTGTTCGGGAATCTCATTGCGCTCAGCCTGGGGCTCACGCCCAACTTCAAGTCGAATTTCGATCTCGCTGAGGACGCCAAGCTGCAGCTCTCCAAGGACACATACGACGATGACCTGGATAACCTCCTGGCCCAGATCGGCGATCAGTACGCGGACCTGTTCCTCGCTGCCAAGAATCTGTCGGACGCCATCCTCCTGTCTGATATTCTCAGGGTGAACACCGAGATTACGAAGGCTCCGCTCTCAGCCTCCATGATCAAGCGCTACGACGAGCACCATCAGGATCTGACCCTCCTGAAGGCGCTGGTCAGGCAGCAGCTCCCCGAGAAGTACAAGGAGATCTTCTTCGATCAGTCGAAGAACGGCTACGCTGGGTACATTGACGGCGGGGCCTCTCAGGAGGAGTTCTACAAGTTCATCAAGCCGATTCTGGAGAAGATGGACGGCACGGAGGAGCTGCTGGTGAAGCTCAAGCGCGAGGACCTCCTGAGGAAGCAGCGGACATTCGATAACGGCAGCATCCCACACCAGATTCATCTCGGGGAGCTGCACGCTATCCTGAGGAGGCAGGAGGACTTCTACCCTTTCCTCAAGGATAACCGCGAGAAGATCGAGAAGATTCTGACTTTCAGGATCCCGTACTACGTCGGCCCACTCGCTAGGGGCAACTCCCGCTTCGCTTGGATGACCCGCAAGTCAGAGGAGACGATCACGCCGTGGAACTTCGAGGAGGTGGTCGACAAGGGCGCTAGCGCTCAGTCGTTCATCGAGAGGATGACGAATTTCGACAAGAACCTGCCAAATGAGAAGGTGCTCCCTAAGCACTCGCTCCTGTACGAGTACTTCACAGTCTACAACGAGCTGACTAAGGTGAAGTATGTGACCGAGGGCATGAGGAAGCCGGCTTTCCTGTCTGGGGAGCAGAAGAAGGCCATCGTGGACCTCCTGTTCAAGACCAACCGGAAGGTCACGGTTAAGCAGCTCAAGGAGGACTACTTCAAGAAGATTGAGTGCTTCGATTCGGTCGAGATCTCTGGCGTTGAGGACCGCTTCAACGCCTCCCTGGGGACCTACCACGATCTCCTGAAGATCATTAAGGATAAGGACTTCCTGGACAACGAGGAGAATGAGGATATCCTCGAGGACATTGTGCTGACACTCACTCTGTTCGAGGACCGGGAGATGATCGAGGAGCGCCTGAAGACTTACGCCCATCTCTTCGATGACAAGGTCATGAAGCAGCTCAAGAGGAGGAGGTACACCGGCTGGGGGAGGCTGAGCAGGAAGCTCATCAACGGCATTCGGGACAAGCAGTCCGGGAAGACGATCCTCGACTTCCTGAAGAGCGATGGCTTCGCGAACCGCAATTTCATGCAGCTGATTCACGATGACAGCCTCACATTCAAGGAGGATATCCAGAAGGCTCAGGTGAGCGGCCAGGGGGACTCGCTGCACGAGCATATCGCGAACCTCGCTGGCTCGCCAGCTATCAAGAAGGGGATTCTGCAGACCGTGAAGGTTGTGGACGAGCTGGTGAAGGTCATGGGCAGGCACAAGCCTGAGAACATCGTCATTGAGATGGCCCGGGAGAATCAGACCACGCAGAAGGGCCAGAAGAACTCACGCGAGAGGATGAAGAGGATCGAGGAGGGCATTAAGGAGCTGGGGTCCCAGATCCTCAAGGAGCACCCGGTGGAGAACACGCAGCTGCAGAATGAGAAGCTCTACCTGTACTACCTCCAGAATGGCCGCGATATGTATGTGGACCAGGAGCTGGATATTAACAGGCTCAGCGATTACGACGTCGATGCCATCGTTCCACAGTCATTCCTGAAGGATGACTCCATTGACAACAAGGTCCTCACCAGGTCGGACAAGAACCGGGGCAAGTCTGATAATGTTCCTTCAGAGGAGGTCGTTAAGAAGATGAAGAACTACTGGCGCCAGCTCCTGAATGCCAAGCTGATCACGCAGCGGAAGTTCGATAACCTCACAAAGGCTGAGAGGGGCGGGCTCTCTGAGCTGGACAAGGCGGGCTTCATCAAGAGGCAGCTGGTCGAGACACGGCAGATCACTAAGCACGTTGCGCAGATTCTCGACTCACGGATGAACACTAAGTACGATGAGAATGACAAGCTGATCCGCGAGGTGAAGGTCATCACCCTGAAGTCAAAGCTCGTCTCCGACTTCAGGAAGGATTTCCAGTTCTACAAGGTTCGGGAGATCAACAATTACCACCATGCCCATGACGCGTACCTGAACGCGGTGGTCGGCACAGCTCTGATCAAGAAGTACCCAAAGCTCGAGAGCGAGTTCGTGTACGGGGACTACAAGGTTTACGATGTGAGGAAGATGATCGCCAAGTCGGAGCAGGAGATTGGCAAGGCTACCGCCAAGTACTTCTTCTACTCTAACATTATGAATTTCTTCAAGACAGAGATCACTCTGGCCAATGGCGAGATCCGGAAGCGCCCCCTCATCGAGACGAACGGCGAGACGGGGGAGATCGTGTGGGACAAGGGCAGGGATTTCGCGACCGTCAGGAAGGTTCTCTCCATGCCACAAGTGAATATCGTCAAGAAGACAGAGGTCCAGACTGGCGGGTTCTCTAAGGAGTCAATTCTGCCTAAGCGGAACAGCGACAAGCTCATCGCCCGCAAGAAGGACTGGGATCCGAAGAAGTACGGCGGGTTCGACAGCCCCACTGTGGCCTACTCGGTCCTGGTTGTGGCGAAGGTTGAGAAGGGCAAGTCCAAGAAGCTCAAGAGCGTGAAGGAGCTGCTGGGGATCACGATTATGGAGCGCTCCAGCTTCGAGAAGAACCCGATCGATTTCCTGGAGGCGAAGGGCTACAAGGAGGTGAAGAAGGACCTGATCATTAAGCTCCCCAAGTACTCACTCTTCGAGCTGGAGAACGGCAGGAAGCGGATGCTGGCTTCCGCTGGCGAGCTGCAGAAGGGGAACGAGCTGGCTCTGCCGTCCAAGTATGTGAACTTCCTCTACCTGGCCTCCCACTACGAGAAGCTCAAGGGCAGCCCCGAGGACAACGAGCAGAAGCAGCTGTTCGTCGAGCAGCACAAGCATTACCTCGACGAGATCATTGAGCAGATTTCCGAGTTCTCCAAGCGCGTGATCCTGGCCGACGCGAATCTGGATAAGGTCCTCTCCGCGTACAACAAGCACCGCGACAAGCCAATCAGGGAGCAGGCTGAGAATATCATTCATCTCTTCACCCTGACGAACCTCGGCGCCCCTGCTGCTTTCAAGTACTTCGACACAACTATCGATCGCAAGAGGTACACAAGCACTAAGGAGGTCCTGGACGCGACCCTCATCCACCAGTCGATTACCGGCCTCTACGAGACGCGCATCGACCTGTCTCAGCTCGGGGGCGACGAATTCTCCGGGAGCGAGACGCCAGGCACCTCCGAGTCGGCCACCCCAGAATCTGCCACAGTGGTGTCCGGCCAAAAGCAGGACCGCCAGGGCGGAGAACGCAGAAGGTCCCAGCTCGATAGGGATCAGTGTGCCTACTGCAAGGAGAAGGGCCACTGGGCCAAAGACTGCCCGAAAAAGCCGCGCGGCCCACGCGGCCCAAGGCCACAAACATCCCTCCTTCCAAAGAAGAAGCGGAAGGTGGAGCTCAGCGGAGGATCTTCCGGAGGATCTAGCGGCTCCGAGACACCAGGAACATCCGAAAGCGCTACACCAGAATCTAGCGGAGGCTCTTCCGGAGGATCTAGG CCTCCCGTCGCGTGCCTCGTACTGAGCCTAGAGGAGGAGTACAGGCTGCATGAGCAAAATCCGAAGCAGCTGCTAGCCCCTGAATGGCTGAGCGCCTTCTCCGAGGTATGGGCGGAGCAGACTGGCATGGGACTGGCCAAGCAGGTGCCTCCTGTGGTGGTAGAGCTGAAGGCGGACGCCAGCCCAGTGAGCGTGAAACAATACCCGATGTCGAGGGAAGCAAAGGAAGGCATTAGACCCCACATCCAGAGACTGCTGCAATTGGGAATCCTGGTACCCTGCCAAAGCCCTTGGAACACGCCACTGCTGCCTGTGCGCAAACCAGGAACTAATGATTACAGACCTGTACAGGATCTAAGGGAAGTTAACAAGCGGGTGCAGGACATACATCCTACGGTCCCAAACCCCTACAACTTGCTGAGCTCTCTGCCCCCCGAGCGCACATGGTACACTGTGCTCGACTTAAAAGATGCGTTCTTCTGCCTTCGCCTCCACCCGAACTCACAGCCACTGTTCGCGTTCGAGTGGCGCGACCCTGAGGGCGGCCACACCGGGCAACTGACGTGGACACGGCTGCCACAGGGTTTCAAGAACAGCCCTACTCTATTTGATGAGGCCCTGCACCGTGATCTGGCCCCCTTCCGGGCGCAGAACCCTCAAATCAGTCTACTGCAGTATGTTGATGACCTGCTGCTAGCCGCTTCTACTCAGGAGCTCTGCCGGGAGGGCACAAAGCGGCTGTTAAATGAGCTGGGCGAGCTGGGATACCGCGTGTCTGCCAAGAAGGCACAATTGTGTCGGACAGAGGTGACATATCTCGGCTACACATTAAGAGAAGGTAAGAGATGGCTCACTGAAGCACGTAAGAAGACTGTCATGCAGATTCCTACCCCTACCACGCCCCGTCAGGTGCGGGAGTTCCTCGGCACCGCCGGCTTCTGTAGACTGTGGATTCCTGGATTTGCCACCCTGGCCGCCCCACTCTACCTGCTGACGAAGGAAAAGGTTCCTTTCACCTGGACTGAGGAACATCAGCGGGCTTTCGAAGACATCAAAGCCGCCCTGCTGGCGGCTCCAGCTCTGGCCCTCCCTGACCTCACCAAGCCCTTCACACTCTATGTGGACGAGAGAGCTGGAGTGGCCAGAGGTGTGCTGACACAGGCACTGGGGCCGTGGAAGAGACCCGTGGCGTACCTATCTAAAAAGCTCGACCCCGTGGCCTCTGGGTGGCCGAGCTGTCTAAAGGCCATCGCTGCTGTAGCTCTACTGGTCAAAGATGCTGACAAGCTGACCCTGGGCCAGCATGTCACCGTCATCGCCCCGCACGCCTTAGAATCCATAGTCCGGCAACCACCAGATAGCGGCGGCAGCAAAAGAACGGCGGACGGCTCTGAGAAGCGCACCGCTGATAGCCAGCATTCAACTCCTCCGAAAACAAAGAGGAAAGTTGAGTTCGAACCGAAGAAGAAAAGGAAGGTGTGA

**Sequence 70 Plasmids sequence of RERV-m5-MLH1dn**

(NLSc-myc-NLSSV40-nCas9(H840A/R221K/N394K)-XTEN-NC-NLS-32aa Linker-RERV-m5-NLSSV40-P2A-MLH1dn)

AAGCTTACTGGTTGTACAATGCCGGCGGCCAAGAGAGTCAAGCTCGACGGCGGGAAGCGCACAGCTGATGGTTCTGAATTCGAGTCCCCTAAGAAAAAGAGAAAAGTGGACAAGAAGTACTCGATCGGCCTCGATATTGGGACTAACTCTGTTGGCTGGGCCGTGATCACCGACGAGTACAAGGTGCCCTCAAAGAAGTTCAAGGTCCTGGGCAACACCGATCGGCATTCCATCAAGAAGAATCTCATTGGCGCTCTCCTGTTCGACAGCGGCGAGACGGCTGAGGCTACGCGGCTCAAGCGCACCGCCCGCAGGCGGTACACGCGCAGGAAGAATCGCATCTGCTACCTGCAGGAGATTTTCTCCAACGAGATGGCGAAGGTTGACGATTCTTTCTTCCACAGGCTGGAGGAGTCATTCCTCGTGGAGGAGGATAAGAAGCACGAGCGGCATCCAATCTTCGGCAACATTGTCGACGAGGTTGCCTACCACGAGAAGTACCCTACGATCTACCATCTGCGGAAGAAGCTCGTGGACTCCACAGATAAGGCGGACCTCCGCCTGATCTACCTCGCTCTGGCCCACATGATTAAGTTCAGGGGCCATTTCCTGATCGAGGGGGATCTCAACCCGGACAATAGCGATGTTGACAAGCTGTTCATCCAGCTCGTGCAGACGTACAACCAGCTCTTCGAGGAGAACCCCATTAATGCGTCAGGCGTCGACGCGAAGGCTATCCTGTCCGCTAGGCTCTCGAAGTCTCGGAAGCTCGAGAACCTGATCGCCCAGCTGCCGGGCGAGAAGAAGAACGGCCTGTTCGGGAATCTCATTGCGCTCAGCCTGGGGCTCACGCCCAACTTCAAGTCGAATTTCGATCTCGCTGAGGACGCCAAGCTGCAGCTCTCCAAGGACACATACGACGATGACCTGGATAACCTCCTGGCCCAGATCGGCGATCAGTACGCGGACCTGTTCCTCGCTGCCAAGAATCTGTCGGACGCCATCCTCCTGTCTGATATTCTCAGGGTGAACACCGAGATTACGAAGGCTCCGCTCTCAGCCTCCATGATCAAGCGCTACGACGAGCACCATCAGGATCTGACCCTCCTGAAGGCGCTGGTCAGGCAGCAGCTCCCCGAGAAGTACAAGGAGATCTTCTTCGATCAGTCGAAGAACGGCTACGCTGGGTACATTGACGGCGGGGCCTCTCAGGAGGAGTTCTACAAGTTCATCAAGCCGATTCTGGAGAAGATGGACGGCACGGAGGAGCTGCTGGTGAAGCTCAAGCGCGAGGACCTCCTGAGGAAGCAGCGGACATTCGATAACGGCAGCATCCCACACCAGATTCATCTCGGGGAGCTGCACGCTATCCTGAGGAGGCAGGAGGACTTCTACCCTTTCCTCAAGGATAACCGCGAGAAGATCGAGAAGATTCTGACTTTCAGGATCCCGTACTACGTCGGCCCACTCGCTAGGGGCAACTCCCGCTTCGCTTGGATGACCCGCAAGTCAGAGGAGACGATCACGCCGTGGAACTTCGAGGAGGTGGTCGACAAGGGCGCTAGCGCTCAGTCGTTCATCGAGAGGATGACGAATTTCGACAAGAACCTGCCAAATGAGAAGGTGCTCCCTAAGCACTCGCTCCTGTACGAGTACTTCACAGTCTACAACGAGCTGACTAAGGTGAAGTATGTGACCGAGGGCATGAGGAAGCCGGCTTTCCTGTCTGGGGAGCAGAAGAAGGCCATCGTGGACCTCCTGTTCAAGACCAACCGGAAGGTCACGGTTAAGCAGCTCAAGGAGGACTACTTCAAGAAGATTGAGTGCTTCGATTCGGTCGAGATCTCTGGCGTTGAGGACCGCTTCAACGCCTCCCTGGGGACCTACCACGATCTCCTGAAGATCATTAAGGATAAGGACTTCCTGGACAACGAGGAGAATGAGGATATCCTCGAGGACATTGTGCTGACACTCACTCTGTTCGAGGACCGGGAGATGATCGAGGAGCGCCTGAAGACTTACGCCCATCTCTTCGATGACAAGGTCATGAAGCAGCTCAAGAGGAGGAGGTACACCGGCTGGGGGAGGCTGAGCAGGAAGCTCATCAACGGCATTCGGGACAAGCAGTCCGGGAAGACGATCCTCGACTTCCTGAAGAGCGATGGCTTCGCGAACCGCAATTTCATGCAGCTGATTCACGATGACAGCCTCACATTCAAGGAGGATATCCAGAAGGCTCAGGTGAGCGGCCAGGGGGACTCGCTGCACGAGCATATCGCGAACCTCGCTGGCTCGCCAGCTATCAAGAAGGGGATTCTGCAGACCGTGAAGGTTGTGGACGAGCTGGTGAAGGTCATGGGCAGGCACAAGCCTGAGAACATCGTCATTGAGATGGCCCGGGAGAATCAGACCACGCAGAAGGGCCAGAAGAACTCACGCGAGAGGATGAAGAGGATCGAGGAGGGCATTAAGGAGCTGGGGTCCCAGATCCTCAAGGAGCACCCGGTGGAGAACACGCAGCTGCAGAATGAGAAGCTCTACCTGTACTACCTCCAGAATGGCCGCGATATGTATGTGGACCAGGAGCTGGATATTAACAGGCTCAGCGATTACGACGTCGATCACATCGTTCCACAGTCATTCCTGAAGGATGACTCCATTGACAACAAGGTCCTCACCAGGTCGGACAAGAACCGGGGCAAGTCTGATAATGTTCCTTCAGAGGAGGTCGTTAAGAAGATGAAGAACTACTGGCGCCAGCTCCTGAATGCCAAGCTGATCACGCAGCGGAAGTTCGATAACCTCACAAAGGCTGAGAGGGGCGGGCTCTCTGAGCTGGACAAGGCGGGCTTCATCAAGAGGCAGCTGGTCGAGACACGGCAGATCACTAAGCACGTTGCGCAGATTCTCGACTCACGGATGAACACTAAGTACGATGAGAATGACAAGCTGATCCGCGAGGTGAAGGTCATCACCCTGAAGTCAAAGCTCGTCTCCGACTTCAGGAAGGATTTCCAGTTCTACAAGGTTCGGGAGATCAACAATTACCACCATGCCCATGACGCGTACCTGAACGCGGTGGTCGGCACAGCTCTGATCAAGAAGTACCCAAAGCTCGAGAGCGAGTTCGTGTACGGGGACTACAAGGTTTACGATGTGAGGAAGATGATCGCCAAGTCGGAGCAGGAGATTGGCAAGGCTACCGCCAAGTACTTCTTCTACTCTAACATTATGAATTTCTTCAAGACAGAGATCACTCTGGCCAATGGCGAGATCCGGAAGCGCCCCCTCATCGAGACGAACGGCGAGACGGGGGAGATCGTGTGGGACAAGGGCAGGGATTTCGCGACCGTCAGGAAGGTTCTCTCCATGCCACAAGTGAATATCGTCAAGAAGACAGAGGTCCAGACTGGCGGGTTCTCTAAGGAGTCAATTCTGCCTAAGCGGAACAGCGACAAGCTCATCGCCCGCAAGAAGGACTGGGATCCGAAGAAGTACGGCGGGTTCGACAGCCCCACTGTGGCCTACTCGGTCCTGGTTGTGGCGAAGGTTGAGAAGGGCAAGTCCAAGAAGCTCAAGAGCGTGAAGGAGCTGCTGGGGATCACGATTATGGAGCGCTCCAGCTTCGAGAAGAACCCGATCGATTTCCTGGAGGCGAAGGGCTACAAGGAGGTGAAGAAGGACCTGATCATTAAGCTCCCCAAGTACTCACTCTTCGAGCTGGAGAACGGCAGGAAGCGGATGCTGGCTTCCGCTGGCGAGCTGCAGAAGGGGAACGAGCTGGCTCTGCCGTCCAAGTATGTGAACTTCCTCTACCTGGCCTCCCACTACGAGAAGCTCAAGGGCAGCCCCGAGGACAACGAGCAGAAGCAGCTGTTCGTCGAGCAGCACAAGCATTACCTCGACGAGATCATTGAGCAGATTTCCGAGTTCTCCAAGCGCGTGATCCTGGCCGACGCGAATCTGGATAAGGTCCTCTCCGCGTACAACAAGCACCGCGACAAGCCAATCAGGGAGCAGGCTGAGAATATCATTCATCTCTTCACCCTGACGAACCTCGGCGCCCCTGCTGCTTTCAAGTACTTCGACACAACTATCGATCGCAAGAGGTACACAAGCACTAAGGAGGTCCTGGACGCGACCCTCATCCACCAGTCGATTACCGGCCTCTACGAGACGCGCATCGACCTGTCTCAGCTCGGGGGCGACGAATTCTCCGGGAGCGAGACGCCAGGCACCTCCGAGTCGGCCACCCCAGAATCTGCCACAGTGGTGTCCGGCCAAAAGCAGGACCGCCAGGGCGGAGAACGCAGAAGGTCCCAGCTCGATAGGGATCAGTGTGCCTACTGCAAGGAGAAGGGCCACTGGGCCAAAGACTGCCCGAAAAAGCCGCGCGGCCCACGCGGCCCAAGGCCACAAACATCCCTCCTTCCAAAGAAGAAGCGGAAGGTGGAGCTCAGCGGAGGATCTTCCGGAGGATCTAGCGGCTCCGAGACACCAGGAACATCCGAAAGCGCTACACCAGAATCTAGCGGAGGCTCTTCCGGAGGATCTAGGCCTCCTCCCGTCGCGTGCCTCGTACTGAGCCTAGAGGAGGAGTACAGGCTGCATGAGCAAAATCCGAAGCAGCTGCTAGCCCCTGAATGGCTGAGCGCCTTCTCCGAGGTATGGGCGGAGCAGACTGGCATGGGACTGGCCAAGCAGGTGCCTCCTGTGGTGGTAGAGCTGAAGGCGGACGCCAGCCCAGTGAGCGTGAAACAATACCCGATGTCGAGGGAAGCAAAGGAAGGCATTAGACCCCACATCCAGAGACTGCTGCAATTGGGAATCCTGGTACCCTGCCAAAGCCCTTGGAACACGCCACTGCTGCCTGTGCGCAAACCAGGAACTAATGATTACAGACCTGTACAGGATCTAAGGGAAGTTAACAAGCGGGTGCAGGACATACATCCTACGGTCCCAAACCCCTACAACTTGCTGAGCTCTCTGCCCCCCGAGCGCACATGGTACACTGTGCTCGACTTAAAAGATGCGTTCTTCTGCCTTCGCCTCCACCCGAACTCACAGCCACTGTTCGCGTTCGAGTGGCGCGACCCTGAGGGCGGCCACACCGGGCAACTGACGTGGACACGGCTGCCACAGGGTTTCAAGAACAGCCCTACTCTATTTAACGAGGCCCTGCACCGTGATCTGGCCCCCTTCCGGGCGCAGAACCCTCAAATCAGTCTACTGCAGTATGCGGATGACCTGCTGCTAGCCGCTTCTACTCAGGAGCTCTGCCGGGAGGGCACAAAGCGGCTGTTAAATGAGCTGGGCGAGCTGGGATACCGCGTGTCTGCCAAGAAGGCACAATTGTGTCGGACAGAGGTGACATATCTCGGCTACACATTAAGAGAAGGTAAGAGATGGCTCACTGAAGCACGTAAGAAGACTGTCATGCAGATTCCTACCCCTACCACGCCCCGTCAGGTGCGGGAGTTCCTCGGCAAGGCCGGCTTCTGTAGACTGTTCATTCCTGGATTTGCCACCCTGGCCGCCCCACTCTACCTGCTGACGAAGCCAAAGGTTCCTTTCACCTGGACTGAGGAACATCAGCGGGCTTTCGAAGACATCAAAGCCGCCCTGCTGGCGGCTCCAGCTCTGGCCCTCCCTGACCTCACCAAGCCCTTCACACTCTATGTGGACGAGAGAGCTGGAGTGGCCAGAGGTGTGCTGACACAGGCACTGGGGCCGTGGAAGAGACCCGTGGCGTACCTATCTAAAAAGCTCGACCCCGTGGCCTCTGGGTGGCCGAGCTGTCTAAAGGCCATCGCTGCTGTAGCTCTACTGGTCAAAGATGCTGACAAGCTGACCCTGGGCCAGCATGTCACCGTCATCGCCCCGCACGCCTTAGAATCCATAGTCCGGCAACCACCAGATCGGTGGATGACAAACGCTAGGATGACGCATTATCAGAGCTTGCTGTTAAACGAAAGAGTGACATTTGCGCCCCCTGCCATCCTTAACCCAGCGACGCTACTGCCGGAGATCCACAACTCAACCCCCATCCACCAGTGCGTCGACATTCTGGCTGAGGAGACCGGCACGCGCAAGGACCTCACGGACAGACCATGGCCTGGTGTGCCGGCATGGTACACCGACGGCAGCTCCTTTGTCGTGGAGGGAAAGCGACGCGCAGGCGCGGCTGTGGTGGACGGCAAGCAAGTCATCTGGGCATCCTCCCTGCCAGAAGGCACCAGCGCTCAGAAAGCGGAGTTGGTGGCCTTGACCCAGGCCCTCAGATTAGCTGAAGGGAAGGCTATTAATATCTACACCGATTCTAGATACGCCTTCGCCACGGCGCACATCCACGGCGCGATCTACAAGCAGCGGGGCCTGCTGACCAGTGCCGGGCGAGACATCAAGAATAAGGAAGAGATTCTTGCCCTGCTGGAGGCCGTCCACCTCCCCAAAAAGGTGGCCATTATCCACTGCCCCGGCCACCAGAAGGGAGAGGATCCAATTACAAAGGGGAACCAGATGGCCGACCTCGTGGCGAAGCAGGTCGCTCAGCAGGTTACCATCCTGGCAGAAAAATCGCAGACCCCTGTGAAGACACCTGTGACAGATGATTCTAACTATAGCGGCGGCAGCAAAAGAACGGCGGACGGCTCTGAGAAGCGCACCGCTGATAGCCAGCATTCAACTCCTCCGAAAACAAAGAGGAAAGTTGAGTTCGAACCGAAGAAGAAAAGGAAGGTGGGAAGCGGAGCTACTAACTTCAGCCTGCTGAAGCAGGCTGGAGACGTGGAGGAGAACCCTGGACCTATGGACGAGCCTTCGCCACGCGGCGGCGGGTGCGCTGGCGAGCCACCACGCATCCGGAGGCTGGAGGAGTCTGTGGTGAACCGCATCGCTGCTGGCGAGGTGATCCAGAGGCCATCATCGGCTGTTAAGGAGCTGATCGAGAACAGCCTCGACGCTGGCGCCTCCAGCGTCTCCGTTGCGGTGAAGGACGGCGGCCTCAAGCTCATCCAGGTGTCCGATGACGGCCATGGCATCAGGTTCGAGGATCTGGCCATCCTGTGCGAGAGGCATACTACCTCAAAGCTGTCTGCTTACGAGGATCTGCAGACCATCAAGTCGATGGGGTTCAGGGGCGAGGCTCTGGCTAGCATGACTTATGTTGGCCATGTTACCGTGACAACGATCACAGAGGGCCAGCTGCACGGCTACAGGGTTTCTTACAGGGATGGCGTTATGGAGAATGAGCCTAAGCCTTGCGCTGCGGTGAAGGGCACTCAGGTCATGGTTGAGAATCTGTTCTACAACATGGTTGCCCGCAAGAAGACACTGCAGAACTCCAATGATGACTACCCCAAGATCGTCGACTTCATCAGCCGGTTCGCCGTCCACCACATCAACGTGACCTTCTCGTGCAGGAAGCATGGCGCCAACAGGGCTGATGTGCACAGCGCTAGCACGTCCTCGAGGCTCGATGCCATCAGGAGCGTCTACGGGGCTTCCGTCGTGCGCGATCTCATCGAGATCAAGGTGTCATACGAGGATGCTGCTGACTCCATCTTCAAGATGGATGGCTACATCTCCAATGCCAACTACGTGGCCAAGAAGATCACCATGATCCTGTTCATCAACGACAGGCTCGTTGACTGCACTGCTCTGAAGCGCGCTATTGAGTTCGTGTACTCTGCTACACTGCCGCAGGCGTCCAAGCCGTTCATCTACATGTCCATTCATCTGCCGTCAGAGCACGTGGATGTCAACATTCACCCGACCAAGAAGGAGGTTAGCCTGCTGAATCAGGAGCGCATCATTGAGACAATCAGGAACGCTATCGAGGAGAAGCTGATGAACTCCAACACCACCAGGATCTTCCAGACTCAGGCGCTCAACCTGTCAGGGATTGCTCAGGCTAACCCACAGAAGGATAAGGTTTCTGAGGCCAGCATGGGGTCTGGCACAAAGTCTCAGAAGATTCCTGTGAGCCAGATGGTCAGGACAGATCCACGCAATCCATCTGGCAGGCTGCACACCTACTGGCACGGGCAGTCTTCAAATCTCGAGAAGAAGTTCGATCTCGTGTCTGTTAGGAATGTTGTTAGGTCAAGGAGGAACCAGAAGGATGCTGGCGATCTGTCAAGCCGCCATGAGCTGCTCGTGGAGATTGATTCTAGCTTCCATCCTGGCCTGCTGGACATTGTCAAGAACTGCACATATGTTGGGCTCGCCGATGAGGCGTTCGCTCTGATTCAGCACAATACCCGCCTGTACCTCGTCAATGTGGTCAATATTAGCAAGGAGCTGATGTACCAGCAGGCTCTGTGCCGCTTCGGGAACTTCAATGCTATTCAGCTCAGCGAGCCAGCTCCACTGCAGGAGCTGCTGGTGATGGCTCTGAAGGACGATGAGCTGATGAGCGATGAGAAGGATGATGAGAAGCTGGAGATTGCTGAGGTCAACACTGAGATTCTCAAGGAGAATGCTGAGATGATTAATGAGTACTTCTCAATTCACATTGATCAGGATGGCAAGCTGACAAGGCTGCCTGTTGTTCTGGACCAGTACACCCCTGATATGGACCGGCTGCCAGAGTTCGTGCTGGCTCTCGGCAATGATGTTACTTGGGATGACGAGAAGGAGTGCTTCAGGACAGTTGCTTCTGCTGTTGGCAACTTCTATGCGCTGCATCCCCCAATCCTGCCAAATCCATCTGGGAATGGCATTCATCTGTACAAGAAGAATAGGGATTCAATGGCTGATGAGCATGCTGAGAATGATCTCATCTCAGATGAGAATGACGTTGATCAGGAGCTGCTCGCGGAGGCTGAGGCTGCTTGGGCCCAGCGCGAGTGGACCATTCAGCATGTCCTGTTCCCATCCATGCGGCTGTTCCTCAAGCCCCCGAAGTCAATGGCTACAGATGGCACGTTCGTGCAGGTTGCTTCCCTGGAGAAGCTCTACAAGATCTTCTGA

**Sequence 68 Plasmids sequence of PE-RERV-m5-OsMLH1dn**

(NLSc-myc-NLSSV40-nCas9(H840A/R221K/N394K)-XTEN-NC-NLS-32aa Linker-RERV-RT-m5-NLSSV40-P2A-OsMLH1dn)

AAGCTTACTGGTTGTACAATGCCGGCGGCCAAGAGAGTCAAGCTCGACGGCGGGAAGCGCACAGCTGATGGTTCTGAATTCGAGTCCCCTAAGAAAAAGAGAAAAGTGGACAAGAAGTACTCGATCGGCCTCGATATTGGGACTAACTCTGTTGGCTGGGCCGTGATCACCGACGAGTACAAGGTGCCCTCAAAGAAGTTCAAGGTCCTGGGCAACACCGATCGGCATTCCATCAAGAAGAATCTCATTGGCGCTCTCCTGTTCGACAGCGGCGAGACGGCTGAGGCTACGCGGCTCAAGCGCACCGCCCGCAGGCGGTACACGCGCAGGAAGAATCGCATCTGCTACCTGCAGGAGATTTTCTCCAACGAGATGGCGAAGGTTGACGATTCTTTCTTCCACAGGCTGGAGGAGTCATTCCTCGTGGAGGAGGATAAGAAGCACGAGCGGCATCCAATCTTCGGCAACATTGTCGACGAGGTTGCCTACCACGAGAAGTACCCTACGATCTACCATCTGCGGAAGAAGCTCGTGGACTCCACAGATAAGGCGGACCTCCGCCTGATCTACCTCGCTCTGGCCCACATGATTAAGTTCAGGGGCCATTTCCTGATCGAGGGGGATCTCAACCCGGACAATAGCGATGTTGACAAGCTGTTCATCCAGCTCGTGCAGACGTACAACCAGCTCTTCGAGGAGAACCCCATTAATGCGTCAGGCGTCGACGCGAAGGCTATCCTGTCCGCTAGGCTCTCGAAGTCTCGGAAGCTCGAGAACCTGATCGCCCAGCTGCCGGGCGAGAAGAAGAACGGCCTGTTCGGGAATCTCATTGCGCTCAGCCTGGGGCTCACGCCCAACTTCAAGTCGAATTTCGATCTCGCTGAGGACGCCAAGCTGCAGCTCTCCAAGGACACATACGACGATGACCTGGATAACCTCCTGGCCCAGATCGGCGATCAGTACGCGGACCTGTTCCTCGCTGCCAAGAATCTGTCGGACGCCATCCTCCTGTCTGATATTCTCAGGGTGAACACCGAGATTACGAAGGCTCCGCTCTCAGCCTCCATGATCAAGCGCTACGACGAGCACCATCAGGATCTGACCCTCCTGAAGGCGCTGGTCAGGCAGCAGCTCCCCGAGAAGTACAAGGAGATCTTCTTCGATCAGTCGAAGAACGGCTACGCTGGGTACATTGACGGCGGGGCCTCTCAGGAGGAGTTCTACAAGTTCATCAAGCCGATTCTGGAGAAGATGGACGGCACGGAGGAGCTGCTGGTGAAGCTCAAGCGCGAGGACCTCCTGAGGAAGCAGCGGACATTCGATAACGGCAGCATCCCACACCAGATTCATCTCGGGGAGCTGCACGCTATCCTGAGGAGGCAGGAGGACTTCTACCCTTTCCTCAAGGATAACCGCGAGAAGATCGAGAAGATTCTGACTTTCAGGATCCCGTACTACGTCGGCCCACTCGCTAGGGGCAACTCCCGCTTCGCTTGGATGACCCGCAAGTCAGAGGAGACGATCACGCCGTGGAACTTCGAGGAGGTGGTCGACAAGGGCGCTAGCGCTCAGTCGTTCATCGAGAGGATGACGAATTTCGACAAGAACCTGCCAAATGAGAAGGTGCTCCCTAAGCACTCGCTCCTGTACGAGTACTTCACAGTCTACAACGAGCTGACTAAGGTGAAGTATGTGACCGAGGGCATGAGGAAGCCGGCTTTCCTGTCTGGGGAGCAGAAGAAGGCCATCGTGGACCTCCTGTTCAAGACCAACCGGAAGGTCACGGTTAAGCAGCTCAAGGAGGACTACTTCAAGAAGATTGAGTGCTTCGATTCGGTCGAGATCTCTGGCGTTGAGGACCGCTTCAACGCCTCCCTGGGGACCTACCACGATCTCCTGAAGATCATTAAGGATAAGGACTTCCTGGACAACGAGGAGAATGAGGATATCCTCGAGGACATTGTGCTGACACTCACTCTGTTCGAGGACCGGGAGATGATCGAGGAGCGCCTGAAGACTTACGCCCATCTCTTCGATGACAAGGTCATGAAGCAGCTCAAGAGGAGGAGGTACACCGGCTGGGGGAGGCTGAGCAGGAAGCTCATCAACGGCATTCGGGACAAGCAGTCCGGGAAGACGATCCTCGACTTCCTGAAGAGCGATGGCTTCGCGAACCGCAATTTCATGCAGCTGATTCACGATGACAGCCTCACATTCAAGGAGGATATCCAGAAGGCTCAGGTGAGCGGCCAGGGGGACTCGCTGCACGAGCATATCGCGAACCTCGCTGGCTCGCCAGCTATCAAGAAGGGGATTCTGCAGACCGTGAAGGTTGTGGACGAGCTGGTGAAGGTCATGGGCAGGCACAAGCCTGAGAACATCGTCATTGAGATGGCCCGGGAGAATCAGACCACGCAGAAGGGCCAGAAGAACTCACGCGAGAGGATGAAGAGGATCGAGGAGGGCATTAAGGAGCTGGGGTCCCAGATCCTCAAGGAGCACCCGGTGGAGAACACGCAGCTGCAGAATGAGAAGCTCTACCTGTACTACCTCCAGAATGGCCGCGATATGTATGTGGACCAGGAGCTGGATATTAACAGGCTCAGCGATTACGACGTCGATCACATCGTTCCACAGTCATTCCTGAAGGATGACTCCATTGACAACAAGGTCCTCACCAGGTCGGACAAGAACCGGGGCAAGTCTGATAATGTTCCTTCAGAGGAGGTCGTTAAGAAGATGAAGAACTACTGGCGCCAGCTCCTGAATGCCAAGCTGATCACGCAGCGGAAGTTCGATAACCTCACAAAGGCTGAGAGGGGCGGGCTCTCTGAGCTGGACAAGGCGGGCTTCATCAAGAGGCAGCTGGTCGAGACACGGCAGATCACTAAGCACGTTGCGCAGATTCTCGACTCACGGATGAACACTAAGTACGATGAGAATGACAAGCTGATCCGCGAGGTGAAGGTCATCACCCTGAAGTCAAAGCTCGTCTCCGACTTCAGGAAGGATTTCCAGTTCTACAAGGTTCGGGAGATCAACAATTACCACCATGCCCATGACGCGTACCTGAACGCGGTGGTCGGCACAGCTCTGATCAAGAAGTACCCAAAGCTCGAGAGCGAGTTCGTGTACGGGGACTACAAGGTTTACGATGTGAGGAAGATGATCGCCAAGTCGGAGCAGGAGATTGGCAAGGCTACCGCCAAGTACTTCTTCTACTCTAACATTATGAATTTCTTCAAGACAGAGATCACTCTGGCCAATGGCGAGATCCGGAAGCGCCCCCTCATCGAGACGAACGGCGAGACGGGGGAGATCGTGTGGGACAAGGGCAGGGATTTCGCGACCGTCAGGAAGGTTCTCTCCATGCCACAAGTGAATATCGTCAAGAAGACAGAGGTCCAGACTGGCGGGTTCTCTAAGGAGTCAATTCTGCCTAAGCGGAACAGCGACAAGCTCATCGCCCGCAAGAAGGACTGGGATCCGAAGAAGTACGGCGGGTTCGACAGCCCCACTGTGGCCTACTCGGTCCTGGTTGTGGCGAAGGTTGAGAAGGGCAAGTCCAAGAAGCTCAAGAGCGTGAAGGAGCTGCTGGGGATCACGATTATGGAGCGCTCCAGCTTCGAGAAGAACCCGATCGATTTCCTGGAGGCGAAGGGCTACAAGGAGGTGAAGAAGGACCTGATCATTAAGCTCCCCAAGTACTCACTCTTCGAGCTGGAGAACGGCAGGAAGCGGATGCTGGCTTCCGCTGGCGAGCTGCAGAAGGGGAACGAGCTGGCTCTGCCGTCCAAGTATGTGAACTTCCTCTACCTGGCCTCCCACTACGAGAAGCTCAAGGGCAGCCCCGAGGACAACGAGCAGAAGCAGCTGTTCGTCGAGCAGCACAAGCATTACCTCGACGAGATCATTGAGCAGATTTCCGAGTTCTCCAAGCGCGTGATCCTGGCCGACGCGAATCTGGATAAGGTCCTCTCCGCGTACAACAAGCACCGCGACAAGCCAATCAGGGAGCAGGCTGAGAATATCATTCATCTCTTCACCCTGACGAACCTCGGCGCCCCTGCTGCTTTCAAGTACTTCGACACAACTATCGATCGCAAGAGGTACACAAGCACTAAGGAGGTCCTGGACGCGACCCTCATCCACCAGTCGATTACCGGCCTCTACGAGACGCGCATCGACCTGTCTCAGCTCGGGGGCGACGAATTCTCCGGGAGCGAGACGCCAGGCACCTCCGAGTCGGCCACCCCAGAATCTGCCACAGTGGTGTCCGGCCAAAAGCAGGACCGCCAGGGCGGAGAACGCAGAAGGTCCCAGCTCGATAGGGATCAGTGTGCCTACTGCAAGGAGAAGGGCCACTGGGCCAAAGACTGCCCGAAAAAGCCGCGCGGCCCACGCGGCCCAAGGCCACAAACATCCCTCCTTCCAAAGAAGAAGCGGAAGGTGGAGCTCAGCGGAGGATCTTCCGGAGGATCTAGCGGCTCCGAGACACCAGGAACATCCGAAAGCGCTACACCAGAATCTAGCGGAGGCTCTTCCGGAGGATCTAGGCCTCCTCCCGTCGCGTGCCTCGTACTGAGCCTAGAGGAGGAGTACAGGCTGCATGAGCAAAATCCGAAGCAGCTGCTAGCCCCTGAATGGCTGAGCGCCTTCTCCGAGGTATGGGCGGAGCAGACTGGCATGGGACTGGCCAAGCAGGTGCCTCCTGTGGTGGTAGAGCTGAAGGCGGACGCCAGCCCAGTGAGCGTGAAACAATACCCGATGTCGAGGGAAGCAAAGGAAGGCATTAGACCCCACATCCAGAGACTGCTGCAATTGGGAATCCTGGTACCCTGCCAAAGCCCTTGGAACACGCCACTGCTGCCTGTGCGCAAACCAGGAACTAATGATTACAGACCTGTACAGGATCTAAGGGAAGTTAACAAGCGGGTGCAGGACATACATCCTACGGTCCCAAACCCCTACAACTTGCTGAGCTCTCTGCCCCCCGAGCGCACATGGTACACTGTGCTCGACTTAAAAGATGCGTTCTTCTGCCTTCGCCTCCACCCGAACTCACAGCCACTGTTCGCGTTCGAGTGGCGCGACCCTGAGGGCGGCCACACCGGGCAACTGACGTGGACACGGCTGCCACAGGGTTTCAAGAACAGCCCTACTCTATTTAACGAGGCCCTGCACCGTGATCTGGCCCCCTTCCGGGCGCAGAACCCTCAAATCAGTCTACTGCAGTATGCGGATGACCTGCTGCTAGCCGCTTCTACTCAGGAGCTCTGCCGGGAGGGCACAAAGCGGCTGTTAAATGAGCTGGGCGAGCTGGGATACCGCGTGTCTGCCAAGAAGGCACAATTGTGTCGGACAGAGGTGACATATCTCGGCTACACATTAAGAGAAGGTAAGAGATGGCTCACTGAAGCACGTAAGAAGACTGTCATGCAGATTCCTACCCCTACCACGCCCCGTCAGGTGCGGGAGTTCCTCGGCAAGGCCGGCTTCTGTAGACTGTTCATTCCTGGATTTGCCACCCTGGCCGCCCCACTCTACCTGCTGACGAAGCCAAAGGTTCCTTTCACCTGGACTGAGGAACATCAGCGGGCTTTCGAAGACATCAAAGCCGCCCTGCTGGCGGCTCCAGCTCTGGCCCTCCCTGACCTCACCAAGCCCTTCACACTCTATGTGGACGAGAGAGCTGGAGTGGCCAGAGGTGTGCTGACACAGGCACTGGGGCCGTGGAAGAGACCCGTGGCGTACCTATCTAAAAAGCTCGACCCCGTGGCCTCTGGGTGGCCGAGCTGTCTAAAGGCCATCGCTGCTGTAGCTCTACTGGTCAAAGATGCTGACAAGCTGACCCTGGGCCAGCATGTCACCGTCATCGCCCCGCACGCCTTAGAATCCATAGTCCGGCAACCACCAGATCGGTGGATGACAAACGCTAGGATGACGCATTATCAGAGCTTGCTGTTAAACGAAAGAGTGACATTTGCGCCCCCTGCCATCCTTAACCCAGCGACGCTACTGCCGGAGATCCACAACTCAACCCCCATCCACCAGTGCGTCGACATTCTGGCTGAGGAGACCGGCACGCGCAAGGACCTCACGGACAGACCATGGCCTGGTGTGCCGGCATGGTACACCGACGGCAGCTCCTTTGTCGTGGAGGGAAAGCGACGCGCAGGCGCGGCTGTGGTGGACGGCAAGCAAGTCATCTGGGCATCCTCCCTGCCAGAAGGCACCAGCGCTCAGAAAGCGGAGTTGGTGGCCTTGACCCAGGCCCTCAGATTAGCTGAAGGGAAGGCTATTAATATCTACACCGATTCTAGATACGCCTTCGCCACGGCGCACATCCACGGCGCGATCTACAAGCAGCGGGGCCTGCTGACCAGTGCCGGGCGAGACATCAAGAATAAGGAAGAGATTCTTGCCCTGCTGGAGGCCGTCCACCTCCCCAAAAAGGTGGCCATTATCCACTGCCCCGGCCACCAGAAGGGAGAGGATCCAATTACAAAGGGGAACCAGATGGCCGACCTCGTGGCGAAGCAGGTCGCTCAGCAGGTTACCATCCTGGCAGAAAAATCGCAGACCCCTGTGAAGACACCTGTGACAGATGATTCTAACTATAGCGGCGGCAGCAAAAGAACGGCGGACGGCTCTGAGAAGCGCACCGCTGATAGCCAGCATTCAACTCCTCCGAAAACAAAGAGGAAAGTTGAGTTCGAACCGAAGAAGAAAAGGAAGGTGGGAAGCGGAGCTACTAACTTCAGCCTGCTGAAGCAGGCTGGAGACGTGGAGGAGAACCCTGGACCTATGGACGAGCCTTCGCCACGCGGCGGCGGGTGCGCTGGCGAGCCACCACGCATCCGGAGGCTGGAGGAGTCTGTGGTGAACCGCATCGCTGCTGGCGAGGTGATCCAGAGGCCATCATCGGCTGTTAAGGAGCTGATCGAGAACAGCCTCGACGCTGGCGCCTCCAGCGTCTCCGTTGCGGTGAAGGACGGCGGCCTCAAGCTCATCCAGGTGTCCGATGACGGCCATGGCATCAGGTTCGAGGATCTGGCCATCCTGTGCGAGAGGCATACTACCTCAAAGCTGTCTGCTTACGAGGATCTGCAGACCATCAAGTCGATGGGGTTCAGGGGCGAGGCTCTGGCTAGCATGACTTATGTTGGCCATGTTACCGTGACAACGATCACAGAGGGCCAGCTGCACGGCTACAGGGTTTCTTACAGGGATGGCGTTATGGAGAATGAGCCTAAGCCTTGCGCTGCGGTGAAGGGCACTCAGGTCATGGTTGAGAATCTGTTCTACAACATGGTTGCCCGCAAGAAGACACTGCAGAACTCCAATGATGACTACCCCAAGATCGTCGACTTCATCAGCCGGTTCGCCGTCCACCACATCAACGTGACCTTCTCGTGCAGGAAGCATGGCGCCAACAGGGCTGATGTGCACAGCGCTAGCACGTCCTCGAGGCTCGATGCCATCAGGAGCGTCTACGGGGCTTCCGTCGTGCGCGATCTCATCGAGATCAAGGTGTCATACGAGGATGCTGCTGACTCCATCTTCAAGATGGATGGCTACATCTCCAATGCCAACTACGTGGCCAAGAAGATCACCATGATCCTGTTCATCAACGACAGGCTCGTTGACTGCACTGCTCTGAAGCGCGCTATTGAGTTCGTGTACTCTGCTACACTGCCGCAGGCGTCCAAGCCGTTCATCTACATGTCCATTCATCTGCCGTCAGAGCACGTGGATGTCAACATTCACCCGACCAAGAAGGAGGTTAGCCTGCTGAATCAGGAGCGCATCATTGAGACAATCAGGAACGCTATCGAGGAGAAGCTGATGAACTCCAACACCACCAGGATCTTCCAGACTCAGGCGCTCAACCTGTCAGGGATTGCTCAGGCTAACCCACAGAAGGATAAGGTTTCTGAGGCCAGCATGGGGTCTGGCACAAAGTCTCAGAAGATTCCTGTGAGCCAGATGGTCAGGACAGATCCACGCAATCCATCTGGCAGGCTGCACACCTACTGGCACGGGCAGTCTTCAAATCTCGAGAAGAAGTTCGATCTCGTGTCTGTTAGGAATGTTGTTAGGTCAAGGAGGAACCAGAAGGATGCTGGCGATCTGTCAAGCCGCCATGAGCTGCTCGTGGAGATTGATTCTAGCTTCCATCCTGGCCTGCTGGACATTGTCAAGAACTGCACATATGTTGGGCTCGCCGATGAGGCGTTCGCTCTGATTCAGCACAATACCCGCCTGTACCTCGTCAATGTGGTCAATATTAGCAAGGAGCTGATGTACCAGCAGGCTCTGTGCCGCTTCGGGAACTTCAATGCTATTCAGCTCAGCGAGCCAGCTCCACTGCAGGAGCTGCTGGTGATGGCTCTGAAGGACGATGAGCTGATGAGCGATGAGAAGGATGATGAGAAGCTGGAGATTGCTGAGGTCAACACTGAGATTCTCAAGGAGAATGCTGAGATGATTAATGAGTACTTCTCAATTCACATTGATCAGGATGGCAAGCTGACAAGGCTGCCTGTTGTTCTGGACCAGTACACCCCTGATATGGACCGGCTGCCAGAGTTCGTGCTGGCTCTCGGCAATGATGTTACTTGGGATGACGAGAAGGAGTGCTTCAGGACAGTTGCTTCTGCTGTTGGCAACTTCTATGCGCTGCATCCCCCAATCCTGCCAAATCCATCTGGGAATGGCATTCATCTGTACAAGAAGAATAGGGATTCAATGGCTGATGAGCATGCTGAGAATGATCTCATCTCAGATGAGAATGACGTTGATCAGGAGCTGCTCGCGGAGGCTGAGGCTGCTTGGGCCCAGCGCGAGTGGACCATTCAGCATGTCCTGTTCCCATCCATGCGGCTGTTCCTCAAGCCCCCGAAGTCAATGGCTACAGATGGCACGTTCGTGCAGGTTGCTTCCCTGGAGAAGCTCTACAAGATCTTCTGA

**Sequence 69 Plasmids sequence of PE-RERV-m5-SB2**

(NLSc-myc-NLSSV40-nCas9(H840A/R221K/N394K)-XTEN-NC-NLS-32aa Linker-RERV-RT-m5-NLSSV40-P2A-OsMLH1-SB-NLSSV40-NLSc-myc-P2A-OsMLH1-SB)

AAGCTTACTGGTTGTACAATGCCGGCGGCCAAGAGAGTCAAGCTCGACGGCGGGAAGCGCACAGCTGATGGTTCTGAATTCGAGTCCCCTAAGAAAAAGAGAAAAGTGGACAAGAAGTACTCGATCGGCCTCGATATTGGGACTAACTCTGTTGGCTGGGCCGTGATCACCGACGAGTACAAGGTGCCCTCAAAGAAGTTCAAGGTCCTGGGCAACACCGATCGGCATTCCATCAAGAAGAATCTCATTGGCGCTCTCCTGTTCGACAGCGGCGAGACGGCTGAGGCTACGCGGCTCAAGCGCACCGCCCGCAGGCGGTACACGCGCAGGAAGAATCGCATCTGCTACCTGCAGGAGATTTTCTCCAACGAGATGGCGAAGGTTGACGATTCTTTCTTCCACAGGCTGGAGGAGTCATTCCTCGTGGAGGAGGATAAGAAGCACGAGCGGCATCCAATCTTCGGCAACATTGTCGACGAGGTTGCCTACCACGAGAAGTACCCTACGATCTACCATCTGCGGAAGAAGCTCGTGGACTCCACAGATAAGGCGGACCTCCGCCTGATCTACCTCGCTCTGGCCCACATGATTAAGTTCAGGGGCCATTTCCTGATCGAGGGGGATCTCAACCCGGACAATAGCGATGTTGACAAGCTGTTCATCCAGCTCGTGCAGACGTACAACCAGCTCTTCGAGGAGAACCCCATTAATGCGTCAGGCGTCGACGCGAAGGCTATCCTGTCCGCTAGGCTCTCGAAGTCTCGGAAGCTCGAGAACCTGATCGCCCAGCTGCCGGGCGAGAAGAAGAACGGCCTGTTCGGGAATCTCATTGCGCTCAGCCTGGGGCTCACGCCCAACTTCAAGTCGAATTTCGATCTCGCTGAGGACGCCAAGCTGCAGCTCTCCAAGGACACATACGACGATGACCTGGATAACCTCCTGGCCCAGATCGGCGATCAGTACGCGGACCTGTTCCTCGCTGCCAAGAATCTGTCGGACGCCATCCTCCTGTCTGATATTCTCAGGGTGAACACCGAGATTACGAAGGCTCCGCTCTCAGCCTCCATGATCAAGCGCTACGACGAGCACCATCAGGATCTGACCCTCCTGAAGGCGCTGGTCAGGCAGCAGCTCCCCGAGAAGTACAAGGAGATCTTCTTCGATCAGTCGAAGAACGGCTACGCTGGGTACATTGACGGCGGGGCCTCTCAGGAGGAGTTCTACAAGTTCATCAAGCCGATTCTGGAGAAGATGGACGGCACGGAGGAGCTGCTGGTGAAGCTCAAGCGCGAGGACCTCCTGAGGAAGCAGCGGACATTCGATAACGGCAGCATCCCACACCAGATTCATCTCGGGGAGCTGCACGCTATCCTGAGGAGGCAGGAGGACTTCTACCCTTTCCTCAAGGATAACCGCGAGAAGATCGAGAAGATTCTGACTTTCAGGATCCCGTACTACGTCGGCCCACTCGCTAGGGGCAACTCCCGCTTCGCTTGGATGACCCGCAAGTCAGAGGAGACGATCACGCCGTGGAACTTCGAGGAGGTGGTCGACAAGGGCGCTAGCGCTCAGTCGTTCATCGAGAGGATGACGAATTTCGACAAGAACCTGCCAAATGAGAAGGTGCTCCCTAAGCACTCGCTCCTGTACGAGTACTTCACAGTCTACAACGAGCTGACTAAGGTGAAGTATGTGACCGAGGGCATGAGGAAGCCGGCTTTCCTGTCTGGGGAGCAGAAGAAGGCCATCGTGGACCTCCTGTTCAAGACCAACCGGAAGGTCACGGTTAAGCAGCTCAAGGAGGACTACTTCAAGAAGATTGAGTGCTTCGATTCGGTCGAGATCTCTGGCGTTGAGGACCGCTTCAACGCCTCCCTGGGGACCTACCACGATCTCCTGAAGATCATTAAGGATAAGGACTTCCTGGACAACGAGGAGAATGAGGATATCCTCGAGGACATTGTGCTGACACTCACTCTGTTCGAGGACCGGGAGATGATCGAGGAGCGCCTGAAGACTTACGCCCATCTCTTCGATGACAAGGTCATGAAGCAGCTCAAGAGGAGGAGGTACACCGGCTGGGGGAGGCTGAGCAGGAAGCTCATCAACGGCATTCGGGACAAGCAGTCCGGGAAGACGATCCTCGACTTCCTGAAGAGCGATGGCTTCGCGAACCGCAATTTCATGCAGCTGATTCACGATGACAGCCTCACATTCAAGGAGGATATCCAGAAGGCTCAGGTGAGCGGCCAGGGGGACTCGCTGCACGAGCATATCGCGAACCTCGCTGGCTCGCCAGCTATCAAGAAGGGGATTCTGCAGACCGTGAAGGTTGTGGACGAGCTGGTGAAGGTCATGGGCAGGCACAAGCCTGAGAACATCGTCATTGAGATGGCCCGGGAGAATCAGACCACGCAGAAGGGCCAGAAGAACTCACGCGAGAGGATGAAGAGGATCGAGGAGGGCATTAAGGAGCTGGGGTCCCAGATCCTCAAGGAGCACCCGGTGGAGAACACGCAGCTGCAGAATGAGAAGCTCTACCTGTACTACCTCCAGAATGGCCGCGATATGTATGTGGACCAGGAGCTGGATATTAACAGGCTCAGCGATTACGACGTCGATCACATCGTTCCACAGTCATTCCTGAAGGATGACTCCATTGACAACAAGGTCCTCACCAGGTCGGACAAGAACCGGGGCAAGTCTGATAATGTTCCTTCAGAGGAGGTCGTTAAGAAGATGAAGAACTACTGGCGCCAGCTCCTGAATGCCAAGCTGATCACGCAGCGGAAGTTCGATAACCTCACAAAGGCTGAGAGGGGCGGGCTCTCTGAGCTGGACAAGGCGGGCTTCATCAAGAGGCAGCTGGTCGAGACACGGCAGATCACTAAGCACGTTGCGCAGATTCTCGACTCACGGATGAACACTAAGTACGATGAGAATGACAAGCTGATCCGCGAGGTGAAGGTCATCACCCTGAAGTCAAAGCTCGTCTCCGACTTCAGGAAGGATTTCCAGTTCTACAAGGTTCGGGAGATCAACAATTACCACCATGCCCATGACGCGTACCTGAACGCGGTGGTCGGCACAGCTCTGATCAAGAAGTACCCAAAGCTCGAGAGCGAGTTCGTGTACGGGGACTACAAGGTTTACGATGTGAGGAAGATGATCGCCAAGTCGGAGCAGGAGATTGGCAAGGCTACCGCCAAGTACTTCTTCTACTCTAACATTATGAATTTCTTCAAGACAGAGATCACTCTGGCCAATGGCGAGATCCGGAAGCGCCCCCTCATCGAGACGAACGGCGAGACGGGGGAGATCGTGTGGGACAAGGGCAGGGATTTCGCGACCGTCAGGAAGGTTCTCTCCATGCCACAAGTGAATATCGTCAAGAAGACAGAGGTCCAGACTGGCGGGTTCTCTAAGGAGTCAATTCTGCCTAAGCGGAACAGCGACAAGCTCATCGCCCGCAAGAAGGACTGGGATCCGAAGAAGTACGGCGGGTTCGACAGCCCCACTGTGGCCTACTCGGTCCTGGTTGTGGCGAAGGTTGAGAAGGGCAAGTCCAAGAAGCTCAAGAGCGTGAAGGAGCTGCTGGGGATCACGATTATGGAGCGCTCCAGCTTCGAGAAGAACCCGATCGATTTCCTGGAGGCGAAGGGCTACAAGGAGGTGAAGAAGGACCTGATCATTAAGCTCCCCAAGTACTCACTCTTCGAGCTGGAGAACGGCAGGAAGCGGATGCTGGCTTCCGCTGGCGAGCTGCAGAAGGGGAACGAGCTGGCTCTGCCGTCCAAGTATGTGAACTTCCTCTACCTGGCCTCCCACTACGAGAAGCTCAAGGGCAGCCCCGAGGACAACGAGCAGAAGCAGCTGTTCGTCGAGCAGCACAAGCATTACCTCGACGAGATCATTGAGCAGATTTCCGAGTTCTCCAAGCGCGTGATCCTGGCCGACGCGAATCTGGATAAGGTCCTCTCCGCGTACAACAAGCACCGCGACAAGCCAATCAGGGAGCAGGCTGAGAATATCATTCATCTCTTCACCCTGACGAACCTCGGCGCCCCTGCTGCTTTCAAGTACTTCGACACAACTATCGATCGCAAGAGGTACACAAGCACTAAGGAGGTCCTGGACGCGACCCTCATCCACCAGTCGATTACCGGCCTCTACGAGACGCGCATCGACCTGTCTCAGCTCGGGGGCGACGAATTCTCCGGGAGCGAGACGCCAGGCACCTCCGAGTCGGCCACCCCAGAATCTGCCACAGTGGTGTCCGGCCAAAAGCAGGACCGCCAGGGCGGAGAACGCAGAAGGTCCCAGCTCGATAGGGATCAGTGTGCCTACTGCAAGGAGAAGGGCCACTGGGCCAAAGACTGCCCGAAAAAGCCGCGCGGCCCACGCGGCCCAAGGCCACAAACATCCCTCCTTCCAAAGAAGAAGCGGAAGGTGGAGCTCAGCGGAGGATCTTCCGGAGGATCTAGCGGCTCCGAGACACCAGGAACATCCGAAAGCGCTACACCAGAATCTAGCGGAGGCTCTTCCGGAGGATCTAGGCCTCCTCCCGTCGCGTGCCTCGTACTGAGCCTAGAGGAGGAGTACAGGCTGCATGAGCAAAATCCGAAGCAGCTGCTAGCCCCTGAATGGCTGAGCGCCTTCTCCGAGGTATGGGCGGAGCAGACTGGCATGGGACTGGCCAAGCAGGTGCCTCCTGTGGTGGTAGAGCTGAAGGCGGACGCCAGCCCAGTGAGCGTGAAACAATACCCGATGTCGAGGGAAGCAAAGGAAGGCATTAGACCCCACATCCAGAGACTGCTGCAATTGGGAATCCTGGTACCCTGCCAAAGCCCTTGGAACACGCCACTGCTGCCTGTGCGCAAACCAGGAACTAATGATTACAGACCTGTACAGGATCTAAGGGAAGTTAACAAGCGGGTGCAGGACATACATCCTACGGTCCCAAACCCCTACAACTTGCTGAGCTCTCTGCCCCCCGAGCGCACATGGTACACTGTGCTCGACTTAAAAGATGCGTTCTTCTGCCTTCGCCTCCACCCGAACTCACAGCCACTGTTCGCGTTCGAGTGGCGCGACCCTGAGGGCGGCCACACCGGGCAACTGACGTGGACACGGCTGCCACAGGGTTTCAAGAACAGCCCTACTCTATTTAACGAGGCCCTGCACCGTGATCTGGCCCCCTTCCGGGCGCAGAACCCTCAAATCAGTCTACTGCAGTATGCGGATGACCTGCTGCTAGCCGCTTCTACTCAGGAGCTCTGCCGGGAGGGCACAAAGCGGCTGTTAAATGAGCTGGGCGAGCTGGGATACCGCGTGTCTGCCAAGAAGGCACAATTGTGTCGGACAGAGGTGACATATCTCGGCTACACATTAAGAGAAGGTAAGAGATGGCTCACTGAAGCACGTAAGAAGACTGTCATGCAGATTCCTACCCCTACCACGCCCCGTCAGGTGCGGGAGTTCCTCGGCAAGGCCGGCTTCTGTAGACTGTTCATTCCTGGATTTGCCACCCTGGCCGCCCCACTCTACCTGCTGACGAAGCCAAAGGTTCCTTTCACCTGGACTGAGGAACATCAGCGGGCTTTCGAAGACATCAAAGCCGCCCTGCTGGCGGCTCCAGCTCTGGCCCTCCCTGACCTCACCAAGCCCTTCACACTCTATGTGGACGAGAGAGCTGGAGTGGCCAGAGGTGTGCTGACACAGGCACTGGGGCCGTGGAAGAGACCCGTGGCGTACCTATCTAAAAAGCTCGACCCCGTGGCCTCTGGGTGGCCGAGCTGTCTAAAGGCCATCGCTGCTGTAGCTCTACTGGTCAAAGATGCTGACAAGCTGACCCTGGGCCAGCATGTCACCGTCATCGCCCCGCACGCCTTAGAATCCATAGTCCGGCAACCACCAGATCGGTGGATGACAAACGCTAGGATGACGCATTATCAGAGCTTGCTGTTAAACGAAAGAGTGACATTTGCGCCCCCTGCCATCCTTAACCCAGCGACGCTACTGCCGGAGATCCACAACTCAACCCCCATCCACCAGTGCGTCGACATTCTGGCTGAGGAGACCGGCACGCGCAAGGACCTCACGGACAGACCATGGCCTGGTGTGCCGGCATGGTACACCGACGGCAGCTCCTTTGTCGTGGAGGGAAAGCGACGCGCAGGCGCGGCTGTGGTGGACGGCAAGCAAGTCATCTGGGCATCCTCCCTGCCAGAAGGCACCAGCGCTCAGAAAGCGGAGTTGGTGGCCTTGACCCAGGCCCTCAGATTAGCTGAAGGGAAGGCTATTAATATCTACACCGATTCTAGATACGCCTTCGCCACGGCGCACATCCACGGCGCGATCTACAAGCAGCGGGGCCTGCTGACCAGTGCCGGGCGAGACATCAAGAATAAGGAAGAGATTCTTGCCCTGCTGGAGGCCGTCCACCTCCCCAAAAAGGTGGCCATTATCCACTGCCCCGGCCACCAGAAGGGAGAGGATCCAATTACAAAGGGGAACCAGATGGCCGACCTCGTGGCGAAGCAGGTCGCTCAGCAGGTTACCATCCTGGCAGAAAAATCGCAGACCCCTGTGAAGACACCTGTGACAGATGATTCTAACTATAGCGGCGGCAGCAAAAGAACGGCGGACGGCTCTGAGAAGCGCACCGCTGATAGCCAGCATTCAACTCCTCCGAAAACAAAGAGGAAAGTTGAGTTCGAACCGAAGAAGAAAAGGAAGGTGGGAAGCGGAGCTACTAACTTCAGCCTGCTGAAGCAGGCTGGAGACGTGGAGGAGAACCCTGGACCTATGTCCGAGGAGGAGGAGAAGAGGAAGATTGAAGAGGGCATGAGGCGGCTCCGCGAGAATTTGAAGAAAAAAAGAAAAGAAGAAGAGGAAAAAGAGAAGAAGGAAACAGAAGAGCTCGCCAAGCTTGCTAAAGAAATAGTGGAGGAAGCAAAGAAAGAGCTGAAGGCGGAGGGGAAGTGGAAGAACAAGGAGGAGATCTGGGAGGCGTTCAAGAAAAAGATCTTAGAGAAATTTGAAAAGCTGGAGAAAAGAACCGCCGACGGCAGCGAATTCGAGTCTCCCAAGAAGAAGAGGAAAGTCGGCTCTGGCCCTGCCGCTAAGAGAGTGAAGCTGGACGGAAGCGGAGCAACAAACTTCTCTCTGCTGAAACAAGCCGGAGATGTCGAAGAGAATCCTGGACCGATGTCCGAGGAGGAGGAGAAGAGGAAGATTGAAGAGGGCATGAGGCGGCTCCGCGAGAATTTGAAGAAAAAAAGAAAAGAAGAAGAGGAAAAAGAGAAGAAGGAAACAGAAGAGCTCGCCAAGCTTGCTAAAGAAATAGTGGAGGAAGCAAAGAAAGAGCTGAAGGCGGAGGGGAAGTGGAAGAACAAGGAGGAGATCTGGGAGGCGTTCAAGAAAAAGATCTTAGAGAAATTTGAAAAGCTGGAGGGAAGCGGAGTTTAA

**Sequence 70 Plasmids sequence of PE-RERV-m5-MCP**

(NLSc-myc-NLSSV40-nCas9(H840A/R221K/N394K)-NLS-T2A-MCP-NLSSV40-XTEN-NC-NLS-32aa Linker-RERV-RT-m5-NLS)

AAGCTTACTGGTTGTACAATGCCGGCGGCCAAGAGAGTCAAGCTCGACGGCGGGAAGCGCACAGCTGATGGTTCTGAATTCGAGTCCCCTAAGAAAAAGAGAAAAGTGGACAAGAAGTACTCGATCGGCCTCGATATTGGGACTAACTCTGTTGGCTGGGCCGTGATCACCGACGAGTACAAGGTGCCCTCAAAGAAGTTCAAGGTCCTGGGCAACACCGATCGGCATTCCATCAAGAAGAATCTCATTGGCGCTCTCCTGTTCGACAGCGGCGAGACGGCTGAGGCTACGCGGCTCAAGCGCACCGCCCGCAGGCGGTACACGCGCAGGAAGAATCGCATCTGCTACCTGCAGGAGATTTTCTCCAACGAGATGGCGAAGGTTGACGATTCTTTCTTCCACAGGCTGGAGGAGTCATTCCTCGTGGAGGAGGATAAGAAGCACGAGCGGCATCCAATCTTCGGCAACATTGTCGACGAGGTTGCCTACCACGAGAAGTACCCTACGATCTACCATCTGCGGAAGAAGCTCGTGGACTCCACAGATAAGGCGGACCTCCGCCTGATCTACCTCGCTCTGGCCCACATGATTAAGTTCAGGGGCCATTTCCTGATCGAGGGGGATCTCAACCCGGACAATAGCGATGTTGACAAGCTGTTCATCCAGCTCGTGCAGACGTACAACCAGCTCTTCGAGGAGAACCCCATTAATGCGTCAGGCGTCGACGCGAAGGCTATCCTGTCCGCTAGGCTCTCGAAGTCTCGGAAGCTCGAGAACCTGATCGCCCAGCTGCCGGGCGAGAAGAAGAACGGCCTGTTCGGGAATCTCATTGCGCTCAGCCTGGGGCTCACGCCCAACTTCAAGTCGAATTTCGATCTCGCTGAGGACGCCAAGCTGCAGCTCTCCAAGGACACATACGACGATGACCTGGATAACCTCCTGGCCCAGATCGGCGATCAGTACGCGGACCTGTTCCTCGCTGCCAAGAATCTGTCGGACGCCATCCTCCTGTCTGATATTCTCAGGGTGAACACCGAGATTACGAAGGCTCCGCTCTCAGCCTCCATGATCAAGCGCTACGACGAGCACCATCAGGATCTGACCCTCCTGAAGGCGCTGGTCAGGCAGCAGCTCCCCGAGAAGTACAAGGAGATCTTCTTCGATCAGTCGAAGAACGGCTACGCTGGGTACATTGACGGCGGGGCCTCTCAGGAGGAGTTCTACAAGTTCATCAAGCCGATTCTGGAGAAGATGGACGGCACGGAGGAGCTGCTGGTGAAGCTCAAGCGCGAGGACCTCCTGAGGAAGCAGCGGACATTCGATAACGGCAGCATCCCACACCAGATTCATCTCGGGGAGCTGCACGCTATCCTGAGGAGGCAGGAGGACTTCTACCCTTTCCTCAAGGATAACCGCGAGAAGATCGAGAAGATTCTGACTTTCAGGATCCCGTACTACGTCGGCCCACTCGCTAGGGGCAACTCCCGCTTCGCTTGGATGACCCGCAAGTCAGAGGAGACGATCACGCCGTGGAACTTCGAGGAGGTGGTCGACAAGGGCGCTAGCGCTCAGTCGTTCATCGAGAGGATGACGAATTTCGACAAGAACCTGCCAAATGAGAAGGTGCTCCCTAAGCACTCGCTCCTGTACGAGTACTTCACAGTCTACAACGAGCTGACTAAGGTGAAGTATGTGACCGAGGGCATGAGGAAGCCGGCTTTCCTGTCTGGGGAGCAGAAGAAGGCCATCGTGGACCTCCTGTTCAAGACCAACCGGAAGGTCACGGTTAAGCAGCTCAAGGAGGACTACTTCAAGAAGATTGAGTGCTTCGATTCGGTCGAGATCTCTGGCGTTGAGGACCGCTTCAACGCCTCCCTGGGGACCTACCACGATCTCCTGAAGATCATTAAGGATAAGGACTTCCTGGACAACGAGGAGAATGAGGATATCCTCGAGGACATTGTGCTGACACTCACTCTGTTCGAGGACCGGGAGATGATCGAGGAGCGCCTGAAGACTTACGCCCATCTCTTCGATGACAAGGTCATGAAGCAGCTCAAGAGGAGGAGGTACACCGGCTGGGGGAGGCTGAGCAGGAAGCTCATCAACGGCATTCGGGACAAGCAGTCCGGGAAGACGATCCTCGACTTCCTGAAGAGCGATGGCTTCGCGAACCGCAATTTCATGCAGCTGATTCACGATGACAGCCTCACATTCAAGGAGGATATCCAGAAGGCTCAGGTGAGCGGCCAGGGGGACTCGCTGCACGAGCATATCGCGAACCTCGCTGGCTCGCCAGCTATCAAGAAGGGGATTCTGCAGACCGTGAAGGTTGTGGACGAGCTGGTGAAGGTCATGGGCAGGCACAAGCCTGAGAACATCGTCATTGAGATGGCCCGGGAGAATCAGACCACGCAGAAGGGCCAGAAGAACTCACGCGAGAGGATGAAGAGGATCGAGGAGGGCATTAAGGAGCTGGGGTCCCAGATCCTCAAGGAGCACCCGGTGGAGAACACGCAGCTGCAGAATGAGAAGCTCTACCTGTACTACCTCCAGAATGGCCGCGATATGTATGTGGACCAGGAGCTGGATATTAACAGGCTCAGCGATTACGACGTCGATGCCATCGTTCCACAGTCATTCCTGAAGGATGACTCCATTGACAACAAGGTCCTCACCAGGTCGGACAAGAACCGGGGCAAGTCTGATAATGTTCCTTCAGAGGAGGTCGTTAAGAAGATGAAGAACTACTGGCGCCAGCTCCTGAATGCCAAGCTGATCACGCAGCGGAAGTTCGATAACCTCACAAAGGCTGAGAGGGGCGGGCTCTCTGAGCTGGACAAGGCGGGCTTCATCAAGAGGCAGCTGGTCGAGACACGGCAGATCACTAAGCACGTTGCGCAGATTCTCGACTCACGGATGAACACTAAGTACGATGAGAATGACAAGCTGATCCGCGAGGTGAAGGTCATCACCCTGAAGTCAAAGCTCGTCTCCGACTTCAGGAAGGATTTCCAGTTCTACAAGGTTCGGGAGATCAACAATTACCACCATGCCCATGACGCGTACCTGAACGCGGTGGTCGGCACAGCTCTGATCAAGAAGTACCCAAAGCTCGAGAGCGAGTTCGTGTACGGGGACTACAAGGTTTACGATGTGAGGAAGATGATCGCCAAGTCGGAGCAGGAGATTGGCAAGGCTACCGCCAAGTACTTCTTCTACTCTAACATTATGAATTTCTTCAAGACAGAGATCACTCTGGCCAATGGCGAGATCCGGAAGCGCCCCCTCATCGAGACGAACGGCGAGACGGGGGAGATCGTGTGGGACAAGGGCAGGGATTTCGCGACCGTCAGGAAGGTTCTCTCCATGCCACAAGTGAATATCGTCAAGAAGACAGAGGTCCAGACTGGCGGGTTCTCTAAGGAGTCAATTCTGCCTAAGCGGAACAGCGACAAGCTCATCGCCCGCAAGAAGGACTGGGATCCGAAGAAGTACGGCGGGTTCGACAGCCCCACTGTGGCCTACTCGGTCCTGGTTGTGGCGAAGGTTGAGAAGGGCAAGTCCAAGAAGCTCAAGAGCGTGAAGGAGCTGCTGGGGATCACGATTATGGAGCGCTCCAGCTTCGAGAAGAACCCGATCGATTTCCTGGAGGCGAAGGGCTACAAGGAGGTGAAGAAGGACCTGATCATTAAGCTCCCCAAGTACTCACTCTTCGAGCTGGAGAACGGCAGGAAGCGGATGCTGGCTTCCGCTGGCGAGCTGCAGAAGGGGAACGAGCTGGCTCTGCCGTCCAAGTATGTGAACTTCCTCTACCTGGCCTCCCACTACGAGAAGCTCAAGGGCAGCCCCGAGGACAACGAGCAGAAGCAGCTGTTCGTCGAGCAGCACAAGCATTACCTCGACGAGATCATTGAGCAGATTTCCGAGTTCTCCAAGCGCGTGATCCTGGCCGACGCGAATCTGGATAAGGTCCTCTCCGCGTACAACAAGCACCGCGACAAGCCAATCAGGGAGCAGGCTGAGAATATCATTCATCTCTTCACCCTGACGAACCTCGGCGCCCCTGCTGCTTTCAAGTACTTCGACACAACTATCGATCGCAAGAGGTACACAAGCACTAAGGAGGTCCTGGACGCGACCCTCATCCACCAGTCGATTACCGGCCTCTACGAGACGCGCATCGACCTGTCTCAGCTCGGGGGCGACGAATTCAAGAGACCAGCGGCAACCAAGAAGGCAGGACAAGCGAAGAAGAAGAAGGGCGGCCGCGAGGGCAGAGGAAGTCTTCTAACATGCGGTGACGTGGAGGAGAATCCCGGCCCTGCCTCAAACTTCACCCAGTTCGTGCTCGTCGACAATGGCGGAACCGGCGATGTGACAGTCGCACCTTCCAACTTCGCCAATGGCATCGCGGAGTGGATTTCCAGCAACTCCAGGTCCCAGGCGTACAAGGTGACCTGCTCCGTCAGGCAGTCATCTGCGCAGAATCGCAAGTACACAATCAAGGTGGAGGTCCCTAAGGGCGCCTGGAGGTCCTACCTCAACATGGAGCTGACCATCCCGATTTTCGCCACAAATAGCGACTGCGAGCTGATCGTGAAGGCGATGCAGGGCCTCCTGAAGGATGGCAACCCGATCCCATCAGCGATTGCCGCCAATTCTGGCATCTACGGAGGTTCTCCAAAGAAGAAGAGGAAGGTTTCAGGCTCTGAGACACCGGGCACATCCGAGTCAGCAACACCAGAGTCCGCCACAGTGGTGTCCGGCCAAAAGCAGGACCGCCAGGGCGGAGAACGCAGAAGGTCCCAGCTCGATAGGGATCAGTGTGCCTACTGCAAGGAGAAGGGCCACTGGGCCAAAGACTGCCCGAAAAAGCCGCGCGGCCCACGCGGCCCAAGGCCACAAACATCCCTCCTTCCAAAGAAGAAGCGGAAGGTGGAGCTCAGCGGAGGATCTTCCGGAGGATCTAGCGGCTCCGAGACACCAGGAACATCCGAAAGCGCTACACCAGAATCTAGCGGAGGCTCTTCCGGAGGATCTAGGCCTCCTCCCGTCGCGTGCCTCGTACTGAGCCTAGAGGAGGAGTACAGGCTGCATGAGCAAAATCCGAAGCAGCTGCTAGCCCCTGAATGGCTGAGCGCCTTCTCCGAGGTATGGGCGGAGCAGACTGGCATGGGACTGGCCAAGCAGGTGCCTCCTGTGGTGGTAGAGCTGAAGGCGGACGCCAGCCCAGTGAGCGTGAAACAATACCCGATGTCGAGGGAAGCAAAGGAAGGCATTAGACCCCACATCCAGAGACTGCTGCAATTGGGAATCCTGGTACCCTGCCAAAGCCCTTGGAACACGCCACTGCTGCCTGTGCGCAAACCAGGAACTAATGATTACAGACCTGTACAGGATCTAAGGGAAGTTAACAAGCGGGTGCAGGACATACATCCTACGGTCCCAAACCCCTACAACTTGCTGAGCTCTCTGCCCCCCGAGCGCACATGGTACACTGTGCTCGACTTAAAAGATGCGTTCTTCTGCCTTCGCCTCCACCCGAACTCACAGCCACTGTTCGCGTTCGAGTGGCGCGACCCTGAGGGCGGCCACACCGGGCAACTGACGTGGACACGGCTGCCACAGGGTTTCAAGAACAGCCCTACTCTATTTAACGAGGCCCTGCACCGTGATCTGGCCCCCTTCCGGGCGCAGAACCCTCAAATCAGTCTACTGCAGTATGCGGATGACCTGCTGCTAGCCGCTTCTACTCAGGAGCTCTGCCGGGAGGGCACAAAGCGGCTGTTAAATGAGCTGGGCGAGCTGGGATACCGCGTGTCTGCCAAGAAGGCACAATTGTGTCGGACAGAGGTGACATATCTCGGCTACACATTAAGAGAAGGTAAGAGATGGCTCACTGAAGCACGTAAGAAGACTGTCATGCAGATTCCTACCCCTACCACGCCCCGTCAGGTGCGGGAGTTCCTCGGCAAGGCCGGCTTCTGTAGACTGTTCATTCCTGGATTTGCCACCCTGGCCGCCCCACTCTACCTGCTGACGAAGCCAAAGGTTCCTTTCACCTGGACTGAGGAACATCAGCGGGCTTTCGAAGACATCAAAGCCGCCCTGCTGGCGGCTCCAGCTCTGGCCCTCCCTGACCTCACCAAGCCCTTCACACTCTATGTGGACGAGAGAGCTGGAGTGGCCAGAGGTGTGCTGACACAGGCACTGGGGCCGTGGAAGAGACCCGTGGCGTACCTATCTAAAAAGCTCGACCCCGTGGCCTCTGGGTGGCCGAGCTGTCTAAAGGCCATCGCTGCTGTAGCTCTACTGGTCAAAGATGCTGACAAGCTGACCCTGGGCCAGCATGTCACCGTCATCGCCCCGCACGCCTTAGAATCCATAGTCCGGCAACCACCAGATCGGTGGATGACAAACGCTAGGATGACGCATTATCAGAGCTTGCTGTTAAACGAAAGAGTGACATTTGCGCCCCCTGCCATCCTTAACCCAGCGACGCTACTGCCGGAGATCCACAACTCAACCCCCATCCACCAGTGCGTCGACATTCTGGCTGAGGAGACCGGCACGCGCAAGGACCTCACGGACAGACCATGGCCTGGTGTGCCGGCATGGTACACCGACGGCAGCTCCTTTGTCGTGGAGGGAAAGCGACGCGCAGGCGCGGCTGTGGTGGACGGCAAGCAAGTCATCTGGGCATCCTCCCTGCCAGAAGGCACCAGCGCTCAGAAAGCGGAGTTGGTGGCCTTGACCCAGGCCCTCAGATTAGCTGAAGGGAAGGCTATTAATATCTACACCGATTCTAGATACGCCTTCGCCACGGCGCACATCCACGGCGCGATCTACAAGCAGCGGGGCCTGCTGACCAGTGCCGGGCGAGACATCAAGAATAAGGAAGAGATTCTTGCCCTGCTGGAGGCCGTCCACCTCCCCAAAAAGGTGGCCATTATCCACTGCCCCGGCCACCAGAAGGGAGAGGATCCAATTACAAAGGGGAACCAGATGGCCGACCTCGTGGCGAAGCAGGTCGCTCAGCAGGTTACCATCCTGGCAGAAAAATCGCAGACCCCTGTGAAGACACCTGTGACAGATGATTCTAACTATAGCGGCGGCAGCAAAAGAACGGCGGACGGCTCTGAGAAGCGCACCGCTGATAGCCAGCATTCAACTCCTCCGAAAACAAAGAGGAAAGTTGAGTTCGAACCGAAGAAGAAAAGGAAGGTGGGCTCCGGCGTACGGAATCGGTTCTTAAGTGGCGGCTCCAAACGGCCAGCGGCGACGAAGAAGGCGGGGCAGGCGAAGAAGAAGAAGTAG

**Sequence 71 Plasmids sequence of pegRNA-MS2**

(35C promoter-tRNAGly RS-spacer-sgRNA scaffold-MS2/f6-RTT+PBS-tevopreQ1-polyT-HDV)

ATGGAGTCAAAGATTCAAATAGAGGACCTAACAGAACTCGCCGTAAAGACTGGCGAACAGTTCATACAGAGTCTCTTACGACTCAATGACAAGAAGAAAATCTTCGTCAACATGGTGGAGCACGACACACTTGTCTACTCCAAAAATATCAAAGATACAGTCTCAGAAGACCAAAGGGCAATTGAGACTTTTCAACAAAGGGTAATATCCGGAAACCTCCTCGGATTCCATTGCCCAGCTATCTGTCACTTTATTGTGAAGATAGTGGAAAAGGAAGGTGGCTCCTACAAATGCCATCATTGCGATAAAGGAAAGGCCATCGTTGAAGATGCCTCTGCCGACAGTGGTCCCAAAGATGGACCCCCACCCACGAGGAGCATCGTGGAAAAAGAAGACGTTCCAACCACGTCTTCAAAGCAAGTGGATTGATGTGATTGGCAGACATACTGTCCCACAAATGAAGATGGAATCTGTAAAAGAAAACGCGTGAAATAATGCGTCTGACAAAGGTTAGGTCGGCTGCCTTTAATCAATACCAAAGTGGTCCCTACCACGATGGAAAAACTGTGCAGTCGGTTTGGCTTTTTCTGACGAACAAATAAGATTCGTGGCCGACAGGTGGGGGTCCACCATGTGAAGGCATCTTCAGACTCCAATAATGGAGCAATGACGTAAGGGCTTACGAAATAAGTAAGGGTAGTTTGGGAAATGTCCACTCACCCGTCAGTCTATAAATACTTAGCCCCTCCCTCATTGTTAAGGGAGCAAAATCTCAGAGAGATAGTCCTAGAGAGAGAAAGAGAGCAAGTAGCCTAGAAGTAGTCAAGGCGGCGAAGTATTCAGGCACGTGGCCAGGAAGAAGAAAAGCCAAGACGACGAAAACAGGTAAGAGCTAAGCATCTAGATAAGTTGAAAACAATCTTCAAAAGTCCCACATCGCTTAGATAAGAAAACGAAGCTGAGTTTATATACAGCTAGAGTCGAAGTAGTGATTGAACAAAGCACCAGTGGTCTAGTGGTAGAATAGTACCCTGCCACGGTACAGACCCGGGTTCGATTCCCGGCTGGTGCATTCCTCGTGCTGGACAAGTGGTTTAAGAGCTATGCTGGAAACAGCATAGCAAGTTTAAATAAGGCTAGTCCGTTATCAACTTGAAAAAGTGGCACCGAGTCGGTGCGGGAGCACATGAGGATCACCCATGTGCGACTCCCACAGTCACTGGGGAGTCTTCCCATCTGGAAAGCACACTTGTCCAGCACGCGGTTCTATCTAGTTACGCGTTAAACCAACTAGAATTTTTTTGGCCGGCATGGTCCCAGCCTCCTCGCTGGCGCCGGCTGGGCAACATGCTTCGGCATGGCGAATGGGACTTTTTTTT

**Sequence 72 Plasmids sequence of pH-35C-RERV-m5-OsEPSPS**

(RB T-DNA repeat-35C promoter-tRNAGly RS-pegRNA-Ubi promoter-NLSc-myc-NLSsv40-nCas9(H840A/ R221K/N394K)-XTEN-NC-NLS-32aa Linker-RERV-RT-m5-NLSvbp-HygR cassette-E9-terminator-CaMV poly(A) signal-LB T-DNA repeat)

GTTTACCCGCCAATATATCCTGTCAAACACTGATAGTTTAAACTGAAGGCGGGAAACGACAATCTGATCCAAGCTCAAGCTGCTCTAGCATTCGCCATTCAGGCTGCGCAACTGTTGGGAAGGGCGATCGGTGCGGGCCTCTTCGCTATTACGCCAGCTGGCGAAAGGGGGATGTGCTGCAAGGCGATTAAGTTGGGTAACGCCAGGGTTTTCCCAGTCACGACGTTGTAAAACGACGGCCAGTGAATTCATGGAGTCAAAGATTCAAATAGAGGACCTAACAGAACTCGCCGTAAAGACTGGCGAACAGTTCATACAGAGTCTCTTACGACTCAATGACAAGAAGAAAATCTTCGTCAACATGGTGGAGCACGACACACTTGTCTACTCCAAAAATATCAAAGATACAGTCTCAGAAGACCAAAGGGCAATTGAGACTTTTCAACAAAGGGTAATATCCGGAAACCTCCTCGGATTCCATTGCCCAGCTATCTGTCACTTTATTGTGAAGATAGTGGAAAAGGAAGGTGGCTCCTACAAATGCCATCATTGCGATAAAGGAAAGGCCATCGTTGAAGATGCCTCTGCCGACAGTGGTCCCAAAGATGGACCCCCACCCACGAGGAGCATCGTGGAAAAAGAAGACGTTCCAACCACGTCTTCAAAGCAAGTGGATTGATGTGATTGGCAGACATACTGTCCCACAAATGAAGATGGAATCTGTAAAAGAAAACGCGTGAAATAATGCGTCTGACAAAGGTTAGGTCGGCTGCCTTTAATCAATACCAAAGTGGTCCCTACCACGATGGAAAAACTGTGCAGTCGGTTTGGCTTTTTCTGACGAACAAATAAGATTCGTGGCCGACAGGTGGGGGTCCACCATGTGAAGGCATCTTCAGACTCCAATAATGGAGCAATGACGTAAGGGCTTACGAAATAAGTAAGGGTAGTTTGGGAAATGTCCACTCACCCGTCAGTCTATAAATACTTAGCCCCTCCCTCATTGTTAAGGGAGCAAAATCTCAGAGAGATAGTCCTAGAGAGAGAAAGAGAGCAAGTAGCCTAGAAGTAGTCAAGGCGGCGAAGTATTCAGGCACGTGGCCAGGAAGAAGAAAAGCCAAGACGACGAAAACAGGTAAGAGCTAAGCATCTAGATAAGTTGAAAACAATCTTCAAAAGTCCCACATCGCTTAGATAAGAAAACGAAGCTGAGTTTATATACAGCTAGAGTCGAAGTAGTGATTGAACAAAGCACCAGTGGTCTAGTGGTAGAATAGTACCCTGCCACGGTACAGACCCGGGTTCGATTCCCGGCTGGTGCAGCAGTCACGGCTGCTGTCAAGTTTAAGAGCTATGCTGGAAACAGCATAGCAAGTTTAAATAAGGCTAGTCCGTTATCAACTTGAAAAAGTGGCACCGAGTCGGTGCACGCTGGAATTGCAATGCGATCGTTGACAGCAGCCCGCGGTTCTATCTAGTTACGCGTTAAACCAACTAGAATTTTTTTGGCCGGCATGGTCCCAGCCTCCTCGCTGGCGCCGGCTGGGCAACATGCTTCGGCATGGCGAATGGGACTTTTTTTTGATATCTCCGGGGCTAATTGAATATGAAGATGAAGATGAAATATTTGGTGTGTCAAATAAAAAGCTGGTGTGCTTAAGTTTGTGTTTTTTTCTTGGCTTGTTGTGTTATGAATTTGTGGCTTTTTCTAATATTAAATGAATGTAAGATCTCATTATAATGAATAAACAAATGTTTCTATAATCCATTGTGAATGTTTTGTTGGATCTCTTCTGCAGCATATAACTACTGTATGTGCTATGGTATGGACTATGGAATATGATTAAAGATAAGAAGCTTGGCGTAATCTGCATGCCTGCAGTGCAGCGTGACCCGGTCGTGCCCCTCTCTAGAGATAATGAGCATTGCATGTCTAAGTTATAAAAAATTACCACATATTTTTTTTGTCACACTTGTTTGAAGTGCAGTTTATCTATCTTTATACATATATTTAAACTTTACTCTACGAATAATATAATCTATAGTACTACAATAATATCAGTGTTTTAGAGAATCATATAAATGAACAGTTAGACATGGTCTAAAGGACAATTGAGTATTTTGACAACAGGACTCTACAGTTTTATCTTTTTAGTGTGCATGTGTTCTCCTTTTTTTTTGCAAATAGCTTCACCTATATAATACTTCATCCATTTTATTAGTACATCCATTTAGGGTTTAGGGTTAATGGTTTTTATAGACTAATTTTTTTAGTACATCTATTTTATTCTATTTTAGCCTCTAAATTAAGAAAACTAAAACTCTATTTTAGTTTTTTTATTTAATAATTTAGATATAAAATAGAATAAAATAAAGTGACTAAAAATTAAACAAATACCCTTTAAGAAATTAAAAAAACTAAGGAAACATTTTTCTTGTTTCGAGTAGATAATGCCAGCCTGTTAAACGCCGTCGACGAGTCTAACGGACACCAACCAGCGAACCAGCAGCGTCGCGTCGGGCCAAGCGAAGCAGACGGCACGGCATCTCTGTCGCTGCCTCTGGACCCCTCTCGAGAGTTCCGCTCCACCGTTGGACTTGCTCCGCTGTCGGCATCCAGAAATTGCGTGGCGGAGCGGCAGACGTGAGCCGGCACGGCAGGCGGCCTCCTCCTCCTCTCACGGCACCGGCAGCTACGGGGGATTCCTTTCCCACCGCTCCTTCGCTTTCCCTTCCTCGCCCGCCGTAATAAATAGACACCCCCTCCACACCCTCTTTCCCCAACCTCGTGTTGTTCGGAGCGCACACACACACAACCAGATCTCCCCCAAATCCACCCGTCGGCACCTCCGCTTCAAGGTACGCCGCTCGTCCTCCCCCCCCCCCCCTCTCTACCTTCTCTAGATCGGCGTTCCGGTCCATGGTTAGGGCCCGGTAGTTCTACTTCTGTTCATGTTTGTGTTAGATCCGTGTTTGTGTTAGATCCGTGCTGCTAGCGTTCGTACACGGATGCGACCTGTACGTCAGACACGTTCTGATTGCTAACTTGCCAGTGTTTCTCTTTGGGGAATCCTGGGATGGCTCTAGCCGTTCCGCAGACGGGATCGATTTCATGATTTTTTTTGTTTCGTTGCATAGGGTTTGGTTTGCCCTTTTCCTTTATTTCAATATATGCCGTGCACTTGTTTGTCGGGTCATCTTTTCATGCTTTTTTTTGTCTTGGTTGTGATGATGTGGTCTGGTTGGGCGGTCGTTCTAGATCGGAGTAGAATTCTGTTTCAAACTACCTGGTGGATTTATTAATTTTGGATCTGTATGTGTGTGCCATACATATTCATAGTTACGAATTGAAGATGATGGATGGAAATATCGATCTAGGATAGGTATACATGTTGATGCGGGTTTTACTGATGCATATACAGAGATGCTTTTTGTTCGCTTGGTTGTGATGATGTGGTGTGGTTGGGCGGTCGTTCATTCGTTCTAGATCGGAGTAGAATACTGTTTCAAACTACCTGGTGTATTTATTAATTTTGGAACTGTATGTGTGTGTCATACATCTTCATAGTTACGAGTTTAAGATGGATGGAAATATCGATCTAGGATAGGTATACATGTTGATGTGGGTTTTACTGATGCATATACATGATGGCATATGCAGCATCTATTCATATGCTCTAACCTTGAGTACCTATCTATTATAATAAACAAGTATGTTTTATAATTATTTTGATCTTGATATACTTGGATGATGGCATATGCAGCAGCTATATGTGGATTTTTTTAGCCCTGCCTTCATACGCTATTTATTTGCTTGGTACTGTTTCTTTTGTCGATGCTCACCCTGTTGTTTGGTGTTACTTCTGCAGCCCTAGGACTGGTTGTACAATGCCGGCGGCCAAGAGAGTCAAGCTCGACGGCGGGAAGCGCACAGCTGATGGTTCTGAATTCGAGTCCCCTAAGAAAAAGAGAAAAGTGGACAAGAAGTACTCGATCGGCCTCGATATTGGGACTAACTCTGTTGGCTGGGCCGTGATCACCGACGAGTACAAGGTGCCCTCAAAGAAGTTCAAGGTCCTGGGCAACACCGATCGGCATTCCATCAAGAAGAATCTCATTGGCGCTCTCCTGTTCGACAGCGGCGAGACGGCTGAGGCTACGCGGCTCAAGCGCACCGCCCGCAGGCGGTACACGCGCAGGAAGAATCGCATCTGCTACCTGCAGGAGATTTTCTCCAACGAGATGGCGAAGGTTGACGATTCTTTCTTCCACAGGCTGGAGGAGTCATTCCTCGTGGAGGAGGATAAGAAGCACGAGCGGCATCCAATCTTCGGCAACATTGTCGACGAGGTTGCCTACCACGAGAAGTACCCTACGATCTACCATCTGCGGAAGAAGCTCGTGGACTCCACAGATAAGGCGGACCTCCGCCTGATCTACCTCGCTCTGGCCCACATGATTAAGTTCAGGGGCCATTTCCTGATCGAGGGGGATCTCAACCCGGACAATAGCGATGTTGACAAGCTGTTCATCCAGCTCGTGCAGACGTACAACCAGCTCTTCGAGGAGAACCCCATTAATGCGTCAGGCGTCGACGCGAAGGCTATCCTGTCCGCTAGGCTCTCGAAGTCTCGGAAGCTCGAGAACCTGATCGCCCAGCTGCCGGGCGAGAAGAAGAACGGCCTGTTCGGGAATCTCATTGCGCTCAGCCTGGGGCTCACGCCCAACTTCAAGTCGAATTTCGATCTCGCTGAGGACGCCAAGCTGCAGCTCTCCAAGGACACATACGACGATGACCTGGATAACCTCCTGGCCCAGATCGGCGATCAGTACGCGGACCTGTTCCTCGCTGCCAAGAATCTGTCGGACGCCATCCTCCTGTCTGATATTCTCAGGGTGAACACCGAGATTACGAAGGCTCCGCTCTCAGCCTCCATGATCAAGCGCTACGACGAGCACCATCAGGATCTGACCCTCCTGAAGGCGCTGGTCAGGCAGCAGCTCCCCGAGAAGTACAAGGAGATCTTCTTCGATCAGTCGAAGAACGGCTACGCTGGGTACATTGACGGCGGGGCCTCTCAGGAGGAGTTCTACAAGTTCATCAAGCCGATTCTGGAGAAGATGGACGGCACGGAGGAGCTGCTGGTGAAGCTCAAGCGCGAGGACCTCCTGAGGAAGCAGCGGACATTCGATAACGGCAGCATCCCACACCAGATTCATCTCGGGGAGCTGCACGCTATCCTGAGGAGGCAGGAGGACTTCTACCCTTTCCTCAAGGATAACCGCGAGAAGATCGAGAAGATTCTGACTTTCAGGATCCCGTACTACGTCGGCCCACTCGCTAGGGGCAACTCCCGCTTCGCTTGGATGACCCGCAAGTCAGAGGAGACGATCACGCCGTGGAACTTCGAGGAGGTGGTCGACAAGGGCGCTAGCGCTCAGTCGTTCATCGAGAGGATGACGAATTTCGACAAGAACCTGCCAAATGAGAAGGTGCTCCCTAAGCACTCGCTCCTGTACGAGTACTTCACAGTCTACAACGAGCTGACTAAGGTGAAGTATGTGACCGAGGGCATGAGGAAGCCGGCTTTCCTGTCTGGGGAGCAGAAGAAGGCCATCGTGGACCTCCTGTTCAAGACCAACCGGAAGGTCACGGTTAAGCAGCTCAAGGAGGACTACTTCAAGAAGATTGAGTGCTTCGATTCGGTCGAGATCTCTGGCGTTGAGGACCGCTTCAACGCCTCCCTGGGGACCTACCACGATCTCCTGAAGATCATTAAGGATAAGGACTTCCTGGACAACGAGGAGAATGAGGATATCCTCGAGGACATTGTGCTGACACTCACTCTGTTCGAGGACCGGGAGATGATCGAGGAGCGCCTGAAGACTTACGCCCATCTCTTCGATGACAAGGTCATGAAGCAGCTCAAGAGGAGGAGGTACACCGGCTGGGGGAGGCTGAGCAGGAAGCTCATCAACGGCATTCGGGACAAGCAGTCCGGGAAGACGATCCTCGACTTCCTGAAGAGCGATGGCTTCGCGAACCGCAATTTCATGCAGCTGATTCACGATGACAGCCTCACATTCAAGGAGGATATCCAGAAGGCTCAGGTGAGCGGCCAGGGGGACTCGCTGCACGAGCATATCGCGAACCTCGCTGGCTCGCCAGCTATCAAGAAGGGGATTCTGCAGACCGTGAAGGTTGTGGACGAGCTGGTGAAGGTCATGGGCAGGCACAAGCCTGAGAACATCGTCATTGAGATGGCCCGGGAGAATCAGACCACGCAGAAGGGCCAGAAGAACTCACGCGAGAGGATGAAGAGGATCGAGGAGGGCATTAAGGAGCTGGGGTCCCAGATCCTCAAGGAGCACCCGGTGGAGAACACGCAGCTGCAGAATGAGAAGCTCTACCTGTACTACCTCCAGAATGGCCGCGATATGTATGTGGACCAGGAGCTGGATATTAACAGGCTCAGCGATTACGACGTCGATGCCATCGTTCCACAGTCATTCCTGAAGGATGACTCCATTGACAACAAGGTCCTCACCAGGTCGGACAAGAACCGGGGCAAGTCTGATAATGTTCCTTCAGAGGAGGTCGTTAAGAAGATGAAGAACTACTGGCGCCAGCTCCTGAATGCCAAGCTGATCACGCAGCGGAAGTTCGATAACCTCACAAAGGCTGAGAGGGGCGGGCTCTCTGAGCTGGACAAGGCGGGCTTCATCAAGAGGCAGCTGGTCGAGACACGGCAGATCACTAAGCACGTTGCGCAGATTCTCGACTCACGGATGAACACTAAGTACGATGAGAATGACAAGCTGATCCGCGAGGTGAAGGTCATCACCCTGAAGTCAAAGCTCGTCTCCGACTTCAGGAAGGATTTCCAGTTCTACAAGGTTCGGGAGATCAACAATTACCACCATGCCCATGACGCGTACCTGAACGCGGTGGTCGGCACAGCTCTGATCAAGAAGTACCCAAAGCTCGAGAGCGAGTTCGTGTACGGGGACTACAAGGTTTACGATGTGAGGAAGATGATCGCCAAGTCGGAGCAGGAGATTGGCAAGGCTACCGCCAAGTACTTCTTCTACTCTAACATTATGAATTTCTTCAAGACAGAGATCACTCTGGCCAATGGCGAGATCCGGAAGCGCCCCCTCATCGAGACGAACGGCGAGACGGGGGAGATCGTGTGGGACAAGGGCAGGGATTTCGCGACCGTCAGGAAGGTTCTCTCCATGCCACAAGTGAATATCGTCAAGAAGACAGAGGTCCAGACTGGCGGGTTCTCTAAGGAGTCAATTCTGCCTAAGCGGAACAGCGACAAGCTCATCGCCCGCAAGAAGGACTGGGATCCGAAGAAGTACGGCGGGTTCGACAGCCCCACTGTGGCCTACTCGGTCCTGGTTGTGGCGAAGGTTGAGAAGGGCAAGTCCAAGAAGCTCAAGAGCGTGAAGGAGCTGCTGGGGATCACGATTATGGAGCGCTCCAGCTTCGAGAAGAACCCGATCGATTTCCTGGAGGCGAAGGGCTACAAGGAGGTGAAGAAGGACCTGATCATTAAGCTCCCCAAGTACTCACTCTTCGAGCTGGAGAACGGCAGGAAGCGGATGCTGGCTTCCGCTGGCGAGCTGCAGAAGGGGAACGAGCTGGCTCTGCCGTCCAAGTATGTGAACTTCCTCTACCTGGCCTCCCACTACGAGAAGCTCAAGGGCAGCCCCGAGGACAACGAGCAGAAGCAGCTGTTCGTCGAGCAGCACAAGCATTACCTCGACGAGATCATTGAGCAGATTTCCGAGTTCTCCAAGCGCGTGATCCTGGCCGACGCGAATCTGGATAAGGTCCTCTCCGCGTACAACAAGCACCGCGACAAGCCAATCAGGGAGCAGGCTGAGAATATCATTCATCTCTTCACCCTGACGAACCTCGGCGCCCCTGCTGCTTTCAAGTACTTCGACACAACTATCGATCGCAAGAGGTACACAAGCACTAAGGAGGTCCTGGACGCGACCCTCATCCACCAGTCGATTACCGGCCTCTACGAGACGCGCATCGACCTGTCTCAGCTCGGGGGCGACGAATTCTCCGGGAGCGAGACGCCAGGCACCTCCGAGTCGGCCACCCCAGAATCTGCCACAGTGGTGTCCGGCCAAAAGCAGGACCGCCAGGGCGGAGAACGCAGAAGGTCCCAGCTCGATAGGGATCAGTGTGCCTACTGCAAGGAGAAGGGCCACTGGGCCAAAGACTGCCCGAAAAAGCCGCGCGGCCCACGCGGCCCAAGGCCACAAACATCCCTCCTTCCAAAGAAGAAGCGGAAGGTGGAGCTCAGCGGAGGATCTTCCGGAGGATCTAGCGGCTCCGAGACACCAGGAACATCCGAAAGCGCTACACCAGAATCTAGCGGAGGCTCTTCCGGAGGATCTAGGCCTCCTCCCGTCGCGTGCCTCGTACTGAGCCTAGAGGAGGAGTACAGGCTGCATGAGCAAAATCCGAAGCAGCTGCTAGCCCCTGAATGGCTGAGCGCCTTCTCCGAGGTATGGGCGGAGCAGACTGGCATGGGACTGGCCAAGCAGGTGCCTCCTGTGGTGGTAGAGCTGAAGGCGGACGCCAGCCCAGTGAGCGTGAAACAATACCCGATGTCGAGGGAAGCAAAGGAAGGCATTAGACCCCACATCCAGAGACTGCTGCAATTGGGAATCCTGGTACCCTGCCAAAGCCCTTGGAACACGCCACTGCTGCCTGTGCGCAAACCAGGAACTAATGATTACAGACCTGTACAGGATCTAAGGGAAGTTAACAAGCGGGTGCAGGACATACATCCTACGGTCCCAAACCCCTACAACTTGCTGAGCTCTCTGCCCCCCGAGCGCACATGGTACACTGTGCTCGACTTAAAAGATGCGTTCTTCTGCCTTCGCCTCCACCCGAACTCACAGCCACTGTTCGCGTTCGAGTGGCGCGACCCTGAGGGCGGCCACACCGGGCAACTGACGTGGACACGGCTGCCACAGGGTTTCAAGAACAGCCCTACTCTATTTAACGAGGCCCTGCACCGTGATCTGGCCCCCTTCCGGGCGCAGAACCCTCAAATCAGTCTACTGCAGTATGCGGATGACCTGCTGCTAGCCGCTTCTACTCAGGAGCTCTGCCGGGAGGGCACAAAGCGGCTGTTAAATGAGCTGGGCGAGCTGGGATACCGCGTGTCTGCCAAGAAGGCACAATTGTGTCGGACAGAGGTGACATATCTCGGCTACACATTAAGAGAAGGTAAGAGATGGCTCACTGAAGCACGTAAGAAGACTGTCATGCAGATTCCTACCCCTACCACGCCCCGTCAGGTGCGGGAGTTCCTCGGCAAGGCCGGCTTCTGTAGACTGTTCATTCCTGGATTTGCCACCCTGGCCGCCCCACTCTACCTGCTGACGAAGCCAAAGGTTCCTTTCACCTGGACTGAGGAACATCAGCGGGCTTTCGAAGACATCAAAGCCGCCCTGCTGGCGGCTCCAGCTCTGGCCCTCCCTGACCTCACCAAGCCCTTCACACTCTATGTGGACGAGAGAGCTGGAGTGGCCAGAGGTGTGCTGACACAGGCACTGGGGCCGTGGAAGAGACCCGTGGCGTACCTATCTAAAAAGCTCGACCCCGTGGCCTCTGGGTGGCCGAGCTGTCTAAAGGCCATCGCTGCTGTAGCTCTACTGGTCAAAGATGCTGACAAGCTGACCCTGGGCCAGCATGTCACCGTCATCGCCCCGCACGCCTTAGAATCCATAGTCCGGCAACCACCAGATCGGTGGATGACAAACGCTAGGATGACGCATTATCAGAGCTTGCTGTTAAACGAAAGAGTGACATTTGCGCCCCCTGCCATCCTTAACCCAGCGACGCTACTGCCGGAGATCCACAACTCAACCCCCATCCACCAGTGCGTCGACATTCTGGCTGAGGAGACCGGCACGCGCAAGGACCTCACGGACAGACCATGGCCTGGTGTGCCGGCATGGTACACCGACGGCAGCTCCTTTGTCGTGGAGGGAAAGCGACGCGCAGGCGCGGCTGTGGTGGACGGCAAGCAAGTCATCTGGGCATCCTCCCTGCCAGAAGGCACCAGCGCTCAGAAAGCGGAGTTGGTGGCCTTGACCCAGGCCCTCAGATTAGCTGAAGGGAAGGCTATTAATATCTACACCGATTCTAGATACGCCTTCGCCACGGCGCACATCCACGGCGCGATCTACAAGCAGCGGGGCCTGCTGACCAGTGCCGGGCGAGACATCAAGAATAAGGAAGAGATTCTTGCCCTGCTGGAGGCCGTCCACCTCCCCAAAAAGGTGGCCATTATCCACTGCCCCGGCCACCAGAAGGGAGAGGATCCAATTACAAAGGGGAACCAGATGGCCGACCTCGTGGCGAAGCAGGTCGCTCAGCAGGTTACCATCCTGGCAGAAAAATCGCAGACCCCTGTGAAGACACCTGTGACAGATGATTCTAACTATAGCGGCGGCAGCAAAAGAACGGCGGACGGCTCTGAGAAGCGCACCGCTGATAGCCAGCATTCAACTCCTCCGAAAACAAAGAGGAAAGTTGAGTTCGAACCGAAGAAGAAAAGGAAGGTGTGAGAGCTCAGAGCTTTCGTTCGTATCATCGGTTTCGACAACGTTCGTCAAGTTCAATGCATCAGTTTCATTGCGCACACACCAGAATCCTACTGAGTTTGAGTATTATGGCATTGGGAAAACTGTTTTTCTTGTACCATTTGTTGTGCTTGTAATTTACTGTGTTTTTTATTCGGTTTTCGCTATCGAACTGTGAAATGGAAATGGATGGAGAAGAGTTAATGAATGATATGGTCCTTTTGTTCATTCTCAAATTAATATTATTTGTTTTTTCTCTTATTTGTTGTGTGTTGAATTTGAAATTATAAGAGATATGCAAACATTTTGTTTTGAGTAAAAATGTGTCAAATCGTGGCCTCTAATGACCGAAGTTAATATGAGGAGTAAAACACTTGTAGTTGTACCATTATGCTTATTCACTAGGCAACAAATATATTTTCAGACCTAGAAAAGCTGCAAATGTTACTGAATACAAGTATGTCCTCTTGTGTTTTAGACATTTATGAACTTTCCTTTATGTAATTTTCCAGAATCCTTGTCAGATTCTAATCATTGCTTTATAATTATAGTTATACTCATGGATTTGTAGTTGAGTATGAAAATATTTTTTAATGCATTTTATGACTTGCCAATTGATTGACAACGAATTCGTAATCATGTCATAGCTGTTTCCTGTGTGAAATTGTTATCCGCTCACAATTCCACACAACATACGAGCCGGAAGCATAAAGTGTAAAGCCTGGGGTGCCTAATGAGTGAGCTAACTCACATTAATTGCGTTGCGCTCACTGCCCGCTTTCCAGTCGGGAAACCTGTCGTGCCAGCTGCATTAATGAATCGGCCAACGCGCGGGGAGAGGCGGTTTGCGTATTGGCTAGAGCAGCTTGCCAACATGGTGGAGCACGACACTCTCGTCTACTCCAAGAATATCAAAGATACAGTCTCAGAAGACCAAAGGGCTATTGAGACTTTTCAACAAAGGGTAATATCGGGAAACCTCCTCGGATTCCATTGCCCAGCTATCTGTCACTTCATCAAAAGGACAGTAGAAAAGGAAGGTGGCACCTACAAATGCCATCATTGCGATAAAGGAAAGGCTATCGTTCAAGATGCCTCTGCCGACAGTGGTCCCAAAGATGGACCCCCACCCACGAGGAGCATCGTGGAAAAAGAAGACGTTCCAACCACGTCTTCAAAGCAAGTGGATTGATGTGAACATGGTGGAGCACGACACTCTCGTCTACTCCAAGAATATCAAAGATACAGTCTCAGAAGACCAAAGGGCTATTGAGACTTTTCAACAAAGGGTAATATCGGGAAACCTCCTCGGATTCCATTGCCCAGCTATCTGTCACTTCATCAAAAGGACAGTAGAAAAGGAAGGTGGCACCTACAAATGCCATCATTGCGATAAAGGAAAGGCTATCGTTCAAGATGCCTCTGCCGACAGTGGTCCCAAAGATGGACCCCCACCCACGAGGAGCATCGTGGAAAAAGAAGACGTTCCAACCACGTCTTCAAAGCAAGTGGATTGATGTGATATCTCCACTGACGTAAGGGATGACGCACAATCCCACTATCCTTCGCAAGACCCTTCCTCTATATAAGGAAGTTCATTTCATTTGGAGAGGACACGCTGAAATCACCAGTCTCTCTCTACAAATCTATCTCTCTCGAGCTTTCGCAGATCCGGGGGGCAATGAGATATGAAAAAGCCTGAACTCACCGCGACGTCTGTCGAGAAGTTTCTGATCGAAAAGTTCGACAGCGTCTCCGACCTGATGCAGCTCTCGGAGGGCGAAGAATCTCGTGCTTTCAGCTTCGATGTAGGAGGGCGTGGATATGTCCTGCGGGTAAATAGCTGCGCCGATGGTTTCTACAAAGATCGTTATGTTTATCGGCACTTTGCATCGGCCGCGCTCCCGATTCCGGAAGTGCTTGACATTGGGGAGTTTAGCGAGAGCCTGACCTATTGCATCTCCCGCCGTTCACAGGGTGTCACGTTGCAAGACCTGCCTGAAACCGAACTGCCCGCTGTTCTACAACCGGTCGCGGAGGCTATGGATGCGATCGCTGCGGCCGATCTTAGCCAGACGAGCGGGTTCGGCCCATTCGGACCGCAAGGAATCGGTCAATACACTACATGGCGTGATTTCATATGCGCGATTGCTGATCCCCATGTGTATCACTGGCAAACTGTGATGGACGACACCGTCAGTGCGTCCGTCGCGCAGGCTCTCGATGAGCTGATGCTTTGGGCCGAGGACTGCCCCGAAGTCCGGCACCTCGTGCACGCGGATTTCGGCTCCAACAATGTCCTGACGGACAATGGCCGCATAACAGCGGTCATTGACTGGAGCGAGGCGATGTTCGGGGATTCCCAATACGAGGTCGCCAACATCTTCTTCTGGAGGCCGTGGTTGGCTTGTATGGAGCAGCAGACGCGCTACTTCGAGCGGAGGCATCCGGAGCTTGCAGGATCGCCACGACTCCGGGCGTATATGCTCCGCATTGGTCTTGACCAACTCTATCAGAGCTTGGTTGACGGCAATTTCGATGATGCAGCTTGGGCGCAGGGTCGATGCGACGCAATCGTCCGATCCGGAGCCGGGACTGTCGGGCGTACACAAATCGCCCGCAGAAGCGCGGCCGTCTGGACCGATGGCTGTGTAGAAGTACTCGCCGATAGTGGAAACCGACGCCCCAGCACTCGTCCGAGGGCAAAGAAATAGAGTAGATGCCGACCGGGATCTGTCGATCGACAAGCTCGAGTTTCTCCATAATAATGTGTGAGTAGTTCCCAGATAAGGGAATTAGGGTTCCTATAGGGTTTCGCTCATGTGTTGAGCATATAAGAAACCCTTAGTATGTATTTGTATTTGTAAAATACTTCTATCAATAAAATTTCTAATTCCTAAAACCAAAATCCAGTACTAAAATCCAGATCCCCCGAATTAATTCGGCGTTAATTCAGTACATTAAAAACGTCCGCAATGTGTTATTAAGTTGTCTAAGCGTCAATTTGTTTACACCACAATATATCCTGCCA

**Sequence 73 Plasmids sequence of pH-35C-RERV-m5-OsALS**

(RB T-DNA repeat-35C promoter-tRNAGly RS-pegRNA-Ubi promoter-NLSc-myc-NLSsv40-nCas9(H840A/ R221K/N394K)-XTEN-NC-NLS-32aa Linker-RERV-RT-m5-NLSvbp-HygR cassette-E9-terminator-CaMV poly(A) signal-LB T-DNA repeat)

GTTTACCCGCCAATATATCCTGTCAAACACTGATAGTTTAAACTGAAGGCGGGAAACGACAATCTGATCCAAGCTCAAGCTGCTCTAGCATTCGCCATTCAGGCTGCGCAACTGTTGGGAAGGGCGATCGGTGCGGGCCTCTTCGCTATTACGCCAGCTGGCGAAAGGGGGATGTGCTGCAAGGCGATTAAGTTGGGTAACGCCAGGGTTTTCCCAGTCACGACGTTGTAAAACGACGGCCAGTGAATTCATGGAGTCAAAGATTCAAATAGAGGACCTAACAGAACTCGCCGTAAAGACTGGCGAACAGTTCATACAGAGTCTCTTACGACTCAATGACAAGAAGAAAATCTTCGTCAACATGGTGGAGCACGACACACTTGTCTACTCCAAAAATATCAAAGATACAGTCTCAGAAGACCAAAGGGCAATTGAGACTTTTCAACAAAGGGTAATATCCGGAAACCTCCTCGGATTCCATTGCCCAGCTATCTGTCACTTTATTGTGAAGATAGTGGAAAAGGAAGGTGGCTCCTACAAATGCCATCATTGCGATAAAGGAAAGGCCATCGTTGAAGATGCCTCTGCCGACAGTGGTCCCAAAGATGGACCCCCACCCACGAGGAGCATCGTGGAAAAAGAAGACGTTCCAACCACGTCTTCAAAGCAAGTGGATTGATGTGATTGGCAGACATACTGTCCCACAAATGAAGATGGAATCTGTAAAAGAAAACGCGTGAAATAATGCGTCTGACAAAGGTTAGGTCGGCTGCCTTTAATCAATACCAAAGTGGTCCCTACCACGATGGAAAAACTGTGCAGTCGGTTTGGCTTTTTCTGACGAACAAATAAGATTCGTGGCCGACAGGTGGGGGTCCACCATGTGAAGGCATCTTCAGACTCCAATAATGGAGCAATGACGTAAGGGCTTACGAAATAAGTAAGGGTAGTTTGGGAAATGTCCACTCACCCGTCAGTCTATAAATACTTAGCCCCTCCCTCATTGTTAAGGGAGCAAAATCTCAGAGAGATAGTCCTAGAGAGAGAAAGAGAGCAAGTAGCCTAGAAGTAGTCAAGGCGGCGAAGTATTCAGGCACGTGGCCAGGAAGAAGAAAAGCCAAGACGACGAAAACAGGTAAGAGCTAAGCATCTAGATAAGTTGAAAACAATCTTCAAAAGTCCCACATCGCTTAGATAAGAAAACGAAGCTGAGTTTATATACAGCTAGAGTCGAAGTAGTGATTGAACAAAGCACCAGTGGTCTAGTGGTAGAATAGTACCCTGCCACGGTACAGACCCGGGTTCGATTCCCGGCTGGTGCAGGGTATGGTGGTGCAATGGGGTTTAAGAGCTATGCTGGAAACAGCATAGCAAGTTTAAATAAGGCTAGTCCGTTATCAACTTGAAAAAGTGGCACCGAGTCGGTGCAAAACCTATCCTCCAATTGCACCACCGCGGTTCTATCTAGTTACGCGTTAAACCAACTAGAATTTTTTTGGCCGGCATGGTCCCAGCCTCCTCGCTGGCGCCGGCTGGGCAACATGCTTCGGCATGGCGAATGGGACTTTTTTTTGATATCTCCGGGGCTAATTGAATATGAAGATGAAGATGAAATATTTGGTGTGTCAAATAAAAAGCTGGTGTGCTTAAGTTTGTGTTTTTTTCTTGGCTTGTTGTGTTATGAATTTGTGGCTTTTTCTAATATTAAATGAATGTAAGATCTCATTATAATGAATAAACAAATGTTTCTATAATCCATTGTGAATGTTTTGTTGGATCTCTTCTGCAGCATATAACTACTGTATGTGCTATGGTATGGACTATGGAATATGATTAAAGATAAGAAGCTTGGCGTAATCTGCATGCCTGCAGTGCAGCGTGACCCGGTCGTGCCCCTCTCTAGAGATAATGAGCATTGCATGTCTAAGTTATAAAAAATTACCACATATTTTTTTTGTCACACTTGTTTGAAGTGCAGTTTATCTATCTTTATACATATATTTAAACTTTACTCTACGAATAATATAATCTATAGTACTACAATAATATCAGTGTTTTAGAGAATCATATAAATGAACAGTTAGACATGGTCTAAAGGACAATTGAGTATTTTGACAACAGGACTCTACAGTTTTATCTTTTTAGTGTGCATGTGTTCTCCTTTTTTTTTGCAAATAGCTTCACCTATATAATACTTCATCCATTTTATTAGTACATCCATTTAGGGTTTAGGGTTAATGGTTTTTATAGACTAATTTTTTTAGTACATCTATTTTATTCTATTTTAGCCTCTAAATTAAGAAAACTAAAACTCTATTTTAGTTTTTTTATTTAATAATTTAGATATAAAATAGAATAAAATAAAGTGACTAAAAATTAAACAAATACCCTTTAAGAAATTAAAAAAACTAAGGAAACATTTTTCTTGTTTCGAGTAGATAATGCCAGCCTGTTAAACGCCGTCGACGAGTCTAACGGACACCAACCAGCGAACCAGCAGCGTCGCGTCGGGCCAAGCGAAGCAGACGGCACGGCATCTCTGTCGCTGCCTCTGGACCCCTCTCGAGAGTTCCGCTCCACCGTTGGACTTGCTCCGCTGTCGGCATCCAGAAATTGCGTGGCGGAGCGGCAGACGTGAGCCGGCACGGCAGGCGGCCTCCTCCTCCTCTCACGGCACCGGCAGCTACGGGGGATTCCTTTCCCACCGCTCCTTCGCTTTCCCTTCCTCGCCCGCCGTAATAAATAGACACCCCCTCCACACCCTCTTTCCCCAACCTCGTGTTGTTCGGAGCGCACACACACACAACCAGATCTCCCCCAAATCCACCCGTCGGCACCTCCGCTTCAAGGTACGCCGCTCGTCCTCCCCCCCCCCCCCTCTCTACCTTCTCTAGATCGGCGTTCCGGTCCATGGTTAGGGCCCGGTAGTTCTACTTCTGTTCATGTTTGTGTTAGATCCGTGTTTGTGTTAGATCCGTGCTGCTAGCGTTCGTACACGGATGCGACCTGTACGTCAGACACGTTCTGATTGCTAACTTGCCAGTGTTTCTCTTTGGGGAATCCTGGGATGGCTCTAGCCGTTCCGCAGACGGGATCGATTTCATGATTTTTTTTGTTTCGTTGCATAGGGTTTGGTTTGCCCTTTTCCTTTATTTCAATATATGCCGTGCACTTGTTTGTCGGGTCATCTTTTCATGCTTTTTTTTGTCTTGGTTGTGATGATGTGGTCTGGTTGGGCGGTCGTTCTAGATCGGAGTAGAATTCTGTTTCAAACTACCTGGTGGATTTATTAATTTTGGATCTGTATGTGTGTGCCATACATATTCATAGTTACGAATTGAAGATGATGGATGGAAATATCGATCTAGGATAGGTATACATGTTGATGCGGGTTTTACTGATGCATATACAGAGATGCTTTTTGTTCGCTTGGTTGTGATGATGTGGTGTGGTTGGGCGGTCGTTCATTCGTTCTAGATCGGAGTAGAATACTGTTTCAAACTACCTGGTGTATTTATTAATTTTGGAACTGTATGTGTGTGTCATACATCTTCATAGTTACGAGTTTAAGATGGATGGAAATATCGATCTAGGATAGGTATACATGTTGATGTGGGTTTTACTGATGCATATACATGATGGCATATGCAGCATCTATTCATATGCTCTAACCTTGAGTACCTATCTATTATAATAAACAAGTATGTTTTATAATTATTTTGATCTTGATATACTTGGATGATGGCATATGCAGCAGCTATATGTGGATTTTTTTAGCCCTGCCTTCATACGCTATTTATTTGCTTGGTACTGTTTCTTTTGTCGATGCTCACCCTGTTGTTTGGTGTTACTTCTGCAGCCCTAGGACTGGTTGTACAATGCCGGCGGCCAAGAGAGTCAAGCTCGACGGCGGGAAGCGCACAGCTGATGGTTCTGAATTCGAGTCCCCTAAGAAAAAGAGAAAAGTGGACAAGAAGTACTCGATCGGCCTCGATATTGGGACTAACTCTGTTGGCTGGGCCGTGATCACCGACGAGTACAAGGTGCCCTCAAAGAAGTTCAAGGTCCTGGGCAACACCGATCGGCATTCCATCAAGAAGAATCTCATTGGCGCTCTCCTGTTCGACAGCGGCGAGACGGCTGAGGCTACGCGGCTCAAGCGCACCGCCCGCAGGCGGTACACGCGCAGGAAGAATCGCATCTGCTACCTGCAGGAGATTTTCTCCAACGAGATGGCGAAGGTTGACGATTCTTTCTTCCACAGGCTGGAGGAGTCATTCCTCGTGGAGGAGGATAAGAAGCACGAGCGGCATCCAATCTTCGGCAACATTGTCGACGAGGTTGCCTACCACGAGAAGTACCCTACGATCTACCATCTGCGGAAGAAGCTCGTGGACTCCACAGATAAGGCGGACCTCCGCCTGATCTACCTCGCTCTGGCCCACATGATTAAGTTCAGGGGCCATTTCCTGATCGAGGGGGATCTCAACCCGGACAATAGCGATGTTGACAAGCTGTTCATCCAGCTCGTGCAGACGTACAACCAGCTCTTCGAGGAGAACCCCATTAATGCGTCAGGCGTCGACGCGAAGGCTATCCTGTCCGCTAGGCTCTCGAAGTCTCGGAAGCTCGAGAACCTGATCGCCCAGCTGCCGGGCGAGAAGAAGAACGGCCTGTTCGGGAATCTCATTGCGCTCAGCCTGGGGCTCACGCCCAACTTCAAGTCGAATTTCGATCTCGCTGAGGACGCCAAGCTGCAGCTCTCCAAGGACACATACGACGATGACCTGGATAACCTCCTGGCCCAGATCGGCGATCAGTACGCGGACCTGTTCCTCGCTGCCAAGAATCTGTCGGACGCCATCCTCCTGTCTGATATTCTCAGGGTGAACACCGAGATTACGAAGGCTCCGCTCTCAGCCTCCATGATCAAGCGCTACGACGAGCACCATCAGGATCTGACCCTCCTGAAGGCGCTGGTCAGGCAGCAGCTCCCCGAGAAGTACAAGGAGATCTTCTTCGATCAGTCGAAGAACGGCTACGCTGGGTACATTGACGGCGGGGCCTCTCAGGAGGAGTTCTACAAGTTCATCAAGCCGATTCTGGAGAAGATGGACGGCACGGAGGAGCTGCTGGTGAAGCTCAAGCGCGAGGACCTCCTGAGGAAGCAGCGGACATTCGATAACGGCAGCATCCCACACCAGATTCATCTCGGGGAGCTGCACGCTATCCTGAGGAGGCAGGAGGACTTCTACCCTTTCCTCAAGGATAACCGCGAGAAGATCGAGAAGATTCTGACTTTCAGGATCCCGTACTACGTCGGCCCACTCGCTAGGGGCAACTCCCGCTTCGCTTGGATGACCCGCAAGTCAGAGGAGACGATCACGCCGTGGAACTTCGAGGAGGTGGTCGACAAGGGCGCTAGCGCTCAGTCGTTCATCGAGAGGATGACGAATTTCGACAAGAACCTGCCAAATGAGAAGGTGCTCCCTAAGCACTCGCTCCTGTACGAGTACTTCACAGTCTACAACGAGCTGACTAAGGTGAAGTATGTGACCGAGGGCATGAGGAAGCCGGCTTTCCTGTCTGGGGAGCAGAAGAAGGCCATCGTGGACCTCCTGTTCAAGACCAACCGGAAGGTCACGGTTAAGCAGCTCAAGGAGGACTACTTCAAGAAGATTGAGTGCTTCGATTCGGTCGAGATCTCTGGCGTTGAGGACCGCTTCAACGCCTCCCTGGGGACCTACCACGATCTCCTGAAGATCATTAAGGATAAGGACTTCCTGGACAACGAGGAGAATGAGGATATCCTCGAGGACATTGTGCTGACACTCACTCTGTTCGAGGACCGGGAGATGATCGAGGAGCGCCTGAAGACTTACGCCCATCTCTTCGATGACAAGGTCATGAAGCAGCTCAAGAGGAGGAGGTACACCGGCTGGGGGAGGCTGAGCAGGAAGCTCATCAACGGCATTCGGGACAAGCAGTCCGGGAAGACGATCCTCGACTTCCTGAAGAGCGATGGCTTCGCGAACCGCAATTTCATGCAGCTGATTCACGATGACAGCCTCACATTCAAGGAGGATATCCAGAAGGCTCAGGTGAGCGGCCAGGGGGACTCGCTGCACGAGCATATCGCGAACCTCGCTGGCTCGCCAGCTATCAAGAAGGGGATTCTGCAGACCGTGAAGGTTGTGGACGAGCTGGTGAAGGTCATGGGCAGGCACAAGCCTGAGAACATCGTCATTGAGATGGCCCGGGAGAATCAGACCACGCAGAAGGGCCAGAAGAACTCACGCGAGAGGATGAAGAGGATCGAGGAGGGCATTAAGGAGCTGGGGTCCCAGATCCTCAAGGAGCACCCGGTGGAGAACACGCAGCTGCAGAATGAGAAGCTCTACCTGTACTACCTCCAGAATGGCCGCGATATGTATGTGGACCAGGAGCTGGATATTAACAGGCTCAGCGATTACGACGTCGATGCCATCGTTCCACAGTCATTCCTGAAGGATGACTCCATTGACAACAAGGTCCTCACCAGGTCGGACAAGAACCGGGGCAAGTCTGATAATGTTCCTTCAGAGGAGGTCGTTAAGAAGATGAAGAACTACTGGCGCCAGCTCCTGAATGCCAAGCTGATCACGCAGCGGAAGTTCGATAACCTCACAAAGGCTGAGAGGGGCGGGCTCTCTGAGCTGGACAAGGCGGGCTTCATCAAGAGGCAGCTGGTCGAGACACGGCAGATCACTAAGCACGTTGCGCAGATTCTCGACTCACGGATGAACACTAAGTACGATGAGAATGACAAGCTGATCCGCGAGGTGAAGGTCATCACCCTGAAGTCAAAGCTCGTCTCCGACTTCAGGAAGGATTTCCAGTTCTACAAGGTTCGGGAGATCAACAATTACCACCATGCCCATGACGCGTACCTGAACGCGGTGGTCGGCACAGCTCTGATCAAGAAGTACCCAAAGCTCGAGAGCGAGTTCGTGTACGGGGACTACAAGGTTTACGATGTGAGGAAGATGATCGCCAAGTCGGAGCAGGAGATTGGCAAGGCTACCGCCAAGTACTTCTTCTACTCTAACATTATGAATTTCTTCAAGACAGAGATCACTCTGGCCAATGGCGAGATCCGGAAGCGCCCCCTCATCGAGACGAACGGCGAGACGGGGGAGATCGTGTGGGACAAGGGCAGGGATTTCGCGACCGTCAGGAAGGTTCTCTCCATGCCACAAGTGAATATCGTCAAGAAGACAGAGGTCCAGACTGGCGGGTTCTCTAAGGAGTCAATTCTGCCTAAGCGGAACAGCGACAAGCTCATCGCCCGCAAGAAGGACTGGGATCCGAAGAAGTACGGCGGGTTCGACAGCCCCACTGTGGCCTACTCGGTCCTGGTTGTGGCGAAGGTTGAGAAGGGCAAGTCCAAGAAGCTCAAGAGCGTGAAGGAGCTGCTGGGGATCACGATTATGGAGCGCTCCAGCTTCGAGAAGAACCCGATCGATTTCCTGGAGGCGAAGGGCTACAAGGAGGTGAAGAAGGACCTGATCATTAAGCTCCCCAAGTACTCACTCTTCGAGCTGGAGAACGGCAGGAAGCGGATGCTGGCTTCCGCTGGCGAGCTGCAGAAGGGGAACGAGCTGGCTCTGCCGTCCAAGTATGTGAACTTCCTCTACCTGGCCTCCCACTACGAGAAGCTCAAGGGCAGCCCCGAGGACAACGAGCAGAAGCAGCTGTTCGTCGAGCAGCACAAGCATTACCTCGACGAGATCATTGAGCAGATTTCCGAGTTCTCCAAGCGCGTGATCCTGGCCGACGCGAATCTGGATAAGGTCCTCTCCGCGTACAACAAGCACCGCGACAAGCCAATCAGGGAGCAGGCTGAGAATATCATTCATCTCTTCACCCTGACGAACCTCGGCGCCCCTGCTGCTTTCAAGTACTTCGACACAACTATCGATCGCAAGAGGTACACAAGCACTAAGGAGGTCCTGGACGCGACCCTCATCCACCAGTCGATTACCGGCCTCTACGAGACGCGCATCGACCTGTCTCAGCTCGGGGGCGACGAATTCTCCGGGAGCGAGACGCCAGGCACCTCCGAGTCGGCCACCCCAGAATCTGCCACAGTGGTGTCCGGCCAAAAGCAGGACCGCCAGGGCGGAGAACGCAGAAGGTCCCAGCTCGATAGGGATCAGTGTGCCTACTGCAAGGAGAAGGGCCACTGGGCCAAAGACTGCCCGAAAAAGCCGCGCGGCCCACGCGGCCCAAGGCCACAAACATCCCTCCTTCCAAAGAAGAAGCGGAAGGTGGAGCTCAGCGGAGGATCTTCCGGAGGATCTAGCGGCTCCGAGACACCAGGAACATCCGAAAGCGCTACACCAGAATCTAGCGGAGGCTCTTCCGGAGGATCTAGGCCTCCTCCCGTCGCGTGCCTCGTACTGAGCCTAGAGGAGGAGTACAGGCTGCATGAGCAAAATCCGAAGCAGCTGCTAGCCCCTGAATGGCTGAGCGCCTTCTCCGAGGTATGGGCGGAGCAGACTGGCATGGGACTGGCCAAGCAGGTGCCTCCTGTGGTGGTAGAGCTGAAGGCGGACGCCAGCCCAGTGAGCGTGAAACAATACCCGATGTCGAGGGAAGCAAAGGAAGGCATTAGACCCCACATCCAGAGACTGCTGCAATTGGGAATCCTGGTACCCTGCCAAAGCCCTTGGAACACGCCACTGCTGCCTGTGCGCAAACCAGGAACTAATGATTACAGACCTGTACAGGATCTAAGGGAAGTTAACAAGCGGGTGCAGGACATACATCCTACGGTCCCAAACCCCTACAACTTGCTGAGCTCTCTGCCCCCCGAGCGCACATGGTACACTGTGCTCGACTTAAAAGATGCGTTCTTCTGCCTTCGCCTCCACCCGAACTCACAGCCACTGTTCGCGTTCGAGTGGCGCGACCCTGAGGGCGGCCACACCGGGCAACTGACGTGGACACGGCTGCCACAGGGTTTCAAGAACAGCCCTACTCTATTTAACGAGGCCCTGCACCGTGATCTGGCCCCCTTCCGGGCGCAGAACCCTCAAATCAGTCTACTGCAGTATGCGGATGACCTGCTGCTAGCCGCTTCTACTCAGGAGCTCTGCCGGGAGGGCACAAAGCGGCTGTTAAATGAGCTGGGCGAGCTGGGATACCGCGTGTCTGCCAAGAAGGCACAATTGTGTCGGACAGAGGTGACATATCTCGGCTACACATTAAGAGAAGGTAAGAGATGGCTCACTGAAGCACGTAAGAAGACTGTCATGCAGATTCCTACCCCTACCACGCCCCGTCAGGTGCGGGAGTTCCTCGGCAAGGCCGGCTTCTGTAGACTGTTCATTCCTGGATTTGCCACCCTGGCCGCCCCACTCTACCTGCTGACGAAGCCAAAGGTTCCTTTCACCTGGACTGAGGAACATCAGCGGGCTTTCGAAGACATCAAAGCCGCCCTGCTGGCGGCTCCAGCTCTGGCCCTCCCTGACCTCACCAAGCCCTTCACACTCTATGTGGACGAGAGAGCTGGAGTGGCCAGAGGTGTGCTGACACAGGCACTGGGGCCGTGGAAGAGACCCGTGGCGTACCTATCTAAAAAGCTCGACCCCGTGGCCTCTGGGTGGCCGAGCTGTCTAAAGGCCATCGCTGCTGTAGCTCTACTGGTCAAAGATGCTGACAAGCTGACCCTGGGCCAGCATGTCACCGTCATCGCCCCGCACGCCTTAGAATCCATAGTCCGGCAACCACCAGATCGGTGGATGACAAACGCTAGGATGACGCATTATCAGAGCTTGCTGTTAAACGAAAGAGTGACATTTGCGCCCCCTGCCATCCTTAACCCAGCGACGCTACTGCCGGAGATCCACAACTCAACCCCCATCCACCAGTGCGTCGACATTCTGGCTGAGGAGACCGGCACGCGCAAGGACCTCACGGACAGACCATGGCCTGGTGTGCCGGCATGGTACACCGACGGCAGCTCCTTTGTCGTGGAGGGAAAGCGACGCGCAGGCGCGGCTGTGGTGGACGGCAAGCAAGTCATCTGGGCATCCTCCCTGCCAGAAGGCACCAGCGCTCAGAAAGCGGAGTTGGTGGCCTTGACCCAGGCCCTCAGATTAGCTGAAGGGAAGGCTATTAATATCTACACCGATTCTAGATACGCCTTCGCCACGGCGCACATCCACGGCGCGATCTACAAGCAGCGGGGCCTGCTGACCAGTGCCGGGCGAGACATCAAGAATAAGGAAGAGATTCTTGCCCTGCTGGAGGCCGTCCACCTCCCCAAAAAGGTGGCCATTATCCACTGCCCCGGCCACCAGAAGGGAGAGGATCCAATTACAAAGGGGAACCAGATGGCCGACCTCGTGGCGAAGCAGGTCGCTCAGCAGGTTACCATCCTGGCAGAAAAATCGCAGACCCCTGTGAAGACACCTGTGACAGATGATTCTAACTATAGCGGCGGCAGCAAAAGAACGGCGGACGGCTCTGAGAAGCGCACCGCTGATAGCCAGCATTCAACTCCTCCGAAAACAAAGAGGAAAGTTGAGTTCGAACCGAAGAAGAAAAGGAAGGTGTGAGAGCTCAGAGCTTTCGTTCGTATCATCGGTTTCGACAACGTTCGTCAAGTTCAATGCATCAGTTTCATTGCGCACACACCAGAATCCTACTGAGTTTGAGTATTATGGCATTGGGAAAACTGTTTTTCTTGTACCATTTGTTGTGCTTGTAATTTACTGTGTTTTTTATTCGGTTTTCGCTATCGAACTGTGAAATGGAAATGGATGGAGAAGAGTTAATGAATGATATGGTCCTTTTGTTCATTCTCAAATTAATATTATTTGTTTTTTCTCTTATTTGTTGTGTGTTGAATTTGAAATTATAAGAGATATGCAAACATTTTGTTTTGAGTAAAAATGTGTCAAATCGTGGCCTCTAATGACCGAAGTTAATATGAGGAGTAAAACACTTGTAGTTGTACCATTATGCTTATTCACTAGGCAACAAATATATTTTCAGACCTAGAAAAGCTGCAAATGTTACTGAATACAAGTATGTCCTCTTGTGTTTTAGACATTTATGAACTTTCCTTTATGTAATTTTCCAGAATCCTTGTCAGATTCTAATCATTGCTTTATAATTATAGTTATACTCATGGATTTGTAGTTGAGTATGAAAATATTTTTTAATGCATTTTATGACTTGCCAATTGATTGACAACGAATTCGTAATCATGTCATAGCTGTTTCCTGTGTGAAATTGTTATCCGCTCACAATTCCACACAACATACGAGCCGGAAGCATAAAGTGTAAAGCCTGGGGTGCCTAATGAGTGAGCTAACTCACATTAATTGCGTTGCGCTCACTGCCCGCTTTCCAGTCGGGAAACCTGTCGTGCCAGCTGCATTAATGAATCGGCCAACGCGCGGGGAGAGGCGGTTTGCGTATTGGCTAGAGCAGCTTGCCAACATGGTGGAGCACGACACTCTCGTCTACTCCAAGAATATCAAAGATACAGTCTCAGAAGACCAAAGGGCTATTGAGACTTTTCAACAAAGGGTAATATCGGGAAACCTCCTCGGATTCCATTGCCCAGCTATCTGTCACTTCATCAAAAGGACAGTAGAAAAGGAAGGTGGCACCTACAAATGCCATCATTGCGATAAAGGAAAGGCTATCGTTCAAGATGCCTCTGCCGACAGTGGTCCCAAAGATGGACCCCCACCCACGAGGAGCATCGTGGAAAAAGAAGACGTTCCAACCACGTCTTCAAAGCAAGTGGATTGATGTGAACATGGTGGAGCACGACACTCTCGTCTACTCCAAGAATATCAAAGATACAGTCTCAGAAGACCAAAGGGCTATTGAGACTTTTCAACAAAGGGTAATATCGGGAAACCTCCTCGGATTCCATTGCCCAGCTATCTGTCACTTCATCAAAAGGACAGTAGAAAAGGAAGGTGGCACCTACAAATGCCATCATTGCGATAAAGGAAAGGCTATCGTTCAAGATGCCTCTGCCGACAGTGGTCCCAAAGATGGACCCCCACCCACGAGGAGCATCGTGGAAAAAGAAGACGTTCCAACCACGTCTTCAAAGCAAGTGGATTGATGTGATATCTCCACTGACGTAAGGGATGACGCACAATCCCACTATCCTTCGCAAGACCCTTCCTCTATATAAGGAAGTTCATTTCATTTGGAGAGGACACGCTGAAATCACCAGTCTCTCTCTACAAATCTATCTCTCTCGAGCTTTCGCAGATCCGGGGGGCAATGAGATATGAAAAAGCCTGAACTCACCGCGACGTCTGTCGAGAAGTTTCTGATCGAAAAGTTCGACAGCGTCTCCGACCTGATGCAGCTCTCGGAGGGCGAAGAATCTCGTGCTTTCAGCTTCGATGTAGGAGGGCGTGGATATGTCCTGCGGGTAAATAGCTGCGCCGATGGTTTCTACAAAGATCGTTATGTTTATCGGCACTTTGCATCGGCCGCGCTCCCGATTCCGGAAGTGCTTGACATTGGGGAGTTTAGCGAGAGCCTGACCTATTGCATCTCCCGCCGTTCACAGGGTGTCACGTTGCAAGACCTGCCTGAAACCGAACTGCCCGCTGTTCTACAACCGGTCGCGGAGGCTATGGATGCGATCGCTGCGGCCGATCTTAGCCAGACGAGCGGGTTCGGCCCATTCGGACCGCAAGGAATCGGTCAATACACTACATGGCGTGATTTCATATGCGCGATTGCTGATCCCCATGTGTATCACTGGCAAACTGTGATGGACGACACCGTCAGTGCGTCCGTCGCGCAGGCTCTCGATGAGCTGATGCTTTGGGCCGAGGACTGCCCCGAAGTCCGGCACCTCGTGCACGCGGATTTCGGCTCCAACAATGTCCTGACGGACAATGGCCGCATAACAGCGGTCATTGACTGGAGCGAGGCGATGTTCGGGGATTCCCAATACGAGGTCGCCAACATCTTCTTCTGGAGGCCGTGGTTGGCTTGTATGGAGCAGCAGACGCGCTACTTCGAGCGGAGGCATCCGGAGCTTGCAGGATCGCCACGACTCCGGGCGTATATGCTCCGCATTGGTCTTGACCAACTCTATCAGAGCTTGGTTGACGGCAATTTCGATGATGCAGCTTGGGCGCAGGGTCGATGCGACGCAATCGTCCGATCCGGAGCCGGGACTGTCGGGCGTACACAAATCGCCCGCAGAAGCGCGGCCGTCTGGACCGATGGCTGTGTAGAAGTACTCGCCGATAGTGGAAACCGACGCCCCAGCACTCGTCCGAGGGCAAAGAAATAGAGTAGATGCCGACCGGGATCTGTCGATCGACAAGCTCGAGTTTCTCCATAATAATGTGTGAGTAGTTCCCAGATAAGGGAATTAGGGTTCCTATAGGGTTTCGCTCATGTGTTGAGCATATAAGAAACCCTTAGTATGTATTTGTATTTGTAAAATACTTCTATCAATAAAATTTCTAATTCCTAAAACCAAAATCCAGTACTAAAATCCAGATCCCCCGAATTAATTCGGCGTTAATTCAGTACATTAAAAACGTCCGCAATGTGTTATTAAGTTGTCTAAGCGTCAATTTGTTTACACCACAATATATCCTGCCA

**Sequence 74 Plasmids sequence of pH-35C-RERV-m5-OsGAPDH**

(RB T-DNA repeat-35C promoter-tRNAGly RS-pegRNA-Ubi promoter-NLSc-myc-NLSsv40-nCas9(H840A/ R221K/N394K)-XTEN-NC-NLS-32aa Linker-RERV-RT-m5-NLSvbp-HygR cassette-E9-terminator-CaMV poly(A) signal-LB T-DNA repeat)

GTTTACCCGCCAATATATCCTGTCAAACACTGATAGTTTAAACTGAAGGCGGGAAACGACAATCTGATCCAAGCTCAAGCTGCTCTAGCATTCGCCATTCAGGCTGCGCAACTGTTGGGAAGGGCGATCGGTGCGGGCCTCTTCGCTATTACGCCAGCTGGCGAAAGGGGGATGTGCTGCAAGGCGATTAAGTTGGGTAACGCCAGGGTTTTCCCAGTCACGACGTTGTAAAACGACGGCCAGTGAATTCATGGAGTCAAAGATTCAAATAGAGGACCTAACAGAACTCGCCGTAAAGACTGGCGAACAGTTCATACAGAGTCTCTTACGACTCAATGACAAGAAGAAAATCTTCGTCAACATGGTGGAGCACGACACACTTGTCTACTCCAAAAATATCAAAGATACAGTCTCAGAAGACCAAAGGGCAATTGAGACTTTTCAACAAAGGGTAATATCCGGAAACCTCCTCGGATTCCATTGCCCAGCTATCTGTCACTTTATTGTGAAGATAGTGGAAAAGGAAGGTGGCTCCTACAAATGCCATCATTGCGATAAAGGAAAGGCCATCGTTGAAGATGCCTCTGCCGACAGTGGTCCCAAAGATGGACCCCCACCCACGAGGAGCATCGTGGAAAAAGAAGACGTTCCAACCACGTCTTCAAAGCAAGTGGATTGATGTGATTGGCAGACATACTGTCCCACAAATGAAGATGGAATCTGTAAAAGAAAACGCGTGAAATAATGCGTCTGACAAAGGTTAGGTCGGCTGCCTTTAATCAATACCAAAGTGGTCCCTACCACGATGGAAAAACTGTGCAGTCGGTTTGGCTTTTTCTGACGAACAAATAAGATTCGTGGCCGACAGGTGGGGGTCCACCATGTGAAGGCATCTTCAGACTCCAATAATGGAGCAATGACGTAAGGGCTTACGAAATAAGTAAGGGTAGTTTGGGAAATGTCCACTCACCCGTCAGTCTATAAATACTTAGCCCCTCCCTCATTGTTAAGGGAGCAAAATCTCAGAGAGATAGTCCTAGAGAGAGAAAGAGAGCAAGTAGCCTAGAAGTAGTCAAGGCGGCGAAGTATTCAGGCACGTGGCCAGGAAGAAGAAAAGCCAAGACGACGAAAACAGGTAAGAGCTAAGCATCTAGATAAGTTGAAAACAATCTTCAAAAGTCCCACATCGCTTAGATAAGAAAACGAAGCTGAGTTTATATACAGCTAGAGTCGAAGTAGTGATTGAACAAAGCACCAGTGGTCTAGTGGTAGAATAGTACCCTGCCACGGTACAGACCCGGGTTCGATTCCCGGCTGGTGCAGAGTATGTCGTGGAGTCCACGTTTAAGAGCTATGCTGGAAACAGCATAGCAAGTTTAAATAAGGCTAGTCCGTTATCAACTTGAAAAAGTGGCACCGAGTCGGTGCAGTGAAGACACCGTTGGACTCCACGCGCGGTTCTATCTAGTTACGCGTTAAACCAACTAGAATTTTTTTGGCCGGCATGGTCCCAGCCTCCTCGCTGGCGCCGGCTGGGCAACATGCTTCGGCATGGCGAATGGGACTTTTTTTTGATATCTCCGGGGCTAATTGAATATGAAGATGAAGATGAAATATTTGGTGTGTCAAATAAAAAGCTGGTGTGCTTAAGTTTGTGTTTTTTTCTTGGCTTGTTGTGTTATGAATTTGTGGCTTTTTCTAATATTAAATGAATGTAAGATCTCATTATAATGAATAAACAAATGTTTCTATAATCCATTGTGAATGTTTTGTTGGATCTCTTCTGCAGCATATAACTACTGTATGTGCTATGGTATGGACTATGGAATATGATTAAAGATAAGAAGCTTGGCGTAATCTGCATGCCTGCAGTGCAGCGTGACCCGGTCGTGCCCCTCTCTAGAGATAATGAGCATTGCATGTCTAAGTTATAAAAAATTACCACATATTTTTTTTGTCACACTTGTTTGAAGTGCAGTTTATCTATCTTTATACATATATTTAAACTTTACTCTACGAATAATATAATCTATAGTACTACAATAATATCAGTGTTTTAGAGAATCATATAAATGAACAGTTAGACATGGTCTAAAGGACAATTGAGTATTTTGACAACAGGACTCTACAGTTTTATCTTTTTAGTGTGCATGTGTTCTCCTTTTTTTTTGCAAATAGCTTCACCTATATAATACTTCATCCATTTTATTAGTACATCCATTTAGGGTTTAGGGTTAATGGTTTTTATAGACTAATTTTTTTAGTACATCTATTTTATTCTATTTTAGCCTCTAAATTAAGAAAACTAAAACTCTATTTTAGTTTTTTTATTTAATAATTTAGATATAAAATAGAATAAAATAAAGTGACTAAAAATTAAACAAATACCCTTTAAGAAATTAAAAAAACTAAGGAAACATTTTTCTTGTTTCGAGTAGATAATGCCAGCCTGTTAAACGCCGTCGACGAGTCTAACGGACACCAACCAGCGAACCAGCAGCGTCGCGTCGGGCCAAGCGAAGCAGACGGCACGGCATCTCTGTCGCTGCCTCTGGACCCCTCTCGAGAGTTCCGCTCCACCGTTGGACTTGCTCCGCTGTCGGCATCCAGAAATTGCGTGGCGGAGCGGCAGACGTGAGCCGGCACGGCAGGCGGCCTCCTCCTCCTCTCACGGCACCGGCAGCTACGGGGGATTCCTTTCCCACCGCTCCTTCGCTTTCCCTTCCTCGCCCGCCGTAATAAATAGACACCCCCTCCACACCCTCTTTCCCCAACCTCGTGTTGTTCGGAGCGCACACACACACAACCAGATCTCCCCCAAATCCACCCGTCGGCACCTCCGCTTCAAGGTACGCCGCTCGTCCTCCCCCCCCCCCCCTCTCTACCTTCTCTAGATCGGCGTTCCGGTCCATGGTTAGGGCCCGGTAGTTCTACTTCTGTTCATGTTTGTGTTAGATCCGTGTTTGTGTTAGATCCGTGCTGCTAGCGTTCGTACACGGATGCGACCTGTACGTCAGACACGTTCTGATTGCTAACTTGCCAGTGTTTCTCTTTGGGGAATCCTGGGATGGCTCTAGCCGTTCCGCAGACGGGATCGATTTCATGATTTTTTTTGTTTCGTTGCATAGGGTTTGGTTTGCCCTTTTCCTTTATTTCAATATATGCCGTGCACTTGTTTGTCGGGTCATCTTTTCATGCTTTTTTTTGTCTTGGTTGTGATGATGTGGTCTGGTTGGGCGGTCGTTCTAGATCGGAGTAGAATTCTGTTTCAAACTACCTGGTGGATTTATTAATTTTGGATCTGTATGTGTGTGCCATACATATTCATAGTTACGAATTGAAGATGATGGATGGAAATATCGATCTAGGATAGGTATACATGTTGATGCGGGTTTTACTGATGCATATACAGAGATGCTTTTTGTTCGCTTGGTTGTGATGATGTGGTGTGGTTGGGCGGTCGTTCATTCGTTCTAGATCGGAGTAGAATACTGTTTCAAACTACCTGGTGTATTTATTAATTTTGGAACTGTATGTGTGTGTCATACATCTTCATAGTTACGAGTTTAAGATGGATGGAAATATCGATCTAGGATAGGTATACATGTTGATGTGGGTTTTACTGATGCATATACATGATGGCATATGCAGCATCTATTCATATGCTCTAACCTTGAGTACCTATCTATTATAATAAACAAGTATGTTTTATAATTATTTTGATCTTGATATACTTGGATGATGGCATATGCAGCAGCTATATGTGGATTTTTTTAGCCCTGCCTTCATACGCTATTTATTTGCTTGGTACTGTTTCTTTTGTCGATGCTCACCCTGTTGTTTGGTGTTACTTCTGCAGCCCTAGGACTGGTTGTACAATGCCGGCGGCCAAGAGAGTCAAGCTCGACGGCGGGAAGCGCACAGCTGATGGTTCTGAATTCGAGTCCCCTAAGAAAAAGAGAAAAGTGGACAAGAAGTACTCGATCGGCCTCGATATTGGGACTAACTCTGTTGGCTGGGCCGTGATCACCGACGAGTACAAGGTGCCCTCAAAGAAGTTCAAGGTCCTGGGCAACACCGATCGGCATTCCATCAAGAAGAATCTCATTGGCGCTCTCCTGTTCGACAGCGGCGAGACGGCTGAGGCTACGCGGCTCAAGCGCACCGCCCGCAGGCGGTACACGCGCAGGAAGAATCGCATCTGCTACCTGCAGGAGATTTTCTCCAACGAGATGGCGAAGGTTGACGATTCTTTCTTCCACAGGCTGGAGGAGTCATTCCTCGTGGAGGAGGATAAGAAGCACGAGCGGCATCCAATCTTCGGCAACATTGTCGACGAGGTTGCCTACCACGAGAAGTACCCTACGATCTACCATCTGCGGAAGAAGCTCGTGGACTCCACAGATAAGGCGGACCTCCGCCTGATCTACCTCGCTCTGGCCCACATGATTAAGTTCAGGGGCCATTTCCTGATCGAGGGGGATCTCAACCCGGACAATAGCGATGTTGACAAGCTGTTCATCCAGCTCGTGCAGACGTACAACCAGCTCTTCGAGGAGAACCCCATTAATGCGTCAGGCGTCGACGCGAAGGCTATCCTGTCCGCTAGGCTCTCGAAGTCTCGGAAGCTCGAGAACCTGATCGCCCAGCTGCCGGGCGAGAAGAAGAACGGCCTGTTCGGGAATCTCATTGCGCTCAGCCTGGGGCTCACGCCCAACTTCAAGTCGAATTTCGATCTCGCTGAGGACGCCAAGCTGCAGCTCTCCAAGGACACATACGACGATGACCTGGATAACCTCCTGGCCCAGATCGGCGATCAGTACGCGGACCTGTTCCTCGCTGCCAAGAATCTGTCGGACGCCATCCTCCTGTCTGATATTCTCAGGGTGAACACCGAGATTACGAAGGCTCCGCTCTCAGCCTCCATGATCAAGCGCTACGACGAGCACCATCAGGATCTGACCCTCCTGAAGGCGCTGGTCAGGCAGCAGCTCCCCGAGAAGTACAAGGAGATCTTCTTCGATCAGTCGAAGAACGGCTACGCTGGGTACATTGACGGCGGGGCCTCTCAGGAGGAGTTCTACAAGTTCATCAAGCCGATTCTGGAGAAGATGGACGGCACGGAGGAGCTGCTGGTGAAGCTCAAGCGCGAGGACCTCCTGAGGAAGCAGCGGACATTCGATAACGGCAGCATCCCACACCAGATTCATCTCGGGGAGCTGCACGCTATCCTGAGGAGGCAGGAGGACTTCTACCCTTTCCTCAAGGATAACCGCGAGAAGATCGAGAAGATTCTGACTTTCAGGATCCCGTACTACGTCGGCCCACTCGCTAGGGGCAACTCCCGCTTCGCTTGGATGACCCGCAAGTCAGAGGAGACGATCACGCCGTGGAACTTCGAGGAGGTGGTCGACAAGGGCGCTAGCGCTCAGTCGTTCATCGAGAGGATGACGAATTTCGACAAGAACCTGCCAAATGAGAAGGTGCTCCCTAAGCACTCGCTCCTGTACGAGTACTTCACAGTCTACAACGAGCTGACTAAGGTGAAGTATGTGACCGAGGGCATGAGGAAGCCGGCTTTCCTGTCTGGGGAGCAGAAGAAGGCCATCGTGGACCTCCTGTTCAAGACCAACCGGAAGGTCACGGTTAAGCAGCTCAAGGAGGACTACTTCAAGAAGATTGAGTGCTTCGATTCGGTCGAGATCTCTGGCGTTGAGGACCGCTTCAACGCCTCCCTGGGGACCTACCACGATCTCCTGAAGATCATTAAGGATAAGGACTTCCTGGACAACGAGGAGAATGAGGATATCCTCGAGGACATTGTGCTGACACTCACTCTGTTCGAGGACCGGGAGATGATCGAGGAGCGCCTGAAGACTTACGCCCATCTCTTCGATGACAAGGTCATGAAGCAGCTCAAGAGGAGGAGGTACACCGGCTGGGGGAGGCTGAGCAGGAAGCTCATCAACGGCATTCGGGACAAGCAGTCCGGGAAGACGATCCTCGACTTCCTGAAGAGCGATGGCTTCGCGAACCGCAATTTCATGCAGCTGATTCACGATGACAGCCTCACATTCAAGGAGGATATCCAGAAGGCTCAGGTGAGCGGCCAGGGGGACTCGCTGCACGAGCATATCGCGAACCTCGCTGGCTCGCCAGCTATCAAGAAGGGGATTCTGCAGACCGTGAAGGTTGTGGACGAGCTGGTGAAGGTCATGGGCAGGCACAAGCCTGAGAACATCGTCATTGAGATGGCCCGGGAGAATCAGACCACGCAGAAGGGCCAGAAGAACTCACGCGAGAGGATGAAGAGGATCGAGGAGGGCATTAAGGAGCTGGGGTCCCAGATCCTCAAGGAGCACCCGGTGGAGAACACGCAGCTGCAGAATGAGAAGCTCTACCTGTACTACCTCCAGAATGGCCGCGATATGTATGTGGACCAGGAGCTGGATATTAACAGGCTCAGCGATTACGACGTCGATGCCATCGTTCCACAGTCATTCCTGAAGGATGACTCCATTGACAACAAGGTCCTCACCAGGTCGGACAAGAACCGGGGCAAGTCTGATAATGTTCCTTCAGAGGAGGTCGTTAAGAAGATGAAGAACTACTGGCGCCAGCTCCTGAATGCCAAGCTGATCACGCAGCGGAAGTTCGATAACCTCACAAAGGCTGAGAGGGGCGGGCTCTCTGAGCTGGACAAGGCGGGCTTCATCAAGAGGCAGCTGGTCGAGACACGGCAGATCACTAAGCACGTTGCGCAGATTCTCGACTCACGGATGAACACTAAGTACGATGAGAATGACAAGCTGATCCGCGAGGTGAAGGTCATCACCCTGAAGTCAAAGCTCGTCTCCGACTTCAGGAAGGATTTCCAGTTCTACAAGGTTCGGGAGATCAACAATTACCACCATGCCCATGACGCGTACCTGAACGCGGTGGTCGGCACAGCTCTGATCAAGAAGTACCCAAAGCTCGAGAGCGAGTTCGTGTACGGGGACTACAAGGTTTACGATGTGAGGAAGATGATCGCCAAGTCGGAGCAGGAGATTGGCAAGGCTACCGCCAAGTACTTCTTCTACTCTAACATTATGAATTTCTTCAAGACAGAGATCACTCTGGCCAATGGCGAGATCCGGAAGCGCCCCCTCATCGAGACGAACGGCGAGACGGGGGAGATCGTGTGGGACAAGGGCAGGGATTTCGCGACCGTCAGGAAGGTTCTCTCCATGCCACAAGTGAATATCGTCAAGAAGACAGAGGTCCAGACTGGCGGGTTCTCTAAGGAGTCAATTCTGCCTAAGCGGAACAGCGACAAGCTCATCGCCCGCAAGAAGGACTGGGATCCGAAGAAGTACGGCGGGTTCGACAGCCCCACTGTGGCCTACTCGGTCCTGGTTGTGGCGAAGGTTGAGAAGGGCAAGTCCAAGAAGCTCAAGAGCGTGAAGGAGCTGCTGGGGATCACGATTATGGAGCGCTCCAGCTTCGAGAAGAACCCGATCGATTTCCTGGAGGCGAAGGGCTACAAGGAGGTGAAGAAGGACCTGATCATTAAGCTCCCCAAGTACTCACTCTTCGAGCTGGAGAACGGCAGGAAGCGGATGCTGGCTTCCGCTGGCGAGCTGCAGAAGGGGAACGAGCTGGCTCTGCCGTCCAAGTATGTGAACTTCCTCTACCTGGCCTCCCACTACGAGAAGCTCAAGGGCAGCCCCGAGGACAACGAGCAGAAGCAGCTGTTCGTCGAGCAGCACAAGCATTACCTCGACGAGATCATTGAGCAGATTTCCGAGTTCTCCAAGCGCGTGATCCTGGCCGACGCGAATCTGGATAAGGTCCTCTCCGCGTACAACAAGCACCGCGACAAGCCAATCAGGGAGCAGGCTGAGAATATCATTCATCTCTTCACCCTGACGAACCTCGGCGCCCCTGCTGCTTTCAAGTACTTCGACACAACTATCGATCGCAAGAGGTACACAAGCACTAAGGAGGTCCTGGACGCGACCCTCATCCACCAGTCGATTACCGGCCTCTACGAGACGCGCATCGACCTGTCTCAGCTCGGGGGCGACGAATTCTCCGGGAGCGAGACGCCAGGCACCTCCGAGTCGGCCACCCCAGAATCTGCCACAGTGGTGTCCGGCCAAAAGCAGGACCGCCAGGGCGGAGAACGCAGAAGGTCCCAGCTCGATAGGGATCAGTGTGCCTACTGCAAGGAGAAGGGCCACTGGGCCAAAGACTGCCCGAAAAAGCCGCGCGGCCCACGCGGCCCAAGGCCACAAACATCCCTCCTTCCAAAGAAGAAGCGGAAGGTGGAGCTCAGCGGAGGATCTTCCGGAGGATCTAGCGGCTCCGAGACACCAGGAACATCCGAAAGCGCTACACCAGAATCTAGCGGAGGCTCTTCCGGAGGATCTAGGCCTCCTCCCGTCGCGTGCCTCGTACTGAGCCTAGAGGAGGAGTACAGGCTGCATGAGCAAAATCCGAAGCAGCTGCTAGCCCCTGAATGGCTGAGCGCCTTCTCCGAGGTATGGGCGGAGCAGACTGGCATGGGACTGGCCAAGCAGGTGCCTCCTGTGGTGGTAGAGCTGAAGGCGGACGCCAGCCCAGTGAGCGTGAAACAATACCCGATGTCGAGGGAAGCAAAGGAAGGCATTAGACCCCACATCCAGAGACTGCTGCAATTGGGAATCCTGGTACCCTGCCAAAGCCCTTGGAACACGCCACTGCTGCCTGTGCGCAAACCAGGAACTAATGATTACAGACCTGTACAGGATCTAAGGGAAGTTAACAAGCGGGTGCAGGACATACATCCTACGGTCCCAAACCCCTACAACTTGCTGAGCTCTCTGCCCCCCGAGCGCACATGGTACACTGTGCTCGACTTAAAAGATGCGTTCTTCTGCCTTCGCCTCCACCCGAACTCACAGCCACTGTTCGCGTTCGAGTGGCGCGACCCTGAGGGCGGCCACACCGGGCAACTGACGTGGACACGGCTGCCACAGGGTTTCAAGAACAGCCCTACTCTATTTAACGAGGCCCTGCACCGTGATCTGGCCCCCTTCCGGGCGCAGAACCCTCAAATCAGTCTACTGCAGTATGCGGATGACCTGCTGCTAGCCGCTTCTACTCAGGAGCTCTGCCGGGAGGGCACAAAGCGGCTGTTAAATGAGCTGGGCGAGCTGGGATACCGCGTGTCTGCCAAGAAGGCACAATTGTGTCGGACAGAGGTGACATATCTCGGCTACACATTAAGAGAAGGTAAGAGATGGCTCACTGAAGCACGTAAGAAGACTGTCATGCAGATTCCTACCCCTACCACGCCCCGTCAGGTGCGGGAGTTCCTCGGCAAGGCCGGCTTCTGTAGACTGTTCATTCCTGGATTTGCCACCCTGGCCGCCCCACTCTACCTGCTGACGAAGCCAAAGGTTCCTTTCACCTGGACTGAGGAACATCAGCGGGCTTTCGAAGACATCAAAGCCGCCCTGCTGGCGGCTCCAGCTCTGGCCCTCCCTGACCTCACCAAGCCCTTCACACTCTATGTGGACGAGAGAGCTGGAGTGGCCAGAGGTGTGCTGACACAGGCACTGGGGCCGTGGAAGAGACCCGTGGCGTACCTATCTAAAAAGCTCGACCCCGTGGCCTCTGGGTGGCCGAGCTGTCTAAAGGCCATCGCTGCTGTAGCTCTACTGGTCAAAGATGCTGACAAGCTGACCCTGGGCCAGCATGTCACCGTCATCGCCCCGCACGCCTTAGAATCCATAGTCCGGCAACCACCAGATCGGTGGATGACAAACGCTAGGATGACGCATTATCAGAGCTTGCTGTTAAACGAAAGAGTGACATTTGCGCCCCCTGCCATCCTTAACCCAGCGACGCTACTGCCGGAGATCCACAACTCAACCCCCATCCACCAGTGCGTCGACATTCTGGCTGAGGAGACCGGCACGCGCAAGGACCTCACGGACAGACCATGGCCTGGTGTGCCGGCATGGTACACCGACGGCAGCTCCTTTGTCGTGGAGGGAAAGCGACGCGCAGGCGCGGCTGTGGTGGACGGCAAGCAAGTCATCTGGGCATCCTCCCTGCCAGAAGGCACCAGCGCTCAGAAAGCGGAGTTGGTGGCCTTGACCCAGGCCCTCAGATTAGCTGAAGGGAAGGCTATTAATATCTACACCGATTCTAGATACGCCTTCGCCACGGCGCACATCCACGGCGCGATCTACAAGCAGCGGGGCCTGCTGACCAGTGCCGGGCGAGACATCAAGAATAAGGAAGAGATTCTTGCCCTGCTGGAGGCCGTCCACCTCCCCAAAAAGGTGGCCATTATCCACTGCCCCGGCCACCAGAAGGGAGAGGATCCAATTACAAAGGGGAACCAGATGGCCGACCTCGTGGCGAAGCAGGTCGCTCAGCAGGTTACCATCCTGGCAGAAAAATCGCAGACCCCTGTGAAGACACCTGTGACAGATGATTCTAACTATAGCGGCGGCAGCAAAAGAACGGCGGACGGCTCTGAGAAGCGCACCGCTGATAGCCAGCATTCAACTCCTCCGAAAACAAAGAGGAAAGTTGAGTTCGAACCGAAGAAGAAAAGGAAGGTGTGAGAGCTCAGAGCTTTCGTTCGTATCATCGGTTTCGACAACGTTCGTCAAGTTCAATGCATCAGTTTCATTGCGCACACACCAGAATCCTACTGAGTTTGAGTATTATGGCATTGGGAAAACTGTTTTTCTTGTACCATTTGTTGTGCTTGTAATTTACTGTGTTTTTTATTCGGTTTTCGCTATCGAACTGTGAAATGGAAATGGATGGAGAAGAGTTAATGAATGATATGGTCCTTTTGTTCATTCTCAAATTAATATTATTTGTTTTTTCTCTTATTTGTTGTGTGTTGAATTTGAAATTATAAGAGATATGCAAACATTTTGTTTTGAGTAAAAATGTGTCAAATCGTGGCCTCTAATGACCGAAGTTAATATGAGGAGTAAAACACTTGTAGTTGTACCATTATGCTTATTCACTAGGCAACAAATATATTTTCAGACCTAGAAAAGCTGCAAATGTTACTGAATACAAGTATGTCCTCTTGTGTTTTAGACATTTATGAACTTTCCTTTATGTAATTTTCCAGAATCCTTGTCAGATTCTAATCATTGCTTTATAATTATAGTTATACTCATGGATTTGTAGTTGAGTATGAAAATATTTTTTAATGCATTTTATGACTTGCCAATTGATTGACAACGAATTCGTAATCATGTCATAGCTGTTTCCTGTGTGAAATTGTTATCCGCTCACAATTCCACACAACATACGAGCCGGAAGCATAAAGTGTAAAGCCTGGGGTGCCTAATGAGTGAGCTAACTCACATTAATTGCGTTGCGCTCACTGCCCGCTTTCCAGTCGGGAAACCTGTCGTGCCAGCTGCATTAATGAATCGGCCAACGCGCGGGGAGAGGCGGTTTGCGTATTGGCTAGAGCAGCTTGCCAACATGGTGGAGCACGACACTCTCGTCTACTCCAAGAATATCAAAGATACAGTCTCAGAAGACCAAAGGGCTATTGAGACTTTTCAACAAAGGGTAATATCGGGAAACCTCCTCGGATTCCATTGCCCAGCTATCTGTCACTTCATCAAAAGGACAGTAGAAAAGGAAGGTGGCACCTACAAATGCCATCATTGCGATAAAGGAAAGGCTATCGTTCAAGATGCCTCTGCCGACAGTGGTCCCAAAGATGGACCCCCACCCACGAGGAGCATCGTGGAAAAAGAAGACGTTCCAACCACGTCTTCAAAGCAAGTGGATTGATGTGAACATGGTGGAGCACGACACTCTCGTCTACTCCAAGAATATCAAAGATACAGTCTCAGAAGACCAAAGGGCTATTGAGACTTTTCAACAAAGGGTAATATCGGGAAACCTCCTCGGATTCCATTGCCCAGCTATCTGTCACTTCATCAAAAGGACAGTAGAAAAGGAAGGTGGCACCTACAAATGCCATCATTGCGATAAAGGAAAGGCTATCGTTCAAGATGCCTCTGCCGACAGTGGTCCCAAAGATGGACCCCCACCCACGAGGAGCATCGTGGAAAAAGAAGACGTTCCAACCACGTCTTCAAAGCAAGTGGATTGATGTGATATCTCCACTGACGTAAGGGATGACGCACAATCCCACTATCCTTCGCAAGACCCTTCCTCTATATAAGGAAGTTCATTTCATTTGGAGAGGACACGCTGAAATCACCAGTCTCTCTCTACAAATCTATCTCTCTCGAGCTTTCGCAGATCCGGGGGGCAATGAGATATGAAAAAGCCTGAACTCACCGCGACGTCTGTCGAGAAGTTTCTGATCGAAAAGTTCGACAGCGTCTCCGACCTGATGCAGCTCTCGGAGGGCGAAGAATCTCGTGCTTTCAGCTTCGATGTAGGAGGGCGTGGATATGTCCTGCGGGTAAATAGCTGCGCCGATGGTTTCTACAAAGATCGTTATGTTTATCGGCACTTTGCATCGGCCGCGCTCCCGATTCCGGAAGTGCTTGACATTGGGGAGTTTAGCGAGAGCCTGACCTATTGCATCTCCCGCCGTTCACAGGGTGTCACGTTGCAAGACCTGCCTGAAACCGAACTGCCCGCTGTTCTACAACCGGTCGCGGAGGCTATGGATGCGATCGCTGCGGCCGATCTTAGCCAGACGAGCGGGTTCGGCCCATTCGGACCGCAAGGAATCGGTCAATACACTACATGGCGTGATTTCATATGCGCGATTGCTGATCCCCATGTGTATCACTGGCAAACTGTGATGGACGACACCGTCAGTGCGTCCGTCGCGCAGGCTCTCGATGAGCTGATGCTTTGGGCCGAGGACTGCCCCGAAGTCCGGCACCTCGTGCACGCGGATTTCGGCTCCAACAATGTCCTGACGGACAATGGCCGCATAACAGCGGTCATTGACTGGAGCGAGGCGATGTTCGGGGATTCCCAATACGAGGTCGCCAACATCTTCTTCTGGAGGCCGTGGTTGGCTTGTATGGAGCAGCAGACGCGCTACTTCGAGCGGAGGCATCCGGAGCTTGCAGGATCGCCACGACTCCGGGCGTATATGCTCCGCATTGGTCTTGACCAACTCTATCAGAGCTTGGTTGACGGCAATTTCGATGATGCAGCTTGGGCGCAGGGTCGATGCGACGCAATCGTCCGATCCGGAGCCGGGACTGTCGGGCGTACACAAATCGCCCGCAGAAGCGCGGCCGTCTGGACCGATGGCTGTGTAGAAGTACTCGCCGATAGTGGAAACCGACGCCCCAGCACTCGTCCGAGGGCAAAGAAATAGAGTAGATGCCGACCGGGATCTGTCGATCGACAAGCTCGAGTTTCTCCATAATAATGTGTGAGTAGTTCCCAGATAAGGGAATTAGGGTTCCTATAGGGTTTCGCTCATGTGTTGAGCATATAAGAAACCCTTAGTATGTATTTGTATTTGTAAAATACTTCTATCAATAAAATTTCTAATTCCTAAAACCAAAATCCAGTACTAAAATCCAGATCCCCCGAATTAATTCGGCGTTAATTCAGTACATTAAAAACGTCCGCAATGTGTTATTAAGTTGTCTAAGCGTCAATTTGTTTACACCACAATATATCCTGCCA

**Sequence 75 Plasmids sequence of pH-35C-RERV-m5-OsACC1**

(RB T-DNA repeat-35C promoter-tRNAGly RS-pegRNA-Ubi promoter-NLSc-myc-NLSsv40-nCas9(H840A/ R221K/N394K)-XTEN-NC-NLS-32aa Linker-RERV-RT-m5-NLSvbp-HygR cassette-E9-terminator-CaMV poly(A) signal-LB T-DNA repeat)

GTTTACCCGCCAATATATCCTGTCAAACACTGATAGTTTAAACTGAAGGCGGGAAACGACAATCTGATCCAAGCTCAAGCTGCTCTAGCATTCGCCATTCAGGCTGCGCAACTGTTGGGAAGGGCGATCGGTGCGGGCCTCTTCGCTATTACGCCAGCTGGCGAAAGGGGGATGTGCTGCAAGGCGATTAAGTTGGGTAACGCCAGGGTTTTCCCAGTCACGACGTTGTAAAACGACGGCCAGTGAATTCATGGAGTCAAAGATTCAAATAGAGGACCTAACAGAACTCGCCGTAAAGACTGGCGAACAGTTCATACAGAGTCTCTTACGACTCAATGACAAGAAGAAAATCTTCGTCAACATGGTGGAGCACGACACACTTGTCTACTCCAAAAATATCAAAGATACAGTCTCAGAAGACCAAAGGGCAATTGAGACTTTTCAACAAAGGGTAATATCCGGAAACCTCCTCGGATTCCATTGCCCAGCTATCTGTCACTTTATTGTGAAGATAGTGGAAAAGGAAGGTGGCTCCTACAAATGCCATCATTGCGATAAAGGAAAGGCCATCGTTGAAGATGCCTCTGCCGACAGTGGTCCCAAAGATGGACCCCCACCCACGAGGAGCATCGTGGAAAAAGAAGACGTTCCAACCACGTCTTCAAAGCAAGTGGATTGATGTGATTGGCAGACATACTGTCCCACAAATGAAGATGGAATCTGTAAAAGAAAACGCGTGAAATAATGCGTCTGACAAAGGTTAGGTCGGCTGCCTTTAATCAATACCAAAGTGGTCCCTACCACGATGGAAAAACTGTGCAGTCGGTTTGGCTTTTTCTGACGAACAAATAAGATTCGTGGCCGACAGGTGGGGGTCCACCATGTGAAGGCATCTTCAGACTCCAATAATGGAGCAATGACGTAAGGGCTTACGAAATAAGTAAGGGTAGTTTGGGAAATGTCCACTCACCCGTCAGTCTATAAATACTTAGCCCCTCCCTCATTGTTAAGGGAGCAAAATCTCAGAGAGATAGTCCTAGAGAGAGAAAGAGAGCAAGTAGCCTAGAAGTAGTCAAGGCGGCGAAGTATTCAGGCACGTGGCCAGGAAGAAGAAAAGCCAAGACGACGAAAACAGGTAAGAGCTAAGCATCTAGATAAGTTGAAAACAATCTTCAAAAGTCCCACATCGCTTAGATAAGAAAACGAAGCTGAGTTTATATACAGCTAGAGTCGAAGTAGTGATTGAACAAAGCACCAGTGGTCTAGTGGTAGAATAGTACCCTGCCACGGTACAGACCCGGGTTCGATTCCCGGCTGGTGCATTCCTCGTGCTGGACAAGTGGTTTAAGAGCTATGCTGGAAACAGCATAGCAAGTTTAAATAAGGCTAGTCCGTTATCAACTTGAAAAAGTGGCACCGAGTCGGTGCATCTGGAAAGCACACTTGTCCAGCACGCGGTTCTATCTAGTTACGCGTTAAACCAACTAGAATTTTTTTGGCCGGCATGGTCCCAGCCTCCTCGCTGGCGCCGGCTGGGCAACATGCTTCGGCATGGCGAATGGGACTTTTTTTTGATATCTCCGGGGCTAATTGAATATGAAGATGAAGATGAAATATTTGGTGTGTCAAATAAAAAGCTGGTGTGCTTAAGTTTGTGTTTTTTTCTTGGCTTGTTGTGTTATGAATTTGTGGCTTTTTCTAATATTAAATGAATGTAAGATCTCATTATAATGAATAAACAAATGTTTCTATAATCCATTGTGAATGTTTTGTTGGATCTCTTCTGCAGCATATAACTACTGTATGTGCTATGGTATGGACTATGGAATATGATTAAAGATAAGAAGCTTGGCGTAATCTGCATGCCTGCAGTGCAGCGTGACCCGGTCGTGCCCCTCTCTAGAGATAATGAGCATTGCATGTCTAAGTTATAAAAAATTACCACATATTTTTTTTGTCACACTTGTTTGAAGTGCAGTTTATCTATCTTTATACATATATTTAAACTTTACTCTACGAATAATATAATCTATAGTACTACAATAATATCAGTGTTTTAGAGAATCATATAAATGAACAGTTAGACATGGTCTAAAGGACAATTGAGTATTTTGACAACAGGACTCTACAGTTTTATCTTTTTAGTGTGCATGTGTTCTCCTTTTTTTTTGCAAATAGCTTCACCTATATAATACTTCATCCATTTTATTAGTACATCCATTTAGGGTTTAGGGTTAATGGTTTTTATAGACTAATTTTTTTAGTACATCTATTTTATTCTATTTTAGCCTCTAAATTAAGAAAACTAAAACTCTATTTTAGTTTTTTTATTTAATAATTTAGATATAAAATAGAATAAAATAAAGTGACTAAAAATTAAACAAATACCCTTTAAGAAATTAAAAAAACTAAGGAAACATTTTTCTTGTTTCGAGTAGATAATGCCAGCCTGTTAAACGCCGTCGACGAGTCTAACGGACACCAACCAGCGAACCAGCAGCGTCGCGTCGGGCCAAGCGAAGCAGACGGCACGGCATCTCTGTCGCTGCCTCTGGACCCCTCTCGAGAGTTCCGCTCCACCGTTGGACTTGCTCCGCTGTCGGCATCCAGAAATTGCGTGGCGGAGCGGCAGACGTGAGCCGGCACGGCAGGCGGCCTCCTCCTCCTCTCACGGCACCGGCAGCTACGGGGGATTCCTTTCCCACCGCTCCTTCGCTTTCCCTTCCTCGCCCGCCGTAATAAATAGACACCCCCTCCACACCCTCTTTCCCCAACCTCGTGTTGTTCGGAGCGCACACACACACAACCAGATCTCCCCCAAATCCACCCGTCGGCACCTCCGCTTCAAGGTACGCCGCTCGTCCTCCCCCCCCCCCCCTCTCTACCTTCTCTAGATCGGCGTTCCGGTCCATGGTTAGGGCCCGGTAGTTCTACTTCTGTTCATGTTTGTGTTAGATCCGTGTTTGTGTTAGATCCGTGCTGCTAGCGTTCGTACACGGATGCGACCTGTACGTCAGACACGTTCTGATTGCTAACTTGCCAGTGTTTCTCTTTGGGGAATCCTGGGATGGCTCTAGCCGTTCCGCAGACGGGATCGATTTCATGATTTTTTTTGTTTCGTTGCATAGGGTTTGGTTTGCCCTTTTCCTTTATTTCAATATATGCCGTGCACTTGTTTGTCGGGTCATCTTTTCATGCTTTTTTTTGTCTTGGTTGTGATGATGTGGTCTGGTTGGGCGGTCGTTCTAGATCGGAGTAGAATTCTGTTTCAAACTACCTGGTGGATTTATTAATTTTGGATCTGTATGTGTGTGCCATACATATTCATAGTTACGAATTGAAGATGATGGATGGAAATATCGATCTAGGATAGGTATACATGTTGATGCGGGTTTTACTGATGCATATACAGAGATGCTTTTTGTTCGCTTGGTTGTGATGATGTGGTGTGGTTGGGCGGTCGTTCATTCGTTCTAGATCGGAGTAGAATACTGTTTCAAACTACCTGGTGTATTTATTAATTTTGGAACTGTATGTGTGTGTCATACATCTTCATAGTTACGAGTTTAAGATGGATGGAAATATCGATCTAGGATAGGTATACATGTTGATGTGGGTTTTACTGATGCATATACATGATGGCATATGCAGCATCTATTCATATGCTCTAACCTTGAGTACCTATCTATTATAATAAACAAGTATGTTTTATAATTATTTTGATCTTGATATACTTGGATGATGGCATATGCAGCAGCTATATGTGGATTTTTTTAGCCCTGCCTTCATACGCTATTTATTTGCTTGGTACTGTTTCTTTTGTCGATGCTCACCCTGTTGTTTGGTGTTACTTCTGCAGCCCTAGGACTGGTTGTACAATGCCGGCGGCCAAGAGAGTCAAGCTCGACGGCGGGAAGCGCACAGCTGATGGTTCTGAATTCGAGTCCCCTAAGAAAAAGAGAAAAGTGGACAAGAAGTACTCGATCGGCCTCGATATTGGGACTAACTCTGTTGGCTGGGCCGTGATCACCGACGAGTACAAGGTGCCCTCAAAGAAGTTCAAGGTCCTGGGCAACACCGATCGGCATTCCATCAAGAAGAATCTCATTGGCGCTCTCCTGTTCGACAGCGGCGAGACGGCTGAGGCTACGCGGCTCAAGCGCACCGCCCGCAGGCGGTACACGCGCAGGAAGAATCGCATCTGCTACCTGCAGGAGATTTTCTCCAACGAGATGGCGAAGGTTGACGATTCTTTCTTCCACAGGCTGGAGGAGTCATTCCTCGTGGAGGAGGATAAGAAGCACGAGCGGCATCCAATCTTCGGCAACATTGTCGACGAGGTTGCCTACCACGAGAAGTACCCTACGATCTACCATCTGCGGAAGAAGCTCGTGGACTCCACAGATAAGGCGGACCTCCGCCTGATCTACCTCGCTCTGGCCCACATGATTAAGTTCAGGGGCCATTTCCTGATCGAGGGGGATCTCAACCCGGACAATAGCGATGTTGACAAGCTGTTCATCCAGCTCGTGCAGACGTACAACCAGCTCTTCGAGGAGAACCCCATTAATGCGTCAGGCGTCGACGCGAAGGCTATCCTGTCCGCTAGGCTCTCGAAGTCTCGGAAGCTCGAGAACCTGATCGCCCAGCTGCCGGGCGAGAAGAAGAACGGCCTGTTCGGGAATCTCATTGCGCTCAGCCTGGGGCTCACGCCCAACTTCAAGTCGAATTTCGATCTCGCTGAGGACGCCAAGCTGCAGCTCTCCAAGGACACATACGACGATGACCTGGATAACCTCCTGGCCCAGATCGGCGATCAGTACGCGGACCTGTTCCTCGCTGCCAAGAATCTGTCGGACGCCATCCTCCTGTCTGATATTCTCAGGGTGAACACCGAGATTACGAAGGCTCCGCTCTCAGCCTCCATGATCAAGCGCTACGACGAGCACCATCAGGATCTGACCCTCCTGAAGGCGCTGGTCAGGCAGCAGCTCCCCGAGAAGTACAAGGAGATCTTCTTCGATCAGTCGAAGAACGGCTACGCTGGGTACATTGACGGCGGGGCCTCTCAGGAGGAGTTCTACAAGTTCATCAAGCCGATTCTGGAGAAGATGGACGGCACGGAGGAGCTGCTGGTGAAGCTCAAGCGCGAGGACCTCCTGAGGAAGCAGCGGACATTCGATAACGGCAGCATCCCACACCAGATTCATCTCGGGGAGCTGCACGCTATCCTGAGGAGGCAGGAGGACTTCTACCCTTTCCTCAAGGATAACCGCGAGAAGATCGAGAAGATTCTGACTTTCAGGATCCCGTACTACGTCGGCCCACTCGCTAGGGGCAACTCCCGCTTCGCTTGGATGACCCGCAAGTCAGAGGAGACGATCACGCCGTGGAACTTCGAGGAGGTGGTCGACAAGGGCGCTAGCGCTCAGTCGTTCATCGAGAGGATGACGAATTTCGACAAGAACCTGCCAAATGAGAAGGTGCTCCCTAAGCACTCGCTCCTGTACGAGTACTTCACAGTCTACAACGAGCTGACTAAGGTGAAGTATGTGACCGAGGGCATGAGGAAGCCGGCTTTCCTGTCTGGGGAGCAGAAGAAGGCCATCGTGGACCTCCTGTTCAAGACCAACCGGAAGGTCACGGTTAAGCAGCTCAAGGAGGACTACTTCAAGAAGATTGAGTGCTTCGATTCGGTCGAGATCTCTGGCGTTGAGGACCGCTTCAACGCCTCCCTGGGGACCTACCACGATCTCCTGAAGATCATTAAGGATAAGGACTTCCTGGACAACGAGGAGAATGAGGATATCCTCGAGGACATTGTGCTGACACTCACTCTGTTCGAGGACCGGGAGATGATCGAGGAGCGCCTGAAGACTTACGCCCATCTCTTCGATGACAAGGTCATGAAGCAGCTCAAGAGGAGGAGGTACACCGGCTGGGGGAGGCTGAGCAGGAAGCTCATCAACGGCATTCGGGACAAGCAGTCCGGGAAGACGATCCTCGACTTCCTGAAGAGCGATGGCTTCGCGAACCGCAATTTCATGCAGCTGATTCACGATGACAGCCTCACATTCAAGGAGGATATCCAGAAGGCTCAGGTGAGCGGCCAGGGGGACTCGCTGCACGAGCATATCGCGAACCTCGCTGGCTCGCCAGCTATCAAGAAGGGGATTCTGCAGACCGTGAAGGTTGTGGACGAGCTGGTGAAGGTCATGGGCAGGCACAAGCCTGAGAACATCGTCATTGAGATGGCCCGGGAGAATCAGACCACGCAGAAGGGCCAGAAGAACTCACGCGAGAGGATGAAGAGGATCGAGGAGGGCATTAAGGAGCTGGGGTCCCAGATCCTCAAGGAGCACCCGGTGGAGAACACGCAGCTGCAGAATGAGAAGCTCTACCTGTACTACCTCCAGAATGGCCGCGATATGTATGTGGACCAGGAGCTGGATATTAACAGGCTCAGCGATTACGACGTCGATGCCATCGTTCCACAGTCATTCCTGAAGGATGACTCCATTGACAACAAGGTCCTCACCAGGTCGGACAAGAACCGGGGCAAGTCTGATAATGTTCCTTCAGAGGAGGTCGTTAAGAAGATGAAGAACTACTGGCGCCAGCTCCTGAATGCCAAGCTGATCACGCAGCGGAAGTTCGATAACCTCACAAAGGCTGAGAGGGGCGGGCTCTCTGAGCTGGACAAGGCGGGCTTCATCAAGAGGCAGCTGGTCGAGACACGGCAGATCACTAAGCACGTTGCGCAGATTCTCGACTCACGGATGAACACTAAGTACGATGAGAATGACAAGCTGATCCGCGAGGTGAAGGTCATCACCCTGAAGTCAAAGCTCGTCTCCGACTTCAGGAAGGATTTCCAGTTCTACAAGGTTCGGGAGATCAACAATTACCACCATGCCCATGACGCGTACCTGAACGCGGTGGTCGGCACAGCTCTGATCAAGAAGTACCCAAAGCTCGAGAGCGAGTTCGTGTACGGGGACTACAAGGTTTACGATGTGAGGAAGATGATCGCCAAGTCGGAGCAGGAGATTGGCAAGGCTACCGCCAAGTACTTCTTCTACTCTAACATTATGAATTTCTTCAAGACAGAGATCACTCTGGCCAATGGCGAGATCCGGAAGCGCCCCCTCATCGAGACGAACGGCGAGACGGGGGAGATCGTGTGGGACAAGGGCAGGGATTTCGCGACCGTCAGGAAGGTTCTCTCCATGCCACAAGTGAATATCGTCAAGAAGACAGAGGTCCAGACTGGCGGGTTCTCTAAGGAGTCAATTCTGCCTAAGCGGAACAGCGACAAGCTCATCGCCCGCAAGAAGGACTGGGATCCGAAGAAGTACGGCGGGTTCGACAGCCCCACTGTGGCCTACTCGGTCCTGGTTGTGGCGAAGGTTGAGAAGGGCAAGTCCAAGAAGCTCAAGAGCGTGAAGGAGCTGCTGGGGATCACGATTATGGAGCGCTCCAGCTTCGAGAAGAACCCGATCGATTTCCTGGAGGCGAAGGGCTACAAGGAGGTGAAGAAGGACCTGATCATTAAGCTCCCCAAGTACTCACTCTTCGAGCTGGAGAACGGCAGGAAGCGGATGCTGGCTTCCGCTGGCGAGCTGCAGAAGGGGAACGAGCTGGCTCTGCCGTCCAAGTATGTGAACTTCCTCTACCTGGCCTCCCACTACGAGAAGCTCAAGGGCAGCCCCGAGGACAACGAGCAGAAGCAGCTGTTCGTCGAGCAGCACAAGCATTACCTCGACGAGATCATTGAGCAGATTTCCGAGTTCTCCAAGCGCGTGATCCTGGCCGACGCGAATCTGGATAAGGTCCTCTCCGCGTACAACAAGCACCGCGACAAGCCAATCAGGGAGCAGGCTGAGAATATCATTCATCTCTTCACCCTGACGAACCTCGGCGCCCCTGCTGCTTTCAAGTACTTCGACACAACTATCGATCGCAAGAGGTACACAAGCACTAAGGAGGTCCTGGACGCGACCCTCATCCACCAGTCGATTACCGGCCTCTACGAGACGCGCATCGACCTGTCTCAGCTCGGGGGCGACGAATTCTCCGGGAGCGAGACGCCAGGCACCTCCGAGTCGGCCACCCCAGAATCTGCCACAGTGGTGTCCGGCCAAAAGCAGGACCGCCAGGGCGGAGAACGCAGAAGGTCCCAGCTCGATAGGGATCAGTGTGCCTACTGCAAGGAGAAGGGCCACTGGGCCAAAGACTGCCCGAAAAAGCCGCGCGGCCCACGCGGCCCAAGGCCACAAACATCCCTCCTTCCAAAGAAGAAGCGGAAGGTGGAGCTCAGCGGAGGATCTTCCGGAGGATCTAGCGGCTCCGAGACACCAGGAACATCCGAAAGCGCTACACCAGAATCTAGCGGAGGCTCTTCCGGAGGATCTAGGCCTCCTCCCGTCGCGTGCCTCGTACTGAGCCTAGAGGAGGAGTACAGGCTGCATGAGCAAAATCCGAAGCAGCTGCTAGCCCCTGAATGGCTGAGCGCCTTCTCCGAGGTATGGGCGGAGCAGACTGGCATGGGACTGGCCAAGCAGGTGCCTCCTGTGGTGGTAGAGCTGAAGGCGGACGCCAGCCCAGTGAGCGTGAAACAATACCCGATGTCGAGGGAAGCAAAGGAAGGCATTAGACCCCACATCCAGAGACTGCTGCAATTGGGAATCCTGGTACCCTGCCAAAGCCCTTGGAACACGCCACTGCTGCCTGTGCGCAAACCAGGAACTAATGATTACAGACCTGTACAGGATCTAAGGGAAGTTAACAAGCGGGTGCAGGACATACATCCTACGGTCCCAAACCCCTACAACTTGCTGAGCTCTCTGCCCCCCGAGCGCACATGGTACACTGTGCTCGACTTAAAAGATGCGTTCTTCTGCCTTCGCCTCCACCCGAACTCACAGCCACTGTTCGCGTTCGAGTGGCGCGACCCTGAGGGCGGCCACACCGGGCAACTGACGTGGACACGGCTGCCACAGGGTTTCAAGAACAGCCCTACTCTATTTAACGAGGCCCTGCACCGTGATCTGGCCCCCTTCCGGGCGCAGAACCCTCAAATCAGTCTACTGCAGTATGCGGATGACCTGCTGCTAGCCGCTTCTACTCAGGAGCTCTGCCGGGAGGGCACAAAGCGGCTGTTAAATGAGCTGGGCGAGCTGGGATACCGCGTGTCTGCCAAGAAGGCACAATTGTGTCGGACAGAGGTGACATATCTCGGCTACACATTAAGAGAAGGTAAGAGATGGCTCACTGAAGCACGTAAGAAGACTGTCATGCAGATTCCTACCCCTACCACGCCCCGTCAGGTGCGGGAGTTCCTCGGCAAGGCCGGCTTCTGTAGACTGTTCATTCCTGGATTTGCCACCCTGGCCGCCCCACTCTACCTGCTGACGAAGCCAAAGGTTCCTTTCACCTGGACTGAGGAACATCAGCGGGCTTTCGAAGACATCAAAGCCGCCCTGCTGGCGGCTCCAGCTCTGGCCCTCCCTGACCTCACCAAGCCCTTCACACTCTATGTGGACGAGAGAGCTGGAGTGGCCAGAGGTGTGCTGACACAGGCACTGGGGCCGTGGAAGAGACCCGTGGCGTACCTATCTAAAAAGCTCGACCCCGTGGCCTCTGGGTGGCCGAGCTGTCTAAAGGCCATCGCTGCTGTAGCTCTACTGGTCAAAGATGCTGACAAGCTGACCCTGGGCCAGCATGTCACCGTCATCGCCCCGCACGCCTTAGAATCCATAGTCCGGCAACCACCAGATCGGTGGATGACAAACGCTAGGATGACGCATTATCAGAGCTTGCTGTTAAACGAAAGAGTGACATTTGCGCCCCCTGCCATCCTTAACCCAGCGACGCTACTGCCGGAGATCCACAACTCAACCCCCATCCACCAGTGCGTCGACATTCTGGCTGAGGAGACCGGCACGCGCAAGGACCTCACGGACAGACCATGGCCTGGTGTGCCGGCATGGTACACCGACGGCAGCTCCTTTGTCGTGGAGGGAAAGCGACGCGCAGGCGCGGCTGTGGTGGACGGCAAGCAAGTCATCTGGGCATCCTCCCTGCCAGAAGGCACCAGCGCTCAGAAAGCGGAGTTGGTGGCCTTGACCCAGGCCCTCAGATTAGCTGAAGGGAAGGCTATTAATATCTACACCGATTCTAGATACGCCTTCGCCACGGCGCACATCCACGGCGCGATCTACAAGCAGCGGGGCCTGCTGACCAGTGCCGGGCGAGACATCAAGAATAAGGAAGAGATTCTTGCCCTGCTGGAGGCCGTCCACCTCCCCAAAAAGGTGGCCATTATCCACTGCCCCGGCCACCAGAAGGGAGAGGATCCAATTACAAAGGGGAACCAGATGGCCGACCTCGTGGCGAAGCAGGTCGCTCAGCAGGTTACCATCCTGGCAGAAAAATCGCAGACCCCTGTGAAGACACCTGTGACAGATGATTCTAACTATAGCGGCGGCAGCAAAAGAACGGCGGACGGCTCTGAGAAGCGCACCGCTGATAGCCAGCATTCAACTCCTCCGAAAACAAAGAGGAAAGTTGAGTTCGAACCGAAGAAGAAAAGGAAGGTGTGAGAGCTCAGAGCTTTCGTTCGTATCATCGGTTTCGACAACGTTCGTCAAGTTCAATGCATCAGTTTCATTGCGCACACACCAGAATCCTACTGAGTTTGAGTATTATGGCATTGGGAAAACTGTTTTTCTTGTACCATTTGTTGTGCTTGTAATTTACTGTGTTTTTTATTCGGTTTTCGCTATCGAACTGTGAAATGGAAATGGATGGAGAAGAGTTAATGAATGATATGGTCCTTTTGTTCATTCTCAAATTAATATTATTTGTTTTTTCTCTTATTTGTTGTGTGTTGAATTTGAAATTATAAGAGATATGCAAACATTTTGTTTTGAGTAAAAATGTGTCAAATCGTGGCCTCTAATGACCGAAGTTAATATGAGGAGTAAAACACTTGTAGTTGTACCATTATGCTTATTCACTAGGCAACAAATATATTTTCAGACCTAGAAAAGCTGCAAATGTTACTGAATACAAGTATGTCCTCTTGTGTTTTAGACATTTATGAACTTTCCTTTATGTAATTTTCCAGAATCCTTGTCAGATTCTAATCATTGCTTTATAATTATAGTTATACTCATGGATTTGTAGTTGAGTATGAAAATATTTTTTAATGCATTTTATGACTTGCCAATTGATTGACAACGAATTCGTAATCATGTCATAGCTGTTTCCTGTGTGAAATTGTTATCCGCTCACAATTCCACACAACATACGAGCCGGAAGCATAAAGTGTAAAGCCTGGGGTGCCTAATGAGTGAGCTAACTCACATTAATTGCGTTGCGCTCACTGCCCGCTTTCCAGTCGGGAAACCTGTCGTGCCAGCTGCATTAATGAATCGGCCAACGCGCGGGGAGAGGCGGTTTGCGTATTGGCTAGAGCAGCTTGCCAACATGGTGGAGCACGACACTCTCGTCTACTCCAAGAATATCAAAGATACAGTCTCAGAAGACCAAAGGGCTATTGAGACTTTTCAACAAAGGGTAATATCGGGAAACCTCCTCGGATTCCATTGCCCAGCTATCTGTCACTTCATCAAAAGGACAGTAGAAAAGGAAGGTGGCACCTACAAATGCCATCATTGCGATAAAGGAAAGGCTATCGTTCAAGATGCCTCTGCCGACAGTGGTCCCAAAGATGGACCCCCACCCACGAGGAGCATCGTGGAAAAAGAAGACGTTCCAACCACGTCTTCAAAGCAAGTGGATTGATGTGAACATGGTGGAGCACGACACTCTCGTCTACTCCAAGAATATCAAAGATACAGTCTCAGAAGACCAAAGGGCTATTGAGACTTTTCAACAAAGGGTAATATCGGGAAACCTCCTCGGATTCCATTGCCCAGCTATCTGTCACTTCATCAAAAGGACAGTAGAAAAGGAAGGTGGCACCTACAAATGCCATCATTGCGATAAAGGAAAGGCTATCGTTCAAGATGCCTCTGCCGACAGTGGTCCCAAAGATGGACCCCCACCCACGAGGAGCATCGTGGAAAAAGAAGACGTTCCAACCACGTCTTCAAAGCAAGTGGATTGATGTGATATCTCCACTGACGTAAGGGATGACGCACAATCCCACTATCCTTCGCAAGACCCTTCCTCTATATAAGGAAGTTCATTTCATTTGGAGAGGACACGCTGAAATCACCAGTCTCTCTCTACAAATCTATCTCTCTCGAGCTTTCGCAGATCCGGGGGGCAATGAGATATGAAAAAGCCTGAACTCACCGCGACGTCTGTCGAGAAGTTTCTGATCGAAAAGTTCGACAGCGTCTCCGACCTGATGCAGCTCTCGGAGGGCGAAGAATCTCGTGCTTTCAGCTTCGATGTAGGAGGGCGTGGATATGTCCTGCGGGTAAATAGCTGCGCCGATGGTTTCTACAAAGATCGTTATGTTTATCGGCACTTTGCATCGGCCGCGCTCCCGATTCCGGAAGTGCTTGACATTGGGGAGTTTAGCGAGAGCCTGACCTATTGCATCTCCCGCCGTTCACAGGGTGTCACGTTGCAAGACCTGCCTGAAACCGAACTGCCCGCTGTTCTACAACCGGTCGCGGAGGCTATGGATGCGATCGCTGCGGCCGATCTTAGCCAGACGAGCGGGTTCGGCCCATTCGGACCGCAAGGAATCGGTCAATACACTACATGGCGTGATTTCATATGCGCGATTGCTGATCCCCATGTGTATCACTGGCAAACTGTGATGGACGACACCGTCAGTGCGTCCGTCGCGCAGGCTCTCGATGAGCTGATGCTTTGGGCCGAGGACTGCCCCGAAGTCCGGCACCTCGTGCACGCGGATTTCGGCTCCAACAATGTCCTGACGGACAATGGCCGCATAACAGCGGTCATTGACTGGAGCGAGGCGATGTTCGGGGATTCCCAATACGAGGTCGCCAACATCTTCTTCTGGAGGCCGTGGTTGGCTTGTATGGAGCAGCAGACGCGCTACTTCGAGCGGAGGCATCCGGAGCTTGCAGGATCGCCACGACTCCGGGCGTATATGCTCCGCATTGGTCTTGACCAACTCTATCAGAGCTTGGTTGACGGCAATTTCGATGATGCAGCTTGGGCGCAGGGTCGATGCGACGCAATCGTCCGATCCGGAGCCGGGACTGTCGGGCGTACACAAATCGCCCGCAGAAGCGCGGCCGTCTGGACCGATGGCTGTGTAGAAGTACTCGCCGATAGTGGAAACCGACGCCCCAGCACTCGTCCGAGGGCAAAGAAATAGAGTAGATGCCGACCGGGATCTGTCGATCGACAAGCTCGAGTTTCTCCATAATAATGTGTGAGTAGTTCCCAGATAAGGGAATTAGGGTTCCTATAGGGTTTCGCTCATGTGTTGAGCATATAAGAAACCCTTAGTATGTATTTGTATTTGTAAAATACTTCTATCAATAAAATTTCTAATTCCTAAAACCAAAATCCAGTACTAAAATCCAGATCCCCCGAATTAATTCGGCGTTAATTCAGTACATTAAAAACGTCCGCAATGTGTTATTAAGTTGTCTAAGCGTCAATTTGTTTACACCACAATATATCCTGCCA

**Sequence 76 Plasmids sequence of pH-35C-RERV-m5-OsCDC48**

(RB T-DNA repeat-35C promoter-tRNAGly RS-pegRNA-Ubi promoter-NLSc-myc-NLSsv40-nCas9(H840A/ R221K/N394K)-XTEN-NC-NLS-32aa Linker- RERV-RT-m5-NLSvbp-HygR cassette-E9-terminator-CaMV poly(A) signal-LB T-DNA repeat)

GTTTACCCGCCAATATATCCTGTCAAACACTGATAGTTTAAACTGAAGGCGGGAAACGACAATCTGATCCAAGCTCAAGCTGCTCTAGCATTCGCCATTCAGGCTGCGCAACTGTTGGGAAGGGCGATCGGTGCGGGCCTCTTCGCTATTACGCCAGCTGGCGAAAGGGGGATGTGCTGCAAGGCGATTAAGTTGGGTAACGCCAGGGTTTTCCCAGTCACGACGTTGTAAAACGACGGCCAGTGAATTCATGGAGTCAAAGATTCAAATAGAGGACCTAACAGAACTCGCCGTAAAGACTGGCGAACAGTTCATACAGAGTCTCTTACGACTCAATGACAAGAAGAAAATCTTCGTCAACATGGTGGAGCACGACACACTTGTCTACTCCAAAAATATCAAAGATACAGTCTCAGAAGACCAAAGGGCAATTGAGACTTTTCAACAAAGGGTAATATCCGGAAACCTCCTCGGATTCCATTGCCCAGCTATCTGTCACTTTATTGTGAAGATAGTGGAAAAGGAAGGTGGCTCCTACAAATGCCATCATTGCGATAAAGGAAAGGCCATCGTTGAAGATGCCTCTGCCGACAGTGGTCCCAAAGATGGACCCCCACCCACGAGGAGCATCGTGGAAAAAGAAGACGTTCCAACCACGTCTTCAAAGCAAGTGGATTGATGTGATTGGCAGACATACTGTCCCACAAATGAAGATGGAATCTGTAAAAGAAAACGCGTGAAATAATGCGTCTGACAAAGGTTAGGTCGGCTGCCTTTAATCAATACCAAAGTGGTCCCTACCACGATGGAAAAACTGTGCAGTCGGTTTGGCTTTTTCTGACGAACAAATAAGATTCGTGGCCGACAGGTGGGGGTCCACCATGTGAAGGCATCTTCAGACTCCAATAATGGAGCAATGACGTAAGGGCTTACGAAATAAGTAAGGGTAGTTTGGGAAATGTCCACTCACCCGTCAGTCTATAAATACTTAGCCCCTCCCTCATTGTTAAGGGAGCAAAATCTCAGAGAGATAGTCCTAGAGAGAGAAAGAGAGCAAGTAGCCTAGAAGTAGTCAAGGCGGCGAAGTATTCAGGCACGTGGCCAGGAAGAAGAAAAGCCAAGACGACGAAAACAGGTAAGAGCTAAGCATCTAGATAAGTTGAAAACAATCTTCAAAAGTCCCACATCGCTTAGATAAGAAAACGAAGCTGAGTTTATATACAGCTAGAGTCGAAGTAGTGATTGAACAAAGCACCAGTGGTCTAGTGGTAGAATAGTACCCTGCCACGGTACAGACCCGGGTTCGATTCCCGGCTGGTGCAGAAGGGGTCAGCGGCGGCGCGTTTAAGAGCTATGCTGGAAACAGCATAGCAAGTTTAAATAAGGCTAGTCCGTTATCAACTTGAAAAAGTGGCACCGAGTCGGTGCCAGCGTCTGGCGCAGGCGCCGCCGCTCGCGGTTCTATCTAGTTACGCGTTAAACCAACTAGAATTTTTTTGGCCGGCATGGTCCCAGCCTCCTCGCTGGCGCCGGCTGGGCAACATGCTTCGGCATGGCGAATGGGACTTTTTTTTGATATCTCCGGGGCTAATTGAATATGAAGATGAAGATGAAATATTTGGTGTGTCAAATAAAAAGCTGGTGTGCTTAAGTTTGTGTTTTTTTCTTGGCTTGTTGTGTTATGAATTTGTGGCTTTTTCTAATATTAAATGAATGTAAGATCTCATTATAATGAATAAACAAATGTTTCTATAATCCATTGTGAATGTTTTGTTGGATCTCTTCTGCAGCATATAACTACTGTATGTGCTATGGTATGGACTATGGAATATGATTAAAGATAAGAAGCTTGGCGTAATCTGCATGCCTGCAGTGCAGCGTGACCCGGTCGTGCCCCTCTCTAGAGATAATGAGCATTGCATGTCTAAGTTATAAAAAATTACCACATATTTTTTTTGTCACACTTGTTTGAAGTGCAGTTTATCTATCTTTATACATATATTTAAACTTTACTCTACGAATAATATAATCTATAGTACTACAATAATATCAGTGTTTTAGAGAATCATATAAATGAACAGTTAGACATGGTCTAAAGGACAATTGAGTATTTTGACAACAGGACTCTACAGTTTTATCTTTTTAGTGTGCATGTGTTCTCCTTTTTTTTTGCAAATAGCTTCACCTATATAATACTTCATCCATTTTATTAGTACATCCATTTAGGGTTTAGGGTTAATGGTTTTTATAGACTAATTTTTTTAGTACATCTATTTTATTCTATTTTAGCCTCTAAATTAAGAAAACTAAAACTCTATTTTAGTTTTTTTATTTAATAATTTAGATATAAAATAGAATAAAATAAAGTGACTAAAAATTAAACAAATACCCTTTAAGAAATTAAAAAAACTAAGGAAACATTTTTCTTGTTTCGAGTAGATAATGCCAGCCTGTTAAACGCCGTCGACGAGTCTAACGGACACCAACCAGCGAACCAGCAGCGTCGCGTCGGGCCAAGCGAAGCAGACGGCACGGCATCTCTGTCGCTGCCTCTGGACCCCTCTCGAGAGTTCCGCTCCACCGTTGGACTTGCTCCGCTGTCGGCATCCAGAAATTGCGTGGCGGAGCGGCAGACGTGAGCCGGCACGGCAGGCGGCCTCCTCCTCCTCTCACGGCACCGGCAGCTACGGGGGATTCCTTTCCCACCGCTCCTTCGCTTTCCCTTCCTCGCCCGCCGTAATAAATAGACACCCCCTCCACACCCTCTTTCCCCAACCTCGTGTTGTTCGGAGCGCACACACACACAACCAGATCTCCCCCAAATCCACCCGTCGGCACCTCCGCTTCAAGGTACGCCGCTCGTCCTCCCCCCCCCCCCCTCTCTACCTTCTCTAGATCGGCGTTCCGGTCCATGGTTAGGGCCCGGTAGTTCTACTTCTGTTCATGTTTGTGTTAGATCCGTGTTTGTGTTAGATCCGTGCTGCTAGCGTTCGTACACGGATGCGACCTGTACGTCAGACACGTTCTGATTGCTAACTTGCCAGTGTTTCTCTTTGGGGAATCCTGGGATGGCTCTAGCCGTTCCGCAGACGGGATCGATTTCATGATTTTTTTTGTTTCGTTGCATAGGGTTTGGTTTGCCCTTTTCCTTTATTTCAATATATGCCGTGCACTTGTTTGTCGGGTCATCTTTTCATGCTTTTTTTTGTCTTGGTTGTGATGATGTGGTCTGGTTGGGCGGTCGTTCTAGATCGGAGTAGAATTCTGTTTCAAACTACCTGGTGGATTTATTAATTTTGGATCTGTATGTGTGTGCCATACATATTCATAGTTACGAATTGAAGATGATGGATGGAAATATCGATCTAGGATAGGTATACATGTTGATGCGGGTTTTACTGATGCATATACAGAGATGCTTTTTGTTCGCTTGGTTGTGATGATGTGGTGTGGTTGGGCGGTCGTTCATTCGTTCTAGATCGGAGTAGAATACTGTTTCAAACTACCTGGTGTATTTATTAATTTTGGAACTGTATGTGTGTGTCATACATCTTCATAGTTACGAGTTTAAGATGGATGGAAATATCGATCTAGGATAGGTATACATGTTGATGTGGGTTTTACTGATGCATATACATGATGGCATATGCAGCATCTATTCATATGCTCTAACCTTGAGTACCTATCTATTATAATAAACAAGTATGTTTTATAATTATTTTGATCTTGATATACTTGGATGATGGCATATGCAGCAGCTATATGTGGATTTTTTTAGCCCTGCCTTCATACGCTATTTATTTGCTTGGTACTGTTTCTTTTGTCGATGCTCACCCTGTTGTTTGGTGTTACTTCTGCAGCCCTAGGACTGGTTGTACAATGCCGGCGGCCAAGAGAGTCAAGCTCGACGGCGGGAAGCGCACAGCTGATGGTTCTGAATTCGAGTCCCCTAAGAAAAAGAGAAAAGTGGACAAGAAGTACTCGATCGGCCTCGATATTGGGACTAACTCTGTTGGCTGGGCCGTGATCACCGACGAGTACAAGGTGCCCTCAAAGAAGTTCAAGGTCCTGGGCAACACCGATCGGCATTCCATCAAGAAGAATCTCATTGGCGCTCTCCTGTTCGACAGCGGCGAGACGGCTGAGGCTACGCGGCTCAAGCGCACCGCCCGCAGGCGGTACACGCGCAGGAAGAATCGCATCTGCTACCTGCAGGAGATTTTCTCCAACGAGATGGCGAAGGTTGACGATTCTTTCTTCCACAGGCTGGAGGAGTCATTCCTCGTGGAGGAGGATAAGAAGCACGAGCGGCATCCAATCTTCGGCAACATTGTCGACGAGGTTGCCTACCACGAGAAGTACCCTACGATCTACCATCTGCGGAAGAAGCTCGTGGACTCCACAGATAAGGCGGACCTCCGCCTGATCTACCTCGCTCTGGCCCACATGATTAAGTTCAGGGGCCATTTCCTGATCGAGGGGGATCTCAACCCGGACAATAGCGATGTTGACAAGCTGTTCATCCAGCTCGTGCAGACGTACAACCAGCTCTTCGAGGAGAACCCCATTAATGCGTCAGGCGTCGACGCGAAGGCTATCCTGTCCGCTAGGCTCTCGAAGTCTCGGAAGCTCGAGAACCTGATCGCCCAGCTGCCGGGCGAGAAGAAGAACGGCCTGTTCGGGAATCTCATTGCGCTCAGCCTGGGGCTCACGCCCAACTTCAAGTCGAATTTCGATCTCGCTGAGGACGCCAAGCTGCAGCTCTCCAAGGACACATACGACGATGACCTGGATAACCTCCTGGCCCAGATCGGCGATCAGTACGCGGACCTGTTCCTCGCTGCCAAGAATCTGTCGGACGCCATCCTCCTGTCTGATATTCTCAGGGTGAACACCGAGATTACGAAGGCTCCGCTCTCAGCCTCCATGATCAAGCGCTACGACGAGCACCATCAGGATCTGACCCTCCTGAAGGCGCTGGTCAGGCAGCAGCTCCCCGAGAAGTACAAGGAGATCTTCTTCGATCAGTCGAAGAACGGCTACGCTGGGTACATTGACGGCGGGGCCTCTCAGGAGGAGTTCTACAAGTTCATCAAGCCGATTCTGGAGAAGATGGACGGCACGGAGGAGCTGCTGGTGAAGCTCAAGCGCGAGGACCTCCTGAGGAAGCAGCGGACATTCGATAACGGCAGCATCCCACACCAGATTCATCTCGGGGAGCTGCACGCTATCCTGAGGAGGCAGGAGGACTTCTACCCTTTCCTCAAGGATAACCGCGAGAAGATCGAGAAGATTCTGACTTTCAGGATCCCGTACTACGTCGGCCCACTCGCTAGGGGCAACTCCCGCTTCGCTTGGATGACCCGCAAGTCAGAGGAGACGATCACGCCGTGGAACTTCGAGGAGGTGGTCGACAAGGGCGCTAGCGCTCAGTCGTTCATCGAGAGGATGACGAATTTCGACAAGAACCTGCCAAATGAGAAGGTGCTCCCTAAGCACTCGCTCCTGTACGAGTACTTCACAGTCTACAACGAGCTGACTAAGGTGAAGTATGTGACCGAGGGCATGAGGAAGCCGGCTTTCCTGTCTGGGGAGCAGAAGAAGGCCATCGTGGACCTCCTGTTCAAGACCAACCGGAAGGTCACGGTTAAGCAGCTCAAGGAGGACTACTTCAAGAAGATTGAGTGCTTCGATTCGGTCGAGATCTCTGGCGTTGAGGACCGCTTCAACGCCTCCCTGGGGACCTACCACGATCTCCTGAAGATCATTAAGGATAAGGACTTCCTGGACAACGAGGAGAATGAGGATATCCTCGAGGACATTGTGCTGACACTCACTCTGTTCGAGGACCGGGAGATGATCGAGGAGCGCCTGAAGACTTACGCCCATCTCTTCGATGACAAGGTCATGAAGCAGCTCAAGAGGAGGAGGTACACCGGCTGGGGGAGGCTGAGCAGGAAGCTCATCAACGGCATTCGGGACAAGCAGTCCGGGAAGACGATCCTCGACTTCCTGAAGAGCGATGGCTTCGCGAACCGCAATTTCATGCAGCTGATTCACGATGACAGCCTCACATTCAAGGAGGATATCCAGAAGGCTCAGGTGAGCGGCCAGGGGGACTCGCTGCACGAGCATATCGCGAACCTCGCTGGCTCGCCAGCTATCAAGAAGGGGATTCTGCAGACCGTGAAGGTTGTGGACGAGCTGGTGAAGGTCATGGGCAGGCACAAGCCTGAGAACATCGTCATTGAGATGGCCCGGGAGAATCAGACCACGCAGAAGGGCCAGAAGAACTCACGCGAGAGGATGAAGAGGATCGAGGAGGGCATTAAGGAGCTGGGGTCCCAGATCCTCAAGGAGCACCCGGTGGAGAACACGCAGCTGCAGAATGAGAAGCTCTACCTGTACTACCTCCAGAATGGCCGCGATATGTATGTGGACCAGGAGCTGGATATTAACAGGCTCAGCGATTACGACGTCGATGCCATCGTTCCACAGTCATTCCTGAAGGATGACTCCATTGACAACAAGGTCCTCACCAGGTCGGACAAGAACCGGGGCAAGTCTGATAATGTTCCTTCAGAGGAGGTCGTTAAGAAGATGAAGAACTACTGGCGCCAGCTCCTGAATGCCAAGCTGATCACGCAGCGGAAGTTCGATAACCTCACAAAGGCTGAGAGGGGCGGGCTCTCTGAGCTGGACAAGGCGGGCTTCATCAAGAGGCAGCTGGTCGAGACACGGCAGATCACTAAGCACGTTGCGCAGATTCTCGACTCACGGATGAACACTAAGTACGATGAGAATGACAAGCTGATCCGCGAGGTGAAGGTCATCACCCTGAAGTCAAAGCTCGTCTCCGACTTCAGGAAGGATTTCCAGTTCTACAAGGTTCGGGAGATCAACAATTACCACCATGCCCATGACGCGTACCTGAACGCGGTGGTCGGCACAGCTCTGATCAAGAAGTACCCAAAGCTCGAGAGCGAGTTCGTGTACGGGGACTACAAGGTTTACGATGTGAGGAAGATGATCGCCAAGTCGGAGCAGGAGATTGGCAAGGCTACCGCCAAGTACTTCTTCTACTCTAACATTATGAATTTCTTCAAGACAGAGATCACTCTGGCCAATGGCGAGATCCGGAAGCGCCCCCTCATCGAGACGAACGGCGAGACGGGGGAGATCGTGTGGGACAAGGGCAGGGATTTCGCGACCGTCAGGAAGGTTCTCTCCATGCCACAAGTGAATATCGTCAAGAAGACAGAGGTCCAGACTGGCGGGTTCTCTAAGGAGTCAATTCTGCCTAAGCGGAACAGCGACAAGCTCATCGCCCGCAAGAAGGACTGGGATCCGAAGAAGTACGGCGGGTTCGACAGCCCCACTGTGGCCTACTCGGTCCTGGTTGTGGCGAAGGTTGAGAAGGGCAAGTCCAAGAAGCTCAAGAGCGTGAAGGAGCTGCTGGGGATCACGATTATGGAGCGCTCCAGCTTCGAGAAGAACCCGATCGATTTCCTGGAGGCGAAGGGCTACAAGGAGGTGAAGAAGGACCTGATCATTAAGCTCCCCAAGTACTCACTCTTCGAGCTGGAGAACGGCAGGAAGCGGATGCTGGCTTCCGCTGGCGAGCTGCAGAAGGGGAACGAGCTGGCTCTGCCGTCCAAGTATGTGAACTTCCTCTACCTGGCCTCCCACTACGAGAAGCTCAAGGGCAGCCCCGAGGACAACGAGCAGAAGCAGCTGTTCGTCGAGCAGCACAAGCATTACCTCGACGAGATCATTGAGCAGATTTCCGAGTTCTCCAAGCGCGTGATCCTGGCCGACGCGAATCTGGATAAGGTCCTCTCCGCGTACAACAAGCACCGCGACAAGCCAATCAGGGAGCAGGCTGAGAATATCATTCATCTCTTCACCCTGACGAACCTCGGCGCCCCTGCTGCTTTCAAGTACTTCGACACAACTATCGATCGCAAGAGGTACACAAGCACTAAGGAGGTCCTGGACGCGACCCTCATCCACCAGTCGATTACCGGCCTCTACGAGACGCGCATCGACCTGTCTCAGCTCGGGGGCGACGAATTCTCCGGGAGCGAGACGCCAGGCACCTCCGAGTCGGCCACCCCAGAATCTGCCACAGTGGTGTCCGGCCAAAAGCAGGACCGCCAGGGCGGAGAACGCAGAAGGTCCCAGCTCGATAGGGATCAGTGTGCCTACTGCAAGGAGAAGGGCCACTGGGCCAAAGACTGCCCGAAAAAGCCGCGCGGCCCACGCGGCCCAAGGCCACAAACATCCCTCCTTCCAAAGAAGAAGCGGAAGGTGGAGCTCAGCGGAGGATCTTCCGGAGGATCTAGCGGCTCCGAGACACCAGGAACATCCGAAAGCGCTACACCAGAATCTAGCGGAGGCTCTTCCGGAGGATCTAGGCCTCCTCCCGTCGCGTGCCTCGTACTGAGCCTAGAGGAGGAGTACAGGCTGCATGAGCAAAATCCGAAGCAGCTGCTAGCCCCTGAATGGCTGAGCGCCTTCTCCGAGGTATGGGCGGAGCAGACTGGCATGGGACTGGCCAAGCAGGTGCCTCCTGTGGTGGTAGAGCTGAAGGCGGACGCCAGCCCAGTGAGCGTGAAACAATACCCGATGTCGAGGGAAGCAAAGGAAGGCATTAGACCCCACATCCAGAGACTGCTGCAATTGGGAATCCTGGTACCCTGCCAAAGCCCTTGGAACACGCCACTGCTGCCTGTGCGCAAACCAGGAACTAATGATTACAGACCTGTACAGGATCTAAGGGAAGTTAACAAGCGGGTGCAGGACATACATCCTACGGTCCCAAACCCCTACAACTTGCTGAGCTCTCTGCCCCCCGAGCGCACATGGTACACTGTGCTCGACTTAAAAGATGCGTTCTTCTGCCTTCGCCTCCACCCGAACTCACAGCCACTGTTCGCGTTCGAGTGGCGCGACCCTGAGGGCGGCCACACCGGGCAACTGACGTGGACACGGCTGCCACAGGGTTTCAAGAACAGCCCTACTCTATTTAACGAGGCCCTGCACCGTGATCTGGCCCCCTTCCGGGCGCAGAACCCTCAAATCAGTCTACTGCAGTATGCGGATGACCTGCTGCTAGCCGCTTCTACTCAGGAGCTCTGCCGGGAGGGCACAAAGCGGCTGTTAAATGAGCTGGGCGAGCTGGGATACCGCGTGTCTGCCAAGAAGGCACAATTGTGTCGGACAGAGGTGACATATCTCGGCTACACATTAAGAGAAGGTAAGAGATGGCTCACTGAAGCACGTAAGAAGACTGTCATGCAGATTCCTACCCCTACCACGCCCCGTCAGGTGCGGGAGTTCCTCGGCAAGGCCGGCTTCTGTAGACTGTTCATTCCTGGATTTGCCACCCTGGCCGCCCCACTCTACCTGCTGACGAAGCCAAAGGTTCCTTTCACCTGGACTGAGGAACATCAGCGGGCTTTCGAAGACATCAAAGCCGCCCTGCTGGCGGCTCCAGCTCTGGCCCTCCCTGACCTCACCAAGCCCTTCACACTCTATGTGGACGAGAGAGCTGGAGTGGCCAGAGGTGTGCTGACACAGGCACTGGGGCCGTGGAAGAGACCCGTGGCGTACCTATCTAAAAAGCTCGACCCCGTGGCCTCTGGGTGGCCGAGCTGTCTAAAGGCCATCGCTGCTGTAGCTCTACTGGTCAAAGATGCTGACAAGCTGACCCTGGGCCAGCATGTCACCGTCATCGCCCCGCACGCCTTAGAATCCATAGTCCGGCAACCACCAGATCGGTGGATGACAAACGCTAGGATGACGCATTATCAGAGCTTGCTGTTAAACGAAAGAGTGACATTTGCGCCCCCTGCCATCCTTAACCCAGCGACGCTACTGCCGGAGATCCACAACTCAACCCCCATCCACCAGTGCGTCGACATTCTGGCTGAGGAGACCGGCACGCGCAAGGACCTCACGGACAGACCATGGCCTGGTGTGCCGGCATGGTACACCGACGGCAGCTCCTTTGTCGTGGAGGGAAAGCGACGCGCAGGCGCGGCTGTGGTGGACGGCAAGCAAGTCATCTGGGCATCCTCCCTGCCAGAAGGCACCAGCGCTCAGAAAGCGGAGTTGGTGGCCTTGACCCAGGCCCTCAGATTAGCTGAAGGGAAGGCTATTAATATCTACACCGATTCTAGATACGCCTTCGCCACGGCGCACATCCACGGCGCGATCTACAAGCAGCGGGGCCTGCTGACCAGTGCCGGGCGAGACATCAAGAATAAGGAAGAGATTCTTGCCCTGCTGGAGGCCGTCCACCTCCCCAAAAAGGTGGCCATTATCCACTGCCCCGGCCACCAGAAGGGAGAGGATCCAATTACAAAGGGGAACCAGATGGCCGACCTCGTGGCGAAGCAGGTCGCTCAGCAGGTTACCATCCTGGCAGAAAAATCGCAGACCCCTGTGAAGACACCTGTGACAGATGATTCTAACTATAGCGGCGGCAGCAAAAGAACGGCGGACGGCTCTGAGAAGCGCACCGCTGATAGCCAGCATTCAACTCCTCCGAAAACAAAGAGGAAAGTTGAGTTCGAACCGAAGAAGAAAAGGAAGGTGTGAGAGCTCAGAGCTTTCGTTCGTATCATCGGTTTCGACAACGTTCGTCAAGTTCAATGCATCAGTTTCATTGCGCACACACCAGAATCCTACTGAGTTTGAGTATTATGGCATTGGGAAAACTGTTTTTCTTGTACCATTTGTTGTGCTTGTAATTTACTGTGTTTTTTATTCGGTTTTCGCTATCGAACTGTGAAATGGAAATGGATGGAGAAGAGTTAATGAATGATATGGTCCTTTTGTTCATTCTCAAATTAATATTATTTGTTTTTTCTCTTATTTGTTGTGTGTTGAATTTGAAATTATAAGAGATATGCAAACATTTTGTTTTGAGTAAAAATGTGTCAAATCGTGGCCTCTAATGACCGAAGTTAATATGAGGAGTAAAACACTTGTAGTTGTACCATTATGCTTATTCACTAGGCAACAAATATATTTTCAGACCTAGAAAAGCTGCAAATGTTACTGAATACAAGTATGTCCTCTTGTGTTTTAGACATTTATGAACTTTCCTTTATGTAATTTTCCAGAATCCTTGTCAGATTCTAATCATTGCTTTATAATTATAGTTATACTCATGGATTTGTAGTTGAGTATGAAAATATTTTTTAATGCATTTTATGACTTGCCAATTGATTGACAACGAATTCGTAATCATGTCATAGCTGTTTCCTGTGTGAAATTGTTATCCGCTCACAATTCCACACAACATACGAGCCGGAAGCATAAAGTGTAAAGCCTGGGGTGCCTAATGAGTGAGCTAACTCACATTAATTGCGTTGCGCTCACTGCCCGCTTTCCAGTCGGGAAACCTGTCGTGCCAGCTGCATTAATGAATCGGCCAACGCGCGGGGAGAGGCGGTTTGCGTATTGGCTAGAGCAGCTTGCCAACATGGTGGAGCACGACACTCTCGTCTACTCCAAGAATATCAAAGATACAGTCTCAGAAGACCAAAGGGCTATTGAGACTTTTCAACAAAGGGTAATATCGGGAAACCTCCTCGGATTCCATTGCCCAGCTATCTGTCACTTCATCAAAAGGACAGTAGAAAAGGAAGGTGGCACCTACAAATGCCATCATTGCGATAAAGGAAAGGCTATCGTTCAAGATGCCTCTGCCGACAGTGGTCCCAAAGATGGACCCCCACCCACGAGGAGCATCGTGGAAAAAGAAGACGTTCCAACCACGTCTTCAAAGCAAGTGGATTGATGTGAACATGGTGGAGCACGACACTCTCGTCTACTCCAAGAATATCAAAGATACAGTCTCAGAAGACCAAAGGGCTATTGAGACTTTTCAACAAAGGGTAATATCGGGAAACCTCCTCGGATTCCATTGCCCAGCTATCTGTCACTTCATCAAAAGGACAGTAGAAAAGGAAGGTGGCACCTACAAATGCCATCATTGCGATAAAGGAAAGGCTATCGTTCAAGATGCCTCTGCCGACAGTGGTCCCAAAGATGGACCCCCACCCACGAGGAGCATCGTGGAAAAAGAAGACGTTCCAACCACGTCTTCAAAGCAAGTGGATTGATGTGATATCTCCACTGACGTAAGGGATGACGCACAATCCCACTATCCTTCGCAAGACCCTTCCTCTATATAAGGAAGTTCATTTCATTTGGAGAGGACACGCTGAAATCACCAGTCTCTCTCTACAAATCTATCTCTCTCGAGCTTTCGCAGATCCGGGGGGCAATGAGATATGAAAAAGCCTGAACTCACCGCGACGTCTGTCGAGAAGTTTCTGATCGAAAAGTTCGACAGCGTCTCCGACCTGATGCAGCTCTCGGAGGGCGAAGAATCTCGTGCTTTCAGCTTCGATGTAGGAGGGCGTGGATATGTCCTGCGGGTAAATAGCTGCGCCGATGGTTTCTACAAAGATCGTTATGTTTATCGGCACTTTGCATCGGCCGCGCTCCCGATTCCGGAAGTGCTTGACATTGGGGAGTTTAGCGAGAGCCTGACCTATTGCATCTCCCGCCGTTCACAGGGTGTCACGTTGCAAGACCTGCCTGAAACCGAACTGCCCGCTGTTCTACAACCGGTCGCGGAGGCTATGGATGCGATCGCTGCGGCCGATCTTAGCCAGACGAGCGGGTTCGGCCCATTCGGACCGCAAGGAATCGGTCAATACACTACATGGCGTGATTTCATATGCGCGATTGCTGATCCCCATGTGTATCACTGGCAAACTGTGATGGACGACACCGTCAGTGCGTCCGTCGCGCAGGCTCTCGATGAGCTGATGCTTTGGGCCGAGGACTGCCCCGAAGTCCGGCACCTCGTGCACGCGGATTTCGGCTCCAACAATGTCCTGACGGACAATGGCCGCATAACAGCGGTCATTGACTGGAGCGAGGCGATGTTCGGGGATTCCCAATACGAGGTCGCCAACATCTTCTTCTGGAGGCCGTGGTTGGCTTGTATGGAGCAGCAGACGCGCTACTTCGAGCGGAGGCATCCGGAGCTTGCAGGATCGCCACGACTCCGGGCGTATATGCTCCGCATTGGTCTTGACCAACTCTATCAGAGCTTGGTTGACGGCAATTTCGATGATGCAGCTTGGGCGCAGGGTCGATGCGACGCAATCGTCCGATCCGGAGCCGGGACTGTCGGGCGTACACAAATCGCCCGCAGAAGCGCGGCCGTCTGGACCGATGGCTGTGTAGAAGTACTCGCCGATAGTGGAAACCGACGCCCCAGCACTCGTCCGAGGGCAAAGAAATAGAGTAGATGCCGACCGGGATCTGTCGATCGACAAGCTCGAGTTTCTCCATAATAATGTGTGAGTAGTTCCCAGATAAGGGAATTAGGGTTCCTATAGGGTTTCGCTCATGTGTTGAGCATATAAGAAACCCTTAGTATGTATTTGTATTTGTAAAATACTTCTATCAATAAAATTTCTAATTCCTAAAACCAAAATCCAGTACTAAAATCCAGATCCCCCGAATTAATTCGGCGTTAATTCAGTACATTAAAAACGTCCGCAATGTGTTATTAAGTTGTCTAAGCGTCAATTTGTTTACACCACAATATATCCTGCCA

**Sequence 77 Plasmids sequence of pH-35C-M-MLV-OsEPSPS**

(RB T-DNA repeat-35C promoter-tRNAGly RS-pegRNA-Ubi promoter-NLSc-myc-NLSsv40-nCas9(H840A/ R221K/N394K)-XTEN-NC-NLS-32aa Linker-M-MLV-RT-NLSvbp-HygR cassette-E9-terminator-CaMV poly(A) signal-LB T-DNA repeat)

GTTTACCCGCCAATATATCCTGTCAAACACTGATAGTTTAAACTGAAGGCGGGAAACGACAATCTGATCCAAGCTCAAGCTGCTCTAGCATTCGCCATTCAGGCTGCGCAACTGTTGGGAAGGGCGATCGGTGCGGGCCTCTTCGCTATTACGCCAGCTGGCGAAAGGGGGATGTGCTGCAAGGCGATTAAGTTGGGTAACGCCAGGGTTTTCCCAGTCACGACGTTGTAAAACGACGGCCAGTGAATTCATGGAGTCAAAGATTCAAATAGAGGACCTAACAGAACTCGCCGTAAAGACTGGCGAACAGTTCATACAGAGTCTCTTACGACTCAATGACAAGAAGAAAATCTTCGTCAACATGGTGGAGCACGACACACTTGTCTACTCCAAAAATATCAAAGATACAGTCTCAGAAGACCAAAGGGCAATTGAGACTTTTCAACAAAGGGTAATATCCGGAAACCTCCTCGGATTCCATTGCCCAGCTATCTGTCACTTTATTGTGAAGATAGTGGAAAAGGAAGGTGGCTCCTACAAATGCCATCATTGCGATAAAGGAAAGGCCATCGTTGAAGATGCCTCTGCCGACAGTGGTCCCAAAGATGGACCCCCACCCACGAGGAGCATCGTGGAAAAAGAAGACGTTCCAACCACGTCTTCAAAGCAAGTGGATTGATGTGATTGGCAGACATACTGTCCCACAAATGAAGATGGAATCTGTAAAAGAAAACGCGTGAAATAATGCGTCTGACAAAGGTTAGGTCGGCTGCCTTTAATCAATACCAAAGTGGTCCCTACCACGATGGAAAAACTGTGCAGTCGGTTTGGCTTTTTCTGACGAACAAATAAGATTCGTGGCCGACAGGTGGGGGTCCACCATGTGAAGGCATCTTCAGACTCCAATAATGGAGCAATGACGTAAGGGCTTACGAAATAAGTAAGGGTAGTTTGGGAAATGTCCACTCACCCGTCAGTCTATAAATACTTAGCCCCTCCCTCATTGTTAAGGGAGCAAAATCTCAGAGAGATAGTCCTAGAGAGAGAAAGAGAGCAAGTAGCCTAGAAGTAGTCAAGGCGGCGAAGTATTCAGGCACGTGGCCAGGAAGAAGAAAAGCCAAGACGACGAAAACAGGTAAGAGCTAAGCATCTAGATAAGTTGAAAACAATCTTCAAAAGTCCCACATCGCTTAGATAAGAAAACGAAGCTGAGTTTATATACAGCTAGAGTCGAAGTAGTGATTGAACAAAGCACCAGTGGTCTAGTGGTAGAATAGTACCCTGCCACGGTACAGACCCGGGTTCGATTCCCGGCTGGTGCAGCAGTCACGGCTGCTGTCAAGTTTAAGAGCTATGCTGGAAACAGCATAGCAAGTTTAAATAAGGCTAGTCCGTTATCAACTTGAAAAAGTGGCACCGAGTCGGTGCACGCTGGAATTGCAATGCGATCGTTGACAGCAGCCCGCGGTTCTATCTAGTTACGCGTTAAACCAACTAGAATTTTTTTGGCCGGCATGGTCCCAGCCTCCTCGCTGGCGCCGGCTGGGCAACATGCTTCGGCATGGCGAATGGGACTTTTTTTTGATATCTCCGGGGCTAATTGAATATGAAGATGAAGATGAAATATTTGGTGTGTCAAATAAAAAGCTGGTGTGCTTAAGTTTGTGTTTTTTTCTTGGCTTGTTGTGTTATGAATTTGTGGCTTTTTCTAATATTAAATGAATGTAAGATCTCATTATAATGAATAAACAAATGTTTCTATAATCCATTGTGAATGTTTTGTTGGATCTCTTCTGCAGCATATAACTACTGTATGTGCTATGGTATGGACTATGGAATATGATTAAAGATAAGAAGCTTGGCGTAATCTGCATGCCTGCAGTGCAGCGTGACCCGGTCGTGCCCCTCTCTAGAGATAATGAGCATTGCATGTCTAAGTTATAAAAAATTACCACATATTTTTTTTGTCACACTTGTTTGAAGTGCAGTTTATCTATCTTTATACATATATTTAAACTTTACTCTACGAATAATATAATCTATAGTACTACAATAATATCAGTGTTTTAGAGAATCATATAAATGAACAGTTAGACATGGTCTAAAGGACAATTGAGTATTTTGACAACAGGACTCTACAGTTTTATCTTTTTAGTGTGCATGTGTTCTCCTTTTTTTTTGCAAATAGCTTCACCTATATAATACTTCATCCATTTTATTAGTACATCCATTTAGGGTTTAGGGTTAATGGTTTTTATAGACTAATTTTTTTAGTACATCTATTTTATTCTATTTTAGCCTCTAAATTAAGAAAACTAAAACTCTATTTTAGTTTTTTTATTTAATAATTTAGATATAAAATAGAATAAAATAAAGTGACTAAAAATTAAACAAATACCCTTTAAGAAATTAAAAAAACTAAGGAAACATTTTTCTTGTTTCGAGTAGATAATGCCAGCCTGTTAAACGCCGTCGACGAGTCTAACGGACACCAACCAGCGAACCAGCAGCGTCGCGTCGGGCCAAGCGAAGCAGACGGCACGGCATCTCTGTCGCTGCCTCTGGACCCCTCTCGAGAGTTCCGCTCCACCGTTGGACTTGCTCCGCTGTCGGCATCCAGAAATTGCGTGGCGGAGCGGCAGACGTGAGCCGGCACGGCAGGCGGCCTCCTCCTCCTCTCACGGCACCGGCAGCTACGGGGGATTCCTTTCCCACCGCTCCTTCGCTTTCCCTTCCTCGCCCGCCGTAATAAATAGACACCCCCTCCACACCCTCTTTCCCCAACCTCGTGTTGTTCGGAGCGCACACACACACAACCAGATCTCCCCCAAATCCACCCGTCGGCACCTCCGCTTCAAGGTACGCCGCTCGTCCTCCCCCCCCCCCCCTCTCTACCTTCTCTAGATCGGCGTTCCGGTCCATGGTTAGGGCCCGGTAGTTCTACTTCTGTTCATGTTTGTGTTAGATCCGTGTTTGTGTTAGATCCGTGCTGCTAGCGTTCGTACACGGATGCGACCTGTACGTCAGACACGTTCTGATTGCTAACTTGCCAGTGTTTCTCTTTGGGGAATCCTGGGATGGCTCTAGCCGTTCCGCAGACGGGATCGATTTCATGATTTTTTTTGTTTCGTTGCATAGGGTTTGGTTTGCCCTTTTCCTTTATTTCAATATATGCCGTGCACTTGTTTGTCGGGTCATCTTTTCATGCTTTTTTTTGTCTTGGTTGTGATGATGTGGTCTGGTTGGGCGGTCGTTCTAGATCGGAGTAGAATTCTGTTTCAAACTACCTGGTGGATTTATTAATTTTGGATCTGTATGTGTGTGCCATACATATTCATAGTTACGAATTGAAGATGATGGATGGAAATATCGATCTAGGATAGGTATACATGTTGATGCGGGTTTTACTGATGCATATACAGAGATGCTTTTTGTTCGCTTGGTTGTGATGATGTGGTGTGGTTGGGCGGTCGTTCATTCGTTCTAGATCGGAGTAGAATACTGTTTCAAACTACCTGGTGTATTTATTAATTTTGGAACTGTATGTGTGTGTCATACATCTTCATAGTTACGAGTTTAAGATGGATGGAAATATCGATCTAGGATAGGTATACATGTTGATGTGGGTTTTACTGATGCATATACATGATGGCATATGCAGCATCTATTCATATGCTCTAACCTTGAGTACCTATCTATTATAATAAACAAGTATGTTTTATAATTATTTTGATCTTGATATACTTGGATGATGGCATATGCAGCAGCTATATGTGGATTTTTTTAGCCCTGCCTTCATACGCTATTTATTTGCTTGGTACTGTTTCTTTTGTCGATGCTCACCCTGTTGTTTGGTGTTACTTCTGCAGCCCTAGGACTGGTTGTACAATGCCGGCGGCCAAGAGAGTCAAGCTCGACGGCGGGAAGCGCACAGCTGATGGTTCTGAATTCGAGTCCCCTAAGAAAAAGAGAAAAGTGGACAAGAAGTACTCGATCGGCCTCGATATTGGGACTAACTCTGTTGGCTGGGCCGTGATCACCGACGAGTACAAGGTGCCCTCAAAGAAGTTCAAGGTCCTGGGCAACACCGATCGGCATTCCATCAAGAAGAATCTCATTGGCGCTCTCCTGTTCGACAGCGGCGAGACGGCTGAGGCTACGCGGCTCAAGCGCACCGCCCGCAGGCGGTACACGCGCAGGAAGAATCGCATCTGCTACCTGCAGGAGATTTTCTCCAACGAGATGGCGAAGGTTGACGATTCTTTCTTCCACAGGCTGGAGGAGTCATTCCTCGTGGAGGAGGATAAGAAGCACGAGCGGCATCCAATCTTCGGCAACATTGTCGACGAGGTTGCCTACCACGAGAAGTACCCTACGATCTACCATCTGCGGAAGAAGCTCGTGGACTCCACAGATAAGGCGGACCTCCGCCTGATCTACCTCGCTCTGGCCCACATGATTAAGTTCAGGGGCCATTTCCTGATCGAGGGGGATCTCAACCCGGACAATAGCGATGTTGACAAGCTGTTCATCCAGCTCGTGCAGACGTACAACCAGCTCTTCGAGGAGAACCCCATTAATGCGTCAGGCGTCGACGCGAAGGCTATCCTGTCCGCTAGGCTCTCGAAGTCTCGGAAGCTCGAGAACCTGATCGCCCAGCTGCCGGGCGAGAAGAAGAACGGCCTGTTCGGGAATCTCATTGCGCTCAGCCTGGGGCTCACGCCCAACTTCAAGTCGAATTTCGATCTCGCTGAGGACGCCAAGCTGCAGCTCTCCAAGGACACATACGACGATGACCTGGATAACCTCCTGGCCCAGATCGGCGATCAGTACGCGGACCTGTTCCTCGCTGCCAAGAATCTGTCGGACGCCATCCTCCTGTCTGATATTCTCAGGGTGAACACCGAGATTACGAAGGCTCCGCTCTCAGCCTCCATGATCAAGCGCTACGACGAGCACCATCAGGATCTGACCCTCCTGAAGGCGCTGGTCAGGCAGCAGCTCCCCGAGAAGTACAAGGAGATCTTCTTCGATCAGTCGAAGAACGGCTACGCTGGGTACATTGACGGCGGGGCCTCTCAGGAGGAGTTCTACAAGTTCATCAAGCCGATTCTGGAGAAGATGGACGGCACGGAGGAGCTGCTGGTGAAGCTCAAGCGCGAGGACCTCCTGAGGAAGCAGCGGACATTCGATAACGGCAGCATCCCACACCAGATTCATCTCGGGGAGCTGCACGCTATCCTGAGGAGGCAGGAGGACTTCTACCCTTTCCTCAAGGATAACCGCGAGAAGATCGAGAAGATTCTGACTTTCAGGATCCCGTACTACGTCGGCCCACTCGCTAGGGGCAACTCCCGCTTCGCTTGGATGACCCGCAAGTCAGAGGAGACGATCACGCCGTGGAACTTCGAGGAGGTGGTCGACAAGGGCGCTAGCGCTCAGTCGTTCATCGAGAGGATGACGAATTTCGACAAGAACCTGCCAAATGAGAAGGTGCTCCCTAAGCACTCGCTCCTGTACGAGTACTTCACAGTCTACAACGAGCTGACTAAGGTGAAGTATGTGACCGAGGGCATGAGGAAGCCGGCTTTCCTGTCTGGGGAGCAGAAGAAGGCCATCGTGGACCTCCTGTTCAAGACCAACCGGAAGGTCACGGTTAAGCAGCTCAAGGAGGACTACTTCAAGAAGATTGAGTGCTTCGATTCGGTCGAGATCTCTGGCGTTGAGGACCGCTTCAACGCCTCCCTGGGGACCTACCACGATCTCCTGAAGATCATTAAGGATAAGGACTTCCTGGACAACGAGGAGAATGAGGATATCCTCGAGGACATTGTGCTGACACTCACTCTGTTCGAGGACCGGGAGATGATCGAGGAGCGCCTGAAGACTTACGCCCATCTCTTCGATGACAAGGTCATGAAGCAGCTCAAGAGGAGGAGGTACACCGGCTGGGGGAGGCTGAGCAGGAAGCTCATCAACGGCATTCGGGACAAGCAGTCCGGGAAGACGATCCTCGACTTCCTGAAGAGCGATGGCTTCGCGAACCGCAATTTCATGCAGCTGATTCACGATGACAGCCTCACATTCAAGGAGGATATCCAGAAGGCTCAGGTGAGCGGCCAGGGGGACTCGCTGCACGAGCATATCGCGAACCTCGCTGGCTCGCCAGCTATCAAGAAGGGGATTCTGCAGACCGTGAAGGTTGTGGACGAGCTGGTGAAGGTCATGGGCAGGCACAAGCCTGAGAACATCGTCATTGAGATGGCCCGGGAGAATCAGACCACGCAGAAGGGCCAGAAGAACTCACGCGAGAGGATGAAGAGGATCGAGGAGGGCATTAAGGAGCTGGGGTCCCAGATCCTCAAGGAGCACCCGGTGGAGAACACGCAGCTGCAGAATGAGAAGCTCTACCTGTACTACCTCCAGAATGGCCGCGATATGTATGTGGACCAGGAGCTGGATATTAACAGGCTCAGCGATTACGACGTCGATGCCATCGTTCCACAGTCATTCCTGAAGGATGACTCCATTGACAACAAGGTCCTCACCAGGTCGGACAAGAACCGGGGCAAGTCTGATAATGTTCCTTCAGAGGAGGTCGTTAAGAAGATGAAGAACTACTGGCGCCAGCTCCTGAATGCCAAGCTGATCACGCAGCGGAAGTTCGATAACCTCACAAAGGCTGAGAGGGGCGGGCTCTCTGAGCTGGACAAGGCGGGCTTCATCAAGAGGCAGCTGGTCGAGACACGGCAGATCACTAAGCACGTTGCGCAGATTCTCGACTCACGGATGAACACTAAGTACGATGAGAATGACAAGCTGATCCGCGAGGTGAAGGTCATCACCCTGAAGTCAAAGCTCGTCTCCGACTTCAGGAAGGATTTCCAGTTCTACAAGGTTCGGGAGATCAACAATTACCACCATGCCCATGACGCGTACCTGAACGCGGTGGTCGGCACAGCTCTGATCAAGAAGTACCCAAAGCTCGAGAGCGAGTTCGTGTACGGGGACTACAAGGTTTACGATGTGAGGAAGATGATCGCCAAGTCGGAGCAGGAGATTGGCAAGGCTACCGCCAAGTACTTCTTCTACTCTAACATTATGAATTTCTTCAAGACAGAGATCACTCTGGCCAATGGCGAGATCCGGAAGCGCCCCCTCATCGAGACGAACGGCGAGACGGGGGAGATCGTGTGGGACAAGGGCAGGGATTTCGCGACCGTCAGGAAGGTTCTCTCCATGCCACAAGTGAATATCGTCAAGAAGACAGAGGTCCAGACTGGCGGGTTCTCTAAGGAGTCAATTCTGCCTAAGCGGAACAGCGACAAGCTCATCGCCCGCAAGAAGGACTGGGATCCGAAGAAGTACGGCGGGTTCGACAGCCCCACTGTGGCCTACTCGGTCCTGGTTGTGGCGAAGGTTGAGAAGGGCAAGTCCAAGAAGCTCAAGAGCGTGAAGGAGCTGCTGGGGATCACGATTATGGAGCGCTCCAGCTTCGAGAAGAACCCGATCGATTTCCTGGAGGCGAAGGGCTACAAGGAGGTGAAGAAGGACCTGATCATTAAGCTCCCCAAGTACTCACTCTTCGAGCTGGAGAACGGCAGGAAGCGGATGCTGGCTTCCGCTGGCGAGCTGCAGAAGGGGAACGAGCTGGCTCTGCCGTCCAAGTATGTGAACTTCCTCTACCTGGCCTCCCACTACGAGAAGCTCAAGGGCAGCCCCGAGGACAACGAGCAGAAGCAGCTGTTCGTCGAGCAGCACAAGCATTACCTCGACGAGATCATTGAGCAGATTTCCGAGTTCTCCAAGCGCGTGATCCTGGCCGACGCGAATCTGGATAAGGTCCTCTCCGCGTACAACAAGCACCGCGACAAGCCAATCAGGGAGCAGGCTGAGAATATCATTCATCTCTTCACCCTGACGAACCTCGGCGCCCCTGCTGCTTTCAAGTACTTCGACACAACTATCGATCGCAAGAGGTACACAAGCACTAAGGAGGTCCTGGACGCGACCCTCATCCACCAGTCGATTACCGGCCTCTACGAGACGCGCATCGACCTGTCTCAGCTCGGGGGCGACGAATTCTCCGGGAGCGAGACGCCAGGCACCTCCGAGTCGGCCACCCCAGAATCTGCCACAGTGGTGTCCGGCCAAAAGCAGGACCGCCAGGGCGGAGAACGCAGAAGGTCCCAGCTCGATAGGGATCAGTGTGCCTACTGCAAGGAGAAGGGCCACTGGGCCAAAGACTGCCCGAAAAAGCCGCGCGGCCCACGCGGCCCAAGGCCACAAACATCCCTCCTTCCAAAGAAGAAGCGGAAGGTGGAGCTCAGCGGAGGATCTTCCGGAGGATCTAGCGGCTCCGAGACACCAGGAACATCCGAAAGCGCTACACCAGAATCTAGCGGAGGCTCTTCCGGAGGATCTAGGCCTACCCTCAACATCGAGGATGAGTATCGCCTCCACGAAACCTCCAAAGAACCGGACGTGTCCCTCGGCAGCACATGGCTCAGCGACTTCCCACAAGCGTGGGCCGAAACCGGCGGCATGGGCCTCGCCGTCCGCCAAGCCCCACTCATTATCCCGCTGAAGGCGACCTCCACACCGGTGTCCATCAAGCAGTACCCGATGAGCCAAGAGGCGAGGCTCGGGATTAAGCCGCACATTCAGCGCCTCCTCGATCAAGGCATTCTCGTGCCGTGCCAATCCCCGTGGAATACACCACTCCTCCCGGTCAAAAAGCCGGGCACCAACGACTATCGCCCGGTCCAAGATCTCCGCGAGGTCAACAAGCGCGTGGAAGACATCCACCCGACCGTCCCGAACCCGTATAATCTGCTCTCCGGGCTCCCACCATCCCACCAGTGGTATACAGTGCTGGACCTCAAAGACGCCTTCTTCTGTCTCCGCCTCCACCCAACAAGCCAGCCGCTCTTCGCCTTCGAGTGGCGCGACCCGGAGATGGGCATCTCCGGCCAACTGACATGGACACGCCTCCCGCAAGGCTTCAAGAACAGCCCGACACTCTTCAACGAGGCGCTCCATAGGGACCTCGCGGATTTTCGCATCCAGCATCCGGACCTCATCCTCCTCCAGTATGCGGATGATCTCCTCCTCGCCGCGACCTCCGAGCTGGATTGTCAACAAGGCACACGCGCGCTCCTCCAAACACTCGGGAACCTCGGCTATCGCGCGTCCGCGAAAAAGGCCCAAATCTGCCAGAAGCAAGTGAAGTACCTCGGGTATCTGCTCAAGGAAGGCCAACGCTGGCTCACCGAAGCGCGCAAAGAAACAGTGATGGGGCAACCGACACCGAAAACACCACGCCAGCTGCGCGAGTTTCTCGGCAAAGCCGGCTTCTGTCGCCTCTTCATCCCGGGCTTTGCCGAGATGGCCGCGCCACTCTACCCACTCACCAAGCCGGGCACACTGTTTAACTGGGGGCCGGATCAGCAGAAAGCCTACCAAGAGATCAAACAAGCGCTCCTCACCGCCCCAGCGCTCGGGCTCCCAGATCTCACAAAGCCGTTCGAGCTGTTCGTCGATGAGAAGCAAGGCTACGCGAAGGGCGTGCTCACACAGAAGCTCGGCCCGTGGAGGAGGCCAGTGGCCTATCTCTCCAAAAAACTCGATCCAGTGGCCGCCGGCTGGCCACCGTGTCTGCGCATGGTCGCCGCGATTGCCGTGCTCACAAAGGATGCCGGCAAACTCACAATGGGCCAGCCGCTGGTGATCCTCGCGCCACATGCCGTGGAAGCCCTCGTCAAACAGCCGCCGGATAGGTGGCTCTCCAATGCGCGCATGACCCATTACCAAGCGCTCCTCCTCGACACCGATCGCGTCCAGTTCGGCCCAGTGGTCGCCCTCAATCCGGCGACACTGCTGCCACTCCCAGAGGAGGGCCTCCAACACAACTGTCTGGATATTCTCGCGGAAGCGCATGGCACAAGGCCAGACCTCACAGATCAACCGCTCAGCGGCGGCAGCAAAAGAACGGCGGACGGCTCTGAGAAGCGCACCGCTGATAGCCAGCATTCAACTCCTCCGAAAACAAAGAGGAAAGTTGAGTTCGAACCGAAGAAGAAAAGGAAGGTGTGAGAGCTCAGAGCTTTCGTTCGTATCATCGGTTTCGACAACGTTCGTCAAGTTCAATGCATCAGTTTCATTGCGCACACACCAGAATCCTACTGAGTTTGAGTATTATGGCATTGGGAAAACTGTTTTTCTTGTACCATTTGTTGTGCTTGTAATTTACTGTGTTTTTTATTCGGTTTTCGCTATCGAACTGTGAAATGGAAATGGATGGAGAAGAGTTAATGAATGATATGGTCCTTTTGTTCATTCTCAAATTAATATTATTTGTTTTTTCTCTTATTTGTTGTGTGTTGAATTTGAAATTATAAGAGATATGCAAACATTTTGTTTTGAGTAAAAATGTGTCAAATCGTGGCCTCTAATGACCGAAGTTAATATGAGGAGTAAAACACTTGTAGTTGTACCATTATGCTTATTCACTAGGCAACAAATATATTTTCAGACCTAGAAAAGCTGCAAATGTTACTGAATACAAGTATGTCCTCTTGTGTTTTAGACATTTATGAACTTTCCTTTATGTAATTTTCCAGAATCCTTGTCAGATTCTAATCATTGCTTTATAATTATAGTTATACTCATGGATTTGTAGTTGAGTATGAAAATATTTTTTAATGCATTTTATGACTTGCCAATTGATTGACAACGAATTCGTAATCATGTCATAGCTGTTTCCTGTGTGAAATTGTTATCCGCTCACAATTCCACACAACATACGAGCCGGAAGCATAAAGTGTAAAGCCTGGGGTGCCTAATGAGTGAGCTAACTCACATTAATTGCGTTGCGCTCACTGCCCGCTTTCCAGTCGGGAAACCTGTCGTGCCAGCTGCATTAATGAATCGGCCAACGCGCGGGGAGAGGCGGTTTGCGTATTGGCTAGAGCAGCTTGCCAACATGGTGGAGCACGACACTCTCGTCTACTCCAAGAATATCAAAGATACAGTCTCAGAAGACCAAAGGGCTATTGAGACTTTTCAACAAAGGGTAATATCGGGAAACCTCCTCGGATTCCATTGCCCAGCTATCTGTCACTTCATCAAAAGGACAGTAGAAAAGGAAGGTGGCACCTACAAATGCCATCATTGCGATAAAGGAAAGGCTATCGTTCAAGATGCCTCTGCCGACAGTGGTCCCAAAGATGGACCCCCACCCACGAGGAGCATCGTGGAAAAAGAAGACGTTCCAACCACGTCTTCAAAGCAAGTGGATTGATGTGAACATGGTGGAGCACGACACTCTCGTCTACTCCAAGAATATCAAAGATACAGTCTCAGAAGACCAAAGGGCTATTGAGACTTTTCAACAAAGGGTAATATCGGGAAACCTCCTCGGATTCCATTGCCCAGCTATCTGTCACTTCATCAAAAGGACAGTAGAAAAGGAAGGTGGCACCTACAAATGCCATCATTGCGATAAAGGAAAGGCTATCGTTCAAGATGCCTCTGCCGACAGTGGTCCCAAAGATGGACCCCCACCCACGAGGAGCATCGTGGAAAAAGAAGACGTTCCAACCACGTCTTCAAAGCAAGTGGATTGATGTGATATCTCCACTGACGTAAGGGATGACGCACAATCCCACTATCCTTCGCAAGACCCTTCCTCTATATAAGGAAGTTCATTTCATTTGGAGAGGACACGCTGAAATCACCAGTCTCTCTCTACAAATCTATCTCTCTCGAGCTTTCGCAGATCCGGGGGGCAATGAGATATGAAAAAGCCTGAACTCACCGCGACGTCTGTCGAGAAGTTTCTGATCGAAAAGTTCGACAGCGTCTCCGACCTGATGCAGCTCTCGGAGGGCGAAGAATCTCGTGCTTTCAGCTTCGATGTAGGAGGGCGTGGATATGTCCTGCGGGTAAATAGCTGCGCCGATGGTTTCTACAAAGATCGTTATGTTTATCGGCACTTTGCATCGGCCGCGCTCCCGATTCCGGAAGTGCTTGACATTGGGGAGTTTAGCGAGAGCCTGACCTATTGCATCTCCCGCCGTTCACAGGGTGTCACGTTGCAAGACCTGCCTGAAACCGAACTGCCCGCTGTTCTACAACCGGTCGCGGAGGCTATGGATGCGATCGCTGCGGCCGATCTTAGCCAGACGAGCGGGTTCGGCCCATTCGGACCGCAAGGAATCGGTCAATACACTACATGGCGTGATTTCATATGCGCGATTGCTGATCCCCATGTGTATCACTGGCAAACTGTGATGGACGACACCGTCAGTGCGTCCGTCGCGCAGGCTCTCGATGAGCTGATGCTTTGGGCCGAGGACTGCCCCGAAGTCCGGCACCTCGTGCACGCGGATTTCGGCTCCAACAATGTCCTGACGGACAATGGCCGCATAACAGCGGTCATTGACTGGAGCGAGGCGATGTTCGGGGATTCCCAATACGAGGTCGCCAACATCTTCTTCTGGAGGCCGTGGTTGGCTTGTATGGAGCAGCAGACGCGCTACTTCGAGCGGAGGCATCCGGAGCTTGCAGGATCGCCACGACTCCGGGCGTATATGCTCCGCATTGGTCTTGACCAACTCTATCAGAGCTTGGTTGACGGCAATTTCGATGATGCAGCTTGGGCGCAGGGTCGATGCGACGCAATCGTCCGATCCGGAGCCGGGACTGTCGGGCGTACACAAATCGCCCGCAGAAGCGCGGCCGTCTGGACCGATGGCTGTGTAGAAGTACTCGCCGATAGTGGAAACCGACGCCCCAGCACTCGTCCGAGGGCAAAGAAATAGAGTAGATGCCGACCGGGATCTGTCGATCGACAAGCTCGAGTTTCTCCATAATAATGTGTGAGTAGTTCCCAGATAAGGGAATTAGGGTTCCTATAGGGTTTCGCTCATGTGTTGAGCATATAAGAAACCCTTAGTATGTATTTGTATTTGTAAAATACTTCTATCAATAAAATTTCTAATTCCTAAAACCAAAATCCAGTACTAAAATCCAGATCCCCCGAATTAATTCGGCGTTAATTCAGTACATTAAAAACGTCCGCAATGTGTTATTAAGTTGTCTAAGCGTCAATTTGTTTACACCACAATATATCCTGCCA

**Sequence 78 Plasmids sequence of pH-35C-M-MLV-OsALS**

(RB T-DNA repeat-35C promoter-tRNAGly RS-pegRNA-Ubi promoter-NLSc-myc-NLSsv40-nCas9(H840A/ R221K/N394K)-XTEN-NC-NLS-32aa Linker-M-MLV-RT-NLSvbp-HygR cassette-E9-terminator-CaMV poly(A) signal-LB T-DNA repeat)

GTTTACCCGCCAATATATCCTGTCAAACACTGATAGTTTAAACTGAAGGCGGGAAACGACAATCTGATCCAAGCTCAAGCTGCTCTAGCATTCGCCATTCAGGCTGCGCAACTGTTGGGAAGGGCGATCGGTGCGGGCCTCTTCGCTATTACGCCAGCTGGCGAAAGGGGGATGTGCTGCAAGGCGATTAAGTTGGGTAACGCCAGGGTTTTCCCAGTCACGACGTTGTAAAACGACGGCCAGTGAATTCATGGAGTCAAAGATTCAAATAGAGGACCTAACAGAACTCGCCGTAAAGACTGGCGAACAGTTCATACAGAGTCTCTTACGACTCAATGACAAGAAGAAAATCTTCGTCAACATGGTGGAGCACGACACACTTGTCTACTCCAAAAATATCAAAGATACAGTCTCAGAAGACCAAAGGGCAATTGAGACTTTTCAACAAAGGGTAATATCCGGAAACCTCCTCGGATTCCATTGCCCAGCTATCTGTCACTTTATTGTGAAGATAGTGGAAAAGGAAGGTGGCTCCTACAAATGCCATCATTGCGATAAAGGAAAGGCCATCGTTGAAGATGCCTCTGCCGACAGTGGTCCCAAAGATGGACCCCCACCCACGAGGAGCATCGTGGAAAAAGAAGACGTTCCAACCACGTCTTCAAAGCAAGTGGATTGATGTGATTGGCAGACATACTGTCCCACAAATGAAGATGGAATCTGTAAAAGAAAACGCGTGAAATAATGCGTCTGACAAAGGTTAGGTCGGCTGCCTTTAATCAATACCAAAGTGGTCCCTACCACGATGGAAAAACTGTGCAGTCGGTTTGGCTTTTTCTGACGAACAAATAAGATTCGTGGCCGACAGGTGGGGGTCCACCATGTGAAGGCATCTTCAGACTCCAATAATGGAGCAATGACGTAAGGGCTTACGAAATAAGTAAGGGTAGTTTGGGAAATGTCCACTCACCCGTCAGTCTATAAATACTTAGCCCCTCCCTCATTGTTAAGGGAGCAAAATCTCAGAGAGATAGTCCTAGAGAGAGAAAGAGAGCAAGTAGCCTAGAAGTAGTCAAGGCGGCGAAGTATTCAGGCACGTGGCCAGGAAGAAGAAAAGCCAAGACGACGAAAACAGGTAAGAGCTAAGCATCTAGATAAGTTGAAAACAATCTTCAAAAGTCCCACATCGCTTAGATAAGAAAACGAAGCTGAGTTTATATACAGCTAGAGTCGAAGTAGTGATTGAACAAAGCACCAGTGGTCTAGTGGTAGAATAGTACCCTGCCACGGTACAGACCCGGGTTCGATTCCCGGCTGGTGCAGGGTATGGTGGTGCAATGGGGTTTAAGAGCTATGCTGGAAACAGCATAGCAAGTTTAAATAAGGCTAGTCCGTTATCAACTTGAAAAAGTGGCACCGAGTCGGTGCAAAACCTATCCTCCAATTGCACCACCGCGGTTCTATCTAGTTACGCGTTAAACCAACTAGAATTTTTTTGGCCGGCATGGTCCCAGCCTCCTCGCTGGCGCCGGCTGGGCAACATGCTTCGGCATGGCGAATGGGACTTTTTTTTGATATCTCCGGGGCTAATTGAATATGAAGATGAAGATGAAATATTTGGTGTGTCAAATAAAAAGCTGGTGTGCTTAAGTTTGTGTTTTTTTCTTGGCTTGTTGTGTTATGAATTTGTGGCTTTTTCTAATATTAAATGAATGTAAGATCTCATTATAATGAATAAACAAATGTTTCTATAATCCATTGTGAATGTTTTGTTGGATCTCTTCTGCAGCATATAACTACTGTATGTGCTATGGTATGGACTATGGAATATGATTAAAGATAAGAAGCTTGGCGTAATCTGCATGCCTGCAGTGCAGCGTGACCCGGTCGTGCCCCTCTCTAGAGATAATGAGCATTGCATGTCTAAGTTATAAAAAATTACCACATATTTTTTTTGTCACACTTGTTTGAAGTGCAGTTTATCTATCTTTATACATATATTTAAACTTTACTCTACGAATAATATAATCTATAGTACTACAATAATATCAGTGTTTTAGAGAATCATATAAATGAACAGTTAGACATGGTCTAAAGGACAATTGAGTATTTTGACAACAGGACTCTACAGTTTTATCTTTTTAGTGTGCATGTGTTCTCCTTTTTTTTTGCAAATAGCTTCACCTATATAATACTTCATCCATTTTATTAGTACATCCATTTAGGGTTTAGGGTTAATGGTTTTTATAGACTAATTTTTTTAGTACATCTATTTTATTCTATTTTAGCCTCTAAATTAAGAAAACTAAAACTCTATTTTAGTTTTTTTATTTAATAATTTAGATATAAAATAGAATAAAATAAAGTGACTAAAAATTAAACAAATACCCTTTAAGAAATTAAAAAAACTAAGGAAACATTTTTCTTGTTTCGAGTAGATAATGCCAGCCTGTTAAACGCCGTCGACGAGTCTAACGGACACCAACCAGCGAACCAGCAGCGTCGCGTCGGGCCAAGCGAAGCAGACGGCACGGCATCTCTGTCGCTGCCTCTGGACCCCTCTCGAGAGTTCCGCTCCACCGTTGGACTTGCTCCGCTGTCGGCATCCAGAAATTGCGTGGCGGAGCGGCAGACGTGAGCCGGCACGGCAGGCGGCCTCCTCCTCCTCTCACGGCACCGGCAGCTACGGGGGATTCCTTTCCCACCGCTCCTTCGCTTTCCCTTCCTCGCCCGCCGTAATAAATAGACACCCCCTCCACACCCTCTTTCCCCAACCTCGTGTTGTTCGGAGCGCACACACACACAACCAGATCTCCCCCAAATCCACCCGTCGGCACCTCCGCTTCAAGGTACGCCGCTCGTCCTCCCCCCCCCCCCCTCTCTACCTTCTCTAGATCGGCGTTCCGGTCCATGGTTAGGGCCCGGTAGTTCTACTTCTGTTCATGTTTGTGTTAGATCCGTGTTTGTGTTAGATCCGTGCTGCTAGCGTTCGTACACGGATGCGACCTGTACGTCAGACACGTTCTGATTGCTAACTTGCCAGTGTTTCTCTTTGGGGAATCCTGGGATGGCTCTAGCCGTTCCGCAGACGGGATCGATTTCATGATTTTTTTTGTTTCGTTGCATAGGGTTTGGTTTGCCCTTTTCCTTTATTTCAATATATGCCGTGCACTTGTTTGTCGGGTCATCTTTTCATGCTTTTTTTTGTCTTGGTTGTGATGATGTGGTCTGGTTGGGCGGTCGTTCTAGATCGGAGTAGAATTCTGTTTCAAACTACCTGGTGGATTTATTAATTTTGGATCTGTATGTGTGTGCCATACATATTCATAGTTACGAATTGAAGATGATGGATGGAAATATCGATCTAGGATAGGTATACATGTTGATGCGGGTTTTACTGATGCATATACAGAGATGCTTTTTGTTCGCTTGGTTGTGATGATGTGGTGTGGTTGGGCGGTCGTTCATTCGTTCTAGATCGGAGTAGAATACTGTTTCAAACTACCTGGTGTATTTATTAATTTTGGAACTGTATGTGTGTGTCATACATCTTCATAGTTACGAGTTTAAGATGGATGGAAATATCGATCTAGGATAGGTATACATGTTGATGTGGGTTTTACTGATGCATATACATGATGGCATATGCAGCATCTATTCATATGCTCTAACCTTGAGTACCTATCTATTATAATAAACAAGTATGTTTTATAATTATTTTGATCTTGATATACTTGGATGATGGCATATGCAGCAGCTATATGTGGATTTTTTTAGCCCTGCCTTCATACGCTATTTATTTGCTTGGTACTGTTTCTTTTGTCGATGCTCACCCTGTTGTTTGGTGTTACTTCTGCAGCCCTAGGACTGGTTGTACAATGCCGGCGGCCAAGAGAGTCAAGCTCGACGGCGGGAAGCGCACAGCTGATGGTTCTGAATTCGAGTCCCCTAAGAAAAAGAGAAAAGTGGACAAGAAGTACTCGATCGGCCTCGATATTGGGACTAACTCTGTTGGCTGGGCCGTGATCACCGACGAGTACAAGGTGCCCTCAAAGAAGTTCAAGGTCCTGGGCAACACCGATCGGCATTCCATCAAGAAGAATCTCATTGGCGCTCTCCTGTTCGACAGCGGCGAGACGGCTGAGGCTACGCGGCTCAAGCGCACCGCCCGCAGGCGGTACACGCGCAGGAAGAATCGCATCTGCTACCTGCAGGAGATTTTCTCCAACGAGATGGCGAAGGTTGACGATTCTTTCTTCCACAGGCTGGAGGAGTCATTCCTCGTGGAGGAGGATAAGAAGCACGAGCGGCATCCAATCTTCGGCAACATTGTCGACGAGGTTGCCTACCACGAGAAGTACCCTACGATCTACCATCTGCGGAAGAAGCTCGTGGACTCCACAGATAAGGCGGACCTCCGCCTGATCTACCTCGCTCTGGCCCACATGATTAAGTTCAGGGGCCATTTCCTGATCGAGGGGGATCTCAACCCGGACAATAGCGATGTTGACAAGCTGTTCATCCAGCTCGTGCAGACGTACAACCAGCTCTTCGAGGAGAACCCCATTAATGCGTCAGGCGTCGACGCGAAGGCTATCCTGTCCGCTAGGCTCTCGAAGTCTCGGAAGCTCGAGAACCTGATCGCCCAGCTGCCGGGCGAGAAGAAGAACGGCCTGTTCGGGAATCTCATTGCGCTCAGCCTGGGGCTCACGCCCAACTTCAAGTCGAATTTCGATCTCGCTGAGGACGCCAAGCTGCAGCTCTCCAAGGACACATACGACGATGACCTGGATAACCTCCTGGCCCAGATCGGCGATCAGTACGCGGACCTGTTCCTCGCTGCCAAGAATCTGTCGGACGCCATCCTCCTGTCTGATATTCTCAGGGTGAACACCGAGATTACGAAGGCTCCGCTCTCAGCCTCCATGATCAAGCGCTACGACGAGCACCATCAGGATCTGACCCTCCTGAAGGCGCTGGTCAGGCAGCAGCTCCCCGAGAAGTACAAGGAGATCTTCTTCGATCAGTCGAAGAACGGCTACGCTGGGTACATTGACGGCGGGGCCTCTCAGGAGGAGTTCTACAAGTTCATCAAGCCGATTCTGGAGAAGATGGACGGCACGGAGGAGCTGCTGGTGAAGCTCAAGCGCGAGGACCTCCTGAGGAAGCAGCGGACATTCGATAACGGCAGCATCCCACACCAGATTCATCTCGGGGAGCTGCACGCTATCCTGAGGAGGCAGGAGGACTTCTACCCTTTCCTCAAGGATAACCGCGAGAAGATCGAGAAGATTCTGACTTTCAGGATCCCGTACTACGTCGGCCCACTCGCTAGGGGCAACTCCCGCTTCGCTTGGATGACCCGCAAGTCAGAGGAGACGATCACGCCGTGGAACTTCGAGGAGGTGGTCGACAAGGGCGCTAGCGCTCAGTCGTTCATCGAGAGGATGACGAATTTCGACAAGAACCTGCCAAATGAGAAGGTGCTCCCTAAGCACTCGCTCCTGTACGAGTACTTCACAGTCTACAACGAGCTGACTAAGGTGAAGTATGTGACCGAGGGCATGAGGAAGCCGGCTTTCCTGTCTGGGGAGCAGAAGAAGGCCATCGTGGACCTCCTGTTCAAGACCAACCGGAAGGTCACGGTTAAGCAGCTCAAGGAGGACTACTTCAAGAAGATTGAGTGCTTCGATTCGGTCGAGATCTCTGGCGTTGAGGACCGCTTCAACGCCTCCCTGGGGACCTACCACGATCTCCTGAAGATCATTAAGGATAAGGACTTCCTGGACAACGAGGAGAATGAGGATATCCTCGAGGACATTGTGCTGACACTCACTCTGTTCGAGGACCGGGAGATGATCGAGGAGCGCCTGAAGACTTACGCCCATCTCTTCGATGACAAGGTCATGAAGCAGCTCAAGAGGAGGAGGTACACCGGCTGGGGGAGGCTGAGCAGGAAGCTCATCAACGGCATTCGGGACAAGCAGTCCGGGAAGACGATCCTCGACTTCCTGAAGAGCGATGGCTTCGCGAACCGCAATTTCATGCAGCTGATTCACGATGACAGCCTCACATTCAAGGAGGATATCCAGAAGGCTCAGGTGAGCGGCCAGGGGGACTCGCTGCACGAGCATATCGCGAACCTCGCTGGCTCGCCAGCTATCAAGAAGGGGATTCTGCAGACCGTGAAGGTTGTGGACGAGCTGGTGAAGGTCATGGGCAGGCACAAGCCTGAGAACATCGTCATTGAGATGGCCCGGGAGAATCAGACCACGCAGAAGGGCCAGAAGAACTCACGCGAGAGGATGAAGAGGATCGAGGAGGGCATTAAGGAGCTGGGGTCCCAGATCCTCAAGGAGCACCCGGTGGAGAACACGCAGCTGCAGAATGAGAAGCTCTACCTGTACTACCTCCAGAATGGCCGCGATATGTATGTGGACCAGGAGCTGGATATTAACAGGCTCAGCGATTACGACGTCGATGCCATCGTTCCACAGTCATTCCTGAAGGATGACTCCATTGACAACAAGGTCCTCACCAGGTCGGACAAGAACCGGGGCAAGTCTGATAATGTTCCTTCAGAGGAGGTCGTTAAGAAGATGAAGAACTACTGGCGCCAGCTCCTGAATGCCAAGCTGATCACGCAGCGGAAGTTCGATAACCTCACAAAGGCTGAGAGGGGCGGGCTCTCTGAGCTGGACAAGGCGGGCTTCATCAAGAGGCAGCTGGTCGAGACACGGCAGATCACTAAGCACGTTGCGCAGATTCTCGACTCACGGATGAACACTAAGTACGATGAGAATGACAAGCTGATCCGCGAGGTGAAGGTCATCACCCTGAAGTCAAAGCTCGTCTCCGACTTCAGGAAGGATTTCCAGTTCTACAAGGTTCGGGAGATCAACAATTACCACCATGCCCATGACGCGTACCTGAACGCGGTGGTCGGCACAGCTCTGATCAAGAAGTACCCAAAGCTCGAGAGCGAGTTCGTGTACGGGGACTACAAGGTTTACGATGTGAGGAAGATGATCGCCAAGTCGGAGCAGGAGATTGGCAAGGCTACCGCCAAGTACTTCTTCTACTCTAACATTATGAATTTCTTCAAGACAGAGATCACTCTGGCCAATGGCGAGATCCGGAAGCGCCCCCTCATCGAGACGAACGGCGAGACGGGGGAGATCGTGTGGGACAAGGGCAGGGATTTCGCGACCGTCAGGAAGGTTCTCTCCATGCCACAAGTGAATATCGTCAAGAAGACAGAGGTCCAGACTGGCGGGTTCTCTAAGGAGTCAATTCTGCCTAAGCGGAACAGCGACAAGCTCATCGCCCGCAAGAAGGACTGGGATCCGAAGAAGTACGGCGGGTTCGACAGCCCCACTGTGGCCTACTCGGTCCTGGTTGTGGCGAAGGTTGAGAAGGGCAAGTCCAAGAAGCTCAAGAGCGTGAAGGAGCTGCTGGGGATCACGATTATGGAGCGCTCCAGCTTCGAGAAGAACCCGATCGATTTCCTGGAGGCGAAGGGCTACAAGGAGGTGAAGAAGGACCTGATCATTAAGCTCCCCAAGTACTCACTCTTCGAGCTGGAGAACGGCAGGAAGCGGATGCTGGCTTCCGCTGGCGAGCTGCAGAAGGGGAACGAGCTGGCTCTGCCGTCCAAGTATGTGAACTTCCTCTACCTGGCCTCCCACTACGAGAAGCTCAAGGGCAGCCCCGAGGACAACGAGCAGAAGCAGCTGTTCGTCGAGCAGCACAAGCATTACCTCGACGAGATCATTGAGCAGATTTCCGAGTTCTCCAAGCGCGTGATCCTGGCCGACGCGAATCTGGATAAGGTCCTCTCCGCGTACAACAAGCACCGCGACAAGCCAATCAGGGAGCAGGCTGAGAATATCATTCATCTCTTCACCCTGACGAACCTCGGCGCCCCTGCTGCTTTCAAGTACTTCGACACAACTATCGATCGCAAGAGGTACACAAGCACTAAGGAGGTCCTGGACGCGACCCTCATCCACCAGTCGATTACCGGCCTCTACGAGACGCGCATCGACCTGTCTCAGCTCGGGGGCGACGAATTCTCCGGGAGCGAGACGCCAGGCACCTCCGAGTCGGCCACCCCAGAATCTGCCACAGTGGTGTCCGGCCAAAAGCAGGACCGCCAGGGCGGAGAACGCAGAAGGTCCCAGCTCGATAGGGATCAGTGTGCCTACTGCAAGGAGAAGGGCCACTGGGCCAAAGACTGCCCGAAAAAGCCGCGCGGCCCACGCGGCCCAAGGCCACAAACATCCCTCCTTCCAAAGAAGAAGCGGAAGGTGGAGCTCAGCGGAGGATCTTCCGGAGGATCTAGCGGCTCCGAGACACCAGGAACATCCGAAAGCGCTACACCAGAATCTAGCGGAGGCTCTTCCGGAGGATCTAGGCCTACCCTCAACATCGAGGATGAGTATCGCCTCCACGAAACCTCCAAAGAACCGGACGTGTCCCTCGGCAGCACATGGCTCAGCGACTTCCCACAAGCGTGGGCCGAAACCGGCGGCATGGGCCTCGCCGTCCGCCAAGCCCCACTCATTATCCCGCTGAAGGCGACCTCCACACCGGTGTCCATCAAGCAGTACCCGATGAGCCAAGAGGCGAGGCTCGGGATTAAGCCGCACATTCAGCGCCTCCTCGATCAAGGCATTCTCGTGCCGTGCCAATCCCCGTGGAATACACCACTCCTCCCGGTCAAAAAGCCGGGCACCAACGACTATCGCCCGGTCCAAGATCTCCGCGAGGTCAACAAGCGCGTGGAAGACATCCACCCGACCGTCCCGAACCCGTATAATCTGCTCTCCGGGCTCCCACCATCCCACCAGTGGTATACAGTGCTGGACCTCAAAGACGCCTTCTTCTGTCTCCGCCTCCACCCAACAAGCCAGCCGCTCTTCGCCTTCGAGTGGCGCGACCCGGAGATGGGCATCTCCGGCCAACTGACATGGACACGCCTCCCGCAAGGCTTCAAGAACAGCCCGACACTCTTCAACGAGGCGCTCCATAGGGACCTCGCGGATTTTCGCATCCAGCATCCGGACCTCATCCTCCTCCAGTATGCGGATGATCTCCTCCTCGCCGCGACCTCCGAGCTGGATTGTCAACAAGGCACACGCGCGCTCCTCCAAACACTCGGGAACCTCGGCTATCGCGCGTCCGCGAAAAAGGCCCAAATCTGCCAGAAGCAAGTGAAGTACCTCGGGTATCTGCTCAAGGAAGGCCAACGCTGGCTCACCGAAGCGCGCAAAGAAACAGTGATGGGGCAACCGACACCGAAAACACCACGCCAGCTGCGCGAGTTTCTCGGCAAAGCCGGCTTCTGTCGCCTCTTCATCCCGGGCTTTGCCGAGATGGCCGCGCCACTCTACCCACTCACCAAGCCGGGCACACTGTTTAACTGGGGGCCGGATCAGCAGAAAGCCTACCAAGAGATCAAACAAGCGCTCCTCACCGCCCCAGCGCTCGGGCTCCCAGATCTCACAAAGCCGTTCGAGCTGTTCGTCGATGAGAAGCAAGGCTACGCGAAGGGCGTGCTCACACAGAAGCTCGGCCCGTGGAGGAGGCCAGTGGCCTATCTCTCCAAAAAACTCGATCCAGTGGCCGCCGGCTGGCCACCGTGTCTGCGCATGGTCGCCGCGATTGCCGTGCTCACAAAGGATGCCGGCAAACTCACAATGGGCCAGCCGCTGGTGATCCTCGCGCCACATGCCGTGGAAGCCCTCGTCAAACAGCCGCCGGATAGGTGGCTCTCCAATGCGCGCATGACCCATTACCAAGCGCTCCTCCTCGACACCGATCGCGTCCAGTTCGGCCCAGTGGTCGCCCTCAATCCGGCGACACTGCTGCCACTCCCAGAGGAGGGCCTCCAACACAACTGTCTGGATATTCTCGCGGAAGCGCATGGCACAAGGCCAGACCTCACAGATCAACCGCTCAGCGGCGGCAGCAAAAGAACGGCGGACGGCTCTGAGAAGCGCACCGCTGATAGCCAGCATTCAACTCCTCCGAAAACAAAGAGGAAAGTTGAGTTCGAACCGAAGAAGAAAAGGAAGGTGTGAGAGCTCAGAGCTTTCGTTCGTATCATCGGTTTCGACAACGTTCGTCAAGTTCAATGCATCAGTTTCATTGCGCACACACCAGAATCCTACTGAGTTTGAGTATTATGGCATTGGGAAAACTGTTTTTCTTGTACCATTTGTTGTGCTTGTAATTTACTGTGTTTTTTATTCGGTTTTCGCTATCGAACTGTGAAATGGAAATGGATGGAGAAGAGTTAATGAATGATATGGTCCTTTTGTTCATTCTCAAATTAATATTATTTGTTTTTTCTCTTATTTGTTGTGTGTTGAATTTGAAATTATAAGAGATATGCAAACATTTTGTTTTGAGTAAAAATGTGTCAAATCGTGGCCTCTAATGACCGAAGTTAATATGAGGAGTAAAACACTTGTAGTTGTACCATTATGCTTATTCACTAGGCAACAAATATATTTTCAGACCTAGAAAAGCTGCAAATGTTACTGAATACAAGTATGTCCTCTTGTGTTTTAGACATTTATGAACTTTCCTTTATGTAATTTTCCAGAATCCTTGTCAGATTCTAATCATTGCTTTATAATTATAGTTATACTCATGGATTTGTAGTTGAGTATGAAAATATTTTTTAATGCATTTTATGACTTGCCAATTGATTGACAACGAATTCGTAATCATGTCATAGCTGTTTCCTGTGTGAAATTGTTATCCGCTCACAATTCCACACAACATACGAGCCGGAAGCATAAAGTGTAAAGCCTGGGGTGCCTAATGAGTGAGCTAACTCACATTAATTGCGTTGCGCTCACTGCCCGCTTTCCAGTCGGGAAACCTGTCGTGCCAGCTGCATTAATGAATCGGCCAACGCGCGGGGAGAGGCGGTTTGCGTATTGGCTAGAGCAGCTTGCCAACATGGTGGAGCACGACACTCTCGTCTACTCCAAGAATATCAAAGATACAGTCTCAGAAGACCAAAGGGCTATTGAGACTTTTCAACAAAGGGTAATATCGGGAAACCTCCTCGGATTCCATTGCCCAGCTATCTGTCACTTCATCAAAAGGACAGTAGAAAAGGAAGGTGGCACCTACAAATGCCATCATTGCGATAAAGGAAAGGCTATCGTTCAAGATGCCTCTGCCGACAGTGGTCCCAAAGATGGACCCCCACCCACGAGGAGCATCGTGGAAAAAGAAGACGTTCCAACCACGTCTTCAAAGCAAGTGGATTGATGTGAACATGGTGGAGCACGACACTCTCGTCTACTCCAAGAATATCAAAGATACAGTCTCAGAAGACCAAAGGGCTATTGAGACTTTTCAACAAAGGGTAATATCGGGAAACCTCCTCGGATTCCATTGCCCAGCTATCTGTCACTTCATCAAAAGGACAGTAGAAAAGGAAGGTGGCACCTACAAATGCCATCATTGCGATAAAGGAAAGGCTATCGTTCAAGATGCCTCTGCCGACAGTGGTCCCAAAGATGGACCCCCACCCACGAGGAGCATCGTGGAAAAAGAAGACGTTCCAACCACGTCTTCAAAGCAAGTGGATTGATGTGATATCTCCACTGACGTAAGGGATGACGCACAATCCCACTATCCTTCGCAAGACCCTTCCTCTATATAAGGAAGTTCATTTCATTTGGAGAGGACACGCTGAAATCACCAGTCTCTCTCTACAAATCTATCTCTCTCGAGCTTTCGCAGATCCGGGGGGCAATGAGATATGAAAAAGCCTGAACTCACCGCGACGTCTGTCGAGAAGTTTCTGATCGAAAAGTTCGACAGCGTCTCCGACCTGATGCAGCTCTCGGAGGGCGAAGAATCTCGTGCTTTCAGCTTCGATGTAGGAGGGCGTGGATATGTCCTGCGGGTAAATAGCTGCGCCGATGGTTTCTACAAAGATCGTTATGTTTATCGGCACTTTGCATCGGCCGCGCTCCCGATTCCGGAAGTGCTTGACATTGGGGAGTTTAGCGAGAGCCTGACCTATTGCATCTCCCGCCGTTCACAGGGTGTCACGTTGCAAGACCTGCCTGAAACCGAACTGCCCGCTGTTCTACAACCGGTCGCGGAGGCTATGGATGCGATCGCTGCGGCCGATCTTAGCCAGACGAGCGGGTTCGGCCCATTCGGACCGCAAGGAATCGGTCAATACACTACATGGCGTGATTTCATATGCGCGATTGCTGATCCCCATGTGTATCACTGGCAAACTGTGATGGACGACACCGTCAGTGCGTCCGTCGCGCAGGCTCTCGATGAGCTGATGCTTTGGGCCGAGGACTGCCCCGAAGTCCGGCACCTCGTGCACGCGGATTTCGGCTCCAACAATGTCCTGACGGACAATGGCCGCATAACAGCGGTCATTGACTGGAGCGAGGCGATGTTCGGGGATTCCCAATACGAGGTCGCCAACATCTTCTTCTGGAGGCCGTGGTTGGCTTGTATGGAGCAGCAGACGCGCTACTTCGAGCGGAGGCATCCGGAGCTTGCAGGATCGCCACGACTCCGGGCGTATATGCTCCGCATTGGTCTTGACCAACTCTATCAGAGCTTGGTTGACGGCAATTTCGATGATGCAGCTTGGGCGCAGGGTCGATGCGACGCAATCGTCCGATCCGGAGCCGGGACTGTCGGGCGTACACAAATCGCCCGCAGAAGCGCGGCCGTCTGGACCGATGGCTGTGTAGAAGTACTCGCCGATAGTGGAAACCGACGCCCCAGCACTCGTCCGAGGGCAAAGAAATAGAGTAGATGCCGACCGGGATCTGTCGATCGACAAGCTCGAGTTTCTCCATAATAATGTGTGAGTAGTTCCCAGATAAGGGAATTAGGGTTCCTATAGGGTTTCGCTCATGTGTTGAGCATATAAGAAACCCTTAGTATGTATTTGTATTTGTAAAATACTTCTATCAATAAAATTTCTAATTCCTAAAACCAAAATCCAGTACTAAAATCCAGATCCCCCGAATTAATTCGGCGTTAATTCAGTACATTAAAAACGTCCGCAATGTGTTATTAAGTTGTCTAAGCGTCAATTTGTTTACACCACAATATATCCTGCCA

**Sequence 79 Plasmids sequence of pH-35C-M-MLV-OsGAPDH**

(RB T-DNA repeat-35C promoter-tRNAGly RS-pegRNA-Ubi promoter-NLSc-myc-NLSsv40-nCas9(H840A/ R221K/N394K)-XTEN-NC-NLS-32aa Linker-M-MLV-RT-NLSvbp-HygR cassette-E9-terminator-CaMV poly(A) signal-LB T-DNA repeat)

GTTTACCCGCCAATATATCCTGTCAAACACTGATAGTTTAAACTGAAGGCGGGAAACGACAATCTGATCCAAGCTCAAGCTGCTCTAGCATTCGCCATTCAGGCTGCGCAACTGTTGGGAAGGGCGATCGGTGCGGGCCTCTTCGCTATTACGCCAGCTGGCGAAAGGGGGATGTGCTGCAAGGCGATTAAGTTGGGTAACGCCAGGGTTTTCCCAGTCACGACGTTGTAAAACGACGGCCAGTGAATTCATGGAGTCAAAGATTCAAATAGAGGACCTAACAGAACTCGCCGTAAAGACTGGCGAACAGTTCATACAGAGTCTCTTACGACTCAATGACAAGAAGAAAATCTTCGTCAACATGGTGGAGCACGACACACTTGTCTACTCCAAAAATATCAAAGATACAGTCTCAGAAGACCAAAGGGCAATTGAGACTTTTCAACAAAGGGTAATATCCGGAAACCTCCTCGGATTCCATTGCCCAGCTATCTGTCACTTTATTGTGAAGATAGTGGAAAAGGAAGGTGGCTCCTACAAATGCCATCATTGCGATAAAGGAAAGGCCATCGTTGAAGATGCCTCTGCCGACAGTGGTCCCAAAGATGGACCCCCACCCACGAGGAGCATCGTGGAAAAAGAAGACGTTCCAACCACGTCTTCAAAGCAAGTGGATTGATGTGATTGGCAGACATACTGTCCCACAAATGAAGATGGAATCTGTAAAAGAAAACGCGTGAAATAATGCGTCTGACAAAGGTTAGGTCGGCTGCCTTTAATCAATACCAAAGTGGTCCCTACCACGATGGAAAAACTGTGCAGTCGGTTTGGCTTTTTCTGACGAACAAATAAGATTCGTGGCCGACAGGTGGGGGTCCACCATGTGAAGGCATCTTCAGACTCCAATAATGGAGCAATGACGTAAGGGCTTACGAAATAAGTAAGGGTAGTTTGGGAAATGTCCACTCACCCGTCAGTCTATAAATACTTAGCCCCTCCCTCATTGTTAAGGGAGCAAAATCTCAGAGAGATAGTCCTAGAGAGAGAAAGAGAGCAAGTAGCCTAGAAGTAGTCAAGGCGGCGAAGTATTCAGGCACGTGGCCAGGAAGAAGAAAAGCCAAGACGACGAAAACAGGTAAGAGCTAAGCATCTAGATAAGTTGAAAACAATCTTCAAAAGTCCCACATCGCTTAGATAAGAAAACGAAGCTGAGTTTATATACAGCTAGAGTCGAAGTAGTGATTGAACAAAGCACCAGTGGTCTAGTGGTAGAATAGTACCCTGCCACGGTACAGACCCGGGTTCGATTCCCGGCTGGTGCAGAGTATGTCGTGGAGTCCACGTTTAAGAGCTATGCTGGAAACAGCATAGCAAGTTTAAATAAGGCTAGTCCGTTATCAACTTGAAAAAGTGGCACCGAGTCGGTGCAGTGAAGACACCGTTGGACTCCACGCGCGGTTCTATCTAGTTACGCGTTAAACCAACTAGAATTTTTTTGGCCGGCATGGTCCCAGCCTCCTCGCTGGCGCCGGCTGGGCAACATGCTTCGGCATGGCGAATGGGACTTTTTTTTGATATCTCCGGGGCTAATTGAATATGAAGATGAAGATGAAATATTTGGTGTGTCAAATAAAAAGCTGGTGTGCTTAAGTTTGTGTTTTTTTCTTGGCTTGTTGTGTTATGAATTTGTGGCTTTTTCTAATATTAAATGAATGTAAGATCTCATTATAATGAATAAACAAATGTTTCTATAATCCATTGTGAATGTTTTGTTGGATCTCTTCTGCAGCATATAACTACTGTATGTGCTATGGTATGGACTATGGAATATGATTAAAGATAAGAAGCTTGGCGTAATCTGCATGCCTGCAGTGCAGCGTGACCCGGTCGTGCCCCTCTCTAGAGATAATGAGCATTGCATGTCTAAGTTATAAAAAATTACCACATATTTTTTTTGTCACACTTGTTTGAAGTGCAGTTTATCTATCTTTATACATATATTTAAACTTTACTCTACGAATAATATAATCTATAGTACTACAATAATATCAGTGTTTTAGAGAATCATATAAATGAACAGTTAGACATGGTCTAAAGGACAATTGAGTATTTTGACAACAGGACTCTACAGTTTTATCTTTTTAGTGTGCATGTGTTCTCCTTTTTTTTTGCAAATAGCTTCACCTATATAATACTTCATCCATTTTATTAGTACATCCATTTAGGGTTTAGGGTTAATGGTTTTTATAGACTAATTTTTTTAGTACATCTATTTTATTCTATTTTAGCCTCTAAATTAAGAAAACTAAAACTCTATTTTAGTTTTTTTATTTAATAATTTAGATATAAAATAGAATAAAATAAAGTGACTAAAAATTAAACAAATACCCTTTAAGAAATTAAAAAAACTAAGGAAACATTTTTCTTGTTTCGAGTAGATAATGCCAGCCTGTTAAACGCCGTCGACGAGTCTAACGGACACCAACCAGCGAACCAGCAGCGTCGCGTCGGGCCAAGCGAAGCAGACGGCACGGCATCTCTGTCGCTGCCTCTGGACCCCTCTCGAGAGTTCCGCTCCACCGTTGGACTTGCTCCGCTGTCGGCATCCAGAAATTGCGTGGCGGAGCGGCAGACGTGAGCCGGCACGGCAGGCGGCCTCCTCCTCCTCTCACGGCACCGGCAGCTACGGGGGATTCCTTTCCCACCGCTCCTTCGCTTTCCCTTCCTCGCCCGCCGTAATAAATAGACACCCCCTCCACACCCTCTTTCCCCAACCTCGTGTTGTTCGGAGCGCACACACACACAACCAGATCTCCCCCAAATCCACCCGTCGGCACCTCCGCTTCAAGGTACGCCGCTCGTCCTCCCCCCCCCCCCCTCTCTACCTTCTCTAGATCGGCGTTCCGGTCCATGGTTAGGGCCCGGTAGTTCTACTTCTGTTCATGTTTGTGTTAGATCCGTGTTTGTGTTAGATCCGTGCTGCTAGCGTTCGTACACGGATGCGACCTGTACGTCAGACACGTTCTGATTGCTAACTTGCCAGTGTTTCTCTTTGGGGAATCCTGGGATGGCTCTAGCCGTTCCGCAGACGGGATCGATTTCATGATTTTTTTTGTTTCGTTGCATAGGGTTTGGTTTGCCCTTTTCCTTTATTTCAATATATGCCGTGCACTTGTTTGTCGGGTCATCTTTTCATGCTTTTTTTTGTCTTGGTTGTGATGATGTGGTCTGGTTGGGCGGTCGTTCTAGATCGGAGTAGAATTCTGTTTCAAACTACCTGGTGGATTTATTAATTTTGGATCTGTATGTGTGTGCCATACATATTCATAGTTACGAATTGAAGATGATGGATGGAAATATCGATCTAGGATAGGTATACATGTTGATGCGGGTTTTACTGATGCATATACAGAGATGCTTTTTGTTCGCTTGGTTGTGATGATGTGGTGTGGTTGGGCGGTCGTTCATTCGTTCTAGATCGGAGTAGAATACTGTTTCAAACTACCTGGTGTATTTATTAATTTTGGAACTGTATGTGTGTGTCATACATCTTCATAGTTACGAGTTTAAGATGGATGGAAATATCGATCTAGGATAGGTATACATGTTGATGTGGGTTTTACTGATGCATATACATGATGGCATATGCAGCATCTATTCATATGCTCTAACCTTGAGTACCTATCTATTATAATAAACAAGTATGTTTTATAATTATTTTGATCTTGATATACTTGGATGATGGCATATGCAGCAGCTATATGTGGATTTTTTTAGCCCTGCCTTCATACGCTATTTATTTGCTTGGTACTGTTTCTTTTGTCGATGCTCACCCTGTTGTTTGGTGTTACTTCTGCAGCCCTAGGACTGGTTGTACAATGCCGGCGGCCAAGAGAGTCAAGCTCGACGGCGGGAAGCGCACAGCTGATGGTTCTGAATTCGAGTCCCCTAAGAAAAAGAGAAAAGTGGACAAGAAGTACTCGATCGGCCTCGATATTGGGACTAACTCTGTTGGCTGGGCCGTGATCACCGACGAGTACAAGGTGCCCTCAAAGAAGTTCAAGGTCCTGGGCAACACCGATCGGCATTCCATCAAGAAGAATCTCATTGGCGCTCTCCTGTTCGACAGCGGCGAGACGGCTGAGGCTACGCGGCTCAAGCGCACCGCCCGCAGGCGGTACACGCGCAGGAAGAATCGCATCTGCTACCTGCAGGAGATTTTCTCCAACGAGATGGCGAAGGTTGACGATTCTTTCTTCCACAGGCTGGAGGAGTCATTCCTCGTGGAGGAGGATAAGAAGCACGAGCGGCATCCAATCTTCGGCAACATTGTCGACGAGGTTGCCTACCACGAGAAGTACCCTACGATCTACCATCTGCGGAAGAAGCTCGTGGACTCCACAGATAAGGCGGACCTCCGCCTGATCTACCTCGCTCTGGCCCACATGATTAAGTTCAGGGGCCATTTCCTGATCGAGGGGGATCTCAACCCGGACAATAGCGATGTTGACAAGCTGTTCATCCAGCTCGTGCAGACGTACAACCAGCTCTTCGAGGAGAACCCCATTAATGCGTCAGGCGTCGACGCGAAGGCTATCCTGTCCGCTAGGCTCTCGAAGTCTCGGAAGCTCGAGAACCTGATCGCCCAGCTGCCGGGCGAGAAGAAGAACGGCCTGTTCGGGAATCTCATTGCGCTCAGCCTGGGGCTCACGCCCAACTTCAAGTCGAATTTCGATCTCGCTGAGGACGCCAAGCTGCAGCTCTCCAAGGACACATACGACGATGACCTGGATAACCTCCTGGCCCAGATCGGCGATCAGTACGCGGACCTGTTCCTCGCTGCCAAGAATCTGTCGGACGCCATCCTCCTGTCTGATATTCTCAGGGTGAACACCGAGATTACGAAGGCTCCGCTCTCAGCCTCCATGATCAAGCGCTACGACGAGCACCATCAGGATCTGACCCTCCTGAAGGCGCTGGTCAGGCAGCAGCTCCCCGAGAAGTACAAGGAGATCTTCTTCGATCAGTCGAAGAACGGCTACGCTGGGTACATTGACGGCGGGGCCTCTCAGGAGGAGTTCTACAAGTTCATCAAGCCGATTCTGGAGAAGATGGACGGCACGGAGGAGCTGCTGGTGAAGCTCAAGCGCGAGGACCTCCTGAGGAAGCAGCGGACATTCGATAACGGCAGCATCCCACACCAGATTCATCTCGGGGAGCTGCACGCTATCCTGAGGAGGCAGGAGGACTTCTACCCTTTCCTCAAGGATAACCGCGAGAAGATCGAGAAGATTCTGACTTTCAGGATCCCGTACTACGTCGGCCCACTCGCTAGGGGCAACTCCCGCTTCGCTTGGATGACCCGCAAGTCAGAGGAGACGATCACGCCGTGGAACTTCGAGGAGGTGGTCGACAAGGGCGCTAGCGCTCAGTCGTTCATCGAGAGGATGACGAATTTCGACAAGAACCTGCCAAATGAGAAGGTGCTCCCTAAGCACTCGCTCCTGTACGAGTACTTCACAGTCTACAACGAGCTGACTAAGGTGAAGTATGTGACCGAGGGCATGAGGAAGCCGGCTTTCCTGTCTGGGGAGCAGAAGAAGGCCATCGTGGACCTCCTGTTCAAGACCAACCGGAAGGTCACGGTTAAGCAGCTCAAGGAGGACTACTTCAAGAAGATTGAGTGCTTCGATTCGGTCGAGATCTCTGGCGTTGAGGACCGCTTCAACGCCTCCCTGGGGACCTACCACGATCTCCTGAAGATCATTAAGGATAAGGACTTCCTGGACAACGAGGAGAATGAGGATATCCTCGAGGACATTGTGCTGACACTCACTCTGTTCGAGGACCGGGAGATGATCGAGGAGCGCCTGAAGACTTACGCCCATCTCTTCGATGACAAGGTCATGAAGCAGCTCAAGAGGAGGAGGTACACCGGCTGGGGGAGGCTGAGCAGGAAGCTCATCAACGGCATTCGGGACAAGCAGTCCGGGAAGACGATCCTCGACTTCCTGAAGAGCGATGGCTTCGCGAACCGCAATTTCATGCAGCTGATTCACGATGACAGCCTCACATTCAAGGAGGATATCCAGAAGGCTCAGGTGAGCGGCCAGGGGGACTCGCTGCACGAGCATATCGCGAACCTCGCTGGCTCGCCAGCTATCAAGAAGGGGATTCTGCAGACCGTGAAGGTTGTGGACGAGCTGGTGAAGGTCATGGGCAGGCACAAGCCTGAGAACATCGTCATTGAGATGGCCCGGGAGAATCAGACCACGCAGAAGGGCCAGAAGAACTCACGCGAGAGGATGAAGAGGATCGAGGAGGGCATTAAGGAGCTGGGGTCCCAGATCCTCAAGGAGCACCCGGTGGAGAACACGCAGCTGCAGAATGAGAAGCTCTACCTGTACTACCTCCAGAATGGCCGCGATATGTATGTGGACCAGGAGCTGGATATTAACAGGCTCAGCGATTACGACGTCGATGCCATCGTTCCACAGTCATTCCTGAAGGATGACTCCATTGACAACAAGGTCCTCACCAGGTCGGACAAGAACCGGGGCAAGTCTGATAATGTTCCTTCAGAGGAGGTCGTTAAGAAGATGAAGAACTACTGGCGCCAGCTCCTGAATGCCAAGCTGATCACGCAGCGGAAGTTCGATAACCTCACAAAGGCTGAGAGGGGCGGGCTCTCTGAGCTGGACAAGGCGGGCTTCATCAAGAGGCAGCTGGTCGAGACACGGCAGATCACTAAGCACGTTGCGCAGATTCTCGACTCACGGATGAACACTAAGTACGATGAGAATGACAAGCTGATCCGCGAGGTGAAGGTCATCACCCTGAAGTCAAAGCTCGTCTCCGACTTCAGGAAGGATTTCCAGTTCTACAAGGTTCGGGAGATCAACAATTACCACCATGCCCATGACGCGTACCTGAACGCGGTGGTCGGCACAGCTCTGATCAAGAAGTACCCAAAGCTCGAGAGCGAGTTCGTGTACGGGGACTACAAGGTTTACGATGTGAGGAAGATGATCGCCAAGTCGGAGCAGGAGATTGGCAAGGCTACCGCCAAGTACTTCTTCTACTCTAACATTATGAATTTCTTCAAGACAGAGATCACTCTGGCCAATGGCGAGATCCGGAAGCGCCCCCTCATCGAGACGAACGGCGAGACGGGGGAGATCGTGTGGGACAAGGGCAGGGATTTCGCGACCGTCAGGAAGGTTCTCTCCATGCCACAAGTGAATATCGTCAAGAAGACAGAGGTCCAGACTGGCGGGTTCTCTAAGGAGTCAATTCTGCCTAAGCGGAACAGCGACAAGCTCATCGCCCGCAAGAAGGACTGGGATCCGAAGAAGTACGGCGGGTTCGACAGCCCCACTGTGGCCTACTCGGTCCTGGTTGTGGCGAAGGTTGAGAAGGGCAAGTCCAAGAAGCTCAAGAGCGTGAAGGAGCTGCTGGGGATCACGATTATGGAGCGCTCCAGCTTCGAGAAGAACCCGATCGATTTCCTGGAGGCGAAGGGCTACAAGGAGGTGAAGAAGGACCTGATCATTAAGCTCCCCAAGTACTCACTCTTCGAGCTGGAGAACGGCAGGAAGCGGATGCTGGCTTCCGCTGGCGAGCTGCAGAAGGGGAACGAGCTGGCTCTGCCGTCCAAGTATGTGAACTTCCTCTACCTGGCCTCCCACTACGAGAAGCTCAAGGGCAGCCCCGAGGACAACGAGCAGAAGCAGCTGTTCGTCGAGCAGCACAAGCATTACCTCGACGAGATCATTGAGCAGATTTCCGAGTTCTCCAAGCGCGTGATCCTGGCCGACGCGAATCTGGATAAGGTCCTCTCCGCGTACAACAAGCACCGCGACAAGCCAATCAGGGAGCAGGCTGAGAATATCATTCATCTCTTCACCCTGACGAACCTCGGCGCCCCTGCTGCTTTCAAGTACTTCGACACAACTATCGATCGCAAGAGGTACACAAGCACTAAGGAGGTCCTGGACGCGACCCTCATCCACCAGTCGATTACCGGCCTCTACGAGACGCGCATCGACCTGTCTCAGCTCGGGGGCGACGAATTCTCCGGGAGCGAGACGCCAGGCACCTCCGAGTCGGCCACCCCAGAATCTGCCACAGTGGTGTCCGGCCAAAAGCAGGACCGCCAGGGCGGAGAACGCAGAAGGTCCCAGCTCGATAGGGATCAGTGTGCCTACTGCAAGGAGAAGGGCCACTGGGCCAAAGACTGCCCGAAAAAGCCGCGCGGCCCACGCGGCCCAAGGCCACAAACATCCCTCCTTCCAAAGAAGAAGCGGAAGGTGGAGCTCAGCGGAGGATCTTCCGGAGGATCTAGCGGCTCCGAGACACCAGGAACATCCGAAAGCGCTACACCAGAATCTAGCGGAGGCTCTTCCGGAGGATCTAGGCCTACCCTCAACATCGAGGATGAGTATCGCCTCCACGAAACCTCCAAAGAACCGGACGTGTCCCTCGGCAGCACATGGCTCAGCGACTTCCCACAAGCGTGGGCCGAAACCGGCGGCATGGGCCTCGCCGTCCGCCAAGCCCCACTCATTATCCCGCTGAAGGCGACCTCCACACCGGTGTCCATCAAGCAGTACCCGATGAGCCAAGAGGCGAGGCTCGGGATTAAGCCGCACATTCAGCGCCTCCTCGATCAAGGCATTCTCGTGCCGTGCCAATCCCCGTGGAATACACCACTCCTCCCGGTCAAAAAGCCGGGCACCAACGACTATCGCCCGGTCCAAGATCTCCGCGAGGTCAACAAGCGCGTGGAAGACATCCACCCGACCGTCCCGAACCCGTATAATCTGCTCTCCGGGCTCCCACCATCCCACCAGTGGTATACAGTGCTGGACCTCAAAGACGCCTTCTTCTGTCTCCGCCTCCACCCAACAAGCCAGCCGCTCTTCGCCTTCGAGTGGCGCGACCCGGAGATGGGCATCTCCGGCCAACTGACATGGACACGCCTCCCGCAAGGCTTCAAGAACAGCCCGACACTCTTCAACGAGGCGCTCCATAGGGACCTCGCGGATTTTCGCATCCAGCATCCGGACCTCATCCTCCTCCAGTATGCGGATGATCTCCTCCTCGCCGCGACCTCCGAGCTGGATTGTCAACAAGGCACACGCGCGCTCCTCCAAACACTCGGGAACCTCGGCTATCGCGCGTCCGCGAAAAAGGCCCAAATCTGCCAGAAGCAAGTGAAGTACCTCGGGTATCTGCTCAAGGAAGGCCAACGCTGGCTCACCGAAGCGCGCAAAGAAACAGTGATGGGGCAACCGACACCGAAAACACCACGCCAGCTGCGCGAGTTTCTCGGCAAAGCCGGCTTCTGTCGCCTCTTCATCCCGGGCTTTGCCGAGATGGCCGCGCCACTCTACCCACTCACCAAGCCGGGCACACTGTTTAACTGGGGGCCGGATCAGCAGAAAGCCTACCAAGAGATCAAACAAGCGCTCCTCACCGCCCCAGCGCTCGGGCTCCCAGATCTCACAAAGCCGTTCGAGCTGTTCGTCGATGAGAAGCAAGGCTACGCGAAGGGCGTGCTCACACAGAAGCTCGGCCCGTGGAGGAGGCCAGTGGCCTATCTCTCCAAAAAACTCGATCCAGTGGCCGCCGGCTGGCCACCGTGTCTGCGCATGGTCGCCGCGATTGCCGTGCTCACAAAGGATGCCGGCAAACTCACAATGGGCCAGCCGCTGGTGATCCTCGCGCCACATGCCGTGGAAGCCCTCGTCAAACAGCCGCCGGATAGGTGGCTCTCCAATGCGCGCATGACCCATTACCAAGCGCTCCTCCTCGACACCGATCGCGTCCAGTTCGGCCCAGTGGTCGCCCTCAATCCGGCGACACTGCTGCCACTCCCAGAGGAGGGCCTCCAACACAACTGTCTGGATATTCTCGCGGAAGCGCATGGCACAAGGCCAGACCTCACAGATCAACCGCTCAGCGGCGGCAGCAAAAGAACGGCGGACGGCTCTGAGAAGCGCACCGCTGATAGCCAGCATTCAACTCCTCCGAAAACAAAGAGGAAAGTTGAGTTCGAACCGAAGAAGAAAAGGAAGGTGTGAGAGCTCAGAGCTTTCGTTCGTATCATCGGTTTCGACAACGTTCGTCAAGTTCAATGCATCAGTTTCATTGCGCACACACCAGAATCCTACTGAGTTTGAGTATTATGGCATTGGGAAAACTGTTTTTCTTGTACCATTTGTTGTGCTTGTAATTTACTGTGTTTTTTATTCGGTTTTCGCTATCGAACTGTGAAATGGAAATGGATGGAGAAGAGTTAATGAATGATATGGTCCTTTTGTTCATTCTCAAATTAATATTATTTGTTTTTTCTCTTATTTGTTGTGTGTTGAATTTGAAATTATAAGAGATATGCAAACATTTTGTTTTGAGTAAAAATGTGTCAAATCGTGGCCTCTAATGACCGAAGTTAATATGAGGAGTAAAACACTTGTAGTTGTACCATTATGCTTATTCACTAGGCAACAAATATATTTTCAGACCTAGAAAAGCTGCAAATGTTACTGAATACAAGTATGTCCTCTTGTGTTTTAGACATTTATGAACTTTCCTTTATGTAATTTTCCAGAATCCTTGTCAGATTCTAATCATTGCTTTATAATTATAGTTATACTCATGGATTTGTAGTTGAGTATGAAAATATTTTTTAATGCATTTTATGACTTGCCAATTGATTGACAACGAATTCGTAATCATGTCATAGCTGTTTCCTGTGTGAAATTGTTATCCGCTCACAATTCCACACAACATACGAGCCGGAAGCATAAAGTGTAAAGCCTGGGGTGCCTAATGAGTGAGCTAACTCACATTAATTGCGTTGCGCTCACTGCCCGCTTTCCAGTCGGGAAACCTGTCGTGCCAGCTGCATTAATGAATCGGCCAACGCGCGGGGAGAGGCGGTTTGCGTATTGGCTAGAGCAGCTTGCCAACATGGTGGAGCACGACACTCTCGTCTACTCCAAGAATATCAAAGATACAGTCTCAGAAGACCAAAGGGCTATTGAGACTTTTCAACAAAGGGTAATATCGGGAAACCTCCTCGGATTCCATTGCCCAGCTATCTGTCACTTCATCAAAAGGACAGTAGAAAAGGAAGGTGGCACCTACAAATGCCATCATTGCGATAAAGGAAAGGCTATCGTTCAAGATGCCTCTGCCGACAGTGGTCCCAAAGATGGACCCCCACCCACGAGGAGCATCGTGGAAAAAGAAGACGTTCCAACCACGTCTTCAAAGCAAGTGGATTGATGTGAACATGGTGGAGCACGACACTCTCGTCTACTCCAAGAATATCAAAGATACAGTCTCAGAAGACCAAAGGGCTATTGAGACTTTTCAACAAAGGGTAATATCGGGAAACCTCCTCGGATTCCATTGCCCAGCTATCTGTCACTTCATCAAAAGGACAGTAGAAAAGGAAGGTGGCACCTACAAATGCCATCATTGCGATAAAGGAAAGGCTATCGTTCAAGATGCCTCTGCCGACAGTGGTCCCAAAGATGGACCCCCACCCACGAGGAGCATCGTGGAAAAAGAAGACGTTCCAACCACGTCTTCAAAGCAAGTGGATTGATGTGATATCTCCACTGACGTAAGGGATGACGCACAATCCCACTATCCTTCGCAAGACCCTTCCTCTATATAAGGAAGTTCATTTCATTTGGAGAGGACACGCTGAAATCACCAGTCTCTCTCTACAAATCTATCTCTCTCGAGCTTTCGCAGATCCGGGGGGCAATGAGATATGAAAAAGCCTGAACTCACCGCGACGTCTGTCGAGAAGTTTCTGATCGAAAAGTTCGACAGCGTCTCCGACCTGATGCAGCTCTCGGAGGGCGAAGAATCTCGTGCTTTCAGCTTCGATGTAGGAGGGCGTGGATATGTCCTGCGGGTAAATAGCTGCGCCGATGGTTTCTACAAAGATCGTTATGTTTATCGGCACTTTGCATCGGCCGCGCTCCCGATTCCGGAAGTGCTTGACATTGGGGAGTTTAGCGAGAGCCTGACCTATTGCATCTCCCGCCGTTCACAGGGTGTCACGTTGCAAGACCTGCCTGAAACCGAACTGCCCGCTGTTCTACAACCGGTCGCGGAGGCTATGGATGCGATCGCTGCGGCCGATCTTAGCCAGACGAGCGGGTTCGGCCCATTCGGACCGCAAGGAATCGGTCAATACACTACATGGCGTGATTTCATATGCGCGATTGCTGATCCCCATGTGTATCACTGGCAAACTGTGATGGACGACACCGTCAGTGCGTCCGTCGCGCAGGCTCTCGATGAGCTGATGCTTTGGGCCGAGGACTGCCCCGAAGTCCGGCACCTCGTGCACGCGGATTTCGGCTCCAACAATGTCCTGACGGACAATGGCCGCATAACAGCGGTCATTGACTGGAGCGAGGCGATGTTCGGGGATTCCCAATACGAGGTCGCCAACATCTTCTTCTGGAGGCCGTGGTTGGCTTGTATGGAGCAGCAGACGCGCTACTTCGAGCGGAGGCATCCGGAGCTTGCAGGATCGCCACGACTCCGGGCGTATATGCTCCGCATTGGTCTTGACCAACTCTATCAGAGCTTGGTTGACGGCAATTTCGATGATGCAGCTTGGGCGCAGGGTCGATGCGACGCAATCGTCCGATCCGGAGCCGGGACTGTCGGGCGTACACAAATCGCCCGCAGAAGCGCGGCCGTCTGGACCGATGGCTGTGTAGAAGTACTCGCCGATAGTGGAAACCGACGCCCCAGCACTCGTCCGAGGGCAAAGAAATAGAGTAGATGCCGACCGGGATCTGTCGATCGACAAGCTCGAGTTTCTCCATAATAATGTGTGAGTAGTTCCCAGATAAGGGAATTAGGGTTCCTATAGGGTTTCGCTCATGTGTTGAGCATATAAGAAACCCTTAGTATGTATTTGTATTTGTAAAATACTTCTATCAATAAAATTTCTAATTCCTAAAACCAAAATCCAGTACTAAAATCCAGATCCCCCGAATTAATTCGGCGTTAATTCAGTACATTAAAAACGTCCGCAATGTGTTATTAAGTTGTCTAAGCGTCAATTTGTTTACACCACAATATATCCTGCCA

**Sequence 80 Plasmids sequence of pH-35C-M-MLV-OsACC1**

(RB T-DNA repeat-35C promoter-tRNAGly RS-pegRNA-Ubi promoter-NLSc-myc-NLSsv40-nCas9(H840A/ R221K/N394K)-XTEN-NC-NLS-32aa Linker-M-MLV-RT-NLSvbp-HygR cassette-E9-terminator-CaMV poly(A) signal-LB T-DNA repeat)

GTTTACCCGCCAATATATCCTGTCAAACACTGATAGTTTAAACTGAAGGCGGGAAACGACAATCTGATCCAAGCTCAAGCTGCTCTAGCATTCGCCATTCAGGCTGCGCAACTGTTGGGAAGGGCGATCGGTGCGGGCCTCTTCGCTATTACGCCAGCTGGCGAAAGGGGGATGTGCTGCAAGGCGATTAAGTTGGGTAACGCCAGGGTTTTCCCAGTCACGACGTTGTAAAACGACGGCCAGTGAATTCATGGAGTCAAAGATTCAAATAGAGGACCTAACAGAACTCGCCGTAAAGACTGGCGAACAGTTCATACAGAGTCTCTTACGACTCAATGACAAGAAGAAAATCTTCGTCAACATGGTGGAGCACGACACACTTGTCTACTCCAAAAATATCAAAGATACAGTCTCAGAAGACCAAAGGGCAATTGAGACTTTTCAACAAAGGGTAATATCCGGAAACCTCCTCGGATTCCATTGCCCAGCTATCTGTCACTTTATTGTGAAGATAGTGGAAAAGGAAGGTGGCTCCTACAAATGCCATCATTGCGATAAAGGAAAGGCCATCGTTGAAGATGCCTCTGCCGACAGTGGTCCCAAAGATGGACCCCCACCCACGAGGAGCATCGTGGAAAAAGAAGACGTTCCAACCACGTCTTCAAAGCAAGTGGATTGATGTGATTGGCAGACATACTGTCCCACAAATGAAGATGGAATCTGTAAAAGAAAACGCGTGAAATAATGCGTCTGACAAAGGTTAGGTCGGCTGCCTTTAATCAATACCAAAGTGGTCCCTACCACGATGGAAAAACTGTGCAGTCGGTTTGGCTTTTTCTGACGAACAAATAAGATTCGTGGCCGACAGGTGGGGGTCCACCATGTGAAGGCATCTTCAGACTCCAATAATGGAGCAATGACGTAAGGGCTTACGAAATAAGTAAGGGTAGTTTGGGAAATGTCCACTCACCCGTCAGTCTATAAATACTTAGCCCCTCCCTCATTGTTAAGGGAGCAAAATCTCAGAGAGATAGTCCTAGAGAGAGAAAGAGAGCAAGTAGCCTAGAAGTAGTCAAGGCGGCGAAGTATTCAGGCACGTGGCCAGGAAGAAGAAAAGCCAAGACGACGAAAACAGGTAAGAGCTAAGCATCTAGATAAGTTGAAAACAATCTTCAAAAGTCCCACATCGCTTAGATAAGAAAACGAAGCTGAGTTTATATACAGCTAGAGTCGAAGTAGTGATTGAACAAAGCACCAGTGGTCTAGTGGTAGAATAGTACCCTGCCACGGTACAGACCCGGGTTCGATTCCCGGCTGGTGCATTCCTCGTGCTGGACAAGTGGTTTAAGAGCTATGCTGGAAACAGCATAGCAAGTTTAAATAAGGCTAGTCCGTTATCAACTTGAAAAAGTGGCACCGAGTCGGTGCATCTGGAAAGCACACTTGTCCAGCACGCGGTTCTATCTAGTTACGCGTTAAACCAACTAGAATTTTTTTGGCCGGCATGGTCCCAGCCTCCTCGCTGGCGCCGGCTGGGCAACATGCTTCGGCATGGCGAATGGGACTTTTTTTTGATATCTCCGGGGCTAATTGAATATGAAGATGAAGATGAAATATTTGGTGTGTCAAATAAAAAGCTGGTGTGCTTAAGTTTGTGTTTTTTTCTTGGCTTGTTGTGTTATGAATTTGTGGCTTTTTCTAATATTAAATGAATGTAAGATCTCATTATAATGAATAAACAAATGTTTCTATAATCCATTGTGAATGTTTTGTTGGATCTCTTCTGCAGCATATAACTACTGTATGTGCTATGGTATGGACTATGGAATATGATTAAAGATAAGAAGCTTGGCGTAATCTGCATGCCTGCAGTGCAGCGTGACCCGGTCGTGCCCCTCTCTAGAGATAATGAGCATTGCATGTCTAAGTTATAAAAAATTACCACATATTTTTTTTGTCACACTTGTTTGAAGTGCAGTTTATCTATCTTTATACATATATTTAAACTTTACTCTACGAATAATATAATCTATAGTACTACAATAATATCAGTGTTTTAGAGAATCATATAAATGAACAGTTAGACATGGTCTAAAGGACAATTGAGTATTTTGACAACAGGACTCTACAGTTTTATCTTTTTAGTGTGCATGTGTTCTCCTTTTTTTTTGCAAATAGCTTCACCTATATAATACTTCATCCATTTTATTAGTACATCCATTTAGGGTTTAGGGTTAATGGTTTTTATAGACTAATTTTTTTAGTACATCTATTTTATTCTATTTTAGCCTCTAAATTAAGAAAACTAAAACTCTATTTTAGTTTTTTTATTTAATAATTTAGATATAAAATAGAATAAAATAAAGTGACTAAAAATTAAACAAATACCCTTTAAGAAATTAAAAAAACTAAGGAAACATTTTTCTTGTTTCGAGTAGATAATGCCAGCCTGTTAAACGCCGTCGACGAGTCTAACGGACACCAACCAGCGAACCAGCAGCGTCGCGTCGGGCCAAGCGAAGCAGACGGCACGGCATCTCTGTCGCTGCCTCTGGACCCCTCTCGAGAGTTCCGCTCCACCGTTGGACTTGCTCCGCTGTCGGCATCCAGAAATTGCGTGGCGGAGCGGCAGACGTGAGCCGGCACGGCAGGCGGCCTCCTCCTCCTCTCACGGCACCGGCAGCTACGGGGGATTCCTTTCCCACCGCTCCTTCGCTTTCCCTTCCTCGCCCGCCGTAATAAATAGACACCCCCTCCACACCCTCTTTCCCCAACCTCGTGTTGTTCGGAGCGCACACACACACAACCAGATCTCCCCCAAATCCACCCGTCGGCACCTCCGCTTCAAGGTACGCCGCTCGTCCTCCCCCCCCCCCCCTCTCTACCTTCTCTAGATCGGCGTTCCGGTCCATGGTTAGGGCCCGGTAGTTCTACTTCTGTTCATGTTTGTGTTAGATCCGTGTTTGTGTTAGATCCGTGCTGCTAGCGTTCGTACACGGATGCGACCTGTACGTCAGACACGTTCTGATTGCTAACTTGCCAGTGTTTCTCTTTGGGGAATCCTGGGATGGCTCTAGCCGTTCCGCAGACGGGATCGATTTCATGATTTTTTTTGTTTCGTTGCATAGGGTTTGGTTTGCCCTTTTCCTTTATTTCAATATATGCCGTGCACTTGTTTGTCGGGTCATCTTTTCATGCTTTTTTTTGTCTTGGTTGTGATGATGTGGTCTGGTTGGGCGGTCGTTCTAGATCGGAGTAGAATTCTGTTTCAAACTACCTGGTGGATTTATTAATTTTGGATCTGTATGTGTGTGCCATACATATTCATAGTTACGAATTGAAGATGATGGATGGAAATATCGATCTAGGATAGGTATACATGTTGATGCGGGTTTTACTGATGCATATACAGAGATGCTTTTTGTTCGCTTGGTTGTGATGATGTGGTGTGGTTGGGCGGTCGTTCATTCGTTCTAGATCGGAGTAGAATACTGTTTCAAACTACCTGGTGTATTTATTAATTTTGGAACTGTATGTGTGTGTCATACATCTTCATAGTTACGAGTTTAAGATGGATGGAAATATCGATCTAGGATAGGTATACATGTTGATGTGGGTTTTACTGATGCATATACATGATGGCATATGCAGCATCTATTCATATGCTCTAACCTTGAGTACCTATCTATTATAATAAACAAGTATGTTTTATAATTATTTTGATCTTGATATACTTGGATGATGGCATATGCAGCAGCTATATGTGGATTTTTTTAGCCCTGCCTTCATACGCTATTTATTTGCTTGGTACTGTTTCTTTTGTCGATGCTCACCCTGTTGTTTGGTGTTACTTCTGCAGCCCTAGGACTGGTTGTACAATGCCGGCGGCCAAGAGAGTCAAGCTCGACGGCGGGAAGCGCACAGCTGATGGTTCTGAATTCGAGTCCCCTAAGAAAAAGAGAAAAGTGGACAAGAAGTACTCGATCGGCCTCGATATTGGGACTAACTCTGTTGGCTGGGCCGTGATCACCGACGAGTACAAGGTGCCCTCAAAGAAGTTCAAGGTCCTGGGCAACACCGATCGGCATTCCATCAAGAAGAATCTCATTGGCGCTCTCCTGTTCGACAGCGGCGAGACGGCTGAGGCTACGCGGCTCAAGCGCACCGCCCGCAGGCGGTACACGCGCAGGAAGAATCGCATCTGCTACCTGCAGGAGATTTTCTCCAACGAGATGGCGAAGGTTGACGATTCTTTCTTCCACAGGCTGGAGGAGTCATTCCTCGTGGAGGAGGATAAGAAGCACGAGCGGCATCCAATCTTCGGCAACATTGTCGACGAGGTTGCCTACCACGAGAAGTACCCTACGATCTACCATCTGCGGAAGAAGCTCGTGGACTCCACAGATAAGGCGGACCTCCGCCTGATCTACCTCGCTCTGGCCCACATGATTAAGTTCAGGGGCCATTTCCTGATCGAGGGGGATCTCAACCCGGACAATAGCGATGTTGACAAGCTGTTCATCCAGCTCGTGCAGACGTACAACCAGCTCTTCGAGGAGAACCCCATTAATGCGTCAGGCGTCGACGCGAAGGCTATCCTGTCCGCTAGGCTCTCGAAGTCTCGGAAGCTCGAGAACCTGATCGCCCAGCTGCCGGGCGAGAAGAAGAACGGCCTGTTCGGGAATCTCATTGCGCTCAGCCTGGGGCTCACGCCCAACTTCAAGTCGAATTTCGATCTCGCTGAGGACGCCAAGCTGCAGCTCTCCAAGGACACATACGACGATGACCTGGATAACCTCCTGGCCCAGATCGGCGATCAGTACGCGGACCTGTTCCTCGCTGCCAAGAATCTGTCGGACGCCATCCTCCTGTCTGATATTCTCAGGGTGAACACCGAGATTACGAAGGCTCCGCTCTCAGCCTCCATGATCAAGCGCTACGACGAGCACCATCAGGATCTGACCCTCCTGAAGGCGCTGGTCAGGCAGCAGCTCCCCGAGAAGTACAAGGAGATCTTCTTCGATCAGTCGAAGAACGGCTACGCTGGGTACATTGACGGCGGGGCCTCTCAGGAGGAGTTCTACAAGTTCATCAAGCCGATTCTGGAGAAGATGGACGGCACGGAGGAGCTGCTGGTGAAGCTCAAGCGCGAGGACCTCCTGAGGAAGCAGCGGACATTCGATAACGGCAGCATCCCACACCAGATTCATCTCGGGGAGCTGCACGCTATCCTGAGGAGGCAGGAGGACTTCTACCCTTTCCTCAAGGATAACCGCGAGAAGATCGAGAAGATTCTGACTTTCAGGATCCCGTACTACGTCGGCCCACTCGCTAGGGGCAACTCCCGCTTCGCTTGGATGACCCGCAAGTCAGAGGAGACGATCACGCCGTGGAACTTCGAGGAGGTGGTCGACAAGGGCGCTAGCGCTCAGTCGTTCATCGAGAGGATGACGAATTTCGACAAGAACCTGCCAAATGAGAAGGTGCTCCCTAAGCACTCGCTCCTGTACGAGTACTTCACAGTCTACAACGAGCTGACTAAGGTGAAGTATGTGACCGAGGGCATGAGGAAGCCGGCTTTCCTGTCTGGGGAGCAGAAGAAGGCCATCGTGGACCTCCTGTTCAAGACCAACCGGAAGGTCACGGTTAAGCAGCTCAAGGAGGACTACTTCAAGAAGATTGAGTGCTTCGATTCGGTCGAGATCTCTGGCGTTGAGGACCGCTTCAACGCCTCCCTGGGGACCTACCACGATCTCCTGAAGATCATTAAGGATAAGGACTTCCTGGACAACGAGGAGAATGAGGATATCCTCGAGGACATTGTGCTGACACTCACTCTGTTCGAGGACCGGGAGATGATCGAGGAGCGCCTGAAGACTTACGCCCATCTCTTCGATGACAAGGTCATGAAGCAGCTCAAGAGGAGGAGGTACACCGGCTGGGGGAGGCTGAGCAGGAAGCTCATCAACGGCATTCGGGACAAGCAGTCCGGGAAGACGATCCTCGACTTCCTGAAGAGCGATGGCTTCGCGAACCGCAATTTCATGCAGCTGATTCACGATGACAGCCTCACATTCAAGGAGGATATCCAGAAGGCTCAGGTGAGCGGCCAGGGGGACTCGCTGCACGAGCATATCGCGAACCTCGCTGGCTCGCCAGCTATCAAGAAGGGGATTCTGCAGACCGTGAAGGTTGTGGACGAGCTGGTGAAGGTCATGGGCAGGCACAAGCCTGAGAACATCGTCATTGAGATGGCCCGGGAGAATCAGACCACGCAGAAGGGCCAGAAGAACTCACGCGAGAGGATGAAGAGGATCGAGGAGGGCATTAAGGAGCTGGGGTCCCAGATCCTCAAGGAGCACCCGGTGGAGAACACGCAGCTGCAGAATGAGAAGCTCTACCTGTACTACCTCCAGAATGGCCGCGATATGTATGTGGACCAGGAGCTGGATATTAACAGGCTCAGCGATTACGACGTCGATGCCATCGTTCCACAGTCATTCCTGAAGGATGACTCCATTGACAACAAGGTCCTCACCAGGTCGGACAAGAACCGGGGCAAGTCTGATAATGTTCCTTCAGAGGAGGTCGTTAAGAAGATGAAGAACTACTGGCGCCAGCTCCTGAATGCCAAGCTGATCACGCAGCGGAAGTTCGATAACCTCACAAAGGCTGAGAGGGGCGGGCTCTCTGAGCTGGACAAGGCGGGCTTCATCAAGAGGCAGCTGGTCGAGACACGGCAGATCACTAAGCACGTTGCGCAGATTCTCGACTCACGGATGAACACTAAGTACGATGAGAATGACAAGCTGATCCGCGAGGTGAAGGTCATCACCCTGAAGTCAAAGCTCGTCTCCGACTTCAGGAAGGATTTCCAGTTCTACAAGGTTCGGGAGATCAACAATTACCACCATGCCCATGACGCGTACCTGAACGCGGTGGTCGGCACAGCTCTGATCAAGAAGTACCCAAAGCTCGAGAGCGAGTTCGTGTACGGGGACTACAAGGTTTACGATGTGAGGAAGATGATCGCCAAGTCGGAGCAGGAGATTGGCAAGGCTACCGCCAAGTACTTCTTCTACTCTAACATTATGAATTTCTTCAAGACAGAGATCACTCTGGCCAATGGCGAGATCCGGAAGCGCCCCCTCATCGAGACGAACGGCGAGACGGGGGAGATCGTGTGGGACAAGGGCAGGGATTTCGCGACCGTCAGGAAGGTTCTCTCCATGCCACAAGTGAATATCGTCAAGAAGACAGAGGTCCAGACTGGCGGGTTCTCTAAGGAGTCAATTCTGCCTAAGCGGAACAGCGACAAGCTCATCGCCCGCAAGAAGGACTGGGATCCGAAGAAGTACGGCGGGTTCGACAGCCCCACTGTGGCCTACTCGGTCCTGGTTGTGGCGAAGGTTGAGAAGGGCAAGTCCAAGAAGCTCAAGAGCGTGAAGGAGCTGCTGGGGATCACGATTATGGAGCGCTCCAGCTTCGAGAAGAACCCGATCGATTTCCTGGAGGCGAAGGGCTACAAGGAGGTGAAGAAGGACCTGATCATTAAGCTCCCCAAGTACTCACTCTTCGAGCTGGAGAACGGCAGGAAGCGGATGCTGGCTTCCGCTGGCGAGCTGCAGAAGGGGAACGAGCTGGCTCTGCCGTCCAAGTATGTGAACTTCCTCTACCTGGCCTCCCACTACGAGAAGCTCAAGGGCAGCCCCGAGGACAACGAGCAGAAGCAGCTGTTCGTCGAGCAGCACAAGCATTACCTCGACGAGATCATTGAGCAGATTTCCGAGTTCTCCAAGCGCGTGATCCTGGCCGACGCGAATCTGGATAAGGTCCTCTCCGCGTACAACAAGCACCGCGACAAGCCAATCAGGGAGCAGGCTGAGAATATCATTCATCTCTTCACCCTGACGAACCTCGGCGCCCCTGCTGCTTTCAAGTACTTCGACACAACTATCGATCGCAAGAGGTACACAAGCACTAAGGAGGTCCTGGACGCGACCCTCATCCACCAGTCGATTACCGGCCTCTACGAGACGCGCATCGACCTGTCTCAGCTCGGGGGCGACGAATTCTCCGGGAGCGAGACGCCAGGCACCTCCGAGTCGGCCACCCCAGAATCTGCCACAGTGGTGTCCGGCCAAAAGCAGGACCGCCAGGGCGGAGAACGCAGAAGGTCCCAGCTCGATAGGGATCAGTGTGCCTACTGCAAGGAGAAGGGCCACTGGGCCAAAGACTGCCCGAAAAAGCCGCGCGGCCCACGCGGCCCAAGGCCACAAACATCCCTCCTTCCAAAGAAGAAGCGGAAGGTGGAGCTCAGCGGAGGATCTTCCGGAGGATCTAGCGGCTCCGAGACACCAGGAACATCCGAAAGCGCTACACCAGAATCTAGCGGAGGCTCTTCCGGAGGATCTAGGCCTACCCTCAACATCGAGGATGAGTATCGCCTCCACGAAACCTCCAAAGAACCGGACGTGTCCCTCGGCAGCACATGGCTCAGCGACTTCCCACAAGCGTGGGCCGAAACCGGCGGCATGGGCCTCGCCGTCCGCCAAGCCCCACTCATTATCCCGCTGAAGGCGACCTCCACACCGGTGTCCATCAAGCAGTACCCGATGAGCCAAGAGGCGAGGCTCGGGATTAAGCCGCACATTCAGCGCCTCCTCGATCAAGGCATTCTCGTGCCGTGCCAATCCCCGTGGAATACACCACTCCTCCCGGTCAAAAAGCCGGGCACCAACGACTATCGCCCGGTCCAAGATCTCCGCGAGGTCAACAAGCGCGTGGAAGACATCCACCCGACCGTCCCGAACCCGTATAATCTGCTCTCCGGGCTCCCACCATCCCACCAGTGGTATACAGTGCTGGACCTCAAAGACGCCTTCTTCTGTCTCCGCCTCCACCCAACAAGCCAGCCGCTCTTCGCCTTCGAGTGGCGCGACCCGGAGATGGGCATCTCCGGCCAACTGACATGGACACGCCTCCCGCAAGGCTTCAAGAACAGCCCGACACTCTTCAACGAGGCGCTCCATAGGGACCTCGCGGATTTTCGCATCCAGCATCCGGACCTCATCCTCCTCCAGTATGCGGATGATCTCCTCCTCGCCGCGACCTCCGAGCTGGATTGTCAACAAGGCACACGCGCGCTCCTCCAAACACTCGGGAACCTCGGCTATCGCGCGTCCGCGAAAAAGGCCCAAATCTGCCAGAAGCAAGTGAAGTACCTCGGGTATCTGCTCAAGGAAGGCCAACGCTGGCTCACCGAAGCGCGCAAAGAAACAGTGATGGGGCAACCGACACCGAAAACACCACGCCAGCTGCGCGAGTTTCTCGGCAAAGCCGGCTTCTGTCGCCTCTTCATCCCGGGCTTTGCCGAGATGGCCGCGCCACTCTACCCACTCACCAAGCCGGGCACACTGTTTAACTGGGGGCCGGATCAGCAGAAAGCCTACCAAGAGATCAAACAAGCGCTCCTCACCGCCCCAGCGCTCGGGCTCCCAGATCTCACAAAGCCGTTCGAGCTGTTCGTCGATGAGAAGCAAGGCTACGCGAAGGGCGTGCTCACACAGAAGCTCGGCCCGTGGAGGAGGCCAGTGGCCTATCTCTCCAAAAAACTCGATCCAGTGGCCGCCGGCTGGCCACCGTGTCTGCGCATGGTCGCCGCGATTGCCGTGCTCACAAAGGATGCCGGCAAACTCACAATGGGCCAGCCGCTGGTGATCCTCGCGCCACATGCCGTGGAAGCCCTCGTCAAACAGCCGCCGGATAGGTGGCTCTCCAATGCGCGCATGACCCATTACCAAGCGCTCCTCCTCGACACCGATCGCGTCCAGTTCGGCCCAGTGGTCGCCCTCAATCCGGCGACACTGCTGCCACTCCCAGAGGAGGGCCTCCAACACAACTGTCTGGATATTCTCGCGGAAGCGCATGGCACAAGGCCAGACCTCACAGATCAACCGCTCAGCGGCGGCAGCAAAAGAACGGCGGACGGCTCTGAGAAGCGCACCGCTGATAGCCAGCATTCAACTCCTCCGAAAACAAAGAGGAAAGTTGAGTTCGAACCGAAGAAGAAAAGGAAGGTGTGAGAGCTCAGAGCTTTCGTTCGTATCATCGGTTTCGACAACGTTCGTCAAGTTCAATGCATCAGTTTCATTGCGCACACACCAGAATCCTACTGAGTTTGAGTATTATGGCATTGGGAAAACTGTTTTTCTTGTACCATTTGTTGTGCTTGTAATTTACTGTGTTTTTTATTCGGTTTTCGCTATCGAACTGTGAAATGGAAATGGATGGAGAAGAGTTAATGAATGATATGGTCCTTTTGTTCATTCTCAAATTAATATTATTTGTTTTTTCTCTTATTTGTTGTGTGTTGAATTTGAAATTATAAGAGATATGCAAACATTTTGTTTTGAGTAAAAATGTGTCAAATCGTGGCCTCTAATGACCGAAGTTAATATGAGGAGTAAAACACTTGTAGTTGTACCATTATGCTTATTCACTAGGCAACAAATATATTTTCAGACCTAGAAAAGCTGCAAATGTTACTGAATACAAGTATGTCCTCTTGTGTTTTAGACATTTATGAACTTTCCTTTATGTAATTTTCCAGAATCCTTGTCAGATTCTAATCATTGCTTTATAATTATAGTTATACTCATGGATTTGTAGTTGAGTATGAAAATATTTTTTAATGCATTTTATGACTTGCCAATTGATTGACAACGAATTCGTAATCATGTCATAGCTGTTTCCTGTGTGAAATTGTTATCCGCTCACAATTCCACACAACATACGAGCCGGAAGCATAAAGTGTAAAGCCTGGGGTGCCTAATGAGTGAGCTAACTCACATTAATTGCGTTGCGCTCACTGCCCGCTTTCCAGTCGGGAAACCTGTCGTGCCAGCTGCATTAATGAATCGGCCAACGCGCGGGGAGAGGCGGTTTGCGTATTGGCTAGAGCAGCTTGCCAACATGGTGGAGCACGACACTCTCGTCTACTCCAAGAATATCAAAGATACAGTCTCAGAAGACCAAAGGGCTATTGAGACTTTTCAACAAAGGGTAATATCGGGAAACCTCCTCGGATTCCATTGCCCAGCTATCTGTCACTTCATCAAAAGGACAGTAGAAAAGGAAGGTGGCACCTACAAATGCCATCATTGCGATAAAGGAAAGGCTATCGTTCAAGATGCCTCTGCCGACAGTGGTCCCAAAGATGGACCCCCACCCACGAGGAGCATCGTGGAAAAAGAAGACGTTCCAACCACGTCTTCAAAGCAAGTGGATTGATGTGAACATGGTGGAGCACGACACTCTCGTCTACTCCAAGAATATCAAAGATACAGTCTCAGAAGACCAAAGGGCTATTGAGACTTTTCAACAAAGGGTAATATCGGGAAACCTCCTCGGATTCCATTGCCCAGCTATCTGTCACTTCATCAAAAGGACAGTAGAAAAGGAAGGTGGCACCTACAAATGCCATCATTGCGATAAAGGAAAGGCTATCGTTCAAGATGCCTCTGCCGACAGTGGTCCCAAAGATGGACCCCCACCCACGAGGAGCATCGTGGAAAAAGAAGACGTTCCAACCACGTCTTCAAAGCAAGTGGATTGATGTGATATCTCCACTGACGTAAGGGATGACGCACAATCCCACTATCCTTCGCAAGACCCTTCCTCTATATAAGGAAGTTCATTTCATTTGGAGAGGACACGCTGAAATCACCAGTCTCTCTCTACAAATCTATCTCTCTCGAGCTTTCGCAGATCCGGGGGGCAATGAGATATGAAAAAGCCTGAACTCACCGCGACGTCTGTCGAGAAGTTTCTGATCGAAAAGTTCGACAGCGTCTCCGACCTGATGCAGCTCTCGGAGGGCGAAGAATCTCGTGCTTTCAGCTTCGATGTAGGAGGGCGTGGATATGTCCTGCGGGTAAATAGCTGCGCCGATGGTTTCTACAAAGATCGTTATGTTTATCGGCACTTTGCATCGGCCGCGCTCCCGATTCCGGAAGTGCTTGACATTGGGGAGTTTAGCGAGAGCCTGACCTATTGCATCTCCCGCCGTTCACAGGGTGTCACGTTGCAAGACCTGCCTGAAACCGAACTGCCCGCTGTTCTACAACCGGTCGCGGAGGCTATGGATGCGATCGCTGCGGCCGATCTTAGCCAGACGAGCGGGTTCGGCCCATTCGGACCGCAAGGAATCGGTCAATACACTACATGGCGTGATTTCATATGCGCGATTGCTGATCCCCATGTGTATCACTGGCAAACTGTGATGGACGACACCGTCAGTGCGTCCGTCGCGCAGGCTCTCGATGAGCTGATGCTTTGGGCCGAGGACTGCCCCGAAGTCCGGCACCTCGTGCACGCGGATTTCGGCTCCAACAATGTCCTGACGGACAATGGCCGCATAACAGCGGTCATTGACTGGAGCGAGGCGATGTTCGGGGATTCCCAATACGAGGTCGCCAACATCTTCTTCTGGAGGCCGTGGTTGGCTTGTATGGAGCAGCAGACGCGCTACTTCGAGCGGAGGCATCCGGAGCTTGCAGGATCGCCACGACTCCGGGCGTATATGCTCCGCATTGGTCTTGACCAACTCTATCAGAGCTTGGTTGACGGCAATTTCGATGATGCAGCTTGGGCGCAGGGTCGATGCGACGCAATCGTCCGATCCGGAGCCGGGACTGTCGGGCGTACACAAATCGCCCGCAGAAGCGCGGCCGTCTGGACCGATGGCTGTGTAGAAGTACTCGCCGATAGTGGAAACCGACGCCCCAGCACTCGTCCGAGGGCAAAGAAATAGAGTAGATGCCGACCGGGATCTGTCGATCGACAAGCTCGAGTTTCTCCATAATAATGTGTGAGTAGTTCCCAGATAAGGGAATTAGGGTTCCTATAGGGTTTCGCTCATGTGTTGAGCATATAAGAAACCCTTAGTATGTATTTGTATTTGTAAAATACTTCTATCAATAAAATTTCTAATTCCTAAAACCAAAATCCAGTACTAAAATCCAGATCCCCCGAATTAATTCGGCGTTAATTCAGTACATTAAAAACGTCCGCAATGTGTTATTAAGTTGTCTAAGCGTCAATTTGTTTACACCACAATATATCCTGCCA

**Sequence 81 Plasmids sequence of pH-35C-M-MLV-OsPDS**

(RB T-DNA repeat-35C promoter-tRNAGly RS-pegRNA-Ubi promoter-NLSc-myc-NLSsv40-nCas9(H840A/ R221K/N394K)-XTEN-NC-NLS-32aa Linker-M-MLV-RT-NLSvbp-HygR cassette-E9-terminator-CaMV poly(A) signal-LB T-DNA repeat)

GTTTACCCGCCAATATATCCTGTCAAACACTGATAGTTTAAACTGAAGGCGGGAAACGACAATCTGATCCAAGCTCAAGCTGCTCTAGCATTCGCCATTCAGGCTGCGCAACTGTTGGGAAGGGCGATCGGTGCGGGCCTCTTCGCTATTACGCCAGCTGGCGAAAGGGGGATGTGCTGCAAGGCGATTAAGTTGGGTAACGCCAGGGTTTTCCCAGTCACGACGTTGTAAAACGACGGCCAGTGAATTCATGGAGTCAAAGATTCAAATAGAGGACCTAACAGAACTCGCCGTAAAGACTGGCGAACAGTTCATACAGAGTCTCTTACGACTCAATGACAAGAAGAAAATCTTCGTCAACATGGTGGAGCACGACACACTTGTCTACTCCAAAAATATCAAAGATACAGTCTCAGAAGACCAAAGGGCAATTGAGACTTTTCAACAAAGGGTAATATCCGGAAACCTCCTCGGATTCCATTGCCCAGCTATCTGTCACTTTATTGTGAAGATAGTGGAAAAGGAAGGTGGCTCCTACAAATGCCATCATTGCGATAAAGGAAAGGCCATCGTTGAAGATGCCTCTGCCGACAGTGGTCCCAAAGATGGACCCCCACCCACGAGGAGCATCGTGGAAAAAGAAGACGTTCCAACCACGTCTTCAAAGCAAGTGGATTGATGTGATTGGCAGACATACTGTCCCACAAATGAAGATGGAATCTGTAAAAGAAAACGCGTGAAATAATGCGTCTGACAAAGGTTAGGTCGGCTGCCTTTAATCAATACCAAAGTGGTCCCTACCACGATGGAAAAACTGTGCAGTCGGTTTGGCTTTTTCTGACGAACAAATAAGATTCGTGGCCGACAGGTGGGGGTCCACCATGTGAAGGCATCTTCAGACTCCAATAATGGAGCAATGACGTAAGGGCTTACGAAATAAGTAAGGGTAGTTTGGGAAATGTCCACTCACCCGTCAGTCTATAAATACTTAGCCCCTCCCTCATTGTTAAGGGAGCAAAATCTCAGAGAGATAGTCCTAGAGAGAGAAAGAGAGCAAGTAGCCTAGAAGTAGTCAAGGCGGCGAAGTATTCAGGCACGTGGCCAGGAAGAAGAAAAGCCAAGACGACGAAAACAGGTAAGAGCTAAGCATCTAGATAAGTTGAAAACAATCTTCAAAAGTCCCACATCGCTTAGATAAGAAAACGAAGCTGAGTTTATATACAGCTAGAGTCGAAGTAGTGATTGAACAAAGCACCAGTGGTCTAGTGGTAGAATAGTACCCTGCCACGGTACAGACCCGGGTTCGATTCCCGGCTGGTGCAGTTGGTCTTTGCTCCTGCAGGTTTAAGAGCTATGCTGGAAACAGCATAGCAAGTTTAAATAAGGCTAGTCCGTTATCAACTTGAAAAAGTGGCACCGAGTCGGTGCGTCCAACCCATTCTGCAGGAGCAAACGCGGTTCTATCTAGTTACGCGTTAAACCAACTAGAATTTTTTTGGCCGGCATGGTCCCAGCCTCCTCGCTGGCGCCGGCTGGGCAACATGCTTCGGCATGGCGAATGGGACTTTTTTTTGATATCTCCGGGGCTAATTGAATATGAAGATGAAGATGAAATATTTGGTGTGTCAAATAAAAAGCTGGTGTGCTTAAGTTTGTGTTTTTTTCTTGGCTTGTTGTGTTATGAATTTGTGGCTTTTTCTAATATTAAATGAATGTAAGATCTCATTATAATGAATAAACAAATGTTTCTATAATCCATTGTGAATGTTTTGTTGGATCTCTTCTGCAGCATATAACTACTGTATGTGCTATGGTATGGACTATGGAATATGATTAAAGATAAGAAGCTTGGCGTAATCTGCATGCCTGCAGTGCAGCGTGACCCGGTCGTGCCCCTCTCTAGAGATAATGAGCATTGCATGTCTAAGTTATAAAAAATTACCACATATTTTTTTTGTCACACTTGTTTGAAGTGCAGTTTATCTATCTTTATACATATATTTAAACTTTACTCTACGAATAATATAATCTATAGTACTACAATAATATCAGTGTTTTAGAGAATCATATAAATGAACAGTTAGACATGGTCTAAAGGACAATTGAGTATTTTGACAACAGGACTCTACAGTTTTATCTTTTTAGTGTGCATGTGTTCTCCTTTTTTTTTGCAAATAGCTTCACCTATATAATACTTCATCCATTTTATTAGTACATCCATTTAGGGTTTAGGGTTAATGGTTTTTATAGACTAATTTTTTTAGTACATCTATTTTATTCTATTTTAGCCTCTAAATTAAGAAAACTAAAACTCTATTTTAGTTTTTTTATTTAATAATTTAGATATAAAATAGAATAAAATAAAGTGACTAAAAATTAAACAAATACCCTTTAAGAAATTAAAAAAACTAAGGAAACATTTTTCTTGTTTCGAGTAGATAATGCCAGCCTGTTAAACGCCGTCGACGAGTCTAACGGACACCAACCAGCGAACCAGCAGCGTCGCGTCGGGCCAAGCGAAGCAGACGGCACGGCATCTCTGTCGCTGCCTCTGGACCCCTCTCGAGAGTTCCGCTCCACCGTTGGACTTGCTCCGCTGTCGGCATCCAGAAATTGCGTGGCGGAGCGGCAGACGTGAGCCGGCACGGCAGGCGGCCTCCTCCTCCTCTCACGGCACCGGCAGCTACGGGGGATTCCTTTCCCACCGCTCCTTCGCTTTCCCTTCCTCGCCCGCCGTAATAAATAGACACCCCCTCCACACCCTCTTTCCCCAACCTCGTGTTGTTCGGAGCGCACACACACACAACCAGATCTCCCCCAAATCCACCCGTCGGCACCTCCGCTTCAAGGTACGCCGCTCGTCCTCCCCCCCCCCCCCTCTCTACCTTCTCTAGATCGGCGTTCCGGTCCATGGTTAGGGCCCGGTAGTTCTACTTCTGTTCATGTTTGTGTTAGATCCGTGTTTGTGTTAGATCCGTGCTGCTAGCGTTCGTACACGGATGCGACCTGTACGTCAGACACGTTCTGATTGCTAACTTGCCAGTGTTTCTCTTTGGGGAATCCTGGGATGGCTCTAGCCGTTCCGCAGACGGGATCGATTTCATGATTTTTTTTGTTTCGTTGCATAGGGTTTGGTTTGCCCTTTTCCTTTATTTCAATATATGCCGTGCACTTGTTTGTCGGGTCATCTTTTCATGCTTTTTTTTGTCTTGGTTGTGATGATGTGGTCTGGTTGGGCGGTCGTTCTAGATCGGAGTAGAATTCTGTTTCAAACTACCTGGTGGATTTATTAATTTTGGATCTGTATGTGTGTGCCATACATATTCATAGTTACGAATTGAAGATGATGGATGGAAATATCGATCTAGGATAGGTATACATGTTGATGCGGGTTTTACTGATGCATATACAGAGATGCTTTTTGTTCGCTTGGTTGTGATGATGTGGTGTGGTTGGGCGGTCGTTCATTCGTTCTAGATCGGAGTAGAATACTGTTTCAAACTACCTGGTGTATTTATTAATTTTGGAACTGTATGTGTGTGTCATACATCTTCATAGTTACGAGTTTAAGATGGATGGAAATATCGATCTAGGATAGGTATACATGTTGATGTGGGTTTTACTGATGCATATACATGATGGCATATGCAGCATCTATTCATATGCTCTAACCTTGAGTACCTATCTATTATAATAAACAAGTATGTTTTATAATTATTTTGATCTTGATATACTTGGATGATGGCATATGCAGCAGCTATATGTGGATTTTTTTAGCCCTGCCTTCATACGCTATTTATTTGCTTGGTACTGTTTCTTTTGTCGATGCTCACCCTGTTGTTTGGTGTTACTTCTGCAGCCCTAGGACTGGTTGTACAATGCCGGCGGCCAAGAGAGTCAAGCTCGACGGCGGGAAGCGCACAGCTGATGGTTCTGAATTCGAGTCCCCTAAGAAAAAGAGAAAAGTGGACAAGAAGTACTCGATCGGCCTCGATATTGGGACTAACTCTGTTGGCTGGGCCGTGATCACCGACGAGTACAAGGTGCCCTCAAAGAAGTTCAAGGTCCTGGGCAACACCGATCGGCATTCCATCAAGAAGAATCTCATTGGCGCTCTCCTGTTCGACAGCGGCGAGACGGCTGAGGCTACGCGGCTCAAGCGCACCGCCCGCAGGCGGTACACGCGCAGGAAGAATCGCATCTGCTACCTGCAGGAGATTTTCTCCAACGAGATGGCGAAGGTTGACGATTCTTTCTTCCACAGGCTGGAGGAGTCATTCCTCGTGGAGGAGGATAAGAAGCACGAGCGGCATCCAATCTTCGGCAACATTGTCGACGAGGTTGCCTACCACGAGAAGTACCCTACGATCTACCATCTGCGGAAGAAGCTCGTGGACTCCACAGATAAGGCGGACCTCCGCCTGATCTACCTCGCTCTGGCCCACATGATTAAGTTCAGGGGCCATTTCCTGATCGAGGGGGATCTCAACCCGGACAATAGCGATGTTGACAAGCTGTTCATCCAGCTCGTGCAGACGTACAACCAGCTCTTCGAGGAGAACCCCATTAATGCGTCAGGCGTCGACGCGAAGGCTATCCTGTCCGCTAGGCTCTCGAAGTCTCGGAAGCTCGAGAACCTGATCGCCCAGCTGCCGGGCGAGAAGAAGAACGGCCTGTTCGGGAATCTCATTGCGCTCAGCCTGGGGCTCACGCCCAACTTCAAGTCGAATTTCGATCTCGCTGAGGACGCCAAGCTGCAGCTCTCCAAGGACACATACGACGATGACCTGGATAACCTCCTGGCCCAGATCGGCGATCAGTACGCGGACCTGTTCCTCGCTGCCAAGAATCTGTCGGACGCCATCCTCCTGTCTGATATTCTCAGGGTGAACACCGAGATTACGAAGGCTCCGCTCTCAGCCTCCATGATCAAGCGCTACGACGAGCACCATCAGGATCTGACCCTCCTGAAGGCGCTGGTCAGGCAGCAGCTCCCCGAGAAGTACAAGGAGATCTTCTTCGATCAGTCGAAGAACGGCTACGCTGGGTACATTGACGGCGGGGCCTCTCAGGAGGAGTTCTACAAGTTCATCAAGCCGATTCTGGAGAAGATGGACGGCACGGAGGAGCTGCTGGTGAAGCTCAAGCGCGAGGACCTCCTGAGGAAGCAGCGGACATTCGATAACGGCAGCATCCCACACCAGATTCATCTCGGGGAGCTGCACGCTATCCTGAGGAGGCAGGAGGACTTCTACCCTTTCCTCAAGGATAACCGCGAGAAGATCGAGAAGATTCTGACTTTCAGGATCCCGTACTACGTCGGCCCACTCGCTAGGGGCAACTCCCGCTTCGCTTGGATGACCCGCAAGTCAGAGGAGACGATCACGCCGTGGAACTTCGAGGAGGTGGTCGACAAGGGCGCTAGCGCTCAGTCGTTCATCGAGAGGATGACGAATTTCGACAAGAACCTGCCAAATGAGAAGGTGCTCCCTAAGCACTCGCTCCTGTACGAGTACTTCACAGTCTACAACGAGCTGACTAAGGTGAAGTATGTGACCGAGGGCATGAGGAAGCCGGCTTTCCTGTCTGGGGAGCAGAAGAAGGCCATCGTGGACCTCCTGTTCAAGACCAACCGGAAGGTCACGGTTAAGCAGCTCAAGGAGGACTACTTCAAGAAGATTGAGTGCTTCGATTCGGTCGAGATCTCTGGCGTTGAGGACCGCTTCAACGCCTCCCTGGGGACCTACCACGATCTCCTGAAGATCATTAAGGATAAGGACTTCCTGGACAACGAGGAGAATGAGGATATCCTCGAGGACATTGTGCTGACACTCACTCTGTTCGAGGACCGGGAGATGATCGAGGAGCGCCTGAAGACTTACGCCCATCTCTTCGATGACAAGGTCATGAAGCAGCTCAAGAGGAGGAGGTACACCGGCTGGGGGAGGCTGAGCAGGAAGCTCATCAACGGCATTCGGGACAAGCAGTCCGGGAAGACGATCCTCGACTTCCTGAAGAGCGATGGCTTCGCGAACCGCAATTTCATGCAGCTGATTCACGATGACAGCCTCACATTCAAGGAGGATATCCAGAAGGCTCAGGTGAGCGGCCAGGGGGACTCGCTGCACGAGCATATCGCGAACCTCGCTGGCTCGCCAGCTATCAAGAAGGGGATTCTGCAGACCGTGAAGGTTGTGGACGAGCTGGTGAAGGTCATGGGCAGGCACAAGCCTGAGAACATCGTCATTGAGATGGCCCGGGAGAATCAGACCACGCAGAAGGGCCAGAAGAACTCACGCGAGAGGATGAAGAGGATCGAGGAGGGCATTAAGGAGCTGGGGTCCCAGATCCTCAAGGAGCACCCGGTGGAGAACACGCAGCTGCAGAATGAGAAGCTCTACCTGTACTACCTCCAGAATGGCCGCGATATGTATGTGGACCAGGAGCTGGATATTAACAGGCTCAGCGATTACGACGTCGATGCCATCGTTCCACAGTCATTCCTGAAGGATGACTCCATTGACAACAAGGTCCTCACCAGGTCGGACAAGAACCGGGGCAAGTCTGATAATGTTCCTTCAGAGGAGGTCGTTAAGAAGATGAAGAACTACTGGCGCCAGCTCCTGAATGCCAAGCTGATCACGCAGCGGAAGTTCGATAACCTCACAAAGGCTGAGAGGGGCGGGCTCTCTGAGCTGGACAAGGCGGGCTTCATCAAGAGGCAGCTGGTCGAGACACGGCAGATCACTAAGCACGTTGCGCAGATTCTCGACTCACGGATGAACACTAAGTACGATGAGAATGACAAGCTGATCCGCGAGGTGAAGGTCATCACCCTGAAGTCAAAGCTCGTCTCCGACTTCAGGAAGGATTTCCAGTTCTACAAGGTTCGGGAGATCAACAATTACCACCATGCCCATGACGCGTACCTGAACGCGGTGGTCGGCACAGCTCTGATCAAGAAGTACCCAAAGCTCGAGAGCGAGTTCGTGTACGGGGACTACAAGGTTTACGATGTGAGGAAGATGATCGCCAAGTCGGAGCAGGAGATTGGCAAGGCTACCGCCAAGTACTTCTTCTACTCTAACATTATGAATTTCTTCAAGACAGAGATCACTCTGGCCAATGGCGAGATCCGGAAGCGCCCCCTCATCGAGACGAACGGCGAGACGGGGGAGATCGTGTGGGACAAGGGCAGGGATTTCGCGACCGTCAGGAAGGTTCTCTCCATGCCACAAGTGAATATCGTCAAGAAGACAGAGGTCCAGACTGGCGGGTTCTCTAAGGAGTCAATTCTGCCTAAGCGGAACAGCGACAAGCTCATCGCCCGCAAGAAGGACTGGGATCCGAAGAAGTACGGCGGGTTCGACAGCCCCACTGTGGCCTACTCGGTCCTGGTTGTGGCGAAGGTTGAGAAGGGCAAGTCCAAGAAGCTCAAGAGCGTGAAGGAGCTGCTGGGGATCACGATTATGGAGCGCTCCAGCTTCGAGAAGAACCCGATCGATTTCCTGGAGGCGAAGGGCTACAAGGAGGTGAAGAAGGACCTGATCATTAAGCTCCCCAAGTACTCACTCTTCGAGCTGGAGAACGGCAGGAAGCGGATGCTGGCTTCCGCTGGCGAGCTGCAGAAGGGGAACGAGCTGGCTCTGCCGTCCAAGTATGTGAACTTCCTCTACCTGGCCTCCCACTACGAGAAGCTCAAGGGCAGCCCCGAGGACAACGAGCAGAAGCAGCTGTTCGTCGAGCAGCACAAGCATTACCTCGACGAGATCATTGAGCAGATTTCCGAGTTCTCCAAGCGCGTGATCCTGGCCGACGCGAATCTGGATAAGGTCCTCTCCGCGTACAACAAGCACCGCGACAAGCCAATCAGGGAGCAGGCTGAGAATATCATTCATCTCTTCACCCTGACGAACCTCGGCGCCCCTGCTGCTTTCAAGTACTTCGACACAACTATCGATCGCAAGAGGTACACAAGCACTAAGGAGGTCCTGGACGCGACCCTCATCCACCAGTCGATTACCGGCCTCTACGAGACGCGCATCGACCTGTCTCAGCTCGGGGGCGACGAATTCTCCGGGAGCGAGACGCCAGGCACCTCCGAGTCGGCCACCCCAGAATCTGCCACAGTGGTGTCCGGCCAAAAGCAGGACCGCCAGGGCGGAGAACGCAGAAGGTCCCAGCTCGATAGGGATCAGTGTGCCTACTGCAAGGAGAAGGGCCACTGGGCCAAAGACTGCCCGAAAAAGCCGCGCGGCCCACGCGGCCCAAGGCCACAAACATCCCTCCTTCCAAAGAAGAAGCGGAAGGTGGAGCTCAGCGGAGGATCTTCCGGAGGATCTAGCGGCTCCGAGACACCAGGAACATCCGAAAGCGCTACACCAGAATCTAGCGGAGGCTCTTCCGGAGGATCTAGGCCTACCCTCAACATCGAGGATGAGTATCGCCTCCACGAAACCTCCAAAGAACCGGACGTGTCCCTCGGCAGCACATGGCTCAGCGACTTCCCACAAGCGTGGGCCGAAACCGGCGGCATGGGCCTCGCCGTCCGCCAAGCCCCACTCATTATCCCGCTGAAGGCGACCTCCACACCGGTGTCCATCAAGCAGTACCCGATGAGCCAAGAGGCGAGGCTCGGGATTAAGCCGCACATTCAGCGCCTCCTCGATCAAGGCATTCTCGTGCCGTGCCAATCCCCGTGGAATACACCACTCCTCCCGGTCAAAAAGCCGGGCACCAACGACTATCGCCCGGTCCAAGATCTCCGCGAGGTCAACAAGCGCGTGGAAGACATCCACCCGACCGTCCCGAACCCGTATAATCTGCTCTCCGGGCTCCCACCATCCCACCAGTGGTATACAGTGCTGGACCTCAAAGACGCCTTCTTCTGTCTCCGCCTCCACCCAACAAGCCAGCCGCTCTTCGCCTTCGAGTGGCGCGACCCGGAGATGGGCATCTCCGGCCAACTGACATGGACACGCCTCCCGCAAGGCTTCAAGAACAGCCCGACACTCTTCAACGAGGCGCTCCATAGGGACCTCGCGGATTTTCGCATCCAGCATCCGGACCTCATCCTCCTCCAGTATGCGGATGATCTCCTCCTCGCCGCGACCTCCGAGCTGGATTGTCAACAAGGCACACGCGCGCTCCTCCAAACACTCGGGAACCTCGGCTATCGCGCGTCCGCGAAAAAGGCCCAAATCTGCCAGAAGCAAGTGAAGTACCTCGGGTATCTGCTCAAGGAAGGCCAACGCTGGCTCACCGAAGCGCGCAAAGAAACAGTGATGGGGCAACCGACACCGAAAACACCACGCCAGCTGCGCGAGTTTCTCGGCAAAGCCGGCTTCTGTCGCCTCTTCATCCCGGGCTTTGCCGAGATGGCCGCGCCACTCTACCCACTCACCAAGCCGGGCACACTGTTTAACTGGGGGCCGGATCAGCAGAAAGCCTACCAAGAGATCAAACAAGCGCTCCTCACCGCCCCAGCGCTCGGGCTCCCAGATCTCACAAAGCCGTTCGAGCTGTTCGTCGATGAGAAGCAAGGCTACGCGAAGGGCGTGCTCACACAGAAGCTCGGCCCGTGGAGGAGGCCAGTGGCCTATCTCTCCAAAAAACTCGATCCAGTGGCCGCCGGCTGGCCACCGTGTCTGCGCATGGTCGCCGCGATTGCCGTGCTCACAAAGGATGCCGGCAAACTCACAATGGGCCAGCCGCTGGTGATCCTCGCGCCACATGCCGTGGAAGCCCTCGTCAAACAGCCGCCGGATAGGTGGCTCTCCAATGCGCGCATGACCCATTACCAAGCGCTCCTCCTCGACACCGATCGCGTCCAGTTCGGCCCAGTGGTCGCCCTCAATCCGGCGACACTGCTGCCACTCCCAGAGGAGGGCCTCCAACACAACTGTCTGGATATTCTCGCGGAAGCGCATGGCACAAGGCCAGACCTCACAGATCAACCGCTCAGCGGCGGCAGCAAAAGAACGGCGGACGGCTCTGAGAAGCGCACCGCTGATAGCCAGCATTCAACTCCTCCGAAAACAAAGAGGAAAGTTGAGTTCGAACCGAAGAAGAAAAGGAAGGTGTGAGAGCTCAGAGCTTTCGTTCGTATCATCGGTTTCGACAACGTTCGTCAAGTTCAATGCATCAGTTTCATTGCGCACACACCAGAATCCTACTGAGTTTGAGTATTATGGCATTGGGAAAACTGTTTTTCTTGTACCATTTGTTGTGCTTGTAATTTACTGTGTTTTTTATTCGGTTTTCGCTATCGAACTGTGAAATGGAAATGGATGGAGAAGAGTTAATGAATGATATGGTCCTTTTGTTCATTCTCAAATTAATATTATTTGTTTTTTCTCTTATTTGTTGTGTGTTGAATTTGAAATTATAAGAGATATGCAAACATTTTGTTTTGAGTAAAAATGTGTCAAATCGTGGCCTCTAATGACCGAAGTTAATATGAGGAGTAAAACACTTGTAGTTGTACCATTATGCTTATTCACTAGGCAACAAATATATTTTCAGACCTAGAAAAGCTGCAAATGTTACTGAATACAAGTATGTCCTCTTGTGTTTTAGACATTTATGAACTTTCCTTTATGTAATTTTCCAGAATCCTTGTCAGATTCTAATCATTGCTTTATAATTATAGTTATACTCATGGATTTGTAGTTGAGTATGAAAATATTTTTTAATGCATTTTATGACTTGCCAATTGATTGACAACGAATTCGTAATCATGTCATAGCTGTTTCCTGTGTGAAATTGTTATCCGCTCACAATTCCACACAACATACGAGCCGGAAGCATAAAGTGTAAAGCCTGGGGTGCCTAATGAGTGAGCTAACTCACATTAATTGCGTTGCGCTCACTGCCCGCTTTCCAGTCGGGAAACCTGTCGTGCCAGCTGCATTAATGAATCGGCCAACGCGCGGGGAGAGGCGGTTTGCGTATTGGCTAGAGCAGCTTGCCAACATGGTGGAGCACGACACTCTCGTCTACTCCAAGAATATCAAAGATACAGTCTCAGAAGACCAAAGGGCTATTGAGACTTTTCAACAAAGGGTAATATCGGGAAACCTCCTCGGATTCCATTGCCCAGCTATCTGTCACTTCATCAAAAGGACAGTAGAAAAGGAAGGTGGCACCTACAAATGCCATCATTGCGATAAAGGAAAGGCTATCGTTCAAGATGCCTCTGCCGACAGTGGTCCCAAAGATGGACCCCCACCCACGAGGAGCATCGTGGAAAAAGAAGACGTTCCAACCACGTCTTCAAAGCAAGTGGATTGATGTGAACATGGTGGAGCACGACACTCTCGTCTACTCCAAGAATATCAAAGATACAGTCTCAGAAGACCAAAGGGCTATTGAGACTTTTCAACAAAGGGTAATATCGGGAAACCTCCTCGGATTCCATTGCCCAGCTATCTGTCACTTCATCAAAAGGACAGTAGAAAAGGAAGGTGGCACCTACAAATGCCATCATTGCGATAAAGGAAAGGCTATCGTTCAAGATGCCTCTGCCGACAGTGGTCCCAAAGATGGACCCCCACCCACGAGGAGCATCGTGGAAAAAGAAGACGTTCCAACCACGTCTTCAAAGCAAGTGGATTGATGTGATATCTCCACTGACGTAAGGGATGACGCACAATCCCACTATCCTTCGCAAGACCCTTCCTCTATATAAGGAAGTTCATTTCATTTGGAGAGGACACGCTGAAATCACCAGTCTCTCTCTACAAATCTATCTCTCTCGAGCTTTCGCAGATCCGGGGGGCAATGAGATATGAAAAAGCCTGAACTCACCGCGACGTCTGTCGAGAAGTTTCTGATCGAAAAGTTCGACAGCGTCTCCGACCTGATGCAGCTCTCGGAGGGCGAAGAATCTCGTGCTTTCAGCTTCGATGTAGGAGGGCGTGGATATGTCCTGCGGGTAAATAGCTGCGCCGATGGTTTCTACAAAGATCGTTATGTTTATCGGCACTTTGCATCGGCCGCGCTCCCGATTCCGGAAGTGCTTGACATTGGGGAGTTTAGCGAGAGCCTGACCTATTGCATCTCCCGCCGTTCACAGGGTGTCACGTTGCAAGACCTGCCTGAAACCGAACTGCCCGCTGTTCTACAACCGGTCGCGGAGGCTATGGATGCGATCGCTGCGGCCGATCTTAGCCAGACGAGCGGGTTCGGCCCATTCGGACCGCAAGGAATCGGTCAATACACTACATGGCGTGATTTCATATGCGCGATTGCTGATCCCCATGTGTATCACTGGCAAACTGTGATGGACGACACCGTCAGTGCGTCCGTCGCGCAGGCTCTCGATGAGCTGATGCTTTGGGCCGAGGACTGCCCCGAAGTCCGGCACCTCGTGCACGCGGATTTCGGCTCCAACAATGTCCTGACGGACAATGGCCGCATAACAGCGGTCATTGACTGGAGCGAGGCGATGTTCGGGGATTCCCAATACGAGGTCGCCAACATCTTCTTCTGGAGGCCGTGGTTGGCTTGTATGGAGCAGCAGACGCGCTACTTCGAGCGGAGGCATCCGGAGCTTGCAGGATCGCCACGACTCCGGGCGTATATGCTCCGCATTGGTCTTGACCAACTCTATCAGAGCTTGGTTGACGGCAATTTCGATGATGCAGCTTGGGCGCAGGGTCGATGCGACGCAATCGTCCGATCCGGAGCCGGGACTGTCGGGCGTACACAAATCGCCCGCAGAAGCGCGGCCGTCTGGACCGATGGCTGTGTAGAAGTACTCGCCGATAGTGGAAACCGACGCCCCAGCACTCGTCCGAGGGCAAAGAAATAGAGTAGATGCCGACCGGGATCTGTCGATCGACAAGCTCGAGTTTCTCCATAATAATGTGTGAGTAGTTCCCAGATAAGGGAATTAGGGTTCCTATAGGGTTTCGCTCATGTGTTGAGCATATAAGAAACCCTTAGTATGTATTTGTATTTGTAAAATACTTCTATCAATAAAATTTCTAATTCCTAAAACCAAAATCCAGTACTAAAATCCAGATCCCCCGAATTAATTCGGCGTTAATTCAGTACATTAAAAACGTCCGCAATGTGTTATTAAGTTGTCTAAGCGTCAATTTGTTTACACCACAATATATCCTGCCA

**Sequence 82 Plasmids sequence of pH-35C-Csy4-RERV-m5-OsACC1 */OsCDC48/OsEPSPS***

(RB T-DNA repeat-35C promoter-Csy4 RS-*OsACC1*-pegRNA-Csy4 RS-*OsCDC48*-pegRNA-Csy4 RS-*OsEPSPS*-pegRNA-Csy4 RS-Ubi promoter-Csy4-P2A-NLSc-myc-NLSsv40-nCas9 (H840A/R221K/N394K)-XTEN-NC-NLS-32aa Linker-RERV-RT-m5-NLSvbp-HygR cassette-E9-terminator-CaMV poly(A) signal-LB T-DNA repeat)

GTTTACCCGCCAATATATCCTGTCAAACACTGATAGTTTAAACTGAAGGCGGGAAACGACAATCTGATCCAAGCTCAAGCTGCTCTAGCATTCGCCATTCAGGCTGCGCAACTGTTGGGAAGGGCGATCGGTGCGGGCCTCTTCGCTATTACGCCAGCTGGCGAAAGGGGGATGTGCTGCAAGGCGATTAAGTTGGGTAACGCCAGGGTTTTCCCAGTCACGACGTTGTAAAACGACGGCCAGTGAATTCATGGAGTCAAAGATTCAAATAGAGGACCTAACAGAACTCGCCGTAAAGACTGGCGAACAGTTCATACAGAGTCTCTTACGACTCAATGACAAGAAGAAAATCTTCGTCAACATGGTGGAGCACGACACACTTGTCTACTCCAAAAATATCAAAGATACAGTCTCAGAAGACCAAAGGGCAATTGAGACTTTTCAACAAAGGGTAATATCCGGAAACCTCCTCGGATTCCATTGCCCAGCTATCTGTCACTTTATTGTGAAGATAGTGGAAAAGGAAGGTGGCTCCTACAAATGCCATCATTGCGATAAAGGAAAGGCCATCGTTGAAGATGCCTCTGCCGACAGTGGTCCCAAAGATGGACCCCCACCCACGAGGAGCATCGTGGAAAAAGAAGACGTTCCAACCACGTCTTCAAAGCAAGTGGATTGATGTGATTGGCAGACATACTGTCCCACAAATGAAGATGGAATCTGTAAAAGAAAACGCGTGAAATAATGCGTCTGACAAAGGTTAGGTCGGCTGCCTTTAATCAATACCAAAGTGGTCCCTACCACGATGGAAAAACTGTGCAGTCGGTTTGGCTTTTTCTGACGAACAAATAAGATTCGTGGCCGACAGGTGGGGGTCCACCATGTGAAGGCATCTTCAGACTCCAATAATGGAGCAATGACGTAAGGGCTTACGAAATAAGTAAGGGTAGTTTGGGAAATGTCCACTCACCCGTCAGTCTATAAATACTTAGCCCCTCCCTCATTGTTAAGGGAGCAAAATCTCAGAGAGATAGTCCTAGAGAGAGAAAGAGAGCAAGTAGCCTAGAAGTAGTCAAGGCGGCGAAGTATTCAGGCACGTGGCCAGGAAGAAGAAAAGCCAAGACGACGAAAACAGGTAAGAGCTAAGCATCTAGATAAGTTGAAAACAATCTTCAAAAGTCCCACATCGCTTAGATAAGAAAACGAAGCTGAGTTTATATACAGCTAGAGTCGAAGTAGTGATTGAACAAAGGTTCACTGCCGTATAGGCAGTTCCTCGTGCTGGACAAGTGGTTTAAGAGCTATGCTGGAAACAGCATAGCAAGTTTAAATAAGGCTAGTCCGTTATCAACTTGAAAAAGTGGCACCGAGTCGGTGCATCTGGAAAGCACACTTGTCCAGCACGCGGTTCTATCTAGTTACGCGTTAAACCAACTAGAATTTTTTTGTTCACTGCCGTATAGGCAGGAAGGGGTCAGCGGCGGCGCGTTTAAGAGCTATGCTGGAAACAGCATAGCAAGTTTAAATAAGGCTAGTCCGTTATCAACTTGAAAAAGTGGCACCGAGTCGGTGCCAGCGTCTGGCGCAGGCGCCGCCGCTCGCGGTTCTATCTAGTTACGCGTTAAACCAACTAGAATTTTTTTGTTCACTGCCGTATAGGCAGGCAGTCACGGCTGCTGTCAAGTTTAAGAGCTATGCTGGAAACAGCATAGCAAGTTTAAATAAGGCTAGTCCGTTATCAACTTGAAAAAGTGGCACCGAGTCGGTGCACGCTGGAACTGCAATGCGATCATTGACAGCAGCCCGCGGTTCTATCTAGTTACGCGTTAAACCAACTAGAATTTTTTTGATATCTCCGGGGCTAATTGAATATGAAGATGAAGATGAAATATTTGGTGTGTCAAATAAAAAGCTGGTGTGCTTAAGTTTGTGTTTTTTTCTTGGCTTGTTGTGTTATGAATTTGTGGCTTTTTCTAATATTAAATGAATGTAAGATCTCATTATAATGAATAAACAAATGTTTCTATAATCCATTGTGAATGTTTTGTTGGATCTCTTCTGCAGCATATAACTACTGTATGTGCTATGGTATGGACTATGGAATATGATTAAAGATAAGAAGCTTGGCGTAATCTGCATGCCTGCAGTGCAGCGTGACCCGGTCGTGCCCCTCTCTAGAGATAATGAGCATTGCATGTCTAAGTTATAAAAAATTACCACATATTTTTTTTGTCACACTTGTTTGAAGTGCAGTTTATCTATCTTTATACATATATTTAAACTTTACTCTACGAATAATATAATCTATAGTACTACAATAATATCAGTGTTTTAGAGAATCATATAAATGAACAGTTAGACATGGTCTAAAGGACAATTGAGTATTTTGACAACAGGACTCTACAGTTTTATCTTTTTAGTGTGCATGTGTTCTCCTTTTTTTTTGCAAATAGCTTCACCTATATAATACTTCATCCATTTTATTAGTACATCCATTTAGGGTTTAGGGTTAATGGTTTTTATAGACTAATTTTTTTAGTACATCTATTTTATTCTATTTTAGCCTCTAAATTAAGAAAACTAAAACTCTATTTTAGTTTTTTTATTTAATAATTTAGATATAAAATAGAATAAAATAAAGTGACTAAAAATTAAACAAATACCCTTTAAGAAATTAAAAAAACTAAGGAAACATTTTTCTTGTTTCGAGTAGATAATGCCAGCCTGTTAAACGCCGTCGACGAGTCTAACGGACACCAACCAGCGAACCAGCAGCGTCGCGTCGGGCCAAGCGAAGCAGACGGCACGGCATCTCTGTCGCTGCCTCTGGACCCCTCTCGAGAGTTCCGCTCCACCGTTGGACTTGCTCCGCTGTCGGCATCCAGAAATTGCGTGGCGGAGCGGCAGACGTGAGCCGGCACGGCAGGCGGCCTCCTCCTCCTCTCACGGCACCGGCAGCTACGGGGGATTCCTTTCCCACCGCTCCTTCGCTTTCCCTTCCTCGCCCGCCGTAATAAATAGACACCCCCTCCACACCCTCTTTCCCCAACCTCGTGTTGTTCGGAGCGCACACACACACAACCAGATCTCCCCCAAATCCACCCGTCGGCACCTCCGCTTCAAGGTACGCCGCTCGTCCTCCCCCCCCCCCCCTCTCTACCTTCTCTAGATCGGCGTTCCGGTCCATGGTTAGGGCCCGGTAGTTCTACTTCTGTTCATGTTTGTGTTAGATCCGTGTTTGTGTTAGATCCGTGCTGCTAGCGTTCGTACACGGATGCGACCTGTACGTCAGACACGTTCTGATTGCTAACTTGCCAGTGTTTCTCTTTGGGGAATCCTGGGATGGCTCTAGCCGTTCCGCAGACGGGATCGATTTCATGATTTTTTTTGTTTCGTTGCATAGGGTTTGGTTTGCCCTTTTCCTTTATTTCAATATATGCCGTGCACTTGTTTGTCGGGTCATCTTTTCATGCTTTTTTTTGTCTTGGTTGTGATGATGTGGTCTGGTTGGGCGGTCGTTCTAGATCGGAGTAGAATTCTGTTTCAAACTACCTGGTGGATTTATTAATTTTGGATCTGTATGTGTGTGCCATACATATTCATAGTTACGAATTGAAGATGATGGATGGAAATATCGATCTAGGATAGGTATACATGTTGATGCGGGTTTTACTGATGCATATACAGAGATGCTTTTTGTTCGCTTGGTTGTGATGATGTGGTGTGGTTGGGCGGTCGTTCATTCGTTCTAGATCGGAGTAGAATACTGTTTCAAACTACCTGGTGTATTTATTAATTTTGGAACTGTATGTGTGTGTCATACATCTTCATAGTTACGAGTTTAAGATGGATGGAAATATCGATCTAGGATAGGTATACATGTTGATGTGGGTTTTACTGATGCATATACATGATGGCATATGCAGCATCTATTCATATGCTCTAACCTTGAGTACCTATCTATTATAATAAACAAGTATGTTTTATAATTATTTTGATCTTGATATACTTGGATGATGGCATATGCAGCAGCTATATGTGGATTTTTTTAGCCCTGCCTTCATACGCTATTTATTTGCTTGGTACTGTTTCTTTTGTCGATGCTCACCCTGTTGTTTGGTGTTACTTCTGCAGCCCTAGGACTGGTTGTACAATGATGGACCACTACCTCGACATCAGGCTCAGGCCAGACCCAGAGTTCCCACCAGCCCAGCTCATGTCCGTCCTCTTCGGCAAGCTCCACCAGGCCCTCGTGGCCCAGGGCGGCGACAGGATCGGCGTGTCCTTCCCAGACCTCGACGAGTCCAGGTCCAGGCTCGGCGAGAGGCTCCGCATCCACGCCTCCGCCGACGACCTCAGGGCCCTCCTCGCCAGGCCGTGGCTGGAGGGCCTCAGGGACCACCTCCAGTTCGGCGAGCCAGCCGTGGTGCCACACCCAACCCCATACAGGCAAGTGTCCAGGGTGCAAGCCAAGTCCAACCCAGAGAGGCTCAGGAGGAGGCTCATGAGGAGGCACGACCTCTCCGAGGAAGAGGCCAGGAAGCGCATCCCAGACACCGTGGCCAGGGCCCTCGACCTCCCATTCGTGACCCTCAGGTCCCAGTCCACCGGCCAGCACTTCCGCCTCTTCATCAGGCACGGCCCACTCCAGGTGACCGCCGAGGAGGGCGGCTTTACCTGCTACGGCCTCTCCAAGGGCGGCTTCGTGCCGTGGTTCGGCTCCGGCGCCACCAACTTCTCCCTCCTCAAGCAAGCCGGCGACGTGGAGGAGAACCCAGGCCCAATGCCGGCGGCCAAGAGAGTCAAGCTCGACGGCGGGAAGCGCACAGCTGATGGTTCTGAATTCGAGTCCCCTAAGAAAAAGAGAAAAGTGGACAAGAAGTACTCGATCGGCCTCGATATTGGGACTAACTCTGTTGGCTGGGCCGTGATCACCGACGAGTACAAGGTGCCCTCAAAGAAGTTCAAGGTCCTGGGCAACACCGATCGGCATTCCATCAAGAAGAATCTCATTGGCGCTCTCCTGTTCGACAGCGGCGAGACGGCTGAGGCTACGCGGCTCAAGCGCACCGCCCGCAGGCGGTACACGCGCAGGAAGAATCGCATCTGCTACCTGCAGGAGATTTTCTCCAACGAGATGGCGAAGGTTGACGATTCTTTCTTCCACAGGCTGGAGGAGTCATTCCTCGTGGAGGAGGATAAGAAGCACGAGCGGCATCCAATCTTCGGCAACATTGTCGACGAGGTTGCCTACCACGAGAAGTACCCTACGATCTACCATCTGCGGAAGAAGCTCGTGGACTCCACAGATAAGGCGGACCTCCGCCTGATCTACCTCGCTCTGGCCCACATGATTAAGTTCAGGGGCCATTTCCTGATCGAGGGGGATCTCAACCCGGACAATAGCGATGTTGACAAGCTGTTCATCCAGCTCGTGCAGACGTACAACCAGCTCTTCGAGGAGAACCCCATTAATGCGTCAGGCGTCGACGCGAAGGCTATCCTGTCCGCTAGGCTCTCGAAGTCTCGGAAGCTCGAGAACCTGATCGCCCAGCTGCCGGGCGAGAAGAAGAACGGCCTGTTCGGGAATCTCATTGCGCTCAGCCTGGGGCTCACGCCCAACTTCAAGTCGAATTTCGATCTCGCTGAGGACGCCAAGCTGCAGCTCTCCAAGGACACATACGACGATGACCTGGATAACCTCCTGGCCCAGATCGGCGATCAGTACGCGGACCTGTTCCTCGCTGCCAAGAATCTGTCGGACGCCATCCTCCTGTCTGATATTCTCAGGGTGAACACCGAGATTACGAAGGCTCCGCTCTCAGCCTCCATGATCAAGCGCTACGACGAGCACCATCAGGATCTGACCCTCCTGAAGGCGCTGGTCAGGCAGCAGCTCCCCGAGAAGTACAAGGAGATCTTCTTCGATCAGTCGAAGAACGGCTACGCTGGGTACATTGACGGCGGGGCCTCTCAGGAGGAGTTCTACAAGTTCATCAAGCCGATTCTGGAGAAGATGGACGGCACGGAGGAGCTGCTGGTGAAGCTCAAGCGCGAGGACCTCCTGAGGAAGCAGCGGACATTCGATAACGGCAGCATCCCACACCAGATTCATCTCGGGGAGCTGCACGCTATCCTGAGGAGGCAGGAGGACTTCTACCCTTTCCTCAAGGATAACCGCGAGAAGATCGAGAAGATTCTGACTTTCAGGATCCCGTACTACGTCGGCCCACTCGCTAGGGGCAACTCCCGCTTCGCTTGGATGACCCGCAAGTCAGAGGAGACGATCACGCCGTGGAACTTCGAGGAGGTGGTCGACAAGGGCGCTAGCGCTCAGTCGTTCATCGAGAGGATGACGAATTTCGACAAGAACCTGCCAAATGAGAAGGTGCTCCCTAAGCACTCGCTCCTGTACGAGTACTTCACAGTCTACAACGAGCTGACTAAGGTGAAGTATGTGACCGAGGGCATGAGGAAGCCGGCTTTCCTGTCTGGGGAGCAGAAGAAGGCCATCGTGGACCTCCTGTTCAAGACCAACCGGAAGGTCACGGTTAAGCAGCTCAAGGAGGACTACTTCAAGAAGATTGAGTGCTTCGATTCGGTCGAGATCTCTGGCGTTGAGGACCGCTTCAACGCCTCCCTGGGGACCTACCACGATCTCCTGAAGATCATTAAGGATAAGGACTTCCTGGACAACGAGGAGAATGAGGATATCCTCGAGGACATTGTGCTGACACTCACTCTGTTCGAGGACCGGGAGATGATCGAGGAGCGCCTGAAGACTTACGCCCATCTCTTCGATGACAAGGTCATGAAGCAGCTCAAGAGGAGGAGGTACACCGGCTGGGGGAGGCTGAGCAGGAAGCTCATCAACGGCATTCGGGACAAGCAGTCCGGGAAGACGATCCTCGACTTCCTGAAGAGCGATGGCTTCGCGAACCGCAATTTCATGCAGCTGATTCACGATGACAGCCTCACATTCAAGGAGGATATCCAGAAGGCTCAGGTGAGCGGCCAGGGGGACTCGCTGCACGAGCATATCGCGAACCTCGCTGGCTCGCCAGCTATCAAGAAGGGGATTCTGCAGACCGTGAAGGTTGTGGACGAGCTGGTGAAGGTCATGGGCAGGCACAAGCCTGAGAACATCGTCATTGAGATGGCCCGGGAGAATCAGACCACGCAGAAGGGCCAGAAGAACTCACGCGAGAGGATGAAGAGGATCGAGGAGGGCATTAAGGAGCTGGGGTCCCAGATCCTCAAGGAGCACCCGGTGGAGAACACGCAGCTGCAGAATGAGAAGCTCTACCTGTACTACCTCCAGAATGGCCGCGATATGTATGTGGACCAGGAGCTGGATATTAACAGGCTCAGCGATTACGACGTCGATGCCATCGTTCCACAGTCATTCCTGAAGGATGACTCCATTGACAACAAGGTCCTCACCAGGTCGGACAAGAACCGGGGCAAGTCTGATAATGTTCCTTCAGAGGAGGTCGTTAAGAAGATGAAGAACTACTGGCGCCAGCTCCTGAATGCCAAGCTGATCACGCAGCGGAAGTTCGATAACCTCACAAAGGCTGAGAGGGGCGGGCTCTCTGAGCTGGACAAGGCGGGCTTCATCAAGAGGCAGCTGGTCGAGACACGGCAGATCACTAAGCACGTTGCGCAGATTCTCGACTCACGGATGAACACTAAGTACGATGAGAATGACAAGCTGATCCGCGAGGTGAAGGTCATCACCCTGAAGTCAAAGCTCGTCTCCGACTTCAGGAAGGATTTCCAGTTCTACAAGGTTCGGGAGATCAACAATTACCACCATGCCCATGACGCGTACCTGAACGCGGTGGTCGGCACAGCTCTGATCAAGAAGTACCCAAAGCTCGAGAGCGAGTTCGTGTACGGGGACTACAAGGTTTACGATGTGAGGAAGATGATCGCCAAGTCGGAGCAGGAGATTGGCAAGGCTACCGCCAAGTACTTCTTCTACTCTAACATTATGAATTTCTTCAAGACAGAGATCACTCTGGCCAATGGCGAGATCCGGAAGCGCCCCCTCATCGAGACGAACGGCGAGACGGGGGAGATCGTGTGGGACAAGGGCAGGGATTTCGCGACCGTCAGGAAGGTTCTCTCCATGCCACAAGTGAATATCGTCAAGAAGACAGAGGTCCAGACTGGCGGGTTCTCTAAGGAGTCAATTCTGCCTAAGCGGAACAGCGACAAGCTCATCGCCCGCAAGAAGGACTGGGATCCGAAGAAGTACGGCGGGTTCGACAGCCCCACTGTGGCCTACTCGGTCCTGGTTGTGGCGAAGGTTGAGAAGGGCAAGTCCAAGAAGCTCAAGAGCGTGAAGGAGCTGCTGGGGATCACGATTATGGAGCGCTCCAGCTTCGAGAAGAACCCGATCGATTTCCTGGAGGCGAAGGGCTACAAGGAGGTGAAGAAGGACCTGATCATTAAGCTCCCCAAGTACTCACTCTTCGAGCTGGAGAACGGCAGGAAGCGGATGCTGGCTTCCGCTGGCGAGCTGCAGAAGGGGAACGAGCTGGCTCTGCCGTCCAAGTATGTGAACTTCCTCTACCTGGCCTCCCACTACGAGAAGCTCAAGGGCAGCCCCGAGGACAACGAGCAGAAGCAGCTGTTCGTCGAGCAGCACAAGCATTACCTCGACGAGATCATTGAGCAGATTTCCGAGTTCTCCAAGCGCGTGATCCTGGCCGACGCGAATCTGGATAAGGTCCTCTCCGCGTACAACAAGCACCGCGACAAGCCAATCAGGGAGCAGGCTGAGAATATCATTCATCTCTTCACCCTGACGAACCTCGGCGCCCCTGCTGCTTTCAAGTACTTCGACACAACTATCGATCGCAAGAGGTACACAAGCACTAAGGAGGTCCTGGACGCGACCCTCATCCACCAGTCGATTACCGGCCTCTACGAGACGCGCATCGACCTGTCTCAGCTCGGGGGCGACGAATTCTCCGGGAGCGAGACGCCAGGCACCTCCGAGTCGGCCACCCCAGAATCTGCCACAGTGGTGTCCGGCCAAAAGCAGGACCGCCAGGGCGGAGAACGCAGAAGGTCCCAGCTCGATAGGGATCAGTGTGCCTACTGCAAGGAGAAGGGCCACTGGGCCAAAGACTGCCCGAAAAAGCCGCGCGGCCCACGCGGCCCAAGGCCACAAACATCCCTCCTTCCAAAGAAGAAGCGGAAGGTGGAGCTCAGCGGAGGATCTTCCGGAGGATCTAGCGGCTCCGAGACACCAGGAACATCCGAAAGCGCTACACCAGAATCTAGCGGAGGCTCTTCCGGAGGATCTAGGCCTCCTCCCGTCGCGTGCCTCGTACTGAGCCTAGAGGAGGAGTACAGGCTGCATGAGCAAAATCCGAAGCAGCTGCTAGCCCCTGAATGGCTGAGCGCCTTCTCCGAGGTATGGGCGGAGCAGACTGGCATGGGACTGGCCAAGCAGGTGCCTCCTGTGGTGGTAGAGCTGAAGGCGGACGCCAGCCCAGTGAGCGTGAAACAATACCCGATGTCGAGGGAAGCAAAGGAAGGCATTAGACCCCACATCCAGAGACTGCTGCAATTGGGAATCCTGGTACCCTGCCAAAGCCCTTGGAACACGCCACTGCTGCCTGTGCGCAAACCAGGAACTAATGATTACAGACCTGTACAGGATCTAAGGGAAGTTAACAAGCGGGTGCAGGACATACATCCTACGGTCCCAAACCCCTACAACTTGCTGAGCTCTCTGCCCCCCGAGCGCACATGGTACACTGTGCTCGACTTAAAAGATGCGTTCTTCTGCCTTCGCCTCCACCCGAACTCACAGCCACTGTTCGCGTTCGAGTGGCGCGACCCTGAGGGCGGCCACACCGGGCAACTGACGTGGACACGGCTGCCACAGGGTTTCAAGAACAGCCCTACTCTATTTAACGAGGCCCTGCACCGTGATCTGGCCCCCTTCCGGGCGCAGAACCCTCAAATCAGTCTACTGCAGTATGCGGATGACCTGCTGCTAGCCGCTTCTACTCAGGAGCTCTGCCGGGAGGGCACAAAGCGGCTGTTAAATGAGCTGGGCGAGCTGGGATACCGCGTGTCTGCCAAGAAGGCACAATTGTGTCGGACAGAGGTGACATATCTCGGCTACACATTAAGAGAAGGTAAGAGATGGCTCACTGAAGCACGTAAGAAGACTGTCATGCAGATTCCTACCCCTACCACGCCCCGTCAGGTGCGGGAGTTCCTCGGCAAGGCCGGCTTCTGTAGACTGTTCATTCCTGGATTTGCCACCCTGGCCGCCCCACTCTACCTGCTGACGAAGCCAAAGGTTCCTTTCACCTGGACTGAGGAACATCAGCGGGCTTTCGAAGACATCAAAGCCGCCCTGCTGGCGGCTCCAGCTCTGGCCCTCCCTGACCTCACCAAGCCCTTCACACTCTATGTGGACGAGAGAGCTGGAGTGGCCAGAGGTGTGCTGACACAGGCACTGGGGCCGTGGAAGAGACCCGTGGCGTACCTATCTAAAAAGCTCGACCCCGTGGCCTCTGGGTGGCCGAGCTGTCTAAAGGCCATCGCTGCTGTAGCTCTACTGGTCAAAGATGCTGACAAGCTGACCCTGGGCCAGCATGTCACCGTCATCGCCCCGCACGCCTTAGAATCCATAGTCCGGCAACCACCAGATCGGTGGATGACAAACGCTAGGATGACGCATTATCAGAGCTTGCTGTTAAACGAAAGAGTGACATTTGCGCCCCCTGCCATCCTTAACCCAGCGACGCTACTGCCGGAGATCCACAACTCAACCCCCATCCACCAGTGCGTCGACATTCTGGCTGAGGAGACCGGCACGCGCAAGGACCTCACGGACAGACCATGGCCTGGTGTGCCGGCATGGTACACCGACGGCAGCTCCTTTGTCGTGGAGGGAAAGCGACGCGCAGGCGCGGCTGTGGTGGACGGCAAGCAAGTCATCTGGGCATCCTCCCTGCCAGAAGGCACCAGCGCTCAGAAAGCGGAGTTGGTGGCCTTGACCCAGGCCCTCAGATTAGCTGAAGGGAAGGCTATTAATATCTACACCGATTCTAGATACGCCTTCGCCACGGCGCACATCCACGGCGCGATCTACAAGCAGCGGGGCCTGCTGACCAGTGCCGGGCGAGACATCAAGAATAAGGAAGAGATTCTTGCCCTGCTGGAGGCCGTCCACCTCCCCAAAAAGGTGGCCATTATCCACTGCCCCGGCCACCAGAAGGGAGAGGATCCAATTACAAAGGGGAACCAGATGGCCGACCTCGTGGCGAAGCAGGTCGCTCAGCAGGTTACCATCCTGGCAGAAAAATCGCAGACCCCTGTGAAGACACCTGTGACAGATGATTCTAACTATAGCGGCGGCAGCAAAAGAACGGCGGACGGCTCTGAGAAGCGCACCGCTGATAGCCAGCATTCAACTCCTCCGAAAACAAAGAGGAAAGTTGAGTTCGAACCGAAGAAGAAAAGGAAGGTGTGAGAGCTCAGAGCTTTCGTTCGTATCATCGGTTTCGACAACGTTCGTCAAGTTCAATGCATCAGTTTCATTGCGCACACACCAGAATCCTACTGAGTTTGAGTATTATGGCATTGGGAAAACTGTTTTTCTTGTACCATTTGTTGTGCTTGTAATTTACTGTGTTTTTTATTCGGTTTTCGCTATCGAACTGTGAAATGGAAATGGATGGAGAAGAGTTAATGAATGATATGGTCCTTTTGTTCATTCTCAAATTAATATTATTTGTTTTTTCTCTTATTTGTTGTGTGTTGAATTTGAAATTATAAGAGATATGCAAACATTTTGTTTTGAGTAAAAATGTGTCAAATCGTGGCCTCTAATGACCGAAGTTAATATGAGGAGTAAAACACTTGTAGTTGTACCATTATGCTTATTCACTAGGCAACAAATATATTTTCAGACCTAGAAAAGCTGCAAATGTTACTGAATACAAGTATGTCCTCTTGTGTTTTAGACATTTATGAACTTTCCTTTATGTAATTTTCCAGAATCCTTGTCAGATTCTAATCATTGCTTTATAATTATAGTTATACTCATGGATTTGTAGTTGAGTATGAAAATATTTTTTAATGCATTTTATGACTTGCCAATTGATTGACAACGAATTCGTAATCATGTCATAGCTGTTTCCTGTGTGAAATTGTTATCCGCTCACAATTCCACACAACATACGAGCCGGAAGCATAAAGTGTAAAGCCTGGGGTGCCTAATGAGTGAGCTAACTCACATTAATTGCGTTGCGCTCACTGCCCGCTTTCCAGTCGGGAAACCTGTCGTGCCAGCTGCATTAATGAATCGGCCAACGCGCGGGGAGAGGCGGTTTGCGTATTGGCTAGAGCAGCTTGCCAACATGGTGGAGCACGACACTCTCGTCTACTCCAAGAATATCAAAGATACAGTCTCAGAAGACCAAAGGGCTATTGAGACTTTTCAACAAAGGGTAATATCGGGAAACCTCCTCGGATTCCATTGCCCAGCTATCTGTCACTTCATCAAAAGGACAGTAGAAAAGGAAGGTGGCACCTACAAATGCCATCATTGCGATAAAGGAAAGGCTATCGTTCAAGATGCCTCTGCCGACAGTGGTCCCAAAGATGGACCCCCACCCACGAGGAGCATCGTGGAAAAAGAAGACGTTCCAACCACGTCTTCAAAGCAAGTGGATTGATGTGAACATGGTGGAGCACGACACTCTCGTCTACTCCAAGAATATCAAAGATACAGTCTCAGAAGACCAAAGGGCTATTGAGACTTTTCAACAAAGGGTAATATCGGGAAACCTCCTCGGATTCCATTGCCCAGCTATCTGTCACTTCATCAAAAGGACAGTAGAAAAGGAAGGTGGCACCTACAAATGCCATCATTGCGATAAAGGAAAGGCTATCGTTCAAGATGCCTCTGCCGACAGTGGTCCCAAAGATGGACCCCCACCCACGAGGAGCATCGTGGAAAAAGAAGACGTTCCAACCACGTCTTCAAAGCAAGTGGATTGATGTGATATCTCCACTGACGTAAGGGATGACGCACAATCCCACTATCCTTCGCAAGACCCTTCCTCTATATAAGGAAGTTCATTTCATTTGGAGAGGACACGCTGAAATCACCAGTCTCTCTCTACAAATCTATCTCTCTCGAGCTTTCGCAGATCCGGGGGGCAATGAGATATGAAAAAGCCTGAACTCACCGCGACGTCTGTCGAGAAGTTTCTGATCGAAAAGTTCGACAGCGTCTCCGACCTGATGCAGCTCTCGGAGGGCGAAGAATCTCGTGCTTTCAGCTTCGATGTAGGAGGGCGTGGATATGTCCTGCGGGTAAATAGCTGCGCCGATGGTTTCTACAAAGATCGTTATGTTTATCGGCACTTTGCATCGGCCGCGCTCCCGATTCCGGAAGTGCTTGACATTGGGGAGTTTAGCGAGAGCCTGACCTATTGCATCTCCCGCCGTTCACAGGGTGTCACGTTGCAAGACCTGCCTGAAACCGAACTGCCCGCTGTTCTACAACCGGTCGCGGAGGCTATGGATGCGATCGCTGCGGCCGATCTTAGCCAGACGAGCGGGTTCGGCCCATTCGGACCGCAAGGAATCGGTCAATACACTACATGGCGTGATTTCATATGCGCGATTGCTGATCCCCATGTGTATCACTGGCAAACTGTGATGGACGACACCGTCAGTGCGTCCGTCGCGCAGGCTCTCGATGAGCTGATGCTTTGGGCCGAGGACTGCCCCGAAGTCCGGCACCTCGTGCACGCGGATTTCGGCTCCAACAATGTCCTGACGGACAATGGCCGCATAACAGCGGTCATTGACTGGAGCGAGGCGATGTTCGGGGATTCCCAATACGAGGTCGCCAACATCTTCTTCTGGAGGCCGTGGTTGGCTTGTATGGAGCAGCAGACGCGCTACTTCGAGCGGAGGCATCCGGAGCTTGCAGGATCGCCACGACTCCGGGCGTATATGCTCCGCATTGGTCTTGACCAACTCTATCAGAGCTTGGTTGACGGCAATTTCGATGATGCAGCTTGGGCGCAGGGTCGATGCGACGCAATCGTCCGATCCGGAGCCGGGACTGTCGGGCGTACACAAATCGCCCGCAGAAGCGCGGCCGTCTGGACCGATGGCTGTGTAGAAGTACTCGCCGATAGTGGAAACCGACGCCCCAGCACTCGTCCGAGGGCAAAGAAATAGAGTAGATGCCGACCGGGATCTGTCGATCGACAAGCTCGAGTTTCTCCATAATAATGTGTGAGTAGTTCCCAGATAAGGGAATTAGGGTTCCTATAGGGTTTCGCTCATGTGTTGAGCATATAAGAAACCCTTAGTATGTATTTGTATTTGTAAAATACTTCTATCAATAAAATTTCTAATTCCTAAAACCAAAATCCAGTACTAAAATCCAGATCCCCCGAATTAATTCGGCGTTAATTCAGTACATTAAAAACGTCCGCAATGTGTTATTAAGTTGTCTAAGCGTCAATTTGTTTACACCACAATATATCCTGCCA

**Sequence 83 Plasmids sequence of pH-35C-enRERV-OsCDC48**

(RB T-DNA repeat-35C promoter-tRNAGly RS-pegRNA-Ubi promoter-NLSc-myc-NLSsv40-nCas9(H840A/ R221K/N394K)-XTEN-NC-NLS-32aa Linker-enRERV-RT-NLSvbp-HygR cassette-E9-terminator-CaMV poly(A) signal-LB T-DNA repeat)

GTTTACCCGCCAATATATCCTGTCAAACACTGATAGTTTAAACTGAAGGCGGGAAACGACAATCTGATCCAAGCTCAAGCTGCTCTAGCATTCGCCATTCAGGCTGCGCAACTGTTGGGAAGGGCGATCGGTGCGGGCCTCTTCGCTATTACGCCAGCTGGCGAAAGGGGGATGTGCTGCAAGGCGATTAAGTTGGGTAACGCCAGGGTTTTCCCAGTCACGACGTTGTAAAACGACGGCCAGTGAATTCATGGAGTCAAAGATTCAAATAGAGGACCTAACAGAACTCGCCGTAAAGACTGGCGAACAGTTCATACAGAGTCTCTTACGACTCAATGACAAGAAGAAAATCTTCGTCAACATGGTGGAGCACGACACACTTGTCTACTCCAAAAATATCAAAGATACAGTCTCAGAAGACCAAAGGGCAATTGAGACTTTTCAACAAAGGGTAATATCCGGAAACCTCCTCGGATTCCATTGCCCAGCTATCTGTCACTTTATTGTGAAGATAGTGGAAAAGGAAGGTGGCTCCTACAAATGCCATCATTGCGATAAAGGAAAGGCCATCGTTGAAGATGCCTCTGCCGACAGTGGTCCCAAAGATGGACCCCCACCCACGAGGAGCATCGTGGAAAAAGAAGACGTTCCAACCACGTCTTCAAAGCAAGTGGATTGATGTGATTGGCAGACATACTGTCCCACAAATGAAGATGGAATCTGTAAAAGAAAACGCGTGAAATAATGCGTCTGACAAAGGTTAGGTCGGCTGCCTTTAATCAATACCAAAGTGGTCCCTACCACGATGGAAAAACTGTGCAGTCGGTTTGGCTTTTTCTGACGAACAAATAAGATTCGTGGCCGACAGGTGGGGGTCCACCATGTGAAGGCATCTTCAGACTCCAATAATGGAGCAATGACGTAAGGGCTTACGAAATAAGTAAGGGTAGTTTGGGAAATGTCCACTCACCCGTCAGTCTATAAATACTTAGCCCCTCCCTCATTGTTAAGGGAGCAAAATCTCAGAGAGATAGTCCTAGAGAGAGAAAGAGAGCAAGTAGCCTAGAAGTAGTCAAGGCGGCGAAGTATTCAGGCACGTGGCCAGGAAGAAGAAAAGCCAAGACGACGAAAACAGGTAAGAGCTAAGCATCTAGATAAGTTGAAAACAATCTTCAAAAGTCCCACATCGCTTAGATAAGAAAACGAAGCTGAGTTTATATACAGCTAGAGTCGAAGTAGTGATTGAACAAAGCACCAGTGGTCTAGTGGTAGAATAGTACCCTGCCACGGTACAGACCCGGGTTCGATTCCCGGCTGGTGCAGAAGGGGTCAGCGGCGGCGCGTTTAAGAGCTATGCTGGAAACAGCATAGCAAGTTTAAATAAGGCTAGTCCGTTATCAACTTGAAAAAGTGGCACCGAGTCGGTGCCAGCGTCTGGCGCAGGCGCCGCCGCTCGCGGTTCTATCTAGTTACGCGTTAAACCAACTAGAATTTTTTTGGCCGGCATGGTCCCAGCCTCCTCGCTGGCGCCGGCTGGGCAACATGCTTCGGCATGGCGAATGGGACTTTTTTTTGATATCTCCGGGGCTAATTGAATATGAAGATGAAGATGAAATATTTGGTGTGTCAAATAAAAAGCTGGTGTGCTTAAGTTTGTGTTTTTTTCTTGGCTTGTTGTGTTATGAATTTGTGGCTTTTTCTAATATTAAATGAATGTAAGATCTCATTATAATGAATAAACAAATGTTTCTATAATCCATTGTGAATGTTTTGTTGGATCTCTTCTGCAGCATATAACTACTGTATGTGCTATGGTATGGACTATGGAATATGATTAAAGATAAGAAGCTTGGCGTAATCTGCATGCCTGCAGTGCAGCGTGACCCGGTCGTGCCCCTCTCTAGAGATAATGAGCATTGCATGTCTAAGTTATAAAAAATTACCACATATTTTTTTTGTCACACTTGTTTGAAGTGCAGTTTATCTATCTTTATACATATATTTAAACTTTACTCTACGAATAATATAATCTATAGTACTACAATAATATCAGTGTTTTAGAGAATCATATAAATGAACAGTTAGACATGGTCTAAAGGACAATTGAGTATTTTGACAACAGGACTCTACAGTTTTATCTTTTTAGTGTGCATGTGTTCTCCTTTTTTTTTGCAAATAGCTTCACCTATATAATACTTCATCCATTTTATTAGTACATCCATTTAGGGTTTAGGGTTAATGGTTTTTATAGACTAATTTTTTTAGTACATCTATTTTATTCTATTTTAGCCTCTAAATTAAGAAAACTAAAACTCTATTTTAGTTTTTTTATTTAATAATTTAGATATAAAATAGAATAAAATAAAGTGACTAAAAATTAAACAAATACCCTTTAAGAAATTAAAAAAACTAAGGAAACATTTTTCTTGTTTCGAGTAGATAATGCCAGCCTGTTAAACGCCGTCGACGAGTCTAACGGACACCAACCAGCGAACCAGCAGCGTCGCGTCGGGCCAAGCGAAGCAGACGGCACGGCATCTCTGTCGCTGCCTCTGGACCCCTCTCGAGAGTTCCGCTCCACCGTTGGACTTGCTCCGCTGTCGGCATCCAGAAATTGCGTGGCGGAGCGGCAGACGTGAGCCGGCACGGCAGGCGGCCTCCTCCTCCTCTCACGGCACCGGCAGCTACGGGGGATTCCTTTCCCACCGCTCCTTCGCTTTCCCTTCCTCGCCCGCCGTAATAAATAGACACCCCCTCCACACCCTCTTTCCCCAACCTCGTGTTGTTCGGAGCGCACACACACACAACCAGATCTCCCCCAAATCCACCCGTCGGCACCTCCGCTTCAAGGTACGCCGCTCGTCCTCCCCCCCCCCCCCTCTCTACCTTCTCTAGATCGGCGTTCCGGTCCATGGTTAGGGCCCGGTAGTTCTACTTCTGTTCATGTTTGTGTTAGATCCGTGTTTGTGTTAGATCCGTGCTGCTAGCGTTCGTACACGGATGCGACCTGTACGTCAGACACGTTCTGATTGCTAACTTGCCAGTGTTTCTCTTTGGGGAATCCTGGGATGGCTCTAGCCGTTCCGCAGACGGGATCGATTTCATGATTTTTTTTGTTTCGTTGCATAGGGTTTGGTTTGCCCTTTTCCTTTATTTCAATATATGCCGTGCACTTGTTTGTCGGGTCATCTTTTCATGCTTTTTTTTGTCTTGGTTGTGATGATGTGGTCTGGTTGGGCGGTCGTTCTAGATCGGAGTAGAATTCTGTTTCAAACTACCTGGTGGATTTATTAATTTTGGATCTGTATGTGTGTGCCATACATATTCATAGTTACGAATTGAAGATGATGGATGGAAATATCGATCTAGGATAGGTATACATGTTGATGCGGGTTTTACTGATGCATATACAGAGATGCTTTTTGTTCGCTTGGTTGTGATGATGTGGTGTGGTTGGGCGGTCGTTCATTCGTTCTAGATCGGAGTAGAATACTGTTTCAAACTACCTGGTGTATTTATTAATTTTGGAACTGTATGTGTGTGTCATACATCTTCATAGTTACGAGTTTAAGATGGATGGAAATATCGATCTAGGATAGGTATACATGTTGATGTGGGTTTTACTGATGCATATACATGATGGCATATGCAGCATCTATTCATATGCTCTAACCTTGAGTACCTATCTATTATAATAAACAAGTATGTTTTATAATTATTTTGATCTTGATATACTTGGATGATGGCATATGCAGCAGCTATATGTGGATTTTTTTAGCCCTGCCTTCATACGCTATTTATTTGCTTGGTACTGTTTCTTTTGTCGATGCTCACCCTGTTGTTTGGTGTTACTTCTGCAGCCCTAGGACTGGTTGTACAATGCCGGCGGCCAAGAGAGTCAAGCTCGACGGCGGGAAGCGCACAGCTGATGGTTCTGAATTCGAGTCCCCTAAGAAAAAGAGAAAAGTGGACAAGAAGTACTCGATCGGCCTCGATATTGGGACTAACTCTGTTGGCTGGGCCGTGATCACCGACGAGTACAAGGTGCCCTCAAAGAAGTTCAAGGTCCTGGGCAACACCGATCGGCATTCCATCAAGAAGAATCTCATTGGCGCTCTCCTGTTCGACAGCGGCGAGACGGCTGAGGCTACGCGGCTCAAGCGCACCGCCCGCAGGCGGTACACGCGCAGGAAGAATCGCATCTGCTACCTGCAGGAGATTTTCTCCAACGAGATGGCGAAGGTTGACGATTCTTTCTTCCACAGGCTGGAGGAGTCATTCCTCGTGGAGGAGGATAAGAAGCACGAGCGGCATCCAATCTTCGGCAACATTGTCGACGAGGTTGCCTACCACGAGAAGTACCCTACGATCTACCATCTGCGGAAGAAGCTCGTGGACTCCACAGATAAGGCGGACCTCCGCCTGATCTACCTCGCTCTGGCCCACATGATTAAGTTCAGGGGCCATTTCCTGATCGAGGGGGATCTCAACCCGGACAATAGCGATGTTGACAAGCTGTTCATCCAGCTCGTGCAGACGTACAACCAGCTCTTCGAGGAGAACCCCATTAATGCGTCAGGCGTCGACGCGAAGGCTATCCTGTCCGCTAGGCTCTCGAAGTCTCGGAAGCTCGAGAACCTGATCGCCCAGCTGCCGGGCGAGAAGAAGAACGGCCTGTTCGGGAATCTCATTGCGCTCAGCCTGGGGCTCACGCCCAACTTCAAGTCGAATTTCGATCTCGCTGAGGACGCCAAGCTGCAGCTCTCCAAGGACACATACGACGATGACCTGGATAACCTCCTGGCCCAGATCGGCGATCAGTACGCGGACCTGTTCCTCGCTGCCAAGAATCTGTCGGACGCCATCCTCCTGTCTGATATTCTCAGGGTGAACACCGAGATTACGAAGGCTCCGCTCTCAGCCTCCATGATCAAGCGCTACGACGAGCACCATCAGGATCTGACCCTCCTGAAGGCGCTGGTCAGGCAGCAGCTCCCCGAGAAGTACAAGGAGATCTTCTTCGATCAGTCGAAGAACGGCTACGCTGGGTACATTGACGGCGGGGCCTCTCAGGAGGAGTTCTACAAGTTCATCAAGCCGATTCTGGAGAAGATGGACGGCACGGAGGAGCTGCTGGTGAAGCTCAAGCGCGAGGACCTCCTGAGGAAGCAGCGGACATTCGATAACGGCAGCATCCCACACCAGATTCATCTCGGGGAGCTGCACGCTATCCTGAGGAGGCAGGAGGACTTCTACCCTTTCCTCAAGGATAACCGCGAGAAGATCGAGAAGATTCTGACTTTCAGGATCCCGTACTACGTCGGCCCACTCGCTAGGGGCAACTCCCGCTTCGCTTGGATGACCCGCAAGTCAGAGGAGACGATCACGCCGTGGAACTTCGAGGAGGTGGTCGACAAGGGCGCTAGCGCTCAGTCGTTCATCGAGAGGATGACGAATTTCGACAAGAACCTGCCAAATGAGAAGGTGCTCCCTAAGCACTCGCTCCTGTACGAGTACTTCACAGTCTACAACGAGCTGACTAAGGTGAAGTATGTGACCGAGGGCATGAGGAAGCCGGCTTTCCTGTCTGGGGAGCAGAAGAAGGCCATCGTGGACCTCCTGTTCAAGACCAACCGGAAGGTCACGGTTAAGCAGCTCAAGGAGGACTACTTCAAGAAGATTGAGTGCTTCGATTCGGTCGAGATCTCTGGCGTTGAGGACCGCTTCAACGCCTCCCTGGGGACCTACCACGATCTCCTGAAGATCATTAAGGATAAGGACTTCCTGGACAACGAGGAGAATGAGGATATCCTCGAGGACATTGTGCTGACACTCACTCTGTTCGAGGACCGGGAGATGATCGAGGAGCGCCTGAAGACTTACGCCCATCTCTTCGATGACAAGGTCATGAAGCAGCTCAAGAGGAGGAGGTACACCGGCTGGGGGAGGCTGAGCAGGAAGCTCATCAACGGCATTCGGGACAAGCAGTCCGGGAAGACGATCCTCGACTTCCTGAAGAGCGATGGCTTCGCGAACCGCAATTTCATGCAGCTGATTCACGATGACAGCCTCACATTCAAGGAGGATATCCAGAAGGCTCAGGTGAGCGGCCAGGGGGACTCGCTGCACGAGCATATCGCGAACCTCGCTGGCTCGCCAGCTATCAAGAAGGGGATTCTGCAGACCGTGAAGGTTGTGGACGAGCTGGTGAAGGTCATGGGCAGGCACAAGCCTGAGAACATCGTCATTGAGATGGCCCGGGAGAATCAGACCACGCAGAAGGGCCAGAAGAACTCACGCGAGAGGATGAAGAGGATCGAGGAGGGCATTAAGGAGCTGGGGTCCCAGATCCTCAAGGAGCACCCGGTGGAGAACACGCAGCTGCAGAATGAGAAGCTCTACCTGTACTACCTCCAGAATGGCCGCGATATGTATGTGGACCAGGAGCTGGATATTAACAGGCTCAGCGATTACGACGTCGATGCCATCGTTCCACAGTCATTCCTGAAGGATGACTCCATTGACAACAAGGTCCTCACCAGGTCGGACAAGAACCGGGGCAAGTCTGATAATGTTCCTTCAGAGGAGGTCGTTAAGAAGATGAAGAACTACTGGCGCCAGCTCCTGAATGCCAAGCTGATCACGCAGCGGAAGTTCGATAACCTCACAAAGGCTGAGAGGGGCGGGCTCTCTGAGCTGGACAAGGCGGGCTTCATCAAGAGGCAGCTGGTCGAGACACGGCAGATCACTAAGCACGTTGCGCAGATTCTCGACTCACGGATGAACACTAAGTACGATGAGAATGACAAGCTGATCCGCGAGGTGAAGGTCATCACCCTGAAGTCAAAGCTCGTCTCCGACTTCAGGAAGGATTTCCAGTTCTACAAGGTTCGGGAGATCAACAATTACCACCATGCCCATGACGCGTACCTGAACGCGGTGGTCGGCACAGCTCTGATCAAGAAGTACCCAAAGCTCGAGAGCGAGTTCGTGTACGGGGACTACAAGGTTTACGATGTGAGGAAGATGATCGCCAAGTCGGAGCAGGAGATTGGCAAGGCTACCGCCAAGTACTTCTTCTACTCTAACATTATGAATTTCTTCAAGACAGAGATCACTCTGGCCAATGGCGAGATCCGGAAGCGCCCCCTCATCGAGACGAACGGCGAGACGGGGGAGATCGTGTGGGACAAGGGCAGGGATTTCGCGACCGTCAGGAAGGTTCTCTCCATGCCACAAGTGAATATCGTCAAGAAGACAGAGGTCCAGACTGGCGGGTTCTCTAAGGAGTCAATTCTGCCTAAGCGGAACAGCGACAAGCTCATCGCCCGCAAGAAGGACTGGGATCCGAAGAAGTACGGCGGGTTCGACAGCCCCACTGTGGCCTACTCGGTCCTGGTTGTGGCGAAGGTTGAGAAGGGCAAGTCCAAGAAGCTCAAGAGCGTGAAGGAGCTGCTGGGGATCACGATTATGGAGCGCTCCAGCTTCGAGAAGAACCCGATCGATTTCCTGGAGGCGAAGGGCTACAAGGAGGTGAAGAAGGACCTGATCATTAAGCTCCCCAAGTACTCACTCTTCGAGCTGGAGAACGGCAGGAAGCGGATGCTGGCTTCCGCTGGCGAGCTGCAGAAGGGGAACGAGCTGGCTCTGCCGTCCAAGTATGTGAACTTCCTCTACCTGGCCTCCCACTACGAGAAGCTCAAGGGCAGCCCCGAGGACAACGAGCAGAAGCAGCTGTTCGTCGAGCAGCACAAGCATTACCTCGACGAGATCATTGAGCAGATTTCCGAGTTCTCCAAGCGCGTGATCCTGGCCGACGCGAATCTGGATAAGGTCCTCTCCGCGTACAACAAGCACCGCGACAAGCCAATCAGGGAGCAGGCTGAGAATATCATTCATCTCTTCACCCTGACGAACCTCGGCGCCCCTGCTGCTTTCAAGTACTTCGACACAACTATCGATCGCAAGAGGTACACAAGCACTAAGGAGGTCCTGGACGCGACCCTCATCCACCAGTCGATTACCGGCCTCTACGAGACGCGCATCGACCTGTCTCAGCTCGGGGGCGACGAATTCTCCGGGAGCGAGACGCCAGGCACCTCCGAGTCGGCCACCCCAGAATCTGCCACAGTGGTGTCCGGCCAAAAGCAGGACCGCCAGGGCGGAGAACGCAGAAGGTCCCAGCTCGATAGGGATCAGTGTGCCTACTGCAAGGAGAAGGGCCACTGGGCCAAAGACTGCCCGAAAAAGCCGCGCGGCCCACGCGGCCCAAGGCCACAAACATCCCTCCTTCCAAAGAAGAAGCGGAAGGTGGAGCTCAGCGGAGGATCTTCCGGAGGATCTAGCGGCTCCGAGACACCAGGAACATCCGAAAGCGCTACACCAGAATCTAGCGGAGGCTCTTCCGGAGGATCTAGGCCTCCTCCCGTCGCGTGCCTCGTACTGAGCCTAGAGGAGGAGTACAGGCTGCATGAGCAAAATCCGAAGCAGCTGCTAGCCCCTGAATGGCTGAGCGCCTTCTCCGAGGTATGGGCGGAGCAGACTGGCATGGGACTGGCCAAGCAGGTGCCTCCTGTGGTGGTAGAGCTGAAGGCGGACGCCAGCCCAGTGAGCGTGAAACAACGCCCGATGTCGAGGGAAGCAAAGGAAGGCATTAGACCCCACATCCAGAGACTGCTGCAATTGGGAATCCTGGTACCCTGCCAAAGCCCTTGGAACACGCCACTGCTGCCTGTGCGCAAACCAGGAACTAATGATTACAGACCTGTACAGGATCTAAGGGAAGTTAACAAGCGGGTGCAGGACATACATCCTACGGTCCCAAACCCCTACAACTTGCTGAGCTCTCTGCCCCCCGAGCGCACAttcTACACTGTGCTCGACTTAAAAGATGCGTTCTTCTGCCTTCGCCTCCACCCGAACTCACAGCCACTGTTCGCGTTCGAGTGGCGCGACCCTGAGGGCGGCCACACCGGGCAACTGACGTGGACACGGCTGCCACAGGGTTTCAAGAACAGCCCTACTCTATTTAACGAGGCCCTGCACCGTGATCTGGCCCCCTTCCGGGCGCAGAACCCTCAAATCAGTCTACTGCAGTATGCGGATGACCTGCTGCTAGCCGCTTCTACTCAGGAGCTCTGCCGGGAGGGCACAAAGCGGCTGTTAAATGAGCTGGGCGAGCTGGGATACCGCGTGTCTGCCAAGAAGGCACAATTGTGTCGGACAGAGGTGACATATCTCGGCTACACATTAAGAGAAGGTAAGAGATGGCTCACTGAAGCACGTAAGAAGACTGTCATGCAGATTCCTACCCCTACCACGCCCCGTCAGGTGCGGGAGTTCCTCGGCAAGGCCGGCTTCTGTAGACTGTTtATTCCTGGATTTGCCACCCTGGCCGCCCCACTCTACCTGCTGACGAAGCCAAAGGTTCCTTTCACCTGGACTGAGGAACATCAGCGGGCTTTCGAAGACATCAAAGCCGCCCTGCTGGCGGCTCCAGCTCTGGCCCTCCCTGACCTCACCAAGCCCTTCACACTCTATGTGGACGAGAGAGCTGGAGTGGCCAGAGGTGTGCTGACACAGGCACTGGGGCCGTGGAAGAGACCCGTGGCGTACCTATCTAAAAAGCTCGACCCCGTGGCCTCTGGGTGGCCGAGCCGCCTAAAGGCCATCGCTGCTGTAGCTCTACTGGTCAAAGATGCTGACAAGCTGACCCTGGGCCAGCATGTCACCGTCATCGCCCCGCACGCCTTAGAATCCATAGTCCGGCAACCACCAGATCGGTGGATGACAAACGCTAGGATGACGCATTATCAGAGCTTGCTGTTAAACGAAAGAGTGACATTTGCGCCCCCTGCCATCCTTAACCCAGCGACGCTACTGCCGGAGATCCACAACTCAACCCCCATCCACCAGTGCGTCGACATTCTGGCTGAGGAGACCGGCACGCGCAAGGACCTCACGGACAGACCATGGCCTGGTGTGCCGGCATGGTACACCGACGGCAGCTCCTTTGTCGTGGAGGGAAAGCGACGCGCAGGCGCGGCTGTGGTGGACGGCAAGCAAGTCATCTGGGCATCCTCCCTGCCAGAAGGCACCAGCGCTCAGAAAGCGGAGTTGGTGGCCTTGACCCAGGCCCTCAGATTAGCTGAAGGGAAGGCTATTAATATCTACACCGATTCTAGATACGCCTTCGCCACGGCGCACATCCACGGCGCGATCTACAAGCAGCGGGGCCTGCTGACCAGTGCCGGGCGAGACATCAAGAATAAGGAAGAGATTCTTGCCCTGCTGGAGGCCGTCCACCTCCCCAAAAAGGTGGCCATTATCCACTGCCCCGGCCACCAGAAGGGAGAGGATCCAATTACAAAGGGGAACCAGATGGCCGACCTCGTGGCGAAGCAGGTCGCTCAGCAGGTTACCATCCTGGCAGAAAAATCGCAGACCCCTGTGAAGACACCTGTGACAGATGATTCTAACTATAGCGGCGGCAGCAAAAGAACGGCGGACGGCTCTGAGAAGCGCACCGCTGATAGCCAGCATTCAACTCCTCCGAAAACAAAGAGGAAAGTTGAGTTCGAACCGAAGAAGAAAAGGAAGGTGTGAGAGCTCAGAGCTTTCGTTCGTATCATCGGTTTCGACAACGTTCGTCAAGTTCAATGCATCAGTTTCATTGCGCACACACCAGAATCCTACTGAGTTTGAGTATTATGGCATTGGGAAAACTGTTTTTCTTGTACCATTTGTTGTGCTTGTAATTTACTGTGTTTTTTATTCGGTTTTCGCTATCGAACTGTGAAATGGAAATGGATGGAGAAGAGTTAATGAATGATATGGTCCTTTTGTTCATTCTCAAATTAATATTATTTGTTTTTTCTCTTATTTGTTGTGTGTTGAATTTGAAATTATAAGAGATATGCAAACATTTTGTTTTGAGTAAAAATGTGTCAAATCGTGGCCTCTAATGACCGAAGTTAATATGAGGAGTAAAACACTTGTAGTTGTACCATTATGCTTATTCACTAGGCAACAAATATATTTTCAGACCTAGAAAAGCTGCAAATGTTACTGAATACAAGTATGTCCTCTTGTGTTTTAGACATTTATGAACTTTCCTTTATGTAATTTTCCAGAATCCTTGTCAGATTCTAATCATTGCTTTATAATTATAGTTATACTCATGGATTTGTAGTTGAGTATGAAAATATTTTTTAATGCATTTTATGACTTGCCAATTGATTGACAACGAATTCGTAATCATGTCATAGCTGTTTCCTGTGTGAAATTGTTATCCGCTCACAATTCCACACAACATACGAGCCGGAAGCATAAAGTGTAAAGCCTGGGGTGCCTAATGAGTGAGCTAACTCACATTAATTGCGTTGCGCTCACTGCCCGCTTTCCAGTCGGGAAACCTGTCGTGCCAGCTGCATTAATGAATCGGCCAACGCGCGGGGAGAGGCGGTTTGCGTATTGGCTAGAGCAGCTTGCCAACATGGTGGAGCACGACACTCTCGTCTACTCCAAGAATATCAAAGATACAGTCTCAGAAGACCAAAGGGCTATTGAGACTTTTCAACAAAGGGTAATATCGGGAAACCTCCTCGGATTCCATTGCCCAGCTATCTGTCACTTCATCAAAAGGACAGTAGAAAAGGAAGGTGGCACCTACAAATGCCATCATTGCGATAAAGGAAAGGCTATCGTTCAAGATGCCTCTGCCGACAGTGGTCCCAAAGATGGACCCCCACCCACGAGGAGCATCGTGGAAAAAGAAGACGTTCCAACCACGTCTTCAAAGCAAGTGGATTGATGTGAACATGGTGGAGCACGACACTCTCGTCTACTCCAAGAATATCAAAGATACAGTCTCAGAAGACCAAAGGGCTATTGAGACTTTTCAACAAAGGGTAATATCGGGAAACCTCCTCGGATTCCATTGCCCAGCTATCTGTCACTTCATCAAAAGGACAGTAGAAAAGGAAGGTGGCACCTACAAATGCCATCATTGCGATAAAGGAAAGGCTATCGTTCAAGATGCCTCTGCCGACAGTGGTCCCAAAGATGGACCCCCACCCACGAGGAGCATCGTGGAAAAAGAAGACGTTCCAACCACGTCTTCAAAGCAAGTGGATTGATGTGATATCTCCACTGACGTAAGGGATGACGCACAATCCCACTATCCTTCGCAAGACCCTTCCTCTATATAAGGAAGTTCATTTCATTTGGAGAGGACACGCTGAAATCACCAGTCTCTCTCTACAAATCTATCTCTCTCGAGCTTTCGCAGATCCGGGGGGCAATGAGATATGAAAAAGCCTGAACTCACCGCGACGTCTGTCGAGAAGTTTCTGATCGAAAAGTTCGACAGCGTCTCCGACCTGATGCAGCTCTCGGAGGGCGAAGAATCTCGTGCTTTCAGCTTCGATGTAGGAGGGCGTGGATATGTCCTGCGGGTAAATAGCTGCGCCGATGGTTTCTACAAAGATCGTTATGTTTATCGGCACTTTGCATCGGCCGCGCTCCCGATTCCGGAAGTGCTTGACATTGGGGAGTTTAGCGAGAGCCTGACCTATTGCATCTCCCGCCGTTCACAGGGTGTCACGTTGCAAGACCTGCCTGAAACCGAACTGCCCGCTGTTCTACAACCGGTCGCGGAGGCTATGGATGCGATCGCTGCGGCCGATCTTAGCCAGACGAGCGGGTTCGGCCCATTCGGACCGCAAGGAATCGGTCAATACACTACATGGCGTGATTTCATATGCGCGATTGCTGATCCCCATGTGTATCACTGGCAAACTGTGATGGACGACACCGTCAGTGCGTCCGTCGCGCAGGCTCTCGATGAGCTGATGCTTTGGGCCGAGGACTGCCCCGAAGTCCGGCACCTCGTGCACGCGGATTTCGGCTCCAACAATGTCCTGACGGACAATGGCCGCATAACAGCGGTCATTGACTGGAGCGAGGCGATGTTCGGGGATTCCCAATACGAGGTCGCCAACATCTTCTTCTGGAGGCCGTGGTTGGCTTGTATGGAGCAGCAGACGCGCTACTTCGAGCGGAGGCATCCGGAGCTTGCAGGATCGCCACGACTCCGGGCGTATATGCTCCGCATTGGTCTTGACCAACTCTATCAGAGCTTGGTTGACGGCAATTTCGATGATGCAGCTTGGGCGCAGGGTCGATGCGACGCAATCGTCCGATCCGGAGCCGGGACTGTCGGGCGTACACAAATCGCCCGCAGAAGCGCGGCCGTCTGGACCGATGGCTGTGTAGAAGTACTCGCCGATAGTGGAAACCGACGCCCCAGCACTCGTCCGAGGGCAAAGAAATAGAGTAGATGCCGACCGGGATCTGTCGATCGACAAGCTCGAGTTTCTCCATAATAATGTGTGAGTAGTTCCCAGATAAGGGAATTAGGGTTCCTATAGGGTTTCGCTCATGTGTTGAGCATATAAGAAACCCTTAGTATGTATTTGTATTTGTAAAATACTTCTATCAATAAAATTTCTAATTCCTAAAACCAAAATCCAGTACTAAAATCCAGATCCCCCGAATTAATTCGGCGTTAATTCAGTACATTAAAAACGTCCGCAATGTGTTATTAAGTTGTCTAAGCGTCAATTTGTTTACACCACAATATATCCTGCCA

**Sequence 84 Plasmids sequence of pH-35C-enRERV-OsEPSPS**

(RB T-DNA repeat-35C promoter-tRNAGly RS-pegRNA-Ubi promoter-NLSc-myc-NLSsv40-nCas9(H840A/ R221K/N394K)-XTEN-NC-NLS-32aa Linker-enRERV-RT-NLSvbp-HygR cassette-E9-terminator-CaMV poly(A) signal-LB T-DNA repeat)

GTTTACCCGCCAATATATCCTGTCAAACACTGATAGTTTAAACTGAAGGCGGGAAACGACAATCTGATCCAAGCTCAAGCTGCTCTAGCATTCGCCATTCAGGCTGCGCAACTGTTGGGAAGGGCGATCGGTGCGGGCCTCTTCGCTATTACGCCAGCTGGCGAAAGGGGGATGTGCTGCAAGGCGATTAAGTTGGGTAACGCCAGGGTTTTCCCAGTCACGACGTTGTAAAACGACGGCCAGTGAATTCATGGAGTCAAAGATTCAAATAGAGGACCTAACAGAACTCGCCGTAAAGACTGGCGAACAGTTCATACAGAGTCTCTTACGACTCAATGACAAGAAGAAAATCTTCGTCAACATGGTGGAGCACGACACACTTGTCTACTCCAAAAATATCAAAGATACAGTCTCAGAAGACCAAAGGGCAATTGAGACTTTTCAACAAAGGGTAATATCCGGAAACCTCCTCGGATTCCATTGCCCAGCTATCTGTCACTTTATTGTGAAGATAGTGGAAAAGGAAGGTGGCTCCTACAAATGCCATCATTGCGATAAAGGAAAGGCCATCGTTGAAGATGCCTCTGCCGACAGTGGTCCCAAAGATGGACCCCCACCCACGAGGAGCATCGTGGAAAAAGAAGACGTTCCAACCACGTCTTCAAAGCAAGTGGATTGATGTGATTGGCAGACATACTGTCCCACAAATGAAGATGGAATCTGTAAAAGAAAACGCGTGAAATAATGCGTCTGACAAAGGTTAGGTCGGCTGCCTTTAATCAATACCAAAGTGGTCCCTACCACGATGGAAAAACTGTGCAGTCGGTTTGGCTTTTTCTGACGAACAAATAAGATTCGTGGCCGACAGGTGGGGGTCCACCATGTGAAGGCATCTTCAGACTCCAATAATGGAGCAATGACGTAAGGGCTTACGAAATAAGTAAGGGTAGTTTGGGAAATGTCCACTCACCCGTCAGTCTATAAATACTTAGCCCCTCCCTCATTGTTAAGGGAGCAAAATCTCAGAGAGATAGTCCTAGAGAGAGAAAGAGAGCAAGTAGCCTAGAAGTAGTCAAGGCGGCGAAGTATTCAGGCACGTGGCCAGGAAGAAGAAAAGCCAAGACGACGAAAACAGGTAAGAGCTAAGCATCTAGATAAGTTGAAAACAATCTTCAAAAGTCCCACATCGCTTAGATAAGAAAACGAAGCTGAGTTTATATACAGCTAGAGTCGAAGTAGTGATTGAACAAAGCACCAGTGGTCTAGTGGTAGAATAGTACCCTGCCACGGTACAGACCCGGGTTCGATTCCCGGCTGGTGCAGCAGTCACGGCTGCTGTCAAGTTTAAGAGCTATGCTGGAAACAGCATAGCAAGTTTAAATAAGGCTAGTCCGTTATCAACTTGAAAAAGTGGCACCGAGTCGGTGCACGCTGGAATTGCAATGCGATCGTTGACAGCAGCCCGCGGTTCTATCTAGTTACGCGTTAAACCAACTAGAATTTTTTTGGCCGGCATGGTCCCAGCCTCCTCGCTGGCGCCGGCTGGGCAACATGCTTCGGCATGGCGAATGGGACTTTTTTTTGATATCTCCGGGGCTAATTGAATATGAAGATGAAGATGAAATATTTGGTGTGTCAAATAAAAAGCTGGTGTGCTTAAGTTTGTGTTTTTTTCTTGGCTTGTTGTGTTATGAATTTGTGGCTTTTTCTAATATTAAATGAATGTAAGATCTCATTATAATGAATAAACAAATGTTTCTATAATCCATTGTGAATGTTTTGTTGGATCTCTTCTGCAGCATATAACTACTGTATGTGCTATGGTATGGACTATGGAATATGATTAAAGATAAGAAGCTTGGCGTAATCTGCATGCCTGCAGTGCAGCGTGACCCGGTCGTGCCCCTCTCTAGAGATAATGAGCATTGCATGTCTAAGTTATAAAAAATTACCACATATTTTTTTTGTCACACTTGTTTGAAGTGCAGTTTATCTATCTTTATACATATATTTAAACTTTACTCTACGAATAATATAATCTATAGTACTACAATAATATCAGTGTTTTAGAGAATCATATAAATGAACAGTTAGACATGGTCTAAAGGACAATTGAGTATTTTGACAACAGGACTCTACAGTTTTATCTTTTTAGTGTGCATGTGTTCTCCTTTTTTTTTGCAAATAGCTTCACCTATATAATACTTCATCCATTTTATTAGTACATCCATTTAGGGTTTAGGGTTAATGGTTTTTATAGACTAATTTTTTTAGTACATCTATTTTATTCTATTTTAGCCTCTAAATTAAGAAAACTAAAACTCTATTTTAGTTTTTTTATTTAATAATTTAGATATAAAATAGAATAAAATAAAGTGACTAAAAATTAAACAAATACCCTTTAAGAAATTAAAAAAACTAAGGAAACATTTTTCTTGTTTCGAGTAGATAATGCCAGCCTGTTAAACGCCGTCGACGAGTCTAACGGACACCAACCAGCGAACCAGCAGCGTCGCGTCGGGCCAAGCGAAGCAGACGGCACGGCATCTCTGTCGCTGCCTCTGGACCCCTCTCGAGAGTTCCGCTCCACCGTTGGACTTGCTCCGCTGTCGGCATCCAGAAATTGCGTGGCGGAGCGGCAGACGTGAGCCGGCACGGCAGGCGGCCTCCTCCTCCTCTCACGGCACCGGCAGCTACGGGGGATTCCTTTCCCACCGCTCCTTCGCTTTCCCTTCCTCGCCCGCCGTAATAAATAGACACCCCCTCCACACCCTCTTTCCCCAACCTCGTGTTGTTCGGAGCGCACACACACACAACCAGATCTCCCCCAAATCCACCCGTCGGCACCTCCGCTTCAAGGTACGCCGCTCGTCCTCCCCCCCCCCCCCTCTCTACCTTCTCTAGATCGGCGTTCCGGTCCATGGTTAGGGCCCGGTAGTTCTACTTCTGTTCATGTTTGTGTTAGATCCGTGTTTGTGTTAGATCCGTGCTGCTAGCGTTCGTACACGGATGCGACCTGTACGTCAGACACGTTCTGATTGCTAACTTGCCAGTGTTTCTCTTTGGGGAATCCTGGGATGGCTCTAGCCGTTCCGCAGACGGGATCGATTTCATGATTTTTTTTGTTTCGTTGCATAGGGTTTGGTTTGCCCTTTTCCTTTATTTCAATATATGCCGTGCACTTGTTTGTCGGGTCATCTTTTCATGCTTTTTTTTGTCTTGGTTGTGATGATGTGGTCTGGTTGGGCGGTCGTTCTAGATCGGAGTAGAATTCTGTTTCAAACTACCTGGTGGATTTATTAATTTTGGATCTGTATGTGTGTGCCATACATATTCATAGTTACGAATTGAAGATGATGGATGGAAATATCGATCTAGGATAGGTATACATGTTGATGCGGGTTTTACTGATGCATATACAGAGATGCTTTTTGTTCGCTTGGTTGTGATGATGTGGTGTGGTTGGGCGGTCGTTCATTCGTTCTAGATCGGAGTAGAATACTGTTTCAAACTACCTGGTGTATTTATTAATTTTGGAACTGTATGTGTGTGTCATACATCTTCATAGTTACGAGTTTAAGATGGATGGAAATATCGATCTAGGATAGGTATACATGTTGATGTGGGTTTTACTGATGCATATACATGATGGCATATGCAGCATCTATTCATATGCTCTAACCTTGAGTACCTATCTATTATAATAAACAAGTATGTTTTATAATTATTTTGATCTTGATATACTTGGATGATGGCATATGCAGCAGCTATATGTGGATTTTTTTAGCCCTGCCTTCATACGCTATTTATTTGCTTGGTACTGTTTCTTTTGTCGATGCTCACCCTGTTGTTTGGTGTTACTTCTGCAGCCCTAGGACTGGTTGTACAATGCCGGCGGCCAAGAGAGTCAAGCTCGACGGCGGGAAGCGCACAGCTGATGGTTCTGAATTCGAGTCCCCTAAGAAAAAGAGAAAAGTGGACAAGAAGTACTCGATCGGCCTCGATATTGGGACTAACTCTGTTGGCTGGGCCGTGATCACCGACGAGTACAAGGTGCCCTCAAAGAAGTTCAAGGTCCTGGGCAACACCGATCGGCATTCCATCAAGAAGAATCTCATTGGCGCTCTCCTGTTCGACAGCGGCGAGACGGCTGAGGCTACGCGGCTCAAGCGCACCGCCCGCAGGCGGTACACGCGCAGGAAGAATCGCATCTGCTACCTGCAGGAGATTTTCTCCAACGAGATGGCGAAGGTTGACGATTCTTTCTTCCACAGGCTGGAGGAGTCATTCCTCGTGGAGGAGGATAAGAAGCACGAGCGGCATCCAATCTTCGGCAACATTGTCGACGAGGTTGCCTACCACGAGAAGTACCCTACGATCTACCATCTGCGGAAGAAGCTCGTGGACTCCACAGATAAGGCGGACCTCCGCCTGATCTACCTCGCTCTGGCCCACATGATTAAGTTCAGGGGCCATTTCCTGATCGAGGGGGATCTCAACCCGGACAATAGCGATGTTGACAAGCTGTTCATCCAGCTCGTGCAGACGTACAACCAGCTCTTCGAGGAGAACCCCATTAATGCGTCAGGCGTCGACGCGAAGGCTATCCTGTCCGCTAGGCTCTCGAAGTCTCGGAAGCTCGAGAACCTGATCGCCCAGCTGCCGGGCGAGAAGAAGAACGGCCTGTTCGGGAATCTCATTGCGCTCAGCCTGGGGCTCACGCCCAACTTCAAGTCGAATTTCGATCTCGCTGAGGACGCCAAGCTGCAGCTCTCCAAGGACACATACGACGATGACCTGGATAACCTCCTGGCCCAGATCGGCGATCAGTACGCGGACCTGTTCCTCGCTGCCAAGAATCTGTCGGACGCCATCCTCCTGTCTGATATTCTCAGGGTGAACACCGAGATTACGAAGGCTCCGCTCTCAGCCTCCATGATCAAGCGCTACGACGAGCACCATCAGGATCTGACCCTCCTGAAGGCGCTGGTCAGGCAGCAGCTCCCCGAGAAGTACAAGGAGATCTTCTTCGATCAGTCGAAGAACGGCTACGCTGGGTACATTGACGGCGGGGCCTCTCAGGAGGAGTTCTACAAGTTCATCAAGCCGATTCTGGAGAAGATGGACGGCACGGAGGAGCTGCTGGTGAAGCTCAAGCGCGAGGACCTCCTGAGGAAGCAGCGGACATTCGATAACGGCAGCATCCCACACCAGATTCATCTCGGGGAGCTGCACGCTATCCTGAGGAGGCAGGAGGACTTCTACCCTTTCCTCAAGGATAACCGCGAGAAGATCGAGAAGATTCTGACTTTCAGGATCCCGTACTACGTCGGCCCACTCGCTAGGGGCAACTCCCGCTTCGCTTGGATGACCCGCAAGTCAGAGGAGACGATCACGCCGTGGAACTTCGAGGAGGTGGTCGACAAGGGCGCTAGCGCTCAGTCGTTCATCGAGAGGATGACGAATTTCGACAAGAACCTGCCAAATGAGAAGGTGCTCCCTAAGCACTCGCTCCTGTACGAGTACTTCACAGTCTACAACGAGCTGACTAAGGTGAAGTATGTGACCGAGGGCATGAGGAAGCCGGCTTTCCTGTCTGGGGAGCAGAAGAAGGCCATCGTGGACCTCCTGTTCAAGACCAACCGGAAGGTCACGGTTAAGCAGCTCAAGGAGGACTACTTCAAGAAGATTGAGTGCTTCGATTCGGTCGAGATCTCTGGCGTTGAGGACCGCTTCAACGCCTCCCTGGGGACCTACCACGATCTCCTGAAGATCATTAAGGATAAGGACTTCCTGGACAACGAGGAGAATGAGGATATCCTCGAGGACATTGTGCTGACACTCACTCTGTTCGAGGACCGGGAGATGATCGAGGAGCGCCTGAAGACTTACGCCCATCTCTTCGATGACAAGGTCATGAAGCAGCTCAAGAGGAGGAGGTACACCGGCTGGGGGAGGCTGAGCAGGAAGCTCATCAACGGCATTCGGGACAAGCAGTCCGGGAAGACGATCCTCGACTTCCTGAAGAGCGATGGCTTCGCGAACCGCAATTTCATGCAGCTGATTCACGATGACAGCCTCACATTCAAGGAGGATATCCAGAAGGCTCAGGTGAGCGGCCAGGGGGACTCGCTGCACGAGCATATCGCGAACCTCGCTGGCTCGCCAGCTATCAAGAAGGGGATTCTGCAGACCGTGAAGGTTGTGGACGAGCTGGTGAAGGTCATGGGCAGGCACAAGCCTGAGAACATCGTCATTGAGATGGCCCGGGAGAATCAGACCACGCAGAAGGGCCAGAAGAACTCACGCGAGAGGATGAAGAGGATCGAGGAGGGCATTAAGGAGCTGGGGTCCCAGATCCTCAAGGAGCACCCGGTGGAGAACACGCAGCTGCAGAATGAGAAGCTCTACCTGTACTACCTCCAGAATGGCCGCGATATGTATGTGGACCAGGAGCTGGATATTAACAGGCTCAGCGATTACGACGTCGATGCCATCGTTCCACAGTCATTCCTGAAGGATGACTCCATTGACAACAAGGTCCTCACCAGGTCGGACAAGAACCGGGGCAAGTCTGATAATGTTCCTTCAGAGGAGGTCGTTAAGAAGATGAAGAACTACTGGCGCCAGCTCCTGAATGCCAAGCTGATCACGCAGCGGAAGTTCGATAACCTCACAAAGGCTGAGAGGGGCGGGCTCTCTGAGCTGGACAAGGCGGGCTTCATCAAGAGGCAGCTGGTCGAGACACGGCAGATCACTAAGCACGTTGCGCAGATTCTCGACTCACGGATGAACACTAAGTACGATGAGAATGACAAGCTGATCCGCGAGGTGAAGGTCATCACCCTGAAGTCAAAGCTCGTCTCCGACTTCAGGAAGGATTTCCAGTTCTACAAGGTTCGGGAGATCAACAATTACCACCATGCCCATGACGCGTACCTGAACGCGGTGGTCGGCACAGCTCTGATCAAGAAGTACCCAAAGCTCGAGAGCGAGTTCGTGTACGGGGACTACAAGGTTTACGATGTGAGGAAGATGATCGCCAAGTCGGAGCAGGAGATTGGCAAGGCTACCGCCAAGTACTTCTTCTACTCTAACATTATGAATTTCTTCAAGACAGAGATCACTCTGGCCAATGGCGAGATCCGGAAGCGCCCCCTCATCGAGACGAACGGCGAGACGGGGGAGATCGTGTGGGACAAGGGCAGGGATTTCGCGACCGTCAGGAAGGTTCTCTCCATGCCACAAGTGAATATCGTCAAGAAGACAGAGGTCCAGACTGGCGGGTTCTCTAAGGAGTCAATTCTGCCTAAGCGGAACAGCGACAAGCTCATCGCCCGCAAGAAGGACTGGGATCCGAAGAAGTACGGCGGGTTCGACAGCCCCACTGTGGCCTACTCGGTCCTGGTTGTGGCGAAGGTTGAGAAGGGCAAGTCCAAGAAGCTCAAGAGCGTGAAGGAGCTGCTGGGGATCACGATTATGGAGCGCTCCAGCTTCGAGAAGAACCCGATCGATTTCCTGGAGGCGAAGGGCTACAAGGAGGTGAAGAAGGACCTGATCATTAAGCTCCCCAAGTACTCACTCTTCGAGCTGGAGAACGGCAGGAAGCGGATGCTGGCTTCCGCTGGCGAGCTGCAGAAGGGGAACGAGCTGGCTCTGCCGTCCAAGTATGTGAACTTCCTCTACCTGGCCTCCCACTACGAGAAGCTCAAGGGCAGCCCCGAGGACAACGAGCAGAAGCAGCTGTTCGTCGAGCAGCACAAGCATTACCTCGACGAGATCATTGAGCAGATTTCCGAGTTCTCCAAGCGCGTGATCCTGGCCGACGCGAATCTGGATAAGGTCCTCTCCGCGTACAACAAGCACCGCGACAAGCCAATCAGGGAGCAGGCTGAGAATATCATTCATCTCTTCACCCTGACGAACCTCGGCGCCCCTGCTGCTTTCAAGTACTTCGACACAACTATCGATCGCAAGAGGTACACAAGCACTAAGGAGGTCCTGGACGCGACCCTCATCCACCAGTCGATTACCGGCCTCTACGAGACGCGCATCGACCTGTCTCAGCTCGGGGGCGACGAATTCTCCGGGAGCGAGACGCCAGGCACCTCCGAGTCGGCCACCCCAGAATCTGCCACAGTGGTGTCCGGCCAAAAGCAGGACCGCCAGGGCGGAGAACGCAGAAGGTCCCAGCTCGATAGGGATCAGTGTGCCTACTGCAAGGAGAAGGGCCACTGGGCCAAAGACTGCCCGAAAAAGCCGCGCGGCCCACGCGGCCCAAGGCCACAAACATCCCTCCTTCCAAAGAAGAAGCGGAAGGTGGAGCTCAGCGGAGGATCTTCCGGAGGATCTAGCGGCTCCGAGACACCAGGAACATCCGAAAGCGCTACACCAGAATCTAGCGGAGGCTCTTCCGGAGGATCTAGGCCTCCTCCCGTCGCGTGCCTCGTACTGAGCCTAGAGGAGGAGTACAGGCTGCATGAGCAAAATCCGAAGCAGCTGCTAGCCCCTGAATGGCTGAGCGCCTTCTCCGAGGTATGGGCGGAGCAGACTGGCATGGGACTGGCCAAGCAGGTGCCTCCTGTGGTGGTAGAGCTGAAGGCGGACGCCAGCCCAGTGAGCGTGAAACAACGCCCGATGTCGAGGGAAGCAAAGGAAGGCATTAGACCCCACATCCAGAGACTGCTGCAATTGGGAATCCTGGTACCCTGCCAAAGCCCTTGGAACACGCCACTGCTGCCTGTGCGCAAACCAGGAACTAATGATTACAGACCTGTACAGGATCTAAGGGAAGTTAACAAGCGGGTGCAGGACATACATCCTACGGTCCCAAACCCCTACAACTTGCTGAGCTCTCTGCCCCCCGAGCGCACAttcTACACTGTGCTCGACTTAAAAGATGCGTTCTTCTGCCTTCGCCTCCACCCGAACTCACAGCCACTGTTCGCGTTCGAGTGGCGCGACCCTGAGGGCGGCCACACCGGGCAACTGACGTGGACACGGCTGCCACAGGGTTTCAAGAACAGCCCTACTCTATTTAACGAGGCCCTGCACCGTGATCTGGCCCCCTTCCGGGCGCAGAACCCTCAAATCAGTCTACTGCAGTATGCGGATGACCTGCTGCTAGCCGCTTCTACTCAGGAGCTCTGCCGGGAGGGCACAAAGCGGCTGTTAAATGAGCTGGGCGAGCTGGGATACCGCGTGTCTGCCAAGAAGGCACAATTGTGTCGGACAGAGGTGACATATCTCGGCTACACATTAAGAGAAGGTAAGAGATGGCTCACTGAAGCACGTAAGAAGACTGTCATGCAGATTCCTACCCCTACCACGCCCCGTCAGGTGCGGGAGTTCCTCGGCAAGGCCGGCTTCTGTAGACTGTTtATTCCTGGATTTGCCACCCTGGCCGCCCCACTCTACCTGCTGACGAAGCCAAAGGTTCCTTTCACCTGGACTGAGGAACATCAGCGGGCTTTCGAAGACATCAAAGCCGCCCTGCTGGCGGCTCCAGCTCTGGCCCTCCCTGACCTCACCAAGCCCTTCACACTCTATGTGGACGAGAGAGCTGGAGTGGCCAGAGGTGTGCTGACACAGGCACTGGGGCCGTGGAAGAGACCCGTGGCGTACCTATCTAAAAAGCTCGACCCCGTGGCCTCTGGGTGGCCGAGCCGCCTAAAGGCCATCGCTGCTGTAGCTCTACTGGTCAAAGATGCTGACAAGCTGACCCTGGGCCAGCATGTCACCGTCATCGCCCCGCACGCCTTAGAATCCATAGTCCGGCAACCACCAGATCGGTGGATGACAAACGCTAGGATGACGCATTATCAGAGCTTGCTGTTAAACGAAAGAGTGACATTTGCGCCCCCTGCCATCCTTAACCCAGCGACGCTACTGCCGGAGATCCACAACTCAACCCCCATCCACCAGTGCGTCGACATTCTGGCTGAGGAGACCGGCACGCGCAAGGACCTCACGGACAGACCATGGCCTGGTGTGCCGGCATGGTACACCGACGGCAGCTCCTTTGTCGTGGAGGGAAAGCGACGCGCAGGCGCGGCTGTGGTGGACGGCAAGCAAGTCATCTGGGCATCCTCCCTGCCAGAAGGCACCAGCGCTCAGAAAGCGGAGTTGGTGGCCTTGACCCAGGCCCTCAGATTAGCTGAAGGGAAGGCTATTAATATCTACACCGATTCTAGATACGCCTTCGCCACGGCGCACATCCACGGCGCGATCTACAAGCAGCGGGGCCTGCTGACCAGTGCCGGGCGAGACATCAAGAATAAGGAAGAGATTCTTGCCCTGCTGGAGGCCGTCCACCTCCCCAAAAAGGTGGCCATTATCCACTGCCCCGGCCACCAGAAGGGAGAGGATCCAATTACAAAGGGGAACCAGATGGCCGACCTCGTGGCGAAGCAGGTCGCTCAGCAGGTTACCATCCTGGCAGAAAAATCGCAGACCCCTGTGAAGACACCTGTGACAGATGATTCTAACTATAGCGGCGGCAGCAAAAGAACGGCGGACGGCTCTGAGAAGCGCACCGCTGATAGCCAGCATTCAACTCCTCCGAAAACAAAGAGGAAAGTTGAGTTCGAACCGAAGAAGAAAAGGAAGGTGTGAGAGCTCAGAGCTTTCGTTCGTATCATCGGTTTCGACAACGTTCGTCAAGTTCAATGCATCAGTTTCATTGCGCACACACCAGAATCCTACTGAGTTTGAGTATTATGGCATTGGGAAAACTGTTTTTCTTGTACCATTTGTTGTGCTTGTAATTTACTGTGTTTTTTATTCGGTTTTCGCTATCGAACTGTGAAATGGAAATGGATGGAGAAGAGTTAATGAATGATATGGTCCTTTTGTTCATTCTCAAATTAATATTATTTGTTTTTTCTCTTATTTGTTGTGTGTTGAATTTGAAATTATAAGAGATATGCAAACATTTTGTTTTGAGTAAAAATGTGTCAAATCGTGGCCTCTAATGACCGAAGTTAATATGAGGAGTAAAACACTTGTAGTTGTACCATTATGCTTATTCACTAGGCAACAAATATATTTTCAGACCTAGAAAAGCTGCAAATGTTACTGAATACAAGTATGTCCTCTTGTGTTTTAGACATTTATGAACTTTCCTTTATGTAATTTTCCAGAATCCTTGTCAGATTCTAATCATTGCTTTATAATTATAGTTATACTCATGGATTTGTAGTTGAGTATGAAAATATTTTTTAATGCATTTTATGACTTGCCAATTGATTGACAACGAATTCGTAATCATGTCATAGCTGTTTCCTGTGTGAAATTGTTATCCGCTCACAATTCCACACAACATACGAGCCGGAAGCATAAAGTGTAAAGCCTGGGGTGCCTAATGAGTGAGCTAACTCACATTAATTGCGTTGCGCTCACTGCCCGCTTTCCAGTCGGGAAACCTGTCGTGCCAGCTGCATTAATGAATCGGCCAACGCGCGGGGAGAGGCGGTTTGCGTATTGGCTAGAGCAGCTTGCCAACATGGTGGAGCACGACACTCTCGTCTACTCCAAGAATATCAAAGATACAGTCTCAGAAGACCAAAGGGCTATTGAGACTTTTCAACAAAGGGTAATATCGGGAAACCTCCTCGGATTCCATTGCCCAGCTATCTGTCACTTCATCAAAAGGACAGTAGAAAAGGAAGGTGGCACCTACAAATGCCATCATTGCGATAAAGGAAAGGCTATCGTTCAAGATGCCTCTGCCGACAGTGGTCCCAAAGATGGACCCCCACCCACGAGGAGCATCGTGGAAAAAGAAGACGTTCCAACCACGTCTTCAAAGCAAGTGGATTGATGTGAACATGGTGGAGCACGACACTCTCGTCTACTCCAAGAATATCAAAGATACAGTCTCAGAAGACCAAAGGGCTATTGAGACTTTTCAACAAAGGGTAATATCGGGAAACCTCCTCGGATTCCATTGCCCAGCTATCTGTCACTTCATCAAAAGGACAGTAGAAAAGGAAGGTGGCACCTACAAATGCCATCATTGCGATAAAGGAAAGGCTATCGTTCAAGATGCCTCTGCCGACAGTGGTCCCAAAGATGGACCCCCACCCACGAGGAGCATCGTGGAAAAAGAAGACGTTCCAACCACGTCTTCAAAGCAAGTGGATTGATGTGATATCTCCACTGACGTAAGGGATGACGCACAATCCCACTATCCTTCGCAAGACCCTTCCTCTATATAAGGAAGTTCATTTCATTTGGAGAGGACACGCTGAAATCACCAGTCTCTCTCTACAAATCTATCTCTCTCGAGCTTTCGCAGATCCGGGGGGCAATGAGATATGAAAAAGCCTGAACTCACCGCGACGTCTGTCGAGAAGTTTCTGATCGAAAAGTTCGACAGCGTCTCCGACCTGATGCAGCTCTCGGAGGGCGAAGAATCTCGTGCTTTCAGCTTCGATGTAGGAGGGCGTGGATATGTCCTGCGGGTAAATAGCTGCGCCGATGGTTTCTACAAAGATCGTTATGTTTATCGGCACTTTGCATCGGCCGCGCTCCCGATTCCGGAAGTGCTTGACATTGGGGAGTTTAGCGAGAGCCTGACCTATTGCATCTCCCGCCGTTCACAGGGTGTCACGTTGCAAGACCTGCCTGAAACCGAACTGCCCGCTGTTCTACAACCGGTCGCGGAGGCTATGGATGCGATCGCTGCGGCCGATCTTAGCCAGACGAGCGGGTTCGGCCCATTCGGACCGCAAGGAATCGGTCAATACACTACATGGCGTGATTTCATATGCGCGATTGCTGATCCCCATGTGTATCACTGGCAAACTGTGATGGACGACACCGTCAGTGCGTCCGTCGCGCAGGCTCTCGATGAGCTGATGCTTTGGGCCGAGGACTGCCCCGAAGTCCGGCACCTCGTGCACGCGGATTTCGGCTCCAACAATGTCCTGACGGACAATGGCCGCATAACAGCGGTCATTGACTGGAGCGAGGCGATGTTCGGGGATTCCCAATACGAGGTCGCCAACATCTTCTTCTGGAGGCCGTGGTTGGCTTGTATGGAGCAGCAGACGCGCTACTTCGAGCGGAGGCATCCGGAGCTTGCAGGATCGCCACGACTCCGGGCGTATATGCTCCGCATTGGTCTTGACCAACTCTATCAGAGCTTGGTTGACGGCAATTTCGATGATGCAGCTTGGGCGCAGGGTCGATGCGACGCAATCGTCCGATCCGGAGCCGGGACTGTCGGGCGTACACAAATCGCCCGCAGAAGCGCGGCCGTCTGGACCGATGGCTGTGTAGAAGTACTCGCCGATAGTGGAAACCGACGCCCCAGCACTCGTCCGAGGGCAAAGAAATAGAGTAGATGCCGACCGGGATCTGTCGATCGACAAGCTCGAGTTTCTCCATAATAATGTGTGAGTAGTTCCCAGATAAGGGAATTAGGGTTCCTATAGGGTTTCGCTCATGTGTTGAGCATATAAGAAACCCTTAGTATGTATTTGTATTTGTAAAATACTTCTATCAATAAAATTTCTAATTCCTAAAACCAAAATCCAGTACTAAAATCCAGATCCCCCGAATTAATTCGGCGTTAATTCAGTACATTAAAAACGTCCGCAATGTGTTATTAAGTTGTCTAAGCGTCAATTTGTTTACACCACAATATATCCTGCCA
