# Supplementary material for: Discovery and Engineering of a Rat Endogenous Retrovirus Reverse Transcriptase for Efficient Prime Editing
Source: Adv Sci (Weinh). 2026 Jun 26:e75888. Online ahead of print. doi: 10.1002/advs.75888 (PMC13335913; doi:10.1002/advs.75888)
Supplement: Supplementary file 4 — Supporting File 4: advs75888‐sup‐0004‐TableS1.pdf. [file ADVS-9999-e75888-s003.pdf]

Table S1 Synthesized protein sequences in the phylogenetic tree

RT ID	Gene ID in phylogenetic tree	domain family	NCBI ID	Domain start and end loci in NCBI annotation	Gene function annotation in NCBI	Species	Full length seq in Phylogenetic trees	synthesis yes or no	Synthesis protein seq	Activity (/: no observed activity; *: slight activity; **: medium activity; ***: strong activity)	Synthesis DNA sequence (plant)
RT1	22	group_II_RT_mat	WP_222823141.1	2-421	group II intron reverse transcriptase/maturase	Caldalkalibacillus thermarum	PALLENILSRSNLTKALKRVEANKGAPGMDGVSTEHLRDYLREHWPAIKQKLLEGTYQPAPVRRVEIPKPDGGVRLLGIPTVIDRFIQQAILQVLTPIFDPHFSSHSYGFRPQRRAHDAVRQAQRYIHEGYKYVVDIDLEKFFDRVHHDILMSRVARRVKDKRVLKLIRAYLKAGIMIAGIKVRSEEGTPQGGTLSPLLANILLDDLDKELEKRGLRFCRYADDCNIYARSRRAGQRVKQSIQKFLEKKLKLKVNEEKSAVDRPWRRKFLGFSFTSQRQARIRLAPQSIQRFKNKIRQLTNPNWSISMEERIKKLNQYMMGWIGYFALIETPSPLKRLEEWIRRRLRLCRWHQWKRVRIRELRALGLKEHEVFEIANTRKGAWTKTPQLHKALSKAYWLAQGLRSLTERYFDVRQDWA	yes	MPALLENILSRSNLTKALKRVEANKGAPGMDGVSTEHLRDYLREHWPAIKQKLLEGTYQPAPVRRVEIPKPDGGVRLLGIPTVIDRFIQQAILQVLTPIFDPHFSSHSYGFRPQRRAHDAVRQAQRYIHEGYKYVVDIDLEKFFDRVHHDILMSRVARRVKDKRVLKLIRAYLKAGIMIAGIKVRSEEGTPQGGTLSPLLANILLDDLDKELEKRGLRFCRYADDCNIYARSRRAGQRVKQSIQKFLEKKLKLKVNEEKSAVDRPWRRKFLGFSFTSQRQARIRLAPQSIQRFKNKIRQLTNPNWSISMEERIKKLNQYMMGWIGYFALIETPSPLKRLEEWIRRRLRLCRWHQWKRVRTRIRELRALGLKEHEVFEIANTRKGAWRTTKTPQLHKALSKAYWLAQGLRSLTERYFDVRQDWRTA	/	ATGCCTGCCCTGCTGGAAAATATTTTGTCTAGATCTAACTTGACCAAAGCTCTAAAAAGAGTAGAGGCGAACAAGGGCGCCCCCGGCATGGATGGAGTGAGCACTGAGCACCTCAGAGACTACCTTAGGGAGCACTGGCCAGCCATCAAACAAAAGCTCCTGGAGGGAACCTACCAGCCAGCCCCTGTGCGCAGAGTGGAGATACCAAAACCTGACGGCGGCGTGAGACTACTGGGCATTCCTACCGTCATCGACAGGTTTATACAGCAGGCTATCCTTCAGGTGCTCACGCCTATCTTTGACCCACACTTCTCATCTCACTCCTACGGCTTCCGTCCGCAGAGAAGGGCACATGATGCCGTGAGACAGGCGCAGCGCTACATTCATGAAGGATACAAGTATGTTGTGGACATCGATCTGGAGAAATTCTTCGACCGCGTCCACCACGATATCCTTATGTCGAGGGTGGCACGGAGAGTCAAGGATAAGCGGGTACTCAAGCTGATTAGAGCTTACTTGAAGGCCGGCATTATGATCGCCGGCATTAAGGTTAGATCCGAAGAAGGAACCCCCCAAGGGGGCACCCTGTCCCCCCTGCTTGCAAACATCCTCTTAGATGATCTCGACAAGGAGCTGGAGAAGCGCGGCCTGCGGTTCTGTAGATACGCCGACGACTGCAACATCTATGCTAGGAGCCGTAGAGCGGGCCAGCGGGTGAAGCAGAGCATTCAGAAGTTCTTGGAAAAGAAATTGAAGCTGAAGGTGAATGAAGAGAAGAGCGCCGTCGACCGGCCATGGCGAAGGAAGTTTCTTGGTTTCTCCTTCACAAGCCAGAGGCAAGCTCGCATCAGACTCGCCCCTCAGTCGATTCAAAGATTCAAGAACAAAATTAGGCAGCTCACCAATCCTAACTGGAGCATCTCAATGGAGGAGCGCATAAAGAAGTTGAACCAGTACATGATGGGGTGGATAGGTTACTTCGCCTTAATCGAGACCCCGAGCCCGCTGAAGCGCCTGGAGGAGTGGATCCGGAGACGGCTGAGACTCTGCAGGTGGCACCAATGGAAGAGAGTGCGGACCCGTATCCGGGAGCTCCGCGCCCTGGGCCTCAAGGAACATGAGGTGTTTGAAATCGCAAACACAAGAAAGGGCGCGTGGAGGACAACAAAAACACCCCAGTTGCACAAGGCCCTGTCAAAGGCTTACTGGCTCGCCCAGGGGCTGCGTAGCCTGACCGAAAGATATTTTGATGTGAGACAAGATTGGCGCACTGCC
RT2	95	group_II_RT_mat	WP_038509608.1	2-319	retron St85 family RNA-directed DNA polymerase	Vibrio coralliilyticus	NLVSKLALYLEKSESEVIRFLSDAPNKYRVYKIPKRSHGHRIIAQPSKELKEYQRAFLELYEFPVHDSAMAYCKGKGIKENALAHSKNSYLLKTDLENFFNSITPPIFWQCMEKCASTVPQFTKQEKVLVEKLIFWCPSKDRNGKLVLSIGAPSSPAISNFCLYQFDDFMSDVCYDHKITYTRYADDLTFSTNEKDLLHTIINSIQYSLNYFFSNSLKLNHSKTVFSSRAHNRHVTGITINNHGRLSLGRERKRYIKHLVNQFKYNQLSESDIFHLQGLLSFARHIEPKFIFRLKDKYTNELVQRIYEAGNEQQNKKQ	yes	MNLVSKLALYLEKSESEVIRFLSDAPNKYRVYKIPKRSHGHRIIAQPSKELKEYQRAFLELYEFPVHDSAMAYCKGKGIKENALAHSKNSYLLKTDLENFFNSITPPIFWQCMEKCASTVPQFTKQEKVLVEKLIFWCPSKDRNGKLVLSIGAPSSPAISNFCLYQFDDFMSDVCYDHKITYTRYADDLTFSTNEKDLLHTIINSIQYSLNYFFSNSLKLNHSKTVFSSRAHNRHVTGITINNHGRLSLGRERKRYIKHLVNQFKYNQLSESDIFHLQGLLSFARHIEPKFIFRLKDKYTNELVQRIYEAGNEQQNKKQ	*	ATGAACCTGGTGTCAAAATTGGCACTGTATTTGGAAAAGAGTGAGTCTGAAGTCATCCGCTTCCTCTCTGATGCACCAAATAAATATCGAGTGTACAAGATTCCAAAGAGATCTCATGGCCATAGGATTATAGCTCAACCTTCTAAGGAACTGAAGGAGTACCAGCGGGCGTTCTTGGAGCTGTACGAGTTCCCCGTCCACGACAGCGCCATGGCGTACTGCAAGGGGAAGGGCATCAAAGAAAATGCTCTCGCCCACAGCAAGAACTCGTACCTTCTCAAGACTGACCTCGAGAATTTTTTCAACAGCATCACGCCGCCGATTTTCTGGCAGTGCATGGAGAAGTGCGCCTCCACAGTGCCGCAGTTCACCAAGCAGGAAAAGGTGCTTGTGGAGAAATTGATTTTTGGTGCCCTTCCAAGGATCGGAACGGCAAGCTTGTCCTCTCTATTGGAGCACCTTCGTCGCCCGCCATCAGCAACTTTTGTTTATATCAATTTGACGACTTTATGTCAGATGTTTGTTATGATCACAAGATTACTTATACTCGCTATGCTGATGATCTTACCTTTTCAACAAATGAAAAAGACCTGCTCCACACCATCATCAATTCAATTCAGTACTCCTTGAACTACTTCTTCTCCAATTCTTTAAAACTGAATCATAGTAAAACGGTGTTCAGCTCGCGTGCGCACAACCGCCATGTTACTGGGATAACAATCAACAACCATGGTAGGTTGTCCCTCGGCAGGGAGAGGAAGAGGTACATCAAGCACCTTGTAAATCAATTCAAGTACAACCAGCTATCCGAGTCCGACATCTTCCACCTGCAGGGTCTTCTCTCATTCGCCAGACATATTGAACCAAAATTTATATTTAGACTGAAAGATAAATACACCAACGAGCTGGTTCAACGGATATATGAGGCGGGAAATGAGCAGCAGAACAAGAAGCAA
RT3	96	group_II_RT_mat	WP_118601765.1	2-427	group II intron reverse transcriptase/maturase	Blautia sp. AF19-1	PDTSSLMKQILSSDNLNRAYLQVVRNKGAEGVDGMKYTELKEHLVKDGEIIKEQLILVPCQSPWNTPLLPIPKPDGGVRNLGVPTVTDRLIQQAIAQVLTPIYEEQFHDHSYGFRPNRCAQQAILTALDMMNDGNDWIVDIDLEKFFDTVNHDKLMTIIGIKDGDVISIVRKYLVSGIMIDDEYEDSIVGTPQGGNLSPLLANIMLNELDKEMEKRGLNFVRYADDCIIMVGSEMSANRVMRNISRFIEEKLGLKVNMTKSKVDRPSGLKYLGFGFYFDSRAHQFKAKPHAKSVAKFKKRMKGLTCRSWGVSNSYKVEKLNQLIRGWINYFKIGSMKILCAKLDANIRYRLRMCIWKHWKTPQNRAKNLMKLGMDRITAYKVGYCSRAYAHVCSCGAVNIAITNKRLASFGLISMLDYYTERCVTC	yes	MPDTSSLMKQILSSDNLNRAYLQVVRNKGAEGVDGMKYTELKEHLVKDGEIIKEQLRTRKYKPQPVRRVEIPKPDGGVRNLGVPTVTDRLIQQAIAQVLTPIYEEQFHDHSYGFRPNRCAQQAILTALDMMNDGNDWIVDIDLEKFFDTVNHDKLMTIIGRTIKDGDVISIVRKYLVSGIMIDDEYEDSIVGTPQGGNLSPLLANIMLNELDKEMEKRGLNFVRYADDCIIMVGSEMSANRVMRNISRFIEEKLGLKVNMTKSKVDRPSGLKYLGFGFYFDSRAHQFKAKPHAKSVAKFKKRMKGLTCRSWGVSNSYKVEKLNQLIRGWINYFKIGSMKILCAKLDANIRYRLRMCIWKHWKTPQNRAKNLMKLGMDRITAYKVGYCSRAYAHVCSCGAVNIAITNKRLASFGLISMLDYYTERCVTC	/	ATGCCTGACACTTCCTCCCTCATGAAGCAGATACTGTCCAGCGACAATCTGAACCGTGCCTACCTCCAGGTGGTGCGGAACAAGGGAGCAGAGGGCGTTGACGGAATGAAATACACCGAGCTCAAGGAGCACCTCGTGAAGGATGGAGAAATCATCAAGGAGCAACTGCGTACCCGGAAGTACAAACCACAGCCAGTGCGGAGAGTTGAAATCCCTAAGCCTGACGGCGGCGTGCGCAACCTGGGCGTGCCTACCGTGACAGATAGACTGATCCAGCAAGCTATCGCGCAGGTTTTGACCCCCATCTATGAGGAGCAGTTCCACGACCACAGCTATGGGTTCAGACCCAACCGGTGTGCACAGCAGGCAATCCTGACCGCCCTCGACATGATGAATGATGGGAACGACTGGATCGTTGATATTGATCTCGAGAAGTTCTTCGACACGGTGAATCATGATAAATTGATGACGATCATTGGCCGCACCATTAAAGATGGCGACGTGATCAGCATAGTGCGCAAGTACCTGGTGAGCGGCATCATGATTGATGATGAATATGAGGACAGCATCGTCGGCACTCCTCAGGGAGGCAACCTCTCTCCACTCCTCGCCAATATAATGTTGAATGAACTTGACAAGGAAATGGAGAAGCGCGGCCTGAACTTTGTGAGGTACGCCGATGACTGCATTATCATGGTGGGCTCAGAGATGTCGGCCAACAGAGTGATGAGAAACATTTCACGGTTCATCGAAGAAAAACTGGGTTTGAAAGTTAACATGACAAAGAGCAAAGTCGACCGCCCCTCTGGCCTTAAGTATCTGGGCTTCGGGTTCTACTTTGACAGCCGAGCTCACCAATTTAAAGCAAAGCCGCACGCCAAGTCTGTGGCGAAGTTTAAGAAGAGGATGAAAGGTCTGACCTGCCGGTCATGGGGTGTCAGCAATTCCTACAAGGTGGAGAAGCTCAACCAGCTCATCAGAGGATGGATTAACTACTTCAAAATCGGTTCCATGAAGATTCTCTGTGCGAAGCTGGATGCTAACATCCGGTATCGCCTGCGCATGTGCATTTGGAAGCATTGGAAAACACCCCAGAATCGGGCCAAGAATCTGATGAAGCTGGGCATGGATAGAATAACGGCCTACAAAGTTGGGTATTGCAGTAGAGCCTACGCGCACGTCTGCAGCTGCGGCGCCGTGAACATTGCCATCACCAACAAAAGACTGGCCTCCTTCGGGCTGATCAGTATGCTGGACTACTACACAGAAAGATGTGTGACTTGT
RT4	98	group_II_RT_mat	XP_049646384.1	1-469	uncharacterized protein LOC126033445, partial	Suncus etruscus	VLKKPLGPLSSKQSLVQGANGSSYRSWTTSRTMDLGKGEIKHSFLVIPNCPSPLMGQDLLTKLRAQITFEETGPEIAFLNPSVQPLTTSVLTIRAEDEYRLFTNDPLPVESQDTDFWLRLVPEAWAETAGLGLSTLQAPVVVELKTTAVPVRVRQYPMSQEAKKGITPHIRKLLQQGVLVKCQSAWNTPLLPVKKPGTGDYRPVQDLRAVNSQVEVIHPTVPNPYNLLSTLSPERVWYSVLDLKDAFFCLSLHPASQPLFAFEWSDPEAGISGQLTWTRLPQGFKNSPTIFDEALHQDLSLFRSQHPQVTLLQYVDDILLAGKTEEDWGKRWLTEARKKVVAQIPAPTTRRQLREFLGTAGFCRLWIPELAIVHCPGHQTGKETVASGNRRADEAAKAAASCMKEIPALIVTEPDNLPPVSTTVDPLSEEPEVGDVKLMVLRQQYQELAQADNFDQEYQEVEAPDHIDF	yes	MVLKKPLGPLSSKQSLVQGANGSSYRSWTTSRTMDLGKGEIKHSFLVIPNCPSPLMGQDLLTKLRAQITFEETGPEIAFLNPSVQPLTTSVLTIRAEDEYRLFTNDPLPVESQDTDFWLRLVPEAWAETAGLGLSTLQAPVVVELKTTAVPVRVRQYPMSQEAKKGITPHIRKLLQQGVLVKCQSAWNTPLLPVKKPGTGDYRPVQDLRAVNSQVEVIHPTVPNPYNLLSTLSPERVWYSVLDLKDAFFCLSLHPASQPLFAFEWSDPEAGISGQLTWTRLPQGFKNSPTIFDEALHQDLSLFRSQHPQVTLLQYVDDILLAGKTEEDWGKRWLTEARKKVVAQIPAPTTRRQLREFLGTAGFCRLWIPELAIVHCPGHQTGKETVASGNRRADEAAKAAASCMKEIPALIVTEPDNLPPVSTTVDPLSEEPEVGDVKLMVLRQQYQELAQADNFDQEYQEVEAPDHIDF	/	ATGGTCCTGAAGAAACCTCTGGGCCCGCTCTCCAGCAAGCAGAGCCTAGTTCAAGGTGCAAATGGTTCTAGCTATAGGTCATGGACGACGAGCAGGACTATGGATTTAGGGAAGGGGGAGATCAAGCATTCATTTCTTGTTATCCCAAATTGTCCTTCCCCTTTGATGGGACAAGATCTGCTGACCAAGTTGCGAGCACAGATCACCTTCGAGGAAACTGGACCTGAAATTGCCTTCCTCAACCCTTCTGTCCAGCCGCTCACCACTTCAGTCTTGACCATCAGGGCTGAGGATGAGTATCGTCTATTCACCAATGATCCACTGCCGGTTGAATCGCAAGACACCGACTTCTGGCTGCGGCTGGTCCCGGAGGCATGGGCAGAGACGGCCGGGCTTGGCCTATCCACACTTCAGGCCCCTGTTGTTGTCGAGCTCAAGACGACTGCTGTTCCTGTAAGAGTGAGGCAGTACCCGATGTCACAAGAAGCAAAAAAAGGCATTACACCGCACATCAGAAAATTGCTGCAGCAAGGCGTCCTCGTGAAATGCCAGTCTGCGTGGAACACACCGTTGTTGCCCGTCAAGAAGCCCGGAACTGGAGACTACCGGCCGGTGCAGGATCTCCGCGCCGTTAACTCACAAGTTGAGGTGATACACCCGACCGTGCCCAACCCATACAACCTCCTTTCCACGCTCTCTCCTGAGAGGGTGTGGTACAGCGTACTCGACCTCAAAGATGCTTTCTTCTGTTTGAGCCTGCATCCAGCAAGTCAACCACTCTTCGCCTTTGAGTGGTCCGACCCCGAAGCTGGCATAAGTGGGCAGCTGACATGGACACGACTACCGCAGGGCTTCAAGAATTCTCCAACAATTTTTGACGAGGCGCTGCACCAGGACCTGAGTTTGTTTCGTTCCCAGCACCCACAGGTGACGCTATTACAATATGTGGATGATATCCTTCTCGCCGGCAAGACTGAGGAGGACTGGGGTAAGCGCTGGCTGACGGAGGCGAGGAAGAAGGTGGTGGCCCAGATACCTGCCCCAACTACAAGAAGACAGCTTCGGGAGTTCTTGGGGACGGCGGGTTTCTGCCGCCTTTGGATCCCAGAATTAGCAATAGTGCACTGCCCAGGACATCAAACTGGTAAAGAAACAGTCGCCTCTGGCAACCGCCGGGCCGACGAAGCGGCGAAGGCCGCCGCATCGTGCATGAAGGAGATACCGGCGCTGATTGTCACCGAGCCCGACAACCTCCCGCCGGTCTCCACCACTGTTGATCCCCTTTCAGAAGAACCAGAAGTTGGCGACGTGAAACTGATGGTGCTCCGCCAGCAATATCAAGAGCTGGCTCAAGCTGATAATTTTGATCAGGAGTACCAGGAGGTGGAGGCTCCTGATCATATTGATTTT
RT5	99	group_II_RT_mat	WP_077616959.1	2-406	group II intron reverse transcriptase/maturase	Caenibacillus caldisaponilyticus	PALLETILSRNNLITALKRVEANKGAPGIDGVPTEQLRDDIRKHWKSIKRQLLEGTYKPAPVRRVEIPKPNGGVRLLGIPTVMDRFIQQAILQVLTPIFDPHFSPYSYGFRPKRRAHDAVRQAQKYIQEGYRYVVDIDLEKFFDRVNHDILMSRVARKVEDKRVLKLIRAYLKAGVMLEGVRVRSEEGTPQGGPLSPLLANILLDDLDKELEKRGLKFCRYADDCNIYVRSPRAGQRVKQSVQKYLEKKLKLKVNEEKSAVDRPWKRKFLGFSFTSQREARIRLAPKSVQRFKNKIRQLTNPNWSLPMEERIRKLNQYTMGWMGYFALIETPSPLKRLEEWIRRRLRLCRWHQWKRVRIRELRALGLKEHEVFEIANTRKGAWTPQLHKALGKAYWLKQG	yes	MPALLETILSRNNLITALKRVEANKGAPGIDGVPTEQLRDDIRKHWKSIKRQLLEGTYKPAPVRRVEIPKPNGGVRLLGIPTVMDRFIQQAILQVLTPIFDPHFSPYSYGFRPKRRAHDAVRQAQKYIQEGYRYVVDIDLEKFFDRVNHDILMSRVARKVEDKRVLKLIRAYLKAGVMLEGVRVRSEEGTPQGGPLSPLLANILLDDLDKELEKRGLKFCRYADDCNIYVRSPRAGQRVKQSVQKYLEKKLKLKVNEEKSAVDRPWKRKFLGFSFTSQREARIRLAPKSVQRFKNKIRQLTNPNWSLPMEERIRKLNQYTMGWMGYFALIETPSPLKRLEEWIRRRLRLCRWHQWKRVRTRIRELRALGLKEHEVFEIANTRKGAWRTTRTPQLHKALGKAYWLKQG	/	ATGCCTGCCCTTCTCGAGACAATTCTCAGCAGGAACAACCTGATCACCGCCCTCAAGAGAGTGGAGGCGAACAAGGGCGCCCCGGGCATTGATGGTGTACCAACAGAGCAGCTCAGAGATGATATCAGAAAGCACTGGAAAAGCATCAAGAGGCAGCTGCTTGAAGGCACATACAAGCCTGCACCAGTCCGGAGAGTGGAGATACCCAAACCAAATGGCGGCGTCAGACTGCTGGGCATCCCCACCGTCATGGATCGGTTCATCCAGCAGGCCATTTTGCAGGTGCTCACCCCTATCTTTGATCCGCACTTTTCGCCCTACTCCTACGGCTTCCGCCCAAAACGCCGCGCACACGACGCGGTGCGGCAGGCGCAGAAGTATATTCAGGAGGGCTACCGCTACGTCGTGGACATCGACCTGGAGAAATTCTTCGACAGAGTGAATCATGATATCCTCATGTCTCGTGTGGCGAGGAAGGTTGAAGACAAGAGAGTTCTGAAGCTGATCCGTGCCTACCTCAAAGCCGGAGTGATGTTAGAGGGTGTGAGAGTAAGGAGTGAAGAAGGAACCCCTCAAGGTGGCCCTTTGAGCCCTCTGCTTGCCAACATTCTGCTGGACGACCTCGACAAGGAACTCGAGAAGAGGGGCCTCAAATTCTGCCGGTATGCTGATGACTGCAACATATATGTGAGGAGCCCACGTGCCGGCCAACGTGTGAAGCAATCTGTTCAGAAGTACCTGGAAAAGAAGCTGAAGCTGAAGGTGAATGAAGAGAAAAGCGCCGTCGACAGACCATGGAAGCGCAAGTTTCTGGGATTCTCCTTCACAAGCCAGAGAGAAGCTAGGATCAGACTGGCACCAAAGAGCGTGCAGCGCTTCAAGAACAAAATCCGCCAATTGACAAATCCAAACTGGAGCCTGCCGATGGAGGAGAGAATCAGGAAGCTGAATCAATACACTATGGGGTGGATGGGGTATTTCGCGCTCATCGAGACTCCCTCGCCCCTGAAACGCCTGGAGGAGTGGATCAGAAGACGCCTGAGATTGTGTCGGTGGCACCAGTGGAAGAGAGTCCGCACGCGGATTCGAGAGCTCCGAGCACTGGGCTTGAAGGAGCATGAAGTTTTTGAAATCGCTAACACGCGAAAGGGCGCATGGAGGACCACCCGGACGCCGCAGCTCCACAAAGCGCTGGGTAAGGCTTACTGGCTCAAACAGGGG

RT 6	100	group_II_RT_mat	WP_205694286.1	61-469	group II intron reverse transcriptase/maturase	Clostridium manihotivorum	MNLALKRVISNKGSHGVDGMTVYKLKQFLKTNWISIRESILNGEYRPMPVRRVEIPKPNGGTRLLGIPTVLDRLIQQAIAQELNSIYDRRFSESSFGFRPQRGAKDAIKKAEQYINDGYRWVVDMLEKFFDKVNHDILMHKLSRSIKDKRVLSLIRKYLQSGIMINGVVVRNEEGTPQGGPLSPLLSNIMLDELDKELEMRGHKFCRYADDCNIYVKSERAGKRVMENITNFIEGKLKLKVNRDKSAVDRPWKRKFLGFTLNLMFGKAYSSISKQSLKRYKDKIREVLSRSKPITLEQRIEKLNQINIGWINYYGIAKCKGIVEHLDIWIRKRLRMCIWKQWKKVRTRYKNLKKLGLEHYEAIKFANTRKGYWRVANSAILNTTLTNKFFSDLGLKSLTHQYIKIHST	yes	MNLALKRVISNKGSHGVDGMTVYKLKQFLKTNWISIRESILNGEYRPMPVRRVEIPKPNGGTRLLGIPTVLDRLIQQAIAQELNSIYDRRFSESSFGFRPQRGAKDAIKKAEQYINDGYRWVVDMLEKFFDKVNHDILMHKLSRSIKDKRVLSLIRKYLQSGIMINGVVVRNEEGTPQGGPLSPLLSNIMLDELDKELEMRGHKFCRYADDCNIYVKSERAGKRVMENITNFIEGKLKLKVNRDKSAVDRPWKRKFLGFTLNLMFGKAYSSISKQSLKRYKDKIREVLSRSKPITLEQRIEKLNQINIGWINYYGIAKCKGIVEHLDIWIRKRLRMCIWKQWKKVRTRYKNLKKLGLEHYEAIKFANTRKGYWRVANSAILNTTLTNKFFSDLGLKSLTHQYIKIHST	**	ATGAACCTCGCGTTAAAGCGGGTAATTTCGAACAAGGGTTCTCATGGTGTTGATGGCATGACCGTCTACAAGCTCAAGCAATTTCTAAAAACAAATTGGATCAGCATCCGGGAATCAATCTTGAATGGCGAGTACCGCCCCATGCCGGTGAGACGGGTGGAAATTCCAAAGCCAAATGGTGGGACGCGCCTGTTGGGAATACCTACTGTTTTGGACCGGCTGATCCAGCAGGCGATTGCGCAGGAACTGAACAGTATATATGATCGTCGCTTCTCCGAAAGTTCCTTCGGCTTCAGGCCACAGCGTGGCGCCAAGGACGCCATAAAGAAGGCGGAGCAGTATATAAATGATGGATATCGATGGGTGGTCGACATGGATTTGGAGAAGTTCTTCGACAAAGTGAACCATGATATTCTGATGCATAAACTCTCTCGGAGCATAAAGGATAAACGAGTCCTATCCCTGATCAGGAAGTACCTCCAATCTGGTATAATGATCAACGGCGTGGTGGTTCGGAATGAGGAGGGAACTCCTCAAGGCGGCCCTCTTTCGCCGCTCCTCAGCAACATCATGCTGGATGAACTTGACAAGGAGCTTGAGATGAGGGGCCACAAGTTCTGCCGGTATGCTGATGATTGTAATATTTATGTGAAGAGTGAACGTGCCGGCAAGAGGGTGATGGAGAACATTACTAATTTCATCGAGGGGAAGCTGAAGCTTAAAGTCAACCGCGACAAGTCAGCTGTTGACCGGCCGTGGAAACGCAAATTCCTCGGATTCACTCTAAATCTCATGTTTGGAAAGGCATATTCTTCGATTTCTAAACAAAGTCTTAAAAGATACAAAGACAAGATTAGAGAAGTTCTTAGCCGCAGCAAGCCCATCACCCTGGAGCAAAGGATTGAAAAGCTCAACCAGATCAATATAGGGTGGATAAACTACTACGGTATTGCTAAGTGCAAGGGCATCGTCGAGCACCTGGACATATGGATTCGAAAACGTCTCAGGATGTGCATCTGGAAGCAGTGGAAGAAAGTTCGTACGAGGTACAAGAACCTTAAGAAGCTGGGTTTAGAACACTACGAGGCCATCAAGTTTGCAAATACAAGAAAAGGGTACTGGCGCGTCGCCAATTCTGCAATATTGAACACAACGCTCACCAACAAATTTTTTTCAGACCTCGGCCTCAAATCATTGACACATCAGTACATCAAAATTCACTCCACC
RT 7	101	group_II_RT_mat	XP_064717083.1	2-392	reverse transcriptase family protein PWA37_004942	Arxiozyma heterogenica	FIKEKPVNVLVDTGSPTSFIRADIADNLKLTRSTAPPFRFRGVVSSESSYTNESTEILLELDDIKIKTPVYITDAITFEIIIGHPIISSHPLLHKILNNKKPVPEYIVSVLTENDTEELDAENAAAVFAIKISETDTDDRFDKLPRWLKEKYSATVRDDLPPKKFAEPPKVEHEIEIKPGARLPRRQPYQTTPKIEQEINLIVADLLEKKFIVPSKSPCSSPVVLVKKKDGTYRLCVDYRALNTVTIKDPFPLPRIDNLLAKIGSSTIFSTLDLHSGYHQIPMKMDDRFKTAFVTPNGKYEYTVMPFGLVNAPSTFARYMADLFRDLPYVCVYLDDILIHSRSVDEHWKHIDIVLQRLTQEGLIVKKKKCNFAAKSVEFWDTLSLLIKSNR	yes	MFIKEKPVNVLVDTGSPTSFIRADIADNLKLTRSTAPPFRFRGVVSSESSYTNESTEILLELDDIKIKTPVYITDAITFEIIIGHPIISSHPLLHKILNNKKPVPEYIVSVLTENDTEELDAENAAAVFAIKISETDTDDRFDKLPRWLKEKYSATVRDDLPPKKFAEPPKVEHEIEIKPGARLPRRQPYQTTPKIEQEINLIVADLLEKKFIVPSKSPCSSPVVLVKKKDGTYRLCVDYRALNTVTIKDPFPLPRIDNLLAKIGSSTIFSTLDLHSGYHQIPMKMDDRFKTAFVTPNGKYEYTVMPFGLVNAPSTFARYMADLFRDLPYVCVYLDDILIHSRSVDEHWKHIDIVLQRLTQEGLIVKKKKCNFAAKSVEFWDTLSLLIKSNR	*	ATGTTCATCAAGGAGAAACCAGTGAATGTTCTGGTGGATACAGGTTCACCAACCTCCTTTATCAGGGCTGATATTGCAGATAATCTGAAGCTCACGCGGTCCACGGCGCCGCCGTTCCGGTTCCGTGGCGTCGTCTCCTCTGAAAGCTCGTATACAAATGAATCAACAGAAATTTTGCTGGAGCTTGATGACATCAAGATAAAAACACCCGTCTACATCACCGACGCCATCACTTTTGAGATAATAATTGGCCACCCCATCATCAGTAGCCACCCGCTGCTCCACAAGATACTGAACAACAAGAAGCCGGTGCCTGAATATATTGTTTCTGTGCTCACTGAAAATGATACTGAGGAGTTGGATGCTGAGAACGCCGCCGCAGTCTTCGCCATTAAAATTTCCGAGACGGACACAGATGATAGGTTCGACAAGCTGCCGCGGTGGCTCAAGGAAAAATATTCAGCAACTGTTAGAGACGATCTTCCTCCAAAAAAATTTGCTGAACCTCCCAAGGTGGAGCATGAGATTGAAATAAAGCCTGGCGCGCGCCTCCCCCGCCGGCAGCCCTATCAAACAACTCCAAAAATTGAGCAAGAAATCAACCTGATAGTTGCTGACCTACTTGAGAAGAAGTTCATAGTTCCAAGCAAGTCGCCGTGCTCCAGCCCTGTGGTTCTTGTAAAAAAGAAGGACGGCACCTATAGATTATGTGTCGACTACCGCGCGCTCAACACCGTCACCATCAAAGATCCATTTCCGCTCCCTCGCATCGATAACCTCCTCGCCAAGATTGGAAGTTCAACTATCTTCTCCACGCTGGATCTACATTCCGGGTACCACCAGATCCCAATGAAGATGGACGACCGCTTCAAGACCGCCTTTGTCACTCCAAATGGAAAGTACGAGTACACGGTGATGCCGTTTGGTCTTGTCAATGCACCTAGCACATTTGCGAGATACATGGCCGACCTCTTCCGTGATTTGCCCTACGTGTGCGTGTACCTCGACGACATTCTCATTCATTCTCGATCTGTTGATGAGCACTGGAAGCATATTGATATCGTCCTCCAGAGGCTCACGCAGGAGGGGCTGATCGTGAAGAAAAAGAAATGCAACTTCGCGGCGAAATCAGTTGAGTTCTGGGACACCCTATCGTTGTTAATCAAGTCGAATAGA
RT 8	102	group_II_RT_mat	WP_318077048.1	2-340	MULTISPECIES: retron St85 family RNA-directed DNA polymerase	Vibrio	YEHFAVSSKELSPRASLLLGFFVERNMNLVKRLARHLGKSELEVIHFLADAPNKYRVYKIPKRSYGHRIIAQPTRELKQYQRAFLELYTFPVHSSATAYCKGKSIKDNALSHVKNHYLLKTDLENFFNSITPSIFWKSIETNSITTPKFSASEIDLVERLIFWRPSKLQGGKLVLSVGAPSSPTISNFCLYQFDEYLSLICKEQNISYTRYADDLTFSTCEKDVLHTVIPLIQSLLDYIFASELKLNRSKTVFSSKAHNRHVTGVTLSNKGTLSLGRERKRYIKHLVHSFKYGKLDDSEIRHLQGMLSFAKHIEPIFIDRLKEKYTNELIKRIYEAGHE	yes	MYEHFAVSSKELSPRASLLLGFFVERNMNLVKRLARHLGKSELEVIHFLADAPNKYRVYKIPKRSYGHRIIAQPTRELKQYQRAFLELYTFPVHSSATAYCKGKSIKDNALSHVKNHYLLKTDLENFFNSITPSIFWKSIETNSITTPKFSASEIDLVERLIFWRPSKLQGGKLVLSVGAPSSPTISNFCLYQFDEYLSLICKEQNISYTRYADDLTFSTCEKDVLHTVIPLIQSLLDYIFASELKLNRSKTVFSSKAHNRHVTGVTLSNKGTLSLGRERKRYIKHLVHSFKYGKLDDSEIRHLQGMLSFAKHIEPIFIDRLKEKYTNELIKRIYEAGHE	/	ATGTACGAGCACTTCGCCGTCAGTAGCAAGGAACTCTCGCCGCGCGCGTCGCTGCTCCTCGGGTTCTTTGTTGAGCGGAACATGAACCTTGTGAAGCGATTGGCGCGGCACCTCGGGAAGAGCGAACTTGAGGTGATTCATTTCCTCGCCGACGCCCCAAATAAATATCGTGTGTACAAGATTCCAAAACGGAGCTATGGCCACCGCATCATTGCTCAACCAACAAGAGAGTTGAAGCAGTACCAAAGAGCATTTCTGGAGCTCTACACCTTTCCGGTGCATTCTTCAGCGACGGCCTACTGCAAAGGAAAATCCATCAAGGACAATGCACTCAGCCACGTCAAGAACCACTACCTGCTGAAGACAGATCTAGAAAATTTCTTCAATTCAATCACCCCTTCGATCTTCTGGAAGTCGATTGAAACAAATAGCATAACGACTCCCAAGTTCAGTGCATCTGAAATCGATCTTGTGGAGCGACTGATATTTTGGAGGCCCTCAAAGCTACAGGGCGGCAAGCTAGTGTTGTCAGTTGGTGCGCCGTCGTCGCCTACTATATCAAACTTCTGCCTATACCAGTTTGATGAGTACCTCAGCCTCATCTGCAAGGAGCAGAACATATCTTATACGAGGTATGCTGACGACCTCACCTTCAGCACTTGTGAGAAAGATGTTCTGCACACCGTCATCCCTCTCATTCAGTCCCTTCTTGACTACATCTTTGCATCAGAGTTGAAGCTTAATAGATCTAAAACTGTCTTCTCCTCCAAGGCGCACAACCGCCATGTAACCGGTGTCACGCTGAGCAACAAGGGAACCTTGAGTTTGGGAAGGGAAAGGAAGAGATATATTAAACATCTTGTTCATAGTTTCAAGTATGGTAAACTTGATGATTCTGAGATCCGCCATTTGCAAGGCATGCTGTCTTTTGCCAAGCACATCGAGCCGATTTTCATCGACCGGCTGAAAGAGAAGTACACAAATGAGTTAATAAAGAGGATTTATGAGGCCGGCCATGAA
RT 9	103	group_II_RT_mat	CAD7676357.1	1-523	unnamed protein product	Nyctereutes procyonoides	RPMLLLSLGSYSQLVAPLSKIEPILAPCRGPNVRLGPGTLGSRPEAKGRCSTAEPPRHPILSILLIFQKAIFCIVHQVFLLVFFFLFLLLFCCCCFYFLSTQHSVLTEAKGPLSSKTSWVQGATGGKLYRWTTERKVHLSTGQVTHSFLLVPDCPYPLLGRDLLSKVGAQIPFQQKGATITGAGGQPLQVLTLRLEDEHRLHEDSPPSVQPLDSEWLTNYPQAWVETAGMGLAVNQPPIIINLKPSATPISIRQYSMSKEAKEGIRPHIQRLLQLGILIPCQSPWNTPLLPVKKPGTGDYRPVQDLREVNWRTEDIHPTVPNPYNLLSTLPPSHVWYTVLDLKDAFFCLRLSSQSQPIFAFEWKDPETGFSGQLTWTRLPQGFKNSPTLFDEALHRDLADFRVGHPDLVLLQYVDDLLLAARTEQDCVKGTGALLERLGELGYRASAKKAQVVMHDCHQILAEVHGTRGDLTDQPLPDAEATWYTDGSSFLRNGWTEAFPTKRETAQVVVKKILEEIFPGLDCPRGA	yes	MRPMLLLSLGSYSQLVAPLSKIEPILAPCRGPNVRLGPGTLGSRPEAKGRCSTAEPPRHPILSILLIFQKAIFCIVHQVFLLVFFFLFLLLFCCCCFYFLSTQHSVLTEAKGPLSSKTSWVQGATGGKLYRWTTERKVHLSTGQVTHSFLLVPDCPYPLLGRDLLSKVGAQIPFQQKGATITGAGGQPLQVLTLRLEDEHRLHEDSPPSVQPLDSEWLTNYPQAWVETAGMGLAVNQPPIIINLKPSATPISIRQYSMSKEAKEGIRPHIQRLLQLGILIPCQSPWNTPLLPVKKPGTGDYRPVQDLREVNWRTEDIHPTVPNPYNLLSTLPPSHVWYTVLDLKDAFFCLRLSSQSQPIFAFEWKDPETGFSGQLTWTRLPQGFKNSPTLFDEALHRDLADFRVGHPDLVLLQYVDDLLLAARTEQDCVKGTGALLERLGELGYRASAKKAQVVMHDCHQILAEVHGTRGDLTDQPLPDAEATWYTDGSSFLRNGWTEAFPTKRETAQVVVKKILEEIFPGLDCPRGA	/	ATGAGGCCTATGCTGCTGCTTTCCTTGGGTTCCTACAGCCAGCTGGTCGCGCCGCTCTCCAAGATTGAGCCCATTCTTGCCCCGTGCCGCGGGCCTAATGTTAGACTGGGCCCCGGAACACTGGGTTCGCGGCCGGAGGCAAAAGGTCGCTGCTCCACGGCCGAGCCGCCCAGACATCCAATTCTCTCCATTCTGCTAATATTTCAGAAGGCCATCTTCTGTATTGTTCACCAGGTTTTTTTGCTCGTCTTCTTCTTCCTGTTTCTTCTCCTCTTCTGCTGTTGCTGCTTCTACTTCCTCTCAACACAGCACTCAGTGCTGACTGAAGCAAAAGGACCACTCTCTTCAAAAACGTCATGGGTTCAAGGAGCAACTGGCGGAAAGCTCTACAGGTGGACTACTGAAAGGAAGGTGCACCTCTCGACGGGTCAAGTCACACATTCATTTCTCCTTGTGCCGGACTGTCCATATCCTCTTCTTGGAAGGGATCTGCTGAGTAAAGTTGGCGCGCAGATCCCTTTCCAGCAGAAAGGTGCTACCATCACCGGCGCCGGCGGCCAGCCACTGCAAGTCCTGACACTGCGGCTTGAGGATGAACACCGTCTACATGAGGACAGCCCTCCCTCCGTACAACCACTGGACTCTGAATGGCTGACCAATTATCCACAGGCGTGGGTGGAGACGGCGGGCATGGGGCTTGCCGTCAACCAGCCGCCAATAATCATCAACCTGAAACCATCGGCGACGCCGATCAGCATCCGGCAGTATTCCATGAGCAAGGAAGCTAAGGAGGGCATCAGGCCGCACATCCAGAGGTTGCTGCAACTCGGTATTTTAATTCCGTGCCAGTCACCCTGGAACACCCCGTTGCTACCAGTGAAGAAGCCAGGGACCGGGGACTACCGCCCGGTGCAGGATTTAAGGGAGGTGAACTGGAGAACAGAAGATATTCACCCAACAGTACCAAATCCTTACAATTTGTTAAGCACCCTCCCGCCCTCTCATGTTTGGTACACCGTCCTCGATTTGAAGGACGCCTTCTTTTGCCTCCGCCTATCGTCACAGAGCCAACCCATATTTGCATTTGAGTGGAAGGATCCAGAGACAGGCTTCTCTGGGCAGCTCACATGGACAAGACTTCCTCAAGGCTTCAAGAACTCCCCCACCCTGTTCGACGAGGCGCTCCACCGGGATTTGGCCGACTTCCGCGTCGGCCACCCCGACCTCGTTCTCCTTCAGTACGTGGACGACTTATTGCTCGCGGCGCGCACCGAGCAAGATTGTGTGAAAGGGACAGGAGCTCTCCTCGAGAGGCTTGGCGAGCTCGGCTATCGAGCATCCGCCAAGAAAGCTCAAGTGGTGATGCATGACTGCCATCAAATATTAGCTGAAGTTCATGGGACCCGTGGTGATCTTACAGATCAACCTTTGCCTGATGCAGAAGCCACCTGGTATACTGATGGAAGTAGTTTCCTTAGAAATGGGTGGACGGAGGCTTTCCCGACCAAGAGGGAAACTGCTCAGGTGGTCGTCAAGAAGATCCTGGAGGAGATATTTCCTGGTCTTGATTGCCCCCGTGGCGCC
RT 10	104	group_II_RT_mat	XP_036063566.1	1-505	uncharacterized protein LOC118596762	Onychomys torridus	METPSDRLPPHGLSRFSPFQRPRPPPTAPPPPLAPPTSSLYPILPRKDPPKTPVLPADPNSPLIDLLTLLTGGGSPRISSTGTAGGGGPGALSNCRSPTRKARDEPAKGATGSRRYRWTTERKVQLASGQVTHSFLHIPDCSHPLLGRDLLTKLKAQIYFDDKGSTVMGPKGTPLQVLALKLEEEYRLFESESSKEPSEGIQGWLREFPSSWAETGGLGLARDQPPLVIQLKASATHVSIKQYPMSREAHEGIKPHIRRLLDQGVLVPCRSPWNTPLLPVKKPGIGDYRPVQDLREVNKRVEDIHPMVLNPYNLLCTLPPTHIWYTVLDLKDAFFCLRLHPQSQLLFAFEWRDPEMGLSGQLTWTRLPQGFKNSPTLFDEALHSDLAEFRVKHPALILLQYVDDLLLAARTQAECLRGTRALLTKLGQKSYRASAKKAQICQSKVIYLVQPATAASGSQVLLRWLPLYILSPSLGSCSTGESSNSRPFNEFRELYSSHQLLAYQI	yes	METPSDRLPPHGLSRFSPFQRPRPPPTAPPPPLAPPTSSLYPILPRKDPPKTPVLPADPNSPLIDLLTLLTGGGSPRISSTGTAGGGGPGALSNCRSPTRKARDEPAKGATGSRRYRWTTERKVQLASGQVTHSFLHIPDCSHPLLGRDLLTKLKAQIYFDDKGSTVMGPKGTPLQVLALKLEEEYRLFESESSKEPSEGIQGWLREFPSSWAETGGLGLARDQPPLVIQLKASATHVSIKQYPMSREAHEGIKPHIRRLLDQGVLVPCRSPWNTPLLPVKKPGIGDYRPVQDLREVNKRVEDIHPMVLNPYNLLCTLPPTHIWYTVLDLKDAFFCLRLHPQSQLLFAFEWRDPEMGLSGQLTWTRLPQGFKNSPTLFDEALHSDLAEFRVKHPALILLQYVDDLLLAARTQAECLRGTRALLTKLGQKSYRASAKKAQICQSKVIYLVQPATAASGSQVLLRWLPLYILSPSLGSCSTGESSNSRPFNEFRELYSSHQLLAYQI	/	ATGGAGACACCCAGCGACAGGTTGCCGCCGCACGGCCTCTCCCGTTTTTCACCCTTCCAGCGCCCCCGCCCACCACCTACTGCACCTCCTCCGCCGCTGGCACCGCCGACGTCGTCGCTGTATCCAATTCTTCCTAGGAAAGATCCACCAAAAACTCCAGTTCTGCCAGCTGATCCGAATTCACCATTGATTGATCTCCTCACATTATTGACTGGCGGCGGCTCGCCGCGGATTTCAAGCACTGGTACAGCCGGCGGTGGAGGGCCAGGCGCCTTAAGCAACTGTAGATCTCCAACCAGGAAGGCCAGGGATGAGCCTGCAAAAGGAGCTACAGGCTCCCGCAGATATAGATGGACCACCGAGCGGAAGGTTCAGCTGGCATCCGGGCAAGTCACTCATTCTTTCCTGCATATTCCTGATTGTAGCCACCCGCTGCTCGGCCGCGACCTCCTCACCAAGCTGAAAGCACAGATCTATTTCGACGACAAGGGGTCGACGGTGATGGGTCCCAAGGGCACGCCGCTCCAAGTTTTGGCGCTAAAACTGGAAGAGGAGTACAGGCTCTTCGAGAGTGAATCATCAAAGGAGCCGTCAGAAGGAATCCAGGGCTGGCTGCGGGAATTCCCCTCCAGCTGGGCTGAAACCGGAGGGCTTGGTCTTGCGCGGGACCAGCCTCCACTGGTGATTCAACTGAAGGCGAGCGCCACCCATGTTTCTATCAAGCAGTATCCCATGAGCCGCGAGGCGCATGAGGGCATCAAACCACACATTAGACGGCTGCTGGATCAAGGAGTGCTCGTACCGTGCCGCTCCCCATGGAACACACCTTTGTTGCCGGTAAAGAAACCTGGTATTGGCGACTACCGACCTGTTCAAGATTTGAGAGAGGTGAACAAGAGGGTGGAGGACATCCACCCAATGGTGCTGAATCCCTACAACTTACTTTGTACTCTTCCCCCAACACATATATGGTACACCGTCCTCGACCTCAAGGACGCCTTTTTCTGCCTCCGCCTCCATCCACAATCTCAACTACTTTTTGCTTTTGAGTGGAGGGACCCGGAGATGGGGCTTTCCGGCCAACTTACATGGACAAGACTTCCTCAAGGGTTCAAGAACTCCCCCACCCTTTTTGATGAAGCTCTGCACAGCGATCTCGCAGAATTTCGCGTCAAGCACCCCGCCCTCATACTCCTGCAGTACGTCGATGATCTATTATTGGCCGCGCGCACGCAGGCCGAGTGCTTGAGGGGAACTAGAGCGCTATTAACGAAATTGGGTCAGAAGAGCTATCGCGCCTCCGCTAAGAAGGCACAAATCTGCCAGAGCAAGGTCATCTACCTCGTGCAGCCGGCGACCGCTGCTTCCGGCAGTCAGGTATTGCTCAGGTGGCTTCCCCTGTACATCCTAAGTCCCTCTCTTGGAAGCTGCAGCACTGGTGAAAGTTCAAATAGTCGTCCGTTCAATGAGTTCCGGGAGCTTTATTCTTCCCACCAGCTTCTGGCATACCAGATA

RT 11	108	ps-ssRNAv_RdRp-like superfamily	AGV92856.1	119-826	polymerase, partial	Echidna ERV	PLSSPLVEEYRLFVEQPAQNLALLDLWREDIPEVWAESNPPGLATTQVPVHVQLTSTALPIRIRQYPISLEARRSLRGSIRKFKAAGILKPVHSPWNTPLLPVRKTGTSEYRMVQDLREVNKRVETIHPTVPNPYTLLSLLPPDWYSVLDLKDAFFCIPLTCQSQLLFAFEWIDIEEGESGQLTWTRLPQGFKNSPTLFDEALSRDLQGYRFDHPTVTLLQYVDDLLIAARSRDECLQATRDLLVTLGSMGYRVSGSKAQLCQEEVTYLGFRIKDGTLAQSRVQAILQIPAPKTKKQVREFLGTVGYCRLWIPSFAELAQPLYAATRGADAPLRWTGTEEEAFQRLKTALLQPPALALPNLDKPFQLFVDEAKGVAKGVLMQTLGPWKRPVAYLSRKLDPLAAGWPRCLRAIAAAALLSKEASKLTFEQSLEITSSHNLEGLLPPDKWLTNARVTQYQVLLLDPPRVIFKQTAALNSIVWAEALPRGTSAQRAELIALTKALEWSRDKTVNIYTDSRYAFATLHVHAMIYKERGLNPATLLPATDDSLPLHHCADTLDALTTTRPDLTDQPLADAEATLFTDGSSYVKKAEYAGAAVVTTLTAGGKAIKNASEILALLTAIWLPKRVAVIHCRGHQQGESLEALGNRLADKTAREVAKKSPAIQASLCDPPPVDWVPADTPQYTKQEEALGQRLGGTTDPT	yes	MPLSSPLVEEYRLFVEQPAQNLALLDLWREDIPEVWAESNPPGLATTQVPVHVQLTSTALPIRIRQYPISLEARRSLRGSIRKFKAAGILKPVHSPWNTPLLPVRKTGTSEYRMVQDLREVNKRVETIHPTVPNPYTLLSLLPPDRTWYSVLDLKDAFFCIPLTCQSQLLFAFEWIDIEEGESGQLTWTRLPQGFKNSPTLFDEALSRDLQGYRFDHPTVTLLQYVDDLLIAARSRDECLQATRDLLVTLGSMGYRVSGSKAQLCQEEVTYLGFRIKDGTRTLAQSRVQAILQIPAPKTKKQVREFLGTVGYCRLWIPSFAELAQPLYAATRGADAPLRWTGTEEEAFQRLKTALLQPPALALPNLDKPFQLFVDEAKGVAKGVLMQTLGPWKRPVAYLSRKLDPLAAGWPRCLRAIAAAALLSKEASKLTFEQSLEITSSHNLEGLLRTPPDKWLTNARVTQYQVLLLDPPRVIFKQTAALNPATLLPATDDSLPLHHCADTLDALTTTRPDLTDQPLADAEATLFTDGSSYVKKAEYAGAAVVTTNSIVWAEALPRGTSAQRAELIALTKALEWSRDKTVNIYTDSRYAFATLHVHAMIYKERGLLTAGGKAIKNASEILALLTAIWLPKRVAVIHCRGHQQGESLEALGNRLADKTAREVAKKSPAIQASLCDPPRTPVDWVPADTPQYTKQEEALGQRLGGTTDPT	/	ATGCCTCTCAGCTCCCCACTGGTGGAGGAATACCGCCTGTTCGTTGAGCAGCCGGCTCAGAACCTTGCCTTACTCGACCTTTGGAGGGAGGACATACCTGAGGTGTGGGCAGAATCCAACCCACCTGGACTGGCCACCACACAGGTTCCTGTGCATGTCCAGCTGACCTCAACCGCCTTACCAATTAGAATACGCCAGTACCCTATATCCCTGGAGGCTAGACGGTCCCTCCGCGGCAGCATCAGGAAATTTAAAGCCGCGGGCATCCTGAAACCTGTTCATAGTCCTTGGAATACGCCTCTGCTGCCAGTGCGCAAGACTGGGACCTCTGAGTATAGGATGGTACAAGATCTGCGAGAAGTGAATAAACGGGTGGAGACCATCCACCCAACTGTGCCTAATCCCTATACGCTGCTGAGCCTGCTCCCTCCTGATAGAACATGGTACTCGGTACTTGATCTCAAGGACGCTTTCTTCTGCATCCCACTCACTTGCCAATCACAGCTGCTTTTCGCATTTGAGTGGATTGATATCGAGGAGGGTGAATCTGGCCAGCTCACGTGGACGAGGCTGCCACAGGGTTTCAAGAATTCTCCGACTCTATTTGATGAGGCTCTGAGTCGCGATCTGCAGGGATATCGTTTCGACCACCCCACTGTGACCCTGCTTCAATATGTGGACGACCTGCTCATAGCTGCGAGGAGCCGGGATGAGTGCCTCCAGGCGACCAGAGACCTCCTCGTCACCCTGGGTTCGATGGGTTACAGGGTGAGCGGCTCTAAGGCGCAACTATGCCAAGAGGAGGTCACTTATCTGGGCTTCCGGATCAAAGATGGCACCCGTACACTGGCTCAGAGCCGGGTTCAGGCGATCTTGCAAATCCCTGCCCCCAAGACTAAGAAGCAGGTCCGGGAGTTCCTGGGAACCGTCGGGTACTGCAGGCTATGGATCCCATCCTTCGCCGAGCTGGCTCAACCTCTCTACGCTGCGACACGGGGAGCCGACGCGCCGCTCCGGTGGACAGGGACAGAGGAAGAAGCCTTCCAGAGACTGAAAACAGCCTTGTTGCAGCCCCCTGCACTAGCTTTGCCCAACCTTGACAAACCTTTCCAACTGTTTGTGGATGAAGCGAAGGGTGTGGCAAAAGGAGTGCTCATGCAGACACTTGGCCCATGGAAGAGACCAGTGGCCTACCTCTCTCGGAAGCTCGACCCGCTGGCCGCCGGCTGGCCTCGATGTTTGAGGGCCATCGCTGCCGCCGCCCTCCTGTCTAAGGAAGCTAGCAAACTTACATTTGAGCAAAGCCTCGAAATTACTTCCAGCCACAATCTTGAGGGCCTACTGCGCACCCCGCCAGACAAATGGCTGACCAACGCACGCGTGACACAGTACCAGGTGCTGCTGCTGGATCCTCCCAGAGTGATCTTCAAGCAAACAGCGGCCCTCAACCCGGCCACCTTGCTGCCTGCGACTGATGATTCGCTGCCGCTCCACCACTGCGCGGATACCTTGGACGCCCTGACCACCACTCGGCCAGATCTAACAGACCAGCCGCTCGCCGATGCCGAAGCTACACTTTTCACAGACGGCTCCTCCTACGTGAAGAAGGCTGAATACGCGGGCGCCGCGGTGGTGACCACAAACAGCATCGTTTGGGCCGAGGCCCTGCCCAGAGGTACTTCCGCGCAGAGAGCTGAGCTGATCGCCCTGACTAAGGCTTTAGAATGGAGCAGAGATAAGACAGTCAACATCTACACGGACTCTAGATATGCTTTTGCTACACTCCACGTCCACGCGATGATTTACAAGGAGCGTGGCCTGCTCACTGCGGGCGGAAGGCCATCAAGAACGCGAGCGAAATTCTTGCCCTGCTCACTGCCATCTGGCTACCTAAACGGGTGGCCGTCATCCATTGTCGAGGCCACCAGCAGGGCGAGAGCCTCGAGGCTCTGGGAAACCGCCTGGCAGACAAGACTGCTAGAGAAGTAGCCAAGAAGTCTCCCGCCATTCAGGCTTCTCTTTGTGATCCACCTAGAACGCCCGTTGATTGGGTGCCGGCTGACACCCCGCAGTACACTAAACAAGAAGAAGCCCTGGGCCAGAGACTAGGCGGCACCACAGACCCAACA
RT 12	110	ps-ssRNAv_RdRp-like superfamily	AEF12615.1	81-335	pol protein	Porcine endogenous retrovirus B	YSPQVKPDQDIQSWLEQFPQAWAETAGMGLAKQVPPQVIQLKASATPVSVRQYPLSREAREGIWPHVQRLIQQGILVPVQSPWNTPLLPVRKPGTNDYRPVQDLREVNKRVQDIHPTVPNPYNLLSALPPERNWYTVLDLKDAFFCLRLHPTSQPLFTFEWRDPGTGRTGQLTWTRLPQGFKNSPTIFDEALHRDLANFRIQHPQVTLLQYVDDLLLAGATKQDCLEGTKALLLELSDLGYRASAKKAQICRREV	yes	MYSPQVKPDQDIQSWLEQFPQAWAETAGMGLAKQVPPQVIQLKASATPVSVRQYPLSREAREGIWPHVQRLIQQGILVPVQSPWNTPLLPVRKPGTNDYRPVQDLREVNKRVQDIHPTVPNPYNLLSALPPERNWYTVLDLKDAFFCLRLHPTSQPLFTFEWRDPGTGRTGQLTWTRLPQGFKNSPTIFDEALHRDLANFRIQHPQVTLLQYVDDLLLAGATKQDCLEGTKALLLELSDLGYRASAKKAQICRREV	/	ATGTATTCGCCGCAGGTGAAGCCGGATCAAGATATTCAAAGCTGGCTCGAGCAGTTCCCTCAGGCGTGGGCGGAGACGGCCGGCATGGGCCTCGCCAAGCAGGTTCCCCCACAGGTCATCCAGCTCAAAGCGTCCGCCACCCCTGTTTCCGTACGGCAGTATCCTTTAAGCCGGGAGGCCAGAGAAGGTATATGGCCACATGTCCAGAGGTTGATACAGCAGGGCATCCTGGTGCCGGTCCAATCTCCATGGAACACACCACTCCTTCCGGTGAGAAAGCCCGGGACAAATGATTATAGGCCAGTGCAGGATCTGCGAGAAGTCAACAAGAGGGTTCAGGACATCCACCCGACTGTTCCAAATCCCTACAACCTCCTCTCGGCGCTGCCGCCGGAGAGGAACTGGTACACCGTCCTTGACCTCAAGGACGCCTTCTTCTGCCTCCGCCTGCATCCTACTTCACAGCCGCTCTTCACCTTTGAGTGGAGGGATCCTGGAACTGGAAGAACAGGTCAATTGACATGGACGCGCTTGCCGCAAGGGTTCAAGAATTCACCCACCATCTTTGATGAGGCGCTCCACCGCGACTTGGCAAATTTTCGTATTCAACACCCACAAGTCACGCTGCTGCAGTACGTGGACGACCTTCTTCTTGCTGGCGCTACAAAACAAGACTGCTTGGAAGGCACGAAAGCACTTTTGCTCGAACTTTCTGATCTTGGGTACCGCGCTAGTGCAAAGAAGGCGCAGATTTGTAGACGCGAGGTG
RT 13	111	ps-ssRNAv_RdRp-like superfamily	XP_027730318.1	505-677	LOW QUALITY PROTEIN: uncharacterized protein LOC114	Vombatus ursinus	GSHMTWLSDFPQAWAETGGMGLAVRQAPLIIPLKATSTPVSIKQYPMSQEARLGIKPHIQRLLDQGILVPCQSPWNTPLLPVKKPGTNDYRPVQDLREVNKRVEDIHPTVPNPYNLLSGLPPSHQWYTVLDLKDAFFCLRLHPTSQPLFAFEWRDPEMGISGQLTWTRLPQGFKNSPTLFDEALHRDLADFRIQHPDLILLQYVDDLLLAATSELDCQQGTRALLQTLGNLGYRASAKKAQICQKQVKYLGYLLKEGQRLTRGSGC	yes	MGSHMTWLSDFPQAWAETGGMGLAVRQAPLIIPLKATSTPVSIKQYPMSQEARLGIKPHIQRLLDQGILVPCQSPWNTPLLPVKKPGTNDYRPVQDLREVNKRVEDIHPTVPNPYNLLSGLPPSHQWYTVLDLKDAFFCLRLHPTSQPLFAFEWRDPEMGISGQLTWTRLPQGFKNSPTLFDEALHRDLADFRIQHPDLILLQYVDDLLLAATSELDCQQGTRALLQTLGNLGYRASAKKAQICQKQVKYLGYLLKEGQRLTRGSGC	/	ATGGGAAGCCACATGACCTGGTTGTCAGATTTCCCGCAGGCGTGGGCAGAAACTGGCGGCATGGGCCTGGCGGTGCGGCAAGCACCGCTCATCATCCCCCTTAAAGCTACTTCTACACCAGTTTCCATCAAGCAGTACCCCATGTCGCAGGAGGCGAGATTAGGTATAAAACCACACATCCAGAGGCTTCTTGATCAAGGAATTCTTGTTCCTTGTCAATCACCATGGAACACCCCTCTTTTACCGGTGAAGAAACCTGGAACAAATGATTATAGACCTGTCCAAGATCTGAGGGAGGTGAACAAGAGGGTGGAGGATATTCATCCAACTGTTCCAAATCCATACAACCTGTTGAGTGGGCTGCCGCCGAGCCATCAGTGGTACACGGTGCTCGACCTGAAAGACGCCTTCTTCTGCCTGCGGCTCCACCCCACGAGTCAACCATTGTTTGCATTTGAGTGGAGGGACCCGGAGATGGGTATTTCTGGTCAGCTGACATGGACAAGGTTGCCACAAGGCTTCAAGAATTCGCCGACGCTCTTTGATGAAGCTTTGCACCGCGACCTCGCCGACTTCCGCATTCAGCATCCTGATTTAATACTCCTCCAGTATGTCGACGACCTTCTCCTGGCCGCCACCTCCGAGCTCGACTGCCAGCAGGGCACCCGGGCGCTCCTGCAAACACTAGGCAACCTCGGGTACCGCGCTTCCGCCAAGAAGGCGCAGATATGTCAGAAGCAAGTCAAGTACCTCGGATATCTATTGAAAGAAGGGCAGCGCCTCACTAGAGGAAGCGGCTGC
RT 14	112	ps-ssRNAv_RdRp-like superfamily	XP_058431757.1	477-735	LOW QUALITY PROTEIN: uncharacterized protein LOC131	Marmota monax	EDEYKLFDKPSVSMKDMDYWLSSYPEAWAETGGMGMAKQRPPIVIHLRAAAIPINIKQYPMSREAYQGIKPHIKRLLDQGILTPCQSPWNTPLLPVKKPGTNDYRPVQDLREVNKRVEDIHPTVPNPYNLLSTLPPTHTWYTVLDLKDAFFCLRLSPQSQALFAFEWKDPEGGISGQLTWTRLPQGFKNSPTLFDEALHQDLADFRVRHPSLIMLQYVDDILLAATSEEDCQAGTEELLQTLGLLGYRASAKKAQICQT	yes	MEDEYKLFDKPSVSMKDMDYWLSSYPEAWAETGGMGMAKQRPPIVIHLRAAAIPINIKQYPMSREAYQGIKPHIKRLLDQGILTPCQSPWNTPLLPVKKPGTNDYRPVQDLREVNKRVEDIHPTVPNPYNLLSTLPPTHTWYTVLDLKDAFFCLRLSPQSQALFAFEWKDPEGGISGQLTWTRLPQGFKNSPTLFDEALHQDLADFRVRHPSLIMLQYVDDILLAATSEEDCQAGTEELLQTLGLLGYRASAKKAQICQT	/	ATGGAAGATGAATACAAGCTCTTCGACAAGCCGTCGGTAAGCATGAAGGACATGGATTACTGGCTCTCAAGTTATCCTGAGGCGTGGGCGGAGACTGGTGGAATGGGCATGGCCAAGCAGAGACCGCCGATAGTCATCCATCTTCGCGCGGCGGCAATTCCCATCAACATCAAGCAATACCCCATGAGCAGAGAAGCATATCAAGGAATAAAACCGCACATCAAAAGACTGCTTGACCAGGGGATTCTTACTCCATGTCAATCTCCATGGAACACCCCACTTCTGCCTGTGAAGAAGCCTGGTACAAATGATTATAGGCCGGTGCAGGACCTGCGAGAGGTGAACAAGAGGGTGGAGGACATTCATCCAACTGTTCCAAATCCATACAACCTGCTGTCCACACTTCCGCCCACGCACACCTGGTACACCGTCCTCGACCTCAAAGATGCTTTCTTCTGCCTCCGGTTGTCACCTCAATCACAGGCGCTCTTTGCATTTGAGTGGAAGGATCCGGAAGGCGGCATTAGCGGGCAGCTGACATGGACGCGGCTCCCGCAGGGTTTCAAGAATTCCCCCACCCTGTTTGATGAAGCTCTGCACCAGGATTTGGCTGACTTCCGTGTCAGGCATCCCTCCCTCATCATGCTACAGTATGTTGATGATATTCTCCTCGCCGCCACCAGTGAAGAGGACTGCCAGGCCGGCACGGAGGAGCTGTTGCAAACATTAGGACTTCTCGGCTACCGCGCCTCCGCAAAAAAAGCGCAGATATGCCAAACT
RT 15	113	ps-ssRNAv_RdRp-like superfamily	AAC16767.1	173-339	pol protein	Sus scrofa	WLRLVPEAWAETAGLGLSTLQAPVVVELKTTAVPVRVRQYPMSQEAKKGITGFLVKCQSAWNTPLLPVKKPGTGDYRPVQDLRAVNSQVEVIHPTVPNPYNLLSTLSPERVWYTVLDLKDAFFCLSLHPASQPLFAFEWSDPEAGISGQLTWTRLPQGFKNSPTIFDEALHQDLSLFRSQHPQVTLLQYVDDILIAGKTEEDCKQATHNLLKELARLGYRASAKKAQLCQREVTFLGYMLRGGKRWLTEARKKVVAQIPAPTT	yes	MWLRLVPEAWAETAGLGLSTLQAPVVVELKTTAVPVRVRQYPMSQEAKKGITGFLVKCQSAWNTPLLPVKKPGTGDYRPVQDLRAVNSQVEVIHPTVPNPYNLLSTLSPERVWYTVLDLKDAFFCLSLHPASQPLFAFEWSDPEAGISGQLTWTRLPQGFKNSPTIFDEALHQDLSLFRSQHPQVTLLQYVDDILIAGKTEEDCKQATHNLLKELARLGYRASAKKAQLCQREVTFLGYMLRGGKRWLTEARKKVVAQIPAPTT	/	ATGTGGCTGCGGCTGGTACCTGAAGCGTGGGCGGAGACGGCGGGGCTTGGTCTCTCGACCCTTCAAGCTCCTGTTGTTGTCGAACTCAAGACGACCGCCGTTCCAGTTCGCGTCCGGCAGTACCCGATGAGCCAGGAGGCCAAGAAAGGAATTACTGGATTTCTTGTCAAGTGTCAAAGTGCATGGAACACCCCGCTCCTCCCCGTCAAAAAACCAGGAACCGGCGACTATCGGCCGGTGCAAGATCTCCGGGCCGTGAACAGTCAAGTGGAGGTGATCCATCCAACTGTTCCAAATCCCTACAACTTGTTGTCAACGCTGTCCCCCGAGAGGGTGTGGTACACCGTCCTCGACTTGAAAGATGCCTTCTTCTGCCTCTCCCTCCACCCTGCATCTCAACCTCTCTTCGCCTTTGAGTGGAGCGACCCAGAAGCCGGCATTTCTGGTCAGCTGACATGGACAAGACTGCCGCAGGGCTTCAAGAATTCACCCACTATATTTGATGAAGCACTGCACCAGGACTTGTCATTGTTTCGATCGCAGCACCCACAAGTTACGCTGCTGCAGTATGTAGATGATATTCTCATCGCCGGGAAAACTGAAGAGGACTGCAAGCAGGCAACACATAATCTTCTGAAGGAGCTGGCCCGCCTCGGCTATCGTGCTTCCGCGAAGAAGGCGCAGCTCTGCCAGAGGGAGGTCACCTTCCTCGGGTACATGCTGCGCGGCGGCAAGAGATGGCTTACTGAAGCGAGGAAGAAGGTGGTGGCTCAGATCCCGGCGCCGACAACG
RT 16	116	ps-ssRNAv_RdRp-like superfamily	WP_074108845.1	51-358	group II intron reverse transcriptase/maturase	Pilosibacter fragilis	IKEQLRTRKYKPQPVRRVEIPKPDGGVRNLGVPTVTDRFVQQAIAQVLTPIYEEQFHAHSYGFRPNRCAQQAILTALDMMNDGNDWIVDIDLEKFFDTVNHDKLMTIIGRTIKDGDVISIVRKYLVSGIMIDDEYEDSIVGTPQGGNLSPLLANIMLNELDMEMETRGLNFVRYADDCIIMVRSEMSANRVMRNISRFIEEKLGLKVNMTKSKVDRPDGLKYLGFGFFFDTQAQQYKARPHAKSIAKLKTKMKWLTRRNWSVSNRYKIEKLNQLTRGWINYFGIGYMKWLCKDMDALIRRRLRMCIWV	yes	MIKEQLRTRKYKPQPVRRVEIPKPDGGVRNLGVPTVTDRFVQQAIAQVLTPIYEEQFHAHSYGFRPNRCAQQAILTALDMMNDGNDWIVDIDLEKFFDTVNHDKLMTIIGRTIKDGDVISIVRKYLVSGIMIDDEYEDSIVGTPQGGNLSPLLANIMLNELDMEMETRGLNFVRYADDCIIMVRSEMSANRVMRNISRFIEEKLGLKVNMTKSKVDRPDGLKYLGFGFFFDTQAQQYKARPHAKSIAKLKTKMKWLTRRNWSVSNRYKIEKLNQLTRGWINYFGIGYMKWLCKDMDALIRRRLRMCIWV	/	ATGATCAAGGAGCAACTTAGGACCAGGAAGTACAAGCCGCAGCCGGTGCGCCGTGTGGAGATCCCCAAGCCAGATGGCGGCGTCAGAAATCTTGGCGTGCCGACGGTAACTGATAGATTTGTGCAGCAAGCAATTGCTCAGGTGCTCACCCCCATCTACGAGGAGCAGTTCCATGCGCACTCCTACGGCTTTCGGCCAAATCGGTGCGCGCAGCAGGCCATCCTCACGGCGCTCGACATGATGAACGACGGAAATGACTGGATTGTTGACATCGACCTGGAGAAATTTTTCGACACCGTCAATCATGACAAGCTGATGACAATAATTGGTCGCACCATTAAAGATGGTGATGTTATATCCATCGTCCGCAAATATCTTGTCTCTGGAATAATGATTGATGATGAATATGAAGATAGTATAGTTGGGACTCCACAAGGTGGCAATTTGAGTCCTCTTCTTGCAAATATTATGCTGAATGAGCTGGACATGGAAATGGAGACAAGAGGGCTGAACTTCGTCCGGTACGCCGACGACTGTATCATCATGGTGCGGTCCGAGATGTCGGCCAACCGCGTCATGAGGAACATTTCACGATTCATTGAAGAAAAATTAGGGCTCAAGGTGAACATGACCAAGAGCAAGGTTGATAGGCCGGACGGCTTGAAGTATTTGGGATTTGGGTTCTTCTTTGATACTCAAGCTCAACAGTATAAGGCGCGGCCTCACGCCAAGTCAATAGCAAAACTAAAAACAAAGATGAAATGGCTCACAAGAAGGAACTGGTCTGTTAGCAATAGGTACAAGATTGAGAAGCTCAACCAGCTCACTCGTGGGTGGATCAACTACTTCGGCATCGGCTACATGAAGTGGCTGTGCAAGGATATGGATGCTTTAATCCGGAGACGCCTCAGGATGTGCATTTGGGTA
RT 17	127	ps-ssRNAv_RdRp-like superfamily	XP017174599.2	1-494	agouti-signaling protein isoform X1	Mus musculus	MVPMGALQPGIPSPVAIPRGYVKLMIDLKDCFFSIPLHPKDCKRFAFTLPMINCIGPSPRFQWKVLPQGMANSPTLCQRYVAQVIDPFRMSYPDLYVVHYMDDILVAGPDQDQLYSASQELINALQNQGLQVSPEKVQIHPPHLFLGFELFPNKILSQKVQVRQDSLQTLNDFQRLLGDINWLRPYLKLTTGELKPLFDILRGDPDPSSPRMLTQEARRSLAKVEQAISEQNIGYFSPELPLQLLVFPTPFSPTGLLWQLKPLFWVHMSASSSKVLPTYPQLVANVLRLGREAALKLFGRDPDVIVLPYDASQVQWLLKNNDDWAVNCISFQGVIDNHYPADKLVQFLHPVVFPKKSSPIPGAMLVFTDGSSSGMAAFYINGKVSRFMTDFSSAQLVKLAAIVKVFEQLPKTSFNLYTDSAYVAASVPLLETVPYIRPSTNASPMFAKLQSLILARNFPFFIGHIRAHSGLPGPLSEGNDIVDQATQVIA---	yes	MVPMGALQPGIPSPVAIPRGYVKLMIDLKDCFFSIPLHPKDCKRFAFTLPMINCIGPSPRFQWKVLPQGMANSPTLCQRYVAQVIDPFRMSYPDLYVVHYMDDILVAGPDQDQLYSASQELINALQNQGLQVSPEKVQIHPPHLFLGFELFPNKILSQKVQVRQDSLQTLNDFQRLLGDINWLRPYLKLTTGELKPLFDILRGDPDPSSPRMLTQEARRSLAKVEQAISEQNIGYFSPELPLQLLVFPTPFSPTGLLWQLKPLFWVHMSASSSKVLPTYPQLVANVLRLGREAALKLFGRDPDVIVLPYDASQVQWLLKNNDDWAVNCISFQGVIDNHYPADKLVQFLHPVVFPKKSSPIPGAMLVFTDGSSSGMAAFYINGKVSRFMTDFSSAQLVKLAAIVKVFEQLPKTSFNLYTDSAYVAASVPLLETVPYIRPSTNASPMFAKLQSLILARNFPFFIGHIRAHSGLPGPLSEGNDIVDQATQVIA	/	ATGGTGCCAATGGGTGCCTTACAGCCGGGCATTCCCAGCCCTGTTGCCATCCCGCGGGGCTACGTGAAGCTCATGATTGACCTGAAGGATTGCTTCTTCAGTATTCCTCTCCACCCAAAGGACTGTAAAAGGTTCGCTTTCACGCTACCGATGATCAACTGCATCGGCCCTTCTCCACGATTTCAGTGGAAGGTGCTGCCACAGGGTATGGCCAACTCGCCCACCCTATGCCAAAGATATGTAGCTCAAGTCATCGACCCCTTTAGGATGTCCTATCCTGACCTGTACGTGGTGCACTACATGGATGATATCTTGGTGGCCGGCCCTGACCAAGACCAGCTTTACTCTGCCTCACAGGAGCTGATCAATGCCCTGCAGAATCAGGGCCTCCAGGTCTCCCCGGAGAAGGTGCAAATTCACCCCCCACACCTCTTCCTTGGGTTTGAACTCTTCCCCAACAAGATACTGTCCCAGAAGGTTCAGGTGAGACAAGATTCACTGCAAACACTTAACGACTTCCAGAGGCTCCTCGGCGACATCAACTGGCTAAGACCATACCTTAAATTAACCACCGGCGAGCTGAAACCTCTGTTTGACATACTAAGAGGAGACCCTGATCCATCTTCACCTCGCATGCTCACACAGGAAGCGAGACGTAGCCTTGCCAAGGTGGAGCAAGCTATCTCCGAGCAGAACATTGGGTATTTCTCCCCTGAACTGCCTCTGCAGCTCCTCGTCTTCCCTACTCCGTTCTCTCCCACCGGCCTGCTCTGGCAACTGAAGCCGCTGTTCTGGGTTCACATGAGCGCCAGCTCGTCTAAAGTGTTACCTACATACCCACAGCTCGTGGCAAATGTTCTCCGCCTGGGTCGGGAGGCCGCCCTAAAGCTGTTTGGAAGGGACCCAGACGTAATCGTGTTGCCTTATGATGCATCCCAGGTTCAATGGCTGCTGAAGAATAATGATGACTGGGCGGTAAACTGTATCTCCTTCCAGGGTGTGATAGACAACCATTACCCTGCCGATAAGCTGGTGCAGTTCCTCCATCCGGTGGTGTTCCCCAAGAAGAGCAGCCCAATCCCTGGCGCCATGTTGGTCTTCACCGACGGCTCCAGCAGCGGAATGGCGGCGTTCTACATCAATGGGAAGGTTTCACGGTTCATGACGGATTTCAGCTCGGCGCAGCTAGTGAAACTCGCCGCCATCGTAAAGGTGTTTGAACAACTCCCTAAAACATCTTTCAACCTTTACACCGATTCTGCATACGTCGCTGCCTCCGTGCCGTTGCTGGAGACCGTCCCTTACATCAGGCCTTCAACCAACGCCTCTCCGATGTTTGCTAAGCTGCAGTCTCTAATCCTGGCAAGAAACTTTCCTTTTTTTATTGGACACATCAGAGCACACTCAGGACTTCCTGGCCCCCTGTCTGAAGGCAATGACATCGTGGACCAGGCTACTCAGGTGATTGCC

RT 18	137	ps-ssRNAv_RdRp-like superfamily	MDX5339534.1	2-262	group II intron reverse transcriptase/maturase	Cyclobacteriaceae bacterium	EEEIDHWHKAKKWIPSLRDRKEITSMYEEKLLNRILDKDNLNQAFKQVKKNKGAAGVDGMTVEELGSYMAINKEEIISQIRQRKYEPNPVLRVEIPKPNGGVRLLGIPTVKDRLIQQAIAQVLTPIFDRKFSEYSYGFRPKRYAEMAILQTLEFLNEGHDWIVDIDLERFFDTVNHDRLMNLISKTVNDGDVISLIRKFLVSGVQIDEEYKETVIGTPQGGNLSPLLSNIMLNELDMELENRGLHFVRYADDCIIMVKSEM	yes	MEEEIDHWHKAKKWIPSLRDRKEITSMYEEKLLNRILDKDNLNQAFKQVKKNKGAAGVDGMTVEELGSYMAINKEEIISQIRQRKYEPNPVLRVEIPKPNGGVRLLGIPTVKDRLIQQAIAQVLTPIFDRKFSEYSYGFRPKRYAEMAILQTLEFLNEGHDWIVDIDLERFFDTVNHDRLMNLISKTVNDGDVISLIRKFLVSGVQIDEEYKETVIGTPQGGNLSPLLSNIMLNELDMELENRGLHFVRYADDCIIMVKSEM	*	ATGGAGGAGGAAATAGATCACTGGCACAAGGCAAAGAAATGGATTCCTTCACTTAGAGATCGCAAGGAGATCACCTCCATGTACGAGGAGAAGCTGCTCAATAGGATCCTTGACAAGGATAATCTCAACCAGGCGTTCAAGCAGGTGAAGAAGAACAAGGGCGCCGCTGGCGTAGATGGCATGACAGTTGAAGAGCTCGGGAGCTACATGGCGATCAACAAAGAAGAAATTATATCACAAATACGTCAAAGGAAATATGAGCCCAACCCGGTCCTCAGAGTGGAGATACCAAAACCAAATGGAGGAGTAAGGTTATTGGGAATTCCAACTGTCAAGGACCGCCTCATCCAGCAGGCTATTGCACAAGTTCTAACACCTATTTTTGATCGTAAATTTTCAGAATATTCTTATGGATTTCGCCCCAAGCGGTACGCGGAGATGGCCATCCTGCAAACACTGGAGTTCTTGAATGAAGGGCATGACTGGATCGTCGACATCGACCTCGAGAGGTTCTTCGACACCGTCAACCATGATCGGCTAATGAACCTTATCAGCAAGACGGTGAATGATGGTGATGTTATAAGTTTAATTAGGAAGTTCCTCGTCTCCGGTGTGCAGATCGATGAGGAGTACAAGGAAACTGTGATCGGCACGCCGCAGGGCGGCAACCTCTCGCCGCTGCTTAGCAATATTATGCTGAATGAGCTGGATATGGAACTTGAGAATAGAGGGTTGCATTTTGTTCGCTACGCCGACGACTGCATCATTATGGTGAAATCTGAAATG
RT 19	138	ps-ssRNAv_RdRp-like superfamily	AAR11472.1	1-223	reverse transcriptase, partial	Colinus virginianus	TPVWIDQWPLPEIKLVALIHLVERELQLGHLEPSLSCWNTPVFVIRKASGSYRLLHDLRAVNAKLIPFGAVQQGAPVLSALPRGWPLMVLDLKDCFFSIPLAGQDREAFAFTLPSVNNQAPARRFQWKVLPQGMACSPTICQLVVGRVLEPLRRRHPSLCMLHYMDDLLIAASDCEKLEMVGKEIISVLEEAGFTISPDKIQREPGVEYLGYRLGSTYVIPAG	yes	MTPVWIDQWPLPEIKLVALIHLVERELQLGHLEPSLSCWNTPVFVIRKASGSYRLLHDLRAVNAKLIPFGAVQQGAPVLSALPRGWPLMVLDLKDCFFSIPLAGQDREAFAFTLPSVNNQAPARRFQWKVLPQGMACSPTICQLVVGRVLEPLRRRHPSLCMLHYMDDLLIAASDCEKLEMVGKEIISVLEEAGFTISPDKIQREPGVEYLGYRLGSTYVIPAG	/	ATGACGCCGGTGTGGATCGACCAGTGGCCGCTGCCGGAAATCAAGCTCGTCGCGCTGATCCACCTCGTGGAGCGGGAACTGCAGCTTGGGCACCTGGAACCAAGTCTCAGCTGCTGGAACACCCCTGTATTTGTTATAAGGAAAGCAAGTGGTAGCTACCGCCTGTTGCATGATCTCCGCGCCGTCAATGCAAAACTTATTCCTTTTGGTGCTGTGCAGCAGGGCGCGCCGGTTCTCTCAGCTTTGCCTCGCGGCTGGCCACTAATGGTGCTCGACCTCAAGGACTGCTTCTTCTCCATTCCCCTCGCCGGGCAAGATAGAGAAGCATTCGCCTTCACTTTGCCCTCGGTGAACAATCAAGCTCCAGCGCGGAGATTTCAATGGAAGGTTCTTCCACAAGGCATGGCGTGTTCCCCCACCATATGCCAGCTGGTGGTGGGCCGTGTCCTTGAACCTTTAAGAAGAAGGCATCCATCCCTCTGCATGCTGCACTACATGGATGATCTTCTCATCGCCGCCTCAGATTGTGAGAAGCTGGAGATGGTTGGAAAGGAAATTATTTCTGTTTTGGAGGAGGCCGGCTTCACAATATCGCCGGACAAGATCCAGAGGGAGCCCGGCGTCGAGTACCTCGGATATCGTTTAGGATCAACATATGTCATCCCGGCGGGG
RT 20	157	retron_St85_RT	HBQ4806382.1	13-406	RNA-directed DNA polymerase	Klebsiella pneumoniae	MDKFKPYSKSKAPISTLHKLAQTLSISIDELNEIAALSEDEKYSRKELPKADGSKRVVYSLHPKMRLLQSRINKRIFKELVIFPPFLFGSVPGKNDGVNSNIKRDYISCAKAHCGARTVLKVDISNFFDNIHKDLVRDVFNDVLNIRGEALEYVTNLCCKGDFVVQGALTSSYIATLCLYEQEGCVYQRALRKNLVYTRLVDDITVSSKINGYDFSQILSHIENMLSNYDLPVNKRKTKVFHCSSEPLKIHGLRVDYNSPRLPGDEVKRIRASLHNLKKLAVKNNTKTSMAYRKEFNRCMGRINKLGRVGHDKYDLFKEQLLAIKPLPSLRDIKIVESAVTSLESSCLIGNGNKHWYKRKYNLTLYKLIILTRSKAFDGVVNAFKERLKKVRPA	yes	MDKFKPYSKSKAPISTLHKLAQTLSISIDELNEIAALSEDEKYSRKELPKADGSKRVVYSLHPKMRLLQSRINKRIFKELVIFPPFLFGSVPGKNDGVNSNIKRDYISCAKAHCGARTVLKVDISNFFDNIHKDLVRDVFNDVLNIRGEALEYVTNLCCKGDFVVQGALTSSYIATLCLYEQEGCVYQRALRKNLVYTRLVDDITVSSKINGYDFSQILSHIENMLSNYDLPVNKRKTKVFHCSSEPLKIHGLRVDYNSPRLPGDEVKRIRASLHNLKKLAVKNNTKTSMAYRKEFNRCMGRINKLGRVGHDKYDLFKEQLLAIKPLPSLRDIKIVESAVTSLESSCLIGNGNKHWYKRKYNLTLYKLIILTRSKAFDGVVNAFKERLKKVRPA	/	ATGATGGACAAGTTTAAGCCGTACAGCAAATCAAAAGCACCAATCAGTACATTACACAAGCTAGCGCAGACCCTATCAATAAGTATAGATGAACTGAATGAAATTGCGGCGCTTTCAGAAGATGAAAAGTACTCCAGAAAAGAACTGCCGAAGGCCGACGGCAGCAAGCGTGTCGTCTACTCTCTTCATCCAAAGATGAGATTGCTCCAATCAAGGATTAATAAACGAATATTTAAAGAGCTCGTCATCTTCCCGCCGTTCTTATTTGGTAGTGTGCCCGGGAAGAATGATGGGGTGAACAGCAATATAAAGAGAGATTATATTTCGTGTGCCAAGGCTCACTGCGGCGCTCGAACAGTATTGAAGGTTGATATCTCTAACTTCTTCGACAACATTCATAAGGATCTTGTCAGAGATGTGTTCAATGACGTGCTCAACATCCGTGGCGAGGCGCTGGAGTATGTCACTAACCTGTGCTGCAAGGGCGACTTTGTGGTCCAGGGGGCACTTACTTCAAGCTACATTGCTACTCTGTGTCTTTATGAGCAGGAGGGCTGCGTCTACCAACGCGCCCTCCGGAAAAATTTGGTTTACACCCGCTTGGTGGACGACATAACTGTTTCTTCGAAAATAAATGGATATGACTTCTCCCAGATCCTTAGTCATATTGAGAACATGCTGTCAAATTATGATCTGCCTGTTAATAAACGCAAGACAAAAGTATTCCACTGTTCTTCCGAGCCACTCAAGATCCATGGACTCCGCGTCGACTACAACTCACCTAGGCTTCCTGGTGACGAGGTGAAAAGGATCAGGGCGTCGCTCCACAACCTGAAGAAACTGGCCGTCAAGAACAACACCAAGACCTCCATGGCATATCGCAAGGAGTTCAACCGGTGCATGGGGCGGATCAACAAGCTCGGCCGCGTTGGACATGATAAGTACGACCTCTTCAAGGAACAACTCCTCGCCATCAAGCCCCTCCCCTCGCTGAGGGATATTAAGATTGTGGAGTCTGCTGTGACGAGCTTGGAAAGTTCCTGCCTCATCGGTAATGGCAACAAACACTGGTACAAGAGGAAATATAATTTGACGTTATACAAACTCATCATCCTAACACGTTCCAAGGCATTTGATGGAGTTGTCAACGCCTTCAAGGAGAGGTTAAAGAAGGTGCGTCCAGCT
RT 21	158	retron_St85_RT	WP_134687572.1	1-220	group II intron reverse transcriptase/maturase, partial	Brevibacillus migulae	QEGTYRPMPVKRVGIPKPGGGIRLLGIPTVMDRFIQQALMQVMTPIFDPHFSPNSYGFRPNKRAHDAVKQAQSYIKEGFRWVVDMDLEKFFDRVNHDILMARVVRKVSDKRVLKLVRAYLQAGVIADGIELETIEGTPQGGPLSPLLANILLDDLDKELTARGLRFVRYADDCNIFVRSRRAGERVMESVIRFAEGKLRLKVNRDKSAVDRPWNRKFLGF	yes	MQEGTYRPMPVKRVGIPKPGGGIRLLGIPTVMDRFIQQALMQVMTPIFDPHFSPNSYGFRPNKRAHDAVKQAQSYIKEGFRWVVDMDLEKFFDRVNHDILMARVVRKVSDKRVLKLVRAYLQAGVIADGIELETIEGTPQGGPLSPLLANILLDDLDKELTARGLRFVRYADDCNIFVRSRRAGERVMESVIRFAEGKLRLKVNRDKSAVDRPWNRKFLGF	/	ATGCAGGAGGGAACATACCGCCCCATGCCGGTGAAGAGAGTGGGCATACCAAAACCTGGTGGCGGCATCCGCTTACTTGGAATACCTACAGTAATGGATCGGTTCATCCAGCAGGCGCTCATGCAGGTCATGACTCCAATATTTGATCCTCACTTCTCCCCAAATTCATATGGATTTAGACCCAACAAGAGGGCACATGACGCCGTCAAGCAAGCGCAAAGCTACATCAAGGAGGGGTTCCGTTGGGTGGTGGACATGGACCTGGAGAAGTTCTTCGACCGCGTCAATCATGATATTCTTATGGCGCGGGTCGTCAGGAAGGTTTCTGACAAGCGGGTACTGAAGCTCGTCCGTGCCTACCTACAAGCTGGTGTTATTGCTGATGGTATTGAGCTGGAAACCATCGAAGGGACGCCGCAGGGCGGGCCGCTGTCGCCGCTACTCGCCAACATCCTCCTTGATGATTTGGATAAAGAATTGACTGCAAGGGGGCTCCGCTTCGTGAGGTATGCCGACGACTGCAACATTTTTGTTCGAAGCCGCCGCGCCGGCGAGAGGGTGATGGAGAGTGTCATCAGATTTGCTGAAGGCAAGCTGCGCCTCAAGGTGAATCGGGACAAATCAGCGGTTGATAGGCCATGGAACAGAAAATTCCTCGGCTTC
RT 22	159	retron_St85_RT	NBH15871.1	1-269	group II intron reverse transcriptase/maturase	Lachnospiraceae bacterium	EKLRNRKYKPQPVRRVEIPKPDGGIGNLGVPTVTVRFIQQAVAQVLIPIYEEQFHEHSYGFRPNRCAQQAIMTALDMINEGNTWIVDIGLEKFFDTVNHDKLVTVIGRTIKDGDVISIIRKFLVSGSMVDDEYKESVIGTPQGGNLSPLLANIMLNELDKEMEKRGLNFVRYADDCIIMVGSEMSAKRVMRNLTKFIEEKLGLKVNMKKSKVDRPGGLKYSGFDIYYDSHAHGFKAKPHTKSVEKFKARMKQLTCRSWGVSNSYKIEKL	yes	MEKLRNRKYKPQPVRRVEIPKPDGGIGNLGVPTVTVRFIQQAVAQVLIPIYEEQFHEHSYGFRPNRCAQQAIMTALDMINEGNTWIVDIGLEKFFDTVNHDKLVTVIGRTIKDGDVISIIRKFLVSGSMVDDEYKESVIGTPQGGNLSPLLANIMLNELDKEMEKRGLNFVRYADDCIIMVGSEMSAKRVMRNLTKFIEEKLGLKVNMKKSKVDRPGGLKYSGFDIYYDSHAHGFKAKPHTKSVEKFKARMKQLTCRSWGVSNSYKIEKL	/	ATGGAAAAGCTGCGCAACAGGAAGTACAAGCCTCAGCCGGTACGGCGGGTGGAGATACCCAAGCCCGACGGCGGCATCGGCAACTTGGGCGTGCCGACTGTTACTGTCCGCTTCATCCAACAAGCTGTTGCTCAAGTGCTGATACCAATATATGAGGAGCAGTTCCATGAGCACAGCTACGGCTTCCGTCCAAATAGGTGCGCGCAGCAGGCCATTATGACTGCTTTGGACATGATCAATGAAGGAAATACATGGATCGTGGATATTGGGCTGGAGAAGTTCTTCGACACGGTGAATCATGATAAGCTCGTCACCGTCATTGGTCGGACCATCAAGGATGGTGATGTCATCTCCATCATTAGAAAATTTCTTGTTTCTGGATCCATGGTTGATGATGAGTACAAGGAGAGCGTAATTGGAACGCCGCAGGGCGGGAACCTTTCTCCTCTCCTCGCCAACATCATGCTAAATGAGCTCGACAAGGAAATGGAGAAGAGAGGTCTAAACTTCGTCAGATATGCCGACGACTGCATCATAATGGTGGGGTCGGAGATGTCAGCAAAGCGGGTGATGAGGAATCTCACCAAGTTTATTGAAGAAAAACTTGGCCTCAAGGTGAACATGAAGAAGTCAAAAGTTGACCGCCCTGGTGGGCTCAAGTATTCCGGTTTTGATATCTACTACGACAGCCACGCGCACGGGTTCAAGGCAAAACCACATACAAAAAGTGTAGAAAAATTTAAGGCGAGGATGAAACAACTGACATGTCGTTCATGGGGCGTCTCCAACAGCTATAAAATTGAGAAGTTA
RT 23	180	retron_St85_RT	WP_132703021.1	5-307	retron St85 family RNA-directed DNA polymerase	Reinekea marinisedimentorum	ERLARDLDISEQDVRGFALTAPNRYKIYRIPKSGSRVIAHPSRMLKAYQRLIISYVTELLPVHSCAYAYRQGVGIKDNARVHAKSSYLLKMDLQNFFNSINPSLFFDVVEKVGLEVADRDKWLLTKLLFWSPNKSSSGRLILSVGAPSSPLVSNFIMYPFDIRMSEYCFANGLKYTRYADDITLSTNKRGCLFDLPDYIKGVLANEYQGKLSVNESKTVFSSKAHNRHVTGITITNKGSLSIGRQRKRYISSLIYQFSSDQLDPDDTLHLKGLLSFAFDVEPDLRRRFEKKYTSKVIHQIL--	yes	MERLARDLDISEQDVRGFALTAPNRYKIYRIPKSGSRVIAHPSRMLKAYQRLIISYVTELLPVHSCAYAYRQGVGIKDNARVHAKSSYLLKMDLQNFFNSINPSLFFDVVEKVGLEVADRDKWLLTKLLFWSPNKSSSGRLILSVGAPSSPLVSNFIMYPFDIRMSEYCFANGLKYTRYADDITLSTNKRGCLFDLPDYIKGVLANEYQGKLSVNESKTVFSSKAHNRHVTGITITNKGSLSIGRQRKRYISSLIYQFSSDQLDPDDTLHLKGLLSFAFDVEPDLRRRFEKKYTSKVIHQIL	/	ATGGAGAGACTGGCAAGGGACTTGGACATTTCAGAGCAAGATGTGAGGGGATTTGCCCTCACCGCCCCCAACAGATACAAGATTTACCGTATCCCAAAAAGCGGTAGCAGAGTAATCGCACATCCGAGTAGAATGCTCAAGGCTTACCAGCGCCTCATCATCAGCTACGTCACGGAGTTACTGCCCGTTCACTCCTGCGCCTACGCATACAGGCAGGGCGTGGGCATCAAAGACAATGCTAGGGTGCATGCCAAGTCATCTTACCTCCTGAAGATGGATTTACAGAATTTCTTCAACTCTATAAACCCTTCCCTATTCTTTGACGTGGTTGAAAAGGTTGGGCTGGAGGTGGCTGACCGCGACAAGTGGCTCTTGACAAAGCTCCTGTTCTGGAGCCCTAATAAAAGCTCTAGTGGCCGGCTGATCTTGAGTGTAGGTGCGCCGTCCTCCCCTCTTGTGAGCAACTTTATCATGTATCCTTTTGACATCAGGATGTCAGAGTACTGCTTCGCCAATGGCCTCAAATACACAAGATATGCCGACGACATCACCCTGTCAACCAACAAGCGCGGCTGTCTCTTCGATTTGCCTGATTATATCAAAGGGGTGCTCGCCAACGAATATCAAGGGAAGCTGTCGGTGAATGAAAGCAAAACAGTCTTCTCCAGCAAGGCGCACAACCGTCACGTCACCGGCATCACTATTACTAACAAAGGATCACTGAGCATAGGCCGGCAGCGCAAGAGATACATTAGCTCCCTGATATACCAGTTTTCTTCTGATCAGTTAGACCCAGATGATACCCTCCACCTGAAGGGCCTGCTGTCCTTCGCGTTCGACGTGGAGCCCGACCTGCGCCGGAGATTTGAGAAGAAGTACACCTCGAAAGTGATCCACCAAATCCTT
RT 24	189	retron_St85_RT	WP_065678249.1	20-310	MULTISPECIES: retron St85 family RNA-directed DNA polymerase	Vibrio	LEATPSKHYKVYKIPKKRLGFIAQPTPAVKIIQKEIVDYLTPKVSIHTSATAYVPGRSVKDNALVHVKSNFLLKVDLENFFNNITPKMLFKSLKKQGIVVSGVDLIVLQQFLFWNISKKRSGKLALSVGAPSSPFVSNVVMLSFDERMTKLCRKSGINYSRYADDLTFSTTQKGLLFEHLFVVRKALKKEFGARLVLNESKTVFSSKAHNRHVTGITLTNNNKISLGRERKRYISALVHKFKLGLLDVEDTLHLQGLISYANHIEASFVQKMSIKYGDNVLTEIIKYSG--	yes	MLEATPSKHYKVYKIPKKRLGFIAQPTPAVKIIQKEIVDYLTPKVSIHTSATAYVPGRSVKDNALVHVKSNFLLKVDLENFFNNITPKMLFKSLKKQGIVVSGVDLIVLQQFLFWNISKKRSGKLALSVGAPSSPFVSNVVMLSFDERMTKLCRKSGINYSRYADDLTFSTTQKGLLFEHLFVVRKALKKEFGARLVLNESKTVFSSKAHNRHVTGITLTNNNKISLGRERKRYISALVHKFKLGLLDVEDTLHLQGLISYANHIEASFVQKMSIKYGDNVLTEIIKYSG	/	ATGCTGGAGGCGACCCCCTCCAAGCATTACAAAGTCTACAAGATTCCTAAGAAAAGACTCGGCTTCATCGCCCAGCCTACACCAGCCGTAAAGATTATTCAAAAGGAGATCGTCGACTACCTCACCCCCAAGGTGAGCATTCACACCAGCGCCACCGCCTACGTGCCTGGAAGATCTGTGAAGGATAATGCATTGGTGCATGTAAAATCGAACTTCCTTCTGAAGGTCGATCTTGAAAATTTCTTCAACAATATAACACCAAAAATGCTCTTCAAGAGCCTGAAGAAACAGGGTATCGTGGTGAGCGGGGTGGACCTCATAGTGCTGCAGCAGTTCCTCTTCTGGAACATCTCAAAGAAGCGGTCTGGGAAGCTCGCCCTCAGTGTCGGCGCACCTTCTTCGCCGTTCGTGAGCAATGTTGTCATGCTGAGCTTTGATGAAAGGATGACAAAGTTGTGCCGCAAGTCGGGTATCAACTACAGCAGATATGCTGACGACCTGACATTCTCCACCACCCAAAAGGGACTGCTGTTTGAGCACCTGTTTGTTGTGAGAAAAGCTCTGAAGAAGGAATTTGGCGCCCGACTGGTGCTGAATGAGTCAAAAACCGTGTTCTCTAGCAAGGCGCACAACCGCCACGTCACCGGCATTACCTTGACAAATAACAACAAAATCTCCCTGGGCCGGGAGAGGAAGAGGTACATCTCCGCGCTGGTCCATAAGTTTAAGCTGGGGCTCCTAGACGTGGAGGATACTCTTCACTTGCAGGGACTGATCAGCTACGCCAACCACATTGAGGCCAGCTTCGTCCAGAAGATGTCAATCAAATATGGTGACAACGTTCTGACAGAAATCATCAAGTACAGCGGC
RT 25	248	retron_St85_RT	WP102948601.1	2-307	retron St85 family RNA-directed DNA polymerase	Aeromonas caviae	GLIKQLAQNLCKSEAEVAHFLLNAPKMYKVYTIPKSGHIAQPSKELKEYQRKYLELQQLPIHEAAMAYREGIGIKQNAIAHKNNPYLLKLDLENFFNSISNLLFWEVWKSIQPLPSSKDQKALEQLLFWCPSKITGGRLVLSIGAPSSPLVSNFFMYQFDCTISEICRHKNIVYTRYADDLTFSTKIKGILFDMPNIVKDHLSLHFGDAININKKKTKFSSKAHNRHVTGITLNNDGKLSLGRERKRYIKHLVHQVKLGCLENEDKQHLVGLLAFVKHIEPMFLESLKNKYSVELITKIIEG--	yes	MGLIKQLAQNLCKSEAEVAHFLLNAPKMYKVYTIPKSGHIAQPSKELKEYQRKYLELQQLPIHEAAMAYREGIGIKQNAIAHKNNPYLLKLDLENFFNSISNLLFWEVWKSIQPLPSSKDQKALEQLLFWCPSKITGGRLVLSIGAPSSPLVSNFFMYQFDCTISEICRHKNIVYTRYADDLTFSTKIKGILFDMPNIVKDHLSLHFGDAININKKKTKFSSKAHNRHVTGITLNNDGKLSLGRERKRYIKHLVHQVKLGCLENEDKQHLVGLLAFVKHIEPMFLESLKNKYSVELITKIIEG	/	ATGGGCCTAATCAAACAACTCGCCCAGAACCTCTGCAAGAGCGAAGCTGAAGTCGCCCATTTCCTCCTCAACGCCCCGAAGATGTACAAGGTTTACACCATTCCAAAGTCCGGCCACATCGCGCAGCCGAGCAAGGAACTAAAGGAGTACCAGCGGAAGTATCTGGAGCTCCAGCAGCTACCTATCCATGAGGCCGCCATGGCGTACAGGGAGGGGATAGGGATAAAGCAAAATGCTATCGCTCACAAAAATAACCCTTACCTGCTGAAGCTCGACCTCGAGAACTTCTTTAACAGTATCTCCAACTTGCTGTTTTGGGAGGTGTGGAAGAGCATACAACCTCTCCCTTCTTCCAAGGATCAGAAGGCGCTGGAGCAGCTGCTGTTCTGGTGCCCTAGCAAAATTACTGGCGGCCGTCTGGTACTCAGCATTGGCGCCCCCTCTTCACCCCTTGTGAGCAATTTTTTCATGTATCAGTTTGATTGCACCATCTCTGAAATATGTCGGCACAAGAATATCGTGTACACAAGATATGCCGATGACCTCACCTTCTCTACTAAGATCAAGGGAATCCTCTTCGACATGCCAAACATCGTGAAAGACCACCTGTCGTTGCACTTCGGCGACGCCATTAACATTAATAAGAAGAAAACAAAGTTCTCCTCCAAAGCTCACAACCGCCACGTCACCGGTATCACGCTGAACAATGATGGAAAGCTTTCCCTGGGCCGGGAGAGAAAAAGATACATCAAGCACTTGGTGCACCAGGTGAAGCTGGGCTGTCTTGAAAATGAAGACAAACAGCATCTGGTAGGCTTACTGGCTTTCGTGAAGCATATCGAGCCGATGTTCCTGGAGAGCTTGAAGAATAAATACTCTGTGGAGTTGATTACCAAGATCATCGAGGGA

RT 26	275	retron_St85_RT	MBL4797689.1	2-309	retron St85 family RNA-directed DNA polymerase	Oleispira sp.	SFLEELASVLQKSEAEVEGFLEGAPKKYYKIPKSGFIAQPSKELKEYQRAFLKIQSLPIHNAAKAYRKNLSIKDNATVHKSSRYFLSMDLENFFNSISSDLFWRVYSRHFDSFSGQDKRVMESLLFWSPGKSNKQGLVLSVGAPSSPLLSNFFMYTFDLIMSNYCDETNILYTRYADDFTFSTNDKNILFSVPEVVKGFLEKCFGRGINVNKRKTVFSSKAHNRHVTGITINNNGDLSLGRERKRYIKHLIFQYSINKLEEGMLQHLQGLLAFSNHIEPMFIFTLNEKYTKELMLRISEERH---	yes	MSFLEELASVLQKSEAEVEGFLEGAPKKYYKIPKSGFIAQPSKELKEYQRAFLKIQSLPIHNAAKAYRKNLSIKDNATVHKSSRYFLSMDLENFFNSISSDLFWRVYSRHFDSFSGQDKRVMESLLFWSPGKSNKQGLVLSVGAPSSPLLSNFFMYTFDLIMSNYCDETNILYTRYADDFTFSTNDKNILFSVPEVVKGFLEKCFGRGINVNKRKTVFSSKAHNRHVTGITINNNGDLSLGRERKRYIKHLIFQYSINKLEEGMLQHLQGLLAFSNHIEPMFIFTLNEKYTKELMLRISEERH	/	ATGAGCTTCCTGGAGGAGCTCGCCTCCGTGCTGCAAAAATCAGAAGCTGAAGTGGAGGGATTCTTGGAGGGTGCCCCCAAGAAGTACTACAAGATACCTAAAAGTGGCTTCATCGCGCAGCCATCAAAGGAGCTGAAGGAGTACCAGCGGGCGTTCCTCAAGATTCAGTCTCTGCCCATCCATAACGCCGCCAAAGCTTACAGAAAGAACCTGAGCATCAAAGATAATGCCACCGTCCACAAGAGCAGCAGGTACTTCCTTTCCATGGATCTGGAAAATTTCTTCAATTCGATCTCCAGCGACCTCTTCTGGAGAGTCTACTCGCGCCATTTTGATTCATTCTCCGGCCAAGACAAGAGGGTGATGGAGTCCCTCCTCTTTTGGAGCCCTGGGAAGTCCAACAAGCAAGGCCTGGTGCTGAGCGTAGGCGCCCCTTCTTCTCCTCTGCTATCGAACTTCTTCATGTACACATTCGACCTCATCATGTCGAACTATTGTGATGAAACAAACATTCTGTACACTAGGTATGCCGACGACTTCACCTTCTCCACCAATGATAAGAATATTCTGTTCAGCGTGCCGGAGGTGGTGAAAGGTTTTCTGGAGAAGTGCTTTGGAAGAGGTATCAACGTTAATAAAAGAAAGACGGTGTTCAGTAGCAAGGCCCACAATCGCCACGTCACCGGCATCACGATCAACAACAATGGCGACCTGTCTTTGGGCCGGGAGAGAAAGAGATATATTAAGCATCTGATCTTCCAGTATAGCATTAATAAGCTTGAAGAAGGAATGCTGCAGCACCTTCAGGGGCTGCTGGCATTCAGCAACCACATTGAGCCAATGTTTATATTTACACTGAATGAAAAATACACCAAGGAATTGATGTTGAGAATCTCAGAGGAGCGGCAC
RT 27	291	RT_Bac_retron_II	WP180344603.1	1-391	reverse transcriptase family protein	Escherichia coli	MSSDRPIKTTASAIPSLKKLSQTLGFSIEELEEIRAIPLEKRYVKLEKPKIDGTMVYRPHFKLKRLQRRINSRIFRPLVIWPVYLYGSVPCDNDDESIKRDYITCASQHCAAKTILKIDIKNFFDNIHQDIVRDVFDKVLNIKDDALDYIVDVCCAGDFIVQGALTSSYIASLCLHDVEADIYARAKRKKLVYTRLVDDITVSSKIHDFDFSQIRKHIEDMLAKKDLPINVEKSGVFITSTKPLVVHGLRIDHSKPRLPSDEVKKIRASLHNLIANASKNNNKTSLAYRIEFNRCMGRINKLGRVGHEKHLVFMSKIKLIRPMPSYKDIVDCKGKLVKLEKLHAKGYNTSDIYFRRYQLVSHLLSLINRSSSFHEVVKDLRKRLNRIKP---	yes	MMSSDRPIKTTASAIPSLKKLSQTLGFSIEELEEIRAIPLEKRYVKLEKPKIDGTMVYRPHFKLKRLQRRINSRIFRPLVIWPVYLYGSVPCDNDDESIKRDYITCASQHCAAKTILKIDIKNFFDNIHQDIVRDVFDKVLNIKDDALDYIVDVCCAGDFIVQGALTSSYIASLCLHDVEADIYARAKRKKLVYTRLVDDITVSSKIHDFDFSQIRKHIEDMLAKKDLPINVEKSGVFITSTKPLVVHGLRIDHSKPRLPSDEVKKIRASLHNLIANASKNNNKTSLAYRIEFNRCMGRINKLGRVGHEKHLVFMSKIKLIRPMPSYKDIVDCKGKLVKLEKLHAKGYNTSDIYFRRYQLVSHLLSLINRSSSFHEVVKDLRKRLNRIKP	/	ATGAGCTCCGACCGCCCTATCAAAACAACGGCGAGCGCTATTCCTTCTCTGAAAAAGCTGTCGCAGACTCTAGGATTCTCTATCGAAGAACTGGAGGAGATCAGGGCTATCCCTTTGGAGAAGAGGTATGTGAAATTGGAAAAACCAAAAATCGACGGCACGATGGTGTATAGACCCCACTTCAAGCTGAAGAGACTGCAGCGTAGAATTAACTCCAGGATATTTCGTCCTCTGGTGATATGGCCGGTGTACCTCTACGGAAGCGTACCTTGTGATAATGATGATGAAAGCATAAAGCGCGACTACATCACCTGCGCCAGCCAGCACTGCGCCGCCAAGACAATCCTCAAGATTGATATCAAGAATTTCTTCGACAACATCCACCAAGATATTGTGAGAGACGTGTTTGACAAGGTGCTCAACATCAAGGATGATGCCTTGGATTACATTGTTGATGTCTGCTGTGCCGGCGACTTCATCGTACAGGGCGCCCTCACCAGTTCTTACATAGCCTCTCTGTGTCTGCATGATGTTGAGGCCGACATCTATGCCCGGGCCAAACGGAAAAAGCTGGTCTACACCAGGCTGGTGGACGACATAACCGTAAGCAGCAAAATCCATGATTTTGATTTCTCGCAGATAAGGAAGCACATCGAGGACATGCTTGCCAAGAAAGATTTACCCATCAATGTGGAGAAAAGCGGCGTCTTCATTACAAGTACAAAACCGCTGGTGGTACACGGGTTGAGAATAGACCACAGCAAGCCACGGCTCCCTTCTGATGAAGTTAAGAAGATCCGCGCTTCTCTGCACAACCTTATTGCAAATGCCTCCAAGAACAACAATAAGACCAGTCTGGCATACAGGATCGAGTTCAATAGGTGCATGGGCCGCATCAACAAGCTCGGCCGGGTCGGCCATGAGAAGCATCTGGTGTTTATGTCGAAGATCAAACTGATCCGGCCCATGCCGTCTTACAAGGACATTGTCGACTGCAAGGGAAAGCTGGTGAAGCTGGAAAAGCTCCACGCTAAGGGTTACAACACCTCCGACATCTACTTCCGTAGGTACCAGCTCGTATCGCACCTCCTGTCGCTGATTAACAGATCAAGTTCCTTCCACGAGGTGGTGAAAGATCTGAGAAAGCGGCTGAACAGAATCAAGCCT
RT 28	294	RT_Bac_retron_II	WP142486604.1	6-392	reverse transcriptase family protein	Leclercia adecarboxylata	PSNKSNSGISTLTSLSKALGFSIDELVEIRSIPLDRRYHKLEKPKSDGSMRIVYRPHHKLKRLQRRINNRIFRELVIWPNFLFGSVPNDNDGEDSLNRDYITCANQHCGAKTLLKVDIKNFFDNIHKELVSDIFSDFFYINAEANDYITDICCAGEFIVQGALTSSYIASLCLHDVETTVYRRAKRKGLVYTRLVDDITVSSKVYDYDFSQIKKHIEDMLAEKDLPINIVKSGVFQVSTKPLLVHGLRVDHNKPRLPSYEVRKIRASLNNLIGNSIKNNNKTSLAYRVEYNRCMGRINKLGRLGHEKHAVFMGRIKKVRPMPSFQDVRECKKKIKWLEIQYSKGFNKSDKYFRVFQITGHVISLINRSDAFSTIAKELRLNKVKP---	yes	MPSNKSNSGISTLTSLSKALGFSIDELVEIRSIPLDRRYHKLEKPKSDGSMRIVYRPHHKLKRLQRRINNRIFRELVIWPNFLFGSVPNDNDGEDSLNRDYITCANQHCGAKTLLKVDIKNFFDNIHKELVSDIFSDFFYINAEANDYITDICCAGEFIVQGALTSSYIASLCLHDVETTVYRRAKRKGLVYTRLVDDITVSSKVYDYDFSQIKKHIEDMLAEKDLPINIVKSGVFQVSTKPLLVHGLRVDHNKPRLPSYEVRKIRASLNNLIGNSIKNNNKTSLAYRVEYNRCMGRINKLGRLGHEKHAVFMGRIKKVRPMPSFQDVRECKKKIKWLEIQYSKGFNKSDKYFRVFQITGHVISLINRSDAFSTIAKELRLNKVKP	/	ATGCCTTCTAACAAATCTAACTCGGGCATCTCCACACTTACCAGCCTGTCCAAGGCTCTTGGTTTCAGCATTGATGAACTTGTGGAGATCAGATCCATCCCTCTTGACCGGCGCTACCACAAACTGGAGAAGCCCAAGTCAGACGGCAGTATGAGGATAGTGTACCGTCCCCACCACAAGCTGAAAAGGCTGCAGAGGAGAATCAACAATAGAATATTCCGAGAGCTGGTGATATGGCCGAATTTCCTCTTCGGCAGCGTTCCTAACGACAATGACGGAGAGGATTCCCTCAACCGTGACTACATAACTTGTGCCAACCAACACTGCGGCGCCAAGACGCTGCTCAAAGTGGACATTAAAAACTTCTTTGATAACATCCATAAGGAGCTGGTATCTGATATCTTCTCCGACTTCTTCTACATCAATGCTGAAGCTAATGATTACATAACAGATATTTGCTGCGCCGGCGAGTTCATTGTCCAGGGCGCGCTCACCTCCTCTTACATCGCCTCACTGTGTCTCCACGACGTTGAGACCACCGTCTATAGACGTGCAAAGCGCAAGGGGCTAGTGTATACCAGACTGGTTGACGACATCACGGTGAGCTCGAAGGTTTACGATTATGATTTTTCTCAGATCAAGAAGCATATAGAAGACATGCTGGCCGAGAAAGATCTGCCCATCAACATCGTGAAAAGCGGCGTCTTCCAGGTTTCGACGAAGCCACTGCTGGTGCACGGTCTGAGAGTCGACCACAATAAGCCTCGTTTGCCTTCTTATGAAGTGCGCAAGATCCGGGCCAGCCTCAACAACCTCATCGGAAACTCCATTAAGAATAACAACAAGACAAGCCTGGCGTACAGGGTGGAGTACAATCGCTGCATGGGAAGAATCAACAAGCTTGGCCGGCTGGGCCATGAAAAGCATGCGGTGTTCATGGGCCGCATCAAGAAGGTGAGACCGATGCCATCATTTCAAGATGTGAGGGAGTGCAAGAAGAAAATAAAATGGCTCGAGATCCAGTACTCGAAGGGATTCAATAAGAGCGACAAGTACTTCCGGGTGTTTCAGATCACCGGCCACGTCATCAGTCTGATTAACAGAAGCGACGCCTTTTCTACCATCGCCAAGGAACTGCGGCTGAACAAAGTGAAACCT
RT 29	339	RT_Bac_retron_II	WP_205077490.1	2-400	reverse transcriptase family protein	Marinobacter salexigens	PIDPRFPLFPEKTSKQAISSLESLAEALSVTVKDLEEVRSLESDKCYRLKEIPKNGGGLRKVYDPHPLIRRLQSRINKRIFLDLIQWPGYLFGSLPNINIDGQLVSRDYIACAQKHCKAKSLLKVDVNNFFDNIHRDHVFDIFRMFLKFSDEVSNCLADICCYNGFLVQGALTSSYIATLCFWQNEGYVVKRLSRKGLIYTRLVDDITVSSKRHDFNFEYAEHHIKMMLVDLDLPANKRKREILRDGIEPLQVHGLRVNYASPRLPADEVRRIRAAVKNVVRMSKINNYSLSYRAMHDRCMGRVNKLARVGHNKHGDFKRELVDVIPLPSVRDLEKAESSISFLEKLDPERLKLRKYTRRYHLAKYRVSIVAYCQEADDLSFRLQKLSVFVGDYEL	yes	MPIDPRFPLFPEKTSKQAISSLESLAEALSVTVKDLEEVRSLESDKCYRLKEIPKNGGGLRKVYDPHPLIRRLQSRINKRIFLDLIQWPGYLFGSLPNINIDGQLVSRDYIACAQKHCKAKSLLKVDVNNFFDNIHRDHVFDIFRMFLKFSDEVSNCLADICCYNGFLVQGALTSSYIATLCFWQNEGYVVKRLSRKGLIYTRLVDDITVSSKRHDFNFEYAEHHIKMMLVDLDLPANKRKREILRDGIEPLQVHGLRVNYASPRLPADEVRRIRAAVKNVVRMSKINNYRTSLSYRAMHDRCMGRVNKLARVGHNKHGDFKRELVDVIPLPSVRDLEKAESSISFLEKLDPERLKLRKYTRRYHLAKYRVSIVARTYCQEADDLSFRLQKLSVFVGDYEL	/	ATGCCTATTGATCCAAGATTCCCCCTATTCCCTGAAAAAACCAGCAAGCAAGCAATCAGTAGCCTTGAGAGCCTGGCTGAGGCGCTGAGCGTCACCGTGAAGGATCTCGAGGAGGTCAGATCCCTGGAATCTGACAAGTGTTACCGGCTGAAGGAGATCCCAAAGAATGGCGGCGGCCTCAGGAAGGTTTATGATCCTCATCCTCTGATCAGACGGCTACAGTCCAGAATCAATAAAAGAATTTTCCTCGACCTGATCCAGTGGCCGGGCTACCTCTTCGGGTCGTTGCCTAACATAAACATCGACGGACAGCTGGTGTCCAGAGACTACATAGCCTGCGCCCAAAAGCATTGCAAAGCAAAAAGCCTGTTAAAGGTCGATGTGAACAACTTCTTTGACAATATCCACCGCGACCACGTTTTCGACATCTTCAGGATGTTTTTAAAGTTCTCAGATGAAGTAAGCAACTGTCTTGCCGACATTTGCTGTTATAATGGCTTCCTGGTGCAGGGCGCCCTGACATCCAGCTACATTGCCACCCTCTGTTTCTGGCAAAATGAAGGTTACGTCGTTAAAAGACTAAGTAGAAAGGGACTTATTTACACCCGCCTGGTGGATGATATCACTGTGTCTAGTAAGCGCCACGACTTCAACTTTGAGTACGCAGAGCACCACATCAAGATGATGCTCGTCGACCTGGACCTCCCTGCTAACAAGAGAAAGAGAGAAATCTTACGCGACGGCATTGAACCTTTACAGGTGCACGGCCTGAGAGTTAACTATGCTTCCCCAGACTGCCCGCCGATGAAGTGAGAAGAATTCGGGCCGCCGTCAAGAACGTGGTAAGGATGTCAAAAATCAATAACTACAGGACCTCCCTGTCTTACCGAGCCATGCATGATCGCTGCATGGGACGGGTGAACAAGCTGGCCCGAGTTGGGCACAACAAGCACGGTGATTTTAAGCGGGAGCTGGTGGACGTTATCCCTCTACCGTCCGTGAGAGATCTCGAGAAGGCTGAAAGCTCCATCTCCTTCTTGGAGAAACTCGACCCGGAGAGGTTAAAGCTGCGAAAATACACAAGACGCTACCACCTCGCCAAATACCGTGTGTCTATCGTCGCCAGGACATACTGCCAGGAGGCTGACGACCTCTCATTCCGGCTGCAGAAGCTCAGCGTGTTTGTCGGCGACTATGAGCTG
RT 30	340	RT_Bac_retron_II	WP_227188504.1	11-285	group II intron reverse transcriptase/maturase	Extibacter muris	LAKENLNTAYLQVVRNKGAEGVDGMTYTELKEHLENNGEIIKDQLRARKYTPRPVRRVEIPKPDGGVRNLGVPTVTDRFVQQAIAQVLTPIYEKQFHDHSYGFRPDRCAQQAIITALNLMNEGNDWIVDIDLEKFFDTVNHDKLMTLIGRTIKEGDVISIIRKFLVSGIMVDDEYKEAVIGTPQGGNLSPLLANIMLNELDKEMEQRGLNFVRYADDCIIMVGSEMSAKRVMRNLTKFIEEKLGLKVNMTKSKVDRPSGLKYLGFGFYFDSKAHQ	yes	MLAKENLNTAYLQVVRNKGAEGVDGMTYTELKEHLENNGEIIKDQLRARKYTPRPVRRVEIPKPDGGVRNLGVPTVTDRFVQQAIAQVLTPIYEKQFHDHSYGFRPDRCAQQAIITALNLMNEGNDWIVDIDLEKFFDTVNHDKLMTLIGRTIKEGDVISIIRKFLVSGIMVDDEYKEAVIGTPQGGNLSPLLANIMLNELDKEMEQRGLNFVRYADDCIIMVGSEMSAKRVMRNLTKFIEEKLGLKVNMTKSKVDRPSGLKYLGFGFYFDSKAHQ	*	ATGCTGGCCAAGGAGAATCTGAACACTGCGTACTTGCAAGTTGTCCGGAACAAGGGCGCGGAGGGGGTGGATGGCATGACATATACTGAACTGAAAGAGCACCTGGAGAACAATGGTGAGATTATCAAGGACCAGCTGCGCGCGAGGAAGTACACCCCGCGGCCGGTGCGCCGGGTTGAAATACCAAAGCCCGACGGCGGCGTAAGAAATTTGGGTGTCCCAACGGTGACCGACAGATTTGTGCAACAAGCAATTGCACAAGTGCTCACTCCAATTTATGAGAAGCAGTTCCATGATCATTCGTACGGCTTCAGACCTGACCGATGTGCTCAGCAGGCCATCATCACCGCCTTAAATCTCATGAATGAAGGAAATGATTGGATCGTCGACATTGATCTTGAAAAGTTCTTTGATACCGTCAACCACGACAAGCTGATGACGCTCATCGGCCGTACAATAAAAGAAGGTGATGTTATAAGCATCATTCGCAAATTCCTCGTCTCCGGCATTATGGTTGATGATGAGTACAAGGAAGCTGTCATCGGGACGCCGCAGGGAGGCAACCTTTCACCCCTCCTCGCCAACATCATGCTCAATGAGCTTGATAAGGAGATGGAGCAGCGCGGCCTCAACTTCGTGAGGTATGCTGACGACTGCATCATAATGGTGGGTTCTGAAATGTCAGCAAAGAGGGTGATGAGGAACTTGACAAAGTTTATTGAGGAGAAACTGGGGCTCAAGGTGAACATGACCAAGAGCAAAGTAGACCGCCCTAGTGGATTGAAATATCTTGGATTTGGGTTCTACTTCGACTCCAAGGCGCACCAG
RT 31	349	RT_LTR	TMC19484.1	344-799	MAG: hypothetical protein E6J3415270	Chloroflexota bacterium	VPDWIRKDYGTVLREELPPQMPPTRSVDHQIPLKPDMPPPFKGIFRLSQLELRELKRQLDQLLKDGKIKPSTSPYGAPVLFVKKKDDKLRMCIDYRALNSQTIQNRYALPRIDELFDRLHGAKIFSKLDLTSGYYQIAIKPKDRHKTAF100RYGHYEFNVMPFGLTNAPATFQTLMNDIFRDLLDVCVIVYLDDILVYSKNKEEHEQHLRQVLQRLKNNQLYAKLSKCTFFTNSIEYLGHIVDGEGLRPNPRLVQALKDFPQPKTLKELQSFLGLANYYRKFIANFSHIALPLTDATRNNTQSNLRPIEWTQSMQTAFEKLKEALTSAPCLALPDPDGEFEVTTDASEDTKAVGAVLMQNGHPVAYESTKLNSHQLNYAVHDKEMCAIMHALERWRPFLLGRHFKVYTDHRSLVHFKTQSNLNQRQLRWQEKAADYDMEILYKPGKENVVADALSR--	yes	MVPDWIRKDYGTVLREELPPQMPPTRSVDHQIPLKPDMPPPFKGIFRLSQLELRELKRQLDQLLKDGKIKPSTSPYGAPVLFVKKKDDKLRMCIDYRALNSQTIQNRYALPRIDELFDRLHGAKIFSKLDLTSGYYQIAIKPKDRHKTAFRYGHYEFNVMPFGLTNAPATFQTLMNDIFRDLLDVCVIVYLDDILVYSKNKEEHEQHLRQVLQRLKNNQLYAKLSKCTFFTNSIEYLGHIVDGEGLRPNPRLVQALKDFPQPKTLKELQSFLGLANYYRKFIANFSHIALPLTDATRNNTQSNLRPIEWTQSMQTAFEKLKEALTSAPCLALPDPDGEFEVTTDASEDTKAVGAVLMQNGHPVAYESTKLNSHQLNYAVHDKEMCAIMHALERWRPFLLGRHFKVYTDHRSLVHFKTQSNLNQRQLRWQEKAADYDMEILYKPGKENVVADALSR	/	ATGGTGCCTGACTGGATTCGGAAGGATTACGGCACGGTGCTGAGGGAGGAGCTCCCTCCTCAGATGCCGCCCACCAGATCAGTGGATCATCAGATTCCTTTGAAGCCTGACATGCCTCCGCCGTTCAAGGGAATCTTTCGTCTTTCTCAGCTGGAGCTGCGGGAATTGAAAAGGCAGTTGGATCAGCTGCTTAAAGACGGCAAAATCAAGCCCTCCACCAGCCCATACGGCGCGCCGGTGCTCTTCGTCAAGAAGAAAGATGATAAGTTGAGGATGTGCATCGACTACCGCGCCCTAAATAGTCAGACAATCCAAAACAGGTACGCCCTGCCTAGGATCGATGAATTGTTTGATCGGCTGCACGGCGCCAAAATCTTCAGCAAACTCGATCTGACCTCAGGCTACTATCAGATCGCTATCAAGCCAAAAGACAGACACAAGACCGCCTTCCGCTATGGTCACTACGAGTTCAACGTCATGCCATTTGGTCTTACCAACGCCCCCGCCACCTTCCAGACACTGATGAATGATATCTTCAGAGACCTTCTAGACGTCTGCGTCATCGTCTATCTCGACGACATTCTGGTATACTCCAAGAACAAGGAAGAGCATGAGCAACACCTCCGGCAGGTGTTGCAGAGACTCAAGAATAACCAACTATACGCAAAGCTCTCAAAATGTACATTCTTCACAAACAGCATCGAGTACCTCGGCCATATTGTGGACGGGGAAGGCCTGCGGCCAAATCCACGGCTGGTTCAGGCGCTGAAGGACTTCCCGCAGCCCAAGACCCTCAAGGAGCTGCAGTCATTCCTGGGCCTGGCCAACTACTACAGAAAATTCATCGCGAACTTCTCTCACATTGCCCTGCCCCTGACAGACGCAACCCGCAATAACACACAGAGCAACCTGAGACCAATAGAGTGGACACAGTCGATGCAGACTGCTTTTGAAAAGCTAAAGGAAGCCTTAACGTCGGCCCCCTGCCTGGCCCTGCCGGACCCTGATGGAGAGTTTGAGGTGACAACCGACGCATCCGAGGACACCAAGGCCGTTGGCGCGGTGCTCATGCAAAATGGACACCCAGTGGCATACGAAAGCACAAAGTTAAACTCCCACCAGCTCAACTATGCTGTGCACGACAAGGAGATGTGTGCTATTATGCATGCCCTGGAGAGATGGCGCCCTTTCCTCCTTGGCCGTCACTTCAAGGTGTACACGGACCATAGAAGCCTCGTCCACTTTAAGACACAAAGCAACCTAAATCAGCGGCAACTGAGATGGCAGGAGAAGGCCGCCGATTATGATATGGAGATCCTCTACAAGCCTGGAAAGGAAAATGTTGTGGCTGACGCCCTGTCCCGA
RT 32	359	RT_LTR	XP_029410360.1	284-529	uncharacterized protein LOC115067138, partial	Nannospalax galili	IDQWLSKYPDTWAETGGMGLAIHQPPLIVELKATETPVAVKQYPMTAEARQGIRPHIKRLLEQGILIPCWAAWNTPLLPVKKPGSGDYRPVQDLREINKRVEDIHPTVPNPYNLLSTLSPSHTWYSVLDLKDAFFCLKLHHQSQPLFTFEWKDSDLGISGQLTWTRLPQGFKNSPTLFDEALHQDLTAFRTLHPHLILLQYVDDILLAAETREECLTGTEALLQELGLMGYRASAKKAQLCQQEVT	yes	MIDQWLSKYPDTWAETGGMGLAIHQPPLIVELKATETPVAVKQYPMTAEARQGIRPHIKRLLEQGILIPCWAAWNTPLLPVKKPGSGDYRPVQDLREINKRVEDIHPTVPNPYNLLSTLSPSHTWYSVLDLKDAFFCLKLHHQSQPLFTFEWKDSDLGISGQLTWTRLPQGFKNSPTLFDEALHQDLTAFRTLHPHLILLQYVDDILLAAETREECLTGTEALLQELGLMGYRASAKKAQLCQQEVT	**	ATGATAGATCAGTGGCTCTCCAAATACCCGGACACGTGGGCTGAAACTGGCGGCATGGGGCTGGCCATCCATCAGCCGCCGCTGATTGTGGAACTCAAAGCTACTGAAACACCTGTTGCGGTGAAGCAGTATCCAATGACAGCTGAGGCCCGCCAAGGTATTAGACCGCACATCAAGAGGTTGCTCGAGCAAGGAATACTTATTCCCTGCTGGGCGGCGTGGAACACACCATTGCTTCCTGTCAAGAAACCTGGAAGCGGCGACTACCGCCCCGTCCAAGATCTCCGCGAGATCAACAAACGTGTAGAAGATATTCATCCAACTGTTCCAAATCCTTATAATTTGCTATCGACTTTAAGTCCATCTCACACATGGTACAGTGTTCTGGACCTGAAAGATGCATTCTTCTGTCTTAAGCTGCACCACCAATCACAACCTCTGTTCACCTTTGAGTGGAAGGATTCTGATCTTGGAATCTCAGGGCAGCTGACCTGGACAAGACTGCCGCAGGGCTTCAAGAACAGCCCCACACTCTTTGATGAAGCACTCCACCAGGACTTAACGGCCTTCCGCACCCTCCATCCTCATCTCATTCTGCTGCAGTACGTCGACGACATCCTGCTCGCTGCCGAGACGAGGGAGGAGTGCCTCACCGGCACCGAGGCGCTCCTTCAAGAACTTGGTCTCATGGGCTATCGGGCATCCGCCAAGAAGGCGCAGCTCTGCCAGCAGGAGGTGACC
RT 33	360	RT_LTR	WP_185380350.1	1-286	group II intron reverse transcriptase/maturase	Listeria booriae	MHEPTLLSQIIHPANLNQAYKQVMKNKGAPGIDDMPITALKAHLALHKETLVHQLQRREYKPQPVKRVEIPKASGGVRLLGIPTVTDRFIQQAIAQVLTPIFDKQFHDNSYGFRPKRYAEMAILQALENMNEGYGWLVDIDLERFFDTVHHDRLMNIVARTVTDGDVISLVRKFLVSGVMVQDEYQETIIGTPQGGNLSPLLSNIMLHELDKELANRDLRFVRYADDCLIFVKSDMAARRVMRSVSKFIEEKLGLIVNVTKSKVRRPEDEETKFLGFGFYFDWNDR	yes	MMHEPTLLSQIIHPANLNQAYKQVMKNKGAPGIDDMPITALKAHLALHKETLVHQLQRREYKPQPVKRVEIPKASGGVRLLGIPTVTDRFIQQAIAQVLTPIFDKQFHDNSYGFRPKRYAEMAILQALENMNEGYGWLVDIDLERFFDTVHHDRLMNIVARTVTDGDVISLVRKFLVSGVMVQDEYQETIIGTPQGGNLSPLLSNIMLHELDKELANRDLRFVRYADDCLIFVKSDMAARRVMRSVSKFIEEKLGLIVNVTKSKVRRPEDEETKFLGFGFYFDWNDR	/	ATGCATGAGCCGACACTTCTTTCACAAATCATCCATCCTGCGAATTTGAATCAAGCATACAAACAAGTAATGAAGAACAAGGGCGCTCCTGGAATTGATGATATGCCAATTACGGCGCTGAAAGCACATTTGGCGCTCCACAAGGAGACTCTTGTGCACCAGCTGCAGCGACGGGAATACAAGCCACAACCAGTGAAGAGGGTGGAGATACCAAAGGCATCTGGAGGGGTGCGCCTCCTCGGCATACCCACAGTCACCGACCGCTTCATCCAGCAGGCCATCGCACAGGTGCTCACTCCTATATTTGACAAGCAGTTCCATGATAATAGCTATGGTTTCCGCCCCAAGAGATATGCTGAGATGGCGATACTTCAAGCTTTGGAGAACATGAATGAAGGATATGGCTGGCTGGTGGACATCGACCTGGAGAGGTTCTTCGACACCGTCCACCACGACAGGCTCATGAACATTGTAGCCAGAACAGTTACTGATGGCGACGTCATTTCACTCGTCAGGAAGTTTCTAGTTTCTGGTGTGATGGTGCAGGATGAGTACCAGGAGACAATCATTGGGACGCCGCAGGGCGGCAACCTGTCGCCGCTGCTGTCCAACATCATGCTGCATGAATTGGATAAAGAGCTCGCCAACAGAGATTTGAGATTTGTTCGTTACGCCGACGACTGCCTCATTTTCGTCAAGAGCGACATGGCCGCGCGCCGCGTCATGAGGAGTGTTTCTAAATTCATTGAGGAGAAGCTCGGATTAATAGTTAATGTTACCAAGTCCAAGGTGCGGCGGCCGGAAGATGAAGAAACAAAATTTCTTGGCTTCGGGTTCTACTTTGATTGGAATGATCGG

RT 34	362	RT_Rtv	CAF25164.1	2-606	pseudo	Avian endogenous retrovirus EAV-HP	PGATVCQLSAQYAIPLRWKPDTRPVWVDQWPLPKEKLDALQTLVARELRLGHIEPSLSRWNTPVFVIQKKSGAFRLLHDLRAVNSQLIPFGVVQQGAPVLSAVPEEWEVTAIDLKDCFFSIPLAEQDREAFAFTVPVSNNQRPTQRYQWRVLPQGMACSPTICQMVVGKILGPLHHTSEASECIILHYMDDLLLAAPTLARLQDLETCVISLLTKAGFTVSSEKIQRGSGVQFLGYKFDEGTVRPTGVNITPRILWDVQKLVGALQWIRGALGIPPRLMQPFYDQLKGSDPREPFTPDMSEAWDEIVTSCLHTALSQYNCKKELEVAVCKCDSGATGLLGQDLGAKPQPLWWNYSVQPVHAFTSWLEVLAKLICKCRLSAVRIFGQEPSVIYLPRSFRDTSPLPEPILLALVGSSATVTFTDSLSIFELARPMQLSLHCRVQASPVDGPTLFTDASSSTGQGAVVWGRGSCWKSVIFQDVTASVQILEVRAVALALRLWPTIPCNIVTDSAFAAKLLLSMGQEGISSTEAAMVLEDSLCTAPVAVMHVRSHSEVPGFFSEGNAAADRAAGTHVFTLKTARELHSTLHIGARALSCGIP	yes	MPGATVCQLSAQYAIPLRWKPDTRPVWVDQWPLPKEKLDALQTLVARELRLGHIEPSLSRWNTPVFVIQKKSGAFRLLHDLRAVNSQLIPFGVVQQGAPVLSAVPEEWEVTAIDLKDCFFSIPLAEQDREAFAFTVPVSNNQRPTQRYQWRVLPQGMACSPTICQMVVGKILGPLHHTSEASECIILHYMDDLLLAAPTLARLQDLETCVISLLTKAGFTVSSEKIQRGSGVQFLGYKFDEGTVRPTGVNITPRIRTLWDVQKLVGALQWIRGALGIPPRLMQPFYDQLKGSDPREPRTFTPDMSEAWDEIVTSCLHTALSQYNCKKELEVAVCKCDSGATGLLGQDLGAKPQPLWWNYSVQPVHAFTSWLEVLAKLICKCRLSAVRIFGQEPSVIYLPRSFRDTSPLPEPILLALVGSSATVTFTDSLSIFELARPMQLSLHCRVQASPVDGPTLFTDASSSTGQGAVVWGRGSCWKSVIFQDVTASVQILEVRAVALALRLWPTIPCNIVTDSAFAAKLLLSMGQEGISSTEAAMVLEDSLCTRTAPVAVMHVRSHSEVPGFFSEGNAAADRAAGTHVFTLKTARELHSTLHIGARALSRTCGIP	/	ATGCCTGGCGCCACCGTATGTCAGCTGAGTGCCCAGTACGCGATACCTCTTCGGTGGAAGCCGGACACAAGACCTGTATGGGTGGATCAATGGCCACTCCCAAAGGAGAAGCTCGACGCGCTGCAGACGCTTGTTGCTAGAGAACTGCGGCTGGGCCATATCGAACCATCCCTCAGCAGATGGAACACTCCTGTATTCGTCATCCAAAAGAAATCTGGAGCTTTTCGGCTGCTGCATGATTTAAGAGCTGTCAATTCACAGCTGATACCTTTCGGGGTGGTGCAGCAAGGAGCCCCAGTACTTTCCGCCGTCCCCGAGGAGTGGGAGGTAACCGCTATCGATCTGAAGGATTGCTTCTTCTCCATCCCCCTCGCGGAGCAGGATCGGGAGGCGTTCGCGTTCACCGTCCCAGTGAGCAACAACCAGAGACCGACCCAGCGATACCAGTGGCGTGTCCTGCCTCAGGGCATGGCGTGTTCCCCTACCATCTGCCAGATGGTTGTGGGCAAGATCCTTGGACCTCTCCACCACACTTCAGAGGCCAGCGAATGTATAATCCTTCACTACATGGATGATTTACTGCTGGCCGCGCCTACACTCGCCCGTCTGCAAGACCTGGAGACCTGCGTGATCTCCCTGCTGACGAAGGCAGGATTTACAGTGAGCTCCGAGAAAATTCAGCGCGGCAGCGGCGTCCAATTCCTGGGCTACAAGTTTGACGAAGGAACCGTCCGTCCCACCGGAGTGAATATCACTCCTCGCATCAGGACCCTCTGGGATGTGCAGAAGCTGGTGGGGGCCCTCCAGTGGATTCGAGGGGCGCTGGGCATCCCTCCAAGACTCATGCAACCCTTCTATGATCAGTTGAAGGGTTCCGACCCCAGAGAACCCCGGACGTTCACCCCCGACATGTCAGAGGCGTGGGACGAGATTGTCACGAGCTGCCTCCACACGGCCCTGTCGCAATACAACTGCAAGAAGGAACTTGAAGTAGCTGTGTGCAAATGTGATTCTGGCGCTACCGGTTTGCTGGGCCAAGACCTGGGCGCCAAGCCACAGCCGCTGTGGTGGAATTATTCTGTCCAGCCAGTCCACGCGTTCACCAGCTGGCTAGAAGTGTTAGCCAAGCTGATCTGTAAGTGCCGGCTCTCCGCCGTGAGGATTTTCGGCCAGGAGCCATCTGTGATATACTTGCCCAGAAGTTTCAGAGACACGTCTCCTCTTCCGGAGCCTATTCTGCTCGCCTTAGTTGGGAGCTCGGCTACCGTGACATTTACGGACTCCCTGTCGATATTTGAGCTGGCGCGGCCAATGCAGCTCAGTCTTCACTGCCGGGTGCAGGCCAGCCCCGTCGATGGCCCTACACTATTCACAGATGCGAGCAGCTCTACGGGCCAGGGCGCCGTAGTTTGGGGCAGAGGTTCCTGCTGGAAATCCGTTATATTCCAGGATGTTACTGCCTCGGTGCAGATCCTGGAGGTGCGCGCAGTGGCATTGGCCCTGCGCCTCTGGCCGACAATCCCCTGCAACATTGTGACCGACAGCGCATTTGCCGCTAAATTGCTGCTGTCGATGGGACAGGAAGGTATCTCATCCACAGAGGCCGCCATGGTCCTCGAGGATTCGCTCTGCACAAGAACCGCTCCGGTGGCCGTGATGCATGTGCGGTCTCATTCGGAGGTGCCTGGATTTTTTTCCGAAGGCAACGCGGCCGCGGACAGAGCGGCCGGCACCCACGTCTTCACCCTTAAGACGGCCAGGGAGTTGCACAGCACCCTGCACATCGGAGCCCGGGCTCTGAGCAGAACTTGCGGCATCCCC
RT 35	363	RT_Rtv	QDK64674.1	200-818	pro-pol, partial	Black Syrian hamster retrovirus	PIPSLPFSLWGRDIMEYMSVRLITDARKFFLGAIMSSFYAKKISWKNDEPVWLNQWPLTTEKIQAIEEIIQEQLQAGHLEESFSPWNTPIFVIRKKSGKWRLFQDLRAVNATMYDMGALQPGLPSPVAVPQGWKVIFIDLQDCFFTIQLHPDDCQRFAFSVPSVNYKEPFKRYQWRVLPQGMKNSPTLCQYFVAKAIEQLRKDHSDAYLVHYMDDILIAHPDIQELDKLVVKLINNLKDHGLVVAPEKVQQKWPINYLGHTMQEDYVSTQKIEIRRDKMLNDFQKVLGNINWIRPYLKITTGDLRPLFMTLNGDPDPKSARILTSKACEALQLVEEKLTEAKVIRIDYNKPLSLLIIEYTPTGCLWQDGVLEWIHLPHTNSRIISTYDILCAALIVKARNRSKEIFGRDMQEIVVPYTRQQFEHLLQESYDWGLALIGYLGIIKFHLPNIQSLIHKPIIFPSNWSTEPIEKAKVIFTDGSSKGNSVMIVQGEPPIIKYEKGLSAQKAEIIAVILAFEKVKEPFNLYTDSRYVAGLFPAIETAMISIEINDLLSKLKELAQKRKFKYHVAHIRGHTKLPEPLAQGNAIADSYTQVFIALENAMASHKLHHQNA	yes	MPIPSLPFSLWGRDIMEYMSVRLITDARKFFLGAIMSSFYAKKISWKNDEPVWLNQWPLTTEKIQAIEEIIQEQLQAGHLEESFSPWNTPIFVIRKKSGKWRLFQDLRAVNATMYDMGALQPGLPSPVAVPQGWKVIFIDLQDCFFTIQLHPDDCQRFAFSVPSVNYKEPFKRYQWRVLPQGMKNSPTLCQYFVAKAIEQLRKDHSDAYLVHYMDDILIAHPDIQELDKLVVKLINNLKDHGLVVAPEKVQQKWPINYLGHTMQEDYVSTQKIEIRRDKMRTLNDFQKVLGNINWIRPYLKITTGDLRPLFMTLNGDPDPKSARILTSKACEALQLVEEKLTEAKVIRIDYNKPLSLLIIRTEYTPTGCLWQDGVLEWIHLPHTNSRIISTYDILCAALIVKARNRSKEIFGRDMQEIVVPYTRQQFEHLLQESYDWGLALIGYLGIIKFHLPNIQSLIHKRTPIIFPSNWSTEPIEKAKVIFTDGSSKGNSVMIVQGEPPIIKYEKGLSAQKAEIIAVILAFEKVKEPFNLYTDSRYVAGLFPAIETAMISIRTEINDLLSKLKELAQKRKFKYHVAHIRGHTKLPEPLAQGNAIADSYTQVFIALENAMASHKLHHQNA	/	ATGCCTATCCCTTCCCTGCCCTTTTCCCTGTGGGGCAGAGACATTATGGAGTACATGAGCGTGCGGCTGATCACGGACGCCCGGAAATTCTTTCTGGGCGCCATCATGAGCAGCTTCTACGCGAAGAAGATCTCGTGGAAGAATGATGAGCCTGTTTGGCTGAACCAGTGGCCCCTGACGACCGAGAAGATCCAAGCTATCGAAGAAATCATTCAGGAACAGCTGCAGGCCGGCCATCTTGAAGAATCGTTCTCCCCTTGGAACACTCCTATATTCGTCATCAGAAAGAAGAGCGGGAAGTGGAGGCTCTTCCAGGATCTTCGCGCGGTGAATGCCACCATGTACGACATGGGTGCGCTGCAGCCCGGCTTGCCTTCACCGGTCGCCGTGCCGCAGGGATGGAAAGTGATCTTTATCGACCTGCAGGATTGTTTCTTCACCATCCAGCTGCACCCCGATGACTGCCAGCGCTTCGCCTTCTCCGTTCCTAGCGTGAATTACAAGGAGCCTTTCAAGAGATACCAATGGAGGGTGCTGCCTCAAGGAATGAAGAACAGCCCAACCCTGTGCCAATATTTCGTCGCCAAGGCCATCGAGCAGCTAAGAAAGGATCATTCCGACGCCTACCTGGTCCATTACATGGATGATATACTCATCGCTCACCCTGACATTCAGGAGTTGGATAAGCTGGTGGTGAAGCTGATCAATAATCTGAAGGACCATGGCCTAGTAGTGGCCCCGGAAAAGGTACAGCAGAAATGGCCAATCAATTACCTGGGCCATACAATGCAAGAAGACTACGTGAGCACACAAAAGATTGAAATAAGACGGGATAAGATGAGGACACTGAACGATTTTCAGAAGGTCCTGGGCAACATCAATTGGATCCGACCATACTTGAAGATTACCACAGGAGACCTCCGTCCTCTGTTCATGACATTGAACGGGGACCCCGACCCAAAGTCCGCCCGCATTCTGACCTCTAAGGCTTGTGAGGCGCTGCAGCTCGTGGAGGAGAAACTGACCGAGGCCAAGGTCATCAGAATTGATTACAACAAACCTCTCAGCTTACTCATAATCAGAACCGAATATACCCCGACCGGTTGCCTCTGGCAGGACGGCGTACTGGAGTGGATCCACCTCCCGCACACCAACTCTAGAATTATCTCTACCTATGATATCCTCTGCGCTGCCCTAATTGTCAAGGCCCGGAACAGAAGCAAGGAAATCTTTGGCCGGGATATGCAGGAGATCGTTGTGCCATACACACGCCAGCAATTTGAGCACCTCCTGCAGGAATCCTATGACTGGGGCTTGGCCCTGATCGGATACCTTGGCATCATCAAGTTCCACCTGCCCAACATCCAAAGCCTGATCCATAAAAGAACACCTATTATCTTTCCTTCTAACTGGAGCACTGAGCCTATCGAAAAAGCAAAGGTGATTTTCACCGACGGCAGCAGCAAAGGAAACTCTGTGATGATTGTGCAGGGCGAACCCCCCATCATCAAGTATGAGAAGGGCCTGAGCGCTCAGAAGGCAGAGATCATTGCCGTCATCCTCGCGTTTGAAAAGGTGAAGGAGCCGTTCAACCTCTACACAGACTCTCGGTACGTTGCTGGGCTGTTCCCTGCAATCGAGACCGCCATGATCAGTATAAGGACCGAAATCAACGACTTACTGTCCAAGCTGAAGGAGCTGGCGCAGAAAAGGAAATTTAAGTACCATGTGGCGCACATAAGAGGCCACACCAAGCTGCCAGAGCCTCTGGCCCAAGGGAACGCCATCGCCGACTCGTACACGCAGGTGTTCATCGCTCTGGAGAATGCAATGGCGTCCCACAAGCTCCACCACCAGAATGCC
RT 36	364	RT_Rtv	XP_010852892.1	2-594	PREDICTED: LOW QUALITY PROTEIN: endogenous retrovirus group K member 11 Pol protein-like	Bison bison bison	PEAIVFAADPITWKSQDPVWVEQWPLTEEKLLAAKALISEQLELGHIEPSNSPWNTPIFVIKKKSGKWRLLQDLRAINATMEDMGALQPGLPSPVAIPEGYNIIVIDLQDCFFTIPLNAEDKKRFAFSLPAENFKQPYLRFQWKVLPQGMKNSPTLCQKFVNAAIEDIRAKYEQLYMIHYMDDILIAHPDRAHLQTVLQDLTQALTDRGLKIAPEKIQVNPPITYLGRVINSETVTHAPLKLRKDHLVTLNDYQKLLGDINWIRPYLKLTTAELKPLFNILRGDPDPTSKRQLTAEAQEALEKVEAALSDSYVKRVNLQTNWQFLCLATPTAPTGVLWQNGPLEWVHLPAQAKKVVASYPALIATLILKGRKRSIELFGKEPAEIVIPYNKEQLDALLMFDEDWQIAIGNYFGQILHHLPSHVLLNFMSKHPVIFPVRCKYSPISDAQMVFTDGSANGRASIVTKDERKILHTQETSVQRAELTAVIEAFVMFAEEEFNLYSDSQYVVRLFPHIETAILPENKTALFHLLTKLQQQIWKRSRAYFIGHIRAHSGLPGPLNALNDLADSLTKVTVASAFEEARASHSLHHQNATA	yes	MPEAIVFAADPITWKSQDPVWVEQWPLTEEKLLAAKALISEQLELGHIEPSNSPWNTPIFVIKKKSGKWRLLQDLRAINATMEDMGALQPGLPSPVAIPEGYNIIVIDLQDCFFTIPLNAEDKKRFAFSLPAENFKQPYLRFQWKVLPQGMKNSPTLCQKFVNAAIEDIRAKYEQLYMIHYMDDILIAHPDRAHLQTVLQDLTQALTDRGLKIAPEKIQVNPPITYLGRVINSETVTHAPLKLRKDHLVTLNDYQKLLGDINWIRPYLKLTTAELKPLFNILRGDPDPTSKRQLTAEAQEALEKVEAALSDSYVKRVNLQTNWQFLCLATPTAPTGVLWQNGPLEWVHLPAQAKKVVASYPALIATLILKGRKRSIELFGKEPAEIVIPYNKEQLDALLMFDEDWQIAIGNYFGQILHHLPSHVLLNFMSKHPVIFPVRCKYSPISDAQMVFTDGSANGRASIVTKDERKILHTQETSVQRAELTAVIEAFVMFAEEEFNLYSDSQYVVRLFPHIETAILPENKTALFHLLTKLQQQIWKRSRAYFIGHIRAHSGLPGPLNALNDLADSLTKVTVASAFEEARASHSLHHQNATA	**	ATGCCTGAAGCCATCGTCTTCGCCGCTGACCCCATCACATGGAAATCACAGGATCCTGTCTGGGTGGAGCAATGGCCACTGACTGAGGAGAAGCTGCTGGCGGCAAAGGCCCTGATTTCTGAGCAGCTTGAGCTTGGGCATATTGAACCTAGCAATAGCCCTTGGAACACACCAATTTTTGTGATCAAGAAGAAAAGCGGGAAATGGAGGCTCCTGCAGGACTTGAGAGCCATCAATGCCACAATGGAGGATATGGGCGCCCTACAGCCCGGCCTGCCTAGCCCAGTGGCCATCCCTGAAGGATACAACATAATCGTGATCGATCTGCAGGACTGCTTCTTCACCATCCCTCTGAACGCCGAGGACAAGAAGCGTTTCGCCTTCTCGCTGCCCGCTGAAAATTTCAAGCAACCATACCTCAGATTCCAGTGGAAGGTGCTTCCTCAGGGAATGAAAAACAGCCCTACTCTGTGCCAAAAGTTTGTTAACGCGGCCATCGAAGACATCCGTGCAAAGTATGAGCAGCTATACATGATCCACTATATGGATGATATCCTAATTGCGCACCCCGACAGGGCCCACCTGCAGACGGTACTGCAGGATCTAACACAAGCCTTGACAGACCGGGGCTTGAAAATCGCCCCTGAGAAGATACAGGTCAACCCGCCGATTACATACCTCGGTCGGGTGATCAACTCCGAGACCGTGACCCACGCGCCTCTAAAGCTGAGAAAGGATCACTTAGTGACCTTAAATGACTACCAGAAGCTGTTAGGGGACATTAACTGGATCCGGCCATACTTAAAACTGACCACCGCCGAACTAAAGCCGCTGTTCAACATCCTACGGGGAGACCCAGATCCCACCTCTAAGAGACAGTTAACCGCCGAAGCACAGGAGGCTCTCGAGAAGGTGGAAGCGGCGCTCTCAGACAGCTATGTGAAGAGAGTCAACTTACAAACAAATTGGCAGTTCTTGTGTCTCGCCACCCCGACCGCGCCCACCGGCGTGCTCTGGCAAAACGGCCCTCTGGAGTGGGTACATCTACCCGCACAGGCCAAGAAGGTGGTCGCCTCTTACCCGGCCCTCATAGCCACCCTTATCCTTAAGGGTAGAAAGCGCTCCATCGAGCTCTTCGGCAAGGAGCCGGCCGAGATCGTGATACCTTACAATAAAGAACAGCTCGACGCTCTGCTGATGTTCGATGAGGACTGGCAGATTGCCATCGGAAACTATTTCGGCCAGATACTGCACCATCTTCCCAGCCATGTCCTGCTGAACTTCATGTCAAAGCACCCGGTGATTTTTCCGGTGAGGTGCAAGTACAGCCCAATTTCCGACGCCCAGATGGTGTTTACAGACGGCAGCGCAAATGGAAGGGCAAGCATCGTGACTAAGGATGAAAGAAAAATCCTCCACACACAGGAAACTTCTGTGCAACGGGCTGAGCTGACCGCCGTTATCGAAGCGTTCGTCATGTTTGCTGAGGAGGAGTTCAATCTCTACAGTGATAGCCAATACGTCGTGCGGTTGTTCCCCCACATAGAGACCGCGATATTGCCGGAGAACAAGACCGCCCTGTTCCATCTGCTGACAAAGCTGCAGCAGCAAATCTGGAAGAGAAGTCGCGCCTACTTCATCGGCCACATCAGGGCCCACAGTGGCCTCCCTGGCCCTCTCAACGCATTAAATGACCTGGCCGACAGCCTCACAAAAGTTACCGTAGCGAGTGCTTTTGAGGAGGCTAGAGCCAGCCACTCGCTGCACCACCAGAACGCGACTGCT
RT 37	365	RT_Rtv	XP_014396142.1	1-377	endogenous retrovirus group K member 6 Pol protein-like	Myotis brandtii	PLDTGSNQEKGLGKESNTSHSSTGFIIGAIAHADPIVWKSSQPVWVEQWPLTSEKIAAARQLIDQQLAEGHIEPSNSPWNTPIFVIKKKSGKWRLLQDLRAINDTMEDMGSLQPGLPSPVAIPQEYCVVVIDLQDCFFTIPLHPADCQRFAFSIPSENFKQPYQRYQWKVLPQGMKNSPTLCQKFVDQALQVIRKKFSDLYLIHYMDDILLAHKDRATLQEILTQTVLALEEHGLKIAPEKIQTEPSFSYLGRILHTSTITHQPLQLRKDHLNTLNDYQKLLGDINWVRSYLKITTLDLKPLFDILKGDSNPKSPRQLTVEGEKAIAKIEQAINKQQLQYLDYSKSWALIILATKYTPTGCLWQEGPLEWIHLPVTPRKIVPSYPSLVATLIIKGRRRSIELFGREVTEIIIPYKREQLDTLLQFEEEWQIAIGNFPGQILHHLPRSPILQFLSLHPFIFPIKCSKEPLSPPASLVFTDGSSNGRAVTIIDSITHVQQTLETSAQELLAVIYAFEQLQQVPFNLYTDSQYIVKLFPHIETASLPQGAIFSLLTQLQTYIHKREKAFYIGHIRAHSCLPGPLSEGNYQADLLTRPVVCTALEEARRSHAIHHQN	yes	MPLDTGSNQEKGLGKESNTSHSSTGFIIGAIAHRTADPIVWKSSQPVWVEQWPLTSEKIAAARQLIDQQLAEGHIEPSNSPWNTPIFVIKKKSGKWRLLQDLRAINDTMEDMGSLQPGLPSPVAIPQEYCVVVIDLQDCFFTIPLHPADCQRFAFSIPSENFKQPYQRYQWKVLPQGMKNSPTLCQKFVDQALQVIRKKFSDLYLIHYMDDILLAHKDRATLQEILTQTVLALEEHGLKIAPEKIQTEPSFSYLGRILHTSTITHQPLQLRKDHLNTLNDYQKLLGDINWVRSYLKITTLDLKPLFDILKGDSNPKSPRQLTVEGEKAIAKIEQAINKQQLQYLDYSKSWALIILATKYTPTGCLWQEGPLEWIHLPVTPRKIVPSYPSLVATLIIKGRRRSIELFGREVTEIIIPYKREQLDTLLQFEEEWQIAIGNFPGQILHHLPRSPILQFLSLHPFIFPIKCSKEPLSPPASLVFTDGSSNGRAVTIIDSITHVQQTLETSAQRTELLAVIYAFEQLQQVPFNLYTDSQYIVKLFPHIETASLPQGRTAIFSLLTQLQTYIHKREKAFYIGHIRAHSCLPGPLSEGNYQADLLTRPVVCTALEEARRSHAIHHQN	**	ATGCCTTTGGACACCGGGAGCAATCAGGAAAAAGGTCTGGGCAAGGAATCAAACACTAGCCACAGCAGTACCGGGTTCATCATCGGGGCCATCGCGCACCGCACCGCCGACCCTATTGTCTGGAAGAGCTCACAGCCGGTGTGGGTTGAGCAATGGCCACTGACCTCTGAGAAGATAGCCGCTGCCCGACAGCTGATTGATCAGCAGCTTGCCGAGGGGCACATTGAACCATCCAACTCGCCGTGGAATACCCCTATATTTGTGATAAAGAAGAAGAGCGGGAAGTGGCGATTGCTGCAGGACCTGCGCGCTATCAATGATACGATGGAGGACATGGGATCTCTGCAGCCCGGCCTGCCTTCTCCTGTGGCAATACCTCAGGAGTACTGCGTGGTGGTCATCGACCTTCAGGATTGCTTCTTCACCATCCCTCTTCACCCCGCGGACTGCCAGCGCTTCGCCTTCAGCATACCCAGCGAAAATTTCAAACAACCTTACCAGAGGTACCAGTGGAAAGTTCTGCCGCAGGGGATGAAGAATTCTCCTACCCTTTGTCAGAAGTTCGTCGACCAGGCCCTCCAGGTCATAAGAAAGAAATTTTCCGACCTCTACCTGATCCACTACATGGATGATATCCTTCTGGCACATAAGGATAGGGCCACCCTTCAAGAGATTCTGACCCAGACGGTGCTCGCCTTGGAGGAGCATGGGCTCAAGATCGCCCCAGAGAAAATCCAAACAGAACCTTCGTTCTCGTACCTGGGCAGAATTTTACACACCAGCACAATCACACACCAGCCTCTGCAACTGAGGAAGGACCACCTCAACACACTGAACGACTACCAAAAGCTGCTGGGCGACATCAACTGGGTGAGAAGCTATCTGAAGATAACCACCCTGGATCTGAAACCACTGTTTGATATCTTGAAGGGCGATTCCAACCCGAAGAGTCCTCGGCAGCTAACGGTGGAGGGAGAGAAGGCTATCGCTAAGATTGAGCAGGCTATCAACAAGCAGCAACTGCAGTATCTTGATTACTCAAAATCTTGGGCGCTGATCATTCTCGCCACGAAATATACCCCAACTGGCTGCCTGTGGCAAGAAGGCCCTCTGGAGTGGATACACTTGCCTGTTACCCCTAGAAAGATTGTGCCCTCATACCCTAGCCTTGTGGCCACCTTGATTATCAAAGGAAGACGTAGATCTATCGAGCTGTTTGGCCGGGAGGTGACTGAAATCATCATCCCATACAAGAGAGAACAGCTTGACACACTTCTCCAGTTTGAAGAAGAATGGCAGATTGCCATCGGCAACTTCCCCGGCCAAATTCTTCACCACCTACCGCGGAGCCCAATCCTGCAGTTCCTCAGCCTGCACCCATTTATCTTCCCCATCAAATGTTCTAAGGAGCCTCTCTCCCCCCCCGCCTCGCTGGTATTCACGGACGGTTCTAGCAATGGCCGCGCCGTCACCATCATCGACTCCATTACTCATGTCCAACAGACCCTGGAGACCAGCGCGCAGAGAACTGAGCTGCTCGCCGTGATCTACGCGTTTGAACAACTGCAGCAGGTGCCGTTCAATCTGTACACAGATTCTCAGTACATCGTGAAGCTATTTCCGCACATTGAAACTGCCTCCCTGCCCCAGGGAAGAACAGCCATCTTCTCCTTGCTGACTCAGTTACAGACATACATACATAAGAGAGAGAAGGCATTCTACATAGGCCACATCCGGGCCCACAGCTGCCTTCCTGGCCCACTGAGTGAAGGCAACTATCAAGCTGACTTACTTACTCGGCCTGTGGTGTGTACAGCTCTGGAGGAGGCAAGGAGATCCCACGCTATCCATCACCAAAAC
RT 38	366	RT_Rtv	NP_056891.1	920-1497	RT-IN	Mason-Pfizer monkey virus	PAPQQCAEPITWKSDEPVWVDQWPLTNDKLAAAQQLVQEQLEAGHITESSSPWNTPIFVIKKKSGKWRLLQDLRAVNATMVLMGALQPGLPSPVAIPQGYLKIIDLKDCFFSIPLHPSDQKRFAFSLPSTNFKEPMQRFQWKVLPQGMANSPTLCQKYVATAIHKVRHAWKQMYIIHYMDDILIAGKDGQQVLQCFDQLKQELTAAGLHIAPEKVQLQDPYTYLGFELNGPKITNQKAVIRKDKLQTLNDFQKLLGDINWLRPYLKLTTGDLKPLFDTLKGDSDPNSHRSLSKEALASLEKVETAIAEQFVTHINYSLPLIFLIFNTALTPTGLFWQDNPIMWIHLPASPKKVLLPYYDAIADLIILGRDHSKKYFGIEPSTIIQPYSKSQIDWLMQNTEMWPIACASFVGILDNHYPPNKLIQFCKLHTFVFPQIISKTPLNNALLVFTDGSSTGMAAYTLTDTTIKFQTNLNSAQLVELQALIAVLSAFPNQPLNIYTDSAYLAHSIPLLETVAQIKHISETAKLFLQCQQLIYNRSIPFYIGHVRAHSGLPGPIAQGNQRADLATKIVASNINTN	yes	MPAPQQCAEPITWKSDEPVWVDQWPLTNDKLAAAQQLVQEQLEAGHITESSSPWNTPIFVIKKKSGKWRLLQDLRAVNATMVLMGALQPGLPSPVAIPQGYLKIIDLKDCFFSIPLHPSDQKRFAFSLPSTNFKEPMQRFQWKVLPQGMANSPTLCQKYVATAIHKVRHAWKQMYIIHYMDDILIAGKDGQQVLQCFDQLKQELTAAGLHIAPEKVQLQDPYTYLGFELNGPKITNQKAVIRKDKLQTLNDFQKLLGDINWLRPYLKLTTGDLKPLFDTLKGDSDPNSHRSLSKEALASLEKVETAIAEQFVTHINYSLPLIFLIFNTALTPTGLFWQDNPIMWIHLPASPKKVLLPYYDAIADLIILGRDHSKKYFGIEPSTIIQPYSKSQIDWLMQNTEMWPIACASFVGILDNHYPPNKLIQFCKLHTFVFPQIISKTPLNNALLVFTDGSSTGMAAYTLTDTTIKFQTNLNSAQLVELQALIAVLSAFPNQPLNIYTDSAYLAHSIPLLETVAQIKHISETAKLFLQCQQLIYNRSIPFYIGHVRAHSGLPGPIAQGNQRADLATKIVASNINTN	*	ATGCCTGCCCCGCAGCAATGCGCCGAACCCATCACATGGAAGAGTGATGAACCTGTGTGGGTGGATCAATGGCCGCTGACGAATGATAAGCTGGCCGCCGCGCAGCAGTTAGTTCAGGAACAACTGGAGGCGGGCCATATCACCGAGTCTAGCAGTCCATGGAACACTCCTATCTTTGTGATCAAGAAGAAAAGCGGGAAGTGGCGGCTGCTGCAAGACCTGCGGGCGGTCAACGCTACCATGGTGCTGATGGGGGCGCTGCAGCCCGGCCTCCCAAGCCCCGTCGCCATCCCACAGGGTTATCTTAAGATAATCATCGACTTGAAGGACTGCTTCTTCTCAATCCCTCTTCACCCTTCGGACCAGAAGAGGTTCGCCTTCTCCCTGCCTAGCACCAACTTCAAGGAACCAATGCAGAGATTTCAATGGAAGGTTCTGCCTCAGGGCATGGCAAACTCCCCCACACTGTGCCAAAAATACGTGGCCACCGCCATCCACAAGGTGCGCCACGCGTGGAAACAAATGTACATTATCCATTACATGGATGATATACTAATTGCGGGCAAGGATGGCCAGCAGGTGCTGCAGTGCTTTGATCAACTCAAGCAGGAGCTCACCGCCGCCGGCCTGCACATCGCGCCTGAGAAAGTGCAGTTGCAGGACCCATACACCTACCTGGGTTTTGAACTGAATGGTCCTAAAATCACTAACCAGAAAGCTGTTATTCGGAAAGACAAGCTGCAAACCCTGAACGATTTCCAGAAGCTACTCGGCGACATCAACTGGCTCCGGCCCTACCTGAAGCTGACCACCGGAGACTTGAAGCCGCTGTTCGACACCCTGAAGGGTGATTCTGATCCTAACTCCCACCGAAGCCTGAGCAAGGAGGCGCTAGCCAGCCTTGAAAAGGTGGAAACCGCGATCGCGGAGCAGTTTGTGACGCACATCAACTATAGCCTGCCGCTAATTTTCCTCATCTTCAATACCGCCCTAACACCAACGGGCCTCTTCTGGCAGGATAACCCTATCATGTGGATTCACTTACCTGCTTCCCCGAAAAAGGTGCTGCTGCCATATTATGATGCCATCGCCGACCTAATCATACTTGGCCGCGACCACTCCAAGAAGTACTTCGGCATCGAGCCTTCTACAATTATCCAGCCTTACTCGAAATCTCAAATTGACTGGTTGATGCAAAATACAGAGATGTGGCCAATCGCTTGTGCCAGCTTCGTCGGTATCCTCGACAACCACTACCCGCCGAACAAGCTCATCCAGTTCTGCAAGCTGCACACTTTTGTGTTCCCTCAAATCATTAGCAAGACGCCCCTCAACAATGCCCTTCTCGTTTTCACTGATGGCTCAAGCACTGGAATGGCCGCCTACACCCTGACAGACACAACCATCAAGTTTCAAACAAATCTGAACTCGGCTCAGCTGGTGGAACTGCAGGCACTGATCGCTGTCTTGAGCGCCTTCCCAAATCAACCACTGAACATTTACACAGACTCTGCCTACCTAGCACACAGCATCCCTCTGCTGGAGACGGTGGCCCAGATCAAGCATATCTCAGAAACTGCTAAACTTTTCCTGCAGTGTCAACAGCTGATATACAACAGATCTATCCCGTTCTACATTGGCCATGTCAGAGCTCATTCGGGCTTGCCCGGCCCTATTGCCCAGGGAAACCAAAGAGCCGACCTTGCGACCAAGATCGTTGCCTCCAACATTAACACAAAC

RT 39	367	RT_Rtv	YP_009243641.1	2-578	putative viral DNA polymerase	Bovine retrovirus CH15	PEPIALSWKSNEPIWIEQWPLTREKLQAAEELVEEQLQKGHIETSTSPWNSPIFVIRKKSGKWRLLTDLRAVNASMFPMGALQPGLPAPVMLPRDWPLLIIDLLDCFFTIPIHPQDKQRFAFSIPKINNAGPHTRYQWRVLPQGMMNSPTICQLYVDAALKPIRKRYPFLHIYHYMDDILFAGPKKEFLQDLLEQLPLYFKPYGLIIAPEKIQLEDISHYLGFVVSTVHPQKMTIRRDNLKTLNDFQKLLGDINWLRPSLGIPTYQLHNLFDTLKGDTDLNSARCLTPAAEEELQFFEHQLKSKFLMRIDPLLPLTLYLINTFMYPTALLGQNEEPCEWLYTRHRFHRSIMSYEQQIAILIQQGRQRCISLSGYDVSTIVLPLTKSQFFFLLQRSEALQIALAQFVGNIEYTLPKGKLWDFFKRHNFVINQIISDIPLEGPNVFTDGSRQRSAYWAEKKYWSQKQNTNSIQKNELLAIIQVLKDFKQDINIIADSAYVVGVVKNIIGAVINSLDKELTTLFKTLQVLLTSRNARIFIVHIRSHSKLPGPLVYGNEQVDKLAAFASPEEEHRYFHNN	yes	MPEPIALSWKSNEPIWIEQWPLTREKLQAAEELVEEQLQKGHIETSTSPWNSPIFVIRKKSGKWRLLTDLRAVNASMFPMGALQPGLPAPVMLPRDWPLLIDLLDCFFTIPIHPQDKQRFAFSIPKINNAGPHTRYQWRVLPQGMMNSPTICQLYVDAALKPIRKRYPFLHIYHYMDDILFAGPKKEFLQDLLEQLPLYFKPYGLIIAPEKIQLEDISHYLGFVVSRTTVHPQKMTIRRDNLKTLNDFQKLLGDINWLRPSLGIPTYQLHNLFDTLKGDTDLNSARCLTPAAEEELQFFEHQLKSKFLMRIDPLLPLTLYLINTFMYPTALLGQNEEPCEWLYTRHRFHRSIMSYEQQIAILIQQGRQRCISLSGYDVSTIVLPLTKSQFFFLLQRSEALQIALAQFVGNIEYTLPKGKLWDFFKRHNFVINQIISDIPLEGPNVFTDGSRQRSAYWAEKKYWSQKQNTNSIQKNELLAIIQVLKDFKQDINIIADSAYVVGVVKNIIGAVINSLDKELTTLFKTLQVLLTSRNARIFIVHIRSHSKLPGPLVYGNEQVDKLAAFASPEEEHRYFHNN	*	ATGCCTGAGCCAATCGCTCTGTCATGGAAGTCAAATGAACCCATTTGGATTGAACAGTGGCCTTTAACCCGGGAGAAGTTACAGGCCGCCGAAGAGCTCGTCGAGGAGCAGCTGCAGAAGGGCCACATCGAGACCTCCACAAGCCCGTGGAACTCTCCCATATTTGTGATCCGAAAGAAAAGCGGCAAATGGAGATTACTGACTGATCTTAGAGCTGTCAATGCGTCCATGTTCCCAATGGGCGCCTTGCAGCCGGGCCTCCCCGCTCCGGTGATGCTCCCTCGCGACTGGCCGCTCCTGATAATCGATCTGCTCGATTGCTTCTTCACCATACCTATCCATCCCCAAGATAAACAGAGATTTGCATTCTCTATCCCAAAGATCAATAATGCAGGGCCACACACCAGATACCAATGGAGGGTGCTGCCTCAGGGAATGATGAATTCCCCTACCATCTGCCAGTTGTACGTCGACGCCGCCTTGAAACCTATCAGGAAGAGATATCCATTTCTCCACATTTACCATTACATGGACGATATCCTGTTCGCCGGCCCCAAGAAGGAATTTCTGCAGGATCTGCTGGAGCAACTGCCGCTGTACTTTAAGCCATATGGACTCATTATTGCTCCTGAAAAGATTCAGCTGGAGGACATCAGTCACTATCTCGGTTTCGTGGTGAGCAGAACGACGGTCCACCCACAGAAGATGACAATACGGCGTGACAACCTGAAAACACTGAATGACTTCCAGAAGCTGTTGGGCGACATCAACTGGCTGCGCCCGAGTCTGGGCATTCCTACTTACCAGCTTCACAACCTGTTCGATACCCTGAAGGGGGATACGGACCTCAACAGCGCGAGATGCCTGACCCCCGCCGCCGAAGAAGAATTGCAGTTCTTTGAGCATCAGCTGAAATCCAAATTTCTCATGAGAATTGATCCTCTCCTGCCTCTTACCCTGTACCTGATCAACACTTTCATGTACCCAACAGCCCTGCTCGGCCAAAATGAGGAGCCTTGTGAGTGGCTCTACACCCGCCACCGCTTCCACCGAAGCATTATGTCATATGAACAGCAAATCGCCATCCTGATACAGCAGGGCAGACAGCGGTGTATAAGCCTCTCGGGCTATGATGTGAGCACAATTGTGCTACCTCTGACTAAGAGCCAATTCTTCTTCCTGCTGCAACGGAGCGAAGCACTGCAAATCGCCCTGGCGCAGTTTGTAGGAAACATTGAGTATACCCTGCCGAAAGGAAAGCTCTGGGATTTTTTCAAGAGACATAATTTCGTTATTAACCAAATCATCTCTGACATCCCGCTGGAGGGCCCCAACGTCTTCACAGATGGGAGCCGGCAGAGATCCGCGTATTGGGCAGAGAAGAAATACTGGTCTCAGAAGCAGAACACAAACTCTATTCAAAAGAATGAACTTCTCGCCATCATCCAGGTTCTGAAAGATTTCAAGCAAGACATCAATATCATCGCCGACTCCGCTTACGTGGTGGGTGTGGTGAAGAACATCATCGGCGCGGTGATCAACTCTCTGGACAAGGAGCTGACAACCCTGTTCAAAACCTTACAGGTGTTACTGACCAGCCGTAACGCTCGCATCTTCATCGTGCATATCAGATCACACAGTAAGCTGCCCGGCCCTTTGGTCTACGGCAACGAGCAGGTAGACAAATTAGCCGCCTTCGCTAGCCCGGAGGAAGAGCACAGATACTTCCATAACAAC
RT 40	381	RT_Rtv	BAF81993.1	19-577	pol partial	Mus musculus	AEKIQWRNDIPVWVDQWSLPKEKIEAASLLVQEQLEAGHLVESHSPWNTPIFIIRKKSGKWRLLQDLRKVNETMVLMGTLQPGLPSPVAIPKGYYKIVIDLKDCFFTIPLHPKDCERFAFSVPSVNFKEPMKRYHWTVLPQGMANSPTLCQRFVAKAIQPVRQQWPNIYIIHFTDDVLMAGKDPQDLLLCYGDLQKALADKGLQIASEKIQTQDPYNYLGFRLTDQAVFPQKIVIRRDNLLNDFQKLLGDINWLRPYLKLTTGELKPLFDILKGSSDPTSPRSLTSEGLLALQLVEKAIEEQFVTYIDYSLPLHLLIFNTTHVPTGLLWQKFPIMWIHSRISPKRNILPYHEAVAQMIITGRRQALTYFGKEPDIIVQPYSVSQDTWLKQHSTDWLLAQLGFEGTLDSHYPQDRLIKFLNVHDMIFPKMTSLQPLNNALLIFTDGSSKGRAGYLISNQQVIVETPGLSAQLAELTAVLKVFQSVHEAFNIFTDSLYVAQSVPLLETCGTFNFNTPSGSLFSELQNIILARKNPFYIGHIRSHSGLPGPLAEGNDRID---	yes	MAEKIQWRNDIPVWVDQWSLPKEKIEAASLLVQEQLEAGHLVESHSPWNTPIFIIRKKSGKWRLLQDLRKVNETMVLMGTLQPGLPSPVAIPKGYYKIVIDLKDCFFTIPLHPKDCERFAFSVPSVNFKEPMKRYHWTVLPQGMANSPTLCQRFVAKAIQPVRQQWPNIYIIHFTDDVLMAGKDPQDLLLCYGDLQKALADKGLQIASEKIQTQDPYNYLGFRLTDQAVFPQKIVIRRDNLLNDFQKLLGDINWLRPYLKLTTGELKPLFDILKGSSDPTSPRSLTSEGLLALQLVEKAIEEQFVTYIDYSLPLHLLIFNTTHVPTGLLWQKFPIMWIHSRISPKRNILPYHEAVAQMIITGRRQALTYFGKEPDIIVQPYSVSQDTWLKQHSTDWLLAQLGFEGTLDSHYPQDRLIKFLNVHDMIFPKMTSLQPLNNALLIFTDGSSKGRAGYLISNQQVIVETPGLSAQLAELTAVLKVFQSVHEAFNIFTDSLYVAQSVPLLETCGTFNFNTPSGSLFSELQNIILARKNPFYIGHIRSHSGLPGPLAEGNDRID	/	ATGGCCGAAAAGATTCAGTGGAGAAATGACATCCCTGTTTGGGTGGATCAATGGAGCCTGCCGAAGGAAAAAATCGAAGCCGCCTCACTGCTCGTCCAGGAGCAGCTGGAAGCGGGCCACCTGGTAGAAAGCCACAGCCCGTGGAACACACCGATTTTCATTATTAGAAAGAAGAGTGGCAAATGGAGACTGCTTCAGGACCTGAGGAAGGTGAATGAGACCATGGTGTTGATGGGAACCCTGCAACCTGGCTTGCCTAGCCCGGTGGCCATCCCAAAGGGCTACTACAAGATCGTCATCGATCTAAAGGACTGCTTCTTCACAATACCTTTGCACCCCAAAGATTGTGAGAGATTCGCGTTCTCCGTGCCGTCTGTCAACTTCAAGGAGCCAATGAAGAGGTACCACTGGACCGTCCTGCCCCAGGGCATGGCGAACTCTCCCACGCTTTGCCAGAGATTTGTCGCCAAGGCTATCCAGCCGGTGCGTCAGCAGTGGCCAAACATCTACATAATCCATTTTACAGATGACGTGCTCATGGCCGGGAAGGATCCACAGGACCTGTTGTTGTGTTATGGTGACCTGCAGAAGGCCCTCGCCGACAAGGGACTCCAAATCGCAAGTGAGAAGATCCAGACTCAGGATCCTTACAACTACCTCGGCTTCCGTCTCACCGACCAGGCGGTGTTCCCGCAGAAGATCGTGATCCGACGCGACAACCTCCTAAACGACTTCCAAAAACTCCTTGGCGATATTAATTGGCTGCGGCCGTACTTAAAGTTAACAACTGGCGAATTAAAGCCTTTGTTCGACATTCTCAAAGGAAGCTCAGACCCAACCTCGCCTAGAAGCCTCACCTCAGAGGGGCTGCTGGCTCTGCAGCTTGTGGAGAAGGCTATCGAGGAACAGTTTGTCACGTACATTGATTATTCACTGCCTCTCCACCTCCTGATATTCAACACCACTCACGTTCCCACCGGCCTGCTTTGGCAGAAGTTTCCTATCATGTGGATACACTCGCGGATCTCGCCTAAAAGAAACATCCTACCTTACCATGAGGCGGTGGCCCAAATGATCATAACTGGTCGGAGGCAGGCTCTCACCTACTTCGGCAAGGAGCCGGACATAATAGTGCAACCATATTCTGTGTCTCAGGACACATGGCTGAAACAACACTCCACCGACTGGCTTCTCGCGCAGCTGGGCTTCGAGGGTACATTAGACTCCCACTATCCCCAAGATCGGTTGATCAAGTTTCTCAACGTGCATGACATGATATTCCCTAAAATGACTAGCCTGCAGCCTTTAAACAATGCACTGCTGATCTTCACCGACGGCTCTTCGAAGGGACGTGCCGGGTACCTCATTTCGAACCAACAGGTGATCGTTGAAACACCCGGCCTGAGCGCACAGCTCGCCGAGCTGACAGCTGTACTGAAGGTTTTCCAGAGTGTGCATGAAGCATTCAATATCTTCACCGACAGCCTCTACGTGGCACAAAGCGTCCCTCTGCTGGAGACCTGCGGCACGTTCAACTTTAACACCCCTTCCGGCAGTCTTTTCTCTGAGTTGCAAAACATCATTCTGGCAAGAAAAAATCCATTCTATATCGGGCACATTAGATCTCATAGCGGACTGCCCGGCCCGCTGGCTGAGGGAAATGATAGAATTGAC
RT 41	415	RT_Rtv	NLL75617.1	2-287	group II intron reverse transcriptase/maturase	Erysipelothrix sp.	EKNLLNIILSDDNLNEAYKRVHKNKGASGVDKMMTSDLKQHLKEHGQEIKEQIRQRKYKPQPVLRVEIPKPDGGVRLLGIPTVTDRFIQQAISQVIGPIFDEQFSDYSYGFRPKRYAEMAIVRALELMNDGYKWIVDIDLEKFFDTVHHDKLMRIVSKTIKDGDVISLIRKYLVSGIVIDEQYHESIIGTPQGGNLSPLLSNIMLNELDKELEIRGLNFVRYADDCLILVGSEKAAKRVMERITKFIEEELGLKVNATKSKVSEPKDIKFLGFAFYWNKYQYQFKA	yes	MEKNLLNIILSDDNLNEAYKRVHKNKGASGVDKMMTSDLKQHLKEHGQEIKEQIRQRKYKPQPVLRVEIPKPDGGVRLLGIPTVTDRFIQQAISQVIGPIFDEQFSDYSYGFRPKRYAEMAIVRALELMNDGYKWIVDIDLEKFFDTVHHDKLMRIVSKTIKDGDVISLIRKYLVSGIVIDEQYHESIIGTPQGGNLSPLLSNIMLNELDKELEIRGLNFVRYADDCLILVGSEKAAKRVMERITKFIEEELGLKVNATKSKVSEPKDIKFLGFAFYWNKYQYQFKA	*	ATGGAGAAGAATCTTTTGAATATTATCCTCTCTGATGACAACCTCAATGAAGCGTACAAACGAGTCCACAAGAACAAAGGCGCCTCGGGTGTCGACAAGATGATGACATCCGACCTCAAGCAGCATTTAAAAGAGCACGGCCAGGAGATTAAGGAGCAGATACGGCAAAGGAAGTATAAACCACAACCTGTGCTGAGAGTGGAGATCCCCAAGCCTGATGGAGGTGTACGGCTGCTCGGGATTCCGACGGTGACTGACCGCTTCATCCAGCAGGCGATATCTCAGGTGATCGGGCCCATCTTTGATGAACAGTTCAGTGACTACAGCTATGGATTTAGACCAAAGAGATATGCTGAGATGGCGATAGTACGAGCGCTGGAGCTCATGAATGATGGCTACAAGTGGATTGTTGATATTGATCTTGAGAAGTTCTTCGACACCGTGCACCATGATAAGCTGATGAGGATTGTGAGCAAGACGATAAAGGATGGTGATGTTATTTCTTTGATAAGGAAATATCTCGTCTCCGGGATCGTCATCGACGAGCAATATCATGAATCAATCATTGGAACTCCTCAAGGCGGCAACTTGTCGCCGCTTCTCAGCAACATTATGCTGAATGAGCTGGACAAAGAATTGGAAATCCGCGGCCTCAACTTCGTCCGCTACGCCGACGACTGCCTCATCCTAGTTGGAAGTGAGAAAGCAGCCAAGAGGGTCATGGAACGTATCACAAAATTTATTGAGGAGGAATTGGGCCTGAAGGTTAATGCTACCAAGTCAAAGGTGTCCGAACCAAAAGATATCAAGTTTCTTGGCTTCGCCTTCTACTGGAACAAGTACCAGTACCAATTCAAGGCT
RT 42	421	RT_ZF REV_like	XP 019063396.1	111-798	LOWQUALITY PROTEIN: uncharacterized protein LOC109118148	Fukomys damarensis	VPLEEEFKLIGEEGKSPEQKWNYLREKFPQVWAETNLPGLAKHQVPIVVQVRATASPVRVRQYPLRLEAKIKISQHIHXLLKAGILIPCQSAWNTPLLPVRKPGTSDYRPVQDLREVNSRVETIHPTVPNPYTLLSLIPPVHIWYSVLDLKDAFFSLPLAPVSQPLFAFEWTDPDTGTTGQLTWTRLPQGFKNSPTLFGEALSKDLQEVREAFPFATILQYVDDLLVATSTEKECLTDTEGLLEALQDLGYRVSWKKAQLCQTKVTYLGYILEKGERKLAESRVSAILQIPVPCSKKQVREFLGAIGYCRIWIPSFAEIARPLHEATAGGNTPLQWTEACQQAFQTLKTALTTAPALALPDLTKPFTLYVAEKNGVAKGVVTQPLGPWKRPIAYLSKRLDTVARGWPSCLRMIAAAATLTREADKLTFGQDLTLITPHAIEPLLHSSPSRWLSNARLLQYQGLLIEQPRIKFATTSNLNPASLLPESDDQLPIHDCLETLENLRGGRPDLTDLALPQPEATYYTDGCSFVREGVRYAGAAIVDQNQQMVWEQGLPQGTSAQKAELIALTKALELGKGKRINIFTDSRYAFATAHVHGQIYQNRGLLTSSGKEIKNKTEILALLQAIWLPREVAIIHCPGHQRNSSPEAIGNNAADRAAKQAAQKSVGKELVLLPTPVLPERPEYTEEE-------------	yes	MVPLEEEFKLIGEEGKSPEQKWNYLREKFPQVWAETNLPGLAKHQVPIVVQVRATASPVRVRQYPLRLEAKIKISQHIHLLKAGILIPCQSAWNTPLLPVRKPGTSDYRPVQDLREVNSRVETIHPTVPNPYTLLSLIPPVHIWYSVLDLKDAFFSLPLAPVSQPLFAFEWTDPDTGTTGQLTWTRLPQGFKNSPTLFGEALSKDLQEVREAFPFATILQYVDDLLVATSTEKECLTDTEGLLEALQDLGYRVSWKKAQLCQTKVTYLGYILEKGERKLAESRVSAILQIPVPCSKKQVREFLGAIGYCRIWIPSFAEIARPLHEATAGGNTPLQWTEACQQAFQTLKTALTTAPALALPDLTKPFTLYVAEKNGVAKGVVTQPLGPWKRPIAYLSKRLDTVARGWPSCLRMIAAAATLTREADKLTFGQDLTLITPHAIEPLLHSSPSRWLSNARLLQYQGLLIEQPRIKFATTSNLNPASLLPESDDQLPIHDCLETLENLRGGRPDLTDLALPQPEATYYTDGCSFVREGVRYAGAAIVDQNQQMVWEQGLPQGTSAQKAELIALTKALELGKGKRINIFTDSRYAFATAHVHGQIYQNRGLLTSSGKEIKNKTEILALLQAIWLPREVAIIHCPGHQRNSSPEAIGNNAADRAAKQAAQKSVGKELVLLPTPVLPERPEYTEEE	/	ATGGTTCCACTGGAAGAGGAGTTCAAACTTATCGGAGAGGAGGGCAAATCGCCTGAACAGAAGTGGAATTATCTGCGGGAAAAGTTCCCTCAGGTCTGGGCTGAAACAAATTTGCCGGGCCTGGCAAAGCATCAGGTACCCATTGTAGTGCAGGTGAGAGCGACAGCAAGCCCGGTGCGCGTGCGGCAATACCCCCTGAGACTGGAAGCCAAAATTAAGATATCGCAGCACATCCATCTCCTGAAGGCTGGCATCTTGATCCCTTGTCAGAGCGCATGGAACACCCCCCTGCTGCCTGTGCGCAAGCCAGGAACCTCTGACTACCGGCCAGTCCAAGACCTGAGAGAAGTTAACTCGCGTGTAGAGACGATTCACCCTACGGTGCCCAACCCGTACACCTTGCTCAGCCTCATCCCGCCTGTGCACATTTGGTACTCAGTGCTCGACCTTAAGGATGCGTTCTTCTCGCTCCCCTTAGCTCCTGTGAGCCAGCCTCTCTTCGCATTTGAGTGGACAGACCCGGACACCGGCACAACCGGGCAGCTGACTTGGACTAGACTACCACAAGGATTCAAGAATTCTCCTACCCTGTTTGGAGAAGCTCTTTCCAAAGATCTGCAGGAGGTGAGGGAGGCCTTCCCTTTCGCTACCATCTTGCAGTATGTGGATGACCTGCTGGTGGCCACCAGCACAGAGAAGGAATGTTTGACAGACACCGAGGGACTGCTGGAGGCGCTGCAGGATTTAGGTTACAGGGTGTCGTGGAAGAAGGCGCAGCTGTGTCAAACAAAGGTGACTTACCTTGGCTACATCTTAGAAAAGGGCGAGAGGAAACTGGCCGAGTCTAGAGTAAGCGCCATCCTGCAGATACCGGTGCCGTGCAGCAAGAAACAAGTTCGAGAGTTCCTCGGCGCCATCGGATACTGCCGGATATGGATTCCTAGTTTTGCCGAAATCGCGCGGCCTCTGCATGAGGCCACCGCGGGCGGCAACACACCTTTGCAGTGGACTGAAGCATGCCAACAGGCGTTCCAGACACTTAAGACGGCGCTGACCACCGCCCCCGCCTTAGCCCTTCCTGATCTGACTAAACCGTTCACCCTATACGTGGCAGAAAAAAATGGTGTTGCCAAAGGAGTCGTCACGCAGCCGCTCGGACCATGGAAAAGACCTATCGCATATTTAAGTAAGAGACTCGATACTGTTGCGAGAGGGTGGCCGAGCTGCCTCCGCATGATCGCAGCCGCCGCCACGTTGACACGGGAAGCCGACAAGTTGACATTTGGTCAAGACCTGACCCTGATAACGCCTCACGCCATTGAACCTCTTCTCCACTCCAGCCCTAGCCGGTGGCTCTCCAACGCCAGACTGCTGCAGTATCAAGGCCTTCTGATTGAGCAGCCCCGCATCAAATTCGCAACCACCAGCAACCTTAACCCTGCCTCTCTGCTGCCAGAGTCAGATGATCAGCTGCCTATCCATGACTGCCTGGAGACCCTTGAGAACCTTAGAGGCGGCCGCCCCGACTTAACCGATCTTGCTTTGCCGCAGCCAGAGGCAACATATTACACTGATGGTTGCTCCTTCGTTCGTGAGGGTGTGAGATATGCCGGCGCCGCCATAGTCGACCAAAATCAGCAGATGGTGTGGGAGCAAGGCCTGCCTCAAGGCACCAGCGCTCAGAAAGCTGAGTTGATCGCCCTAACAAAAGCCCTGGAGCTCGGAAAGGGAAAGAGAATCAATATCTTCACAGATTCTCGCTACGCGTTTGCCACGGCGCACGTCCACGGTCAGATATACCAGAACAGAGGCCTGCTCACCAGCTCGGGGAAGGAAATCAAGAACAAGACAGAAATTCTTGCCCTGCTGCAGGCCATCTGGCTGCCCCGGGAGGTGGCCATCATCCACTGCCCTGGCCACCAAAGAAACAGTTCCCCAGAGGCTATCGGCAACAATGCTGCTGACCGCGCTGCCAAGCAGGCCGCTCAGAAGAGTGTGGGCAAGGAGCTTGTGCTATTACCAACACCAGTGCTGCCCGAAAGACCCGAGTACACCGAGGAAGAA
RT 43	439	RT_ZF REV_like	XP_038956651.1	620-1342	LOW QUALITY PROTEIN: uncharacterized protein LOC120099849	Rattus norvegicus	PSKGPKVTWEEAPVACLVLSLEEEYRLHEQNPKQLLAPEWLSAFSEVWAEQTGMGLAKQVPPVVVELKADASPVSVKQYPMSREAKEGIRPHIQRLLQLGILVPCQSPWNTPLLPVRKPGTNDYRPVQDLREVNKRVQDIHPTVPNPYNLLSSLPPEWYTVLDLKDAFFCLRLHPNSQPLFAFEWRDPEGGHTGQLTWTRLPQGFKNSPTLFDEALHRDLAPFRAQNPQISLLQYVDDLLLAASTQELCREGTKRLLNELGELGYRVSAKKAQLCEVTYLGYTLREGKRWLTEARKKTVMQIPTPTTPRQVREFLGTAGFCRLWIPGFATLAAPLYLLTKEKVPFTWTEEHQRAFEDIKAALLAAPALALPDLTKPFTLYVDERAGVARGVLTQALGPWKRPVAYLSKKLDPVASGWPSCLKAIAAVALLVKDADKLTLGQHVTVIAPHALESIVRQPPDRWMTNARMTHYQSLLLNERVTFAPPAILNPATLLPEIHNSTPIHQCVDILAEETGTRKDLTDRPWPGVPAWYTDGSSFVVEGKRRAGAAVVDGKQVIWASSLPEGTSAQKAELVALTQALRLAEGKAINIYTDSRYAFATAHIHGAIYKQRGLLTSAGRDIKNKEEILALLEAVHLPKKVAIIHCPGHQKGEDPITKGNQMADLVAKQVAQQVTILAEKSQTPVKTPVTDDSNYIYSANDFKILENMVYRKQAQEFSFDG	yes	MPSKGPKVTWEEAPVACLVLSLEEEYRLHEQNPKQLLAPEWLSAFSEVWAEQTGMGLAKQVPPVVVELKADASPVSVKQYPMSREAKEGIRPHIQRLLQLGILVPCQSPWNTPLLPVRKPGTNDYRPVQDLREVNKRVQDIHPTVPNPYNLLSSLPPERTWYTVLDLKDAFFCLRLHPNSQPLFAFEWRDPEGGHTGQLTWTRLPQGFKNSPTLFDEALHRDLAPFRAQNPQISLLQYVDDLLLAASTQELCREGTKRLLNELGELGYRVSAKKAQLCRTEVTYLGYTLREGKRWLTEARKKTVMQIPTPTTPRQVREFLGTAGFCRLWIPGFATLAAPLYLLTKEKVPFTWTEEHQRAFEDIKAALLAAPALALPDLTKPFTLYVDERAGVARGVLTQALGPWKRPVAYLSKKLDPVASGWPSCLKAIAAVALLVKDADKLTLGQHVTVIAPHALESIVRQPPDRWMTNARMTHYQSLLLNERVTFAPPAILNPATLLPEIHNSTPIHQCVDILAEETGTRKDLTDRPWPGVPAWYTDGSSFVVEGKRRAGAAVVDGKQVIWASSLPEGTSAQKAELVALTQALRLAEGKAINIYTDSRYAFATAHIHGAIYKQRGLLTSAGRDIKNKEEILALLEAVHLPKKVAIIHCPGHQKGEDPITKGNQMADLVAKQVAQQVTILAEKSQTPVKTPVTDDSNYIYSANDFKILENMVYRKQAQEFSFDG	/	ATGCCTAGCAAGGGCCCCAAGGTGACTTGGGAGGAGGCGCCCGTGGCGTGCTTAGTGCTCAGCCTGGAGGAAGAGTATCGGCTACATGAACAAAATCCAAAGCAACTGCTGGCTCCTGAATGGCTCTCCGCCTTCTCTGAAGTTTGGGCTGAGCAGACGGGCATGGGGTTAGCCAAACAGGTGCCGCCGGTGGTTGTGGAGCTGAAGGCCGACGCTTCCCCGGTGAGCGTAAAGCAGTACCCGATGTCGCGGGAAGCAAAGGAAGGCATCAGACCTCACATTCAAAGGCTGCTGCAGCTCGGGATCCTGGTGCCATGCCAGAGCCCATGGAACACACCTCTCCTTCCTGTGAGGAAGCCGGGCACCAATGACTACCGGCCCGTGCAGGATTTGCGGGAGGTCAATAAGAGGGTTCAGGACATCCACCCTACCGTGCCAAACCCCTACAACCTTCTGAGCAGCCTGCCACCGGAGAGGACCTGGTACACCGTGCTTGACCTCAAGGATGCATTCTTCTGCCTCAGATTGCACCCGAACTCCCAGCCACTGTTTGCTTTCGAGTGGAGGGATCCTGAGGGTGGCCATACCGGTCAGCTGACTTGGACGCGACTGCCACAGGGATTTAAGAATAGCCCAACCTTATTTGATGAGGCACTCCACCGCGACCTGGCGCCCTTCAGAGCTCAGAACCCTCAGATTAGTTTGCTGCAGTATGTGGACGACCTGCTGCTGGCCGCAAGCACTCAGGAACTCTGCAGGGAAGGCACCAAACGGCTGCTGAACGAACTGGGCGAGTTGGGCTATCGCGTCTCAGCTAAGAAGGCCCAGCTTTGTCGTACTGAGGTAACATACCTGGGGTACACCCTGAGAGAGGGCAAGCGGTGGCTGACCGAAGCCAGAAAGAAGACGGTGATGCAAATCCCAACTCCCACAACCCCTCGCCAGGTGAGAGAATTTCTGGGTACCGCCGGCTTCTGTCGGCTATGGATTCCTGGATTCGCCACCCTGGCTGCACCCCTCTACCTTCTGACCAAGGAGAAGGTTCCCTTCACATGGACAGAAGAGCATCAGCGTGCTTTTGAAGATATCAAGGCTGCTCTGCTCGCCGCCCCTGCCCTGGCTCTGCCAGACCTCACTAAGCCCTTCACGCTTTACGTGGATGAAAGAGCCGGCGTCGCCCGTGGCGTGCTCACCCAGGCCCTCGGCCCGTGGAAGAGGCCCGTCGCCTACCTGAGCAAGAAGCTGGACCCGGTGGCCAGCGGCTGGCCATCCTGCCTGAAAGCCATCGCCGCCGTGGCGCTGCTGGTGAAGGACGCCGACAAGCTGACCCTGGGACAGCACGTCACAGTCATCGCCCCCCACGCCCTGGAGTCCATCGTCAGACAACCACCTGATCGATGGATGACAAATGCTAGAATGACGCACTACCAGTCCCTACTTCTAAATGAGCGGGTGACGTTCGCGCCTCCTGCAATTCTCAACCCCGCCACGCTGCTGCCAGAGATCCACAACTCGACTCCTATCCACCAGTGTGTCGACATCCTGGCGGAGGAGACCGGCACCAGGAAAGATCTGACCGACAGACCTTGGCCTGGAGTCCCGGCGTGGTATACAGACGGCAGTTCTTTTGTGGTAGAGGGAAAGCGCCGGGCCGGCGCAGCCGTGGTGGATGGGAAGCAGGTAATCTGGGCCAGCAGTCTGCCAGAGGGAACGTCTGCACAGAAAGCTGAGCTCGTGGCCCTAACCCAGGCTCTCCGGCTGGCCGAAGGCAAGGCCATCAACATTTACACTGACAGCCGCTATGCGTTTGCTACAGCCCACATCCACGGCGCCATCTACAAGCAACGTGGACTACTCACAAGCGCGGGCAGAGACATTAAAAACAAAGAAGAAATCCTCGCCCTGCTGGAGGCCGTCCATCTCCCGAAGAAGGTCGCTATAATTCACTGCCCTGGGCACCAAAAAGGAGAGGACCCCATCACAAAAGGGAACCAAATGGCGGACCTCGTTGCAAAACAGGTCGCCCAGCAAGTGACCATCCTCGCAGAGAAATCTCAAACACCTGTGAAAACACCTGTGACCGATGATAGTAACTACATCTACTCAGCCAATGACTTCAAGATTCTGGAGAACATGGTATACAGAAAACAGGCGCAGGAGTTCTCCTTCGACGGT

RT 44	450	RT_ZF REV_like	XP_027286823.1	31-731	uncharacterized protein LOC113837321	Cricetulus griseus	PRSEVKVTGPEGIPLTILTMSIEDEYRLHEKNSNNQETLDHWLAEFPQAWAETGGMGLAINQAPIIVTLKAAILPASVRQYPMPKEAREGIRPHIKRLLEQGILVPCKSPWNTPLLPVRKPGTNDYRPVQDLREVNKRIEDIHPTVPNPYNLLSGLPPNYTWYTVLDLKDAFFCLRLHPTSQPIFAFEWQDADLGISGQLTWTRLPQGFKNSPTLFDEALHQDLAGFRVRYPALILLQYVDDILLAAKTKEECKEGTRALLQTLGSLGYRASAKKAQICQKQVTYLGYKIKDGRRWLTEARMRAILDIPTPQNPRQLREFLGTAGFCRLWIPGFAEMAAPLYPLTRPGVAFKWEEPQKKAFTNIKKALLESPALGLPDLAKPFELFIDEKGGYAKGVLTQKLGPWRRPTAYLSKKLDPVASGWPPCLRMIAAIALLVKDSYKLTLGQPLTIHAPHAVEAVIRQPPDRWLTNARMTHYQTMLLDKDRVHFGPLVTLNPATLLPLPGEPEAHNCLQVLAEAHGARPDLTDQPLPSPDHIWFTDGSSFLHQGKRRAGAAVTTENQVVWAQALPPGTSAQRAELIALTQALKLAEGKRLTVYTDSRYAFATAHIHGEIYRRRGLLTSEGKDIKNKEEILALLRALHLPSALSIIHCPGHQKGDSLEARGNRRADLAAREAALTTDTTSLLALEPTNDRPTPSWD	yes	MPRSEVKVTGPEGIPLTILTMSIEDEYRLHEKRTNSNNQETLDHWLAEFPQAWAETGGMGLAINQAPIIVTLKAAILPASVRQYPMPKEAREGIRPHIKRLLEQGILVPCKSPWNTPLLPVRKPGTNDYRPVQDLREVNKRIEDIHPTVPNPYNLLSGLPPNYTWYTVLDLKDAFFCLRLHPTSQPIFAFEWQDADLGISGQLTWTRLPQGFKNSPTLFDEALHQDLAGFRVRYPALILLQYVDDILLAAKTKEECKEGTRALLQTLGSLGYRASAKKAQICQKQVTYLGYKIKDGRRWLTEARMRAILDIPTPQNPRQLREFLGTAGFCRLWIPGFAEMAAPLYPLTRPGVAFKWEEPQKKAFTNIKKALLESPALGLPDLAKPFELFIDEKGGYAKGVLTQKLGPWRRPTAYLSKKLDPVASGWPPCLRMIAAIALLVKDSYKLTLGQPLTIHAPHAVEAVIRQPPDRWLTNARMTHYQTMLLDKDRVHFGPLVTLNPATLLPLPGEPEAHNCLQVLAEAHGARPDLTDQPLPSPDHIWFTDGSSFLHQGKRRAGAAVTTENQVVWAQALPPGTSAQRAELIALTQALKLAEGKRLTVYTDSRYAFATAHIHGEIYRRRGLLTSEGKDIKNKEEILALLRALHLPSALSIIHCPGHQKGDSLEARGNRRADLAAREAALTTDTTSLLALEPTNDRPTPSWD	/	ATGCCTAGATCTGAAGTTAAGGTTACAGGGCCGGAAGGAATCCCTTTAACTATCCTGACAATGTCTATTGAGGATGAATACCGCCTGCATGAAAAGAGGACCAACAGCAACAATCAGGAGACCCTCGACCACTGGCTTGCGGAGTTTCCTCAGGCGTGGGCAGAAACCGGCGGGATGGGTTTAGCCATCAATCAGGCGCCCATAATAGTTACTCTGAAGGCCGCCATTCTGCCCGCCTCTGTGAGACAATATCCTATGCCAAAAGAGGCGAGGGAAGGTATACGGCCACACATAAAGAGATTGCTGGAGCAAGGGATCCTGGTCCCGTGCAAAAGCCCGTGGAACACGCCGCTGCTGCCTGTGAGGAAACCAGGCACTAATGATTACCGCCCCGTGCAAGATTTACGAGAGGTGAATAAGAGGATCGAAGACATCCACCCAACCGTGCCAAATCCATACAACTTACTGTCTGGTCTGCCGCCTAACTACACATGGTACACGGTATTGGACTTGAAGGACGCCTTCTTCTGCCTTAGATTACACCCGACCTCTCAGCCGATTTTTGCATTCGAATGGCAGGACGCTGATCTCGGCATCTCCGGCCAGCTCACCTGGACCAGACTGCCCCAGGGTTTCAAAAACAGCCCTACACTGTTTGATGAGGCCCTTCATCAAGACCTCGCCGGGTTCCGCGTAAGATATCCCGCCCTGATTCTCCTGCAGTACGTTGATGATATCCTGTTGGCCGCGAAGACAAAGGAAGAGTGCAAGGAGGGCACAAGAGCCCTGTTACAGACGCTTGGCTCGCTGGGCTATAGGGCTTCCGCTAAGAAGGCTCAGATTTGTCAAAAGCAGGTGACGTATCTGGGATACAAAATCAAGGATGGCAGAAGATGGTTGACCGAAGCCAGAATGCGCGCGATCCTGGACATTCCAACCCCACAAAACCCTAGACAACTACGGGAATTTCTGGGCACAGCTGGCTTCTGTAGGCTGTGGATCCCTGGCTTCGCGGAGATGGCGGCCCCATTGTACCCTCTGACACGGCCTGGCGTCGCTTTCAAGTGGGAGGAACCACAGAAAAAAGCGTTCACTAATATTAAAAAGGCCCTGCTCGAGTCTCCCGCGCTGGGCTTACCTGACCTGGCAAAGCCTTTTGAGCTCTTCATCGACGAGAAGGGCGGCTACGCCAAGGGAGTCCTGACCCAGAAGCTGGGCCCATGGAGACGGCCCACCGCGTACCTCAGCAAAAAGCTCGATCCGGTGGCTAGCGGATGGCCACCTTGTCTAAGGATGATTGCGGCAATCGCCCTGCTGGTGAAAGATTCCTACAAACTCACCCTGGGCCAACCCTTAACTATACACGCCCCCCACGCCGTGGAGGCCGTTATCCGCCAGCCGCCCGATAGATGGTTAACCAATGCTCGCATGACCCACTACCAGACCATGTTACTGGACAAGGACCGGGTGCATTTTGGCCCCCTCGTGACACTCAACCCAGCGACCTTGTTGCCTCTCCCCGGAGAGCCGGAGGCTCACAACTGCCTGCAGGTGCTGGCCGAGGCGCACGGCGCTAGACCCGACCTGACTGATCAGCCACTGCCAAGCCCTGACCATATTTGGTTCACCGACGGGAGTAGCTTCCTCCACCAAGGAAAAAGACGGGCTGGTGCCGCTGTGACTACAGAGAACCAGGTGGTCTGGGCGCAGGCACTGCCACCCGGAACCAGCGCCCAAAGAGCTGAGCTCATCGCTCTGACGCAGGCCCTGAAGCTGGCCGAAGGCAAGCGCCTGACCGTCTATACTGACAGCCGGTATGCTTTCGCCACAGCACATATCCACGGTGAGATCTACAGAAGAAGGGGCTTACTGACTAGCGAAGGAAAGGATATCAAGAACAAGGAAGAAATTTTAGCCTTGCTCCGGGCGTTGCACCTGCCCTCTGCCCTCAGCATCATCCATTGCCCTGGCCACCAGAAAGGCGACTCCTTAGAGGCTCGTGGGAACCGGCGCGCCGACCTGGCTGCCAGAGAAGCAGCCTTGACAACTGACACAACCTCTCTGCTGGCCCTGGAGCCTACAAACGACCGACCTACGCCTTCATGGGAC
RT 45	486	RT_ZF REV_like	XP_041910541.1	459-1146	LOW QUALITY PROTEIN: uncharacterized protein LOC119808351	Arvicola amphibius	PGPQGQPLHVLTLNLEDEYRLHEPPRDPKTALETFWLSKFPQAWAETGGMGLATQQAPLIISLKATATPVSIKQYPMSLEAKTGIRPHIRRLLDQGILTPCQSPWNTPLLPVKKPGTGDYRPVQDLREVNKRVEDIHPTVPNPYNLLNGLSPSHNWYSVLDLKDAFFCLRLHPESQPIFAFEWKDPELGISGQLTWTRLPQGFKNSPTLFDEALHRDLADFRIQHPTLILLQYVDDLLLAATSEQECKEGTKALLLTLGNLGYKASAKKAQICQKQVTYLGYQIKEGQRWLTEARKATVMNMPVPGTPRQLREFLGTAGFCRLWIPGFAEIAAPLYPLTKSGTLFRWGEDQQKAYQEIKRALLTAPALGLPDLTKPFELFVDEKQGYAKGVLTQKLGPWRRPVAYLSKKLDPVASGWPPCLRMVAAIAVLTKDAGKLTLGQPLTILAPHAVEALVKQPPDRWLSNARMTHYQAMLLDTDRVHFGPVVTLNPATLLPLPEGGAKHDCLEILAEMHGTRPDLTDQPLQNADFTWYTDGSSFLQDGQRRAGAAVTTETEVIWAETLPTGTSAQRAELIALTQALRLAEGKKLNVYTDSRYAFATAHIHGEIYRRRGLLTSEGKEIKNRNEILALLKALFLPQRLSIIHCPGHQKGDSPKARGNRMADRAAREAAMGTEILKRLGASQDSVKG	yes	MPGPQGQPLHVLTLNLEDEYRLHEPPRDPKTALETFWLSKFPQAWAETGGMGLATQQAPLIISLKATATPVSIKQYPMSLEAKTGIRPHIRRLLDQGILTPCQSPWNTPLLPVKKPGTGDYRPVQDLREVNKRVEDIHPTVPNPYNLLNGLSPSHNWYSVLDLKDAFFCLRLHPESQPIFAFEWKDPELGISGQLTWTRLPQGFKNSPTLFDEALHRDLADFRIQHPTLILLQYVDDLLLAATSEQECKEGTKALLLTLGNLGYKASAKKAQICQKQVTYLGYQIKEGQRWLTEARKATVMNMPVPGTPRQLREFLGTAGFCRLWIPGFAEIAAPLYPLTKSGTLFRWGEDQQKAYQEIKRALLTAPALGLPDLTKPFELFVDEKQGYAKGVLTQKLGPWRRPVAYLSKKLDPVASGWPPCLRMVAAIAVLTKDAGKLTLGQPLTILAPHAVEALVKQPPDRWLSNARMTHYQAMLLDTDRVHFGPVVTLNPATLLPLPEGGAKHDCLEILAEMHGTRPDLTDQPLQNADFTWYTDGSSFLQDGQRRAGAAVTTETEVIWAETLPTGTSAQRAELIALTQALRLAEGKKLNVYTDSRYAFATAHIHGEIYRRRGLLTSEGKEIKNRNEILALLKALFLPQRLSIIHCPGHQKGDSPKARGNRMADRAAREAAMGTEILKRLGASQDSVKG	*	ATGCCTGGCCCGCAGGGACAGCCATTGCACGTCCTGACCCTGAACTTGGAAGATGAGTACAGACTGCATGAACCTCCACGCGACCCAAAAACCGCCCTGGAAACATTCTGGTTGAGCAAGTTCCCTCAGGCGTGGGCGGAAACTGGCGGCATGGGGCTTGCAACGCAGCAGGCTCCTCTCATCATCAGTTTGAAGGCGACCGCCACTCCTGTAAGCATAAAACAATACCCAATGTCACTGGAAGCCAAGACAGGAATCCGCCCCCACATTCGGAGACTGTTGGACCAGGGCATATTAACACCATGTCAAAGCCCCTGGAACACTCCTCTGCTGCCTGTAAAGAAACCTGGCACGGGCGACTACAGGCCGGTGCAGGACTTAAGAGAGGTGAACAAGAGGGTGGAGGACATCCACCCTACAGTCCCTAATCCTTACAACCTTCTGAATGGCCTCAGCCCCTCCCACAACTGGTATTCCGTGTTAGACCTCAAGGACGCCTTCTTCTGTCTGAGACTGCACCCTGAAAGCCAACCAATCTTTGCTTTCGAGTGGAAGGATCCAGAGTTGGGAATAAGCGGCCAGCTAACATGGACGAGACTGCCCCAGGGTTTCAAGAATTCACCCACGCTGTTTGATGAAGCCTTACACCGTGACCTGGCCGACTTCCGCATCCAGCACCCGACTCTCATCCTCCTGCAGTATGTTGATGATTTGCTGCTCGCCGCCACCTCGGAGCAGGAATGCAAGGAGGGCACCAAGGCCCTGCTGCTGACTCTGGGAAACCTAGGATACAAGGCTTCCGCAAAAAAGGCTCAGATCTGCCAGAAACAGGTGACATACCTTGGATATCAAATCAAGGAGGGGCAGAGATGGCTTACCGAAGCCAGGAAGGCTACAGTGATGAATATGCCAGTGCCCGGCACCCCGAGGCAGCTCAGAGAGTTCCTTGGCACCGCCGGTTTTTGTCGGCTCTGGATCCCGGGCTTCGCTGAGATCGCCGCCCCTCTGTACCCGCTAACCAAAAGCGGCACCCTGTTCCGGTGGGGTGAGGATCAGCAAAAGGCTTATCAGGAGATCAAGCGGGCCTTACTTACCGCGCCCGCGCTGGGCTTGCCCGACCTGACAAAGCCATTCGAGTTGTTCGTCGACGAGAAGCAAGGCTACGCTAAAGGCGTGCTGACCCAGAAGCTCGGCCCATGGAGACGGCCGGTCGCCTACCTAAGCAAGAAGCTGGACCCTGTCGCGAGCGGGTGGCCGCCGTGCCTGCGCATGGTGGCGGCCATCGCGGTGCTGACCAAGGATGCCGGAAAGCTGACCCTCGGTCAACCTCTCACCATCCTGGCGCCCCACGCGGTCGAGGCCCTAGTTAAGCAGCCGCCTGACAGATGGCTCTCCAACGCACGGATGACACACTACCAAGCAATGCTACTGGATACCGATCGAGTGCATTTTGGTCCCGTGGTGACATTAAACCCTGCCACTCTGCTGCCATTACCTGAAGGCGGTGCCAAGCATGACTGCTTGGAGATTCTGGCGGAGATGCATGGAACTCGGCCTGATCTGACTGATCAACCACTGCAGAATGCTGATTTCACCTGGTACACCGATGGTAGTTCTTTTTGCAGGACGGTCAGCGTCGAGCCGGGGCCGCCGTAACGACAGAGACCGAAGTCATTTGGGCCGAGACTCTGCCAACCGGAACAAGTGCGCAGCGTGCCGAGCTGATAGCTCTTACACAAGCGCTGCGACTGGCCGAAGGCAAGAAGTTGAACGTCTACACAGACAGCAGATACGCGTTCGCCACGGCGCACATTCACGGCGAGATATATCGAAGACGGGGCCTCTTGACCTCTGAAGGAAAGGAGATTAAGAACAGAAATGAAATCCTGGCCCTGCTGAAGGCGCTGTTCCTGCCTCAGAGACTGTCTATTATTCATTGCCCCGGCCACCAGAAAGGTGACTCCCCTAAAGCTAGAGGCAACCGCATGGCAGACCGCGCAGCACGGGAAGCGGCCATGGGCACTGAAATATTGAAAAGACTAGGCGCCAGCCAAGATTCTGTGAAGGGA
RT 46	487	RT_ZF REV_like	XP_038956947.1	631-1316	LOW QUALITY PROTEIN: uncharacterized protein LOC120099849	Rattus norvegicus	PPVACLVLSLEEEYRLHEQNPKQLLAPEWLSAFSEVWAEQTGMGLAKQVPPVVVELKADASPVSVKQYPMSREAKEGIRPHIQRLLQLGILVPCQSPWNTPLLPVRKPGTNDYRPVQDLREVNKRVQDIHPTVPNPYNLLSSLPPEWYTVLDLKDAFFCLRLHPNSQPLFAFEWRDPEGGHTGQLTWTRLPQGFKNSPTLFDEALHRDLAPFRAQNPQISLLQYVDDLLLAASTQELCREGTKRLLNELGELGYRVSAKKAQLCEVTYLGYTLREGKRWLTEARKKTVMQIPTPTTPRQVREFLGTAGFCRLWIPGFATLAAPLYLLTKEKVPFTWTEEHQRAFEDIKAALLAAPALALPDLTKPFTLYVDERAGVARGVLTQALGPWKRPVAYLSKKLDPVASGWPSCLKAIAAVALLVKDADKLTLGQHVTVIAPHALESIVRQPPDRWMTNARMTHYQSLLLNERVTFAPPAILNPATLLPEIHNSTPIHQCVDILAEETGTRKDLTDRPWPGVPAWYTDGSSFVVEGKRRAGAAVVDGKQVIWASSLPEGTSAQKAELVALTQALRLAEGKAINIYTDSRYAFATAHIHGAIYKQRGLLTSAGRDIKNKEEILALLEAVHLPKKVAIIHCPGHQKGEDPITKGNQMADLVAKQVAQQVTILAEKSQTPVKTPVTDDSNY	yes	MPPVACLVLSLEEEYRLHEQNPKQLLAPEWLSAFSEVWAEQTGMGLAKQVPPVVVELKADASPVSVKQYPMSREAKEGIRPHIQRLLQLGILVPCQSPWNTPLLPVRKPGTNDYRPVQDLREVNKRVQDIHPTVPNPYNLLSSLPPERTWYTVLDLKDAFFCLRLHPNSQPLFAFEWRDPEGGHTGQLTWTRLPQGFKNSPTLFDEALHRDLAPFRAQNPQISLLQYVDDLLLAASTQELCREGTKRLLNELGELGYRVSAKKAQLCRTEVTYLGYTLREGKRWLTEARKKTVMQIPTPTTPRQVREFLGTAGFCRLWIPGFATLAAPLYLLTKEKVPFTWTEEHQRAFEDIKAALLAAPALALPDLTKPFTLYVDERAGVARGVLTQALGPWKRPVAYLSKKLDPVASGWPSCLKAIAAVALLVKDADKLTLGQHVTVIAPHALESIVRQPPDRWMTNARMTHYQSLLLNERVTFAPPAILNPATLLPEIHNSTPIHQCVDILAEETGTRKDLTDRPWPGVPAWYTDGSSFVVEGKRRAGAAVVDGKQVIWASSLPEGTSAQKAELVALTQALRLAEGKAINIYTDSRYAFATAHIHGAIYKQRGLLTSAGRDIKNKEEILALLEAVHLPKKVAIIHCPGHQKGEDPITKGNQMADLVAKQVAQQVTILAEKSQTPVKTPVTDDSNY	***	ATGCCTCCCGTCGCGTGCCTCGTACTGAGCCTAGAGGAGGAGTACAGGCTGCATGAGCAAAATCCGAAGCAGCTGCTAGCCCCTGAATGGCTGAGCGCCTTCTCCGAGGTATGGGCGGAGCAGACTGGCATGGGACTGGCCAAGCAGGTGCCTCCTGTGGTGGTAGAGCTGAAGGCGGACGCCAGCCCAGTGAGCGTGAAACAATACCCGATGTCGAGGGAAGCAAAGGAAGGCATTAGACCCCACATCCAGAGACTGCTGCAATTGGGAATCCTGGTACCCTGCCAAAGCCCTTGGAACACGCCACTGCTGCCTGTGCGCAAACCAGGAACTAATGATTACAGACCTGTACAGGATCTAAGGGAAGTTAACAAGCGGGTGCAGGACATACATCCTACGGTCCCAAACCCCTACAACTTGCTGAGCTCTCTGCCCCCCGAGCGCACATGGTACACTGTGCTCGACTTAAAAGATGCGTTCTTCTGCCTTCGCCTCCACCCGAACTCACAGCCACTGTTCGCGTTCGAGTGGCGCGACCCTGAGGGCGGCCACACCGGGCAACTGACGTGGACACGGCTGCCACAGGGTTTCAAGAACAGCCCTACTCTATTTGATGAGGCCCTGCACCGTGATCTGGCCCCCTTCCGGGCGCAGAACCCTCAAATCAGTCTACTGCAGTATGTTGATGACCTGCTGCTAGCCGCTTCTACTCAGGAGCTCTGCCGGGAGGGCACAAAGCGGCTGTTAAATGAGCTGGGCGAGCTGGGATACCGCGTGTCTGCCAAGAAGGCACAATTGTGTCGGACAGAGGTGACATATCTCGGCTACACATTAAGAGAAGGTAAGAGATGGCTCACTGAAGCACGTAAGAAGACTGTCATGCAGATTCCTACCCCTACCACGCCCCGTCAGGTGCGGGAGTTCCTCGGCACCGCCGGCTTCTGTAGACTGTGGATTCCTGGATTTGCCACCCTGGCCGCCCCACTCTACCTGCTGACGAAGGAAAAGGTTCCTTTCACCTGGACTGAGGAACATCAGCGGGCTTTCGAAGACATCAAAGCCGCCCTGCTGGCGGCTCCAGCTCTGGCCCTCCCTGACCTCACCAAGCCCTTCACACTCTATGTGGACGAGAGAGCTGGAGTGGCCAGAGGTGTGCTGACACAGGCACTGGGGCCGTGGAAGAGACCCGTGGCGTACCTATCTAAAAAGCTCGACCCCGTGGCCTCTGGGTGGCCGAGCTGTCTAAAGGCCATCGCTGCTGTAGCTCTACTGGTCAAAGATGCTGACAAGCTGACCCTGGGCCAGCATGTCACCGTCATCGCCCCGCACGCCTTAGAATCCATAGTCCGGCAACCACCAGATCGGTGGATGACAAACGCTAGGATGACGCATTATCAGAGCTTGCTGTTAAACGAAAGAGTGACATTTGCGCCCCCTGCCATCCTTAACCCAGCGACGCTACTGCCGGAGATCCACAACTCAACCCCCATCCACCAGTGCGTCGACATTCTGGCTGAGGAGACCGGCACGCGCAAGGACCTCACGGACAGACCATGGCCTGGTGTGCCGGCATGGTACACCGACGGCAGCTCCTTTGTCGTGGAGGGAAAGCGACGCGCAGGCGCGGCTGTGGTGGACGGCAAGCAAGTCATCTGGGCATCCTCCCTGCCAGAAGGCACCAGCGCTCAGAAAGCGGAGTTGGTGGCCTTGACCCAGGCCCTCAGATTAGCTGAAGGGAAGGCTATTAATATCTACACCGATTCTAGATACGCCTTCGCCACGGCGCACATCCACGGCGCGATCTACAAGCAGCGGGGCCTGCTGACCAGTGCCGGGCGAGACATCAAGAATAAGGAAGAGATTCTTGCCCTGCTGGAGGCCGTCCACCTCCCCAAAAAGGTGGCCATTATCCACTGCCCCGGCCACCAGAAGGGAGAGGATCCAATTACAAAGGGGAACCAGATGGCCGACCTCGTGGCGAAGCAGGTCGCTCAGCAGGTTACCATCCTGGCAGAAAAATCGCAGACCCCTGTGAAGACACCTGTGACAGATGATTCTAACTAT
RT 47	497	RT_ZF REV_like	XP_044913633.1	461-1186	LOW QUALITY PROTEIN: uncharacterized protein LOC123385412, partial	Felis catus	PNRDGQPIQVLTVSLQDEHRLFETPVTTNLLEAWLQDFPQAWAETGGLGRAKCQAPIIIDLKPTAMPVSIRQYPMSKEAHMGIQPHITRFLELGVLRPCRSPWNTPLLPVKKPGTRDYRPVQDLREVNKMDIHPTVPNPYNLLSTLSPDWYTVLDLKDAFFCLPLAPQSQELFAFEWRDPKRGISGQLTWTRLPQGFKNSPTLFDEALHRDLTDFQHPEVTLLQYVDDLLLAAPTKEACILGTRHLLRELGEKGYRASAKKAQICQTKVTYLGYILSEGKRWLTPGRIETVARIPPPQNPREVREFLGTAGFCRLWIPGFAELAAPLYALTKESAPFTWQEKHQSAFEALKEALLSAPALGLPDTSKPFTLFIDEKQGIAKGVLTQKLGPWKRPVAYLSKKLDPVAAGWPPCLRIMAATAMLVKDSAKLTLGQPLTVITPHALEAVVRQPPDRWITNARLTHYQALLLDTDRIQFGPPVTLNPATLLPAPEDQQSAHDCRQVLAEIHGTREDLKDQELPDADHSWYTDGSSYIDSGTRRAGAAVVDGHHIIWAQSLPPGTSAQKAELIALTKALELSEGKKANIYTDSRYAFATAHTHGSIYERRGLLTSEGKEIKNKAEIIALLKALFLPQRVAIIHCPGHQKGQGPIATGNRQADQVARQVAAIGAVLNQDTKDWEKEGKIVLPRKEALAMIQQMHAWTHLSNQKLKSLIEKTDFLIPK	yes	MPNRDGQPIQVLTVSLQDEHRLFETPVTTNLLEAWLQDFPQAWAETGGLGRAKCQAPIIIDLKPTAMPVSIRQYPMSKEAHMGIQPHITRFLELGVLRPCRSPWNTPLLPVKKPGTRDYRPVQDLREVNKRTMDIHPTVPNPYNLLSTLSPDRTWYTVLDLKDAFFCLPLAPQSQELFAFEWRDPKRGISGQLTWTRLPQGFKNSPTLFDEALHRDLTDFRTQHPEVTLLQYVDDLLLAAPTKEACILGTRHLLRELGEKGYRASAKKAQICQTKVTYLGYILSEGKRWLTPGRIETVARIPPPQNPREVREFLGTAGFCRLWIPGFAELAAPLYALTKESAPFTWQEKHQSAFEALKEALLSAPALGLPDTSKPFTLFIDEKQGIAKGVLTQKLGPWKRPVAYLSKKLDPVAAGWPPCLRIMAATAMLVKDSAKLTLGQPLTVITPHALEAVVRQPPDRWITNARLTHYQALLLDTDRIQFGPPVTLNPATLLPAPEDQQSAHDCRQVLAEIHGTREDLKDQELPDADHSWYTDGSSYIDSGTRRAGAAVVDGHHIIWAQSLPPGTSAQKAELIALTKALELSEGKKANIYTDSRYAFATAHTHGSIYERRGLLTSEGKEIKNKAEIIALLKALFLPQRVAIIHCPGHQKGQGPIATGNRQADQVARQVAAIGAVLNQDTKDWEKEGKIVLPRKEALAMIQQMHAWTHLSNQKLKSLIEKTDFLIPK	**	ATGCCTAACAGGGATGGCCAACCAATTCAGGTGCTCACCGTGAGCCTGCAGGATGAACATAGACTTTTTGAAACACCGGTGACCACAAACCTGCTCGAAGCCTGGCTCCAAGACTTCCCACAAGCGTGGGCAGAGACCGGCGGACTAGGCAGGGCCAAATGTCAAGCACCCATCATCATTGATCTCAAACCTACGGCGATGCCAGTCTCCATACGCCAGTACCCGATGTCGAAGGAAGCGCACATGGGAATCCAGCCGCATATCACAAGATTTCTCGAATTAGGAGTCCTGCGTCCCTGTCGTAGCCCATGGAACACACCTCTGCTGCCTGTCAAGAAGCCAGGCACCAGGGACTACAGACCTGTTCAAGATCTGCGTGAGGTGAACAAGCGGACAATGGATATTCACCCAACAGTGCCGAACCCATACAATCTTCTGTCTACTCTGAGCCCAGACCGCACCTGGTACACAGTGCTGGACTTGAAGGACGCGTTCTTCTGTCTGCCTCTCGCCCCACAAAGCCAGGAACTCTTCGCTTTCGAGTGGAGGGACCCGAAGAGAGGGATAAGCGGCCAGCTCACATGGACACGGCTGCCCCAGGGATTCAAGAACTCCCCTACATTATTTGATGAGGCCCTACACCGCGACCTCACCGACTTTAGAACACAGCACCCGGAGGTTACTCTGTTGCAGTATGTGGATGATTTGCTCCTGGCCGCCCCTACCAAGGAGGCGTGCATTCTAGGCACCAGGCACCTGTTGAGAGAACTTGGTGAAAAGGGGTACCGCGCCTCAGCCAAGAAAGCGCAGATCTGCCAGACTAAGGTGACATACCTGGGCTACATCCTGTCTGAGGGAAAGAGATGGCTGACTCCCGGTCGGATCGAGACGGTGGCAAGAATCCCACCCCCTCAGAACCCTAGGGAAGTAAGGGAGTTCCTGGGCACCGCTGGATTCTGCAGACTCTGGATCCCTGGCTTTGCCGAGCTGGCAGCCCCCCTCTATGCCCTCACGAAAGAAAGTGCCCCGTTCACCTGGCAAGAGAAGCATCAAAGCGCATTTGAGGCTCTCAAGGAGGCTCTGTTATCCGCCCCTGCCCTGGGTCTGCCGGACACCTCTAAGCCGTTCACGTTGTTCATCGACGAGAAGCAGGGCATCGCTAAGGGAGTGCTCACTCAGAAATTAGGCCCGTGGAAGCGCCCAGTCGCCTACCTGAGCAAGAAACTCGACCCTGTGGCCGCCGGGTGGCCGCCGTGCTTGAGAATTATGGCCGCGACCGCCATGCTGGTTAAGGACTCCGCTAAGCTGACGCTGGGTCAGCCTCTGACTGTTATCACTCCCCACGCGTTAGAGGCGGTGGTGCGACAACCTCCAGACCGATGGATCACAAATGCTAGACTGACTCACTACCAAGCCCTGCTGCTCGATACAGATCGGATCCAATTTGGCCCTCCTGTCACCCTCAACCCGGCCACCCTGCTGCCTGCGCCTGAGGACCAGCAATCTGCTCATGATTGCCGCCAGGTGTTAGCGGAGATACATGGCACGAGGGAGGATCTGAAAGACCAGGAGCTGCCTGACGCCGACCACAGCTGGTACACGGACGGGAGCTCTTACATCGATTCTGGCACAAGACGAGCCGGGGCCGCCGTGGTGGACGGCCACCACATTATATGGGCACAGTCACTGCCCCCCGGAACCTCCGCCCAAAAAGCTGAGCTAATCGCCCTGACTAAGGCCCTGGAGCTAAGCGAGGGCAAAAAGGCAAATATCTATACTGACAGCAGATATGCTTTCGCCACCGCCCACACGCACGGTTCCATCTACGAGCGGCGGGGCCTGCTGACTTCAGAAGGCAAAGAGATCAAGAATAAGGCCGAGATCATCGCGCTGTTGAAGGCACTGTTCCTACCCCAGAGAGTGGCGATCATTCACTGCCCTGGCCACCAGAAGGGGCAGGGTCCTATTGCAACCGGCAACAGACAGGCAGACCAAGTCGCCAGACAGGTCGCGGCCATCGGCGCAGTTCTCAACCAGGATACAAAAGACTGGGAGAAAGAAGGCAAGATTGTGCTGCCCAGAAAGGAAGCCCTAGCAATGATTCAGCAGATGCATGCTTGGACGCACCTCTCTAATCAGAAATTGAAGAGCCTTATTGAAAAAACAGATTTCCTTATCCCGAAG

RT 48	508	RT_ZF REV_like	XP 06314129 9.1	646-1311	LOW QUALITY PROTEIN: uncharacterized protein LOC134486339	Rattus norvegicus	FNLADEYRIHEKGKKMDNPQWWLSRFPEAWAETGGVGMASQVPPIVIAVKSGATPISVRQYPMGKEAKEGIRPHINKFLQLGILTPCRSSWNTPLLPVKKPGTRDYRPVQDLREVNKRVEDVHPIVPNPYTLLSTLPPERIWYTVLDLKDAFFSMRLHPTSQHLFAFEWKDPETGHSGQLTWTRLPQGFKNSPTLFDEALHRDLMIFNNPQLTLLQYVDDLLLGAETQDECQKGTEQLLSELGRLGYRASAKKAQLCEVTYLGYILRDGKRWLTEARKKTVLQIPVPQTPRQVREFLGTAGFCRLWIPGFAALAAPLHTLTRLETEFQWKPEHQMAFDAIKRALLTAPALVLPDLTKPFMLFVDERAGVAKGVLTQTLGPWKRPVAYLSKKLDPVASGWPSCLKIIAAVATLIKDSDKLTLGQPVTIIAPHALESVIRQPPDRWITNARLTHYQSLLLNDRVSFGTPVILNPATLLPETTGQNPIHVCTDILAEASSTREDLTDVPLPGCPSWFTDGSNFLVEGKRKAGAAVVDGRTIWSSGLPEGTSAQKAELIALTQALRLAEGKNINIYTDSRYAFATAHVHGAIYRQRGLLTSTGKEIKNKDEILSLLEAIHLPRKVAVIHCPGHQGGQDAVAKGNRMADATAKQAALGAVILTVVNK-----------------------------------	yes	MFNLADEYRIHEKGKKMDNPQWWLSRFPEAWAETGGVGMASQVPPIVIAVKSGATPISVRQYPMGKEAKEGIRPHINKFLQLGILTPCRSSWNTPLLPVKKPGTRDYRPVQDLREVNKRVEDVHPIVPNPYTLLSTLPPERIWYTVLDLKDAFFSMRLHPTSQHLFAFEWKDPETGHSGQLTWTRLPQGFKNSPTLFDEALHRDLMIFNNPQLTLLQYVDDLLLGAETQDECQKGTEQLLSELGRLGYRASAKKAQLCEVTYLGYILRDGKRWLTEARKKTVLQIPVPQTPRQVREFLGTAGFCRLWIPGFAALAAPLHTLTRLETEFQWKPEHQMAFDAIKRALLTAPALVLPDLTKPFMLFVDERAGVAKGVLTQTLGPWKRPVAYLSKKLDPVASGWPSCLKIIAAVATLIKDSDKLTLGQPVTIIAPHALESVIRQPPDRWITNARLTHYQSLLLNDRVSFGTPVILNPATLLPETTGQNPIHVCTDILAEASSTREDLTDVPLPGCPSWFTDGSNFLVEGKRKAGAAVVDGRTIWSSGLPEGTSAQKAELIALTQALRLAEGKNINIYTDSRYAFATAHVHGAIYRQRGLLTSTGKEIKNKDEILSLLEAIHLPRKVAVIHCPGHQGGQDAVAKGNRMADATAKQAALGAVILTVVNK	/	ATGTTCAATCTCGCCGACGAATACCGTATTCATGAGAAGGGAAAGAAGATGGATAACCCACAGTGGTGGTTGAGTAGGTTCCCGGAGGCGTGGGCAGAAACGGGCGGCGTGGGTATGGCAAGCCAGGTGCCTCCAATTGTTATCGCTGTGAAGTCTGGCGCCACCCCGATCTCTGTCAGGCAGTATCCCATGGGGAAAGAGGCAAAAGAGGGGATACGACCTCATATTAACAAATTTCTTCAGCTGGGCATCTTGACCCCATGTAGAAGCAGCTGGAATACTCCCCTGCTTCCCGTGAAGAAACCTGGCACTAGAGACTACCGGCCCGTGCAAGATTTGAGAGAGGTGAACAAAAGAGTAGAAGATGTGCACCCTATCGTGCCGAACCCTTATACCCTGCTGAGCACGCTGCCTCCAGAGAGGATATGGTACACCGTCCTTGACCTCAAGGACGCCTTCTTCTCCATGAGGCTCCACCCTACCAGCCAGCACCTGTTTGCGTTTGAGTGGAAGGATCCAGAAACCGGCCACTCTGGACAGTTAACATGGACCAGACTGCCGCAGGGATTCAAGAATAGTCCGACGCTCTTTGATGAAGCTTTGCACCGCGACCTCATGATATTCAACAATCCTCAGCTGACGCTGCTTCAGTACGTGGACGATCTGCTGCTGGGCGCCGAAACCCAGGACGAGTGCCAAAAGGGCACAGAGCAGCTGCTGTCTGAGCTGGGTCGACTAGGGTACAGAGCTTCGGCCAAAAAAGCACAGCTTTGTGAGGTGACATATCTGGGCTACATCCTCAGAGATGGCAAGAGATGGTTAACGGAGGCCCGGAAGAAGACAGTGCTGCAAATCCCTGTCCCTCAGACACCGCGTCAGGTTAGAGAATTCCTCGGCACGGCCGGTTTCTGCCGCCTGTGGATCCCTGGCTTCGCGGCCCTGGCGGCGCCCCTCCACACATTAACACGGCTGGAAACAGAATTTCAATGGAAGCCAGAGCACCAGATGGCGTTCGACGCCATCAAGCGGGCCCTCCTCACGGCTCCGGCTTTGGTGCTGCCAGACCTGACCAAGCCGTTCATGTTGTTCGTCGACGAGAGAGCCGGCGTAGCCAAAGGAGTGCTGACACAGACACTAGGCCCATGGAAGCGCCCTGTGGCATACCTCAGCAAAAAGCTGGACCCCGTCGCGAGCGGCTGGCCATCGTGCCTCAAAATAATCGCCGCCGTGGCCACCCTCATCAAAGACTCCGACAAGCTCACCCTCGGCCAACCGGTCACTATCATCGCCCCCCATGCCCTCGAATCCGTGATCAGACAGCCTCCTGATCGGTGGATAACGAACGCGAGACTGACCCACTACCAATCCCTTCTGCTAAATGATAGAGTGTCCTTTGGGACGCCCGTAATTCTCAACCCCGCCACTCTGCTGCCGGAGACCACGGGCCAGAACCCTATCCATGTCTGCACCGACATTCTTGCCGAGGCCAGCTCAACAAGAGAGGACCTCACCGACGTACCTCTCCCCGGCTGCCCTTCATGGTTCACAGACGGAAGTAATTTCCTGGTGGAGGGGAAAAGGAAGGCGGGCGCCGCCGTGGTTGATGGTAGGACTATCTGGAGCAGCGGCCTGCCTGAAGGTACCAGCGCACAGAAGGCCGAGCTCATCGCTCTAACACAAGCATTACGGCTGGCAGAAGGAAAAAATATTAACATTTACACAGATTCTCGATATGCTTTCGCCACCGCGCACGTGCATGGGGCCATCTACAGGCAGCGAGGCTTACTGACTAGCACAGGCAAGGAAATAAAAAACAAGGATGAGATCTTATCCCTGCTAGAGGCCATCCACCTGCCTCGCAAGGTTGCCGTTATCCACTGCCCCGGTCACCAAGGCGGCCAGGATGCCGTGGCCAAGGGGAACAGAATGGCCGATGCCACCGCTAAGCAGGCTGCTTTGGGTGCCGTGATCCTCACCGTGGTAAACAAG
RT 49	558	RT_ZF REV_like	WP_2374 6085 0.1	2-313	retron St85 family RNA-directed DNA polymerase	Vibrio parahaemolyticus	NLVKQLAQHLGKSEYEVSLFLVDAPNKYRVYKIPKRTYGHRVIAQPSKELKKYQRAFLDIYPFPVHSAAMAYCEGKSIKDNAQAHVQNDYLLKTDLENFFNSITPTIFWSCIESCSLDTPSFTTNEKKLVESLLFWCPSKTKNGKLVLSVGAPSSPTISNFCLYEFDRYLSTICNFQNITYTRYADDLTFSTKKKDILYTVIPSLQDLLVKLFSQSIRLNHSKTVFSSRAHNRHVTGVTLNNDRRLSLGRQRKRYIKHLVNQFKYEQLDEVDTRHLQGLLAFAKHIEPTFIKRLKDKYSSELIKRIYEAGNE	yes	MNLVKQLAQHLGKSEYEVSLFLVDAPNKYRVYKIPKRTYGHRVIAQPSKELKKYQRAFLDIYPFPVHSAAMAYCEGKSIKDNAQAHVQNDYLLKTDLENFFNSITPTIFWSCIESCSLDTPSFTTNEKKLVESLLFWCPSKTKNGKLVLSVGAPSSPTISNFCLYEFDRYLSTICNFQNITYTRYADDLTFSTKKKDILYTVIPSLQDLLVKLFSQSIRLNHSKTVFSSRAHNRHVTGVTLNNDRRLSLGRQRKRYIKHLVNQFKYEQLDEVDTRHLQGLLAFAKHIEPTFIKRLKDKYSSELIKRIYEAGNE	*	ATGAACCTCGTCAAGCAGCTGGCGCAGCACCTCGGCAAGTCTGAGTACGAGGTGAGTTTGTTCCTCGTCGACGCGCCCAACAAGTACCGCGTGTACAAGATCCCCAAGAGGACATATGGCCACCGCGTCATCGCTCAACCATCAAAGGAGCTGAAAAAATACCAGCGGGCATTTCTTGACATTTACCCGTTCCCGGTGCACTCCGCCGCCATGGCGTACTGCGAGGGGAAGAGTATAAAAGATAATGCTCAAGCACATGTGCAGAATGATTATCTTCTCAAAACTGATTTAGAAAATTTCTTCAACTCGATAACTCCAACTATATTTTGGAGCTGTATTGAATCATGTAGTCTCGACACGCCTTCTTTTACAACAAATGAGAAGAAACTGGTGGAGAGCCTGCTCTTCTGGTGCCCTTCCAAAACAAAGAATGGAAAATTGGTTCTTTCAGTTGGTGCACCATCGTCGCCGACGATAAGCAACTTCTGCCTCTATGAGTTTGATAGATACCTCTCTACCATCTGCAACTTTCAAAATATCACCTACACACGGTATGCTGACGACCTCACCTTCTCCACCAAGAAGAAGGATATTCTGTACACTGTTATTCCTTCACTGCAGGATCTTTTGGTGAAGCTCTTTTCTCAATCTATTCGTCTTAACCATTCCAAGACGGTGTTCTCATCCCGCGCGCACAACAGACATGTCACCGGCGTCACTCTGAACAATGACCGCCGTCTCTCTTTGGGGAGGCAGAGGAAACGGTACATCAAGCATCTTGTAAACCAGTTCAAGTATGAGCAGCTTGATGAAGTTGACACAAGGCATCTGCAAGGTCTTCTCGCCTTCGCCAAGCACATCGAGCCGACGTTCATCAAACGGCTGAAGGACAAGTACAGCTCGGAACTGATCAAAAGAATTTATGAAGCTGGAAATGAA
RT 50	559	RT_ZF REV_like	WP_0756 8130 5.1	57-309	group II intron reverse transcriptase/maturase	Roseburia sp. 831b	TRKYKPQPVRRVEIPKPDGGVRNLGVPTVTDRFIQQAIAQVLTPIYEEQFHDHSYGFRPNRCAQQAILTALDMMNEGNDWIVDIDLEKFFDTVNHDKLMTIIGRTIKDGDVISIVRKYLVSGIMIDDEYEDSIVGTPQGGNLSPLLANIMLNELDKEMEKRGLNFVRYADDCIIMVGSEMSANRVMRNISRFIKEKLGLKVNMTKSKVDKPQGLKYLGFGFYFDSRAHQFKAKPHAKSVAKFKKRMKELTCRS	yes	MTRKYKPQPVRRVEIPKPDGGVRNLGVPTVTDRFIQQAIAQVLTPIYEEQFHDHSYGFRPNRCAQQAILTALDMMNEGNDWIVDIDLEKFFDTVNHDKLMTIIGRTIKDGDVISIVRKYLVSGIMIDDEYEDSIVGTPQGGNLSPLLANIMLNELDKEMEKRGLNFVRYADDCIIMVGSEMSANRVMRNISRFIKEKLGLKVNMTKSKVDKPQGLKYLGFGFYFDSRAHQFKAKPHAKSVAKFKKRMKELTCRS	*	ATGACAAGGAAATACAAGCCGCAACCTGTGCGCCGGGTGGAGATTCCCAAGCCTGATGGTGGCGTAAGAAATTTAGGTGTTCCTACGGTGACTGATAGATTTATTCAGCAAGCTATTGCTCAAGTGCTGACACCAATATATGAGGAGCAGTTCCACGACCACTCATATGGTTTTCGTCCCAACCGTTGTGCGCAGCAGGCCATCCTCACTGCGCTCGACATGATGAATGAAGGAAATGATTGGATCGTGGACATCGACCTTGAAAAATTCTTCGACACGGTCAATCATGATAAGCTGATGACAATAATTGGCCGCACCATCAAGGACGGCGACGTCATCTCCATCGTCAGGAAGTACCTGGTATCCGGGATAATGATTGATGATGAGTACGAGGATTCTATTGTTGGAACGCCGCAGGGCGGCAACTTGTCGCCGCTTCTTGCAAATATTATGCTGAATGAACTGGACAAGGAAATGGAGAAGCGGGGCCTCAACTTCGTCCGCTACGCCGACGACTGCATCATCATGGTTGGATCAGAGATGAGCGCCAACAGAGTGATGAGGAACATTTCTCGCTTCATCAAAGAAAAGCTCGGTCTCAAGGTGAACATGACCAAGAGTAAGGTTGATAAACCACAAGGGTTGAAATATCTTGGATTTGGCTTCTACTTTGATTCGCGGGCGCACCAGTTTAAAGCAAAGCCACATGCTAAATCAGTCGCCAAGTTCAAGAAGAGGATGAAGGAGTTGACCTGCCGAAGC
RT 51	562	RT_ZF REV_like	AGP 2548 0.1	114-806	pol protein, partial	Mus caroli	PTPIACLVLNLEEEYRLHEQGPKQLPDPEWLTAFSGVWAEQAGMGLAKQVPPVVVELKADASPVSVRQYPMSREAKEGIRPHIQRLIEQGILVPCQSPWNTPLLPVRKPGTNDYRPVQDLREVNKRVQDIHPTVPNPYNLLSSLPPEWYTVLDLKDAFFCLRLHPNSQPLFAFEWRDPEGGHTGQLTWTRLPQGFKNSPTLFDEALHRDLAPFRAQNPQISLLQYVDDLLVAASTRELCLDGTEKLLNELGELGYRVSAKKAQLCEVTYLGYTLREGKRWLTEARKKTVMLIPTPTTPRQVREFLGTAGFCRLWIPGFATLAAPLYPLTKEKVPFTWTEEHQKAFDSIKAALLAAPALALPDLTKSFTLYVDERAGVARGVLTQTLGPWKRPVAYLSKKLDPVASGWPSCLKAIAAVALLVKDADKLTLGQHVTVIAPHALESIVRQPPDRWTTNARMTHYQSLLLNDRVTFAPPAILNPATLLPLVDDSVPVHRCADVLAEEAGTRKDLIDQPWPGVPNWYTDGSSFVVEGKRRAGAAVVDGKKVIWASSLPEGTSAQKAELLALTQALQMAEGKAINIYTDSRYAFATAHIHGAIYKQRGLLTSAGKDIKNKEEILALLEAIHLPKKVAIIHCPGHQKGQDPVAQGNQMADIAAKQAAQGVMALAGKSGPSSGPSPVTHPDCIFCQKE	yes	MPTPIACLVLNLEEEYRLHEQGPKQLPDPEWLTAFSGVWAEQAGMGLAKQVPPVVVELKADASPVSVRQYPMSREAKEGIRPHIQRLIEQGILVPCQSPWNTPLLPVRKPGTNDYRPVQDLREVNKRVQDIHPTVPNPYNLLSSLPPERTWYTVLDLKDAFFCLRLHPNSQPLFAFEWRDPEGGHTGQLTWTRLPQGFKNSPTLFDEALHRDLAPFRAQNPQISLLQYVDDLLVAASTRELCLDGTEKLLNELGELGYRVSAKKAQLCRTEVTYLGYTLREGKRWLTEARKKTVMLIPTPTTPRQVREFLGTAGFCRLWIPGFATLAAPLYPLTKEKVPFTWTEEHQKAFDSIKAALLAAPALALPDLTKSFTLYVDERAGVARGVLTQTLGPWKRPVAYLSKKLDPVASGWPSCLKAIAAVALLVKDADKLTLGQHVTVIAPHALESIVRQPPDRWTTNARMTHYQSLLLNDRVTFAPPAILNPATLLPLVDDSVPVHRCADVLAEEAGTRKDLIDQPWPGVPNWYTDGSSFVVEGKRRAGAAVVDGKKVIWASSLPEGTSAQKAELLALTQALQMAEGKAINIYTDSRYAFATAHIHGAIYKQRGLLTSAGKDIKNKEEILALLEAIHLPKKVAIIHCPGHQKGQDPVAQGNQMADIAAKQAAQGVMALAGKSGPSSGPSPVTHPDCIFCQKE	**	ATGCCTACACCCATCGCTTGCCTGGTACTCAACCTCGAGGAGGAGTATCGGCTTCATGAACAAGGCCCTAAACAGCTGCCAGACCCTGAATGGCTGACCGCCTTTTCCGGCGTATGGGCCGAGCAGGCGGGCATGGGACTGGCAAAGCAGGTACCTCCTGTAGTCGTGGAGCTGAAGGCTGACGCCAGCCCCGTCTCCGTGCGTCAGTACCCCATGTCAAGAGAAGCTAAGGAAGGCATCAGACCACACATCCAGAGACTGATTGAACAGGGCATCCTAGTCCCGTGCCAGTCGCCTTGGAACACTCCACTGTTACCTGTGAGAAAGCCGGGCACCAACGACTACAGACCCGTGCAAGACCTGCGGGAGGTCAACAAGAGAGTGCAGGACATCCATCCCACCGTGCCAAATCCGTATAACCTTCTGAGCAGCCTTCCTCCTGAGAGAACGTGGTACACTGTGCTCGATTTAAAAGACGCGTTCTTCTGTCTGCGTCTTCACCCGAACAGCCAGCCTCTCTTCGCATTTGAGTGGCGTGACCCGGAGGGGGGCCACACCGGCCAGCTGACCTGGACACGGCTGCCGCAAGGATTCAAGAACTCACCTACCCTGTTTGACGAAGCGCTGCACCGCGACCTGGCACCCTTCAGAGCTCAGAACCCACAAATCAGCTTACTCCAATATGTGGATGATTTACTGGTGGCAGCCTCCACAAGAGAACTGTGCTTGGATGGAACTGAAAAACTGCTTAATGAACTTGGTGAACTGGGCTACCGTGTGAGCGCCAAGAAGGCTCAGCTCTGCCGGACAGAGGTGACATACCTGGGATACACCTTGAGAGAGGGAAAGAGGTGGCTCACTGAAGCAAGAAAGAAAACTGTGATGCTCATTCCAACACCTACGACCCCCAGACAGGTGAGAGAGTTCCTGGGCACAGCAGGGTTCTGCAGGCTGTGGATCCCAGGCTTCGCAACCCTGGCGGCTCCACTCTACCCTCTGACCAAGGAGAAGGTTCCGTTCACTTGGACAGAGGAGCATCAGAAGGCTTTCGATTCTATCAAGGCCGCCCTCTTAGCTGCCCCTGCCCTCGCGTTACCTGACCTCACCAAGAGCTTCACCCTATACGTGGACGAGAGGGCCGGCGTAGCCCGCGGCGTGCTGACACAAACACTGGGCCCGTGGAAAAGGCCCGTCGCGTACTTAAGCAAGAAATTGGATCCAGTGGCCAGCGGCTGGCCGTCCTGTCTGAAGGCTATTGCTGCGGTCGCCCTGCTGGTGAAGGATGCTGACAAGTTGACCTTGGGCCAGCACGTCACTGTCATCGCGCCCCATGCCTTGGAGTCTATCGTCCGGCAGCCGCCTGACCGCTGGACCACCAACGCCCGGATGACGCACTACCAGTCCCTGCTGCTGAATGATCGGGTTACATTTGCCCCTCCTGCTATCCTCAACCCTGCCACCCTGCTGCCTCTTGTCGATGATTCCGTGCCTGTTCACAGATGCGCCGATGTCCTGGCAGAAGAGGCCGGCACCAGGAAGGACCTGATCGACCAGCCATGGCCTGGCGTGCCCAACTGGTATACTGATGGATCCTCGTTTGTGGTGGAAGGGAAGCGGCGTGCAGGCGCCGCCGTGGTGGACGGCAAAAAGGTGATATGGGCCTCCTCACTGCCAGAGGGCACCAGTGCTCAGAAGGCAGAGCTGCTGGCCCTAACCCAGGCACTCCAGATGGCTGAAGGTAAGGCCATCAATATATACACCGACAGCAGATATGCCTTCGCCACCGCGCACATCCATGGAGCCATCTACAAGCAGCGCGGCTTGCTCACAAGTGCCGGCAAGGATATTAAAAATAAAGAAGAGATCCTCGCCCTTCTGGAGGCGATCCACCTCCCAAAAAAGGTGGCTATAATTCACTGCCCCGGGCACCAGAAAGGACAAGATCCTGTTGCCCAGGGCAACCAAATGGCAGATATTGCTGCTAAGCAAGCAGCACAAGGCGTCATGGCGTTGGCCGGCAAAAGCGGCCCTAGCTCCGGTCCGTCACCTGTGACTCACCCGGACTGCATCTTTTGTCAAAAGGAA
RT 52	563	RT_ZF REV_like	WP_3258 4058 0.1	2-394	MULTISPECIES: reverse transcriptase family protein	Enterobacter	DKLKPYSKSKAPITSLSKLAQTLSVSVDQLNEIAGLPDEEKYVLKELPKADGSKRIVYSLHPKMRLLQSRVNKRIFKELVVFPCFLYGSVPGKNDGLNSHIKRDYVSCAKAHCGAKTVLKVDISNFFDNIHKDLVSDVLENVLNIKDEALKYLTTLCCKGDFIVQGALTSSYIATLCLYEREGDIVQRALRKNLVYTRLVDDITVSSKINDYDFSQILNHIENMLSEHDLPINKRKTKIFHCSSEPIKIHGLRIDYKSPRLPSDEVKRIRASLHNLKQLSIKNNTKTSIAYRKEFNRCMGRVHKLGRVGHEKYEQFKQQLLAIKPMPSKRDVAVVEAAVNSLEISFFNGNNGKHWYKRKYNLTLYKMIIFTRSDAFDEVVEGFRVRLSKVRPI	yes	MDKLKPYSKSKAPITSLSKLAQTLSVSVDQLNEIAGLPDEEKYVLKELPKADGSKRIVYSLHPKMRLLQSRVNKRIFKELVVFPCFLYGSVPGKNDGLNSHIKRDYVSCAKAHCGAKTVLKVDISNFFDNIHKDLVSDVLENVLNIKDEALKYLTTLCCKGDFIVQGALTSSYIATLCLYEREGDIVQRALRKNLVYTRLVDDITVSSKINDYDFSQILNHIENMLSEHDLPINKRKTKIFHCSSEPIKIHGLRIDYKSPRLPSDEVKRIRASLHNLKQLSIKNNTKTSIAYRKEFNRCMGRVHKLGRVGHEKYEQFKQQLLAIKPMPSKRDVAVVEAAVNSLEISFFNGNNGKHWYKRKYNLTLYKMIIFTRSDAFDEVVEGFRVRLSKVRPI	/	ATGGACAAGCTGAAACCATATAGCAAGAGCAAAGCTCCTATAACTTCGTTGTCAAAGCTTGCTCAGACGCTGTCTGTATCAGTAGACCAGCTGAATGAGATTGCAGGGCTCCCTGATGAGGAGAAGTATGTGTTGAAGGAGTTGCCCAAGGCCGACGGCTCAAAAAGGATCGTCTACTCCCTTCATCCAAAAATGAGGTTATTGCAAAGTCGTGTCAATAAGCGCATCTTCAAGGAACTCGTGGTGTTCCCGTGCTTCCTCTATGGTTCTGTGCCGGGAAAAAATGACGGTCTTAATTCTCATATAAAAAGAGACTATGTTTCTTGTGCCAAGGCTCATTGTGGAGCAAAAACTGTTCTGAAGGTTGATATCAGCAACTTCTTCGACAACATCCACAAGGATTTAGTTTCTGATGTACTTGAAAATGTTTTGAATATAAAGGACGAGGCCCTTAAGTACCTCACCACCCTGTGCTGCAAGGGCGACTTCATCGTGCAGGGTGCGCTAACAAGCTCCTACATCGCGACGCTCTGTCTGTACGAGAGGGAGGGGGATATTGTTCAACGCGCGCTGAGAAAGAATCTTGTGTACACGAGATTGGTCGACGACATCACCGTCTCCTCCAAGATAAATGATTATGATTTTTCACAGATACTCAACCACATTGAGAACATGCTTTCCGAGCATGATCTCCCGATCAACAAGAGGAAGACGAAAATTTTCCACTGCTCCTCTGAGCCCATCAAGATTCATGGTCTACGTATTGATTACAAATCGCCGCGCCTCCCTAGTGATGAGGTGAAGAGGATACGGGCATCGCTCCACAACCTCAAGCAACTGTCTATAAAGAACAATACAAAGACATCCATCGCCTACAGAAAAGAGTTCAACCGGTGCATGGGGCGAGTCCACAAACTAGGACGCGTCGGCCATGAAAAATATGAACAATTTAAGCAGCAGCTCCTCGCCATCAAACCCATGCCAAGTAAGCGGGACGTCGCTGTCGTGGAGGCGGCGGTGAATTCATTGGAAATTTCCTTTTTTAATGGCAACAACGGCAAGCACTGGTACAAGCGAAAGTACAACCTGACCCTCTACAAGATGATCATTTTTACTCGCAGCGATGCTTTTGATGAAGTTGTTGAAGGCTTCCGCGTGAGATTAAGCAAGGTGCGGCCCATT

RT53	564	RT_ZFREV_like	QDA02050.1	74-786	pol protein	Flying-fox retrovirus	PEEDRATLCLVLGLEEEYRLHEKPVPSSVDPSWLQLFPDVWAEKGGMGLANRVPPIVVELKSDALPVAVRQYPMSREAREGIRPHIQRFLDLGVLVPCQSPWNTPLLPVKKPGTSDYRPVQDLREINKRVQDIHPTVPNPYNLLSSLPPNHTWYSVLDLKDAFFCLKLHPNSQLLFAFEWRDPEKGHTGQLTWTRLPQGFKNSPTLFDEALHRDLASFRASNPQVVLLQYVDDLLVAAPTYKDCKEGTQKLLQELSELGYRVSAKKAQLCQREVTYLGYLLKEGKRWLTPARKATVMEIPTPTTPRQVREFLGTAGFCRLWIPGFASLAAPLYPLTKESTPFLWTEEHRRAFDQIKEALLTAPALALPDLTKPFALYVDERAGVARGVLTQTLGPWRRPVAYLSKKLDPVASGWPTCLKAVAAVALLLKDADKLTLGQSVTVIASHSLESIVRQPPDRWMTNARMTHYQSLLLNERVSFAPPAVLNPATLLPAESGAAPVHECSEILAEETGTRQDLTDQPLPGVPAWYTDGSSFITEGKRRAGAAIVDGKVWMSSLPEGTSAQKAELIALTQALRLADGKDINIYTDSRYAFATAHIHGAIYRQRGLLTSAGKEIKNKEEILALLEAIHLPKRVAIIHCPGHQKGNDPVAIGNRRADEAAKQAALAVRVLAETIEPQGQLGSTQDRPGELTPDQGKNFIRRVHQLTHLG	yes	MPEEDRATLCLVLGLEEEYRLHEKPVPSSVDPSWLQLFPDVWAEKGGMGLANRVPPIVVELKSDALPVAVRQYPMSREAREGIRPHIQRFLDLGVLVPCQSPWNTPLLPVKKPGTSDYRPVQDLREINKRVQDIHPTVPNPYNLLSSLPPNHTWYSVLDLKDAFFCLKLHPNSQLLFAFEWRDPEKGHTGQLTWTRLPQGFKNSPTLFDEALHRDLASFRASNPQVVLLQYVDDLLVAAPTYKDCKEGTQKLLQELSELGYRVSAKKAQLCQREVTYLGYLLKEGKRWLTPARKATVMEIPTPTTPRQVREFLGTAGFCRLWIPGFASLAAPLYPLTKESTPFLWTEEHRRAFDQIKEALLTAPALALPDLTKPFALYVDERAGVARGVLTQTLGPWRRPVAYLSKKLDPVASGWPTCLKAVAAVALLLKDADKLTLGQSVTVIASHSLESIVRQPPDRWMTNARMTHYQSLLLNERVSFAPPAVLNPATLLPAESGAAPVHECSEILAEETGTRQDLTDQPLPGVPAWYTDGSSFITEGKRRAGAAIVDGKRTVWMSSLPEGTSAQKAELIALTQALRLADGKDINIYTDSRYAFATAHIHGAIYRQRGLLTSAGKEIKNKEEILALLEAIHLPKRVAIIHCPGHQKGNDPVAIGNRRADEAAKQAALAVRVLAETIEPQGQLGSTQDRTRPGELTPDQGKNFIRRVHQLTHLG	**	ATGCCTGAGGAAGACCGCGCTACATTGTGTCTTGTGCTTGGCCTGGAGGAGGAGTACCGCCTACATGAGAAGCCTGTGCCTTCCTCCGTCGACCCCTCTTGGCTCCAGCTGTTCCCGGACGTTTGGGCGGAGAAAGGCGGCATGGGGCTAGCCAACCGGGTCCCCCCTATCGTAGTGGAGCTAAAGAGTGATGCCCTGCCAGTCGCCGTAAGGCAGTACCCGATGTCGCGAGAAGCTAGAGAAGGTATCCGGCCGCACATTCAGCGCTTCCTGGACCTTGGTGTTCTAGTGCCATGCCAGAGTCCGTGGAACACACCACTGCTTCCTGTGAAGAAGCCCGGCACCTCTGACTACCGCCCCGTGCAGGATCTGAGAGAAATTAATAAGAGAGTTCAAGATATACACCCAACAGTGCCCAACCCATACAACTTGCTGTCCAGCCTGCCCCCAAATCATACGTGGTATAGCGTTCTAGATCTCAAAGACGCGTTCTTCTGCCTCAAACTGCATCCGAACTCCCAGCTCCTCTTCGCCTTTGAGTGGAGGGATCCGGAGAAAGGACACACCGGCCAACTAACATGGACACGGCTGCCGCAGGGCTTCAAGAACTCTCCTACTCTCTTCGATGAGGCACTCCACAGAGACCTGGCCTCCTTCCGCGCCAGCAACCCCCAGGTGGTGCTGCTGCAATATGTGGACGACCTGCTGGTGGCGGCCCCTACCTACAAGGACTGCAAGGAGGGAACTCAAAAACTACTCCAGGAGCTCTCGGAATTAGGTTACAGGGTCAGCGCGAAGAAGGCGCAGCTTTGTCAGAGAGAGGTAACGTACCTCGGATACCTGCTAAAGGAGGGCAAGCGGTGGCTGACCCCTGCTCGTAAGGCCACGGTGATGGAGATCCCCACCCCCACCACGCCTCGCCAGGTGCGTGAGTTCCTTGGTACTGCCGGTTTCTGTAGACTCTGGATTCCTGGTTTTGCTAGTCTGGCCGCCCCGCTGTATCCACTCACCAAGGAAAGCACTCCCTTCCTCTGGACAGAGGAACACCGCCGCGCATTCGACCAAATCAAGGAGGCCCTGCTCACGGCCCCTGCCCTCGCCCTCCCAGACCTGACTAAGCCGTTTGCCCTCTATGTGGATGAACGGGCCGGCGTGGCGAGAGGCGTGTTAACGCAGACACTGGGGCCTTGGCGGCGGCCTGTGGCCTACCTGTCAAAGAAGCTGGACCCCGTGGCTTCCGGCTGGCCCACGTGCCTCAAGGCAGTTGCTGCCGTCGCGCTGTTACTCAAGGATGCAGATAAGCTGACCCTCGGACAATCAGTGACAGTCATCGCTAGCCACAGCCTAGAGTCTATCGTGAGACAGCCGCCAGACAGGTGGATGACGAACGCCAGGATGACACACTACCAGTCGCTGCTGCTTAATGAACGTGTGAGCTTTGCCCCCCCGGCCGTACTGAACCCTGCCACCTTGCTGCCAGCAGAGTCTGGCGCGGCGCCTGTTCACGAATGCTCTGAAATTCTGGCTGAGGAGACCGGCACAAGACAGGACCTCACCGATCAACCTCTGCCGGGAGTGCCCGCGTGGTACACCGACGGCTCTAGCTTCATCACTGAAGGCAAAAGAAGGGCCGGCGCGGCCATTGTGGATGGAAAGCGGACGGTATGGATGTCCTCCCTGCCTGAAGGAACGAGCGCACAAAAGGCTGAACTGATCGCACTGACACAGGCTTTGCGCCTGGCCGACGGAAGGATATCAATATTTACACTGATTCTAGATACGCGTTCGCCACGGCCCATATCCATGGCGCCATATATCGGCAGCGGGGTCTGCTTACCTCCGCCGGCAAGGAAATCAAAAATAAAGAAGAGATTCTCGCACTGCTGGAGGCTATCCACCTGCCTAAAAGAGTAGCCATCATTCACTGCCCTGGCCACCAGAAAGGCAATGATCCAGTGGCTATCGGAAACCGGCGGGCTGACGAGGCCGCCAAGCAGGCCGCGCTAGCCGTGAGGGTGCTCGCAGAGACTATCGAACCCCAGGGACAGCTGGGCTCGACCCAAGATAGAACGCGGCCAGGTGAATTAACCCCTGATCAGGGAAAGAATTTTATCAGGAGAGTGCATCAGCTGACGCACCTCGGC
RT54	565	RT_ZFREV_like	YP_009513211.1	76-785	pol protein	Koala retrovirus	PRPAMCLVLNLEEEYRLHEKPVPPSIDPSWLQLFPMVWAEKAGMGLANQVPPVVVELKSDASPVAVRQYPMSKEAREGIRPHIQRFLDLGILVPCQSPWNTPLLPVKKPGTNDYRPVQDLREVNKRVQDIHPTVPNPYNLLSSLPPSHTWYSVLDLKDAFFCLKLHPNSQPLFAFEWRDPEKGNTGQLTWTRLPQGFKNSPTLFDEALHRDLASFRALNPQVVMLQYVDDLLVAAPTYRDCKEGTRRLLQELSKLGYRVSAKKAQLCREEVTYLGYLLKGGKRWLTPARKATVMKIPTPTTPRQVREFLGTAGFCRLWIPGFASLAAPLYPLTREKVPFTWTEAHQEAFGRIKEALLSAPALALPDLTKPFALYVDEKEGVARGVLTQTLGPWRRPVAYLSKKLDPVASGWPTCLKAIAAVALLLKDADKLTLGQNVLVIAPHNLESIVRQPPDRWMTNARMTHYQSLLLNERVSFAPPAILNPATLLPVESDDTPIHICSEILAEETGTRPDLRDQPLPGVPAWYTDGSSFIMDGRRQAGAAIVDNKVWASNLPEGTSAQKAELIALTQALRLAEGKSINIYTDSRYAFATAHVHGAIYKQRGLLTSAGKDIKNKEEILALLEAIHLPKRVAIIHCPGHQRGTDPVATGNRKADEAAKQAAQSTRILTETTKNQEHFEPTRGKIKPRELTPDQGREFIQRLHQLTHLG	yes	MPRPAMCLVLNLEEEYRLHEKPVPPSIDPSWLQLFPMVWAEKAGMGLANQVPPVVVELKSDASPVAVRQYPMSKEAREGIRPHIQRFLDLGILVPCQSPWNTPLLPVKKPGTNDYRPVQDLREVNKRVQDIHPTVPNPYNLLSSLPPSHTWYSVLDLKDAFFCLKLHPNSQPLFAFEWRDPEKGNTGQLTWTRLPQGFKNSPTLFDEALHRDLASFRALNPQVVMLQYVDDLLVAAPTYRDCKEGTRRLLQELSKLGYRVSAKKAQLCREEVTYLGYLLKGGKRWLTPARKATVMKIPTPTTPRQVREFLGTAGFCRLWIPGFASLAAPLYPLTREKVPFTWTEAHQEAFGRIKEALLSAPALALPDLTKPFALYVDEKEGVARGVLTQTLGPWRRPVAYLSKKLDPVASGWPTCLKAIAAVALLLKDADKLTLGQNVLVIAPHNLESIVRQPPDRWMTNARMTHYQSLLLNERVSFAPPAILNPATLLPVESDDTPIHICSEILAEETGTRPDLRDQPLPGVPAWYTDGSSFIMDGRRQAGAAIVDNKRTVWASNLPEGTSAQKAELIALTQALRLAEGKSINIYTDSRYAFATAHVHGAIYKQRGLLTSAGKDIKNKEEILALLEAIHLPKRVAIIHCPGHQRGTDPVATGNRKADEAAKQAAQSTRILTETTKNQEHFEPTRGKIKPRELTPDQGREFIQRLHQLTHLG	*	ATGCCTCGGCCGGCGATGTGCTTGGTGCTCAACCTCGAGGAAGAATACAGGCTCCATGAGAAACCAGTGCCCCCCAGCATAGATCCTTCGTGGCTTCAGCTGTTCCCGATGGTGTGGGCGGAGAAGGCTGGGATGGGGCTGGCCAACCAGGTTCCTCCTGTGGTGGTCGAACTCAAGTCAGACGCCAGCCCGGTTGCCGTGAGGCAGTACCCGATGTCAAAGGAGGCAAGAGAGGGTATTCGCCCTCACATCCAGAGATTTCTGGATTTAGGCATCTTGGTCCCATGTCAGAGCCCATGGAACACCCCTCTTCTTCCCGTGAAAAAGCCCGGCACAAATGATTACAGACCAGTACAAGATCTTAGAGAAGTCAATAAGCGTGTGCAGGACATTCACCCGACAGTGCCTAACCCATACAATTTGCTGTCGAGCCTACCGCCTAGCCACACCTGGTATTCAGTGCTAGATTTAAAGGACGCATTCTTCTGTCTGAAGCTCCATCCTAACTCTCAACCCCTTTTCGCCTTCGAATGGAGAGATCCTGAAAAGGGCAACACTGGGCAGCTCACATGGACGAGACTGCCACAGGGATTCAAGAATAGCCCTACCCTTTTTGATGAGGCTCTGCACAGGGACCTAGCCAGCTTCCGCGCCCTGAACCCTCAGGTCGTGATGCTTCAATATGTTGACGACCTACTGGTCGCGGCTCCAACATATCGCGACTGCAAAGAGGGCACACGTAGGCTGTTACAGGAGCTGTCTAAGCTGGGCTACCGCGTGAGCGCTAAAAAGGCTCAGCTTTGCCGGGAGGAGGTAACTTATCTGGGCTACTTGCTGAAAGGTGGCAAAAGATGGCTCACACCAGCTCGGAAGGCGACGGTCATGAAAATCCCTACCCCCACCACTCCTCGTCAGGTTCGGGAGTTCCTCGGCACGGCGGGCTTCTGCAGGTTATGGATCCCTGGATTCGCCAGCTTAGCTGCCCCTCTCTACCCGCTGACCAGAGAGAAGGTGCCTTTCACATGGACAGAGGCCCACCAAGAGGCATTCGGCAGGATAAAGGAGGCGCTGTTGAGCGCTCCGGCCTTAGCTCTGCCGGACCTGACTAAGCCGTTCGCCCTGTACGTTGATGAAAAGGAAGGAGTGGCGCGCGGGGTGCTGACACAGACTCTCGGCCCGTGGAGACGCCCTGTCGCGTACCTTAGCAAAAAACTAGACCCGGTGGCTTCTGGGTGGCCGACTTGCCTCAAAGCAATCGCCGCCGTCGCCCTCCTGCTGAAGGACGCCGACAAGTTAACTCTGGGTCAGAATGTACTCGTGATCGCACCACACAACCTGGAGTCCATCGTGAGACAGCCGCCTGATCGGTGGATGACCAACGCCAGGATGACGCACTACCAGAGTCTGCTGCTGAATGAACGCGTCTCTTTTGCTCCTCCTGCAATTTTGAACCCTGCCACTCTACTGCCCGTGGAGTCAGATGACACCCCTATACATATTTGCTCTGAAATCCTGGCCGAGGAGACCGGCACGCGACCTGATCTGCGAGACCAACCACTTCCTGGAGTGCCGGCGTGGTACACCGACGGCAGCTCTTTCATCATGGATGGGAGACGCCAGGCCGGCGCGGCCATCGTCGACAACAAGCGCACCGTCTGGGCATCCAACCTGCCCGAGGGAACATCGGCCCAGAAGGCCGAGCTCATCGCCCTGACCCAGGCTCTGAGGCTAGCTGAAGGGAAGTCTATCAATATCTACACAGACAGCCGGTATGCATTTGCTACCGCGCACGTCCATGGGGCCATTTACAAGCAAAGAGGACTGTTGACATCTGCCGGTAAAGATATCAAAAATAAGGAAGAAATACTCGCTCTGCTAGAGGCCATCCATTTGCCAAAGAGAGTCGCCATTATCCACTGTCCTGGCCATCAGCGCGGCACAGATCCAGTGGCCACCGGAAACAGGAAGGCTGACGAAGCAGCCAAGCAGGCTGCGCAGTCCACCCGGATCCTCACCGAAACAACAAAGAACCAAGAACACTTTGAACCCACTCGGGGGAAGATCAAGCCGCGGGAGCTGACCCCTGACCAGGGCCGGGAGTTTATTCAGCGGTTACACCAGTTAACCCACCTCGGC
