# Supplementary material for: Discovery and Engineering of a Rat Endogenous Retrovirus Reverse Transcriptase for Efficient Prime Editing
Source: Adv Sci (Weinh). 2026 Jun 26:e75888. Online ahead of print. doi: 10.1002/advs.75888 (PMC13335913; doi:10.1002/advs.75888)
Supplement: Supplementary file 5 — Supporting File 5: advs75888‐sup‐0005‐TableS2.docx. [file ADVS-9999-e75888-s008.docx]

**Table S2.** Off-target editing efficiency of sites predicted by Cas-OFFinder.

| Target site | Spacer with PAM sequence | Number of mismatches compared to the spacer sequence | Editing type | Editing efficiency |
| --- | --- | --- | --- | --- |
| *OsACC1* | TTCCTCGTGCTGGACAAGTG**TGG** | 0 | +6 G to C | 82.5% |
| *OsACC1*-Off-T1 | GTGCGCGTGCTGGACAAGTG**CGG** | 3 | No mutants were found | 0.0% |
| *OsALS* | GGGTATGGTGGTGCAATGGG**AGG** | 0 | +1 G to T | 57.1% |
| *OsALS*-Off-T1 | GGGCATGGTGGTGCAGTGGG**AGG** | 2 | No mutants were found | 0.0% |
| *OsALS*-Off-T2 | GGGTTTGGGGGTGCAATGTG**CGG** | 3 |  | 0.0% |
| *OsALS*-Off-T3 | GGGTGTGGTGCTGCATTGGG**TGG** | 3 |  | 0.0% |
| *OsEPSPS* | GCAGTCACGGCTGCTGTCAA**TGG** | 0 | +4 T to C、+6 G to A、+17 G to A | 64.4% |
| *OsEPSPS*-Off-T1 | GCAGTCACTGCGGCTGCCAA**CGG** | 3 | No mutants were found | 0.0% |
| *OsEPSPS*-Off-T2 | GCAGTGATGTCTGCTGTCAA**TGG** | 3 |  | 0.0% |
| *OsGAPDH* | GAGTATGTCGTGGAGTCCAC**CGG** | 0 | +3 C to A | 77.7% |
| *OsGAPDH*-Off-T1 | GAGTTTGTTGTGGAGTCCAC**TGG** | 2 | No mutants were found | 0.0% |
| *OsGAPDH*-Off-T2 | GAGAATGACGTGGCGTCCAT**TGG** | 4 |  | 0.0% |
| *OsCDC48*-T1 | GAAGGGGTCAGCGGCGGCGC**CGG** | 0 | +5 G to T | 49.0% |
| *OsCDC48*-Off-T1 | GAAGGGGTCGGCGGCGGCGGCGG | 2 | No mutants were found | 0.0% |
| *OsCDC48-*Off-T2 | GAAGGGGTCGGCGGCGGCGGAGG | 2 |  | 0.0% |
| *OsCDC48*-Off-T3 | GAAGGGGGCAGCGGCGGCGGCGG | 2 |  | 0.0% |
| *OsPDS* | GTTGGTCTTTGCTCCTGCAG**AGG** | 0 | +4-6 GGA del | 82.1% |
| *OsPDS*-Off-T1 | GTTGTTCTCTGCACCTGCAG**GGG** | 3 | No mutants were found | 0.0% |
