# Supplementary material for: Discovery and Engineering of a Rat Endogenous Retrovirus Reverse Transcriptase for Efficient Prime Editing
Source: Adv Sci (Weinh). 2026 Jun 26:e75888. Online ahead of print. doi: 10.1002/advs.75888 (PMC13335913; doi:10.1002/advs.75888)
Supplement: Supplementary file 6 — Supporting File 6: advs75888‐sup‐0006‐TableS3.docx. [file ADVS-9999-e75888-s005.docx]

**Table S3.** PCR primers used for NGS sequencing and target locus amplification.

| Frist round PCR primer | | |
| --- | --- | --- |
| Locus | Fwd-primer | Rev-primer |
| OsCDC48-T1 | GCGTGCATGCAAATACGCCA | CCATGCTCATCCACGACACAT |
| OsCDC48-T2 | CTTGTTGAGCTCCCATTGCG | TCATCAGGAACACCAATGTC |
| OsACC1 | CCAAGGGAAATGGTTAGGTGGT | AGAACATTGCCTTTTGCAGTCC |
| OsALS | TCATCACCAACCACCTCTTCCG | CCTTGGGGATGTCGACCAGCA |
| OsEPSPS-T1 | GGCACAACAGTGGTGGACAAC | CCACCAGGAAGTCCTCCAATTC |
| OsEPSPS-T2 | CCATTGTCTTCCACAGTGGCT | CGAGTTCCCTCAACAGTCAACA |
| OsEPSPS-T3 | CCATTGTCTTCCACAGTGGCT | CGAGTTCCCTCAACAGTCAACA |
| OsGAPDH | GGTCGTGTGATTTGCAGACC | TGTGGCACATCTCTGCCTAC |
| OsPDS | GTGCATGTGTTACAGAAGGAATCCA | CAAGGTTCACAGTCCGGGAT |
| OsACC1-Off-T1 | TCGCGACAACGGGAAAGGCAG | CGACAGCGACAAGGATGACAGG |
| OsALS-Off-T1 | GGCGACGCAGCACTACACCTACAG | AATACGAACTCCGGCCATCACC |
| OsALS-Off-T2 | TCCCAAAGTAGTCCGGTTTTAACTGAG | GTGTTCTCGGAAGGAAATTGGTGCT |
| OsALS-Off-T3 | TGGTTTCTGGCCAACCGACGTC | GTTGCCGAAGAGCGAGACGG |
| OsEPSPS-Off-T1 | TAGAATGTCTCACATCCAGTATTAGATTGG | GACAGTTTACAAGGATTTAGGCGTG |
| OsEPSPS-Off-T2 | CAAGGCAGCAAAACCATATGAACAAG | TATCAAGCTTCCAACTCCAGGTGC |
| OsGAPDH-Off-T1 | CCTGCAGACATACATGTTCAAGTAT | ATCACCACACAATTTGCAACAGT |
| OsGAPDH-Off-T2 | ACGGGTGGTGATGACAAATTG | TGCGCACAACACTCATAGG |
| OsCDC48-Off-T1 | GAACACCACCCACCGCTCCT | CATGTTGACCCGGCGCATTAA |
| OsCDC48-Off-T2 | CCCCATGAATACGCTGCATTG | ACTCCTGCTTTGACCAGTTTT |
| OsCDC48-Off-T3 | CCCTTGCTCATTTGACCCAGT | TCGATCTGGCTGCCTTGACCA |
| OsPDS-Off-T1 | TGGAGAATGGCGCCGCATAC | GAAACCGTTGTCGTCGGTCAC |
| OsNRT1.1B | CTATGTTACGAGATGGAGCG | ATGTGGAGGAGGAGGTGCTA |
| HsHEK3 | GCTACGCCTGTGATGGGCTAATT | CCTCTGTTGAGCTCGACCCTG |
| HsRNF2 | CATAACCTGATCACCTCCCAAA | ACAGAAGTCAGGAATGCTTGA |
| HsDNMT1 | TGTGAGGATTGAGTGAGTTGCACG | GCGAACCTCACACAACAGCT |
| HsVEGFA | GACTCCACAGTGCATACGTGGG | CCATTCACCCAGCTTCCCTGT |
| HsEMX1 | AACTCGTAGAGTCCCATGTC | GAGCTGGATGCCCGTGTCATT |
| HsRP11 | GCTTGAGCCCATGAGTTAGAGGC | CACTGTGCTGGGCAGTTTAGG |
| HsFAS | CGCGCAAGAGTGACACACAGGT | GCGGGGTCAGCACTTCGCAT |
| HsCISH | ACTGGTTCCCTCCAGCCGCC | ACTTGCTAGCCTGGGGCAGG |
| HsPD1 | GCTCGTGGTGACCGAAGGGGAC | CTGGGGTGCTTCCAGAGCTAGAGG |
| HsTGFBR | AAAGGTCGCTTTGCTGAGGTC | CTGCCCACTGTTAGCCAGGT |
| Second round PCR primer | | |
| Locus | Fwd-primer | Rev-primer |
| OsCDC48-T1 | NNNNNNGCCGACATCCGCAAGTACC | NNNNNNACTGAACAAAAGCACGGAGG |
| OsCDC48-T2 | NNNNNNTACTGCTTTATGGACCCCCT | NNNNNNGGTGCTATGGAGTCTATCTC |
| OsACC1 | NNNNNNACCCTGGTCAGCTTGATTCC | NNNNNNCAACAATAGTCGAGCCAGCC |
| OsALS | NNNNNNTTCAGGAGCTGGCATTGATC | NNNNNNAGGAATATTGAACCCCTTAG |
| OsEPSPS-T1 | NNNNNNGGCTCTCTGTGGAAGCAGAT | NNNNNNCGTTGCATTTCCACCAGCAG |
| OsEPSPS-T2 | NNNNNNGGTCCCGTGTCCTTTCATTC | NNNNNNTGGCCATCCTGTGATCATCG |
| OsEPSPS-T3 |  |  |
| OsGAPDH | NNNNNNATGTTTGATCCTGTGCAATTT | NNNNNNGGGAACAATATGTAACAATATGC |
| OsPDS | NNNNNNCCGTTCAATGCTGGAGTTGG | NNNNNNCCTCACCTTGGTGTCTTCAC |
| *OsACC1*-Off-T1 | NNNNNNCTCAGCCTCAACCTGGGGCA | NNNNNNCCAGGCTCTCCACCGAGCAC |
| *OsALS*-Off-T1 | NNNNNNCCTCCTGATGAACATCCAG | NNNNNNACGAAGTCCGGGTACACCTC |
| *OsALS*-Off-T2 | NNNNNNGGCATCCCATCAAACACCTG | NNNNNNGTTAGGCCGGATGCACATGC |
| *OsALS*-Off-T3 | NNNNNNTAATGCATGCGCTCGCACTC | NNNNNNCGGCCTTGAACTCCAGCAG |
| *OsEPSPS*-Off-T1 | NNNNNNGGACGGAGGGATCACATCTC | NNNNNNGGCATGATAAAACTGAGGAGG |
| *OsEPSPS*-Off-T2 | NNNNNNGAGTGATATTGTGCTAGACCAG | NNNNNNGTACTCGTAGACAGTACAGAC |
| *OsGAPDH*-Off-T1 | NNNNNNCCTTCTCTTCGGTGAGAAGG | NNNNNNACAACAAACATGGGGGCATC |
| *OsGAPDH*-Off-T2 | NNNNNNCTTAGTAGACCCAGCTATCGG | NNNNNNCTCTTATTGGGATGATGGCC |
| *OsGAPDH*-Off-T3 | NNNNNNCCCACTATCAGTCTGACCAG | NNNNNNCGTAACATCTTGTGCGAGTCG |
| *OsCDC48*-Off-T1 | NNNNNNGGAGAATGCCATCTCTGCAA | NNNNNNTGGTCGGCGTAGGTGAGGAG |
| *OsCDC48*-Off-T2 | NNNNNNTTACCAGGAAGCCGCGGAAG | NNNNNNCGACCACGGCTCGCTATGG |
| *OsCDC48*-Off-T3 | NNNNNNAGGAGCGGCGACATCAGTG | NNNNNNGGAGGTTGGGATCCGCACAG |
| *OsPDS*-Off-T1 | NNNNNNCTGACTGTGTATATCTTGCTTCC | NNNNNNGCCCACTCCACATCCCTTTT |
| *OsNRT1.1B* | NNNNNNGATATACGGTCTCGCAAGCC | NNNNNNTCCACTTTAATTTACTTATG |
| *HsHEK3* | NNNNNNGGAAACGCCCATGCAATTAGTC | NNNNNNCTAGGAAAAGCTGTCCTGCGACG |
| *HsRNF2* | NNNNNNCAGACCATAGCACTTCCCTT | NNNNNNCCCAGTTTACACGTCTCATATGC |
| *HsDNMT1* | NNNNNNTCCCCAGAGTGACTTTTCCT | NNNNNNTCAGCCAAGGCCACAAACA |
| *HsVEGFA* | NNNNNNCTGGACACTTCCCAAAGGAC | NNNNNNCCAAACAGCTACATATTTGGG |
| *HsEMX1* | NNNNNNTGAAGGTGTGGTTCCAGAAC | NNNNNNTTTGTGGTTGCCCACCCTAG |
| *HsRP11* | NNNNNNCTTTCCAGAGTCCAGTGAG | NNNNNNGGTCTCCAATTGTCTACCACC |
| *HsFAS* | NNNNNNAGGCGGAGCTGCCTCTTCT | NNNNNNACTTTGCCTATCCCCGGGA |
| *HsCISH* | NNNNNNTTCCCTCCAGCCGCCGCC | NNNNNNTCACCTCTGGCCCGTCAAGC |
| *HsPD1* | NNNNNNTGGTACCGCATGAGCCCCA | NNNNNNAGATGGCCCCACAGAGGTA |
| *HsTGFBR* | NNNNNNACATACTCCAGTTCCTGACG | NNNNNNTCACTGTGGAGGTGAGCAAT |
| TRAP-PE | NNNNNNTATATATCTTGTGGAAAGGACGAAACACCG | NNNNNNTAGAAGGCACAGTCGAGGCTGATCAGCGTT |
| DMS-tile01 | NNNNNNCTGCCAGTCTCCCTGGAACACTCCG | NNNNNNAGAAGGCATCCTTGAGGTCCAGGAC |
| DMS-tile02 | NNNNNNGTGGAGGGACCCTGAAGGTGGTCAC | NNNNNNCCCTGCAGAGCTCCTGGGTGCTGGC |
| DMS-tile03 | NNNNNNCTGTAGAACAGAGGTGACCTACCTG | NNNNNNCTGTCCAAGTGAATGGCACCTTTTC |
| DMS-tile04 | NNNNNNGAAGAGACCCGTGGCATACTTAAGT | NNNNNNGTAACAGTGACTGATAGTGAGTCAT |
| Quantitative real‑time PCR primer | | |
| *HsTNF-α* | TAGCCCATGTTGTAGCAAACC | ATGAGGTACAGGCCCTCTGAT |
| *HsIL-1β* | AGCTGATGGCCCTAAACAGA | TGGTGGTCGGAGATTCGTAG |
| *HsIL-6* | CCACTCACCTCTTCAGAACG | CATCTTTGGAAGGTTCAGGTTG |
| *HsGAPDH* | ACAACTTTGGTATCGTGGAAGG | GCCATCACGCCACAGTTTC |

| Amplicons | Unedited nucleotide sequence (5' to 3') |
| --- | --- |
| *OsCDC48*-T1 | GCCGACATCCGCAAGTACCAGGCCTTCGCCCAGACTCTGCAGCAGTCTCGTGGGTTCGGCACCGAGTTTAGGTTCGCTGACCAGCCAGCGTCTGGCGCCGGCGCCGCCGCTGACCCCTTCGCATCCGCTGCCGCCGCAGCTGACGATGATGATTTATATAGTTAGATCGCTGTGCTATTTATATTTAACCTCCGTGCTTTTGTTCAGT |
| *OsCDC48*-T2 | TACTGCTTTATGGACCCCCTGGCTCTGGAAAGACCCTCATTGCTAGAGCTGTTGCTAATGAAACAGGTGCTTTCTTCTTTCTGATTAATGGCCCGGAGATTATGTCAAAGCTAGCAGGAGAAAGTGAGAGTAATCTCAGGAAGGCATTTGAAGAAGCTGAGAAGAATGCACCATCAATCATTTTCATCGATGAGATAGACTCCATAGCACC |
| *OsACC1* | ACCCTGGTCAGCTTGATTCCCGTGAGCAATCTGTTCCTCGTGCTGGACAAGTGTGGTTTCCAGATTCTGCAACCAAGACTGCGCAGGCATTGCTGGACTTCAACCGTGAAGGATTACCTCTGTTCATCCTCGCTAACTGGAGAGGCTTCTCTGGTGGACAAAGAGATCTTTTTGAAGGAATTCTTCAGGCTGGCTCGACTATTGTTG |
| *HsHEK3*-T1*, HsHEK3*-T2 & *HsHEK3*-T3 | GGAAACGCCCATGCAATTAGTCTATTTCTGCTGCAAGTAAGCATGCATTTGTAGGCTTGATGCTTTTTTTCTGCTTCTCCAGCCCTGGCCTGGGTCAATCCTTGGGGCCCAGACTGAGCACGTGATGGCAGAGGAAAGGAAGCCCTGCTTCCTCCAGAGGGCGTCGCAGGACAGCTTTTCCTAG |
| *HsRNF2*-T1 *& HsRNF2*-T2 | CAGACCATAGCACTTCCCTTCCAAATACTAAAATTGTTTTTCTCTCTTCTTTATTTCCAGCAATGTCTCAGGCTGTGCAGACAAACGGAACTCAACCATTAAGCAAAACATGGGAACTCAGTTTATATGAGTTACAACGAACACCTCAGGTAATGACTAAGATGACTGCCAAGGGGCATATGAGACGTGTAAACTGGG |
| *HsDNMT1* | TCCCCAGAGTGACTTTTCCTTTTATTTCCCTTCAGCTAAAATAAAGGAGGAGGAAGCTGCTAAGGACTAGTTCTGCCCTCCCGTCACCCCTGTTTCTGGCACCAGGAATCCCCAACATGCACTGATGTTGTGTTTTTAACATGTCAATCTGTCCGTTCACATGTGTGGTACATGGTGTTTGTGGCCTTGGCTGA |
| *HsVEGFA* | CTGGACACTTCCCAAAGGACCCCAGTCACTCCAGCCTGTTGGCTGCCGCTCACTTTGATGTCTGCAGGCCAGATGAGGGCTCCAGATGGCACATTGTCAGAGGGACACACTGTGGCCCCTGTGCCCAGCCCTGGGCTCTCTGTACATGAAGCAACTCCAGTCCCAAATATGTAGCTGTTTGG |
| *HsEMX1*-T1 & *HsEMX1*-T2 | TGAAGGTGTGGTTCCAGAACCGGAGGACAAAGTACAAACGGCAGAAGCTGGAGGAGGAAGGGCCTGAGTCCGAGCAGAAGAAGAAGGGCTCCCATCACATCAACCGGTGGCGCATTGCCACGAAGCAGGCCAATGGGGAGGACATCGATGTCACCTCCAATGACTAGGGTGGGCAACCACAAA |
| *OsALS* | TTCAGGAGCTGGCATTGATCCGCATTGAGAACCTCCCTGTGAAGGTGATGGTGTTGAACAACCAACATTTGGGTATGGTGGTGCAATGGGAGGATAGGTTTTACAAGGCGAATAGGGCGCATACATACTTGGGCAACCCGGAATGTGAGAGCGAGATATATCCAGATTTTGTGACTATTGCTAAGGGGTTCAATATTCCT |
| *OsEPSPS* | GGCTCTCTGTGGAAGCAGATAAAGTTGCAAAAAGAGCTGTAGTCGTTGGCTGTGGTGGCAAGTTTCCTGTTGAGAAGGATGCGAAAGAGGAAGTGCAACTCTTCTTGGGGAACGCTGGAACTGCAATGCGACCATTGACAGCAGCCGTGACTGCTGCTGGTGGAAATGCAACG |
| *OsGAPDH* | ATGTTTGATCCTGTGCAATTTGAAAGGAACCCTGACGAGATTCCGTGGGCTGAGGCTGGTGCTGAGTATGTCGTGGAGTCCACCGGTGTCTTCACTGACAAGGAGAAGGCTGCTGCTCACTTGAAGGTATTATCACTGTTGCTTTTCATTCCAACAAATTGGCATATTGTTACATATTGTTCCC |
| *OsPDS* | CCGTTCAATGCTGGAGTTGGTCTTTGCTCCTGCAGAGGAATGGGTTGGACGGAGTGACACTGAAATCATCGAAGCAACTATGCAAGAGCTAGCCAAGCTATTTCCTGATGAAATTGCTGCTGATCAGAGTAAAGCAAAGATTCTGAAGTATCATGTTGTGAAGACACCAAGGTGAGG |
| *HsRP11* | CTTTCCAGAGTCCAGTGAGTATTGTCTAAGGAGAGCCTATTACTAAAGAGTTCTTTGAGTTCTATCAAGGGCAATCTGGGTGGAATGGTTGGGAGATAATACCTATGGGAATGGGTTGATCAGTGAAAAGCACATGAAAAGACAGACTCTACAGATGTTTGGCTGTGAAGGAAATGAGAGATAGGTGGTAGACAATTGGAGACC |
| *OsEPSPS*-T2 & *OsEPSPS*-T3 | GGTCCCGTGTCCTTTCATTCTTCAAGTAGTTTGTTCATAAGTTGAATTCTCCTTCAATGATGTTTAAATTCATCATCTTCTTTTTTGGTGTTGTGCCAGCTGGGAGCATCGGTTGAAGAAGGTCCTGACTACTGCATCATCACCCCACCGGAGAAGCTGAACATCACGGCAATCGACACCTACGATGATCACAGGATGGCCA |
| *HsFAS*-T1 | AGGCGGAGCTGCCTCTTCTCCCGCGGGTTGGTGGACCCGCTCAGTACGGAGTTGGGGAAGCTCTTTCACTTCGGAGGATTGCTCAACAACCATGCTGGGCATCTGGACCCTCCTACCTCTGGTGAGCCCTCTCCTGCCCGGGTGGAGGCTTACCCCGTCTTAGTCCCGGGGATAGGCAAAGT |
| *HsCISH* | TTCCCTCCAGCCGCCGCCGTCCAGCCGAGTCCCCACTCCGGAGTCGCCGCTGCCGCGGGGACATGGTCCTCTGCGTTCAGGGGTAAGCGCGGCTCTCTGCCCCCTTCCCGGCCACCACGAGGGGCGCACGGGAGAACAGGGGCTTCGTGCTAGCTGCCGGGCATTGGGAGGGCTTGACGGGCCAGAGGTGA |
| *HsPD1*-T5 | TGGTACCGCATGAGCCCCAGCAACCAGACGGACAAGCTGGCCGCCTTCCCCGAGGACCGCAGCCAGCCCGGCCAGGACTGCCGCTTCCGTGTCACACAACTGCCCAACGGGCGTGACTTCCACATGAGCGTGGTCAGGGCCCGGCGCAATGACAGCGGCACCTACCTCTGTGGGGCCATCT |
| *HsTGFBR*-T3 | ACATACTCCAGTTCCTGACGGCTGAGGAGCGGAAGACGGAGTTGGGGAAACAATACTGGCTGATCACCGCCTTCCACGCCAAGGGCAACCTACAGGAGTACCTGACGCGGCATGTCATCAGCTGGGAGGACCTGCGCAAGCTGGGCAGCTCCCTCGCCCGGGGGATTGCTCACCTCCACAGTGA |
| *OsNRT1.1B* | GATATACGGTCTCGCAAGCCTGTAGGTGTGTATATACTTGGACCAGCACTACTAGATATCTAAACCATTAAGGTAGGTCAAATCAAGTTATTTTTAGGAAGCCTAATAAGTAGCCCAGTCCCAACGACGTATGGTCGCAGCCATCTGCCAGGTATATAGCTAGAGTCGACATACACATGCCCATAAGTAAATTAAAGTGGA |
| *HSSLC9B2* | GATGAACACTGGGTGTACATTTGTATATGCACATTAAGATAGCTTTTATAATGAAATACTTTATTAATTTGATGCTAAGAAAGTATCTGGAAAGCCAACTTAATCTCATTAGAAAAGAGTCATGAGCAGAAAAACTTAAAATGAAATTCACCTTTTCGCTGGTCCATCCCATGCCTGCAAGGAAAGCCATGACCAACGTGCACAGTCCTCCTGATCCAGGGAAA |
| *HSCTNNA1* | GTGTCATTGCTCTCCAAGAGAAGGATGTGGATGGCCTGGACCGCACAGCTGGTGCAATTCGAGGCCGGGCAGCCCGGGTCATTCACGTAGTCACCTCAGAGATGGACAACTATGAGCCAGGAGTCTACACAGAGAAGGTTCTGGAAGCCACTAAGCTGCTCTCCAACACAGGTACGGGAACTCTCCCTTT |
| *HSMAPK3* | CTAATCATCCCCAACTCAGCCAGCCGGGCCTCCCTCACCCAGAATGTGGTTGAGCTGATCCAGGTAGTGCTTGCCAGGGAAGATGGGCCGGTTAGAGAGCATCTCAGCCAGAATGCAGCCCACAGACCAGATGTCGATGGACTTGGTATAGCCCTGGGGGAGAGGAGGAAGTGGTGAGCTCCTGGGCCAGCCTCAACAGGGTCCTGTTGTCT |
| *HSOR56B4* | AAGTGCTCAGAGCCTTGGACATTGCTTCTGCTGAGTTCAGCCTCAGCACAGAGTGAAGGATTACAGCATAGGAAGAAAATACCAGAGCCATATCACTCCCAACCAAGACCCATGCTAGCATCAGTTGGTAAAATTTGTTCACAGTGATGTCATCACAAGCCAGGCTGATAACCCCCAAGTTAG |
| *HSSS18* | TGGTTTTTCAGGCCCACCACAGCAGTACTCAGGCCAGGAAGACTATTACGGGGACCAATACAGTCATGGTGGACAAGGTCCTCCAGAAGGCATGAACCAGCAATATTACCCTGATGGTAATCTCTCCTGATGTTAACTTTCCCATTTTCTATCCCTGCCTAATATTAATGTAGAGAGTTATTTAAAACATTTCAGTGAGGACGAAGAAACT |
| *HSCLP1* | CACTGAGGTGGCTTATGTCTCCAAGGACACTCCTATGTTGCTTTACCTCAACACTCACACAGCCTTGGAACAGATGCGGAGGCAAGCGGAAAAGGAAGAAGAGCGAGGTCCCCGAGTGATGGTAGTGGGCCCCACTGATGTGGGCAAGTCTACAGTGTGTCGCCTTCTGCTCAACTACGCAGTGCGTTTGGGCCGCCGTCCCACTTATGTGGAGCTGGAT |
